# Comparative genomics provides new insights into the diversity, physiology, and sexuality of the only industrially exploited tremellomycete: *Phaffia rhodozyma*

**DOI:** 10.1186/s12864-016-3244-7

**Published:** 2016-11-09

**Authors:** Nicolás Bellora, Martín Moliné, Márcia David-Palma, Marco A. Coelho, Chris Todd Hittinger, José P. Sampaio, Paula Gonçalves, Diego Libkind

**Affiliations:** 1 Laboratorio de Microbiología Aplicada, Biotecnología y Bioinformática de Levaduras, Instituto Andino-Patagónico de Tecnologías Biológicas y Geoambientales (IPATEC), CONICET – UNComahue, Quintral 1250, 8400 Bariloche, Argentina; 2 UCIBIO-REQUIMTE, Departamento de Ciências da Vida, Faculdade de Ciências e Tecnologia, Universidade Nova de Lisboa, Caparica, Portugal; 3 Laboratory of Genetics, Genome Center of Wisconsin, DOE Great Lakes Bioenergy Research Center, Wisconsin Energy Institute, J. F. Crow Institute for the Study of Evolution, University of Wisconsin–Madison, Madison, WI USA

**Keywords:** *Xanthophyllomyces dendrorhous*, Mycosporines, Aquaculture, Phylogenomics, Basidiomycete, Mating type, Photoprotection, Yeast, Type strain

## Abstract

**Background:**

The class Tremellomycete (Agaricomycotina) encompasses more than 380 fungi. Although there are a few edible *Tremella* spp., the only species with current biotechnological use is the astaxanthin-producing yeast *Phaffia rhodozyma* (Cystofilobasidiales). Besides astaxanthin, a carotenoid pigment with potent antioxidant activity and great value for aquaculture and pharmaceutical industries, *P. rhodozyma* possesses multiple exceptional traits of fundamental and applied interest. The aim of this study was to obtain, and analyze two new genome sequences of representative strains from the northern (CBS 7918^T^, the type strain) and southern hemispheres (CRUB 1149) and compre them to a previously published genome sequence (strain CBS 6938). Photoprotection and antioxidant related genes, as well as genes involved in sexual reproduction were analyzed.

**Results:**

Both genomes had ca. 19 Mb and 6000 protein coding genes, similar to CBS 6938. Compared to other fungal genomes *P. rhodozyma* strains and other Cystofilobasidiales have the highest number of intron-containing genes and highest number of introns per gene. The Patagonian strain showed 4.4 % of nucleotide sequence divergence compared to the European strains which differed from each other by only 0.073 %. All known genes related to the synthesis of astaxanthin were annotated. A hitherto unknown gene cluster potentially responsible for photoprotection (mycosporines) was found in the newly sequenced *P. rhodozyma* strains but was absent in the non-mycosporinogenic strain CBS 6938. A broad battery of enzymes that act as scavengers of free radical oxygen species were detected, including catalases and superoxide dismutases (SODs). Additionally, genes involved in sexual reproduction were found and annotated.

**Conclusions:**

A draft genome sequence of the type strain of *P. rhodozyma* is now available, and comparison with that of the Patagonian population suggests the latter deserves to be assigned to a distinct variety. An unexpected genetic trait regarding high occurrence of introns in *P. rhodozyma* and other Cystofilobasidiales was revealed. New genomic insights into fungal homothallism were also provided. The genetic basis of several additional photoprotective and antioxidant strategies were described, indicating that *P. rhodozyma* is one of the fungi most well-equipped to cope with environmental oxidative stress, a factor that has probably contributed to shaping its genome.

**Electronic supplementary material:**

The online version of this article (doi:10.1186/s12864-016-3244-7) contains supplementary material, which is available to authorized users.

## Background

The basidiomycetous yeast *Phaffia rhodozyma* (synonym *Xanthophyllomyces dendrorhous*) belongs to a basal lineage of the Agaricomycotina within the Tremellomycetes and possess a set of unique characteristics of outstanding scientific interest and technological value. It is best known as one of the few currently commercially exploited natural sources of astaxanthin, an economically important pigment widely used in aquaculture and pharmaceutical industries [1], with an expected global market size for 2015 of a quarter-billion dollars [2]. *P. rhodozyma* is so far the only astaxanthinogenic yeast known [3], and this carotenoid pigment is considered one of the most potent free reactive oxygen species (ROS) scavenger. Recently, numerous reports have demonstrated that astaxanthin, when used as a nutritional supplement, can act as an anticancer agent; reduce the risk of diabetes, cardiovascular diseases, and neurodegenerative disorders; and stimulate immunization [4].

This exceptional property of *P. rhodozyma* is supposed to have evolved as a result of its adaptation to live in association with plant substrates, particularly tree exudates in mountain environments where ROS are generated by high levels of UV radiation (UVR) [5], and/or the phylloplane of mountain trees where cells are directly affected by UV radiation [6, 7]. In line with this hypothesis, additional photoprotective strategies were found in *P. rhodozyma*, such as the synthesis of an antioxidant compound named Phaffiol [8] and the accumulation of mycosporine-glutaminol-glucoside (MGG), a UVB-screening compound that also has antioxidant properties [9, 10].

The microbial phylogeography and ecology of *P. rhodozyma* are also interesting due to the strong association, and possible co-evolution, of the yeast with specific tree species of birch in the Northern Hemisphere [11–13] and southern beech (*Nothofagus* spp.) in the Southern Hemisphere [6, 7, 14]. Many genetically distinct, natural populations of *P. rhodozyma* are known worldwide, but most of the diversity is found in the Southern Hemisphere, mainly in Australasia, whereas Holarctic populations are mostly genetically uniform [6]. The population structure of this yeast seems to be driven by adaptation to the different niches as a result of long-distance dispersal, and the observed genetic diversity correlates with host tree genera, rather than with geography [6].

The sexual stage of *P. rhodozyma* is unusual because it does not involve a unicellular to filamentous stage transition, an exception among basidiomycetous yeasts that might be related to the adaptive loss of filamentous structures that are normally related to the exploitation of solid substrates. In most basidiomycetous yeasts, the sexual cycle is initiated by mating of two compatible strains of distinct mating types (heterothallism) followed by the formation of a dikaryotic mycelium [15], but in the case of *Phaffia*, no such compatibility system appears to be necessary. *P. rhodozyma* has an homothallic mating behavior [16] usually involving the conjugation between the mother cell and its bud (pedogamy) on polyol-rich media [17], followed by the formation of a slender, non-septate basidium (holobasidium), with basidiospores arising terminally on its apex. Occasionally, basidial formation may result from the conjugation of identical but independent cells or without apparent conjugation (one single cell, usually larger than the vegetative cells originates the basidium) [17]. In heterothallic basidiomycetous yeasts, sexual identity is determined by mating type-specific genes encoding pheromone/receptors (P/R) and homeodomain (HD) transcription [15]. However, the presence/absence and function of these genes in homothallic basidiomycetes, including *P. rhodozyma* has not yet been fully elucidated.

Currently, two type strains have been designated for this yeast (CBS 5905 and CBS 7918^T^) since, for some time, it was believed these strains were not conspecific [18]. It was later determined that both strains belong to the same species [19], a confusion resulting from the fact that the anamorphic strain CBS 5905 is a hybrid or admixed strain derived from two genetically distinct lineages of *P. rhodozyma* [6]. Thus, we consider the Holarctic strain CBS 7918^T^ to be the valid type strain for the species. Here we selected it for genome sequencing to compare it to the previously published genome sequence of another Holarctic isolate, CBS 6938 [20]. We also sought to obtain and analyze the draft genome sequence of a representative *P. rhodozyma* strain from the Southern Hemisphere (CRUB 1149) to compare it to its counterparts from the Northern Hemisphere. In particular, we focused on genes related to photoprotection and antioxidant activities, as well as genes involved in sexual reproduction.

## Results and discussion

### Genome sequencing, assembly, and gene prediction

Here we report the genome sequence of the type strain, CBS 7918^T^, as a Holarctic representative of *P. rhodozyma*, as well as that of the Patagonian strain CRUB 1149 as a representative from the Southern Hemisphere. For comparative purposes, we also re-analyzed the recently published genome sequence of the Holarctic strain CBS 6938 [20] using the same bioinformatic framework. Further information on the strains is depicted in Table 1 and will be discussed in the following sections.

**Table 1:** Detailed information on the three strains of *P. rhodozyma* included in this study

Strain	Alternative codes	Origin and isolation reference	Lineage^a^	Genome reference
CBS 6938	UCD 77-61	Sap of *Betula* sp. stumps, Finland (Golubev et al., 1995 [17])	C2	Sharma et al., 2015 [20]
CBS 7918^T^	VKM Y-2786, JCM 9681, KCTC 17160, NCYC 2774	Exudate of *Betula verrucosa*, Moscow, Russia (Golubev et al., 1995 [17])	C2	This study
CRUB 1149	CBS 10596	Water from Lake Ilon, surrounded by *Nothofagus pumilio*, Patagonia, Argentina (Libkind et al., 2007 [19])	A	This study

^a^Based on David-Palma et al. [6] phylogenetic grouping using MLST

The *de novo* genome assembly of CBS 7918^T^ yielded an 18.7-Mb genome with 10.5-fold depth of coverage and 47.2 % GC content. This corresponded to 343 scaffolds with N50 = 104.1Kb, L50 = 55, and very low number of undefined nucleotides (total of 788 Ns (0.004 %)). Repeated and low complexity sequences cover 2.37 % of the genome, according to Repeat Masker. The CRUB 1149 *de novo* genome assembly yielded an 18.9-Mb genome assembly with a 17.5-fold depth of coverage and 47.1 % GC content. This corresponded to 305 scaffolds, N50 = 132.8Kb, and L50 = 43, and 2.47 % of repeated and low complexity sequences (Table 2). Both genomes obtained here showed similar characteristics such as GC content and percentage of repeated and low complexity sequences, but were smaller (3.0–3.9 %) than that of CBS 6938 (19.5 Mb) and had a higher number of scaffolds (Table 2).

**Table 2:** Comparative analysis of genomes, assemblies, and genes statistics for *P. rhodozyma* and other fungi

										Density			Introns	Intron	Exon	% correct
#	Species	Strain	Size (Mb)	Scaf.	N50 (Kb)	%N	%GC	%repeat	Genes	Gene/Mb	ILG	%ILG	/Gene	Length	Length	CDS
1	*Malassezia globosa*	CBS 7966	9.0	67	654.6	0.00	52.06	0.98	4195	468.3	2030	48.39	2.1	81	508	99.7
2	*Ustilago maydis*	strain 521	19.7	274	127.5	0.00	54.03	2.9	6454	327.9	4640	71.89	1.7	229	583	97.9
3	*Pseudozyma hubeiensis*	SY62	18.4	74	445.6	0.04	56.51	1.21	6313	342.3	4703	74.5	1.7	230	573	99.6
4	*Wallemia sebi*	CBS 633.66	9.8	56	337.4	0.17	40.01	0.79	5062	515.7	374	7.39	3.2	56	345	99.8
5	*Tremella mesenterica*	DSM-1558	28.6	45	1622.7	2.28	46.73	6.52	8975	313.4	1101	12.27	5.2	129	287	99
6	*Cryptococcus neoformans*	JEC21	19.1	14	1439.0	0.01	48.54	5.33	6933	363.9	343	4.95	5.4	69	257	100
7	*Cryptococcus gattii*	WM276	18.4	14	1333.1	0.07	47.88	4.53	6474	352.3	228	3.52	5.4	70	260	99.7
8	*Kwoniella heveanensis*	CBS 569	25.3	n.a.	n.a.	0.24	n.a.	n.a.	7702	n.a.	456	5.92	5.9	n.a.	n.a.	n.a.
9	*Cryptococcus dejecticola*	CBS 10117	23.9	n.a.	n.a.	0.02	n.a.	n.a.	8426	n.a.	554	6.57	5.6	n.a.	n.a.	n.a.
10	*Cryptococcus pinus*	CBS 10737	20.8	n.a.	n.a.	0.06	n.a.	n.a.	7667	n.a.	403	5.26	5.7	n.a.	n.a.	n.a.
11	*Cryptococcus bestiolae*	CBS 10118	24.4	n.a.	n.a.	0.06	n.a.	n.a.	8834	n.a.	671	7.6	5.8	n.a.	n.a.	n.a.
12	*Kwoniella mangrovensis*	CBS 10435	22.7	n.a.	n.a.	0.22	n.a.	n.a.	8242	n.a.	507	6.15	5.8	n.a.	n.a.	n.a.
13	*Cryptococcus flavescens*	NRRL Y-50378	22.8	712	71.4	0.02	58.47	0.82	8588	376.8	701	8.16	5.5	63	239	94.6
14	*Cryptococcus laurentii*	RY1	19.1	1152	32.4	0.00	56.13	1	7288	380.7	435	5.97	5.5	63	240	96.7
15	*Trichosporon asahii*	CBS 8904	25.3	194	3223.9	1.00	59.5	1.52	8520	336.8	1513	17.76	3.2	118	353	97.7
16	*Cryptococcus vishniacii*	ANT03-052	19.7	50	1080.6	0.22	52.93	0.74	6421	326.1	248	3.86	6	79	243	99.9
17	*Phaffia rhodozyma*	CBS 6938	19.5	257	2088.2	1.65	47.31	2.52	6260	321	183	2.92	7.4	112	199	97.5
18	*Phaffia rhodozyma*	CBS 7918 T	18.7	343	104.1	0.00	47.21	2.37	5980	319	97	1.62	7.5	110	199	98.3
19	*Phaffia rhodozyma*	CRUB 1149	18.9	305	132.9	0.01	47.11	2.47	6016	318	108	1.8	7.5	110	199	98.4
20	*Mrakia blollopis*	SK-4	30.5	167	1718.1	0.00	53.7	7.46	9335	306.3	229	2.45	8.2	118	177	99.4
21	*Schizophyllum commune*	H4-8	38.7	25	2560.2	0.15	57.53	4.49	12999	336.1	953	7.33	5.5	85	243	99.9
22	*Coprinopsis cinerea*	okayama7#130	36.2	67	3468.1	0.00	51.67	5.06	12265	339.3	1017	8.29	5.5	80	251	99.9
23	*Stereum hirsutum*	FP-91666 SS1	46.5	159	1799.0	1.86	51.31	1.66	13115	282	929	7.08	6.2	99	226	99.6

*Scaf.* scaffolds, *ILG* intron less genes, *n.a*. not available due to the unpublished condition of the assemblies

We also recovered, for the first time, contigs for mtDNA, rDNA cluster, and pDK1 plasmid assembled with coverages of ~1500X, ~6000X and ~2000X, respectively. The excess of sequencing coverage of these contigs suggests a copy-number ratio of 45:1 mtDNA:nDNA and the existence of ~182 clusters of rDNA in the genome of *P. rhodozyma* CBS 7918^T^. Sequences encoding all specific rRNA subunits were successfully found in both mitochondrial and nuclear contigs. The resulting CBS 7918^T^ rDNA assembly yielded 9042 nucleotides and contains the entire gene cluster: 18S, 5.8S, 28S, and 5S including IGS and ITSs regions. The partial rDNA regions of IGS, ITS, and 26S from the same strain (AF139633, AF075496, and NR077107) showed 100 % similarity to the assembled rDNA operon. Such alignments cover 2017 nts that represents 22 % of the assembled operon. Moreover, we obtained the complete CRUB 1149 rDNA cluster, which has 0.35 % mismatches relative to the CBS. This is the first report of the complete rDNA cluster (which includes phylogenetic barcodes routinely used by taxonomists) of the type strain of *P. rhodozyma*.

We predicted 5980 coding genes for CBS 7918^T^ and 6016 for CRUB 1149, both having less genes than CBS 6938 (*n* = 6385). Reciprocal best blast hits of only canonical CDS obtained using *de novo* gene predictions (6098 / 6260 for CBS 6938; 5877 / 5980 to CBS 7918^T^ and 5916 / 6016 for CRUB 1149) that the three strains share 5463 genes. The number of genes shared between CBS 7918^T^ and the CBS 6938 strains and not present in CRUB 1149 is 213. On the other hand, the CBS 7918^T^ and the CRUB 1149 share 68 genes, among this group are 3 genes responsible of mycosporine synthesis (see section). The mean percentages of identical amino acids were lower for the Patagonian strain being 98.6 % for CBS 7918^T^ and 95.0 % for CRUB 1149 relatives to the CBS 6938. The lower number of common genes and the higher dissimilarity detected in the Patagonian strain is probably related to the fact that it belongs to a genetically divergent lineage. In order to retrieve putative *Phaffia* orphan genes, we worked with the common set of predicted genes of the three strains (*n* = 5463) and kept genes with expression evidence from Sharma et al. [20] (RNA-seq of CBS 6938) that were not present in any of the other tremellomycetous yeasts and lacked hits in the NCBI nr database (see methods). This resulted in a set of 283 orphan genes that is shared among the three strains of *P. rhodozyma*, is transcribed, and contains putative CDSs that do not have known relatives in any other branch of the tree of life (Additional file 1: Table S1). However, we found that 66 % (*n* = 188) of the proteins included in this set contain at least one known protein domain (see methods), suggesting that many may have distantly related sequences that simply missed our detection threshold. Further analyses are required to determine the origin and function of these genes [21].

The density of genes per Mb for the three genomes was similar, ranging between 318 and 321 genes/Mb, and most of the genes (97–98.4 %) contained introns (7.5 introns/gene, 111 avg. intronic length and 199 avg. exonic length). The proportion of intron-containing genes and number of introns per genes are, together with species of the sister genus *Mrakia* among the highest described in any fungal species, suggesting that this might not be an exclusive trait of *P. rhodozyma*, but rather of the Cystofilobasidiales. To further evaluate this hypothesis, we used the available annotations and ran *de novo* predictions of 77 Basidiomycota and Ascomycota genomes (those listed in Table 2 plus 48 additional genomes). The average percentage of intron-less genes (ILG) and introns per gene (IPG) of the Ascomycota was 62.93 % and 1.63 respectively, while for Basidiomycota, we found a lower proportion of ILG (19.81 %) and a higher average number of IPG (4.35). Among the latter, tremellomycetous fungi showed the lowest %ILG (4.32 all or 7.52 without considering Cystofilobasidiales) and the highest IPG number (6 all or 5.22 without considering Cystofilobasidiales). Members of the Cystofilobasidiales were an extreme case among Tremellomycetes fungi with %ILG = 2.19 and IPG = 7.65. Thus, *P. rhodozyma* represents an unusual case of a yeast where almost all genes possess multiple introns. Moreover, the density of introns in the Cystofilobasidiales ranks relatively highly among all eukaryotic organisms, suggesting intron gain relative to the Last Eukaryotic Common Ancestor (LECA) [22].

To assess the quality of our genome assemblies and annotations, we retrieved 205 public sequences of *P. rhodozyma* CBS 7918^T^ from NCBI and successfully mapped them against our assembly (BlastN e-values between 3e-07 and 0.0) (Additional file 2: Table S2). Reciprocal best BLAST hits identified 2938 (49.1 %) orthologous proteins in *C. neoformans* and 1417 (23.7 %) in *S. cerevisiae*; 1328 (22.2 %) of these proteins were in common among the three taxa. We found 49 tRNAs dispersed among the scaffolds, corresponding to 12 anticodon species. Another suggested measure for quantitative assessment of the genome completeness is the number of core eukaryotic genes (CEGs) present. Applying the models defined by Parra et al. [23], we found 247 and 245 out of 248 confident CEGs, respectively, for the type and CRUB strains. The average CEGs for other tremellomycetous species with complete genomes available is 244. Thus, several analytical parameters indicate that the two assemblies and annotations of *P. rhodozyma* obtained here are adequate tools for comparative genomics and for mining of genes of fundamental and/or applied interest.

### Genomic diversity within *P. rhodozyma*

**Fig. 1:**
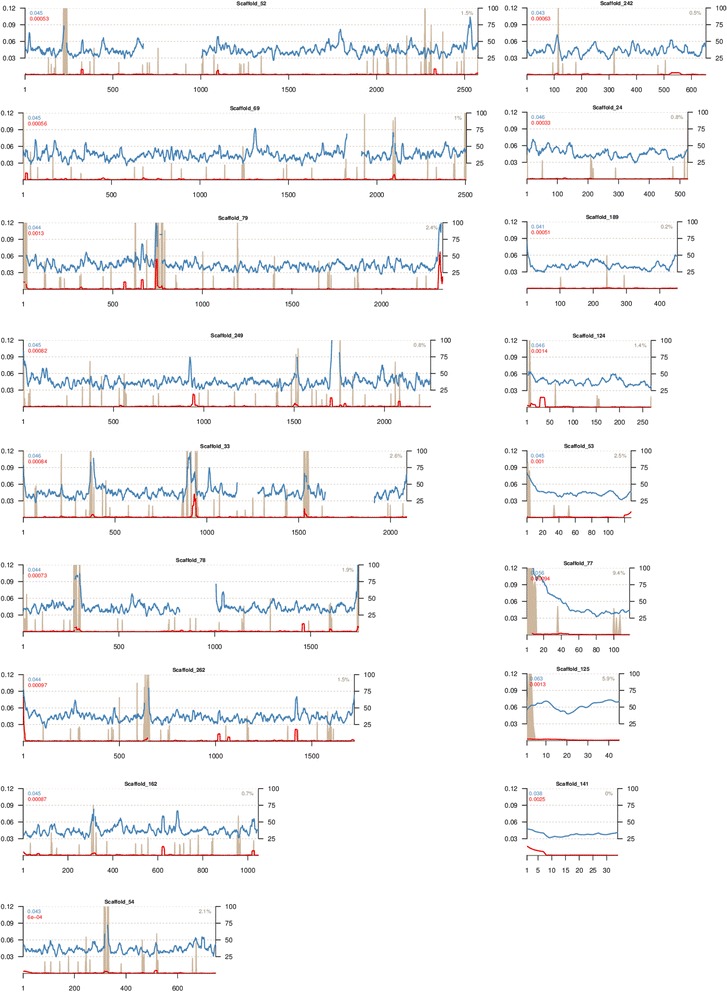
Genome sequence divergence using sliding window analysis of 3 strains of *P. rhodozyma*. Strains CBS 7918^T^ (*red*) and CRUB 1149 (*blue*) are plotted against the 17 largest scaffolds of CBS 6938 (*x axis*), and nucleotide sequence divergence (*left y axis*) is relative to the genome of this latter strain. Colored numbers represent the mean divergence for each of the two strains for each scaffold. Grey areas indicate regions of the CBS 6938 genome enriched in Ns (percentage of Ns in *right y axis*)

Previous publications, based on multiple molecular techniques and various DNA markers, demonstrated the existence of genetic heterogeneity within *P. rhodozyma* [6, 18, 19]. Due to our new data, it is now possible to apply comparative genomics on multiple strains of *P. rhodozyma*, including the type strain of the species and additional Holarctic and Southern Hemisphere representatives. We used two alternative strategies to analyze the divergence between the reported genome of the strain CBS 6938 [20] and our assemblies (see methods). As expected, whole-genome alignments and an alignment-free method (Kr) retrieved similar divergence values: 0.00073 or Kr = 0.00076 between both Holarctic strains, respectively, but 0.044 (4.4 %) or Kr = 0.06 between the Patagonian strain CRUB 1149 and the Holarctic strain CBS 6938. The divergence along the genome in the 3-way alignment showed variable values (0.03 to 0.12) when CRUB 1149 was compared to the Holarctic strain CBS 6938, while consistently low values were found between the two Holarctic strains (Fig. 1). The higher divergence values fall near the regions with gaps in the reference scaffolds of CBS 6938, even though gaps were not considered in the final calculations. Both Holarctic strains showed low genomic divergence, which is in agreement with previous reports [19], particularly with David-Palma et al. [6] whose MLST approach demonstrated that both strains belong to the same lineage (named C2 in the original report). The southern strain belongs to a separate clade (lineage A) and, as expected, was quite more divergent from the Holarctic strains. The average level of divergence found at the genome level (4.4 %) fully agrees with that found for the seven-gene (3187 bp) MLST dataset of David-Palma et al. [6]. Thus, here we validated the gene selection of our previous study and support the conclusions drawn from it.

**Fig. 2:**
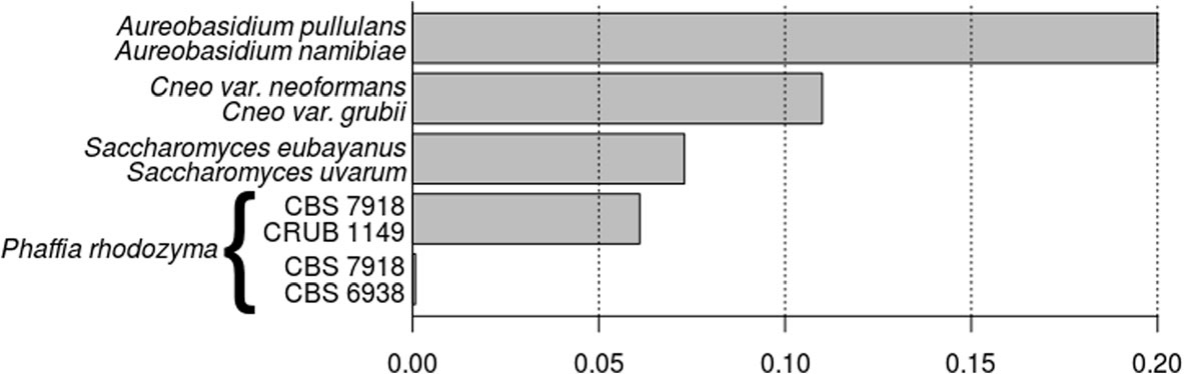
Level of genomic sequence divergence of 3 *P. rhodozyma* strains and other cases of closely related yeasts using alignment-free method (Kr). Cneo, *Cryptococcus neoformans*

To determine whether the southern population is conspecific to the Holarctic population, we compared Kr values of very closely related species or varieties of a single species, with those of Holarctic vs. southern *P. rhodozyma* strains (Kr = 0.057–0.061) (Fig. 2). The four varieties of *Aureobasidium pullulans*, recently elevated to the rank of species [24], share average Kr values of 0.2, similar to those separating *S. cerevisiae* from the psychrotolerant species *S. uvarum* and *S. eubayanus* (data not shown). These two latter taxa are the most closely related known *Saccharomyces* species with an average genome sequence divergence of ~7 %; their status as separate taxa was demonstrated by the lack of fertility of the interspecific hybrid, which produces ~93 % of unviable spores [25]. The Kr values between *S. eubayanus* and *S. uvarum* (0.073) obtained here are in agreement with previous findings and are similar to those of the most divergent *P. rhodozyma* strains. However, recent reports showed large intraspecific diversity (as high as 1 %) within both *Saccharomyces* species [26–28], so Kr values are prone to vary considerably, depending on the lineages used for comparison. The only Basidiomycota cases we could retrieve for comparison was that of the sister species *Cryptococcus gattii* and *C. neoformans*, which have Kr values 2-fold higher than *P. rhodozyma* (0.14); similarly, two varieties of *C. neoformans* (var. *grubii* and var. *neoformans*) have Kr values (0.11) closer to that of our study case. In conclusion, although the Holarctic and Southern Hemisphere strains of *P. rhodozyma* differ substantially from each other at the genomic level, based on comparison with well-studied taxa, the conspecificity of the strains remains supported in our opinion. However, the proposal of distinct varieties could be appropriate for *P. rhodozyma*, even more if the exclusive geographic distribution of certain populations is considered (e.g. Patagonia). In our opinion, such decisions should wait until the genome sequences of the two additional lineages of *P. rhodozyma* are obtained, specifically the Australasian and Japanese populations described by David-Palma et al. [6] (lineages B and D, respectively). Based on MLST analysis, the two strains isolated from *Cornus* spp. in Japan are genetically more divergent from the type strain than from the Patagonian population [6], already suggesting more than two varieties of *P. rhodozyma* might be proposed in the future.

### Photoprotective and antioxidant strategies in *P. rhodozyma*

There is evidence that *P. rhodozyma* has evolved strategies to cope with high levels of environmental oxidative and UV radiation (UVR) stress [9, 29, 30]. However, with the exception of the elucidation of most of the genes involved in astaxanthin synthesis due to applied interests, there is scarce knowledge on the genetic basis of complementary strategies against oxidative stress. For example, the genes encoding the enzymes involved in the synthesis of mycosporine-glutaminol-glucoside (MGG) and ROS scavenging enzymes, such as catalases and superoxide dismutases (SODs), remain uncharacterized. Our genome mining of newly sequenced strains of *P. rhodozyma* allowed the localization of all known genes related to the synthesis of astaxanthin (Additional file 3: Table S3). We were also able to identify a hitherto unknown gene cluster that may be responsible for MGG synthesis in yeasts, as well as a battery of enzymes (catalases and SODs) whose orthologs in other fungal species act as scavengers of free radical oxygen species (ROS) (Additional file 3: Table S3).

### Astaxanthin

The complete set of genes responsible for astaxanthin biosynthesis were annotated for both *Phaffia* strains (Additional file 3: Table S3) and compared to those of CBS 6938. With the exception of the crtR enzyme (0.13 % amino acid sequence divergence), we did not find any nonsynonymous differences between the Holarctic *P. rhodozyma* strains for the seven genes studied. On the other hand, the CRUB 1149 strain showed aminoacidic dissimilarities when compared to CBS 6938 for all these genes (values ranged 0.39–2.52 %). Nucleotide sequence variability has already been reported for some of these genes, and partial sequences of *idi*, *crtI*, and *crtS* proved to be valuable molecular markers for genetic differentiation of the distinct lineages of *P. rhodozyma* [6].

Although astaxanthin biosynthesis has been elucidated at the genetic level, the complex regulatory mechanism controlling this process is scarcely known, and it is a focus of ongoing research [31]. The repressive effect of glucose on the expression of the *crtYB*, *crtI*, and *crtS* genes was demonstrated in *P. rhodozyma* and potential Mig1-binding sites in the promoter regions of the three genes were found, suggesting that transcriptional regulation mechanisms may be involved in this inhibition [31, 32]. We located a putative homolog (*G04777_PC*) of the *Cryptococcus gattii* Mig1 gene. Moreover, we were able to identify a gene that encodes for a beta-carotene 15,15′-monooxygenase (*G04735_P*), which is related with the β-carotene cleavage oxygenases produced in *Fusarium fujikuroi* by the gene *CARX* [33] and in *Ustilago maydis* by the gene *Cco1* [34]. These enzymes are related to the production of retinal via the cleavage of different carotenoids with at least one β-ionone ring. Retinal is one of the best-known apocarotenoid and serves as a chromophore for opsins, which are involved in several photoreceptor functions. In *Fusarium fujikuroi*, the production of retinal is involved in the regulation of the carotenoid biosynthetic pathway via a negative feedback mechanism [33]. The presence in *P. rhodozyma* of this gene, which has proven regulatory functions in the carotenoid biosynthesis of other fungi, leads us to speculate that it may play a similar role in this yeast. To the best of our knowledge this gene has been scarcely studied in basidiomycetous fungi. It also represents a potential new target for genetic engineering approaches aimed at the improvement of astaxanthin yields in *P. rhodozyma*.

### Mycosporines

Mycosporines (MYC) synthesis has recently been shown to occur through two distinct pathways in cyanobacteria [35, 36]. Blaskus & Walsh [37], proposed a biosynthetic route in cyanobacteria that consists of a specific cluster of three genes that encodes a DHQS homolog (2-epi-5-epi-valiolone synthase, EEVS-like) O-methyltransferase (O-MT) and a gene encoding an ATP-grasp [37]. In the *P. rhodozyma* CBS 7918^T^ genome, we identified an 8-kb-long gene cluster in scaffold 175 that encodes at least three proteins (EEVS-like, O-MT and ATP-Grasp) that are homologous to those responsible for mycosporine synthesis in cyanobacteria [37]. This finding is consistent with the fact that *P. rhodozyma* produces the sunscreen molecule mycosporine-glutaminol-glucoside (MGG) [9]. An identical cluster of genes was detected in the Patagonian strain CRUB 1149, which, along with 15 other *P. rhodozyma* isolates tested, also produces MGG. Thus, we were surprised when we failed to detect any evidence of the putative MGG cluster in the >100x coverage genome assembly of CBS 6938, either using the original assembly, or our own assembly. RNA-seq data from the same strain also failed to reveal the expression of genes from a MGG cluster. When we experimentally tested CBS 6938 for mycosporine synthesis, we found that it was negative, a result that agrees with the absence of the gene cluster in its genome. The strain CBS 6938 represents the first exception to this hypothesis that all strains of *P. rhodozyma* can synthesize MGG [9, 38], although it might not be the only one because additional *P. rhodozyma* MGG-negative isolates have recently been recovered from Antarctic environments [39]. These results reinforce the hypothesis that the synthesis of MGG in yeasts is not essential and is rather an adaptation for coping with environmental stress conditions, specifically UV light and/or oxidative damage. A positive correlation between MGG synthesis and UVR tolerance has been previously reported for yeast [10].

The present study provides the first insight into a set of genes that could potentially be responsible for the synthesis of MGG in *P. rhodozyma*, an interesting photoprotective compound with potential application in cosmetics. Similar molecules named mycosporine-like amino acids (MAAs) are being already exploited as active ingredients of commercial sunscreen products, such as Helioguard and Helionori [36]. Thus, although our findings need experimental validation (ongoing work in our lab), it definitely represents a step forward towards a better understanding of the molecular basis of the biosynthetic pathways that give rise to the evolutionarily and industrially important metabolite.

### Catalase enzymes

**Fig. 3:**
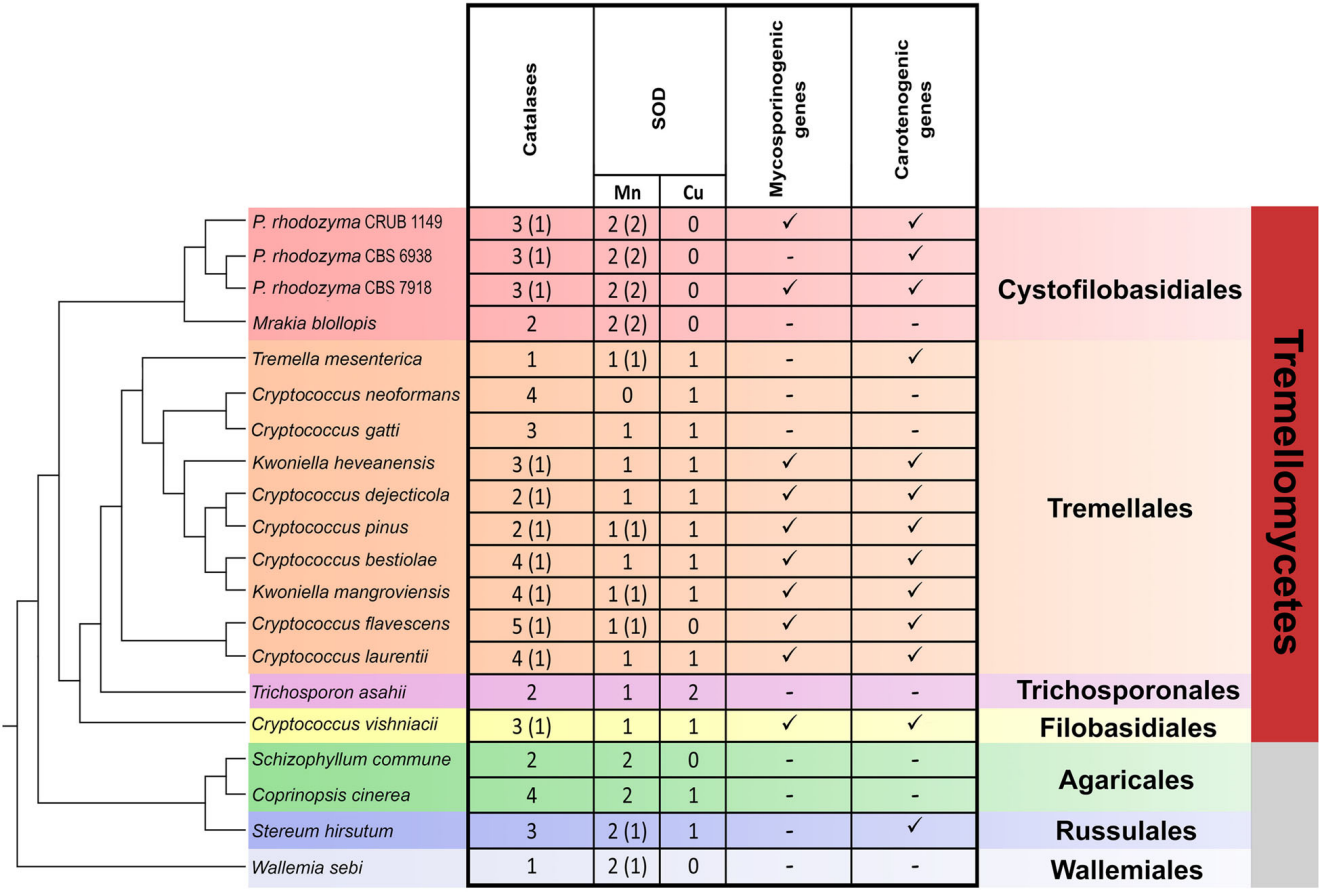
Cladogram of the Tremellomycetes based on 248 protein sequences belonging to CEGs and the presence of photoprotective and antioxidant related genes. * lack of mycosporines was confirmed experimentally in this study. Numbers in brackets correspond to proteins predicted to have similar functions. All branches had maximum *a posteriori* support values

**Fig. 4:**
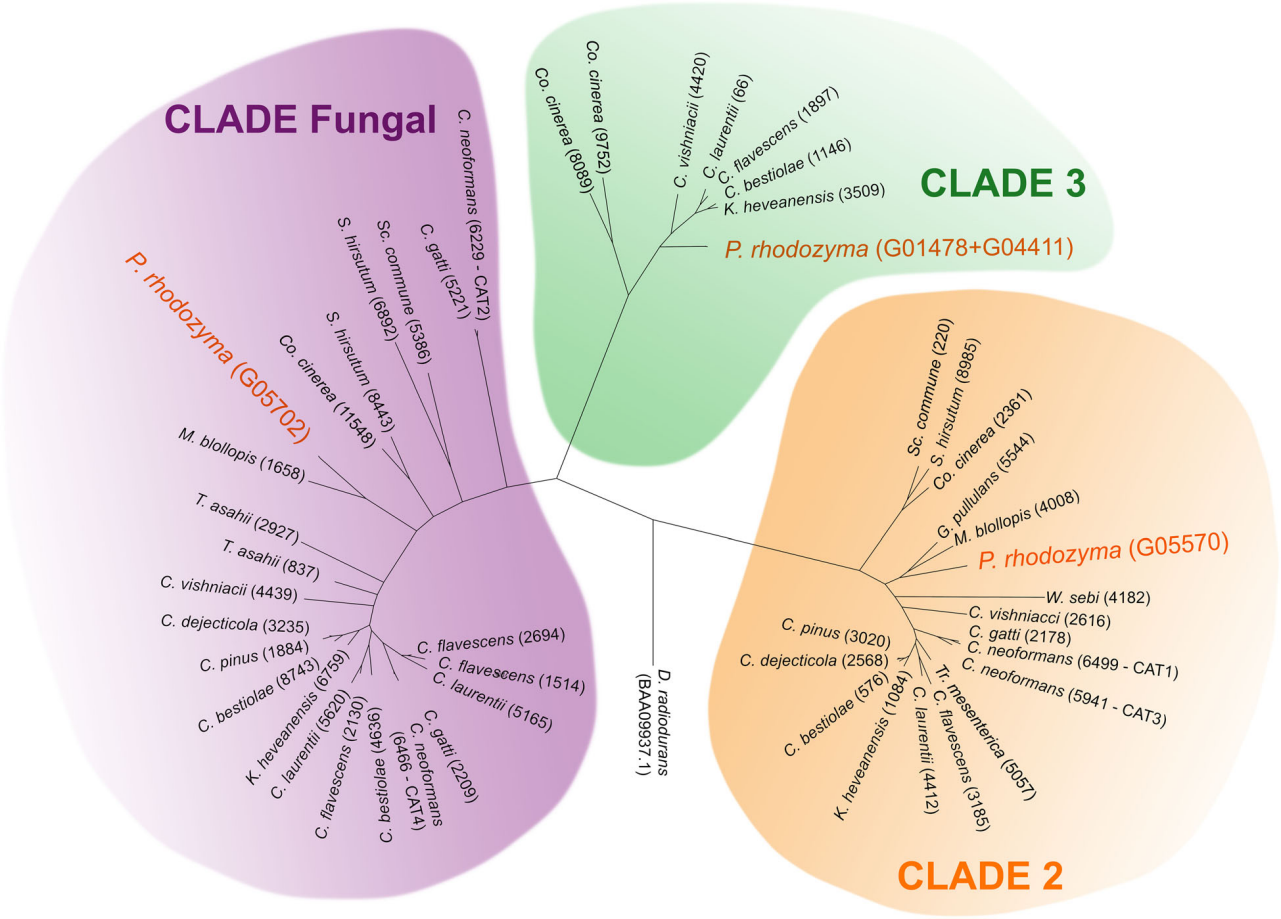
Phylogenetic tree based on protein sequences of 3 different clades of catalases from *P. rhodozyma* CBS 7918^T^ and other fungi. C.: *Cryptococcus*; Co.: *Coprionopsis*; K.: *Kwoniella*; M.: *Mrakia*; S.: *Stereum*; Sc.: *Schizophyllum*; T.: *Thichosporon*; Tr.: *Tremella*; W.: *Wallemia*

When compared to related fungi (Tremellomycetes), several antioxidant enzymes were found in *P. rhodozyma* (Additional file 3: Table S3), suggesting that this yeast has multiple antioxidant strategies, probably due to the environmental stress imposed in its natural habitat. The enzymatic ability of *P. rhodozyma* to withstand hydrogen peroxide (H_2_O_2_) has been examined before [45] and a significantly lower catalase activity in *P. rhodozyma* when compared with *Saccharomyces cerevisiae* was observed. Other authors have suggested that astaxanthin is produced by *P. rhodozyma* to supplement its catalase deficiency and defend against H_2_O_2_-induced oxidative stress [40]. Contrary to these expectations, the analysis of the genome revealed a surprising diversity of catalases, suggesting that *P. rhodozyma* is well adapted to cope with oxidative stress and that it may possess even more catalases than the sister genus *Mrakia* (Cystofilobasidiales) (Fig. 3). We identified at least three genes in *P. rhodozyma* that, based on comparative analysis to previously known catalases, seem to belong to three different categories (Fig. 4). Most fungi have several monofunctional heme-catalases. In Ascomycota, there are two large groups: 1) the large subunit catalases, such as those present in Pezizomycotina, which are associated with spore germination and cell differentiation; and 2) the small-size subunit catalases located in the cytosol or the peroxisome that are common in Saccharomycotina [41]. In *P. rhodozyma*, the large-subunit catalase (716 aa encoded on scaffold 210), possess a glutamine amidotransferase (GATase1)-like domain at the C-terminus. This catalase is also present in other Cystofilobasidiales (*Mrakia*) and most Tremellales. It belongs to Clade 2 (cd08155) and is related to the spore-specific catalases (CAT 1 and CAT 3) found in *C. neoformans* [42, 43]. The second catalase belongs to fungal clade cd08157, has a small subunit size (524aa encoded on scaffold 230), and is related with the peroxisomal (CAT 2) and cytoplasmatic (CAT 4) catalases of *C. neoformans*, as well as to Cta1 and Ctt1 of *S. cerevisiae*, respectively [42]*.* This catalase probably has a cytosolic localization in *P. rhodozyma* because it is more closely related to *C. neoformans* CAT 4. Finally, the third *P. rhodozyma* catalase, which has a relatively small subunit (589aa, found partially in scaffolds 19 and 118 and manually curated as depicted in Additional file 3: Table S3) belongs to the Clade 3 (cd08156). This family includes the most versatile and abundant monofunctional catalases, and representatives are found in all three kingdoms of life [44]. Surprisingly, the catalases of Clade 3 are only present in 8 out of 20 species of analyzed Tremellomycetes genomes. Notably, although *P. rhodozyma* has fewer catalases than the well-studied pathogen *C. neoformans* (which has four catalases from two classes)*,* all three catalases of the former are of different types thus possibly providing higher plasticity to cope with H_2_O_2_. It is worth studying whether having single-copy genes of three different classes of catalases (as in the case of *P. rhodozyma*), rather than two classes with two copies each (as in *C. neoformans*), implies a fitness advantage under oxidative stress conditions.

A sequence matching the conserved domain cd08152, which is related to the y4iL protein of *Rhizobium* sp. NGR234, was also found in *P. rhodozyma* (encoded on scaffold 66). This protein, of bacterial origin, shares the catalase fold and heme binding motif, suggesting that it might have also catalase activity. This y4iL-like gene is present in other 8 species of the Tremellales studied, as well as in the 3 genomes of *P. rhodozyma*. Although the activity of this gene is uncharacterized, it possibly contributes to the H_2_O_2_ - inactivating mechanisms of *P. rhodozyma*.

### Superoxide dismutase enzymes

The presence of Mn-SOD and the absence of Cu/Zn-SOD in *P. rhodozyma* was previously described reported [45, 46]. Given that it has been reported that Mn-SOD is present only in the mitochondria, while Cu/Zn-SOD is cytosolic [47], it was proposed that *P. rhodozyma* would be hypersensitive to oxidative stress. Interestingly, genome sequence analysis revealed the presence of two different genes encoding Mn-SODs, confirming the possible existence of two isozymes [45]. Signal peptide sequences analysis suggests that one Mn-SOD could be localized to the mitochondria and the other to the cytosol. In the ascomycetes, multiple gene duplication events of the gene encoding the mitochondrial Mn-SODs were coupled with the subsequent loss of the amino-terminal mitochondrial targeting sequence, and it has been proposed that basidiomycetes also underwent a late gene duplication [48]. The two isozymes we observed in *P. rhodozyma* likely correspond to this latter duplication event, which also provides an evolutionary mechanism for their possible differential subcellular localization. It is remarkable that both Mn-SODs are present in *Wallemia sebi* and *Stereum hirsutum*, suggesting that the duplication is a plesiomorphic character in the Agaricomycotina. However, Tremellales lost the gene encoding the cytosolic Mn-SOD, while Cystofilobasidiales lost the gene encoding the cystosolic Cu-SOD. SOD activity in *P. rhodozyma* is known to confer resistance to KCN and H_2_O_2_, two compounds that affect Cu/Zn-SOD but not Mn-SOD. The existence of three different types of catalases and the lack of H_2_O_2_-sensitive SOD enzymes suggests that interaction with this reactive species has been important in shaping *P. rhodozyma* genome.

### Genes involved in sexual reproduction

**Fig. 5:**
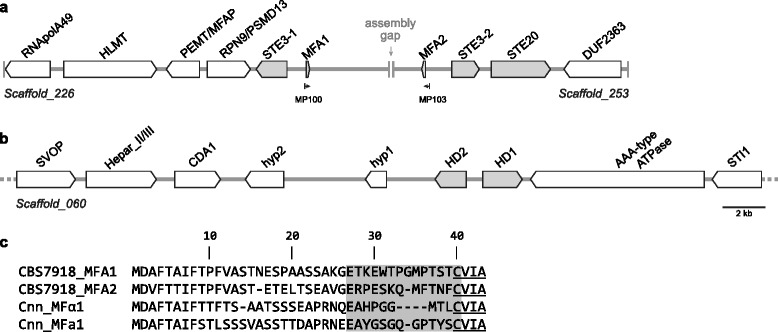
Gene content and organization around the putative mating type genes of *P. rhodozyma*. **a** Genomic organization of the DNA regions with putative mating pheromone receptors (Ste3-1 and Ste3-2), pheromone precursors (Mfa1 and Mfa2) and **b** homeodomain transcription factors (HD1 and HD2). Genes are indicated by arrows showing the direction of transcription. Arrows in grey indicate putative mating related genes while those in white depict additional genes apparently unrelated to mating. Hyp1-2 are hypothetical predicted proteins of uncertain function. The end of a scaffold is indicated by a solid vertical bar. The contiguity of the scaffolds harboring the two sets of pheromone and receptor genes was confirmed by PCR with primers MP100 and MP103. **c** Sequence alignment of the putative pheromone precursors from *P. rhodozyma* (CBS 7918^T^) and *C. neoformans* var. *neoformans* (Cnn Mfα1 - XP_570122.1; Cnn Mfa1- AAG42766.1). The predicted mature pheromones are shaded in grey and the CAAX-motifs for C-terminal processing are underlined

In the genome assembly of the *P. rhodozyma* type strain, CBS 7918^T^, we identified three scaffolds harboring putative homologs of genes that determine sexual identity in basidiomycetes [15], namely the mating pheromone and receptors (*P*/*R*) and the *HD1/HD2* homeodomain (HD) transcription factors, which are usually encoded at the mating-type (*MAT*) loci. Two divergently transcribed genes, *HD1* and *HD2*, were found on scaffold 60, mirroring the arrangement observed at *HD* loci of most basidiomycetes [15] (Fig. 5; Additional file 4: Table S4). Based on sequence similarity with *MAT* genes of *C. neoformans*, we also identified for the first time two putative genes encoding pheromone precursors and two receptor genes. These genes were located on two different scaffolds (numbered 226 and 253), each of which contains a putative pheromone receptor (*STE3*) and a pheromone precursor gene (*MFA*). However, in the genome of strain CBS 6938 [20], the two sets of genes (*STE3-1/MFA1* and *STE3-2/MFA2*) are located on the same scaffold approximately 5 kb apart (Fig. 5). Using PCR, we were able to confirm that the two gene sets are similarly positioned in strain CBS 7918^T^, suggesting that these two scaffolds are linked. The two pheromone precursor genes (*MFA1* and *MFA2*) are predicted to encode proteins that are about 58 % identical and yield different mature pheromones that undergo post-translational modification (farnesylation) at the cysteine residue of the CAAX motif (Fig. 5). Likewise, the two pheromone receptor genes also encode different proteins with about 50 % sequence identity. All putative *MAT* genes are devoid of inactivating mutations. Both pheromone receptors are predicted to have seven transmembrane domains (Additional file 5: Figure S1), which is similar to their counterparts in other fungi, and the homeodomain transcription factors encoded by the *HD1* and *HD2* genes have the expected domain structure (Additional file 5: Figure S1) [15]. Genetic analyses allowed us to propose recently a role for these genes in sexual reproduction of *P. rhodozyma* [49].

Our genome survey also identified orthologs encoding components of the conserved pheromone response pathway that is activated upon pheromone/receptor interaction during mating in *C. neoformans*, namely the genes encoding the subunits of the heterotrimeric G protein (*GPA1-3*, *STE4*, and *STE18*) and those that compose the MAP kinase module itself (*STE11*, *STE7*, and *CPK1*). Moreover, we identified a gene encoding a p21-activated kinase (*STE20*) in the vicinity of the *STE3-2* gene, which is consistent with observations in other basidiomycetes, viz. species of the pathogenic *Cryptococcus neoformans* complex [50] and of the sensu lato *Kwoniella* clade [51] in Tremellales (Agaricomycotina), as well as in yeast species in the Pucciniomycotina [52, 53]. A final set of orthologs encoding transcription factors that have key roles in mating in *S. cerevisiae* (Ste12) [54], *C. neoformans* (Mat2 and Znf2) [55], and *U. maydis* (Prf1) [56] were also found and are listed in Additional file 4: Table S4. The analysis of the genome assembly of *P. rhodozyma* strain CRUB 1149 yielded identical results to those obtained with the type strain (CBS 7918^T^).

## Conclusions

The present study provides new genomic insights into several biological and genetic aspects of the industrially relevant yeast *P. rhodozyma*, an early diverging Agaricomycete with exceptional physiological and ecological properties. By analyzing, for the first time, the valid type strain of the species together with a genetically distinct lineage from the Southern Hemisphere, comparative genomic analyses within the species and among the Tremellomycetes become possible. Indication of conspecificity of the northern and southern strains was obtained, though due to the relatively high level of genomic divergence detected, these strains might be considered different varieties in the future. The proportion of intron-containing genes and the number of introns per gene in *P. rhodoyma* are the highest hitherto known for fungi, having values similar to those found in humans. Although it remains to be confirmed using a larger set of genomes, available data suggest that this trait might not be species-specific but rather might be shared with other members of the Cystofilobasidiales. Genome mining provided the first insight into the genetic basis of the synthesis of mycosporine-glutaminol-glucoside, another biotechnologically important molecule due to its antioxidant and UV sunscreen activities. We also observed that a putative cluster of genes found in MGG-producing strains was absent in non-mycosporinogenic ones. Further studies are in progress to elucidate the biosynthetic pathway for MGG synthesis in yeasts. The study of genes encoding additional enzymes that protect against oxidative stress revealed an unexpected diversity of catalases and the loss of H_2_O_2_-sensitive superoxide dismutases. Our results indicate that the *P. rhodozyma* genome is enriched in antioxidant mechanisms, in particular those most effective to cope with H_2_O_2_, suggesting that the environmental interaction with this reactive species has been of great relevance to the evolution of *P. rhodozyma*.

## Methods

### Strain, culture conditions, and DNA sequencing

Yeasts were cultured 72 h in 15 ml YM broth (g/l, yeast extract 3; malt extract 3; peptone 5; dextrose 10) at 20 °C, and genomic DNA was extracted using a modified phenol:chloroform:isoamyl alcohol method [57]. DNA was dissolved in TE buffer (10 mM Tris–HCl, 1 mM EDTA, pH 7.6) with RNase A (100 μg/ml). Paired-end Illumina libraries with an average length of 455 bp, as measured by Agilent 2100 Bioanalyzer, were constructed following Hittinger et al. [58]. The genome of *Phaffia rhodozyma* CBS 7918^T^ and CRUB 1149 were sequenced by using Illumina GA II_X_ paired-end reads. A total of 6,995,372 paired-end reads with a length of 115 nucleotides were generated for a combined depth of coverage of 41X-fold for the CBS 7918^T^ and 9638996 paired-end reads with same length for the CRUB 1149 with a coverage of 57X-fold.

### Genome assembly and correction

De novo genome assembly was performed with SPAdes 3.1.1 [59], including adapter removal, trimming, quality filtering, and error correction, resulting in 6805670 paired-end reads with a mean length of 115 nt and an estimated insert size of 317 nt, yielding 769 contigs with length > =200 bp for the CBS 7918^T^ strain. Out of 361 scaffolds with a length >2Kb, we selected 343 with a median read coverage of 10.5, standard deviation of 5.1, a minimum of 5.2, and maximum of 71.2. The average GC content was 47.3 % with a standard deviation of 2.7 %. The remainder 18 scaffolds had coverages between 357 and 2074 and, together with shorter contigs, were considered as non-nuclear DNA for contig extension rounds (see below). The assembly of the 9356088 CRUB 1149 corrected reads with mean length of 115 nt and an estimated insert size of 327 nt, yielded 642 contigs with length > =200 bp. Out of 322 scaffolds with length >2Kb we kept 305 with average coverage of 17.5. Mitochondrial DNA, pDK1 plasmids, and regions of rDNA nuclear clusters were assembled using custom scripts. Such scripts performed: read alignment to NCBI deposited sequences, selection of seed contigs, extension of contigs by multiple sequence alignment, and information content (IC) calculation. Reads were aligned to 28S-5S ribosomal RNA ITS 1 partial sequence and to pDK1 plasmid sequence with Blat v34 [60] with the default parameters (NCBI accession numbers AF139633.1 and AJ278424.1 respectively). *Cryptococcus neoformans* mitochondrial proteins were downloaded from Broad Institute, and alignments were performed with Blast v2.2.17 (parameter: -Q4) [61] and Blat to mitochondrial-encoded rRNAs, RNL, and RNS. A seed read with maximum coverage were selected for each target sequence. Rounds of contig extension were performed by aligning reads with Lastz v1.03.54 [62] (parameters: --step = 10 --seed = match12 --notransition --exact = 20 --noytrim --match = 1,5 --ambiguous = n --coverage = 40..100 –identity = 95) fixing positions with IC > 0.5 and a minimum coverage of 100.

### Gene prediction and functional annotation

Ab initio gene prediction with GeneMark-ES v2.3e [63] was self-trained on the genome scaffolds (parameters: --min_contig 8000 --max_nnn 1000). Repeats and low-complexity sequences were retrieved by RepeatMasker v4.0.3 [64] using the RepBase library [65]. tRNAs were predicted by tRNAscan-SE v1.23 [66]. ncRNAs, including rRNAs, were predicted with HMMER v3.1b1 [67]. Automatic annotation of genes was performed by recording the best reciprocal blastp hits (e-value < 10^−5^, identity > 50 %) to the *Cryptococcus neoformans* (Broad Institute) and *Saccharomyces cerevisiae* (SGD) proteomes. Blast2GO [68] was used to retrieve functional annotations. Additionally, multiple sequence alignments were generated by MAFFT v6.935b [69]. Enzymatic domains were predicted using PRIAM [70] (parameters: -pt 0.5 -mo -1 -mp 70 -cc T -cg T -e T -cm T), and related pathways were retrieved from the KEGG public database. No automatic improvements or consensus gene models were made by combining evidence due to low abundance of available mRNAs or ESTs. However, manual curation was applied to all genes with transcript sequences deposited in NCBI. We used tblastn and blastp to map genes involved in meiosis, mating, and the synthesis of photoprotective and antioxidant compounds from available sequences of *Phaffia* sp., *Cryptococcus,* sp., *Ustilago maydis*, *Neurospora crassa* and *Saccharomyces cerevisiae* (NCBI, JGI, and SGD databases, all permission granted). Protein domains were predicted with PFAM [71].

### Genes survey

In order to compare between gene sets we use 77 fungal genomes, 23 of Tremellomycetes show at Table 2. Since gene prediction strategies differed between genome submissions, and it would introduce inconsistencies in the comparative analyses, we predicted such gene structures with GeneMark-ES v2.3e [63] using same parameters applied to the two strains sequenced in the present study. Gene features listed in Table 2 were retrieved from de-novo predictions. We selected just canonical CDS (ATG-STOP) to analyze gene orthology in *Phaffia* strains. Best reciprocal hits (BRH), blastp (e-value < 10^−5^), were calculated between the three possible pairs of gene sets using as primary and secondary selective criterion the e-value and number of nucleotides aligned respectively. Shared genes among the 3 strains or present only in two genomes were selected by pairs of consistent BRH. The putative set of orphan genes was defined from common genes for the 3 *Phaffia* strains with transcriptional evidence, at least 50 % of read to gene sequence coverage, from CBS 6938 RNA-seq data [20]. Predictions present in any other Tremellomycetes were filtered out using blastp (e-value < 10^−5^). A second filter using blastp with the nr NCBI database discarded any gene with a hit (e-value < 10^−5^) to any other species. Among the resulting 286 genes, we found 188 containing at least one PFAM domain.

### Comparative genomics

Pairwise genome-wide alignments were produced with Blat using the default parameters [60]. Sequence divergence among the 3 *P. rhodozyma* genomes was estimated by applying a sliding windows approach using the longest scaffolds of the strain CBS 6938 as references, corresponding to 17 scaffolds that covers 98.73 % of its assembly. We used a window size and step of 1Kb; windows with less than 500 bp unambiguously called between the 2 alignments were discarded. Finally, the divergence value for each datapoint was calculated as the average for such window plus the five windows on each flank [25]. Alignment-free pairwise distances, Kr, based on shustrings was calculated with GenomeTools [72]. Although Kr values above 0.5 are generally regarded as unreliable, we used a more restrictive threshold of 0.3 because we found inconsistent values between genome triads at high Kr levels.

### Phylogenetic tree reconstruction

A total of 21 proteomes were obtained from JGI and UCSC databases (permissions granted). The set of 248 core eukaryotic genes (CEGs) were scanned with HMMER, applying the specific models and thresholds defined by Parra et al., [23]. Multiple alignments were produced using MUSCLE [73] (default parameters) for the common 210 orthologous proteins. These alignments were concatenated, gaps were eliminated, and the resulting length was 60,298 columns. The phylogeny was inferred using MrBayes v3.2.2 [74]. The tree was reported as a cladogram. Alignment and other parameters are available as supplementary material.

### Analysis of photoprotective and antioxidant genes

Genes involved in the synthesis of photoprotective and antioxidant metabolites or enzymes were identified in the genome assemblies of *P. rhodozyma* CBS 7918^T^ and CRUB 1149, by tblastn or by blastp using the predicted proteins. Mycosporine genes were detected using the protein sequences of the genes Ava_3856, Ava_3857 and Ava_3858 from *Anabaena variabilis* [37]. The carotenoid genes *crtE,* c*rtI,* and *crtYB* were used to identify candidate genes for carotenoid production in Tremellomycetes. Catalases were detected using representative proteins of the families cd08154, cd08155, cd08156, cd08157, and cd08152; these sequences were downloaded from the Conserved Domains Database (CDD) of NCBI. Superoxide dismutases were identified using representative proteins of the families PF00080, PF00081, PF02777, and PF09055 as queries; these sequences were downloaded from the PFAM database of EMBL-EBI. Catalases were categorized using phylogenetic analyses that compared *P. rhodozyma* protein sequences with the predicted protein sequences from other Tremellomycetes. Sequences were aligned using MUSCLE [73], and unrooted phylogenetic trees were constructed using RAxML v. 7.3.5 [75] with the PROTGAMMAWAG model of amino acid substitutions, eliminating columns with gaps. Branch supports were determined using 1000 rapid bootstrap replicates. The subcellular localization of the superoxide dismutases were predicted using TargetP 1.1 (http://www.cbs.dtu.dk/services/TargetP/) [76].

### Identification of genes involved in sexual reproduction

The genomic regions containing the genes that determine sexual identity in basidiomycetes (genes encoding the homeodomain transcription factors *HD1/HD2* and the mating pheromones/receptors) were identified in the genome assemblies of *P. rhodozyma* CBS 7918^T^ and CRUB 1149 (or in local databases of proteins resulting from genome annotation) by reciprocal tblastn and blastp, respectively, using *C. neoformans* Mat proteins (Sxi1, Sxi2, Mfa1, and Ste3) as queries (Additional file 4: Table S4). Pheromone precursor genes failing detection by blast due to their short length and highly variable sequences were identified manually upon inspection of the genomic regions in the vicinity of pheromone receptor genes by searching for the existence of ORFs whose deduced protein sequences contained a conserved CAAX motif at the C-terminus. To ascertain the contiguity of the scaffolds harboring the two sets of pheromone and receptor genes, a pair of primers (MP100 5′-TCCATCCTCAACTGATTGC-3′ and MP103 5′-TTCATCTTGTCAGACAGC-3′) were used to amplify and partially sequence the intervening region between both pheromone precursor genes. Standard PCR and cycling conditions were used with Phusion® High-Fidelity DNA Polymerase using an annealing temperature of 51 °C and extension for 90 s. Protein sequences of genes involved in the pheromone signaling cascade in *C. neoformans* [77], were used to identify the corresponding putative orthologs in *P. rhodozyma* by blast searches (Additional file 4: Table S4).

## Additional files

Additional file 1: Table S1. List of *P. rhodozyma* putative orphan genes. (XLS 44 kb)
Additional file 2: Table S2. NCBI public sequences found in *P. rhodozyma* CBS 7918^T^. (XLS 1327 kb)
Additional file 3: Table S3. List of photoprotective and antioxidant genes of *P. rhodozyma*. (XLS 1304 kb)
Additional file 4: Table S4. List of mating genes of *P. rhodozyma*. (XLSX 192 kb)
Additional file 5: Figure S1. Secondary protein structure features of the homeodomain transcription factors and pheromone receptors of *P. rhodozyma*. (a) Regions of HD1 and HD2 proteins corresponding to the homeodomain and the three typical helical regions (grey). Comparison of these features was performed with the homeodomain transcription factors of *Kwoniella heveanensis* (Kwohev) (HD1 - ACZ51528 and HD2 - ACZ51529, respectively). (b) Pheromone receptor proteins highlighting the seven transmembrane regions (green) as predicted by HMMTOP software (http://www.enzim.hu/hmmtop/). (PDF 782 kb)
Additional file 6: List of orphan genes with links to PFAM (related to Additional file 1: Table S1). (ZIP 1428 kb)
Additional file 7: Protein multiple sequence alignment used for cladogram construction (related to Fig. 3). (NEX 1505 kb)
Additional file 8: FASTA alignments of catalase proteins (related to Fig. 4). (FASTA 157 kb)
