# Supplementary material for: Comparative genomics provides new insights into the diversity, physiology, and sexuality of the only industrially exploited tremellomycete: Phaffia rhodozyma
Source: BMC Genomics. 2016 Nov 9;17:901. doi: 10.1186/s12864-016-3244-7 (PMC5103461; doi:10.1186/s12864-016-3244-7)
Supplement: Additional file 5: Figure S1. — Secondary protein structure features of the homeodomain transcription factors and pheromone receptors of P. rhodozyma. (a) Regions of HD1 and HD2 proteins corresponding to the homeodomain and the three typical helical regions (grey). Comparison of these features was performed with the homeodomain transcription factors of Kwoniella heveanensis (Kwohev) (HD1 - ACZ51528 and HD2 - ACZ51529, respectively). (b) Pheromone receptor proteins highlighting the seven transmembrane regions (green) as predicted by HMMTOP software (http://www.enzim.hu/hmmtop/). (PDF 782 kb) [file 12864_2016_3244_MOESM5_ESM.pdf]

a

HD1

```
Pharho - PSSFRHLRDWFIVHLDHPYPSAQEKEELALARNLTKNSINLWFNNMRRRSGWM
Kwohev - EPDHSLIRIWFLHNISYPYPTVSMKEHLASKAGITRAKVDSDLTNFRRRAGWT
          ...  :* **: ::.:***:.. **.**   .:*: .::  :.*:***:** 
              Helix 1              Helix 2          Helix 3
```

HD2

```
Pharho - SDDEQAPVSVRRSSFTKNTIDTLQALYDLNQYPNPIELKTIAVKVGLQPKQIRAWFQNRRNRRS
Kwohev - VSQAASPR--KLSSFSTRSLLVLDAAYSRSKSLSQAETVIIAQAAGISPHQVRTWFQNKRNRGK
          .:  :*   : ***:..:: .*:* *. .:   .  *   **  .*:.*:*:*:****:*** .
                            Helix 1          Helix 2        Helix 3
```

b

Ste3-1

MKD**AIFPVFASIALLLLVLSCYP**HFRTGN **IGAIALVAWCFASNFVYLV**DC
LIYWDTVENLTPVWCD**IMVKIQATTQTGLAAACLCI**NRRLAIISCSKQTS
ATSKSRRW**AFWSDIMICVLAPVIVAVVSYCV**QSHRYNIVENFGCSGSPWM
DVYAILGLHGSP**VLLGAISFVYGAIAIYNFIA**QRRRFQVVLQQNSSLNTS
RFVR**LIGVAGVNIVISLLFAIRETVLTA**HSVYPTVSWDYIHYDFDLVFTY
DSFFLLGDPQAWVE**LNLSRWLPCVASFIYFAFFG**MHEDMLSYYTYVWARL
SQALLRTKERIFGQPLTVHDPSQYPKLGTAVASPSECGWSQDKISDEEQV
FPHSRTEISEKSNIDEGSLPRFEKICL

Ste3-2

MRD**ALFPIYSGLAILLLLMACGP**HVRARNV**GAISLMTWCMLANITYFINA**
LVYQNTAEDLTPIWCD**VVVKIQCAVQTGLAASCLCI**NRRLATISSPTQSR
QTETSRQW**AFWSDVAICLLPTTLVLIV**SYCVQAHRYNIVENFGCFPATWL
ELY**AILGLFVPPILCAAGSFICGGFAI**YNFLAQRRRFQAVLQQHSSSLNS
SRFLR**LIGVAAVDMVLSLPFGIY**EIIHNSYNLQPTYSWADLHHSFDLVQE
TDQSILNAQPGSWASINLSRWTTT**LAAFIYFAFFGMHEDALSFHASTW**NK
ITAAFSYIWMRAFGTSEPPSYDSSHLTELGTTVASVSDLNRFCKSSKDEE
AYFAYENMIGSDKSDSDKGVDSKISVTVRIQKTIS
