# Supplementary material for: Comparative genomics provides new insights into the diversity, physiology, and sexuality of the only industrially exploited tremellomycete: Phaffia rhodozyma
Source: BMC Genomics. 2016 Nov 9;17:901. doi: 10.1186/s12864-016-3244-7 (PMC5103461; doi:10.1186/s12864-016-3244-7)
Supplement: Additional file 6: — List of orphan genes with links to PFAM (related to Additional file 1: Table S1). (ZIP 1428 kb) [file 12864_2016_3244_MOESM6_ESM.zip › putative_orphan_genes_CBS7918.html]

| # | Gene | Length (AA) | Blast | Domains | Scaffold | Start | End | Strand |
| --- | --- | --- | --- | --- | --- | --- | --- | --- |
| 1 | **G00023\_P** | 1031 | blastp vs nr (<1e-3) | PFAM (0 hits) | scaffold\_001 | 92359 | 95813 | + |
| 2 | **G00082\_P** | 1580 | blastp vs nr (<1e-3) | PFAM (0 hits) | scaffold\_001 | 269795 | 275192 | - |
| 3 | **G00111\_P** | 220 | blastp vs nr (<1e-3) | PFAM (0 hits) | scaffold\_001 | 368685 | 369450 | + |
| 4 | **G00129\_P** | 815 | blastp vs nr (<1e-3) | PFAM (5 hits) | scaffold\_002 | 37385 | 39988 | + |
| 5 | **G00132\_P** | 1036 | blastp vs nr (<1e-3) | PFAM (1 hits) | scaffold\_002 | 43886 | 47668 | - |
| 6 | **G00141\_P** | 364 | blastp vs nr (<1e-3) | PFAM (0 hits) | scaffold\_002 | 67389 | 68562 | - |
| 7 | **G00168\_P** | 453 | blastp vs nr (<1e-3) | PFAM (2 hits) | scaffold\_002 | 166202 | 167727 | - |
| 8 | **G00190\_P** | 563 | blastp vs nr (<1e-3) | PFAM (0 hits) | scaffold\_002 | 233511 | 235321 | + |
| 9 | **G00192\_P** | 412 | blastp vs nr (<1e-3) | PFAM (2 hits) | scaffold\_002 | 240093 | 241846 | + |
| 10 | **G00209\_P** | 590 | blastp vs nr (<1e-3) | PFAM (0 hits) | scaffold\_002 | 291402 | 293532 | + |
| 11 | **G00232\_P** | 217 | blastp vs nr (<1e-3) | PFAM (2 hits) | scaffold\_003 | 16160 | 17263 | - |
| 12 | **G00252\_P** | 1137 | blastp vs nr (<1e-3) | PFAM (2 hits) | scaffold\_003 | 89719 | 93208 | - |
| 13 | **G00297\_P** | 275 | blastp vs nr (<1e-3) | PFAM (0 hits) | scaffold\_003 | 235730 | 237000 | + |
| 14 | **G00304\_P** | 1051 | blastp vs nr (<1e-3) | PFAM (2 hits) | scaffold\_003 | 265737 | 269287 | - |
| 15 | **G00333\_P** | 243 | blastp vs nr (<1e-3) | PFAM (2 hits) | scaffold\_004 | 48692 | 49583 | - |
| 16 | **G00346\_P** | 944 | blastp vs nr (<1e-3) | PFAM (0 hits) | scaffold\_004 | 84362 | 87894 | - |
| 17 | **G00358\_P** | 870 | blastp vs nr (<1e-3) | PFAM (0 hits) | scaffold\_004 | 132657 | 135393 | + |
| 18 | **G00362\_P** | 250 | blastp vs nr (<1e-3) | PFAM (2 hits) | scaffold\_004 | 146172 | 147208 | + |
| 19 | **G00399\_P** | 351 | blastp vs nr (<1e-3) | PFAM (0 hits) | scaffold\_004 | 255876 | 256928 | - |
| 20 | **G00401\_P** | 197 | blastp vs nr (<1e-3) | PFAM (3 hits) | scaffold\_004 | 262416 | 263226 | + |
| 21 | **G00410\_P** | 215 | blastp vs nr (<1e-3) | PFAM (4 hits) | scaffold\_005 | 3940 | 4841 | - |
| 22 | **G00488\_P** | 152 | blastp vs nr (<1e-3) | PFAM (0 hits) | scaffold\_005 | 263204 | 263896 | + |
| 23 | **G00513\_P** | 606 | blastp vs nr (<1e-3) | PFAM (0 hits) | scaffold\_006 | 84968 | 87219 | + |
| 24 | **G00515\_P** | 481 | blastp vs nr (<1e-3) | PFAM (1 hits) | scaffold\_006 | 94201 | 95933 | + |
| 25 | **G00526\_P** | 450 | blastp vs nr (<1e-3) | PFAM (1 hits) | scaffold\_006 | 125697 | 127202 | + |
| 26 | **G00541\_P** | 789 | blastp vs nr (<1e-3) | PFAM (5 hits) | scaffold\_006 | 171056 | 173823 | - |
| 27 | **G00542\_P** | 1003 | blastp vs nr (<1e-3) | PFAM (0 hits) | scaffold\_006 | 174029 | 177349 | + |
| 28 | **G00561\_P** | 352 | blastp vs nr (<1e-3) | PFAM (3 hits) | scaffold\_006 | 235519 | 236574 | - |
| 29 | **G00592\_P** | 171 | blastp vs nr (<1e-3) | PFAM (0 hits) | scaffold\_007 | 53355 | 54388 | - |
| 30 | **G00597\_P** | 297 | blastp vs nr (<1e-3) | PFAM (0 hits) | scaffold\_007 | 69032 | 70283 | + |
| 31 | **G00619\_P** | 692 | blastp vs nr (<1e-3) | PFAM (2 hits) | scaffold\_007 | 131044 | 133410 | - |
| 32 | **G00620\_P** | 76 | blastp vs nr (<1e-3) | PFAM (0 hits) | scaffold\_007 | 133560 | 133887 | + |
| 33 | **G00632\_P** | 420 | blastp vs nr (<1e-3) | PFAM (1 hits) | scaffold\_007 | 173127 | 174881 | - |
| 34 | **G00640\_P** | 824 | blastp vs nr (<1e-3) | PFAM (1 hits) | scaffold\_007 | 205803 | 210494 | + |
| 35 | **G00647\_P** | 961 | blastp vs nr (<1e-3) | PFAM (3 hits) | scaffold\_007 | 226917 | 231632 | + |
| 36 | **G00676\_P** | 311 | blastp vs nr (<1e-3) | PFAM (2 hits) | scaffold\_008 | 68099 | 69169 | - |
| 37 | **G00685\_P** | 439 | blastp vs nr (<1e-3) | PFAM (1 hits) | scaffold\_008 | 98657 | 101267 | - |
| 38 | **G00746\_P** | 336 | blastp vs nr (<1e-3) | PFAM (0 hits) | scaffold\_009 | 31239 | 32246 | + |
| 39 | **G00785\_P** | 497 | blastp vs nr (<1e-3) | PFAM (0 hits) | scaffold\_009 | 131038 | 132709 | + |
| 40 | **G00834\_P** | 508 | blastp vs nr (<1e-3) | PFAM (0 hits) | scaffold\_010 | 19776 | 21299 | + |
| 41 | **G00898\_P** | 436 | blastp vs nr (<1e-3) | PFAM (0 hits) | scaffold\_010 | 199110 | 200417 | + |
| 42 | **G00950\_P** | 364 | blastp vs nr (<1e-3) | PFAM (0 hits) | scaffold\_011 | 91667 | 92911 | - |
| 43 | **G00960\_P** | 86 | blastp vs nr (<1e-3) | PFAM (1 hits) | scaffold\_011 | 119791 | 120328 | - |
| 44 | **G00969\_P** | 99 | blastp vs nr (<1e-3) | PFAM (0 hits) | scaffold\_011 | 143623 | 144001 | + |
| 45 | **G01012\_P** | 1268 | blastp vs nr (<1e-3) | PFAM (30 hits) | scaffold\_012 | 31680 | 35923 | + |
| 46 | **G01021\_P** | 340 | blastp vs nr (<1e-3) | PFAM (0 hits) | scaffold\_012 | 76824 | 78048 | + |
| 47 | **G01076\_P** | 692 | blastp vs nr (<1e-3) | PFAM (3 hits) | scaffold\_013 | 23233 | 25568 | - |
| 48 | **G01103\_P** | 180 | blastp vs nr (<1e-3) | PFAM (0 hits) | scaffold\_013 | 97422 | 98069 | + |
| 49 | **G01111\_P** | 149 | blastp vs nr (<1e-3) | PFAM (9 hits) | scaffold\_013 | 126189 | 126858 | - |
| 50 | **G01128\_P** | 119 | blastp vs nr (<1e-3) | PFAM (5 hits) | scaffold\_013 | 177804 | 178342 | - |
| 51 | **G01150\_P** | 282 | blastp vs nr (<1e-3) | PFAM (15 hits) | scaffold\_014 | 25320 | 26628 | - |
| 52 | **G01167\_P** | 171 | blastp vs nr (<1e-3) | PFAM (1 hits) | scaffold\_014 | 63890 | 64503 | + |
| 53 | **G01183\_P** | 381 | blastp vs nr (<1e-3) | PFAM (2 hits) | scaffold\_014 | 119368 | 120700 | - |
| 54 | **G01194\_P** | 562 | blastp vs nr (<1e-3) | PFAM (0 hits) | scaffold\_014 | 156376 | 158178 | + |
| 55 | **G01200\_P** | 188 | blastp vs nr (<1e-3) | PFAM (5 hits) | scaffold\_014 | 176191 | 176965 | - |
| 56 | **G01210\_P** | 260 | blastp vs nr (<1e-3) | PFAM (63 hits) | scaffold\_014 | 195876 | 196948 | - |
| 57 | **G01242\_P** | 685 | blastp vs nr (<1e-3) | PFAM (6 hits) | scaffold\_015 | 81270 | 83702 | + |
| 58 | **G01261\_P** | 166 | blastp vs nr (<1e-3) | PFAM (2 hits) | scaffold\_015 | 149740 | 150398 | + |
| 59 | **G01289\_P** | 590 | blastp vs nr (<1e-3) | PFAM (9 hits) | scaffold\_016 | 17596 | 19365 | - |
| 60 | **G01299\_P** | 200 | blastp vs nr (<1e-3) | PFAM (0 hits) | scaffold\_016 | 46498 | 47337 | + |
| 61 | **G01377\_P** | 512 | blastp vs nr (<1e-3) | PFAM (2 hits) | scaffold\_017 | 84777 | 86756 | + |
| 62 | **G01395\_P** | 107 | blastp vs nr (<1e-3) | PFAM (0 hits) | scaffold\_017 | 131169 | 131682 | + |
| 63 | **G01428\_P** | 457 | blastp vs nr (<1e-3) | PFAM (3 hits) | scaffold\_018 | 34391 | 36055 | - |
| 64 | **G01486\_P** | 397 | blastp vs nr (<1e-3) | PFAM (0 hits) | scaffold\_019 | 24111 | 25619 | + |
| 65 | **G01488\_P** | 516 | blastp vs nr (<1e-3) | PFAM (3 hits) | scaffold\_019 | 28027 | 29891 | + |
| 66 | **G01493\_P** | 852 | blastp vs nr (<1e-3) | PFAM (0 hits) | scaffold\_019 | 41167 | 44104 | - |
| 67 | **G01507\_P** | 337 | blastp vs nr (<1e-3) | PFAM (4 hits) | scaffold\_019 | 81956 | 83783 | - |
| 68 | **G01521\_P** | 318 | blastp vs nr (<1e-3) | PFAM (3 hits) | scaffold\_019 | 114167 | 115457 | + |
| 69 | **G01533\_P** | 758 | blastp vs nr (<1e-3) | PFAM (0 hits) | scaffold\_019 | 146269 | 148614 | - |
| 70 | **G01559\_P** | 841 | blastp vs nr (<1e-3) | PFAM (0 hits) | scaffold\_020 | 44973 | 47602 | + |
| 71 | **G01612\_P** | 543 | blastp vs nr (<1e-3) | PFAM (0 hits) | scaffold\_021 | 34571 | 36491 | + |
| 72 | **G01638\_P** | 180 | blastp vs nr (<1e-3) | PFAM (0 hits) | scaffold\_021 | 110888 | 111532 | - |
| 73 | **G01645\_P** | 177 | blastp vs nr (<1e-3) | PFAM (0 hits) | scaffold\_021 | 138684 | 139378 | - |
| 74 | **G01663\_P** | 425 | blastp vs nr (<1e-3) | PFAM (3 hits) | scaffold\_022 | 39074 | 40722 | - |
| 75 | **G01670\_P** | 944 | blastp vs nr (<1e-3) | PFAM (14 hits) | scaffold\_022 | 56754 | 60024 | - |
| 76 | **G01676\_P** | 692 | blastp vs nr (<1e-3) | PFAM (0 hits) | scaffold\_022 | 79381 | 81535 | + |
| 77 | **G01702\_P** | 87 | blastp vs nr (<1e-3) | PFAM (1 hits) | scaffold\_022 | 150264 | 150619 | + |
| 78 | **G01725\_P** | 207 | blastp vs nr (<1e-3) | PFAM (8 hits) | scaffold\_023 | 63508 | 64296 | + |
| 79 | **G01736\_P** | 422 | blastp vs nr (<1e-3) | PFAM (2 hits) | scaffold\_023 | 101855 | 103416 | - |
| 80 | **G01744\_P** | 332 | blastp vs nr (<1e-3) | PFAM (0 hits) | scaffold\_023 | 126194 | 127300 | - |
| 81 | **G01756\_P** | 246 | blastp vs nr (<1e-3) | PFAM (6 hits) | scaffold\_024 | 5408 | 6524 | + |
| 82 | **G01771\_P** | 284 | blastp vs nr (<1e-3) | PFAM (5 hits) | scaffold\_024 | 50468 | 51488 | + |
| 83 | **G01789\_P** | 145 | blastp vs nr (<1e-3) | PFAM (1 hits) | scaffold\_024 | 100444 | 101065 | - |
| 84 | **G01806\_P** | 422 | blastp vs nr (<1e-3) | PFAM (4 hits) | scaffold\_025 | 4955 | 6989 | + |
| 85 | **G01819\_P** | 973 | blastp vs nr (<1e-3) | PFAM (0 hits) | scaffold\_025 | 39036 | 41954 | - |
| 86 | **G01831\_P** | 341 | blastp vs nr (<1e-3) | PFAM (2 hits) | scaffold\_025 | 81904 | 82926 | - |
| 87 | **G01838\_P** | 837 | blastp vs nr (<1e-3) | PFAM (5 hits) | scaffold\_025 | 98420 | 101711 | - |
| 88 | **G01859\_P** | 276 | blastp vs nr (<1e-3) | PFAM (2 hits) | scaffold\_026 | 871 | 2080 | + |
| 89 | **G01873\_P** | 198 | blastp vs nr (<1e-3) | PFAM (1 hits) | scaffold\_026 | 52252 | 52915 | - |
| 90 | **G01889\_P** | 582 | blastp vs nr (<1e-3) | PFAM (3 hits) | scaffold\_026 | 107343 | 109259 | - |
| 91 | **G01956\_P** | 434 | blastp vs nr (<1e-3) | PFAM (0 hits) | scaffold\_028 | 31647 | 33294 | - |
| 92 | **G01966\_P** | 447 | blastp vs nr (<1e-3) | PFAM (0 hits) | scaffold\_028 | 58554 | 59966 | - |
| 93 | **G01971\_P** | 163 | blastp vs nr (<1e-3) | PFAM (9 hits) | scaffold\_028 | 71686 | 72295 | + |
| 94 | **G01972\_P** | 301 | blastp vs nr (<1e-3) | PFAM (0 hits) | scaffold\_028 | 72566 | 73898 | - |
| 95 | **G02033\_P** | 163 | blastp vs nr (<1e-3) | PFAM (1 hits) | scaffold\_029 | 108870 | 109736 | - |
| 96 | **G02048\_P** | 117 | blastp vs nr (<1e-3) | PFAM (1 hits) | scaffold\_030 | 16082 | 16628 | + |
| 97 | **G02088\_P** | 253 | blastp vs nr (<1e-3) | PFAM (0 hits) | scaffold\_030 | 132430 | 133686 | - |
| 98 | **G02098\_P** | 728 | blastp vs nr (<1e-3) | PFAM (13 hits) | scaffold\_031 | 25736 | 28391 | + |
| 99 | **G02106\_P** | 556 | blastp vs nr (<1e-3) | PFAM (4 hits) | scaffold\_031 | 49599 | 52668 | + |
| 100 | **G02123\_P** | 394 | blastp vs nr (<1e-3) | PFAM (1 hits) | scaffold\_031 | 101659 | 103321 | - |
| 101 | **G02129\_P** | 585 | blastp vs nr (<1e-3) | PFAM (28 hits) | scaffold\_031 | 118929 | 120683 | + |
| 102 | **G02131\_P** | 143 | blastp vs nr (<1e-3) | PFAM (1 hits) | scaffold\_031 | 125343 | 125882 | + |
| 103 | **G02132\_P** | 298 | blastp vs nr (<1e-3) | PFAM (0 hits) | scaffold\_031 | 126296 | 127910 | + |
| 104 | **G02135\_P** | 55 | blastp vs nr (<1e-3) | PFAM (0 hits) | scaffold\_031 | 135151 | 135410 | - |
| 105 | **G02136\_P** | 299 | blastp vs nr (<1e-3) | PFAM (1 hits) | scaffold\_031 | 135971 | 137035 | + |
| 106 | **G02143\_P** | 429 | blastp vs nr (<1e-3) | PFAM (1 hits) | scaffold\_032 | 13824 | 15264 | + |
| 107 | **G02152\_P** | 99 | blastp vs nr (<1e-3) | PFAM (0 hits) | scaffold\_032 | 38795 | 39500 | + |
| 108 | **G02171\_P** | 314 | blastp vs nr (<1e-3) | PFAM (3 hits) | scaffold\_032 | 103899 | 105110 | - |
| 109 | **G02198\_P** | 217 | blastp vs nr (<1e-3) | PFAM (6 hits) | scaffold\_033 | 67533 | 68264 | + |
| 110 | **G02221\_P** | 483 | blastp vs nr (<1e-3) | PFAM (2 hits) | scaffold\_034 | 2881 | 4479 | + |
| 111 | **G02259\_P** | 136 | blastp vs nr (<1e-3) | PFAM (2 hits) | scaffold\_035 | 4350 | 4757 | + |
| 112 | **G02333\_P** | 612 | blastp vs nr (<1e-3) | PFAM (1 hits) | scaffold\_036 | 105291 | 107574 | - |
| 113 | **G02353\_P** | 282 | blastp vs nr (<1e-3) | PFAM (3 hits) | scaffold\_037 | 30356 | 31612 | + |
| 114 | **G02379\_P** | 361 | blastp vs nr (<1e-3) | PFAM (0 hits) | scaffold\_037 | 119347 | 120716 | - |
| 115 | **G02395\_P** | 327 | blastp vs nr (<1e-3) | PFAM (0 hits) | scaffold\_038 | 59182 | 60761 | - |
| 116 | **G02403\_P** | 1325 | blastp vs nr (<1e-3) | PFAM (2 hits) | scaffold\_038 | 89948 | 94004 | - |
| 117 | **G02408\_P** | 735 | blastp vs nr (<1e-3) | PFAM (4 hits) | scaffold\_038 | 101752 | 104336 | - |
| 118 | **G02416\_P** | 365 | blastp vs nr (<1e-3) | PFAM (15 hits) | scaffold\_039 | 231 | 2108 | - |
| 119 | **G02422\_P** | 657 | blastp vs nr (<1e-3) | PFAM (6 hits) | scaffold\_039 | 16355 | 19823 | - |
| 120 | **G02436\_P** | 371 | blastp vs nr (<1e-3) | PFAM (29 hits) | scaffold\_039 | 62299 | 63861 | + |
| 121 | **G02446\_P** | 217 | blastp vs nr (<1e-3) | PFAM (0 hits) | scaffold\_039 | 93980 | 94814 | - |
| 122 | **G02497\_P** | 360 | blastp vs nr (<1e-3) | PFAM (0 hits) | scaffold\_041 | 11980 | 13347 | - |
| 123 | **G02518\_P** | 101 | blastp vs nr (<1e-3) | PFAM (0 hits) | scaffold\_041 | 74683 | 75099 | + |
| 124 | **G02527\_P** | 930 | blastp vs nr (<1e-3) | PFAM (0 hits) | scaffold\_041 | 114031 | 117123 | + |
| 125 | **G02530\_P** | 419 | blastp vs nr (<1e-3) | PFAM (2 hits) | scaffold\_042 | 1493 | 2819 | + |
| 126 | **G02549\_P** | 233 | blastp vs nr (<1e-3) | PFAM (1 hits) | scaffold\_042 | 55859 | 56696 | + |
| 127 | **G02556\_P** | 462 | blastp vs nr (<1e-3) | PFAM (0 hits) | scaffold\_042 | 72589 | 74459 | + |
| 128 | **G02653\_P** | 224 | blastp vs nr (<1e-3) | PFAM (3 hits) | scaffold\_045 | 45310 | 46404 | - |
| 129 | **G02662\_P** | 601 | blastp vs nr (<1e-3) | PFAM (3 hits) | scaffold\_045 | 71302 | 73734 | - |
| 130 | **G02713\_P** | 974 | blastp vs nr (<1e-3) | PFAM (6 hits) | scaffold\_047 | 20496 | 23530 | - |
| 131 | **G02748\_P** | 505 | blastp vs nr (<1e-3) | PFAM (0 hits) | scaffold\_048 | 43356 | 44977 | - |
| 132 | **G02751\_P** | 526 | blastp vs nr (<1e-3) | PFAM (1 hits) | scaffold\_048 | 52300 | 53877 | + |
| 133 | **G02767\_P** | 1031 | blastp vs nr (<1e-3) | PFAM (16 hits) | scaffold\_048 | 100656 | 103894 | + |
| 134 | **G02779\_P** | 741 | blastp vs nr (<1e-3) | PFAM (7 hits) | scaffold\_049 | 45339 | 47765 | - |
| 135 | **G02822\_P** | 1056 | blastp vs nr (<1e-3) | PFAM (2 hits) | scaffold\_050 | 98728 | 101895 | - |
| 136 | **G02824\_P** | 377 | blastp vs nr (<1e-3) | PFAM (22 hits) | scaffold\_050 | 105442 | 107383 | - |
| 137 | **G02844\_P** | 588 | blastp vs nr (<1e-3) | PFAM (8 hits) | scaffold\_051 | 65149 | 67341 | - |
| 138 | **G02845\_P** | 403 | blastp vs nr (<1e-3) | PFAM (0 hits) | scaffold\_051 | 67772 | 69165 | - |
| 139 | **G02859\_P** | 138 | blastp vs nr (<1e-3) | PFAM (8 hits) | scaffold\_051 | 105453 | 105866 | - |
| 140 | **G02866\_P** | 438 | blastp vs nr (<1e-3) | PFAM (18 hits) | scaffold\_052 | 21062 | 23041 | - |
| 141 | **G02875\_P** | 477 | blastp vs nr (<1e-3) | PFAM (0 hits) | scaffold\_052 | 42363 | 43899 | + |
| 142 | **G02893\_P** | 126 | blastp vs nr (<1e-3) | PFAM (2 hits) | scaffold\_053 | 1119 | 1576 | - |
| 143 | **G02906\_P** | 1146 | blastp vs nr (<1e-3) | PFAM (7 hits) | scaffold\_053 | 37292 | 40812 | + |
| 144 | **G02913\_P** | 285 | blastp vs nr (<1e-3) | PFAM (1 hits) | scaffold\_053 | 62926 | 64244 | - |
| 145 | **G02917\_P** | 413 | blastp vs nr (<1e-3) | PFAM (1 hits) | scaffold\_053 | 73699 | 75162 | - |
| 146 | **G02994\_P** | 89 | blastp vs nr (<1e-3) | PFAM (4 hits) | scaffold\_056 | 10058 | 10536 | - |
| 147 | **G03005\_P** | 394 | blastp vs nr (<1e-3) | PFAM (5 hits) | scaffold\_056 | 44077 | 45447 | + |
| 148 | **G03030\_P** | 384 | blastp vs nr (<1e-3) | PFAM (3 hits) | scaffold\_057 | 24960 | 26205 | + |
| 149 | **G03036\_P** | 757 | blastp vs nr (<1e-3) | PFAM (21 hits) | scaffold\_057 | 48330 | 50755 | - |
| 150 | **G03095\_P** | 687 | blastp vs nr (<1e-3) | PFAM (1 hits) | scaffold\_059 | 25613 | 28362 | - |
| 151 | **G03136\_P** | 456 | blastp vs nr (<1e-3) | PFAM (0 hits) | scaffold\_060 | 46661 | 48429 | - |
| 152 | **G03137\_P** | 223 | blastp vs nr (<1e-3) | PFAM (0 hits) | scaffold\_060 | 52308 | 53273 | - |
| 153 | **G03142\_P** | 1219 | blastp vs nr (<1e-3) | PFAM (0 hits) | scaffold\_060 | 73538 | 77808 | + |
| 154 | **G03270\_P** | 708 | blastp vs nr (<1e-3) | PFAM (4 hits) | scaffold\_065 | 27572 | 29912 | - |
| 155 | **G03273\_P** | 893 | blastp vs nr (<1e-3) | PFAM (39 hits) | scaffold\_065 | 36808 | 39763 | + |
| 156 | **G03278\_P** | 182 | blastp vs nr (<1e-3) | PFAM (0 hits) | scaffold\_065 | 52489 | 53247 | + |
| 157 | **G03324\_P** | 90 | blastp vs nr (<1e-3) | PFAM (1 hits) | scaffold\_067 | 34808 | 35154 | + |
| 158 | **G03335\_P** | 117 | blastp vs nr (<1e-3) | PFAM (12 hits) | scaffold\_067 | 63362 | 63828 | - |
| 159 | **G03402\_P** | 124 | blastp vs nr (<1e-3) | PFAM (0 hits) | scaffold\_070 | 847 | 1318 | + |
| 160 | **G03444\_P** | 973 | blastp vs nr (<1e-3) | PFAM (1 hits) | scaffold\_071 | 42328 | 45501 | - |
| 161 | **G03462\_P** | 366 | blastp vs nr (<1e-3) | PFAM (3 hits) | scaffold\_072 | 14894 | 15991 | + |
| 162 | **G03468\_P** | 560 | blastp vs nr (<1e-3) | PFAM (1 hits) | scaffold\_072 | 46699 | 48772 | - |
| 163 | **G03481\_P** | 777 | blastp vs nr (<1e-3) | PFAM (0 hits) | scaffold\_073 | 1062 | 3619 | + |
| 164 | **G03552\_P** | 671 | blastp vs nr (<1e-3) | PFAM (4 hits) | scaffold\_076 | 181 | 2279 | + |
| 165 | **G03566\_P** | 92 | blastp vs nr (<1e-3) | PFAM (9 hits) | scaffold\_076 | 38287 | 38651 | + |
| 166 | **G03591\_P** | 198 | blastp vs nr (<1e-3) | PFAM (1 hits) | scaffold\_077 | 26048 | 26816 | - |
| 167 | **G03610\_P** | 540 | blastp vs nr (<1e-3) | PFAM (20 hits) | scaffold\_078 | 5643 | 8510 | + |
| 168 | **G03613\_P** | 482 | blastp vs nr (<1e-3) | PFAM (0 hits) | scaffold\_078 | 18083 | 19824 | - |
| 169 | **G03616\_P** | 356 | blastp vs nr (<1e-3) | PFAM (2 hits) | scaffold\_078 | 24189 | 25486 | - |
| 170 | **G03621\_P** | 287 | blastp vs nr (<1e-3) | PFAM (2 hits) | scaffold\_078 | 35233 | 36093 | + |
| 171 | **G03680\_P** | 99 | blastp vs nr (<1e-3) | PFAM (0 hits) | scaffold\_081 | 522 | 899 | - |
| 172 | **G03713\_P** | 539 | blastp vs nr (<1e-3) | PFAM (9 hits) | scaffold\_082 | 47752 | 49570 | - |
| 173 | **G03726\_P** | 196 | blastp vs nr (<1e-3) | PFAM (3 hits) | scaffold\_083 | 21928 | 23550 | + |
| 174 | **G03747\_P** | 641 | blastp vs nr (<1e-3) | PFAM (0 hits) | scaffold\_084 | 20290 | 22746 | + |
| 175 | **G03761\_P** | 613 | blastp vs nr (<1e-3) | PFAM (3 hits) | scaffold\_084 | 65198 | 67126 | + |
| 176 | **G03783\_P** | 1269 | blastp vs nr (<1e-3) | PFAM (2 hits) | scaffold\_086 | 8417 | 13139 | - |
| 177 | **G03789\_P** | 381 | blastp vs nr (<1e-3) | PFAM (0 hits) | scaffold\_086 | 24244 | 25697 | + |
| 178 | **G03801\_P** | 848 | blastp vs nr (<1e-3) | PFAM (57 hits) | scaffold\_086 | 62676 | 65314 | + |
| 179 | **G03814\_P** | 495 | blastp vs nr (<1e-3) | PFAM (18 hits) | scaffold\_087 | 29740 | 31330 | - |
| 180 | **G03815\_P** | 528 | blastp vs nr (<1e-3) | PFAM (0 hits) | scaffold\_087 | 31780 | 33959 | - |
| 181 | **G03818\_P** | 380 | blastp vs nr (<1e-3) | PFAM (4 hits) | scaffold\_087 | 43998 | 45232 | + |
| 182 | **G03825\_P** | 399 | blastp vs nr (<1e-3) | PFAM (2 hits) | scaffold\_087 | 61877 | 63302 | + |
| 183 | **G03833\_P** | 147 | blastp vs nr (<1e-3) | PFAM (1 hits) | scaffold\_088 | 16862 | 17540 | + |
| 184 | **G03849\_P** | 336 | blastp vs nr (<1e-3) | PFAM (2 hits) | scaffold\_088 | 57200 | 58294 | + |
| 185 | **G03875\_P** | 76 | blastp vs nr (<1e-3) | PFAM (2 hits) | scaffold\_090 | 9738 | 10136 | - |
| 186 | **G03952\_P** | 1204 | blastp vs nr (<1e-3) | PFAM (4 hits) | scaffold\_093 | 17215 | 21279 | - |
| 187 | **G03963\_P** | 977 | blastp vs nr (<1e-3) | PFAM (7 hits) | scaffold\_093 | 59863 | 63167 | - |
| 188 | **G03980\_P** | 283 | blastp vs nr (<1e-3) | PFAM (3 hits) | scaffold\_094 | 55300 | 56335 | + |
| 189 | **G04008\_P** | 880 | blastp vs nr (<1e-3) | PFAM (0 hits) | scaffold\_096 | 19403 | 22291 | - |
| 190 | **G04010\_P** | 256 | blastp vs nr (<1e-3) | PFAM (4 hits) | scaffold\_096 | 31172 | 32604 | - |
| 191 | **G04018\_P** | 321 | blastp vs nr (<1e-3) | PFAM (0 hits) | scaffold\_096 | 47286 | 48643 | + |
| 192 | **G04031\_P** | 554 | blastp vs nr (<1e-3) | PFAM (22 hits) | scaffold\_097 | 14910 | 17157 | - |
| 193 | **G04047\_P** | 312 | blastp vs nr (<1e-3) | PFAM (3 hits) | scaffold\_098 | 8921 | 10173 | - |
| 194 | **G04056\_P** | 258 | blastp vs nr (<1e-3) | PFAM (1 hits) | scaffold\_098 | 48335 | 49544 | + |
| 195 | **G04057\_P** | 316 | blastp vs nr (<1e-3) | PFAM (11 hits) | scaffold\_098 | 49849 | 51033 | + |
| 196 | **G04061\_P** | 235 | blastp vs nr (<1e-3) | PFAM (4 hits) | scaffold\_098 | 59885 | 60674 | + |
| 197 | **G04102\_P** | 89 | blastp vs nr (<1e-3) | PFAM (0 hits) | scaffold\_100 | 56468 | 56826 | + |
| 198 | **G04110\_P** | 851 | blastp vs nr (<1e-3) | PFAM (2 hits) | scaffold\_101 | 18683 | 21307 | + |
| 199 | **G04122\_P** | 626 | blastp vs nr (<1e-3) | PFAM (0 hits) | scaffold\_101 | 56938 | 58912 | + |
| 200 | **G04221\_P** | 131 | blastp vs nr (<1e-3) | PFAM (2 hits) | scaffold\_106 | 53613 | 54391 | - |
| 201 | **G04250\_P** | 390 | blastp vs nr (<1e-3) | PFAM (2 hits) | scaffold\_108 | 39507 | 41066 | + |
| 202 | **G04263\_P** | 453 | blastp vs nr (<1e-3) | PFAM (5 hits) | scaffold\_109 | 26804 | 28512 | - |
| 203 | **G04269\_P** | 1110 | blastp vs nr (<1e-3) | PFAM (2 hits) | scaffold\_109 | 44687 | 48473 | - |
| 204 | **G04279\_P** | 289 | blastp vs nr (<1e-3) | PFAM (3 hits) | scaffold\_110 | 18968 | 20301 | + |
| 205 | **G04292\_P** | 424 | blastp vs nr (<1e-3) | PFAM (5 hits) | scaffold\_110 | 48347 | 49859 | - |
| 206 | **G04325\_P** | 427 | blastp vs nr (<1e-3) | PFAM (2 hits) | scaffold\_112 | 39237 | 40738 | + |
| 207 | **G04332\_P** | 387 | blastp vs nr (<1e-3) | PFAM (13 hits) | scaffold\_113 | 3639 | 5197 | + |
| 208 | **G04363\_P** | 217 | blastp vs nr (<1e-3) | PFAM (4 hits) | scaffold\_114 | 43625 | 44363 | - |
| 209 | **G04373\_P** | 126 | blastp vs nr (<1e-3) | PFAM (1 hits) | scaffold\_115 | 24463 | 24947 | + |
| 210 | **G04434\_P** | 324 | blastp vs nr (<1e-3) | PFAM (0 hits) | scaffold\_119 | 17768 | 18836 | + |
| 211 | **G04446\_P** | 129 | blastp vs nr (<1e-3) | PFAM (1 hits) | scaffold\_119 | 50204 | 50683 | + |
| 212 | **G04472\_P** | 546 | blastp vs nr (<1e-3) | PFAM (10 hits) | scaffold\_121 | 17868 | 20529 | - |
| 213 | **G04485\_P** | 163 | blastp vs nr (<1e-3) | PFAM (2 hits) | scaffold\_121 | 50057 | 51289 | - |
| 214 | **G04494\_P** | 123 | blastp vs nr (<1e-3) | PFAM (0 hits) | scaffold\_122 | 24281 | 24821 | + |
| 215 | **G04543\_P** | 286 | blastp vs nr (<1e-3) | PFAM (2 hits) | scaffold\_125 | 21084 | 22326 | + |
| 216 | **G04555\_P** | 398 | blastp vs nr (<1e-3) | PFAM (1 hits) | scaffold\_126 | 27585 | 29216 | + |
| 217 | **G04558\_P** | 577 | blastp vs nr (<1e-3) | PFAM (6 hits) | scaffold\_126 | 35397 | 37352 | + |
| 218 | **G04559\_P** | 440 | blastp vs nr (<1e-3) | PFAM (0 hits) | scaffold\_126 | 37961 | 39388 | - |
| 219 | **G04564\_P** | 382 | blastp vs nr (<1e-3) | PFAM (0 hits) | scaffold\_127 | 497 | 2150 | - |
| 220 | **G04581\_P** | 307 | blastp vs nr (<1e-3) | PFAM (1 hits) | scaffold\_128 | 9818 | 10738 | + |
| 221 | **G04588\_P** | 219 | blastp vs nr (<1e-3) | PFAM (4 hits) | scaffold\_128 | 30552 | 31717 | + |
| 222 | **G04612\_P** | 490 | blastp vs nr (<1e-3) | PFAM (7 hits) | scaffold\_130 | 30282 | 32034 | + |
| 223 | **G04670\_P** | 144 | blastp vs nr (<1e-3) | PFAM (1 hits) | scaffold\_134 | 7499 | 8224 | - |
| 224 | **G04674\_P** | 266 | blastp vs nr (<1e-3) | PFAM (1 hits) | scaffold\_134 | 16504 | 17301 | - |
| 225 | **G04721\_P** | 362 | blastp vs nr (<1e-3) | PFAM (1 hits) | scaffold\_137 | 10832 | 12388 | + |
| 226 | **G04771\_P** | 93 | blastp vs nr (<1e-3) | PFAM (6 hits) | scaffold\_140 | 24275 | 24661 | + |
| 227 | **G04790\_P** | 172 | blastp vs nr (<1e-3) | PFAM (0 hits) | scaffold\_141 | 40762 | 41520 | + |
| 228 | **G04795\_P** | 445 | blastp vs nr (<1e-3) | PFAM (0 hits) | scaffold\_142 | 8216 | 9807 | + |
| 229 | **G04803\_P** | 645 | blastp vs nr (<1e-3) | PFAM (0 hits) | scaffold\_142 | 28273 | 30308 | - |
| 230 | **G04818\_P** | 403 | blastp vs nr (<1e-3) | PFAM (2 hits) | scaffold\_143 | 31563 | 32981 | + |
| 231 | **G04829\_P** | 795 | blastp vs nr (<1e-3) | PFAM (0 hits) | scaffold\_144 | 17633 | 20231 | + |
| 232 | **G04880\_P** | 98 | blastp vs nr (<1e-3) | PFAM (0 hits) | scaffold\_148 | 13646 | 14084 | - |
| 233 | **G04912\_P** | 666 | blastp vs nr (<1e-3) | PFAM (0 hits) | scaffold\_150 | 26566 | 28563 | + |
| 234 | **G04932\_P** | 583 | blastp vs nr (<1e-3) | PFAM (3 hits) | scaffold\_152 | 355 | 2103 | - |
| 235 | **G04938\_P** | 220 | blastp vs nr (<1e-3) | PFAM (8 hits) | scaffold\_152 | 14335 | 15189 | + |
| 236 | **G04975\_P** | 446 | blastp vs nr (<1e-3) | PFAM (2 hits) | scaffold\_155 | 7744 | 9659 | + |
| 237 | **G04982\_P** | 531 | blastp vs nr (<1e-3) | PFAM (0 hits) | scaffold\_155 | 24996 | 26588 | + |
| 238 | **G04983\_P** | 538 | blastp vs nr (<1e-3) | PFAM (0 hits) | scaffold\_155 | 28010 | 29732 | - |
| 239 | **G04985\_P** | 608 | blastp vs nr (<1e-3) | PFAM (1 hits) | scaffold\_155 | 33704 | 35944 | - |
| 240 | **G05010\_P** | 171 | blastp vs nr (<1e-3) | PFAM (1 hits) | scaffold\_157 | 26415 | 27328 | - |
| 241 | **G05020\_P** | 228 | blastp vs nr (<1e-3) | PFAM (0 hits) | scaffold\_158 | 19768 | 20742 | + |
| 242 | **G05071\_P** | 522 | blastp vs nr (<1e-3) | PFAM (0 hits) | scaffold\_162 | 32748 | 34384 | + |
| 243 | **G05118\_P** | 212 | blastp vs nr (<1e-3) | PFAM (42 hits) | scaffold\_166 | 28540 | 29649 | + |
| 244 | **G05132\_P** | 253 | blastp vs nr (<1e-3) | PFAM (0 hits) | scaffold\_168 | 1222 | 2190 | + |
| 245 | **G05133\_P** | 200 | blastp vs nr (<1e-3) | PFAM (6 hits) | scaffold\_168 | 3567 | 4369 | - |
| 246 | **G05174\_P** | 240 | blastp vs nr (<1e-3) | PFAM (2 hits) | scaffold\_171 | 5854 | 7185 | - |
| 247 | **G05176\_P** | 771 | blastp vs nr (<1e-3) | PFAM (1 hits) | scaffold\_171 | 9047 | 11998 | - |
| 248 | **G05204\_P** | 363 | blastp vs nr (<1e-3) | PFAM (7 hits) | scaffold\_173 | 34620 | 35965 | + |
| 249 | **G05213\_P** | 454 | blastp vs nr (<1e-3) | PFAM (6 hits) | scaffold\_174 | 28560 | 30107 | - |
| 250 | **G05227\_P** | 173 | blastp vs nr (<1e-3) | PFAM (3 hits) | scaffold\_175 | 30055 | 30640 | - |
| 251 | **G05235\_P** | 162 | blastp vs nr (<1e-3) | PFAM (4 hits) | scaffold\_176 | 18910 | 19492 | - |
| 252 | **G05237\_P** | 491 | blastp vs nr (<1e-3) | PFAM (1 hits) | scaffold\_176 | 25022 | 26705 | + |
| 253 | **G05272\_P** | 143 | blastp vs nr (<1e-3) | PFAM (2 hits) | scaffold\_180 | 2839 | 3360 | - |
| 254 | **G05295\_P** | 247 | blastp vs nr (<1e-3) | PFAM (0 hits) | scaffold\_182 | 12761 | 14068 | - |
| 255 | **G05304\_P** | 86 | blastp vs nr (<1e-3) | PFAM (0 hits) | scaffold\_183 | 9438 | 9785 | + |
| 256 | **G05355\_P** | 68 | blastp vs nr (<1e-3) | PFAM (31 hits) | scaffold\_187 | 19401 | 19801 | + |
| 257 | **G05366\_P** | 377 | blastp vs nr (<1e-3) | PFAM (3 hits) | scaffold\_188 | 20749 | 21984 | - |
| 258 | **G05374\_P** | 123 | blastp vs nr (<1e-3) | PFAM (1 hits) | scaffold\_189 | 11952 | 12619 | + |
| 259 | **G05391\_P** | 458 | blastp vs nr (<1e-3) | PFAM (1 hits) | scaffold\_190 | 21016 | 22389 | + |
| 260 | **G05400\_P** | 789 | blastp vs nr (<1e-3) | PFAM (0 hits) | scaffold\_191 | 12827 | 15252 | - |
| 261 | **G05441\_P** | 63 | blastp vs nr (<1e-3) | PFAM (0 hits) | scaffold\_195 | 19674 | 19953 | - |
| 262 | **G05467\_P** | 127 | blastp vs nr (<1e-3) | PFAM (7 hits) | scaffold\_198 | 3192 | 3572 | + |
| 263 | **G05470\_P** | 333 | blastp vs nr (<1e-3) | PFAM (3 hits) | scaffold\_198 | 12511 | 13771 | - |
| 264 | **G05502\_P** | 167 | blastp vs nr (<1e-3) | PFAM (2 hits) | scaffold\_202 | 703 | 1495 | + |
| 265 | **G05503\_P** | 295 | blastp vs nr (<1e-3) | PFAM (1 hits) | scaffold\_202 | 1995 | 3575 | - |
| 266 | **G05523\_P** | 243 | blastp vs nr (<1e-3) | PFAM (0 hits) | scaffold\_204 | 23751 | 24791 | - |
| 267 | **G05542\_P** | 348 | blastp vs nr (<1e-3) | PFAM (2 hits) | scaffold\_206 | 21498 | 23159 | + |
| 268 | **G05553\_P** | 205 | blastp vs nr (<1e-3) | PFAM (2 hits) | scaffold\_208 | 18305 | 18919 | + |
| 269 | **G05573\_P** | 410 | blastp vs nr (<1e-3) | PFAM (0 hits) | scaffold\_211 | 693 | 2444 | + |
| 270 | **G05580\_P** | 136 | blastp vs nr (<1e-3) | PFAM (0 hits) | scaffold\_212 | 1364 | 2038 | + |
| 271 | **G05584\_P** | 143 | blastp vs nr (<1e-3) | PFAM (9 hits) | scaffold\_212 | 12553 | 13083 | + |
| 272 | **G05590\_P** | 106 | blastp vs nr (<1e-3) | PFAM (175 hits) | scaffold\_213 | 5534 | 6382 | + |
| 273 | **G05668\_P** | 157 | blastp vs nr (<1e-3) | PFAM (4 hits) | scaffold\_224 | 16467 | 17651 | - |
| 274 | **G05688\_P** | 570 | blastp vs nr (<1e-3) | PFAM (10 hits) | scaffold\_228 | 3343 | 5432 | + |
| 275 | **G05766\_P** | 944 | blastp vs nr (<1e-3) | PFAM (0 hits) | scaffold\_244 | 5780 | 8760 | + |
| 276 | **G05788\_P** | 315 | blastp vs nr (<1e-3) | PFAM (1 hits) | scaffold\_250 | 6544 | 8186 | - |
| 277 | **G05803\_P** | 188 | blastp vs nr (<1e-3) | PFAM (0 hits) | scaffold\_254 | 6020 | 6583 | - |
| 278 | **G05849\_P** | 103 | blastp vs nr (<1e-3) | PFAM (2 hits) | scaffold\_268 | 2484 | 2899 | + |
| 279 | **G05867\_P** | 560 | blastp vs nr (<1e-3) | PFAM (5 hits) | scaffold\_274 | 4299 | 6214 | + |
| 280 | **G05909\_P** | 381 | blastp vs nr (<1e-3) | PFAM (0 hits) | scaffold\_296 | 1196 | 2494 | - |
| 281 | **G05949\_P** | 473 | blastp vs nr (<1e-3) | PFAM (1 hits) | scaffold\_321 | 1636 | 3670 | + |
| 282 | **G05950\_P** | 739 | blastp vs nr (<1e-3) | PFAM (2 hits) | scaffold\_322 | 218 | 2833 | - |
| 283 | **G05972\_P** | 119 | blastp vs nr (<1e-3) | PFAM (0 hits) | scaffold\_337 | 33 | 754 | - |
