# Supplementary material for: Comparative genomics provides new insights into the diversity, physiology, and sexuality of the only industrially exploited tremellomycete: Phaffia rhodozyma
Source: BMC Genomics. 2016 Nov 9;17:901. doi: 10.1186/s12864-016-3244-7 (PMC5103461; doi:10.1186/s12864-016-3244-7)
Supplement: Additional file 6: — List of orphan genes with links to PFAM (related to Additional file 1: Table S1). (ZIP 1428 kb) [file 12864_2016_3244_MOESM6_ESM.zip › BLAST_HTML_FTR/G00111_P.html]

BLAST Search Results


```
BLASTP 2.2.27+


Reference:
Stephen F. Altschul, Thomas L. Madden, Alejandro A. Schäffer,
Jinghui Zhang, Zheng Zhang, Webb Miller, and David J. Lipman (1997),
"Gapped BLAST and PSI-BLAST: a new generation of protein database
search programs", Nucleic Acids Res. 25:3389-3402.


Reference for
composition-based statistics:
Alejandro A. Schäffer, L. Aravind, Thomas L. Madden, Sergei
Shavirin, John L. Spouge, Yuri I. Wolf, Eugene V. Koonin, and
Stephen F. Altschul (2001), "Improving the accuracy of PSI-BLAST
protein database searches with composition-based statistics and
other refinements", Nucleic Acids Res. 29:2994-3005.


Database: nr
           71,551,133 sequences; 26,053,659,533 total letters


Query= G00111_P

Length=220
                                                                      Score     E
Sequences producing significant alignments:                          (Bits)  Value

emb|CED84242.1|  hypothetical protein [Xanthophyllomyces dendrorh...   402    6e-140
ref|XP_003605528.1|  50S ribosomal protein L18 family protein [Me...  37.4    7.6   
ref|WP_036611491.1|  transcriptional regulator [Paenibacillus sp....  37.4    7.8   


 >emb|CED84242.1| hypothetical protein [Xanthophyllomyces dendrorhous]
Length=200

 Score =  402 bits (1033),  Expect = 6e-140, Method: Compositional matrix adjust.
 Identities = 198/198 (100%), Positives = 198/198 (100%), Gaps = 0/198 (0%)

Query  1    MSSSSCLPSPPPVSEPMHLEPYQKQYIGDGWTSVYSKNQPEDYLTSRMSTLSSNSIEGDH  60
            MSSSSCLPSPPPVSEPMHLEPYQKQYIGDGWTSVYSKNQPEDYLTSRMSTLSSNSIEGDH
Sbjct  1    MSSSSCLPSPPPVSEPMHLEPYQKQYIGDGWTSVYSKNQPEDYLTSRMSTLSSNSIEGDH  60

Query  61   IRPPLPTPTASEAGDDPSEEDPLADFTLILPRQPRDFMLPPHSATSFIPTDRSKHRSFSV  120
            IRPPLPTPTASEAGDDPSEEDPLADFTLILPRQPRDFMLPPHSATSFIPTDRSKHRSFSV
Sbjct  61   IRPPLPTPTASEAGDDPSEEDPLADFTLILPRQPRDFMLPPHSATSFIPTDRSKHRSFSV  120

Query  121  STAVLSSAATSRRPSLFSPLEEEARPVFGLSKAERTYSADMYKLLEEGLKMLKARQFQEN  180
            STAVLSSAATSRRPSLFSPLEEEARPVFGLSKAERTYSADMYKLLEEGLKMLKARQFQEN
Sbjct  121  STAVLSSAATSRRPSLFSPLEEEARPVFGLSKAERTYSADMYKLLEEGLKMLKARQFQEN  180

Query  181  KVKGKIGGMMIIRTSKPK  198
            KVKGKIGGMMIIRTSKPK
Sbjct  181  KVKGKIGGMMIIRTSKPK  198


>ref|XP_003605528.1| 50S ribosomal protein L18 family protein [Medicago truncatula]
 gb|AES87725.1| 50S ribosomal protein L18 family protein [Medicago truncatula]
Length=199

 Score = 37.4 bits (85),  Expect = 7.6, Method: Compositional matrix adjust.
 Identities = 42/152 (28%), Positives = 69/152 (45%), Gaps = 15/152 (10%)

Query  45   TSRMSTLSSNSIEGDHIRPPLPTPTASEAGDDPSEEDPLADFTLILPRQPRDFMLPPHSA  104
            T+ +S  +SN+ +G  IR      +  EA   PS +       + +    R F+L    +
Sbjct  56   TTTLSPSNSNNPKGTFIRAAWTRRSRGEAEKKPSRKSWKRRTDMYM----RPFLLDIFFS  111

Query  105  TSFIPTDRSKHRSFSVSTAVLSSAATSRRPSLFSPLEEEARPVFGLSKAERTYSADMYKL  164
              F+   +  HR  S    V ++ A   R SL S ++ EA  V G   AER+  AD+Y +
Sbjct  112  KKFVHA-KVTHRGTSKVICVATTNAKDLRNSLPSLIDPEACRVIGRLIAERSKEADVYAM  170

Query  165  LEEGLKMLKARQFQENKVKGKIGGMMIIRTSK  196
              E  K        + +++G++G  +II T K
Sbjct  171  AYEPRK--------KERIEGRLG--IIIDTVK  192


>ref|WP_036611491.1| transcriptional regulator [Paenibacillus sp. FSL H7-689]
 gb|ETT50488.1| hypothetical protein C170_16100 [Paenibacillus sp. FSL H7-689]
Length=305

 Score = 37.4 bits (85),  Expect = 7.8, Method: Compositional matrix adjust.
 Identities = 23/74 (31%), Positives = 36/74 (49%), Gaps = 3/74 (4%)

Query  16   PMHLEPYQKQYIGDGWTSVYSKNQP-EDYLTSRMSTLSSNSIEGDHIRPPLPTPTASEAG  74
            P HLEP +  +    W  VY   Q  +DY   R+S + +  +  +H +P L  P  +  G
Sbjct  155  PRHLEPVRLHFKYRNWY-VYGFCQTRQDYREFRLSRMMNVQLTSEHFQPHLELPQET-VG  212

Query  75   DDPSEEDPLADFTL  88
             DPS +D ++D   
Sbjct  213  SDPSWQDQVSDVVF  226


Lambda      K        H        a         alpha
   0.316    0.132    0.387    0.792     4.96 

Gapped
Lambda      K        H        a         alpha    sigma
   0.267   0.0410    0.140     1.90     42.6     43.6 

Effective search space used: 1175905893600


  Database: nr
    Posted date:  Sep 23, 2015 12:05 AM
  Number of letters in database: 26,053,659,533
  Number of sequences in database:  71,551,133


Matrix: BLOSUM62
Gap Penalties: Existence: 11, Extension: 1
Neighboring words threshold: 11
Window for multiple hits: 40
```
