# Supplementary material for: Comparative genomics provides new insights into the diversity, physiology, and sexuality of the only industrially exploited tremellomycete: Phaffia rhodozyma
Source: BMC Genomics. 2016 Nov 9;17:901. doi: 10.1186/s12864-016-3244-7 (PMC5103461; doi:10.1186/s12864-016-3244-7)
Supplement: Additional file 6: — List of orphan genes with links to PFAM (related to Additional file 1: Table S1). (ZIP 1428 kb) [file 12864_2016_3244_MOESM6_ESM.zip › BLAST_HTML_FTR/G01670_P.html]

BLAST Search Results


```
BLASTP 2.2.27+


Reference:
Stephen F. Altschul, Thomas L. Madden, Alejandro A. Schäffer,
Jinghui Zhang, Zheng Zhang, Webb Miller, and David J. Lipman (1997),
"Gapped BLAST and PSI-BLAST: a new generation of protein database
search programs", Nucleic Acids Res. 25:3389-3402.


Reference for
composition-based statistics:
Alejandro A. Schäffer, L. Aravind, Thomas L. Madden, Sergei
Shavirin, John L. Spouge, Yuri I. Wolf, Eugene V. Koonin, and
Stephen F. Altschul (2001), "Improving the accuracy of PSI-BLAST
protein database searches with composition-based statistics and
other refinements", Nucleic Acids Res. 29:2994-3005.


Database: nr
           71,551,133 sequences; 26,053,659,533 total letters


Query= G01670_P

Length=944
                                                                      Score     E
Sequences producing significant alignments:                          (Bits)  Value

emb|CDZ97256.1|  Shugoshin, C-terminal [Xanthophyllomyces dendror...  1489    0.0  
gb|KIJ31844.1|  hypothetical protein M422DRAFT_36101 [Sphaerobolu...  44.7    0.55 
gb|KIJ98039.1|  hypothetical protein K443DRAFT_681088 [Laccaria a...  40.8    7.9  
gb|KIJ63388.1|  hypothetical protein HYDPIDRAFT_29636 [Hydnomerul...  40.8    8.6  


 >emb|CDZ97256.1| Shugoshin, C-terminal [Xanthophyllomyces dendrorhous]
Length=892

 Score = 1489 bits (3856),  Expect = 0.0, Method: Compositional matrix adjust.
 Identities = 880/915 (96%), Positives = 880/915 (96%), Gaps = 35/915 (4%)

Query  1    MPSPSKVGAKKPRYLIEVERLRERLHGITLKHNDVVINNVAHRQKIKELENEVYDLRDEV  60
            MPSPSKVGAKKPRYLIEVERLRERLHGITLKHNDVVINNVAHRQKIKELENEVYDLRDEV
Sbjct  1    MPSPSKVGAKKPRYLIEVERLRERLHGITLKHNDVVINNVAHRQKIKELENEVYDLRDEV  60

Query  61   IRLSERNIRLEAEVGRLRKVEFKSKQDRNGSLQNFLNKEEDAKILLHVLSTLIVSAPALT  120
            IRLSERNIRLEAEVGRLRKVEFKSKQDRNGSLQNFLNKEEDAKILLHVLSTLIVSAPALT
Sbjct  61   IRLSERNIRLEAEVGRLRKVEFKSKQDRNGSLQNFLNKEEDAKILLHVLSTLIVSAPALT  120

Query  121  VLRDEILSNVAPSPPSSTSPLLNRSALPTPLSPHQPFAYWQPKLNPQGTVKASASPVGID  180
            VLRDEILSNVAPSPPSSTSPLLNRSALPTPLSPHQPFAYWQPKLNPQGTVKASASPVGID
Sbjct  121  VLRDEILSNVAPSPPSSTSPLLNRSALPTPLSPHQPFAYWQPKLNPQGTVKASASPVGID  180

Query  181  NTLKEEDEPSEDFAQPGSLYMALNQQTVEPPSSSTSLSSIASSSSSRSLSQEAYQPLNPP  240
            NTLKEEDEPSEDFAQPGSLYMALNQQTVEPPSSSTSLSSIASSSSSRSLSQEAYQPLNPP
Sbjct  181  NTLKEEDEPSEDFAQPGSLYMALNQQTVEPPSSSTSLSSIASSSSSRSLSQEAYQPLNPP  240

Query  241  PTQSAVLARSQSRSSRAARRSSGFLLPPGPNPTQQRTESGLPSNSTSQSAFQSSSGGSEA  300
            PTQSAVLARSQSRSSRAARRSSGFLLPPGPNPTQQRTESGLPSNSTSQSAFQSSSGGSEA
Sbjct  241  PTQSAVLARSQSRSSRAARRSSGFLLPPGPNPTQQRTESGLPSNSTSQSAFQSSSGGSEA  300

Query  301  RSTAELSAHKSKGLEKGDVLGEWETVEVKQEPLDEARFLNALNGEMIKKTVRVGDVELFD  360
            RSTAELSAHKSKGLEKGDVLGEWETVEVKQEPLDEARFLNALNGEMIKKTVRVGDVELFD
Sbjct  301  RSTAELSAHKSKGLEKGDVLGEWETVEVKQEPLDEARFLNALNGEMIKKTVRVGDVELFD  360

Query  361  EISPEDEDKKTVETTIGRAVVPSIPMELTQRPTLTKPVALAIRKGMSSGSLENCRSTSQS  420
            EISPEDEDKKTVETTIGRAVVPSIPMELTQRPTLTKPVALAIRKGMSSGSLENC      
Sbjct  361  EISPEDEDKKTVETTIGRAVVPSIPMELTQRPTLTKPVALAIRKGMSSGSLENC------  414

Query  421  LLQSTPVSNVTDSNTSRLKSTLTAPPFEGSSVSKMIKPPSTATKSLVLPLIASNVPSIAG  480
                                         SSVSKMIKPPSTATKSLVLPLIASNVPSIAG
Sbjct  415  -----------------------------SSVSKMIKPPSTATKSLVLPLIASNVPSIAG  445

Query  481  SSSAPVLPSNSTTISRSSVSKSKTSLVGSMKPPNPKTGFSLISHSTSASSSSSTISAAHT  540
            SSSAPVLPSNSTTISRSSVSKSKTSLVGSMKPPNPKTGFSLISHSTSASSSSSTISAAHT
Sbjct  446  SSSAPVLPSNSTTISRSSVSKSKTSLVGSMKPPNPKTGFSLISHSTSASSSSSTISAAHT  505

Query  541  VVAEEGPIKLNGIYNTSQAESKGGSSMVKMKDVADQLRKGIVGPIDRNSPPEKSKLKGKS  600
            VVAEEGPIKLNGIYNTSQAESKGGSSMVKMKDVADQLRKGIVGPIDRNSPPEKSKLKGKS
Sbjct  506  VVAEEGPIKLNGIYNTSQAESKGGSSMVKMKDVADQLRKGIVGPIDRNSPPEKSKLKGKS  565

Query  601  KAEEEEFVAGVDMDDIGVGYRDDEDQEGEEDRRRMRRRASPATYISEFRQDTTLSRPTLY  660
            KAEEEEFVAGVDMDDIGVGYRDDEDQEGEEDRRRMRRRASPATYISEFRQDTTLSRPTLY
Sbjct  566  KAEEEEFVAGVDMDDIGVGYRDDEDQEGEEDRRRMRRRASPATYISEFRQDTTLSRPTLY  625

Query  661  TPPPEDAPSGTTISSVPSRSASWSTTSKSTEEIQGRRGRERKSVNYKEPSLTTKMRKPAD  720
            TPPPEDAPSGTTISSVPSRSASWSTTSKSTEEIQGRRGRERKSVNYKEPSLTTKMRKPAD
Sbjct  626  TPPPEDAPSGTTISSVPSRSASWSTTSKSTEEIQGRRGRERKSVNYKEPSLTTKMRKPAD  685

Query  721  DTTSKPKSRNILPSSSRASSQEVEHDLLSSMDAKNPALVVPRNDLVRRKSSLPKKPPIDN  780
            DTTSKPKSRNILPSSSRASSQEVEHDLLSSMDAKNPALVVPRNDLVRRKSSLPKKPPIDN
Sbjct  686  DTTSKPKSRNILPSSSRASSQEVEHDLLSSMDAKNPALVVPRNDLVRRKSSLPKKPPIDN  745

Query  781  TMTIGRTGSGSHAKMSFSEEEEEEEPEDEDEDEDDYEEIDDREPKAYEEMRPEARRDTGR  840
            TMTIGRTGSGSHAKMSFSEEEEEEEPEDEDEDEDDYEEIDDREPKAYEEMRPEARRDTGR
Sbjct  746  TMTIGRTGSGSHAKMSFSEEEEEEEPEDEDEDEDDYEEIDDREPKAYEEMRPEARRDTGR  805

Query  841  YSSTSVDTQEVGGDSYDEEDDDHDGAIRRLKIITPAPRASTSSSSSTATRRRDSGLTAGS  900
            YSSTSVDTQEVGGDSYDEEDDDHDGAIRRLKIITPAPRASTSSSSSTATRRRDSGLTAGS
Sbjct  806  YSSTSVDTQEVGGDSYDEEDDDHDGAIRRLKIITPAPRASTSSSSSTATRRRDSGLTAGS  865

Query  901  KKGRIRVPSGDEDDV  915
            KKGRIRVPSGDEDDV
Sbjct  866  KKGRIRVPSGDEDDV  880


>gb|KIJ31844.1| hypothetical protein M422DRAFT_36101 [Sphaerobolus stellatus 
SS14]
Length=524

 Score = 44.7 bits (104),  Expect = 0.55, Method: Compositional matrix adjust.
 Identities = 32/62 (52%), Positives = 36/62 (58%), Gaps = 7/62 (11%)

Query  672  TISSVPSRSASWSTTS------KSTEEIQGRRGRERKSVNYKEPSLTTKMRKP-ADDTTS  724
            T S+VP+ SA   T S      K TE   GR  R RKSVNY EP L TKMRKP +   T+
Sbjct  374  TTSAVPTSSAVSMTDSEMNSPDKITEVSGGRERRTRKSVNYAEPKLNTKMRKPDSSSDTA  433

Query  725  KP  726
            KP
Sbjct  434  KP  435


>gb|KIJ98039.1| hypothetical protein K443DRAFT_681088 [Laccaria amethystina LaAM-08-1]
Length=643

 Score = 40.8 bits (94),  Expect = 7.9, Method: Compositional matrix adjust.
 Identities = 26/48 (54%), Positives = 28/48 (58%), Gaps = 4/48 (8%)

Query  675  SVPSRSASWSTTSKSTEE----IQGRRGRERKSVNYKEPSLTTKMRKP  718
            S+P+  AS S T  S  E      GR  R RKSVNY EP L TKMRKP
Sbjct  386  SLPTPRASSSPTPASEAESATTAAGREKRTRKSVNYAEPKLNTKMRKP  433


>gb|KIJ63388.1| hypothetical protein HYDPIDRAFT_29636 [Hydnomerulius pinastri 
MD-312]
Length=584

 Score = 40.8 bits (94),  Expect = 8.6, Method: Compositional matrix adjust.
 Identities = 27/51 (53%), Positives = 30/51 (59%), Gaps = 3/51 (6%)

Query  668  PSGTTISSVPSRSASWSTTSKSTEEIQGRRGRERKSVNYKEPSLTTKMRKP  718
            P+  T S VP  +AS    S+S     GR  R RKSVNY EP L TKMRKP
Sbjct  403  PTPRTSSPVPPGAAS---ASESEAPPGGRERRTRKSVNYAEPKLNTKMRKP  450


Lambda      K        H        a         alpha
   0.306    0.123    0.330    0.792     4.96 

Gapped
Lambda      K        H        a         alpha    sigma
   0.267   0.0410    0.140     1.90     42.6     43.6 

Effective search space used: 11380064934960


  Database: nr
    Posted date:  Sep 23, 2015 12:05 AM
  Number of letters in database: 26,053,659,533
  Number of sequences in database:  71,551,133


Matrix: BLOSUM62
Gap Penalties: Existence: 11, Extension: 1
Neighboring words threshold: 11
Window for multiple hits: 40
```
