# Supplementary material for: Comparative genomics provides new insights into the diversity, physiology, and sexuality of the only industrially exploited tremellomycete: Phaffia rhodozyma
Source: BMC Genomics. 2016 Nov 9;17:901. doi: 10.1186/s12864-016-3244-7 (PMC5103461; doi:10.1186/s12864-016-3244-7)
Supplement: Additional file 6: — List of orphan genes with links to PFAM (related to Additional file 1: Table S1). (ZIP 1428 kb) [file 12864_2016_3244_MOESM6_ESM.zip › BLAST_HTML_FTR/G00526_P.html]

BLAST Search Results


```
BLASTP 2.2.27+


Reference:
Stephen F. Altschul, Thomas L. Madden, Alejandro A. Schäffer,
Jinghui Zhang, Zheng Zhang, Webb Miller, and David J. Lipman (1997),
"Gapped BLAST and PSI-BLAST: a new generation of protein database
search programs", Nucleic Acids Res. 25:3389-3402.


Reference for
composition-based statistics:
Alejandro A. Schäffer, L. Aravind, Thomas L. Madden, Sergei
Shavirin, John L. Spouge, Yuri I. Wolf, Eugene V. Koonin, and
Stephen F. Altschul (2001), "Improving the accuracy of PSI-BLAST
protein database searches with composition-based statistics and
other refinements", Nucleic Acids Res. 29:2994-3005.


Database: nr
           71,551,133 sequences; 26,053,659,533 total letters


Query= G00526_P

Length=450
                                                                      Score     E
Sequences producing significant alignments:                          (Bits)  Value

emb|CED83838.1|  hypothetical protein [Xanthophyllomyces dendrorh...   486    1e-168
emb|CED83837.1|  hypothetical protein [Xanthophyllomyces dendrorh...  63.5    2e-08 
ref|XP_003651733.1|  hypothetical protein THITE_2112332 [Thielavi...  39.3    8.0   


 >emb|CED83838.1| hypothetical protein [Xanthophyllomyces dendrorhous]
Length=244

 Score =  486 bits (1251),  Expect = 1e-168, Method: Compositional matrix adjust.
 Identities = 244/244 (100%), Positives = 244/244 (100%), Gaps = 0/244 (0%)

Query  206  MDEVDWENSGMFNRFGTDVVVDEVTLTRRDPADRSSGKDGPVGLNHSVALLRLDHSPSSS  265
            MDEVDWENSGMFNRFGTDVVVDEVTLTRRDPADRSSGKDGPVGLNHSVALLRLDHSPSSS
Sbjct  1    MDEVDWENSGMFNRFGTDVVVDEVTLTRRDPADRSSGKDGPVGLNHSVALLRLDHSPSSS  60

Query  266  PQADESPLLSPSQMRGDLSMPSVSPPWMPDTKSPRIDKPAAYDGMPSSEANSSFFPDFHS  325
            PQADESPLLSPSQMRGDLSMPSVSPPWMPDTKSPRIDKPAAYDGMPSSEANSSFFPDFHS
Sbjct  61   PQADESPLLSPSQMRGDLSMPSVSPPWMPDTKSPRIDKPAAYDGMPSSEANSSFFPDFHS  120

Query  326  SSIFGGEIVKAPSPIETPELCPSSTTPNAGLDSGNIPTTHSHSSSSSTDSHLNQTSPPPS  385
            SSIFGGEIVKAPSPIETPELCPSSTTPNAGLDSGNIPTTHSHSSSSSTDSHLNQTSPPPS
Sbjct  121  SSIFGGEIVKAPSPIETPELCPSSTTPNAGLDSGNIPTTHSHSSSSSTDSHLNQTSPPPS  180

Query  386  LPTLPLLQSSSIGPSSSASPVSCPIPTTTAPPLAKRFLSGFSFGGINKVFKHSTKEAGAG  445
            LPTLPLLQSSSIGPSSSASPVSCPIPTTTAPPLAKRFLSGFSFGGINKVFKHSTKEAGAG
Sbjct  181  LPTLPLLQSSSIGPSSSASPVSCPIPTTTAPPLAKRFLSGFSFGGINKVFKHSTKEAGAG  240

Query  446  HGFF  449
            HGFF
Sbjct  241  HGFF  244


>emb|CED83837.1| hypothetical protein [Xanthophyllomyces dendrorhous]
Length=168

 Score = 63.5 bits (153),  Expect = 2e-08, Method: Compositional matrix adjust.
 Identities = 43/73 (59%), Positives = 48/73 (66%), Gaps = 14/73 (19%)

Query  14   SMSLSFSKAPSEESLDDPSFIPTSPRRIC-HLRPCLVHSSDSSGSS--WTRLDHQSTGHV  70
            SMSLSFSKAPSEESLDDPSFIPTSPRRIC  LR  L    D  G S  WTR       H+
Sbjct  104  SMSLSFSKAPSEESLDDPSFIPTSPRRICRQLRIQL----DEIGPSKYWTR----QVIHI  155

Query  71   K---SFISLKTSP  80
                ++I+ +TSP
Sbjct  156  AQDLAYITKRTSP  168


>ref|XP_003651733.1| hypothetical protein THITE_2112332 [Thielavia terrestris NRRL 
8126]
 gb|AEO65397.1| hypothetical protein THITE_2112332 [Thielavia terrestris NRRL 
8126]
Length=481

 Score = 39.3 bits (90),  Expect = 8.0, Method: Compositional matrix adjust.
 Identities = 43/153 (28%), Positives = 71/153 (46%), Gaps = 23/153 (15%)

Query  272  PLLSPSQMRGDLSMPSVSPPWMPDTKSP-RIDKPAAYDGMPSSEANSSFFPDFHSSSIFG  330
            PL+SP+   G++S+ S+  P  P +  P R D+P + D  PS            S++   
Sbjct  79   PLVSPTTSNGNMSVASIVSPTTPGSADPRRFDRPQSLDSAPS------------SATFLS  126

Query  331  GEIVKAPSPIETPELCPSSTTPNAGLDS---GNIP-TTHSHSSSSSTDSHLNQTSPPPSL  386
            GE+ +A S  E+ +     +  N G      GN P  +H+HS++S   +   Q +P P L
Sbjct  127  GEVPEALSRRESVD-----SRINQGFHDMRLGNSPYASHNHSTTSIHTTLQQQRNPRPGL  181

Query  387  PTLPLLQ-SSSIGPSSSASPVSCPIPTTTAPPL  418
              L + + S+   PS+  +P   P     AP +
Sbjct  182  DNLAVHRISNGYQPSADRNPEGHPKTMRIAPAI  214


Lambda      K        H        a         alpha
   0.311    0.127    0.381    0.792     4.96 

Gapped
Lambda      K        H        a         alpha    sigma
   0.267   0.0410    0.140     1.90     42.6     43.6 

Effective search space used: 4450296375096


  Database: nr
    Posted date:  Sep 23, 2015 12:05 AM
  Number of letters in database: 26,053,659,533
  Number of sequences in database:  71,551,133


Matrix: BLOSUM62
Gap Penalties: Existence: 11, Extension: 1
Neighboring words threshold: 11
Window for multiple hits: 40
```
