# Supplementary material for: Comparative genomics provides new insights into the diversity, physiology, and sexuality of the only industrially exploited tremellomycete: Phaffia rhodozyma
Source: BMC Genomics. 2016 Nov 9;17:901. doi: 10.1186/s12864-016-3244-7 (PMC5103461; doi:10.1186/s12864-016-3244-7)
Supplement: Additional file 6: — List of orphan genes with links to PFAM (related to Additional file 1: Table S1). (ZIP 1428 kb) [file 12864_2016_3244_MOESM6_ESM.zip › BLAST_HTML_FTR/G04581_P.html]

BLAST Search Results


```
BLASTP 2.2.27+


Reference:
Stephen F. Altschul, Thomas L. Madden, Alejandro A. Schäffer,
Jinghui Zhang, Zheng Zhang, Webb Miller, and David J. Lipman (1997),
"Gapped BLAST and PSI-BLAST: a new generation of protein database
search programs", Nucleic Acids Res. 25:3389-3402.


Reference for
composition-based statistics:
Alejandro A. Schäffer, L. Aravind, Thomas L. Madden, Sergei
Shavirin, John L. Spouge, Yuri I. Wolf, Eugene V. Koonin, and
Stephen F. Altschul (2001), "Improving the accuracy of PSI-BLAST
protein database searches with composition-based statistics and
other refinements", Nucleic Acids Res. 29:2994-3005.


Database: nr
           71,551,133 sequences; 26,053,659,533 total letters


Query= G04581_P

Length=307
                                                                      Score     E
Sequences producing significant alignments:                          (Bits)  Value

emb|CED83914.1|  hypothetical protein [Xanthophyllomyces dendrorh...   491    9e-172
ref|NP_001260034.1|  dumpy, isoform S [Drosophila melanogaster] >...  43.5    0.28  
ref|NP_001260040.1|  dumpy, isoform Y [Drosophila melanogaster] >...  43.5    0.29  
ref|XP_002132955.1|  GA26108 [Drosophila pseudoobscura pseudoobsc...  43.5    0.31  
ref|XP_002037833.1|  GM18084 [Drosophila sechellia] >gb|EDW54251....  41.6    1.4   
ref|NP_001260031.1|  dumpy, isoform P [Drosophila melanogaster] >...  41.2    1.5   
ref|NP_001260035.1|  dumpy, isoform T [Drosophila melanogaster] >...  41.2    1.5   
ref|NP_001260030.1|  dumpy, isoform O [Drosophila melanogaster] >...  41.2    1.5   
ref|NP_001260032.1|  dumpy, isoform Q [Drosophila melanogaster] >...  41.2    1.5   
ref|NP_001260036.1|  dumpy, isoform U [Drosophila melanogaster] >...  41.2    1.6   
ref|NP_001260033.1|  dumpy, isoform R [Drosophila melanogaster] >...  41.2    1.6   
ref|NP_001260037.1|  dumpy, isoform V [Drosophila melanogaster] >...  40.0    3.6   


 >emb|CED83914.1| hypothetical protein [Xanthophyllomyces dendrorhous]
Length=330

 Score =  491 bits (1265),  Expect = 9e-172, Method: Compositional matrix adjust.
 Identities = 242/243 (99%), Positives = 243/243 (100%), Gaps = 0/243 (0%)

Query  64   RQLLPTSSPLTSLPFAQEADIEVPVAAVPDDPRQLFKPSVLREENLVPLAFPPDHPWESL  123
            RQLLPTSSPLTSLPFAQEADIEVPVAAVPDDPRQLFKPSVLREENLVPLAFPPDHPWESL
Sbjct  88   RQLLPTSSPLTSLPFAQEADIEVPVAAVPDDPRQLFKPSVLREENLVPLAFPPDHPWESL  147

Query  124  SIHDKIRPNDQRHNPLPTFPRPISVPAYPEPVLPSSPRPVDHLPTVRYRDLPPETMAELY  183
            SIHDKIRPNDQRHNPLPTFPRPISVPAYPEPVLPSSPRPVDHLPTVRYRDLPPETMAELY
Sbjct  148  SIHDKIRPNDQRHNPLPTFPRPISVPAYPEPVLPSSPRPVDHLPTVRYRDLPPETMAELY  207

Query  184  RARIMARPVKGKDQGWVQKAWDMFDAEVDDPNGWVLVREKMGLPPTRADRPASILFWWEN  243
            RARIMARPVKGKDQGWVQKAWDMFDAEVDDPNGWVLVREKMGLPPTRADRPASILFWWEN
Sbjct  208  RARIMARPVKGKDQGWVQKAWDMFDAEVDDPNGWVLVREKMGLPPTRADRPASILFWWEN  267

Query  244  LEEEQLWRWLVPFFWVWALVPIIGTGLLVSFFAYDGAFEDENGFRFGKLGWGVPEPPVRK  303
            LEEEQLWRWLVPFFWVWALVPIIGTGLLVSFFAYDGAFEDENGFRFGKLGWGVP+PPVRK
Sbjct  268  LEEEQLWRWLVPFFWVWALVPIIGTGLLVSFFAYDGAFEDENGFRFGKLGWGVPQPPVRK  327

Query  304  ELA  306
            ELA
Sbjct  328  ELA  330


>ref|NP_001260034.1| dumpy, isoform S [Drosophila melanogaster]
 gb|AGB92570.1| dumpy, isoform S [Drosophila melanogaster]
Length=18641

 Score = 43.5 bits (101),  Expect = 0.28, Method: Composition-based stats.
 Identities = 46/184 (25%), Positives = 73/184 (40%), Gaps = 29/184 (16%)

Query  4      SIPSYRTFASCHVRYSKDPSVPPSPSPSSPASSPS----PSSHSSSSDTPRPSLYRRQES  59
              +IPSY T  +      + P   PS     P + PS    PS    +  TP+  +Y    +
Sbjct  14764  NIPSYPTPVA---PTPQSPIYIPSQEQPKPTTRPSVINVPSVPQPAYPTPQAPVY--DVN  14818

Query  60     FHAQRQLLPTSSPLTSLPFAQEADIEVPVAAVPDDPRQLFKPSVLREENL-------VPL  112
              +     ++P    + ++P        VP+ A P   R +F PS +            +P 
Sbjct  14819  YPTSPSVIPHQPGVVNIP-------SVPLPAPPVKQRPVFVPSPVHPTPAPQPGVVNIPS  14871

Query  113    AFPPDHPWESLSIHDKIRPNDQRHNPLPTFPRPISVPAYPEPVLPSS------PRPVDHL  166
                 P HP     + ++    D  + P P+ P  I++P+ P PV P        P PV H+
Sbjct  14872  VAQPVHPTYQPPVVERPAIYDVYYPPPPSRPGVINIPSPPRPVYPVPQQPIYVPAPVLHI  14931

Query  167    PTVR  170
              P  R
Sbjct  14932  PAPR  14935


 Score = 39.3 bits (90),  Expect = 7.3, Method: Composition-based stats.
 Identities = 37/150 (25%), Positives = 59/150 (39%), Gaps = 13/150 (9%)

Query  18     YSKDPSVPPSPSPSSPASSPSPSSHSSSSDTPRPSL----YRRQESFHAQRQLLPTSSPL  73
              Y  + S  PSP P  P     PS+       P P +    Y    +   Q  +L   S  
Sbjct  14710  YDVNYSTTPSPIPQKPGVVNIPSAPQPVHPAPNPPVHEFNYPTPPAVPQQPGVLNIPSYP  14769

Query  74     TSLPFAQEADIEVPVAAVPDDPRQLFKPSVLREENLVPLAFPPDHPWESLSIHDKIRPND  133
              T +    ++ I +P     + P+   +PSV+     VP    P +P     ++D   P  
Sbjct  14770  TPVAPTPQSPIYIPSQ---EQPKPTTRPSVIN----VPSVPQPAYPTPQAPVYDVNYPTS  14822

Query  134    QRHNPLPTFPRPISVPAYPEPVLPSSPRPV  163
                 + +P  P  +++P+ P P  P   RPV
Sbjct  14823  P--SVIPHQPGVVNIPSVPLPAPPVKQRPV  14850


>ref|NP_001260040.1| dumpy, isoform Y [Drosophila melanogaster]
 gb|AGB92576.1| dumpy, isoform Y [Drosophila melanogaster]
Length=18095

 Score = 43.5 bits (101),  Expect = 0.29, Method: Composition-based stats.
 Identities = 46/184 (25%), Positives = 73/184 (40%), Gaps = 29/184 (16%)

Query  4      SIPSYRTFASCHVRYSKDPSVPPSPSPSSPASSPS----PSSHSSSSDTPRPSLYRRQES  59
              +IPSY T  +      + P   PS     P + PS    PS    +  TP+  +Y    +
Sbjct  14218  NIPSYPTPVA---PTPQSPIYIPSQEQPKPTTRPSVINVPSVPQPAYPTPQAPVY--DVN  14272

Query  60     FHAQRQLLPTSSPLTSLPFAQEADIEVPVAAVPDDPRQLFKPSVLREENL-------VPL  112
              +     ++P    + ++P        VP+ A P   R +F PS +            +P 
Sbjct  14273  YPTSPSVIPHQPGVVNIP-------SVPLPAPPVKQRPVFVPSPVHPTPAPQPGVVNIPS  14325

Query  113    AFPPDHPWESLSIHDKIRPNDQRHNPLPTFPRPISVPAYPEPVLPSS------PRPVDHL  166
                 P HP     + ++    D  + P P+ P  I++P+ P PV P        P PV H+
Sbjct  14326  VAQPVHPTYQPPVVERPAIYDVYYPPPPSRPGVINIPSPPRPVYPVPQQPIYVPAPVLHI  14385

Query  167    PTVR  170
              P  R
Sbjct  14386  PAPR  14389


 Score = 39.3 bits (90),  Expect = 7.2, Method: Composition-based stats.
 Identities = 37/150 (25%), Positives = 59/150 (39%), Gaps = 13/150 (9%)

Query  18     YSKDPSVPPSPSPSSPASSPSPSSHSSSSDTPRPSL----YRRQESFHAQRQLLPTSSPL  73
              Y  + S  PSP P  P     PS+       P P +    Y    +   Q  +L   S  
Sbjct  14164  YDVNYSTTPSPIPQKPGVVNIPSAPQPVHPAPNPPVHEFNYPTPPAVPQQPGVLNIPSYP  14223

Query  74     TSLPFAQEADIEVPVAAVPDDPRQLFKPSVLREENLVPLAFPPDHPWESLSIHDKIRPND  133
              T +    ++ I +P     + P+   +PSV+     VP    P +P     ++D   P  
Sbjct  14224  TPVAPTPQSPIYIPSQ---EQPKPTTRPSVIN----VPSVPQPAYPTPQAPVYDVNYPTS  14276

Query  134    QRHNPLPTFPRPISVPAYPEPVLPSSPRPV  163
                 + +P  P  +++P+ P P  P   RPV
Sbjct  14277  P--SVIPHQPGVVNIPSVPLPAPPVKQRPV  14304


>ref|XP_002132955.1| GA26108 [Drosophila pseudoobscura pseudoobscura]
 gb|EDY70357.1| GA26108 [Drosophila pseudoobscura pseudoobscura]
Length=17011

 Score = 43.5 bits (101),  Expect = 0.31, Method: Composition-based stats.
 Identities = 41/147 (28%), Positives = 67/147 (46%), Gaps = 20/147 (14%)

Query  23     SVPPSPSPSSPASSPSPSSHSSSSDTPRPSLYRRQESFHAQRQLLPTSSPLTSLPFAQEA  82
              ++P  P P+ P   P+P  H  S  T RPS      + H+  Q  P  +P+  +      
Sbjct  13232  NIPSMPQPTYP--HPNPPIHDVSYPTQRPSPVPGVINVHSVPQPTPVPNPVPGV------  13283

Query  83     DIEVPVAAVPDDPRQLFKPSVLREENLVPLAFPPDHPWESLSIHDKIRPNDQRHNPLPT-  141
               I +P    P  P  +  P V+     +P    P +P  +  IHD   P  Q   P+P+ 
Sbjct  13284  -INIPSQQSP--PNIVPTPGVVN----IPSVPQPTYPTYNPPIHDVSYPTPQ---PIPSP  13333

Query  142    FPRPISVPAYPEPVLPSSPRPVDHLPT  168
               P  +++P+ P+P +PSS   V ++P+
Sbjct  13334  APGVVNIPSAPQP-MPSSIPGVINIPS  13359


 Score = 42.4 bits (98),  Expect = 0.77, Method: Composition-based stats.
 Identities = 45/162 (28%), Positives = 66/162 (41%), Gaps = 30/162 (19%)

Query  22     PSVP-PSPSPS----------SPASSPSPSSHSSSSDTPRPSLYRRQESFHAQRQLLPTS  70
              PSVP P+P+P            P  +P P   +  S  P+P     Q   +        S
Sbjct  12987  PSVPQPTPAPQPGVINIPSVPQPTPAPQPGVFNIPS-VPQPIPPSHQPPVY-------LS  13038

Query  71     SPLTSLPFA-QEADIEVPVAAVPDDPRQLF------KPSVLREENLV--PLAFPPDHPWE  121
              +PL S+P A Q   I +P    P  P           P   R   +V  P    P +P  
Sbjct  13039  TPLNSVPPAPQPGIINIPSVPQPRPPIHGVHYPTPQTPIPERPVGVVNIPSVVQPVYPTP  13098

Query  122    SLSIHDKIRPNDQRHNPLPTFPRPISVPAYPEPVLPSSPRPV  163
                 ++D   P    H+P+P  P  +++P+ P+PV P S RP+
Sbjct  13099  QPPVYDVNYPTP--HSPVPQRPVIVNIPSLPQPVAPVSQRPI  13138


>ref|XP_002037833.1| GM18084 [Drosophila sechellia]
 gb|EDW54251.1| GM18084 [Drosophila sechellia]
Length=14551

 Score = 41.6 bits (96),  Expect = 1.4, Method: Composition-based stats.
 Identities = 36/144 (25%), Positives = 58/144 (40%), Gaps = 22/144 (15%)

Query  23     SVPPSPSPSSPASSPSPSSHSSSSDTPRPSLYRRQESFHAQRQLLPTSSPLTSLPFAQEA  82
              ++P  P P  P+  P P  H + + TP            A R + PTS            
Sbjct  11171  NIPSVPQPVYPSPQP-PVYHVNYATTPVSQQPGVVNIPSAHRPVPPTS------------  11217

Query  83     DIEVPV-AAVPDDPRQLFKPSVLREENLVPLAFPPDHPWESLSIHDKIRPNDQRHNPLPT  141
                + PV    P +P    +P V+     +P    P +P     I+D   P  Q  +P+P 
Sbjct  11218  --QRPVFVTSPGNPTPTPQPGVIN----IPSVSQPGYPTPQSPIYDANYPTTQ--SPIPQ  11269

Query  142    FPRPISVPAYPEPVLPSSPRPVDH  165
               P  +++P+ P P  P+   PV++
Sbjct  11270  QPGVVNIPSLPSPAYPAPNPPVNY  11293


 Score = 40.8 bits (94),  Expect = 2.3, Method: Composition-based stats.
 Identities = 42/161 (26%), Positives = 64/161 (40%), Gaps = 24/161 (15%)

Query  22     PSVPPS------PSPSSPASSPSPSSHSSSSDTPRPSLYRRQESFHAQRQLLPTSSPLTS  75
              P+ PP       PSP SP+++P P +     + P P       +   Q  ++   S    
Sbjct  11316  PATPPQHPPVFIPSPESPSTAPKPGAPVYDVNYPTPP-----SAIPHQPGVVNIPSVPPP  11370

Query  76     LPFAQEADIEVPVAAVPDDPRQLFKPSVLREENLVPLAFPPDHPWESLSIHDKIRPNDQR  135
               P   +  + VP  A P    Q   P V+     +P    P HP     + ++    D  
Sbjct  11371  PPPVMQRPVFVPSPAHPTPGPQ---PGVVN----IPSVVQPVHPTYQSPVVERPAIYDVY  11423

Query  136    HNPLPTFPRPISVPAYPEPVLPSS------PRPVDHLPTVR  170
              + P P+ P  I++P+ P PV P        P PV H+P  R
Sbjct  11424  YPPPPSRPGVINIPSPPRPVYPVPQQPIYVPSPVLHIPAPR  11464


>ref|NP_001260031.1| dumpy, isoform P [Drosophila melanogaster]
 gb|AGB92567.1| dumpy, isoform P [Drosophila melanogaster]
Length=20710

 Score = 41.2 bits (95),  Expect = 1.5, Method: Composition-based stats.
 Identities = 46/184 (25%), Positives = 73/184 (40%), Gaps = 29/184 (16%)

Query  4      SIPSYRTFASCHVRYSKDPSVPPSPSPSSPASSPS----PSSHSSSSDTPRPSLYRRQES  59
              +IPSY T  +      + P   PS     P + PS    PS    +  TP+  +Y    +
Sbjct  16833  NIPSYPTPVA---PTPQSPIYIPSQEQPKPTTRPSVINVPSVPQPAYPTPQAPVY--DVN  16887

Query  60     FHAQRQLLPTSSPLTSLPFAQEADIEVPVAAVPDDPRQLFKPSVLREENL-------VPL  112
              +     ++P    + ++P        VP+ A P   R +F PS +            +P 
Sbjct  16888  YPTSPSVIPHQPGVVNIP-------SVPLPAPPVKQRPVFVPSPVHPTPAPQPGVVNIPS  16940

Query  113    AFPPDHPWESLSIHDKIRPNDQRHNPLPTFPRPISVPAYPEPVLPSS------PRPVDHL  166
                 P HP     + ++    D  + P P+ P  I++P+ P PV P        P PV H+
Sbjct  16941  VAQPVHPTYQPPVVERPAIYDVYYPPPPSRPGVINIPSPPRPVYPVPQQPIYVPAPVLHI  17000

Query  167    PTVR  170
              P  R
Sbjct  17001  PAPR  17004


>ref|NP_001260035.1| dumpy, isoform T [Drosophila melanogaster]
 gb|AGB92571.1| dumpy, isoform T [Drosophila melanogaster]
Length=22300

 Score = 41.2 bits (95),  Expect = 1.5, Method: Composition-based stats.
 Identities = 46/184 (25%), Positives = 73/184 (40%), Gaps = 29/184 (16%)

Query  4      SIPSYRTFASCHVRYSKDPSVPPSPSPSSPASSPS----PSSHSSSSDTPRPSLYRRQES  59
              +IPSY T  +      + P   PS     P + PS    PS    +  TP+  +Y    +
Sbjct  18423  NIPSYPTPVA---PTPQSPIYIPSQEQPKPTTRPSVINVPSVPQPAYPTPQAPVY--DVN  18477

Query  60     FHAQRQLLPTSSPLTSLPFAQEADIEVPVAAVPDDPRQLFKPSVLREENL-------VPL  112
              +     ++P    + ++P        VP+ A P   R +F PS +            +P 
Sbjct  18478  YPTSPSVIPHQPGVVNIP-------SVPLPAPPVKQRPVFVPSPVHPTPAPQPGVVNIPS  18530

Query  113    AFPPDHPWESLSIHDKIRPNDQRHNPLPTFPRPISVPAYPEPVLPSS------PRPVDHL  166
                 P HP     + ++    D  + P P+ P  I++P+ P PV P        P PV H+
Sbjct  18531  VAQPVHPTYQPPVVERPAIYDVYYPPPPSRPGVINIPSPPRPVYPVPQQPIYVPAPVLHI  18590

Query  167    PTVR  170
              P  R
Sbjct  18591  PAPR  18594


>ref|NP_001260030.1| dumpy, isoform O [Drosophila melanogaster]
 gb|AGB92566.1| dumpy, isoform O [Drosophila melanogaster]
Length=22743

 Score = 41.2 bits (95),  Expect = 1.5, Method: Composition-based stats.
 Identities = 46/184 (25%), Positives = 73/184 (40%), Gaps = 29/184 (16%)

Query  4      SIPSYRTFASCHVRYSKDPSVPPSPSPSSPASSPS----PSSHSSSSDTPRPSLYRRQES  59
              +IPSY T  +      + P   PS     P + PS    PS    +  TP+  +Y    +
Sbjct  18866  NIPSYPTPVA---PTPQSPIYIPSQEQPKPTTRPSVINVPSVPQPAYPTPQAPVY--DVN  18920

Query  60     FHAQRQLLPTSSPLTSLPFAQEADIEVPVAAVPDDPRQLFKPSVLREENL-------VPL  112
              +     ++P    + ++P        VP+ A P   R +F PS +            +P 
Sbjct  18921  YPTSPSVIPHQPGVVNIP-------SVPLPAPPVKQRPVFVPSPVHPTPAPQPGVVNIPS  18973

Query  113    AFPPDHPWESLSIHDKIRPNDQRHNPLPTFPRPISVPAYPEPVLPSS------PRPVDHL  166
                 P HP     + ++    D  + P P+ P  I++P+ P PV P        P PV H+
Sbjct  18974  VAQPVHPTYQPPVVERPAIYDVYYPPPPSRPGVINIPSPPRPVYPVPQQPIYVPAPVLHI  19033

Query  167    PTVR  170
              P  R
Sbjct  19034  PAPR  19037


>ref|NP_001260032.1| dumpy, isoform Q [Drosophila melanogaster]
 gb|AGB92568.1| dumpy, isoform Q [Drosophila melanogaster]
Length=22949

 Score = 41.2 bits (95),  Expect = 1.5, Method: Composition-based stats.
 Identities = 46/184 (25%), Positives = 73/184 (40%), Gaps = 29/184 (16%)

Query  4      SIPSYRTFASCHVRYSKDPSVPPSPSPSSPASSPS----PSSHSSSSDTPRPSLYRRQES  59
              +IPSY T  +      + P   PS     P + PS    PS    +  TP+  +Y    +
Sbjct  19072  NIPSYPTPVA---PTPQSPIYIPSQEQPKPTTRPSVINVPSVPQPAYPTPQAPVY--DVN  19126

Query  60     FHAQRQLLPTSSPLTSLPFAQEADIEVPVAAVPDDPRQLFKPSVLREENL-------VPL  112
              +     ++P    + ++P        VP+ A P   R +F PS +            +P 
Sbjct  19127  YPTSPSVIPHQPGVVNIP-------SVPLPAPPVKQRPVFVPSPVHPTPAPQPGVVNIPS  19179

Query  113    AFPPDHPWESLSIHDKIRPNDQRHNPLPTFPRPISVPAYPEPVLPSS------PRPVDHL  166
                 P HP     + ++    D  + P P+ P  I++P+ P PV P        P PV H+
Sbjct  19180  VAQPVHPTYQPPVVERPAIYDVYYPPPPSRPGVINIPSPPRPVYPVPQQPIYVPAPVLHI  19239

Query  167    PTVR  170
              P  R
Sbjct  19240  PAPR  19243


>ref|NP_001260036.1| dumpy, isoform U [Drosophila melanogaster]
 gb|AGB92572.1| dumpy, isoform U [Drosophila melanogaster]
Length=21657

 Score = 41.2 bits (95),  Expect = 1.6, Method: Composition-based stats.
 Identities = 46/184 (25%), Positives = 73/184 (40%), Gaps = 29/184 (16%)

Query  4      SIPSYRTFASCHVRYSKDPSVPPSPSPSSPASSPS----PSSHSSSSDTPRPSLYRRQES  59
              +IPSY T  +      + P   PS     P + PS    PS    +  TP+  +Y    +
Sbjct  17780  NIPSYPTPVA---PTPQSPIYIPSQEQPKPTTRPSVINVPSVPQPAYPTPQAPVY--DVN  17834

Query  60     FHAQRQLLPTSSPLTSLPFAQEADIEVPVAAVPDDPRQLFKPSVLREENL-------VPL  112
              +     ++P    + ++P        VP+ A P   R +F PS +            +P 
Sbjct  17835  YPTSPSVIPHQPGVVNIP-------SVPLPAPPVKQRPVFVPSPVHPTPAPQPGVVNIPS  17887

Query  113    AFPPDHPWESLSIHDKIRPNDQRHNPLPTFPRPISVPAYPEPVLPSS------PRPVDHL  166
                 P HP     + ++    D  + P P+ P  I++P+ P PV P        P PV H+
Sbjct  17888  VAQPVHPTYQPPVVERPAIYDVYYPPPPSRPGVINIPSPPRPVYPVPQQPIYVPAPVLHI  17947

Query  167    PTVR  170
              P  R
Sbjct  17948  PAPR  17951


>ref|NP_001260033.1| dumpy, isoform R [Drosophila melanogaster]
 gb|AGB92569.1| dumpy, isoform R [Drosophila melanogaster]
Length=22830

 Score = 41.2 bits (95),  Expect = 1.6, Method: Composition-based stats.
 Identities = 46/184 (25%), Positives = 73/184 (40%), Gaps = 29/184 (16%)

Query  4      SIPSYRTFASCHVRYSKDPSVPPSPSPSSPASSPS----PSSHSSSSDTPRPSLYRRQES  59
              +IPSY T  +      + P   PS     P + PS    PS    +  TP+  +Y    +
Sbjct  18953  NIPSYPTPVA---PTPQSPIYIPSQEQPKPTTRPSVINVPSVPQPAYPTPQAPVY--DVN  19007

Query  60     FHAQRQLLPTSSPLTSLPFAQEADIEVPVAAVPDDPRQLFKPSVLREENL-------VPL  112
              +     ++P    + ++P        VP+ A P   R +F PS +            +P 
Sbjct  19008  YPTSPSVIPHQPGVVNIP-------SVPLPAPPVKQRPVFVPSPVHPTPAPQPGVVNIPS  19060

Query  113    AFPPDHPWESLSIHDKIRPNDQRHNPLPTFPRPISVPAYPEPVLPSS------PRPVDHL  166
                 P HP     + ++    D  + P P+ P  I++P+ P PV P        P PV H+
Sbjct  19061  VAQPVHPTYQPPVVERPAIYDVYYPPPPSRPGVINIPSPPRPVYPVPQQPIYVPAPVLHI  19120

Query  167    PTVR  170
              P  R
Sbjct  19121  PAPR  19124


>ref|NP_001260037.1| dumpy, isoform V [Drosophila melanogaster]
 gb|AGB92573.1| dumpy, isoform V [Drosophila melanogaster]
Length=20404

 Score = 40.0 bits (92),  Expect = 3.6, Method: Composition-based stats.
 Identities = 46/184 (25%), Positives = 73/184 (40%), Gaps = 29/184 (16%)

Query  4      SIPSYRTFASCHVRYSKDPSVPPSPSPSSPASSPS----PSSHSSSSDTPRPSLYRRQES  59
              +IPSY T  +      + P   PS     P + PS    PS    +  TP+  +Y    +
Sbjct  16527  NIPSYPTPVA---PTPQSPIYIPSQEQPKPTTRPSVINVPSVPQPAYPTPQAPVY--DVN  16581

Query  60     FHAQRQLLPTSSPLTSLPFAQEADIEVPVAAVPDDPRQLFKPSVLREENL-------VPL  112
              +     ++P    + ++P        VP+ A P   R +F PS +            +P 
Sbjct  16582  YPTSPSVIPHQPGVVNIP-------SVPLPAPPVKQRPVFVPSPVHPTPAPQPGVVNIPS  16634

Query  113    AFPPDHPWESLSIHDKIRPNDQRHNPLPTFPRPISVPAYPEPVLPSS------PRPVDHL  166
                 P HP     + ++    D  + P P+ P  I++P+ P PV P        P PV H+
Sbjct  16635  VAQPVHPTYQPPVVERPAIYDVYYPPPPSRPGVINIPSPPRPVYPVPQQPIYVPAPVLHI  16694

Query  167    PTVR  170
              P  R
Sbjct  16695  PAPR  16698


Lambda      K        H        a         alpha
   0.319    0.137    0.451    0.792     4.96 

Gapped
Lambda      K        H        a         alpha    sigma
   0.267   0.0410    0.140     1.90     42.6     43.6 

Effective search space used: 2405395364531


  Database: nr
    Posted date:  Sep 23, 2015 12:05 AM
  Number of letters in database: 26,053,659,533
  Number of sequences in database:  71,551,133


Matrix: BLOSUM62
Gap Penalties: Existence: 11, Extension: 1
Neighboring words threshold: 11
Window for multiple hits: 40
```
