# Supplementary material for: Comparative genomics provides new insights into the diversity, physiology, and sexuality of the only industrially exploited tremellomycete: Phaffia rhodozyma
Source: BMC Genomics. 2016 Nov 9;17:901. doi: 10.1186/s12864-016-3244-7 (PMC5103461; doi:10.1186/s12864-016-3244-7)
Supplement: Additional file 6: — List of orphan genes with links to PFAM (related to Additional file 1: Table S1). (ZIP 1428 kb) [file 12864_2016_3244_MOESM6_ESM.zip › BLAST_HTML_FTR/G00401_P.html]

BLAST Search Results


```
BLASTP 2.2.27+


Reference:
Stephen F. Altschul, Thomas L. Madden, Alejandro A. Schäffer,
Jinghui Zhang, Zheng Zhang, Webb Miller, and David J. Lipman (1997),
"Gapped BLAST and PSI-BLAST: a new generation of protein database
search programs", Nucleic Acids Res. 25:3389-3402.


Reference for
composition-based statistics:
Alejandro A. Schäffer, L. Aravind, Thomas L. Madden, Sergei
Shavirin, John L. Spouge, Yuri I. Wolf, Eugene V. Koonin, and
Stephen F. Altschul (2001), "Improving the accuracy of PSI-BLAST
protein database searches with composition-based statistics and
other refinements", Nucleic Acids Res. 29:2994-3005.


Database: nr
           71,551,133 sequences; 26,053,659,533 total letters


Query= G00401_P

Length=197
                                                                      Score     E
Sequences producing significant alignments:                          (Bits)  Value

emb|CED83291.1|  hypothetical protein [Xanthophyllomyces dendrorh...   390    1e-135
ref|WP_044656983.1|  UDP-N-acetyl-D-galactosamine dehydrogenase, ...  37.4    7.1   


 >emb|CED83291.1| hypothetical protein [Xanthophyllomyces dendrorhous]
Length=196

 Score =  390 bits (1002),  Expect = 1e-135, Method: Compositional matrix adjust.
 Identities = 196/196 (100%), Positives = 196/196 (100%), Gaps = 0/196 (0%)

Query  1    MAPTRSRQALFPVYVDPAPSLSLSSCNIPSIEHSSVRTSPQKRKASSKSVPSFKEKPVDL  60
            MAPTRSRQALFPVYVDPAPSLSLSSCNIPSIEHSSVRTSPQKRKASSKSVPSFKEKPVDL
Sbjct  1    MAPTRSRQALFPVYVDPAPSLSLSSCNIPSIEHSSVRTSPQKRKASSKSVPSFKEKPVDL  60

Query  61   VVVAGNGLKKRDGMSDLGKVGKNGERVVRREVLRDVSQEWGVVGCEPEGFKNASVIKKPF  120
            VVVAGNGLKKRDGMSDLGKVGKNGERVVRREVLRDVSQEWGVVGCEPEGFKNASVIKKPF
Sbjct  61   VVVAGNGLKKRDGMSDLGKVGKNGERVVRREVLRDVSQEWGVVGCEPEGFKNASVIKKPF  120

Query  121  DPVKPGKLRIFADSASLPSSRPSASINTNDTVTATNAQPSRKPFSVYTSPSRTTSAITRP  180
            DPVKPGKLRIFADSASLPSSRPSASINTNDTVTATNAQPSRKPFSVYTSPSRTTSAITRP
Sbjct  121  DPVKPGKLRIFADSASLPSSRPSASINTNDTVTATNAQPSRKPFSVYTSPSRTTSAITRP  180

Query  181  KGLGLGGPGAARGLRS  196
            KGLGLGGPGAARGLRS
Sbjct  181  KGLGLGGPGAARGLRS  196


>ref|WP_044656983.1| UDP-N-acetyl-D-galactosamine dehydrogenase, partial [Bacteroides 
acidifaciens]
Length=339

 Score = 37.4 bits (85),  Expect = 7.1, Method: Compositional matrix adjust.
 Identities = 25/93 (27%), Positives = 46/93 (49%), Gaps = 3/93 (3%)

Query  37   RTSPQKRKASSKSVPSFKEKPVDL---VVVAGNGLKKRDGMSDLGKVGKNGERVVRREVL  93
             T  + +K +S S P   EK   L   V+ AG  L     +++  KV +N +R +    +
Sbjct  170  HTVEKIKKVTSGSTPEIGEKIDHLYSSVITAGTHLASSIKVAEAAKVIENSQRDINIAFV  229

Query  94   RDVSQEWGVVGCEPEGFKNASVIKKPFDPVKPG  126
             ++S+ + ++G + +   +A+  K  F P KPG
Sbjct  230  NELSKIFNLMGIDTQEVLDAAATKWNFLPFKPG  262


Lambda      K        H        a         alpha
   0.314    0.131    0.378    0.792     4.96 

Gapped
Lambda      K        H        a         alpha    sigma
   0.267   0.0410    0.140     1.90     42.6     43.6 

Effective search space used: 874136925585


  Database: nr
    Posted date:  Sep 23, 2015 12:05 AM
  Number of letters in database: 26,053,659,533
  Number of sequences in database:  71,551,133


Matrix: BLOSUM62
Gap Penalties: Existence: 11, Extension: 1
Neighboring words threshold: 11
Window for multiple hits: 40
```
