# Supplementary material for: Comparative genomics provides new insights into the diversity, physiology, and sexuality of the only industrially exploited tremellomycete: Phaffia rhodozyma
Source: BMC Genomics. 2016 Nov 9;17:901. doi: 10.1186/s12864-016-3244-7 (PMC5103461; doi:10.1186/s12864-016-3244-7)
Supplement: Additional file 6: — List of orphan genes with links to PFAM (related to Additional file 1: Table S1). (ZIP 1428 kb) [file 12864_2016_3244_MOESM6_ESM.zip › BLAST_HTML_FTR/G05020_P.html]

BLAST Search Results


```
BLASTP 2.2.27+


Reference:
Stephen F. Altschul, Thomas L. Madden, Alejandro A. Schäffer,
Jinghui Zhang, Zheng Zhang, Webb Miller, and David J. Lipman (1997),
"Gapped BLAST and PSI-BLAST: a new generation of protein database
search programs", Nucleic Acids Res. 25:3389-3402.


Reference for
composition-based statistics:
Alejandro A. Schäffer, L. Aravind, Thomas L. Madden, Sergei
Shavirin, John L. Spouge, Yuri I. Wolf, Eugene V. Koonin, and
Stephen F. Altschul (2001), "Improving the accuracy of PSI-BLAST
protein database searches with composition-based statistics and
other refinements", Nucleic Acids Res. 29:2994-3005.


Database: nr
           71,551,133 sequences; 26,053,659,533 total letters


Query= G05020_P

Length=228
                                                                      Score     E
Sequences producing significant alignments:                          (Bits)  Value

emb|CED85245.1|  hypothetical protein [Xanthophyllomyces dendrorh...   469    6e-166
emb|CCO27746.1|  hypothetical protein BN14_01733 [Rhizoctonia sol...  40.8    0.15  
gb|KDN42115.1|  hypothetical protein RSAG8_06973, partial [Rhizoc...  40.8    0.16  
gb|EUC61719.1|  transmembrane protein, putative [Rhizoctonia sola...  40.4    0.22  
emb|CEL51676.1|  hypothetical protein RSOLAG1IB_00211 [Rhizoctoni...  39.7    0.40  
ref|WP_052349612.1|  hypothetical protein [Synechococcus sp. NKBG...  38.5    1.3   


 >emb|CED85245.1| hypothetical protein [Xanthophyllomyces dendrorhous]
Length=227

 Score =  469 bits (1208),  Expect = 6e-166, Method: Compositional matrix adjust.
 Identities = 227/227 (100%), Positives = 227/227 (100%), Gaps = 0/227 (0%)

Query  1    MSSTLLSKSSRDKIENLPAYRSASSPVEYTMSQLPLHVNLPGNQAANFPKAFNPERALLY  60
            MSSTLLSKSSRDKIENLPAYRSASSPVEYTMSQLPLHVNLPGNQAANFPKAFNPERALLY
Sbjct  1    MSSTLLSKSSRDKIENLPAYRSASSPVEYTMSQLPLHVNLPGNQAANFPKAFNPERALLY  60

Query  61   QRAMARSLPKKTFLSKCVTLAVFATVGAIAYHLLFIEDYGHDNVMFAEARKERLRQISLK  120
            QRAMARSLPKKTFLSKCVTLAVFATVGAIAYHLLFIEDYGHDNVMFAEARKERLRQISLK
Sbjct  61   QRAMARSLPKKTFLSKCVTLAVFATVGAIAYHLLFIEDYGHDNVMFAEARKERLRQISLK  120

Query  121  RPLTAKEQAILDRPAIETHELVSDVSFSDLFRRMGQRFRDREEDRLARQDAYKRKIGLLD  180
            RPLTAKEQAILDRPAIETHELVSDVSFSDLFRRMGQRFRDREEDRLARQDAYKRKIGLLD
Sbjct  121  RPLTAKEQAILDRPAIETHELVSDVSFSDLFRRMGQRFRDREEDRLARQDAYKRKIGLLD  180

Query  181  ELDGTSAATVGTSDSGTGLNWQRYQRKSNVREALPEDVEWDKVKSLN  227
            ELDGTSAATVGTSDSGTGLNWQRYQRKSNVREALPEDVEWDKVKSLN
Sbjct  181  ELDGTSAATVGTSDSGTGLNWQRYQRKSNVREALPEDVEWDKVKSLN  227


>emb|CCO27746.1| hypothetical protein BN14_01733 [Rhizoctonia solani AG-1 IB]
Length=111

 Score = 40.8 bits (94),  Expect = 0.15, Method: Compositional matrix adjust.
 Identities = 25/74 (34%), Positives = 38/74 (51%), Gaps = 0/74 (0%)

Query  71   KTFLSKCVTLAVFATVGAIAYHLLFIEDYGHDNVMFAEARKERLRQISLKRPLTAKEQAI  130
            +T L + V +A +  VG I+ + +F  DYG    +F+ AR+   RQ +    L+  EQA 
Sbjct  33   RTKLHRYVAVASYTAVGFISIYNIFYADYGEQEHVFSPARRWLDRQKTAFWTLSPAEQAA  92

Query  131  LDRPAIETHELVSD  144
             DR   +  EL  D
Sbjct  93   ADRLKRQQQELPRD  106


>gb|KDN42115.1| hypothetical protein RSAG8_06973, partial [Rhizoctonia solani 
AG-8 WAC10335]
Length=108

 Score = 40.8 bits (94),  Expect = 0.16, Method: Compositional matrix adjust.
 Identities = 21/60 (35%), Positives = 34/60 (57%), Gaps = 0/60 (0%)

Query  74   LSKCVTLAVFATVGAIAYHLLFIEDYGHDNVMFAEARKERLRQISLKRPLTAKEQAILDR  133
            + + V +A +A+VG I+ + +F  DYG    +F+ AR+   RQ +    L+  EQA  DR
Sbjct  35   IHRYVAIASYASVGIISVYNVFFTDYGEQEHVFSPARRWLDRQKAAFWTLSPAEQAAADR  94


>gb|EUC61719.1| transmembrane protein, putative [Rhizoctonia solani AG-3 Rhs1AP]
 gb|KEP53226.1| putative transmembrane protein [Rhizoctonia solani 123E]
Length=108

 Score = 40.4 bits (93),  Expect = 0.22, Method: Compositional matrix adjust.
 Identities = 21/65 (32%), Positives = 36/65 (55%), Gaps = 0/65 (0%)

Query  69   PKKTFLSKCVTLAVFATVGAIAYHLLFIEDYGHDNVMFAEARKERLRQISLKRPLTAKEQ  128
            P +  L + V +A + +VG I+ + +F  DYG +  +F+ AR+   +Q +    L+  EQ
Sbjct  30   PTRPKLHRYVAIASYVSVGVISVYNIFYADYGKEEHVFSPARRWLDQQKTAFWTLSPAEQ  89

Query  129  AILDR  133
            A  DR
Sbjct  90   AAADR  94


>emb|CEL51676.1| hypothetical protein RSOLAG1IB_00211 [Rhizoctonia solani AG-1 
IB]
Length=111

 Score = 39.7 bits (91),  Expect = 0.40, Method: Compositional matrix adjust.
 Identities = 24/71 (34%), Positives = 36/71 (51%), Gaps = 0/71 (0%)

Query  74   LSKCVTLAVFATVGAIAYHLLFIEDYGHDNVMFAEARKERLRQISLKRPLTAKEQAILDR  133
            L + V +A +  VG I+ + +F  DYG    +F+ AR+   RQ +    L+  EQA  DR
Sbjct  36   LHRYVAVASYTAVGFISIYNIFYADYGEQEHVFSPARRWLDRQKTAFWTLSPAEQAAADR  95

Query  134  PAIETHELVSD  144
               +  EL  D
Sbjct  96   LKRQQQELPRD  106


>ref|WP_052349612.1| hypothetical protein [Synechococcus sp. NKBG042902]
Length=128

 Score = 38.5 bits (88),  Expect = 1.3, Method: Compositional matrix adjust.
 Identities = 23/94 (24%), Positives = 48/94 (51%), Gaps = 10/94 (11%)

Query  78   VTLAVFA-TVGAIAYHLLFIEDYGHDNVMFAEARKERLRQISLKRPLTAKEQAI------  130
            V L+V   TV  + + ++ ++D  ++N + A  +K R+ Q    + L  ++Q +      
Sbjct  35   VFLSVIGYTVSRVKFSIMSVKDKCNENTLKALRKKLRINQTQFAKALGVRQQTVSEWERG  94

Query  131  LDRPAIETHELVSDVSFSDLFRRMGQRFRDREED  164
            + RPA+   ++ +     D+  R+G +FRD  +D
Sbjct  95   IYRPALSIEQIKT---LEDMLSRVGLKFRDLPDD  125


Lambda      K        H        a         alpha
   0.320    0.133    0.379    0.792     4.96 

Gapped
Lambda      K        H        a         alpha    sigma
   0.267   0.0410    0.140     1.90     42.6     43.6 

Effective search space used: 1301335855584


  Database: nr
    Posted date:  Sep 23, 2015 12:05 AM
  Number of letters in database: 26,053,659,533
  Number of sequences in database:  71,551,133


Matrix: BLOSUM62
Gap Penalties: Existence: 11, Extension: 1
Neighboring words threshold: 11
Window for multiple hits: 40
```
