# Supplementary material for: Comparative genomics provides new insights into the diversity, physiology, and sexuality of the only industrially exploited tremellomycete: Phaffia rhodozyma
Source: BMC Genomics. 2016 Nov 9;17:901. doi: 10.1186/s12864-016-3244-7 (PMC5103461; doi:10.1186/s12864-016-3244-7)
Supplement: Additional file 6: — List of orphan genes with links to PFAM (related to Additional file 1: Table S1). (ZIP 1428 kb) [file 12864_2016_3244_MOESM6_ESM.zip › BLAST_HTML_FTR/G01012_P.html]

BLAST Search Results


```
BLASTP 2.2.27+


Reference:
Stephen F. Altschul, Thomas L. Madden, Alejandro A. Schäffer,
Jinghui Zhang, Zheng Zhang, Webb Miller, and David J. Lipman (1997),
"Gapped BLAST and PSI-BLAST: a new generation of protein database
search programs", Nucleic Acids Res. 25:3389-3402.


Reference for
composition-based statistics:
Alejandro A. Schäffer, L. Aravind, Thomas L. Madden, Sergei
Shavirin, John L. Spouge, Yuri I. Wolf, Eugene V. Koonin, and
Stephen F. Altschul (2001), "Improving the accuracy of PSI-BLAST
protein database searches with composition-based statistics and
other refinements", Nucleic Acids Res. 29:2994-3005.


Database: nr
           71,551,133 sequences; 26,053,659,533 total letters


Query= G01012_P

Length=1268
                                                                      Score     E
Sequences producing significant alignments:                          (Bits)  Value

emb|CDZ97213.1|  isoform aa [Xanthophyllomyces dendrorhous]           2568    0.0  


 >emb|CDZ97213.1| isoform aa [Xanthophyllomyces dendrorhous]
Length=1267

 Score = 2568 bits (6656),  Expect = 0.0, Method: Compositional matrix adjust.
 Identities = 1267/1267 (100%), Positives = 1267/1267 (100%), Gaps = 0/1267 (0%)

Query  1     MKRSVVTTPKSSLSRRPGPPPPGWLTQSKRKLAGLSFSNFKSAPSYDDHLFCTSTSHLAS  60
             MKRSVVTTPKSSLSRRPGPPPPGWLTQSKRKLAGLSFSNFKSAPSYDDHLFCTSTSHLAS
Sbjct  1     MKRSVVTTPKSSLSRRPGPPPPGWLTQSKRKLAGLSFSNFKSAPSYDDHLFCTSTSHLAS  60

Query  61    PLSNPLLPPTSLAFENQAWLQTGPDPRMLDVRARKYGLDKDFHAAHFAHEGIEVWDLLGR  120
             PLSNPLLPPTSLAFENQAWLQTGPDPRMLDVRARKYGLDKDFHAAHFAHEGIEVWDLLGR
Sbjct  61    PLSNPLLPPTSLAFENQAWLQTGPDPRMLDVRARKYGLDKDFHAAHFAHEGIEVWDLLGR  120

Query  121   KALSTAPPLPPPIRSESVKPLTTNHPMTSQGARTGKRTEREVRDLKAQSVSTFTASSSIS  180
             KALSTAPPLPPPIRSESVKPLTTNHPMTSQGARTGKRTEREVRDLKAQSVSTFTASSSIS
Sbjct  121   KALSTAPPLPPPIRSESVKPLTTNHPMTSQGARTGKRTEREVRDLKAQSVSTFTASSSIS  180

Query  181   DLPGTSPSRYQPLSSNHPLHSSVEPHPPSIPISSSLDIPARQPPTASIDSLSPASTPDAI  240
             DLPGTSPSRYQPLSSNHPLHSSVEPHPPSIPISSSLDIPARQPPTASIDSLSPASTPDAI
Sbjct  181   DLPGTSPSRYQPLSSNHPLHSSVEPHPPSIPISSSLDIPARQPPTASIDSLSPASTPDAI  240

Query  241   PSIQASSSTSSLSPSHIPDVLPELASHTTTRSSKSLPHLQTTLPSTPDPLTHTSSVGRST  300
             PSIQASSSTSSLSPSHIPDVLPELASHTTTRSSKSLPHLQTTLPSTPDPLTHTSSVGRST
Sbjct  241   PSIQASSSTSSLSPSHIPDVLPELASHTTTRSSKSLPHLQTTLPSTPDPLTHTSSVGRST  300

Query  301   RTKRSTRKSPANDLTSPQSDLFRIALSGATSPTPPVPPRSTHISDTKHYFNQRIRPPLRK  360
             RTKRSTRKSPANDLTSPQSDLFRIALSGATSPTPPVPPRSTHISDTKHYFNQRIRPPLRK
Sbjct  301   RTKRSTRKSPANDLTSPQSDLFRIALSGATSPTPPVPPRSTHISDTKHYFNQRIRPPLRK  360

Query  361   KLSHISLPPTTASLADLQYILPASAENKAPVDQSHSSSSAGKAIRQYCLEDKTAEAIRLF  420
             KLSHISLPPTTASLADLQYILPASAENKAPVDQSHSSSSAGKAIRQYCLEDKTAEAIRLF
Sbjct  361   KLSHISLPPTTASLADLQYILPASAENKAPVDQSHSSSSAGKAIRQYCLEDKTAEAIRLF  420

Query  421   EKVYPGHSPSMSSSYSLASSQPSSSQQPSINLYLFIINALIRTNQHALAQGWMSLTTSKQ  480
             EKVYPGHSPSMSSSYSLASSQPSSSQQPSINLYLFIINALIRTNQHALAQGWMSLTTSKQ
Sbjct  421   EKVYPGHSPSMSSSYSLASSQPSSSQQPSINLYLFIINALIRTNQHALAQGWMSLTTSKQ  480

Query  481   GAEYSTETYLALFRLFTWSRDHAAAHELYNRMMALRSNPLEDKTFARTVMRFLMRAGPAE  540
             GAEYSTETYLALFRLFTWSRDHAAAHELYNRMMALRSNPLEDKTFARTVMRFLMRAGPAE
Sbjct  481   GAEYSTETYLALFRLFTWSRDHAAAHELYNRMMALRSNPLEDKTFARTVMRFLMRAGPAE  540

Query  541   KDSSISWYWLREMQAKEIPIGFEEWTIILRAHAYASELDHVQSVWRLARETAPISKDSPI  600
             KDSSISWYWLREMQAKEIPIGFEEWTIILRAHAYASELDHVQSVWRLARETAPISKDSPI
Sbjct  541   KDSSISWYWLREMQAKEIPIGFEEWTIILRAHAYASELDHVQSVWRLARETAPISKDSPI  600

Query  601   KERVDYTRFLTTYLDAHRRMGGSWEELRELAREFLADRAVIPDGSLNLSLMRAACKSGFL  660
             KERVDYTRFLTTYLDAHRRMGGSWEELRELAREFLADRAVIPDGSLNLSLMRAACKSGFL
Sbjct  601   KERVDYTRFLTTYLDAHRRMGGSWEELRELAREFLADRAVIPDGSLNLSLMRAACKSGFL  660

Query  661   DDARQIMKIMEDRAGIAPDSRHFTTLIVELINPNLPPQPGASKAVAALPLTKSAVEKPLS  720
             DDARQIMKIMEDRAGIAPDSRHFTTLIVELINPNLPPQPGASKAVAALPLTKSAVEKPLS
Sbjct  661   DDARQIMKIMEDRAGIAPDSRHFTTLIVELINPNLPPQPGASKAVAALPLTKSAVEKPLS  720

Query  721   GSQRVKRTARQLSREIFALIGEMQARHLSLEYPVYINIIRYLSSDVSTKPKAIELCRRLI  780
             GSQRVKRTARQLSREIFALIGEMQARHLSLEYPVYINIIRYLSSDVSTKPKAIELCRRLI
Sbjct  721   GSQRVKRTARQLSREIFALIGEMQARHLSLEYPVYINIIRYLSSDVSTKPKAIELCRRLI  780

Query  781   STLPGPGEGWSTENKWAKRWSINSPDPVTGIFTELVNAYAAQTQPGLVLETYEQLKGKKA  840
             STLPGPGEGWSTENKWAKRWSINSPDPVTGIFTELVNAYAAQTQPGLVLETYEQLKGKKA
Sbjct  781   STLPGPGEGWSTENKWAKRWSINSPDPVTGIFTELVNAYAAQTQPGLVLETYEQLKGKKA  840

Query  841   WEVSLKIVGTLLDRVGSTISIKEQELLWRSAVVLAQEEVESSSSVERRRLKRQLDNGGLP  900
             WEVSLKIVGTLLDRVGSTISIKEQELLWRSAVVLAQEEVESSSSVERRRLKRQLDNGGLP
Sbjct  841   WEVSLKIVGTLLDRVGSTISIKEQELLWRSAVVLAQEEVESSSSVERRRLKRQLDNGGLP  900

Query  901   VKEDPSITAAGVRAPFANSSSSLTDSLIDPSALAPRTPASPPNVANTTTRTPIALPTGSY  960
             VKEDPSITAAGVRAPFANSSSSLTDSLIDPSALAPRTPASPPNVANTTTRTPIALPTGSY
Sbjct  901   VKEDPSITAAGVRAPFANSSSSLTDSLIDPSALAPRTPASPPNVANTTTRTPIALPTGSY  960

Query  961   KPPSSLLSRPLTAYIRSLLSVPSRPGTYQLRYITEQCLGITKLGFGLDSTCWSYLALAYV  1020
             KPPSSLLSRPLTAYIRSLLSVPSRPGTYQLRYITEQCLGITKLGFGLDSTCWSYLALAYV
Sbjct  961   KPPSSLLSRPLTAYIRSLLSVPSRPGTYQLRYITEQCLGITKLGFGLDSTCWSYLALAYV  1020

Query  1021  EAGLLDEAFFVLNDVILFKHEHIRRQYEAWTGRTLEGETLRESRARLLDKRQLFKGDFLC  1080
             EAGLLDEAFFVLNDVILFKHEHIRRQYEAWTGRTLEGETLRESRARLLDKRQLFKGDFLC
Sbjct  1021  EAGLLDEAFFVLNDVILFKHEHIRRQYEAWTGRTLEGETLRESRARLLDKRQLFKGDFLC  1080

Query  1081  PSIERHTPEFKEMTLTSYRMFTPDKVLRRIKEINDMNRLRLPWSPDQRRSFDLQSGSHGA  1140
             PSIERHTPEFKEMTLTSYRMFTPDKVLRRIKEINDMNRLRLPWSPDQRRSFDLQSGSHGA
Sbjct  1081  PSIERHTPEFKEMTLTSYRMFTPDKVLRRIKEINDMNRLRLPWSPDQRRSFDLQSGSHGA  1140

Query  1141  STTRSDDGDLIFLTESDLSLSSDGPTSATLTMSPTAGQRSLLTAQGFDPKSLAPGLWLPH  1200
             STTRSDDGDLIFLTESDLSLSSDGPTSATLTMSPTAGQRSLLTAQGFDPKSLAPGLWLPH
Sbjct  1141  STTRSDDGDLIFLTESDLSLSSDGPTSATLTMSPTAGQRSLLTAQGFDPKSLAPGLWLPH  1200

Query  1201  RSILSALSSIVKEFEARAVDLETDALLRRASGKEDPRGKLMEWQTTYRRAWRFIELWRRR  1260
             RSILSALSSIVKEFEARAVDLETDALLRRASGKEDPRGKLMEWQTTYRRAWRFIELWRRR
Sbjct  1201  RSILSALSSIVKEFEARAVDLETDALLRRASGKEDPRGKLMEWQTTYRRAWRFIELWRRR  1260

Query  1261  GHDRGGR  1267
             GHDRGGR
Sbjct  1261  GHDRGGR  1267


Lambda      K        H        a         alpha
   0.316    0.130    0.383    0.792     4.96 

Gapped
Lambda      K        H        a         alpha    sigma
   0.267   0.0410    0.140     1.90     42.6     43.6 

Effective search space used: 15901861463670


  Database: nr
    Posted date:  Sep 23, 2015 12:05 AM
  Number of letters in database: 26,053,659,533
  Number of sequences in database:  71,551,133


Matrix: BLOSUM62
Gap Penalties: Existence: 11, Extension: 1
Neighboring words threshold: 11
Window for multiple hits: 40
```
