# Supplementary material for: Comparative genomics provides new insights into the diversity, physiology, and sexuality of the only industrially exploited tremellomycete: Phaffia rhodozyma
Source: BMC Genomics. 2016 Nov 9;17:901. doi: 10.1186/s12864-016-3244-7 (PMC5103461; doi:10.1186/s12864-016-3244-7)
Supplement: Additional file 6: — List of orphan genes with links to PFAM (related to Additional file 1: Table S1). (ZIP 1428 kb) [file 12864_2016_3244_MOESM6_ESM.zip › BLAST_HTML_FTR/G04446_P.html]

BLAST Search Results


```
BLASTP 2.2.27+


Reference:
Stephen F. Altschul, Thomas L. Madden, Alejandro A. Schäffer,
Jinghui Zhang, Zheng Zhang, Webb Miller, and David J. Lipman (1997),
"Gapped BLAST and PSI-BLAST: a new generation of protein database
search programs", Nucleic Acids Res. 25:3389-3402.


Reference for
composition-based statistics:
Alejandro A. Schäffer, L. Aravind, Thomas L. Madden, Sergei
Shavirin, John L. Spouge, Yuri I. Wolf, Eugene V. Koonin, and
Stephen F. Altschul (2001), "Improving the accuracy of PSI-BLAST
protein database searches with composition-based statistics and
other refinements", Nucleic Acids Res. 29:2994-3005.


Database: nr
           71,551,133 sequences; 26,053,659,533 total letters


Query= G04446_P

Length=129
                                                                      Score     E
Sequences producing significant alignments:                          (Bits)  Value

emb|CED82665.1|  hypothetical protein [Xanthophyllomyces dendrorh...   239    3e-78
gb|KNB42508.1|  hypothetical protein JH06_3844, partial [Blastocy...  40.0    0.27 
ref|WP_033684374.1|  amylopullulanase [Streptococcus mitis] >gb|K...  39.3    0.57 


 >emb|CED82665.1| hypothetical protein [Xanthophyllomyces dendrorhous]
Length=128

 Score =  239 bits (610),  Expect = 3e-78, Method: Compositional matrix adjust.
 Identities = 128/128 (100%), Positives = 128/128 (100%), Gaps = 0/128 (0%)

Query  1    MFTTLASTTTTTTAALLPKRLKRWTRPSCRTSSSASGSSSEDELTRPANLTPRERRPIEP  60
            MFTTLASTTTTTTAALLPKRLKRWTRPSCRTSSSASGSSSEDELTRPANLTPRERRPIEP
Sbjct  1    MFTTLASTTTTTTAALLPKRLKRWTRPSCRTSSSASGSSSEDELTRPANLTPRERRPIEP  60

Query  61   TSPLVTSPTEFIPSTFSRSSPMSRSTRPKLSRADTPTPLSPRSSQAIGVNLRVGGWIGVP  120
            TSPLVTSPTEFIPSTFSRSSPMSRSTRPKLSRADTPTPLSPRSSQAIGVNLRVGGWIGVP
Sbjct  61   TSPLVTSPTEFIPSTFSRSSPMSRSTRPKLSRADTPTPLSPRSSQAIGVNLRVGGWIGVP  120

Query  121  SSSLITSP  128
            SSSLITSP
Sbjct  121  SSSLITSP  128


>gb|KNB42508.1| hypothetical protein JH06_3844, partial [Blastocystis sp. ST4]
Length=1136

 Score = 40.0 bits (92),  Expect = 0.27, Method: Composition-based stats.
 Identities = 36/107 (34%), Positives = 51/107 (48%), Gaps = 14/107 (13%)

Query  15   ALLPKRLKRWTRPSCRTSSSASGSSSEDELTRPAN--LTPRERRP----IEPTSPLVTSP  68
            A + +  K W  PSCR +  AS +S     TRP +  L+PR R P    ++P+ PLV   
Sbjct  570  AYMNEHFKNWLCPSCRDTKEASVTSHSRSSTRPGSPLLSPRLRPPTTPELKPSFPLVLE-  628

Query  69   TEFIPSTFSRSSPMSRSTRPKLSRADTPTPLSPRSSQAIGVNLRVGG  115
                 S  +  S   R + P L  A +P P+SP   +  G +L  GG
Sbjct  629  ----SSPENADSSQRRDSIPVL--APSP-PISPLIRKPAGTDLSHGG  668


>ref|WP_033684374.1| amylopullulanase [Streptococcus mitis]
 gb|KEQ39540.1| putative pullulanase [Streptococcus mitis]
Length=1298

 Score = 39.3 bits (90),  Expect = 0.57, Method: Composition-based stats.
 Identities = 36/103 (35%), Positives = 47/103 (46%), Gaps = 18/103 (17%)

Query  3    TTLASTTTTTTAALLPKRLKRWTRPSCRTSSSASGSSSEDELTRPAN----LTPRERRP-  57
            TT AS + TTTA   P            T ++AS    E +L+ P N    L  +E +P 
Sbjct  50   TTNASNSETTTALAQP-----------LTDTTASTGKQESDLSVPKNANASLEKKEEKPA  98

Query  58   IEPTSPLVTSPTEFIPSTFS-RSSPMSRSTRPKLSRADTPTPL  99
             EPTSP   SP +  P T   RSS  + STRP  +      P+
Sbjct  99   TEPTSP-AASPADSAPQTGQDRSSEPTTSTRPVTTETKVEEPI  140


Lambda      K        H        a         alpha
   0.314    0.125    0.366    0.792     4.96 

Gapped
Lambda      K        H        a         alpha    sigma
   0.267   0.0410    0.140     1.90     42.6     43.6 

Effective search space used: 654714264532


  Database: nr
    Posted date:  Sep 23, 2015 12:05 AM
  Number of letters in database: 26,053,659,533
  Number of sequences in database:  71,551,133


Matrix: BLOSUM62
Gap Penalties: Existence: 11, Extension: 1
Neighboring words threshold: 11
Window for multiple hits: 40
```
