# Supplementary material for: Comparative genomics provides new insights into the diversity, physiology, and sexuality of the only industrially exploited tremellomycete: Phaffia rhodozyma
Source: BMC Genomics. 2016 Nov 9;17:901. doi: 10.1186/s12864-016-3244-7 (PMC5103461; doi:10.1186/s12864-016-3244-7)
Supplement: Additional file 6: — List of orphan genes with links to PFAM (related to Additional file 1: Table S1). (ZIP 1428 kb) [file 12864_2016_3244_MOESM6_ESM.zip › BLAST_HTML_FTR/G02767_P.html]

BLAST Search Results


```
BLASTP 2.2.27+


Reference:
Stephen F. Altschul, Thomas L. Madden, Alejandro A. Schäffer,
Jinghui Zhang, Zheng Zhang, Webb Miller, and David J. Lipman (1997),
"Gapped BLAST and PSI-BLAST: a new generation of protein database
search programs", Nucleic Acids Res. 25:3389-3402.


Reference for
composition-based statistics:
Alejandro A. Schäffer, L. Aravind, Thomas L. Madden, Sergei
Shavirin, John L. Spouge, Yuri I. Wolf, Eugene V. Koonin, and
Stephen F. Altschul (2001), "Improving the accuracy of PSI-BLAST
protein database searches with composition-based statistics and
other refinements", Nucleic Acids Res. 29:2994-3005.


Database: nr
           71,551,133 sequences; 26,053,659,533 total letters


Query= G02767_P

Length=1031
                                                                      Score     E
Sequences producing significant alignments:                          (Bits)  Value

emb|CED84195.1|  Armadillo-like helical [Xanthophyllomyces dendro...  2084    0.0  


 >emb|CED84195.1| Armadillo-like helical [Xanthophyllomyces dendrorhous]
Length=1040

 Score = 2084 bits (5400),  Expect = 0.0, Method: Compositional matrix adjust.
 Identities = 1030/1030 (100%), Positives = 1030/1030 (100%), Gaps = 0/1030 (0%)

Query  1     MDGDETLGSPLLMRRTITNVENNLIAQPIESPETTTTTPTPGFLPPPLTQGNYNESLSGN  60
             MDGDETLGSPLLMRRTITNVENNLIAQPIESPETTTTTPTPGFLPPPLTQGNYNESLSGN
Sbjct  11    MDGDETLGSPLLMRRTITNVENNLIAQPIESPETTTTTPTPGFLPPPLTQGNYNESLSGN  70

Query  61    HSSSTNSSSDPLGLFVKRPVPLRQSGCPPVSYQPVSCGDPNIACDPYKLTRTPSLSSSSS  120
             HSSSTNSSSDPLGLFVKRPVPLRQSGCPPVSYQPVSCGDPNIACDPYKLTRTPSLSSSSS
Sbjct  71    HSSSTNSSSDPLGLFVKRPVPLRQSGCPPVSYQPVSCGDPNIACDPYKLTRTPSLSSSSS  130

Query  121   YGVDVSSPTCLSDGLSSSDGLYSPESVSPIAIQQNLNQSAIFGPAPSQMSLLSQILLSHS  180
             YGVDVSSPTCLSDGLSSSDGLYSPESVSPIAIQQNLNQSAIFGPAPSQMSLLSQILLSHS
Sbjct  131   YGVDVSSPTCLSDGLSSSDGLYSPESVSPIAIQQNLNQSAIFGPAPSQMSLLSQILLSHS  190

Query  181   HQSKPLSYRTPNNSNPTTAHHSLQSTPLSSNTSSPAGYGNIRQLKGRRMSNGACDILDQV  240
             HQSKPLSYRTPNNSNPTTAHHSLQSTPLSSNTSSPAGYGNIRQLKGRRMSNGACDILDQV
Sbjct  191   HQSKPLSYRTPNNSNPTTAHHSLQSTPLSSNTSSPAGYGNIRQLKGRRMSNGACDILDQV  250

Query  241   GEEDGEAVDCPSNILDLHPSDVRGGPEKEESSLGLVPSDYPSPAPVHIRFGEAAVLPKRP  300
             GEEDGEAVDCPSNILDLHPSDVRGGPEKEESSLGLVPSDYPSPAPVHIRFGEAAVLPKRP
Sbjct  251   GEEDGEAVDCPSNILDLHPSDVRGGPEKEESSLGLVPSDYPSPAPVHIRFGEAAVLPKRP  310

Query  301   PLPLSDFAPPVTILASLAKTQSTDATVSKKPTLKFADSGSTTPLSMPSAQHSTAHSETSS  360
             PLPLSDFAPPVTILASLAKTQSTDATVSKKPTLKFADSGSTTPLSMPSAQHSTAHSETSS
Sbjct  311   PLPLSDFAPPVTILASLAKTQSTDATVSKKPTLKFADSGSTTPLSMPSAQHSTAHSETSS  370

Query  361   RSSPAPVPPEVTCVPVKKPQLTFAVATTSARPAMLAKESKELMKSLQDSGDEDDDPDEEG  420
             RSSPAPVPPEVTCVPVKKPQLTFAVATTSARPAMLAKESKELMKSLQDSGDEDDDPDEEG
Sbjct  371   RSSPAPVPPEVTCVPVKKPQLTFAVATTSARPAMLAKESKELMKSLQDSGDEDDDPDEEG  430

Query  421   DEDEDEDEDEEDDEDDDDDDDDDEEDIEDDEEDEDEDGKEEEDEVEQVDGFDVEGDGYEE  480
             DEDEDEDEDEEDDEDDDDDDDDDEEDIEDDEEDEDEDGKEEEDEVEQVDGFDVEGDGYEE
Sbjct  431   DEDEDEDEDEEDDEDDDDDDDDDEEDIEDDEEDEDEDGKEEEDEVEQVDGFDVEGDGYEE  490

Query  481   DDEGEGLQDDEGISAPIGRKYRFKRESLFGQSDSEDDELLEDEQVGTLRSSRPTGGFPPD  540
             DDEGEGLQDDEGISAPIGRKYRFKRESLFGQSDSEDDELLEDEQVGTLRSSRPTGGFPPD
Sbjct  491   DDEGEGLQDDEGISAPIGRKYRFKRESLFGQSDSEDDELLEDEQVGTLRSSRPTGGFPPD  550

Query  541   SYRQIDQSRDQQSGTPKLQQGWVETTLKKRRSSLLTLHPNNASELAFSVSRAESSIPSSH  600
             SYRQIDQSRDQQSGTPKLQQGWVETTLKKRRSSLLTLHPNNASELAFSVSRAESSIPSSH
Sbjct  551   SYRQIDQSRDQQSGTPKLQQGWVETTLKKRRSSLLTLHPNNASELAFSVSRAESSIPSSH  610

Query  601   RGDSTCQQFQTPVLSFITTGRNRRSAYSPRRGGFKGGYSPGLKKRNTSVASSPRTRVDRD  660
             RGDSTCQQFQTPVLSFITTGRNRRSAYSPRRGGFKGGYSPGLKKRNTSVASSPRTRVDRD
Sbjct  611   RGDSTCQQFQTPVLSFITTGRNRRSAYSPRRGGFKGGYSPGLKKRNTSVASSPRTRVDRD  670

Query  661   DFLPSHLKSEKGKEKERMAERKGREVATSSRSGTEGGSGTAGRCSRHRSPPPSGRECRKS  720
             DFLPSHLKSEKGKEKERMAERKGREVATSSRSGTEGGSGTAGRCSRHRSPPPSGRECRKS
Sbjct  671   DFLPSHLKSEKGKEKERMAERKGREVATSSRSGTEGGSGTAGRCSRHRSPPPSGRECRKS  730

Query  721   GGERREGSRSQTSKAPQRAQTSELAITRPAALPLPPSPILRNPYEFTNPVAPKQVNIYGI  780
             GGERREGSRSQTSKAPQRAQTSELAITRPAALPLPPSPILRNPYEFTNPVAPKQVNIYGI
Sbjct  731   GGERREGSRSQTSKAPQRAQTSELAITRPAALPLPPSPILRNPYEFTNPVAPKQVNIYGI  790

Query  781   RSTSYSVPRRAPDGHLTPPIFLTDTENWMDIQAISHNRPGRLSERQTSLSAVSSAFRQCT  840
             RSTSYSVPRRAPDGHLTPPIFLTDTENWMDIQAISHNRPGRLSERQTSLSAVSSAFRQCT
Sbjct  791   RSTSYSVPRRAPDGHLTPPIFLTDTENWMDIQAISHNRPGRLSERQTSLSAVSSAFRQCT  850

Query  841   STCESDDPSAAKRRNSYSLKVDVTQAVRQAKSDVWSDGAVSDHPSDVAGALTQALQQKLA  900
             STCESDDPSAAKRRNSYSLKVDVTQAVRQAKSDVWSDGAVSDHPSDVAGALTQALQQKLA
Sbjct  851   STCESDDPSAAKRRNSYSLKVDVTQAVRQAKSDVWSDGAVSDHPSDVAGALTQALQQKLA  910

Query  901   QQPLSPVSRPFAPPSTSFLKRHTSLLGTIAIAQPQQRLQPVPCIIPELSSPETLLPSTNQ  960
             QQPLSPVSRPFAPPSTSFLKRHTSLLGTIAIAQPQQRLQPVPCIIPELSSPETLLPSTNQ
Sbjct  911   QQPLSPVSRPFAPPSTSFLKRHTSLLGTIAIAQPQQRLQPVPCIIPELSSPETLLPSTNQ  970

Query  961   PLHGTTNHKALKTTIVHSKTANTTPINLDPSLSLPLQKRSVSQPPLVKSKPIPCPAAKVP  1020
             PLHGTTNHKALKTTIVHSKTANTTPINLDPSLSLPLQKRSVSQPPLVKSKPIPCPAAKVP
Sbjct  971   PLHGTTNHKALKTTIVHSKTANTTPINLDPSLSLPLQKRSVSQPPLVKSKPIPCPAAKVP  1030

Query  1021  GDWDGLAHDF  1030
             GDWDGLAHDF
Sbjct  1031  GDWDGLAHDF  1040


Lambda      K        H        a         alpha
   0.309    0.127    0.367    0.792     4.96 

Gapped
Lambda      K        H        a         alpha    sigma
   0.267   0.0410    0.140     1.90     42.6     43.6 

Effective search space used: 12644516594400


  Database: nr
    Posted date:  Sep 23, 2015 12:05 AM
  Number of letters in database: 26,053,659,533
  Number of sequences in database:  71,551,133


Matrix: BLOSUM62
Gap Penalties: Existence: 11, Extension: 1
Neighboring words threshold: 11
Window for multiple hits: 40
```
