# Supplementary material for: Comparative genomics provides new insights into the diversity, physiology, and sexuality of the only industrially exploited tremellomycete: Phaffia rhodozyma
Source: BMC Genomics. 2016 Nov 9;17:901. doi: 10.1186/s12864-016-3244-7 (PMC5103461; doi:10.1186/s12864-016-3244-7)
Supplement: Additional file 6: — List of orphan genes with links to PFAM (related to Additional file 1: Table S1). (ZIP 1428 kb) [file 12864_2016_3244_MOESM6_ESM.zip › BLAST_HTML_FTR/G01789_P.html]

BLAST Search Results


```
BLASTP 2.2.27+


Reference:
Stephen F. Altschul, Thomas L. Madden, Alejandro A. Schäffer,
Jinghui Zhang, Zheng Zhang, Webb Miller, and David J. Lipman (1997),
"Gapped BLAST and PSI-BLAST: a new generation of protein database
search programs", Nucleic Acids Res. 25:3389-3402.


Reference for
composition-based statistics:
Alejandro A. Schäffer, L. Aravind, Thomas L. Madden, Sergei
Shavirin, John L. Spouge, Yuri I. Wolf, Eugene V. Koonin, and
Stephen F. Altschul (2001), "Improving the accuracy of PSI-BLAST
protein database searches with composition-based statistics and
other refinements", Nucleic Acids Res. 29:2994-3005.


Database: nr
           71,551,133 sequences; 26,053,659,533 total letters


Query= G01789_P

Length=145
                                                                      Score     E
Sequences producing significant alignments:                          (Bits)  Value

emb|CDZ96993.1|  hypothetical protein [Xanthophyllomyces dendrorh...   290    4e-98
gb|KNE96238.1|  hypothetical protein PSTG_10501 [Puccinia striifo...  40.0    0.42 
ref|XP_006797936.1|  PREDICTED: abnormal spindle-like microcephal...  38.5    1.4  


 >emb|CDZ96993.1| hypothetical protein [Xanthophyllomyces dendrorhous]
Length=144

 Score =  290 bits (743),  Expect = 4e-98, Method: Compositional matrix adjust.
 Identities = 144/144 (100%), Positives = 144/144 (100%), Gaps = 0/144 (0%)

Query  1    MPLRRTKTVYTSLDSTLSCPSSPESGRFPLPVSVKNWAQINSSCSNSPFVPSSSANPNRG  60
            MPLRRTKTVYTSLDSTLSCPSSPESGRFPLPVSVKNWAQINSSCSNSPFVPSSSANPNRG
Sbjct  1    MPLRRTKTVYTSLDSTLSCPSSPESGRFPLPVSVKNWAQINSSCSNSPFVPSSSANPNRG  60

Query  61   PSDPCSSFPHSRLQTEHTDRHTYTCPQFSLELASHSILRPTEDGSIYFSPKHPQLYSRSR  120
            PSDPCSSFPHSRLQTEHTDRHTYTCPQFSLELASHSILRPTEDGSIYFSPKHPQLYSRSR
Sbjct  61   PSDPCSSFPHSRLQTEHTDRHTYTCPQFSLELASHSILRPTEDGSIYFSPKHPQLYSRSR  120

Query  121  PYPRGSGSDGAEEYFPAFSRGVTA  144
            PYPRGSGSDGAEEYFPAFSRGVTA
Sbjct  121  PYPRGSGSDGAEEYFPAFSRGVTA  144


>gb|KNE96238.1| hypothetical protein PSTG_10501 [Puccinia striiformis f. sp. 
tritici PST-78]
Length=1265

 Score = 40.0 bits (92),  Expect = 0.42, Method: Compositional matrix adjust.
 Identities = 28/99 (28%), Positives = 46/99 (46%), Gaps = 6/99 (6%)

Query  30   LPVSVKNWAQINSSCSNSPFVPSSSANPNRGPSDPCSSFPHSR----LQTEHTDRHTYTC  85
            L  S+K   ++     NS  +PS +  P + P+DP ++ P S     + TE  D  T T 
Sbjct  574  LVQSLKAHIEMTDQLLNSLTIPSGATTPQQAPADPLNTSPGSSSTAGINTEDKD-QTATA  632

Query  86   PQFSLELASHSILRPTEDGSIYFSPKHPQLYSRSRPYPR  124
             Q  +E+  H++ R  ED  + F     ++  R R + R
Sbjct  633  SQRQIEV-KHALKRSVEDLGVMFDDYLTKINKRERYHIR  670


>ref|XP_006797936.1| PREDICTED: abnormal spindle-like microcephaly-associated protein 
homolog [Neolamprologus brichardi]
Length=2591

 Score = 38.5 bits (88),  Expect = 1.4, Method: Composition-based stats.
 Identities = 35/115 (30%), Positives = 49/115 (43%), Gaps = 12/115 (10%)

Query  14   DSTLSCPSSPESGRFPLPVSVKNWAQINSSCSNSPFVP---SSSANPNRGPSDPCSSFPH  70
            D +  C S PES   P+  S K      S C + P VP   SSS +P    S    +FP 
Sbjct  469  DGSCPCESGPESPSLPVIDSDKGI----SPCDDKPQVPEFISSSTSPRLAASPAPITFPV  524

Query  71   SRLQTEHTDRHTYTC---PQFSLELASHSILRPTEDGSIYFSPKHPQLYSRSRPY  122
            S        R +++    P   L   + ++  PT  GS +  P H  L S+S+ Y
Sbjct  525  SSPPPLAPSRFSFSVTSPPHSVLAPVAFTVPSPTPVGSSF--PHHHDLLSKSKVY  577


Lambda      K        H        a         alpha
   0.317    0.131    0.412    0.792     4.96 

Gapped
Lambda      K        H        a         alpha    sigma
   0.267   0.0410    0.140     1.90     42.6     43.6 

Effective search space used: 636406221605


  Database: nr
    Posted date:  Sep 23, 2015 12:05 AM
  Number of letters in database: 26,053,659,533
  Number of sequences in database:  71,551,133


Matrix: BLOSUM62
Gap Penalties: Existence: 11, Extension: 1
Neighboring words threshold: 11
Window for multiple hits: 40
```
