# Supplementary material for: Comparative genomics provides new insights into the diversity, physiology, and sexuality of the only industrially exploited tremellomycete: Phaffia rhodozyma
Source: BMC Genomics. 2016 Nov 9;17:901. doi: 10.1186/s12864-016-3244-7 (PMC5103461; doi:10.1186/s12864-016-3244-7)
Supplement: Additional file 6: — List of orphan genes with links to PFAM (related to Additional file 1: Table S1). (ZIP 1428 kb) [file 12864_2016_3244_MOESM6_ESM.zip › BLAST_HTML_FTR/G03825_P.html]

BLAST Search Results


```
BLASTP 2.2.27+


Reference:
Stephen F. Altschul, Thomas L. Madden, Alejandro A. Schäffer,
Jinghui Zhang, Zheng Zhang, Webb Miller, and David J. Lipman (1997),
"Gapped BLAST and PSI-BLAST: a new generation of protein database
search programs", Nucleic Acids Res. 25:3389-3402.


Reference for
composition-based statistics:
Alejandro A. Schäffer, L. Aravind, Thomas L. Madden, Sergei
Shavirin, John L. Spouge, Yuri I. Wolf, Eugene V. Koonin, and
Stephen F. Altschul (2001), "Improving the accuracy of PSI-BLAST
protein database searches with composition-based statistics and
other refinements", Nucleic Acids Res. 29:2994-3005.


Database: nr
           71,551,133 sequences; 26,053,659,533 total letters


Query= G03825_P

Length=399
                                                                      Score     E
Sequences producing significant alignments:                          (Bits)  Value

emb|CED83689.1|  hypothetical protein [Xanthophyllomyces dendrorh...   748    0.0  
ref|WP_013353670.1|  MULTISPECIES: RpiR family transcriptional re...  47.4    0.014
ref|WP_045512145.1|  RpiR family transcriptional regulator [Bacil...  47.4    0.015
ref|WP_045208952.1|  RpiR family transcriptional regulator [Bacil...  42.7    0.38 
ref|WP_025853459.1|  RpiR family transcriptional regulator [Bacil...  42.7    0.39 
gb|KJD58719.1|  RpiR family transcriptional regulator [Bacillus a...  42.7    0.40 
ref|WP_007613885.1|  MULTISPECIES: RpiR family transcriptional re...  42.7    0.41 
ref|WP_052827874.1|  RpiR family transcriptional regulator [Bacil...  42.7    0.42 
ref|WP_004009887.1|  DNA helicase UvrD [Mobiluncus curtisii] >gb|...  39.7    7.3  
gb|KNZ46545.1|  hypothetical protein VP01_717g3 [Puccinia sorghi]     38.1    9.5  


 >emb|CED83689.1| hypothetical protein [Xanthophyllomyces dendrorhous]
Length=429

 Score =  748 bits (1931),  Expect = 0.0, Method: Compositional matrix adjust.
 Identities = 398/425 (94%), Positives = 398/425 (94%), Gaps = 27/425 (6%)

Query  1    MLCSSPRDLSPIHHFGNPFGSTHTSLHKQRANLEVKKPMSLGRISPLCSRLASTSQTSLI  60
            MLCSSPRDLSPIHHFGNPFGSTHTSLHKQRANLEVKKPMSLGRISPLCSRLASTSQTSLI
Sbjct  5    MLCSSPRDLSPIHHFGNPFGSTHTSLHKQRANLEVKKPMSLGRISPLCSRLASTSQTSLI  64

Query  61   RRSQVIRLYSTPCSSSSSSSSSVGEATPTGKKPRKASWSRTRHAERDYRALGLPPSKIES  120
            RRSQVIRLYSTPCSSSSSSSSSVGEATPTGKKPRKASWSRTRHAERDYRALGLPPSKIES
Sbjct  65   RRSQVIRLYSTPCSSSSSSSSSVGEATPTGKKPRKASWSRTRHAERDYRALGLPPSKIES  124

Query  121  YERVLKKPKAVQFAVDPTRESVGPERKQRKSAKAIRKERLRGQEVEVRDYLKEKEKEERT  180
            YERVLKKPKAVQFAVDPTRESVGPERKQRKSAKAIRKERLRGQEVEVRDYLKEKEKEERT
Sbjct  125  YERVLKKPKAVQFAVDPTRESVGPERKQRKSAKAIRKERLRGQEVEVRDYLKEKEKEERT  184

Query  181  KRLNTLEDTS---------------------------TLPDYERETPYPAHKVCEWILAH  213
            KRLNTLEDTS                           TLPDYERETPYPAHKVCEWILAH
Sbjct  185  KRLNTLEDTSFARRRNGWCWEWFQEGKDLGDIGNMVSTLPDYERETPYPAHKVCEWILAH  244

Query  214  LSLPPRSDPSSPTEPGRESIQEPTDSFHLLTPESWARLSAPHAFYSSQSYPDLINSRINR  273
            LSLPPRSDPSSPTEPGRESIQEPTDSFHLLTPESWARLSAPHAFYSSQSYPDLINSRINR
Sbjct  245  LSLPPRSDPSSPTEPGRESIQEPTDSFHLLTPESWARLSAPHAFYSSQSYPDLINSRINR  304

Query  274  IRSTDSHIYDLLYSRGWRGEGLGEPLGASDGIESKDVVDSVRTKDEVGLGIRGIDRVPIP  333
            IRSTDSHIYDLLYSRGWRGEGLGEPLGASDGIESKDVVDSVRTKDEVGLGIRGIDRVPIP
Sbjct  305  IRSTDSHIYDLLYSRGWRGEGLGEPLGASDGIESKDVVDSVRTKDEVGLGIRGIDRVPIP  364

Query  334  MNVLRKFIDRADQDKLTFEDEIARRRDESAVEPETSRIIRVLDALRRKEFKDEAMRQKKE  393
            MNVLRKFIDRADQDKLTFEDEIARRRDESAVEPETSRIIRVLDALRRKEFKDEAMRQKKE
Sbjct  365  MNVLRKFIDRADQDKLTFEDEIARRRDESAVEPETSRIIRVLDALRRKEFKDEAMRQKKE  424

Query  394  RSSIE  398
            RSSIE
Sbjct  425  RSSIE  429


>ref|WP_013353670.1| MULTISPECIES: RpiR family transcriptional regulator [Bacillus 
subtilis group]
 emb|CBI44390.1| Uncharacterized HTH-type transcriptional regulator RBAM_031420 
[Bacillus amyloliquefaciens DSM 7]
 gb|AEB25618.1| hypothetical protein BAMTA208_17330 [Bacillus amyloliquefaciens 
TA208]
 gb|AEB65081.1| Uncharacterized HTH-type transcriptional regulator [Bacillus 
amyloliquefaciens LL3]
 gb|AEK90654.1| RpiR family transcriptional regulator [Bacillus amyloliquefaciens 
XH7]
 gb|AIW35264.1| RpiR family transcriptional regulator [Bacillus subtilis]
Length=285

 Score = 47.4 bits (111),  Expect = 0.014, Method: Compositional matrix adjust.
 Identities = 36/112 (32%), Positives = 53/112 (47%), Gaps = 17/112 (15%)

Query  230  RESIQEPTDSFHLLTPESWAR-LSAPHA-----FY--------SSQSYPDLINSRINRIR  275
            R +I   TD+FHLL P    + +   H+     FY        ++ +Y   + + IN I 
Sbjct  106  RANISGLTDTFHLLDPADVEKAVEMIHSADRIEFYGNGGSGLIATDAYHKFMRTGINCIA  165

Query  276  STDSHIYDLLYSRGWRGEGLGEPLGASDGIESKDVVDSVRTKDEVGLGIRGI  327
             TDSH   +  S G  G G    +G S    +KDV+D+V+T   +G G  GI
Sbjct  166  HTDSHFQAM--SAGLLGPG-SAVIGISHSGSNKDVLDAVKTAKSLGAGTIGI  214


>ref|WP_045512145.1| RpiR family transcriptional regulator [Bacillus amyloliquefaciens]
Length=285

 Score = 47.4 bits (111),  Expect = 0.015, Method: Compositional matrix adjust.
 Identities = 36/112 (32%), Positives = 53/112 (47%), Gaps = 17/112 (15%)

Query  230  RESIQEPTDSFHLLTPESWAR-LSAPHA-----FY--------SSQSYPDLINSRINRIR  275
            R +I   TD+FHLL P    + +   H+     FY        ++ +Y   + + IN I 
Sbjct  106  RANISGLTDTFHLLDPADVEKAVEMIHSADRIEFYGNGGSGLIATDAYHKFMRTGINCIA  165

Query  276  STDSHIYDLLYSRGWRGEGLGEPLGASDGIESKDVVDSVRTKDEVGLGIRGI  327
             TDSH   +  S G  G G    +G S    +KDV+D+V+T   +G G  GI
Sbjct  166  HTDSHFQAM--SAGLLGPG-SAVIGISHSGSNKDVLDAVKTAKSLGAGTIGI  214


>ref|WP_045208952.1| RpiR family transcriptional regulator [Bacillus methylotrophicus]
 gb|KJR68135.1| RpiR family transcriptional regulator [Bacillus methylotrophicus]
Length=285

 Score = 42.7 bits (99),  Expect = 0.38, Method: Compositional matrix adjust.
 Identities = 35/112 (31%), Positives = 51/112 (46%), Gaps = 17/112 (15%)

Query  230  RESIQEPTDSFHLLTPESWAR-LSAPH-----AFY--------SSQSYPDLINSRINRIR  275
            R +I   TD+FHLL P    + +   H      FY        ++ +Y   + + IN I 
Sbjct  106  RTNISGLTDTFHLLDPADIEKAVEMIHRADRIEFYGNGGSGLIATDAYHKFMRTGINCIA  165

Query  276  STDSHIYDLLYSRGWRGEGLGEPLGASDGIESKDVVDSVRTKDEVGLGIRGI  327
             TDSH   +  S G  G      +G S    +KDV+D+V+T   +G G  GI
Sbjct  166  HTDSHFQAM--SAGLLGPD-SAVIGISHSGSNKDVLDAVKTAKSLGAGTIGI  214


>ref|WP_025853459.1| RpiR family transcriptional regulator [Bacillus methylotrophicus]
Length=285

 Score = 42.7 bits (99),  Expect = 0.39, Method: Compositional matrix adjust.
 Identities = 35/112 (31%), Positives = 51/112 (46%), Gaps = 17/112 (15%)

Query  230  RESIQEPTDSFHLLTPESWAR-LSAPH-----AFY--------SSQSYPDLINSRINRIR  275
            R +I   TD+FHLL P    + +   H      FY        ++ +Y   + + IN I 
Sbjct  106  RTNISGLTDTFHLLDPADVEKAVEMIHRADRIEFYGNGGSGLIATDAYHKFMRTGINCIA  165

Query  276  STDSHIYDLLYSRGWRGEGLGEPLGASDGIESKDVVDSVRTKDEVGLGIRGI  327
             TDSH   +  S G  G      +G S    +KDV+D+V+T   +G G  GI
Sbjct  166  HTDSHFQAM--SAGLLGPD-SAVIGISHSGSNKDVLDAVKTAKSLGAGTIGI  214


>gb|KJD58719.1| RpiR family transcriptional regulator [Bacillus amyloliquefaciens]
Length=285

 Score = 42.7 bits (99),  Expect = 0.40, Method: Compositional matrix adjust.
 Identities = 35/112 (31%), Positives = 51/112 (46%), Gaps = 17/112 (15%)

Query  230  RESIQEPTDSFHLLTPESWAR-LSAPH-----AFY--------SSQSYPDLINSRINRIR  275
            R +I   TD+FHLL P    + +   H      FY        ++ +Y   + + IN I 
Sbjct  106  RTNISGLTDTFHLLDPADVEKAVEMIHRADRIEFYGNGGSGLIATDAYHKFMRTGINCIA  165

Query  276  STDSHIYDLLYSRGWRGEGLGEPLGASDGIESKDVVDSVRTKDEVGLGIRGI  327
             TDSH   +  S G  G      +G S    +KDV+D+V+T   +G G  GI
Sbjct  166  HTDSHFQAM--SAGLLGPD-SAVIGISHSGSNKDVLDAVKTAKSLGAGTIGI  214


>ref|WP_007613885.1| MULTISPECIES: RpiR family transcriptional regulator [Bacillus]
 gb|EIF14747.1| RpiR family transcriptional regulator [Bacillus sp. 5B6]
Length=285

 Score = 42.7 bits (99),  Expect = 0.41, Method: Compositional matrix adjust.
 Identities = 35/112 (31%), Positives = 51/112 (46%), Gaps = 17/112 (15%)

Query  230  RESIQEPTDSFHLLTPESWAR-LSAPH-----AFY--------SSQSYPDLINSRINRIR  275
            R +I   TD+FHLL P    + +   H      FY        ++ +Y   + + IN I 
Sbjct  106  RTNISGLTDTFHLLDPADVEKAVEMIHRADRIEFYGNGGSGLIATDAYHKFMRTGINCIA  165

Query  276  STDSHIYDLLYSRGWRGEGLGEPLGASDGIESKDVVDSVRTKDEVGLGIRGI  327
             TDSH   +  S G  G      +G S    +KDV+D+V+T   +G G  GI
Sbjct  166  HTDSHFQAM--SAGLLGPD-SAVIGISHSGSNKDVLDAVKTAKSLGAGTIGI  214


>ref|WP_052827874.1| RpiR family transcriptional regulator [Bacillus amyloliquefaciens]
 gb|KNX32556.1| RpiR family transcriptional regulator [Bacillus amyloliquefaciens]
Length=285

 Score = 42.7 bits (99),  Expect = 0.42, Method: Compositional matrix adjust.
 Identities = 35/112 (31%), Positives = 51/112 (46%), Gaps = 17/112 (15%)

Query  230  RESIQEPTDSFHLLTPESWAR-LSAPH-----AFY--------SSQSYPDLINSRINRIR  275
            R +I   TD+FHLL P    + +   H      FY        ++ +Y   + + IN I 
Sbjct  106  RTNISGLTDTFHLLDPADVEKAVEMIHRADRIEFYGNGGSGLIATDAYHKFMRTGINCIA  165

Query  276  STDSHIYDLLYSRGWRGEGLGEPLGASDGIESKDVVDSVRTKDEVGLGIRGI  327
             TDSH   +  S G  G      +G S    +KDV+D+V+T   +G G  GI
Sbjct  166  HTDSHFQAM--SAGLLGPD-SAVIGISHSGSNKDVLDAVKTAKSLGAGTIGI  214


>ref|WP_004009887.1| DNA helicase UvrD [Mobiluncus curtisii]
 gb|EFU79390.1| hypothetical protein HMPREF0388_1493 [Mobiluncus curtisii ATCC 
51333]
Length=1164

 Score = 39.7 bits (91),  Expect = 7.3, Method: Compositional matrix adjust.
 Identities = 32/114 (28%), Positives = 53/114 (46%), Gaps = 15/114 (13%)

Query  266  LINSRINRIRSTDSHIYDLLYSRGWRGEGLGEPLGASDGIESKDVVDSVRTKDEVGLGIR  325
            L+   + R+R  DS +YDL          L  P  + +   S  V+DS  T D+ G GI+
Sbjct  472  LVGWDVWRMRRMDSSLYDL---------QLQPPADSENAAGSTGVMDSADTADDEGSGIQ  522

Query  326  GIDRVPIPMNVLRKFIDRADQDKLTFEDEIARRRDESAVEPETSRIIRVLDALR  379
             +  +P     LR+ I   +   +   DEI      SA+ P+ ++++ +  ALR
Sbjct  523  IVGNLPYIQQFLRQVISGQNDFSVRSVDEII-----SAI-PDAAQLVDLAQALR  570


>gb|KNZ46545.1| hypothetical protein VP01_717g3 [Puccinia sorghi]
Length=199

 Score = 38.1 bits (87),  Expect = 9.5, Method: Compositional matrix adjust.
 Identities = 19/43 (44%), Positives = 25/43 (58%), Gaps = 1/43 (2%)

Query  184  NTLEDTSTLPDYERETPYPAHKVCEWILAHLSLPPRSDPSSPT  226
            N  +D+  LP +    P P H+V  W+LA L LPP  DPS+ T
Sbjct  58   NEADDSLDLPLFLGPAPSPPHRVVPWVLAQLGLPPL-DPSALT  99


Lambda      K        H        a         alpha
   0.315    0.131    0.377    0.792     4.96 

Gapped
Lambda      K        H        a         alpha    sigma
   0.267   0.0410    0.140     1.90     42.6     43.6 

Effective search space used: 3716158701264


  Database: nr
    Posted date:  Sep 23, 2015 12:05 AM
  Number of letters in database: 26,053,659,533
  Number of sequences in database:  71,551,133


Matrix: BLOSUM62
Gap Penalties: Existence: 11, Extension: 1
Neighboring words threshold: 11
Window for multiple hits: 40
```
