# Supplementary material for: Comparative genomics provides new insights into the diversity, physiology, and sexuality of the only industrially exploited tremellomycete: Phaffia rhodozyma
Source: BMC Genomics. 2016 Nov 9;17:901. doi: 10.1186/s12864-016-3244-7 (PMC5103461; doi:10.1186/s12864-016-3244-7)
Supplement: Additional file 6: — List of orphan genes with links to PFAM (related to Additional file 1: Table S1). (ZIP 1428 kb) [file 12864_2016_3244_MOESM6_ESM.zip › BLAST_HTML_FTR/G00152_P.html]

BLAST Search Results


```
BLASTP 2.2.27+


Reference:
Stephen F. Altschul, Thomas L. Madden, Alejandro A. Schäffer,
Jinghui Zhang, Zheng Zhang, Webb Miller, and David J. Lipman (1997),
"Gapped BLAST and PSI-BLAST: a new generation of protein database
search programs", Nucleic Acids Res. 25:3389-3402.


Reference for
composition-based statistics:
Alejandro A. Schäffer, L. Aravind, Thomas L. Madden, Sergei
Shavirin, John L. Spouge, Yuri I. Wolf, Eugene V. Koonin, and
Stephen F. Altschul (2001), "Improving the accuracy of PSI-BLAST
protein database searches with composition-based statistics and
other refinements", Nucleic Acids Res. 29:2994-3005.


Database: nr
           71,551,133 sequences; 26,053,659,533 total letters


Query= G00152_P

Length=319
                                                                      Score     E
Sequences producing significant alignments:                          (Bits)  Value

emb|CED83999.1|  hypothetical protein [Xanthophyllomyces dendrorh...   613    0.0  
gb|KIO12349.1|  hypothetical protein M404DRAFT_993351 [Pisolithus...  50.4    0.001
ref|XP_007356192.1|  hypothetical protein AURDEDRAFT_117314 [Auri...  50.4    0.002
gb|KIK29023.1|  hypothetical protein PISMIDRAFT_6929 [Pisolithus ...  50.4    0.002
gb|KIK81810.1|  hypothetical protein PAXRUDRAFT_832598 [Paxillus ...  49.3    0.004
gb|KIJ69743.1|  hypothetical protein HYDPIDRAFT_79187 [Hydnomerul...  48.1    0.010
gb|EJU05695.1|  hypothetical protein DACRYDRAFT_113751 [Dacryopin...  46.6    0.023
ref|XP_009543904.1|  hypothetical protein HETIRDRAFT_458084 [Hete...  46.6    0.024
gb|KIL70514.1|  hypothetical protein M378DRAFT_156664 [Amanita mu...  46.2    0.033
gb|KLO05354.1|  hypothetical protein SCHPADRAFT_1002955 [Schizopo...  45.8    0.042
gb|KIM52063.1|  hypothetical protein SCLCIDRAFT_1224075, partial ...  44.7    0.087
ref|XP_007382561.1|  hypothetical protein PUNSTDRAFT_112822 [Punc...  44.7    0.097
ref|XP_007776392.1|  hypothetical protein W97_00286 [Coniosporium...  44.7    0.11 
gb|EPS98778.1|  hypothetical protein FOMPIDRAFT_1164916 [Fomitops...  43.9    0.19 
gb|KIM75198.1|  hypothetical protein PILCRDRAFT_827443 [Piloderma...  43.9    0.22 
gb|KNZ78643.1|  hypothetical protein J132_11084 [Termitomyces sp....  42.7    0.44 
gb|KIJ63384.1|  hypothetical protein HYDPIDRAFT_156232 [Hydnomeru...  42.4    0.59 
ref|XP_007765165.1|  hypothetical protein CONPUDRAFT_162884 [Coni...  42.4    0.59 
gb|KDQ21887.1|  hypothetical protein BOTBODRAFT_150912 [Botryobas...  41.6    0.99 
ref|XP_002470021.1|  predicted protein [Postia placenta Mad-698-R...  41.2    1.4  
gb|KIK43482.1|  hypothetical protein CY34DRAFT_23518 [Suillus lut...  41.2    1.5  
ref|XP_007392551.1|  hypothetical protein PHACADRAFT_206218 [Phan...  40.8    1.8  
emb|CUA77085.1|  Glutamate receptor 3,4 [Rhizoctonia solani]          40.4    2.5  
gb|KIJ94391.1|  hypothetical protein K443DRAFT_683808 [Laccaria a...  40.4    2.7  
gb|KJA29582.1|  hypothetical protein HYPSUDRAFT_210505 [Hypholoma...  40.0    2.8  
ref|WP_019174944.1|  hypothetical protein [Brevibacterium massili...  40.0    3.2  
gb|KGB79050.1|  hypothetical protein CNBG_4888 [Cryptococcus gatt...  38.5    3.2  
gb|KIR35505.1|  hypothetical protein I352_01780 [Cryptococcus gat...  38.1    4.4  
gb|KIR97300.1|  hypothetical protein L804_05482 [Cryptococcus gat...  38.1    4.5  
ref|XP_001876478.1|  predicted protein [Laccaria bicolor S238N-H8...  39.7    4.6  
gb|KIK93572.1|  hypothetical protein PAXRUDRAFT_33996 [Paxillus r...  39.7    4.7  
gb|KIJ12580.1|  hypothetical protein PAXINDRAFT_177171 [Paxillus ...  39.3    5.0  


 >emb|CED83999.1| hypothetical protein [Xanthophyllomyces dendrorhous]
Length=328

 Score =  613 bits (1581),  Expect = 0.0, Method: Compositional matrix adjust.
 Identities = 318/328 (97%), Positives = 318/328 (97%), Gaps = 10/328 (3%)

Query  1    MNDQDHKSEPRITSFLSEFKFTFPPWPKPAGLLSFEEFKSVGHQASAGIEETKASEDEGS  60
            MNDQDHKSEPRITSFLSEFKFTFPPWPKPAGLLSFEEFKSVGHQASAGIEETKASEDEGS
Sbjct  1    MNDQDHKSEPRITSFLSEFKFTFPPWPKPAGLLSFEEFKSVGHQASAGIEETKASEDEGS  60

Query  61   SEHHLDGLESIIPDLSIRKDQKLSSAEDREVYRATILQVKDIFGGNGIHGKEPDDTISYD  120
            SEHHLDGLESIIPDLSIRKDQKLSSAEDREVYRATILQVKDIFGGNGIHGKEPDDTISYD
Sbjct  61   SEHHLDGLESIIPDLSIRKDQKLSSAEDREVYRATILQVKDIFGGNGIHGKEPDDTISYD  120

Query  121  FGPYDS----------ATREWEISRNQILIDDQAYLIVWAHWKHFAQTERTTTDNQSSDD  170
            FGPYDS          ATREWEISRNQILIDDQAYLIVWAHWKHFAQTERTTTDNQSSDD
Sbjct  121  FGPYDSQNDPAVRLYRATREWEISRNQILIDDQAYLIVWAHWKHFAQTERTTTDNQSSDD  180

Query  171  GRDDDVVLIDEEGNISQFNDGLDGGDVVDDVVKCRFLDDIEGTVKLFLSSRFNPDLVNIL  230
            GRDDDVVLIDEEGNISQFNDGLDGGDVVDDVVKCRFLDDIEGTVKLFLSSRFNPDLVNIL
Sbjct  181  GRDDDVVLIDEEGNISQFNDGLDGGDVVDDVVKCRFLDDIEGTVKLFLSSRFNPDLVNIL  240

Query  231  WSDERLAAAPKIILSFLKYIVAHKVLPEVHYQSQIQETICFLAAELDSSDSFQAGSDSNN  290
            WSDERLAAAPKIILSFLKYIVAHKVLPEVHYQSQIQETICFLAAELDSSDSFQAGSDSNN
Sbjct  241  WSDERLAAAPKIILSFLKYIVAHKVLPEVHYQSQIQETICFLAAELDSSDSFQAGSDSNN  300

Query  291  SLKYEKLLPLHHQKEVAAGWGYDSETEE  318
            SLKYEKLLPLHHQKEVAAGWGYDSETEE
Sbjct  301  SLKYEKLLPLHHQKEVAAGWGYDSETEE  328


>gb|KIO12349.1| hypothetical protein M404DRAFT_993351 [Pisolithus tinctorius 
Marx 270]
Length=688

 Score = 50.4 bits (119),  Expect = 0.001, Method: Compositional matrix adjust.
 Identities = 22/64 (34%), Positives = 40/64 (63%), Gaps = 2/64 (3%)

Query  206  FLDDIEGTVKLFLSSRFNPDLVNILWSDERLAAAPKIILSFLKYIVAHKVLPEVHYQSQI  265
            FL D E +V++FLSS        ++WS+  L +AP ++  FL++++ ++VLPE  YQ  +
Sbjct  258  FLSDPEKSVRIFLSSHMREK--GLIWSERNLISAPHLLSFFLRFVLRNRVLPEQAYQRGV  315

Query  266  QETI  269
            +  +
Sbjct  316  KRAL  319


>ref|XP_007356192.1| hypothetical protein AURDEDRAFT_117314 [Auricularia delicata 
TFB-10046 SS5]
 gb|EJD35702.1| hypothetical protein AURDEDRAFT_117314 [Auricularia delicata 
TFB-10046 SS5]
Length=593

 Score = 50.4 bits (119),  Expect = 0.002, Method: Compositional matrix adjust.
 Identities = 77/294 (26%), Positives = 120/294 (41%), Gaps = 41/294 (14%)

Query  21   FTFPPWPK-PAG--LLSFEEFKSVGHQASAGIEETKASEDEGSSEHHLDGLESIIPDLSI  77
            + FPPWP+ PAG  ++ F++FK VG              D+G  E  LDG+   IP +++
Sbjct  32   YRFPPWPQVPAGVTIVPFKDFKDVG----------IILRDDG--EEELDGMG--IPTIAL  77

Query  78   RKDQKLSSAEDREVYRATILQV-KDIFGGNGI--HGKEPDDTISYDFGPYDSA-TREWEI  133
                    A  R   +    Q  K+   G  +  H +  +   S    PYD   TR   +
Sbjct  78   SVVHGDGGAGSRSAKKRRRAQAEKNAREGIKLTWHEQWEEFDASTPVRPYDQRLTRG--V  135

Query  134  SRNQILIDDQA-------YLIVWAHWKHF---AQTERTTTDNQSSDDGRDDDVVLIDEEG  183
               Q + D Q+          V+  ++ F         T   Q+ D+G   D   +D   
Sbjct  136  RLEQAVTDFQSGRQWNSHLQSVFDFFRQFLGIVANPNQTKQKQAVDEGDFSDDEDMDAPT  195

Query  184  NISQFNDGLDGGDVVDDVVKCRFLDDIEGTVKLFLSSRFNPDLVNILWSDERLAAAPKII  243
             ++   +  D     D  +  RFLDD E  +K+FLSS ++     ++W   +L   P +I
Sbjct  196  AMAHIIED-DRDPWADHALMNRFLDDPERAIKVFLSSFYSSK--GLMWVPAKLKDGPILI  252

Query  244  LSFLKYIVAHKVLPEVHYQSQIQETICFLAAELDSSDSFQAGSDSNNSLKYEKL  297
              FL+Y++ H+VL E     +    +  LA      D     SD   SL  EK 
Sbjct  253  GLFLQYLIKHRVLSESRKALERALAVAQLA-----RDELPRTSDVGQSLSPEKF  301


>gb|KIK29023.1| hypothetical protein PISMIDRAFT_6929 [Pisolithus microcarpus 
441]
Length=688

 Score = 50.4 bits (119),  Expect = 0.002, Method: Compositional matrix adjust.
 Identities = 23/64 (36%), Positives = 39/64 (61%), Gaps = 2/64 (3%)

Query  206  FLDDIEGTVKLFLSSRFNPDLVNILWSDERLAAAPKIILSFLKYIVAHKVLPEVHYQSQI  265
            FL D E +V++FLSS        ++WS+  L  AP ++  FL++++ ++VLPE  YQ  I
Sbjct  257  FLSDPEKSVRIFLSSHMREK--GLIWSERNLTFAPHLLSFFLRFVLRNRVLPEQAYQRGI  314

Query  266  QETI  269
            +  +
Sbjct  315  KRAL  318


>gb|KIK81810.1| hypothetical protein PAXRUDRAFT_832598 [Paxillus rubicundulus 
Ve08.2h10]
Length=703

 Score = 49.3 bits (116),  Expect = 0.004, Method: Compositional matrix adjust.
 Identities = 27/74 (36%), Positives = 42/74 (57%), Gaps = 5/74 (7%)

Query  206  FLDDIEGTVKLFLSSRFNPDLVNILWSDERLAAAPKIILSFLKYIVAHKVLPEVHYQSQI  265
            FL D E  V++FLSS        ++WS+  L  AP++I  FL +++ +++LPE  YQ  +
Sbjct  257  FLADPEKAVRIFLSSYMREH--GLIWSERSLNYAPRLISFFLSFVLRNRILPEQSYQRGL  314

Query  266  Q---ETICFLAAEL  276
            +   ETI     EL
Sbjct  315  KRALETINLAKKEL  328


>gb|KIJ69743.1| hypothetical protein HYDPIDRAFT_79187 [Hydnomerulius pinastri 
MD-312]
Length=720

 Score = 48.1 bits (113),  Expect = 0.010, Method: Compositional matrix adjust.
 Identities = 22/64 (34%), Positives = 38/64 (59%), Gaps = 2/64 (3%)

Query  206  FLDDIEGTVKLFLSSRFNPDLVNILWSDERLAAAPKIILSFLKYIVAHKVLPEVHYQSQI  265
            FL+D E  ++ FLSS        ++WS+  L  AP+++  FL +I+ ++VLPE  YQ  +
Sbjct  263  FLNDPEKAIRTFLSSYMRE--YGLIWSERNLIYAPRLLGFFLSFILRNRVLPEQTYQRGL  320

Query  266  QETI  269
            +  +
Sbjct  321  KRAL  324


>gb|EJU05695.1| hypothetical protein DACRYDRAFT_113751 [Dacryopinax sp. DJM-731 
SS1]
Length=612

 Score = 46.6 bits (109),  Expect = 0.023, Method: Compositional matrix adjust.
 Identities = 30/102 (29%), Positives = 55/102 (54%), Gaps = 11/102 (11%)

Query  196  DVVDDVVKC-RFLDDIEGTVKLFLSSRFNPDLVNILWSDERLAAAPKIILSFLKYIVAHK  254
            DV D+  +C RFLD    T+K F SSR++     + WS+ +L   P I+  +++Y++  +
Sbjct  252  DVWDE--RCARFLDRTIDTLKRFFSSRYH--YAGLCWSEAKLRDGPIIVSLYIRYLLKDR  307

Query  255  VLPEVHYQSQIQETICFL----AAELDSSDSFQAGSDSNNSL  292
            V+PE      +   + FL      +L ++ +F  G++ N S+
Sbjct  308  VVPEC--TDNLTRALAFLEETIKPDLLATRAFSQGAEDNFSI  347


>ref|XP_009543904.1| hypothetical protein HETIRDRAFT_458084 [Heterobasidion irregulare 
TC 32-1]
 gb|ETW84208.1| hypothetical protein HETIRDRAFT_458084 [Heterobasidion irregulare 
TC 32-1]
Length=637

 Score = 46.6 bits (109),  Expect = 0.024, Method: Compositional matrix adjust.
 Identities = 19/69 (28%), Positives = 39/69 (57%), Gaps = 2/69 (3%)

Query  205  RFLDDIEGTVKLFLSSRFNPDLVNILWSDERLAAAPKIILSFLKYIVAHKVLPEVHYQSQ  264
            RFL++ E ++K+F SS F      ++WS+ R  A P ++  F+++++  +V PE  Y   
Sbjct  249  RFLNNPEQSMKVFFSSFFR--QRGLIWSEPRCTAFPHLVHFFIRFLIRCRVFPEPEYMEA  306

Query  265  IQETICFLA  273
            ++  +  + 
Sbjct  307  LKAALAVVG  315


>gb|KIL70514.1| hypothetical protein M378DRAFT_156664 [Amanita muscaria Koide 
BX008]
Length=738

 Score = 46.2 bits (108),  Expect = 0.033, Method: Compositional matrix adjust.
 Identities = 21/67 (31%), Positives = 42/67 (63%), Gaps = 4/67 (6%)

Query  206  FLDDIEGTVKLFLSSRFNPDLVNILWSDERLAAAPKIILSFLKYIVAHKVLPEVHYQSQI  265
            FL+D E  VK+FLSS    +   ++W+D  L+  P+++  FL+YI+ +K+L  + Y+  +
Sbjct  271  FLNDPETVVKMFLSSYMRKE--GLVWADSNLSITPRLLFFFLRYILRNKLL--LEYEVGL  326

Query  266  QETICFL  272
            ++ +  +
Sbjct  327  KKALVVV  333


>gb|KLO05354.1| hypothetical protein SCHPADRAFT_1002955 [Schizopora paradoxa]
Length=685

 Score = 45.8 bits (107),  Expect = 0.042, Method: Compositional matrix adjust.
 Identities = 28/94 (30%), Positives = 51/94 (54%), Gaps = 13/94 (14%)

Query  206  FLDDIEGTVKLFLSSRFNPDLVNILWSDERLAAAPKIILSFLKYIVAHKVLPEVHYQSQI  265
            FL D E ++KLF SS F      ++WS+ ++   P ++L FL++I+ +K LPE+    ++
Sbjct  258  FLVDPEFSMKLFFSSYFREK--GMIWSEPKIRDYPILLLFFLRFIIRNKALPEL--DREL  313

Query  266  QETICFLAAELDSSDSFQAGSDSNNSLKYEKLLP  299
            Q+ +  +          QA  +   + K+ KL+P
Sbjct  314  QKAVAAVE---------QAKIELPKAFKFAKLVP  338


>gb|KIM52063.1| hypothetical protein SCLCIDRAFT_1224075, partial [Scleroderma 
citrinum Foug A]
Length=442

 Score = 44.7 bits (104),  Expect = 0.087, Method: Compositional matrix adjust.
 Identities = 25/74 (34%), Positives = 41/74 (55%), Gaps = 5/74 (7%)

Query  206  FLDDIEGTVKLFLSSRFNPDLVNILWSDERLAAAPKIILSFLKYIVAHKVLPEVHYQSQI  265
            FL D E +V++FLSS        ++WS+  L  AP ++  F+ +++ ++V PE  YQ  +
Sbjct  15   FLKDPEKSVRIFLSSHMREK--GLIWSERNLTYAPHLLSFFIGFLLRNRVFPEQTYQRGL  72

Query  266  Q---ETICFLAAEL  276
            +   ETI     EL
Sbjct  73   KRAFETIQQAKKEL  86


>ref|XP_007382561.1| hypothetical protein PUNSTDRAFT_112822 [Punctularia strigosozonata 
HHB-11173 SS5]
 gb|EIN11049.1| hypothetical protein PUNSTDRAFT_112822 [Punctularia strigosozonata 
HHB-11173 SS5]
Length=548

 Score = 44.7 bits (104),  Expect = 0.097, Method: Compositional matrix adjust.
 Identities = 34/121 (28%), Positives = 52/121 (43%), Gaps = 15/121 (12%)

Query  199  DDVVKCRFLDDIEGTVKLFLSSRFNPDLVNILWSDERLAAAPKIILSFLKYIVAHKVLPE  258
            DD     F+ D E ++K+FLSS F      ++WS  R   APK++  FL +I+  KV P 
Sbjct  205  DDNRMLAFIKDPETSIKVFLSSFFRDK--GLIWSVPRCRDAPKLLHFFLNFIIRTKVFPG  262

Query  259  VHYQSQIQETICFLA-AELDSSDSFQAGSDSNNSLKYEKLLPLHHQKEVAAGWGYDSETE  317
            +  Q +    +   A  EL  + +              +LLP    +     WG  +E  
Sbjct  263  MGEQFEKAAAVALKAETELPMTKTIS------------ELLPGDFSRACTTTWGSVTEYY  310

Query  318  E  318
            E
Sbjct  311  E  311


>ref|XP_007776392.1| hypothetical protein W97_00286 [Coniosporium apollinis CBS 100218]
 gb|EON61075.1| hypothetical protein W97_00286 [Coniosporium apollinis CBS 100218]
Length=610

 Score = 44.7 bits (104),  Expect = 0.11, Method: Compositional matrix adjust.
 Identities = 24/62 (39%), Positives = 31/62 (50%), Gaps = 4/62 (6%)

Query  209  DIEGTVKLFLSSRFNPDLVNILWSDERLAAAPKIILSFLKYIVAHKVLPEVHYQSQIQET  268
            D  G VK + SS   P    + WSD+RLA   K+I +F  YI+ H V PE +        
Sbjct  307  DFTGVVKGYFSSYQQP----LEWSDQRLAIHAKVIRNFYNYILHHNVCPEYYGDVMEARN  362

Query  269  IC  270
            IC
Sbjct  363  IC  364


>gb|EPS98778.1| hypothetical protein FOMPIDRAFT_1164916 [Fomitopsis pinicola 
FP-58527 SS1]
Length=670

 Score = 43.9 bits (102),  Expect = 0.19, Method: Compositional matrix adjust.
 Identities = 27/111 (24%), Positives = 52/111 (47%), Gaps = 11/111 (10%)

Query  206  FLDDIEGTVKLFLSSRFNPDLVNILWSDERLAAAPKIILSFLKYIVAHKVLPEVHYQSQI  265
            F +D +  VK+F SS +      ++WS++R+   P +I  +LK+++ ++VLPE  ++  +
Sbjct  272  FFNDADSCVKVFFSSYYREK--GLVWSEQRVRDGPILIGFWLKFLLRNRVLPEPEHEKSL  329

Query  266  QETICFLAAELDSSDSFQAGSDSNNSLKYEKLLPLHHQKEVAAGWGYDSET  316
            +  +  +          QA  +   +    K LP        A WG   +T
Sbjct  330  RRALLVIE---------QARKELPQTFVIGKALPDAVGAGCEALWGSKGQT  371


>gb|KIM75198.1| hypothetical protein PILCRDRAFT_827443 [Piloderma croceum F 1598]
Length=740

 Score = 43.9 bits (102),  Expect = 0.22, Method: Compositional matrix adjust.
 Identities = 19/71 (27%), Positives = 39/71 (55%), Gaps = 2/71 (3%)

Query  206  FLDDIEGTVKLFLSSRFNPDLVNILWSDERLAAAPKIILSFLKYIVAHKVLPEVHYQSQI  265
            FL+D E +  +FLSS        ++W+D  L   P++   F+ +++ ++VLPE +++  +
Sbjct  275  FLNDPEKSTMIFLSSYMREQ--GMIWADRNLVTGPRLTAFFINFLLRNRVLPESNHERGL  332

Query  266  QETICFLAAEL  276
            +  +   A  L
Sbjct  333  RRALEVTAIAL  343


>gb|KNZ78643.1| hypothetical protein J132_11084 [Termitomyces sp. J132]
Length=563

 Score = 42.7 bits (99),  Expect = 0.44, Method: Compositional matrix adjust.
 Identities = 16/67 (24%), Positives = 41/67 (61%), Gaps = 2/67 (3%)

Query  206  FLDDIEGTVKLFLSSRFNPDLVNILWSDERLAAAPKIILSFLKYIVAHKVLPEVHYQSQI  265
            FL+D E  +++FLSS        ++W+D+ L   P+++  F+++++ ++V PE  +  ++
Sbjct  229  FLNDPELKMRIFLSSYMRKQ--GLIWTDKNLINIPRLVEFFVRFLIRNRVFPEPDFDREL  286

Query  266  QETICFL  272
            + ++  +
Sbjct  287  RRSLAVI  293


>gb|KIJ63384.1| hypothetical protein HYDPIDRAFT_156232 [Hydnomerulius pinastri 
MD-312]
Length=620

 Score = 42.4 bits (98),  Expect = 0.59, Method: Compositional matrix adjust.
 Identities = 70/297 (24%), Positives = 121/297 (41%), Gaps = 58/297 (20%)

Query  5    DHKSEPRITSF--LSEFKFTFPPWPKP---AGLLSFEEFKSVGHQ-------------AS  46
            +HK    I S   + E    FPP+P P     +L F++FK+ G++             A 
Sbjct  8    EHKKSITIVSAPAVDELSLRFPPFPAPPQGVAILPFKDFKAFGYKRVANESGQEIEVDAF  67

Query  47   AGIEETKASEDEGSSEHHLDGLESIIPDLSIRKDQKLSSAEDREVYRATILQVKDIFGGN  106
            AG    K + +E +++   D  +      S   + +L    +      +       F GN
Sbjct  68   AGQPTAKVASEEEAAQRRKDKKKRKNAGQSTDANGRLIPWWEEWEESESSRTASVTFNGN  127

Query  107  GIHGKEPDDTISYDFGPYDSA-----TREW-EISRNQILIDDQ--------AYLIVWAHW  152
                      +SY    Y +A      R W EI+    +I D         A + ++   
Sbjct  128  ----------MSYVDRVYQAADDFRVGRTWPEIATGVRIIWDHFRVYVGLLASMPIYRKP  177

Query  153  KHFAQTERTTTDN--QSSDD------GRDDDVVLI-DEEGNISQFNDGL----DGGDVVD  199
            K  ++ E    +N  ++SDD       R  +V +I D    I+  N  L    D   +  
Sbjct  178  KGRSRGEVDGPNNGGEASDDEDDAPRARQTNVTIIQDHLEQIAHPNKVLEPNADDSSIEA  237

Query  200  DVVKCRFLDDIEGTVKLFLSSRFNPDLVNILWSDERLAAAPKIILSFLKYIVAHKVL  256
             +++  F+DDIE TVK+FLSS        ++W++  L  AP ++  FL++++ + V 
Sbjct  238  HLLRA-FIDDIEKTVKVFLSSHMRD--TGLIWTERNLFIAPTVLHFFLRFMLRNGVF  291


>ref|XP_007765165.1| hypothetical protein CONPUDRAFT_162884 [Coniophora puteana RWD-64-598 
SS2]
 gb|EIW85795.1| hypothetical protein CONPUDRAFT_162884 [Coniophora puteana RWD-64-598 
SS2]
Length=682

 Score = 42.4 bits (98),  Expect = 0.59, Method: Compositional matrix adjust.
 Identities = 23/68 (34%), Positives = 37/68 (54%), Gaps = 2/68 (3%)

Query  206  FLDDIEGTVKLFLSSRFNPDLVNILWSDERLAAAPKIILSFLKYIVAHKVLPEVHYQSQI  265
            F+DDIEG VK+FL +        ++WS + L  AP II  FL++++ + V  +    S  
Sbjct  271  FVDDIEGGVKMFLGAYMRDK--GLMWSSQNLNIAPIIISFFLRFLLRNAVFEDSRDHSDS  328

Query  266  QETICFLA  273
             +   F+A
Sbjct  329  LKRALFVA  336


>gb|KDQ21887.1| hypothetical protein BOTBODRAFT_150912 [Botryobasidium botryosum 
FD-172 SS1]
Length=590

 Score = 41.6 bits (96),  Expect = 0.99, Method: Compositional matrix adjust.
 Identities = 20/65 (31%), Positives = 36/65 (55%), Gaps = 4/65 (6%)

Query  205  RFLDDIEGTVKLFLSSRFNPDLVNILWSDERLAAAPKIILSFLKYIVAHKVLPEVHYQSQ  264
            RF D+ E ++K+F SS F      ++WS+ +L   P ++  FL Y + H   PE  Y  +
Sbjct  201  RFFDEPEESMKIFFSSYFRDK--GLIWSEAKLRDGPLLVSIFLSYAIRHSAFPE--YTRE  256

Query  265  IQETI  269
            +++ +
Sbjct  257  LKKAL  261


>ref|XP_002470021.1| predicted protein [Postia placenta Mad-698-R]
 gb|EED84771.1| predicted protein [Postia placenta Mad-698-R]
Length=792

 Score = 41.2 bits (95),  Expect = 1.4, Method: Compositional matrix adjust.
 Identities = 19/52 (37%), Positives = 33/52 (63%), Gaps = 2/52 (4%)

Query  207  LDDIEGTVKLFLSSRFNPDLVNILWSDERLAAAPKIILSFLKYIVAHKVLPE  258
             DD E  +K+FLSS F+     ++WS++ +  AP +I  F+ +I+ + +LPE
Sbjct  131  FDDTEKNIKIFLSSYFHDK--GLIWSEQCVHDAPILIGFFINFIICNHILPE  180


>gb|KIK43482.1| hypothetical protein CY34DRAFT_23518 [Suillus luteus UH-Slu-Lm8-n1]
Length=692

 Score = 41.2 bits (95),  Expect = 1.5, Method: Compositional matrix adjust.
 Identities = 19/64 (30%), Positives = 37/64 (58%), Gaps = 2/64 (3%)

Query  206  FLDDIEGTVKLFLSSRFNPDLVNILWSDERLAAAPKIILSFLKYIVAHKVLPEVHYQSQI  265
            FL+D E +V +FLSS        ++WS+  L  AP ++  FL +++ ++VL +  ++  I
Sbjct  258  FLNDPEKSVTIFLSSHMREQ--GLIWSERNLVNAPHLLSFFLNFVLRNRVLQKASHERGI  315

Query  266  QETI  269
            +  +
Sbjct  316  RNAL  319


>ref|XP_007392551.1| hypothetical protein PHACADRAFT_206218 [Phanerochaete carnosa 
HHB-10118-sp]
 gb|EKM60003.1| hypothetical protein PHACADRAFT_206218 [Phanerochaete carnosa 
HHB-10118-sp]
Length=705

 Score = 40.8 bits (94),  Expect = 1.8, Method: Compositional matrix adjust.
 Identities = 23/70 (33%), Positives = 36/70 (51%), Gaps = 2/70 (3%)

Query  206  FLDDIEGTVKLFLSSRFNPDLVNILWSDERLAAAPKIILSFLKYIVAHKVLPEVHYQSQI  265
            FL+D E TVK+F SS +       + S E+    P +I  FL+Y++ ++V PE     + 
Sbjct  247  FLNDPEQTVKIFFSSHWRDK--GYVHSKEKCKEGPILIAFFLRYLIRNRVFPEEEAALKR  304

Query  266  QETICFLAAE  275
               +C  A E
Sbjct  305  AAALCDRAKE  314


>emb|CUA77085.1| Glutamate receptor 3,4 [Rhizoctonia solani]
Length=1337

 Score = 40.4 bits (93),  Expect = 2.5, Method: Compositional matrix adjust.
 Identities = 27/90 (30%), Positives = 43/90 (48%), Gaps = 2/90 (2%)

Query  44   QASAGIEETKASEDEGSSEHHLDGLESIIPDLSIRKDQKL--SSAEDREVYRATILQVKD  101
            Q  A    ++A E   +S   ++ + S +  + + KD  L  + AE  E   A+IL    
Sbjct  79   QIPALSANSRAREGRRASRKGINHVLSAVDSIELEKDPNLNETGAETEEGSLASILNEDI  138

Query  102  IFGGNGIHGKEPDDTISYDFGPYDSATREW  131
            + G N +H  EPD+T+  D  P + AT  W
Sbjct  139  LRGFNEVHAWEPDETVEVDTPPSNPATHPW  168


>gb|KIJ94391.1| hypothetical protein K443DRAFT_683808 [Laccaria amethystina LaAM-08-1]
Length=650

 Score = 40.4 bits (93),  Expect = 2.7, Method: Compositional matrix adjust.
 Identities = 20/76 (26%), Positives = 46/76 (61%), Gaps = 6/76 (8%)

Query  195  GDVVDDVVKCR----FLDDIEGTVKLFLSSRFNPDLVNILWSDERLAAAPKIILSFLKYI  250
            G++ D  V+ R    F+D+ E T+K+F+SS        ++W+++ L A P+++  ++ ++
Sbjct  193  GELTDANVRDRKILSFVDNPETTIKIFMSSYSRHK--GLIWAEDNLKAIPRLMGFWIDFL  250

Query  251  VAHKVLPEVHYQSQIQ  266
            + +KVLP     ++++
Sbjct  251  IRNKVLPSSERNAKLK  266


>gb|KJA29582.1| hypothetical protein HYPSUDRAFT_210505 [Hypholoma sublateritium 
FD-334 SS-4]
Length=632

 Score = 40.0 bits (92),  Expect = 2.8, Method: Compositional matrix adjust.
 Identities = 22/68 (32%), Positives = 37/68 (54%), Gaps = 2/68 (3%)

Query  206  FLDDIEGTVKLFLSSRFNPDLVNILWSDERLAAAPKIILSFLKYIVAHKVLPEVHYQSQI  265
            F D+ E + K+F++S      +  +W D  L   P++IL F+ +++  KVLPEV  + + 
Sbjct  241  FFDNPELSFKVFMTSHAR--EMGYIWYDANLDCMPRVILFFVNFLLRSKVLPEVDRELRR  298

Query  266  QETICFLA  273
               I  LA
Sbjct  299  SVEILKLA  306


>ref|WP_019174944.1| hypothetical protein [Brevibacterium massiliense]
Length=1004

 Score = 40.0 bits (92),  Expect = 3.2, Method: Composition-based stats.
 Identities = 25/93 (27%), Positives = 45/93 (48%), Gaps = 2/93 (2%)

Query  60   SSEHHLDGLESIIPDLSIRKDQKLSSAEDREVYRATILQVKDIFGGNGIHGKEPDDTISY  119
            ++EH    + S +P L+  +D+ L SA D EV R   L   ++     ++  +P D++  
Sbjct  897  AAEHPGLRITSTLPALAAARDEGLVSASDHEVLRTAWLMATEVRNAITLYRGKPSDSLPS  956

Query  120  DFGPYDSATR--EWEISRNQILIDDQAYLIVWA  150
            D    ++A R   +     Q+L+DD   +  WA
Sbjct  957  DIRELEAAARLMGYPQGSAQLLVDDYLRVTRWA  989


>gb|KGB79050.1| hypothetical protein CNBG_4888 [Cryptococcus gattii R265]
 gb|KIR38770.1| hypothetical protein I313_05408 [Cryptococcus gattii Ram5]
 gb|KIR70954.1| hypothetical protein I310_05366 [Cryptococcus gattii CA1014]
 gb|KIY57782.1| hypothetical protein I307_02855 [Cryptococcus gattii 99/473]
Length=165

 Score = 38.5 bits (88),  Expect = 3.2, Method: Compositional matrix adjust.
 Identities = 27/74 (36%), Positives = 39/74 (53%), Gaps = 4/74 (5%)

Query  33   LSFEEFKSVGHQA--SAGIEETKASEDEGSSEHHLDG--LESIIPDLSIRKDQKLSSAED  88
            L FEE   +  Q+  S+    T  + DE    HHL    + S  P  SI  D KL+  ED
Sbjct  14   LPFEETDHLPPQSPRSSHPSTTTLATDELFGSHHLPAPAISSTHPSPSIEDDHKLAIIED  73

Query  89   REVYRATILQVKDI  102
            R+V RA++L++ D+
Sbjct  74   RQVIRASVLEMADL  87


>gb|KIR35505.1| hypothetical protein I352_01780 [Cryptococcus gattii MMRL2647]
Length=165

 Score = 38.1 bits (87),  Expect = 4.4, Method: Compositional matrix adjust.
 Identities = 27/74 (36%), Positives = 39/74 (53%), Gaps = 4/74 (5%)

Query  33   LSFEEFKSVGHQA--SAGIEETKASEDEGSSEHHLDG--LESIIPDLSIRKDQKLSSAED  88
            L FEE   +  Q+  S+    T  + DE    HHL    + S  P  SI  D KL+  ED
Sbjct  14   LPFEETDHLPPQSPRSSHPSTTTLATDELFGFHHLPAPAISSTHPSPSIEDDHKLAITED  73

Query  89   REVYRATILQVKDI  102
            R+V RA++L++ D+
Sbjct  74   RQVIRASVLEMADL  87


>gb|KIR97300.1| hypothetical protein L804_05482 [Cryptococcus gattii 2001/935-1]
Length=165

 Score = 38.1 bits (87),  Expect = 4.5, Method: Compositional matrix adjust.
 Identities = 27/74 (36%), Positives = 39/74 (53%), Gaps = 4/74 (5%)

Query  33   LSFEEFKSVGHQA--SAGIEETKASEDEGSSEHHLDG--LESIIPDLSIRKDQKLSSAED  88
            L FEE   +  Q+  S+    T  + DE    HHL    + S  P  SI  D KL+  ED
Sbjct  14   LPFEETDHLPPQSPRSSHPSTTTLATDELFGFHHLPAPAISSTHPSPSIEDDHKLAITED  73

Query  89   REVYRATILQVKDI  102
            R+V RA++L++ D+
Sbjct  74   RQVIRASVLEMADL  87


>ref|XP_001876478.1| predicted protein [Laccaria bicolor S238N-H82]
 gb|EDR12214.1| predicted protein [Laccaria bicolor S238N-H82]
Length=652

 Score = 39.7 bits (91),  Expect = 4.6, Method: Compositional matrix adjust.
 Identities = 21/67 (31%), Positives = 41/67 (61%), Gaps = 6/67 (9%)

Query  195  GDVVDDVVKCR----FLDDIEGTVKLFLSSRFNPDLVNILWSDERLAAAPKIILSFLKYI  250
            G++ D  VK R    F+DD E T+K+F+SS        ++W+++ L A P+++  ++ ++
Sbjct  205  GELADANVKERKISSFVDDPETTIKIFMSSYSRHK--GLIWAEDNLKAIPRLMGFWIDFL  262

Query  251  VAHKVLP  257
            + +KV P
Sbjct  263  LRNKVFP  269


>gb|KIK93572.1| hypothetical protein PAXRUDRAFT_33996 [Paxillus rubicundulus 
Ve08.2h10]
Length=624

 Score = 39.7 bits (91),  Expect = 4.7, Method: Compositional matrix adjust.
 Identities = 18/51 (35%), Positives = 31/51 (61%), Gaps = 2/51 (4%)

Query  206  FLDDIEGTVKLFLSSRFNPDLVNILWSDERLAAAPKIILSFLKYIVAHKVL  256
            F+DD+E TVK FLSS        ++W++  L  AP ++  FL++++ + V 
Sbjct  243  FIDDMEKTVKTFLSSHMRDS--GLIWTERNLLIAPTVLHFFLRFMLRNGVF  291


>gb|KIJ12580.1| hypothetical protein PAXINDRAFT_177171 [Paxillus involutus ATCC 
200175]
Length=641

 Score = 39.3 bits (90),  Expect = 5.0, Method: Compositional matrix adjust.
 Identities = 18/51 (35%), Positives = 31/51 (61%), Gaps = 2/51 (4%)

Query  206  FLDDIEGTVKLFLSSRFNPDLVNILWSDERLAAAPKIILSFLKYIVAHKVL  256
            F+DD+E TVK FLSS        ++W++  L  AP ++  FL++++ + V 
Sbjct  261  FIDDMEKTVKTFLSSHMRDS--GLIWTERNLLIAPTVLHFFLRFMLRNGVF  309


Lambda      K        H        a         alpha
   0.316    0.135    0.401    0.792     4.96 

Gapped
Lambda      K        H        a         alpha    sigma
   0.267   0.0410    0.140     1.90     42.6     43.6 

Effective search space used: 2589247239527


  Database: nr
    Posted date:  Sep 23, 2015 12:05 AM
  Number of letters in database: 26,053,659,533
  Number of sequences in database:  71,551,133


Matrix: BLOSUM62
Gap Penalties: Existence: 11, Extension: 1
Neighboring words threshold: 11
Window for multiple hits: 40
```
