# Supplementary material for: Comparative genomics provides new insights into the diversity, physiology, and sexuality of the only industrially exploited tremellomycete: Phaffia rhodozyma
Source: BMC Genomics. 2016 Nov 9;17:901. doi: 10.1186/s12864-016-3244-7 (PMC5103461; doi:10.1186/s12864-016-3244-7)
Supplement: Additional file 6: — List of orphan genes with links to PFAM (related to Additional file 1: Table S1). (ZIP 1428 kb) [file 12864_2016_3244_MOESM6_ESM.zip › BLAST_HTML_FTR/G02132_P.html]

BLAST Search Results


```
BLASTP 2.2.27+


Reference:
Stephen F. Altschul, Thomas L. Madden, Alejandro A. Schäffer,
Jinghui Zhang, Zheng Zhang, Webb Miller, and David J. Lipman (1997),
"Gapped BLAST and PSI-BLAST: a new generation of protein database
search programs", Nucleic Acids Res. 25:3389-3402.


Reference for
composition-based statistics:
Alejandro A. Schäffer, L. Aravind, Thomas L. Madden, Sergei
Shavirin, John L. Spouge, Yuri I. Wolf, Eugene V. Koonin, and
Stephen F. Altschul (2001), "Improving the accuracy of PSI-BLAST
protein database searches with composition-based statistics and
other refinements", Nucleic Acids Res. 29:2994-3005.


Database: nr
           71,551,133 sequences; 26,053,659,533 total letters


Query= G02132_P

Length=298
                                                                      Score     E
Sequences producing significant alignments:                          (Bits)  Value

emb|CED82142.1|  hypothetical protein [Xanthophyllomyces dendrorh...   495    1e-172
ref|WP_036898417.1|  hypothetical protein [Prevotella sp. S7 MS 2]    38.1    6.2   


 >emb|CED82142.1| hypothetical protein [Xanthophyllomyces dendrorhous]
Length=376

 Score =  495 bits (1274),  Expect = 1e-172, Method: Compositional matrix adjust.
 Identities = 269/296 (91%), Positives = 271/296 (92%), Gaps = 23/296 (8%)

Query  1    MFSTLAIARSSASSSRRSCVSLRELNTVSDTLNLKGPHRTRLRNPTKPLAKPSPSSSLPV  60
            MFSTLAIARSSASSSRRSCVSLRELNTVSDTLNLKGPHRTRLRNPTKPLAKPSPSSSLPV
Sbjct  1    MFSTLAIARSSASSSRRSCVSLRELNTVSDTLNLKGPHRTRLRNPTKPLAKPSPSSSLPV  60

Query  61   SSTEALPASTLRVAENLSPDKPKDQTGNFAFLDKFFLEAAQAYKDRALNALNHRPYVYVK  120
            SSTEALPASTLRVAENLSPDKPKDQTGNFAFLDKFFLEAAQAYKDRALNALNHRPYVYVK
Sbjct  61   SSTEALPASTLRVAENLSPDKPKDQTGNFAFLDKFFLEAAQAYKDRALNALNHRPYVYVK  120

Query  121  PSVLPGEPLRTNIGTKPGQLLVKFPEALERTPYLKIVANGPLTREYIDHTFSELDIRTKS  180
            PSVLPGEPLRTNIGTKPGQLLVKFPEALERTPYLKIVANGPLTREYIDHTFSELDIRTKS
Sbjct  121  PSVLPGEPLRTNIGTKPGQLLVKFPEALERTPYLKIVANGPLTREYIDHTFSELDIRTKS  180

Query  181  VCLLSQQAPDEMFDLGRFVFQVELSSLMNLAMAVGVHWPHFEIISAHLAEDHVAFFHS--  238
            VCLLSQQAPDEMFDLGRFVFQVELSSLMNLAMAVGVHWPHFEIISAHLAEDHV F +S  
Sbjct  181  VCLLSQQAPDEMFDLGRFVFQVELSSLMNLAMAVGVHWPHFEIISAHLAEDHVGFSNSKT  240

Query  239  ---------------------QDDVEFEKIPTATKLPTTDPINTLPIGSHGCHIKV  273
                                 QDDVEFEKIPTATKLPTTDPINTLPIGSHGCHIK+
Sbjct  241  NKLLKANRTKNISLSTYELYFQDDVEFEKIPTATKLPTTDPINTLPIGSHGCHIKI  296


>ref|WP_036898417.1| hypothetical protein [Prevotella sp. S7 MS 2]
Length=219

 Score = 38.1 bits (87),  Expect = 6.2, Method: Compositional matrix adjust.
 Identities = 36/129 (28%), Positives = 61/129 (47%), Gaps = 15/129 (12%)

Query  134  GTKPGQLLVKFPEALERTPYLKIVANGPLTREYIDHTFSELDIRTKSVCLLSQQAPDEMF  193
            G+K  QL VK  E   R P  +I  +  LT+ Y DH           + LL+  APD+MF
Sbjct  10   GSKLSQLQVK--EIFNRFP--EIDYDLILTKSYGDHNMQ--------ISLLNGAAPDDMF  57

Query  194  DLG--RFVFQVELSSLMNLAMAVGVHW-PHFEIISAHLAEDHVAFFHSQDDVEFEKIPTA  250
              G  + +   E    ++ A  + V+  P  EII+ + A D      S++ ++  ++P  
Sbjct  58   TRGLDQLILTGEADIAIHSAKDLPVNLNPELEIIALYEAFDKTDSLVSRNHLKLNELPAG  117

Query  251  TKLPTTDPI  259
            + + T+ P+
Sbjct  118  SSIGTSSPL  126


Lambda      K        H        a         alpha
   0.321    0.134    0.395    0.792     4.96 

Gapped
Lambda      K        H        a         alpha    sigma
   0.267   0.0410    0.140     1.90     42.6     43.6 

Effective search space used: 2293488566684


  Database: nr
    Posted date:  Sep 23, 2015 12:05 AM
  Number of letters in database: 26,053,659,533
  Number of sequences in database:  71,551,133


Matrix: BLOSUM62
Gap Penalties: Existence: 11, Extension: 1
Neighboring words threshold: 11
Window for multiple hits: 40
```
