# Supplementary material for: Comparative genomics provides new insights into the diversity, physiology, and sexuality of the only industrially exploited tremellomycete: Phaffia rhodozyma
Source: BMC Genomics. 2016 Nov 9;17:901. doi: 10.1186/s12864-016-3244-7 (PMC5103461; doi:10.1186/s12864-016-3244-7)
Supplement: Additional file 6: — List of orphan genes with links to PFAM (related to Additional file 1: Table S1). (ZIP 1428 kb) [file 12864_2016_3244_MOESM6_ESM.zip › BLAST_HTML_FTR/G03789_P.html]

BLAST Search Results


```
BLASTP 2.2.27+


Reference:
Stephen F. Altschul, Thomas L. Madden, Alejandro A. Schäffer,
Jinghui Zhang, Zheng Zhang, Webb Miller, and David J. Lipman (1997),
"Gapped BLAST and PSI-BLAST: a new generation of protein database
search programs", Nucleic Acids Res. 25:3389-3402.


Reference for
composition-based statistics:
Alejandro A. Schäffer, L. Aravind, Thomas L. Madden, Sergei
Shavirin, John L. Spouge, Yuri I. Wolf, Eugene V. Koonin, and
Stephen F. Altschul (2001), "Improving the accuracy of PSI-BLAST
protein database searches with composition-based statistics and
other refinements", Nucleic Acids Res. 29:2994-3005.


Database: nr
           71,551,133 sequences; 26,053,659,533 total letters


Query= G03789_P

Length=381
                                                                      Score     E
Sequences producing significant alignments:                          (Bits)  Value

emb|CDZ98064.1|  hypothetical protein [Xanthophyllomyces dendrorh...   417    6e-142
ref|XP_003191206.1|  hypothetical protein CGB_A1800W [Cryptococcu...  43.5    0.23  
emb|CEL63680.1|  hypothetical protein RSOLAG1IB_05441 [Rhizoctoni...  43.5    0.33  
gb|KIR59865.1|  hypothetical protein I314_04300 [Cryptococcus gat...  41.6    0.83  
gb|KIR54225.1|  hypothetical protein I315_03284 [Cryptococcus gat...  41.2    1.1   
gb|KIR80023.1|  hypothetical protein I306_02987 [Cryptococcus gat...  41.2    1.2   
gb|KIR85507.1|  hypothetical protein I308_04203 [Cryptococcus gat...  41.2    1.3   
emb|CUA69132.1|  hypothetical protein RSOLAG22IIIB_03810 [Rhizoct...  41.2    1.6   
ref|XP_012047557.1|  hypothetical protein CNAG_03930 [Cryptococcu...  38.5    9.5   


 >emb|CDZ98064.1| hypothetical protein [Xanthophyllomyces dendrorhous]
Length=290

 Score =  417 bits (1073),  Expect = 6e-142, Method: Compositional matrix adjust.
 Identities = 280/282 (99%), Positives = 280/282 (99%), Gaps = 0/282 (0%)

Query  1    MTDKQPSSSNDIEAPPTAAQLAAFQARADLSVVMLRNMMGKMVKGWVPPTAEELNQTDPQ  60
            MTDKQPSSSNDIEAPPTAAQLAAFQARADLSVVMLRNMMGKMVKGWVPPTAEELNQTDPQ
Sbjct  1    MTDKQPSSSNDIEAPPTAAQLAAFQARADLSVVMLRNMMGKMVKGWVPPTAEELNQTDPQ  60

Query  61   VKPNATRKLINKLVPRSSAASSSTLAPSSSATTSFEQHQQSQQNNKRYRSSAVANLINQP  120
            VKPNATRKLINKLVPRSSAASSSTLAPSSSATTSFEQHQQSQQNNKRYRSSAVANLINQP
Sbjct  61   VKPNATRKLINKLVPRSSAASSSTLAPSSSATTSFEQHQQSQQNNKRYRSSAVANLINQP  120

Query  121  APSEKNTTNGTSNGNGNGTGSKRPGRDNQDSDSDEDEAEDRSRAVGKKAKLSNTGIDQFG  180
            APSEKNTTNGTSNGNGNGTGSKRPGRDNQDSDSDEDEAE RSRAVGKKAKLSNTGIDQFG
Sbjct  121  APSEKNTTNGTSNGNGNGTGSKRPGRDNQDSDSDEDEAESRSRAVGKKAKLSNTGIDQFG  180

Query  181  GKNKKKKKQLQQQQVPPPPSSTAVATDKGAGNKSKDTEPGPESRPEQEQINPFRLFASPK  240
            GKNKKKKKQLQQQQVPPPPSSTAVATDKGAGNKSKDTEPGPESRPEQEQINPFRLFASPK
Sbjct  181  GKNKKKKKQLQQQQVPPPPSSTAVATDKGAGNKSKDTEPGPESRPEQEQINPFRLFASPK  240

Query  241  TSLPKPNTAPKPPSSSTSISTPASATVAVSKPASIPASSMSS  282
            TSLPKPNTAPKPPSSSTSISTPASATVAVSKPASIPASSM S
Sbjct  241  TSLPKPNTAPKPPSSSTSISTPASATVAVSKPASIPASSMFS  282


>ref|XP_003191206.1| hypothetical protein CGB_A1800W [Cryptococcus gattii WM276]
 gb|ADV19419.1| Hypothetical Protein CGB_A1800W [Cryptococcus gattii WM276]
Length=293

 Score = 43.5 bits (101),  Expect = 0.23, Method: Compositional matrix adjust.
 Identities = 32/77 (42%), Positives = 40/77 (52%), Gaps = 16/77 (21%)

Query  133  NGNGNGTGSKRPG-----RDNQDSDS-------DEDEAEDRSRAVGKKAKLSNTGIDQFG  180
             GNG    SK+ G     +D +D  S       DEDE E R ++VGK  K  N  +D FG
Sbjct  79   GGNGLAGLSKKLGNQKKNKDKEDGGSLQPQQPQDEDEEESRVKSVGKGKKSVNGVLDMFG  138

Query  181  GKNKKKKKQLQQQQVPP  197
            GK K+KK    +QQV P
Sbjct  139  GKKKRKK----EQQVHP  151


>emb|CEL63680.1| hypothetical protein RSOLAG1IB_05441 [Rhizoctonia solani AG-1 
IB]
Length=443

 Score = 43.5 bits (101),  Expect = 0.33, Method: Compositional matrix adjust.
 Identities = 51/176 (29%), Positives = 82/176 (47%), Gaps = 29/176 (16%)

Query  21   LAAFQARADLSVVMLRNMMGKMVKGWVPPTAEE-LNQTDPQVKPNATRKLINKLVPRSSA  79
            +   Q+  D+SV +    +G +V+ W+PP + + LNQ+  Q     +RK + + + R   
Sbjct  24   IETIQSTVDMSVSL---ALG-LVQTWMPPASSKPLNQSVIQ-----SRKKLEEYMRRPPR  74

Query  80   ASSSTLAPSSSATTSFEQHQQSQQNNKRYRSSAVANLINQPAPSEKNTTNGTSNGNGNGT  139
                T  P+  A  +   H ++Q+   R   S  A        +E+ T         N  
Sbjct  75   LGVGTPIPT--AQNAQSTHHETQKLKNRLVGSGAARRK-----AEEET-------KLNSA  120

Query  140  GSKRPGRDNQDSDSDEDEAEDRSRAVGKK-AKLSNTGIDQF-GGKNKKKKKQLQQQ  193
            GSKRPG ++ D    EDE+E R+ A+ KK A+    G+  F GGK+ K K   + Q
Sbjct  121  GSKRPGHNDSDG---EDESESRASAMKKKQARAGVNGLSVFQGGKHSKLKSDAEPQ  173


>gb|KIR59865.1| hypothetical protein I314_04300 [Cryptococcus gattii CA1873]
Length=293

 Score = 41.6 bits (96),  Expect = 0.83, Method: Compositional matrix adjust.
 Identities = 31/77 (40%), Positives = 39/77 (51%), Gaps = 16/77 (21%)

Query  133  NGNGNGTGSKRPG-----RDNQDSDS-------DEDEAEDRSRAVGKKAKLSNTGIDQFG  180
             GNG    SK+ G     +D +D  S       DEDE E R ++VGK  K  N  +D FG
Sbjct  79   GGNGFAGLSKKLGNEKKNKDKEDGGSLPSQQPQDEDEEESRVKSVGKGKKSVNGVLDMFG  138

Query  181  GKNKKKKKQLQQQQVPP  197
            GK K+K     +QQV P
Sbjct  139  GKKKRKT----EQQVHP  151


>gb|KIR54225.1| hypothetical protein I315_03284 [Cryptococcus gattii Ru294]
 gb|KIY34334.1| hypothetical protein I305_03114 [Cryptococcus gattii E566]
Length=293

 Score = 41.2 bits (95),  Expect = 1.1, Method: Compositional matrix adjust.
 Identities = 31/77 (40%), Positives = 39/77 (51%), Gaps = 16/77 (21%)

Query  133  NGNGNGTGSKRPG-----RDNQDSDS-------DEDEAEDRSRAVGKKAKLSNTGIDQFG  180
             GNG    SK+ G     +D +D  S       DEDE E R ++VGK  K  N  +D FG
Sbjct  79   GGNGLAGLSKKLGNEKKNKDKEDGGSLQPQQPQDEDEEESRVKSVGKGKKSVNGVLDMFG  138

Query  181  GKNKKKKKQLQQQQVPP  197
            GK K+K     +QQV P
Sbjct  139  GKKKRKT----EQQVHP  151


>gb|KIR80023.1| hypothetical protein I306_02987 [Cryptococcus gattii EJB2]
 gb|KJE05306.1| hypothetical protein I311_00985 [Cryptococcus gattii NT-10]
Length=293

 Score = 41.2 bits (95),  Expect = 1.2, Method: Compositional matrix adjust.
 Identities = 31/77 (40%), Positives = 39/77 (51%), Gaps = 16/77 (21%)

Query  133  NGNGNGTGSKRPG-----RDNQDSDS-------DEDEAEDRSRAVGKKAKLSNTGIDQFG  180
             GNG    SK+ G     +D +D  S       DEDE E R ++VGK  K  N  +D FG
Sbjct  79   GGNGLAGLSKKLGNQKKNKDKEDGGSLQPQQPQDEDEEESRVKSVGKGKKSVNGVLDMFG  138

Query  181  GKNKKKKKQLQQQQVPP  197
            GK K+K     +QQV P
Sbjct  139  GKKKRKT----EQQVHP  151


>gb|KIR85507.1| hypothetical protein I308_04203 [Cryptococcus gattii IND107]
Length=293

 Score = 41.2 bits (95),  Expect = 1.3, Method: Compositional matrix adjust.
 Identities = 31/77 (40%), Positives = 39/77 (51%), Gaps = 16/77 (21%)

Query  133  NGNGNGTGSKRPG-----RDNQDSDS-------DEDEAEDRSRAVGKKAKLSNTGIDQFG  180
             GNG    SK+ G     +D +D  S       DEDE E R ++VGK  K  N  +D FG
Sbjct  79   GGNGLAGLSKKLGNEKKNKDKEDGGSLPPQQPQDEDEEESRVKSVGKGKKSVNGVLDMFG  138

Query  181  GKNKKKKKQLQQQQVPP  197
            GK K+K     +QQV P
Sbjct  139  GKKKRK----TEQQVHP  151


>emb|CUA69132.1| hypothetical protein RSOLAG22IIIB_03810 [Rhizoctonia solani]
Length=444

 Score = 41.2 bits (95),  Expect = 1.6, Method: Compositional matrix adjust.
 Identities = 51/167 (31%), Positives = 74/167 (44%), Gaps = 30/167 (18%)

Query  24   FQARADLSVVMLRNMMGKMVKGWVPPTAEE-LNQTDPQVKPNATRKLINKLVPRSSAASS  82
             Q+  D+S+    ++   +V+ W+PP +   L+Q+  Q     +RK + + + R      
Sbjct  26   IQSTVDMSL----SLALSLVQSWMPPASSTPLDQSVVQ-----SRKKLEEYMRRPPRLGV  76

Query  83   STLAPSSSATTSFEQHQQSQQNNKRYRSSAVANLINQPAPSEKNTTNGTSNGNGNGTGSK  142
             T  P++  T     H+  +  NK   S A      +                 NGTGSK
Sbjct  77   GTPIPTAQHTQP-TYHETQKLKNKLVGSGAARRKAEE-------------ESKLNGTGSK  122

Query  143  RPGRDNQDSDSDEDEAEDRSRAVGKKAKLSNTGIDQF-GGKNKKKKK  188
            RP   N DSD +EDE E  SRA   K K + TGI+ F GGK  K  K
Sbjct  123  RP--PNNDSD-EEDEPE--SRASAMKKKQARTGINVFAGGKGLKHSK  164


>ref|XP_012047557.1| hypothetical protein CNAG_03930 [Cryptococcus neoformans var. 
grubii H99]
 gb|AFR93430.1| hypothetical protein CNAG_03930 [Cryptococcus neoformans var. 
grubii H99]
Length=296

 Score = 38.5 bits (88),  Expect = 9.5, Method: Compositional matrix adjust.
 Identities = 22/44 (50%), Positives = 26/44 (59%), Gaps = 4/44 (9%)

Query  154  DEDEAEDRSRAVGKKAKLSNTGIDQFGGKNKKKKKQLQQQQVPP  197
            DEDE E R ++VGK  K  N   D FGGK K+K     +QQV P
Sbjct  112  DEDEEESRVKSVGKGKKNVNGVFDMFGGKKKRKA----EQQVHP  151


Lambda      K        H        a         alpha
   0.300    0.117    0.316    0.792     4.96 

Gapped
Lambda      K        H        a         alpha    sigma
   0.267   0.0410    0.140     1.90     42.6     43.6 

Effective search space used: 3475736195593


  Database: nr
    Posted date:  Sep 23, 2015 12:05 AM
  Number of letters in database: 26,053,659,533
  Number of sequences in database:  71,551,133


Matrix: BLOSUM62
Gap Penalties: Existence: 11, Extension: 1
Neighboring words threshold: 11
Window for multiple hits: 40
```
