# Supplementary material for: Comparative genomics provides new insights into the diversity, physiology, and sexuality of the only industrially exploited tremellomycete: Phaffia rhodozyma
Source: BMC Genomics. 2016 Nov 9;17:901. doi: 10.1186/s12864-016-3244-7 (PMC5103461; doi:10.1186/s12864-016-3244-7)
Supplement: Additional file 6: — List of orphan genes with links to PFAM (related to Additional file 1: Table S1). (ZIP 1428 kb) [file 12864_2016_3244_MOESM6_ESM.zip › BLAST_HTML_FTR/G02379_P.html]

BLAST Search Results


```
BLASTP 2.2.27+


Reference:
Stephen F. Altschul, Thomas L. Madden, Alejandro A. Schäffer,
Jinghui Zhang, Zheng Zhang, Webb Miller, and David J. Lipman (1997),
"Gapped BLAST and PSI-BLAST: a new generation of protein database
search programs", Nucleic Acids Res. 25:3389-3402.


Reference for
composition-based statistics:
Alejandro A. Schäffer, L. Aravind, Thomas L. Madden, Sergei
Shavirin, John L. Spouge, Yuri I. Wolf, Eugene V. Koonin, and
Stephen F. Altschul (2001), "Improving the accuracy of PSI-BLAST
protein database searches with composition-based statistics and
other refinements", Nucleic Acids Res. 29:2994-3005.


Database: nr
           71,551,133 sequences; 26,053,659,533 total letters


Query= G02379_P

Length=361
                                                                      Score     E
Sequences producing significant alignments:                          (Bits)  Value

emb|CED83542.1|  hypothetical protein [Xanthophyllomyces dendrorh...   732    0.0  
ref|WP_038025467.1|  ATP-dependent DNA helicase RecQ [Tenacibacul...  45.4    0.090
ref|WP_012881193.1|  adenylyl-sulfate kinase [Sinorhizobium melil...  42.0    1.0  
ref|WP_026030561.1|  adenylyl-sulfate kinase [Sinorhizobium melil...  40.8    2.3  
ref|WP_027991250.1|  adenylyl-sulfate kinase [Sinorhizobium melil...  39.7    5.0  
gb|KND18803.1|  hypothetical protein ADZ37_13075 [Pannonibacter p...  39.3    6.8  
ref|WP_050473291.1|  hypothetical protein [Pannonibacter phragmit...  39.3    6.8  


 >emb|CED83542.1| hypothetical protein [Xanthophyllomyces dendrorhous]
Length=360

 Score =  732 bits (1889),  Expect = 0.0, Method: Compositional matrix adjust.
 Identities = 360/360 (100%), Positives = 360/360 (100%), Gaps = 0/360 (0%)

Query  1    MSVLRRAVASSSSSVTQSVLSARRQICLTASLSAKKAKSGRVNINSTPSIQELDQLAEEE  60
            MSVLRRAVASSSSSVTQSVLSARRQICLTASLSAKKAKSGRVNINSTPSIQELDQLAEEE
Sbjct  1    MSVLRRAVASSSSSVTQSVLSARRQICLTASLSAKKAKSGRVNINSTPSIQELDQLAEEE  60

Query  61   SRPPAEHDANPNSFFNLGRDIIFLRGATTFFRNTRSTMLRDLANQIQTPAETREFFEYLK  120
            SRPPAEHDANPNSFFNLGRDIIFLRGATTFFRNTRSTMLRDLANQIQTPAETREFFEYLK
Sbjct  61   SRPPAEHDANPNSFFNLGRDIIFLRGATTFFRNTRSTMLRDLANQIQTPAETREFFEYLK  120

Query  121  IWRLNGGQIDWDLAQLVIERSMASSSPYPVLYALMNPTSFGLHISSPSRPAYIVPTYEST  180
            IWRLNGGQIDWDLAQLVIERSMASSSPYPVLYALMNPTSFGLHISSPSRPAYIVPTYEST
Sbjct  121  IWRLNGGQIDWDLAQLVIERSMASSSPYPVLYALMNPTSFGLHISSPSRPAYIVPTYEST  180

Query  181  PPSAQETSEILKKIREEVSTRGDILLFDRRKPHFTTLSTPTPMAKVPASIEPVGSIPVPA  240
            PPSAQETSEILKKIREEVSTRGDILLFDRRKPHFTTLSTPTPMAKVPASIEPVGSIPVPA
Sbjct  181  PPSAQETSEILKKIREEVSTRGDILLFDRRKPHFTTLSTPTPMAKVPASIEPVGSIPVPA  240

Query  241  FFRDFVRLLFVGSEEAGRSRDLTKEEVAWVAEQTDRHLPLDPVITSLLLNHYSHLPDNSG  300
            FFRDFVRLLFVGSEEAGRSRDLTKEEVAWVAEQTDRHLPLDPVITSLLLNHYSHLPDNSG
Sbjct  241  FFRDFVRLLFVGSEEAGRSRDLTKEEVAWVAEQTDRHLPLDPVITSLLLNHYSHLPDNSG  300

Query  301  QAAYGNLQSRLSAVVPETFAQQELTAEEKSVVRDQLEASAFPLKWAGEVLLPLAQTQAEA  360
            QAAYGNLQSRLSAVVPETFAQQELTAEEKSVVRDQLEASAFPLKWAGEVLLPLAQTQAEA
Sbjct  301  QAAYGNLQSRLSAVVPETFAQQELTAEEKSVVRDQLEASAFPLKWAGEVLLPLAQTQAEA  360


>ref|WP_038025467.1| ATP-dependent DNA helicase RecQ [Tenacibaculum maritimum]
Length=726

 Score = 45.4 bits (106),  Expect = 0.090, Method: Compositional matrix adjust.
 Identities = 40/152 (26%), Positives = 69/152 (45%), Gaps = 28/152 (18%)

Query  156  NPTSFGL---HISSPSRPAYIVPTYESTPPSAQETSEILKKIREEVSTRGDILLFDRRKP  212
            NP+SF +   HI + S    I+   +ST  S  +  ++LK +R++V+ +  +       P
Sbjct  501  NPSSFMMTKDHIYNDSDDGTIITNAKSTAISDDKLVKLLKDLRKKVAVKQGV-------P  553

Query  213  HFTTLSTPT--PMA-KVPASIEP------VGSIPVPAFFRDFVRLL--FVGSEEAGRSRD  261
             F     P+   MA K P ++E       VG      F +DF++L+  +V   +  R  D
Sbjct  554  PFAVFQDPSIDDMALKYPMTLEELSKVHGVGEGKARKFGKDFIKLITAYVEENDIIRPDD  613

Query  262  LTKEEVA-------WVAEQTDRHLPLDPVITS  286
            L  +          ++ + TDR LPL+ +  S
Sbjct  614  LIVKSTGVNSGLKLYIIQNTDRKLPLNDIAQS  645


>ref|WP_012881193.1| adenylyl-sulfate kinase [Sinorhizobium meliloti]
 ref|YP_003329317.1| sulfate adenylate transferase subunit 1 [Sinorhizobium meliloti]
 gb|ABA55990.1| sulfate adenylate transferase subunit 1 [Sinorhizobium meliloti]
 emb|CDH80913.1| sulfate adenylate transferase subunit 1 [Sinorhizobium meliloti 
RU11/001]
Length=636

 Score = 42.0 bits (97),  Expect = 1.0, Method: Compositional matrix adjust.
 Identities = 44/157 (28%), Positives = 67/157 (43%), Gaps = 13/157 (8%)

Query  167  PSRPAYIVPTYESTPPSAQETSEILKKIREEVSTRGDILLFDRRKPHFTTL--STPTPMA  224
            P+  A IV      P +A E +  L   RE   +RGD+L    + P F+    +    MA
Sbjct  285  PAAIARIVTMDGDLPEAAAEQAVTLVLDREIDISRGDVLTHSGQTPEFSNQFQAELVWMA  344

Query  225  KVPAS-----IEPVGSIPVPAFFRDFVRLLFVGSEEAGRSRDLTKEEVAWVAEQTDRHLP  279
              PA      +  +GS  VPA   D      V + +   ++ L   EV  V   TDR + 
Sbjct  345  DEPAYPGRSYLLKIGSQVVPATITDLKHRTNVNTLKKAAAKRLELNEVGTVTIATDRPIA  404

Query  280  LDPVITS------LLLNHYSHLPDNSGQAAYGNLQSR  310
             DP  T+      +L++   +L   +G  AYG  ++R
Sbjct  405  FDPYNTNRLTGGFILIDRIHNLTLGAGTIAYGLQRAR  441


>ref|WP_026030561.1| adenylyl-sulfate kinase [Sinorhizobium meliloti]
Length=622

 Score = 40.8 bits (94),  Expect = 2.3, Method: Compositional matrix adjust.
 Identities = 43/153 (28%), Positives = 65/153 (42%), Gaps = 13/153 (8%)

Query  171  AYIVPTYESTPPSAQETSEILKKIREEVSTRGDILLFDRRKPHFTTL--STPTPMAKVPA  228
            A IV      P +A E +  L   RE   +RGD+L    + P F+    +    MA  PA
Sbjct  275  ARIVTMDGDLPEAAAEQAVTLVLDREIDISRGDVLTHAGQTPEFSNQFQAELVWMADEPA  334

Query  229  S-----IEPVGSIPVPAFFRDFVRLLFVGSEEAGRSRDLTKEEVAWVAEQTDRHLPLDPV  283
                  +  +GS  VPA   D      V + +   ++ L   EV  V   TDR +  DP 
Sbjct  335  YPGRSYLLKIGSQVVPAIITDLKHRTNVNTLKKAAAKRLELNEVGTVTIATDRPIAFDPY  394

Query  284  ITS------LLLNHYSHLPDNSGQAAYGNLQSR  310
             T+      +L++   +L   +G  AYG  ++R
Sbjct  395  NTNRLTGGFILIDRIHNLTLGAGTIAYGLQRAR  427


>ref|WP_027991250.1| adenylyl-sulfate kinase [Sinorhizobium meliloti]
Length=636

 Score = 39.7 bits (91),  Expect = 5.0, Method: Compositional matrix adjust.
 Identities = 43/153 (28%), Positives = 65/153 (42%), Gaps = 13/153 (8%)

Query  171  AYIVPTYESTPPSAQETSEILKKIREEVSTRGDILLFDRRKPHFTTL--STPTPMAKVPA  228
            A IV      P +A E +  L   RE   +RGD+L    + P F+    +    MA  PA
Sbjct  289  ARIVTMDGDLPEAAAEQAVTLVLDREIDISRGDVLTHAGQTPEFSNQFQAELVWMADEPA  348

Query  229  S-----IEPVGSIPVPAFFRDFVRLLFVGSEEAGRSRDLTKEEVAWVAEQTDRHLPLDPV  283
                  +  +GS  VPA   D      V + +   ++ L   EV  V   TDR +  DP 
Sbjct  349  YPGRSYLLKIGSQVVPATITDLKHRTNVNTLKKAAAKRLELNEVGTVTIATDRPIAFDPY  408

Query  284  ITS------LLLNHYSHLPDNSGQAAYGNLQSR  310
             T+      +L++   +L   +G  AYG  ++R
Sbjct  409  NTNRLTGGFILIDRIHNLTLGAGTIAYGLQRAR  441


>gb|KND18803.1| hypothetical protein ADZ37_13075 [Pannonibacter phragmitetus]
Length=727

 Score = 39.3 bits (90),  Expect = 6.8, Method: Compositional matrix adjust.
 Identities = 58/231 (25%), Positives = 89/231 (39%), Gaps = 21/231 (9%)

Query  83   FLRGATTFFRNTRSTMLRDLANQIQTPAETREFFEYLKIWRLNGGQIDW--DLAQLVIER  140
            F  GA + F     T   ++ ++ + PAE R     L I +L+   + W  D+       
Sbjct  443  FHTGAISLFEGRTFT---NVVDRGEYPAEARA---TLTIDKLSETLVRWVVDIYHNTPHG  496

Query  141  SMASSSPYPVLYALMNPTSFGLHISSPSRPAYIVPTYESTPPSA-------QETSEILKK  193
             +   SP  V   L N     +        AY   T E    S        +  S  L++
Sbjct  497  GLGGRSPREVWLELKNKVGVPMSPRKDEMRAYFGITVERDLRSEGIQLYCNKYQSTELQR  556

Query  194  IREEVSTRGDILL-FDRRKPHFTTLSTPTPMAKVPASIEPVGSIPVPAFFRDFVRLLFVG  252
            IR E   R  + + FD       ++ TP    KVP +   +  IP+ A   +  R+L   
Sbjct  557  IRREFRLRKKLRVRFDPDDLGHISVETPKGWLKVPCTTPEMHGIPLEAHVENRQRMLARY  616

Query  253  SEEAGRSRDLTKEEVAWVAEQTD----RHLPLDPVITSLLLNHY-SHLPDN  298
             EEA RS  + K+ +A +    D    R     PV T+  L H  +H  D+
Sbjct  617  GEEAARSAPIVKKALADLRRLADESRARRGIASPVFTATNLAHLEAHFSDD  667


>ref|WP_050473291.1| hypothetical protein [Pannonibacter phragmitetus]
Length=729

 Score = 39.3 bits (90),  Expect = 6.8, Method: Compositional matrix adjust.
 Identities = 58/231 (25%), Positives = 89/231 (39%), Gaps = 21/231 (9%)

Query  83   FLRGATTFFRNTRSTMLRDLANQIQTPAETREFFEYLKIWRLNGGQIDW--DLAQLVIER  140
            F  GA + F     T   ++ ++ + PAE R     L I +L+   + W  D+       
Sbjct  445  FHTGAISLFEGRTFT---NVVDRGEYPAEARA---TLTIDKLSETLVRWVVDIYHNTPHG  498

Query  141  SMASSSPYPVLYALMNPTSFGLHISSPSRPAYIVPTYESTPPSA-------QETSEILKK  193
             +   SP  V   L N     +        AY   T E    S        +  S  L++
Sbjct  499  GLGGRSPREVWLELKNKVGVPMSPRKDEMRAYFGITVERDLRSEGIQLYCNKYQSTELQR  558

Query  194  IREEVSTRGDILL-FDRRKPHFTTLSTPTPMAKVPASIEPVGSIPVPAFFRDFVRLLFVG  252
            IR E   R  + + FD       ++ TP    KVP +   +  IP+ A   +  R+L   
Sbjct  559  IRREFRLRKKLRVRFDPDDLGHISVETPKGWLKVPCTTPEMHGIPLEAHVENRQRMLARY  618

Query  253  SEEAGRSRDLTKEEVAWVAEQTD----RHLPLDPVITSLLLNHY-SHLPDN  298
             EEA RS  + K+ +A +    D    R     PV T+  L H  +H  D+
Sbjct  619  GEEAARSAPIVKKALADLRRLADESRARRGIASPVFTATNLAHLEAHFSDD  669


Lambda      K        H        a         alpha
   0.317    0.130    0.372    0.792     4.96 

Gapped
Lambda      K        H        a         alpha    sigma
   0.267   0.0410    0.140     1.90     42.6     43.6 

Effective search space used: 3172178449253


  Database: nr
    Posted date:  Sep 23, 2015 12:05 AM
  Number of letters in database: 26,053,659,533
  Number of sequences in database:  71,551,133


Matrix: BLOSUM62
Gap Penalties: Existence: 11, Extension: 1
Neighboring words threshold: 11
Window for multiple hits: 40
```
