# Supplementary material for: Comparative genomics provides new insights into the diversity, physiology, and sexuality of the only industrially exploited tremellomycete: Phaffia rhodozyma
Source: BMC Genomics. 2016 Nov 9;17:901. doi: 10.1186/s12864-016-3244-7 (PMC5103461; doi:10.1186/s12864-016-3244-7)
Supplement: Additional file 6: — List of orphan genes with links to PFAM (related to Additional file 1: Table S1). (ZIP 1428 kb) [file 12864_2016_3244_MOESM6_ESM.zip › BLAST_HTML_FTR/G02859_P.html]

BLAST Search Results


```
BLASTP 2.2.27+


Reference:
Stephen F. Altschul, Thomas L. Madden, Alejandro A. Schäffer,
Jinghui Zhang, Zheng Zhang, Webb Miller, and David J. Lipman (1997),
"Gapped BLAST and PSI-BLAST: a new generation of protein database
search programs", Nucleic Acids Res. 25:3389-3402.


Reference for
composition-based statistics:
Alejandro A. Schäffer, L. Aravind, Thomas L. Madden, Sergei
Shavirin, John L. Spouge, Yuri I. Wolf, Eugene V. Koonin, and
Stephen F. Altschul (2001), "Improving the accuracy of PSI-BLAST
protein database searches with composition-based statistics and
other refinements", Nucleic Acids Res. 29:2994-3005.


Database: nr
           71,551,133 sequences; 26,053,659,533 total letters


Query= G02859_P

Length=138
                                                                      Score     E
Sequences producing significant alignments:                          (Bits)  Value

emb|CED84697.1|  hypothetical protein [Xanthophyllomyces dendrorh...   167    9e-50
ref|WP_034776919.1|  cell division protein FtsX [Idiomarina salin...  39.7    0.40 
gb|ESU44496.1|  Hypothetical protein GSB_94003, partial [Giardia ...  39.7    0.48 
gb|EET02546.1|  High cysteine protein [Giardia intestinalis ATCC ...  39.7    0.50 
gb|ESU35948.1|  Hypothetical protein DHA2_94003 [Giardia intestin...  39.3    0.64 
ref|XP_008086746.1|  hypothetical protein GLAREA_01339 [Glarea lo...  39.3    0.66 
ref|WP_052196457.1|  chemotaxis protein [Halomonas hydrothermalis]    38.5    1.1  
gb|KHG34691.1|  membrane protein [Sulfurospirillum sp. MES]           38.1    1.5  
gb|KHJ50571.1|  chemotaxis protein [Halomonas hydrothermalis]         38.1    1.6  
ref|WP_028997722.1|  chain-length determining protein [Azohydromo...  37.4    3.2  
ref|XP_004235119.1|  PREDICTED: receptor-like protein kinase [Sol...  37.0    4.6  
ref|WP_053777402.1|  FAD-dependent oxidoreductase [Gordonia sp. N...  36.6    5.7  
ref|XP_001748963.1|  hypothetical protein [Monosiga brevicollis M...  36.2    6.2  
ref|XP_011127335.1|  hypothetical protein AOL_s00210g231 [Arthrob...  35.8    7.3  


 >emb|CED84697.1| hypothetical protein [Xanthophyllomyces dendrorhous]
Length=128

 Score =  167 bits (422),  Expect = 9e-50, Method: Compositional matrix adjust.
 Identities = 91/118 (77%), Positives = 94/118 (80%), Gaps = 10/118 (8%)

Query  1    MSVLSSPVIMATIALLVTNVAHGLPMNDQPLVRAYSDLDPAPDVPTTESRSGSDGGSKVS  60
            MSVLSSPVIMATIALLVTNVAHGLPMNDQPLVRAYSDLDPAPDVPTTESRSGSDGGSKVS
Sbjct  1    MSVLSSPVIMATIALLVTNVAHGLPMNDQPLVRAYSDLDPAPDVPTTESRSGSDGGSKVS  60

Query  61   STTIIVVAVILGVLVTLGVGGMMSMLVR----------RNRRRTDVAKFSSVGCQTKF  108
            STTIIVVAVILGVLVTLGVGGMMSML R           NR     + F+   C  + 
Sbjct  61   STTIIVVAVILGVLVTLGVGGMMSMLDRNLQGFMIQESHNRSTRSPSSFNGFLCSNQL  118


>ref|WP_034776919.1| cell division protein FtsX [Idiomarina salinarum]
 gb|KFZ30156.1| cell division protein FtsX [Idiomarina salinarum]
Length=405

 Score = 39.7 bits (91),  Expect = 0.40, Method: Compositional matrix adjust.
 Identities = 28/86 (33%), Positives = 42/86 (49%), Gaps = 15/86 (17%)

Query  47   TESRSGSDGGSKVSSTTIIVVAVILGVLVTLGVGGMMSMLVRRNRRRTDVAKFSSVGCQT  106
            TE+R  S  G +   T + +  V+L ++  LG+ GM++  V  NRRR  +          
Sbjct  267  TETREDSYRGHQALQTVLWITVVLLTIITALGIVGMVTFNV--NRRRKQI----------  314

Query  107  KFGSRRGLG-RRDDLPGYELTEPLPV  131
              G+RR LG  R D+ GY + E   V
Sbjct  315  --GTRRALGASRGDIMGYFMIENFMV  338


>gb|ESU44496.1| Hypothetical protein GSB_94003, partial [Giardia intestinalis]
Length=548

 Score = 39.7 bits (91),  Expect = 0.48, Method: Composition-based stats.
 Identities = 21/55 (38%), Positives = 35/55 (64%), Gaps = 1/55 (2%)

Query  50   RSGSDGGSKVSSTTIIVVAVILGVLVTLGVGGMMS-MLVRRNRRRTDVAKFSSVG  103
            + GSDG + V S+++I +AVI+ +L+ L  GG+ +  LV R +RR    + +S G
Sbjct  471  KKGSDGITCVPSSSVIALAVIVPILLILTAGGLCTYFLVCRGKRRRPAKQAASAG  525


>gb|EET02546.1| High cysteine protein [Giardia intestinalis ATCC 50581]
Length=677

 Score = 39.7 bits (91),  Expect = 0.50, Method: Composition-based stats.
 Identities = 21/55 (38%), Positives = 35/55 (64%), Gaps = 1/55 (2%)

Query  50   RSGSDGGSKVSSTTIIVVAVILGVLVTLGVGGMMS-MLVRRNRRRTDVAKFSSVG  103
            + GSDG + V S+++I +AVI+ +L+ L  GG+ +  LV R +RR    + +S G
Sbjct  600  KKGSDGITCVPSSSVIALAVIVPILLILTAGGLCTYFLVCRGKRRRPAKQAASAG  654


>gb|ESU35948.1| Hypothetical protein DHA2_94003 [Giardia intestinalis]
Length=677

 Score = 39.3 bits (90),  Expect = 0.64, Method: Composition-based stats.
 Identities = 22/55 (40%), Positives = 35/55 (64%), Gaps = 1/55 (2%)

Query  50   RSGSDGGSKVSSTTIIVVAVILGVLVTLGVGGM-MSMLVRRNRRRTDVAKFSSVG  103
            +  SDG + V S+++I +AVI+ +L+ L VGG+ +  LV R +RR    + SS G
Sbjct  600  KKSSDGITCVPSSSVIALAVIVPILLVLTVGGLCIYFLVCRGKRREPAKQASSKG  654


>ref|XP_008086746.1| hypothetical protein GLAREA_01339 [Glarea lozoyensis ATCC 20868]
 gb|EPE25427.1| hypothetical protein GLAREA_01339 [Glarea lozoyensis ATCC 20868]
Length=590

 Score = 39.3 bits (90),  Expect = 0.66, Method: Compositional matrix adjust.
 Identities = 30/95 (32%), Positives = 49/95 (52%), Gaps = 11/95 (12%)

Query  7    PVIMATIALLVTNVAHGLP----MNDQPLVRAYSDLDPAPDVPTTESRSGSDGGSKVSST  62
            P +MAT+ ++VT  +   P     ++QP V   ++   +  +PTT +R    G S +  T
Sbjct  449  PSLMATVTMMVTPTSAIQPGTTATSNQPPVTGVANEASSTRLPTTSTR----GVSLI--T  502

Query  63   TIIVVAVILGVLVTLGVGGMMSMLVRRNRRRTDVA  97
             +I+V  I+G+    GV      L RR R+R D+A
Sbjct  503  LVIIVFAIVGLFAIFGVVAFC-FLRRRKRKRADIA  536


>ref|WP_052196457.1| chemotaxis protein [Halomonas hydrothermalis]
Length=542

 Score = 38.5 bits (88),  Expect = 1.1, Method: Compositional matrix adjust.
 Identities = 29/75 (39%), Positives = 39/75 (52%), Gaps = 6/75 (8%)

Query  18   TNVAHGLPMND-QPLVRAYSDLDPAPDVPTTESRSGSDGGSKVSSTTI--IVVAVILGVL  74
            +N A G   ND  PLVR YS+     DV         D  S + S  I  IVV ++ G L
Sbjct  170  SNFARG---NDGSPLVRTYSEAFAPWDVYVASGVFVGDVNSTIVSQLIETIVVGIVAGAL  226

Query  75   VTLGVGGMMSMLVRR  89
            VTL   GM+++++RR
Sbjct  227  VTLAFWGMITLILRR  241


>gb|KHG34691.1| membrane protein [Sulfurospirillum sp. MES]
Length=265

 Score = 38.1 bits (87),  Expect = 1.5, Method: Compositional matrix adjust.
 Identities = 20/51 (39%), Positives = 25/51 (49%), Gaps = 4/51 (8%)

Query  74   LVTLGVGGMMSMLVRRNRRRTDVAKFSSVGCQTKFGSRRGLGRRDDLPGYE  124
            L  LG+GG+M+ L  RN  R D    S +G    FG   G+   D  PGY 
Sbjct  67   LFALGLGGIMAYLTHRNNTRLD----SLIGVLWAFGMAFGIIMTDLTPGYN  113


>gb|KHJ50571.1| chemotaxis protein [Halomonas hydrothermalis]
Length=513

 Score = 38.1 bits (87),  Expect = 1.6, Method: Composition-based stats.
 Identities = 29/75 (39%), Positives = 39/75 (52%), Gaps = 6/75 (8%)

Query  18   TNVAHGLPMND-QPLVRAYSDLDPAPDVPTTESRSGSDGGSKVSSTTI--IVVAVILGVL  74
            +N A G   ND  PLVR YS+     DV         D  S + S  I  IVV ++ G L
Sbjct  141  SNFARG---NDGSPLVRTYSEAFAPWDVYVASGVFVGDVNSTIVSQLIETIVVGIVAGAL  197

Query  75   VTLGVGGMMSMLVRR  89
            VTL   GM+++++RR
Sbjct  198  VTLAFWGMITLILRR  212


>ref|WP_028997722.1| chain-length determining protein [Azohydromonas australica]
Length=473

 Score = 37.4 bits (85),  Expect = 3.2, Method: Compositional matrix adjust.
 Identities = 24/69 (35%), Positives = 36/69 (52%), Gaps = 10/69 (14%)

Query  66   VVAVILGVLVTLGVGGMMSMLVRRNRRRTDVAKFSS---VGCQTK-------FGSRRGLG  115
            +VAV LG ++ +GV  M+  + RR R   DVA+ +    +G   K       FG+RRG  
Sbjct  401  IVAVFLGSILAVGVALMLEFMNRRVRSPNDVAQLADLHVIGYLPKPDRKTWLFGARRGAS  460

Query  116  RRDDLPGYE  124
             +  L GY+
Sbjct  461  MQTRLVGYQ  469


>ref|XP_004235119.1| PREDICTED: receptor-like protein kinase [Solanum lycopersicum]
Length=1115

 Score = 37.0 bits (84),  Expect = 4.6, Method: Composition-based stats.
 Identities = 18/41 (44%), Positives = 24/41 (59%), Gaps = 0/41 (0%)

Query  3    VLSSPVIMATIALLVTNVAHGLPMNDQPLVRAYSDLDPAPD  43
            VLS  VI   I LLVT + H   +N  PLV+  S+++  PD
Sbjct  784  VLSMAVIAVAIYLLVTRIQHASLLNKHPLVKYQSEIEDLPD  824


>ref|WP_053777402.1| FAD-dependent oxidoreductase [Gordonia sp. NB4-1Y]
 gb|KOY49557.1| FAD-dependent oxidoreductase [Gordonia sp. NB4-1Y]
Length=542

 Score = 36.6 bits (83),  Expect = 5.7, Method: Compositional matrix adjust.
 Identities = 29/83 (35%), Positives = 43/83 (52%), Gaps = 11/83 (13%)

Query  51   SGSDGGSKVSSTTIIVVAVILGVLVTLGVGGMMSMLVRRNRRRTDVAKFSSVGCQTKFGS  110
            +G+DG S+ + TT+I    IL  L  LG GG    L+  +R RT ++  S +G   + G+
Sbjct  285  TGTDGQSRCA-TTVIAACHILTTLDLLGAGGFAPDLL--DRWRTQISVGSGMGMAVRVGA  341

Query  111  RRGLGRRDDLPGY-ELTEPLPVY  132
            R       DLP Y +L   LP +
Sbjct  342  R-------DLPVYRDLPSDLPAH  357


>ref|XP_001748963.1| hypothetical protein [Monosiga brevicollis MX1]
 gb|EDQ86293.1| predicted protein [Monosiga brevicollis MX1]
Length=381

 Score = 36.2 bits (82),  Expect = 6.2, Method: Compositional matrix adjust.
 Identities = 19/70 (27%), Positives = 35/70 (50%), Gaps = 1/70 (1%)

Query  29   QPLVRAYSDLDPAPDVPTTESRSGSDGGSKVSSTTIIVVAVILGVLVTLGVGGMMSMLVR  88
            +P  + + D+  +   P   ++  +DGG K  S + + V  +  V   L VG +++ +V 
Sbjct  273  EPCDKEFFDMLHSKSSPAPSTQPTADGGKKKKSNSAMAVTAVTAVACVLAVGALVAYVVH  332

Query  89   RNRR-RTDVA  97
            R R  R D+A
Sbjct  333  RRRSARVDLA  342


>ref|XP_011127335.1| hypothetical protein AOL_s00210g231 [Arthrobotrys oligospora 
ATCC 24927]
 gb|EGX44070.1| hypothetical protein AOL_s00210g231 [Arthrobotrys oligospora 
ATCC 24927]
Length=155

 Score = 35.8 bits (81),  Expect = 7.3, Method: Compositional matrix adjust.
 Identities = 31/103 (30%), Positives = 51/103 (50%), Gaps = 8/103 (8%)

Query  25   PMNDQPLVRAYSDLDPAPDVPTTESRSGSDGGSKVSSTTIIVVAVILGVLVTLGVGGMMS  84
            P    P+ +A        D  T E++  + GGSK+S+  I  V + + VLV + + G + 
Sbjct  59   PATTNPISKATPS-----DTSTAETQGEAAGGSKLSAGAIAGVTIGVTVLV-VALVGFLF  112

Query  85   MLVRRNRRRTDVAKFSSVGCQTKFGSRRGLGRRDDLPGYELTE  127
            +L RR  RR  V   S+     +  +  G+G  +D+PG +L E
Sbjct  113  ILFRRKGRRLHVPVLSTSQSH-ETNNWGGIGPDNDIPG-QLNE  153


Lambda      K        H        a         alpha
   0.322    0.137    0.393    0.792     4.96 

Gapped
Lambda      K        H        a         alpha    sigma
   0.267   0.0410    0.140     1.90     42.6     43.6 

Effective search space used: 653936249190


  Database: nr
    Posted date:  Sep 23, 2015 12:05 AM
  Number of letters in database: 26,053,659,533
  Number of sequences in database:  71,551,133


Matrix: BLOSUM62
Gap Penalties: Existence: 11, Extension: 1
Neighboring words threshold: 11
Window for multiple hits: 40
```
