# Supplementary material for: Comparative genomics provides new insights into the diversity, physiology, and sexuality of the only industrially exploited tremellomycete: Phaffia rhodozyma
Source: BMC Genomics. 2016 Nov 9;17:901. doi: 10.1186/s12864-016-3244-7 (PMC5103461; doi:10.1186/s12864-016-3244-7)
Supplement: Additional file 6: — List of orphan genes with links to PFAM (related to Additional file 1: Table S1). (ZIP 1428 kb) [file 12864_2016_3244_MOESM6_ESM.zip › BLAST_HTML_FTR/G03007_P.html]

BLAST Search Results


```
BLASTP 2.2.27+


Reference:
Stephen F. Altschul, Thomas L. Madden, Alejandro A. Schäffer,
Jinghui Zhang, Zheng Zhang, Webb Miller, and David J. Lipman (1997),
"Gapped BLAST and PSI-BLAST: a new generation of protein database
search programs", Nucleic Acids Res. 25:3389-3402.


Reference for
composition-based statistics:
Alejandro A. Schäffer, L. Aravind, Thomas L. Madden, Sergei
Shavirin, John L. Spouge, Yuri I. Wolf, Eugene V. Koonin, and
Stephen F. Altschul (2001), "Improving the accuracy of PSI-BLAST
protein database searches with composition-based statistics and
other refinements", Nucleic Acids Res. 29:2994-3005.


Database: nr
           71,551,133 sequences; 26,053,659,533 total letters


Query= G03007_P

Length=364
                                                                      Score     E
Sequences producing significant alignments:                          (Bits)  Value

emb|CED82646.1|  FAS1 domain [Xanthophyllomyces dendrorhous]           723    0.0  
gb|KII85364.1|  hypothetical protein PLICRDRAFT_178446 [Plicaturo...  89.4    3e-16


 >emb|CED82646.1| FAS1 domain [Xanthophyllomyces dendrorhous]
Length=363

 Score =  723 bits (1867),  Expect = 0.0, Method: Compositional matrix adjust.
 Identities = 363/363 (100%), Positives = 363/363 (100%), Gaps = 0/363 (0%)

Query  1    MSTTAWAYWAQTADHALIPLFKEIEKAGANYTIAVPTIAGFRTFTTSKGVNTSDLATLNS  60
            MSTTAWAYWAQTADHALIPLFKEIEKAGANYTIAVPTIAGFRTFTTSKGVNTSDLATLNS
Sbjct  1    MSTTAWAYWAQTADHALIPLFKEIEKAGANYTIAVPTIAGFRTFTTSKGVNTSDLATLNS  60

Query  61   KGWKSTWADAYPYHFLKSTVKTTAVKSNQTIILDSYKTSPAGDWPLQMVYYMDGNIKAKD  120
            KGWKSTWADAYPYHFLKSTVKTTAVKSNQTIILDSYKTSPAGDWPLQMVYYMDGNIKAKD
Sbjct  61   KGWKSTWADAYPYHFLKSTVKTTAVKSNQTIILDSYKTSPAGDWPLQMVYYMDGNIKAKD  120

Query  121  VSAASTSSSSSNSTTSTSGHGSSSSNSTTSASGHGSSSSNSTTVVASSTSTATDSAASAA  180
            VSAASTSSSSSNSTTSTSGHGSSSSNSTTSASGHGSSSSNSTTVVASSTSTATDSAASAA
Sbjct  121  VSAASTSSSSSNSTTSTSGHGSSSSNSTTSASGHGSSSSNSTTVVASSTSTATDSAASAA  180

Query  181  TSTTTRTHNHKRARGGKGTTTSSVDMSGIFNGTFGDSFYGQYNVTIPDTTVGTFGIAQAV  240
            TSTTTRTHNHKRARGGKGTTTSSVDMSGIFNGTFGDSFYGQYNVTIPDTTVGTFGIAQAV
Sbjct  181  TSTTTRTHNHKRARGGKGTTTSSVDMSGIFNGTFGDSFYGQYNVTIPDTTVGTFGIAQAV  240

Query  241  SGWVNEPKWLKDTLSDLSINYTSLVALYPSFANLSTSRYTLFLPPSSSIPTYSGAAFESF  300
            SGWVNEPKWLKDTLSDLSINYTSLVALYPSFANLSTSRYTLFLPPSSSIPTYSGAAFESF
Sbjct  241  SGWVNEPKWLKDTLSDLSINYTSLVALYPSFANLSTSRYTLFLPPSSSIPTYSGAAFESF  300

Query  301  VGKHLVKSLAYSPMYGSGGANLTAQDGSTIAWTDGGVNGATVLHRDVLTNAMVLQLIDQP  360
            VGKHLVKSLAYSPMYGSGGANLTAQDGSTIAWTDGGVNGATVLHRDVLTNAMVLQLIDQP
Sbjct  301  VGKHLVKSLAYSPMYGSGGANLTAQDGSTIAWTDGGVNGATVLHRDVLTNAMVLQLIDQP  360

Query  361  FTN  363
            FTN
Sbjct  361  FTN  363


>gb|KII85364.1| hypothetical protein PLICRDRAFT_178446 [Plicaturopsis crispa 
FD-325 SS-3]
Length=414

 Score = 89.4 bits (220),  Expect = 3e-16, Method: Compositional matrix adjust.
 Identities = 49/132 (37%), Positives = 73/132 (55%), Gaps = 3/132 (2%)

Query  1    MSTTAWAYWAQTADHALIPLFKEIEKAGANYTIAVPTIAGFRTFTTSKGVNTSDLATLNS  60
             +TTA+  W Q A   ++P+ K         TI VP+ A F  F TS+G N SD A++++
Sbjct  42   FTTTAFGAWIQLAADTVLPILKN--PNSKELTIVVPSNAAFNDFVTSRGGNISDPASIST  99

Query  61   KGWKSTWADAYPYHFLKSTVKTTAVKSNQTIILDSYKTSPAGDWPLQMVYYMDGNIKAKD  120
              W   W D +PY F +  + TT + SN T +LDS KTS  GDWPL +    D + +A D
Sbjct  100  AEWVQVWEDVFPYFFFQG-IYTTDLLSNTTTVLDSVKTSALGDWPLSIGLRFDEDQEAAD  158

Query  121  VSAASTSSSSSN  132
             S ++ S++  +
Sbjct  159  NSTSTASNTRRD  170


 Score = 54.3 bits (129),  Expect = 9e-05, Method: Compositional matrix adjust.
 Identities = 50/180 (28%), Positives = 79/180 (44%), Gaps = 32/180 (18%)

Query  209  IFNGTFGDSFYGQYNVTIPDTTVGTFGIAQAVSGWVNEPKWLKDTLSDLSIN-YTSLVAL  267
            ++NGT   + YG  N+ IPDT +      Q V  W   P+ L  T++ L++  ++++ + 
Sbjct  195  VYNGTVYSTPYGSGNIIIPDTPLTDTIYIQVVDYWPTLPRNLSATIAALNVTAWSAISSR  254

Query  268  YPSFANLSTS-RYTLFLPPSSSIP---TYSGAAFESFVGKHLVK----------------  307
             PS A L T   YTL LPP  ++    + S +A  +F+  H++                 
Sbjct  255  LPSIAGLETQYNYTLLLPPQDALSEALSLSDSALLAFMDAHVISGARVVLPAAGQNYTSA  314

Query  308  ---SLAYSPMYGSGGANLTAQDGSTIAWTDGGVNG--------ATVLHRDVLTNAMVLQL  356
               S+  S   G  G+     DGS  A   G   G        A V  RDVLT+  ++Q+
Sbjct  315  AGGSVKISQTAGVDGSQTGVADGSQTAVVGGSQTGIVVAGSVEAKVTARDVLTDGGIVQV  374


Lambda      K        H        a         alpha
   0.311    0.123    0.357    0.792     4.96 

Gapped
Lambda      K        H        a         alpha    sigma
   0.267   0.0410    0.140     1.90     42.6     43.6 

Effective search space used: 3217712111204


  Database: nr
    Posted date:  Sep 23, 2015 12:05 AM
  Number of letters in database: 26,053,659,533
  Number of sequences in database:  71,551,133


Matrix: BLOSUM62
Gap Penalties: Existence: 11, Extension: 1
Neighboring words threshold: 11
Window for multiple hits: 40
```
