# Supplementary material for: Comparative genomics provides new insights into the diversity, physiology, and sexuality of the only industrially exploited tremellomycete: Phaffia rhodozyma
Source: BMC Genomics. 2016 Nov 9;17:901. doi: 10.1186/s12864-016-3244-7 (PMC5103461; doi:10.1186/s12864-016-3244-7)
Supplement: Additional file 6: — List of orphan genes with links to PFAM (related to Additional file 1: Table S1). (ZIP 1428 kb) [file 12864_2016_3244_MOESM6_ESM.zip › BLAST_HTML_FTR/G01725_P.html]

BLAST Search Results


```
BLASTP 2.2.27+


Reference:
Stephen F. Altschul, Thomas L. Madden, Alejandro A. Schäffer,
Jinghui Zhang, Zheng Zhang, Webb Miller, and David J. Lipman (1997),
"Gapped BLAST and PSI-BLAST: a new generation of protein database
search programs", Nucleic Acids Res. 25:3389-3402.


Reference for
composition-based statistics:
Alejandro A. Schäffer, L. Aravind, Thomas L. Madden, Sergei
Shavirin, John L. Spouge, Yuri I. Wolf, Eugene V. Koonin, and
Stephen F. Altschul (2001), "Improving the accuracy of PSI-BLAST
protein database searches with composition-based statistics and
other refinements", Nucleic Acids Res. 29:2994-3005.


Database: nr
           71,551,133 sequences; 26,053,659,533 total letters


Query= G01725_P

Length=207
                                                                      Score     E
Sequences producing significant alignments:                          (Bits)  Value

emb|CDZ96208.1|  hypothetical protein [Xanthophyllomyces dendrorh...   377    2e-129
ref|XP_011981174.1|  PREDICTED: aftiphilin isoform X2 [Ovis aries...  42.7    0.16  
ref|XP_011981170.1|  PREDICTED: aftiphilin isoform X1 [Ovis aries...  42.7    0.17  
ref|XP_005686857.1|  PREDICTED: aftiphilin isoform X2 [Capra hircus]  42.0    0.36  
ref|XP_005686856.1|  PREDICTED: aftiphilin isoform X1 [Capra hircus]  41.6    0.39  
ref|XP_005963508.1|  PREDICTED: aftiphilin isoform X1 [Pantholops...  41.2    0.50  
ref|XP_012332656.1|  PREDICTED: aftiphilin isoform X2 [Aotus nanc...  41.2    0.50  
ref|XP_012332654.1|  PREDICTED: aftiphilin isoform X1 [Aotus nanc...  41.2    0.52  
ref|XP_005963509.1|  PREDICTED: aftiphilin isoform X2 [Pantholops...  41.2    0.54  
ref|XP_005963510.1|  PREDICTED: aftiphilin isoform X3 [Pantholops...  41.2    0.56  
ref|XP_004005887.1|  PREDICTED: aftiphilin isoform X1 [Ovis aries...  40.8    0.69  
ref|XP_004005886.1|  PREDICTED: aftiphilin isoform X2 [Ovis aries]    40.8    0.69  
ref|XP_004029369.1|  PREDICTED: aftiphilin isoform 1 [Gorilla gor...  40.4    1.0   
ref|XP_004029370.1|  PREDICTED: aftiphilin isoform 2 [Gorilla gor...  40.4    1.1   
ref|XP_007189803.1|  PREDICTED: aftiphilin isoform X5 [Balaenopte...  40.4    1.1   
ref|XP_007464175.1|  PREDICTED: aftiphilin isoform X2 [Lipotes ve...  40.4    1.2   
ref|XP_007464174.1|  PREDICTED: aftiphilin isoform X1 [Lipotes ve...  40.0    1.2   
ref|XP_008252563.1|  PREDICTED: aftiphilin isoform X3 [Oryctolagu...  40.0    1.3   
ref|XP_007189802.1|  PREDICTED: aftiphilin isoform X4 [Balaenopte...  40.0    1.4   
ref|XP_007189799.1|  PREDICTED: aftiphilin isoform X1 [Balaenopte...  40.0    1.5   
ref|XP_008252562.1|  PREDICTED: aftiphilin isoform X2 [Oryctolagu...  40.0    1.5   
ref|XP_002709726.1|  PREDICTED: aftiphilin isoform X1 [Oryctolagu...  39.7    1.6   
dbj|BAE45715.1|  putative protein product of Nbla10388 [Homo sapi...  39.7    1.8   
ref|XP_005322125.1|  PREDICTED: aftiphilin isoform X3 [Ictidomys ...  39.7    1.8   
ref|XP_013212663.1|  PREDICTED: aftiphilin isoform X1 [Ictidomys ...  39.7    1.8   
ref|XP_013212665.1|  PREDICTED: aftiphilin isoform X2 [Ictidomys ...  39.7    1.9   
ref|XP_013005489.1|  PREDICTED: aftiphilin [Cavia porcellus]          39.7    1.9   
ref|XP_005575804.1|  PREDICTED: aftiphilin isoform X3 [Macaca fas...  39.3    2.5   
ref|XP_011894996.1|  PREDICTED: aftiphilin isoform X3 [Cercocebus...  39.3    2.5   
ref|XP_003262504.1|  PREDICTED: aftiphilin [Nomascus leucogenys]      39.3    2.6   
emb|CAE46209.1|  hypothetical protein [Homo sapiens]                  39.3    2.6   
gb|EAW99940.1|  aftiphilin protein, isoform CRA_d [Homo sapiens]      39.3    2.7   
ref|NP_001244656.1|  aftiphilin [Macaca mulatta] >ref|XP_00557580...  39.3    2.7   
ref|XP_011894995.1|  PREDICTED: aftiphilin isoform X2 [Cercocebus...  39.3    2.7   
ref|XP_011894993.1|  PREDICTED: aftiphilin isoform X1 [Cercocebus...  39.3    2.7   
ref|XP_011840879.1|  PREDICTED: aftiphilin [Mandrillus leucophaeus]   39.3    2.7   
ref|XP_005575802.1|  PREDICTED: aftiphilin isoform X1 [Macaca fas...  39.3    2.7   
gb|EHH22164.1|  hypothetical protein EGK_05380 [Macaca mulatta]       39.3    2.7   
gb|EHH55612.1|  hypothetical protein EGM_04853 [Macaca fascicularis]  39.3    2.7   
ref|XP_004321595.1|  PREDICTED: aftiphilin-like, partial [Tursiop...  39.3    2.7   
ref|NP_060127.3|  aftiphilin isoform b [Homo sapiens] >dbj|BAA909...  39.3    2.7   
ref|XP_007968638.1|  PREDICTED: aftiphilin isoform X3 [Chlorocebu...  39.3    2.8   
ref|XP_004280676.1|  PREDICTED: aftiphilin isoform X1 [Orcinus orca]  39.3    2.8   
ref|XP_003908780.1|  PREDICTED: aftiphilin isoform X1 [Papio anub...  39.3    2.8   
ref|XP_004280677.1|  PREDICTED: aftiphilin isoform X2 [Orcinus orca]  39.3    2.8   
ref|XP_003908779.1|  PREDICTED: aftiphilin isoform X2 [Papio anubis]  39.3    2.8   
ref|NP_001002243.1|  aftiphilin isoform c [Homo sapiens]              39.3    2.8   
gb|AAH47529.1|  Aftiphilin [Homo sapiens]                             39.3    2.8   
ref|XP_007968636.1|  PREDICTED: aftiphilin isoform X1 [Chlorocebu...  39.3    2.8   
ref|XP_009235528.1|  PREDICTED: LOW QUALITY PROTEIN: aftiphilin-l...  38.9    2.8   
dbj|BAD96541.1|  aftiphilin protein isoform b variant [Homo sapiens]  39.3    2.8   
ref|XP_011800696.1|  PREDICTED: aftiphilin [Colobus angolensis pa...  39.3    2.8   
ref|XP_005264437.1|  PREDICTED: aftiphilin isoform X1 [Homo sapie...  39.3    2.8   
ref|XP_515513.2|  PREDICTED: aftiphilin isoform X4 [Pan troglodytes]  38.9    2.8   
ref|XP_009440833.1|  PREDICTED: aftiphilin isoform X3 [Pan troglo...  38.9    2.8   
ref|XP_008954570.1|  PREDICTED: aftiphilin isoform X3 [Pan paniscus]  38.9    2.8   
ref|XP_007968637.1|  PREDICTED: aftiphilin isoform X2 [Chlorocebu...  38.9    2.9   
ref|XP_003830944.1|  PREDICTED: aftiphilin isoform X4 [Pan paniscus]  38.9    2.9   
ref|NP_982261.2|  aftiphilin isoform a [Homo sapiens]                 38.9    2.9   
gb|AAR14726.1|  brain aftiphilin [Homo sapiens]                       38.9    2.9   
ref|XP_008954568.1|  PREDICTED: aftiphilin isoform X1 [Pan paniscus]  38.9    2.9   
ref|XP_001165739.1|  PREDICTED: aftiphilin isoform X2 [Pan troglo...  38.9    3.0   
ref|XP_003830945.1|  PREDICTED: aftiphilin isoform X2 [Pan paniscus]  38.9    3.0   
ref|XP_009440832.1|  PREDICTED: aftiphilin isoform X1 [Pan troglo...  38.9    3.0   
ref|XP_007954155.1|  PREDICTED: aftiphilin isoform X1 [Orycteropu...  38.9    3.3   
ref|XP_007954156.1|  PREDICTED: aftiphilin isoform X2 [Orycteropu...  38.9    3.4   
ref|XP_002757747.1|  PREDICTED: aftiphilin isoform X2 [Callithrix...  38.5    4.2   
ref|XP_002757746.1|  PREDICTED: aftiphilin isoform X1 [Callithrix...  38.5    4.2   
ref|XP_010331840.1|  PREDICTED: aftiphilin isoform X1 [Saimiri bo...  38.5    4.3   
ref|XP_010331842.1|  PREDICTED: aftiphilin isoform X2 [Saimiri bo...  38.5    4.4   
ref|WP_011035095.1|  hypothetical protein [Methanosarcina mazei] ...  35.4    5.6   
sp|A8Z5Z0|LON_SULMW  RecName: Full=Lon protease; AltName: Full=AT...  38.1    5.8   
ref|XP_010356484.1|  PREDICTED: aftiphilin [Rhinopithecus roxellana]  37.7    6.9   
ref|XP_012620378.1|  PREDICTED: aftiphilin isoform X4 [Microcebus...  37.7    7.9   
ref|XP_012620376.1|  PREDICTED: aftiphilin isoform X2 [Microcebus...  37.7    8.0   
ref|XP_012620377.1|  PREDICTED: aftiphilin isoform X3 [Microcebus...  37.7    8.6   
ref|XP_012620375.1|  PREDICTED: aftiphilin isoform X1 [Microcebus...  37.7    8.6   
ref|XP_007124965.1|  PREDICTED: aftiphilin isoform X5 [Physeter c...  37.7    8.7   
ref|XP_007124964.1|  PREDICTED: aftiphilin isoform X4 [Physeter c...  37.7    8.7   
ref|XP_007124961.1|  PREDICTED: aftiphilin isoform X1 [Physeter c...  37.4    9.2   
ref|XP_007124963.1|  PREDICTED: aftiphilin isoform X3 [Physeter c...  37.4    9.3   


 >emb|CDZ96208.1| hypothetical protein [Xanthophyllomyces dendrorhous]
Length=261

 Score =  377 bits (969),  Expect = 2e-129, Method: Compositional matrix adjust.
 Identities = 186/186 (100%), Positives = 186/186 (100%), Gaps = 0/186 (0%)

Query  21   QLNVPSSPSPVNRTVSTIMFSRAKRIKLAKRVRQTQLVNMINQINHIKKINKINKINKTD  80
            QLNVPSSPSPVNRTVSTIMFSRAKRIKLAKRVRQTQLVNMINQINHIKKINKINKINKTD
Sbjct  76   QLNVPSSPSPVNRTVSTIMFSRAKRIKLAKRVRQTQLVNMINQINHIKKINKINKINKTD  135

Query  81   KTKKIKNIKKVESYRMTSKPVFVLPSVKLFRSSSRAAWASPTTKSSLGKRIRHHSADPSI  140
            KTKKIKNIKKVESYRMTSKPVFVLPSVKLFRSSSRAAWASPTTKSSLGKRIRHHSADPSI
Sbjct  136  KTKKIKNIKKVESYRMTSKPVFVLPSVKLFRSSSRAAWASPTTKSSLGKRIRHHSADPSI  195

Query  141  KLCDEPARSNKKPKQWAENSLDLEGSPSVPAQRPQSVNIPSWIEARRNEHQEQSDSTPSD  200
            KLCDEPARSNKKPKQWAENSLDLEGSPSVPAQRPQSVNIPSWIEARRNEHQEQSDSTPSD
Sbjct  196  KLCDEPARSNKKPKQWAENSLDLEGSPSVPAQRPQSVNIPSWIEARRNEHQEQSDSTPSD  255

Query  201  ILGRAT  206
            ILGRAT
Sbjct  256  ILGRAT  261


>ref|XP_011981174.1| PREDICTED: aftiphilin isoform X2 [Ovis aries musimon]
Length=907

 Score = 42.7 bits (99),  Expect = 0.16, Method: Compositional matrix adjust.
 Identities = 38/155 (25%), Positives = 73/155 (47%), Gaps = 17/155 (11%)

Query  17   THNTQLNVPSSPSPVNRTVSTIMFSRAKRIKLAKRVRQTQLVNMINQINHIKKINKINKI  76
            TH+T+ N+  +PSP         FSR +RI+L     +     + ++   I++ NKIN++
Sbjct  214  THSTEYNLDYAPSPAEEFADFATFSRKERIQL----EEIGCAVLNDEALTIQENNKINRV  269

Query  77   NKTDKTKKIKNIKKVESYRMTSKPVFVLP---SVKLFRSSSRAAWASPTTKSS--LGKRI  131
            N+ +  K++   +  +    T++   V     S+   R  SR     PT +       R+
Sbjct  270  NELNSVKEVSLDRSFDDKGDTARKDQVCVSEISIASDRGFSRGKQGLPTLQQDEFFNSRV  329

Query  132  RHHS---ADPSIKLCDEPARSNKKPKQWAENSLDL  163
            +  +   ADP+     E + ++++ +  AE +LDL
Sbjct  330  QSEAWSLADPA-----EKSEASRREQCEAEENLDL  359


>ref|XP_011981170.1| PREDICTED: aftiphilin isoform X1 [Ovis aries musimon]
 ref|XP_011981171.1| PREDICTED: aftiphilin isoform X1 [Ovis aries musimon]
 ref|XP_011981173.1| PREDICTED: aftiphilin isoform X1 [Ovis aries musimon]
Length=935

 Score = 42.7 bits (99),  Expect = 0.17, Method: Compositional matrix adjust.
 Identities = 38/155 (25%), Positives = 73/155 (47%), Gaps = 17/155 (11%)

Query  17   THNTQLNVPSSPSPVNRTVSTIMFSRAKRIKLAKRVRQTQLVNMINQINHIKKINKINKI  76
            TH+T+ N+  +PSP         FSR +RI+L     +     + ++   I++ NKIN++
Sbjct  214  THSTEYNLDYAPSPAEEFADFATFSRKERIQL----EEIGCAVLNDEALTIQENNKINRV  269

Query  77   NKTDKTKKIKNIKKVESYRMTSKPVFVLP---SVKLFRSSSRAAWASPTTKSS--LGKRI  131
            N+ +  K++   +  +    T++   V     S+   R  SR     PT +       R+
Sbjct  270  NELNSVKEVSLDRSFDDKGDTARKDQVCVSEISIASDRGFSRGKQGLPTLQQDEFFNSRV  329

Query  132  RHHS---ADPSIKLCDEPARSNKKPKQWAENSLDL  163
            +  +   ADP+     E + ++++ +  AE +LDL
Sbjct  330  QSEAWSLADPA-----EKSEASRREQCEAEENLDL  359


>ref|XP_005686857.1| PREDICTED: aftiphilin isoform X2 [Capra hircus]
Length=907

 Score = 42.0 bits (97),  Expect = 0.36, Method: Compositional matrix adjust.
 Identities = 38/155 (25%), Positives = 73/155 (47%), Gaps = 17/155 (11%)

Query  17   THNTQLNVPSSPSPVNRTVSTIMFSRAKRIKLAKRVRQTQLVNMINQINHIKKINKINKI  76
            TH+T+ N+  +PSP         FSR +RI+L     +     + ++   I++ NKIN++
Sbjct  214  THSTEYNLDYAPSPAEEFADFATFSRKERIQL----EEIGCAVLNDEALTIQENNKINRV  269

Query  77   NKTDKTKKIKNIKKVESYRMTSKPVFVLP---SVKLFRSSSRAAWASPTTKSS--LGKRI  131
            N+ +  K++   +  +    T++   V     S+   R  SR     PT +       R+
Sbjct  270  NELNSIKEVSLDRSFDDKGDTARKDQVCVSEISIASDRGFSRGKQGLPTLQQDEFFNSRV  329

Query  132  RHHS---ADPSIKLCDEPARSNKKPKQWAENSLDL  163
            +  +   ADP+     E + ++++ +  AE +LDL
Sbjct  330  QSEAWSLADPA-----ENSEASRREQCKAEENLDL  359


>ref|XP_005686856.1| PREDICTED: aftiphilin isoform X1 [Capra hircus]
Length=935

 Score = 41.6 bits (96),  Expect = 0.39, Method: Compositional matrix adjust.
 Identities = 38/155 (25%), Positives = 73/155 (47%), Gaps = 17/155 (11%)

Query  17   THNTQLNVPSSPSPVNRTVSTIMFSRAKRIKLAKRVRQTQLVNMINQINHIKKINKINKI  76
            TH+T+ N+  +PSP         FSR +RI+L     +     + ++   I++ NKIN++
Sbjct  214  THSTEYNLDYAPSPAEEFADFATFSRKERIQL----EEIGCAVLNDEALTIQENNKINRV  269

Query  77   NKTDKTKKIKNIKKVESYRMTSKPVFVLP---SVKLFRSSSRAAWASPTTKSS--LGKRI  131
            N+ +  K++   +  +    T++   V     S+   R  SR     PT +       R+
Sbjct  270  NELNSIKEVSLDRSFDDKGDTARKDQVCVSEISIASDRGFSRGKQGLPTLQQDEFFNSRV  329

Query  132  RHHS---ADPSIKLCDEPARSNKKPKQWAENSLDL  163
            +  +   ADP+     E + ++++ +  AE +LDL
Sbjct  330  QSEAWSLADPA-----ENSEASRREQCKAEENLDL  359


>ref|XP_005963508.1| PREDICTED: aftiphilin isoform X1 [Pantholops hodgsonii]
Length=935

 Score = 41.2 bits (95),  Expect = 0.50, Method: Compositional matrix adjust.
 Identities = 38/155 (25%), Positives = 72/155 (46%), Gaps = 17/155 (11%)

Query  17   THNTQLNVPSSPSPVNRTVSTIMFSRAKRIKLAKRVRQTQLVNMINQINHIKKINKINKI  76
            TH+T+ N+  +PSP         FSR +RI+L     +     + ++   I++ NKIN++
Sbjct  214  THSTEYNLDYAPSPAEEFADFATFSRKERIQL----EEIGCAVLNDEALTIQENNKINRV  269

Query  77   NKTDKTKKIKNIKKVESYRMTSKPVFVLP---SVKLFRSSSRAAWASPTTKSS--LGKRI  131
            N+ +  K++   +  +    T++   V     S+   R  SR     PT +       R+
Sbjct  270  NELNSIKEVSLDRSFDDKGDTARKDQVCVSEISIASDRGFSRGKQGLPTLQQDEFFNSRV  329

Query  132  RHHS---ADPSIKLCDEPARSNKKPKQWAENSLDL  163
            +  +   ADP+     E + ++++ +  AE  LDL
Sbjct  330  QSEAWSLADPA-----ENSEASRREQCKAEEHLDL  359


>ref|XP_012332656.1| PREDICTED: aftiphilin isoform X2 [Aotus nancymaae]
Length=907

 Score = 41.2 bits (95),  Expect = 0.50, Method: Compositional matrix adjust.
 Identities = 22/69 (32%), Positives = 37/69 (54%), Gaps = 13/69 (19%)

Query  15   PL-THNTQLNVPSSPSPVNRTVSTIMFSRAKRIKL------------AKRVRQTQLVNMI  61
            PL TH+T+ N+ S PSP +       FSR +RI+L            A  +R+   +N +
Sbjct  211  PLSTHSTECNLDSVPSPADEFADFATFSRKERIQLEEIGCAVLNDREALTIRENNKINRV  270

Query  62   NQINHIKKI  70
            N++N +K++
Sbjct  271  NELNSVKEV  279


>ref|XP_012332654.1| PREDICTED: aftiphilin isoform X1 [Aotus nancymaae]
 ref|XP_012332655.1| PREDICTED: aftiphilin isoform X1 [Aotus nancymaae]
Length=935

 Score = 41.2 bits (95),  Expect = 0.52, Method: Compositional matrix adjust.
 Identities = 22/69 (32%), Positives = 37/69 (54%), Gaps = 13/69 (19%)

Query  15   PL-THNTQLNVPSSPSPVNRTVSTIMFSRAKRIKL------------AKRVRQTQLVNMI  61
            PL TH+T+ N+ S PSP +       FSR +RI+L            A  +R+   +N +
Sbjct  211  PLSTHSTECNLDSVPSPADEFADFATFSRKERIQLEEIGCAVLNDREALTIRENNKINRV  270

Query  62   NQINHIKKI  70
            N++N +K++
Sbjct  271  NELNSVKEV  279


>ref|XP_005963509.1| PREDICTED: aftiphilin isoform X2 [Pantholops hodgsonii]
Length=907

 Score = 41.2 bits (95),  Expect = 0.54, Method: Compositional matrix adjust.
 Identities = 38/155 (25%), Positives = 72/155 (46%), Gaps = 17/155 (11%)

Query  17   THNTQLNVPSSPSPVNRTVSTIMFSRAKRIKLAKRVRQTQLVNMINQINHIKKINKINKI  76
            TH+T+ N+  +PSP         FSR +RI+L     +     + ++   I++ NKIN++
Sbjct  214  THSTEYNLDYAPSPAEEFADFATFSRKERIQL----EEIGCAVLNDEALTIQENNKINRV  269

Query  77   NKTDKTKKIKNIKKVESYRMTSKPVFVLP---SVKLFRSSSRAAWASPTTKSS--LGKRI  131
            N+ +  K++   +  +    T++   V     S+   R  SR     PT +       R+
Sbjct  270  NELNSIKEVSLDRSFDDKGDTARKDQVCVSEISIASDRGFSRGKQGLPTLQQDEFFNSRV  329

Query  132  RHHS---ADPSIKLCDEPARSNKKPKQWAENSLDL  163
            +  +   ADP+     E + ++++ +  AE  LDL
Sbjct  330  QSEAWSLADPA-----ENSEASRREQCKAEEHLDL  359


>ref|XP_005963510.1| PREDICTED: aftiphilin isoform X3 [Pantholops hodgsonii]
Length=908

 Score = 41.2 bits (95),  Expect = 0.56, Method: Compositional matrix adjust.
 Identities = 38/155 (25%), Positives = 72/155 (46%), Gaps = 17/155 (11%)

Query  17   THNTQLNVPSSPSPVNRTVSTIMFSRAKRIKLAKRVRQTQLVNMINQINHIKKINKINKI  76
            TH+T+ N+  +PSP         FSR +RI+L     +     + ++   I++ NKIN++
Sbjct  214  THSTEYNLDYAPSPAEEFADFATFSRKERIQL----EEIGCAVLNDEALTIQENNKINRV  269

Query  77   NKTDKTKKIKNIKKVESYRMTSKPVFVLP---SVKLFRSSSRAAWASPTTKSS--LGKRI  131
            N+ +  K++   +  +    T++   V     S+   R  SR     PT +       R+
Sbjct  270  NELNSIKEVSLDRSFDDKGDTARKDQVCVSEISIASDRGFSRGKQGLPTLQQDEFFNSRV  329

Query  132  RHHS---ADPSIKLCDEPARSNKKPKQWAENSLDL  163
            +  +   ADP+     E + ++++ +  AE  LDL
Sbjct  330  QSEAWSLADPA-----ENSEASRREQCKAEEHLDL  359


>ref|XP_004005887.1| PREDICTED: aftiphilin isoform X1 [Ovis aries]
 ref|XP_012023733.1| PREDICTED: aftiphilin isoform X1 [Ovis aries]
 ref|XP_012023742.1| PREDICTED: aftiphilin isoform X1 [Ovis aries]
Length=935

 Score = 40.8 bits (94),  Expect = 0.69, Method: Compositional matrix adjust.
 Identities = 37/155 (24%), Positives = 72/155 (46%), Gaps = 17/155 (11%)

Query  17   THNTQLNVPSSPSPVNRTVSTIMFSRAKRIKLAKRVRQTQLVNMINQINHIKKINKINKI  76
            TH+ + N+  +PSP         FSR +RI+L     +     + ++   I++ NKIN++
Sbjct  214  THSAEYNLDYAPSPAEEFADFATFSRKERIQL----EEIGCAVLNDEALTIQENNKINRV  269

Query  77   NKTDKTKKIKNIKKVESYRMTSKPVFVLP---SVKLFRSSSRAAWASPTTKSS--LGKRI  131
            N+ +  K++   +  +    T++   V     S+   R  SR     PT +       R+
Sbjct  270  NELNSVKEVSLDRSFDDKGDTARKDQVCVSEISIASDRGFSRGKQGLPTLQQDEFFNSRV  329

Query  132  RHHS---ADPSIKLCDEPARSNKKPKQWAENSLDL  163
            +  +   ADP+     E + ++++ +  AE +LDL
Sbjct  330  QSEAWSLADPA-----EKSEASRREQCEAEENLDL  359


>ref|XP_004005886.1| PREDICTED: aftiphilin isoform X2 [Ovis aries]
Length=907

 Score = 40.8 bits (94),  Expect = 0.69, Method: Compositional matrix adjust.
 Identities = 37/155 (24%), Positives = 72/155 (46%), Gaps = 17/155 (11%)

Query  17   THNTQLNVPSSPSPVNRTVSTIMFSRAKRIKLAKRVRQTQLVNMINQINHIKKINKINKI  76
            TH+ + N+  +PSP         FSR +RI+L     +     + ++   I++ NKIN++
Sbjct  214  THSAEYNLDYAPSPAEEFADFATFSRKERIQL----EEIGCAVLNDEALTIQENNKINRV  269

Query  77   NKTDKTKKIKNIKKVESYRMTSKPVFVLP---SVKLFRSSSRAAWASPTTKSS--LGKRI  131
            N+ +  K++   +  +    T++   V     S+   R  SR     PT +       R+
Sbjct  270  NELNSVKEVSLDRSFDDKGDTARKDQVCVSEISIASDRGFSRGKQGLPTLQQDEFFNSRV  329

Query  132  RHHS---ADPSIKLCDEPARSNKKPKQWAENSLDL  163
            +  +   ADP+     E + ++++ +  AE +LDL
Sbjct  330  QSEAWSLADPA-----EKSEASRREQCEAEENLDL  359


>ref|XP_004029369.1| PREDICTED: aftiphilin isoform 1 [Gorilla gorilla gorilla]
Length=910

 Score = 40.4 bits (93),  Expect = 1.0, Method: Compositional matrix adjust.
 Identities = 22/69 (32%), Positives = 41/69 (59%), Gaps = 3/69 (4%)

Query  17   THNTQLNVPSSPSPVNRTVSTIMFSRAKRIKLAKRVRQTQLVNMINQINHIKKINKINKI  76
            TH+T+ N+ S PSP         FS+ +RI+L +   +  ++N    +N I++ NKIN++
Sbjct  214  THSTEYNLDSVPSPAEEFADFATFSKKERIQLEE--IECAVLNDREALN-IRENNKINRV  270

Query  77   NKTDKTKKI  85
            N+ +  K++
Sbjct  271  NELNSVKEV  279


>ref|XP_004029370.1| PREDICTED: aftiphilin isoform 2 [Gorilla gorilla gorilla]
Length=937

 Score = 40.4 bits (93),  Expect = 1.1, Method: Compositional matrix adjust.
 Identities = 22/69 (32%), Positives = 41/69 (59%), Gaps = 3/69 (4%)

Query  17   THNTQLNVPSSPSPVNRTVSTIMFSRAKRIKLAKRVRQTQLVNMINQINHIKKINKINKI  76
            TH+T+ N+ S PSP         FS+ +RI+L +   +  ++N    +N I++ NKIN++
Sbjct  214  THSTEYNLDSVPSPAEEFADFATFSKKERIQLEE--IECAVLNDREALN-IRENNKINRV  270

Query  77   NKTDKTKKI  85
            N+ +  K++
Sbjct  271  NELNSVKEV  279


>ref|XP_007189803.1| PREDICTED: aftiphilin isoform X5 [Balaenoptera acutorostrata 
scammoni]
Length=806

 Score = 40.4 bits (93),  Expect = 1.1, Method: Compositional matrix adjust.
 Identities = 26/101 (26%), Positives = 47/101 (47%), Gaps = 14/101 (14%)

Query  15   PL-THNTQLNVPSSPSPVNRTVSTIMFSRAKRIKL------------AKRVRQTQLVNMI  61
            PL TH+T+ N+ S+PSP         FS+ +RI+L            A  +++   +N +
Sbjct  212  PLSTHSTEYNLDSAPSPAEEFADFATFSKKERIQLEEIGCAVLNDREAVTIQENNKINRV  271

Query  62   NQINHIKKINKINKINKTDKTKKIKNIKKVESYRMTSKPVF  102
            N++N +K+++    I+  + T     +  V    M S   F
Sbjct  272  NELNSVKEVSLDRSIDDKEDTAGEDQV-CVSEISMVSNGAF  311


>ref|XP_007464175.1| PREDICTED: aftiphilin isoform X2 [Lipotes vexillifer]
Length=907

 Score = 40.4 bits (93),  Expect = 1.2, Method: Compositional matrix adjust.
 Identities = 29/102 (28%), Positives = 49/102 (48%), Gaps = 16/102 (16%)

Query  15   PL-THNTQLNVPSSPSPVNRTVSTIMFSRAKRIKL------------AKRVRQTQLVNMI  61
            PL TH+T+ N+ S+PSP         FS+ +RI+L            A  +++   +N +
Sbjct  211  PLSTHSTEYNLDSAPSPAEEFADFATFSKKERIQLEEIGCAVLNDREALTIQENNKINRV  270

Query  62   NQINHIKKINKINKI-NKTDKTKKIKNIKKVESYRMTSKPVF  102
            N++N +K+++    I NK D   + +    V    M S  VF
Sbjct  271  NELNSVKEVSLDRSIDNKGDSAGEDQVC--VSEISMVSNGVF  310


>ref|XP_007464174.1| PREDICTED: aftiphilin isoform X1 [Lipotes vexillifer]
Length=935

 Score = 40.0 bits (92),  Expect = 1.2, Method: Compositional matrix adjust.
 Identities = 29/102 (28%), Positives = 49/102 (48%), Gaps = 16/102 (16%)

Query  15   PL-THNTQLNVPSSPSPVNRTVSTIMFSRAKRIKL------------AKRVRQTQLVNMI  61
            PL TH+T+ N+ S+PSP         FS+ +RI+L            A  +++   +N +
Sbjct  211  PLSTHSTEYNLDSAPSPAEEFADFATFSKKERIQLEEIGCAVLNDREALTIQENNKINRV  270

Query  62   NQINHIKKINKINKI-NKTDKTKKIKNIKKVESYRMTSKPVF  102
            N++N +K+++    I NK D   + +    V    M S  VF
Sbjct  271  NELNSVKEVSLDRSIDNKGDSAGEDQVC--VSEISMVSNGVF  310


>ref|XP_008252563.1| PREDICTED: aftiphilin isoform X3 [Oryctolagus cuniculus]
Length=817

 Score = 40.0 bits (92),  Expect = 1.3, Method: Compositional matrix adjust.
 Identities = 26/104 (25%), Positives = 48/104 (46%), Gaps = 13/104 (13%)

Query  15   PL-THNTQLNVPSSPSPVNRTVSTIMFSRAKRIKL------------AKRVRQTQLVNMI  61
            PL TH+T+ N+ S+PSP         FS+ +RI+L            A  +++   +N +
Sbjct  204  PLSTHSTEYNIDSAPSPAEEFADFATFSKQERIQLEEIGYAVLSDREALTIQENNKINRV  263

Query  62   NQINHIKKINKINKINKTDKTKKIKNIKKVESYRMTSKPVFVLP  105
            +++N +K+++     +    T     +   E   MTS+   V P
Sbjct  264  SELNSVKEVSLSRSCDNKGDTDGDDQVCVSEISVMTSRGFSVEP  307


>ref|XP_007189802.1| PREDICTED: aftiphilin isoform X4 [Balaenoptera acutorostrata 
scammoni]
Length=907

 Score = 40.0 bits (92),  Expect = 1.4, Method: Compositional matrix adjust.
 Identities = 26/101 (26%), Positives = 47/101 (47%), Gaps = 14/101 (14%)

Query  15   PL-THNTQLNVPSSPSPVNRTVSTIMFSRAKRIKL------------AKRVRQTQLVNMI  61
            PL TH+T+ N+ S+PSP         FS+ +RI+L            A  +++   +N +
Sbjct  212  PLSTHSTEYNLDSAPSPAEEFADFATFSKKERIQLEEIGCAVLNDREAVTIQENNKINRV  271

Query  62   NQINHIKKINKINKINKTDKTKKIKNIKKVESYRMTSKPVF  102
            N++N +K+++    I+  + T     +  V    M S   F
Sbjct  272  NELNSVKEVSLDRSIDDKEDTAGEDQV-CVSEISMVSNGAF  311


>ref|XP_007189799.1| PREDICTED: aftiphilin isoform X1 [Balaenoptera acutorostrata 
scammoni]
 ref|XP_007189800.1| PREDICTED: aftiphilin isoform X2 [Balaenoptera acutorostrata 
scammoni]
 ref|XP_007189801.1| PREDICTED: aftiphilin isoform X3 [Balaenoptera acutorostrata 
scammoni]
Length=935

 Score = 40.0 bits (92),  Expect = 1.5, Method: Compositional matrix adjust.
 Identities = 26/101 (26%), Positives = 47/101 (47%), Gaps = 14/101 (14%)

Query  15   PL-THNTQLNVPSSPSPVNRTVSTIMFSRAKRIKL------------AKRVRQTQLVNMI  61
            PL TH+T+ N+ S+PSP         FS+ +RI+L            A  +++   +N +
Sbjct  212  PLSTHSTEYNLDSAPSPAEEFADFATFSKKERIQLEEIGCAVLNDREAVTIQENNKINRV  271

Query  62   NQINHIKKINKINKINKTDKTKKIKNIKKVESYRMTSKPVF  102
            N++N +K+++    I+  + T     +  V    M S   F
Sbjct  272  NELNSVKEVSLDRSIDDKEDTAGEDQV-CVSEISMVSNGAF  311


>ref|XP_008252562.1| PREDICTED: aftiphilin isoform X2 [Oryctolagus cuniculus]
Length=898

 Score = 40.0 bits (92),  Expect = 1.5, Method: Compositional matrix adjust.
 Identities = 26/104 (25%), Positives = 48/104 (46%), Gaps = 13/104 (13%)

Query  15   PL-THNTQLNVPSSPSPVNRTVSTIMFSRAKRIKL------------AKRVRQTQLVNMI  61
            PL TH+T+ N+ S+PSP         FS+ +RI+L            A  +++   +N +
Sbjct  204  PLSTHSTEYNIDSAPSPAEEFADFATFSKQERIQLEEIGYAVLSDREALTIQENNKINRV  263

Query  62   NQINHIKKINKINKINKTDKTKKIKNIKKVESYRMTSKPVFVLP  105
            +++N +K+++     +    T     +   E   MTS+   V P
Sbjct  264  SELNSVKEVSLSRSCDNKGDTDGDDQVCVSEISVMTSRGFSVEP  307


>ref|XP_002709726.1| PREDICTED: aftiphilin isoform X1 [Oryctolagus cuniculus]
 ref|XP_008252559.1| PREDICTED: aftiphilin isoform X1 [Oryctolagus cuniculus]
 ref|XP_008252560.1| PREDICTED: aftiphilin isoform X1 [Oryctolagus cuniculus]
 ref|XP_008252561.1| PREDICTED: aftiphilin isoform X1 [Oryctolagus cuniculus]
Length=926

 Score = 39.7 bits (91),  Expect = 1.6, Method: Compositional matrix adjust.
 Identities = 26/104 (25%), Positives = 48/104 (46%), Gaps = 13/104 (13%)

Query  15   PL-THNTQLNVPSSPSPVNRTVSTIMFSRAKRIKL------------AKRVRQTQLVNMI  61
            PL TH+T+ N+ S+PSP         FS+ +RI+L            A  +++   +N +
Sbjct  204  PLSTHSTEYNIDSAPSPAEEFADFATFSKQERIQLEEIGYAVLSDREALTIQENNKINRV  263

Query  62   NQINHIKKINKINKINKTDKTKKIKNIKKVESYRMTSKPVFVLP  105
            +++N +K+++     +    T     +   E   MTS+   V P
Sbjct  264  SELNSVKEVSLSRSCDNKGDTDGDDQVCVSEISVMTSRGFSVEP  307


>dbj|BAE45715.1| putative protein product of Nbla10388 [Homo sapiens]
Length=581

 Score = 39.7 bits (91),  Expect = 1.8, Method: Compositional matrix adjust.
 Identities = 21/69 (30%), Positives = 36/69 (52%), Gaps = 13/69 (19%)

Query  15   PL-THNTQLNVPSSPSPVNRTVSTIMFSRAKRIKL------------AKRVRQTQLVNMI  61
            PL TH+T+ N+ S PSP         FS+ +RI+L            A  +R+   +N +
Sbjct  138  PLSTHSTEYNLDSVPSPAEEFADFATFSKKERIQLEEIECAVLNDREALTIRENNKINRV  197

Query  62   NQINHIKKI  70
            N++N +K++
Sbjct  198  NELNSVKEV  206


>ref|XP_005322125.1| PREDICTED: aftiphilin isoform X3 [Ictidomys tridecemlineatus]
Length=932

 Score = 39.7 bits (91),  Expect = 1.8, Method: Compositional matrix adjust.
 Identities = 22/76 (29%), Positives = 45/76 (59%), Gaps = 3/76 (4%)

Query  17   THNTQLNVPSSPSPVNRTVSTIMFSRAKRIKLAKRVRQTQLVNMINQINHIKKINKINKI  76
            TH+T+ N+  +PSP         FS+ +RI+L + +R   ++N  + +  I++ NKIN++
Sbjct  214  THSTEYNLDFAPSPAEEFADFATFSQKERIQL-EEIR-CAVLNERDALT-IQENNKINRV  270

Query  77   NKTDKTKKIKNIKKVE  92
            N+ +  K++  +K  +
Sbjct  271  NELNSVKEVSLVKSFD  286


>ref|XP_013212663.1| PREDICTED: aftiphilin isoform X1 [Ictidomys tridecemlineatus]
 ref|XP_013212664.1| PREDICTED: aftiphilin isoform X1 [Ictidomys tridecemlineatus]
Length=904

 Score = 39.7 bits (91),  Expect = 1.8, Method: Compositional matrix adjust.
 Identities = 24/79 (30%), Positives = 47/79 (59%), Gaps = 4/79 (5%)

Query  15   PL-THNTQLNVPSSPSPVNRTVSTIMFSRAKRIKLAKRVRQTQLVNMINQINHIKKINKI  73
            PL TH+T+ N+  +PSP         FS+ +RI+L + +R   ++N  + +  I++ NKI
Sbjct  211  PLGTHSTEYNLDFAPSPAEEFADFATFSQKERIQL-EEIR-CAVLNERDALT-IQENNKI  267

Query  74   NKINKTDKTKKIKNIKKVE  92
            N++N+ +  K++  +K  +
Sbjct  268  NRVNELNSVKEVSLVKSFD  286


>ref|XP_013212665.1| PREDICTED: aftiphilin isoform X2 [Ictidomys tridecemlineatus]
Length=803

 Score = 39.7 bits (91),  Expect = 1.9, Method: Compositional matrix adjust.
 Identities = 24/79 (30%), Positives = 47/79 (59%), Gaps = 4/79 (5%)

Query  15   PL-THNTQLNVPSSPSPVNRTVSTIMFSRAKRIKLAKRVRQTQLVNMINQINHIKKINKI  73
            PL TH+T+ N+  +PSP         FS+ +RI+L + +R   ++N  + +  I++ NKI
Sbjct  211  PLGTHSTEYNLDFAPSPAEEFADFATFSQKERIQL-EEIR-CAVLNERDALT-IQENNKI  267

Query  74   NKINKTDKTKKIKNIKKVE  92
            N++N+ +  K++  +K  +
Sbjct  268  NRVNELNSVKEVSLVKSFD  286


>ref|XP_013005489.1| PREDICTED: aftiphilin [Cavia porcellus]
Length=863

 Score = 39.7 bits (91),  Expect = 1.9, Method: Compositional matrix adjust.
 Identities = 22/69 (32%), Positives = 41/69 (59%), Gaps = 3/69 (4%)

Query  17   THNTQLNVPSSPSPVNRTVSTIMFSRAKRIKLAKRVRQTQLVNMINQINHIKKINKINKI  76
            TH+T+ N+ S PSP         FS+ +RI+L +   +  ++N  N I  +++ NKIN++
Sbjct  214  THSTEYNLDSVPSPAEEFADFATFSKKERIQLEE--IECAVLNNSN-ILTVQENNKINRV  270

Query  77   NKTDKTKKI  85
            N+ +  K++
Sbjct  271  NEVNSIKEV  279


>ref|XP_005575804.1| PREDICTED: aftiphilin isoform X3 [Macaca fascicularis]
Length=806

 Score = 39.3 bits (90),  Expect = 2.5, Method: Compositional matrix adjust.
 Identities = 21/69 (30%), Positives = 36/69 (52%), Gaps = 13/69 (19%)

Query  15   PL-THNTQLNVPSSPSPVNRTVSTIMFSRAKRIKL------------AKRVRQTQLVNMI  61
            PL TH+T+ N+ S PSP         FS+ +RI+L            A  +R+   +N +
Sbjct  211  PLSTHSTEYNLDSVPSPAEEFADFATFSKKERIQLEEIECAILNNREALTIRENNKINRV  270

Query  62   NQINHIKKI  70
            N++N +K++
Sbjct  271  NELNSVKEV  279


>ref|XP_011894996.1| PREDICTED: aftiphilin isoform X3 [Cercocebus atys]
Length=806

 Score = 39.3 bits (90),  Expect = 2.5, Method: Compositional matrix adjust.
 Identities = 21/69 (30%), Positives = 36/69 (52%), Gaps = 13/69 (19%)

Query  15   PL-THNTQLNVPSSPSPVNRTVSTIMFSRAKRIKL------------AKRVRQTQLVNMI  61
            PL TH+T+ N+ S PSP         FS+ +RI+L            A  +R+   +N +
Sbjct  211  PLSTHSTEYNLDSVPSPAEEFADFATFSKKERIQLEEIECAILNNREALTIRENNKINRV  270

Query  62   NQINHIKKI  70
            N++N +K++
Sbjct  271  NELNSVKEV  279


>ref|XP_003262504.1| PREDICTED: aftiphilin [Nomascus leucogenys]
Length=935

 Score = 39.3 bits (90),  Expect = 2.6, Method: Compositional matrix adjust.
 Identities = 21/85 (25%), Positives = 42/85 (49%), Gaps = 15/85 (18%)

Query  17   THNTQLNVPSSPSPVNRTVSTIMFSRAKRIKL------------AKRVRQTQLVNMINQI  64
            TH+T+ N+ S PSP         FS+ +RI+L            A  +R+   +N +N++
Sbjct  214  THSTEYNLDSVPSPAEEFADFATFSKKERIQLEEIECAVLNDREALTIRENNKINRVNEL  273

Query  65   NHIKKIN---KINKINKTDKTKKIK  86
            N +K+++    ++    TD   +++
Sbjct  274  NSVKEVSLGRSLDNKGDTDGEDQVR  298


>emb|CAE46209.1| hypothetical protein [Homo sapiens]
Length=820

 Score = 39.3 bits (90),  Expect = 2.6, Method: Compositional matrix adjust.
 Identities = 21/69 (30%), Positives = 36/69 (52%), Gaps = 13/69 (19%)

Query  15   PL-THNTQLNVPSSPSPVNRTVSTIMFSRAKRIKL------------AKRVRQTQLVNMI  61
            PL TH+T+ N+ S PSP         FS+ +RI+L            A  +R+   +N +
Sbjct  211  PLSTHSTEYNLDSVPSPAEEFADFATFSKKERIQLEEIECAVLNDREALTIRENNKINRV  270

Query  62   NQINHIKKI  70
            N++N +K++
Sbjct  271  NELNSVKEV  279


>gb|EAW99940.1| aftiphilin protein, isoform CRA_d [Homo sapiens]
Length=844

 Score = 39.3 bits (90),  Expect = 2.7, Method: Compositional matrix adjust.
 Identities = 21/69 (30%), Positives = 36/69 (52%), Gaps = 13/69 (19%)

Query  15   PL-THNTQLNVPSSPSPVNRTVSTIMFSRAKRIKL------------AKRVRQTQLVNMI  61
            PL TH+T+ N+ S PSP         FS+ +RI+L            A  +R+   +N +
Sbjct  211  PLSTHSTEYNLDSVPSPAEEFADFATFSKKERIQLEEIECAVLNDREALTIRENNKINRV  270

Query  62   NQINHIKKI  70
            N++N +K++
Sbjct  271  NELNSVKEV  279


>ref|NP_001244656.1| aftiphilin [Macaca mulatta]
 ref|XP_005575803.1| PREDICTED: aftiphilin isoform X2 [Macaca fascicularis]
 ref|XP_011711714.1| PREDICTED: aftiphilin isoform X2 [Macaca nemestrina]
Length=907

 Score = 39.3 bits (90),  Expect = 2.7, Method: Compositional matrix adjust.
 Identities = 21/69 (30%), Positives = 36/69 (52%), Gaps = 13/69 (19%)

Query  15   PL-THNTQLNVPSSPSPVNRTVSTIMFSRAKRIKL------------AKRVRQTQLVNMI  61
            PL TH+T+ N+ S PSP         FS+ +RI+L            A  +R+   +N +
Sbjct  211  PLSTHSTEYNLDSVPSPAEEFADFATFSKKERIQLEEIECAILNNREALTIRENNKINRV  270

Query  62   NQINHIKKI  70
            N++N +K++
Sbjct  271  NELNSVKEV  279


>ref|XP_011894995.1| PREDICTED: aftiphilin isoform X2 [Cercocebus atys]
Length=907

 Score = 39.3 bits (90),  Expect = 2.7, Method: Compositional matrix adjust.
 Identities = 21/69 (30%), Positives = 36/69 (52%), Gaps = 13/69 (19%)

Query  15   PL-THNTQLNVPSSPSPVNRTVSTIMFSRAKRIKL------------AKRVRQTQLVNMI  61
            PL TH+T+ N+ S PSP         FS+ +RI+L            A  +R+   +N +
Sbjct  211  PLSTHSTEYNLDSVPSPAEEFADFATFSKKERIQLEEIECAILNNREALTIRENNKINRV  270

Query  62   NQINHIKKI  70
            N++N +K++
Sbjct  271  NELNSVKEV  279


>ref|XP_011894993.1| PREDICTED: aftiphilin isoform X1 [Cercocebus atys]
 ref|XP_011894994.1| PREDICTED: aftiphilin isoform X1 [Cercocebus atys]
Length=935

 Score = 39.3 bits (90),  Expect = 2.7, Method: Compositional matrix adjust.
 Identities = 19/66 (29%), Positives = 34/66 (52%), Gaps = 12/66 (18%)

Query  17   THNTQLNVPSSPSPVNRTVSTIMFSRAKRIKL------------AKRVRQTQLVNMINQI  64
            TH+T+ N+ S PSP         FS+ +RI+L            A  +R+   +N +N++
Sbjct  214  THSTEYNLDSVPSPAEEFADFATFSKKERIQLEEIECAILNNREALTIRENNKINRVNEL  273

Query  65   NHIKKI  70
            N +K++
Sbjct  274  NSVKEV  279


>ref|XP_011840879.1| PREDICTED: aftiphilin [Mandrillus leucophaeus]
Length=935

 Score = 39.3 bits (90),  Expect = 2.7, Method: Compositional matrix adjust.
 Identities = 19/66 (29%), Positives = 34/66 (52%), Gaps = 12/66 (18%)

Query  17   THNTQLNVPSSPSPVNRTVSTIMFSRAKRIKL------------AKRVRQTQLVNMINQI  64
            TH+T+ N+ S PSP         FS+ +RI+L            A  +R+   +N +N++
Sbjct  214  THSTEYNLDSVPSPAEEFADFATFSKKERIQLEEIECAILNNREALTIRENNKINRVNEL  273

Query  65   NHIKKI  70
            N +K++
Sbjct  274  NSVKEV  279


>ref|XP_005575802.1| PREDICTED: aftiphilin isoform X1 [Macaca fascicularis]
 ref|XP_011711713.1| PREDICTED: aftiphilin isoform X1 [Macaca nemestrina]
Length=935

 Score = 39.3 bits (90),  Expect = 2.7, Method: Compositional matrix adjust.
 Identities = 19/66 (29%), Positives = 34/66 (52%), Gaps = 12/66 (18%)

Query  17   THNTQLNVPSSPSPVNRTVSTIMFSRAKRIKL------------AKRVRQTQLVNMINQI  64
            TH+T+ N+ S PSP         FS+ +RI+L            A  +R+   +N +N++
Sbjct  214  THSTEYNLDSVPSPAEEFADFATFSKKERIQLEEIECAILNNREALTIRENNKINRVNEL  273

Query  65   NHIKKI  70
            N +K++
Sbjct  274  NSVKEV  279


>gb|EHH22164.1| hypothetical protein EGK_05380 [Macaca mulatta]
Length=936

 Score = 39.3 bits (90),  Expect = 2.7, Method: Compositional matrix adjust.
 Identities = 19/66 (29%), Positives = 34/66 (52%), Gaps = 12/66 (18%)

Query  17   THNTQLNVPSSPSPVNRTVSTIMFSRAKRIKL------------AKRVRQTQLVNMINQI  64
            TH+T+ N+ S PSP         FS+ +RI+L            A  +R+   +N +N++
Sbjct  214  THSTEYNLDSVPSPAEEFADFATFSKKERIQLEEIECAILNNREALTIRENNKINRVNEL  273

Query  65   NHIKKI  70
            N +K++
Sbjct  274  NSVKEV  279


>gb|EHH55612.1| hypothetical protein EGM_04853 [Macaca fascicularis]
Length=936

 Score = 39.3 bits (90),  Expect = 2.7, Method: Compositional matrix adjust.
 Identities = 19/66 (29%), Positives = 34/66 (52%), Gaps = 12/66 (18%)

Query  17   THNTQLNVPSSPSPVNRTVSTIMFSRAKRIKL------------AKRVRQTQLVNMINQI  64
            TH+T+ N+ S PSP         FS+ +RI+L            A  +R+   +N +N++
Sbjct  214  THSTEYNLDSVPSPAEEFADFATFSKKERIQLEEIECAILNNREALTIRENNKINRVNEL  273

Query  65   NHIKKI  70
            N +K++
Sbjct  274  NSVKEV  279


>ref|XP_004321595.1| PREDICTED: aftiphilin-like, partial [Tursiops truncatus]
Length=886

 Score = 39.3 bits (90),  Expect = 2.7, Method: Compositional matrix adjust.
 Identities = 20/69 (29%), Positives = 37/69 (54%), Gaps = 13/69 (19%)

Query  15   PL-THNTQLNVPSSPSPVNRTVSTIMFSRAKRIKL------------AKRVRQTQLVNMI  61
            PL TH+T+ N+ S+PSP         FS+ +RI+L            A  +++   +N +
Sbjct  211  PLSTHSTEYNLDSAPSPAEEFADFATFSKKERIQLEEIGCAVLNDREALTIQENNKINRV  270

Query  62   NQINHIKKI  70
            N++N +K++
Sbjct  271  NELNSVKEV  279


>ref|NP_060127.3| aftiphilin isoform b [Homo sapiens]
 dbj|BAA90936.1| unnamed protein product [Homo sapiens]
 gb|EAW99938.1| aftiphilin protein, isoform CRA_b [Homo sapiens]
 gb|EAW99941.1| aftiphilin protein, isoform CRA_b [Homo sapiens]
Length=909

 Score = 39.3 bits (90),  Expect = 2.7, Method: Compositional matrix adjust.
 Identities = 19/66 (29%), Positives = 34/66 (52%), Gaps = 12/66 (18%)

Query  17   THNTQLNVPSSPSPVNRTVSTIMFSRAKRIKL------------AKRVRQTQLVNMINQI  64
            TH+T+ N+ S PSP         FS+ +RI+L            A  +R+   +N +N++
Sbjct  214  THSTEYNLDSVPSPAEEFADFATFSKKERIQLEEIECAVLNDREALTIRENNKINRVNEL  273

Query  65   NHIKKI  70
            N +K++
Sbjct  274  NSVKEV  279


>ref|XP_007968638.1| PREDICTED: aftiphilin isoform X3 [Chlorocebus sabaeus]
Length=806

 Score = 39.3 bits (90),  Expect = 2.8, Method: Compositional matrix adjust.
 Identities = 21/69 (30%), Positives = 36/69 (52%), Gaps = 13/69 (19%)

Query  15   PL-THNTQLNVPSSPSPVNRTVSTIMFSRAKRIKL------------AKRVRQTQLVNMI  61
            PL TH+T+ N+ S PSP         FS+ +RI+L            A  +R+   +N +
Sbjct  211  PLSTHSTEYNLDSVPSPAEEFADFATFSKKERIQLEEIECAILNNREALTIRENNKINRV  270

Query  62   NQINHIKKI  70
            N++N +K++
Sbjct  271  NELNSVKEV  279


>ref|XP_004280676.1| PREDICTED: aftiphilin isoform X1 [Orcinus orca]
Length=934

 Score = 39.3 bits (90),  Expect = 2.8, Method: Compositional matrix adjust.
 Identities = 20/69 (29%), Positives = 37/69 (54%), Gaps = 13/69 (19%)

Query  15   PL-THNTQLNVPSSPSPVNRTVSTIMFSRAKRIKL------------AKRVRQTQLVNMI  61
            PL TH+T+ N+ S+PSP         FS+ +RI+L            A  +++   +N +
Sbjct  211  PLSTHSTEYNLDSAPSPAEEFADFATFSKKERIQLEEIGCAVLNDREALTIQENNKINRV  270

Query  62   NQINHIKKI  70
            N++N +K++
Sbjct  271  NELNSVKEV  279


>ref|XP_003908780.1| PREDICTED: aftiphilin isoform X1 [Papio anubis]
 ref|XP_009182573.1| PREDICTED: aftiphilin isoform X1 [Papio anubis]
Length=935

 Score = 39.3 bits (90),  Expect = 2.8, Method: Compositional matrix adjust.
 Identities = 19/66 (29%), Positives = 34/66 (52%), Gaps = 12/66 (18%)

Query  17   THNTQLNVPSSPSPVNRTVSTIMFSRAKRIKL------------AKRVRQTQLVNMINQI  64
            TH+T+ N+ S PSP         FS+ +RI+L            A  +R+   +N +N++
Sbjct  214  THSTEYNLDSVPSPAEEFADFATFSKKERIQLEEIECAILNNREALTIRENNKINRVNEL  273

Query  65   NHIKKI  70
            N +K++
Sbjct  274  NSVKEV  279


>ref|XP_004280677.1| PREDICTED: aftiphilin isoform X2 [Orcinus orca]
Length=906

 Score = 39.3 bits (90),  Expect = 2.8, Method: Compositional matrix adjust.
 Identities = 20/69 (29%), Positives = 37/69 (54%), Gaps = 13/69 (19%)

Query  15   PL-THNTQLNVPSSPSPVNRTVSTIMFSRAKRIKL------------AKRVRQTQLVNMI  61
            PL TH+T+ N+ S+PSP         FS+ +RI+L            A  +++   +N +
Sbjct  211  PLSTHSTEYNLDSAPSPAEEFADFATFSKKERIQLEEIGCAVLNDREALTIQENNKINRV  270

Query  62   NQINHIKKI  70
            N++N +K++
Sbjct  271  NELNSVKEV  279


>ref|XP_003908779.1| PREDICTED: aftiphilin isoform X2 [Papio anubis]
Length=907

 Score = 39.3 bits (90),  Expect = 2.8, Method: Compositional matrix adjust.
 Identities = 21/69 (30%), Positives = 36/69 (52%), Gaps = 13/69 (19%)

Query  15   PL-THNTQLNVPSSPSPVNRTVSTIMFSRAKRIKL------------AKRVRQTQLVNMI  61
            PL TH+T+ N+ S PSP         FS+ +RI+L            A  +R+   +N +
Sbjct  211  PLSTHSTEYNLDSVPSPAEEFADFATFSKKERIQLEEIECAILNNREALTIRENNKINRV  270

Query  62   NQINHIKKI  70
            N++N +K++
Sbjct  271  NELNSVKEV  279


>ref|NP_001002243.1| aftiphilin isoform c [Homo sapiens]
Length=908

 Score = 39.3 bits (90),  Expect = 2.8, Method: Compositional matrix adjust.
 Identities = 19/66 (29%), Positives = 34/66 (52%), Gaps = 12/66 (18%)

Query  17   THNTQLNVPSSPSPVNRTVSTIMFSRAKRIKL------------AKRVRQTQLVNMINQI  64
            TH+T+ N+ S PSP         FS+ +RI+L            A  +R+   +N +N++
Sbjct  214  THSTEYNLDSVPSPAEEFADFATFSKKERIQLEEIECAVLNDREALTIRENNKINRVNEL  273

Query  65   NHIKKI  70
            N +K++
Sbjct  274  NSVKEV  279


>gb|AAH47529.1| Aftiphilin [Homo sapiens]
Length=909

 Score = 39.3 bits (90),  Expect = 2.8, Method: Compositional matrix adjust.
 Identities = 19/66 (29%), Positives = 34/66 (52%), Gaps = 12/66 (18%)

Query  17   THNTQLNVPSSPSPVNRTVSTIMFSRAKRIKL------------AKRVRQTQLVNMINQI  64
            TH+T+ N+ S PSP         FS+ +RI+L            A  +R+   +N +N++
Sbjct  214  THSTEYNLDSVPSPAEEFADFATFSKKERIQLEEIECAVLNDREALTIRENNKINRVNEL  273

Query  65   NHIKKI  70
            N +K++
Sbjct  274  NSVKEV  279


>ref|XP_007968636.1| PREDICTED: aftiphilin isoform X1 [Chlorocebus sabaeus]
Length=935

 Score = 39.3 bits (90),  Expect = 2.8, Method: Compositional matrix adjust.
 Identities = 19/66 (29%), Positives = 34/66 (52%), Gaps = 12/66 (18%)

Query  17   THNTQLNVPSSPSPVNRTVSTIMFSRAKRIKL------------AKRVRQTQLVNMINQI  64
            TH+T+ N+ S PSP         FS+ +RI+L            A  +R+   +N +N++
Sbjct  214  THSTEYNLDSVPSPAEEFADFATFSKKERIQLEEIECAILNNREALTIRENNKINRVNEL  273

Query  65   NHIKKI  70
            N +K++
Sbjct  274  NSVKEV  279


>ref|XP_009235528.1| PREDICTED: LOW QUALITY PROTEIN: aftiphilin-like, partial [Pongo 
abelii]
Length=705

 Score = 38.9 bits (89),  Expect = 2.8, Method: Compositional matrix adjust.
 Identities = 21/69 (30%), Positives = 36/69 (52%), Gaps = 13/69 (19%)

Query  15   PL-THNTQLNVPSSPSPVNRTVSTIMFSRAKRIKL------------AKRVRQTQLVNMI  61
            PL TH+T+ N+ S PSP         FS+ +RI+L            A  +R+   +N +
Sbjct  107  PLSTHSTEYNLDSVPSPAEEFADFATFSKKERIQLEEIECAVLNDREALTIRENNKINRV  166

Query  62   NQINHIKKI  70
            N++N +K++
Sbjct  167  NELNSVKEV  175


>dbj|BAD96541.1| aftiphilin protein isoform b variant [Homo sapiens]
Length=909

 Score = 39.3 bits (90),  Expect = 2.8, Method: Compositional matrix adjust.
 Identities = 19/66 (29%), Positives = 34/66 (52%), Gaps = 12/66 (18%)

Query  17   THNTQLNVPSSPSPVNRTVSTIMFSRAKRIKL------------AKRVRQTQLVNMINQI  64
            TH+T+ N+ S PSP         FS+ +RI+L            A  +R+   +N +N++
Sbjct  214  THSTEYNLDSVPSPAEEFADFATFSKKERIQLEEIECAVLNDREALTIRENNKINRVNEL  273

Query  65   NHIKKI  70
            N +K++
Sbjct  274  NSVKEV  279


>ref|XP_011800696.1| PREDICTED: aftiphilin [Colobus angolensis palliatus]
Length=935

 Score = 39.3 bits (90),  Expect = 2.8, Method: Compositional matrix adjust.
 Identities = 19/66 (29%), Positives = 34/66 (52%), Gaps = 12/66 (18%)

Query  17   THNTQLNVPSSPSPVNRTVSTIMFSRAKRIKL------------AKRVRQTQLVNMINQI  64
            TH+T+ N+ S PSP         FS+ +RI+L            A  +R+   +N +N++
Sbjct  214  THSTEYNLDSVPSPAEEFADFATFSKKERIQLEEIECAILNNREALTIRENNKINRVNEL  273

Query  65   NHIKKI  70
            N +K++
Sbjct  274  NSVKEV  279


>ref|XP_005264437.1| PREDICTED: aftiphilin isoform X1 [Homo sapiens]
 sp|Q6ULP2|AFTIN_HUMAN RecName: Full=Aftiphilin
 gb|EAW99937.1| aftiphilin protein, isoform CRA_a [Homo sapiens]
 dbj|BAI45975.1| aftiphilin [synthetic construct]
Length=937

 Score = 39.3 bits (90),  Expect = 2.8, Method: Compositional matrix adjust.
 Identities = 19/66 (29%), Positives = 34/66 (52%), Gaps = 12/66 (18%)

Query  17   THNTQLNVPSSPSPVNRTVSTIMFSRAKRIKL------------AKRVRQTQLVNMINQI  64
            TH+T+ N+ S PSP         FS+ +RI+L            A  +R+   +N +N++
Sbjct  214  THSTEYNLDSVPSPAEEFADFATFSKKERIQLEEIECAVLNDREALTIRENNKINRVNEL  273

Query  65   NHIKKI  70
            N +K++
Sbjct  274  NSVKEV  279


>ref|XP_515513.2| PREDICTED: aftiphilin isoform X4 [Pan troglodytes]
Length=908

 Score = 38.9 bits (89),  Expect = 2.8, Method: Compositional matrix adjust.
 Identities = 19/66 (29%), Positives = 34/66 (52%), Gaps = 12/66 (18%)

Query  17   THNTQLNVPSSPSPVNRTVSTIMFSRAKRIKL------------AKRVRQTQLVNMINQI  64
            TH+T+ N+ S PSP         FS+ +RI+L            A  +R+   +N +N++
Sbjct  214  THSTEYNLDSVPSPAEEFADFATFSKKERIQLEEIECAVLNDREALTIRENNKINRVNEL  273

Query  65   NHIKKI  70
            N +K++
Sbjct  274  NSVKEV  279


>ref|XP_009440833.1| PREDICTED: aftiphilin isoform X3 [Pan troglodytes]
Length=909

 Score = 38.9 bits (89),  Expect = 2.8, Method: Compositional matrix adjust.
 Identities = 19/66 (29%), Positives = 34/66 (52%), Gaps = 12/66 (18%)

Query  17   THNTQLNVPSSPSPVNRTVSTIMFSRAKRIKL------------AKRVRQTQLVNMINQI  64
            TH+T+ N+ S PSP         FS+ +RI+L            A  +R+   +N +N++
Sbjct  214  THSTEYNLDSVPSPAEEFADFATFSKKERIQLEEIECAVLNDREALTIRENNKINRVNEL  273

Query  65   NHIKKI  70
            N +K++
Sbjct  274  NSVKEV  279


>ref|XP_008954570.1| PREDICTED: aftiphilin isoform X3 [Pan paniscus]
Length=909

 Score = 38.9 bits (89),  Expect = 2.8, Method: Compositional matrix adjust.
 Identities = 19/66 (29%), Positives = 34/66 (52%), Gaps = 12/66 (18%)

Query  17   THNTQLNVPSSPSPVNRTVSTIMFSRAKRIKL------------AKRVRQTQLVNMINQI  64
            TH+T+ N+ S PSP         FS+ +RI+L            A  +R+   +N +N++
Sbjct  214  THSTEYNLDSVPSPAEEFADFATFSKKERIQLEEIECAVLNDREALTIRENNKINRVNEL  273

Query  65   NHIKKI  70
            N +K++
Sbjct  274  NSVKEV  279


>ref|XP_007968637.1| PREDICTED: aftiphilin isoform X2 [Chlorocebus sabaeus]
Length=907

 Score = 38.9 bits (89),  Expect = 2.9, Method: Compositional matrix adjust.
 Identities = 19/66 (29%), Positives = 34/66 (52%), Gaps = 12/66 (18%)

Query  17   THNTQLNVPSSPSPVNRTVSTIMFSRAKRIKL------------AKRVRQTQLVNMINQI  64
            TH+T+ N+ S PSP         FS+ +RI+L            A  +R+   +N +N++
Sbjct  214  THSTEYNLDSVPSPAEEFADFATFSKKERIQLEEIECAILNNREALTIRENNKINRVNEL  273

Query  65   NHIKKI  70
            N +K++
Sbjct  274  NSVKEV  279


>ref|XP_003830944.1| PREDICTED: aftiphilin isoform X4 [Pan paniscus]
Length=908

 Score = 38.9 bits (89),  Expect = 2.9, Method: Compositional matrix adjust.
 Identities = 19/66 (29%), Positives = 34/66 (52%), Gaps = 12/66 (18%)

Query  17   THNTQLNVPSSPSPVNRTVSTIMFSRAKRIKL------------AKRVRQTQLVNMINQI  64
            TH+T+ N+ S PSP         FS+ +RI+L            A  +R+   +N +N++
Sbjct  214  THSTEYNLDSVPSPAEEFADFATFSKKERIQLEEIECAVLNDREALTIRENNKINRVNEL  273

Query  65   NHIKKI  70
            N +K++
Sbjct  274  NSVKEV  279


>ref|NP_982261.2| aftiphilin isoform a [Homo sapiens]
Length=936

 Score = 38.9 bits (89),  Expect = 2.9, Method: Compositional matrix adjust.
 Identities = 19/66 (29%), Positives = 34/66 (52%), Gaps = 12/66 (18%)

Query  17   THNTQLNVPSSPSPVNRTVSTIMFSRAKRIKL------------AKRVRQTQLVNMINQI  64
            TH+T+ N+ S PSP         FS+ +RI+L            A  +R+   +N +N++
Sbjct  214  THSTEYNLDSVPSPAEEFADFATFSKKERIQLEEIECAVLNDREALTIRENNKINRVNEL  273

Query  65   NHIKKI  70
            N +K++
Sbjct  274  NSVKEV  279


>gb|AAR14726.1| brain aftiphilin [Homo sapiens]
Length=936

 Score = 38.9 bits (89),  Expect = 2.9, Method: Compositional matrix adjust.
 Identities = 19/66 (29%), Positives = 34/66 (52%), Gaps = 12/66 (18%)

Query  17   THNTQLNVPSSPSPVNRTVSTIMFSRAKRIKL------------AKRVRQTQLVNMINQI  64
            TH+T+ N+ S PSP         FS+ +RI+L            A  +R+   +N +N++
Sbjct  214  THSTEYNLDSVPSPAEEFADFATFSKKERIQLEEIECAVLNDREALTIRENNKINRVNEL  273

Query  65   NHIKKI  70
            N +K++
Sbjct  274  NSVKEV  279


>ref|XP_008954568.1| PREDICTED: aftiphilin isoform X1 [Pan paniscus]
Length=937

 Score = 38.9 bits (89),  Expect = 2.9, Method: Compositional matrix adjust.
 Identities = 19/66 (29%), Positives = 34/66 (52%), Gaps = 12/66 (18%)

Query  17   THNTQLNVPSSPSPVNRTVSTIMFSRAKRIKL------------AKRVRQTQLVNMINQI  64
            TH+T+ N+ S PSP         FS+ +RI+L            A  +R+   +N +N++
Sbjct  214  THSTEYNLDSVPSPAEEFADFATFSKKERIQLEEIECAVLNDREALTIRENNKINRVNEL  273

Query  65   NHIKKI  70
            N +K++
Sbjct  274  NSVKEV  279


>ref|XP_001165739.1| PREDICTED: aftiphilin isoform X2 [Pan troglodytes]
Length=936

 Score = 38.9 bits (89),  Expect = 3.0, Method: Compositional matrix adjust.
 Identities = 19/66 (29%), Positives = 34/66 (52%), Gaps = 12/66 (18%)

Query  17   THNTQLNVPSSPSPVNRTVSTIMFSRAKRIKL------------AKRVRQTQLVNMINQI  64
            TH+T+ N+ S PSP         FS+ +RI+L            A  +R+   +N +N++
Sbjct  214  THSTEYNLDSVPSPAEEFADFATFSKKERIQLEEIECAVLNDREALTIRENNKINRVNEL  273

Query  65   NHIKKI  70
            N +K++
Sbjct  274  NSVKEV  279


>ref|XP_003830945.1| PREDICTED: aftiphilin isoform X2 [Pan paniscus]
Length=936

 Score = 38.9 bits (89),  Expect = 3.0, Method: Compositional matrix adjust.
 Identities = 19/66 (29%), Positives = 34/66 (52%), Gaps = 12/66 (18%)

Query  17   THNTQLNVPSSPSPVNRTVSTIMFSRAKRIKL------------AKRVRQTQLVNMINQI  64
            TH+T+ N+ S PSP         FS+ +RI+L            A  +R+   +N +N++
Sbjct  214  THSTEYNLDSVPSPAEEFADFATFSKKERIQLEEIECAVLNDREALTIRENNKINRVNEL  273

Query  65   NHIKKI  70
            N +K++
Sbjct  274  NSVKEV  279


>ref|XP_009440832.1| PREDICTED: aftiphilin isoform X1 [Pan troglodytes]
Length=937

 Score = 38.9 bits (89),  Expect = 3.0, Method: Compositional matrix adjust.
 Identities = 19/66 (29%), Positives = 34/66 (52%), Gaps = 12/66 (18%)

Query  17   THNTQLNVPSSPSPVNRTVSTIMFSRAKRIKL------------AKRVRQTQLVNMINQI  64
            TH+T+ N+ S PSP         FS+ +RI+L            A  +R+   +N +N++
Sbjct  214  THSTEYNLDSVPSPAEEFADFATFSKKERIQLEEIECAVLNDREALTIRENNKINRVNEL  273

Query  65   NHIKKI  70
            N +K++
Sbjct  274  NSVKEV  279


>ref|XP_007954155.1| PREDICTED: aftiphilin isoform X1 [Orycteropus afer afer]
Length=931

 Score = 38.9 bits (89),  Expect = 3.3, Method: Compositional matrix adjust.
 Identities = 23/69 (33%), Positives = 41/69 (59%), Gaps = 3/69 (4%)

Query  17   THNTQLNVPSSPSPVNRTVSTIMFSRAKRIKLAKRVRQTQLVNMINQINHIKKINKINKI  76
            TH+T+  + S+PSP         FS+ +RI+L + +  T L +   +I  I++ NKIN+I
Sbjct  214  THSTEYTLDSAPSPAEEFADFATFSKKERIQL-EEIGCTVLND--KKILTIQENNKINRI  270

Query  77   NKTDKTKKI  85
            N+ +  K++
Sbjct  271  NELNSVKEL  279


>ref|XP_007954156.1| PREDICTED: aftiphilin isoform X2 [Orycteropus afer afer]
Length=903

 Score = 38.9 bits (89),  Expect = 3.4, Method: Compositional matrix adjust.
 Identities = 25/72 (35%), Positives = 43/72 (60%), Gaps = 4/72 (6%)

Query  15   PL-THNTQLNVPSSPSPVNRTVSTIMFSRAKRIKLAKRVRQTQLVNMINQINHIKKINKI  73
            PL TH+T+  + S+PSP         FS+ +RI+L + +  T L +   +I  I++ NKI
Sbjct  211  PLSTHSTEYTLDSAPSPAEEFADFATFSKKERIQL-EEIGCTVLND--KKILTIQENNKI  267

Query  74   NKINKTDKTKKI  85
            N+IN+ +  K++
Sbjct  268  NRINELNSVKEL  279


>ref|XP_002757747.1| PREDICTED: aftiphilin isoform X2 [Callithrix jacchus]
Length=907

 Score = 38.5 bits (88),  Expect = 4.2, Method: Compositional matrix adjust.
 Identities = 18/66 (27%), Positives = 34/66 (52%), Gaps = 12/66 (18%)

Query  17   THNTQLNVPSSPSPVNRTVSTIMFSRAKRIKL------------AKRVRQTQLVNMINQI  64
            TH T+ N+ S PSP +       FS+ +R++L            A  +R+   +N +N++
Sbjct  214  THTTECNLDSVPSPADEFADFATFSKKERMQLEEIGCAVLNDREALTIRENNKINRVNEL  273

Query  65   NHIKKI  70
            N +K++
Sbjct  274  NSVKEV  279


>ref|XP_002757746.1| PREDICTED: aftiphilin isoform X1 [Callithrix jacchus]
 ref|XP_008978859.1| PREDICTED: aftiphilin isoform X1 [Callithrix jacchus]
Length=935

 Score = 38.5 bits (88),  Expect = 4.2, Method: Compositional matrix adjust.
 Identities = 18/66 (27%), Positives = 34/66 (52%), Gaps = 12/66 (18%)

Query  17   THNTQLNVPSSPSPVNRTVSTIMFSRAKRIKL------------AKRVRQTQLVNMINQI  64
            TH T+ N+ S PSP +       FS+ +R++L            A  +R+   +N +N++
Sbjct  214  THTTECNLDSVPSPADEFADFATFSKKERMQLEEIGCAVLNDREALTIRENNKINRVNEL  273

Query  65   NHIKKI  70
            N +K++
Sbjct  274  NSVKEV  279


>ref|XP_010331840.1| PREDICTED: aftiphilin isoform X1 [Saimiri boliviensis boliviensis]
Length=935

 Score = 38.5 bits (88),  Expect = 4.3, Method: Compositional matrix adjust.
 Identities = 23/72 (32%), Positives = 43/72 (60%), Gaps = 4/72 (6%)

Query  15   PL-THNTQLNVPSSPSPVNRTVSTIMFSRAKRIKLAKRVRQTQLVNMINQINHIKKINKI  73
            PL TH+T+ N+ S PSP +       FS+ +RI+L +   +  ++N    +  I++ NKI
Sbjct  211  PLSTHSTECNLDSVPSPADEFTDFATFSKKERIQLEE--IECAVLNDREALT-IQENNKI  267

Query  74   NKINKTDKTKKI  85
            N++N+ +  K++
Sbjct  268  NRVNELNSVKEV  279


>ref|XP_010331842.1| PREDICTED: aftiphilin isoform X2 [Saimiri boliviensis boliviensis]
Length=907

 Score = 38.5 bits (88),  Expect = 4.4, Method: Compositional matrix adjust.
 Identities = 23/72 (32%), Positives = 43/72 (60%), Gaps = 4/72 (6%)

Query  15   PL-THNTQLNVPSSPSPVNRTVSTIMFSRAKRIKLAKRVRQTQLVNMINQINHIKKINKI  73
            PL TH+T+ N+ S PSP +       FS+ +RI+L +   +  ++N    +  I++ NKI
Sbjct  211  PLSTHSTECNLDSVPSPADEFTDFATFSKKERIQLEE--IECAVLNDREALT-IQENNKI  267

Query  74   NKINKTDKTKKI  85
            N++N+ +  K++
Sbjct  268  NRVNELNSVKEV  279


>ref|WP_011035095.1| hypothetical protein [Methanosarcina mazei]
 gb|AAM32896.1| hypothetical protein MM_3200 [Methanosarcina mazei Go1]
 gb|KKG01686.1| hypothetical protein DU31_11655 [Methanosarcina mazei]
 gb|KKG39438.1| hypothetical protein DU39_17735 [Methanosarcina mazei]
 gb|KKG45154.1| hypothetical protein DU41_18970 [Methanosarcina mazei]
 gb|KKG88940.1| hypothetical protein DU57_00130 [Methanosarcina mazei]
 gb|KKH53349.1| hypothetical protein DU76_10500 [Methanosarcina mazei]
Length=68

 Score = 35.4 bits (80),  Expect = 5.6, Method: Composition-based stats.
 Identities = 16/51 (31%), Positives = 29/51 (57%), Gaps = 0/51 (0%)

Query  52   VRQTQLVNMINQINHIKKINKINKINKTDKTKKIKNIKKVESYRMTSKPVF  102
            V + ++VN I  +N I+ +NKI  +NK +   KI+ + K+E    +   +F
Sbjct  15   VNKIEMVNKIEMVNKIEMVNKIEMVNKIEMVNKIEMVNKIEMVNKSEIGIF  65


 Score = 35.0 bits (79),  Expect = 9.5, Method: Composition-based stats.
 Identities = 16/54 (30%), Positives = 31/54 (57%), Gaps = 0/54 (0%)

Query  39  MFSRAKRIKLAKRVRQTQLVNMINQINHIKKINKINKINKTDKTKKIKNIKKVE  92
           + +R + +   + V + ++VN I  +N I+ +NKI  +NK +   KI+ + K E
Sbjct  8   LINRFEMVNKIEMVNKIEMVNKIEMVNKIEMVNKIEMVNKIEMVNKIEMVNKSE  61


>sp|A8Z5Z0|LON_SULMW RecName: Full=Lon protease; AltName: Full=ATP-dependent protease 
La
 gb|ABS30541.1| ATP-dependent protease [Candidatus Sulcia muelleri GWSS]
Length=855

 Score = 38.1 bits (87),  Expect = 5.8, Method: Composition-based stats.
 Identities = 21/64 (33%), Positives = 32/64 (50%), Gaps = 0/64 (0%)

Query  48   LAKRVRQTQLVNMINQINHIKKINKINKINKTDKTKKIKNIKKVESYRMTSKPVFVLPSV  107
            +AK   QT  +   N+ N I K NKINK NK +K  KI    K+  +  T+   ++    
Sbjct  98   IAKLNIQTFNIYSFNKFNKINKFNKINKFNKINKFNKINKFNKINKFNKTNNIYYIGTVA  157

Query  108  KLFR  111
            K+ +
Sbjct  158  KILK  161


>ref|XP_010356484.1| PREDICTED: aftiphilin [Rhinopithecus roxellana]
Length=826

 Score = 37.7 bits (86),  Expect = 6.9, Method: Compositional matrix adjust.
 Identities = 20/69 (29%), Positives = 35/69 (51%), Gaps = 13/69 (19%)

Query  15   PL-THNTQLNVPSSPSPVNRTVSTIMFSRAKRIKL------------AKRVRQTQLVNMI  61
            PL TH+T+ N+   PSP         FS+ +RI+L            A  +R+   +N +
Sbjct  211  PLSTHSTEYNLDCVPSPAEEFADFATFSKKERIQLEEIECAILNNREALTIRENNKINRV  270

Query  62   NQINHIKKI  70
            N++N +K++
Sbjct  271  NELNSVKEV  279


>ref|XP_012620378.1| PREDICTED: aftiphilin isoform X4 [Microcebus murinus]
Length=804

 Score = 37.7 bits (86),  Expect = 7.9, Method: Compositional matrix adjust.
 Identities = 20/69 (29%), Positives = 36/69 (52%), Gaps = 13/69 (19%)

Query  15   PL-THNTQLNVPSSPSPVNRTVSTIMFSRAKRIKL------------AKRVRQTQLVNMI  61
            PL TH+T+ N+ S PSP         FS+ +RI+L            A  +++   +N +
Sbjct  210  PLSTHSTEYNLDSVPSPAEEFADFATFSKKERIQLEEIGHAVLNDREALTIQENNKINRV  269

Query  62   NQINHIKKI  70
            N++N +K++
Sbjct  270  NELNSVKEV  278


>ref|XP_012620376.1| PREDICTED: aftiphilin isoform X2 [Microcebus murinus]
Length=905

 Score = 37.7 bits (86),  Expect = 8.0, Method: Compositional matrix adjust.
 Identities = 20/69 (29%), Positives = 36/69 (52%), Gaps = 13/69 (19%)

Query  15   PL-THNTQLNVPSSPSPVNRTVSTIMFSRAKRIKL------------AKRVRQTQLVNMI  61
            PL TH+T+ N+ S PSP         FS+ +RI+L            A  +++   +N +
Sbjct  210  PLSTHSTEYNLDSVPSPAEEFADFATFSKKERIQLEEIGHAVLNDREALTIQENNKINRV  269

Query  62   NQINHIKKI  70
            N++N +K++
Sbjct  270  NELNSVKEV  278


>ref|XP_012620377.1| PREDICTED: aftiphilin isoform X3 [Microcebus murinus]
Length=834

 Score = 37.7 bits (86),  Expect = 8.6, Method: Compositional matrix adjust.
 Identities = 20/69 (29%), Positives = 36/69 (52%), Gaps = 13/69 (19%)

Query  15   PL-THNTQLNVPSSPSPVNRTVSTIMFSRAKRIKL------------AKRVRQTQLVNMI  61
            PL TH+T+ N+ S PSP         FS+ +RI+L            A  +++   +N +
Sbjct  210  PLSTHSTEYNLDSVPSPAEEFADFATFSKKERIQLEEIGHAVLNDREALTIQENNKINRV  269

Query  62   NQINHIKKI  70
            N++N +K++
Sbjct  270  NELNSVKEV  278


>ref|XP_012620375.1| PREDICTED: aftiphilin isoform X1 [Microcebus murinus]
Length=933

 Score = 37.7 bits (86),  Expect = 8.6, Method: Compositional matrix adjust.
 Identities = 18/66 (27%), Positives = 34/66 (52%), Gaps = 12/66 (18%)

Query  17   THNTQLNVPSSPSPVNRTVSTIMFSRAKRIKL------------AKRVRQTQLVNMINQI  64
            TH+T+ N+ S PSP         FS+ +RI+L            A  +++   +N +N++
Sbjct  213  THSTEYNLDSVPSPAEEFADFATFSKKERIQLEEIGHAVLNDREALTIQENNKINRVNEL  272

Query  65   NHIKKI  70
            N +K++
Sbjct  273  NSVKEV  278


>ref|XP_007124965.1| PREDICTED: aftiphilin isoform X5 [Physeter catodon]
Length=865

 Score = 37.7 bits (86),  Expect = 8.7, Method: Compositional matrix adjust.
 Identities = 17/66 (26%), Positives = 35/66 (53%), Gaps = 12/66 (18%)

Query  17   THNTQLNVPSSPSPVNRTVSTIMFSRAKRIKL------------AKRVRQTQLVNMINQI  64
            +H+T+ N+ S+PSP         FS+ +RI+L            A  +++   +N +N++
Sbjct  214  SHSTEYNLDSAPSPAEEFADFATFSKKERIQLEEIGCAVLNDREALTIQENNKINRVNEL  273

Query  65   NHIKKI  70
            N +K++
Sbjct  274  NSVKEV  279


>ref|XP_007124964.1| PREDICTED: aftiphilin isoform X4 [Physeter catodon]
Length=893

 Score = 37.7 bits (86),  Expect = 8.7, Method: Compositional matrix adjust.
 Identities = 17/66 (26%), Positives = 35/66 (53%), Gaps = 12/66 (18%)

Query  17   THNTQLNVPSSPSPVNRTVSTIMFSRAKRIKL------------AKRVRQTQLVNMINQI  64
            +H+T+ N+ S+PSP         FS+ +RI+L            A  +++   +N +N++
Sbjct  214  SHSTEYNLDSAPSPAEEFADFATFSKKERIQLEEIGCAVLNDREALTIQENNKINRVNEL  273

Query  65   NHIKKI  70
            N +K++
Sbjct  274  NSVKEV  279


>ref|XP_007124961.1| PREDICTED: aftiphilin isoform X1 [Physeter catodon]
 ref|XP_007124962.1| PREDICTED: aftiphilin isoform X2 [Physeter catodon]
Length=934

 Score = 37.4 bits (85),  Expect = 9.2, Method: Compositional matrix adjust.
 Identities = 17/66 (26%), Positives = 35/66 (53%), Gaps = 12/66 (18%)

Query  17   THNTQLNVPSSPSPVNRTVSTIMFSRAKRIKL------------AKRVRQTQLVNMINQI  64
            +H+T+ N+ S+PSP         FS+ +RI+L            A  +++   +N +N++
Sbjct  214  SHSTEYNLDSAPSPAEEFADFATFSKKERIQLEEIGCAVLNDREALTIQENNKINRVNEL  273

Query  65   NHIKKI  70
            N +K++
Sbjct  274  NSVKEV  279


>ref|XP_007124963.1| PREDICTED: aftiphilin isoform X3 [Physeter catodon]
Length=906

 Score = 37.4 bits (85),  Expect = 9.3, Method: Compositional matrix adjust.
 Identities = 17/66 (26%), Positives = 35/66 (53%), Gaps = 12/66 (18%)

Query  17   THNTQLNVPSSPSPVNRTVSTIMFSRAKRIKL------------AKRVRQTQLVNMINQI  64
            +H+T+ N+ S+PSP         FS+ +RI+L            A  +++   +N +N++
Sbjct  214  SHSTEYNLDSAPSPAEEFADFATFSKKERIQLEEIGCAVLNDREALTIQENNKINRVNEL  273

Query  65   NHIKKI  70
            N +K++
Sbjct  274  NSVKEV  279


Lambda      K        H        a         alpha
   0.314    0.125    0.353    0.792     4.96 

Gapped
Lambda      K        H        a         alpha    sigma
   0.267   0.0410    0.140     1.90     42.6     43.6 

Effective search space used: 1012598240896


  Database: nr
    Posted date:  Sep 23, 2015 12:05 AM
  Number of letters in database: 26,053,659,533
  Number of sequences in database:  71,551,133


Matrix: BLOSUM62
Gap Penalties: Existence: 11, Extension: 1
Neighboring words threshold: 11
Window for multiple hits: 40
```
