# Supplementary material for: Comparative genomics provides new insights into the diversity, physiology, and sexuality of the only industrially exploited tremellomycete: Phaffia rhodozyma
Source: BMC Genomics. 2016 Nov 9;17:901. doi: 10.1186/s12864-016-3244-7 (PMC5103461; doi:10.1186/s12864-016-3244-7)
Supplement: Additional file 6: — List of orphan genes with links to PFAM (related to Additional file 1: Table S1). (ZIP 1428 kb) [file 12864_2016_3244_MOESM6_ESM.zip › BLAST_HTML_FTR/G01873_P.html]

BLAST Search Results


```
BLASTP 2.2.27+


Reference:
Stephen F. Altschul, Thomas L. Madden, Alejandro A. Schäffer,
Jinghui Zhang, Zheng Zhang, Webb Miller, and David J. Lipman (1997),
"Gapped BLAST and PSI-BLAST: a new generation of protein database
search programs", Nucleic Acids Res. 25:3389-3402.


Reference for
composition-based statistics:
Alejandro A. Schäffer, L. Aravind, Thomas L. Madden, Sergei
Shavirin, John L. Spouge, Yuri I. Wolf, Eugene V. Koonin, and
Stephen F. Altschul (2001), "Improving the accuracy of PSI-BLAST
protein database searches with composition-based statistics and
other refinements", Nucleic Acids Res. 29:2994-3005.


Database: nr
           71,551,133 sequences; 26,053,659,533 total letters


Query= G01873_P

Length=198
                                                                      Score     E
Sequences producing significant alignments:                          (Bits)  Value

emb|CED82371.1|  hypothetical protein [Xanthophyllomyces dendrorh...   399    5e-139
gb|KIO20690.1|  hypothetical protein M407DRAFT_29682 [Tulasnella ...  43.1    0.073 
ref|WP_011154895.1|  cell division protein FtsK [Ehrlichia rumina...  42.4    0.21  
ref|WP_011255376.1|  cell division protein FtsK [Ehrlichia rumina...  41.6    0.42  
ref|XP_012055151.1|  PREDICTED: LOW QUALITY PROTEIN: uncharacteri...  38.9    3.5   


 >emb|CED82371.1| hypothetical protein [Xanthophyllomyces dendrorhous]
Length=197

 Score =  399 bits (1024),  Expect = 5e-139, Method: Compositional matrix adjust.
 Identities = 197/197 (100%), Positives = 197/197 (100%), Gaps = 0/197 (0%)

Query  1    MSHSSAHHWSHSHSHAYPPPSVSVDQSASFSSSTFPAEDQPSQTEGGYSDAEELLDQDVD  60
            MSHSSAHHWSHSHSHAYPPPSVSVDQSASFSSSTFPAEDQPSQTEGGYSDAEELLDQDVD
Sbjct  1    MSHSSAHHWSHSHSHAYPPPSVSVDQSASFSSSTFPAEDQPSQTEGGYSDAEELLDQDVD  60

Query  61   DNDVVDESIDDQESEDGSDEALSDTGFEDKMESQLGLWKPTEQEFKATKDLLVSWDWETQ  120
            DNDVVDESIDDQESEDGSDEALSDTGFEDKMESQLGLWKPTEQEFKATKDLLVSWDWETQ
Sbjct  61   DNDVVDESIDDQESEDGSDEALSDTGFEDKMESQLGLWKPTEQEFKATKDLLVSWDWETQ  120

Query  121  TVKEPDQGQVGTEQELVAVAHKAMRRALYSLDRDQWRYDSWPADEQTFAPPFPHRSQRGH  180
            TVKEPDQGQVGTEQELVAVAHKAMRRALYSLDRDQWRYDSWPADEQTFAPPFPHRSQRGH
Sbjct  121  TVKEPDQGQVGTEQELVAVAHKAMRRALYSLDRDQWRYDSWPADEQTFAPPFPHRSQRGH  180

Query  181  NQSQTQGQSQGISVGRL  197
            NQSQTQGQSQGISVGRL
Sbjct  181  NQSQTQGQSQGISVGRL  197


>gb|KIO20690.1| hypothetical protein M407DRAFT_29682 [Tulasnella calospora MUT 
4182]
Length=237

 Score = 43.1 bits (100),  Expect = 0.073, Method: Compositional matrix adjust.
 Identities = 41/144 (28%), Positives = 61/144 (42%), Gaps = 32/144 (22%)

Query  61   DNDVVDESIDDQESEDGSDEALSDTGFEDKMESQLGLWKPTEQEFKATKDLLVSWDWETQ  120
            DN + DES         SD  L D  +  K+E ++GL +PTE E +A +  L  +DWE  
Sbjct  28   DNHIEDES---------SDLELDDQAYALKIEQEIGLGQPTEAEMQANQTTL--YDWEKM  76

Query  121  TVKEPDQGQVGTEQELVAVAHKAMRRALYSLDR-DQWRYDSWPADEQTFA----------  169
                  +G V  EQ     A  ++R  +  + R D++R  +  A   T +          
Sbjct  77   ------KGDVRAEQAAKVTALSSLRDTVQKMSREDRFRATNLYAPPATVSQPTISAYPLT  130

Query  170  ----PPFPHRSQRGHNQSQTQGQS  189
                PP P  +Q G     T+G S
Sbjct  131  GPNGPPPPSVNQAGPTGGGTRGVS  154


>ref|WP_011154895.1| cell division protein FtsK [Ehrlichia ruminantium]
 emb|CAH57927.1| putative cell division protein FtsK [Ehrlichia ruminantium str. 
Welgevonden]
Length=855

 Score = 42.4 bits (98),  Expect = 0.21, Method: Composition-based stats.
 Identities = 24/53 (45%), Positives = 33/53 (62%), Gaps = 4/53 (8%)

Query  39   DQPSQTEGGYSDAEELLDQDVDDNDVVDESIDDQESEDG--SDEALSDTGFED  89
            DQ  + E  ++D E+L DQD +D   +DE + DQ+ ED   +DE LSD  FED
Sbjct  256  DQDFEDES-FAD-EDLSDQDFEDESFIDEDLSDQDFEDDGFTDEDLSDQDFED  306


 Score = 41.6 bits (96),  Expect = 0.40, Method: Composition-based stats.
 Identities = 20/41 (49%), Positives = 29/41 (71%), Gaps = 2/41 (5%)

Query  52   EELLDQDVDDNDVVDESIDDQESEDGS--DEALSDTGFEDK  90
            E+LLD++++  D VD+ + DQ+ ED S  DE LSD  FED+
Sbjct  237  EDLLDKELETGDFVDKDLSDQDFEDESFADEDLSDQDFEDE  277


 Score = 40.0 bits (92),  Expect = 1.1, Method: Composition-based stats.
 Identities = 22/46 (48%), Positives = 28/46 (61%), Gaps = 3/46 (7%)

Query  46   GGYSDAEELLDQDVDDNDVVDESIDDQESEDGS--DEALSDTGFED  89
            G + D ++L DQD +D    DE + DQ+ ED S  DE LSD  FED
Sbjct  247  GDFVD-KDLSDQDFEDESFADEDLSDQDFEDESFIDEDLSDQDFED  291


>ref|WP_011255376.1| cell division protein FtsK [Ehrlichia ruminantium]
 emb|CAI27658.1| DNA translocase ftsK [Ehrlichia ruminantium str. Gardel]
Length=855

 Score = 41.6 bits (96),  Expect = 0.42, Method: Composition-based stats.
 Identities = 20/41 (49%), Positives = 29/41 (71%), Gaps = 2/41 (5%)

Query  52   EELLDQDVDDNDVVDESIDDQESEDGS--DEALSDTGFEDK  90
            E+LLD++++  D VD+ + DQ+ ED S  DE LSD  FED+
Sbjct  237  EDLLDKELETGDFVDKDLSDQDFEDESFADEDLSDQDFEDE  277


 Score = 40.4 bits (93),  Expect = 0.90, Method: Composition-based stats.
 Identities = 22/47 (47%), Positives = 29/47 (62%), Gaps = 3/47 (6%)

Query  46   GGYSDAEELLDQDVDDNDVVDESIDDQESEDGS--DEALSDTGFEDK  90
            G + D ++L DQD +D    DE + DQ+ ED S  DE LSD  FED+
Sbjct  247  GDFVD-KDLSDQDFEDESFADEDLSDQDFEDESFADEDLSDQDFEDE  292


>ref|XP_012055151.1| PREDICTED: LOW QUALITY PROTEIN: uncharacterized protein LOC105618219 
[Atta cephalotes]
Length=2156

 Score = 38.9 bits (89),  Expect = 3.5, Method: Compositional matrix adjust.
 Identities = 26/106 (25%), Positives = 48/106 (45%), Gaps = 0/106 (0%)

Query  57    QDVDDNDVVDESIDDQESEDGSDEALSDTGFEDKMESQLGLWKPTEQEFKATKDLLVSWD  116
             Q +D   VV ESID + +   S +  + T  +  ++S +G    T+   K  K  L    
Sbjct  1582  QTMDQKKVVKESIDSKGTTVPSRKVTNHTILKSTLKSPIGKNLKTKNVVKVRKSDLRILK  1641

Query  117   WETQTVKEPDQGQVGTEQELVAVAHKAMRRALYSLDRDQWRYDSWP  162
              ET++ K P +  + ++Q    +   A +R L+  D ++   +  P
Sbjct  1642  KETRSSKRPTRSSIESKQRSTKIQSPACKRRLFESDSEKELQEELP  1687


Lambda      K        H        a         alpha
   0.310    0.126    0.380    0.792     4.96 

Gapped
Lambda      K        H        a         alpha    sigma
   0.267   0.0410    0.140     1.90     42.6     43.6 

Effective search space used: 890030324232


  Database: nr
    Posted date:  Sep 23, 2015 12:05 AM
  Number of letters in database: 26,053,659,533
  Number of sequences in database:  71,551,133


Matrix: BLOSUM62
Gap Penalties: Existence: 11, Extension: 1
Neighboring words threshold: 11
Window for multiple hits: 40
```
