# Supplementary material for: Comparative genomics provides new insights into the diversity, physiology, and sexuality of the only industrially exploited tremellomycete: Phaffia rhodozyma
Source: BMC Genomics. 2016 Nov 9;17:901. doi: 10.1186/s12864-016-3244-7 (PMC5103461; doi:10.1186/s12864-016-3244-7)
Supplement: Additional file 6: — List of orphan genes with links to PFAM (related to Additional file 1: Table S1). (ZIP 1428 kb) [file 12864_2016_3244_MOESM6_ESM.zip › BLAST_HTML_FTR/G00640_P.html]

BLAST Search Results


```
BLASTP 2.2.27+


Reference:
Stephen F. Altschul, Thomas L. Madden, Alejandro A. Schäffer,
Jinghui Zhang, Zheng Zhang, Webb Miller, and David J. Lipman (1997),
"Gapped BLAST and PSI-BLAST: a new generation of protein database
search programs", Nucleic Acids Res. 25:3389-3402.


Reference for
composition-based statistics:
Alejandro A. Schäffer, L. Aravind, Thomas L. Madden, Sergei
Shavirin, John L. Spouge, Yuri I. Wolf, Eugene V. Koonin, and
Stephen F. Altschul (2001), "Improving the accuracy of PSI-BLAST
protein database searches with composition-based statistics and
other refinements", Nucleic Acids Res. 29:2994-3005.


Database: nr
           71,551,133 sequences; 26,053,659,533 total letters


Query= G00640_P

Length=824
                                                                      Score     E
Sequences producing significant alignments:                          (Bits)  Value

emb|CED82534.1|  hypothetical protein [Xanthophyllomyces dendrorh...  1071    0.0  
emb|CED82526.1|  hypothetical protein [Xanthophyllomyces dendrorh...   127    9e-27
emb|CED82543.1|  hypothetical protein [Xanthophyllomyces dendrorh...  75.9    6e-11


 >emb|CED82534.1| hypothetical protein [Xanthophyllomyces dendrorhous]
Length=916

 Score = 1071 bits (2771),  Expect = 0.0, Method: Compositional matrix adjust.
 Identities = 514/516 (99%), Positives = 514/516 (99%), Gaps = 0/516 (0%)

Query  308  TLLVHNLPSNIQKDQIDSCLDSLGGMPPMKLLRWESNRTAIFSLSNPSEESRESIKRVHG  367
            T  VHNLPSNIQKDQIDSCLDSLGGMPPMKLLRWESNRTAIFSLSNPSEESRESIKRVHG
Sbjct  401  TFRVHNLPSNIQKDQIDSCLDSLGGMPPMKLLRWESNRTAIFSLSNPSEESRESIKRVHG  460

Query  368  NSIHGKTITIEILPQDLRSSTKGALAVEDVSIQQSPTHDGDYRENVPKDTGRLVDELSYL  427
            NSIHGKTITIEILPQDLRSSTKGALAVEDVSIQQSPTHDGDYRENVPKDTGRLVDELSYL
Sbjct  461  NSIHGKTITIEILPQDLRSSTKGALAVEDVSIQQSPTHDGDYRENVPKDTGRLVDELSYL  520

Query  428  KSLFASYAPQYLQLYVMSQAKFQRSFLKGISQGTITNTKQMYKSDEQDHQTTKRLLIESD  487
            KSLFASYAPQYLQLYVMSQAKFQRSFLKGISQGTITNTKQMYKSDEQDHQTTKRLLIESD
Sbjct  521  KSLFASYAPQYLQLYVMSQAKFQRSFLKGISQGTITNTKQMYKSDEQDHQTTKRLLIESD  580

Query  488  LLSVREILYHPNCGLFTPTSFDFDRRYSPPYLTECAKMFYKEVRGYPDGSLMQGLRSKSE  547
            LLSVREILYHPNCGLFTPTSFDFDRRYSPPYLTECAKMFYKEVRGYPDGSLMQGLRSKSE
Sbjct  581  LLSVREILYHPNCGLFTPTSFDFDRRYSPPYLTECAKMFYKEVRGYPDGSLMQGLRSKSE  640

Query  548  LSARSMDVSEWTVKFGRIVQDLVQSGVSDSYSVPVVKDLEKQIANYSTSIRTHIDDARDL  607
            LSARSMDVSEWTVKFGRIVQDLVQSGVSDSYSVPVVKDLEKQIANYSTSIRTHIDDARDL
Sbjct  641  LSARSMDVSEWTVKFGRIVQDLVQSGVSDSYSVPVVKDLEKQIANYSTSIRTHIDDARDL  700

Query  608  LMVVSYIKYCRAVYYQSHQVDGQKPMNVFNDLVQGFDRRFEAHKKNLKGLQKLRNNIDRG  667
            LMVVSYIKYCRAVYYQSHQVDGQKPMNVFNDLVQGFDRRFEAHKKNLKGLQKLRNNIDRG
Sbjct  701  LMVVSYIKYCRAVYYQSHQVDGQKPMNVFNDLVQGFDRRFEAHKKNLKGLQKLRNNIDRG  760

Query  668  LIRHGLQPCVDFLHMLVSTRARAEYVEGLLEIVRHPTHGFNGAFPFDGEVIPNPPYATTL  727
            LIRHGLQPCVDFLHMLVSTRARAEYVEGLLEIVRHPTHGFNGAFPFDGEVIPNPPYATTL
Sbjct  761  LIRHGLQPCVDFLHMLVSTRARAEYVEGLLEIVRHPTHGFNGAFPFDGEVIPNPPYATTL  820

Query  728  ARTFGSLIKANWQQRSKTHKVTASKMRLQSVLRTTDIQKLAPIYVPSLLSSFVRSEQIKA  787
            ARTFGSLIKANWQQRSKTHKVTASKMRLQSVLRTTDIQKLAPIYVPSLLSSFVRSEQIKA
Sbjct  821  ARTFGSLIKANWQQRSKTHKVTASKMRLQSVLRTTDIQKLAPIYVPSLLSSFVRSEQIKA  880

Query  788  GRLMENPPKRMTVQVNESVVKPTRAVAKLRKNKGKT  823
            GRLMENPPKRMTVQVNESVVKPTRAVAKLRKNKGKT
Sbjct  881  GRLMENPPKRMTVQVNESVVKPTRAVAKLRKNKGKT  916


 Score =  597 bits (1538),  Expect = 0.0, Method: Compositional matrix adjust.
 Identities = 327/391 (84%), Positives = 345/391 (88%), Gaps = 6/391 (2%)

Query  1    MLPGKIQRRYINLFLGSSFQRSSSSSSLVTLKGKPCYSPPPSASFFRSRSSTAASRLKEN  60
            MLPGKIQRRYINLFLGSSFQRSSSSSSLVTLKGKPCYSPPPSASFFRSRSSTAASRLKEN
Sbjct  1    MLPGKIQRRYINLFLGSSFQRSSSSSSLVTLKGKPCYSPPPSASFFRSRSSTAASRLKEN  60

Query  61   GVSVRTETKEIPRDGHQVITSIFPLPPDMNSYSLRNFFWSNSINVLDARKSTGEVSGLIQ  120
            GVSVRTETKEIPRDGHQVITSIFPLPPDMNSYSLRNFFWSNSINVLDARKSTGEVSGLIQ
Sbjct  61   GVSVRTETKEIPRDGHQVITSIFPLPPDMNSYSLRNFFWSNSINVLDARKSTGEVSGLIQ  120

Query  121  TSQEDQQKLLSSIHDIQAHWGNLTAVPASPAMTAIVPSPKQERKFLEALSTSKRGQNPRV  180
            TSQEDQQKLLSSIHDIQAHWGNLTAVPASPAMTAIVPSPKQERKFLEALSTSKRGQNPRV
Sbjct  121  TSQEDQQKLLSSIHDIQAHWGNLTAVPASPAMTAIVPSPKQERKFLEALSTSKRGQNPRV  180

Query  181  VSSSAQVGLANKYESRDISRSDSRILFLSSIPELVTMQISFSKKFPRKHIANDLRELLLR  240
            VSSSAQVGLANKYESRDISRSDSRILFLSSIPELVTMQISFSKKFPRKHIANDLRELLLR
Sbjct  181  VSSSAQVGLANKYESRDISRSDSRILFLSSIPELVTMQISFSKKFPRKHIANDLRELLLR  240

Query  241  EGWAVPSIAMLNQQILVDIGGISVYELKKMVMRVQPELQSDMLDIAPLYWMSSSSDKNAN  300
            EGWAVPSIAMLNQQILVDIGGISVYELKKMVMRVQPELQSDMLDIAPLYWMSSSSDKNAN
Sbjct  241  EGWAVPSIAMLNQQILVDIGGISVYELKKMVMRVQPELQSDMLDIAPLYWMSSSSDKNAN  300

Query  301  RNCLSGRTLLVHNL-PSNIQKDQIDSCLDSLGGMPPMKLLRWE---SNRTAIFSLSNPSE  356
            RNCLSGRTLL+ +L P+   +D +      +  + P  + R        TA+  + +   
Sbjct  301  RNCLSGRTLLISSLSPAMSCQDIMLYIRTLIPDIHPNSINRIRKKIGECTAVIPVQD--R  358

Query  357  ESRESIKRVHGNSIHGKTITIEILPQDLRSS  387
            E+ +SI  +HG    G+ + I ++P    SS
Sbjct  359  ETLQSILGLHGRCFGGRPVNIRVIPAKTDSS  389


>emb|CED82526.1| hypothetical protein [Xanthophyllomyces dendrorhous]
Length=980

 Score =  127 bits (320),  Expect = 9e-27, Method: Compositional matrix adjust.
 Identities = 94/335 (28%), Positives = 162/335 (48%), Gaps = 27/335 (8%)

Query  77   QVITSIFPLPPDMNSYSLRNFFWSNSINVLDARKSTGEVSGLIQTSQEDQQKLLSSIHDI  136
            QVIT I  LP +++S + R+FFWS  ++V++A K+TG+ SG+IQTS  DQ  +  ++   
Sbjct  135  QVITYIHRLPLELDSITFRDFFWSRGVHVVEAWKATGKRSGIIQTSISDQDLVCDTMDGT  194

Query  137  QAHWGNLTAVPASPAMTAIVPSPKQERKFLEALSTSKRG---------QNPRV-------  180
            QA WG L A P   A T I  S   E  F +A S +             NP+        
Sbjct  195  QAPWGLLIAKPGDTASTMIKLSEADEHAFQKASSQNPDSSYTIDKNVTSNPKAESLDDMT  254

Query  181  -VSSSAQVGLANKYESRDISRSDSRILFLSSIPELVTMQISFSKKFPRKHIANDLRELLL  239
             V+S A V +++  +S ++ +     L+L  IP + T+QI    K         + E L 
Sbjct  255  DVASEASVKISSDNQSDELRQE----LYLQRIPNMKTLQIDIDPKAEVTLNETAVIEALT  310

Query  240  REGWAVPSIAMLNQQILVDIGGISVYELKKMVMRVQPELQSDMLDIAPLYWMSSSSDKNA  299
              G+ VP+  + + +IL D+G +S    ++++ +  P +++++L++    W SS   ++A
Sbjct  311  SRGYKVPAYHIKDNRILFDVGDLSAEGFREILAKSHPTIKANVLEVVAFSWRSSFFSESA  370

Query  300  NRNCLSGRTLLVHNLPSNIQKDQIDSCLDSLGGMPPMKLLRWESNRT----AIFSLSNPS  355
                 S  +LL+ +L        +   L  L     +  +RW   R     AI ++S   
Sbjct  371  LYEVNSRTSLLITSLSPTTSGKLVLRYLLELAPQLKLSSIRWIEKRVRETWAIVTVSG--  428

Query  356  EESRESIKRVHGNSIHGKTITIEILPQDLRSSTKG  390
            +++ ES+  VH   I G  + +  +P + + S KG
Sbjct  429  QQAFESVLNVHQQPIGGVPVDVRAMPIETKGSIKG  463


 Score = 86.3 bits (212),  Expect = 9e-14, Method: Compositional matrix adjust.
 Identities = 111/479 (23%), Positives = 179/479 (37%), Gaps = 73/479 (15%)

Query  311  VHNLPSNIQKDQIDSCLDSLGGMPPMKLLRWESNRTAIFSLSNPSEESRESIKRVHGNSI  370
            +HN P N+  DQ+ +        P ++ +   + R A F L NPS E+R  +  +  + I
Sbjct  474  IHNFPFNLTDDQVRNLFSGGEKSPAVEFIGRINPRAATFKLLNPSVEARRDLMSMQMSVI  533

Query  371  HGKTITIEIL-PQDLRSSTKGALAVEDVSIQQSPTH-DGD---------YRENVPKDTG-  418
            HG+ + +EI+ PQDL        ++    I +S    DG          +R+N  KD   
Sbjct  534  HGRALQLEIVTPQDLEKPKHYTNSIAGSQISRSSKRTDGPDAGPRLLQRFRKNNYKDAAP  593

Query  419  -----------------------------RLVDELSYLKSLFA--------------SYA  435
                                         RL  EL  L+  F                  
Sbjct  594  VMGNDELIRTMRVFSLSCYIILRNAMEIIRLGQELKGLQFAFVRAKREEGLQEGPNPEIV  653

Query  436  PQYLQLYVMSQAKFQ--RSFLKGISQGTITNTKQMYKSDEQDHQTTKRLLIESDLLSVRE  493
            P +L+    S    +  RS    +      N +Q  +S +Q  +   R  IE  L   RE
Sbjct  654  PNFLKRICASPTNLEPWRSLRYEVFTAQKMNHQQQLRS-QQIAENEVRQKIEQGL---RE  709

Query  494  ILYHPNCGLFTPTSFDFDRRYSPPYLTECAKMFYKEVRGYPDGSLMQGLRSKSELSARSM  553
            I+YHP  G+     FDF     PPY    +KMF + V   P       L S ++L+   +
Sbjct  710  IMYHPTHGIHALRPFDFKSLPQPPYRFPLSKMFQRIVLQDPSV-----LVSMNDLTPNPL  764

Query  554  DVSEWTVKFGRIVQDLVQSGVSDSYSVPVVKDLEKQIANYSTSIRTHIDDARDLLMVVSY  613
               +   +  +   D+ +   SD  S      L  ++  +S       +  R    +  +
Sbjct  765  SQVDQKSELQQAPHDVHRVRTSDPLSNTERMLLRGRMRAFSPFFFDIFESYRKRSELSIF  824

Query  614  IKYCRAVYYQSHQVDGQKPMNVFNDLVQGFDRRFEAHKKNLKGLQKLRNNIDRGLIRHGL  673
            ++  +  Y Q     G       ++++  F  R  +   +L  L  LR  + + + +  L
Sbjct  825  LQRLQQAYSQLEDKKGGD-----SEIIPKFHYRLWSILGDLGSLVSLRTEVCQHIEKQKL  879

Query  674  Q-PCVDFLHMLVSTRARAEYVEGLLEIVRHPTHGFNGAFPFDGEVIPNPPYATTLARTF  731
                VD L  +   +A   Y EGL E++ HPTHG +  F  +      PPY   LA  F
Sbjct  880  DIASVDILR-IAQIQAAKSYYEGLKEVIYHPTHGLDAKFSLNYRHPVEPPYQNELACRF  937


 Score = 50.1 bits (118),  Expect = 0.012, Method: Compositional matrix adjust.
 Identities = 40/173 (23%), Positives = 81/173 (47%), Gaps = 5/173 (3%)

Query  564  RIVQDLVQSGVSDSYSVPVVKDLEKQIANYSTSIRTHIDDARDLLMVVSYIKYCRAVYYQ  623
            R++Q   ++   D+  V    +L + +  +S S    + +A +++ +   +K  +  + +
Sbjct  578  RLLQRFRKNNYKDAAPVMGNDELIRTMRVFSLSCYIILRNAMEIIRLGQELKGLQFAFVR  637

Query  624  SHQVDG-QKPMNVFNDLVQGFDRRFEAHKKNLKGLQKLRNNIDRGLIRHGLQPCVDFLHM  682
            + + +G Q+  N   ++V  F +R  A   NL+  + LR  +      +  Q        
Sbjct  638  AKREEGLQEGPN--PEIVPNFLKRICASPTNLEPWRSLRYEVFTAQKMNHQQQLRS--QQ  693

Query  683  LVSTRARAEYVEGLLEIVRHPTHGFNGAFPFDGEVIPNPPYATTLARTFGSLI  735
            +     R +  +GL EI+ HPTHG +   PFD + +P PPY   L++ F  ++
Sbjct  694  IAENEVRQKIEQGLREIMYHPTHGIHALRPFDFKSLPQPPYRFPLSKMFQRIV  746


>emb|CED82543.1| hypothetical protein [Xanthophyllomyces dendrorhous]
Length=419

 Score = 75.9 bits (185),  Expect = 6e-11, Method: Compositional matrix adjust.
 Identities = 46/103 (45%), Positives = 59/103 (57%), Gaps = 0/103 (0%)

Query  63   SVRTETKEIPRDGHQVITSIFPLPPDMNSYSLRNFFWSNSINVLDARKSTGEVSGLIQTS  122
            + R   KE      QV+T I  LPP+++S +LR FFW++ I VL+A K  G+ SG+IQTS
Sbjct  49   TTRPAEKEPKSQPVQVVTCIHLLPPELDSNTLRKFFWTSGIRVLEAWKPIGKRSGMIQTS  108

Query  123  QEDQQKLLSSIHDIQAHWGNLTAVPASPAMTAIVPSPKQERKF  165
             EDQ K L  I+     WG L    A  A  AIV SP +E  F
Sbjct  109  FEDQFKALRRINREPRPWGILQTQSAGLATVAIVLSPLEESSF  151


Lambda      K        H        a         alpha
   0.318    0.132    0.379    0.792     4.96 

Gapped
Lambda      K        H        a         alpha    sigma
   0.267   0.0410    0.140     1.90     42.6     43.6 

Effective search space used: 9698037559992


  Database: nr
    Posted date:  Sep 23, 2015 12:05 AM
  Number of letters in database: 26,053,659,533
  Number of sequences in database:  71,551,133


Matrix: BLOSUM62
Gap Penalties: Existence: 11, Extension: 1
Neighboring words threshold: 11
Window for multiple hits: 40
```
