# Supplementary material for: Comparative genomics provides new insights into the diversity, physiology, and sexuality of the only industrially exploited tremellomycete: Phaffia rhodozyma
Source: BMC Genomics. 2016 Nov 9;17:901. doi: 10.1186/s12864-016-3244-7 (PMC5103461; doi:10.1186/s12864-016-3244-7)
Supplement: Additional file 6: — List of orphan genes with links to PFAM (related to Additional file 1: Table S1). (ZIP 1428 kb) [file 12864_2016_3244_MOESM6_ESM.zip › BLAST_HTML_FTR/G00120_P.html]

BLAST Search Results


```
BLASTP 2.2.27+


Reference:
Stephen F. Altschul, Thomas L. Madden, Alejandro A. Schäffer,
Jinghui Zhang, Zheng Zhang, Webb Miller, and David J. Lipman (1997),
"Gapped BLAST and PSI-BLAST: a new generation of protein database
search programs", Nucleic Acids Res. 25:3389-3402.


Reference for
composition-based statistics:
Alejandro A. Schäffer, L. Aravind, Thomas L. Madden, Sergei
Shavirin, John L. Spouge, Yuri I. Wolf, Eugene V. Koonin, and
Stephen F. Altschul (2001), "Improving the accuracy of PSI-BLAST
protein database searches with composition-based statistics and
other refinements", Nucleic Acids Res. 29:2994-3005.


Database: nr
           71,551,133 sequences; 26,053,659,533 total letters


Query= G00120_P

Length=146
                                                                      Score     E
Sequences producing significant alignments:                          (Bits)  Value

emb|CDZ97809.1|  Transposase IS605, OrfB, C-terminal [Xanthophyll...  50.4    1e-04
emb|CDZ97488.1|  isocitrate dehydrogenase [Xanthophyllomyces dend...  45.8    0.006
emb|CED85469.1|  hypothetical protein [Xanthophyllomyces dendrorh...  42.7    0.052
ref|XP_007595345.1|  hypothetical protein CFIO01_08907 [Colletotr...  37.7    2.7  


 >emb|CDZ97809.1| Transposase IS605, OrfB, C-terminal [Xanthophyllomyces dendrorhous]
Length=634

 Score = 50.4 bits (119),  Expect = 1e-04, Method: Compositional matrix adjust.
 Identities = 35/93 (38%), Positives = 45/93 (48%), Gaps = 9/93 (10%)

Query  60   KPVIEQTIGELDISLDNGGLAERGTQWSLSQVMEAPSGNGPRRSEAAAVNENQCGRVNS-  118
            + ++  T G LD+SLDNGGLAERGTQWSLS           R +    +       V++ 
Sbjct  389  RTLLSTTAGGLDMSLDNGGLAERGTQWSLSLKSWRHLAGMDRDARKQRLWMKNHAEVSTT  448

Query  119  --------SPWPDGLRAATASRCRSYFWHSTFF  143
                    S   D LRAA     +SY W +TFF
Sbjct  449  LRDLPSFRSASLDRLRAALVIHSQSYSWLATFF  481


>emb|CDZ97488.1| isocitrate dehydrogenase [Xanthophyllomyces dendrorhous]
Length=1099

 Score = 45.8 bits (107),  Expect = 0.006, Method: Compositional matrix adjust.
 Identities = 21/30 (70%), Positives = 25/30 (83%), Gaps = 0/30 (0%)

Query  60   KPVIEQTIGELDISLDNGGLAERGTQWSLS  89
            + ++  T G LD+SLDNGGLAERGTQWSLS
Sbjct  484  RTLLSTTAGGLDMSLDNGGLAERGTQWSLS  513


>emb|CED85469.1| hypothetical protein [Xanthophyllomyces dendrorhous]
Length=487

 Score = 42.7 bits (99),  Expect = 0.052, Method: Compositional matrix adjust.
 Identities = 19/30 (63%), Positives = 24/30 (80%), Gaps = 0/30 (0%)

Query  60   KPVIEQTIGELDISLDNGGLAERGTQWSLS  89
            + ++  T G +D+SLDNGGLAE GTQWSLS
Sbjct  456  RTLLSTTAGGIDMSLDNGGLAEHGTQWSLS  485


>ref|XP_007595345.1| hypothetical protein CFIO01_08907 [Colletotrichum fioriniae PJ7]
 gb|EXF81002.1| hypothetical protein CFIO01_08907 [Colletotrichum fioriniae PJ7]
Length=1120

 Score = 37.7 bits (86),  Expect = 2.7, Method: Compositional matrix adjust.
 Identities = 19/43 (44%), Positives = 24/43 (56%), Gaps = 0/43 (0%)

Query  24   PGRSVWTGLERPPERLDLHRLHLTSTTTGGDRKVKAKPVIEQT  66
            P  SV TG   PP R D H  H ++TT       +A+PVIEQ+
Sbjct  645  PAASVSTGAGNPPHRSDSHDSHASATTIASSVASEAEPVIEQS  687


Lambda      K        H        a         alpha
   0.319    0.135    0.429    0.792     4.96 

Gapped
Lambda      K        H        a         alpha    sigma
   0.267   0.0410    0.140     1.90     42.6     43.6 

Effective search space used: 654589256508


  Database: nr
    Posted date:  Sep 23, 2015 12:05 AM
  Number of letters in database: 26,053,659,533
  Number of sequences in database:  71,551,133


Matrix: BLOSUM62
Gap Penalties: Existence: 11, Extension: 1
Neighboring words threshold: 11
Window for multiple hits: 40
```
