# Supplementary material for: Comparative genomics provides new insights into the diversity, physiology, and sexuality of the only industrially exploited tremellomycete: Phaffia rhodozyma
Source: BMC Genomics. 2016 Nov 9;17:901. doi: 10.1186/s12864-016-3244-7 (PMC5103461; doi:10.1186/s12864-016-3244-7)
Supplement: Additional file 6: — List of orphan genes with links to PFAM (related to Additional file 1: Table S1). (ZIP 1428 kb) [file 12864_2016_3244_MOESM6_ESM.zip › BLAST_HTML_FTR/G00216_P.html]

BLAST Search Results


```
BLASTP 2.2.27+


Reference:
Stephen F. Altschul, Thomas L. Madden, Alejandro A. Schäffer,
Jinghui Zhang, Zheng Zhang, Webb Miller, and David J. Lipman (1997),
"Gapped BLAST and PSI-BLAST: a new generation of protein database
search programs", Nucleic Acids Res. 25:3389-3402.


Reference for
composition-based statistics:
Alejandro A. Schäffer, L. Aravind, Thomas L. Madden, Sergei
Shavirin, John L. Spouge, Yuri I. Wolf, Eugene V. Koonin, and
Stephen F. Altschul (2001), "Improving the accuracy of PSI-BLAST
protein database searches with composition-based statistics and
other refinements", Nucleic Acids Res. 29:2994-3005.


Database: nr
           71,551,133 sequences; 26,053,659,533 total letters


Query= G00216_P

Length=194
                                                                      Score     E
Sequences producing significant alignments:                          (Bits)  Value

emb|CED84066.1|  Tetratricopeptide-like helical [Xanthophyllomyce...   310    3e-104
ref|WP_008293009.1|  adenylyl cyclase [Congregibacter litoralis] ...  40.8    0.65  
ref|WP_050752366.1|  hypothetical protein [Scardovia inopinata] >...  39.7    1.8   
gb|EEH07821.1|  DnaJ and TPR domain-containing protein [Histoplas...  39.3    1.8   
gb|EER42779.1|  DnaJ and TPR domain-containing protein [Histoplas...  39.3    1.8   
gb|KNC95962.1|  hypothetical protein SPPG_08567 [Spizellomyces pu...  39.3    1.9   
ref|XP_006884633.1|  PREDICTED: suppressor of G2 allele of SKP1 h...  38.9    2.1   
gb|KNG46503.1|  cysteine synthase 2 [Stemphylium lycopersici]         39.3    2.1   
gb|EGE86291.1|  DnaJ like subfamily C member 3 [Blastomyces derma...  38.9    2.7   
ref|XP_002620698.1|  DnaJ domain-containing protein [Blastomyces ...  38.9    2.7   
ref|WP_009025162.1|  adenylyl cyclase [gamma proteobacterium NOR5...  38.5    3.5   
ref|XP_006176337.1|  PREDICTED: suppressor of G2 allele of SKP1 h...  38.1    3.7   
gb|KLJ13223.1|  DnaJ like subfamily C member 3 [Emmonsia parva UA...  38.5    3.8   
gb|EPS26558.1|  hypothetical protein PDE_01495 [Penicillium oxali...  38.5    3.8   
ref|XP_006215630.1|  PREDICTED: suppressor of G2 allele of SKP1 h...  38.1    3.9   
gb|EIE91049.1|  hypothetical protein RO3G_15760 [Rhizopus delemar...  38.1    3.9   
gb|EPY86814.1|  fermitin family 3 short form-like protein [Camelu...  37.7    6.9   
ref|XP_002789233.1|  DnaJ domain-containing protein [Paracoccidio...  37.4    7.2   
ref|XP_008614527.1|  hypothetical protein SDRG_10321 [Saprolegnia...  37.4    7.7   
gb|EEH23396.1|  hypothetical protein PABG_05607 [Paracoccidioides...  37.4    7.7   
ref|XP_010761479.1|  hypothetical protein PADG_05928 [Paracoccidi...  37.4    8.3   
ref|XP_009016225.1|  hypothetical protein HELRODRAFT_185497 [Helo...  37.0    9.4   
ref|WP_007147424.1|  hypothetical protein [Scardovia wiggsiae] >g...  37.4    9.4   
gb|KOO33536.1|  ion channel [Chrysochromulina sp. CCMP291]            37.4    9.5   


 >emb|CED84066.1| Tetratricopeptide-like helical [Xanthophyllomyces dendrorhous]
Length=195

 Score =  310 bits (795),  Expect = 3e-104, Method: Compositional matrix adjust.
 Identities = 166/168 (99%), Positives = 166/168 (99%), Gaps = 2/168 (1%)

Query  1    MTTISADLSALSIKDLGSVPLEELLTTASTYLVGSPQQSLPYLNQALILSPSSAPIHAQR  60
            MTTISADLSALSIKDLGSVPLEELLTTASTYLVGSPQQSLPYLNQALILSPSSAPIHAQR
Sbjct  1    MTTISADLSALSIKDLGSVPLEELLTTASTYLVGSPQQSLPYLNQALILSPSSAPIHAQR  60

Query  61   AVAYTLLENWPRAYFDACQGLDDDAEGLTDELRAELIGLAGKCAAERMIWDLA--HLEEA  118
            AVAYTLLENWPRAYFDACQGLDDDAEGLTDELRAELIGLAGKCAAERMIWDLA  HLEEA
Sbjct  61   AVAYTLLENWPRAYFDACQGLDDDAEGLTDELRAELIGLAGKCAAERMIWDLAVIHLEEA  120

Query  119  IGLTRTSALLALQAKALSNLQKTSVSPVRTRMLNQRKTKFLEEAFPGI  166
            IGLTRTSALLALQAKALSNLQKTSVSPVRTRMLNQRKTKFLEEAFPGI
Sbjct  121  IGLTRTSALLALQAKALSNLQKTSVSPVRTRMLNQRKTKFLEEAFPGI  168


>ref|WP_008293009.1| adenylyl cyclase [Congregibacter litoralis]
 gb|EAQ98184.1| putative integral membrane protein [Congregibacter litoralis 
KT71]
Length=741

 Score = 40.8 bits (94),  Expect = 0.65, Method: Compositional matrix adjust.
 Identities = 19/38 (50%), Positives = 27/38 (71%), Gaps = 0/38 (0%)

Query  37   QQSLPYLNQALILSPSSAPIHAQRAVAYTLLENWPRAY  74
            Q++L  LNQA+ + P+ AP +A RA AYTLL + P +Y
Sbjct  308  QRALELLNQAIEIDPTYAPAYASRAKAYTLLSDRPGSY  345


>ref|WP_050752366.1| hypothetical protein [Scardovia inopinata]
 gb|EFG26947.2| hypothetical protein HMPREF9020_00577 [Scardovia inopinata F0304]
 dbj|BAR06553.1| conserved hypothetical protein [Scardovia inopinata JCM 12537]
Length=1271

 Score = 39.7 bits (91),  Expect = 1.8, Method: Compositional matrix adjust.
 Identities = 14/44 (32%), Positives = 30/44 (68%), Gaps = 0/44 (0%)

Query  31    YLVGSPQQSLPYLNQALILSPSSAPIHAQRAVAYTLLENWPRAY  74
             + +G P++++ +LN+A+  +PS   +H ++AV   L+E+W  A+
Sbjct  1081  FTMGKPKEAVKHLNKAVSYAPSYTMVHLRQAVGLALIEDWDSAF  1124


>gb|EEH07821.1| DnaJ and TPR domain-containing protein [Histoplasma capsulatum 
G186AR]
Length=525

 Score = 39.3 bits (90),  Expect = 1.8, Method: Compositional matrix adjust.
 Identities = 22/59 (37%), Positives = 31/59 (53%), Gaps = 1/59 (2%)

Query  19  VPLEELLTTASTYLV-GSPQQSLPYLNQALILSPSSAPIHAQRAVAYTLLENWPRAYFD  76
            PL  L++TA +YL  G PQ +LPY + A+   P++     QR  AY  L    +A  D
Sbjct  30  TPLSSLISTAKSYLKNGVPQDALPYFDAAISRDPTNYLTIFQRGAAYLSLGKNTKALLD  88


>gb|EER42779.1| DnaJ and TPR domain-containing protein [Histoplasma capsulatum 
H143]
 gb|EGC42854.1| DnaJ and TPR domain-containing protein [Histoplasma capsulatum 
H88]
Length=525

 Score = 39.3 bits (90),  Expect = 1.8, Method: Compositional matrix adjust.
 Identities = 22/59 (37%), Positives = 31/59 (53%), Gaps = 1/59 (2%)

Query  19  VPLEELLTTASTYLV-GSPQQSLPYLNQALILSPSSAPIHAQRAVAYTLLENWPRAYFD  76
            PL  L++TA +YL  G PQ +LPY + A+   P++     QR  AY  L    +A  D
Sbjct  30  TPLSSLISTAKSYLKNGVPQDALPYFDAAISRDPTNYLTIFQRGAAYLSLGKNTKALLD  88


>gb|KNC95962.1| hypothetical protein SPPG_08567 [Spizellomyces punctatus DAOM 
BR117]
Length=473

 Score = 39.3 bits (90),  Expect = 1.9, Method: Compositional matrix adjust.
 Identities = 40/157 (25%), Positives = 74/157 (47%), Gaps = 12/157 (8%)

Query  2    TTISADLSALSIKDLGSVPLEELLTTASTYLVGSPQQSLPYLNQALILSPSSAPIHAQRA  61
            ++ SA +SA   KD G   + E     + +  G  ++++   ++++ L P+SA +   RA
Sbjct  113  SSKSAKVSA-DQKDFGERAILEKEKGNAWFKKGDYKRAILCYSKSMQLDPTSAVLPVNRA  171

Query  62   VAYTLLENWPRAYFDACQGLDDDAEGLTDELRAELIGLA----GKCAAERMIWDLAHL--  115
            +AY  L+ +  A  D  QG++ D + +    R    G+A    GK    R   + A +  
Sbjct  172  LAYLKLDRFAEAEADCTQGIEMDPKNVKALWRR---GIARAKLGKAEEARQDLEAAVVLE  228

Query  116  --EEAIGLTRTSALLALQAKALSNLQKTSVSPVRTRM  150
               ++I    +  L +++ KA  N +K    PVR R+
Sbjct  229  PTNKSIKADLSDVLTSIKEKATKNDRKNPTRPVRRRL  265


>ref|XP_006884633.1| PREDICTED: suppressor of G2 allele of SKP1 homolog [Elephantulus 
edwardii]
Length=333

 Score = 38.9 bits (89),  Expect = 2.1, Method: Compositional matrix adjust.
 Identities = 38/124 (31%), Positives = 51/124 (41%), Gaps = 7/124 (6%)

Query  35   SPQQSLPYLNQALILSPSSAPIHAQRAVAYTLLENWPRAYFDACQGLDDDAEGLTDELRA  94
            +PQ +L  LNQAL   P  A  + QRA  + LL N+  A  DA + L  +    T  LR 
Sbjct  26   NPQAALEELNQALEQKPDDAQYYCQRAYCHILLGNYCDAAVDAKKSLRLNPNNSTALLRK  85

Query  95   ELIGLAGKCAAERMIWDLAHLEEAIGLTRTSALLALQAKALSNLQKTSVSPVRTRMLNQR  154
             +    GK      +  L    E   L  T+    +  K     Q  S S VR +   Q 
Sbjct  86   GICEYHGK----NYVAALETFTEGQKLDGTNDNFIVWIKRCQEAQNGSESEVRAQ---QS  138

Query  155  KTKF  158
            K K+
Sbjct  139  KIKY  142


>gb|KNG46503.1| cysteine synthase 2 [Stemphylium lycopersici]
Length=640

 Score = 39.3 bits (90),  Expect = 2.1, Method: Compositional matrix adjust.
 Identities = 22/71 (31%), Positives = 34/71 (48%), Gaps = 10/71 (14%)

Query  57   HAQRAVAYTLLENWPRAYFDACQGLDDDA-----EGLTDELR--AELIGLAGKCAAERMI  109
            +++    Y ++ NW   + D C+GL DD      EGL  ++R  A  IG+ G   AE   
Sbjct  547  YSEHTFQYPMIMNWAHPFLDRCEGLPDDCRLDDVEGLRVKMRMFARFIGMRG---AETPT  603

Query  110  WDLAHLEEAIG  120
            W+     E +G
Sbjct  604  WEYERQMEILG  614


>gb|EGE86291.1| DnaJ like subfamily C member 3 [Blastomyces dermatitidis ATCC 
18188]
Length=525

 Score = 38.9 bits (89),  Expect = 2.7, Method: Compositional matrix adjust.
 Identities = 21/59 (36%), Positives = 31/59 (53%), Gaps = 1/59 (2%)

Query  19  VPLEELLTTASTYLV-GSPQQSLPYLNQALILSPSSAPIHAQRAVAYTLLENWPRAYFD  76
            PL  L++TA +YL  G+PQ +LPY + A+   P++     QR   Y  L    +A  D
Sbjct  30  TPLSSLISTAKSYLKNGAPQDALPYFDVAISRDPTNYLTIFQRGATYLSLGKNAKALLD  88


>ref|XP_002620698.1| DnaJ domain-containing protein [Blastomyces dermatitidis SLH14081]
 gb|EEQ75763.1| DnaJ domain-containing protein [Blastomyces dermatitidis SLH14081]
 gb|EEQ90235.1| DnaJ domain-containing protein [Blastomyces dermatitidis ER-3]
 gb|EQL28933.1| DnaJ like subfamily C member 3 [Blastomyces dermatitidis ATCC 
26199]
 gb|EQL28934.1| DnaJ like subfamily C member 3, variant [Blastomyces dermatitidis 
ATCC 26199]
Length=525

 Score = 38.9 bits (89),  Expect = 2.7, Method: Compositional matrix adjust.
 Identities = 21/59 (36%), Positives = 31/59 (53%), Gaps = 1/59 (2%)

Query  19  VPLEELLTTASTYLV-GSPQQSLPYLNQALILSPSSAPIHAQRAVAYTLLENWPRAYFD  76
            PL  L++TA +YL  G+PQ +LPY + A+   P++     QR   Y  L    +A  D
Sbjct  30  TPLSSLISTAKSYLKNGAPQDALPYFDVAISRDPTNYLTIFQRGATYLSLGKNAKALLD  88


>ref|WP_009025162.1| adenylyl cyclase [gamma proteobacterium NOR5-3]
 gb|EED30404.1| TPR repeat-containing protein [gamma proteobacterium NOR5-3]
Length=740

 Score = 38.5 bits (88),  Expect = 3.5, Method: Compositional matrix adjust.
 Identities = 18/38 (47%), Positives = 27/38 (71%), Gaps = 0/38 (0%)

Query  37   QQSLPYLNQALILSPSSAPIHAQRAVAYTLLENWPRAY  74
            Q++L  L+QA+ + P+ AP +A RA AYTLL + P +Y
Sbjct  308  QRALELLDQAIEIDPTYAPAYASRAKAYTLLSDRPGSY  345


>ref|XP_006176337.1| PREDICTED: suppressor of G2 allele of SKP1 homolog isoform X2 
[Camelus ferus]
Length=333

 Score = 38.1 bits (87),  Expect = 3.7, Method: Compositional matrix adjust.
 Identities = 38/123 (31%), Positives = 51/123 (41%), Gaps = 7/123 (6%)

Query  36   PQQSLPYLNQALILSPSSAPIHAQRAVAYTLLENWPRAYFDACQGLDDDAEGLTDELRAE  95
            PQ +L  L +AL   P  A  + QRA  + LL N+P A  DA + L+ +    T  LR  
Sbjct  27   PQAALEELTKALEQKPDDAQYYCQRAYCHILLGNYPDAVADAKKSLELNPNSSTAMLRKG  86

Query  96   LIGLAGKCAAERMIWDLAHLEEAIGLTRTSALLALQAKALSNLQKTSVSPVRTRMLNQRK  155
            +     K  A      L    E   L    A   +  K     Q+ S S VRT   +Q K
Sbjct  87   ICEYHEKNYAVA----LETFTEGQKLDGADADFVVWIKRCQEAQEGSQSEVRT---HQSK  139

Query  156  TKF  158
             K+
Sbjct  140  IKY  142


>gb|KLJ13223.1| DnaJ like subfamily C member 3 [Emmonsia parva UAMH 139]
Length=526

 Score = 38.5 bits (88),  Expect = 3.8, Method: Compositional matrix adjust.
 Identities = 21/59 (36%), Positives = 31/59 (53%), Gaps = 1/59 (2%)

Query  19  VPLEELLTTASTYLV-GSPQQSLPYLNQALILSPSSAPIHAQRAVAYTLLENWPRAYFD  76
            PL  L++TA +YL  G+PQ +LPY + A+   P++     QR   Y  L    +A  D
Sbjct  30  TPLSSLVSTAKSYLKNGAPQDALPYFDVAISRDPTNYLTIFQRGATYLSLGKNAKALLD  88


>gb|EPS26558.1| hypothetical protein PDE_01495 [Penicillium oxalicum 114-2]
Length=526

 Score = 38.5 bits (88),  Expect = 3.8, Method: Compositional matrix adjust.
 Identities = 30/92 (33%), Positives = 46/92 (50%), Gaps = 7/92 (8%)

Query  3   TISADLSALS----IKDLGSVPLEELLTTASTYLV-GSPQQSLPYLNQALILSPSSAPIH  57
           T++A L+ LS    ++     PL EL+ +A  +L  GSP+ +LPY + A+   PS+    
Sbjct  7   TVTAVLACLSTGSALQIPADTPLSELIASAKAHLAKGSPRDALPYFDAAVSRDPSNYITL  66

Query  58  AQRAVAYTLLENWPRAY--FDACQGLDDDAEG  87
            QR  AY  +    +A   F+    L  D EG
Sbjct  67  FQRGAAYLSIGKNSQASEDFNRVLKLKPDFEG  98


>ref|XP_006215630.1| PREDICTED: suppressor of G2 allele of SKP1 homolog isoform X2 
[Vicugna pacos]
Length=333

 Score = 38.1 bits (87),  Expect = 3.9, Method: Compositional matrix adjust.
 Identities = 38/123 (31%), Positives = 50/123 (41%), Gaps = 7/123 (6%)

Query  36   PQQSLPYLNQALILSPSSAPIHAQRAVAYTLLENWPRAYFDACQGLDDDAEGLTDELRAE  95
            PQ +L  L +AL   P  A  + QRA  + LL N+P A  DA + L+ +    T  LR  
Sbjct  27   PQAALEELTKALEQKPDDAQYYCQRAYCHILLGNYPDAVADAKKSLELNPNSSTAMLRKG  86

Query  96   LIGLAGKCAAERMIWDLAHLEEAIGLTRTSALLALQAKALSNLQKTSVSPVRTRMLNQRK  155
            +     K  A      L    E   L    A   +  K     Q  S S VRT   +Q K
Sbjct  87   ICEYHEKNYAAA----LETFTEGQKLDGADADFVVWIKRCQEAQDGSQSEVRT---HQSK  139

Query  156  TKF  158
             K+
Sbjct  140  VKY  142


>gb|EIE91049.1| hypothetical protein RO3G_15760 [Rhizopus delemar RA 99-880]
Length=513

 Score = 38.1 bits (87),  Expect = 3.9, Method: Compositional matrix adjust.
 Identities = 27/98 (28%), Positives = 48/98 (49%), Gaps = 12/98 (12%)

Query  22   EELLTTAS-TYLVGSPQQSLPYLNQALILSPSSAPIHAQRAVAYTLLENWPRAYFDACQG  80
            EE+   A+  Y +G  ++++   +QA+  SP ++  +  RA AY + + +  A FD+   
Sbjct  15   EEIKNLANEQYKLGRYEEAIKLYSQAIDASPKTSTFYNNRAAAYLMQKKYKEATFDSRTA  74

Query  81   LDDDAEGLTDELRAELIGLAGKCAAERMIWDLAHLEEA  118
            L+       D   A+    AGKC       ++ +LEEA
Sbjct  75   LE------LDPTNAKAYARAGKCQ-----LNMGNLEEA  101


>gb|EPY86814.1| fermitin family 3 short form-like protein [Camelus ferus]
Length=1265

 Score = 37.7 bits (86),  Expect = 6.9, Method: Compositional matrix adjust.
 Identities = 24/77 (31%), Positives = 42/77 (55%), Gaps = 2/77 (3%)

Query  8    LSALSIKDLGSV-PLEELLTTASTYL-VGSPQQSLPYLNQALILSPSSAPIHAQRAVAYT  65
            L  LSIK  G+  P+ EL    +  L  G+   +L   ++A+ L P +  +++ R+ AY 
Sbjct  42   LHGLSIKSGGAAGPVNELKEKGNKALSAGNIDDALQCYSEAIKLDPQNHVLYSNRSAAYA  101

Query  66   LLENWPRAYFDACQGLD  82
               ++ +AY DAC+ +D
Sbjct  102  KKGDYQKAYEDACKTVD  118


>ref|XP_002789233.1| DnaJ domain-containing protein [Paracoccidioides sp. 'lutzii' 
Pb01]
 gb|EEH39567.1| DnaJ domain-containing protein [Paracoccidioides sp. 'lutzii' 
Pb01]
Length=524

 Score = 37.4 bits (85),  Expect = 7.2, Method: Compositional matrix adjust.
 Identities = 30/91 (33%), Positives = 45/91 (49%), Gaps = 5/91 (5%)

Query  1    MTTISADLSALSIKDLGS-VPLEELLTTASTYLV-GSPQQSLPYLNQALILSPSSAPIHA  58
            +  ISA  + L   D+ S  PL  L++TA T+L  G+PQ +LPY + A+   P++     
Sbjct  12   LAVISA-TNGLQSSDIPSDTPLSSLISTAKTHLKNGAPQDALPYFDAAISRDPANYLTIF  70

Query  59   QRAVAYTLLENWPRAY--FDACQGLDDDAEG  87
            QR   Y  L    +A   F+    +  D EG
Sbjct  71   QRGATYLSLGKSAKALQDFNEVLKIKPDFEG  101


>ref|XP_008614527.1| hypothetical protein SDRG_10321 [Saprolegnia diclina VS20]
 gb|EQC32125.1| hypothetical protein SDRG_10321 [Saprolegnia diclina VS20]
Length=432

 Score = 37.4 bits (85),  Expect = 7.7, Method: Compositional matrix adjust.
 Identities = 20/78 (26%), Positives = 39/78 (50%), Gaps = 0/78 (0%)

Query  4    ISADLSALSIKDLGSVPLEELLTTASTYLVGSPQQSLPYLNQALILSPSSAPIHAQRAVA  63
            +S DL  L++ +     L E       + +G   +++ Y +++L   P+SA +HA RA+ 
Sbjct  208  LSVDLDVLTLTEREVYALHEKTKGNECFKLGENDEAILYYSRSLAYDPTSAIVHANRALT  267

Query  64   YTLLENWPRAYFDACQGL  81
            +  L+N+  A  D    +
Sbjct  268  HLRLKNFASAEDDCSSAI  285


>gb|EEH23396.1| hypothetical protein PABG_05607 [Paracoccidioides brasiliensis 
Pb03]
Length=524

 Score = 37.4 bits (85),  Expect = 7.7, Method: Compositional matrix adjust.
 Identities = 30/91 (33%), Positives = 45/91 (49%), Gaps = 5/91 (5%)

Query  1    MTTISADLSALSIKDLGS-VPLEELLTTASTYLV-GSPQQSLPYLNQALILSPSSAPIHA  58
            +  ISA  + L   D+ S  PL  L++TA T+L  G+PQ +LPY + A+   P++     
Sbjct  12   LAVISA-TNGLQSSDIPSDTPLSSLISTAKTHLKNGAPQDALPYFDAAISRDPANYLTIF  70

Query  59   QRAVAYTLLENWPRAY--FDACQGLDDDAEG  87
            QR   Y  L    +A   F+    +  D EG
Sbjct  71   QRGATYLSLGKSAKALQDFNEVLKIKPDFEG  101


>ref|XP_010761479.1| hypothetical protein PADG_05928 [Paracoccidioides brasiliensis 
Pb18]
 gb|EEH49849.1| hypothetical protein PADG_05928 [Paracoccidioides brasiliensis 
Pb18]
Length=523

 Score = 37.4 bits (85),  Expect = 8.3, Method: Compositional matrix adjust.
 Identities = 30/91 (33%), Positives = 45/91 (49%), Gaps = 5/91 (5%)

Query  1    MTTISADLSALSIKDLGS-VPLEELLTTASTYLV-GSPQQSLPYLNQALILSPSSAPIHA  58
            +  ISA  + L   D+ S  PL  L++TA T+L  G+PQ +LPY + A+   P++     
Sbjct  12   LAVISA-TNGLQSSDIPSDTPLSSLISTAKTHLKNGAPQDALPYFDAAISRDPANYLTIF  70

Query  59   QRAVAYTLLENWPRAY--FDACQGLDDDAEG  87
            QR   Y  L    +A   F+    +  D EG
Sbjct  71   QRGATYLSLGKSAKALQDFNEVLKIKPDFEG  101


>ref|XP_009016225.1| hypothetical protein HELRODRAFT_185497 [Helobdella robusta]
 gb|ESO05592.1| hypothetical protein HELRODRAFT_185497 [Helobdella robusta]
Length=391

 Score = 37.0 bits (84),  Expect = 9.4, Method: Compositional matrix adjust.
 Identities = 29/74 (39%), Positives = 39/74 (53%), Gaps = 9/74 (12%)

Query  28   ASTYL-VGSPQQSLPYLNQALILSPSSAPIHAQRAVAYTLLENWPRAYFD---ACQ-GLD  82
            AS Y+ +  P  ++   N+AL L+P SA  +  R  AY LL  W  AY D   +CQ   D
Sbjct  159  ASIYVKLLKPNAAIRDCNKALELNPDSAQPYKWRGKAYRLLGEWEHAYQDLNKSCQLDYD  218

Query  83   DDAEGLTDELRAEL  96
            DD    T+E R E+
Sbjct  219  DD----TNEARREV  228


>ref|WP_007147424.1| hypothetical protein [Scardovia wiggsiae]
 gb|EJD65592.1| hypothetical protein HMPREF9156_00356 [Scardovia wiggsiae F0424]
Length=1255

 Score = 37.4 bits (85),  Expect = 9.4, Method: Compositional matrix adjust.
 Identities = 15/44 (34%), Positives = 29/44 (66%), Gaps = 0/44 (0%)

Query  31    YLVGSPQQSLPYLNQALILSPSSAPIHAQRAVAYTLLENWPRAY  74
             +L+G P+++  +LN+A+  +PS    H ++AV   L+E+W  A+
Sbjct  1065  FLMGQPRKAAEHLNKAVSYAPSYTMAHLRQAVGLALVEDWDSAF  1108


>gb|KOO33536.1| ion channel [Chrysochromulina sp. CCMP291]
Length=699

 Score = 37.4 bits (85),  Expect = 9.5, Method: Compositional matrix adjust.
 Identities = 29/84 (35%), Positives = 43/84 (51%), Gaps = 9/84 (11%)

Query  21   LEELLTTASTYLVGSPQQSLPYLNQALILSPSSAPIHAQRAVAYTLLENWPRAYFDACQG  80
            L+EL T  + Y  G   ++L   +QA+   PS+  ++  RA  + +L  + RA  D C  
Sbjct  359  LQELGT--ARYKAGDYAEALKLYSQAIGTEPSNGALYGNRAACWMMLSKFERAVTD-C--  413

Query  81   LDDDAEGLTDELRAELIGLAGKCA  104
                AEGL  E +AEL  L G+ A
Sbjct  414  ----AEGLRYEKQAELGKLRGRQA  433


Lambda      K        H        a         alpha
   0.316    0.132    0.377    0.792     4.96 

Gapped
Lambda      K        H        a         alpha    sigma
   0.267   0.0410    0.140     1.90     42.6     43.6 

Effective search space used: 826456729644


  Database: nr
    Posted date:  Sep 23, 2015 12:05 AM
  Number of letters in database: 26,053,659,533
  Number of sequences in database:  71,551,133


Matrix: BLOSUM62
Gap Penalties: Existence: 11, Extension: 1
Neighboring words threshold: 11
Window for multiple hits: 40
```
