# Supplementary material for: Comparative genomics provides new insights into the diversity, physiology, and sexuality of the only industrially exploited tremellomycete: Phaffia rhodozyma
Source: BMC Genomics. 2016 Nov 9;17:901. doi: 10.1186/s12864-016-3244-7 (PMC5103461; doi:10.1186/s12864-016-3244-7)
Supplement: Additional file 6: — List of orphan genes with links to PFAM (related to Additional file 1: Table S1). (ZIP 1428 kb) [file 12864_2016_3244_MOESM6_ESM.zip › BLAST_HTML_FTR/G02136_P.html]

BLAST Search Results


```
BLASTP 2.2.27+


Reference:
Stephen F. Altschul, Thomas L. Madden, Alejandro A. Schäffer,
Jinghui Zhang, Zheng Zhang, Webb Miller, and David J. Lipman (1997),
"Gapped BLAST and PSI-BLAST: a new generation of protein database
search programs", Nucleic Acids Res. 25:3389-3402.


Reference for
composition-based statistics:
Alejandro A. Schäffer, L. Aravind, Thomas L. Madden, Sergei
Shavirin, John L. Spouge, Yuri I. Wolf, Eugene V. Koonin, and
Stephen F. Altschul (2001), "Improving the accuracy of PSI-BLAST
protein database searches with composition-based statistics and
other refinements", Nucleic Acids Res. 29:2994-3005.


Database: nr
           71,551,133 sequences; 26,053,659,533 total letters


Query= G02136_P

Length=299
                                                                      Score     E
Sequences producing significant alignments:                          (Bits)  Value

emb|CED82138.1|  hypothetical protein [Xanthophyllomyces dendrorh...   580    0.0  
ref|XP_007413583.1|  hypothetical protein MELLADRAFT_78668 [Melam...  40.8    1.4  


 >emb|CED82138.1| hypothetical protein [Xanthophyllomyces dendrorhous]
Length=298

 Score =  580 bits (1496),  Expect = 0.0, Method: Compositional matrix adjust.
 Identities = 298/298 (100%), Positives = 298/298 (100%), Gaps = 0/298 (0%)

Query  1    MPISVCRGDSFKSDLLSDARPIVIPILPSRVVRFVDPFSSSDEEAEQSSEDDDRQGFLYK  60
            MPISVCRGDSFKSDLLSDARPIVIPILPSRVVRFVDPFSSSDEEAEQSSEDDDRQGFLYK
Sbjct  1    MPISVCRGDSFKSDLLSDARPIVIPILPSRVVRFVDPFSSSDEEAEQSSEDDDRQGFLYK  60

Query  61   HQVDSSSAHTHRKHRSRPKSDHSSRPSIEISLGDLLRRLDRMGYEPTSPSNSPPRSSRSS  120
            HQVDSSSAHTHRKHRSRPKSDHSSRPSIEISLGDLLRRLDRMGYEPTSPSNSPPRSSRSS
Sbjct  61   HQVDSSSAHTHRKHRSRPKSDHSSRPSIEISLGDLLRRLDRMGYEPTSPSNSPPRSSRSS  120

Query  121  FSSTSSTNPSSSSSVLSLSSGPSGSPSTSYPPTILHSPYLPGPLPVTGSDDPSTFGPSHT  180
            FSSTSSTNPSSSSSVLSLSSGPSGSPSTSYPPTILHSPYLPGPLPVTGSDDPSTFGPSHT
Sbjct  121  FSSTSSTNPSSSSSVLSLSSGPSGSPSTSYPPTILHSPYLPGPLPVTGSDDPSTFGPSHT  180

Query  181  APRARSTSPMSVASVDPISSHVVPATSFPQETTSSTVLHPNKNRPKARSMSISILPVLHE  240
            APRARSTSPMSVASVDPISSHVVPATSFPQETTSSTVLHPNKNRPKARSMSISILPVLHE
Sbjct  181  APRARSTSPMSVASVDPISSHVVPATSFPQETTSSTVLHPNKNRPKARSMSISILPVLHE  240

Query  241  HVDERPDSRSFTLLTSGGRPIDRNSSDRDDWQTDRKRKRRCSAEGRLEVLKEEREEAL  298
            HVDERPDSRSFTLLTSGGRPIDRNSSDRDDWQTDRKRKRRCSAEGRLEVLKEEREEAL
Sbjct  241  HVDERPDSRSFTLLTSGGRPIDRNSSDRDDWQTDRKRKRRCSAEGRLEVLKEEREEAL  298


>ref|XP_007413583.1| hypothetical protein MELLADRAFT_78668 [Melampsora larici-populina 
98AG31]
 gb|EGG03123.1| hypothetical protein MELLADRAFT_78668 [Melampsora larici-populina 
98AG31]
Length=388

 Score = 40.8 bits (94),  Expect = 1.4, Method: Compositional matrix adjust.
 Identities = 51/190 (27%), Positives = 86/190 (45%), Gaps = 16/190 (8%)

Query  107  TSPSNSPPRSSRSSFSSTSSTNPSSSSSVLSLSSGPSGSPSTSYPPTILHSPYLPGPLPV  166
            +SPS++ P  + + FSSTS   P + +S++S ++G   S S S        P+       
Sbjct  24   SSPSSTRPHLNPNLFSSTSQEEPLTMNSMISTTTGYHPSSSQSLDHQAQRIPWSSSHEDG  83

Query  167  TGSDDPSTFGPSHTA---PRARSTSPMSVASVDPISSHVVPATSFPQETTSSTVLHPNK-  222
            +   +    G SHT    PR+RS+SP    + DP       A S P  +T++ + H ++ 
Sbjct  84   SNEREEPIAGSSHTHWNPPRSRSSSP---HNQDP------QADSNPLHSTTTEINHEDEL  134

Query  223  NRPKARSMSISILPVLHEHVDERPDSRSFTLLTSGGRPIDRNSSDRDDWQTDRKRKRRCS  282
            N    + ++ +     ++HV     S + T     G  +DRNS   D W+ +RK   +  
Sbjct  135  NSKIDKRLAEASTLAWNDHVTGNEGSDACTNGNGNGESVDRNS---DQWKQNRKNNHKEV  191

Query  283  AEGRLEVLKE  292
               R E + E
Sbjct  192  ERRRRETINE  201


Lambda      K        H        a         alpha
   0.311    0.127    0.365    0.792     4.96 

Gapped
Lambda      K        H        a         alpha    sigma
   0.267   0.0410    0.140     1.90     42.6     43.6 

Effective search space used: 2308881107400


  Database: nr
    Posted date:  Sep 23, 2015 12:05 AM
  Number of letters in database: 26,053,659,533
  Number of sequences in database:  71,551,133


Matrix: BLOSUM62
Gap Penalties: Existence: 11, Extension: 1
Neighboring words threshold: 11
Window for multiple hits: 40
```
