# Supplementary material for: Comparative genomics provides new insights into the diversity, physiology, and sexuality of the only industrially exploited tremellomycete: Phaffia rhodozyma
Source: BMC Genomics. 2016 Nov 9;17:901. doi: 10.1186/s12864-016-3244-7 (PMC5103461; doi:10.1186/s12864-016-3244-7)
Supplement: Additional file 6: — List of orphan genes with links to PFAM (related to Additional file 1: Table S1). (ZIP 1428 kb) [file 12864_2016_3244_MOESM6_ESM.zip › BLAST_HTML_FTR/G03359_P.html]

BLAST Search Results


```
BLASTP 2.2.27+


Reference:
Stephen F. Altschul, Thomas L. Madden, Alejandro A. Schäffer,
Jinghui Zhang, Zheng Zhang, Webb Miller, and David J. Lipman (1997),
"Gapped BLAST and PSI-BLAST: a new generation of protein database
search programs", Nucleic Acids Res. 25:3389-3402.


Reference for
composition-based statistics:
Alejandro A. Schäffer, L. Aravind, Thomas L. Madden, Sergei
Shavirin, John L. Spouge, Yuri I. Wolf, Eugene V. Koonin, and
Stephen F. Altschul (2001), "Improving the accuracy of PSI-BLAST
protein database searches with composition-based statistics and
other refinements", Nucleic Acids Res. 29:2994-3005.


Database: nr
           71,551,133 sequences; 26,053,659,533 total letters


Query= G03359_P

Length=837
                                                                      Score     E
Sequences producing significant alignments:                          (Bits)  Value

emb|CED82471.1|  Zn(2)-C6 fungal-type DNA-binding domain [Xanthop...  1183    0.0  


 >emb|CED82471.1| Zn(2)-C6 fungal-type DNA-binding domain [Xanthophyllomyces dendrorhous]
Length=850

 Score = 1183 bits (3060),  Expect = 0.0, Method: Compositional matrix adjust.
 Identities = 695/700 (99%), Positives = 696/700 (99%), Gaps = 3/700 (0%)

Query  121  HYYPLYQDNQSENKAIPSSGRTDYTGRQQPNSGPTYIRSKDWSPLIASTAWADQSGSIES  180
            H+ P   DNQSENKAIPSSGRTDYTGRQQPNSGPTYIRSKDWSPLIASTAWADQSGSIES
Sbjct  75   HFTP---DNQSENKAIPSSGRTDYTGRQQPNSGPTYIRSKDWSPLIASTAWADQSGSIES  131

Query  181  ALLAQQPSVDSTFLVDQGEALDGISIWGEEFTENRYENPAVGPSRPVHLFHVFTPSSSSS  240
            ALLAQQPSVDSTFLVDQGEALDGISIWGEEFTENRYENPAVGPSRPVHLFHVFTPSSSSS
Sbjct  132  ALLAQQPSVDSTFLVDQGEALDGISIWGEEFTENRYENPAVGPSRPVHLFHVFTPSSSSS  191

Query  241  SSSSSAYRFPFRRQSHNTSSFVNSNLIPTPISSTSSSSTSSVNNQLNSSAHDQQAMSAPF  300
            SSSSSAYRFPFRRQSHNTSSFVNSNLIPTPISSTSSSSTSSVNNQLNSSAHDQQAMSAPF
Sbjct  192  SSSSSAYRFPFRRQSHNTSSFVNSNLIPTPISSTSSSSTSSVNNQLNSSAHDQQAMSAPF  251

Query  301  VATGPFEETTFNRYQQMFNEKQASERRFNIASSSSAVPSTGAPKDYVTTGPSVDLSIAPS  360
            VATGPFEETTFNRYQQMFNEKQASERRFNIASSSSAVPSTGAPKDYVTTGPSVDLSIAPS
Sbjct  252  VATGPFEETTFNRYQQMFNEKQASERRFNIASSSSAVPSTGAPKDYVTTGPSVDLSIAPS  311

Query  361  VFSPSSSTPAVHSERRASFHHYPQQQQQQQQQQQNHQRRLSITDVKLWPHQASSALFSHH  420
            VFSPSSSTPAVHSERRASFHHYPQQQQQQQQQQQNHQRRLSITDVKLWPHQASSALFSHH
Sbjct  312  VFSPSSSTPAVHSERRASFHHYPQQQQQQQQQQQNHQRRLSITDVKLWPHQASSALFSHH  371

Query  421  FPTGPSETSGSDVSRFPYGHNPPASGFSSTLSSSPSTSSLPSSLGLTPKTSSTLSTVQEG  480
            FPTGPSETSGSDVSRFPYGHNPPASGFSSTLSSSPSTSSLPSSLGLTPKTSSTLSTVQEG
Sbjct  372  FPTGPSETSGSDVSRFPYGHNPPASGFSSTLSSSPSTSSLPSSLGLTPKTSSTLSTVQEG  431

Query  481  CLPVQDISGSWTTESSLVPSSDLNTDSNNHLKSIQSAQSGYWMNNPSLMYAPLPSGSPAH  540
            CLPVQDISGSWTTESSLVPSSDLNTDSNNHLKSIQSAQSGYWMNNPSLMYAPLPSGSPAH
Sbjct  432  CLPVQDISGSWTTESSLVPSSDLNTDSNNHLKSIQSAQSGYWMNNPSLMYAPLPSGSPAH  491

Query  541  QQTPLGVTHSSSSSSSSSSIPAQSFLGPSSSSSSSPYSANTAPFRSSNDYASHPIGLQIT  600
            QQTPLGVTHSSSSSSSSSSIPAQSFLGPSSSSSSSPYSANTAPFRSSNDYASHPIGLQIT
Sbjct  492  QQTPLGVTHSSSSSSSSSSIPAQSFLGPSSSSSSSPYSANTAPFRSSNDYASHPIGLQIT  551

Query  601  QEQSRLLLLGQQRLQAQQKCFTPEAPESSSSSASASSLTAVGSPFASDHRRSSETHGLPL  660
            QEQSRLLLLGQQRLQAQQKCFTPEAPESSSSSASASSLTAVGSPFASDHRRSSETHGLPL
Sbjct  552  QEQSRLLLLGQQRLQAQQKCFTPEAPESSSSSASASSLTAVGSPFASDHRRSSETHGLPL  611

Query  661  MTTAALAAATIEPRSFQVGSYPPTTSVGLTNLSMMNASLPTDLPNELSRSSSFEQTGEDS  720
            MTTAALAAATIEPRSFQVGSYPPTTSVGLTNLSMMNASLPTDLPNELSRSSSFEQTGEDS
Sbjct  612  MTTAALAAATIEPRSFQVGSYPPTTSVGLTNLSMMNASLPTDLPNELSRSSSFEQTGEDS  671

Query  721  HQAFNWTATSSSGSSSMTYNEKDPTWAVVRATKVELHEIGSGSEKGLTAKRKETNHSTKG  780
            HQAFNWTATSSSGSSSMTYNEKDPTWAVVRATKVELHEIGSGSEKGLTAKRKETNHSTKG
Sbjct  672  HQAFNWTATSSSGSSSMTYNEKDPTWAVVRATKVELHEIGSGSEKGLTAKRKETNHSTKG  731

Query  781  GSTSIDRKCSKKKTISRSSATSVAESGNDLKEPEASEQVD  820
            GSTSIDRKCSKKKTISRSSATSVAESGNDLKEPEASEQVD
Sbjct  732  GSTSIDRKCSKKKTISRSSATSVAESGNDLKEPEASEQVD  771


Lambda      K        H        a         alpha
   0.307    0.119    0.341    0.792     4.96 

Gapped
Lambda      K        H        a         alpha    sigma
   0.267   0.0410    0.140     1.90     42.6     43.6 

Effective search space used: 9887908777281


  Database: nr
    Posted date:  Sep 23, 2015 12:05 AM
  Number of letters in database: 26,053,659,533
  Number of sequences in database:  71,551,133


Matrix: BLOSUM62
Gap Penalties: Existence: 11, Extension: 1
Neighboring words threshold: 11
Window for multiple hits: 40
```
