# Supplementary material for: Comparative genomics provides new insights into the diversity, physiology, and sexuality of the only industrially exploited tremellomycete: Phaffia rhodozyma
Source: BMC Genomics. 2016 Nov 9;17:901. doi: 10.1186/s12864-016-3244-7 (PMC5103461; doi:10.1186/s12864-016-3244-7)
Supplement: Additional file 6: — List of orphan genes with links to PFAM (related to Additional file 1: Table S1). (ZIP 1428 kb) [file 12864_2016_3244_MOESM6_ESM.zip › BLAST_HTML_FTR/G04453_P.html]

BLAST Search Results


```
BLASTP 2.2.27+


Reference:
Stephen F. Altschul, Thomas L. Madden, Alejandro A. Schäffer,
Jinghui Zhang, Zheng Zhang, Webb Miller, and David J. Lipman (1997),
"Gapped BLAST and PSI-BLAST: a new generation of protein database
search programs", Nucleic Acids Res. 25:3389-3402.


Reference for
composition-based statistics:
Alejandro A. Schäffer, L. Aravind, Thomas L. Madden, Sergei
Shavirin, John L. Spouge, Yuri I. Wolf, Eugene V. Koonin, and
Stephen F. Altschul (2001), "Improving the accuracy of PSI-BLAST
protein database searches with composition-based statistics and
other refinements", Nucleic Acids Res. 29:2994-3005.


Database: nr
           71,551,133 sequences; 26,053,659,533 total letters


Query= G04453_P

Length=674
                                                                      Score     E
Sequences producing significant alignments:                          (Bits)  Value

emb|CDZ96473.1|  hypothetical protein [Xanthophyllomyces dendrorh...  1316    0.0  
emb|CDZ96936.1|  hypothetical protein [Xanthophyllomyces dendrorh...   187    2e-49
emb|CED84443.1|  hypothetical protein [Xanthophyllomyces dendrorh...   142    4e-32
emb|CED82137.1|  hypothetical protein [Xanthophyllomyces dendrorh...   119    2e-25
emb|CDZ98114.1|  hypothetical protein, partial [Xanthophyllomyces...  58.5    2e-06
gb|KLT45648.1|  hypothetical protein CC85DRAFT_325375 [Trichospor...  51.2    0.003
emb|CDZ97489.1|  hypothetical protein [Xanthophyllomyces dendrorh...  48.9    0.018
ref|XP_007842263.1|  hypothetical protein Moror_78 [Moniliophthor...  47.4    0.054
gb|EUC53667.1|  hypothetical protein RSOL_015180 [Rhizoctonia sol...  46.6    0.098
gb|KEP54147.1|  hypothetical protein V565_021480 [Rhizoctonia sol...  46.6    0.10 
gb|KDQ12168.1|  hypothetical protein BOTBODRAFT_425963 [Botryobas...  45.4    0.22 
emb|CUA77612.1|  hypothetical protein RSOLAG22IIIB_02629 [Rhizoct...  45.4    0.23 
ref|XP_007265774.1|  hypothetical protein FOMMEDRAFT_155294 [Fomi...  45.1    0.25 
ref|XP_011406271.1|  PREDICTED: deleted in malignant brain tumors...  43.5    1.1  
ref|XP_008397172.1|  PREDICTED: nucleolar and spindle-associated ...  40.4    5.6  
ref|XP_008397173.1|  PREDICTED: nucleolar and spindle-associated ...  40.4    5.8  


 >emb|CDZ96473.1| hypothetical protein [Xanthophyllomyces dendrorhous]
Length=660

 Score = 1316 bits (3405),  Expect = 0.0, Method: Compositional matrix adjust.
 Identities = 658/664 (99%), Positives = 660/664 (99%), Gaps = 4/664 (1%)

Query  1    MSLTSNELNSLKRTDVQKLCKTLGIKGANQKTDTLIGLILHHYDTIGEDVASIKTSLNSR  60
            MSLTSNELNSLKRTDVQKLCKTLGIKGANQKTDTLIGLILHHYDTIGEDVASIKTSLNSR
Sbjct  1    MSLTSNELNSLKRTDVQKLCKTLGIKGANQKTDTLIGLILHHYDTIGEDVASIKTSLNSR  60

Query  61   LLRPTASSWAKQASQKGMLQKRAVSPKRIVSRKRSSSYKARPKKEAPKSIKNVLAQRHEK  120
            LLRPTASSWAKQASQKGMLQKRAVSPKRIVSRKRSSSYKARPKKEAPKSIKNVLAQRHEK
Sbjct  61   LLRPTASSWAKQASQKGMLQKRAVSPKRIVSRKRSSSYKARPKKEAPKSIKNVLAQRHEK  120

Query  121  PALIESTTAWNEATSDRKADGDEALKDEVMRAEAGSPDKNIKVATTELTSTSNPTSTSKP  180
            PALIESTTAWNEATSDRKADGDEALKDEVMRAEA    +NIKVATTELTSTSNPTSTSKP
Sbjct  121  PALIESTTAWNEATSDRKADGDEALKDEVMRAEA----ENIKVATTELTSTSNPTSTSKP  176

Query  181  NEVDITSLLFPSSSACTCMNSTVMLSSQPLVDPRSVWDALTELTAKIHSLPTKEESDLDM  240
            NEVDITSLLFPSSSACTCMNSTVMLSSQPLVDPRSVWDALTELTAKIHSLPTKEESDLDM
Sbjct  177  NEVDITSLLFPSSSACTCMNSTVMLSSQPLVDPRSVWDALTELTAKIHSLPTKEESDLDM  236

Query  241  QRFYDALERTDVAIKSVSSRIDVSDRMRNLTEQESVKKGDVEAMVNRLIDSRLEQIEQSV  300
            QRFYDALERTDVAIKSVSSRIDVSDRMRNLTEQESVKKGDVEAMVNRLIDSRLEQIEQSV
Sbjct  237  QRFYDALERTDVAIKSVSSRIDVSDRMRNLTEQESVKKGDVEAMVNRLIDSRLEQIEQSV  296

Query  301  ACRLKAMEDKLTAQIFEKHAPQVETMVLSDLSMGLGGQRHQISSKRAWDTVGFPALISSV  360
            ACRLKAMEDKLTAQIFEKHAPQVETMVLSDLSMGLGGQRHQISSKRAWDTVGFPALISSV
Sbjct  297  ACRLKAMEDKLTAQIFEKHAPQVETMVLSDLSMGLGGQRHQISSKRAWDTVGFPALISSV  356

Query  361  SIANDFTVDRETLITSTPSSMASFKSSFPLFSPPVFSVATALDDHRPKRSKIDSLVTGGM  420
            SIANDFTVDRETLITSTPSSMASFK+SFPLFSPPVFSVATALDDHRPKRSKIDSLVTGGM
Sbjct  357  SIANDFTVDRETLITSTPSSMASFKTSFPLFSPPVFSVATALDDHRPKRSKIDSLVTGGM  416

Query  421  AANTVGGLLKDLPERTISRDDTSLISSATQSSIASPFAPVIAPTIDTSAPVASTLIAGVT  480
            AANTVGGLLKDLPERTISRDDTSLISSATQSSIASPFAPVIAPTIDTSAPVASTLIAGVT
Sbjct  417  AANTVGGLLKDLPERTISRDDTSLISSATQSSIASPFAPVIAPTIDTSAPVASTLIAGVT  476

Query  481  SPQVCRTPLLVTGQSARSIAPLPLAFRPGSLKPKDNNHYSRTAPSSRQPTAASALKCTAG  540
            SPQVCRTPLLVTGQSARSIAPLPLAFRPGSLKPKDNNHYSRTAPSSRQPTAASALKCTAG
Sbjct  477  SPQVCRTPLLVTGQSARSIAPLPLAFRPGSLKPKDNNHYSRTAPSSRQPTAASALKCTAG  536

Query  541  SQRATGVKDGQPSDTISYPSFSPFERQTVQSLLPPAPAGAPLWISPMASPRPSYRYRPKA  600
            SQRATGVKDGQPSDTISYPSFSPFERQTVQSLLPPAPAGAPLWISPMASPRPSYRYRPKA
Sbjct  537  SQRATGVKDGQPSDTISYPSFSPFERQTVQSLLPPAPAGAPLWISPMASPRPSYRYRPKA  596

Query  601  KQGPTKEGVTPLPLSATGAHNARLGLNQNIEIAPPTPAGPKTMFGTEKETFGRMFGDEMT  660
            KQGPTKEGVTPLPLSATGAHNARLGLNQNIEIAPPTPAGPKTMFGTEKETFGRMFGDEMT
Sbjct  597  KQGPTKEGVTPLPLSATGAHNARLGLNQNIEIAPPTPAGPKTMFGTEKETFGRMFGDEMT  656

Query  661  PWMS  664
            PWMS
Sbjct  657  PWMS  660


>emb|CDZ96936.1| hypothetical protein [Xanthophyllomyces dendrorhous]
Length=356

 Score =  187 bits (476),  Expect = 2e-49, Method: Compositional matrix adjust.
 Identities = 139/378 (37%), Positives = 196/378 (52%), Gaps = 87/378 (23%)

Query  1    MSLTSNELNSLKRTDVQKLCKTLGIKGANQKTDTLIGLILHHY-----------------  43
            MS TS EL+ LKR DVQKLCKTLGIKGANQKT+ LIGLIL HY                 
Sbjct  1    MSPTSIELHGLKRVDVQKLCKTLGIKGANQKTEALIGLILQHYANSQSTTSEGEQAPPAE  60

Query  44   ---------DTIGEDVASIK------------------------TSL--------NSRLL  62
                       +GE +AS +                        +SL        NSRLL
Sbjct  61   LSSVESGSSGAVGEGIASAQSRSTPSHPAYRPLISMTGRKTSSNSSLHVSRNGKSNSRLL  120

Query  63   RPTASSWAKQASQKGMLQKRAVSPKRIVSRKRSSSYKARPKKEAPKSIKNVLAQRHEKPA  122
            +PTASS AK+ +            K+ +S+K S++  ARPKKEA + ++  L +  EKP 
Sbjct  121  QPTASSRAKKMT------------KQTISQKPSTTCNARPKKEAGQPVEATLPEDQEKPM  168

Query  123  LIESTTAWNEATSDRKADGDEALKDEVMRAEAGSPDKNI-------KVATTELTSTSNPT  175
             +E+         D +A+GDEA++D  +  +A  PD  I       ++AT E T +    
Sbjct  169  PVEAEADEYGEEPDDRANGDEAMEDRTVPPKAARPDVLISEDKLETEIATVEPTPSPRLP  228

Query  176  STSKPNEVDITSLLFPSSSACTCMNSTVMLSSQPLVDPRSVWDALTELTAKIHSLPTKEE  235
            STS+P E         ++SAC    S+++  S  L D  SV  +L ELTA++  LPT+EE
Sbjct  229  STSEPVES------IEAASACVYTRSSLVQPSDLLTDLLSVRTSLAELTARVDDLPTREE  282

Query  236  SDLDMQRFYDALERTDVAIKSVSSRIDVSDRMRNLTEQESVKKGDVEAMVNRLIDSRLEQ  295
            ++L+ +  YDAL  TD  ++ + + I V  RM +  E+ SVKK D+EAMV+++++SRL+ 
Sbjct  283  TELNKRSVYDALACTDATVEFIGAEIMVFGRMSSRAERLSVKKEDIEAMVDQMVESRLKL  342

Query  296  IEQSVACRLKAMEDKLTA  313
            IE     RLK +E   T 
Sbjct  343  IE----SRLKLIEQSRTG  356


>emb|CED84443.1| hypothetical protein [Xanthophyllomyces dendrorhous]
Length=674

 Score =  142 bits (359),  Expect = 4e-32, Method: Compositional matrix adjust.
 Identities = 73/134 (54%), Positives = 84/134 (63%), Gaps = 8/134 (6%)

Query  538  TAGSQRATGVKDGQPSDTISYPSFSPFERQTVQSLLPPAPAGAPLWISPMASPRPSYRYR  597
            T+ S   TGV   QP D  S+P FSP +RQTVQSLLPP P   P W+SPMA  R  +R  
Sbjct  540  TSASLTPTGVCLNQPGDRFSHPPFSPSDRQTVQSLLPPVPVDGPFWMSPMAPSRQLHRPT  599

Query  598  PKAKQ--------GPTKEGVTPLPLSATGAHNARLGLNQNIEIAPPTPAGPKTMFGTEKE  649
              +KQ        G  ++   P  L  TG HN +LG  Q+ EIAP TP GPKTMFGTEKE
Sbjct  600  SGSKQETPKRATKGAAQKVAAPFTLIPTGTHNIQLGPGQDNEIAPLTPPGPKTMFGTEKE  659

Query  650  TFGRMFGDEMTPWM  663
            TFGR+F DEMTPWM
Sbjct  660  TFGRLFSDEMTPWM  673


>emb|CED82137.1| hypothetical protein [Xanthophyllomyces dendrorhous]
Length=429

 Score =  119 bits (299),  Expect = 2e-25, Method: Compositional matrix adjust.
 Identities = 79/203 (39%), Positives = 101/203 (50%), Gaps = 20/203 (10%)

Query  464  TIDTSAPVASTLIAGVTSP-QVCRTPLLVTGQSARSIAPLPLAFRPGSLKPKDNNHYSRT  522
            T+D SAPV+STL  G + P +   TP     + AR + PLP A   G  +  + N +   
Sbjct  234  TVDMSAPVSSTLTGGRSYPDRQASTPTSSAAEPARPVIPLPSASHLGLTQTTNGNDW---  290

Query  523  APSSRQPTAASALKCTAGSQRATGVKDGQPSDTISYPSFSPFERQTVQSLLPPAPAGAPL  582
                   T  +  +    S    G  + QP D  S+P FSP +RQTVQSLLPP P   P 
Sbjct  291  -------TPLTNPQVIVASTPDHGPVN-QPGDRFSHPPFSPSDRQTVQSLLPPVPVDGPF  342

Query  583  WISPMASPRPSYRYRPKAKQ--------GPTKEGVTPLPLSATGAHNARLGLNQNIEIAP  634
            W+SPMA  R  +R    +KQ        G  ++  TP  L   G HN +LG  QN EIAP
Sbjct  343  WMSPMAPSRQLHRPTSGSKQETPKRATKGAAQKVATPFTLIPMGTHNIQLGPGQNNEIAP  402

Query  635  PTPAGPKTMFGTEKETFGRMFGD  657
              P G K +FGTEKE   R+  D
Sbjct  403  SMPPGSKKIFGTEKENIARLLSD  425


 Score =  112 bits (280),  Expect = 5e-23, Method: Compositional matrix adjust.
 Identities = 75/178 (42%), Positives = 106/178 (60%), Gaps = 15/178 (8%)

Query  218  DALTELTAKIHSLPTKEESDLDMQRFYDALERTDVAIKSVSSRIDVSDRMRNLTEQESVK  277
            DAL ELTA++  LPT+EE++ + +  YDAL  TD  ++ + + I V  RM +  E+ SVK
Sbjct  90   DALAELTARVDDLPTREETEPNKRSVYDALACTDATVEFIGAEIMVFGRMSSRAERLSVK  149

Query  278  KGDVEAMVN-------RLIDSRLEQIEQSVACRLKAMEDKLTAQIFEKH--APQVETMVL  328
            K D+EAMV+       +LI+SRL+ IEQS A RLK +ED+L A++ +    A Q E M +
Sbjct  150  KEDIEAMVDQMVESRLKLIESRLKLIEQSFASRLKTVEDRLEARLCQSQTCASQPEAMKV  209

Query  329  SDLSMGLGGQRHQIS---SKRAWDTVGFPALISSVSIANDFTVDRETLITSTPSSMAS  383
            SDLS+ L  Q  Q+     KRA +TV   A +SS         DR+    STP+S A+
Sbjct  210  SDLSLDLERQSEQVEQVLGKRARNTVDMSAPVSSTLTGGRSYPDRQ---ASTPTSSAA  264


 Score = 69.7 bits (169),  Expect = 4e-09, Method: Compositional matrix adjust.
 Identities = 39/75 (52%), Positives = 47/75 (63%), Gaps = 2/75 (3%)

Query  1   MSLTSNELNSLKRTDVQKLCKTLGIKGANQKTDTLIGLILHHYDTIGEDVASIKTSLNSR  60
           M+ TS EL+ LKR DVQKLCKTLGIKGANQKT  LI  IL HY  +   V++  + L S 
Sbjct  1   MTPTSTELHKLKRADVQKLCKTLGIKGANQKTKNLIDRILQHYTDVEPSVST--SGLQSS  58

Query  61  LLRPTASSWAKQASQ  75
              P+  +    ASQ
Sbjct  59  ASLPSEENSLSNASQ  73


>emb|CDZ98114.1| hypothetical protein, partial [Xanthophyllomyces dendrorhous]
Length=136

 Score = 58.5 bits (140),  Expect = 2e-06, Method: Compositional matrix adjust.
 Identities = 38/101 (38%), Positives = 58/101 (57%), Gaps = 12/101 (12%)

Query  58   NSRLLRPTASSWAKQASQKGMLQKRAVSPKRIVSRKRSSSYKARPKKEAPKSIKNVLAQR  117
            NSRLL+PTASS AK+ +            K+ +S+K S++  ARPKKEA + ++  L + 
Sbjct  21   NSRLLQPTASSRAKKMT------------KQTISQKPSTTCNARPKKEAGQPVEATLPED  68

Query  118  HEKPALIESTTAWNEATSDRKADGDEALKDEVMRAEAGSPD  158
             EKP  +E+         D +A+GDEA++D  +  +A  PD
Sbjct  69   QEKPMPVEAEADEYGEEPDDRANGDEAMEDRTVPPKAARPD  109


>gb|KLT45648.1| hypothetical protein CC85DRAFT_325375 [Trichosporon oleaginosus]
Length=580

 Score = 51.2 bits (121),  Expect = 0.003, Method: Compositional matrix adjust.
 Identities = 20/42 (48%), Positives = 29/42 (69%), Gaps = 0/42 (0%)

Query  2   SLTSNELNSLKRTDVQKLCKTLGIKGANQKTDTLIGLILHHY  43
           + T +EL  L+R D+Q+LCKT  IKGAN K+D LI  +  ++
Sbjct  6   AYTRDELQGLRRADLQRLCKTYNIKGANAKSDVLITTLADYF  47


>emb|CDZ97489.1| hypothetical protein [Xanthophyllomyces dendrorhous]
Length=607

 Score = 48.9 bits (115),  Expect = 0.018, Method: Compositional matrix adjust.
 Identities = 22/40 (55%), Positives = 28/40 (70%), Gaps = 0/40 (0%)

Query  4   TSNELNSLKRTDVQKLCKTLGIKGANQKTDTLIGLILHHY  43
           T   L  LKR  +QKLCK+L IKGAN+K+  +I +IL HY
Sbjct  7   TRESLQMLKRVSLQKLCKSLFIKGANKKSVEIIEVILAHY  46


>ref|XP_007842263.1| hypothetical protein Moror_78 [Moniliophthora roreri MCA 2997]
 gb|ESK98328.1| hypothetical protein Moror_78 [Moniliophthora roreri MCA 2997]
Length=641

 Score = 47.4 bits (111),  Expect = 0.054, Method: Compositional matrix adjust.
 Identities = 32/75 (43%), Positives = 42/75 (56%), Gaps = 7/75 (9%)

Query  1   MSLTSNELNSLKRTDVQKLCKTLGIKGANQKTDTLIGLILH----HYDT--IGEDVASIK  54
           M  T ++L SLKR D+QKLCK  G+K AN KT+ LI L+L     H     + E   S+ 
Sbjct  1   MGHTRSQLESLKRADLQKLCKDYGVK-ANLKTEALIELLLETSKPHTRAAPVQESRRSVS  59

Query  55  TSLNSRLLRPTASSW  69
           T ++SR   P  SS 
Sbjct  60  TRVSSRNTAPRISSM  74


>gb|EUC53667.1| hypothetical protein RSOL_015180 [Rhizoctonia solani AG-3 Rhs1AP]
Length=630

 Score = 46.6 bits (109),  Expect = 0.098, Method: Compositional matrix adjust.
 Identities = 23/40 (58%), Positives = 26/40 (65%), Gaps = 1/40 (3%)

Query  4   TSNELNSLKRTDVQKLCKTLGIKGANQKTDTLIGLILHHY  43
           T  EL  LKR  +Q  CK LGIK AN K++ LI LIL HY
Sbjct  5   TRVELEGLKRAALQSKCKELGIK-ANSKSEMLIDLILEHY  43


>gb|KEP54147.1| hypothetical protein V565_021480 [Rhizoctonia solani 123E]
Length=632

 Score = 46.6 bits (109),  Expect = 0.10, Method: Compositional matrix adjust.
 Identities = 23/40 (58%), Positives = 26/40 (65%), Gaps = 1/40 (3%)

Query  4   TSNELNSLKRTDVQKLCKTLGIKGANQKTDTLIGLILHHY  43
           T  EL  LKR  +Q  CK LGIK AN K++ LI LIL HY
Sbjct  5   TRVELEGLKRAALQSKCKELGIK-ANSKSEMLIDLILEHY  43


>gb|KDQ12168.1| hypothetical protein BOTBODRAFT_425963 [Botryobasidium botryosum 
FD-172 SS1]
Length=840

 Score = 45.4 bits (106),  Expect = 0.22, Method: Compositional matrix adjust.
 Identities = 24/53 (45%), Positives = 34/53 (64%), Gaps = 1/53 (2%)

Query  6   NELNSLKRTDVQKLCKTLGIKGANQKTDTLIGLILHHYDTIGEDVASIKTSLN  58
           ++L SLKR ++Q+L K   IK AN KTDTL+ L+L  +   GE  A++  S N
Sbjct  4   SKLESLKRAELQRLAKEHNIK-ANLKTDTLVDLLLKAFADAGELSATVSHSAN  55


>emb|CUA77612.1| hypothetical protein RSOLAG22IIIB_02629 [Rhizoctonia solani]
Length=687

 Score = 45.4 bits (106),  Expect = 0.23, Method: Compositional matrix adjust.
 Identities = 21/42 (50%), Positives = 28/42 (67%), Gaps = 1/42 (2%)

Query  4   TSNELNSLKRTDVQKLCKTLGIKGANQKTDTLIGLILHHYDT  45
           T  +L  LKR+ +Q  CK LGIK AN K++ LI L+L HY +
Sbjct  5   TRAQLEGLKRSALQAKCKELGIK-ANSKSEVLIELVLEHYQS  45


>ref|XP_007265774.1| hypothetical protein FOMMEDRAFT_155294 [Fomitiporia mediterranea 
MF3/22]
 gb|EJD04173.1| hypothetical protein FOMMEDRAFT_155294 [Fomitiporia mediterranea 
MF3/22]
Length=683

 Score = 45.1 bits (105),  Expect = 0.25, Method: Compositional matrix adjust.
 Identities = 21/38 (55%), Positives = 28/38 (74%), Gaps = 1/38 (3%)

Query  3   LTSNELNSLKRTDVQKLCKTLGIKGANQKTDTLIGLIL  40
            T  +L SLKR+D+QKLCK  G++ AN KT+ LI L+L
Sbjct  8   FTREDLVSLKRSDIQKLCKEYGVR-ANMKTEALIELLL  44


>ref|XP_011406271.1| PREDICTED: deleted in malignant brain tumors 1 protein-like [Amphimedon 
queenslandica]
Length=3189

 Score = 43.5 bits (101),  Expect = 1.1, Method: Composition-based stats.
 Identities = 43/162 (27%), Positives = 68/162 (42%), Gaps = 21/162 (13%)

Query  446   SSATQSSIASPFAPVIAPTIDTSAPVASTLIAGVTSPQVCRTPLLVTGQSARSIAPLPLA  505
             S  T +S A+ F+     T+ TSA   +T+     +  +  T L+ T  +A+S  P P  
Sbjct  2679  SIPTSTSAAAGFSTSSLATMTTSAVSPTTIAPSTVTSGITSTLLVTTTSAAQSSTPQP--  2736

Query  506   FRPGSLKPKDNNHYSRTAP---SSRQPTAASALKCTAGSQRATGVKDGQPSDTISYPSFS  562
                 S  P  ++    + P   S++QP++      TA  Q +T V+   P      PS +
Sbjct  2737  ----STTPVQSSTAQSSTPLQSSTQQPSSTPVQSSTA--QSSTPVQSSTPQ-----PSST  2785

Query  563   PFERQTVQSLLP-----PAPAGAPLWISPMASPRPSYRYRPK  599
             P +  T QS  P     P P+  P+  S   S  P     P+
Sbjct  2786  PVQSSTAQSSTPVQSSTPQPSSTPVQSSTAQSSTPVQSSTPQ  2827


>ref|XP_008397172.1| PREDICTED: nucleolar and spindle-associated protein 1 isoform 
X1 [Poecilia reticulata]
Length=434

 Score = 40.4 bits (93),  Expect = 5.6, Method: Compositional matrix adjust.
 Identities = 28/88 (32%), Positives = 46/88 (52%), Gaps = 6/88 (7%)

Query  7   ELNSLKRTDVQKLCKTLGIKGANQKTDTLIGLILHHYDTIGEDVASIKTSLNSRLLRPTA  66
           +L+S+K  +++ + K LG+KG N KTD L+  I  HY+   E+    +     R++   A
Sbjct  4   DLDSMKYAELRSIAKELGVKG-NMKTDKLLKAIKQHYEKEKENDEEAQV----RVVEXAA  58

Query  67  SSWAKQASQKGMLQKRAVSPKRIVSRKR  94
           S    Q S+    Q+ A SP   V+ +R
Sbjct  59  SDRG-QESKDDASQQEASSPAVFVNTRR  85


>ref|XP_008397173.1| PREDICTED: nucleolar and spindle-associated protein 1 isoform 
X2 [Poecilia reticulata]
Length=433

 Score = 40.4 bits (93),  Expect = 5.8, Method: Compositional matrix adjust.
 Identities = 28/88 (32%), Positives = 46/88 (52%), Gaps = 6/88 (7%)

Query  7   ELNSLKRTDVQKLCKTLGIKGANQKTDTLIGLILHHYDTIGEDVASIKTSLNSRLLRPTA  66
           +L+S+K  +++ + K LG+KG N KTD L+  I  HY+   E+    +     R++   A
Sbjct  4   DLDSMKYAELRSIAKELGVKG-NMKTDKLLKAIKQHYEKEKENDEEAQV----RVVEXAA  58

Query  67  SSWAKQASQKGMLQKRAVSPKRIVSRKR  94
           S    Q S+    Q+ A SP   V+ +R
Sbjct  59  SDRG-QESKDDASQQEASSPAVFVNTRR  85


Lambda      K        H        a         alpha
   0.312    0.126    0.355    0.792     4.96 

Gapped
Lambda      K        H        a         alpha    sigma
   0.267   0.0410    0.140     1.90     42.6     43.6 

Effective search space used: 7610267547804


  Database: nr
    Posted date:  Sep 23, 2015 12:05 AM
  Number of letters in database: 26,053,659,533
  Number of sequences in database:  71,551,133


Matrix: BLOSUM62
Gap Penalties: Existence: 11, Extension: 1
Neighboring words threshold: 11
Window for multiple hits: 40
```
