# Supplementary material for: Comparative genomics provides new insights into the diversity, physiology, and sexuality of the only industrially exploited tremellomycete: Phaffia rhodozyma
Source: BMC Genomics. 2016 Nov 9;17:901. doi: 10.1186/s12864-016-3244-7 (PMC5103461; doi:10.1186/s12864-016-3244-7)
Supplement: Additional file 6: — List of orphan genes with links to PFAM (related to Additional file 1: Table S1). (ZIP 1428 kb) [file 12864_2016_3244_MOESM6_ESM.zip › BLAST_HTML_FTR/G04279_P.html]

BLAST Search Results


```
BLASTP 2.2.27+


Reference:
Stephen F. Altschul, Thomas L. Madden, Alejandro A. Schäffer,
Jinghui Zhang, Zheng Zhang, Webb Miller, and David J. Lipman (1997),
"Gapped BLAST and PSI-BLAST: a new generation of protein database
search programs", Nucleic Acids Res. 25:3389-3402.


Reference for
composition-based statistics:
Alejandro A. Schäffer, L. Aravind, Thomas L. Madden, Sergei
Shavirin, John L. Spouge, Yuri I. Wolf, Eugene V. Koonin, and
Stephen F. Altschul (2001), "Improving the accuracy of PSI-BLAST
protein database searches with composition-based statistics and
other refinements", Nucleic Acids Res. 29:2994-3005.


Database: nr
           71,551,133 sequences; 26,053,659,533 total letters


Query= G04279_P

Length=289
                                                                      Score     E
Sequences producing significant alignments:                          (Bits)  Value

emb|CDZ97873.1|  hypothetical protein [Xanthophyllomyces dendrorh...   536    0.0  
ref|WP_025422424.1|  ATP-dependent helicase [Sodalis praecaptivus...  43.1    0.30 
ref|WP_050061463.1|  hypothetical protein [Acidobacteriaceae bact...  40.0    2.2  
ref|WP_023438375.1|  metallo beta-lactamase superfamily hydrolase...  39.7    3.0  
ref|XP_006696106.1|  putative peroxisome biosynthesis protein [Ch...  39.7    3.7  
ref|XP_011295998.1|  PREDICTED: dystonin isoform X23 [Musca domes...  39.7    4.2  
ref|XP_011295997.1|  PREDICTED: dystonin isoform X22 [Musca domes...  39.7    4.3  
ref|XP_013108704.1|  PREDICTED: dystonin isoform X38 [Stomoxys ca...  38.9    6.9  
ref|XP_013108702.1|  PREDICTED: microtubule-actin cross-linking f...  38.9    7.5  
ref|XP_005191832.1|  PREDICTED: uncharacterized protein LOC101893...  38.9    7.5  
ref|WP_019664586.1|  hypothetical protein [Rhodococcus sp. 29MFTs...  38.5    7.5  
ref|XP_013108703.1|  PREDICTED: microtubule-actin cross-linking f...  38.9    7.5  
ref|XP_011295992.1|  PREDICTED: uncharacterized protein LOC101893...  38.9    7.6  
ref|XP_011295993.1|  PREDICTED: uncharacterized protein LOC101893...  38.9    7.6  
ref|XP_011295995.1|  PREDICTED: uncharacterized protein LOC101893...  38.9    7.7  
ref|XP_011295986.1|  PREDICTED: uncharacterized protein LOC101893...  38.9    7.7  


 >emb|CDZ97873.1| hypothetical protein [Xanthophyllomyces dendrorhous]
Length=293

 Score =  536 bits (1380),  Expect = 0.0, Method: Compositional matrix adjust.
 Identities = 287/293 (98%), Positives = 288/293 (98%), Gaps = 5/293 (2%)

Query  1    MPNSGLFRYSHDHDDPDQLMIHSKQEVIDILVQPSNRFHAKAVLESYAQHKLSINNKVSL  60
            MPNSGLFRYSHDHDDPDQLMIHSKQEVIDILVQPSNRFHAKAVLESYAQHKLSINNKVSL
Sbjct  1    MPNSGLFRYSHDHDDPDQLMIHSKQEVIDILVQPSNRFHAKAVLESYAQHKLSINNKVSL  60

Query  61   SNKLHTIEQQLVAHNAPNYAVE-----WNLSLRPSRVYGHPSNLVLDPIVRSSSSIYSSH  115
            SNKLHTIEQQLVAHNAPNYAVE     WNLSLRPSRVYG+PSNLVLDPIVRSSSSIYSSH
Sbjct  61   SNKLHTIEQQLVAHNAPNYAVEFISLRWNLSLRPSRVYGNPSNLVLDPIVRSSSSIYSSH  120

Query  116  SSPNVSYIEAQMSSDNMPTFMDGPSSSSSLPSPSSSSFRPTRTISTAPTPKALSPPRKSS  175
            SSPNVSYIEAQMSSDNMPTFMDGPSSSSSLPSPSSSSFRPTRTISTAPTPKALSPPRKSS
Sbjct  121  SSPNVSYIEAQMSSDNMPTFMDGPSSSSSLPSPSSSSFRPTRTISTAPTPKALSPPRKSS  180

Query  176  DPKRSTSKDRPFWLTAVEQAHRSNCEHLNSCANSLSGFSMISADSWEEESRALAQAQQHF  235
            DPKRSTSKDRPFWLTAVEQAHRSNCEHLNSCANSLSGFSMISADSWEEESRALAQAQQHF
Sbjct  181  DPKRSTSKDRPFWLTAVEQAHRSNCEHLNSCANSLSGFSMISADSWEEESRALAQAQQHF  240

Query  236  ETEGLIGRQYAESVEPDGNHGRSRGDSYQVAVGEEWDRQWLEKNAPDDLKLRK  288
            ETEGLIGRQYAESVEPDGNHGRSRGDSYQVAVGEEWDRQWLEKNAPDDLKLRK
Sbjct  241  ETEGLIGRQYAESVEPDGNHGRSRGDSYQVAVGEEWDRQWLEKNAPDDLKLRK  293


>ref|WP_025422424.1| ATP-dependent helicase [Sodalis praecaptivus]
 gb|AHF77290.1| DEAD/DEAH box helicase [Sodalis praecaptivus]
Length=1633

 Score = 43.1 bits (100),  Expect = 0.30, Method: Composition-based stats.
 Identities = 31/99 (31%), Positives = 46/99 (46%), Gaps = 6/99 (6%)

Query  149   SSSSFRPTRTISTAPTPKALSPPRKSSDPKRSTSKDRPFWLTAVEQAHRSNCEHLNSCAN  208
             SS S+ P R ++ A TP    P R S  P+R      P W  A  QA RS        A 
Sbjct  1357  SSDSWLPLRALTGAHTPSRARPTRASRHPRRQVPGWVPVWTPASAQAARSPWG-----AA  1411

Query  209   SLSG-FSMISADSWEEESRALAQAQQHFETEGLIGRQYA  246
             +L+G +S+++A+      R L   +  F+  G++ R  A
Sbjct  1412  ALTGRWSLLAAEPVNSTLRLLTWVESLFDRYGVVSRGVA  1450


>ref|WP_050061463.1| hypothetical protein [Acidobacteriaceae bacterium S15]
Length=353

 Score = 40.0 bits (92),  Expect = 2.2, Method: Compositional matrix adjust.
 Identities = 38/146 (26%), Positives = 66/146 (45%), Gaps = 13/146 (9%)

Query  140  SSSSSLPSPSSSSFRPTRTISTAPTPKALSPPRKSSDPKRSTSKDRPFWLTAVEQAHRSN  199
            S ++  PS S+ S RP R    AP P+++  PR+S+DP+R      P     ++      
Sbjct  44   SRTNHAPSRSAPSRRPERQREAAPRPRSVKDPRESTDPRRPQRNKIPSQTIGMQLRPEER  103

Query  200  CEHLNSCANSLSGFSMIS----ADS-WEEESRALAQAQQHFETEGLIGRQYAESVEPDGN  254
               L      L  F ++     ADS ++ + R L +   +  ++GL+  ++  ++  DG 
Sbjct  104  KVMLE-----LGKFRVVRTRDLADSVYDGKQRKLGEDLNYLRSKGLVETRHI-NLRRDGT  157

Query  255  HGRSRGDSYQVAVGEEWDRQWLEKNA  280
              R + D  +VA      R WL K+ 
Sbjct  158  --RRQIDRAEVATLTRDGRAWLRKSG  181


>ref|WP_023438375.1| metallo beta-lactamase superfamily hydrolase [Clostridium tetani]
 emb|CDI49585.1| metallo beta-lactamase superfamily hydrolase [Clostridium tetani 
12124569]
Length=454

 Score = 39.7 bits (91),  Expect = 3.0, Method: Compositional matrix adjust.
 Identities = 35/132 (27%), Positives = 51/132 (39%), Gaps = 13/132 (10%)

Query  77   PNYAVEWNLSLRPSRVYGHPSNLVLDPIVRSSSSIYSSHSSPNVSYIEAQMSSDNMPTFM  136
            P YAV   +S      YGHP+   L+ +      +Y +    NV       S  N  TF 
Sbjct  328  PKYAV---ISCGKGNKYGHPTQETLNKLKDKGIKVYRTDECSNV----IATSDGNNITFN  380

Query  137  DGPSSSSSLPSPSSSSFRPTRTISTAPTPKALSPPRKSSDPKRSTSKDRPFWLTA-----  191
              P S   + + + S+ +PT  +   P PKA   P     P     K +P   T      
Sbjct  381  TKPGSYKGVDN-TKSTRKPTSKVQPKPVPKATPKPTAKPQPTLPVVKSQPNSKTVHITNT  439

Query  192  VEQAHRSNCEHL  203
             ++ HR+ C  L
Sbjct  440  GKKYHRNGCRSL  451


>ref|XP_006696106.1| putative peroxisome biosynthesis protein [Chaetomium thermophilum 
var. thermophilum DSM 1495]
 gb|EGS19161.1| putative peroxisome biosynthesis protein [Chaetomium thermophilum 
var. thermophilum DSM 1495]
Length=1503

 Score = 39.7 bits (91),  Expect = 3.7, Method: Compositional matrix adjust.
 Identities = 29/87 (33%), Positives = 40/87 (46%), Gaps = 5/87 (6%)

Query  77   PNYAVEWNLSLRPSRVYGHPSNLVLDPIVRSSSSIYSSHSSPNVSYIEAQMSSDNMPTFM  136
            P+  V W    RP+RVYG P      P+ R  SS YSS     +S+ EAQ+   + P   
Sbjct  458  PDEEVAW----RPARVYGLPEAYASRPMARVPSSKYSSDGR-RMSFFEAQVQKPSSPVVY  512

Query  137  DGPSSSSSLPSPSSSSFRPTRTISTAP  163
              P   ++L SP      P +  + AP
Sbjct  513  LSPIMLANLDSPPYLRLSPIKRPAQAP  539


>ref|XP_011295998.1| PREDICTED: dystonin isoform X23 [Musca domestica]
Length=5205

 Score = 39.7 bits (91),  Expect = 4.2, Method: Compositional matrix adjust.
 Identities = 19/43 (44%), Positives = 25/43 (58%), Gaps = 0/43 (0%)

Query  133   PTFMDGPSSSSSLPSPSSSSFRPTRTISTAPTPKALSPPRKSS  175
             PT   G S SSS+P+ +   ++P R IS   TP  +  PRKSS
Sbjct  5135  PTRNGGMSRSSSIPALTGYGYKPRRNISGTSTPSGMQTPRKSS  5177


>ref|XP_011295997.1| PREDICTED: dystonin isoform X22 [Musca domestica]
Length=5208

 Score = 39.7 bits (91),  Expect = 4.3, Method: Compositional matrix adjust.
 Identities = 19/43 (44%), Positives = 25/43 (58%), Gaps = 0/43 (0%)

Query  133   PTFMDGPSSSSSLPSPSSSSFRPTRTISTAPTPKALSPPRKSS  175
             PT   G S SSS+P+ +   ++P R IS   TP  +  PRKSS
Sbjct  5138  PTRNGGMSRSSSIPALTGYGYKPRRNISGTSTPSGMQTPRKSS  5180


>ref|XP_013108704.1| PREDICTED: dystonin isoform X38 [Stomoxys calcitrans]
Length=5175

 Score = 38.9 bits (89),  Expect = 6.9, Method: Compositional matrix adjust.
 Identities = 18/43 (42%), Positives = 25/43 (58%), Gaps = 0/43 (0%)

Query  133   PTFMDGPSSSSSLPSPSSSSFRPTRTISTAPTPKALSPPRKSS  175
             PT   G S SSS+P+ +   ++P R +S   TP  +  PRKSS
Sbjct  5105  PTRNGGMSRSSSIPALTGFGYKPRRNVSGTSTPSGMQTPRKSS  5147


>ref|XP_013108702.1| PREDICTED: microtubule-actin cross-linking factor 1 isoform X36 
[Stomoxys calcitrans]
Length=5205

 Score = 38.9 bits (89),  Expect = 7.5, Method: Compositional matrix adjust.
 Identities = 18/43 (42%), Positives = 25/43 (58%), Gaps = 0/43 (0%)

Query  133   PTFMDGPSSSSSLPSPSSSSFRPTRTISTAPTPKALSPPRKSS  175
             PT   G S SSS+P+ +   ++P R +S   TP  +  PRKSS
Sbjct  5135  PTRNGGMSRSSSIPALTGFGYKPRRNVSGTSTPSGMQTPRKSS  5177


>ref|XP_005191832.1| PREDICTED: uncharacterized protein LOC101893893 isoform X9 [Musca 
domestica]
Length=8847

 Score = 38.9 bits (89),  Expect = 7.5, Method: Compositional matrix adjust.
 Identities = 19/43 (44%), Positives = 25/43 (58%), Gaps = 0/43 (0%)

Query  133   PTFMDGPSSSSSLPSPSSSSFRPTRTISTAPTPKALSPPRKSS  175
             PT   G S SSS+P+ +   ++P R IS   TP  +  PRKSS
Sbjct  8777  PTRNGGMSRSSSIPALTGYGYKPRRNISGTSTPSGMQTPRKSS  8819


>ref|WP_019664586.1| hypothetical protein [Rhodococcus sp. 29MFTsu3.1]
Length=399

 Score = 38.5 bits (88),  Expect = 7.5, Method: Compositional matrix adjust.
 Identities = 25/82 (30%), Positives = 40/82 (49%), Gaps = 3/82 (4%)

Query  59   SLSNKLHTIEQQLVAHNAPNYAVEWNLSLRPSRVYGHPSNLVLDPIVRSSSSI-YSSHSS  117
            S++N L+    QL    A NY +E   SL+PS ++GH +N  L P++     I     + 
Sbjct  76   SVNNGLYVHTSQLTVEMAVNYWLESKHSLKPSSLHGHRTN--LQPVISELGQIPVQKLTK  133

Query  118  PNVSYIEAQMSSDNMPTFMDGP  139
            PNV  +   + +  +PT    P
Sbjct  134  PNVESLVRNLRNGGLPTKQGRP  155


>ref|XP_013108703.1| PREDICTED: microtubule-actin cross-linking factor 1 isoform X37 
[Stomoxys calcitrans]
Length=5201

 Score = 38.9 bits (89),  Expect = 7.5, Method: Compositional matrix adjust.
 Identities = 18/43 (42%), Positives = 25/43 (58%), Gaps = 0/43 (0%)

Query  133   PTFMDGPSSSSSLPSPSSSSFRPTRTISTAPTPKALSPPRKSS  175
             PT   G S SSS+P+ +   ++P R +S   TP  +  PRKSS
Sbjct  5131  PTRNGGMSRSSSIPALTGFGYKPRRNVSGTSTPSGMQTPRKSS  5173


>ref|XP_011295992.1| PREDICTED: uncharacterized protein LOC101893893 isoform X16 [Musca 
domestica]
Length=8823

 Score = 38.9 bits (89),  Expect = 7.6, Method: Compositional matrix adjust.
 Identities = 19/43 (44%), Positives = 25/43 (58%), Gaps = 0/43 (0%)

Query  133   PTFMDGPSSSSSLPSPSSSSFRPTRTISTAPTPKALSPPRKSS  175
             PT   G S SSS+P+ +   ++P R IS   TP  +  PRKSS
Sbjct  8753  PTRNGGMSRSSSIPALTGYGYKPRRNISGTSTPSGMQTPRKSS  8795


>ref|XP_011295993.1| PREDICTED: uncharacterized protein LOC101893893 isoform X17 [Musca 
domestica]
Length=8846

 Score = 38.9 bits (89),  Expect = 7.6, Method: Compositional matrix adjust.
 Identities = 19/43 (44%), Positives = 25/43 (58%), Gaps = 0/43 (0%)

Query  133   PTFMDGPSSSSSLPSPSSSSFRPTRTISTAPTPKALSPPRKSS  175
             PT   G S SSS+P+ +   ++P R IS   TP  +  PRKSS
Sbjct  8776  PTRNGGMSRSSSIPALTGYGYKPRRNISGTSTPSGMQTPRKSS  8818


>ref|XP_011295995.1| PREDICTED: uncharacterized protein LOC101893893 isoform X20 [Musca 
domestica]
Length=8826

 Score = 38.9 bits (89),  Expect = 7.7, Method: Compositional matrix adjust.
 Identities = 19/43 (44%), Positives = 25/43 (58%), Gaps = 0/43 (0%)

Query  133   PTFMDGPSSSSSLPSPSSSSFRPTRTISTAPTPKALSPPRKSS  175
             PT   G S SSS+P+ +   ++P R IS   TP  +  PRKSS
Sbjct  8756  PTRNGGMSRSSSIPALTGYGYKPRRNISGTSTPSGMQTPRKSS  8798


>ref|XP_011295986.1| PREDICTED: uncharacterized protein LOC101893893 isoform X8 [Musca 
domestica]
Length=8850

 Score = 38.9 bits (89),  Expect = 7.7, Method: Compositional matrix adjust.
 Identities = 19/43 (44%), Positives = 25/43 (58%), Gaps = 0/43 (0%)

Query  133   PTFMDGPSSSSSLPSPSSSSFRPTRTISTAPTPKALSPPRKSS  175
             PT   G S SSS+P+ +   ++P R IS   TP  +  PRKSS
Sbjct  8780  PTRNGGMSRSSSIPALTGYGYKPRRNISGTSTPSGMQTPRKSS  8822


Lambda      K        H        a         alpha
   0.312    0.126    0.372    0.792     4.96 

Gapped
Lambda      K        H        a         alpha    sigma
   0.267   0.0410    0.140     1.90     42.6     43.6 

Effective search space used: 2154955700240


  Database: nr
    Posted date:  Sep 23, 2015 12:05 AM
  Number of letters in database: 26,053,659,533
  Number of sequences in database:  71,551,133


Matrix: BLOSUM62
Gap Penalties: Existence: 11, Extension: 1
Neighboring words threshold: 11
Window for multiple hits: 40
```
