# Supplementary material for: Comparative genomics provides new insights into the diversity, physiology, and sexuality of the only industrially exploited tremellomycete: Phaffia rhodozyma
Source: BMC Genomics. 2016 Nov 9;17:901. doi: 10.1186/s12864-016-3244-7 (PMC5103461; doi:10.1186/s12864-016-3244-7)
Supplement: Additional file 6: — List of orphan genes with links to PFAM (related to Additional file 1: Table S1). (ZIP 1428 kb) [file 12864_2016_3244_MOESM6_ESM.zip › BLAST_HTML_FTR/G04110_P.html]

BLAST Search Results


```
BLASTP 2.2.27+


Reference:
Stephen F. Altschul, Thomas L. Madden, Alejandro A. Schäffer,
Jinghui Zhang, Zheng Zhang, Webb Miller, and David J. Lipman (1997),
"Gapped BLAST and PSI-BLAST: a new generation of protein database
search programs", Nucleic Acids Res. 25:3389-3402.


Reference for
composition-based statistics:
Alejandro A. Schäffer, L. Aravind, Thomas L. Madden, Sergei
Shavirin, John L. Spouge, Yuri I. Wolf, Eugene V. Koonin, and
Stephen F. Altschul (2001), "Improving the accuracy of PSI-BLAST
protein database searches with composition-based statistics and
other refinements", Nucleic Acids Res. 29:2994-3005.


Database: nr
           71,551,133 sequences; 26,053,659,533 total letters


Query= G04110_P

Length=851
                                                                      Score     E
Sequences producing significant alignments:                          (Bits)  Value

emb|CDZ97025.1|  hypothetical protein [Xanthophyllomyces dendrorh...  1514    0.0  


 >emb|CDZ97025.1| hypothetical protein [Xanthophyllomyces dendrorhous]
Length=855

 Score = 1514 bits (3921),  Expect = 0.0, Method: Compositional matrix adjust.
 Identities = 849/855 (99%), Positives = 850/855 (99%), Gaps = 5/855 (1%)

Query  1    MPSTLPDQTATAVSAPAPPQLKPMSLPVASISGSSPDGTNFNAKSKPKQARKKPFKSRAR  60
            MPSTLPDQTATAVSAPAPPQLKPMSLPVASISGSSPDGTNFNAKSKPKQARKKPFKSRAR
Sbjct  1    MPSTLPDQTATAVSAPAPPQLKPMSLPVASISGSSPDGTNFNAKSKPKQARKKPFKSRAR  60

Query  61   QLGRRIREGSVDDDEEQGSSSRASRPVLHSDSRSSSLNDQGRLRNTHTDTDDDDDVEVTF  120
            QLGRRIREGSVDDDEEQGSSSRASRPVLHSDSRSSSLNDQGRLRNTHTDTDDDDDVEVTF
Sbjct  61   QLGRRIREGSVDDDEEQGSSSRASRPVLHSDSRSSSLNDQGRLRNTHTDTDDDDDVEVTF  120

Query  121  GAPAGRKELKLGLASSRQNSSTSISKGKGRESPVKTTLDLATAKLPRILQLPSPPSSNTS  180
            GAPAGRKELKLGLASSRQNSSTSISKGKGRESPVKTTLDLATAKLPRILQLPSPPSSNTS
Sbjct  121  GAPAGRKELKLGLASSRQNSSTSISKGKGRESPVKTTLDLATAKLPRILQLPSPPSSNTS  180

Query  181  NGSTSNSAIPILPPPELSSTDTPASKNPKKKRNKKSIAVSSSQTNPLSTSKTSKPLPRPI  240
            NGSTSNSAIPILPPPELSSTDTPASKNPKKKRNKKSIAVSSSQTNPLSTSKTSKPLPRPI
Sbjct  181  NGSTSNSAIPILPPPELSSTDTPASKNPKKKRNKKSIAVSSSQTNPLSTSKTSKPLPRPI  240

Query  241  TSPNDQHSPSEKQNDGLRSYPASRASIDSSLSNINPSTTCITVQSNDPTLVDEIPKKKKK  300
            TSPNDQHSPSEKQNDGLRSYPASRASIDSSLSNINPSTTCITVQSNDPTLVDEIPKKKKK
Sbjct  241  TSPNDQHSPSEKQNDGLRSYPASRASIDSSLSNINPSTTCITVQSNDPTLVDEIPKKKKK  300

Query  301  NSRQKMRERKEREAAAVAAGSHQANAEESASPDVVPKSTVPSHSIKPSTPTSSAKPFVPS  360
            NSRQKMRERKEREAAAVAAGSHQANAEESASPDVVPKSTVPSHSIKPSTPTSSAKPFVPS
Sbjct  301  NSRQKMRERKEREAAAVAAGSHQANAEESASPDVVPKSTVPSHSIKPSTPTSSAKPFVPS  360

Query  361  KPQSKGVANESNAVSTGEPNTGVLEATQNRPPPPVQQLSYTAEKRQQAAELRMRRATEKN  420
            KPQSKGVANESNAVSTGEPNTGVLEATQNRPPPPVQQLSYTAEKRQQAAELRMRRATEKN
Sbjct  361  KPQSKGVANESNAVSTGEPNTGVLEATQNRPPPPVQQLSYTAEKRQQAAELRMRRATEKN  420

Query  421  PPPNRSLSATHTRPAPSVSRAVGLHSGENQVVLKPSEMDTPVPMTKSKNALKRERLERKK  480
            PPPNRSLSATHTRPAPSVSRAVGLHSGENQVVLKPSEMDTPVPMTKSKNALKRERLERKK
Sbjct  421  PPPNRSLSATHTRPAPSVSRAVGLHSGENQVVLKPSEMDTPVPMTKSKNALKRERLERKK  480

Query  481  ERLAAASATEDTHTTEKATPKAKAVRKSESRSAVPAGGKNEVESSGLFKEKLPRGKEKGR  540
            ERLAAASATEDTHTTEKATPKAKAVRKSESRSAVPAGGKNEVESSGLFKEKLPRGKEKGR
Sbjct  481  ERLAAASATEDTHTTEKATPKAKAVRKSESRSAVPAGGKNEVESSGLFKEKLPRGKEKGR  540

Query  541  EKEKKKNRMVDVQVVEVVKEEVTEKINEVEFGYMR-----QQQQQQQQQQHESAKATDFV  595
            EKEKKKNRMVDVQVVEVVKEEVTEKINEVEFGYMR     QQQQQQQQQQHESAKATDFV
Sbjct  541  EKEKKKNRMVDVQVVEVVKEEVTEKINEVEFGYMRQQQQQQQQQQQQQQQHESAKATDFV  600

Query  596  ISSRAEPTSPALATLERTILSHPPDHSASPAFDESPRFSSIAGPMPLSFPEAPCRSSGSH  655
            ISSRAEPTSPALATLERTILSHPPDHSASPAFDESPRFSSIAGPMPLSFPEAPCRSSGSH
Sbjct  601  ISSRAEPTSPALATLERTILSHPPDHSASPAFDESPRFSSIAGPMPLSFPEAPCRSSGSH  660

Query  656  IAPATYPQMNASYQQSPSPNMLSNLIVPPGYLLDSGIYYDQQTGQPYIYAYPSQTQPAPF  715
            IAPATYPQ+NASYQQSPSPNMLSNLIVPPGYLLDSGIYYDQQTGQPYIYAYPSQTQPAPF
Sbjct  661  IAPATYPQVNASYQQSPSPNMLSNLIVPPGYLLDSGIYYDQQTGQPYIYAYPSQTQPAPF  720

Query  716  YPSLPTHNASSLQNSSSHQHWAQPRLSMDQTSPMYYPTMFPSNISIPPSGYPPSLNDLQN  775
            YPSLPTHNASSLQNSSSHQHWAQPRLSMDQTSPMYYPTMFPSNISIPPSGYPPSLNDLQN
Sbjct  721  YPSLPTHNASSLQNSSSHQHWAQPRLSMDQTSPMYYPTMFPSNISIPPSGYPPSLNDLQN  780

Query  776  SYAAAQLGNMMAESMAAASYQTSVSPGPSRMSHLPQIQQQPLLSSASSSVAASPLFVPRR  835
            SYAAAQLGNMMAESMAAASYQTSVSPGPSRMSHLPQIQQQPLLSSASSSVAASPLFVPRR
Sbjct  781  SYAAAQLGNMMAESMAAASYQTSVSPGPSRMSHLPQIQQQPLLSSASSSVAASPLFVPRR  840

Query  836  QGSSAVKVRMDDGNC  850
            QGSSAVKVRMDDGNC
Sbjct  841  QGSSAVKVRMDDGNC  855


Lambda      K        H        a         alpha
   0.306    0.121    0.335    0.792     4.96 

Gapped
Lambda      K        H        a         alpha    sigma
   0.267   0.0410    0.140     1.90     42.6     43.6 

Effective search space used: 10092385472823


  Database: nr
    Posted date:  Sep 23, 2015 12:05 AM
  Number of letters in database: 26,053,659,533
  Number of sequences in database:  71,551,133


Matrix: BLOSUM62
Gap Penalties: Existence: 11, Extension: 1
Neighboring words threshold: 11
Window for multiple hits: 40
```
