# Supplementary material for: Comparative genomics provides new insights into the diversity, physiology, and sexuality of the only industrially exploited tremellomycete: Phaffia rhodozyma
Source: BMC Genomics. 2016 Nov 9;17:901. doi: 10.1186/s12864-016-3244-7 (PMC5103461; doi:10.1186/s12864-016-3244-7)
Supplement: Additional file 6: — List of orphan genes with links to PFAM (related to Additional file 1: Table S1). (ZIP 1428 kb) [file 12864_2016_3244_MOESM6_ESM.zip › BLAST_HTML_FTR/G02171_P.html]

BLAST Search Results


```
BLASTP 2.2.27+


Reference:
Stephen F. Altschul, Thomas L. Madden, Alejandro A. Schäffer,
Jinghui Zhang, Zheng Zhang, Webb Miller, and David J. Lipman (1997),
"Gapped BLAST and PSI-BLAST: a new generation of protein database
search programs", Nucleic Acids Res. 25:3389-3402.


Reference for
composition-based statistics:
Alejandro A. Schäffer, L. Aravind, Thomas L. Madden, Sergei
Shavirin, John L. Spouge, Yuri I. Wolf, Eugene V. Koonin, and
Stephen F. Altschul (2001), "Improving the accuracy of PSI-BLAST
protein database searches with composition-based statistics and
other refinements", Nucleic Acids Res. 29:2994-3005.


Database: nr
           71,551,133 sequences; 26,053,659,533 total letters


Query= G02171_P

Length=314
                                                                      Score     E
Sequences producing significant alignments:                          (Bits)  Value

emb|CED85264.1|  hypothetical protein [Xanthophyllomyces dendrorh...   616    0.0  
ref|WP_053325187.1|  MFS transporter [Paenibacillus peoriae] >gb|...  40.8    1.3  
ref|XP_010452377.1|  PREDICTED: cationic amino acid transporter 6...  39.3    5.6  
ref|XP_010423539.1|  PREDICTED: cationic amino acid transporter 6...  39.3    5.9  


 >emb|CED85264.1| hypothetical protein [Xanthophyllomyces dendrorhous]
Length=313

 Score =  616 bits (1588),  Expect = 0.0, Method: Compositional matrix adjust.
 Identities = 313/313 (100%), Positives = 313/313 (100%), Gaps = 0/313 (0%)

Query  1    MAPVSPTAFFSFSSLALSLAQSADPSTNRFVLSATSLLAILLLRARSLLRSLSSSPVQIT  60
            MAPVSPTAFFSFSSLALSLAQSADPSTNRFVLSATSLLAILLLRARSLLRSLSSSPVQIT
Sbjct  1    MAPVSPTAFFSFSSLALSLAQSADPSTNRFVLSATSLLAILLLRARSLLRSLSSSPVQIT  60

Query  61   APSAADASLGFLGLFLWASNPLESSTSPNVIHYGIKSCLVVFVALTLCGLYLEADDDAGR  120
            APSAADASLGFLGLFLWASNPLESSTSPNVIHYGIKSCLVVFVALTLCGLYLEADDDAGR
Sbjct  61   APSAADASLGFLGLFLWASNPLESSTSPNVIHYGIKSCLVVFVALTLCGLYLEADDDAGR  120

Query  121  EDKQCLRAVTEKSPLLPRREMSIGQSSTVDAPIRIDIHNTLLWITLLLTAFIAFSTSSIL  180
            EDKQCLRAVTEKSPLLPRREMSIGQSSTVDAPIRIDIHNTLLWITLLLTAFIAFSTSSIL
Sbjct  121  EDKQCLRAVTEKSPLLPRREMSIGQSSTVDAPIRIDIHNTLLWITLLLTAFIAFSTSSIL  180

Query  181  TWILGASPAMLPLIPAALSITLSLSSITLSRPISAFFSELTLLTSFHAVLSYTIQVSNAP  240
            TWILGASPAMLPLIPAALSITLSLSSITLSRPISAFFSELTLLTSFHAVLSYTIQVSNAP
Sbjct  181  TWILGASPAMLPLIPAALSITLSLSSITLSRPISAFFSELTLLTSFHAVLSYTIQVSNAP  240

Query  241  TCGVWDPLSRPDLKDVVPALCMIAKVLYYASWTTVVMLFYKISKDHLDTISGPDPILSSK  300
            TCGVWDPLSRPDLKDVVPALCMIAKVLYYASWTTVVMLFYKISKDHLDTISGPDPILSSK
Sbjct  241  TCGVWDPLSRPDLKDVVPALCMIAKVLYYASWTTVVMLFYKISKDHLDTISGPDPILSSK  300

Query  301  KTDADVEVALFGI  313
            KTDADVEVALFGI
Sbjct  301  KTDADVEVALFGI  313


>ref|WP_053325187.1| MFS transporter [Paenibacillus peoriae]
 gb|ALA41724.1| MFS transporter [Paenibacillus peoriae]
Length=407

 Score = 40.8 bits (94),  Expect = 1.3, Method: Compositional matrix adjust.
 Identities = 36/134 (27%), Positives = 63/134 (47%), Gaps = 26/134 (19%)

Query  97   SCLVVFVALTLCGLYLEADDDAGREDKQCLRAVTEKSPLLPRREMSIGQSSTVDAPIRID  156
            S L + V + LC  Y++ ++ A R+                R +  IG+S      +RI 
Sbjct  160  SILSIIVCIFLCFFYMDEENVAHRKTSN------------TRIKTIIGES------VRIG  201

Query  157  IHNTLLWITLLLTAFIAFSTSSILTW----ILGASPA---MLPLIPAALSITLSLSSITL  209
              N  +WI  ++  FI+FS S+  T+    ++G S     ++  I A  S+ ++L S  L
Sbjct  202  FENKSIWIVFMIGLFISFSNSAGNTFQQPRLVGLSEQGIWIMGFIKAGYSLCMTLGS-YL  260

Query  210  SRPISAFFSELTLL  223
             R + A FS++ +L
Sbjct  261  VRKLGARFSDVHIL  274


>ref|XP_010452377.1| PREDICTED: cationic amino acid transporter 6, chloroplastic [Camelina 
sativa]
Length=587

 Score = 39.3 bits (90),  Expect = 5.6, Method: Compositional matrix adjust.
 Identities = 25/66 (38%), Positives = 38/66 (58%), Gaps = 11/66 (17%)

Query  71   FLGLFLWASNPLESSTSPNVIHYGIKSCLVVFVALTLCGLYLEADDDAG-----REDKQC  125
            FL +FL     L S  +P+   +G+ SCL+V V L L G++  +D +A      RED+Q 
Sbjct  526  FLNIFL-----LGSLDAPSYARFGVFSCLIVLVYL-LYGVHASSDAEANGSFDVREDRQV  579

Query  126  LRAVTE  131
            L+ +TE
Sbjct  580  LKELTE  585


>ref|XP_010423539.1| PREDICTED: cationic amino acid transporter 6, chloroplastic-like 
[Camelina sativa]
Length=588

 Score = 39.3 bits (90),  Expect = 5.9, Method: Compositional matrix adjust.
 Identities = 25/66 (38%), Positives = 38/66 (58%), Gaps = 11/66 (17%)

Query  71   FLGLFLWASNPLESSTSPNVIHYGIKSCLVVFVALTLCGLYLEADDDAG-----REDKQC  125
            FL +FL     L S  +P+   +G+ SCL+V V L L G++  +D +A      RED+Q 
Sbjct  527  FLNIFL-----LGSLDAPSYARFGVFSCLIVLVYL-LYGVHASSDAEANGSFGVREDRQV  580

Query  126  LRAVTE  131
            L+ +TE
Sbjct  581  LKELTE  586


Lambda      K        H        a         alpha
   0.323    0.134    0.394    0.792     4.96 

Gapped
Lambda      K        H        a         alpha    sigma
   0.267   0.0410    0.140     1.90     42.6     43.6 

Effective search space used: 2512642291612


  Database: nr
    Posted date:  Sep 23, 2015 12:05 AM
  Number of letters in database: 26,053,659,533
  Number of sequences in database:  71,551,133


Matrix: BLOSUM62
Gap Penalties: Existence: 11, Extension: 1
Neighboring words threshold: 11
Window for multiple hits: 40
```
