# Supplementary material for: Comparative genomics provides new insights into the diversity, physiology, and sexuality of the only industrially exploited tremellomycete: Phaffia rhodozyma
Source: BMC Genomics. 2016 Nov 9;17:901. doi: 10.1186/s12864-016-3244-7 (PMC5103461; doi:10.1186/s12864-016-3244-7)
Supplement: Additional file 6: — List of orphan genes with links to PFAM (related to Additional file 1: Table S1). (ZIP 1428 kb) [file 12864_2016_3244_MOESM6_ESM.zip › BLAST_HTML_FTR/G03136_P.html]

BLAST Search Results


```
BLASTP 2.2.27+


Reference:
Stephen F. Altschul, Thomas L. Madden, Alejandro A. Schäffer,
Jinghui Zhang, Zheng Zhang, Webb Miller, and David J. Lipman (1997),
"Gapped BLAST and PSI-BLAST: a new generation of protein database
search programs", Nucleic Acids Res. 25:3389-3402.


Reference for
composition-based statistics:
Alejandro A. Schäffer, L. Aravind, Thomas L. Madden, Sergei
Shavirin, John L. Spouge, Yuri I. Wolf, Eugene V. Koonin, and
Stephen F. Altschul (2001), "Improving the accuracy of PSI-BLAST
protein database searches with composition-based statistics and
other refinements", Nucleic Acids Res. 29:2994-3005.


Database: nr
           71,551,133 sequences; 26,053,659,533 total letters


Query= G03136_P

Length=456
                                                                      Score     E
Sequences producing significant alignments:                          (Bits)  Value

emb|CDZ96692.1|  hypothetical protein [Xanthophyllomyces dendrorh...   884    0.0  
gb|AAY56492.1|  membrane bound nitrate reductase, partial [uncult...  41.6    0.98 
gb|AAY56518.1|  membrane bound nitrate reductase, partial [uncult...  40.8    1.4  
gb|AAP79322.1|  putative dissimilatory membrane-bound nitrate red...  39.3    4.9  
emb|CED82486.1|  hypothetical protein [Xanthophyllomyces dendrorh...  39.3    9.0  


 >emb|CDZ96692.1| hypothetical protein [Xanthophyllomyces dendrorhous]
Length=441

 Score =  884 bits (2283),  Expect = 0.0, Method: Compositional matrix adjust.
 Identities = 441/455 (97%), Positives = 441/455 (97%), Gaps = 14/455 (3%)

Query  1    MSYNFTSSPLCTPSLSLISTASCASSDAPFSPPGICGMPPSPSPANRTAPLTRSLRMEKG  60
            MSYNFTSSPLCTPSLSLISTASCASSDAPFSPPGICGMPPSPSPANRTAPLTRSLRMEKG
Sbjct  1    MSYNFTSSPLCTPSLSLISTASCASSDAPFSPPGICGMPPSPSPANRTAPLTRSLRMEKG  60

Query  61   HPLQELFMTRPPTNNLFLKQSQIMYLGKARSESIQQHEVVLGQCAREDRRTDEAKRAEHV  120
            HPLQELFM              IMYLGKARSESIQQHEVVLGQCAREDRRTDEAKRAEHV
Sbjct  61   HPLQELFM--------------IMYLGKARSESIQQHEVVLGQCAREDRRTDEAKRAEHV  106

Query  121  GRTKLAGRVGRTKLAERMESSRPNRKLRKTARMKIDSSTWKPPRENVSFNDLSSKSLAVS  180
            GRTKLAGRVGRTKLAERMESSRPNRKLRKTARMKIDSSTWKPPRENVSFNDLSSKSLAVS
Sbjct  107  GRTKLAGRVGRTKLAERMESSRPNRKLRKTARMKIDSSTWKPPRENVSFNDLSSKSLAVS  166

Query  181  TATSSRTRTVSREQLATCRAPSRSHRNPRVEGRLILGELEWPQRRLSCVSPTVPLLSSPS  240
            TATSSRTRTVSREQLATCRAPSRSHRNPRVEGRLILGELEWPQRRLSCVSPTVPLLSSPS
Sbjct  167  TATSSRTRTVSREQLATCRAPSRSHRNPRVEGRLILGELEWPQRRLSCVSPTVPLLSSPS  226

Query  241  SKFLESSPTTSPLLGKRARQPTESDLYIDSVDMFLLATKSVTSQMEDVSEYSGFKDSSLD  300
            SKFLESSPTTSPLLGKRARQPTESDLYIDSVDMFLLATKSVTSQMEDVSEYSGFKDSSLD
Sbjct  227  SKFLESSPTTSPLLGKRARQPTESDLYIDSVDMFLLATKSVTSQMEDVSEYSGFKDSSLD  286

Query  301  KPSGESSRLAKRAKYSLCVSPKITSQPSPSIQWASAKSGSSCMELYKSEHRRPTALSQQR  360
            KPSGESSRLAKRAKYSLCVSPKITSQPSPSIQWASAKSGSSCMELYKSEHRRPTALSQQR
Sbjct  287  KPSGESSRLAKRAKYSLCVSPKITSQPSPSIQWASAKSGSSCMELYKSEHRRPTALSQQR  346

Query  361  ARPVADKEYIGSVEAWVKHVESARLYCDRHKRASSRRSLKNTLEAGSSSRPVHKPDQILP  420
            ARPVADKEYIGSVEAWVKHVESARLYCDRHKRASSRRSLKNTLEAGSSSRPVHKPDQILP
Sbjct  347  ARPVADKEYIGSVEAWVKHVESARLYCDRHKRASSRRSLKNTLEAGSSSRPVHKPDQILP  406

Query  421  PITHSVTYMEVESDLSVLGMNRKDEDVFSSASVTL  455
            PITHSVTYMEVESDLSVLGMNRKDEDVFSSASVTL
Sbjct  407  PITHSVTYMEVESDLSVLGMNRKDEDVFSSASVTL  441


>gb|AAY56492.1| membrane bound nitrate reductase, partial [uncultured bacterium]
Length=214

 Score = 41.6 bits (96),  Expect = 0.98, Method: Compositional matrix adjust.
 Identities = 33/125 (26%), Positives = 57/125 (46%), Gaps = 11/125 (9%)

Query  119  HVGRTKLAGRVGRTKLAERMESSRPNRKLRKTARMKIDSSTWKPPRENVSFNDLSSKSLA  178
            +VG+ KL  + G T LA  ++  RP R +  T+     S  W+   E ++ ++++S +  
Sbjct  1    YVGQEKLRPQTGWTPLAFALDWHRPPRHMNSTSYWYAHSDQWR--YEKLTVDEIASPTSD  58

Query  179  VSTATSSRTRTVSREQLATCRAPSRSHRNPRVEGRLILGE---------LEWPQRRLSCV  229
            V  A +     V  E++    +  +   NP   GRL+ GE         L+  + +LSC 
Sbjct  59   VDVAGTMIDFNVRAERMGWLPSAPQLETNPLEIGRLLKGEAPGTRVARDLKSGELKLSCE  118

Query  230  SPTVP  234
             P  P
Sbjct  119  DPDNP  123


>gb|AAY56518.1| membrane bound nitrate reductase, partial [uncultured bacterium]
Length=214

 Score = 40.8 bits (94),  Expect = 1.4, Method: Compositional matrix adjust.
 Identities = 33/125 (26%), Positives = 57/125 (46%), Gaps = 11/125 (9%)

Query  119  HVGRTKLAGRVGRTKLAERMESSRPNRKLRKTARMKIDSSTWKPPRENVSFNDLSSKSLA  178
            +VG+ KL  + G T LA  ++  RP R +  T+     S  W+   E ++ ++++S +  
Sbjct  1    YVGQEKLRPQTGWTPLAFALDWHRPPRHMNSTSYWYAYSDQWR--YEKLTVDEIASPTSD  58

Query  179  VSTATSSRTRTVSREQLATCRAPSRSHRNPRVEGRLILGE---------LEWPQRRLSCV  229
            V  A +     V  E++    +  +   NP   GRL+ GE         L+  + +LSC 
Sbjct  59   VDVAGTMIDFNVRAERMGWLPSAPQLETNPLEIGRLLKGEAPGTRVARDLKSGELKLSCE  118

Query  230  SPTVP  234
             P  P
Sbjct  119  DPDNP  123


>gb|AAP79322.1| putative dissimilatory membrane-bound nitrate reductase [uncultured 
bacterium]
Length=214

 Score = 39.3 bits (90),  Expect = 4.9, Method: Compositional matrix adjust.
 Identities = 33/125 (26%), Positives = 56/125 (45%), Gaps = 11/125 (9%)

Query  119  HVGRTKLAGRVGRTKLAERMESSRPNRKLRKTARMKIDSSTWKPPRENVSFNDLSSKSLA  178
            +VG+ KL  + G T LA  ++  RP R +  T+     S  W+   E +S  +++S +  
Sbjct  1    YVGQEKLRPQTGWTPLASALDWHRPPRHMNGTSFWYAHSDQWR--YEKLSVGEIASPTSD  58

Query  179  VSTATSSRTRTVSREQLATCRAPSRSHRNPRVEGRLILGE---------LEWPQRRLSCV  229
            V TA +     +  E++    +  +   NP   GR + GE         L+  + +LSC 
Sbjct  59   VDTAGTLIDFNIRAERMGWLPSAPQLETNPLEVGRSLKGEAAGLKVAHGLKSGELKLSCE  118

Query  230  SPTVP  234
             P  P
Sbjct  119  DPDNP  123


>emb|CED82486.1| hypothetical protein [Xanthophyllomyces dendrorhous]
 emb|CDZ96935.1| hypothetical protein [Xanthophyllomyces dendrorhous]
 emb|CDZ97473.1| hypothetical protein [Xanthophyllomyces dendrorhous]
Length=420

 Score = 39.3 bits (90),  Expect = 9.0, Method: Compositional matrix adjust.
 Identities = 72/287 (25%), Positives = 105/287 (37%), Gaps = 77/287 (27%)

Query  22   SCASSDAPFSPPGICGMPPSPSPANRTAPLTRSLRMEKGHPLQELFMTRPPTNNLFLKQS  81
            S AS DAP        MP SP+P +R AP  + LR  K +P++ L     P N       
Sbjct  60   SYASLDAPHFSSDTQPMPASPTPVSRNAPAAKPLRPAKRYPVEGL-----PINE------  108

Query  82   QIMYLGKARSESIQQHEVVLGQCAREDRRTDEAKRAEHVGRTKLAGRVGRTKLAERMESS  141
                                        R + A RA    R +   R GR + AE M   
Sbjct  109  ----------------------------RANVASRARQGDRPQPVKRAGRVRKAEAMRLP  140

Query  142  RPNRKLRKTARMKIDSSTWK------PPRENVSFNDLSSKSLAV----STATSSRTRTVS  191
                + R+ + +K  +   +       P  +   +   + S         A+SS T  V 
Sbjct  141  TTTTECRERSYLKSLTPIQRFELRGMEPMTDAEMDAFLNPSAIPKPLPQAASSSWTEMVD  200

Query  192  REQLATCRAPSRSHRNPRV-------------------------EGRLILGELEWPQRRL  226
            RE+L   R P  S +  RV                         + R  L   +     L
Sbjct  201  RERLQVPRPPFESPKRRRVIDDDKDDNDDDGSDEEKRRSDVRPAKRRFDLSSAKLSGSAL  260

Query  227  SCVSPTVP-LLSSPSSKFLESSPTTSPLLGKRARQPTESDLYIDSVD  272
            S  SP  P +LS+P  +  +SSP +SPL GK+ R+PT   + ++ VD
Sbjct  261  S--SPPAPSVLSAPPVERSDSSPASSPLFGKQTRRPTIPTIKVELVD  305


Lambda      K        H        a         alpha
   0.314    0.126    0.360    0.792     4.96 

Gapped
Lambda      K        H        a         alpha    sigma
   0.267   0.0410    0.140     1.90     42.6     43.6 

Effective search space used: 4540505085402


  Database: nr
    Posted date:  Sep 23, 2015 12:05 AM
  Number of letters in database: 26,053,659,533
  Number of sequences in database:  71,551,133


Matrix: BLOSUM62
Gap Penalties: Existence: 11, Extension: 1
Neighboring words threshold: 11
Window for multiple hits: 40
```
