# Supplementary material for: Comparative genomics provides new insights into the diversity, physiology, and sexuality of the only industrially exploited tremellomycete: Phaffia rhodozyma
Source: BMC Genomics. 2016 Nov 9;17:901. doi: 10.1186/s12864-016-3244-7 (PMC5103461; doi:10.1186/s12864-016-3244-7)
Supplement: Additional file 6: — List of orphan genes with links to PFAM (related to Additional file 1: Table S1). (ZIP 1428 kb) [file 12864_2016_3244_MOESM6_ESM.zip › BLAST_HTML_FTR/G01428_P.html]

BLAST Search Results


```
BLASTP 2.2.27+


Reference:
Stephen F. Altschul, Thomas L. Madden, Alejandro A. Schäffer,
Jinghui Zhang, Zheng Zhang, Webb Miller, and David J. Lipman (1997),
"Gapped BLAST and PSI-BLAST: a new generation of protein database
search programs", Nucleic Acids Res. 25:3389-3402.


Reference for
composition-based statistics:
Alejandro A. Schäffer, L. Aravind, Thomas L. Madden, Sergei
Shavirin, John L. Spouge, Yuri I. Wolf, Eugene V. Koonin, and
Stephen F. Altschul (2001), "Improving the accuracy of PSI-BLAST
protein database searches with composition-based statistics and
other refinements", Nucleic Acids Res. 29:2994-3005.


Database: nr
           71,551,133 sequences; 26,053,659,533 total letters


Query= G01428_P

Length=457
                                                                      Score     E
Sequences producing significant alignments:                          (Bits)  Value

emb|CDZ96723.1|  hypothetical protein [Xanthophyllomyces dendrorh...   656    0.0  
emb|CDZ96231.1|  hypothetical protein [Xanthophyllomyces dendrorh...  53.1    6e-04
gb|EJY55488.1|  alpha/beta hydrolase fold protein [Alicyclobacill...  41.2    1.6  


 >emb|CDZ96723.1| hypothetical protein [Xanthophyllomyces dendrorhous]
Length=400

 Score =  656 bits (1693),  Expect = 0.0, Method: Compositional matrix adjust.
 Identities = 375/464 (81%), Positives = 382/464 (82%), Gaps = 72/464 (16%)

Query  1    MLSKSVVRSRRSLSTLAASAFLTTNGVACRPSSSSSPSKFSQPKHKLLDKLIRSAGSSTE  60
            MLSKSVVRSRRSLSTLAASAFLTTNGVACRPSSSSSPSKFSQPKHKLLDKLIRSAGSSTE
Sbjct  1    MLSKSVVRSRRSLSTLAASAFLTTNGVACRPSSSSSPSKFSQPKHKLLDKLIRSAGSSTE  60

Query  61   LNKAVRIVQEWTQVSLPPTEVTLAWLNSLLDRARDLNDVPFSLDVLSDPITYGLNLPSQT  120
            LNKAVRIVQEWTQVSLPPTEVTLAWLNSLLDRARDLNDVPFSLDVLSDPITYGLNLPSQT
Sbjct  61   LNKAVRIVQEWTQVSLPPTEVTLAWLNSLLDRARDLNDVPFSLDVLSDPITYGLNLPSQT  120

Query  121  PPSIFRKLLYNLLHPPVASTSTTSDHDQFHTSRPSRVHLNAFMDLANRHAPGDPLILLLA  180
            PPSIFRKLLYNLLHPPVASTSTTSDHDQFHTSRPSRVHLNAFMDLANRHAPGDPLILLLA
Sbjct  121  PPSIFRKLLYNLLHPPVASTSTTSDHDQFHTSRPSRVHLNAFMDLANRHAPGDPLILLLA  180

Query  181  LEGYISLWEEGIGRRVWIEQEIRHYRNRWIPKADGGDIKLTLNAEESAWMINTLDNLRPF  240
            LEGYISLWEEGIGRRVWIEQEIRHYRNRWIPKADGGDIKLTLNAEESAWMINTLDNLRPF
Sbjct  181  LEGYISLWEEGIGRRVWIEQEIRHYRNRWIPKADGGDIKLTLNAEESAWMINTLDNLRPF  240

Query  241  VADALLESWGRLEMPDPGVRF---HRSRIH-----SLLSRYDHLPIHHPSSSSASSSSSS  292
            VADALLESWGRLEMPDPGV +   H  R +     +LL+ Y HLP+HH            
Sbjct  241  VADALLESWGRLEMPDPGVSYLEAHTQRANLPLTSNLLAHYIHLPLHHHP----------  290

Query  293  LSDPPIALSSHSFHSSLASSYGKSYLEAHTQRANLPLTSNLLAHYIHLPLHHHPTDPFII  352
             +DP I LS+                                   IHL            
Sbjct  291  -TDPFIILSA-----------------------------------IHL------------  302

Query  353  LSAIHLYSLDLPRYRDQIDNLLNSLPNENPWAARSSTSGTGSGRELSPVERTRMRSSLDR  412
                  YSLDLPRYRDQIDNLLNSLPNENPWAARSSTSGTGSGRELSPVERTRMRSSLDR
Sbjct  303  ------YSLDLPRYRDQIDNLLNSLPNENPWAARSSTSGTGSGRELSPVERTRMRSSLDR  356

Query  413  VGSLLGDKKIFWESFDPVDVEVGVDVGGLSVGRAALDLRSRAIP  456
            VGSLLGDKKIFWESFDPVDVEVGVDVGGLSVGRAALDLRSRAIP
Sbjct  357  VGSLLGDKKIFWESFDPVDVEVGVDVGGLSVGRAALDLRSRAIP  400


>emb|CDZ96231.1| hypothetical protein [Xanthophyllomyces dendrorhous]
Length=838

 Score = 53.1 bits (126),  Expect = 6e-04, Method: Compositional matrix adjust.
 Identities = 57/231 (25%), Positives = 100/231 (43%), Gaps = 14/231 (6%)

Query  202  IRHYRNRWIPKADGGDIKLTLNAEESAWMINTLDNLRPFVADALLESWGRLEMPDPGVRF  261
            +R  ++ W      G+IKL      S W++  LD++R FV   L   W +  +P PG+ F
Sbjct  606  VRTGKDTWWSGLSPGEIKL-----HSRWLLEALDSVRSFVPPELALLW-KAGIPSPGLDF  659

Query  262  HRSRIHSLLSRYDHLPIHHPSSSSASSSSSSLSDPPIALSSHSFHSSLASSYGKSYLEAH  321
            HR+R+  +    D      P + +    ++ +S+  I  + H       SS  +  +   
Sbjct  660  HRARLREVFLLVDRAK-PKPYTITLERLNNDVSNDSITNNKHKGRHQHRSSSQEDTMSDL  718

Query  322  TQRANLPLTSNLLAHYIHLPLHHHPTDPFIILSAIHLYSLDLPRYR--DQIDNLLNSLPN  379
                 LPL   L+  ++  PL  H +DP I+L ++ +    +P      +I   + +LP 
Sbjct  719  GLPKALPLVPRLIESFLDSPLASHHSDPIIVLFSLGIRMKMIPDESALREIHWTIRNLPK  778

Query  380  -ENPW---AARSSTSGTGSGRELSPVERTRMRSSLDRVGSLLGDKKIFWES  426
                W    A +S    G+G  +S  +R    + L  V  LL +K   W++
Sbjct  779  LAKSWDKVIASTSRQIEGAG-WISKQDRIVAEACLGMVEELLNEKVELWQN  828


>gb|EJY55488.1| alpha/beta hydrolase fold protein [Alicyclobacillus hesperidum 
URH17-3-68]
Length=282

 Score = 41.2 bits (95),  Expect = 1.6, Method: Compositional matrix adjust.
 Identities = 32/106 (30%), Positives = 50/106 (47%), Gaps = 2/106 (2%)

Query  148  QFHTSRPSRVHLNAFMDLANRHAPGDPLILLLALEGYISLWEEGIGRRVWIEQEIRHYRN  207
            ++  SR  R  L A       H  G PLIL++ L G    W +G  RR+  ++++  + N
Sbjct  11   RWEESRVPRADLGAVELYYEVHGEGQPLILIMGLGGNADWWGDGFVRRLAAKRQVIAFDN  70

Query  208  RWIPK-ADGGDIKLTLNAEESAWMINTLDNLRPFVADALLESWGRL  252
            R   + A  GD   TL AE +  ++  + +L   VAD    S G +
Sbjct  71   RGAARTALRGDEHFTL-AEMAGDVVGLMRHLDIAVADVFGVSMGGM  115


Lambda      K        H        a         alpha
   0.318    0.133    0.399    0.792     4.96 

Gapped
Lambda      K        H        a         alpha    sigma
   0.267   0.0410    0.140     1.90     42.6     43.6 

Effective search space used: 4555539870453


  Database: nr
    Posted date:  Sep 23, 2015 12:05 AM
  Number of letters in database: 26,053,659,533
  Number of sequences in database:  71,551,133


Matrix: BLOSUM62
Gap Penalties: Existence: 11, Extension: 1
Neighboring words threshold: 11
Window for multiple hits: 40
```
