# Supplementary material for: Comparative genomics provides new insights into the diversity, physiology, and sexuality of the only industrially exploited tremellomycete: Phaffia rhodozyma
Source: BMC Genomics. 2016 Nov 9;17:901. doi: 10.1186/s12864-016-3244-7 (PMC5103461; doi:10.1186/s12864-016-3244-7)
Supplement: Additional file 6: — List of orphan genes with links to PFAM (related to Additional file 1: Table S1). (ZIP 1428 kb) [file 12864_2016_3244_MOESM6_ESM.zip › BLAST_HTML_FTR/G00362_P.html]

BLAST Search Results


```
BLASTP 2.2.27+


Reference:
Stephen F. Altschul, Thomas L. Madden, Alejandro A. Schäffer,
Jinghui Zhang, Zheng Zhang, Webb Miller, and David J. Lipman (1997),
"Gapped BLAST and PSI-BLAST: a new generation of protein database
search programs", Nucleic Acids Res. 25:3389-3402.


Reference for
composition-based statistics:
Alejandro A. Schäffer, L. Aravind, Thomas L. Madden, Sergei
Shavirin, John L. Spouge, Yuri I. Wolf, Eugene V. Koonin, and
Stephen F. Altschul (2001), "Improving the accuracy of PSI-BLAST
protein database searches with composition-based statistics and
other refinements", Nucleic Acids Res. 29:2994-3005.


Database: nr
           71,551,133 sequences; 26,053,659,533 total letters


Query= G00362_P

Length=250
                                                                      Score     E
Sequences producing significant alignments:                          (Bits)  Value

emb|CED83251.1|  RNA recognition motif domain [Xanthophyllomyces ...   506    1e-179
emb|CDS29517.1|  heterogeneous nuclear ribonucleoprotein [Hymenol...  46.2    0.018 
gb|KKQ87190.1|  Glycine-rich RNA-binding protein GRP1A [Parcubact...  42.7    0.040 
gb|KKW30655.1|  RNP-1 like protein RNA-binding protein [Parcubact...  42.4    0.050 
ref|XP_007665524.1|  PREDICTED: DAZ-associated protein 1 [Ornitho...  44.3    0.088 
ref|XP_002367750.1|  U1 small nuclear ribonucleoprotein, putative...  43.1    0.12  
emb|CDH59702.1|  rna-binding domain-containing protein [Lichtheim...  43.5    0.14  
ref|XP_008887446.1|  RNA recognition motif-containing protein [Ha...  43.1    0.14  
gb|KKR09175.1|  RNA-binding protein (RRM domain) [Peregrinibacter...  41.2    0.15  
gb|KKS42347.1|  RRM domain-containing RNA-binding protein [Parcub...  40.8    0.18  
gb|KKQ21192.1|  RNP-1 like protein RNA-binding protein [Parcubact...  40.8    0.18  
gb|KKP64586.1|  RNP-1 like protein RNA-binding protein [Parcubact...  40.8    0.19  
emb|CDS07137.1|  hypothetical protein LRAMOSA09660 [Absidia idaho...  43.1    0.19  
ref|XP_010223297.1|  PREDICTED: DAZ-associated protein 1, partial...  42.7    0.21  
gb|KGL84381.1|  DAZ-associated protein 1, partial [Tinamus guttatus]  42.7    0.23  
ref|XP_008985159.1|  PREDICTED: DAZ-associated protein 1 isoform ...  42.4    0.31  
ref|XP_012291431.1|  PREDICTED: DAZ-associated protein 1 isoform ...  42.4    0.31  
ref|XP_010330750.1|  PREDICTED: DAZ-associated protein 1 isoform ...  42.4    0.33  
gb|AEK69207.1|  boule-like protein 3 [Macrostomum lignano]            40.8    0.35  
gb|KKS04378.1|  RNP-1 like protein RNA-binding protein [Parcubact...  40.0    0.36  
ref|XP_008985154.1|  PREDICTED: DAZ-associated protein 1 isoform ...  42.0    0.37  
ref|XP_012291429.1|  PREDICTED: DAZ-associated protein 1 isoform ...  42.0    0.38  
ref|XP_012291427.1|  PREDICTED: DAZ-associated protein 1 isoform ...  42.0    0.39  
gb|KKS50256.1|  Glycine-rich RNA-binding protein GRP1A [Parcubact...  39.7    0.39  
ref|XP_008985158.1|  PREDICTED: DAZ-associated protein 1 isoform ...  42.0    0.39  
ref|XP_008985157.1|  PREDICTED: DAZ-associated protein 1 isoform ...  42.0    0.39  
ref|XP_012291430.1|  PREDICTED: DAZ-associated protein 1 isoform ...  42.0    0.40  
ref|XP_012291428.1|  PREDICTED: DAZ-associated protein 1 isoform ...  42.0    0.40  
ref|XP_010330747.1|  PREDICTED: DAZ-associated protein 1 isoform ...  42.0    0.40  
ref|XP_008985156.1|  PREDICTED: DAZ-associated protein 1 isoform ...  42.0    0.40  
ref|XP_008928902.1|  PREDICTED: DAZ-associated protein 1 [Manacus...  42.0    0.41  
ref|XP_010330749.1|  PREDICTED: DAZ-associated protein 1 isoform ...  42.0    0.41  
ref|XP_010330748.1|  PREDICTED: DAZ-associated protein 1 isoform ...  42.0    0.41  
emb|CCK67910.1|  hypothetical protein KNAG_0A02210 [Kazachstania ...  42.4    0.41  
ref|XP_013800785.1|  PREDICTED: DAZ-associated protein 1 isoform ...  42.0    0.44  
ref|XP_010442944.1|  PREDICTED: LOW QUALITY PROTEIN: RNA-binding ...  39.7    0.44  
gb|KDQ60783.1|  hypothetical protein JAAARDRAFT_190929 [Jaapia ar...  40.4    0.46  
pdb|2DGS|A  Chain A, Solution Structure Of The Second Rna Binding...  39.7    0.47  
gb|KKS54071.1|  RNA-binding protein [Parcubacteria bacterium GW20...  39.7    0.48  
gb|KKQ04002.1|  RNP-1 like protein RNA-binding protein [Parcubact...  39.7    0.48  
ref|XP_012958411.1|  PREDICTED: DAZ-associated protein 1 [Anas pl...  42.0    0.49  
gb|ESA01207.1|  hypothetical protein GLOINDRAFT_262119 [Rhizophag...  41.6    0.49  
ref|XP_007764737.1|  RNA-binding domain-containing protein [Conio...  40.4    0.49  
ref|XP_013800775.1|  PREDICTED: DAZ-associated protein 1 isoform ...  41.6    0.49  
ref|XP_005433987.1|  PREDICTED: LOW QUALITY PROTEIN: DAZ-associat...  41.6    0.50  
ref|XP_013055534.1|  PREDICTED: DAZ-associated protein 1 isoform ...  41.6    0.52  
ref|XP_008985161.1|  PREDICTED: DAZ-associated protein 1 isoform ...  41.6    0.52  
ref|XP_008945735.1|  PREDICTED: DAZ-associated protein 1, partial...  41.6    0.53  
ref|XP_009865967.1|  PREDICTED: DAZ-associated protein 1, partial...  41.6    0.53  
ref|XP_009936272.1|  PREDICTED: LOW QUALITY PROTEIN: DAZ-associat...  41.6    0.53  
gb|EMT22954.1|  28 kDa ribonucleoprotein, chloroplastic [Aegilops...  42.0    0.53  
ref|XP_010723322.1|  PREDICTED: DAZ-associated protein 1 [Meleagr...  41.6    0.55  
gb|KFW86571.1|  DAZ-associated protein 1, partial [Manacus vitell...  41.6    0.56  
ref|XP_013055533.1|  PREDICTED: DAZ-associated protein 1 isoform ...  41.6    0.56  
ref|XP_007862855.1|  hypothetical protein GLOTRDRAFT_35766 [Gloeo...  39.7    0.56  
ref|XP_010409941.1|  PREDICTED: DAZ-associated protein 1 [Corvus ...  41.6    0.57  
ref|XP_010149380.1|  PREDICTED: DAZ-associated protein 1 [Eurypyg...  41.6    0.57  
ref|XP_010135175.1|  PREDICTED: DAZ-associated protein 1, partial...  41.2    0.57  
gb|KFW04747.1|  DAZ-associated protein 1, partial [Eurypyga helia...  41.6    0.58  
gb|KKR02014.1|  RNP-1 like protein RNA-binding protein [Parcubact...  39.3    0.58  
ref|XP_002768166.1|  pre-mRNA-splicing factor SF2, putative [Perk...  41.6    0.58  
ref|XP_009665809.1|  PREDICTED: DAZ-associated protein 1, partial...  41.6    0.58  
ref|XP_012761430.1|  RNA-binding protein, putative [Plasmodium re...  41.6    0.58  
ref|XP_010125130.1|  PREDICTED: DAZ-associated protein 1, partial...  41.2    0.59  
emb|CEM35350.1|  unnamed protein product [Vitrella brassicaformis...  41.6    0.60  
ref|XP_013800769.1|  PREDICTED: DAZ-associated protein 1 isoform ...  41.6    0.60  
ref|XP_009278158.1|  PREDICTED: DAZ-associated protein 1 [Aptenod...  41.6    0.61  
ref|XP_009907611.1|  PREDICTED: heterogeneous nuclear ribonucleop...  39.3    0.61  
ref|XP_005060220.1|  PREDICTED: DAZ-associated protein 1 [Ficedul...  41.6    0.61  
ref|XP_006017617.1|  PREDICTED: DAZ-associated protein 1 [Alligat...  41.6    0.61  
gb|KFQ36552.1|  DAZ-associated protein 1, partial [Merops nubicus]    41.2    0.61  
gb|ETW56723.1|  hypothetical protein PFUGPA_01513 [Plasmodium fal...  41.2    0.61  
ref|XP_012397671.1|  PREDICTED: DAZ-associated protein 1 [Sarcoph...  41.6    0.62  
ref|XP_009897503.1|  PREDICTED: LOW QUALITY PROTEIN: DAZ-associat...  41.6    0.62  
ref|XP_010003630.1|  PREDICTED: DAZ-associated protein 1 [Chaetur...  41.6    0.62  
emb|CEP60191.1|  LALA0S01e05072g1_1 [Lachancea lanzarotensis]         41.2    0.63  
ref|XP_009924112.1|  PREDICTED: DAZ-associated protein 1, partial...  41.2    0.64  
ref|XP_010071977.1|  PREDICTED: DAZ-associated protein 1 [Pterocl...  41.2    0.65  
ref|XP_006267895.1|  PREDICTED: DAZ-associated protein 1 isoform ...  41.2    0.65  
ref|XP_009566610.1|  PREDICTED: DAZ-associated protein 1 [Cuculus...  41.2    0.66  
ref|XP_009327196.1|  PREDICTED: DAZ-associated protein 1 [Pygosce...  41.2    0.66  
ref|XP_008639317.1|  PREDICTED: DAZ-associated protein 1 [Corvus ...  41.2    0.67  
gb|KFR13441.1|  DAZ-associated protein 1, partial [Opisthocomus h...  41.2    0.67  
ref|XP_011594903.1|  PREDICTED: DAZ-associated protein 1 [Aquila ...  41.2    0.68  
ref|XP_009882852.1|  PREDICTED: DAZ-associated protein 1 [Charadr...  41.2    0.68  
ref|XP_009957872.1|  PREDICTED: DAZ-associated protein 1 [Leptoso...  41.2    0.68  
ref|XP_009639347.1|  PREDICTED: DAZ-associated protein 1 [Egretta...  41.2    0.68  
ref|XP_005428644.1|  PREDICTED: DAZ-associated protein 1 [Geospiz...  41.2    0.69  
ref|XP_013157363.1|  PREDICTED: DAZ-associated protein 1 [Falco p...  41.2    0.70  
ref|XP_010561581.1|  PREDICTED: DAZ-associated protein 1 [Haliaee...  41.2    0.70  
ref|XP_001351452.1|  RNA binding protein, putative [Plasmodium fa...  41.2    0.70  
ref|XP_009980991.1|  PREDICTED: DAZ-associated protein 1, partial...  41.2    0.70  
ref|XP_009490696.1|  PREDICTED: DAZ-associated protein 1, partial...  41.2    0.70  
ref|XP_010193137.1|  PREDICTED: DAZ-associated protein 1 [Mesitor...  41.2    0.71  
ref|XP_009467366.1|  PREDICTED: DAZ-associated protein 1 [Nipponi...  41.2    0.71  
gb|EOA98730.1|  DAZ-associated protein 1, partial [Anas platyrhyn...  41.2    0.71  
ref|XP_009577426.1|  PREDICTED: DAZ-associated protein 1, partial...  41.2    0.71  
ref|XP_011526210.1|  PREDICTED: DAZ-associated protein 1 isoform ...  41.2    0.71  
ref|XP_006898062.1|  PREDICTED: DAZ-associated protein 1 [Elephan...  41.6    0.71  
gb|ETW38431.1|  hypothetical protein PFTANZ_00862 [Plasmodium fal...  40.8    0.72  
ref|XP_012986147.1|  PREDICTED: DAZ-associated protein 1 [Melopsi...  41.2    0.72  
gb|KFU97824.1|  DAZ-associated protein 1, partial [Pterocles gutt...  41.2    0.72  
ref|XP_006401670.1|  hypothetical protein EUTSA_v10015559mg [Eutr...  39.7    0.73  
ref|XP_013055532.1|  PREDICTED: DAZ-associated protein 1 isoform ...  41.2    0.73  
ref|XP_009708903.1|  PREDICTED: DAZ-associated protein 1 [Cariama...  41.2    0.73  
ref|XP_013055531.1|  PREDICTED: DAZ-associated protein 1 isoform ...  41.2    0.73  
ref|XP_009500835.1|  PREDICTED: DAZ-associated protein 1, partial...  41.2    0.74  
ref|XP_007382467.1|  hypothetical protein PUNSTDRAFT_100868 [Punc...  41.2    0.74  
ref|XP_002194831.1|  PREDICTED: DAZ-associated protein 1 [Taeniop...  41.2    0.74  
ref|XP_006267896.1|  PREDICTED: DAZ-associated protein 1 isoform ...  41.2    0.74  
gb|KFP05016.1|  DAZ-associated protein 1, partial [Calypte anna]      41.2    0.74  
ref|XP_011373487.1|  PREDICTED: DAZ-associated protein 1 isoform ...  41.2    0.74  
gb|KKR04684.1|  RNP-1 like protein RNA-binding protein [Parcubact...  38.9    0.75  
ref|XP_004955408.1|  PREDICTED: RNA-binding protein 1-like [Setar...  41.2    0.78  
ref|XP_007072131.1|  PREDICTED: LOW QUALITY PROTEIN: DAZ-associat...  40.8    0.78  
ref|NP_001026599.1|  DAZ-associated protein 1 [Gallus gallus] >em...  41.2    0.79  
ref|XP_008497449.1|  PREDICTED: DAZ-associated protein 1 [Calypte...  41.2    0.79  
ref|XP_011526208.1|  PREDICTED: DAZ-associated protein 1 isoform ...  41.2    0.79  
gb|AAD49731.1|AF169290_1  hnRNP A2/B1 protein [Sus scrofa]            38.5    0.79  
ref|XP_008509609.1|  PREDICTED: DAZ-associated protein 1, partial...  41.2    0.81  
ref|XP_007489409.1|  PREDICTED: DAZ-associated protein 1 isoform ...  41.2    0.81  
ref|XP_005531237.1|  PREDICTED: DAZ-associated protein 1 [Pseudop...  41.2    0.82  
ref|XP_011526209.1|  PREDICTED: DAZ-associated protein 1 isoform ...  41.2    0.82  
ref|XP_007489410.1|  PREDICTED: DAZ-associated protein 1 isoform ...  41.2    0.82  
ref|XP_005497184.1|  PREDICTED: DAZ-associated protein 1 [Zonotri...  41.2    0.82  
ref|XP_005983429.1|  PREDICTED: heterogeneous nuclear ribonucleop...  40.8    0.82  
ref|XP_011373485.1|  PREDICTED: DAZ-associated protein 1 isoform ...  41.2    0.83  
ref|XP_011526206.1|  PREDICTED: DAZ-associated protein 1 isoform ...  41.2    0.83  
ref|XP_007489412.1|  PREDICTED: DAZ-associated protein 1 isoform ...  41.2    0.83  
ref|XP_007489411.1|  PREDICTED: DAZ-associated protein 1 isoform ...  41.2    0.84  
ref|XP_013260251.1|  hypothetical protein A1O9_05579 [Exophiala a...  41.2    0.84  
ref|XP_011526207.1|  PREDICTED: DAZ-associated protein 1 isoform ...  41.2    0.84  
ref|XP_007075779.1|  PREDICTED: DAZ-associated protein 1 [Panther...  41.2    0.84  
ref|XP_011373486.1|  PREDICTED: DAZ-associated protein 1 isoform ...  41.2    0.85  
ref|XP_008709425.1|  PREDICTED: LOW QUALITY PROTEIN: DAZ-associat...  40.8    0.85  
ref|XP_004695951.2|  PREDICTED: DAZ-associated protein 1 isoform ...  40.8    0.86  
ref|XP_005866756.1|  PREDICTED: LOW QUALITY PROTEIN: DAZ associat...  40.8    0.86  
gb|KKS23261.1|  RNP-1 like protein RNA-binding protein [Parcubact...  38.9    0.86  
ref|XP_005682549.2|  PREDICTED: uncharacterized protein LOC102176...  41.2    0.87  
ref|XP_013216845.1|  PREDICTED: LOW QUALITY PROTEIN: DAZ-associat...  41.2    0.87  
ref|XP_006125020.1|  PREDICTED: DAZ-associated protein 1 isoform ...  40.8    0.89  
gb|ELV09335.1|  DAZ-associated protein 1, partial [Tupaia chinensis]  40.8    0.89  
ref|XP_003230223.2|  PREDICTED: DAZ-associated protein 1, partial...  40.4    0.90  
ref|XP_006125019.1|  PREDICTED: DAZ-associated protein 1 isoform ...  40.8    0.90  
ref|XP_008161267.1|  PREDICTED: DAZ-associated protein 1 isoform ...  40.8    0.91  
ref|XP_012585271.1|  PREDICTED: DAZ-associated protein 1 isoform ...  40.8    0.92  
ref|XP_007614942.1|  PREDICTED: LOW QUALITY PROTEIN: DAZ-associat...  40.8    0.92  
ref|XP_012585270.1|  PREDICTED: DAZ-associated protein 1 isoform ...  40.8    0.93  
ref|XP_013918739.1|  PREDICTED: DAZ-associated protein 1 isoform ...  40.8    0.94  
ref|XP_008161260.1|  PREDICTED: DAZ-associated protein 1 isoform ...  40.8    0.95  
ref|XP_010373256.1|  PREDICTED: DAZ-associated protein 1 [Rhinopi...  40.8    0.96  
ref|XP_007431853.1|  PREDICTED: DAZ-associated protein 1 [Python ...  40.8    0.96  
ref|XP_009014475.1|  hypothetical protein HELRODRAFT_76754 [Helob...  40.8    0.96  
ref|XP_010307268.1|  PREDICTED: DAZ-associated protein 1, partial...  40.8    0.97  
ref|XP_007176686.1|  PREDICTED: DAZ-associated protein 1 [Balaeno...  40.8    0.98  
ref|XP_008964813.1|  PREDICTED: LOW QUALITY PROTEIN: DAZ-associat...  40.8    0.99  
gb|ACO11942.1|  RNA-binding protein squid [Lepeophtheirus salmonis]   40.4    1.0   
ref|XP_004441492.1|  PREDICTED: DAZ-associated protein 1 [Ceratot...  40.8    1.0   
ref|XP_008059032.1|  PREDICTED: DAZ-associated protein 1 isoform ...  40.8    1.0   
ref|XP_013820910.1|  PREDICTED: DAZ-associated protein 1 [Capra h...  40.8    1.0   
ref|XP_011286492.1|  PREDICTED: DAZ-associated protein 1 isoform ...  40.8    1.0   
ref|XP_007992805.1|  PREDICTED: DAZ-associated protein 1 isoform ...  40.8    1.0   
gb|EUB59616.1|  Heterogeneous nuclear ribonucleoproteins A2/B1 [E...  40.4    1.0   
ref|XP_006050586.1|  PREDICTED: DAZ-associated protein 1 isoform ...  40.8    1.0   
gb|KKU49817.1|  RNA-binding protein [Parcubacteria bacterium GW20...  38.5    1.0   
ref|XP_005587455.1|  PREDICTED: DAZ-associated protein 1 isoform ...  40.4    1.0   
ref|XP_004417802.2|  PREDICTED: DAZ-associated protein 1 isoform ...  40.8    1.0   
gb|KKP24625.1|  RNP-1 like protein RNA-binding protein [Parcubact...  38.5    1.0   
ref|XP_005890529.1|  PREDICTED: DAZ-associated protein 1 [Bos mutus]  40.8    1.0   
ref|XP_008161256.1|  PREDICTED: DAZ-associated protein 1 isoform ...  40.8    1.0   
ref|XP_009250816.1|  PREDICTED: DAZ-associated protein 1 isoform ...  40.8    1.1   
ref|XP_010633232.1|  PREDICTED: DAZ-associated protein 1 isoform ...  40.8    1.1   
ref|NP_733829.1|  DAZ-associated protein 1 isoform a [Homo sapien...  40.8    1.1   
ref|XP_009250817.1|  PREDICTED: DAZ-associated protein 1 isoform ...  40.8    1.1   
ref|XP_009250815.1|  PREDICTED: DAZ-associated protein 1 isoform ...  40.8    1.1   
emb|CBY09798.1|  unnamed protein product [Oikopleura dioica]          40.4    1.1   
ref|XP_012789609.1|  PREDICTED: DAZ-associated protein 1 isoform ...  40.4    1.1   
ref|XP_013918737.1|  PREDICTED: DAZ-associated protein 1 isoform ...  40.8    1.1   
ref|XP_011971526.1|  PREDICTED: DAZ-associated protein 1 isoform ...  40.4    1.1   
emb|CDS21411.1|  heterogeneous nuclear ribonucleoprotein [Echinoc...  40.8    1.1   
ref|XP_004285911.1|  PREDICTED: DAZ-associated protein 1 isoform ...  40.4    1.1   
ref|XP_004595974.1|  PREDICTED: DAZ-associated protein 1 [Ochoton...  40.8    1.1   
ref|XP_005302407.1|  PREDICTED: DAZ-associated protein 1 isoform ...  40.8    1.1   
ref|XP_005661445.1|  PREDICTED: DAZ-associated protein 1 isoform ...  40.4    1.1   
ref|XP_010633230.1|  PREDICTED: DAZ-associated protein 1 isoform ...  40.8    1.1   
emb|CDS15233.1|  Heterogeneous nuclear ribonucleoprotein A2 [Echi...  40.4    1.1   
ref|XP_009250813.1|  PREDICTED: DAZ-associated protein 1 isoform ...  40.8    1.1   
gb|KIK08600.1|  hypothetical protein K443DRAFT_672131 [Laccaria a...  39.3    1.1   
ref|XP_004654943.1|  PREDICTED: DAZ-associated protein 1 isoform ...  40.8    1.1   
ref|XP_005890797.1|  PREDICTED: heterogeneous nuclear ribonucleop...  40.4    1.1   
emb|CDJ02783.1|  Heterogeneous nuclear ribonucleoprotein A2 [Echi...  40.4    1.1   
ref|XP_010633233.1|  PREDICTED: DAZ-associated protein 1 isoform ...  40.4    1.1   
ref|XP_006171826.1|  PREDICTED: LOW QUALITY PROTEIN: DAZ-associat...  40.8    1.1   
ref|XP_010633231.1|  PREDICTED: DAZ-associated protein 1 isoform ...  40.4    1.1   
ref|XP_009250814.1|  PREDICTED: DAZ-associated protein 1 isoform ...  40.4    1.1   
ref|XP_012411610.1|  PREDICTED: DAZ-associated protein 1 [Trichec...  40.8    1.1   
ref|XP_004654941.1|  PREDICTED: DAZ-associated protein 1 isoform ...  40.4    1.1   
dbj|BAB71295.1|  unnamed protein product [Homo sapiens]               40.4    1.2   
ref|XP_004717042.1|  PREDICTED: DAZ-associated protein 1 [Echinop...  40.8    1.2   
gb|KFO28898.1|  DAZ-associated protein 1 [Fukomys damarensis]         40.4    1.2   
gb|AAH77252.1|  LOC398218 protein [Xenopus laevis]                    40.4    1.2   
ref|XP_005083368.1|  PREDICTED: DAZ-associated protein 1 isoform ...  40.4    1.2   
ref|XP_012815278.1|  PREDICTED: DAZ-associated protein 1 isoform ...  40.4    1.2   
ref|XP_006206658.1|  PREDICTED: DAZ-associated protein 1 [Vicugna...  40.8    1.2   
ref|XP_006050584.1|  PREDICTED: DAZ-associated protein 1 isoform ...  40.4    1.2   
ref|XP_012659962.1|  PREDICTED: DAZ-associated protein 1 isoform ...  40.4    1.2   
ref|XP_005302410.1|  PREDICTED: DAZ-associated protein 1 isoform ...  40.4    1.2   
ref|XP_004866011.1|  PREDICTED: DAZ-associated protein 1 isoform ...  40.4    1.2   
ref|XP_010079059.1|  PREDICTED: heterogeneous nuclear ribonucleop...  39.3    1.2   
ref|XP_012601176.1|  PREDICTED: DAZ-associated protein 1 isoform ...  40.4    1.2   
ref|XP_005406028.2|  PREDICTED: DAZ-associated protein 1 isoform ...  40.4    1.2   
ref|XP_012500916.1|  PREDICTED: DAZ-associated protein 1 isoform ...  40.4    1.2   
ref|XP_011228551.1|  PREDICTED: DAZ-associated protein 1, partial...  40.4    1.2   
ref|XP_003788744.1|  PREDICTED: DAZ-associated protein 1 isoform ...  40.4    1.2   
gb|AAF81071.1|AF225910_1  DAZ-associated protein 1 [Mus musculus]     40.4    1.2   
ref|XP_007641385.1|  PREDICTED: DAZ-associated protein 1 isoform ...  40.4    1.2   
ref|XP_012601177.1|  PREDICTED: DAZ-associated protein 1 isoform ...  40.4    1.2   
ref|XP_005611795.1|  PREDICTED: DAZ-associated protein 1 [Equus c...  40.8    1.2   
ref|XP_005587449.1|  PREDICTED: DAZ-associated protein 1 isoform ...  40.4    1.2   
gb|EPQ08082.1|  DAZ-associated protein 1 [Myotis brandtii]            40.4    1.2   
gb|EHB02473.1|  DAZ-associated protein 1 [Heterocephalus glaber]      40.4    1.2   
ref|XP_012500913.1|  PREDICTED: DAZ-associated protein 1 isoform ...  40.4    1.2   
ref|XP_006050585.1|  PREDICTED: DAZ-associated protein 1 isoform ...  40.4    1.2   
dbj|GAN04573.1|  ribosome biogenesis protein Nop4 [Mucor ambiguus]    40.8    1.2   
ref|XP_012815270.1|  PREDICTED: DAZ-associated protein 1 isoform ...  40.4    1.2   
gb|ELR60208.1|  DAZ-associated protein 1, partial [Bos mutus]         40.4    1.2   
ref|XP_004462795.1|  PREDICTED: DAZ-associated protein 1 isoform ...  40.4    1.2   
dbj|BAE31119.1|  unnamed protein product [Mus musculus] >dbj|BAE3...  40.4    1.2   
ref|XP_004632736.1|  PREDICTED: DAZ-associated protein 1 isoform ...  40.4    1.2   
ref|XP_004866009.1|  PREDICTED: DAZ-associated protein 1 isoform ...  40.4    1.2   
ref|XP_012601175.1|  PREDICTED: DAZ-associated protein 1 isoform ...  40.4    1.2   
ref|XP_011286478.1|  PREDICTED: DAZ-associated protein 1 isoform ...  40.4    1.2   
ref|NP_001020913.1|  DAZ-associated protein 1 [Rattus norvegicus]...  40.4    1.2   
ref|NP_001116077.1|  DAZ-associated protein 1 isoform c [Mus musc...  40.4    1.2   
ref|XP_008148910.1|  PREDICTED: DAZ-associated protein 1 [Eptesic...  40.4    1.3   
gb|ETN57998.1|  heterogeneous nuclear ribonucleoprotein [Anophele...  40.4    1.3   
ref|XP_012394575.1|  PREDICTED: DAZ-associated protein 1 isoform ...  40.4    1.3   
ref|XP_010600162.1|  PREDICTED: DAZ-associated protein 1 isoform ...  40.4    1.3   
ref|XP_004866012.1|  PREDICTED: DAZ-associated protein 1 isoform ...  40.4    1.3   
ref|XP_004654942.1|  PREDICTED: DAZ-associated protein 1 isoform ...  40.4    1.3   
ref|XP_012500914.1|  PREDICTED: DAZ-associated protein 1 isoform ...  40.4    1.3   
ref|XP_004654940.1|  PREDICTED: DAZ-associated protein 1 isoform ...  40.4    1.3   
ref|XP_004632734.1|  PREDICTED: DAZ-associated protein 1 isoform ...  40.4    1.3   
ref|XP_008835384.1|  PREDICTED: DAZ-associated protein 1 isoform ...  40.4    1.3   
ref|XP_007130188.1|  PREDICTED: DAZ-associated protein 1 [Physete...  40.4    1.3   
ref|XP_005406030.2|  PREDICTED: DAZ-associated protein 1 isoform ...  40.4    1.3   
ref|XP_011286487.1|  PREDICTED: DAZ-associated protein 1 isoform ...  40.4    1.3   
ref|XP_005406027.2|  PREDICTED: DAZ-associated protein 1 isoform ...  40.4    1.3   
ref|XP_003422575.1|  PREDICTED: DAZ-associated protein 1 isoform ...  40.4    1.3   
ref|XP_012815264.1|  PREDICTED: DAZ-associated protein 1 isoform ...  40.4    1.3   
ref|XP_004462796.1|  PREDICTED: DAZ-associated protein 1 isoform ...  40.4    1.3   
gb|EGV99500.1|  DAZ-associated protein 1 [Cricetulus griseus]         40.4    1.3   
ref|XP_012878145.1|  PREDICTED: DAZ-associated protein 1 [Dipodom...  40.4    1.3   
ref|XP_012500917.1|  PREDICTED: DAZ-associated protein 1 isoform ...  40.4    1.3   
ref|XP_008835383.1|  PREDICTED: DAZ-associated protein 1 isoform ...  40.4    1.3   
ref|XP_004866010.1|  PREDICTED: DAZ-associated protein 1 isoform ...  40.4    1.3   
ref|XP_006514134.1|  PREDICTED: DAZ-associated protein 1 isoform ...  40.4    1.3   
ref|XP_011373488.1|  PREDICTED: DAZ-associated protein 1 isoform ...  40.4    1.3   
ref|XP_010842936.1|  PREDICTED: DAZ-associated protein 1 [Bison b...  40.4    1.3   
ref|XP_010996088.1|  PREDICTED: DAZ-associated protein 1 [Camelus...  40.4    1.3   
ref|XP_005962666.1|  PREDICTED: DAZ-associated protein 1 [Panthol...  40.4    1.3   
ref|XP_005259588.1|  PREDICTED: DAZ-associated protein 1 isoform ...  40.4    1.3   
ref|XP_006514133.1|  PREDICTED: DAZ-associated protein 1 isoform ...  40.4    1.3   
ref|XP_007952754.1|  PREDICTED: DAZ-associated protein 1 isoform ...  40.4    1.3   
ref|XP_011971524.1|  PREDICTED: DAZ-associated protein 1 isoform ...  40.4    1.3   
ref|XP_004632737.1|  PREDICTED: DAZ-associated protein 1 isoform ...  40.4    1.3   
ref|XP_005406029.2|  PREDICTED: DAZ-associated protein 1 isoform ...  40.4    1.3   
ref|NP_001116076.1|  DAZ-associated protein 1 isoform b [Mus musc...  40.4    1.3   
ref|XP_004059702.1|  PREDICTED: DAZ-associated protein 1 [Gorilla...  40.4    1.3   
ref|XP_012416105.1|  PREDICTED: DAZ-associated protein 1 isoform ...  40.4    1.3   
ref|XP_011852801.1|  PREDICTED: DAZ-associated protein 1 isoform ...  40.4    1.3   
ref|XP_007952755.1|  PREDICTED: DAZ-associated protein 1 isoform ...  40.4    1.3   
ref|XP_004325534.1|  PREDICTED: DAZ-associated protein 1 [Tursiop...  40.4    1.3   
ref|XP_008059023.1|  PREDICTED: DAZ-associated protein 1 isoform ...  40.4    1.3   
ref|XP_012815276.1|  PREDICTED: DAZ-associated protein 1 isoform ...  40.4    1.3   
ref|XP_010600161.1|  PREDICTED: DAZ-associated protein 1 isoform ...  40.4    1.3   
ref|NP_001179960.1|  DAZ-associated protein 1 [Bos taurus] >ref|X...  40.4    1.3   
ref|XP_004285910.1|  PREDICTED: DAZ-associated protein 1 isoform ...  40.4    1.3   
ref|XP_004614857.1|  PREDICTED: DAZ-associated protein 1 isoform ...  40.4    1.3   
ref|XP_010964942.1|  PREDICTED: DAZ-associated protein 1 [Camelus...  40.4    1.3   
ref|XP_008763345.1|  PREDICTED: DAZ-associated protein 1 isoform ...  40.4    1.3   
ref|XP_007992800.1|  PREDICTED: DAZ-associated protein 1 isoform ...  40.4    1.3   
ref|XP_012815282.1|  PREDICTED: DAZ-associated protein 1 isoform ...  40.4    1.3   
ref|XP_730072.1|  hypothetical protein [Plasmodium yoelii yoelii ...  40.4    1.3   
ref|XP_003461015.1|  PREDICTED: LOW QUALITY PROTEIN: DAZ-associat...  40.4    1.3   
ref|XP_006241031.1|  PREDICTED: DAZ-associated protein 1 isoform ...  40.4    1.3   
ref|NP_061832.2|  DAZ-associated protein 1 isoform b [Homo sapien...  40.4    1.3   
ref|XP_012815275.1|  PREDICTED: DAZ-associated protein 1 isoform ...  40.4    1.3   
ref|XP_006241032.1|  PREDICTED: DAZ-associated protein 1 isoform ...  40.4    1.4   
ref|XP_004778124.2|  PREDICTED: DAZ-associated protein 1 [Mustela...  40.4    1.4   
ref|XP_004632735.1|  PREDICTED: DAZ-associated protein 1 isoform ...  40.4    1.4   
ref|XP_001094635.2|  PREDICTED: DAZ-associated protein 1 isoform ...  40.4    1.4   
gb|KKR70998.1|  hypothetical protein UU12_C0011G0018 [Microgenoma...  38.5    1.4   
ref|XP_006928237.2|  PREDICTED: DAZ-associated protein 1 isoform ...  40.4    1.4   
ref|XP_007992801.1|  PREDICTED: DAZ-associated protein 1 isoform ...  40.4    1.4   
ref|XP_012815289.1|  PREDICTED: DAZ-associated protein 1 isoform ...  40.4    1.4   
ref|XP_012500918.1|  PREDICTED: DAZ-associated protein 1 isoform ...  40.4    1.4   
ref|XP_012394576.1|  PREDICTED: DAZ-associated protein 1 isoform ...  40.4    1.4   
gb|EHH29422.1|  Deleted in azoospermia-associated protein 1, part...  40.4    1.4   
gb|AAF78364.1|AF181719_1  DAZ associated protein 1 [Homo sapiens]     40.4    1.4   
ref|XP_005587453.1|  PREDICTED: DAZ-associated protein 1 isoform ...  40.4    1.4   
ref|NP_001231435.1|  DAZ-associated protein 1 [Sus scrofa] >ref|X...  40.4    1.4   
ref|XP_006876783.1|  PREDICTED: DAZ-associated protein 1 [Chrysoc...  40.4    1.4   
ref|XP_011971525.1|  PREDICTED: DAZ-associated protein 1 isoform ...  40.4    1.4   
ref|NP_001082088.1|  DAZ-associated protein 1 [Xenopus laevis] >s...  40.4    1.4   
ref|XP_007992799.1|  PREDICTED: DAZ-associated protein 1 isoform ...  40.4    1.4   
ref|NP_001006737.1|  DAZ-associated protein 1 [Xenopus (Silurana)...  40.4    1.4   
ref|XP_005587452.1|  PREDICTED: DAZ-associated protein 1 isoform ...  40.4    1.4   
gb|KFO31525.1|  Heterogeneous nuclear ribonucleoproteins A2/B1 [F...  40.0    1.4   
emb|CDQ57941.1|  unnamed protein product [Oncorhynchus mykiss]        40.4    1.4   
ref|XP_005633188.1|  PREDICTED: DAZ-associated protein 1 isoform ...  40.4    1.4   
ref|XP_007992797.1|  PREDICTED: DAZ-associated protein 1 isoform ...  40.4    1.4   
ref|XP_005633189.1|  PREDICTED: DAZ-associated protein 1 isoform ...  40.0    1.4   
ref|XP_012815291.1|  PREDICTED: DAZ-associated protein 1 isoform ...  40.0    1.5   
ref|XP_006746000.1|  PREDICTED: DAZ-associated protein 1, partial...  40.4    1.5   
ref|XP_011928275.1|  PREDICTED: DAZ-associated protein 1 isoform ...  40.0    1.5   
ref|XP_006764920.1|  PREDICTED: DAZ-associated protein 1 [Myotis ...  40.4    1.5   
ref|XP_005587454.1|  PREDICTED: DAZ-associated protein 1 isoform ...  40.0    1.5   
ref|XP_005587450.1|  PREDICTED: DAZ-associated protein 1 isoform ...  40.0    1.5   
ref|XP_563821.4|  AGAP002374-PA [Anopheles gambiae str. PEST] >gb...  40.0    1.5   
ref|XP_005587451.1|  PREDICTED: DAZ-associated protein 1 isoform ...  40.0    1.5   
ref|XP_012033849.1|  PREDICTED: LOW QUALITY PROTEIN: DAZ-associat...  40.0    1.5   
gb|ACI34285.1|  DAZ-associated protein 1 [Salmo salar]                40.4    1.5   
gb|EUB60328.1|  Heterogeneous nuclear ribonucleoprotein A/B [Echi...  40.4    1.5   
gb|EFB16942.1|  hypothetical protein PANDA_012747, partial [Ailur...  40.0    1.5   
gb|EKF33681.1|  RNA-binding protein, putative [Trypanosoma cruzi ...  40.0    1.5   
gb|ELK29445.1|  DAZ-associated protein 1 [Myotis davidii]             40.0    1.6   
gb|KKP58055.1|  RNP-1 like protein RNA-binding protein [Parcubact...  38.1    1.6   
gb|KKR17749.1|  hypothetical protein UT47_C0005G0014 [candidate d...  37.7    1.6   
gb|KNB43638.1|  hypothetical protein JH06_2463 [Blastocystis sp. ...  39.7    1.6   
ref|XP_007524863.1|  PREDICTED: DAZ-associated protein 1 [Erinace...  40.0    1.6   
ref|XP_008520166.1|  PREDICTED: heterogeneous nuclear ribonucleop...  40.0    1.7   
gb|KKQ30092.1|  RNP-1 like protein RNA-binding protein [Microgeno...  37.7    1.7   
emb|CEG80975.1|  hypothetical protein RMATCC62417_15233 [Rhizopus...  40.0    1.7   
ref|WP_011447420.1|  RNA recognition motif-containing protein [Me...  37.7    1.7   
gb|KKQ59399.1|  Glycine-rich RNA-binding protein GRP1A [Parcubact...  38.1    1.7   
dbj|BAF57616.1|  pRM10 protein [Dugesia japonica]                     39.7    1.8   
gb|KFK36614.1|  hypothetical protein AALP_AA4G146600 [Arabis alpina]  40.0    1.9   
ref|XP_012659820.1|  PREDICTED: heterogeneous nuclear ribonucleop...  39.7    1.9   
gb|KKS99150.1|  RNP-1 like protein RNA-binding protein [Parcubact...  37.7    2.0   
emb|CDZ98709.1|  Cyclophilin-type peptidyl-prolyl cis-trans isome...  38.5    2.0   
ref|XP_007505394.1|  PREDICTED: heterogeneous nuclear ribonucleop...  39.7    2.0   
ref|XP_008651987.1|  PREDICTED: DAZ-associated protein 1-like [Ze...  39.7    2.0   
ref|WP_013625605.1|  RNA-binding protein [Syntrophobotulus glycol...  37.4    2.0   
dbj|GAD81376.1|  putative RNA-binding protein [Vibrio ezurae NBRC...  37.7    2.1   
ref|NP_001270102.1|  uncharacterized protein LOC101925647 [Macaca...  39.7    2.1   
ref|XP_003296044.1|  hypothetical protein PTT_04470 [Pyrenophora ...  38.5    2.2   
ref|XP_007980026.1|  PREDICTED: heterogeneous nuclear ribonucleop...  39.7    2.2   
ref|XP_007240323.1|  PREDICTED: DAZ-associated protein 1 [Astyana...  39.7    2.2   
ref|XP_007505397.1|  PREDICTED: heterogeneous nuclear ribonucleop...  39.7    2.2   
ref|XP_005872032.1|  PREDICTED: heterogeneous nuclear ribonucleop...  39.7    2.2   
ref|XP_004604316.1|  PREDICTED: heterogeneous nuclear ribonucleop...  39.7    2.2   
gb|KKU53925.1|  RNP-1 like protein RNA-binding protein [Parcubact...  37.4    2.2   
gb|EDK98637.1|  mCG119114, isoform CRA_a, partial [Mus musculus]      39.7    2.3   
ref|XP_007896923.1|  PREDICTED: DAZ-associated protein 1 [Callorh...  39.7    2.4   
ref|XP_007010457.1|  Pentatricopeptide repeat-containing protein ...  39.7    2.4   
dbj|BAF82118.1|  unnamed protein product [Homo sapiens]               39.7    2.4   
ref|XP_007010458.1|  Pentatricopeptide repeat-containing protein ...  40.0    2.4   
ref|XP_010585512.1|  PREDICTED: heterogeneous nuclear ribonucleop...  39.3    2.4   
ref|XP_003387203.1|  PREDICTED: ribonucleoprotein PTB-binding 1-l...  39.7    2.4   
ref|XP_006832261.1|  PREDICTED: heterogeneous nuclear ribonucleop...  39.7    2.5   
ref|XP_004702786.1|  PREDICTED: heterogeneous nuclear ribonucleop...  39.7    2.5   
ref|XP_005872031.1|  PREDICTED: heterogeneous nuclear ribonucleop...  39.3    2.5   
ref|XP_005957731.1|  PREDICTED: heterogeneous nuclear ribonucleop...  39.7    2.5   
ref|NP_001127331.1|  heterogeneous nuclear ribonucleoproteins A2/...  39.3    2.5   
gb|ERE66050.1|  heterogeneous nuclear ribonucleoprotein A2/B1-lik...  39.3    2.5   
ref|XP_007629134.1|  PREDICTED: heterogeneous nuclear ribonucleop...  39.3    2.5   
gb|AAH00506.3|  HNRNPA2B1 protein [Homo sapiens] >gb|AIC59017.1| ...  39.3    2.5   
ref|XP_012381164.1|  PREDICTED: heterogeneous nuclear ribonucleop...  39.3    2.5   
gb|AAI06633.1|  MGC52881 protein [Xenopus laevis]                     39.3    2.5   
ref|XP_008332016.1|  PREDICTED: DAZ-associated protein 1 [Cynoglo...  39.7    2.5   
ref|XP_012406864.1|  PREDICTED: heterogeneous nuclear ribonucleop...  39.3    2.5   
ref|XP_008259775.1|  PREDICTED: heterogeneous nuclear ribonucleop...  39.3    2.5   
ref|XP_002713772.1|  PREDICTED: heterogeneous nuclear ribonucleop...  39.3    2.5   
ref|XP_004582487.1|  PREDICTED: heterogeneous nuclear ribonucleop...  39.3    2.5   
ref|XP_008146169.1|  PREDICTED: LOW QUALITY PROTEIN: heterogeneou...  39.3    2.6   
ref|XP_013245664.1|  RNA-binding domain-containing protein [Tille...  38.1    2.6   
ref|NP_112533.1|  heterogeneous nuclear ribonucleoproteins A2/B1 ...  39.3    2.6   
ref|XP_010585509.1|  PREDICTED: heterogeneous nuclear ribonucleop...  39.3    2.6   
ref|XP_004460014.1|  PREDICTED: heterogeneous nuclear ribonucleop...  39.3    2.6   
ref|XP_007627984.1|  PREDICTED: heterogeneous nuclear ribonucleop...  39.3    2.6   
ref|XP_004323805.1|  PREDICTED: heterogeneous nuclear ribonucleop...  38.9    2.6   
ref|XP_004762582.1|  PREDICTED: heterogeneous nuclear ribonucleop...  39.3    2.6   
ref|XP_004673136.1|  PREDICTED: heterogeneous nuclear ribonucleop...  39.3    2.7   
ref|XP_005155755.1|  PREDICTED: DAZ-associated protein 1 isoform ...  39.3    2.7   
ref|XP_008520170.1|  PREDICTED: heterogeneous nuclear ribonucleop...  39.3    2.7   
ref|XP_010711105.1|  PREDICTED: heterogeneous nuclear ribonucleop...  39.3    2.7   
ref|XP_004676845.1|  PREDICTED: heterogeneous nuclear ribonucleop...  39.3    2.7   
ref|XP_006138439.1|  PREDICTED: heterogeneous nuclear ribonucleop...  39.3    2.7   
ref|XP_012694970.1|  PREDICTED: DAZ-associated protein 1 isoform ...  39.7    2.7   
gb|KIM87119.1|  hypothetical protein PILCRDRAFT_815579 [Piloderma...  38.1    2.7   
ref|XP_009000503.1|  PREDICTED: heterogeneous nuclear ribonucleop...  39.3    2.7   
gb|KDO79201.1|  hypothetical protein CISIN_1g026534mg [Citrus sin...  38.9    2.7   
ref|XP_012369102.1|  PREDICTED: heterogeneous nuclear ribonucleop...  39.3    2.7   
gb|EMP41569.1|  Heterogeneous nuclear ribonucleoproteins A2/B1 [C...  39.3    2.8   
ref|XP_006138440.1|  PREDICTED: heterogeneous nuclear ribonucleop...  39.3    2.8   
ref|XP_013018153.1|  RNA-binding protein [Schizosaccharomyces oct...  39.7    2.8   
ref|XP_011390640.1|  hypothetical protein UMAG_04149 [Ustilago ma...  38.9    2.8   
ref|XP_007053723.1|  PREDICTED: heterogeneous nuclear ribonucleop...  39.3    2.8   
gb|EHB07204.1|  Heterogeneous nuclear ribonucleoproteins A2/B1, p...  39.3    2.8   
ref|XP_012694971.1|  PREDICTED: DAZ-associated protein 1 isoform ...  39.3    2.8   
ref|XP_008168427.1|  PREDICTED: heterogeneous nuclear ribonucleop...  39.3    2.9   
ref|NP_001079471.1|  heterogeneous nuclear ribonucleoprotein A2 h...  39.3    2.9   
ref|NP_001098083.1|  heterogeneous nuclear ribonucleoproteins A2/...  39.3    2.9   
ref|XP_005022144.1|  PREDICTED: heterogeneous nuclear ribonucleop...  39.3    2.9   
ref|XP_010796565.1|  PREDICTED: heterogeneous nuclear ribonucleop...  39.3    2.9   
ref|XP_009000508.1|  PREDICTED: heterogeneous nuclear ribonucleop...  39.3    2.9   
ref|XP_006066117.1|  PREDICTED: heterogeneous nuclear ribonucleop...  39.3    2.9   
ref|XP_006066111.1|  PREDICTED: heterogeneous nuclear ribonucleop...  39.3    2.9   
ref|XP_012735448.1|  PREDICTED: DAZ-associated protein 1 isoform ...  39.3    2.9   
ref|XP_006077789.1|  PREDICTED: heterogeneous nuclear ribonucleop...  39.3    2.9   
ref|XP_012694968.1|  PREDICTED: DAZ-associated protein 1 isoform ...  39.3    3.0   
ref|XP_012735446.1|  PREDICTED: DAZ-associated protein 1 isoform ...  39.3    3.0   
ref|XP_013862731.1|  PREDICTED: DAZ-associated protein 1 isoform ...  39.3    3.0   
dbj|BAF79678.1|  heterogeneous nuclear ribonucleoprotein B0b [Rat...  39.3    3.0   
ref|XP_005868816.1|  PREDICTED: heterogeneous nuclear ribonucleop...  38.9    3.0   
ref|XP_008404863.1|  PREDICTED: DAZ-associated protein 1 isoform ...  39.3    3.1   
gb|KKR72967.1|  hypothetical protein UU16_C0034G0001, partial [Mi...  37.0    3.1   
ref|XP_012694969.1|  PREDICTED: DAZ-associated protein 1 isoform ...  39.3    3.1   
ref|XP_011472319.1|  PREDICTED: DAZ-associated protein 1 isoform ...  39.3    3.1   
ref|XP_009304077.1|  PREDICTED: DAZ-associated protein 1 isoform ...  39.3    3.1   
gb|KKW35176.1|  hypothetical protein UY82_C0047G0008 [Parcubacter...  37.0    3.1   
ref|XP_011472320.1|  PREDICTED: DAZ-associated protein 1 isoform ...  39.3    3.1   
ref|NP_001026156.1|  heterogeneous nuclear ribonucleoproteins A2/...  39.3    3.1   
ref|XP_004067962.1|  PREDICTED: DAZ-associated protein 1 isoform ...  39.3    3.2   
ref|XP_006265410.1|  PREDICTED: heterogeneous nuclear ribonucleop...  39.3    3.2   
gb|KKF29087.1|  DAZ-associated protein 1 [Larimichthys crocea]        39.3    3.2   
gb|EAW93838.1|  heterogeneous nuclear ribonucleoprotein A2/B1, is...  38.9    3.2   
ref|XP_005154202.1|  PREDICTED: heterogeneous nuclear ribonucleop...  39.3    3.2   
ref|XP_010575873.1|  PREDICTED: heterogeneous nuclear ribonucleop...  39.3    3.2   
ref|XP_008259774.1|  PREDICTED: heterogeneous nuclear ribonucleop...  39.3    3.2   
ref|XP_004582488.1|  PREDICTED: heterogeneous nuclear ribonucleop...  39.3    3.2   
ref|XP_009867724.1|  PREDICTED: heterogeneous nuclear ribonucleop...  39.3    3.2   
ref|XP_012735449.1|  PREDICTED: DAZ-associated protein 1 isoform ...  39.3    3.2   
ref|XP_005480939.1|  PREDICTED: heterogeneous nuclear ribonucleop...  39.3    3.2   
ref|XP_013226257.1|  PREDICTED: heterogeneous nuclear ribonucleop...  39.3    3.2   
ref|XP_009304078.1|  PREDICTED: DAZ-associated protein 1 isoform ...  39.3    3.2   
gb|EGV94722.1|  Heterogeneous nuclear ribonucleoproteins A2/B1 [C...  38.9    3.2   
ref|XP_012735447.1|  PREDICTED: DAZ-associated protein 1 isoform ...  39.3    3.2   
ref|XP_008839716.1|  PREDICTED: heterogeneous nuclear ribonucleop...  39.3    3.2   
ref|XP_007268070.1|  RNA-binding domain-containing protein [Fomit...  39.3    3.3   
ref|XP_006265413.1|  PREDICTED: heterogeneous nuclear ribonucleop...  38.9    3.3   
ref|XP_005513536.1|  PREDICTED: heterogeneous nuclear ribonucleop...  39.3    3.3   
ref|XP_005730294.1|  PREDICTED: DAZ-associated protein 1 isoform ...  39.3    3.3   
ref|XP_005868817.1|  PREDICTED: heterogeneous nuclear ribonucleop...  38.9    3.3   
ref|XP_004702787.1|  PREDICTED: heterogeneous nuclear ribonucleop...  38.9    3.3   
ref|XP_006102708.1|  PREDICTED: heterogeneous nuclear ribonucleop...  38.9    3.3   
ref|XP_013796827.1|  PREDICTED: heterogeneous nuclear ribonucleop...  38.9    3.4   
ref|XP_012306874.1|  PREDICTED: heterogeneous nuclear ribonucleop...  38.9    3.4   
gb|EAZ32021.1|  hypothetical protein OsJ_16200 [Oryza sativa Japo...  39.3    3.4   
gb|KKQ92747.1|  RNP-1 like protein RNA-binding protein [Microgeno...  37.4    3.4   
gb|EPY87708.1|  hypothetical protein CB1_000224010 [Camelus ferus]    39.3    3.4   
ref|XP_012757110.1|  hypothetical protein SAMD00019534_023580 [Ac...  39.3    3.4   
emb|CBY14172.1|  unnamed protein product [Oikopleura dioica]          39.3    3.4   
ref|XP_006102707.1|  PREDICTED: heterogeneous nuclear ribonucleop...  38.9    3.4   
ref|XP_005993138.1|  PREDICTED: heterogeneous nuclear ribonucleop...  38.9    3.4   
ref|XP_010575880.1|  PREDICTED: heterogeneous nuclear ribonucleop...  38.9    3.4   
ref|XP_012957476.1|  PREDICTED: heterogeneous nuclear ribonucleop...  38.9    3.5   
ref|XP_009674729.1|  PREDICTED: heterogeneous nuclear ribonucleop...  38.9    3.5   
ref|XP_005730296.1|  PREDICTED: DAZ-associated protein 1 isoform ...  39.3    3.5   
gb|AAI53535.1|  Dazap1 protein [Danio rerio]                          38.9    3.5   
ref|XP_004738433.1|  PREDICTED: heterogeneous nuclear ribonucleop...  38.9    3.5   
ref|XP_003430726.1|  PREDICTED: heterogeneous nuclear ribonucleop...  38.9    3.5   
ref|NP_001053909.1|  Os04g0620700 [Oryza sativa Japonica Group] >...  39.3    3.5   
ref|XP_013862733.1|  PREDICTED: DAZ-associated protein 1 isoform ...  39.3    3.5   
ref|XP_005730293.1|  PREDICTED: DAZ-associated protein 1 isoform ...  39.3    3.5   
ref|XP_005993137.1|  PREDICTED: heterogeneous nuclear ribonucleop...  38.9    3.6   
ref|XP_005730292.1|  PREDICTED: DAZ-associated protein 1 isoform ...  39.3    3.6   
ref|NP_872591.1|  heterogeneous nuclear ribonucleoproteins A2/B1 ...  38.9    3.6   
emb|CAH66982.1|  H0714H04.9 [Oryza sativa Indica Group]               39.3    3.6   
ref|XP_004185942.1|  protein HRB1, putative [Entamoeba invadens I...  38.9    3.6   
gb|AAC26867.1|  heterogenous nuclear ribonucleoprotein A2/B1 [Mus...  38.9    3.6   
ref|XP_006103816.1|  PREDICTED: heterogeneous nuclear ribonucleop...  38.9    3.6   
ref|XP_009907006.1|  PREDICTED: heterogeneous nuclear ribonucleop...  38.9    3.6   
gb|KOM19822.1|  hypothetical protein XA68_3589 [Ophiocordyceps un...  38.9    3.6   
ref|NP_002128.1|  heterogeneous nuclear ribonucleoproteins A2/B1 ...  38.9    3.7   
ref|XP_010585511.1|  PREDICTED: heterogeneous nuclear ribonucleop...  38.9    3.7   
ref|XP_007505396.1|  PREDICTED: heterogeneous nuclear ribonucleop...  38.9    3.7   
ref|NP_058086.2|  heterogeneous nuclear ribonucleoproteins A2/B1 ...  38.9    3.7   
ref|XP_005993135.1|  PREDICTED: heterogeneous nuclear ribonucleop...  38.9    3.7   
ref|XP_001510026.1|  PREDICTED: heterogeneous nuclear ribonucleop...  38.9    3.7   
emb|CAJ82537.1|  heterogeneous nuclear ribonucleoprotein A2/B1 [X...  38.9    3.7   
ref|XP_013862730.1|  PREDICTED: DAZ-associated protein 1 isoform ...  38.9    3.7   
gb|ELR50605.1|  Heterogeneous nuclear ribonucleoproteins A2/B1, p...  38.9    3.7   
ref|NP_001016926.2|  heterogeneous nuclear ribonucleoproteins A2/...  38.9    3.7   
ref|XP_005041037.1|  PREDICTED: heterogeneous nuclear ribonucleop...  38.9    3.7   
ref|XP_009488704.1|  PREDICTED: heterogeneous nuclear ribonucleop...  38.9    3.7   
ref|XP_010397320.1|  PREDICTED: heterogeneous nuclear ribonucleop...  38.9    3.7   
ref|XP_005155753.1|  PREDICTED: DAZ-associated protein 1 isoform ...  38.9    3.7   
gb|AAI14332.1|  Dazap1 protein [Danio rerio]                          38.9    3.7   
ref|XP_009969866.1|  PREDICTED: heterogeneous nuclear ribonucleop...  38.9    3.7   
dbj|BAF79677.1|  heterogeneous nuclear ribonucleoprotein B0a [Rat...  38.9    3.7   
gb|KFP34332.1|  Heterogeneous nuclear ribonucleoproteins A2/B1, p...  38.5    3.7   
gb|ELW68462.1|  Heterogeneous nuclear ribonucleoproteins A2/B1 [T...  38.9    3.7   
dbj|BAK62302.1|  heterogeneous nuclear ribonucleoproteins A2/B1 [...  38.9    3.8   
ref|XP_006265412.1|  PREDICTED: heterogeneous nuclear ribonucleop...  38.9    3.8   
ref|XP_005421803.1|  PREDICTED: heterogeneous nuclear ribonucleop...  38.9    3.8   
gb|KDD74158.1|  hypothetical protein H632_c1522p0 [Helicosporidiu...  38.1    3.8   
gb|KFQ47092.1|  Heterogeneous nuclear ribonucleoproteins A2/B1, p...  38.9    3.8   
ref|XP_013796829.1|  PREDICTED: heterogeneous nuclear ribonucleop...  38.9    3.8   


 >emb|CED83251.1| RNA recognition motif domain [Xanthophyllomyces dendrorhous]
Length=249

 Score =  506 bits (1302),  Expect = 1e-179, Method: Compositional matrix adjust.
 Identities = 248/249 (99%), Positives = 249/249 (100%), Gaps = 0/249 (0%)

Query  1    MNTLALTTAKKMVFGPRNYIHQSIFTRLIHSSLPVTARSFARGARDLRASSLRTNASLQH  60
            MNTLALTTAKKMVFGPRNYIHQSIFT+LIHSSLPVTARSFARGARDLRASSLRTNASLQH
Sbjct  1    MNTLALTTAKKMVFGPRNYIHQSIFTQLIHSSLPVTARSFARGARDLRASSLRTNASLQH  60

Query  61   ALADEPILEDSVTIPSISASAFSSSTSTPLKSTALLDTDTNGSSRVDQTLFVAGIPNVAR  120
            ALADEPILEDSVTIPSISASAFSSSTSTPLKSTALLDTDTNGSSRVDQTLFVAGIPNVAR
Sbjct  61   ALADEPILEDSVTIPSISASAFSSSTSTPLKSTALLDTDTNGSSRVDQTLFVAGIPNVAR  120

Query  121  EQHLTEYFEDLGIIVRDVFVPSAFNQNGQKRKHNRGFGFVEVGTVKEHQQLIRMSKNKKI  180
            EQHLTEYFEDLGIIVRDVFVPSAFNQNGQKRKHNRGFGFVEVGTVKEHQQLIRMSKNKKI
Sbjct  121  EQHLTEYFEDLGIIVRDVFVPSAFNQNGQKRKHNRGFGFVEVGTVKEHQQLIRMSKNKKI  180

Query  181  EFDLKPAKWDIERARSYGTPSPSIAPKIESKPKDSKARPDDKARSRSAIEPQGTEKKKAR  240
            EFDLKPAKWDIERARSYGTPSPSIAPKIESKPKDSKARPDDKARSRSAIEPQGTEKKKAR
Sbjct  181  EFDLKPAKWDIERARSYGTPSPSIAPKIESKPKDSKARPDDKARSRSAIEPQGTEKKKAR  240

Query  241  RRIPSFSRR  249
            RRIPSFSRR
Sbjct  241  RRIPSFSRR  249


>emb|CDS29517.1| heterogeneous nuclear ribonucleoprotein [Hymenolepis microstoma]
Length=558

 Score = 46.2 bits (108),  Expect = 0.018, Method: Compositional matrix adjust.
 Identities = 39/122 (32%), Positives = 56/122 (46%), Gaps = 24/122 (20%)

Query  97   DTDTNGSSRVDQTLFVAGIPNVAREQHLTEYFEDLGIIVRDVFVPSAFNQNGQKRKHNRG  156
            + + +GS R ++ +FV  +     EQ+LT+YF   G +V        F + G    H+RG
Sbjct  41   ENNFSGSPRPNKKIFVGALTPETTEQNLTDYFSKFGELVSCAI--KVFRETG----HSRG  94

Query  157  FGFV---------EVGTVKEHQQLIRMSKNKKIEFDLKPAKW--DIERARSYGTPSPSIA  205
            FGFV         +V ++ EH         KKI  D KPAK   D +R    G   P + 
Sbjct  95   FGFVIFKNEESVKKVLSIPEH-----FINGKKI--DPKPAKCPKDTQRKVFVGGLDPGVT  147

Query  206  PK  207
            PK
Sbjct  148  PK  149


>gb|KKQ87190.1| Glycine-rich RNA-binding protein GRP1A [Parcubacteria bacterium 
GW2011_GWF2_38_8]
Length=112

 Score = 42.7 bits (99),  Expect = 0.040, Method: Compositional matrix adjust.
 Identities = 23/72 (32%), Positives = 37/72 (51%), Gaps = 6/72 (8%)

Query  110  LFVAGIPNVAREQHLTEYFEDLGIIVRDVFVPSAFNQNGQKRKHNRGFGFVEVGTVKEHQ  169
            L+V G+P   +E  L E+F   G +V  V +    +        ++GFGFVE+ T +E Q
Sbjct  5    LYVGGLPYSVQEDALKEHFAQAGNVVSSVIIMDKMS------GRSKGFGFVEMATQEEAQ  58

Query  170  QLIRMSKNKKIE  181
              I M  +++ E
Sbjct  59   NAISMFNDQEFE  70


>gb|KKW30655.1| RNP-1 like protein RNA-binding protein [Parcubacteria (Uhrbacteria) 
bacterium GW2011_GWD2_52_7]
Length=85

 Score = 42.4 bits (98),  Expect = 0.050, Method: Compositional matrix adjust.
 Identities = 26/65 (40%), Positives = 34/65 (52%), Gaps = 6/65 (9%)

Query  110  LFVAGIPNVAREQHLTEYFEDLGIIVRDVFVPSAFNQNGQKRKHNRGFGFVEVGTVKEHQ  169
            +FV GIP  A E  L  +F   G +V  VF+P    + G+KR    GFGFVE  T  E  
Sbjct  7    VFVGGIPYAATEDELKAHFSAAGTVV-SVFLPIE-KETGRKR----GFGFVEFNTPDEQD  60

Query  170  QLIRM  174
              ++M
Sbjct  61   NAVKM  65


>ref|XP_007665524.1| PREDICTED: DAZ-associated protein 1 [Ornithorhynchus anatinus]
Length=446

 Score = 44.3 bits (103),  Expect = 0.088, Method: Compositional matrix adjust.
 Identities = 34/125 (27%), Positives = 58/125 (46%), Gaps = 31/125 (25%)

Query  98   TDTNGSSRVDQTLFVAGIPNVAREQHLTEYFEDLGIIVRDVFVPSAFNQNGQKRKHNRGF  157
            +D N S+++    FV GIP+   E  L EYF+  G++   V +  A      +++  RGF
Sbjct  146  SDNNKSNKI----FVGGIPHNCGETELREYFKKFGVVSEVVMIYDA------EKQRPRGF  195

Query  158  GFVEVGTVKEHQQLIRMSKNKKIEFDLKPAKWDIERARSYGTPSPSIAPKIESKPKDSKA  217
            GF+      E QQ +  + N     D+   K +++RA                +P+DSK+
Sbjct  196  GFITF----EDQQSVDQAVNMHFH-DIMGKKVEVKRA----------------EPRDSKS  234

Query  218  RPDDK  222
            +P  +
Sbjct  235  QPQGQ  239


>ref|XP_002367750.1| U1 small nuclear ribonucleoprotein, putative [Toxoplasma gondii 
ME49]
 gb|EPR61412.1| RNA recognition motif-containing protein [Toxoplasma gondii GT1]
 gb|EPT29996.1| RNA recognition motif-containing protein [Toxoplasma gondii ME49]
 8 more sequence titles

gb|ESS33244.1| RNA recognition motif-containing protein [Toxoplasma gondii VEG]
 gb|KFG33620.1| RNA recognition motif-containing protein [Toxoplasma gondii p89]
 gb|KFG36767.1| RNA recognition motif-containing protein [Toxoplasma gondii FOU]
 gb|KFG38203.1| RNA recognition motif-containing protein [Toxoplasma gondii GAB2-2007-GAL-DOM2]
 gb|KFG59161.1| RNA recognition motif-containing protein [Toxoplasma gondii RUB]
 gb|KFH00449.1| RNA recognition motif-containing protein [Toxoplasma gondii VAND]
 gb|KFH07522.1| RNA recognition motif-containing protein [Toxoplasma gondii MAS]
 tpe|CEL73692.1| TPA: U1 small nuclear ribonucleoprotein, putative [Toxoplasma 
gondii VEG]

Length=274

 Score = 43.1 bits (100),  Expect = 0.12, Method: Compositional matrix adjust.
 Identities = 34/112 (30%), Positives = 54/112 (48%), Gaps = 9/112 (8%)

Query  108  QTLFVAGIPNVAREQHLTEYFEDLGIIVRDVFVPSAFNQNGQKRKHNRGFGFVEVGTVKE  167
            +TLFV GI     E+ L   FE  G I R   +   +++NG+     RG+GF+E    ++
Sbjct  98   RTLFVGGISYDTTEKKLKREFEQYGSIKRVRLI---YDRNGKP----RGYGFIEFENDRD  150

Query  168  HQQLIRMSKNKKIEFDLKPAKWDIERARSYGTPSPSIAPKIESKPKDSKARP  219
             ++  + +  KKI  D +    D+ERAR+     P        KP+ S  +P
Sbjct  151  MKEAYKNADGKKI--DGRRVLVDVERARTVPGWLPRRLGGGRGKPRGSNTKP  200


>emb|CDH59702.1| rna-binding domain-containing protein [Lichtheimia corymbifera 
JMRC:FSU:9682]
Length=725

 Score = 43.5 bits (101),  Expect = 0.14, Method: Compositional matrix adjust.
 Identities = 43/144 (30%), Positives = 65/144 (45%), Gaps = 22/144 (15%)

Query  95   LLDTDTNGSSRVD-QTLFVAGIPNVAREQHLTEYFEDLGIIVRDVFVPSAFNQNGQKRK-  152
            + + D+N S  V   TLFV  +P  A  + L E+F D+G I R+ FV +  ++   K   
Sbjct  1    MANDDSNSSKPVAVSTLFVGNLPRNATSKQLEEFFSDIGPI-RNCFVVADRSKGKVKEDD  59

Query  153  ---HNRGFGFVEVGTVKEHQQLIRMSKNKK-----IEFDLKPAKWDIERARSYGTPSPSI  204
                N+G G+V      + QQ I   KN K     ++ D    K  +   R    PS ++
Sbjct  60   PQFQNKGVGYVHFAVTDDAQQAIEKLKNAKFLGRRLKLDYAQRKSALGEKRQ--KPSSAL  117

Query  205  APKIESKPKDSKARPDDKARSRSA  228
            A   E         P+ KAR+R+A
Sbjct  118  AIPEE---------PNKKARTRAA  132


>ref|XP_008887446.1| RNA recognition motif-containing protein [Hammondia hammondi]
 gb|KEP61972.1| RNA recognition motif-containing protein [Hammondia hammondi]
Length=274

 Score = 43.1 bits (100),  Expect = 0.14, Method: Compositional matrix adjust.
 Identities = 34/112 (30%), Positives = 54/112 (48%), Gaps = 9/112 (8%)

Query  108  QTLFVAGIPNVAREQHLTEYFEDLGIIVRDVFVPSAFNQNGQKRKHNRGFGFVEVGTVKE  167
            +TLFV GI     E+ L   FE  G I R   +   +++NG+     RG+GF+E    ++
Sbjct  98   RTLFVGGISYDTTEKKLRREFEQYGSIKRVRLI---YDRNGKP----RGYGFIEFENDRD  150

Query  168  HQQLIRMSKNKKIEFDLKPAKWDIERARSYGTPSPSIAPKIESKPKDSKARP  219
             ++  + +  KKI  D +    D+ERAR+     P        KP+ S  +P
Sbjct  151  MKEAYKNADGKKI--DGRRVLVDVERARTVPGWLPRRLGGGRGKPRGSNTKP  200


>gb|KKR09175.1| RNA-binding protein (RRM domain) [Peregrinibacteria bacterium 
GW2011_GWF2_39_17]
 gb|KKR24619.1| RNA-binding protein (RRM domain) [Peregrinibacteria bacterium 
GW2011_GWE2_39_6]
Length=107

 Score = 41.2 bits (95),  Expect = 0.15, Method: Compositional matrix adjust.
 Identities = 25/72 (35%), Positives = 34/72 (47%), Gaps = 6/72 (8%)

Query  110  LFVAGIPNVAREQHLTEYFEDLGIIVRDVFVPSAFNQNGQKRKHNRGFGFVEVGTVKEHQ  169
            L+V GIP    E+ L   F   G +V    +   F+        +RGFGFVE+ T  E Q
Sbjct  5    LYVGGIPYRTTEEALRAAFSQAGTVVSVKIITDKFS------GRSRGFGFVEMETEAEGQ  58

Query  170  QLIRMSKNKKIE  181
            + I M   K+ E
Sbjct  59   KAIEMWHGKEFE  70


>gb|KKS42347.1| RRM domain-containing RNA-binding protein [Parcubacteria (Kuenenbacteria) 
bacterium GW2011_GWA2_42_15]
Length=111

 Score = 40.8 bits (94),  Expect = 0.18, Method: Compositional matrix adjust.
 Identities = 24/71 (34%), Positives = 35/71 (49%), Gaps = 6/71 (8%)

Query  110  LFVAGIPNVAREQHLTEYFEDLGIIVRDVFVPSAFNQNGQKRKHNRGFGFVEVGTVKEHQ  169
            LFV  +P  A +Q LTE F   G +V    V + FN        ++GFGFVE+ T +E  
Sbjct  5    LFVGNLPYEATDQSLTELFSQSGTVVSASVVINKFN------NRSKGFGFVEMSTQEEAN  58

Query  170  QLIRMSKNKKI  180
              I     +++
Sbjct  59   AAIETLNGQEM  69


>gb|KKQ21192.1| RNP-1 like protein RNA-binding protein [Parcubacteria bacterium 
GW2011_GWC1_36_9]
 gb|KKQ27188.1| RNP-1 like protein RNA-binding protein [Parcubacteria bacterium 
GW2011_GWB1_37_13]
 gb|KKQ33720.1| RNP-1 like protein RNA-binding protein [Parcubacteria (Nomurabacteria) 
bacterium GW2011_GWA1_37_20]
 gb|KKQ47874.1| RNP-1 like protein RNA-binding protein [Parcubacteria (Yanofskybacteria) 
bacterium GW2011_GWC2_37_9]
Length=107

 Score = 40.8 bits (94),  Expect = 0.18, Method: Compositional matrix adjust.
 Identities = 23/72 (32%), Positives = 36/72 (50%), Gaps = 6/72 (8%)

Query  110  LFVAGIPNVAREQHLTEYFEDLGIIVRDVFVPSAFNQNGQKRKHNRGFGFVEVGTVKEHQ  169
            L+V G+P   +E  L E F   G +V  V +    +        ++GFGFVE+ T  E Q
Sbjct  5    LYVGGLPYSTQEGALKELFAQAGNVVSAVIIMDKMS------GRSKGFGFVEMATNDEAQ  58

Query  170  QLIRMSKNKKIE  181
            + I M  +++ E
Sbjct  59   KAISMFNDQEFE  70


>gb|KKP64586.1| RNP-1 like protein RNA-binding protein [Parcubacteria (Nomurabacteria) 
bacterium GW2011_GWF2_35_12]
 gb|KKP72110.1| RNP-1 like protein RNA-binding protein [Parcubacteria (Nomurabacteria) 
bacterium GW2011_GWB1_35_20]
 gb|KKP76423.1| RNP-1 like protein RNA-binding protein [Parcubacteria bacterium 
GW2011_GWC1_35_21]
 gb|KKP77525.1| RNP-1 like protein RNA-binding protein [Parcubacteria (Nomurabacteria) 
bacterium GW2011_GWC2_35_35]
 gb|KKP85334.1| RNP-1 like protein RNA-binding protein [Parcubacteria bacterium 
GW2011_GWD2_35_7]
 gb|KKP98087.1| RNP-1 like protein RNA-binding protein [Parcubacteria (Nomurabacteria) 
bacterium GW2011_GWA1_36_15]
Length=112

 Score = 40.8 bits (94),  Expect = 0.19, Method: Compositional matrix adjust.
 Identities = 22/72 (31%), Positives = 35/72 (49%), Gaps = 6/72 (8%)

Query  110  LFVAGIPNVAREQHLTEYFEDLGIIVRDVFVPSAFNQNGQKRKHNRGFGFVEVGTVKEHQ  169
            L+V G+P   +E  L E F   G +   V +    +        ++GFGFVE+ T +E Q
Sbjct  5    LYVGGLPYSVQEDALKELFAQAGNVTSAVIIMDKMS------GRSKGFGFVEMATAEEAQ  58

Query  170  QLIRMSKNKKIE  181
              I M  +++ E
Sbjct  59   AAISMFNDQEFE  70


>emb|CDS07137.1| hypothetical protein LRAMOSA09660 [Absidia idahoensis var. thermophila]
Length=759

 Score = 43.1 bits (100),  Expect = 0.19, Method: Compositional matrix adjust.
 Identities = 39/118 (33%), Positives = 57/118 (48%), Gaps = 11/118 (9%)

Query  109  TLFVAGIPNVAREQHLTEYFEDLGIIVRDVFVPSAFNQNGQKRK----HNRGFGFVEVGT  164
            TLFV  +P  A  + L E+F D+G I R+ FV +  ++   K       N+G G+V    
Sbjct  17   TLFVGNLPRNATSKQLEEFFSDIGPI-RNCFVVADRSKGKVKEDDPQFQNKGVGYVHFAV  75

Query  165  VKEHQQLIRMSKNKKIEFDLKPAKWDI-ERARSYGTP--SPSIAPKI-ESKPKDSKAR  218
              + QQ +   KN K  F  +  K D  +R  + G     PS A +I E +P + KAR
Sbjct  76   TDDAQQALEKFKNAK--FMGRRLKLDYAQRKSALGEKRQKPSSALQIPEEQPSNKKAR  131


>ref|XP_010223297.1| PREDICTED: DAZ-associated protein 1, partial [Tinamus guttatus]
Length=415

 Score = 42.7 bits (99),  Expect = 0.21, Method: Compositional matrix adjust.
 Identities = 34/118 (29%), Positives = 56/118 (47%), Gaps = 19/118 (16%)

Query  98   TDTNGSSRVDQTLFVAGIPNVAREQHLTEYFEDLGIIVRDVFVPSAFNQNGQKRKHNRGF  157
            +D N S+++    FV GIP+   E  L EYF+  G++   V +  A      +++  RGF
Sbjct  114  SDNNKSNKI----FVGGIPHNCGETELREYFKKFGVVTEVVMIYDA------EKQRPRGF  163

Query  158  GFVEVGTVKEHQQLIRMSKNKKIEFDLKPAKWDIERARSYG----TPSPSIAPKIESK  211
            GF+      E +Q +  + N     D+   K +++RA        TP P  A + ES+
Sbjct  164  GFITF----EDEQSVDQAVNMHFH-DIMGKKVEVKRAEPRDSKSQTPGPPGASQWESR  216


>gb|KGL84381.1| DAZ-associated protein 1, partial [Tinamus guttatus]
Length=398

 Score = 42.7 bits (99),  Expect = 0.23, Method: Compositional matrix adjust.
 Identities = 34/118 (29%), Positives = 56/118 (47%), Gaps = 19/118 (16%)

Query  98   TDTNGSSRVDQTLFVAGIPNVAREQHLTEYFEDLGIIVRDVFVPSAFNQNGQKRKHNRGF  157
            +D N S+++    FV GIP+   E  L EYF+  G++   V +  A      +++  RGF
Sbjct  97   SDNNKSNKI----FVGGIPHNCGETELREYFKKFGVVTEVVMIYDA------EKQRPRGF  146

Query  158  GFVEVGTVKEHQQLIRMSKNKKIEFDLKPAKWDIERARSYG----TPSPSIAPKIESK  211
            GF+      E +Q +  + N     D+   K +++RA        TP P  A + ES+
Sbjct  147  GFITF----EDEQSVDQAVNMHFH-DIMGKKVEVKRAEPRDSKSQTPGPPGASQWESR  199


>ref|XP_008985159.1| PREDICTED: DAZ-associated protein 1 isoform X5 [Callithrix jacchus]
 ref|XP_008985160.1| PREDICTED: DAZ-associated protein 1 isoform X5 [Callithrix jacchus]
Length=378

 Score = 42.4 bits (98),  Expect = 0.31, Method: Compositional matrix adjust.
 Identities = 30/110 (27%), Positives = 51/110 (46%), Gaps = 27/110 (25%)

Query  110  LFVAGIPNVAREQHLTEYFEDLGIIVRDVFVPSAFNQNGQKRKHNRGFGFVEVGTVKEHQ  169
            +FV GIP+   E  L EYF+  G++   V +  A      +++  RGFGF+      E +
Sbjct  115  IFVGGIPHNCGETELREYFKKFGVVTEVVMIYDA------EKQRPRGFGFITF----EDE  164

Query  170  QLIRMSKNKKIEFDLKPAKWDIERARSYGTPSPSIAPKIESKPKDSKARP  219
            Q +  + N     D+   K +++RA                +P+DSK++P
Sbjct  165  QSVDQAVNMHFH-DIMGKKVEVKRA----------------EPRDSKSQP  197


>ref|XP_012291431.1| PREDICTED: DAZ-associated protein 1 isoform X5 [Aotus nancymaae]
Length=369

 Score = 42.4 bits (98),  Expect = 0.31, Method: Compositional matrix adjust.
 Identities = 30/110 (27%), Positives = 51/110 (46%), Gaps = 27/110 (25%)

Query  110  LFVAGIPNVAREQHLTEYFEDLGIIVRDVFVPSAFNQNGQKRKHNRGFGFVEVGTVKEHQ  169
            +FV GIP+   E  L EYF+  G++   V +  A      +++  RGFGF+      E +
Sbjct  106  IFVGGIPHNCGETELREYFKKFGVVTEVVMIYDA------EKQRPRGFGFITF----EDE  155

Query  170  QLIRMSKNKKIEFDLKPAKWDIERARSYGTPSPSIAPKIESKPKDSKARP  219
            Q +  + N     D+   K +++RA                +P+DSK++P
Sbjct  156  QSVDQAVNMHFH-DIMGKKVEVKRA----------------EPRDSKSQP  188


>ref|XP_010330750.1| PREDICTED: DAZ-associated protein 1 isoform X4 [Saimiri boliviensis 
boliviensis]
Length=377

 Score = 42.4 bits (98),  Expect = 0.33, Method: Compositional matrix adjust.
 Identities = 30/110 (27%), Positives = 51/110 (46%), Gaps = 27/110 (25%)

Query  110  LFVAGIPNVAREQHLTEYFEDLGIIVRDVFVPSAFNQNGQKRKHNRGFGFVEVGTVKEHQ  169
            +FV GIP+   E  L EYF+  G++   V +  A      +++  RGFGF+      E +
Sbjct  114  IFVGGIPHNCGETELREYFKKFGVVTEVVMIYDA------EKQRPRGFGFITF----EDE  163

Query  170  QLIRMSKNKKIEFDLKPAKWDIERARSYGTPSPSIAPKIESKPKDSKARP  219
            Q +  + N     D+   K +++RA                +P+DSK++P
Sbjct  164  QSVDQAVNMHFH-DIMGKKVEVKRA----------------EPRDSKSQP  196


>gb|AEK69207.1| boule-like protein 3 [Macrostomum lignano]
Length=162

 Score = 40.8 bits (94),  Expect = 0.35, Method: Compositional matrix adjust.
 Identities = 30/96 (31%), Positives = 49/96 (51%), Gaps = 10/96 (10%)

Query  78   SASAFSSSTSTPLKSTALLDTDTNGSSRVDQTLFVAGIPNVAREQHLTEYFEDLGIIVRD  137
            S +A  +   TP+ + A  +T+  G+  +   +FV GIP+ A E  L E+F  LG  VRD
Sbjct  38   STTAIVAMPGTPVSTAA--NTELGGT-LIPNRVFVGGIPSSATEAELMEFFSALG-EVRD  93

Query  138  VFVPSAFNQNGQKRKHNRGFGFVEVGTVKEHQQLIR  173
            V + +       K   ++G+GFV   + +    +IR
Sbjct  94   VKIIA------DKSGTSKGYGFVTFESAELADSIIR  123


>gb|KKS04378.1| RNP-1 like protein RNA-binding protein [Parcubacteria (Nomurabacteria) 
bacterium GW2011_GWA2_41_25]
Length=106

 Score = 40.0 bits (92),  Expect = 0.36, Method: Compositional matrix adjust.
 Identities = 22/72 (31%), Positives = 36/72 (50%), Gaps = 6/72 (8%)

Query  110  LFVAGIPNVAREQHLTEYFEDLGIIVRDVFVPSAFNQNGQKRKHNRGFGFVEVGTVKEHQ  169
            L+V G+P   +E  L E F   G +V  V +    +        ++GFGFVE+ +  E Q
Sbjct  5    LYVGGLPYSTQEDALKELFAQAGNVVSAVIIMDKMS------GRSKGFGFVEMSSQDEAQ  58

Query  170  QLIRMSKNKKIE  181
            + I M  +++ E
Sbjct  59   KAISMFNDQEFE  70


>ref|XP_008985154.1| PREDICTED: DAZ-associated protein 1 isoform X1 [Callithrix jacchus]
Length=407

 Score = 42.0 bits (97),  Expect = 0.37, Method: Compositional matrix adjust.
 Identities = 30/110 (27%), Positives = 51/110 (46%), Gaps = 27/110 (25%)

Query  110  LFVAGIPNVAREQHLTEYFEDLGIIVRDVFVPSAFNQNGQKRKHNRGFGFVEVGTVKEHQ  169
            +FV GIP+   E  L EYF+  G++   V +  A      +++  RGFGF+      E +
Sbjct  115  IFVGGIPHNCGETELREYFKKFGVVTEVVMIYDA------EKQRPRGFGFITF----EDE  164

Query  170  QLIRMSKNKKIEFDLKPAKWDIERARSYGTPSPSIAPKIESKPKDSKARP  219
            Q +  + N     D+   K +++RA                +P+DSK++P
Sbjct  165  QSVDQAVNMHFH-DIMGKKVEVKRA----------------EPRDSKSQP  197


>ref|XP_012291429.1| PREDICTED: DAZ-associated protein 1 isoform X3 [Aotus nancymaae]
Length=397

 Score = 42.0 bits (97),  Expect = 0.38, Method: Compositional matrix adjust.
 Identities = 30/110 (27%), Positives = 51/110 (46%), Gaps = 27/110 (25%)

Query  110  LFVAGIPNVAREQHLTEYFEDLGIIVRDVFVPSAFNQNGQKRKHNRGFGFVEVGTVKEHQ  169
            +FV GIP+   E  L EYF+  G++   V +  A      +++  RGFGF+      E +
Sbjct  105  IFVGGIPHNCGETELREYFKKFGVVTEVVMIYDA------EKQRPRGFGFITF----EDE  154

Query  170  QLIRMSKNKKIEFDLKPAKWDIERARSYGTPSPSIAPKIESKPKDSKARP  219
            Q +  + N     D+   K +++RA                +P+DSK++P
Sbjct  155  QSVDQAVNMHFH-DIMGKKVEVKRA----------------EPRDSKSQP  187


>ref|XP_012291427.1| PREDICTED: DAZ-associated protein 1 isoform X1 [Aotus nancymaae]
Length=398

 Score = 42.0 bits (97),  Expect = 0.39, Method: Compositional matrix adjust.
 Identities = 30/110 (27%), Positives = 51/110 (46%), Gaps = 27/110 (25%)

Query  110  LFVAGIPNVAREQHLTEYFEDLGIIVRDVFVPSAFNQNGQKRKHNRGFGFVEVGTVKEHQ  169
            +FV GIP+   E  L EYF+  G++   V +  A      +++  RGFGF+      E +
Sbjct  106  IFVGGIPHNCGETELREYFKKFGVVTEVVMIYDA------EKQRPRGFGFITF----EDE  155

Query  170  QLIRMSKNKKIEFDLKPAKWDIERARSYGTPSPSIAPKIESKPKDSKARP  219
            Q +  + N     D+   K +++RA                +P+DSK++P
Sbjct  156  QSVDQAVNMHFH-DIMGKKVEVKRA----------------EPRDSKSQP  188


>gb|KKS50256.1| Glycine-rich RNA-binding protein GRP1A [Parcubacteria bacterium 
GW2011_GWC1_42_21]
 gb|KKS57970.1| Glycine-rich RNA-binding protein GRP1A [Parcubacteria (Nomurabacteria) 
bacterium GW2011_GWF1_42_40]
 gb|KKT00667.1| Glycine-rich RNA-binding protein GRP1A [Parcubacteria (Nomurabacteria) 
bacterium GW2011_GWA1_43_17]
 gb|KKT07646.1| Glycine-rich RNA-binding protein GRP1A [Parcubacteria (Nomurabacteria) 
bacterium GW2011_GWB1_43_19]
 gb|KKT11826.1| Glycine-rich RNA-binding protein GRP1A [Parcubacteria (Nomurabacteria) 
bacterium GW2011_GWF2_43_24]
 gb|KKT18421.1| Glycine-rich RNA-binding protein GRP1A [Parcubacteria (Nomurabacteria) 
bacterium GW2011_GWA2_43_66]
Length=104

 Score = 39.7 bits (91),  Expect = 0.39, Method: Compositional matrix adjust.
 Identities = 22/72 (31%), Positives = 36/72 (50%), Gaps = 6/72 (8%)

Query  110  LFVAGIPNVAREQHLTEYFEDLGIIVRDVFVPSAFNQNGQKRKHNRGFGFVEVGTVKEHQ  169
            L+V G+P   +E  L E F   G +V  V +    +        ++GFGFVE+ +  E Q
Sbjct  5    LYVGGLPYSTQEDALKELFAQAGNVVSAVIIMDKMS------GRSKGFGFVEMSSQDEAQ  58

Query  170  QLIRMSKNKKIE  181
            + I M  +++ E
Sbjct  59   KAISMFNDQEFE  70


>ref|XP_008985158.1| PREDICTED: DAZ-associated protein 1 isoform X4 [Callithrix jacchus]
Length=405

 Score = 42.0 bits (97),  Expect = 0.39, Method: Compositional matrix adjust.
 Identities = 30/110 (27%), Positives = 51/110 (46%), Gaps = 27/110 (25%)

Query  110  LFVAGIPNVAREQHLTEYFEDLGIIVRDVFVPSAFNQNGQKRKHNRGFGFVEVGTVKEHQ  169
            +FV GIP+   E  L EYF+  G++   V +  A      +++  RGFGF+      E +
Sbjct  114  IFVGGIPHNCGETELREYFKKFGVVTEVVMIYDA------EKQRPRGFGFITF----EDE  163

Query  170  QLIRMSKNKKIEFDLKPAKWDIERARSYGTPSPSIAPKIESKPKDSKARP  219
            Q +  + N     D+   K +++RA                +P+DSK++P
Sbjct  164  QSVDQAVNMHFH-DIMGKKVEVKRA----------------EPRDSKSQP  196


>ref|XP_008985157.1| PREDICTED: DAZ-associated protein 1 isoform X3 [Callithrix jacchus]
Length=406

 Score = 42.0 bits (97),  Expect = 0.39, Method: Compositional matrix adjust.
 Identities = 30/110 (27%), Positives = 51/110 (46%), Gaps = 27/110 (25%)

Query  110  LFVAGIPNVAREQHLTEYFEDLGIIVRDVFVPSAFNQNGQKRKHNRGFGFVEVGTVKEHQ  169
            +FV GIP+   E  L EYF+  G++   V +  A      +++  RGFGF+      E +
Sbjct  114  IFVGGIPHNCGETELREYFKKFGVVTEVVMIYDA------EKQRPRGFGFITF----EDE  163

Query  170  QLIRMSKNKKIEFDLKPAKWDIERARSYGTPSPSIAPKIESKPKDSKARP  219
            Q +  + N     D+   K +++RA                +P+DSK++P
Sbjct  164  QSVDQAVNMHFH-DIMGKKVEVKRA----------------EPRDSKSQP  196


>ref|XP_012291430.1| PREDICTED: DAZ-associated protein 1 isoform X4 [Aotus nancymaae]
Length=396

 Score = 42.0 bits (97),  Expect = 0.40, Method: Compositional matrix adjust.
 Identities = 30/110 (27%), Positives = 51/110 (46%), Gaps = 27/110 (25%)

Query  110  LFVAGIPNVAREQHLTEYFEDLGIIVRDVFVPSAFNQNGQKRKHNRGFGFVEVGTVKEHQ  169
            +FV GIP+   E  L EYF+  G++   V +  A      +++  RGFGF+      E +
Sbjct  105  IFVGGIPHNCGETELREYFKKFGVVTEVVMIYDA------EKQRPRGFGFITF----EDE  154

Query  170  QLIRMSKNKKIEFDLKPAKWDIERARSYGTPSPSIAPKIESKPKDSKARP  219
            Q +  + N     D+   K +++RA                +P+DSK++P
Sbjct  155  QSVDQAVNMHFH-DIMGKKVEVKRA----------------EPRDSKSQP  187


>ref|XP_012291428.1| PREDICTED: DAZ-associated protein 1 isoform X2 [Aotus nancymaae]
Length=397

 Score = 42.0 bits (97),  Expect = 0.40, Method: Compositional matrix adjust.
 Identities = 30/110 (27%), Positives = 51/110 (46%), Gaps = 27/110 (25%)

Query  110  LFVAGIPNVAREQHLTEYFEDLGIIVRDVFVPSAFNQNGQKRKHNRGFGFVEVGTVKEHQ  169
            +FV GIP+   E  L EYF+  G++   V +  A      +++  RGFGF+      E +
Sbjct  106  IFVGGIPHNCGETELREYFKKFGVVTEVVMIYDA------EKQRPRGFGFITF----EDE  155

Query  170  QLIRMSKNKKIEFDLKPAKWDIERARSYGTPSPSIAPKIESKPKDSKARP  219
            Q +  + N     D+   K +++RA                +P+DSK++P
Sbjct  156  QSVDQAVNMHFH-DIMGKKVEVKRA----------------EPRDSKSQP  188


>ref|XP_010330747.1| PREDICTED: DAZ-associated protein 1 isoform X1 [Saimiri boliviensis 
boliviensis]
Length=406

 Score = 42.0 bits (97),  Expect = 0.40, Method: Compositional matrix adjust.
 Identities = 30/110 (27%), Positives = 51/110 (46%), Gaps = 27/110 (25%)

Query  110  LFVAGIPNVAREQHLTEYFEDLGIIVRDVFVPSAFNQNGQKRKHNRGFGFVEVGTVKEHQ  169
            +FV GIP+   E  L EYF+  G++   V +  A      +++  RGFGF+      E +
Sbjct  114  IFVGGIPHNCGETELREYFKKFGVVTEVVMIYDA------EKQRPRGFGFITF----EDE  163

Query  170  QLIRMSKNKKIEFDLKPAKWDIERARSYGTPSPSIAPKIESKPKDSKARP  219
            Q +  + N     D+   K +++RA                +P+DSK++P
Sbjct  164  QSVDQAVNMHFH-DIMGKKVEVKRA----------------EPRDSKSQP  196


>ref|XP_008985156.1| PREDICTED: DAZ-associated protein 1 isoform X2 [Callithrix jacchus]
Length=406

 Score = 42.0 bits (97),  Expect = 0.40, Method: Compositional matrix adjust.
 Identities = 30/110 (27%), Positives = 51/110 (46%), Gaps = 27/110 (25%)

Query  110  LFVAGIPNVAREQHLTEYFEDLGIIVRDVFVPSAFNQNGQKRKHNRGFGFVEVGTVKEHQ  169
            +FV GIP+   E  L EYF+  G++   V +  A      +++  RGFGF+      E +
Sbjct  115  IFVGGIPHNCGETELREYFKKFGVVTEVVMIYDA------EKQRPRGFGFITF----EDE  164

Query  170  QLIRMSKNKKIEFDLKPAKWDIERARSYGTPSPSIAPKIESKPKDSKARP  219
            Q +  + N     D+   K +++RA                +P+DSK++P
Sbjct  165  QSVDQAVNMHFH-DIMGKKVEVKRA----------------EPRDSKSQP  197


>ref|XP_008928902.1| PREDICTED: DAZ-associated protein 1 [Manacus vitellinus]
Length=437

 Score = 42.0 bits (97),  Expect = 0.41, Method: Compositional matrix adjust.
 Identities = 36/137 (26%), Positives = 62/137 (45%), Gaps = 34/137 (25%)

Query  98   TDTNGSSRVDQTLFVAGIPNVAREQHLTEYFEDLGIIVRDVFVPSAFNQNGQKRKHNRGF  157
            +D N S+++    FV GIP+   E  L EYF+  G++   V +  A      +++  RGF
Sbjct  131  SDNNKSNKI----FVGGIPHNCGETELREYFKKFGVVTEVVMIYDA------EKQRPRGF  180

Query  158  GFVEVGTVKEHQQLIRMSKNKKIEFDLKPAKWDIERARSYGTPSPSIAPKIESKPKDSKA  217
            GF+      E +Q +  + N     D+   K +++RA                +P+DSK+
Sbjct  181  GFITF----EDEQSVDQAVNMHFH-DIMGKKVEVKRA----------------EPRDSKS  219

Query  218  R---PDDKARSRSAIEP  231
            +   P   ++ R  I P
Sbjct  220  QTPGPPGASQWRGRIMP  236


>ref|XP_010330749.1| PREDICTED: DAZ-associated protein 1 isoform X3 [Saimiri boliviensis 
boliviensis]
Length=405

 Score = 42.0 bits (97),  Expect = 0.41, Method: Compositional matrix adjust.
 Identities = 30/110 (27%), Positives = 51/110 (46%), Gaps = 27/110 (25%)

Query  110  LFVAGIPNVAREQHLTEYFEDLGIIVRDVFVPSAFNQNGQKRKHNRGFGFVEVGTVKEHQ  169
            +FV GIP+   E  L EYF+  G++   V +  A      +++  RGFGF+      E +
Sbjct  113  IFVGGIPHNCGETELREYFKKFGVVTEVVMIYDA------EKQRPRGFGFITF----EDE  162

Query  170  QLIRMSKNKKIEFDLKPAKWDIERARSYGTPSPSIAPKIESKPKDSKARP  219
            Q +  + N     D+   K +++RA                +P+DSK++P
Sbjct  163  QSVDQAVNMHFH-DIMGKKVEVKRA----------------EPRDSKSQP  195


>ref|XP_010330748.1| PREDICTED: DAZ-associated protein 1 isoform X2 [Saimiri boliviensis 
boliviensis]
Length=405

 Score = 42.0 bits (97),  Expect = 0.41, Method: Compositional matrix adjust.
 Identities = 30/110 (27%), Positives = 51/110 (46%), Gaps = 27/110 (25%)

Query  110  LFVAGIPNVAREQHLTEYFEDLGIIVRDVFVPSAFNQNGQKRKHNRGFGFVEVGTVKEHQ  169
            +FV GIP+   E  L EYF+  G++   V +  A      +++  RGFGF+      E +
Sbjct  114  IFVGGIPHNCGETELREYFKKFGVVTEVVMIYDA------EKQRPRGFGFITF----EDE  163

Query  170  QLIRMSKNKKIEFDLKPAKWDIERARSYGTPSPSIAPKIESKPKDSKARP  219
            Q +  + N     D+   K +++RA                +P+DSK++P
Sbjct  164  QSVDQAVNMHFH-DIMGKKVEVKRA----------------EPRDSKSQP  196


>emb|CCK67910.1| hypothetical protein KNAG_0A02210 [Kazachstania naganishii CBS 
8797]
Length=682

 Score = 42.4 bits (98),  Expect = 0.41, Method: Compositional matrix adjust.
 Identities = 37/129 (29%), Positives = 61/129 (47%), Gaps = 24/129 (19%)

Query  108  QTLFVAGIPNVAREQHLTEYFEDL-----GIIVRDVFVPSAFNQNGQKRKHNRGFGFVEV  162
            QTLFV  IP    ++ LTE+F +       ++V+DV             K +RGFGFV  
Sbjct  17   QTLFVRAIPFAVTDEQLTEFFANFAPTKHAVVVKDV------------NKKSRGFGFVSF  64

Query  163  GTVKEHQQLIRMSKNKKIEFDLKPAKWDI----ERARSYGTPSPSI-APKIESKPKDSKA  217
             + ++ ++ +  ++ +K+   L   + DI    ER +     S  I APK E K K +  
Sbjct  65   ASEEDTKEALLKARKEKLNGQL--LRVDIAKRRERNKRKEDASNGIAAPKPEHKEKRTYG  122

Query  218  RPDDKARSR  226
              DD+ + +
Sbjct  123  DEDDQFKGK  131


>ref|XP_013800785.1| PREDICTED: DAZ-associated protein 1 isoform X3 [Apteryx australis 
mantelli]
Length=393

 Score = 42.0 bits (97),  Expect = 0.44, Method: Compositional matrix adjust.
 Identities = 26/88 (30%), Positives = 42/88 (48%), Gaps = 14/88 (16%)

Query  98   TDTNGSSRVDQTLFVAGIPNVAREQHLTEYFEDLGIIVRDVFVPSAFNQNGQKRKHNRGF  157
            +D N S+++    FV GIP+   E  L EYF+  G++   V +  A      +++  RGF
Sbjct  108  SDNNKSNKI----FVGGIPHNCGETELREYFKKFGVVTEVVMIYDA------EKQRPRGF  157

Query  158  GFVEVGTVKEHQQLIRMS----KNKKIE  181
            GF+     +   Q + M       KK+E
Sbjct  158  GFITFEDEQSVDQAVNMHFHDIMGKKVE  185


>ref|XP_010442944.1| PREDICTED: LOW QUALITY PROTEIN: RNA-binding protein 24 [Camelina 
sativa]
Length=100

 Score = 39.7 bits (91),  Expect = 0.44, Method: Compositional matrix adjust.
 Identities = 27/82 (33%), Positives = 37/82 (45%), Gaps = 9/82 (11%)

Query  110  LFVAGIPNVAREQHLTEYFEDLGIIVRDVFVPSAFNQNGQKRKHNRGFGFVEVGTVKEHQ  169
            ++VAG+P+V R + L  YFE  G IV    V     Q       ++GFGFV   T KE  
Sbjct  11   IYVAGLPSVTRTEGLRSYFEQFGEIVYANVVCDGATQ------RSKGFGFV---TFKEAD  61

Query  170  QLIRMSKNKKIEFDLKPAKWDI  191
               R  +N     D + A   +
Sbjct  62   SATRACENPNHMIDXRMANCKL  83


>gb|KDQ60783.1| hypothetical protein JAAARDRAFT_190929 [Jaapia argillacea MUCL 
33604]
Length=154

 Score = 40.4 bits (93),  Expect = 0.46, Method: Compositional matrix adjust.
 Identities = 24/70 (34%), Positives = 33/70 (47%), Gaps = 0/70 (0%)

Query  103  SSRVDQTLFVAGIPNVAREQHLTEYFEDLGIIVRDVFVPSAFNQNGQKRKHNRGFGFVEV  162
             +R  +T+FV GI +   E  L E F   G I+     P A NQ+ Q    +RGF FV  
Sbjct  4    GTRSKKTVFVGGIGDDVDEGALVEAFTPFGDIIEVQLPPVATNQHQQSEAKHRGFAFVTY  63

Query  163  GTVKEHQQLI  172
               ++ Q  I
Sbjct  64   SAPEDAQDAI  73


>pdb|2DGS|A Chain A, Solution Structure Of The Second Rna Binding Domain 
In Daz- Associated Protein 1
Length=99

 Score = 39.7 bits (91),  Expect = 0.47, Method: Compositional matrix adjust.
 Identities = 25/85 (29%), Positives = 42/85 (49%), Gaps = 11/85 (13%)

Query  110  LFVAGIPNVAREQHLTEYFEDLGIIVRDVFVPSAFNQNGQKRKHNRGFGFVEVGTVKEHQ  169
            +FV GIP+   E  L EYF+  G++   V +  A      +++  RGFGF+      E +
Sbjct  13   IFVGGIPHNCGETELREYFKKFGVVTEVVMIYDA------EKQRPRGFGFITF----EDE  62

Query  170  QLIRMSKNKKIEFDLKPAKWDIERA  194
            Q +  + N     D+   K +++RA
Sbjct  63   QSVDQAVNMHFH-DIMGKKVEVKRA  86


>gb|KKS54071.1| RNA-binding protein [Parcubacteria bacterium GW2011_GWA2_42_28]
 gb|KKT56260.1| RNA-binding protein [Parcubacteria bacterium GW2011_GWC2_44_22]
Length=109

 Score = 39.7 bits (91),  Expect = 0.48, Method: Compositional matrix adjust.
 Identities = 25/89 (28%), Positives = 41/89 (46%), Gaps = 10/89 (11%)

Query  110  LFVAGIPNVAREQHLTEYFEDLGIIVRDVFVPSAFNQNGQKRKHNRGFGFVEVGTVKEHQ  169
            L+V  +P       L+E F   G++V    +   F+        ++GFGFVE+ T +E +
Sbjct  5    LYVGSLPYAVTGDQLSELFSQAGMVVSANIILDKFS------GRSKGFGFVEMSTEEESK  58

Query  170  QLIRMSKNKKIEFDLKPAKWDIERARSYG  198
            + I M  N    +DL+     +  AR  G
Sbjct  59   KAIEMFNN----YDLEGRNLVVNEARPMG  83


>gb|KKQ04002.1| RNP-1 like protein RNA-binding protein [Parcubacteria bacterium 
GW2011_GWA2_36_24]
 gb|KKQ06458.1| RNP-1 like protein RNA-binding protein [Parcubacteria bacterium 
GW2011_GWB1_36_5]
Length=106

 Score = 39.7 bits (91),  Expect = 0.48, Method: Compositional matrix adjust.
 Identities = 23/72 (32%), Positives = 35/72 (49%), Gaps = 6/72 (8%)

Query  110  LFVAGIPNVAREQHLTEYFEDLGIIVRDVFVPSAFNQNGQKRKHNRGFGFVEVGTVKEHQ  169
            L+V G+P    E  L E F   G +V  V +        +    ++GFGFVE+ T  E Q
Sbjct  5    LYVGGLPYSTVENALKELFAQAGNVVSAVIIMD------KMSGRSKGFGFVEMSTNDEAQ  58

Query  170  QLIRMSKNKKIE  181
            + I M  +++ E
Sbjct  59   KAISMFNDQEFE  70


>ref|XP_012958411.1| PREDICTED: DAZ-associated protein 1 [Anas platyrhynchos]
Length=436

 Score = 42.0 bits (97),  Expect = 0.49, Method: Compositional matrix adjust.
 Identities = 28/97 (29%), Positives = 48/97 (49%), Gaps = 15/97 (15%)

Query  98   TDTNGSSRVDQTLFVAGIPNVAREQHLTEYFEDLGIIVRDVFVPSAFNQNGQKRKHNRGF  157
            +D N S+++    FV GIP+   E  L EYF+  G++   V +  A      +++  RGF
Sbjct  135  SDNNKSNKI----FVGGIPHNCGETELREYFKKFGVVTEVVMIYDA------EKQRPRGF  184

Query  158  GFVEVGTVKEHQQLIRMSKNKKIEFDLKPAKWDIERA  194
            GF+      E +Q +  + N     D+   K +++RA
Sbjct  185  GFITF----EDEQSVDQAVNMHFH-DIMGKKVEVKRA  216


>gb|ESA01207.1| hypothetical protein GLOINDRAFT_262119 [Rhizophagus irregularis 
DAOM 181602]
 gb|EXX69322.1| Ist3p [Rhizophagus irregularis DAOM 197198w]
Length=260

 Score = 41.6 bits (96),  Expect = 0.49, Method: Compositional matrix adjust.
 Identities = 33/126 (26%), Positives = 53/126 (42%), Gaps = 13/126 (10%)

Query  81   AFSSSTSTPLKSTALLDTDTNGSSRVDQTLFVAGIPNVAREQHLTEYFEDLGIIVRDVFV  140
            +++S    P +    +D  ++   R   TL+V  +P   REQ + E FE  G + R V V
Sbjct  101  SYASERRGPPRRNKPIDRGSDKERRDSTTLYVGNLPYAFREQDVAEMFERYGRL-RKVTV  159

Query  141  PSAFNQNGQKRKHNRGFGFVEVGTVKEHQQLIRM-------SKNKKIEFDLKPAKWDIER  193
                 Q       N+GF FVE    ++ +             +  K+++D+   K D  R
Sbjct  160  -----QIDHYTGRNKGFAFVEFEDRRDAEDAFDKYNGTNVEGRRLKLDWDIGLGKKDQHR  214

Query  194  ARSYGT  199
               YGT
Sbjct  215  REKYGT  220


>ref|XP_007764737.1| RNA-binding domain-containing protein [Coniophora puteana RWD-64-598 
SS2]
 gb|EIW85162.1| RNA-binding domain-containing protein [Coniophora puteana RWD-64-598 
SS2]
Length=151

 Score = 40.4 bits (93),  Expect = 0.49, Method: Compositional matrix adjust.
 Identities = 26/66 (39%), Positives = 36/66 (55%), Gaps = 2/66 (3%)

Query  108  QTLFVAGIPNVAREQHLTEYFEDLGIIVRDVFVPSAF-NQNGQKRKHNRGFGFVEVGTVK  166
            +T+F+ GI +   E  L E F   G I+ +V +PSA  N N Q    +RGFGFV  G+  
Sbjct  9    KTIFIGGIGDDIDENALYETFSPFGDII-EVQLPSAATNPNQQTEAKHRGFGFVTFGSPA  67

Query  167  EHQQLI  172
            + Q  I
Sbjct  68   DAQDAI  73


>ref|XP_013800775.1| PREDICTED: DAZ-associated protein 1 isoform X2 [Apteryx australis 
mantelli]
Length=399

 Score = 41.6 bits (96),  Expect = 0.49, Method: Compositional matrix adjust.
 Identities = 28/97 (29%), Positives = 48/97 (49%), Gaps = 15/97 (15%)

Query  98   TDTNGSSRVDQTLFVAGIPNVAREQHLTEYFEDLGIIVRDVFVPSAFNQNGQKRKHNRGF  157
            +D N S+++    FV GIP+   E  L EYF+  G++   V +  A      +++  RGF
Sbjct  108  SDNNKSNKI----FVGGIPHNCGETELREYFKKFGVVTEVVMIYDA------EKQRPRGF  157

Query  158  GFVEVGTVKEHQQLIRMSKNKKIEFDLKPAKWDIERA  194
            GF+      E +Q +  + N     D+   K +++RA
Sbjct  158  GFITF----EDEQSVDQAVNMHFH-DIMGKKVEVKRA  189


>ref|XP_005433987.1| PREDICTED: LOW QUALITY PROTEIN: DAZ-associated protein 1 [Falco 
cherrug]
Length=390

 Score = 41.6 bits (96),  Expect = 0.50, Method: Compositional matrix adjust.
 Identities = 26/88 (30%), Positives = 42/88 (48%), Gaps = 14/88 (16%)

Query  98   TDTNGSSRVDQTLFVAGIPNVAREQHLTEYFEDLGIIVRDVFVPSAFNQNGQKRKHNRGF  157
            +D N S+++    FV GIP+   E  L EYF+  G++   V +  A      +++  RGF
Sbjct  103  SDNNKSNKI----FVGGIPHNCGETELREYFKKFGVVTEVVMIYDA------EKQRPRGF  152

Query  158  GFVEVGTVKEHQQLIRMS----KNKKIE  181
            GF+     +   Q + M       KK+E
Sbjct  153  GFITFEDEQSVDQAVNMHFHDIMGKKVE  180


>ref|XP_013055534.1| PREDICTED: DAZ-associated protein 1 isoform X4 [Anser cygnoides 
domesticus]
Length=378

 Score = 41.6 bits (96),  Expect = 0.52, Method: Compositional matrix adjust.
 Identities = 28/97 (29%), Positives = 48/97 (49%), Gaps = 15/97 (15%)

Query  98   TDTNGSSRVDQTLFVAGIPNVAREQHLTEYFEDLGIIVRDVFVPSAFNQNGQKRKHNRGF  157
            +D N S+++    FV GIP+   E  L EYF+  G++   V +  A      +++  RGF
Sbjct  108  SDNNKSNKI----FVGGIPHNCGETELREYFKKFGVVTEVVMIYDA------EKQRPRGF  157

Query  158  GFVEVGTVKEHQQLIRMSKNKKIEFDLKPAKWDIERA  194
            GF+      E +Q +  + N     D+   K +++RA
Sbjct  158  GFITF----EDEQSVDQAVNMHFH-DIMGKKVEVKRA  189


>ref|XP_008985161.1| PREDICTED: DAZ-associated protein 1 isoform X6 [Callithrix jacchus]
 ref|XP_008985162.1| PREDICTED: DAZ-associated protein 1 isoform X6 [Callithrix jacchus]
Length=366

 Score = 41.6 bits (96),  Expect = 0.52, Method: Compositional matrix adjust.
 Identities = 30/110 (27%), Positives = 51/110 (46%), Gaps = 27/110 (25%)

Query  110  LFVAGIPNVAREQHLTEYFEDLGIIVRDVFVPSAFNQNGQKRKHNRGFGFVEVGTVKEHQ  169
            +FV GIP+   E  L EYF+  G++   V +  A      +++  RGFGF+      E +
Sbjct  74   IFVGGIPHNCGETELREYFKKFGVVTEVVMIYDA------EKQRPRGFGFITF----EDE  123

Query  170  QLIRMSKNKKIEFDLKPAKWDIERARSYGTPSPSIAPKIESKPKDSKARP  219
            Q +  + N     D+   K +++RA                +P+DSK++P
Sbjct  124  QSVDQAVNMHFH-DIMGKKVEVKRA----------------EPRDSKSQP  156


>ref|XP_008945735.1| PREDICTED: DAZ-associated protein 1, partial [Merops nubicus]
Length=346

 Score = 41.6 bits (96),  Expect = 0.53, Method: Compositional matrix adjust.
 Identities = 26/88 (30%), Positives = 42/88 (48%), Gaps = 14/88 (16%)

Query  98   TDTNGSSRVDQTLFVAGIPNVAREQHLTEYFEDLGIIVRDVFVPSAFNQNGQKRKHNRGF  157
            +D N S+++    FV GIP+   E  L EYF+  G++   V +  A      +++  RGF
Sbjct  97   SDNNKSNKI----FVGGIPHNCGETELREYFKKFGVVTEVVMIYDA------EKQRPRGF  146

Query  158  GFVEVGTVKEHQQLIRMS----KNKKIE  181
            GF+     +   Q + M       KK+E
Sbjct  147  GFITFEDEQSVDQAVNMHFHDIMGKKVE  174


>ref|XP_009865967.1| PREDICTED: DAZ-associated protein 1, partial [Apaloderma vittatum]
Length=369

 Score = 41.6 bits (96),  Expect = 0.53, Method: Compositional matrix adjust.
 Identities = 28/97 (29%), Positives = 48/97 (49%), Gaps = 15/97 (15%)

Query  98   TDTNGSSRVDQTLFVAGIPNVAREQHLTEYFEDLGIIVRDVFVPSAFNQNGQKRKHNRGF  157
            +D N S+++    FV GIP+   E  L EYF+  G++   V +  A      +++  RGF
Sbjct  97   SDNNKSNKI----FVGGIPHNCGETELREYFKKFGVVTEVVMIYDA------EKQRPRGF  146

Query  158  GFVEVGTVKEHQQLIRMSKNKKIEFDLKPAKWDIERA  194
            GF+      E +Q +  + N     D+   K +++RA
Sbjct  147  GFITF----EDEQSVDQAVNMHFH-DIMGKKVEVKRA  178


>ref|XP_009936272.1| PREDICTED: LOW QUALITY PROTEIN: DAZ-associated protein 1 [Opisthocomus 
hoazin]
Length=395

 Score = 41.6 bits (96),  Expect = 0.53, Method: Compositional matrix adjust.
 Identities = 26/88 (30%), Positives = 42/88 (48%), Gaps = 14/88 (16%)

Query  98   TDTNGSSRVDQTLFVAGIPNVAREQHLTEYFEDLGIIVRDVFVPSAFNQNGQKRKHNRGF  157
            +D N S+++    FV GIP+   E  L EYF+  G++   V +  A      +++  RGF
Sbjct  108  SDNNKSNKI----FVGGIPHNCGETELREYFKKFGVVTEVVMIYDA------EKQRPRGF  157

Query  158  GFVEVGTVKEHQQLIRMS----KNKKIE  181
            GF+     +   Q + M       KK+E
Sbjct  158  GFITFEDEQSVDQAVNMHFHDIMGKKVE  185


>gb|EMT22954.1| 28 kDa ribonucleoprotein, chloroplastic [Aegilops tauschii]
Length=580

 Score = 42.0 bits (97),  Expect = 0.53, Method: Compositional matrix adjust.
 Identities = 25/92 (27%), Positives = 44/92 (48%), Gaps = 6/92 (7%)

Query  109  TLFVAGIPNVAREQHLTEYFEDLGIIVRDVFVPSAFNQNGQKRKHNRGFGFVEVGTVKEH  168
            T++V  +P    +  L   F+  G++V   F    ++    K   +RGFG+V + TV+E 
Sbjct  407  TVYVGNLPYHTDDDSLALNFQHAGVVV---FSEVIYDD---KTGQSRGFGYVTMSTVQEA  460

Query  169  QQLIRMSKNKKIEFDLKPAKWDIERARSYGTP  200
            ++ +RM     I   ++P    I   R  G+P
Sbjct  461  EKAVRMYHGYAIYGSVRPLTVYITAPRQSGSP  492


>ref|XP_010723322.1| PREDICTED: DAZ-associated protein 1 [Meleagris gallopavo]
Length=460

 Score = 41.6 bits (96),  Expect = 0.55, Method: Compositional matrix adjust.
 Identities = 28/97 (29%), Positives = 48/97 (49%), Gaps = 15/97 (15%)

Query  98   TDTNGSSRVDQTLFVAGIPNVAREQHLTEYFEDLGIIVRDVFVPSAFNQNGQKRKHNRGF  157
            +D N S+++    FV GIP+   E  L EYF+  G++   V +  A      +++  RGF
Sbjct  159  SDNNKSNKI----FVGGIPHNCGETELREYFKKFGVVTEVVMIYDA------EKQRPRGF  208

Query  158  GFVEVGTVKEHQQLIRMSKNKKIEFDLKPAKWDIERA  194
            GF+      E +Q +  + N     D+   K +++RA
Sbjct  209  GFITF----EDEQSVDQAVNMHFH-DIMGKKVEVKRA  240


>gb|KFW86571.1| DAZ-associated protein 1, partial [Manacus vitellinus]
Length=380

 Score = 41.6 bits (96),  Expect = 0.56, Method: Compositional matrix adjust.
 Identities = 36/137 (26%), Positives = 62/137 (45%), Gaps = 34/137 (25%)

Query  98   TDTNGSSRVDQTLFVAGIPNVAREQHLTEYFEDLGIIVRDVFVPSAFNQNGQKRKHNRGF  157
            +D N S+++    FV GIP+   E  L EYF+  G++   V +  A      +++  RGF
Sbjct  97   SDNNKSNKI----FVGGIPHNCGETELREYFKKFGVVTEVVMIYDA------EKQRPRGF  146

Query  158  GFVEVGTVKEHQQLIRMSKNKKIEFDLKPAKWDIERARSYGTPSPSIAPKIESKPKDSKA  217
            GF+      E +Q +  + N     D+   K +++RA                +P+DSK+
Sbjct  147  GFITF----EDEQSVDQAVNMHFH-DIMGKKVEVKRA----------------EPRDSKS  185

Query  218  R---PDDKARSRSAIEP  231
            +   P   ++ R  I P
Sbjct  186  QTPGPPGASQWRGRIMP  202


>ref|XP_013055533.1| PREDICTED: DAZ-associated protein 1 isoform X3 [Anser cygnoides 
domesticus]
Length=389

 Score = 41.6 bits (96),  Expect = 0.56, Method: Compositional matrix adjust.
 Identities = 26/88 (30%), Positives = 42/88 (48%), Gaps = 14/88 (16%)

Query  98   TDTNGSSRVDQTLFVAGIPNVAREQHLTEYFEDLGIIVRDVFVPSAFNQNGQKRKHNRGF  157
            +D N S+++    FV GIP+   E  L EYF+  G++   V +  A      +++  RGF
Sbjct  108  SDNNKSNKI----FVGGIPHNCGETELREYFKKFGVVTEVVMIYDA------EKQRPRGF  157

Query  158  GFVEVGTVKEHQQLIRMS----KNKKIE  181
            GF+     +   Q + M       KK+E
Sbjct  158  GFITFEDEQSVDQAVNMHFHDIMGKKVE  185


>ref|XP_007862855.1| hypothetical protein GLOTRDRAFT_35766 [Gloeophyllum trabeum ATCC 
11539]
 gb|EPQ58727.1| hypothetical protein GLOTRDRAFT_35766 [Gloeophyllum trabeum ATCC 
11539]
Length=131

 Score = 39.7 bits (91),  Expect = 0.56, Method: Compositional matrix adjust.
 Identities = 21/70 (30%), Positives = 34/70 (49%), Gaps = 0/70 (0%)

Query  103  SSRVDQTLFVAGIPNVAREQHLTEYFEDLGIIVRDVFVPSAFNQNGQKRKHNRGFGFVEV  162
             +R  +T+FV G+ +   E  L E F   G ++      +A NQ  Q    +RGFGF+  
Sbjct  4    GTRTKKTIFVGGLGDDVDEAVLVETFATFGDVIEVQLPQAATNQYQQAEAKHRGFGFITY  63

Query  163  GTVKEHQQLI  172
             + ++ Q  I
Sbjct  64   SSAEDAQDAI  73


>ref|XP_010409941.1| PREDICTED: DAZ-associated protein 1 [Corvus cornix cornix]
Length=416

 Score = 41.6 bits (96),  Expect = 0.57, Method: Compositional matrix adjust.
 Identities = 28/97 (29%), Positives = 48/97 (49%), Gaps = 15/97 (15%)

Query  98   TDTNGSSRVDQTLFVAGIPNVAREQHLTEYFEDLGIIVRDVFVPSAFNQNGQKRKHNRGF  157
            +D N S+++    FV GIP+   E  L EYF+  G++   V +  A      +++  RGF
Sbjct  115  SDNNKSNKI----FVGGIPHNCGETELREYFKKFGVVTEVVMIYDA------EKQRPRGF  164

Query  158  GFVEVGTVKEHQQLIRMSKNKKIEFDLKPAKWDIERA  194
            GF+      E +Q +  + N     D+   K +++RA
Sbjct  165  GFITF----EDEQSVDQAVNMHFH-DIMGKKVEVKRA  196


>ref|XP_010149380.1| PREDICTED: DAZ-associated protein 1 [Eurypyga helias]
Length=360

 Score = 41.6 bits (96),  Expect = 0.57, Method: Compositional matrix adjust.
 Identities = 26/88 (30%), Positives = 42/88 (48%), Gaps = 14/88 (16%)

Query  98   TDTNGSSRVDQTLFVAGIPNVAREQHLTEYFEDLGIIVRDVFVPSAFNQNGQKRKHNRGF  157
            +D N S+++    FV GIP+   E  L EYF+  G++   V +  A      +++  RGF
Sbjct  114  SDNNKSNKI----FVGGIPHNCGETELREYFKKFGVVTEVVMIYDA------EKQRPRGF  163

Query  158  GFVEVGTVKEHQQLIRMS----KNKKIE  181
            GF+     +   Q + M       KK+E
Sbjct  164  GFITFEDEQSVDQAVNMHFHDIMGKKVE  191


>ref|XP_010135175.1| PREDICTED: DAZ-associated protein 1, partial [Buceros rhinoceros 
silvestris]
Length=328

 Score = 41.2 bits (95),  Expect = 0.57, Method: Compositional matrix adjust.
 Identities = 28/97 (29%), Positives = 48/97 (49%), Gaps = 15/97 (15%)

Query  98   TDTNGSSRVDQTLFVAGIPNVAREQHLTEYFEDLGIIVRDVFVPSAFNQNGQKRKHNRGF  157
            +D N S+++    FV GIP+   E  L EYF+  G++   V +  A      +++  RGF
Sbjct  98   SDNNKSNKI----FVGGIPHNCGETELREYFKKFGVVTEVVMIYDA------EKQRPRGF  147

Query  158  GFVEVGTVKEHQQLIRMSKNKKIEFDLKPAKWDIERA  194
            GF+      E +Q +  + N     D+   K +++RA
Sbjct  148  GFITF----EDEQSVDQAVNMHFH-DIMGKKVEVKRA  179


>gb|KFW04747.1| DAZ-associated protein 1, partial [Eurypyga helias]
 gb|KFZ65253.1| DAZ-associated protein 1, partial [Podiceps cristatus]
Length=342

 Score = 41.6 bits (96),  Expect = 0.58, Method: Compositional matrix adjust.
 Identities = 26/88 (30%), Positives = 42/88 (48%), Gaps = 14/88 (16%)

Query  98   TDTNGSSRVDQTLFVAGIPNVAREQHLTEYFEDLGIIVRDVFVPSAFNQNGQKRKHNRGF  157
            +D N S+++    FV GIP+   E  L EYF+  G++   V +  A      +++  RGF
Sbjct  97   SDNNKSNKI----FVGGIPHNCGETELREYFKKFGVVTEVVMIYDA------EKQRPRGF  146

Query  158  GFVEVGTVKEHQQLIRMS----KNKKIE  181
            GF+     +   Q + M       KK+E
Sbjct  147  GFITFEDEQSVDQAVNMHFHDIMGKKVE  174


>gb|KKR02014.1| RNP-1 like protein RNA-binding protein [Parcubacteria (Nomurabacteria) 
bacterium GW2011_GWD2_39_12]
 gb|KKR20947.1| RNP-1 like protein RNA-binding protein [Parcubacteria (Nomurabacteria) 
bacterium GW2011_GWC2_39_41]
 gb|KKR37174.1| RNP-1 like protein RNA-binding protein [Parcubacteria (Nomurabacteria) 
bacterium GW2011_GWE2_40_10]
 6 more sequence titles

gb|KKR38896.1| RNP-1 like protein RNA-binding protein [Parcubacteria (Nomurabacteria) 
bacterium GW2011_GWB1_40_11]
 gb|KKR40138.1| RNP-1 like protein RNA-binding protein [Parcubacteria bacterium 
GW2011_GWC1_40_11]
 gb|KKR59283.1| RNP-1 like protein RNA-binding protein [Parcubacteria (Nomurabacteria) 
bacterium GW2011_GWF2_40_31]
 gb|KKR66551.1| RNP-1 like protein RNA-binding protein [Parcubacteria bacterium 
GW2011_GWF1_40_5]
 gb|KKR74956.1| RNP-1 like protein RNA-binding protein [Parcubacteria (Nomurabacteria) 
bacterium GW2011_GWA1_40_8]
 gb|KKR84081.1| RNP-1 like protein RNA-binding protein [Parcubacteria (Nomurabacteria) 
bacterium GW2011_GWA2_40_97]

Length=106

 Score = 39.3 bits (90),  Expect = 0.58, Method: Compositional matrix adjust.
 Identities = 22/72 (31%), Positives = 35/72 (49%), Gaps = 6/72 (8%)

Query  110  LFVAGIPNVAREQHLTEYFEDLGIIVRDVFVPSAFNQNGQKRKHNRGFGFVEVGTVKEHQ  169
            L+V G+P   +E  L E F   G +V  V +    +        ++GFGFVE+    E Q
Sbjct  5    LYVGGLPYSTQEDALKELFAQAGSVVSAVIIMDKMS------GRSKGFGFVEMSNNDEAQ  58

Query  170  QLIRMSKNKKIE  181
            + I M  +++ E
Sbjct  59   KAISMFNDQEFE  70


>ref|XP_002768166.1| pre-mRNA-splicing factor SF2, putative [Perkinsus marinus ATCC 
50983]
 gb|EER00884.1| pre-mRNA-splicing factor SF2, putative [Perkinsus marinus ATCC 
50983]
Length=354

 Score = 41.6 bits (96),  Expect = 0.58, Method: Compositional matrix adjust.
 Identities = 27/109 (25%), Positives = 51/109 (47%), Gaps = 10/109 (9%)

Query  86   TSTPLKSTALLDTDTNGSSRV--DQTLFVAGIPNVAREQHLTEYFEDLGIIVRDVFVPSA  143
            T+ P +    +    +G SR   D+ LF+ G+P    +QH+  YF   G +   + +   
Sbjct  7    TAPPQQGGNYVSGGGSGRSRESDDRKLFIGGLPGACEKQHMDSYFSQFGPVEHTMVM---  63

Query  144  FNQNGQKRKHNRGFGFVEVGTVKEHQQLIRMSKNKKIE--FDLKPAKWD  190
            +++N  +   +RGFGFV    + + +  +    +  +E   D+K A  D
Sbjct  64   YDRNTGR---SRGFGFVVYSQLTDMETCLASGPHVLLEKTVDVKRASQD  109


>ref|XP_009665809.1| PREDICTED: DAZ-associated protein 1, partial [Struthio camelus 
australis]
Length=412

 Score = 41.6 bits (96),  Expect = 0.58, Method: Compositional matrix adjust.
 Identities = 28/97 (29%), Positives = 48/97 (49%), Gaps = 15/97 (15%)

Query  98   TDTNGSSRVDQTLFVAGIPNVAREQHLTEYFEDLGIIVRDVFVPSAFNQNGQKRKHNRGF  157
            +D N S+++    FV GIP+   E  L EYF+  G++   V +  A      +++  RGF
Sbjct  111  SDNNKSNKI----FVGGIPHNCGETELREYFKKFGVVTEVVMIYDA------EKQRPRGF  160

Query  158  GFVEVGTVKEHQQLIRMSKNKKIEFDLKPAKWDIERA  194
            GF+      E +Q +  + N     D+   K +++RA
Sbjct  161  GFITF----EDEQSVDQAVNMHFH-DIMGKKVEVKRA  192


>ref|XP_012761430.1| RNA-binding protein, putative [Plasmodium reichenowi]
 emb|CDO62790.1| RNA-binding protein, putative [Plasmodium reichenowi]
Length=363

 Score = 41.6 bits (96),  Expect = 0.58, Method: Compositional matrix adjust.
 Identities = 20/64 (31%), Positives = 31/64 (48%), Gaps = 6/64 (9%)

Query  110  LFVAGIPNVAREQHLTEYFEDLGIIVRDVFVPSAFNQNGQKRKHNRGFGFVEVGTVKEHQ  169
             F+ GIP     +H+TEYFE  G +   V           + K NRGF FV + +    +
Sbjct  121  FFIGGIPQYITSKHITEYFEQYGTVQHVVIAQD------HETKRNRGFAFVTMASHINKE  174

Query  170  QLIR  173
            +++R
Sbjct  175  RILR  178


>ref|XP_010125130.1| PREDICTED: DAZ-associated protein 1, partial [Chlamydotis macqueenii]
Length=282

 Score = 41.2 bits (95),  Expect = 0.59, Method: Compositional matrix adjust.
 Identities = 28/97 (29%), Positives = 48/97 (49%), Gaps = 15/97 (15%)

Query  98   TDTNGSSRVDQTLFVAGIPNVAREQHLTEYFEDLGIIVRDVFVPSAFNQNGQKRKHNRGF  157
            +D N S+++    FV GIP+   E  L EYF+  G++   V +  A      +++  RGF
Sbjct  99   SDNNKSNKI----FVGGIPHNCGETELREYFKKFGVVTEVVMIYDA------EKQRPRGF  148

Query  158  GFVEVGTVKEHQQLIRMSKNKKIEFDLKPAKWDIERA  194
            GF+      E +Q +  + N     D+   K +++RA
Sbjct  149  GFITF----EDEQSVDQAVNMHFH-DIMGKKVEVKRA  180


>emb|CEM35350.1| unnamed protein product [Vitrella brassicaformis CCMP3155]
Length=471

 Score = 41.6 bits (96),  Expect = 0.60, Method: Compositional matrix adjust.
 Identities = 29/86 (34%), Positives = 45/86 (52%), Gaps = 12/86 (14%)

Query  110  LFVAGIPNVAREQHLTEYFEDLGIIVRDVFVPSAFNQNGQKRKHNRGFGFVEVGTVKEHQ  169
            +FV G+P+V  ++ L EYF   G I +D  V    + N    KH RGFGFV+    +   
Sbjct  116  IFVGGLPSVCNKEMLEEYFSSFGAI-KDCIVMVDRDTN----KH-RGFGFVDFEDPETVD  169

Query  170  QLIRMSKNKKIEFDLKPAKW-DIERA  194
            ++I+   +  IE      KW +++RA
Sbjct  170  EVIKRFNDHYIE-----NKWIEVKRA  190


>ref|XP_013800769.1| PREDICTED: DAZ-associated protein 1 isoform X1 [Apteryx australis 
mantelli]
Length=409

 Score = 41.6 bits (96),  Expect = 0.60, Method: Compositional matrix adjust.
 Identities = 28/97 (29%), Positives = 48/97 (49%), Gaps = 15/97 (15%)

Query  98   TDTNGSSRVDQTLFVAGIPNVAREQHLTEYFEDLGIIVRDVFVPSAFNQNGQKRKHNRGF  157
            +D N S+++    FV GIP+   E  L EYF+  G++   V +  A      +++  RGF
Sbjct  108  SDNNKSNKI----FVGGIPHNCGETELREYFKKFGVVTEVVMIYDA------EKQRPRGF  157

Query  158  GFVEVGTVKEHQQLIRMSKNKKIEFDLKPAKWDIERA  194
            GF+      E +Q +  + N     D+   K +++RA
Sbjct  158  GFITF----EDEQSVDQAVNMHFH-DIMGKKVEVKRA  189


>ref|XP_009278158.1| PREDICTED: DAZ-associated protein 1 [Aptenodytes forsteri]
Length=427

 Score = 41.6 bits (96),  Expect = 0.61, Method: Compositional matrix adjust.
 Identities = 28/97 (29%), Positives = 48/97 (49%), Gaps = 15/97 (15%)

Query  98   TDTNGSSRVDQTLFVAGIPNVAREQHLTEYFEDLGIIVRDVFVPSAFNQNGQKRKHNRGF  157
            +D N S+++    FV GIP+   E  L EYF+  G++   V +  A      +++  RGF
Sbjct  126  SDNNKSNKI----FVGGIPHNCGETELREYFKKFGVVTEVVMIYDA------EKQRPRGF  175

Query  158  GFVEVGTVKEHQQLIRMSKNKKIEFDLKPAKWDIERA  194
            GF+      E +Q +  + N     D+   K +++RA
Sbjct  176  GFITF----EDEQSVDQAVNMHFH-DIMGKKVEVKRA  207


>ref|XP_009907611.1| PREDICTED: heterogeneous nuclear ribonucleoproteins A2/B1-like, 
partial [Picoides pubescens]
Length=118

 Score = 39.3 bits (90),  Expect = 0.61, Method: Compositional matrix adjust.
 Identities = 26/72 (36%), Positives = 36/72 (50%), Gaps = 6/72 (8%)

Query  89   PLKSTALLDTDTNGSSRVDQTLFVAGIPNVAREQHLTEYFEDLGIIVRDVFVPSAFNQNG  148
            P ++ A  ++   G+    + LFV GI     E HL +YFE+ G I  D        Q+G
Sbjct  42   PKRAVAREESGKPGAHVTVKKLFVGGIKEDTEEHHLRDYFEEYGKI--DTIEIITDRQSG  99

Query  149  QKRKHNRGFGFV  160
            +K    RGFGFV
Sbjct  100  KK----RGFGFV  107


>ref|XP_005060220.1| PREDICTED: DAZ-associated protein 1 [Ficedula albicollis]
Length=418

 Score = 41.6 bits (96),  Expect = 0.61, Method: Compositional matrix adjust.
 Identities = 28/97 (29%), Positives = 48/97 (49%), Gaps = 15/97 (15%)

Query  98   TDTNGSSRVDQTLFVAGIPNVAREQHLTEYFEDLGIIVRDVFVPSAFNQNGQKRKHNRGF  157
            +D N S+++    FV GIP+   E  L EYF+  G++   V +  A      +++  RGF
Sbjct  117  SDNNKSNKI----FVGGIPHNCGETELREYFKKFGVVTEVVMIYDA------EKQRPRGF  166

Query  158  GFVEVGTVKEHQQLIRMSKNKKIEFDLKPAKWDIERA  194
            GF+      E +Q +  + N     D+   K +++RA
Sbjct  167  GFITF----EDEQSVDQAVNMHFH-DIMGKKVEVKRA  198


>ref|XP_006017617.1| PREDICTED: DAZ-associated protein 1 [Alligator sinensis]
Length=402

 Score = 41.6 bits (96),  Expect = 0.61, Method: Compositional matrix adjust.
 Identities = 28/97 (29%), Positives = 48/97 (49%), Gaps = 15/97 (15%)

Query  98   TDTNGSSRVDQTLFVAGIPNVAREQHLTEYFEDLGIIVRDVFVPSAFNQNGQKRKHNRGF  157
            +D N S+++    FV GIP+   E  L EYF+  G++   V +  A      +++  RGF
Sbjct  101  SDNNKSNKI----FVGGIPHNCGETELREYFKKFGVVTEVVMIYDA------EKQRPRGF  150

Query  158  GFVEVGTVKEHQQLIRMSKNKKIEFDLKPAKWDIERA  194
            GF+      E +Q +  + N     D+   K +++RA
Sbjct  151  GFITF----EDEQSVDQAVNMHFH-DIMGKKVEVKRA  182


>gb|KFQ36552.1| DAZ-associated protein 1, partial [Merops nubicus]
Length=322

 Score = 41.2 bits (95),  Expect = 0.61, Method: Compositional matrix adjust.
 Identities = 26/88 (30%), Positives = 42/88 (48%), Gaps = 14/88 (16%)

Query  98   TDTNGSSRVDQTLFVAGIPNVAREQHLTEYFEDLGIIVRDVFVPSAFNQNGQKRKHNRGF  157
            +D N S+++    FV GIP+   E  L EYF+  G++   V +  A      +++  RGF
Sbjct  97   SDNNKSNKI----FVGGIPHNCGETELREYFKKFGVVTEVVMIYDA------EKQRPRGF  146

Query  158  GFVEVGTVKEHQQLIRMS----KNKKIE  181
            GF+     +   Q + M       KK+E
Sbjct  147  GFITFEDEQSVDQAVNMHFHDIMGKKVE  174


>gb|ETW56723.1| hypothetical protein PFUGPA_01513 [Plasmodium falciparum Palo 
Alto/Uganda]
Length=268

 Score = 41.2 bits (95),  Expect = 0.61, Method: Compositional matrix adjust.
 Identities = 25/86 (29%), Positives = 41/86 (48%), Gaps = 9/86 (10%)

Query  90   LKSTALLDTDTNGSSRVDQ--TLFVAGIPNVAREQHLTEYFEDLGIIVRDVFVPSAFNQN  147
            L ST + +   N S  +++    F+ GIP     +H+TEYFE  G +   V         
Sbjct  5    LASTGINEC-LNNSKNLNEPFKFFIGGIPQYITSKHITEYFEQYGTVQHVVIAQD-----  58

Query  148  GQKRKHNRGFGFVEVGTVKEHQQLIR  173
              + K NRGF FV + +    ++++R
Sbjct  59   -HETKRNRGFAFVTMASHINKERILR  83


>ref|XP_012397671.1| PREDICTED: DAZ-associated protein 1 [Sarcophilus harrisii]
Length=558

 Score = 41.6 bits (96),  Expect = 0.62, Method: Compositional matrix adjust.
 Identities = 28/97 (29%), Positives = 48/97 (49%), Gaps = 15/97 (15%)

Query  98   TDTNGSSRVDQTLFVAGIPNVAREQHLTEYFEDLGIIVRDVFVPSAFNQNGQKRKHNRGF  157
            +D N S+++    FV GIP+   E  L EYF+  G++   V +  A      +++  RGF
Sbjct  258  SDNNKSNKI----FVGGIPHNCGETELREYFKKFGVVTEVVMIYDA------EKQRPRGF  307

Query  158  GFVEVGTVKEHQQLIRMSKNKKIEFDLKPAKWDIERA  194
            GF+      E +Q +  + N     D+   K +++RA
Sbjct  308  GFITF----EDEQSVDQAVNMHFH-DIMGKKVEVKRA  339


>ref|XP_009897503.1| PREDICTED: LOW QUALITY PROTEIN: DAZ-associated protein 1 [Picoides 
pubescens]
Length=435

 Score = 41.6 bits (96),  Expect = 0.62, Method: Compositional matrix adjust.
 Identities = 28/97 (29%), Positives = 48/97 (49%), Gaps = 15/97 (15%)

Query  98   TDTNGSSRVDQTLFVAGIPNVAREQHLTEYFEDLGIIVRDVFVPSAFNQNGQKRKHNRGF  157
            +D N S+++    FV GIP+   E  L EYF+  G++   V +  A      +++  RGF
Sbjct  126  SDNNKSNKI----FVGGIPHNCGETELREYFKKFGVVTEVVMIYDA------EKQRPRGF  175

Query  158  GFVEVGTVKEHQQLIRMSKNKKIEFDLKPAKWDIERA  194
            GF+      E +Q +  + N     D+   K +++RA
Sbjct  176  GFITF----EDEQSVDQAVNMHFH-DIMGKKVEVKRA  207


>ref|XP_010003630.1| PREDICTED: DAZ-associated protein 1 [Chaetura pelagica]
Length=418

 Score = 41.6 bits (96),  Expect = 0.62, Method: Compositional matrix adjust.
 Identities = 28/97 (29%), Positives = 48/97 (49%), Gaps = 15/97 (15%)

Query  98   TDTNGSSRVDQTLFVAGIPNVAREQHLTEYFEDLGIIVRDVFVPSAFNQNGQKRKHNRGF  157
            +D N S+++    FV GIP+   E  L EYF+  G++   V +  A      +++  RGF
Sbjct  117  SDNNKSNKI----FVGGIPHNCGETELREYFKKFGVVTEVVMIYDA------EKQRPRGF  166

Query  158  GFVEVGTVKEHQQLIRMSKNKKIEFDLKPAKWDIERA  194
            GF+      E +Q +  + N     D+   K +++RA
Sbjct  167  GFITF----EDEQSVDQAVNMHFH-DIMGKKVEVKRA  198


>emb|CEP60191.1| LALA0S01e05072g1_1 [Lachancea lanzarotensis]
Length=352

 Score = 41.2 bits (95),  Expect = 0.63, Method: Compositional matrix adjust.
 Identities = 31/96 (32%), Positives = 47/96 (49%), Gaps = 8/96 (8%)

Query  106  VDQTLFVAGIPNVAREQHLTEYFEDLGIIVRDVFVPSAFNQNGQKRKHNRGFGFVEVGTV  165
            +  TL V+G P   R + L   FE +G +VR + +P A ++ G      R + FVE  T 
Sbjct  3    IGSTLHVSGFPRGVRTRDLAPDFESVGRVVR-IEMPPARSEFG------RPYAFVEYETP  55

Query  166  KEHQQLIRMSKNKKIEFDLKPAKWDIERARSYGTPS  201
            +E Q  I     + + FD + A   ++ ARS   PS
Sbjct  56   EEAQDAIHQLNQRPLSFDPQ-AIITVQVARSEARPS  90


>ref|XP_009924112.1| PREDICTED: DAZ-associated protein 1, partial [Haliaeetus albicilla]
 gb|KFP92974.1| DAZ-associated protein 1, partial [Haliaeetus albicilla]
Length=398

 Score = 41.2 bits (95),  Expect = 0.64, Method: Compositional matrix adjust.
 Identities = 28/97 (29%), Positives = 48/97 (49%), Gaps = 15/97 (15%)

Query  98   TDTNGSSRVDQTLFVAGIPNVAREQHLTEYFEDLGIIVRDVFVPSAFNQNGQKRKHNRGF  157
            +D N S+++    FV GIP+   E  L EYF+  G++   V +  A      +++  RGF
Sbjct  97   SDNNKSNKI----FVGGIPHNCGETELREYFKKFGVVTEVVMIYDA------EKQRPRGF  146

Query  158  GFVEVGTVKEHQQLIRMSKNKKIEFDLKPAKWDIERA  194
            GF+      E +Q +  + N     D+   K +++RA
Sbjct  147  GFITF----EDEQSVDQAVNMHFH-DIMGKKVEVKRA  178


>ref|XP_010071977.1| PREDICTED: DAZ-associated protein 1 [Pterocles gutturalis]
Length=410

 Score = 41.2 bits (95),  Expect = 0.65, Method: Compositional matrix adjust.
 Identities = 28/97 (29%), Positives = 48/97 (49%), Gaps = 15/97 (15%)

Query  98   TDTNGSSRVDQTLFVAGIPNVAREQHLTEYFEDLGIIVRDVFVPSAFNQNGQKRKHNRGF  157
            +D N S+++    FV GIP+   E  L EYF+  G++   V +  A      +++  RGF
Sbjct  109  SDNNKSNKI----FVGGIPHNCGETELREYFKKFGVVTEVVMIYDA------EKQRPRGF  158

Query  158  GFVEVGTVKEHQQLIRMSKNKKIEFDLKPAKWDIERA  194
            GF+      E +Q +  + N     D+   K +++RA
Sbjct  159  GFITF----EDEQSVDQAVNMHFH-DIMGKKVEVKRA  190


>ref|XP_006267895.1| PREDICTED: DAZ-associated protein 1 isoform X1 [Alligator mississippiensis]
Length=409

 Score = 41.2 bits (95),  Expect = 0.65, Method: Compositional matrix adjust.
 Identities = 28/97 (29%), Positives = 48/97 (49%), Gaps = 15/97 (15%)

Query  98   TDTNGSSRVDQTLFVAGIPNVAREQHLTEYFEDLGIIVRDVFVPSAFNQNGQKRKHNRGF  157
            +D N S+++    FV GIP+   E  L EYF+  G++   V +  A      +++  RGF
Sbjct  108  SDNNKSNKI----FVGGIPHNCGETELREYFKKFGVVTEVVMIYDA------EKQRPRGF  157

Query  158  GFVEVGTVKEHQQLIRMSKNKKIEFDLKPAKWDIERA  194
            GF+      E +Q +  + N     D+   K +++RA
Sbjct  158  GFITF----EDEQSVDQAVNMHFH-DIMGKKVEVKRA  189


>ref|XP_009566610.1| PREDICTED: DAZ-associated protein 1 [Cuculus canorus]
Length=409

 Score = 41.2 bits (95),  Expect = 0.66, Method: Compositional matrix adjust.
 Identities = 28/97 (29%), Positives = 48/97 (49%), Gaps = 15/97 (15%)

Query  98   TDTNGSSRVDQTLFVAGIPNVAREQHLTEYFEDLGIIVRDVFVPSAFNQNGQKRKHNRGF  157
            +D N S+++    FV GIP+   E  L EYF+  G++   V +  A      +++  RGF
Sbjct  108  SDNNKSNKI----FVGGIPHNCGETELREYFKKFGVVTEVVMIYDA------EKQRPRGF  157

Query  158  GFVEVGTVKEHQQLIRMSKNKKIEFDLKPAKWDIERA  194
            GF+      E +Q +  + N     D+   K +++RA
Sbjct  158  GFITF----EDEQSVDQAVNMHFH-DIMGKKVEVKRA  189


>ref|XP_009327196.1| PREDICTED: DAZ-associated protein 1 [Pygoscelis adeliae]
Length=437

 Score = 41.2 bits (95),  Expect = 0.66, Method: Compositional matrix adjust.
 Identities = 28/97 (29%), Positives = 48/97 (49%), Gaps = 15/97 (15%)

Query  98   TDTNGSSRVDQTLFVAGIPNVAREQHLTEYFEDLGIIVRDVFVPSAFNQNGQKRKHNRGF  157
            +D N S+++    FV GIP+   E  L EYF+  G++   V +  A      +++  RGF
Sbjct  136  SDNNKSNKI----FVGGIPHNCGETELREYFKKFGVVTEVVMIYDA------EKQRPRGF  185

Query  158  GFVEVGTVKEHQQLIRMSKNKKIEFDLKPAKWDIERA  194
            GF+      E +Q +  + N     D+   K +++RA
Sbjct  186  GFITF----EDEQSVDQAVNMHFH-DIMGKKVEVKRA  217


>ref|XP_008639317.1| PREDICTED: DAZ-associated protein 1 [Corvus brachyrhynchos]
Length=417

 Score = 41.2 bits (95),  Expect = 0.67, Method: Compositional matrix adjust.
 Identities = 28/97 (29%), Positives = 48/97 (49%), Gaps = 15/97 (15%)

Query  98   TDTNGSSRVDQTLFVAGIPNVAREQHLTEYFEDLGIIVRDVFVPSAFNQNGQKRKHNRGF  157
            +D N S+++    FV GIP+   E  L EYF+  G++   V +  A      +++  RGF
Sbjct  116  SDNNKSNKI----FVGGIPHNCGETELREYFKKFGVVTEVVMIYDA------EKQRPRGF  165

Query  158  GFVEVGTVKEHQQLIRMSKNKKIEFDLKPAKWDIERA  194
            GF+      E +Q +  + N     D+   K +++RA
Sbjct  166  GFITF----EDEQSVDQAVNMHFH-DIMGKKVEVKRA  197


>gb|KFR13441.1| DAZ-associated protein 1, partial [Opisthocomus hoazin]
Length=398

 Score = 41.2 bits (95),  Expect = 0.67, Method: Compositional matrix adjust.
 Identities = 28/97 (29%), Positives = 48/97 (49%), Gaps = 15/97 (15%)

Query  98   TDTNGSSRVDQTLFVAGIPNVAREQHLTEYFEDLGIIVRDVFVPSAFNQNGQKRKHNRGF  157
            +D N S+++    FV GIP+   E  L EYF+  G++   V +  A      +++  RGF
Sbjct  97   SDNNKSNKI----FVGGIPHNCGETELREYFKKFGVVTEVVMIYDA------EKQRPRGF  146

Query  158  GFVEVGTVKEHQQLIRMSKNKKIEFDLKPAKWDIERA  194
            GF+      E +Q +  + N     D+   K +++RA
Sbjct  147  GFITF----EDEQSVDQAVNMHFH-DIMGKKVEVKRA  178


>ref|XP_011594903.1| PREDICTED: DAZ-associated protein 1 [Aquila chrysaetos canadensis]
Length=405

 Score = 41.2 bits (95),  Expect = 0.68, Method: Compositional matrix adjust.
 Identities = 28/97 (29%), Positives = 48/97 (49%), Gaps = 15/97 (15%)

Query  98   TDTNGSSRVDQTLFVAGIPNVAREQHLTEYFEDLGIIVRDVFVPSAFNQNGQKRKHNRGF  157
            +D N S+++    FV GIP+   E  L EYF+  G++   V +  A      +++  RGF
Sbjct  104  SDNNKSNKI----FVGGIPHNCGETELREYFKKFGVVTEVVMIYDA------EKQRPRGF  153

Query  158  GFVEVGTVKEHQQLIRMSKNKKIEFDLKPAKWDIERA  194
            GF+      E +Q +  + N     D+   K +++RA
Sbjct  154  GFITF----EDEQSVDQAVNMHFH-DIMGKKVEVKRA  185


>ref|XP_009882852.1| PREDICTED: DAZ-associated protein 1 [Charadrius vociferus]
Length=408

 Score = 41.2 bits (95),  Expect = 0.68, Method: Compositional matrix adjust.
 Identities = 28/97 (29%), Positives = 48/97 (49%), Gaps = 15/97 (15%)

Query  98   TDTNGSSRVDQTLFVAGIPNVAREQHLTEYFEDLGIIVRDVFVPSAFNQNGQKRKHNRGF  157
            +D N S+++    FV GIP+   E  L EYF+  G++   V +  A      +++  RGF
Sbjct  107  SDNNKSNKI----FVGGIPHNCGETELREYFKKFGVVTEVVMIYDA------EKQRPRGF  156

Query  158  GFVEVGTVKEHQQLIRMSKNKKIEFDLKPAKWDIERA  194
            GF+      E +Q +  + N     D+   K +++RA
Sbjct  157  GFITF----EDEQSVDQAVNMHFH-DIMGKKVEVKRA  188


>ref|XP_009957872.1| PREDICTED: DAZ-associated protein 1 [Leptosomus discolor]
Length=400

 Score = 41.2 bits (95),  Expect = 0.68, Method: Compositional matrix adjust.
 Identities = 28/97 (29%), Positives = 48/97 (49%), Gaps = 15/97 (15%)

Query  98   TDTNGSSRVDQTLFVAGIPNVAREQHLTEYFEDLGIIVRDVFVPSAFNQNGQKRKHNRGF  157
            +D N S+++    FV GIP+   E  L EYF+  G++   V +  A      +++  RGF
Sbjct  99   SDNNKSNKI----FVGGIPHNCGETELREYFKKFGVVTEVVMIYDA------EKQRPRGF  148

Query  158  GFVEVGTVKEHQQLIRMSKNKKIEFDLKPAKWDIERA  194
            GF+      E +Q +  + N     D+   K +++RA
Sbjct  149  GFITF----EDEQSVDQAVNMHFH-DIMGKKVEVKRA  180


>ref|XP_009639347.1| PREDICTED: DAZ-associated protein 1 [Egretta garzetta]
Length=403

 Score = 41.2 bits (95),  Expect = 0.68, Method: Compositional matrix adjust.
 Identities = 28/97 (29%), Positives = 48/97 (49%), Gaps = 15/97 (15%)

Query  98   TDTNGSSRVDQTLFVAGIPNVAREQHLTEYFEDLGIIVRDVFVPSAFNQNGQKRKHNRGF  157
            +D N S+++    FV GIP+   E  L EYF+  G++   V +  A      +++  RGF
Sbjct  102  SDNNKSNKI----FVGGIPHNCGETELREYFKKFGVVTEVVMIYDA------EKQRPRGF  151

Query  158  GFVEVGTVKEHQQLIRMSKNKKIEFDLKPAKWDIERA  194
            GF+      E +Q +  + N     D+   K +++RA
Sbjct  152  GFITF----EDEQSVDQAVNMHFH-DIMGKKVEVKRA  183


>ref|XP_005428644.1| PREDICTED: DAZ-associated protein 1 [Geospiza fortis]
Length=496

 Score = 41.2 bits (95),  Expect = 0.69, Method: Compositional matrix adjust.
 Identities = 26/88 (30%), Positives = 42/88 (48%), Gaps = 14/88 (16%)

Query  98   TDTNGSSRVDQTLFVAGIPNVAREQHLTEYFEDLGIIVRDVFVPSAFNQNGQKRKHNRGF  157
            +D N S+++    FV GIP+   E  L EYF+  G++   V +  A      +++  RGF
Sbjct  165  SDNNKSNKI----FVGGIPHNCGETELREYFKKFGVVTEVVMIYDA------EKQRPRGF  214

Query  158  GFVEVGTVKEHQQLIRMS----KNKKIE  181
            GF+     +   Q + M       KK+E
Sbjct  215  GFITFEDEQSVDQAVNMHFHDIMGKKVE  242


>ref|XP_013157363.1| PREDICTED: DAZ-associated protein 1 [Falco peregrinus]
Length=404

 Score = 41.2 bits (95),  Expect = 0.70, Method: Compositional matrix adjust.
 Identities = 28/97 (29%), Positives = 48/97 (49%), Gaps = 15/97 (15%)

Query  98   TDTNGSSRVDQTLFVAGIPNVAREQHLTEYFEDLGIIVRDVFVPSAFNQNGQKRKHNRGF  157
            +D N S+++    FV GIP+   E  L EYF+  G++   V +  A      +++  RGF
Sbjct  103  SDNNKSNKI----FVGGIPHNCGETELREYFKKFGVVTEVVMIYDA------EKQRPRGF  152

Query  158  GFVEVGTVKEHQQLIRMSKNKKIEFDLKPAKWDIERA  194
            GF+      E +Q +  + N     D+   K +++RA
Sbjct  153  GFITF----EDEQSVDQAVNMHFH-DIMGKKVEVKRA  184


>ref|XP_010561581.1| PREDICTED: DAZ-associated protein 1 [Haliaeetus leucocephalus]
Length=426

 Score = 41.2 bits (95),  Expect = 0.70, Method: Compositional matrix adjust.
 Identities = 28/97 (29%), Positives = 48/97 (49%), Gaps = 15/97 (15%)

Query  98   TDTNGSSRVDQTLFVAGIPNVAREQHLTEYFEDLGIIVRDVFVPSAFNQNGQKRKHNRGF  157
            +D N S+++    FV GIP+   E  L EYF+  G++   V +  A      +++  RGF
Sbjct  125  SDNNKSNKI----FVGGIPHNCGETELREYFKKFGVVTEVVMIYDA------EKQRPRGF  174

Query  158  GFVEVGTVKEHQQLIRMSKNKKIEFDLKPAKWDIERA  194
            GF+      E +Q +  + N     D+   K +++RA
Sbjct  175  GFITF----EDEQSVDQAVNMHFH-DIMGKKVEVKRA  206


>ref|XP_001351452.1| RNA binding protein, putative [Plasmodium falciparum 3D7]
 emb|CAD49181.1| RNA binding protein, putative [Plasmodium falciparum 3D7]
 gb|ETW20286.1| hypothetical protein PFFVO_00762 [Plasmodium falciparum Vietnam 
Oak-Knoll (FVO)]
 gb|ETW44781.1| hypothetical protein PFNF135_00836 [Plasmodium falciparum NF135/5.C10]
 gb|EUT91380.1| hypothetical protein PFAG_00714 [Plasmodium falciparum Santa 
Lucia]
Length=358

 Score = 41.2 bits (95),  Expect = 0.70, Method: Compositional matrix adjust.
 Identities = 20/64 (31%), Positives = 31/64 (48%), Gaps = 6/64 (9%)

Query  110  LFVAGIPNVAREQHLTEYFEDLGIIVRDVFVPSAFNQNGQKRKHNRGFGFVEVGTVKEHQ  169
             F+ GIP     +H+TEYFE  G +   V           + K NRGF FV + +    +
Sbjct  116  FFIGGIPQYITSKHITEYFEQYGTVQHVVIAQD------HETKRNRGFAFVTMASHINKE  169

Query  170  QLIR  173
            +++R
Sbjct  170  RILR  173


>ref|XP_009980991.1| PREDICTED: DAZ-associated protein 1, partial [Tauraco erythrolophus]
 gb|KFV00955.1| DAZ-associated protein 1, partial [Tauraco erythrolophus]
Length=398

 Score = 41.2 bits (95),  Expect = 0.70, Method: Compositional matrix adjust.
 Identities = 28/97 (29%), Positives = 48/97 (49%), Gaps = 15/97 (15%)

Query  98   TDTNGSSRVDQTLFVAGIPNVAREQHLTEYFEDLGIIVRDVFVPSAFNQNGQKRKHNRGF  157
            +D N S+++    FV GIP+   E  L EYF+  G++   V +  A      +++  RGF
Sbjct  97   SDNNKSNKI----FVGGIPHNCGETELREYFKKFGVVTEVVMIYDA------EKQRPRGF  146

Query  158  GFVEVGTVKEHQQLIRMSKNKKIEFDLKPAKWDIERA  194
            GF+      E +Q +  + N     D+   K +++RA
Sbjct  147  GFITF----EDEQSVDQAVNMHFH-DIMGKKVEVKRA  178


>ref|XP_009490696.1| PREDICTED: DAZ-associated protein 1, partial [Pelecanus crispus]
 ref|XP_009967361.1| PREDICTED: DAZ-associated protein 1, partial [Tyto alba]
 ref|XP_010171482.1| PREDICTED: DAZ-associated protein 1, partial [Caprimulgus carolinensis]
 12 more sequence titles

ref|XP_010196193.1| PREDICTED: DAZ-associated protein 1, partial [Colius striatus]
 ref|XP_010295144.1| PREDICTED: DAZ-associated protein 1, partial [Phaethon lepturus]
 gb|KFM07908.1| DAZ-associated protein 1, partial [Aptenodytes forsteri]
 gb|KFP17837.1| DAZ-associated protein 1, partial [Egretta garzetta]
 gb|KFP23467.1| DAZ-associated protein 1, partial [Colius striatus]
 gb|KFQ29653.1| DAZ-associated protein 1, partial [Mesitornis unicolor]
 gb|KFQ57925.1| DAZ-associated protein 1, partial [Pelecanus crispus]
 gb|KFR01454.1| DAZ-associated protein 1, partial [Nipponia nippon]
 gb|KFV54182.1| DAZ-associated protein 1, partial [Tyto alba]
 gb|KFV77535.1| DAZ-associated protein 1, partial [Struthio camelus australis]
 gb|KFW70524.1| DAZ-associated protein 1, partial [Pygoscelis adeliae]
 gb|KFZ63375.1| DAZ-associated protein 1, partial [Caprimulgus carolinensis]

Length=398

 Score = 41.2 bits (95),  Expect = 0.70, Method: Compositional matrix adjust.
 Identities = 28/97 (29%), Positives = 48/97 (49%), Gaps = 15/97 (15%)

Query  98   TDTNGSSRVDQTLFVAGIPNVAREQHLTEYFEDLGIIVRDVFVPSAFNQNGQKRKHNRGF  157
            +D N S+++    FV GIP+   E  L EYF+  G++   V +  A      +++  RGF
Sbjct  97   SDNNKSNKI----FVGGIPHNCGETELREYFKKFGVVTEVVMIYDA------EKQRPRGF  146

Query  158  GFVEVGTVKEHQQLIRMSKNKKIEFDLKPAKWDIERA  194
            GF+      E +Q +  + N     D+   K +++RA
Sbjct  147  GFITF----EDEQSVDQAVNMHFH-DIMGKKVEVKRA  178


>ref|XP_010193137.1| PREDICTED: DAZ-associated protein 1 [Mesitornis unicolor]
Length=409

 Score = 41.2 bits (95),  Expect = 0.71, Method: Compositional matrix adjust.
 Identities = 28/97 (29%), Positives = 48/97 (49%), Gaps = 15/97 (15%)

Query  98   TDTNGSSRVDQTLFVAGIPNVAREQHLTEYFEDLGIIVRDVFVPSAFNQNGQKRKHNRGF  157
            +D N S+++    FV GIP+   E  L EYF+  G++   V +  A      +++  RGF
Sbjct  108  SDNNKSNKI----FVGGIPHNCGETELREYFKKFGVVTEVVMIYDA------EKQRPRGF  157

Query  158  GFVEVGTVKEHQQLIRMSKNKKIEFDLKPAKWDIERA  194
            GF+      E +Q +  + N     D+   K +++RA
Sbjct  158  GFITF----EDEQSVDQAVNMHFH-DIMGKKVEVKRA  189


>ref|XP_009467366.1| PREDICTED: DAZ-associated protein 1 [Nipponia nippon]
Length=412

 Score = 41.2 bits (95),  Expect = 0.71, Method: Compositional matrix adjust.
 Identities = 28/97 (29%), Positives = 48/97 (49%), Gaps = 15/97 (15%)

Query  98   TDTNGSSRVDQTLFVAGIPNVAREQHLTEYFEDLGIIVRDVFVPSAFNQNGQKRKHNRGF  157
            +D N S+++    FV GIP+   E  L EYF+  G++   V +  A      +++  RGF
Sbjct  111  SDNNKSNKI----FVGGIPHNCGETELREYFKKFGVVTEVVMIYDA------EKQRPRGF  160

Query  158  GFVEVGTVKEHQQLIRMSKNKKIEFDLKPAKWDIERA  194
            GF+      E +Q +  + N     D+   K +++RA
Sbjct  161  GFITF----EDEQSVDQAVNMHFH-DIMGKKVEVKRA  192


>gb|EOA98730.1| DAZ-associated protein 1, partial [Anas platyrhynchos]
Length=390

 Score = 41.2 bits (95),  Expect = 0.71, Method: Compositional matrix adjust.
 Identities = 28/97 (29%), Positives = 48/97 (49%), Gaps = 15/97 (15%)

Query  98   TDTNGSSRVDQTLFVAGIPNVAREQHLTEYFEDLGIIVRDVFVPSAFNQNGQKRKHNRGF  157
            +D N S+++    FV GIP+   E  L EYF+  G++   V +  A      +++  RGF
Sbjct  89   SDNNKSNKI----FVGGIPHNCGETELREYFKKFGVVTEVVMIYDA------EKQRPRGF  138

Query  158  GFVEVGTVKEHQQLIRMSKNKKIEFDLKPAKWDIERA  194
            GF+      E +Q +  + N     D+   K +++RA
Sbjct  139  GFITF----EDEQSVDQAVNMHFH-DIMGKKVEVKRA  170


>ref|XP_009577426.1| PREDICTED: DAZ-associated protein 1, partial [Fulmarus glacialis]
 gb|KFW06037.1| DAZ-associated protein 1, partial [Fulmarus glacialis]
Length=398

 Score = 41.2 bits (95),  Expect = 0.71, Method: Compositional matrix adjust.
 Identities = 28/97 (29%), Positives = 48/97 (49%), Gaps = 15/97 (15%)

Query  98   TDTNGSSRVDQTLFVAGIPNVAREQHLTEYFEDLGIIVRDVFVPSAFNQNGQKRKHNRGF  157
            +D N S+++    FV GIP+   E  L EYF+  G++   V +  A      +++  RGF
Sbjct  97   SDNNKSNKI----FVGGIPHNCGETELREYFKKFGVVTEVVMIYDA------EKQRPRGF  146

Query  158  GFVEVGTVKEHQQLIRMSKNKKIEFDLKPAKWDIERA  194
            GF+      E +Q +  + N     D+   K +++RA
Sbjct  147  GFITF----EDEQSVDQAVNMHFH-DIMGKKVEVKRA  178


>ref|XP_011526210.1| PREDICTED: DAZ-associated protein 1 isoform X5 [Homo sapiens]
 ref|XP_011526211.1| PREDICTED: DAZ-associated protein 1 isoform X5 [Homo sapiens]
Length=521

 Score = 41.2 bits (95),  Expect = 0.71, Method: Compositional matrix adjust.
 Identities = 23/76 (30%), Positives = 36/76 (47%), Gaps = 10/76 (13%)

Query  110  LFVAGIPNVAREQHLTEYFEDLGIIVRDVFVPSAFNQNGQKRKHNRGFGFVEVGTVKEHQ  169
            +FV GIP+   E  L EYF+  G++   V +  A      +++  RGFGF+     +   
Sbjct  258  IFVGGIPHNCGETELREYFKKFGVVTEVVMIYDA------EKQRPRGFGFITFEDEQSVD  311

Query  170  QLIRMS----KNKKIE  181
            Q + M       KK+E
Sbjct  312  QAVNMHFHDIMGKKVE  327


>ref|XP_006898062.1| PREDICTED: DAZ-associated protein 1 [Elephantulus edwardii]
Length=692

 Score = 41.6 bits (96),  Expect = 0.71, Method: Compositional matrix adjust.
 Identities = 29/110 (26%), Positives = 51/110 (46%), Gaps = 27/110 (25%)

Query  110  LFVAGIPNVAREQHLTEYFEDLGIIVRDVFVPSAFNQNGQKRKHNRGFGFVEVGTVKEHQ  169
            +FV GIP+   E  L EYF+  G++   V +  A      +++  RGFGF+      E +
Sbjct  400  IFVGGIPHNCGETELREYFKKFGVVTEVVMIYDA------EKQRPRGFGFITF----EDE  449

Query  170  QLIRMSKNKKIEFDLKPAKWDIERARSYGTPSPSIAPKIESKPKDSKARP  219
            Q +  + N     D+   K +++RA                +P+D+K++P
Sbjct  450  QSVDQAVNMHFH-DIMGKKVEVKRA----------------EPRDNKSQP  482


>gb|ETW38431.1| hypothetical protein PFTANZ_00862 [Plasmodium falciparum Tanzania 
(2000708)]
 gb|EUR78646.1| hypothetical protein PFBG_00741 [Plasmodium falciparum 7G8]
Length=291

 Score = 40.8 bits (94),  Expect = 0.72, Method: Compositional matrix adjust.
 Identities = 20/64 (31%), Positives = 31/64 (48%), Gaps = 6/64 (9%)

Query  110  LFVAGIPNVAREQHLTEYFEDLGIIVRDVFVPSAFNQNGQKRKHNRGFGFVEVGTVKEHQ  169
             F+ GIP     +H+TEYFE  G +   V           + K NRGF FV + +    +
Sbjct  116  FFIGGIPQYITSKHITEYFEQYGTVQHVVIAQD------HETKRNRGFAFVTMASHINKE  169

Query  170  QLIR  173
            +++R
Sbjct  170  RILR  173


>ref|XP_012986147.1| PREDICTED: DAZ-associated protein 1 [Melopsittacus undulatus]
Length=367

 Score = 41.2 bits (95),  Expect = 0.72, Method: Compositional matrix adjust.
 Identities = 28/97 (29%), Positives = 48/97 (49%), Gaps = 15/97 (15%)

Query  98   TDTNGSSRVDQTLFVAGIPNVAREQHLTEYFEDLGIIVRDVFVPSAFNQNGQKRKHNRGF  157
            +D N S+++    FV GIP+   E  L EYF+  G++   V +  A      +++  RGF
Sbjct  66   SDNNKSNKI----FVGGIPHNCGETELREYFKKFGVVTEVVMIYDA------EKQRPRGF  115

Query  158  GFVEVGTVKEHQQLIRMSKNKKIEFDLKPAKWDIERA  194
            GF+      E +Q +  + N     D+   K +++RA
Sbjct  116  GFITF----EDEQSVDQAVNMHFH-DIMGKKVEVKRA  147


>gb|KFU97824.1| DAZ-associated protein 1, partial [Pterocles gutturalis]
Length=398

 Score = 41.2 bits (95),  Expect = 0.72, Method: Compositional matrix adjust.
 Identities = 28/97 (29%), Positives = 48/97 (49%), Gaps = 15/97 (15%)

Query  98   TDTNGSSRVDQTLFVAGIPNVAREQHLTEYFEDLGIIVRDVFVPSAFNQNGQKRKHNRGF  157
            +D N S+++    FV GIP+   E  L EYF+  G++   V +  A      +++  RGF
Sbjct  97   SDNNKSNKI----FVGGIPHNCGETELREYFKKFGVVTEVVMIYDA------EKQRPRGF  146

Query  158  GFVEVGTVKEHQQLIRMSKNKKIEFDLKPAKWDIERA  194
            GF+      E +Q +  + N     D+   K +++RA
Sbjct  147  GFITF----EDEQSVDQAVNMHFH-DIMGKKVEVKRA  178


>ref|XP_006401670.1| hypothetical protein EUTSA_v10015559mg [Eutrema salsugineum]
 gb|ESQ43123.1| hypothetical protein EUTSA_v10015559mg [Eutrema salsugineum]
Length=141

 Score = 39.7 bits (91),  Expect = 0.73, Method: Compositional matrix adjust.
 Identities = 25/81 (31%), Positives = 39/81 (48%), Gaps = 9/81 (11%)

Query  107  DQTLFVAGIPNVAREQHLTEYFEDLGIIVRDVFVPSAFNQNGQKRKHNRGFGFVEVGTVK  166
            D  ++VAG+P + R + L  YFE  G I+    V        +  + +RGFGFV   T +
Sbjct  8    DTKIYVAGLPWITRTEGLRSYFEQFGEIINANVVCD------RATRRSRGFGFV---TFR  58

Query  167  EHQQLIRMSKNKKIEFDLKPA  187
            E +   R  +N   + D + A
Sbjct  59   EAESARRACENPNPKIDGRVA  79


>ref|XP_013055532.1| PREDICTED: DAZ-associated protein 1 isoform X2 [Anser cygnoides 
domesticus]
Length=408

 Score = 41.2 bits (95),  Expect = 0.73, Method: Compositional matrix adjust.
 Identities = 28/97 (29%), Positives = 48/97 (49%), Gaps = 15/97 (15%)

Query  98   TDTNGSSRVDQTLFVAGIPNVAREQHLTEYFEDLGIIVRDVFVPSAFNQNGQKRKHNRGF  157
            +D N S+++    FV GIP+   E  L EYF+  G++   V +  A      +++  RGF
Sbjct  107  SDNNKSNKI----FVGGIPHNCGETELREYFKKFGVVTEVVMIYDA------EKQRPRGF  156

Query  158  GFVEVGTVKEHQQLIRMSKNKKIEFDLKPAKWDIERA  194
            GF+      E +Q +  + N     D+   K +++RA
Sbjct  157  GFITF----EDEQSVDQAVNMHFH-DIMGKKVEVKRA  188


>ref|XP_009708903.1| PREDICTED: DAZ-associated protein 1 [Cariama cristata]
Length=409

 Score = 41.2 bits (95),  Expect = 0.73, Method: Compositional matrix adjust.
 Identities = 28/97 (29%), Positives = 48/97 (49%), Gaps = 15/97 (15%)

Query  98   TDTNGSSRVDQTLFVAGIPNVAREQHLTEYFEDLGIIVRDVFVPSAFNQNGQKRKHNRGF  157
            +D N S+++    FV GIP+   E  L EYF+  G++   V +  A      +++  RGF
Sbjct  108  SDNNKSNKI----FVGGIPHNCGETELREYFKKFGVVTEVVMIYDA------EKQRPRGF  157

Query  158  GFVEVGTVKEHQQLIRMSKNKKIEFDLKPAKWDIERA  194
            GF+      E +Q +  + N     D+   K +++RA
Sbjct  158  GFITF----EDEQSVDQAVNMHFH-DIMGKKVEVKRA  189


>ref|XP_013055531.1| PREDICTED: DAZ-associated protein 1 isoform X1 [Anser cygnoides 
domesticus]
Length=409

 Score = 41.2 bits (95),  Expect = 0.73, Method: Compositional matrix adjust.
 Identities = 28/97 (29%), Positives = 48/97 (49%), Gaps = 15/97 (15%)

Query  98   TDTNGSSRVDQTLFVAGIPNVAREQHLTEYFEDLGIIVRDVFVPSAFNQNGQKRKHNRGF  157
            +D N S+++    FV GIP+   E  L EYF+  G++   V +  A      +++  RGF
Sbjct  108  SDNNKSNKI----FVGGIPHNCGETELREYFKKFGVVTEVVMIYDA------EKQRPRGF  157

Query  158  GFVEVGTVKEHQQLIRMSKNKKIEFDLKPAKWDIERA  194
            GF+      E +Q +  + N     D+   K +++RA
Sbjct  158  GFITF----EDEQSVDQAVNMHFH-DIMGKKVEVKRA  189


>ref|XP_009500835.1| PREDICTED: DAZ-associated protein 1, partial [Phalacrocorax carbo]
 ref|XP_009815888.1| PREDICTED: DAZ-associated protein 1, partial [Gavia stellata]
 gb|KFO64740.1| DAZ-associated protein 1, partial [Corvus brachyrhynchos]
 8 more sequence titles

gb|KFO81609.1| DAZ-associated protein 1, partial [Cuculus canorus]
 gb|KFP62225.1| DAZ-associated protein 1, partial [Cariama cristata]
 gb|KFQ16351.1| DAZ-associated protein 1, partial [Leptosomus discolor]
 gb|KFQ86461.1| DAZ-associated protein 1, partial [Phoenicopterus ruber ruber]
 gb|KFU95450.1| DAZ-associated protein 1, partial [Chaetura pelagica]
 gb|KFV45099.1| DAZ-associated protein 1, partial [Gavia stellata]
 gb|KFW89875.1| DAZ-associated protein 1, partial [Phalacrocorax carbo]
 gb|KGL92869.1| DAZ-associated protein 1, partial [Charadrius vociferus]

Length=398

 Score = 41.2 bits (95),  Expect = 0.74, Method: Compositional matrix adjust.
 Identities = 28/97 (29%), Positives = 48/97 (49%), Gaps = 15/97 (15%)

Query  98   TDTNGSSRVDQTLFVAGIPNVAREQHLTEYFEDLGIIVRDVFVPSAFNQNGQKRKHNRGF  157
            +D N S+++    FV GIP+   E  L EYF+  G++   V +  A      +++  RGF
Sbjct  97   SDNNKSNKI----FVGGIPHNCGETELREYFKKFGVVTEVVMIYDA------EKQRPRGF  146

Query  158  GFVEVGTVKEHQQLIRMSKNKKIEFDLKPAKWDIERA  194
            GF+      E +Q +  + N     D+   K +++RA
Sbjct  147  GFITF----EDEQSVDQAVNMHFH-DIMGKKVEVKRA  178


>ref|XP_007382467.1| hypothetical protein PUNSTDRAFT_100868 [Punctularia strigosozonata 
HHB-11173 SS5]
 gb|EIN10955.1| hypothetical protein PUNSTDRAFT_100868 [Punctularia strigosozonata 
HHB-11173 SS5]
Length=466

 Score = 41.2 bits (95),  Expect = 0.74, Method: Compositional matrix adjust.
 Identities = 26/84 (31%), Positives = 41/84 (49%), Gaps = 5/84 (6%)

Query  97   DTDTNGSSRVDQTLFVAGIPNVAREQHLTEYFEDLGIIVRDVFVPSAFNQNGQK----RK  152
            D  T  S++   +++V  +     E  L  +F+ +G I R + +P     NGQ+    RK
Sbjct  97   DAKTESSAKRQHSVWVGNLSFKTTEDALKAFFDGVGDITR-IHLPLKPPVNGQRGPGVRK  155

Query  153  HNRGFGFVEVGTVKEHQQLIRMSK  176
             NRGF +V+  T    Q  I MS+
Sbjct  156  ENRGFAYVDFATAGAKQVAIAMSE  179


>ref|XP_002194831.1| PREDICTED: DAZ-associated protein 1 [Taeniopygia guttata]
Length=410

 Score = 41.2 bits (95),  Expect = 0.74, Method: Compositional matrix adjust.
 Identities = 28/97 (29%), Positives = 48/97 (49%), Gaps = 15/97 (15%)

Query  98   TDTNGSSRVDQTLFVAGIPNVAREQHLTEYFEDLGIIVRDVFVPSAFNQNGQKRKHNRGF  157
            +D N S+++    FV GIP+   E  L EYF+  G++   V +  A      +++  RGF
Sbjct  109  SDNNKSNKI----FVGGIPHNCGETELREYFKKFGVVTEVVMIYDA------EKQRPRGF  158

Query  158  GFVEVGTVKEHQQLIRMSKNKKIEFDLKPAKWDIERA  194
            GF+      E +Q +  + N     D+   K +++RA
Sbjct  159  GFITF----EDEQSVDQAVNMHFH-DIMGKKVEVKRA  190


>ref|XP_006267896.1| PREDICTED: DAZ-associated protein 1 isoform X2 [Alligator mississippiensis]
Length=348

 Score = 41.2 bits (95),  Expect = 0.74, Method: Compositional matrix adjust.
 Identities = 28/97 (29%), Positives = 48/97 (49%), Gaps = 15/97 (15%)

Query  98   TDTNGSSRVDQTLFVAGIPNVAREQHLTEYFEDLGIIVRDVFVPSAFNQNGQKRKHNRGF  157
            +D N S+++    FV GIP+   E  L EYF+  G++   V +  A      +++  RGF
Sbjct  108  SDNNKSNKI----FVGGIPHNCGETELREYFKKFGVVTEVVMIYDA------EKQRPRGF  157

Query  158  GFVEVGTVKEHQQLIRMSKNKKIEFDLKPAKWDIERA  194
            GF+      E +Q +  + N     D+   K +++RA
Sbjct  158  GFITF----EDEQSVDQAVNMHFH-DIMGKKVEVKRA  189


>gb|KFP05016.1| DAZ-associated protein 1, partial [Calypte anna]
Length=398

 Score = 41.2 bits (95),  Expect = 0.74, Method: Compositional matrix adjust.
 Identities = 28/97 (29%), Positives = 48/97 (49%), Gaps = 15/97 (15%)

Query  98   TDTNGSSRVDQTLFVAGIPNVAREQHLTEYFEDLGIIVRDVFVPSAFNQNGQKRKHNRGF  157
            +D N S+++    FV GIP+   E  L EYF+  G++   V +  A      +++  RGF
Sbjct  97   SDNNKSNKI----FVGGIPHNCGETELREYFKKFGVVTEVVMIYDA------EKQRPRGF  146

Query  158  GFVEVGTVKEHQQLIRMSKNKKIEFDLKPAKWDIERA  194
            GF+      E +Q +  + N     D+   K +++RA
Sbjct  147  GFITF----EDEQSVDQAVNMHFH-DIMGKKVEVKRA  178


>ref|XP_011373487.1| PREDICTED: DAZ-associated protein 1 isoform X3 [Pteropus vampyrus]
Length=456

 Score = 41.2 bits (95),  Expect = 0.74, Method: Compositional matrix adjust.
 Identities = 25/85 (29%), Positives = 42/85 (49%), Gaps = 11/85 (13%)

Query  110  LFVAGIPNVAREQHLTEYFEDLGIIVRDVFVPSAFNQNGQKRKHNRGFGFVEVGTVKEHQ  169
            +FV GIP+   E  L EYF+  G++   V +  A      +++  RGFGF+      E +
Sbjct  196  IFVGGIPHNCGETELREYFKKFGVVTEVVMIYDA------EKQRPRGFGFITF----EDE  245

Query  170  QLIRMSKNKKIEFDLKPAKWDIERA  194
            Q +  + N     D+   K +++RA
Sbjct  246  QSVDQAVNMHFH-DIMGKKVEVKRA  269


>gb|KKR04684.1| RNP-1 like protein RNA-binding protein [Parcubacteria bacterium 
GW2011_GWC2_39_14]
Length=85

 Score = 38.9 bits (89),  Expect = 0.75, Method: Compositional matrix adjust.
 Identities = 23/76 (30%), Positives = 34/76 (45%), Gaps = 6/76 (8%)

Query  106  VDQTLFVAGIPNVAREQHLTEYFEDLGIIVRDVFVPSAFNQNGQKRKHNRGFGFVEVGTV  165
            + Q L+V G+P    E  L E FE  G +     +    +        +RGFGFVE+ + 
Sbjct  1    MGQKLYVGGLPYTTTEGALKEAFEKAGSVASVKIITDKMS------GRSRGFGFVEMSSD  54

Query  166  KEHQQLIRMSKNKKIE  181
             E +  I M   K+ E
Sbjct  55   DEAEAAIDMWNGKEFE  70


>ref|XP_004955408.1| PREDICTED: RNA-binding protein 1-like [Setaria italica]
Length=382

 Score = 41.2 bits (95),  Expect = 0.78, Method: Compositional matrix adjust.
 Identities = 25/69 (36%), Positives = 35/69 (51%), Gaps = 16/69 (23%)

Query  98   TDTNGSSRVDQTLFVAGIPNVAREQHLTEYFEDLG-----IIVRDVFVPSAFNQNGQKRK  152
            T   G++   + LFV GIP+ A+E  L E+F   G     I++RD           ++  
Sbjct  13   TGEEGAAGESRKLFVGGIPSGAQEGELREHFARYGEVRSVIVMRD-----------RETG  61

Query  153  HNRGFGFVE  161
            H RGFGFVE
Sbjct  62   HGRGFGFVE  70


>ref|XP_007072131.1| PREDICTED: LOW QUALITY PROTEIN: DAZ-associated protein 1 [Chelonia 
mydas]
Length=339

 Score = 40.8 bits (94),  Expect = 0.78, Method: Compositional matrix adjust.
 Identities = 23/76 (30%), Positives = 36/76 (47%), Gaps = 10/76 (13%)

Query  110  LFVAGIPNVAREQHLTEYFEDLGIIVRDVFVPSAFNQNGQKRKHNRGFGFVEVGTVKEHQ  169
            +FV GIP+   E  L EYF+  G++   V +  A      +++  RGFGF+     +   
Sbjct  74   IFVGGIPHNCGETELREYFKKFGVVTEVVMIYDA------EKQRPRGFGFITFEDEQSVD  127

Query  170  QLIRMS----KNKKIE  181
            Q + M       KK+E
Sbjct  128  QAVNMHFHDIMGKKVE  143


>ref|NP_001026599.1| DAZ-associated protein 1 [Gallus gallus]
 emb|CAG31151.1| hypothetical protein RCJMB04_2n4 [Gallus gallus]
Length=409

 Score = 41.2 bits (95),  Expect = 0.79, Method: Compositional matrix adjust.
 Identities = 28/97 (29%), Positives = 48/97 (49%), Gaps = 15/97 (15%)

Query  98   TDTNGSSRVDQTLFVAGIPNVAREQHLTEYFEDLGIIVRDVFVPSAFNQNGQKRKHNRGF  157
            +D N S+++    FV GIP+   E  L EYF+  G++   V +  A      +++  RGF
Sbjct  108  SDNNKSNKI----FVGGIPHNCGETELREYFKKFGVVTEVVMIYDA------EKQRPRGF  157

Query  158  GFVEVGTVKEHQQLIRMSKNKKIEFDLKPAKWDIERA  194
            GF+      E +Q +  + N     D+   K +++RA
Sbjct  158  GFITF----EDEQSVDQAVNMHFH-DIMGKKVEVKRA  189


>ref|XP_008497449.1| PREDICTED: DAZ-associated protein 1 [Calypte anna]
Length=402

 Score = 41.2 bits (95),  Expect = 0.79, Method: Compositional matrix adjust.
 Identities = 28/97 (29%), Positives = 48/97 (49%), Gaps = 15/97 (15%)

Query  98   TDTNGSSRVDQTLFVAGIPNVAREQHLTEYFEDLGIIVRDVFVPSAFNQNGQKRKHNRGF  157
            +D N S+++    FV GIP+   E  L EYF+  G++   V +  A      +++  RGF
Sbjct  101  SDNNKSNKI----FVGGIPHNCGETELREYFKKFGVVTEVVMIYDA------EKQRPRGF  150

Query  158  GFVEVGTVKEHQQLIRMSKNKKIEFDLKPAKWDIERA  194
            GF+      E +Q +  + N     D+   K +++RA
Sbjct  151  GFITF----EDEQSVDQAVNMHFH-DIMGKKVEVKRA  182


>ref|XP_011526208.1| PREDICTED: DAZ-associated protein 1 isoform X3 [Homo sapiens]
Length=549

 Score = 41.2 bits (95),  Expect = 0.79, Method: Compositional matrix adjust.
 Identities = 23/76 (30%), Positives = 36/76 (47%), Gaps = 10/76 (13%)

Query  110  LFVAGIPNVAREQHLTEYFEDLGIIVRDVFVPSAFNQNGQKRKHNRGFGFVEVGTVKEHQ  169
            +FV GIP+   E  L EYF+  G++   V +  A      +++  RGFGF+     +   
Sbjct  257  IFVGGIPHNCGETELREYFKKFGVVTEVVMIYDA------EKQRPRGFGFITFEDEQSVD  310

Query  170  QLIRMS----KNKKIE  181
            Q + M       KK+E
Sbjct  311  QAVNMHFHDIMGKKVE  326


>gb|AAD49731.1|AF169290_1 hnRNP A2/B1 protein [Sus scrofa]
Length=84

 Score = 38.5 bits (88),  Expect = 0.79, Method: Compositional matrix adjust.
 Identities = 26/72 (36%), Positives = 36/72 (50%), Gaps = 6/72 (8%)

Query  89   PLKSTALLDTDTNGSSRVDQTLFVAGIPNVAREQHLTEYFEDLGIIVRDVFVPSAFNQNG  148
            P ++ A  ++   G+    + LFV GI     E HL +YFE+ G I  D        Q+G
Sbjct  7    PKRAVAREESGKPGAHVTVKKLFVGGIKEDTEEHHLRDYFEEYGKI--DTIEIITDRQSG  64

Query  149  QKRKHNRGFGFV  160
            +K    RGFGFV
Sbjct  65   KK----RGFGFV  72


>ref|XP_008509609.1| PREDICTED: DAZ-associated protein 1, partial [Equus przewalskii]
Length=459

 Score = 41.2 bits (95),  Expect = 0.81, Method: Compositional matrix adjust.
 Identities = 23/76 (30%), Positives = 36/76 (47%), Gaps = 10/76 (13%)

Query  110  LFVAGIPNVAREQHLTEYFEDLGIIVRDVFVPSAFNQNGQKRKHNRGFGFVEVGTVKEHQ  169
            +FV GIP+   E  L EYF+  G++   V +  A      +++  RGFGF+     +   
Sbjct  272  IFVGGIPHNCGETELREYFKKFGVVTEVVMIYDA------EKQRPRGFGFITFEDEQSVD  325

Query  170  QLIRMS----KNKKIE  181
            Q + M       KK+E
Sbjct  326  QAVNMHFHDIMGKKVE  341


>ref|XP_007489409.1| PREDICTED: DAZ-associated protein 1 isoform X1 [Monodelphis domestica]
Length=407

 Score = 41.2 bits (95),  Expect = 0.81, Method: Compositional matrix adjust.
 Identities = 28/97 (29%), Positives = 48/97 (49%), Gaps = 15/97 (15%)

Query  98   TDTNGSSRVDQTLFVAGIPNVAREQHLTEYFEDLGIIVRDVFVPSAFNQNGQKRKHNRGF  157
            +D N S+++    FV GIP+   E  L EYF+  G++   V +  A      +++  RGF
Sbjct  107  SDNNKSNKI----FVGGIPHNCGETELREYFKKFGVVTEVVMIYDA------EKQRPRGF  156

Query  158  GFVEVGTVKEHQQLIRMSKNKKIEFDLKPAKWDIERA  194
            GF+      E +Q +  + N     D+   K +++RA
Sbjct  157  GFITF----EDEQSVDQAVNMHFH-DIMGKKVEVKRA  188


>ref|XP_005531237.1| PREDICTED: DAZ-associated protein 1 [Pseudopodoces humilis]
Length=409

 Score = 41.2 bits (95),  Expect = 0.82, Method: Compositional matrix adjust.
 Identities = 28/97 (29%), Positives = 48/97 (49%), Gaps = 15/97 (15%)

Query  98   TDTNGSSRVDQTLFVAGIPNVAREQHLTEYFEDLGIIVRDVFVPSAFNQNGQKRKHNRGF  157
            +D N S+++    FV GIP+   E  L EYF+  G++   V +  A      +++  RGF
Sbjct  108  SDNNKSNKI----FVGGIPHNCGETELREYFKKFGVVTEVVMIYDA------EKQRPRGF  157

Query  158  GFVEVGTVKEHQQLIRMSKNKKIEFDLKPAKWDIERA  194
            GF+      E +Q +  + N     D+   K +++RA
Sbjct  158  GFITF----EDEQSVDQAVNMHFH-DIMGKKVEVKRA  189


>ref|XP_011526209.1| PREDICTED: DAZ-associated protein 1 isoform X4 [Homo sapiens]
Length=548

 Score = 41.2 bits (95),  Expect = 0.82, Method: Compositional matrix adjust.
 Identities = 23/76 (30%), Positives = 36/76 (47%), Gaps = 10/76 (13%)

Query  110  LFVAGIPNVAREQHLTEYFEDLGIIVRDVFVPSAFNQNGQKRKHNRGFGFVEVGTVKEHQ  169
            +FV GIP+   E  L EYF+  G++   V +  A      +++  RGFGF+     +   
Sbjct  257  IFVGGIPHNCGETELREYFKKFGVVTEVVMIYDA------EKQRPRGFGFITFEDEQSVD  310

Query  170  QLIRMS----KNKKIE  181
            Q + M       KK+E
Sbjct  311  QAVNMHFHDIMGKKVE  326


>ref|XP_007489410.1| PREDICTED: DAZ-associated protein 1 isoform X2 [Monodelphis domestica]
Length=406

 Score = 41.2 bits (95),  Expect = 0.82, Method: Compositional matrix adjust.
 Identities = 28/97 (29%), Positives = 48/97 (49%), Gaps = 15/97 (15%)

Query  98   TDTNGSSRVDQTLFVAGIPNVAREQHLTEYFEDLGIIVRDVFVPSAFNQNGQKRKHNRGF  157
            +D N S+++    FV GIP+   E  L EYF+  G++   V +  A      +++  RGF
Sbjct  106  SDNNKSNKI----FVGGIPHNCGETELREYFKKFGVVTEVVMIYDA------EKQRPRGF  155

Query  158  GFVEVGTVKEHQQLIRMSKNKKIEFDLKPAKWDIERA  194
            GF+      E +Q +  + N     D+   K +++RA
Sbjct  156  GFITF----EDEQSVDQAVNMHFH-DIMGKKVEVKRA  187


>ref|XP_005497184.1| PREDICTED: DAZ-associated protein 1 [Zonotrichia albicollis]
Length=411

 Score = 41.2 bits (95),  Expect = 0.82, Method: Compositional matrix adjust.
 Identities = 26/88 (30%), Positives = 42/88 (48%), Gaps = 14/88 (16%)

Query  98   TDTNGSSRVDQTLFVAGIPNVAREQHLTEYFEDLGIIVRDVFVPSAFNQNGQKRKHNRGF  157
            +D N S+++    FV GIP+   E  L EYF+  G++   V +  A      +++  RGF
Sbjct  149  SDNNKSNKI----FVGGIPHNCGETELREYFKKFGVVTEVVMIYDA------EKQRPRGF  198

Query  158  GFVEVGTVKEHQQLIRMS----KNKKIE  181
            GF+     +   Q + M       KK+E
Sbjct  199  GFITFEDEQSVDQAVNMHFHDIMGKKVE  226


>ref|XP_005983429.1| PREDICTED: heterogeneous nuclear ribonucleoproteins A2/B1-like 
[Pantholops hodgsonii]
Length=347

 Score = 40.8 bits (94),  Expect = 0.82, Method: Compositional matrix adjust.
 Identities = 25/51 (49%), Positives = 27/51 (53%), Gaps = 6/51 (12%)

Query  110  LFVAGIPNVAREQHLTEYFEDLGIIVRDVFVPSAFNQNGQKRKHNRGFGFV  160
            LFV GI    RE HL EYFE  G I  D        Q+G+K    RGFGFV
Sbjct  122  LFVGGIKEDTREHHLREYFEKYGKI--DAIEIITDRQSGKK----RGFGFV  166


>ref|XP_011373485.1| PREDICTED: DAZ-associated protein 1 isoform X1 [Pteropus vampyrus]
Length=487

 Score = 41.2 bits (95),  Expect = 0.83, Method: Compositional matrix adjust.
 Identities = 25/85 (29%), Positives = 42/85 (49%), Gaps = 11/85 (13%)

Query  110  LFVAGIPNVAREQHLTEYFEDLGIIVRDVFVPSAFNQNGQKRKHNRGFGFVEVGTVKEHQ  169
            +FV GIP+   E  L EYF+  G++   V +  A      +++  RGFGF+      E +
Sbjct  196  IFVGGIPHNCGETELREYFKKFGVVTEVVMIYDA------EKQRPRGFGFITF----EDE  245

Query  170  QLIRMSKNKKIEFDLKPAKWDIERA  194
            Q +  + N     D+   K +++RA
Sbjct  246  QSVDQAVNMHFH-DIMGKKVEVKRA  269


>ref|XP_011526206.1| PREDICTED: DAZ-associated protein 1 isoform X1 [Homo sapiens]
Length=550

 Score = 41.2 bits (95),  Expect = 0.83, Method: Compositional matrix adjust.
 Identities = 23/76 (30%), Positives = 36/76 (47%), Gaps = 10/76 (13%)

Query  110  LFVAGIPNVAREQHLTEYFEDLGIIVRDVFVPSAFNQNGQKRKHNRGFGFVEVGTVKEHQ  169
            +FV GIP+   E  L EYF+  G++   V +  A      +++  RGFGF+     +   
Sbjct  258  IFVGGIPHNCGETELREYFKKFGVVTEVVMIYDA------EKQRPRGFGFITFEDEQSVD  311

Query  170  QLIRMS----KNKKIE  181
            Q + M       KK+E
Sbjct  312  QAVNMHFHDIMGKKVE  327


>ref|XP_007489412.1| PREDICTED: DAZ-associated protein 1 isoform X4 [Monodelphis domestica]
Length=405

 Score = 41.2 bits (95),  Expect = 0.83, Method: Compositional matrix adjust.
 Identities = 28/97 (29%), Positives = 48/97 (49%), Gaps = 15/97 (15%)

Query  98   TDTNGSSRVDQTLFVAGIPNVAREQHLTEYFEDLGIIVRDVFVPSAFNQNGQKRKHNRGF  157
            +D N S+++    FV GIP+   E  L EYF+  G++   V +  A      +++  RGF
Sbjct  106  SDNNKSNKI----FVGGIPHNCGETELREYFKKFGVVTEVVMIYDA------EKQRPRGF  155

Query  158  GFVEVGTVKEHQQLIRMSKNKKIEFDLKPAKWDIERA  194
            GF+      E +Q +  + N     D+   K +++RA
Sbjct  156  GFITF----EDEQSVDQAVNMHFH-DIMGKKVEVKRA  187


>ref|XP_007489411.1| PREDICTED: DAZ-associated protein 1 isoform X3 [Monodelphis domestica]
Length=406

 Score = 41.2 bits (95),  Expect = 0.84, Method: Compositional matrix adjust.
 Identities = 28/97 (29%), Positives = 48/97 (49%), Gaps = 15/97 (15%)

Query  98   TDTNGSSRVDQTLFVAGIPNVAREQHLTEYFEDLGIIVRDVFVPSAFNQNGQKRKHNRGF  157
            +D N S+++    FV GIP+   E  L EYF+  G++   V +  A      +++  RGF
Sbjct  107  SDNNKSNKI----FVGGIPHNCGETELREYFKKFGVVTEVVMIYDA------EKQRPRGF  156

Query  158  GFVEVGTVKEHQQLIRMSKNKKIEFDLKPAKWDIERA  194
            GF+      E +Q +  + N     D+   K +++RA
Sbjct  157  GFITF----EDEQSVDQAVNMHFH-DIMGKKVEVKRA  188


>ref|XP_013260251.1| hypothetical protein A1O9_05579 [Exophiala aquamarina CBS 119918]
 gb|KEF57661.1| hypothetical protein A1O9_05579 [Exophiala aquamarina CBS 119918]
Length=486

 Score = 41.2 bits (95),  Expect = 0.84, Method: Compositional matrix adjust.
 Identities = 36/119 (30%), Positives = 53/119 (45%), Gaps = 21/119 (18%)

Query  104  SRVDQ-TLFVAGIPNVAREQHLTEYFEDLGII-----VRDVFVPSAFNQNGQKRKHNRGF  157
            S VDQ ++FV  +P     Q L   FED G I     +R VF   A N           F
Sbjct  268  SAVDQKSIFVGNLPEGTTRQDLHTLFEDFGTIIQVNVIRKVFSDDAVNN----------F  317

Query  158  GFVEVGTVKEHQQLIRMSKN-KKIEFDLKPAKWDIERA-RSYGTPSPSIAPKIESKPKD  214
            GFVE  TV+E +    + +  K ++  ++P ++   R  R+   P+    P   S P+D
Sbjct  318  GFVEFSTVQEAEHASNIERTFKGVKLRVEPKEYSARRGPRTTYIPA---TPAHTSTPRD  373


>ref|XP_011526207.1| PREDICTED: DAZ-associated protein 1 isoform X2 [Homo sapiens]
Length=549

 Score = 41.2 bits (95),  Expect = 0.84, Method: Compositional matrix adjust.
 Identities = 23/76 (30%), Positives = 36/76 (47%), Gaps = 10/76 (13%)

Query  110  LFVAGIPNVAREQHLTEYFEDLGIIVRDVFVPSAFNQNGQKRKHNRGFGFVEVGTVKEHQ  169
            +FV GIP+   E  L EYF+  G++   V +  A      +++  RGFGF+     +   
Sbjct  258  IFVGGIPHNCGETELREYFKKFGVVTEVVMIYDA------EKQRPRGFGFITFEDEQSVD  311

Query  170  QLIRMS----KNKKIE  181
            Q + M       KK+E
Sbjct  312  QAVNMHFHDIMGKKVE  327


>ref|XP_007075779.1| PREDICTED: DAZ-associated protein 1 [Panthera tigris altaica]
Length=414

 Score = 41.2 bits (95),  Expect = 0.84, Method: Compositional matrix adjust.
 Identities = 25/85 (29%), Positives = 42/85 (49%), Gaps = 11/85 (13%)

Query  110  LFVAGIPNVAREQHLTEYFEDLGIIVRDVFVPSAFNQNGQKRKHNRGFGFVEVGTVKEHQ  169
            +FV GIP+   E  L EYF+  G++   V +  A      +++  RGFGF+      E +
Sbjct  154  IFVGGIPHNCGETELREYFKKFGVVTEVVMIYDA------EKQRPRGFGFITF----EDE  203

Query  170  QLIRMSKNKKIEFDLKPAKWDIERA  194
            Q +  + N     D+   K +++RA
Sbjct  204  QSVDQAVNMHFH-DIMGKKVEVKRA  227


>ref|XP_011373486.1| PREDICTED: DAZ-associated protein 1 isoform X2 [Pteropus vampyrus]
Length=486

 Score = 41.2 bits (95),  Expect = 0.85, Method: Compositional matrix adjust.
 Identities = 25/85 (29%), Positives = 42/85 (49%), Gaps = 11/85 (13%)

Query  110  LFVAGIPNVAREQHLTEYFEDLGIIVRDVFVPSAFNQNGQKRKHNRGFGFVEVGTVKEHQ  169
            +FV GIP+   E  L EYF+  G++   V +  A      +++  RGFGF+      E +
Sbjct  195  IFVGGIPHNCGETELREYFKKFGVVTEVVMIYDA------EKQRPRGFGFITF----EDE  244

Query  170  QLIRMSKNKKIEFDLKPAKWDIERA  194
            Q +  + N     D+   K +++RA
Sbjct  245  QSVDQAVNMHFH-DIMGKKVEVKRA  268


>ref|XP_008709425.1| PREDICTED: LOW QUALITY PROTEIN: DAZ-associated protein 1 [Ursus 
maritimus]
Length=384

 Score = 40.8 bits (94),  Expect = 0.85, Method: Compositional matrix adjust.
 Identities = 25/85 (29%), Positives = 42/85 (49%), Gaps = 11/85 (13%)

Query  110  LFVAGIPNVAREQHLTEYFEDLGIIVRDVFVPSAFNQNGQKRKHNRGFGFVEVGTVKEHQ  169
            +FV GIP+   E  L EYF+  G++   V +  A      +++  RGFGF+      E +
Sbjct  128  IFVGGIPHNCGETELREYFKKFGVVTEVVMIYDA------EKQRPRGFGFITF----EDE  177

Query  170  QLIRMSKNKKIEFDLKPAKWDIERA  194
            Q +  + N     D+   K +++RA
Sbjct  178  QSVDQAVNMHFH-DIMGKKVEVKRA  201


>ref|XP_004695951.2| PREDICTED: DAZ-associated protein 1 isoform X3 [Condylura cristata]
Length=334

 Score = 40.8 bits (94),  Expect = 0.86, Method: Compositional matrix adjust.
 Identities = 23/76 (30%), Positives = 36/76 (47%), Gaps = 10/76 (13%)

Query  110  LFVAGIPNVAREQHLTEYFEDLGIIVRDVFVPSAFNQNGQKRKHNRGFGFVEVGTVKEHQ  169
            +FV GIP+   E  L EYF+  G++   V +  A      +++  RGFGF+     +   
Sbjct  74   IFVGGIPHNCGETELREYFKKFGVVTEVVMIYDA------EKQRPRGFGFITFEDEQSVD  127

Query  170  QLIRMS----KNKKIE  181
            Q + M       KK+E
Sbjct  128  QAVNMHFHDIMGKKVE  143


>ref|XP_005866756.1| PREDICTED: LOW QUALITY PROTEIN: DAZ associated protein 1 [Myotis 
brandtii]
Length=413

 Score = 40.8 bits (94),  Expect = 0.86, Method: Compositional matrix adjust.
 Identities = 25/85 (29%), Positives = 42/85 (49%), Gaps = 11/85 (13%)

Query  110  LFVAGIPNVAREQHLTEYFEDLGIIVRDVFVPSAFNQNGQKRKHNRGFGFVEVGTVKEHQ  169
            +FV GIP+   E  L EYF+  G++   V +  A      +++  RGFGF+      E +
Sbjct  150  IFVGGIPHNCGETELREYFKKFGVVTEVVMIYDA------EKQRPRGFGFITF----EDE  199

Query  170  QLIRMSKNKKIEFDLKPAKWDIERA  194
            Q +  + N     D+   K +++RA
Sbjct  200  QSVDQAVNMHFH-DIMGKKVEVKRA  223


>gb|KKS23261.1| RNP-1 like protein RNA-binding protein [Parcubacteria (Nomurabacteria) 
bacterium GW2011_GWC2_41_8]
Length=106

 Score = 38.9 bits (89),  Expect = 0.86, Method: Compositional matrix adjust.
 Identities = 21/72 (29%), Positives = 35/72 (49%), Gaps = 6/72 (8%)

Query  110  LFVAGIPNVAREQHLTEYFEDLGIIVRDVFVPSAFNQNGQKRKHNRGFGFVEVGTVKEHQ  169
            L+V G+P   +E  L E F   G +   V +    +        ++GFGFVE+ +  E Q
Sbjct  5    LYVGGLPYSTQEDALKELFAQAGSVTSAVIIMDKMS------GRSKGFGFVEMSSQDEAQ  58

Query  170  QLIRMSKNKKIE  181
            + I M  +++ E
Sbjct  59   KAISMFNDQEFE  70


>ref|XP_005682549.2| PREDICTED: uncharacterized protein LOC102176222 [Capra hircus]
Length=788

 Score = 41.2 bits (95),  Expect = 0.87, Method: Compositional matrix adjust.
 Identities = 27/72 (38%), Positives = 36/72 (50%), Gaps = 6/72 (8%)

Query  89   PLKSTALLDTDTNGSSRVDQTLFVAGIPNVAREQHLTEYFEDLGIIVRDVFVPSAFNQNG  148
            P ++ A   ++  GS    + LFV G+    +E HL EYFE  G I  D        Q+G
Sbjct  101  PKRAVAREKSEKQGSLVTVKKLFVGGLKEDTKEHHLREYFEKYGKI--DAIEIITDRQSG  158

Query  149  QKRKHNRGFGFV  160
            +K    RGFGFV
Sbjct  159  KK----RGFGFV  166


>ref|XP_013216845.1| PREDICTED: LOW QUALITY PROTEIN: DAZ-associated protein 1 [Ictidomys 
tridecemlineatus]
Length=451

 Score = 41.2 bits (95),  Expect = 0.87, Method: Compositional matrix adjust.
 Identities = 25/85 (29%), Positives = 42/85 (49%), Gaps = 11/85 (13%)

Query  110  LFVAGIPNVAREQHLTEYFEDLGIIVRDVFVPSAFNQNGQKRKHNRGFGFVEVGTVKEHQ  169
            +FV GIP+   E  L EYF+  G++   V +  A      +++  RGFGF+      E +
Sbjct  164  IFVGGIPHNCGETELREYFKKFGVVTEVVMIYDA------EKQRPRGFGFITF----EDE  213

Query  170  QLIRMSKNKKIEFDLKPAKWDIERA  194
            Q +  + N     D+   K +++RA
Sbjct  214  QSVDQAVNMHFH-DIMGKKVEVKRA  237


>ref|XP_006125020.1| PREDICTED: DAZ-associated protein 1 isoform X2 [Pelodiscus sinensis]
Length=387

 Score = 40.8 bits (94),  Expect = 0.89, Method: Compositional matrix adjust.
 Identities = 25/85 (29%), Positives = 42/85 (49%), Gaps = 11/85 (13%)

Query  110  LFVAGIPNVAREQHLTEYFEDLGIIVRDVFVPSAFNQNGQKRKHNRGFGFVEVGTVKEHQ  169
            +FV GIP+   E  L EYF+  G++   V +  A      +++  RGFGF+      E +
Sbjct  73   IFVGGIPHNCGETELREYFKKFGVVTEVVMIYDA------EKQRPRGFGFITF----EDE  122

Query  170  QLIRMSKNKKIEFDLKPAKWDIERA  194
            Q +  + N     D+   K +++RA
Sbjct  123  QSVDQAVNMHFH-DIMGKKVEVKRA  146


>gb|ELV09335.1| DAZ-associated protein 1, partial [Tupaia chinensis]
Length=391

 Score = 40.8 bits (94),  Expect = 0.89, Method: Compositional matrix adjust.
 Identities = 25/85 (29%), Positives = 42/85 (49%), Gaps = 11/85 (13%)

Query  110  LFVAGIPNVAREQHLTEYFEDLGIIVRDVFVPSAFNQNGQKRKHNRGFGFVEVGTVKEHQ  169
            +FV GIP+   E  L EYF+  G++   V +  A      +++  RGFGF+      E +
Sbjct  83   IFVGGIPHNCGETELREYFKKFGVVTEVVMIYDA------EKQRPRGFGFITF----EDE  132

Query  170  QLIRMSKNKKIEFDLKPAKWDIERA  194
            Q +  + N     D+   K +++RA
Sbjct  133  QSVDQAVNMHFH-DIMGKKVEVKRA  156


>ref|XP_003230223.2| PREDICTED: DAZ-associated protein 1, partial [Anolis carolinensis]
Length=231

 Score = 40.4 bits (93),  Expect = 0.90, Method: Compositional matrix adjust.
 Identities = 25/85 (29%), Positives = 42/85 (49%), Gaps = 11/85 (13%)

Query  110  LFVAGIPNVAREQHLTEYFEDLGIIVRDVFVPSAFNQNGQKRKHNRGFGFVEVGTVKEHQ  169
            +FV GIP+   E  L EYF+  G++   V +  A      +++  RGFGF+      E +
Sbjct  114  IFVGGIPHNCGETELREYFKKFGVVTEVVMIYDA------EKQRPRGFGFITF----EDE  163

Query  170  QLIRMSKNKKIEFDLKPAKWDIERA  194
            Q +  + N     D+   K +++RA
Sbjct  164  QSVDQAVNMHFH-DIMGKKVEVKRA  187


>ref|XP_006125019.1| PREDICTED: DAZ-associated protein 1 isoform X1 [Pelodiscus sinensis]
Length=388

 Score = 40.8 bits (94),  Expect = 0.90, Method: Compositional matrix adjust.
 Identities = 25/85 (29%), Positives = 42/85 (49%), Gaps = 11/85 (13%)

Query  110  LFVAGIPNVAREQHLTEYFEDLGIIVRDVFVPSAFNQNGQKRKHNRGFGFVEVGTVKEHQ  169
            +FV GIP+   E  L EYF+  G++   V +  A      +++  RGFGF+      E +
Sbjct  74   IFVGGIPHNCGETELREYFKKFGVVTEVVMIYDA------EKQRPRGFGFITF----EDE  123

Query  170  QLIRMSKNKKIEFDLKPAKWDIERA  194
            Q +  + N     D+   K +++RA
Sbjct  124  QSVDQAVNMHFH-DIMGKKVEVKRA  147


>ref|XP_008161267.1| PREDICTED: DAZ-associated protein 1 isoform X4 [Chrysemys picta 
bellii]
Length=377

 Score = 40.8 bits (94),  Expect = 0.91, Method: Compositional matrix adjust.
 Identities = 25/86 (29%), Positives = 42/86 (49%), Gaps = 11/86 (13%)

Query  110  LFVAGIPNVAREQHLTEYFEDLGIIVRDVFVPSAFNQNGQKRKHNRGFGFVEVGTVKEHQ  169
            +FV GIP+   E  L EYF+  G++   V +  A      +++  RGFGF+      E +
Sbjct  115  IFVGGIPHNCGETELREYFKKFGVVTEVVMIYDA------EKQRPRGFGFITF----EDE  164

Query  170  QLIRMSKNKKIEFDLKPAKWDIERAR  195
            Q +  + N     D+   K +++RA 
Sbjct  165  QSVDQAVNMHFH-DIMGKKVEVKRAE  189


>ref|XP_012585271.1| PREDICTED: DAZ-associated protein 1 isoform X2 [Condylura cristata]
Length=346

 Score = 40.8 bits (94),  Expect = 0.92, Method: Compositional matrix adjust.
 Identities = 23/76 (30%), Positives = 36/76 (47%), Gaps = 10/76 (13%)

Query  110  LFVAGIPNVAREQHLTEYFEDLGIIVRDVFVPSAFNQNGQKRKHNRGFGFVEVGTVKEHQ  169
            +FV GIP+   E  L EYF+  G++   V +  A      +++  RGFGF+     +   
Sbjct  73   IFVGGIPHNCGETELREYFKKFGVVTEVVMIYDA------EKQRPRGFGFITFEDEQSVD  126

Query  170  QLIRMS----KNKKIE  181
            Q + M       KK+E
Sbjct  127  QAVNMHFHDIMGKKVE  142


>ref|XP_007614942.1| PREDICTED: LOW QUALITY PROTEIN: DAZ-associated protein 1 isoform 
X2 [Cricetulus griseus]
Length=413

 Score = 40.8 bits (94),  Expect = 0.92, Method: Compositional matrix adjust.
 Identities = 31/112 (28%), Positives = 51/112 (46%), Gaps = 13/112 (12%)

Query  110  LFVAGIPNVAREQHLTEYFEDLGIIVRDVFVPSAFNQNGQKRKHNRGFGFVEVGTVKEHQ  169
            +FV GIP+   E  L EYF+  G++   V +  A      +++  RGFGF+      E +
Sbjct  115  IFVGGIPHNCGETELREYFKKFGVVTEVVMIYDA------EKQRPRGFGFITF----EDE  164

Query  170  QLIRMSKNKKIEFDLKPAKWDIERA--RSYGTPSPSIAPKIESKPKDSKARP  219
            Q +  + N     D+   K +++RA  R     +P  A   +  P  S+  P
Sbjct  165  QSVDQAVNMHFH-DIMGKKVEVKRAEPRDSKNQAPGQAGASQGGPGGSRVAP  215


>ref|XP_012585270.1| PREDICTED: DAZ-associated protein 1 isoform X1 [Condylura cristata]
Length=347

 Score = 40.8 bits (94),  Expect = 0.93, Method: Compositional matrix adjust.
 Identities = 23/76 (30%), Positives = 36/76 (47%), Gaps = 10/76 (13%)

Query  110  LFVAGIPNVAREQHLTEYFEDLGIIVRDVFVPSAFNQNGQKRKHNRGFGFVEVGTVKEHQ  169
            +FV GIP+   E  L EYF+  G++   V +  A      +++  RGFGF+     +   
Sbjct  74   IFVGGIPHNCGETELREYFKKFGVVTEVVMIYDA------EKQRPRGFGFITFEDEQSVD  127

Query  170  QLIRMS----KNKKIE  181
            Q + M       KK+E
Sbjct  128  QAVNMHFHDIMGKKVE  143


>ref|XP_013918739.1| PREDICTED: DAZ-associated protein 1 isoform X2 [Thamnophis sirtalis]
Length=376

 Score = 40.8 bits (94),  Expect = 0.94, Method: Compositional matrix adjust.
 Identities = 25/86 (29%), Positives = 42/86 (49%), Gaps = 11/86 (13%)

Query  110  LFVAGIPNVAREQHLTEYFEDLGIIVRDVFVPSAFNQNGQKRKHNRGFGFVEVGTVKEHQ  169
            +FV GIP+   E  L EYF+  G++   V +  A      +++  RGFGF+      E +
Sbjct  114  IFVGGIPHNCGETELREYFKKFGVVTEVVMIYDA------EKQRPRGFGFITF----EDE  163

Query  170  QLIRMSKNKKIEFDLKPAKWDIERAR  195
            Q +  + N     D+   K +++RA 
Sbjct  164  QSVDQAVNMHFH-DIMGKKVEVKRAE  188


>ref|XP_008161260.1| PREDICTED: DAZ-associated protein 1 isoform X3 [Chrysemys picta 
bellii]
 ref|XP_008161261.1| PREDICTED: DAZ-associated protein 1 isoform X3 [Chrysemys picta 
bellii]
Length=378

 Score = 40.8 bits (94),  Expect = 0.95, Method: Compositional matrix adjust.
 Identities = 25/86 (29%), Positives = 42/86 (49%), Gaps = 11/86 (13%)

Query  110  LFVAGIPNVAREQHLTEYFEDLGIIVRDVFVPSAFNQNGQKRKHNRGFGFVEVGTVKEHQ  169
            +FV GIP+   E  L EYF+  G++   V +  A      +++  RGFGF+      E +
Sbjct  116  IFVGGIPHNCGETELREYFKKFGVVTEVVMIYDA------EKQRPRGFGFITF----EDE  165

Query  170  QLIRMSKNKKIEFDLKPAKWDIERAR  195
            Q +  + N     D+   K +++RA 
Sbjct  166  QSVDQAVNMHFH-DIMGKKVEVKRAE  190


>ref|XP_010373256.1| PREDICTED: DAZ-associated protein 1 [Rhinopithecus roxellana]
Length=520

 Score = 40.8 bits (94),  Expect = 0.96, Method: Compositional matrix adjust.
 Identities = 23/76 (30%), Positives = 36/76 (47%), Gaps = 10/76 (13%)

Query  110  LFVAGIPNVAREQHLTEYFEDLGIIVRDVFVPSAFNQNGQKRKHNRGFGFVEVGTVKEHQ  169
            +FV GIP+   E  L EYF+  G++   V +  A      +++  RGFGF+     +   
Sbjct  229  IFVGGIPHNCGETELREYFKKFGVVTEVVMIYDA------EKQRPRGFGFITFEDEQSVD  282

Query  170  QLIRMS----KNKKIE  181
            Q + M       KK+E
Sbjct  283  QAVNMHFHDIMGKKVE  298


>ref|XP_007431853.1| PREDICTED: DAZ-associated protein 1 [Python bivittatus]
Length=407

 Score = 40.8 bits (94),  Expect = 0.96, Method: Compositional matrix adjust.
 Identities = 25/85 (29%), Positives = 42/85 (49%), Gaps = 11/85 (13%)

Query  110  LFVAGIPNVAREQHLTEYFEDLGIIVRDVFVPSAFNQNGQKRKHNRGFGFVEVGTVKEHQ  169
            +FV GIP+   E  L EYF+  G++   V +  A      +++  RGFGF+      E +
Sbjct  114  IFVGGIPHNCGETELREYFKKFGVVTEVVMIYDA------EKQRPRGFGFITF----EDE  163

Query  170  QLIRMSKNKKIEFDLKPAKWDIERA  194
            Q +  + N     D+   K +++RA
Sbjct  164  QSVDQAVNMHFH-DIMGKKVEVKRA  187


>ref|XP_009014475.1| hypothetical protein HELRODRAFT_76754 [Helobdella robusta]
 gb|ESO07097.1| hypothetical protein HELRODRAFT_76754 [Helobdella robusta]
Length=333

 Score = 40.8 bits (94),  Expect = 0.96, Method: Compositional matrix adjust.
 Identities = 32/107 (30%), Positives = 51/107 (48%), Gaps = 16/107 (15%)

Query  110  LFVAGIPNVAREQHLTEYFEDLGIIV---RDVFVPSAFNQNGQKRKHNRGFGFVEVGTVK  166
            LF+ G+P   R++H  E  E++  +V   RDV V      + + ++ NRGF FVE     
Sbjct  142  LFLGGLP---RDKHRPEILENISKLVPGVRDVIV----KPDARDKQKNRGFAFVEF----  190

Query  167  EHQQLIRMSKNKKIEFDLKPAKWDIERARSYGTPSPSIAPKIESKPK  213
            E   L  M++ K +   ++   WD   A  +  P P +  +I  K K
Sbjct  191  ETHHLAAMARRKLVNKGIR--MWDQVIAVDWAEPEPEVDREIMEKVK  235


>ref|XP_010307268.1| PREDICTED: DAZ-associated protein 1, partial [Balearica regulorum 
gibbericeps]
 gb|KFO14092.1| DAZ-associated protein 1, partial [Balearica regulorum gibbericeps]
Length=398

 Score = 40.8 bits (94),  Expect = 0.97, Method: Compositional matrix adjust.
 Identities = 28/96 (29%), Positives = 47/96 (49%), Gaps = 15/96 (16%)

Query  99   DTNGSSRVDQTLFVAGIPNVAREQHLTEYFEDLGIIVRDVFVPSAFNQNGQKRKHNRGFG  158
            D N S+++    FV GIP+   E  L EYF+  G++   V +  A      +++  RGFG
Sbjct  98   DNNKSNKI----FVGGIPHNCGETELREYFKKFGVVTEVVMIYDA------EKQRPRGFG  147

Query  159  FVEVGTVKEHQQLIRMSKNKKIEFDLKPAKWDIERA  194
            F+      E +Q +  + N     D+   K +++RA
Sbjct  148  FITF----EDEQSVDQAVNMHFH-DIMGKKVEVKRA  178


>ref|XP_007176686.1| PREDICTED: DAZ-associated protein 1 [Balaenoptera acutorostrata 
scammoni]
Length=337

 Score = 40.8 bits (94),  Expect = 0.98, Method: Compositional matrix adjust.
 Identities = 23/76 (30%), Positives = 36/76 (47%), Gaps = 10/76 (13%)

Query  110  LFVAGIPNVAREQHLTEYFEDLGIIVRDVFVPSAFNQNGQKRKHNRGFGFVEVGTVKEHQ  169
            +FV GIP+   E  L EYF+  G++   V +  A      +++  RGFGF+     +   
Sbjct  74   IFVGGIPHNCGETELREYFKKFGVVTEVVMIYDA------EKQRPRGFGFITFEDEQSVD  127

Query  170  QLIRMS----KNKKIE  181
            Q + M       KK+E
Sbjct  128  QAVNMHFHDIMGKKVE  143


>ref|XP_008964813.1| PREDICTED: LOW QUALITY PROTEIN: DAZ-associated protein 1 [Pan 
paniscus]
Length=481

 Score = 40.8 bits (94),  Expect = 0.99, Method: Compositional matrix adjust.
 Identities = 23/76 (30%), Positives = 36/76 (47%), Gaps = 10/76 (13%)

Query  110  LFVAGIPNVAREQHLTEYFEDLGIIVRDVFVPSAFNQNGQKRKHNRGFGFVEVGTVKEHQ  169
            +FV GIP+   E  L EYF+  G++   V +  A      +++  RGFGF+     +   
Sbjct  189  IFVGGIPHNCGETELREYFKKFGVVTEVVMIYDA------EKQRPRGFGFITFEDEQSVD  242

Query  170  QLIRMS----KNKKIE  181
            Q + M       KK+E
Sbjct  243  QAVNMHFHDIMGKKVE  258


>gb|ACO11942.1| RNA-binding protein squid [Lepeophtheirus salmonis]
Length=268

 Score = 40.4 bits (93),  Expect = 1.0, Method: Compositional matrix adjust.
 Identities = 27/80 (34%), Positives = 36/80 (45%), Gaps = 12/80 (15%)

Query  103  SSRVDQTLFVAGIPNVAREQHLTEYFEDLGIIVRDVFVPSAFNQNGQKRKHNRGFGFVEV  162
            SS  D+ LFV G+P  A E  L EYFE  G +   V       +  Q    +RGF FV  
Sbjct  12   SSEDDRKLFVGGLPQEASENDLKEYFEAYGSVTSTVL------KMDQMTGRSRGFAFVVF  65

Query  163  GT------VKEHQQLIRMSK  176
             T      V E + +++  K
Sbjct  66   ATPESLESVLEQEHVVKGKK  85


>ref|XP_004441492.1| PREDICTED: DAZ-associated protein 1 [Ceratotherium simum simum]
Length=472

 Score = 40.8 bits (94),  Expect = 1.0, Method: Compositional matrix adjust.
 Identities = 23/76 (30%), Positives = 36/76 (47%), Gaps = 10/76 (13%)

Query  110  LFVAGIPNVAREQHLTEYFEDLGIIVRDVFVPSAFNQNGQKRKHNRGFGFVEVGTVKEHQ  169
            +FV GIP+   E  L EYF+  G++   V +  A      +++  RGFGF+     +   
Sbjct  181  IFVGGIPHNCGETELREYFKKFGVVTEVVMIYDA------EKQRPRGFGFITFEDEQSVD  234

Query  170  QLIRMS----KNKKIE  181
            Q + M       KK+E
Sbjct  235  QAVNMHFHDIMGKKVE  250


>ref|XP_008059032.1| PREDICTED: DAZ-associated protein 1 isoform X2 [Tarsius syrichta]
Length=337

 Score = 40.8 bits (94),  Expect = 1.0, Method: Compositional matrix adjust.
 Identities = 23/76 (30%), Positives = 36/76 (47%), Gaps = 10/76 (13%)

Query  110  LFVAGIPNVAREQHLTEYFEDLGIIVRDVFVPSAFNQNGQKRKHNRGFGFVEVGTVKEHQ  169
            +FV GIP+   E  L EYF+  G++   V +  A      +++  RGFGF+     +   
Sbjct  74   IFVGGIPHNCGETELREYFKKFGVVTEVVMIYDA------EKQRPRGFGFITFEDEQSVD  127

Query  170  QLIRMS----KNKKIE  181
            Q + M       KK+E
Sbjct  128  QAVNMHFHDIMGKKVE  143


>ref|XP_013820910.1| PREDICTED: DAZ-associated protein 1 [Capra hircus]
Length=384

 Score = 40.8 bits (94),  Expect = 1.0, Method: Compositional matrix adjust.
 Identities = 23/76 (30%), Positives = 36/76 (47%), Gaps = 10/76 (13%)

Query  110  LFVAGIPNVAREQHLTEYFEDLGIIVRDVFVPSAFNQNGQKRKHNRGFGFVEVGTVKEHQ  169
            +FV GIP+   E  L EYF+  G++   V +  A      +++  RGFGF+     +   
Sbjct  115  IFVGGIPHNCGETELREYFKKFGVVTEVVMIYDA------EKQRPRGFGFITFEDEQSVD  168

Query  170  QLIRMS----KNKKIE  181
            Q + M       KK+E
Sbjct  169  QAVNMHFHDIMGKKVE  184


>ref|XP_011286492.1| PREDICTED: DAZ-associated protein 1 isoform X4 [Felis catus]
Length=393

 Score = 40.8 bits (94),  Expect = 1.0, Method: Compositional matrix adjust.
 Identities = 23/76 (30%), Positives = 36/76 (47%), Gaps = 10/76 (13%)

Query  110  LFVAGIPNVAREQHLTEYFEDLGIIVRDVFVPSAFNQNGQKRKHNRGFGFVEVGTVKEHQ  169
            +FV GIP+   E  L EYF+  G++   V +  A      +++  RGFGF+     +   
Sbjct  133  IFVGGIPHNCGETELREYFKKFGVVTEVVMIYDA------EKQRPRGFGFITFEDEQSVD  186

Query  170  QLIRMS----KNKKIE  181
            Q + M       KK+E
Sbjct  187  QAVNMHFHDIMGKKVE  202


>ref|XP_007992805.1| PREDICTED: DAZ-associated protein 1 isoform X8 [Chlorocebus sabaeus]
 ref|XP_009191300.1| PREDICTED: DAZ-associated protein 1 isoform X6 [Papio anubis]
 ref|XP_011928273.1| PREDICTED: DAZ-associated protein 1 isoform X6 [Cercocebus atys]
Length=375

 Score = 40.8 bits (94),  Expect = 1.0, Method: Compositional matrix adjust.
 Identities = 25/86 (29%), Positives = 42/86 (49%), Gaps = 11/86 (13%)

Query  110  LFVAGIPNVAREQHLTEYFEDLGIIVRDVFVPSAFNQNGQKRKHNRGFGFVEVGTVKEHQ  169
            +FV GIP+   E  L EYF+  G++   V +  A      +++  RGFGF+      E +
Sbjct  115  IFVGGIPHNCGETELREYFKKFGVVTEVVMIYDA------EKQRPRGFGFITF----EDE  164

Query  170  QLIRMSKNKKIEFDLKPAKWDIERAR  195
            Q +  + N     D+   K +++RA 
Sbjct  165  QSVDQAVNMHFH-DIMGKKVEVKRAE  189


>gb|EUB59616.1| Heterogeneous nuclear ribonucleoproteins A2/B1 [Echinococcus 
granulosus]
Length=293

 Score = 40.4 bits (93),  Expect = 1.0, Method: Compositional matrix adjust.
 Identities = 31/87 (36%), Positives = 44/87 (51%), Gaps = 11/87 (13%)

Query  101  NGSSRVDQT--LFVAGIPNVAREQHLTEYFEDLGIIVRDVFVPSAFNQNGQKRKHNRGFG  158
            N  SR +Q   LF+ G+ +   E HL EY+   G IV  V +  +  Q+G+    +RGFG
Sbjct  8    NNDSRTEQYRKLFIGGLTHNTTEDHLKEYYSAWGEIVDVVVMKDS--QSGR----SRGFG  61

Query  159  FVEVGTVKEHQQLIRMSKNKKIEFDLK  185
            FV   T KE + +     N+  E D K
Sbjct  62   FV---TYKEPEMVDTAQANRPHEIDGK  85


>ref|XP_006050586.1| PREDICTED: DAZ-associated protein 1 isoform X3 [Bubalus bubalis]
Length=373

 Score = 40.8 bits (94),  Expect = 1.0, Method: Compositional matrix adjust.
 Identities = 25/85 (29%), Positives = 42/85 (49%), Gaps = 11/85 (13%)

Query  110  LFVAGIPNVAREQHLTEYFEDLGIIVRDVFVPSAFNQNGQKRKHNRGFGFVEVGTVKEHQ  169
            +FV GIP+   E  L EYF+  G++   V +  A      +++  RGFGF+      E +
Sbjct  110  IFVGGIPHNCGETELREYFKKFGVVTEVVMIYDA------EKQRPRGFGFITF----EDE  159

Query  170  QLIRMSKNKKIEFDLKPAKWDIERA  194
            Q +  + N     D+   K +++RA
Sbjct  160  QSVDQAVNMHFH-DIMGKKVEVKRA  183


>gb|KKU49817.1| RNA-binding protein [Parcubacteria bacterium GW2011_GWA1_47_10]
 gb|KKW09767.1| RNA-binding protein [Parcubacteria bacterium GW2011_GWB1_49_7]
Length=97

 Score = 38.5 bits (88),  Expect = 1.0, Method: Compositional matrix adjust.
 Identities = 22/72 (31%), Positives = 35/72 (49%), Gaps = 6/72 (8%)

Query  110  LFVAGIPNVAREQHLTEYFEDLGIIVRDVFVPSAFNQNGQKRKHNRGFGFVEVGTVKEHQ  169
            L+V GIP  + E  +TE F   G +V    +        +    ++GFGFVE+ + +E Q
Sbjct  5    LYVGGIPYTSTEATMTEAFSKAGNVVSAAIIID------RMTGRSKGFGFVEMSSEEEAQ  58

Query  170  QLIRMSKNKKIE  181
            + I M   +  E
Sbjct  59   KAIDMFNGQDFE  70


>ref|XP_005587455.1| PREDICTED: DAZ-associated protein 1 isoform X7 [Macaca fascicularis]
 ref|XP_005587456.1| PREDICTED: DAZ-associated protein 1 isoform X8 [Macaca fascicularis]
 ref|XP_011852802.1| PREDICTED: DAZ-associated protein 1 isoform X2 [Mandrillus leucophaeus]
 gb|EAW69504.1| DAZ associated protein 1, isoform CRA_d, partial [Homo sapiens]
Length=337

 Score = 40.4 bits (93),  Expect = 1.0, Method: Compositional matrix adjust.
 Identities = 23/76 (30%), Positives = 36/76 (47%), Gaps = 10/76 (13%)

Query  110  LFVAGIPNVAREQHLTEYFEDLGIIVRDVFVPSAFNQNGQKRKHNRGFGFVEVGTVKEHQ  169
            +FV GIP+   E  L EYF+  G++   V +  A      +++  RGFGF+     +   
Sbjct  74   IFVGGIPHNCGETELREYFKKFGVVTEVVMIYDA------EKQRPRGFGFITFEDEQSVD  127

Query  170  QLIRMS----KNKKIE  181
            Q + M       KK+E
Sbjct  128  QAVNMHFHDIMGKKVE  143


>ref|XP_004417802.2| PREDICTED: DAZ-associated protein 1 isoform X4 [Odobenus rosmarus 
divergens]
 ref|XP_013977470.1| PREDICTED: DAZ-associated protein 1 isoform X3 [Canis lupus familiaris]
Length=375

 Score = 40.8 bits (94),  Expect = 1.0, Method: Compositional matrix adjust.
 Identities = 25/86 (29%), Positives = 42/86 (49%), Gaps = 11/86 (13%)

Query  110  LFVAGIPNVAREQHLTEYFEDLGIIVRDVFVPSAFNQNGQKRKHNRGFGFVEVGTVKEHQ  169
            +FV GIP+   E  L EYF+  G++   V +  A      +++  RGFGF+      E +
Sbjct  115  IFVGGIPHNCGETELREYFKKFGVVTEVVMIYDA------EKQRPRGFGFITF----EDE  164

Query  170  QLIRMSKNKKIEFDLKPAKWDIERAR  195
            Q +  + N     D+   K +++RA 
Sbjct  165  QSVDQAVNMHFH-DIMGKKVEVKRAE  189


>gb|KKP24625.1| RNP-1 like protein RNA-binding protein [Parcubacteria (Nomurabacteria) 
bacterium GW2011_GWF2_30_133]
 gb|KKP29042.1| RNP-1 like protein RNA-binding protein [Parcubacteria (Nomurabacteria) 
bacterium GW2011_GWE2_31_40]
 gb|KKP30548.1| RNP-1 like protein RNA-binding protein [Parcubacteria (Nomurabacteria) 
bacterium GW2011_GWF1_31_48]
 gb|KKP35033.1| RNP-1 like protein RNA-binding protein [Parcubacteria (Nomurabacteria) 
bacterium GW2011_GWE1_32_28]
Length=107

 Score = 38.5 bits (88),  Expect = 1.0, Method: Compositional matrix adjust.
 Identities = 22/72 (31%), Positives = 33/72 (46%), Gaps = 6/72 (8%)

Query  110  LFVAGIPNVAREQHLTEYFEDLGIIVRDVFVPSAFNQNGQKRKHNRGFGFVEVGTVKEHQ  169
            L+V G+P   +E  L E F   G +   V +    +        ++GFGFVE+ T  E Q
Sbjct  5    LYVGGLPYSVQEDALKELFAQAGSVTSAVIIMDKMS------GRSKGFGFVEMATEDEAQ  58

Query  170  QLIRMSKNKKIE  181
              I M   ++ E
Sbjct  59   GAISMFNEQEFE  70


>ref|XP_005890529.1| PREDICTED: DAZ-associated protein 1 [Bos mutus]
Length=462

 Score = 40.8 bits (94),  Expect = 1.0, Method: Compositional matrix adjust.
 Identities = 25/85 (29%), Positives = 42/85 (49%), Gaps = 11/85 (13%)

Query  110  LFVAGIPNVAREQHLTEYFEDLGIIVRDVFVPSAFNQNGQKRKHNRGFGFVEVGTVKEHQ  169
            +FV GIP+   E  L EYF+  G++   V +  A      +++  RGFGF+      E +
Sbjct  171  IFVGGIPHNCGETELREYFKKFGVVTEVVMIYDA------EKQRPRGFGFITF----EDE  220

Query  170  QLIRMSKNKKIEFDLKPAKWDIERA  194
            Q +  + N     D+   K +++RA
Sbjct  221  QSVDQAVNMHFH-DIMGKKVEVKRA  244


>ref|XP_008161256.1| PREDICTED: DAZ-associated protein 1 isoform X2 [Chrysemys picta 
bellii]
Length=408

 Score = 40.8 bits (94),  Expect = 1.0, Method: Compositional matrix adjust.
 Identities = 25/85 (29%), Positives = 42/85 (49%), Gaps = 11/85 (13%)

Query  110  LFVAGIPNVAREQHLTEYFEDLGIIVRDVFVPSAFNQNGQKRKHNRGFGFVEVGTVKEHQ  169
            +FV GIP+   E  L EYF+  G++   V +  A      +++  RGFGF+      E +
Sbjct  115  IFVGGIPHNCGETELREYFKKFGVVTEVVMIYDA------EKQRPRGFGFITF----EDE  164

Query  170  QLIRMSKNKKIEFDLKPAKWDIERA  194
            Q +  + N     D+   K +++RA
Sbjct  165  QSVDQAVNMHFH-DIMGKKVEVKRA  188


>ref|XP_009250816.1| PREDICTED: DAZ-associated protein 1 isoform X4 [Pongo abelii]
Length=411

 Score = 40.8 bits (94),  Expect = 1.1, Method: Compositional matrix adjust.
 Identities = 25/85 (29%), Positives = 42/85 (49%), Gaps = 11/85 (13%)

Query  110  LFVAGIPNVAREQHLTEYFEDLGIIVRDVFVPSAFNQNGQKRKHNRGFGFVEVGTVKEHQ  169
            +FV GIP+   E  L EYF+  G++   V +  A      +++  RGFGF+      E +
Sbjct  122  IFVGGIPHNCGETELREYFKKFGVVTEVVMIYDA------EKQRPRGFGFITF----EDE  171

Query  170  QLIRMSKNKKIEFDLKPAKWDIERA  194
            Q +  + N     D+   K +++RA
Sbjct  172  QSVDQAVNMHFH-DIMGKKVEVKRA  195


>ref|XP_010633232.1| PREDICTED: DAZ-associated protein 1 isoform X3 [Fukomys damarensis]
Length=403

 Score = 40.8 bits (94),  Expect = 1.1, Method: Compositional matrix adjust.
 Identities = 25/85 (29%), Positives = 42/85 (49%), Gaps = 11/85 (13%)

Query  110  LFVAGIPNVAREQHLTEYFEDLGIIVRDVFVPSAFNQNGQKRKHNRGFGFVEVGTVKEHQ  169
            +FV GIP+   E  L EYF+  G++   V +  A      +++  RGFGF+      E +
Sbjct  111  IFVGGIPHNCGETELREYFKKFGVVTEVVMIYDA------EKQRPRGFGFITF----EDE  160

Query  170  QLIRMSKNKKIEFDLKPAKWDIERA  194
            Q +  + N     D+   K +++RA
Sbjct  161  QSVDQAVNMHFH-DIMGKKVEVKRA  184


>ref|NP_733829.1| DAZ-associated protein 1 isoform a [Homo sapiens]
 ref|XP_003914632.1| PREDICTED: DAZ-associated protein 1 isoform X5 [Papio anubis]
 ref|XP_007992802.1| PREDICTED: DAZ-associated protein 1 isoform X7 [Chlorocebus sabaeus]
 7 more sequence titles

ref|XP_007992804.1| PREDICTED: DAZ-associated protein 1 isoform X7 [Chlorocebus sabaeus]
 ref|XP_009191299.1| PREDICTED: DAZ-associated protein 1 isoform X5 [Papio anubis]
 ref|XP_011747183.1| PREDICTED: DAZ-associated protein 1 isoform X6 [Macaca nemestrina]
 ref|XP_011747191.1| PREDICTED: DAZ-associated protein 1 isoform X6 [Macaca nemestrina]
 ref|XP_011928270.1| PREDICTED: DAZ-associated protein 1 isoform X5 [Cercocebus atys]
 ref|XP_011928272.1| PREDICTED: DAZ-associated protein 1 isoform X5 [Cercocebus atys]
 gb|EAW69503.1| DAZ associated protein 1, isoform CRA_c [Homo sapiens]

Length=378

 Score = 40.8 bits (94),  Expect = 1.1, Method: Compositional matrix adjust.
 Identities = 25/86 (29%), Positives = 42/86 (49%), Gaps = 11/86 (13%)

Query  110  LFVAGIPNVAREQHLTEYFEDLGIIVRDVFVPSAFNQNGQKRKHNRGFGFVEVGTVKEHQ  169
            +FV GIP+   E  L EYF+  G++   V +  A      +++  RGFGF+      E +
Sbjct  115  IFVGGIPHNCGETELREYFKKFGVVTEVVMIYDA------EKQRPRGFGFITF----EDE  164

Query  170  QLIRMSKNKKIEFDLKPAKWDIERAR  195
            Q +  + N     D+   K +++RA 
Sbjct  165  QSVDQAVNMHFH-DIMGKKVEVKRAE  189


>ref|XP_009250817.1| PREDICTED: DAZ-associated protein 1 isoform X5 [Pongo abelii]
Length=410

 Score = 40.8 bits (94),  Expect = 1.1, Method: Compositional matrix adjust.
 Identities = 25/85 (29%), Positives = 42/85 (49%), Gaps = 11/85 (13%)

Query  110  LFVAGIPNVAREQHLTEYFEDLGIIVRDVFVPSAFNQNGQKRKHNRGFGFVEVGTVKEHQ  169
            +FV GIP+   E  L EYF+  G++   V +  A      +++  RGFGF+      E +
Sbjct  122  IFVGGIPHNCGETELREYFKKFGVVTEVVMIYDA------EKQRPRGFGFITF----EDE  171

Query  170  QLIRMSKNKKIEFDLKPAKWDIERA  194
            Q +  + N     D+   K +++RA
Sbjct  172  QSVDQAVNMHFH-DIMGKKVEVKRA  195


>ref|XP_009250815.1| PREDICTED: DAZ-associated protein 1 isoform X3 [Pongo abelii]
Length=413

 Score = 40.8 bits (94),  Expect = 1.1, Method: Compositional matrix adjust.
 Identities = 25/85 (29%), Positives = 42/85 (49%), Gaps = 11/85 (13%)

Query  110  LFVAGIPNVAREQHLTEYFEDLGIIVRDVFVPSAFNQNGQKRKHNRGFGFVEVGTVKEHQ  169
            +FV GIP+   E  L EYF+  G++   V +  A      +++  RGFGF+      E +
Sbjct  121  IFVGGIPHNCGETELREYFKKFGVVTEVVMIYDA------EKQRPRGFGFITF----EDE  170

Query  170  QLIRMSKNKKIEFDLKPAKWDIERA  194
            Q +  + N     D+   K +++RA
Sbjct  171  QSVDQAVNMHFH-DIMGKKVEVKRA  194


>emb|CBY09798.1| unnamed protein product [Oikopleura dioica]
Length=367

 Score = 40.4 bits (93),  Expect = 1.1, Method: Compositional matrix adjust.
 Identities = 26/101 (26%), Positives = 46/101 (46%), Gaps = 15/101 (15%)

Query  108  QTLFVAGIPNVAREQHLTEYFEDLGIIVRDVFVPSAFNQNGQKRKHNRGFGFVEVGTVKE  167
            + LFV G+P    E+ L EYF   G +V  +           K K  R FGFV   +++E
Sbjct  124  EKLFVGGLPVSFSEEELGEYFSQYGTVVESLV----------KTKEGRSFGFVTFASIEE  173

Query  168  HQQLIRMSKNKKIEFDLKPAKWDIERARSYGTPSPSIAPKI  208
                + +  +K  + D+     +++RA +     P ++P +
Sbjct  174  ANAALEIKTHKIGDRDV-----EVKRAIANTRAPPMVSPTM  209


>ref|XP_012789609.1| PREDICTED: DAZ-associated protein 1 isoform X2 [Sorex araneus]
Length=375

 Score = 40.4 bits (93),  Expect = 1.1, Method: Compositional matrix adjust.
 Identities = 25/86 (29%), Positives = 42/86 (49%), Gaps = 11/86 (13%)

Query  110  LFVAGIPNVAREQHLTEYFEDLGIIVRDVFVPSAFNQNGQKRKHNRGFGFVEVGTVKEHQ  169
            +FV GIP+   E  L EYF+  G++   V +  A      +++  RGFGF+      E +
Sbjct  115  IFVGGIPHNCGETELREYFKKFGVVTEVVMIYDA------EKQRPRGFGFITF----EDE  164

Query  170  QLIRMSKNKKIEFDLKPAKWDIERAR  195
            Q +  + N     D+   K +++RA 
Sbjct  165  QSVDQAVNMHFH-DIMGKKVEVKRAE  189


>ref|XP_013918737.1| PREDICTED: DAZ-associated protein 1 isoform X1 [Thamnophis sirtalis]
Length=407

 Score = 40.8 bits (94),  Expect = 1.1, Method: Compositional matrix adjust.
 Identities = 25/85 (29%), Positives = 42/85 (49%), Gaps = 11/85 (13%)

Query  110  LFVAGIPNVAREQHLTEYFEDLGIIVRDVFVPSAFNQNGQKRKHNRGFGFVEVGTVKEHQ  169
            +FV GIP+   E  L EYF+  G++   V +  A      +++  RGFGF+      E +
Sbjct  114  IFVGGIPHNCGETELREYFKKFGVVTEVVMIYDA------EKQRPRGFGFITF----EDE  163

Query  170  QLIRMSKNKKIEFDLKPAKWDIERA  194
            Q +  + N     D+   K +++RA
Sbjct  164  QSVDQAVNMHFH-DIMGKKVEVKRA  187


>ref|XP_011971526.1| PREDICTED: DAZ-associated protein 1 isoform X5 [Ovis aries musimon]
Length=378

 Score = 40.4 bits (93),  Expect = 1.1, Method: Compositional matrix adjust.
 Identities = 25/86 (29%), Positives = 42/86 (49%), Gaps = 11/86 (13%)

Query  110  LFVAGIPNVAREQHLTEYFEDLGIIVRDVFVPSAFNQNGQKRKHNRGFGFVEVGTVKEHQ  169
            +FV GIP+   E  L EYF+  G++   V +  A      +++  RGFGF+      E +
Sbjct  115  IFVGGIPHNCGETELREYFKKFGVVTEVVMIYDA------EKQRPRGFGFITF----EDE  164

Query  170  QLIRMSKNKKIEFDLKPAKWDIERAR  195
            Q +  + N     D+   K +++RA 
Sbjct  165  QSVDQAVNMHFH-DIMGKKVEVKRAE  189


>emb|CDS21411.1| heterogeneous nuclear ribonucleoprotein [Echinococcus granulosus]
Length=752

 Score = 40.8 bits (94),  Expect = 1.1, Method: Compositional matrix adjust.
 Identities = 36/122 (30%), Positives = 55/122 (45%), Gaps = 24/122 (20%)

Query  97   DTDTNGSSRVDQTLFVAGIPNVAREQHLTEYFEDLGIIVRDVFVPSAFNQNGQKRKHNRG  156
            + + +GS R ++ +FV  +     EQ+L +YF   G IV        +   G     +RG
Sbjct  42   ENNCSGSPRSNKKIFVGALTPDTTEQNLIDYFSKFGEIVSCAV--KLYRDTGC----SRG  95

Query  157  FGFV---------EVGTVKEHQQLIRMSKNKKIEFDLKPAKW--DIERARSYGTPSPSIA  205
            FGF+         +V ++ EH     +   KKI  D KPAK   D +R    G   P ++
Sbjct  96   FGFIVFKSDESVKKVLSIPEH-----IVNGKKI--DPKPAKCPKDTQRKVFVGGLDPDVS  148

Query  206  PK  207
            PK
Sbjct  149  PK  150


>ref|XP_004285911.1| PREDICTED: DAZ-associated protein 1 isoform X4 [Orcinus orca]
 ref|XP_007460448.1| PREDICTED: DAZ-associated protein 1 isoform X2 [Lipotes vexillifer]
Length=378

 Score = 40.4 bits (93),  Expect = 1.1, Method: Compositional matrix adjust.
 Identities = 25/86 (29%), Positives = 42/86 (49%), Gaps = 11/86 (13%)

Query  110  LFVAGIPNVAREQHLTEYFEDLGIIVRDVFVPSAFNQNGQKRKHNRGFGFVEVGTVKEHQ  169
            +FV GIP+   E  L EYF+  G++   V +  A      +++  RGFGF+      E +
Sbjct  115  IFVGGIPHNCGETELREYFKKFGVVTEVVMIYDA------EKQRPRGFGFITF----EDE  164

Query  170  QLIRMSKNKKIEFDLKPAKWDIERAR  195
            Q +  + N     D+   K +++RA 
Sbjct  165  QSVDQAVNMHFH-DIMGKKVEVKRAE  189


>ref|XP_004595974.1| PREDICTED: DAZ-associated protein 1 [Ochotona princeps]
Length=420

 Score = 40.8 bits (94),  Expect = 1.1, Method: Compositional matrix adjust.
 Identities = 23/76 (30%), Positives = 36/76 (47%), Gaps = 10/76 (13%)

Query  110  LFVAGIPNVAREQHLTEYFEDLGIIVRDVFVPSAFNQNGQKRKHNRGFGFVEVGTVKEHQ  169
            +FV GIP+   E  L EYF+  G++   V +  A      +++  RGFGF+     +   
Sbjct  128  IFVGGIPHNCGETELREYFKKFGVVTEVVMIYDA------EKQRPRGFGFITFEDEQSVD  181

Query  170  QLIRMS----KNKKIE  181
            Q + M       KK+E
Sbjct  182  QAVNMHFHDIMGKKVE  197


>ref|XP_005302407.1| PREDICTED: DAZ-associated protein 1 isoform X1 [Chrysemys picta 
bellii]
Length=409

 Score = 40.8 bits (94),  Expect = 1.1, Method: Compositional matrix adjust.
 Identities = 25/85 (29%), Positives = 42/85 (49%), Gaps = 11/85 (13%)

Query  110  LFVAGIPNVAREQHLTEYFEDLGIIVRDVFVPSAFNQNGQKRKHNRGFGFVEVGTVKEHQ  169
            +FV GIP+   E  L EYF+  G++   V +  A      +++  RGFGF+      E +
Sbjct  116  IFVGGIPHNCGETELREYFKKFGVVTEVVMIYDA------EKQRPRGFGFITF----EDE  165

Query  170  QLIRMSKNKKIEFDLKPAKWDIERA  194
            Q +  + N     D+   K +++RA
Sbjct  166  QSVDQAVNMHFH-DIMGKKVEVKRA  189


>ref|XP_005661445.1| PREDICTED: DAZ-associated protein 1 isoform X2 [Sus scrofa]
Length=378

 Score = 40.4 bits (93),  Expect = 1.1, Method: Compositional matrix adjust.
 Identities = 25/86 (29%), Positives = 42/86 (49%), Gaps = 11/86 (13%)

Query  110  LFVAGIPNVAREQHLTEYFEDLGIIVRDVFVPSAFNQNGQKRKHNRGFGFVEVGTVKEHQ  169
            +FV GIP+   E  L EYF+  G++   V +  A      +++  RGFGF+      E +
Sbjct  115  IFVGGIPHNCGETELREYFKKFGVVTEVVMIYDA------EKQRPRGFGFITF----EDE  164

Query  170  QLIRMSKNKKIEFDLKPAKWDIERAR  195
            Q +  + N     D+   K +++RA 
Sbjct  165  QSVDQAVNMHFH-DIMGKKVEVKRAE  189


>ref|XP_010633230.1| PREDICTED: DAZ-associated protein 1 isoform X1 [Fukomys damarensis]
Length=404

 Score = 40.8 bits (94),  Expect = 1.1, Method: Compositional matrix adjust.
 Identities = 25/85 (29%), Positives = 42/85 (49%), Gaps = 11/85 (13%)

Query  110  LFVAGIPNVAREQHLTEYFEDLGIIVRDVFVPSAFNQNGQKRKHNRGFGFVEVGTVKEHQ  169
            +FV GIP+   E  L EYF+  G++   V +  A      +++  RGFGF+      E +
Sbjct  112  IFVGGIPHNCGETELREYFKKFGVVTEVVMIYDA------EKQRPRGFGFITF----EDE  161

Query  170  QLIRMSKNKKIEFDLKPAKWDIERA  194
            Q +  + N     D+   K +++RA
Sbjct  162  QSVDQAVNMHFH-DIMGKKVEVKRA  185


>emb|CDS15233.1| Heterogeneous nuclear ribonucleoprotein A2 [Echinococcus granulosus]
Length=326

 Score = 40.4 bits (93),  Expect = 1.1, Method: Compositional matrix adjust.
 Identities = 31/87 (36%), Positives = 44/87 (51%), Gaps = 11/87 (13%)

Query  101  NGSSRVDQT--LFVAGIPNVAREQHLTEYFEDLGIIVRDVFVPSAFNQNGQKRKHNRGFG  158
            N  SR +Q   LF+ G+ +   E HL EY+   G IV  V +  +  Q+G+    +RGFG
Sbjct  8    NNDSRTEQYRKLFIGGLTHNTTEDHLKEYYSAWGEIVDVVVMKDS--QSGR----SRGFG  61

Query  159  FVEVGTVKEHQQLIRMSKNKKIEFDLK  185
            FV   T KE + +     N+  E D K
Sbjct  62   FV---TYKEPEMVDTAQANRPHEIDGK  85


>ref|XP_009250813.1| PREDICTED: DAZ-associated protein 1 isoform X1 [Pongo abelii]
Length=414

 Score = 40.8 bits (94),  Expect = 1.1, Method: Compositional matrix adjust.
 Identities = 25/85 (29%), Positives = 42/85 (49%), Gaps = 11/85 (13%)

Query  110  LFVAGIPNVAREQHLTEYFEDLGIIVRDVFVPSAFNQNGQKRKHNRGFGFVEVGTVKEHQ  169
            +FV GIP+   E  L EYF+  G++   V +  A      +++  RGFGF+      E +
Sbjct  122  IFVGGIPHNCGETELREYFKKFGVVTEVVMIYDA------EKQRPRGFGFITF----EDE  171

Query  170  QLIRMSKNKKIEFDLKPAKWDIERA  194
            Q +  + N     D+   K +++RA
Sbjct  172  QSVDQAVNMHFH-DIMGKKVEVKRA  195


>gb|KIK08600.1| hypothetical protein K443DRAFT_672131 [Laccaria amethystina LaAM-08-1]
Length=149

 Score = 39.3 bits (90),  Expect = 1.1, Method: Compositional matrix adjust.
 Identities = 25/65 (38%), Positives = 36/65 (55%), Gaps = 2/65 (3%)

Query  109  TLFVAGIPNVAREQHLTEYFEDLGIIVRDVFVPSAF-NQNGQKRKHNRGFGFVEVGTVKE  167
            T+FV GI +   E  + + F   G IV+ V +PSA  NQ+ Q +  +RGF FV  G+  +
Sbjct  10   TVFVGGISDDTDETAIYQSFSTFGEIVQ-VQLPSAVTNQSQQSQAKHRGFAFVTYGSSSD  68

Query  168  HQQLI  172
             Q  I
Sbjct  69   AQDAI  73


>ref|XP_004654943.1| PREDICTED: DAZ-associated protein 1 isoform X2 [Jaculus jaculus]
Length=411

 Score = 40.8 bits (94),  Expect = 1.1, Method: Compositional matrix adjust.
 Identities = 25/85 (29%), Positives = 42/85 (49%), Gaps = 11/85 (13%)

Query  110  LFVAGIPNVAREQHLTEYFEDLGIIVRDVFVPSAFNQNGQKRKHNRGFGFVEVGTVKEHQ  169
            +FV GIP+   E  L EYF+  G++   V +  A      +++  RGFGF+      E +
Sbjct  114  IFVGGIPHNCGETELREYFKKFGVVTEVVMIYDA------EKQRPRGFGFITF----EDE  163

Query  170  QLIRMSKNKKIEFDLKPAKWDIERA  194
            Q +  + N     D+   K +++RA
Sbjct  164  QSVDQAVNMHFH-DIMGKKVEVKRA  187


>ref|XP_005890797.1| PREDICTED: heterogeneous nuclear ribonucleoproteins A2/B1-like 
[Bos mutus]
Length=266

 Score = 40.4 bits (93),  Expect = 1.1, Method: Compositional matrix adjust.
 Identities = 28/72 (39%), Positives = 35/72 (49%), Gaps = 6/72 (8%)

Query  89   PLKSTALLDTDTNGSSRVDQTLFVAGIPNVAREQHLTEYFEDLGIIVRDVFVPSAFNQNG  148
            P ++ A   ++  GS    + LFV GI     E HL EYFE  G I  D        Q+G
Sbjct  101  PKRAVAREKSEKQGSLVNVKKLFVGGIKEDTEEHHLREYFEKYGKI--DAIEIITDRQSG  158

Query  149  QKRKHNRGFGFV  160
            +K    RGFGFV
Sbjct  159  KK----RGFGFV  166


>emb|CDJ02783.1| Heterogeneous nuclear ribonucleoprotein A2 [Echinococcus multilocularis]
 emb|CDS40065.1| Heterogeneous nuclear ribonucleoprotein A2 [Echinococcus multilocularis]
Length=333

 Score = 40.4 bits (93),  Expect = 1.1, Method: Compositional matrix adjust.
 Identities = 31/87 (36%), Positives = 44/87 (51%), Gaps = 11/87 (13%)

Query  101  NGSSRVDQT--LFVAGIPNVAREQHLTEYFEDLGIIVRDVFVPSAFNQNGQKRKHNRGFG  158
            N  SR +Q   LF+ G+ +   E HL EY+   G IV  V +  +  Q+G+    +RGFG
Sbjct  8    NNDSRTEQYRKLFIGGLTHNTTEDHLKEYYSAWGEIVDVVVMKDS--QSGR----SRGFG  61

Query  159  FVEVGTVKEHQQLIRMSKNKKIEFDLK  185
            FV   T KE + +     N+  E D K
Sbjct  62   FV---TYKEPEMVDTAQANRPHEIDGK  85


>ref|XP_010633233.1| PREDICTED: DAZ-associated protein 1 isoform X4 [Fukomys damarensis]
Length=402

 Score = 40.4 bits (93),  Expect = 1.1, Method: Compositional matrix adjust.
 Identities = 25/85 (29%), Positives = 42/85 (49%), Gaps = 11/85 (13%)

Query  110  LFVAGIPNVAREQHLTEYFEDLGIIVRDVFVPSAFNQNGQKRKHNRGFGFVEVGTVKEHQ  169
            +FV GIP+   E  L EYF+  G++   V +  A      +++  RGFGF+      E +
Sbjct  111  IFVGGIPHNCGETELREYFKKFGVVTEVVMIYDA------EKQRPRGFGFITF----EDE  160

Query  170  QLIRMSKNKKIEFDLKPAKWDIERA  194
            Q +  + N     D+   K +++RA
Sbjct  161  QSVDQAVNMHFH-DIMGKKVEVKRA  184


>ref|XP_006171826.1| PREDICTED: LOW QUALITY PROTEIN: DAZ-associated protein 1 [Tupaia 
chinensis]
Length=515

 Score = 40.8 bits (94),  Expect = 1.1, Method: Compositional matrix adjust.
 Identities = 23/76 (30%), Positives = 36/76 (47%), Gaps = 10/76 (13%)

Query  110  LFVAGIPNVAREQHLTEYFEDLGIIVRDVFVPSAFNQNGQKRKHNRGFGFVEVGTVKEHQ  169
            +FV GIP+   E  L EYF+  G++   V +  A      +++  RGFGF+     +   
Sbjct  224  IFVGGIPHNCGETELREYFKKFGVVTEVVMIYDA------EKQRPRGFGFITFEDEQSVD  277

Query  170  QLIRMS----KNKKIE  181
            Q + M       KK+E
Sbjct  278  QAVNMHFHDIMGKKVE  293


>ref|XP_010633231.1| PREDICTED: DAZ-associated protein 1 isoform X2 [Fukomys damarensis]
Length=403

 Score = 40.4 bits (93),  Expect = 1.1, Method: Compositional matrix adjust.
 Identities = 25/85 (29%), Positives = 42/85 (49%), Gaps = 11/85 (13%)

Query  110  LFVAGIPNVAREQHLTEYFEDLGIIVRDVFVPSAFNQNGQKRKHNRGFGFVEVGTVKEHQ  169
            +FV GIP+   E  L EYF+  G++   V +  A      +++  RGFGF+      E +
Sbjct  112  IFVGGIPHNCGETELREYFKKFGVVTEVVMIYDA------EKQRPRGFGFITF----EDE  161

Query  170  QLIRMSKNKKIEFDLKPAKWDIERA  194
            Q +  + N     D+   K +++RA
Sbjct  162  QSVDQAVNMHFH-DIMGKKVEVKRA  185


>ref|XP_009250814.1| PREDICTED: DAZ-associated protein 1 isoform X2 [Pongo abelii]
Length=413

 Score = 40.4 bits (93),  Expect = 1.1, Method: Compositional matrix adjust.
 Identities = 25/85 (29%), Positives = 42/85 (49%), Gaps = 11/85 (13%)

Query  110  LFVAGIPNVAREQHLTEYFEDLGIIVRDVFVPSAFNQNGQKRKHNRGFGFVEVGTVKEHQ  169
            +FV GIP+   E  L EYF+  G++   V +  A      +++  RGFGF+      E +
Sbjct  122  IFVGGIPHNCGETELREYFKKFGVVTEVVMIYDA------EKQRPRGFGFITF----EDE  171

Query  170  QLIRMSKNKKIEFDLKPAKWDIERA  194
            Q +  + N     D+   K +++RA
Sbjct  172  QSVDQAVNMHFH-DIMGKKVEVKRA  195


>ref|XP_012411610.1| PREDICTED: DAZ-associated protein 1 [Trichechus manatus latirostris]
Length=428

 Score = 40.8 bits (94),  Expect = 1.1, Method: Compositional matrix adjust.
 Identities = 25/85 (29%), Positives = 42/85 (49%), Gaps = 11/85 (13%)

Query  110  LFVAGIPNVAREQHLTEYFEDLGIIVRDVFVPSAFNQNGQKRKHNRGFGFVEVGTVKEHQ  169
            +FV GIP+   E  L EYF+  G++   V +  A      +++  RGFGF+      E +
Sbjct  137  IFVGGIPHNCGETELREYFKKFGVVTEVVMIYDA------EKQRPRGFGFITF----EDE  186

Query  170  QLIRMSKNKKIEFDLKPAKWDIERA  194
            Q +  + N     D+   K +++RA
Sbjct  187  QSVDQAVNMHFH-DIMGKKVEVKRA  210


>ref|XP_004654941.1| PREDICTED: DAZ-associated protein 1 isoform X1 [Jaculus jaculus]
Length=412

 Score = 40.4 bits (93),  Expect = 1.1, Method: Compositional matrix adjust.
 Identities = 25/85 (29%), Positives = 42/85 (49%), Gaps = 11/85 (13%)

Query  110  LFVAGIPNVAREQHLTEYFEDLGIIVRDVFVPSAFNQNGQKRKHNRGFGFVEVGTVKEHQ  169
            +FV GIP+   E  L EYF+  G++   V +  A      +++  RGFGF+      E +
Sbjct  115  IFVGGIPHNCGETELREYFKKFGVVTEVVMIYDA------EKQRPRGFGFITF----EDE  164

Query  170  QLIRMSKNKKIEFDLKPAKWDIERA  194
            Q +  + N     D+   K +++RA
Sbjct  165  QSVDQAVNMHFH-DIMGKKVEVKRA  188


>dbj|BAB71295.1| unnamed protein product [Homo sapiens]
Length=289

 Score = 40.4 bits (93),  Expect = 1.2, Method: Compositional matrix adjust.
 Identities = 23/76 (30%), Positives = 36/76 (47%), Gaps = 10/76 (13%)

Query  110  LFVAGIPNVAREQHLTEYFEDLGIIVRDVFVPSAFNQNGQKRKHNRGFGFVEVGTVKEHQ  169
            +FV GIP+   E  L EYF+  G++   V +  A      +++  RGFGF+     +   
Sbjct  26   IFVGGIPHNCGETELREYFKKFGVVTEVVMIYDA------EKQRPRGFGFITFEDEQSVD  79

Query  170  QLIRMS----KNKKIE  181
            Q + M       KK+E
Sbjct  80   QAVNMHFHDIMGKKVE  95


>ref|XP_004717042.1| PREDICTED: DAZ-associated protein 1 [Echinops telfairi]
Length=454

 Score = 40.8 bits (94),  Expect = 1.2, Method: Compositional matrix adjust.
 Identities = 25/85 (29%), Positives = 42/85 (49%), Gaps = 11/85 (13%)

Query  110  LFVAGIPNVAREQHLTEYFEDLGIIVRDVFVPSAFNQNGQKRKHNRGFGFVEVGTVKEHQ  169
            +FV GIP+   E  L EYF+  G++   V +  A      +++  RGFGF+      E +
Sbjct  163  IFVGGIPHNCGETELREYFKKFGVVTEVVMIYDA------EKQRPRGFGFITF----EDE  212

Query  170  QLIRMSKNKKIEFDLKPAKWDIERA  194
            Q +  + N     D+   K +++RA
Sbjct  213  QSVDQAVNMHFH-DIMGKKVEVKRA  236


>gb|KFO28898.1| DAZ-associated protein 1 [Fukomys damarensis]
Length=410

 Score = 40.4 bits (93),  Expect = 1.2, Method: Compositional matrix adjust.
 Identities = 25/85 (29%), Positives = 42/85 (49%), Gaps = 11/85 (13%)

Query  110  LFVAGIPNVAREQHLTEYFEDLGIIVRDVFVPSAFNQNGQKRKHNRGFGFVEVGTVKEHQ  169
            +FV GIP+   E  L EYF+  G++   V +  A      +++  RGFGF+      E +
Sbjct  119  IFVGGIPHNCGETELREYFKKFGVVTEVVMIYDA------EKQRPRGFGFITF----EDE  168

Query  170  QLIRMSKNKKIEFDLKPAKWDIERA  194
            Q +  + N     D+   K +++RA
Sbjct  169  QSVDQAVNMHFH-DIMGKKVEVKRA  192


>gb|AAH77252.1| LOC398218 protein [Xenopus laevis]
Length=405

 Score = 40.4 bits (93),  Expect = 1.2, Method: Compositional matrix adjust.
 Identities = 25/85 (29%), Positives = 41/85 (48%), Gaps = 11/85 (13%)

Query  110  LFVAGIPNVAREQHLTEYFEDLGIIVRDVFVPSAFNQNGQKRKHNRGFGFVEVGTVKEHQ  169
            +FV GIP+   E  L EYF   G++   V +  A      +++  RGFGF+      E +
Sbjct  116  IFVGGIPHNCGETELKEYFNRFGVVTEVVMIYDA------EKQRPRGFGFITF----EDE  165

Query  170  QLIRMSKNKKIEFDLKPAKWDIERA  194
            Q +  + N     D+   K +++RA
Sbjct  166  QSVDQAVNMHFH-DIMGKKVEVKRA  189


>ref|XP_005083368.1| PREDICTED: DAZ-associated protein 1 isoform X1 [Mesocricetus 
auratus]
 ref|XP_005359121.1| PREDICTED: DAZ-associated protein 1 isoform X1 [Microtus ochrogaster]
 ref|XP_006514132.1| PREDICTED: DAZ-associated protein 1 isoform X1 [Mus musculus]
Length=407

 Score = 40.4 bits (93),  Expect = 1.2, Method: Compositional matrix adjust.
 Identities = 25/85 (29%), Positives = 42/85 (49%), Gaps = 11/85 (13%)

Query  110  LFVAGIPNVAREQHLTEYFEDLGIIVRDVFVPSAFNQNGQKRKHNRGFGFVEVGTVKEHQ  169
            +FV GIP+   E  L EYF+  G++   V +  A      +++  RGFGF+      E +
Sbjct  115  IFVGGIPHNCGETELREYFKKFGVVTEVVMIYDA------EKQRPRGFGFITF----EDE  164

Query  170  QLIRMSKNKKIEFDLKPAKWDIERA  194
            Q +  + N     D+   K +++RA
Sbjct  165  QSVDQAVNMHFH-DIMGKKVEVKRA  188


>ref|XP_012815278.1| PREDICTED: DAZ-associated protein 1 isoform X5 [Xenopus (Silurana) 
tropicalis]
Length=370

 Score = 40.4 bits (93),  Expect = 1.2, Method: Compositional matrix adjust.
 Identities = 25/85 (29%), Positives = 42/85 (49%), Gaps = 11/85 (13%)

Query  110  LFVAGIPNVAREQHLTEYFEDLGIIVRDVFVPSAFNQNGQKRKHNRGFGFVEVGTVKEHQ  169
            +FV GIP+   E  L EYF+  G++   V +  A      +++  RGFGF+      E +
Sbjct  75   IFVGGIPHNCGETELREYFKRFGVVTEVVMIYDA------EKQRPRGFGFITF----EDE  124

Query  170  QLIRMSKNKKIEFDLKPAKWDIERA  194
            Q +  + N     D+   K +++RA
Sbjct  125  QSVDQAVNMHFH-DIMGKKVEVKRA  148


>ref|XP_006206658.1| PREDICTED: DAZ-associated protein 1 [Vicugna pacos]
Length=528

 Score = 40.8 bits (94),  Expect = 1.2, Method: Compositional matrix adjust.
 Identities = 25/85 (29%), Positives = 42/85 (49%), Gaps = 11/85 (13%)

Query  110  LFVAGIPNVAREQHLTEYFEDLGIIVRDVFVPSAFNQNGQKRKHNRGFGFVEVGTVKEHQ  169
            +FV GIP+   E  L EYF+  G++   V +  A      +++  RGFGF+      E +
Sbjct  237  IFVGGIPHNCGETELREYFKKFGVVTEVVMIYDA------EKQRPRGFGFITF----EDE  286

Query  170  QLIRMSKNKKIEFDLKPAKWDIERA  194
            Q +  + N     D+   K +++RA
Sbjct  287  QSVDQAVNMHFH-DIMGKKVEVKRA  310


>ref|XP_006050584.1| PREDICTED: DAZ-associated protein 1 isoform X1 [Bubalus bubalis]
 tpg|DAA27535.1| TPA: DAZ associated protein 1 [Bos taurus]
Length=444

 Score = 40.4 bits (93),  Expect = 1.2, Method: Compositional matrix adjust.
 Identities = 25/85 (29%), Positives = 42/85 (49%), Gaps = 11/85 (13%)

Query  110  LFVAGIPNVAREQHLTEYFEDLGIIVRDVFVPSAFNQNGQKRKHNRGFGFVEVGTVKEHQ  169
            +FV GIP+   E  L EYF+  G++   V +  A      +++  RGFGF+      E +
Sbjct  153  IFVGGIPHNCGETELREYFKKFGVVTEVVMIYDA------EKQRPRGFGFITF----EDE  202

Query  170  QLIRMSKNKKIEFDLKPAKWDIERA  194
            Q +  + N     D+   K +++RA
Sbjct  203  QSVDQAVNMHFH-DIMGKKVEVKRA  226


>ref|XP_012659962.1| PREDICTED: DAZ-associated protein 1 isoform X2 [Otolemur garnettii]
Length=406

 Score = 40.4 bits (93),  Expect = 1.2, Method: Compositional matrix adjust.
 Identities = 25/85 (29%), Positives = 42/85 (49%), Gaps = 11/85 (13%)

Query  110  LFVAGIPNVAREQHLTEYFEDLGIIVRDVFVPSAFNQNGQKRKHNRGFGFVEVGTVKEHQ  169
            +FV GIP+   E  L EYF+  G++   V +  A      +++  RGFGF+      E +
Sbjct  115  IFVGGIPHNCGETELREYFKKFGVVTEVVMIYDA------EKQRPRGFGFITF----EDE  164

Query  170  QLIRMSKNKKIEFDLKPAKWDIERA  194
            Q +  + N     D+   K +++RA
Sbjct  165  QSVDQAVNMHFH-DIMGKKVEVKRA  188


>ref|XP_005302410.1| PREDICTED: DAZ-associated protein 1 isoform X6 [Chrysemys picta 
bellii]
Length=348

 Score = 40.4 bits (93),  Expect = 1.2, Method: Compositional matrix adjust.
 Identities = 25/85 (29%), Positives = 42/85 (49%), Gaps = 11/85 (13%)

Query  110  LFVAGIPNVAREQHLTEYFEDLGIIVRDVFVPSAFNQNGQKRKHNRGFGFVEVGTVKEHQ  169
            +FV GIP+   E  L EYF+  G++   V +  A      +++  RGFGF+      E +
Sbjct  116  IFVGGIPHNCGETELREYFKKFGVVTEVVMIYDA------EKQRPRGFGFITF----EDE  165

Query  170  QLIRMSKNKKIEFDLKPAKWDIERA  194
            Q +  + N     D+   K +++RA
Sbjct  166  QSVDQAVNMHFH-DIMGKKVEVKRA  189


>ref|XP_004866011.1| PREDICTED: DAZ-associated protein 1 isoform X3 [Heterocephalus 
glaber]
Length=406

 Score = 40.4 bits (93),  Expect = 1.2, Method: Compositional matrix adjust.
 Identities = 25/85 (29%), Positives = 42/85 (49%), Gaps = 11/85 (13%)

Query  110  LFVAGIPNVAREQHLTEYFEDLGIIVRDVFVPSAFNQNGQKRKHNRGFGFVEVGTVKEHQ  169
            +FV GIP+   E  L EYF+  G++   V +  A      +++  RGFGF+      E +
Sbjct  114  IFVGGIPHNCGETELREYFKKFGVVTEVVMIYDA------EKQRPRGFGFITF----EDE  163

Query  170  QLIRMSKNKKIEFDLKPAKWDIERA  194
            Q +  + N     D+   K +++RA
Sbjct  164  QSVDQAVNMHFH-DIMGKKVEVKRA  187


>ref|XP_010079059.1| PREDICTED: heterogeneous nuclear ribonucleoproteins A2/B1-like, 
partial [Pterocles gutturalis]
Length=160

 Score = 39.3 bits (90),  Expect = 1.2, Method: Compositional matrix adjust.
 Identities = 26/72 (36%), Positives = 36/72 (50%), Gaps = 6/72 (8%)

Query  89   PLKSTALLDTDTNGSSRVDQTLFVAGIPNVAREQHLTEYFEDLGIIVRDVFVPSAFNQNG  148
            P ++ A  ++   G+    + LFV GI     E HL +YFE+ G I  D        Q+G
Sbjct  83   PKRAVAREESGKPGAHVTVKKLFVGGIKEDTEEHHLRDYFEEYGKI--DTIEIITDRQSG  140

Query  149  QKRKHNRGFGFV  160
            +K    RGFGFV
Sbjct  141  KK----RGFGFV  148


>ref|XP_012601176.1| PREDICTED: DAZ-associated protein 1 isoform X2 [Microcebus murinus]
Length=409

 Score = 40.4 bits (93),  Expect = 1.2, Method: Compositional matrix adjust.
 Identities = 25/85 (29%), Positives = 42/85 (49%), Gaps = 11/85 (13%)

Query  110  LFVAGIPNVAREQHLTEYFEDLGIIVRDVFVPSAFNQNGQKRKHNRGFGFVEVGTVKEHQ  169
            +FV GIP+   E  L EYF+  G++   V +  A      +++  RGFGF+      E +
Sbjct  117  IFVGGIPHNCGETELREYFKKFGVVTEVVMIYDA------EKQRPRGFGFITF----EDE  166

Query  170  QLIRMSKNKKIEFDLKPAKWDIERA  194
            Q +  + N     D+   K +++RA
Sbjct  167  QSVDQAVNMHFH-DIMGKKVEVKRA  190


>ref|XP_005406028.2| PREDICTED: DAZ-associated protein 1 isoform X2 [Chinchilla lanigera]
Length=406

 Score = 40.4 bits (93),  Expect = 1.2, Method: Compositional matrix adjust.
 Identities = 25/85 (29%), Positives = 42/85 (49%), Gaps = 11/85 (13%)

Query  110  LFVAGIPNVAREQHLTEYFEDLGIIVRDVFVPSAFNQNGQKRKHNRGFGFVEVGTVKEHQ  169
            +FV GIP+   E  L EYF+  G++   V +  A      +++  RGFGF+      E +
Sbjct  114  IFVGGIPHNCGETELREYFKKFGVVTEVVMIYDA------EKQRPRGFGFITF----EDE  163

Query  170  QLIRMSKNKKIEFDLKPAKWDIERA  194
            Q +  + N     D+   K +++RA
Sbjct  164  QSVDQAVNMHFH-DIMGKKVEVKRA  187


>ref|XP_012500916.1| PREDICTED: DAZ-associated protein 1 isoform X3 [Propithecus coquereli]
Length=406

 Score = 40.4 bits (93),  Expect = 1.2, Method: Compositional matrix adjust.
 Identities = 25/85 (29%), Positives = 42/85 (49%), Gaps = 11/85 (13%)

Query  110  LFVAGIPNVAREQHLTEYFEDLGIIVRDVFVPSAFNQNGQKRKHNRGFGFVEVGTVKEHQ  169
            +FV GIP+   E  L EYF+  G++   V +  A      +++  RGFGF+      E +
Sbjct  114  IFVGGIPHNCGETELREYFKKFGVVTEVVMIYDA------EKQRPRGFGFITF----EDE  163

Query  170  QLIRMSKNKKIEFDLKPAKWDIERA  194
            Q +  + N     D+   K +++RA
Sbjct  164  QSVDQAVNMHFH-DIMGKKVEVKRA  187


>ref|XP_011228551.1| PREDICTED: DAZ-associated protein 1, partial [Ailuropoda melanoleuca]
Length=435

 Score = 40.4 bits (93),  Expect = 1.2, Method: Compositional matrix adjust.
 Identities = 23/76 (30%), Positives = 36/76 (47%), Gaps = 10/76 (13%)

Query  110  LFVAGIPNVAREQHLTEYFEDLGIIVRDVFVPSAFNQNGQKRKHNRGFGFVEVGTVKEHQ  169
            +FV GIP+   E  L EYF+  G++   V +  A      +++  RGFGF+     +   
Sbjct  144  IFVGGIPHNCGETELREYFKKFGVVTEVVMIYDA------EKQRPRGFGFITFEDEQSVD  197

Query  170  QLIRMS----KNKKIE  181
            Q + M       KK+E
Sbjct  198  QAVNMHFHDIMGKKVE  213


>ref|XP_003788744.1| PREDICTED: DAZ-associated protein 1 isoform X1 [Otolemur garnettii]
Length=407

 Score = 40.4 bits (93),  Expect = 1.2, Method: Compositional matrix adjust.
 Identities = 25/85 (29%), Positives = 42/85 (49%), Gaps = 11/85 (13%)

Query  110  LFVAGIPNVAREQHLTEYFEDLGIIVRDVFVPSAFNQNGQKRKHNRGFGFVEVGTVKEHQ  169
            +FV GIP+   E  L EYF+  G++   V +  A      +++  RGFGF+      E +
Sbjct  115  IFVGGIPHNCGETELREYFKKFGVVTEVVMIYDA------EKQRPRGFGFITF----EDE  164

Query  170  QLIRMSKNKKIEFDLKPAKWDIERA  194
            Q +  + N     D+   K +++RA
Sbjct  165  QSVDQAVNMHFH-DIMGKKVEVKRA  188


>gb|AAF81071.1|AF225910_1 DAZ-associated protein 1 [Mus musculus]
Length=405

 Score = 40.4 bits (93),  Expect = 1.2, Method: Compositional matrix adjust.
 Identities = 25/85 (29%), Positives = 42/85 (49%), Gaps = 11/85 (13%)

Query  110  LFVAGIPNVAREQHLTEYFEDLGIIVRDVFVPSAFNQNGQKRKHNRGFGFVEVGTVKEHQ  169
            +FV GIP+   E  L EYF+  G++   V +  A      +++  RGFGF+      E +
Sbjct  114  IFVGGIPHNCGETELREYFKKFGVVTEVVMIYDA------EKQRPRGFGFITF----EDE  163

Query  170  QLIRMSKNKKIEFDLKPAKWDIERA  194
            Q +  + N     D+   K +++RA
Sbjct  164  QSVDQAVNMHFH-DIMGKKVEVKRA  187


>ref|XP_007641385.1| PREDICTED: DAZ-associated protein 1 isoform X1 [Cricetulus griseus]
Length=406

 Score = 40.4 bits (93),  Expect = 1.2, Method: Compositional matrix adjust.
 Identities = 25/85 (29%), Positives = 42/85 (49%), Gaps = 11/85 (13%)

Query  110  LFVAGIPNVAREQHLTEYFEDLGIIVRDVFVPSAFNQNGQKRKHNRGFGFVEVGTVKEHQ  169
            +FV GIP+   E  L EYF+  G++   V +  A      +++  RGFGF+      E +
Sbjct  115  IFVGGIPHNCGETELREYFKKFGVVTEVVMIYDA------EKQRPRGFGFITF----EDE  164

Query  170  QLIRMSKNKKIEFDLKPAKWDIERA  194
            Q +  + N     D+   K +++RA
Sbjct  165  QSVDQAVNMHFH-DIMGKKVEVKRA  188


>ref|XP_012601177.1| PREDICTED: DAZ-associated protein 1 isoform X3 [Microcebus murinus]
Length=408

 Score = 40.4 bits (93),  Expect = 1.2, Method: Compositional matrix adjust.
 Identities = 25/85 (29%), Positives = 42/85 (49%), Gaps = 11/85 (13%)

Query  110  LFVAGIPNVAREQHLTEYFEDLGIIVRDVFVPSAFNQNGQKRKHNRGFGFVEVGTVKEHQ  169
            +FV GIP+   E  L EYF+  G++   V +  A      +++  RGFGF+      E +
Sbjct  117  IFVGGIPHNCGETELREYFKKFGVVTEVVMIYDA------EKQRPRGFGFITF----EDE  166

Query  170  QLIRMSKNKKIEFDLKPAKWDIERA  194
            Q +  + N     D+   K +++RA
Sbjct  167  QSVDQAVNMHFH-DIMGKKVEVKRA  190


>ref|XP_005611795.1| PREDICTED: DAZ-associated protein 1 [Equus caballus]
Length=621

 Score = 40.8 bits (94),  Expect = 1.2, Method: Compositional matrix adjust.
 Identities = 23/76 (30%), Positives = 36/76 (47%), Gaps = 10/76 (13%)

Query  110  LFVAGIPNVAREQHLTEYFEDLGIIVRDVFVPSAFNQNGQKRKHNRGFGFVEVGTVKEHQ  169
            +FV GIP+   E  L EYF+  G++   V +  A      +++  RGFGF+     +   
Sbjct  330  IFVGGIPHNCGETELREYFKKFGVVTEVVMIYDA------EKQRPRGFGFITFEDEQSVD  383

Query  170  QLIRMS----KNKKIE  181
            Q + M       KK+E
Sbjct  384  QAVNMHFHDIMGKKVE  399


>ref|XP_005587449.1| PREDICTED: DAZ-associated protein 1 isoform X1 [Macaca fascicularis]
Length=418

 Score = 40.4 bits (93),  Expect = 1.2, Method: Compositional matrix adjust.
 Identities = 25/85 (29%), Positives = 42/85 (49%), Gaps = 11/85 (13%)

Query  110  LFVAGIPNVAREQHLTEYFEDLGIIVRDVFVPSAFNQNGQKRKHNRGFGFVEVGTVKEHQ  169
            +FV GIP+   E  L EYF+  G++   V +  A      +++  RGFGF+      E +
Sbjct  126  IFVGGIPHNCGETELREYFKKFGVVTEVVMIYDA------EKQRPRGFGFITF----EDE  175

Query  170  QLIRMSKNKKIEFDLKPAKWDIERA  194
            Q +  + N     D+   K +++RA
Sbjct  176  QSVDQAVNMHFH-DIMGKKVEVKRA  199


>gb|EPQ08082.1| DAZ-associated protein 1 [Myotis brandtii]
Length=441

 Score = 40.4 bits (93),  Expect = 1.2, Method: Compositional matrix adjust.
 Identities = 25/85 (29%), Positives = 42/85 (49%), Gaps = 11/85 (13%)

Query  110  LFVAGIPNVAREQHLTEYFEDLGIIVRDVFVPSAFNQNGQKRKHNRGFGFVEVGTVKEHQ  169
            +FV GIP+   E  L EYF+  G++   V +  A      +++  RGFGF+      E +
Sbjct  150  IFVGGIPHNCGETELREYFKKFGVVTEVVMIYDA------EKQRPRGFGFITF----EDE  199

Query  170  QLIRMSKNKKIEFDLKPAKWDIERA  194
            Q +  + N     D+   K +++RA
Sbjct  200  QSVDQAVNMHFH-DIMGKKVEVKRA  223


>gb|EHB02473.1| DAZ-associated protein 1 [Heterocephalus glaber]
Length=404

 Score = 40.4 bits (93),  Expect = 1.2, Method: Compositional matrix adjust.
 Identities = 25/85 (29%), Positives = 42/85 (49%), Gaps = 11/85 (13%)

Query  110  LFVAGIPNVAREQHLTEYFEDLGIIVRDVFVPSAFNQNGQKRKHNRGFGFVEVGTVKEHQ  169
            +FV GIP+   E  L EYF+  G++   V +  A      +++  RGFGF+      E +
Sbjct  112  IFVGGIPHNCGETELREYFKKFGVVTEVVMIYDA------EKQRPRGFGFITF----EDE  161

Query  170  QLIRMSKNKKIEFDLKPAKWDIERA  194
            Q +  + N     D+   K +++RA
Sbjct  162  QSVDQAVNMHFH-DIMGKKVEVKRA  185


>ref|XP_012500913.1| PREDICTED: DAZ-associated protein 1 isoform X1 [Propithecus coquereli]
 ref|XP_012601178.1| PREDICTED: DAZ-associated protein 1 isoform X4 [Microcebus murinus]
Length=407

 Score = 40.4 bits (93),  Expect = 1.2, Method: Compositional matrix adjust.
 Identities = 25/85 (29%), Positives = 42/85 (49%), Gaps = 11/85 (13%)

Query  110  LFVAGIPNVAREQHLTEYFEDLGIIVRDVFVPSAFNQNGQKRKHNRGFGFVEVGTVKEHQ  169
            +FV GIP+   E  L EYF+  G++   V +  A      +++  RGFGF+      E +
Sbjct  115  IFVGGIPHNCGETELREYFKKFGVVTEVVMIYDA------EKQRPRGFGFITF----EDE  164

Query  170  QLIRMSKNKKIEFDLKPAKWDIERA  194
            Q +  + N     D+   K +++RA
Sbjct  165  QSVDQAVNMHFH-DIMGKKVEVKRA  188


>ref|XP_006050585.1| PREDICTED: DAZ-associated protein 1 isoform X2 [Bubalus bubalis]
Length=400

 Score = 40.4 bits (93),  Expect = 1.2, Method: Compositional matrix adjust.
 Identities = 25/85 (29%), Positives = 42/85 (49%), Gaps = 11/85 (13%)

Query  110  LFVAGIPNVAREQHLTEYFEDLGIIVRDVFVPSAFNQNGQKRKHNRGFGFVEVGTVKEHQ  169
            +FV GIP+   E  L EYF+  G++   V +  A      +++  RGFGF+      E +
Sbjct  109  IFVGGIPHNCGETELREYFKKFGVVTEVVMIYDA------EKQRPRGFGFITF----EDE  158

Query  170  QLIRMSKNKKIEFDLKPAKWDIERA  194
            Q +  + N     D+   K +++RA
Sbjct  159  QSVDQAVNMHFH-DIMGKKVEVKRA  182


>dbj|GAN04573.1| ribosome biogenesis protein Nop4 [Mucor ambiguus]
Length=805

 Score = 40.8 bits (94),  Expect = 1.2, Method: Compositional matrix adjust.
 Identities = 35/113 (31%), Positives = 55/113 (49%), Gaps = 10/113 (9%)

Query  109  TLFVAGIPNVAREQHLTEYFEDLGIIVRDVFVP---SAFNQNGQKRKHNRGFGFVEVGTV  165
            TLFV G+P  A  + L ++F ++G I R  FV    S     GQ  K N+GFG+V     
Sbjct  29   TLFVRGLPFEATSKDLEDFFGEIGPI-RKCFVVTDRSVAPIEGQAPK-NKGFGYVHYALE  86

Query  166  KEHQQLIRMSKN-----KKIEFDLKPAKWDIERARSYGTPSPSIAPKIESKPK  213
            ++ Q  I   KN     +K++ +L   K +  R  +   P+P      +++PK
Sbjct  87   EDAQSAITKLKNVKFQGRKLKIELAKRKSETVREDTKKQPAPVATAVADAEPK  139


>ref|XP_012815270.1| PREDICTED: DAZ-associated protein 1 isoform X2 [Xenopus (Silurana) 
tropicalis]
Length=410

 Score = 40.4 bits (93),  Expect = 1.2, Method: Compositional matrix adjust.
 Identities = 25/85 (29%), Positives = 42/85 (49%), Gaps = 11/85 (13%)

Query  110  LFVAGIPNVAREQHLTEYFEDLGIIVRDVFVPSAFNQNGQKRKHNRGFGFVEVGTVKEHQ  169
            +FV GIP+   E  L EYF+  G++   V +  A      +++  RGFGF+      E +
Sbjct  115  IFVGGIPHNCGETELREYFKRFGVVTEVVMIYDA------EKQRPRGFGFITF----EDE  164

Query  170  QLIRMSKNKKIEFDLKPAKWDIERA  194
            Q +  + N     D+   K +++RA
Sbjct  165  QSVDQAVNMHFH-DIMGKKVEVKRA  188


>gb|ELR60208.1| DAZ-associated protein 1, partial [Bos mutus]
Length=386

 Score = 40.4 bits (93),  Expect = 1.2, Method: Compositional matrix adjust.
 Identities = 23/76 (30%), Positives = 36/76 (47%), Gaps = 10/76 (13%)

Query  110  LFVAGIPNVAREQHLTEYFEDLGIIVRDVFVPSAFNQNGQKRKHNRGFGFVEVGTVKEHQ  169
            +FV GIP+   E  L EYF+  G++   V +  A      +++  RGFGF+     +   
Sbjct  105  IFVGGIPHNCGETELREYFKKFGVVTEVVMIYDA------EKQRPRGFGFITFEDEQSVD  158

Query  170  QLIRMS----KNKKIE  181
            Q + M       KK+E
Sbjct  159  QAVNMHFHDIMGKKVE  174


>ref|XP_004462795.1| PREDICTED: DAZ-associated protein 1 isoform X1 [Dasypus novemcinctus]
Length=366

 Score = 40.4 bits (93),  Expect = 1.2, Method: Compositional matrix adjust.
 Identities = 23/76 (30%), Positives = 36/76 (47%), Gaps = 10/76 (13%)

Query  110  LFVAGIPNVAREQHLTEYFEDLGIIVRDVFVPSAFNQNGQKRKHNRGFGFVEVGTVKEHQ  169
            +FV GIP+   E  L EYF+  G++   V +  A      +++  RGFGF+     +   
Sbjct  74   IFVGGIPHNCGETELREYFKKFGVVTEVVMIYDA------EKQRPRGFGFITFEDEQSVD  127

Query  170  QLIRMS----KNKKIE  181
            Q + M       KK+E
Sbjct  128  QAVNMHFHDIMGKKVE  143


>dbj|BAE31119.1| unnamed protein product [Mus musculus]
 dbj|BAE31140.1| unnamed protein product [Mus musculus]
Length=405

 Score = 40.4 bits (93),  Expect = 1.2, Method: Compositional matrix adjust.
 Identities = 23/76 (30%), Positives = 36/76 (47%), Gaps = 10/76 (13%)

Query  110  LFVAGIPNVAREQHLTEYFEDLGIIVRDVFVPSAFNQNGQKRKHNRGFGFVEVGTVKEHQ  169
            +FV GIP+   E  L EYF+  G++   V +  A      +++  RGFGF+     +   
Sbjct  114  IFVGGIPHNCGETELREYFKKFGVVTEVVMIYDA------EKQRPRGFGFITFEDEQSVD  167

Query  170  QLIRMS----KNKKIE  181
            Q + M       KK+E
Sbjct  168  QAVNMHFRDIMGKKVE  183


>ref|XP_004632736.1| PREDICTED: DAZ-associated protein 1 isoform X3 [Octodon degus]
Length=406

 Score = 40.4 bits (93),  Expect = 1.2, Method: Compositional matrix adjust.
 Identities = 25/85 (29%), Positives = 42/85 (49%), Gaps = 11/85 (13%)

Query  110  LFVAGIPNVAREQHLTEYFEDLGIIVRDVFVPSAFNQNGQKRKHNRGFGFVEVGTVKEHQ  169
            +FV GIP+   E  L EYF+  G++   V +  A      +++  RGFGF+      E +
Sbjct  114  IFVGGIPHNCGETELREYFKKFGVVTEVVMIYDA------EKQRPRGFGFITF----EDE  163

Query  170  QLIRMSKNKKIEFDLKPAKWDIERA  194
            Q +  + N     D+   K +++RA
Sbjct  164  QSVDQAVNMHFH-DIMGKKVEVKRA  187


>ref|XP_004866009.1| PREDICTED: DAZ-associated protein 1 isoform X1 [Heterocephalus 
glaber]
Length=407

 Score = 40.4 bits (93),  Expect = 1.2, Method: Compositional matrix adjust.
 Identities = 25/85 (29%), Positives = 42/85 (49%), Gaps = 11/85 (13%)

Query  110  LFVAGIPNVAREQHLTEYFEDLGIIVRDVFVPSAFNQNGQKRKHNRGFGFVEVGTVKEHQ  169
            +FV GIP+   E  L EYF+  G++   V +  A      +++  RGFGF+      E +
Sbjct  115  IFVGGIPHNCGETELREYFKKFGVVTEVVMIYDA------EKQRPRGFGFITF----EDE  164

Query  170  QLIRMSKNKKIEFDLKPAKWDIERA  194
            Q +  + N     D+   K +++RA
Sbjct  165  QSVDQAVNMHFH-DIMGKKVEVKRA  188


>ref|XP_012601175.1| PREDICTED: DAZ-associated protein 1 isoform X1 [Microcebus murinus]
Length=409

 Score = 40.4 bits (93),  Expect = 1.2, Method: Compositional matrix adjust.
 Identities = 25/85 (29%), Positives = 42/85 (49%), Gaps = 11/85 (13%)

Query  110  LFVAGIPNVAREQHLTEYFEDLGIIVRDVFVPSAFNQNGQKRKHNRGFGFVEVGTVKEHQ  169
            +FV GIP+   E  L EYF+  G++   V +  A      +++  RGFGF+      E +
Sbjct  118  IFVGGIPHNCGETELREYFKKFGVVTEVVMIYDA------EKQRPRGFGFITF----EDE  167

Query  170  QLIRMSKNKKIEFDLKPAKWDIERA  194
            Q +  + N     D+   K +++RA
Sbjct  168  QSVDQAVNMHFH-DIMGKKVEVKRA  191


>ref|XP_011286478.1| PREDICTED: DAZ-associated protein 1 isoform X1 [Felis catus]
Length=423

 Score = 40.4 bits (93),  Expect = 1.2, Method: Compositional matrix adjust.
 Identities = 25/85 (29%), Positives = 42/85 (49%), Gaps = 11/85 (13%)

Query  110  LFVAGIPNVAREQHLTEYFEDLGIIVRDVFVPSAFNQNGQKRKHNRGFGFVEVGTVKEHQ  169
            +FV GIP+   E  L EYF+  G++   V +  A      +++  RGFGF+      E +
Sbjct  132  IFVGGIPHNCGETELREYFKKFGVVTEVVMIYDA------EKQRPRGFGFITF----EDE  181

Query  170  QLIRMSKNKKIEFDLKPAKWDIERA  194
            Q +  + N     D+   K +++RA
Sbjct  182  QSVDQAVNMHFH-DIMGKKVEVKRA  205


>ref|NP_001020913.1| DAZ-associated protein 1 [Rattus norvegicus]
 ref|NP_573451.2| DAZ-associated protein 1 isoform a [Mus musculus]
 ref|XP_005083371.1| PREDICTED: DAZ-associated protein 1 isoform X4 [Mesocricetus 
auratus]
 ref|XP_005359124.1| PREDICTED: DAZ-associated protein 1 isoform X4 [Microtus ochrogaster]
 gb|AAH98930.1| DAZ associated protein 1 [Rattus norvegicus]
 gb|EDL31575.1| DAZ associated protein 1, isoform CRA_d [Mus musculus]
 gb|EDL89301.1| DAZ associated protein 1, isoform CRA_a [Rattus norvegicus]
Length=405

 Score = 40.4 bits (93),  Expect = 1.2, Method: Compositional matrix adjust.
 Identities = 25/85 (29%), Positives = 42/85 (49%), Gaps = 11/85 (13%)

Query  110  LFVAGIPNVAREQHLTEYFEDLGIIVRDVFVPSAFNQNGQKRKHNRGFGFVEVGTVKEHQ  169
            +FV GIP+   E  L EYF+  G++   V +  A      +++  RGFGF+      E +
Sbjct  114  IFVGGIPHNCGETELREYFKKFGVVTEVVMIYDA------EKQRPRGFGFITF----EDE  163

Query  170  QLIRMSKNKKIEFDLKPAKWDIERA  194
            Q +  + N     D+   K +++RA
Sbjct  164  QSVDQAVNMHFH-DIMGKKVEVKRA  187


>ref|NP_001116077.1| DAZ-associated protein 1 isoform c [Mus musculus]
 ref|XP_005083370.1| PREDICTED: DAZ-associated protein 1 isoform X3 [Mesocricetus 
auratus]
 ref|XP_005359123.1| PREDICTED: DAZ-associated protein 1 isoform X3 [Microtus ochrogaster]
 dbj|BAE28294.1| unnamed protein product [Mus musculus]
 gb|EDL31573.1| DAZ associated protein 1, isoform CRA_b [Mus musculus]
Length=406

 Score = 40.4 bits (93),  Expect = 1.2, Method: Compositional matrix adjust.
 Identities = 25/85 (29%), Positives = 42/85 (49%), Gaps = 11/85 (13%)

Query  110  LFVAGIPNVAREQHLTEYFEDLGIIVRDVFVPSAFNQNGQKRKHNRGFGFVEVGTVKEHQ  169
            +FV GIP+   E  L EYF+  G++   V +  A      +++  RGFGF+      E +
Sbjct  114  IFVGGIPHNCGETELREYFKKFGVVTEVVMIYDA------EKQRPRGFGFITF----EDE  163

Query  170  QLIRMSKNKKIEFDLKPAKWDIERA  194
            Q +  + N     D+   K +++RA
Sbjct  164  QSVDQAVNMHFH-DIMGKKVEVKRA  187


>ref|XP_008148910.1| PREDICTED: DAZ-associated protein 1 [Eptesicus fuscus]
Length=407

 Score = 40.4 bits (93),  Expect = 1.3, Method: Compositional matrix adjust.
 Identities = 25/85 (29%), Positives = 42/85 (49%), Gaps = 11/85 (13%)

Query  110  LFVAGIPNVAREQHLTEYFEDLGIIVRDVFVPSAFNQNGQKRKHNRGFGFVEVGTVKEHQ  169
            +FV GIP+   E  L EYF+  G++   V +  A      +++  RGFGF+      E +
Sbjct  115  IFVGGIPHNCGETELREYFKKFGVVTEVVMIYDA------EKQRPRGFGFITF----EDE  164

Query  170  QLIRMSKNKKIEFDLKPAKWDIERA  194
            Q +  + N     D+   K +++RA
Sbjct  165  QSVDQAVNMHFH-DIMGKKVEVKRA  188


>gb|ETN57998.1| heterogeneous nuclear ribonucleoprotein [Anopheles darlingi]
Length=369

 Score = 40.4 bits (93),  Expect = 1.3, Method: Compositional matrix adjust.
 Identities = 31/92 (34%), Positives = 47/92 (51%), Gaps = 12/92 (13%)

Query  110  LFVAGIPNVAREQHLTEYFEDLGIIVRDVFVPSAFNQNGQKRKHNRGFGFVEVGTVKEHQ  169
            LFV G+ +   E+HL EYF   G ++    V      NG+K    RGFGFVE        
Sbjct  122  LFVGGLRDDFDEEHLREYFSKYGNVISACIVTDK--DNGKK----RGFGFVEFDDYDPVD  175

Query  170  QLIRMSKNKKIE---FDLKPA--KWDIERARS  196
            ++I + K+  I+    D+K A  K D++R ++
Sbjct  176  KII-LQKSHTIQNKLLDVKKALPKQDMDRYKN  206


>ref|XP_012394575.1| PREDICTED: DAZ-associated protein 1 isoform X1 [Orcinus orca]
Length=463

 Score = 40.4 bits (93),  Expect = 1.3, Method: Compositional matrix adjust.
 Identities = 23/76 (30%), Positives = 36/76 (47%), Gaps = 10/76 (13%)

Query  110  LFVAGIPNVAREQHLTEYFEDLGIIVRDVFVPSAFNQNGQKRKHNRGFGFVEVGTVKEHQ  169
            +FV GIP+   E  L EYF+  G++   V +  A      +++  RGFGF+     +   
Sbjct  172  IFVGGIPHNCGETELREYFKKFGVVTEVVMIYDA------EKQRPRGFGFITFEDEQSVD  225

Query  170  QLIRMS----KNKKIE  181
            Q + M       KK+E
Sbjct  226  QAVNMHFHDIMGKKVE  241


>ref|XP_010600162.1| PREDICTED: DAZ-associated protein 1 isoform X3 [Loxodonta africana]
Length=366

 Score = 40.4 bits (93),  Expect = 1.3, Method: Compositional matrix adjust.
 Identities = 23/76 (30%), Positives = 36/76 (47%), Gaps = 10/76 (13%)

Query  110  LFVAGIPNVAREQHLTEYFEDLGIIVRDVFVPSAFNQNGQKRKHNRGFGFVEVGTVKEHQ  169
            +FV GIP+   E  L EYF+  G++   V +  A      +++  RGFGF+     +   
Sbjct  74   IFVGGIPHNCGETELREYFKKFGVVTEVVMIYDA------EKQRPRGFGFITFEDEQSVD  127

Query  170  QLIRMS----KNKKIE  181
            Q + M       KK+E
Sbjct  128  QAVNMHFHDIMGKKVE  143


>ref|XP_004866012.1| PREDICTED: DAZ-associated protein 1 isoform X4 [Heterocephalus 
glaber]
Length=405

 Score = 40.4 bits (93),  Expect = 1.3, Method: Compositional matrix adjust.
 Identities = 25/85 (29%), Positives = 42/85 (49%), Gaps = 11/85 (13%)

Query  110  LFVAGIPNVAREQHLTEYFEDLGIIVRDVFVPSAFNQNGQKRKHNRGFGFVEVGTVKEHQ  169
            +FV GIP+   E  L EYF+  G++   V +  A      +++  RGFGF+      E +
Sbjct  114  IFVGGIPHNCGETELREYFKKFGVVTEVVMIYDA------EKQRPRGFGFITF----EDE  163

Query  170  QLIRMSKNKKIEFDLKPAKWDIERA  194
            Q +  + N     D+   K +++RA
Sbjct  164  QSVDQAVNMHFH-DIMGKKVEVKRA  187


>ref|XP_004654942.1| PREDICTED: DAZ-associated protein 1 isoform X4 [Jaculus jaculus]
Length=405

 Score = 40.4 bits (93),  Expect = 1.3, Method: Compositional matrix adjust.
 Identities = 25/85 (29%), Positives = 42/85 (49%), Gaps = 11/85 (13%)

Query  110  LFVAGIPNVAREQHLTEYFEDLGIIVRDVFVPSAFNQNGQKRKHNRGFGFVEVGTVKEHQ  169
            +FV GIP+   E  L EYF+  G++   V +  A      +++  RGFGF+      E +
Sbjct  114  IFVGGIPHNCGETELREYFKKFGVVTEVVMIYDA------EKQRPRGFGFITF----EDE  163

Query  170  QLIRMSKNKKIEFDLKPAKWDIERA  194
            Q +  + N     D+   K +++RA
Sbjct  164  QSVDQAVNMHFH-DIMGKKVEVKRA  187


>ref|XP_012500914.1| PREDICTED: DAZ-associated protein 1 isoform X2 [Propithecus coquereli]
Length=406

 Score = 40.4 bits (93),  Expect = 1.3, Method: Compositional matrix adjust.
 Identities = 25/85 (29%), Positives = 42/85 (49%), Gaps = 11/85 (13%)

Query  110  LFVAGIPNVAREQHLTEYFEDLGIIVRDVFVPSAFNQNGQKRKHNRGFGFVEVGTVKEHQ  169
            +FV GIP+   E  L EYF+  G++   V +  A      +++  RGFGF+      E +
Sbjct  115  IFVGGIPHNCGETELREYFKKFGVVTEVVMIYDA------EKQRPRGFGFITF----EDE  164

Query  170  QLIRMSKNKKIEFDLKPAKWDIERA  194
            Q +  + N     D+   K +++RA
Sbjct  165  QSVDQAVNMHFH-DIMGKKVEVKRA  188


>ref|XP_004654940.1| PREDICTED: DAZ-associated protein 1 isoform X3 [Jaculus jaculus]
Length=406

 Score = 40.4 bits (93),  Expect = 1.3, Method: Compositional matrix adjust.
 Identities = 25/85 (29%), Positives = 42/85 (49%), Gaps = 11/85 (13%)

Query  110  LFVAGIPNVAREQHLTEYFEDLGIIVRDVFVPSAFNQNGQKRKHNRGFGFVEVGTVKEHQ  169
            +FV GIP+   E  L EYF+  G++   V +  A      +++  RGFGF+      E +
Sbjct  115  IFVGGIPHNCGETELREYFKKFGVVTEVVMIYDA------EKQRPRGFGFITF----EDE  164

Query  170  QLIRMSKNKKIEFDLKPAKWDIERA  194
            Q +  + N     D+   K +++RA
Sbjct  165  QSVDQAVNMHFH-DIMGKKVEVKRA  188


>ref|XP_004632734.1| PREDICTED: DAZ-associated protein 1 isoform X1 [Octodon degus]
Length=407

 Score = 40.4 bits (93),  Expect = 1.3, Method: Compositional matrix adjust.
 Identities = 25/85 (29%), Positives = 42/85 (49%), Gaps = 11/85 (13%)

Query  110  LFVAGIPNVAREQHLTEYFEDLGIIVRDVFVPSAFNQNGQKRKHNRGFGFVEVGTVKEHQ  169
            +FV GIP+   E  L EYF+  G++   V +  A      +++  RGFGF+      E +
Sbjct  115  IFVGGIPHNCGETELREYFKKFGVVTEVVMIYDA------EKQRPRGFGFITF----EDE  164

Query  170  QLIRMSKNKKIEFDLKPAKWDIERA  194
            Q +  + N     D+   K +++RA
Sbjct  165  QSVDQAVNMHFH-DIMGKKVEVKRA  188


>ref|XP_008835384.1| PREDICTED: DAZ-associated protein 1 isoform X2 [Nannospalax galili]
Length=402

 Score = 40.4 bits (93),  Expect = 1.3, Method: Compositional matrix adjust.
 Identities = 23/76 (30%), Positives = 36/76 (47%), Gaps = 10/76 (13%)

Query  110  LFVAGIPNVAREQHLTEYFEDLGIIVRDVFVPSAFNQNGQKRKHNRGFGFVEVGTVKEHQ  169
            +FV GIP+   E  L EYF+  G++   V +  A      +++  RGFGF+     +   
Sbjct  114  IFVGGIPHNCGETELREYFKKFGVVTEVVMIYDA------EKQRPRGFGFITFEDEQSVD  167

Query  170  QLIRMS----KNKKIE  181
            Q + M       KK+E
Sbjct  168  QAVNMHFHDIMGKKVE  183


>ref|XP_007130188.1| PREDICTED: DAZ-associated protein 1 [Physeter catodon]
Length=412

 Score = 40.4 bits (93),  Expect = 1.3, Method: Compositional matrix adjust.
 Identities = 23/76 (30%), Positives = 36/76 (47%), Gaps = 10/76 (13%)

Query  110  LFVAGIPNVAREQHLTEYFEDLGIIVRDVFVPSAFNQNGQKRKHNRGFGFVEVGTVKEHQ  169
            +FV GIP+   E  L EYF+  G++   V +  A      +++  RGFGF+     +   
Sbjct  121  IFVGGIPHNCGETELREYFKKFGVVTEVVMIYDA------EKQRPRGFGFITFEDEQSVD  174

Query  170  QLIRMS----KNKKIE  181
            Q + M       KK+E
Sbjct  175  QAVNMHFHDIMGKKVE  190


>ref|XP_005406030.2| PREDICTED: DAZ-associated protein 1 isoform X4 [Chinchilla lanigera]
Length=405

 Score = 40.4 bits (93),  Expect = 1.3, Method: Compositional matrix adjust.
 Identities = 25/85 (29%), Positives = 42/85 (49%), Gaps = 11/85 (13%)

Query  110  LFVAGIPNVAREQHLTEYFEDLGIIVRDVFVPSAFNQNGQKRKHNRGFGFVEVGTVKEHQ  169
            +FV GIP+   E  L EYF+  G++   V +  A      +++  RGFGF+      E +
Sbjct  114  IFVGGIPHNCGETELREYFKKFGVVTEVVMIYDA------EKQRPRGFGFITF----EDE  163

Query  170  QLIRMSKNKKIEFDLKPAKWDIERA  194
            Q +  + N     D+   K +++RA
Sbjct  164  QSVDQAVNMHFH-DIMGKKVEVKRA  187


>ref|XP_011286487.1| PREDICTED: DAZ-associated protein 1 isoform X3 [Felis catus]
Length=424

 Score = 40.4 bits (93),  Expect = 1.3, Method: Compositional matrix adjust.
 Identities = 23/76 (30%), Positives = 36/76 (47%), Gaps = 10/76 (13%)

Query  110  LFVAGIPNVAREQHLTEYFEDLGIIVRDVFVPSAFNQNGQKRKHNRGFGFVEVGTVKEHQ  169
            +FV GIP+   E  L EYF+  G++   V +  A      +++  RGFGF+     +   
Sbjct  133  IFVGGIPHNCGETELREYFKKFGVVTEVVMIYDA------EKQRPRGFGFITFEDEQSVD  186

Query  170  QLIRMS----KNKKIE  181
            Q + M       KK+E
Sbjct  187  QAVNMHFHDIMGKKVE  202


>ref|XP_005406027.2| PREDICTED: DAZ-associated protein 1 isoform X1 [Chinchilla lanigera]
Length=407

 Score = 40.4 bits (93),  Expect = 1.3, Method: Compositional matrix adjust.
 Identities = 25/85 (29%), Positives = 42/85 (49%), Gaps = 11/85 (13%)

Query  110  LFVAGIPNVAREQHLTEYFEDLGIIVRDVFVPSAFNQNGQKRKHNRGFGFVEVGTVKEHQ  169
            +FV GIP+   E  L EYF+  G++   V +  A      +++  RGFGF+      E +
Sbjct  115  IFVGGIPHNCGETELREYFKKFGVVTEVVMIYDA------EKQRPRGFGFITF----EDE  164

Query  170  QLIRMSKNKKIEFDLKPAKWDIERA  194
            Q +  + N     D+   K +++RA
Sbjct  165  QSVDQAVNMHFH-DIMGKKVEVKRA  188


>ref|XP_003422575.1| PREDICTED: DAZ-associated protein 1 isoform X1 [Loxodonta africana]
Length=407

 Score = 40.4 bits (93),  Expect = 1.3, Method: Compositional matrix adjust.
 Identities = 25/85 (29%), Positives = 42/85 (49%), Gaps = 11/85 (13%)

Query  110  LFVAGIPNVAREQHLTEYFEDLGIIVRDVFVPSAFNQNGQKRKHNRGFGFVEVGTVKEHQ  169
            +FV GIP+   E  L EYF+  G++   V +  A      +++  RGFGF+      E +
Sbjct  115  IFVGGIPHNCGETELREYFKKFGVVTEVVMIYDA------EKQRPRGFGFITF----EDE  164

Query  170  QLIRMSKNKKIEFDLKPAKWDIERA  194
            Q +  + N     D+   K +++RA
Sbjct  165  QSVDQAVNMHFH-DIMGKKVEVKRA  188


>ref|XP_012815264.1| PREDICTED: DAZ-associated protein 1 isoform X1 [Xenopus (Silurana) 
tropicalis]
Length=411

 Score = 40.4 bits (93),  Expect = 1.3, Method: Compositional matrix adjust.
 Identities = 25/85 (29%), Positives = 42/85 (49%), Gaps = 11/85 (13%)

Query  110  LFVAGIPNVAREQHLTEYFEDLGIIVRDVFVPSAFNQNGQKRKHNRGFGFVEVGTVKEHQ  169
            +FV GIP+   E  L EYF+  G++   V +  A      +++  RGFGF+      E +
Sbjct  116  IFVGGIPHNCGETELREYFKRFGVVTEVVMIYDA------EKQRPRGFGFITF----EDE  165

Query  170  QLIRMSKNKKIEFDLKPAKWDIERA  194
            Q +  + N     D+   K +++RA
Sbjct  166  QSVDQAVNMHFH-DIMGKKVEVKRA  189


Lambda      K        H        a         alpha
   0.318    0.131    0.366    0.792     4.96 

Gapped
Lambda      K        H        a         alpha    sigma
   0.267   0.0410    0.140     1.90     42.6     43.6 

Effective search space used: 1600171227146


  Database: nr
    Posted date:  Sep 23, 2015 12:05 AM
  Number of letters in database: 26,053,659,533
  Number of sequences in database:  71,551,133


Matrix: BLOSUM62
Gap Penalties: Existence: 11, Extension: 1
Neighboring words threshold: 11
Window for multiple hits: 40
```
