# Supplementary material for: Comparative genomics provides new insights into the diversity, physiology, and sexuality of the only industrially exploited tremellomycete: Phaffia rhodozyma
Source: BMC Genomics. 2016 Nov 9;17:901. doi: 10.1186/s12864-016-3244-7 (PMC5103461; doi:10.1186/s12864-016-3244-7)
Supplement: Additional file 6: — List of orphan genes with links to PFAM (related to Additional file 1: Table S1). (ZIP 1428 kb) [file 12864_2016_3244_MOESM6_ESM.zip › BLAST_HTML_FTR/G00542_P.html]

BLAST Search Results


```
BLASTP 2.2.27+


Reference:
Stephen F. Altschul, Thomas L. Madden, Alejandro A. Schäffer,
Jinghui Zhang, Zheng Zhang, Webb Miller, and David J. Lipman (1997),
"Gapped BLAST and PSI-BLAST: a new generation of protein database
search programs", Nucleic Acids Res. 25:3389-3402.


Reference for
composition-based statistics:
Alejandro A. Schäffer, L. Aravind, Thomas L. Madden, Sergei
Shavirin, John L. Spouge, Yuri I. Wolf, Eugene V. Koonin, and
Stephen F. Altschul (2001), "Improving the accuracy of PSI-BLAST
protein database searches with composition-based statistics and
other refinements", Nucleic Acids Res. 29:2994-3005.


Database: nr
           71,551,133 sequences; 26,053,659,533 total letters


Query= G00542_P

Length=1003
                                                                      Score     E
Sequences producing significant alignments:                          (Bits)  Value

emb|CED83857.1|  hypothetical protein [Xanthophyllomyces dendrorh...  1456    0.0  


 >emb|CED83857.1| hypothetical protein [Xanthophyllomyces dendrorhous]
Length=957

 Score = 1456 bits (3768),  Expect = 0.0, Method: Compositional matrix adjust.
 Identities = 948/979 (97%), Positives = 949/979 (97%), Gaps = 22/979 (2%)

Query  24    MTVHPTTDSSTTHPTIAYTPSIVPDTFSSSSSADSSLIVSNTSPVSAVMAPIHSSLHSPA  83
             MTVHPTTDSSTTHPTIAYTPSIVPDTFSSSSSADSSLIVSNTSPVSAVMAPIHSSLHSPA
Sbjct  1     MTVHPTTDSSTTHPTIAYTPSIVPDTFSSSSSADSSLIVSNTSPVSAVMAPIHSSLHSPA  60

Query  84    VSVGESHVRPSPFSLDSPIGTGGNLLSTLPPDSTLARELESGFDVWDGPPGHPFKLDGPV  143
             VSVGESHVRPSPFSLDSPIGTGGNLLSTLPPDSTLARELESGFDVWDGPPGHPFKLDGPV
Sbjct  61    VSVGESHVRPSPFSLDSPIGTGGNLLSTLPPDSTLARELESGFDVWDGPPGHPFKLDGPV  120

Query  144   SSLGGRSEKAIQSSGERSYQEEDRAHTPSLQRLDKPGGVTWSSSSRETSSNPKQFSSLST  203
             SSLGGRSEKAIQSSGERSYQEEDRAHTPSLQRLDKPGGVTWSSSSRETSSNPKQFSSLST
Sbjct  121   SSLGGRSEKAIQSSGERSYQEEDRAHTPSLQRLDKPGGVTWSSSSRETSSNPKQFSSLST  180

Query  204   FTPNPTAGPSYHPFQDPTLLLSAPAASSDFPASSVSSAAPSPPNVSTPYPVGVGVSQVEP  263
             FTPNPTAGPSYHPFQDPTLLLSAPAASSDFPASSVSSAAPSPPNVSTPYPVGVGVSQVEP
Sbjct  181   FTPNPTAGPSYHPFQDPTLLLSAPAASSDFPASSVSSAAPSPPNVSTPYPVGVGVSQVEP  240

Query  264   PFEARARPKPKPRMSVREKPTDSGGIILTPATENSTKSVGGSKDWSMGPPLVLPSSRAPS  323
             PFEARARPKPKPRMSVREKPTDSGGIILTPATENSTKSVGGSKDWSMGPPLVLPSSRAPS
Sbjct  241   PFEARARPKPKPRMSVREKPTDSGGIILTPATENSTKSVGGSKDWSMGPPLVLPSSRAPS  300

Query  324   GLTPEGIYHTPTLSPLSIRSVTSAESDKEITGQIEKPSRKMDKLPVKRKTRDLSDEEEGD  383
             GLTPEGIYHTPTLSPLSIRSVTSAESDKEITGQIEKPSRKMDKLPVKRKTRDLSDEEEGD
Sbjct  301   GLTPEGIYHTPTLSPLSIRSVTSAESDKEITGQIEKPSRKMDKLPVKRKTRDLSDEEEGD  360

Query  384   REEECEKRRLSEPRGRKHSASTKGNGSTKEERTKDRRRAPENTGDDESDPIGLSSSSKKS  443
             REEECEKRRLSEPRGRKHSASTKGNGSTKEERTKDRRRAPENTGDDESDPIGLSSSSKKS
Sbjct  361   REEECEKRRLSEPRGRKHSASTKGNGSTKEERTKDRRRAPENTGDDESDPIGLSSSSKKS  420

Query  444   LSGKMAEVIDLVASSEPGDIPQTTLKSKTKTKDVSKADLKAKSKINGKGKEVDRRSGIKS  503
             LSGKMAEVIDLVASSEPGDIPQTTLKSKTKTKDVSKADLKAKSKINGKGKEVDRRSGIKS
Sbjct  421   LSGKMAEVIDLVASSEPGDIPQTTLKSKTKTKDVSKADLKAKSKINGKGKEVDRRSGIKS  480

Query  504   ATATPPRSSSSSSGKAVRAKRPMMVPSSPTSAASEPEAELTPAPVSKPPTPQPQKAKTLA  563
             ATATPPRSSSSSSGKAVRAKRPMMVPSSPTSAASEPEAELTPAPVSKPPTPQPQKAKTLA
Sbjct  481   ATATPPRSSSSSSGKAVRAKRPMMVPSSPTSAASEPEAELTPAPVSKPPTPQPQKAKTLA  540

Query  564   EGGMSVPHLVDSQSPSLSPPPPLLNEISAEINPEFKGASASTTIVIKKSKKKGPPKEYAY  623
             EGGMSVPHLVDSQSPSLSPPPPLLNEISAEINPEFKGASASTTIVIKKSKKKGPPKEYAY
Sbjct  541   EGGMSVPHLVDSQSPSLSPPPPLLNEISAEINPEFKGASASTTIVIKKSKKKGPPKEYAY  600

Query  624   ETDEEEPTSRPVLAPASVWMDEHSTRRRGKPQVSYQEIPPDEIDERLMEIRSSSTGLGRD  683
             ETDEEEPTSRPVLAPASVWMDEHSTRRRGKPQVSYQEIPPDEIDERLMEIRSSSTGLGRD
Sbjct  601   ETDEEEPTSRPVLAPASVWMDEHSTRRRGKPQVSYQEIPPDEIDERLMEIRSSSTGLGRD  660

Query  684   MKRNGEGVLTDDENPAKRDKKGYDDEARLVDQANIESNKEVLATSIEEIQGAGEAQDPAG  743
             MKRNGEGVLTDDENPAKRDKKGYDDEARLVDQANIESNKEVLATSIEEIQGAGEAQDPAG
Sbjct  661   MKRNGEGVLTDDENPAKRDKKGYDDEARLVDQANIESNKEVLATSIEEIQGAGEAQDPAG  720

Query  744   EASAKNSKGLTAKKAPTKKGKVKGKGKGKASAKEIETEDQVVVEEEDMIEVEKEEEVEQI  803
             EASAKNSKGLTAKKAPTKKGKVKGKGKGKASAKEIETEDQVVVEEEDMIEVEKEEEVEQI
Sbjct  721   EASAKNSKGLTAKKAPTKKGKVKGKGKGKASAKEIETEDQVVVEEEDMIEVEKEEEVEQI  780

Query  804   EPDEEAQKEDVDEDEDEDEKKDSSASTPTQVPPPVPPPATTTATATKSSLKRSQGPALSS  863
             EPDEEAQKEDVDEDEDEDEKKDSSASTPTQVPPPVPPPATTTATATKSSLKRSQGPALSS
Sbjct  781   EPDEEAQKEDVDEDEDEDEKKDSSASTPTQVPPPVPPPATTTATATKSSLKRSQGPALSS  840

Query  864   SNSSLAAESPRVSIPRHRPPFKREGSAISGLSSPGPGKVQSGNEALRGTSLASIIQKHST  923
             SNSSLAAESPRVSIPRHRPPFKREGSAISGLSSPGPGKVQSGNEALRGTSLASIIQKHST
Sbjct  841   SNSSLAAESPRVSIPRHRPPFKREGSAISGLSSPGPGKVQSGNEALRGTSLASIIQKHST  900

Query  924   PLRSPSIQTSGLVRPSGLSRRHRIVPLHHNILPQPKKLPPPPVKKVAKKKGDLCEDDFSE  983
             PLRSPSIQTSGL +                             KKVAKKKGDLCEDDFSE
Sbjct  901   PLRSPSIQTSGLPKKLPPPPV----------------------KKVAKKKGDLCEDDFSE  938

Query  984   GEWEEMEKERLKRERDWCE  1002
             GEWEEMEKERLKRERDWCE
Sbjct  939   GEWEEMEKERLKRERDWCE  957


Lambda      K        H        a         alpha
   0.304    0.124    0.345    0.792     4.96 

Gapped
Lambda      K        H        a         alpha    sigma
   0.267   0.0410    0.140     1.90     42.6     43.6 

Effective search space used: 12237566635040


  Database: nr
    Posted date:  Sep 23, 2015 12:05 AM
  Number of letters in database: 26,053,659,533
  Number of sequences in database:  71,551,133


Matrix: BLOSUM62
Gap Penalties: Existence: 11, Extension: 1
Neighboring words threshold: 11
Window for multiple hits: 40
```
