# Supplementary material for: Comparative genomics provides new insights into the diversity, physiology, and sexuality of the only industrially exploited tremellomycete: Phaffia rhodozyma
Source: BMC Genomics. 2016 Nov 9;17:901. doi: 10.1186/s12864-016-3244-7 (PMC5103461; doi:10.1186/s12864-016-3244-7)
Supplement: Additional file 6: — List of orphan genes with links to PFAM (related to Additional file 1: Table S1). (ZIP 1428 kb) [file 12864_2016_3244_MOESM6_ESM.zip › BLAST_HTML_FTR/G03137_P.html]

BLAST Search Results


```
BLASTP 2.2.27+


Reference:
Stephen F. Altschul, Thomas L. Madden, Alejandro A. Schäffer,
Jinghui Zhang, Zheng Zhang, Webb Miller, and David J. Lipman (1997),
"Gapped BLAST and PSI-BLAST: a new generation of protein database
search programs", Nucleic Acids Res. 25:3389-3402.


Reference for
composition-based statistics:
Alejandro A. Schäffer, L. Aravind, Thomas L. Madden, Sergei
Shavirin, John L. Spouge, Yuri I. Wolf, Eugene V. Koonin, and
Stephen F. Altschul (2001), "Improving the accuracy of PSI-BLAST
protein database searches with composition-based statistics and
other refinements", Nucleic Acids Res. 29:2994-3005.


Database: nr
           71,551,133 sequences; 26,053,659,533 total letters


Query= G03137_P

Length=223
                                                                      Score     E
Sequences producing significant alignments:                          (Bits)  Value

emb|CDZ96690.1|  hypothetical protein [Xanthophyllomyces dendrorh...   451    5e-159
gb|KOF80723.1|  hypothetical protein OCBIM_22027544mg [Octopus bi...  42.7    0.19  
ref|XP_007911178.1|  hypothetical protein UCRPA7_391 [Togninia mi...  37.4    6.1   
ref|WP_046106863.1|  beta-galactosidase [Devosia geojensis] >gb|K...  38.1    6.5   


 >emb|CDZ96690.1| hypothetical protein [Xanthophyllomyces dendrorhous]
Length=222

 Score =  451 bits (1161),  Expect = 5e-159, Method: Compositional matrix adjust.
 Identities = 222/222 (100%), Positives = 222/222 (100%), Gaps = 0/222 (0%)

Query  1    MFPQTEADKHYAIVQAELMQLIPLGASAPSADHQSGSNGKPGSSLSSIRPSYAEVESTSP  60
            MFPQTEADKHYAIVQAELMQLIPLGASAPSADHQSGSNGKPGSSLSSIRPSYAEVESTSP
Sbjct  1    MFPQTEADKHYAIVQAELMQLIPLGASAPSADHQSGSNGKPGSSLSSIRPSYAEVESTSP  60

Query  61   FTPALTFSKKLEQGDAFNEEDFDESDYSDDGEDMPQTPWVPQAPLHSDRDFFLSFQPLAP  120
            FTPALTFSKKLEQGDAFNEEDFDESDYSDDGEDMPQTPWVPQAPLHSDRDFFLSFQPLAP
Sbjct  61   FTPALTFSKKLEQGDAFNEEDFDESDYSDDGEDMPQTPWVPQAPLHSDRDFFLSFQPLAP  120

Query  121  LSIPRPVSWASDFPSLNNQTFENNFLGLRLDLPHSITAEAQSSTQTRLSNRPRVSTVPVE  180
            LSIPRPVSWASDFPSLNNQTFENNFLGLRLDLPHSITAEAQSSTQTRLSNRPRVSTVPVE
Sbjct  121  LSIPRPVSWASDFPSLNNQTFENNFLGLRLDLPHSITAEAQSSTQTRLSNRPRVSTVPVE  180

Query  181  SNAIPERAPSHVYRSWSDLETTSQPDLKNNDSHKMDKHVRPS  222
            SNAIPERAPSHVYRSWSDLETTSQPDLKNNDSHKMDKHVRPS
Sbjct  181  SNAIPERAPSHVYRSWSDLETTSQPDLKNNDSHKMDKHVRPS  222


>gb|KOF80723.1| hypothetical protein OCBIM_22027544mg [Octopus bimaculoides]
Length=679

 Score = 42.7 bits (99),  Expect = 0.19, Method: Compositional matrix adjust.
 Identities = 41/130 (32%), Positives = 62/130 (48%), Gaps = 6/130 (5%)

Query  41   PGSSLSSIRPSYAEVESTSPFTPALTFSKKLEQGDAFNEEDFDESDYSDDGEDMPQTPWV  100
            P  S +SI P + E++STS F+ +LT +     G+A ++ D  +SDY       P TP  
Sbjct  546  PDRSSASISPCFLELKSTSNFSKSLTDTDPNLTGEA-DKHDNRKSDYHWLRFQEPITP--  602

Query  101  PQAPLHSDRDFFLSFQPLAPLSIPRPVSWASDFPSLNNQTFENNFLGLRLDLPHSITAEA  160
              AP   DRD  L+F PL P +  +        P + N  ++ N + +  D   S TA  
Sbjct  603  --APSSFDRD-SLTFDPLHPSNSSKGPRTEMQIPCMQNCDYDGNLIPVYGDQSASSTANG  659

Query  161  QSSTQTRLSN  170
             +S +  L N
Sbjct  660  CNSMKQNLCN  669


>ref|XP_007911178.1| hypothetical protein UCRPA7_391 [Togninia minima UCRPA7]
 gb|EOO04113.1| hypothetical protein UCRPA7_391 [Togninia minima UCRPA7]
Length=201

 Score = 37.4 bits (85),  Expect = 6.1, Method: Compositional matrix adjust.
 Identities = 19/65 (29%), Positives = 35/65 (54%), Gaps = 1/65 (2%)

Query  57   STSPFTPALTFSKKLEQGDAFNEEDFDESDYSDDGEDMPQTPWV-PQAPLHSDRDFFLSF  115
            S  P  P ++  +++E+G  F E+D   +D +   +D+   PW+ P A +  D D +L  
Sbjct  55   SLVPMRPQVSAERRMEEGRYFPEDDLQHNDSAQGAQDLTDKPWLWPVAEVLFDADDYLEA  114

Query  116  QPLAP  120
            +P +P
Sbjct  115  EPASP  119


>ref|WP_046106863.1| beta-galactosidase [Devosia geojensis]
 gb|KKB13479.1| beta-galactosidase [Devosia geojensis]
Length=678

 Score = 38.1 bits (87),  Expect = 6.5, Method: Compositional matrix adjust.
 Identities = 26/87 (30%), Positives = 40/87 (46%), Gaps = 7/87 (8%)

Query  9    KHYAIVQAELMQLIPLGASAPSADHQSGSNGKPGSSLSSIRPSYAEVESTSPFTPALTFS  68
            KH   ++ E  Q++ LG+     D    S      + SSI P+YA +E   PF   L  +
Sbjct  288  KHPDALEYECAQMVALGSKCLVGDQLHPSGAINADTYSSIAPAYARIEKLEPF---LEGA  344

Query  69   KKLEQGDAFNEEDFDESD----YSDDG  91
            +++ +    + E FD S      SDDG
Sbjct  345  RQVSEIAILSAEHFDASGDRNHVSDDG  371


Lambda      K        H        a         alpha
   0.313    0.128    0.381    0.792     4.96 

Gapped
Lambda      K        H        a         alpha    sigma
   0.267   0.0410    0.140     1.90     42.6     43.6 

Effective search space used: 1222942129344


  Database: nr
    Posted date:  Sep 23, 2015 12:05 AM
  Number of letters in database: 26,053,659,533
  Number of sequences in database:  71,551,133


Matrix: BLOSUM62
Gap Penalties: Existence: 11, Extension: 1
Neighboring words threshold: 11
Window for multiple hits: 40
```
