# Supplementary material for: Comparative genomics provides new insights into the diversity, physiology, and sexuality of the only industrially exploited tremellomycete: Phaffia rhodozyma
Source: BMC Genomics. 2016 Nov 9;17:901. doi: 10.1186/s12864-016-3244-7 (PMC5103461; doi:10.1186/s12864-016-3244-7)
Supplement: Additional file 6: — List of orphan genes with links to PFAM (related to Additional file 1: Table S1). (ZIP 1428 kb) [file 12864_2016_3244_MOESM6_ESM.zip › BLAST_HTML_FTR/G03747_P.html]

BLAST Search Results


```
BLASTP 2.2.27+


Reference:
Stephen F. Altschul, Thomas L. Madden, Alejandro A. Schäffer,
Jinghui Zhang, Zheng Zhang, Webb Miller, and David J. Lipman (1997),
"Gapped BLAST and PSI-BLAST: a new generation of protein database
search programs", Nucleic Acids Res. 25:3389-3402.


Reference for
composition-based statistics:
Alejandro A. Schäffer, L. Aravind, Thomas L. Madden, Sergei
Shavirin, John L. Spouge, Yuri I. Wolf, Eugene V. Koonin, and
Stephen F. Altschul (2001), "Improving the accuracy of PSI-BLAST
protein database searches with composition-based statistics and
other refinements", Nucleic Acids Res. 29:2994-3005.


Database: nr
           71,551,133 sequences; 26,053,659,533 total letters


Query= G03747_P

Length=641
                                                                      Score     E
Sequences producing significant alignments:                          (Bits)  Value

emb|CDZ98831.1|  zinc finger protein 740 [Xanthophyllomyces dendr...  1193    0.0  


 >emb|CDZ98831.1| zinc finger protein 740 [Xanthophyllomyces dendrorhous]
Length=656

 Score = 1193 bits (3087),  Expect = 0.0, Method: Compositional matrix adjust.
 Identities = 626/656 (95%), Positives = 628/656 (96%), Gaps = 16/656 (2%)

Query  1    MSFELCRRNFEQRLPGVAVILSSAFEPLSTMDGSTCRSDHSSPRYFPYSLPRPPRKPCID  60
            MSFELCRRNFEQRLPGVAVILSSAFEPLSTMDGSTCRSDHSSPRYFPYSLPRPPRKPCID
Sbjct  1    MSFELCRRNFEQRLPGVAVILSSAFEPLSTMDGSTCRSDHSSPRYFPYSLPRPPRKPCID  60

Query  61   LPPEQNPPRESSYSHCTGSFVSAGAQGFEPSGLRHQHGEFHRQSSSCYQVQPPWRPDVPG  120
            LPPEQNPPRESSYSHCTGSFVSAGAQGFEPSGLRHQHGEFHRQSSSCYQVQPPWRPDVPG
Sbjct  61   LPPEQNPPRESSYSHCTGSFVSAGAQGFEPSGLRHQHGEFHRQSSSCYQVQPPWRPDVPG  120

Query  121  PRLEVLNNIEPREGSQLKLHKQWPEPQQKQHADEMRVGLQRIEPHRSNDERLKTTLNDGT  180
            PRLEVLNNIEPREGSQLKLHKQWPEPQQKQHADEMRVGLQRIEPHRSNDERLKTTLNDGT
Sbjct  121  PRLEVLNNIEPREGSQLKLHKQWPEPQQKQHADEMRVGLQRIEPHRSNDERLKTTLNDGT  180

Query  181  DRSCLSYISLSKGSVPLFSNGPTPFRDALQTSIPATESTPWVHSQLPQQSPKYWQDSHPD  240
            DRSCLSYISLSKGSVPLFSNGPTPFRDALQTSIPATESTPWVHSQLPQQSPKYWQDSHP+
Sbjct  181  DRSCLSYISLSKGSVPLFSNGPTPFRDALQTSIPATESTPWVHSQLPQQSPKYWQDSHPN  240

Query  241  PHRYQPGSRWHFSGSINQTNRTAFDHSDTYPSPRQQLSAFPPSISPPYSAVRSALPTIGH  300
            PHRYQPGSRWHFSGSINQTNRTAFDHSDTYPSPRQQLSAFPPSISPPYSAVRSALPTIGH
Sbjct  241  PHRYQPGSRWHFSGSINQTNRTAFDHSDTYPSPRQQLSAFPPSISPPYSAVRSALPTIGH  300

Query  301  VPLTPHESTPDPLPEEKVTETYPSPPSTDRSFRQISSFAPLPLSLEDLVDPAPVKSDTVD  360
            VPLTPHESTPDPLPEEKVTETYPSPPSTDRSFRQISSFAPLPLSLEDLVDPAPVKSDTVD
Sbjct  301  VPLTPHESTPDPLPEEKVTETYPSPPSTDRSFRQISSFAPLPLSLEDLVDPAPVKSDTVD  360

Query  361  DDVRMSGQILVGLAKGRTPVLGLGVGSDCPPTKMSSVTVSADETLNEAEAAGERDESEDG  420
            DDVRMSGQILVGLAKGRTPVLGLGVGSDCPPTKMSSVTVSADETLNEAEAAGERDESEDG
Sbjct  361  DDVRMSGQILVGLAKGRTPVLGLGVGSDCPPTKMSSVTVSADETLNEAEAAGERDESEDG  420

Query  421  SESNDEIIPTTRTPSPMPVYRSAEATLTQLKRTKKRASTLPVSATSTATEPSFRRSWTRG  480
            SESNDEIIPTTRTPSPMPVYRSAEATLTQLKRTKKRASTLPVSATSTATEPSFRRSWTRG
Sbjct  421  SESNDEIIPTTRTPSPMPVYRSAEATLTQLKRTKKRASTLPVSATSTATEPSFRRSWTRG  480

Query  481  LPSASGSVSTPVTKTASSSQIPIEPTAVTSSGSVGTSSSGSVGTSDRSTKSVTPKNSKRR  540
            LPSASGSVSTPVTKTASSSQIPIEPTAVTSSGSVGTSSSGSVGTSDRSTKSVTPKNSKRR
Sbjct  481  LPSASGSVSTPVTKTASSSQIPIEPTAVTSSGSVGTSSSGSVGTSDRSTKSVTPKNSKRR  540

Query  541  TKKPKTKVSMENYDPEHPRPFGCDMCLERMTMCDT--------SASIVATSPI-------  585
            TKKPKTKVSMENYDPEHPRPFGCDMCLER    +         S       PI       
Sbjct  541  TKKPKTKVSMENYDPEHPRPFGCDMCLERFLRKNDYVRHQRIHSGDKPYVCPICSTGFMR  600

Query  586  -DALLRHIDHTSVCAAQAPSREEYVFGLGGLKRRKSFSISTASESSHARNHKKRKV  640
             DALLRHIDHTSVCAAQAPSREEYVFGLGGLKRRKSFSISTASESSHARNHKKRKV
Sbjct  601  NDALLRHIDHTSVCAAQAPSREEYVFGLGGLKRRKSFSISTASESSHARNHKKRKV  656


Lambda      K        H        a         alpha
   0.312    0.128    0.383    0.792     4.96 

Gapped
Lambda      K        H        a         alpha    sigma
   0.267   0.0410    0.140     1.90     42.6     43.6 

Effective search space used: 7172943719568


  Database: nr
    Posted date:  Sep 23, 2015 12:05 AM
  Number of letters in database: 26,053,659,533
  Number of sequences in database:  71,551,133


Matrix: BLOSUM62
Gap Penalties: Existence: 11, Extension: 1
Neighboring words threshold: 11
Window for multiple hits: 40
```
