# Supplementary material for: Comparative genomics provides new insights into the diversity, physiology, and sexuality of the only industrially exploited tremellomycete: Phaffia rhodozyma
Source: BMC Genomics. 2016 Nov 9;17:901. doi: 10.1186/s12864-016-3244-7 (PMC5103461; doi:10.1186/s12864-016-3244-7)
Supplement: Additional file 6: — List of orphan genes with links to PFAM (related to Additional file 1: Table S1). (ZIP 1428 kb) [file 12864_2016_3244_MOESM6_ESM.zip › BLAST_HTML_FTR/G00190_P.html]

BLAST Search Results


```
BLASTP 2.2.27+


Reference:
Stephen F. Altschul, Thomas L. Madden, Alejandro A. Schäffer,
Jinghui Zhang, Zheng Zhang, Webb Miller, and David J. Lipman (1997),
"Gapped BLAST and PSI-BLAST: a new generation of protein database
search programs", Nucleic Acids Res. 25:3389-3402.


Reference for
composition-based statistics:
Alejandro A. Schäffer, L. Aravind, Thomas L. Madden, Sergei
Shavirin, John L. Spouge, Yuri I. Wolf, Eugene V. Koonin, and
Stephen F. Altschul (2001), "Improving the accuracy of PSI-BLAST
protein database searches with composition-based statistics and
other refinements", Nucleic Acids Res. 29:2994-3005.


Database: nr
           71,551,133 sequences; 26,053,659,533 total letters


Query= G00190_P

Length=563
                                                                      Score     E
Sequences producing significant alignments:                          (Bits)  Value

emb|CED84040.1|  hypothetical protein [Xanthophyllomyces dendrorh...   797    0.0  
gb|EDL04856.1|  mCG49710 [Mus musculus]                               40.4    5.8  
emb|CCF53829.1|  related to SPT3-general transcriptional adaptor ...  39.7    8.5  


 >emb|CED84040.1| hypothetical protein [Xanthophyllomyces dendrorhous]
Length=397

 Score =  797 bits (2059),  Expect = 0.0, Method: Compositional matrix adjust.
 Identities = 396/397 (99%), Positives = 397/397 (100%), Gaps = 0/397 (0%)

Query  166  MMTINTTIDTPFALPPSIIHPLLLSAQTLYLDPSVLLQTLIEPFLQTIPLALTNTLAALS  225
            MMTINTTIDTPFALPPSIIHPLLLSAQTLYLDPSVLLQTLIEPFLQTIPLALTNTLAALS
Sbjct  1    MMTINTTIDTPFALPPSIIHPLLLSAQTLYLDPSVLLQTLIEPFLQTIPLALTNTLAALS  60

Query  226  RSRASAEGMRARSLGEEGWVGDVLEGYQDVMSAWARCLGGERGKAVVREDRILGKEFKEQ  285
            RSRASAEGMRARSLGEEGWVGDVLEGYQDVMSAWARCLGGERGKAVVREDRILGKEFKEQ
Sbjct  61   RSRASAEGMRARSLGEEGWVGDVLEGYQDVMSAWARCLGGERGKAVVREDRILGKEFKEQ  120

Query  286  IIESLNPPSPLPSPVLSRLPSFDRTSSIDRASSPTPSDSSASSITSVQTTTPTSYRLQRQ  345
            IIESLNPPSPLPSPVLSRLPSFDRTSSIDRASSPTPSDSSASSITSVQTTTPTSYRLQRQ
Sbjct  121  IIESLNPPSPLPSPVLSRLPSFDRTSSIDRASSPTPSDSSASSITSVQTTTPTSYRLQRQ  180

Query  346  HTTNQDKESGNEAGKGKGKGRAKAGTVKSRWNGLDSGVEGLSGDESYEEEQNLEKVHLST  405
            HTTN+DKESGNEAGKGKGKGRAKAGTVKSRWNGLDSGVEGLSGDESYEEEQNLEKVHLST
Sbjct  181  HTTNRDKESGNEAGKGKGKGRAKAGTVKSRWNGLDSGVEGLSGDESYEEEQNLEKVHLST  240

Query  406  AQKIPFSSTSSSATNPITDKLAHEESRQDEGRTSYTSTSFGSKLVLLQKFLQPRSSSSPL  465
            AQKIPFSSTSSSATNPITDKLAHEESRQDEGRTSYTSTSFGSKLVLLQKFLQPRSSSSPL
Sbjct  241  AQKIPFSSTSSSATNPITDKLAHEESRQDEGRTSYTSTSFGSKLVLLQKFLQPRSSSSPL  300

Query  466  PETLPASPMRHSLSKQSSTDLMNPNSYLLIFLRAIKSSFKAGLFLWIIWHIIYRVRAGWF  525
            PETLPASPMRHSLSKQSSTDLMNPNSYLLIFLRAIKSSFKAGLFLWIIWHIIYRVRAGWF
Sbjct  301  PETLPASPMRHSLSKQSSTDLMNPNSYLLIFLRAIKSSFKAGLFLWIIWHIIYRVRAGWF  360

Query  526  RPSSVPSMTSAGGGVVSGVGRAEEIRRRLRRGILARF  562
            RPSSVPSMTSAGGGVVSGVGRAEEIRRRLRRGILARF
Sbjct  361  RPSSVPSMTSAGGGVVSGVGRAEEIRRRLRRGILARF  397


>gb|EDL04856.1| mCG49710 [Mus musculus]
Length=915

 Score = 40.4 bits (93),  Expect = 5.8, Method: Compositional matrix adjust.
 Identities = 45/174 (26%), Positives = 72/174 (41%), Gaps = 10/174 (6%)

Query  211  QTIPLALTNTLAALSRSRASAEGMRARSLGEEGWVGDVLEGYQDVMSAWARCLGGERGKA  270
            + +P AL +      R  A +EGMR R  GEE  + +V +  QD +      L  E+  +
Sbjct  18   RVVPTALRDHPQLRGREVARSEGMRGRGAGEEQELSEVDKQLQDELEMLLERL-REKDTS  76

Query  271  VVREDRILGKEFKEQIIESLNPPSPLPSPVLSRLPSFDRTSSIDRASSPTPSDSSASSIT  330
            + R      +E + QI  S    + +P P+    P + +   I    +P  +   A+ I 
Sbjct  77   LYRPAL---EELRRQIHSSTTSMTSVPKPLKFLRPHYGKLKEIYENMAPGENKCFAADII  133

Query  331  SVQTTTPTSYR--LQRQHTTNQDKESGNEAGKGKGKGRAKAGTVKSRWNGLDSG  382
            SV   T +  R  L+ +   +Q++     A  G    R  AG V   W  LD  
Sbjct  134  SVLAMTMSGERECLKYRLVGSQEE----LASWGHEYVRHLAGEVTKEWQELDDA  183


>emb|CCF53829.1| related to SPT3-general transcriptional adaptor or co-activator 
[Ustilago hordei]
Length=432

 Score = 39.7 bits (91),  Expect = 8.5, Method: Compositional matrix adjust.
 Identities = 27/88 (31%), Positives = 41/88 (47%), Gaps = 6/88 (7%)

Query  103  DERARDTEGLALFGQIRKRCLDIYQPSPSSSASISLSRPSPTKNGHASLVSSSSHQNGPQ  162
            D+ +RDTEG A   + +    D+Y P     A+ +       KNG A+    +  +    
Sbjct  313  DDNSRDTEGEAAEKRAKMEASDMYGP-----AATTAGEADEAKNGEAADAQEADDEANTT  367

Query  163  LNSMMTINTTIDTPFALPPSIIHPLLLS  190
             N+  T +  + T F LPPS+  PLL S
Sbjct  368  ANAKGT-DDELCTLFTLPPSVETPLLAS  394


Lambda      K        H        a         alpha
   0.315    0.130    0.379    0.792     4.96 

Gapped
Lambda      K        H        a         alpha    sigma
   0.267   0.0410    0.140     1.90     42.6     43.6 

Effective search space used: 6060914893495


  Database: nr
    Posted date:  Sep 23, 2015 12:05 AM
  Number of letters in database: 26,053,659,533
  Number of sequences in database:  71,551,133


Matrix: BLOSUM62
Gap Penalties: Existence: 11, Extension: 1
Neighboring words threshold: 11
Window for multiple hits: 40
```
