# Supplementary material for: Comparative genomics provides new insights into the diversity, physiology, and sexuality of the only industrially exploited tremellomycete: Phaffia rhodozyma
Source: BMC Genomics. 2016 Nov 9;17:901. doi: 10.1186/s12864-016-3244-7 (PMC5103461; doi:10.1186/s12864-016-3244-7)
Supplement: Additional file 6: — List of orphan genes with links to PFAM (related to Additional file 1: Table S1). (ZIP 1428 kb) [file 12864_2016_3244_MOESM6_ESM.zip › BLAST_HTML_FTR/G03402_P.html]

BLAST Search Results


```
BLASTP 2.2.27+


Reference:
Stephen F. Altschul, Thomas L. Madden, Alejandro A. Schäffer,
Jinghui Zhang, Zheng Zhang, Webb Miller, and David J. Lipman (1997),
"Gapped BLAST and PSI-BLAST: a new generation of protein database
search programs", Nucleic Acids Res. 25:3389-3402.


Reference for
composition-based statistics:
Alejandro A. Schäffer, L. Aravind, Thomas L. Madden, Sergei
Shavirin, John L. Spouge, Yuri I. Wolf, Eugene V. Koonin, and
Stephen F. Altschul (2001), "Improving the accuracy of PSI-BLAST
protein database searches with composition-based statistics and
other refinements", Nucleic Acids Res. 29:2994-3005.


Database: nr
           71,551,133 sequences; 26,053,659,533 total letters


Query= G03402_P

Length=124
                                                                      Score     E
Sequences producing significant alignments:                          (Bits)  Value

emb|CDZ98680.1|  hypothetical protein [Xanthophyllomyces dendrorh...   240    6e-79
ref|WP_043143755.1|  peptide chain release factor 1 [Ponticoccus ...  42.7    0.031
ref|WP_024814749.1|  deoxyribonuclease [Acidovorax sp. JHL-3]         38.9    0.65 
ref|WP_028708495.1|  hypothetical protein [Propionicicella superf...  37.7    1.1  
gb|ESQ09827.1|  hypothetical protein N838_02250 [uncultured Thioh...  36.2    4.5  


 >emb|CDZ98680.1| hypothetical protein [Xanthophyllomyces dendrorhous]
Length=123

 Score =  240 bits (613),  Expect = 6e-79, Method: Compositional matrix adjust.
 Identities = 123/123 (100%), Positives = 123/123 (100%), Gaps = 0/123 (0%)

Query  1    MSPGQSKMSTDEPSLASLSDLVATLPPTIPSLEAWADLQLTFQTIAKAKALLQDPVFQTL  60
            MSPGQSKMSTDEPSLASLSDLVATLPPTIPSLEAWADLQLTFQTIAKAKALLQDPVFQTL
Sbjct  1    MSPGQSKMSTDEPSLASLSDLVATLPPTIPSLEAWADLQLTFQTIAKAKALLQDPVFQTL  60

Query  61   PEPPKPDSLAPLPLVPMMSAPSAEPTIANTVATVVVKEEKNVGNEVEEVGLTSQDKEHQM  120
            PEPPKPDSLAPLPLVPMMSAPSAEPTIANTVATVVVKEEKNVGNEVEEVGLTSQDKEHQM
Sbjct  61   PEPPKPDSLAPLPLVPMMSAPSAEPTIANTVATVVVKEEKNVGNEVEEVGLTSQDKEHQM  120

Query  121  DIS  123
            DIS
Sbjct  121  DIS  123


>ref|WP_043143755.1| peptide chain release factor 1 [Ponticoccus sp. UMTAT08]
 gb|KHQ52238.1| Peptide chain release factor 1 [Ponticoccus sp. UMTAT08]
Length=349

 Score = 42.7 bits (99),  Expect = 0.031, Method: Compositional matrix adjust.
 Identities = 25/69 (36%), Positives = 35/69 (51%), Gaps = 3/69 (4%)

Query  5   QSKMSTDEPSLASLSDLVATLPPTIPSLEAWADLQLTFQTIAKAKALLQDPVFQTLPEPP  64
           +++MS  E  +A+L    A L P +  +  W  L      IA+A+ALL DP  + L E  
Sbjct  18  EARMSAGEGDIAALGREYAELRPVVSQIAEWERL---VAEIAEAEALLADPEMKALAEEE  74

Query  65  KPDSLAPLP  73
            PD  A LP
Sbjct  75  LPDLRARLP  83


>ref|WP_024814749.1| deoxyribonuclease [Acidovorax sp. JHL-3]
Length=1132

 Score = 38.9 bits (89),  Expect = 0.65, Method: Composition-based stats.
 Identities = 26/70 (37%), Positives = 37/70 (53%), Gaps = 5/70 (7%)

Query  4     GQSKMSTDEPSLASLSDLVATLPPTIPSLEAWADLQLTFQTIAKAKALLQDPVFQTLPEP  63
             G SK   +    AS  DL+A +   I  L+ W + +   QT A+ +  + D ++QTLPEP
Sbjct  1048  GLSKTDRERIKQAS-KDLLAGVLAVIAPLDRWTEKE---QTQAEVETFVLDQIYQTLPEP  1103

Query  64    P-KPDSLAPL  72
             P  PD  A L
Sbjct  1104  PYSPDDKASL  1113


>ref|WP_028708495.1| hypothetical protein [Propionicicella superfundia]
Length=215

 Score = 37.7 bits (86),  Expect = 1.1, Method: Compositional matrix adjust.
 Identities = 29/84 (35%), Positives = 44/84 (52%), Gaps = 4/84 (5%)

Query  41   TFQTIAKAKALLQDPVFQTLPEPPKPDSLA-PLPLVPMMSAPSAEPT-IANTVATVVVKE  98
            T++T      + +DP+    P P  PDSLA  +PLV  M     + T  ANT+ T+ +K 
Sbjct  40   TYRTFGWTVEVREDPLDADTPRPL-PDSLARSVPLVGRMYRAVEDSTPQANTI-TLRLKR  97

Query  99   EKNVGNEVEEVGLTSQDKEHQMDI  122
            ++++ N  E VGL  Q +    DI
Sbjct  98   DRHIANRAEVVGLQHQAEAALADI  121


>gb|ESQ09827.1| hypothetical protein N838_02250 [uncultured Thiohalocapsa sp. 
PB-PSB1]
Length=479

 Score = 36.2 bits (82),  Expect = 4.5, Method: Composition-based stats.
 Identities = 32/103 (31%), Positives = 47/103 (46%), Gaps = 13/103 (13%)

Query  15   LASLSDLVATLPPTIPSLEAWADLQLTFQTIAKAKALLQDPVFQTLPEPPKPDSLAPLP-  73
            L SLS   ATL P  P  EA  D+Q          A +Q+P+    PE  +  +L P+P 
Sbjct  48   LISLSGFAATLSPAAP--EAIPDMQ---------AATVQEPMADANPEQSQKPTLVPVPE  96

Query  74   LVPMMSAPSAEP-TIANTVATVVVKEEKNVGNEVEEVGLTSQD  115
            L P  S   AE  +  N V + + K  + +G+  E +    Q+
Sbjct  97   LAPEGSPEGAEERSPGNKVHSEITKRAQKLGDIEERLARLQQE  139


Lambda      K        H        a         alpha
   0.312    0.128    0.359    0.792     4.96 

Gapped
Lambda      K        H        a         alpha    sigma
   0.267   0.0410    0.140     1.90     42.6     43.6 

Effective search space used: 644902712190


  Database: nr
    Posted date:  Sep 23, 2015 12:05 AM
  Number of letters in database: 26,053,659,533
  Number of sequences in database:  71,551,133


Matrix: BLOSUM62
Gap Penalties: Existence: 11, Extension: 1
Neighboring words threshold: 11
Window for multiple hits: 40
```
