# Supplementary material for: Comparative genomics provides new insights into the diversity, physiology, and sexuality of the only industrially exploited tremellomycete: Phaffia rhodozyma
Source: BMC Genomics. 2016 Nov 9;17:901. doi: 10.1186/s12864-016-3244-7 (PMC5103461; doi:10.1186/s12864-016-3244-7)
Supplement: Additional file 6: — List of orphan genes with links to PFAM (related to Additional file 1: Table S1). (ZIP 1428 kb) [file 12864_2016_3244_MOESM6_ESM.zip › BLAST_HTML_FTR/G04612_P.html]

BLAST Search Results


```
BLASTP 2.2.27+


Reference:
Stephen F. Altschul, Thomas L. Madden, Alejandro A. Schäffer,
Jinghui Zhang, Zheng Zhang, Webb Miller, and David J. Lipman (1997),
"Gapped BLAST and PSI-BLAST: a new generation of protein database
search programs", Nucleic Acids Res. 25:3389-3402.


Reference for
composition-based statistics:
Alejandro A. Schäffer, L. Aravind, Thomas L. Madden, Sergei
Shavirin, John L. Spouge, Yuri I. Wolf, Eugene V. Koonin, and
Stephen F. Altschul (2001), "Improving the accuracy of PSI-BLAST
protein database searches with composition-based statistics and
other refinements", Nucleic Acids Res. 29:2994-3005.


Database: nr
           71,551,133 sequences; 26,053,659,533 total letters


Query= G04612_P

Length=490
                                                                      Score     E
Sequences producing significant alignments:                          (Bits)  Value

emb|CDZ96166.1|  hypothetical protein [Xanthophyllomyces dendrorh...   996    0.0  
gb|KIY62836.1|  hypothetical protein CYLTODRAFT_494379 [Cylindrob...  43.5    0.28 


 >emb|CDZ96166.1| hypothetical protein [Xanthophyllomyces dendrorhous]
Length=489

 Score =  996 bits (2575),  Expect = 0.0, Method: Compositional matrix adjust.
 Identities = 489/489 (100%), Positives = 489/489 (100%), Gaps = 0/489 (0%)

Query  1    MPIEIPQLNLSGKNFEHPIANTGPPSKTTSAYVISSLLQPPRRNAQLHLNNRLTLDQHAQ  60
            MPIEIPQLNLSGKNFEHPIANTGPPSKTTSAYVISSLLQPPRRNAQLHLNNRLTLDQHAQ
Sbjct  1    MPIEIPQLNLSGKNFEHPIANTGPPSKTTSAYVISSLLQPPRRNAQLHLNNRLTLDQHAQ  60

Query  61   PWFSNALGWDTVGDQSSRKPSQRLTNTERRTNGAMDDEVPRQIRKARSSHSITAKGKERQ  120
            PWFSNALGWDTVGDQSSRKPSQRLTNTERRTNGAMDDEVPRQIRKARSSHSITAKGKERQ
Sbjct  61   PWFSNALGWDTVGDQSSRKPSQRLTNTERRTNGAMDDEVPRQIRKARSSHSITAKGKERQ  120

Query  121  IDDEYYSGDQSEGEDEDEEEEWFERDVARRASAKKSYREQLKAQTLDFLRNPDPRYYDLP  180
            IDDEYYSGDQSEGEDEDEEEEWFERDVARRASAKKSYREQLKAQTLDFLRNPDPRYYDLP
Sbjct  121  IDDEYYSGDQSEGEDEDEEEEWFERDVARRASAKKSYREQLKAQTLDFLRNPDPRYYDLP  180

Query  181  NSELLKTILHSATHYYTSQSPPLLDELGYNQYLAHMARLARHANKQTRRRRRLRRKQFAQ  240
            NSELLKTILHSATHYYTSQSPPLLDELGYNQYLAHMARLARHANKQTRRRRRLRRKQFAQ
Sbjct  181  NSELLKTILHSATHYYTSQSPPLLDELGYNQYLAHMARLARHANKQTRRRRRLRRKQFAQ  240

Query  241  GGGNFGQDGQDEEDGGSSSSSSSSSSESYSSSSASSSTLSSSGSSFSSRLTQTSKALRRT  300
            GGGNFGQDGQDEEDGGSSSSSSSSSSESYSSSSASSSTLSSSGSSFSSRLTQTSKALRRT
Sbjct  241  GGGNFGQDGQDEEDGGSSSSSSSSSSESYSSSSASSSTLSSSGSSFSSRLTQTSKALRRT  300

Query  301  RSQSTVAETSKSCPDSTSDLSRPPSRASTKTKGTPRTLPKSIDTDDCDPRMSGTRGKKPR  360
            RSQSTVAETSKSCPDSTSDLSRPPSRASTKTKGTPRTLPKSIDTDDCDPRMSGTRGKKPR
Sbjct  301  RSQSTVAETSKSCPDSTSDLSRPPSRASTKTKGTPRTLPKSIDTDDCDPRMSGTRGKKPR  360

Query  361  LASGMKKVVLRDSAGPKKNKKRRTRWRAVSKLDMHKSLDGDSLIALGILLEEHVRHQVLT  420
            LASGMKKVVLRDSAGPKKNKKRRTRWRAVSKLDMHKSLDGDSLIALGILLEEHVRHQVLT
Sbjct  361  LASGMKKVVLRDSAGPKKNKKRRTRWRAVSKLDMHKSLDGDSLIALGILLEEHVRHQVLT  420

Query  421  RSYSPFQSALSPTPEPTQSSSPPSFSHSDGGESRPEGTSKEGATAEEGDITGTESANSDV  480
            RSYSPFQSALSPTPEPTQSSSPPSFSHSDGGESRPEGTSKEGATAEEGDITGTESANSDV
Sbjct  421  RSYSPFQSALSPTPEPTQSSSPPSFSHSDGGESRPEGTSKEGATAEEGDITGTESANSDV  480

Query  481  FDMRDSDTE  489
            FDMRDSDTE
Sbjct  481  FDMRDSDTE  489


>gb|KIY62836.1| hypothetical protein CYLTODRAFT_494379 [Cylindrobasidium torrendii 
FP15055 ss-10]
Length=279

 Score = 43.5 bits (101),  Expect = 0.28, Method: Compositional matrix adjust.
 Identities = 23/64 (36%), Positives = 38/64 (59%), Gaps = 6/64 (9%)

Query  364  GMKKVVLRDSAGPKKNKKRRTR------WRAVSKLDMHKSLDGDSLIALGILLEEHVRHQ  417
            G  ++ L +S   ++ KKR  R      WR   + DMH+++DG +L A+G+LL+E+V   
Sbjct  188  GESEIELPESEDWQEPKKRHVRTGKSEEWRKTRRRDMHRAMDGSALTAIGVLLQEYVGTL  247

Query  418  VLTR  421
            + TR
Sbjct  248  LETR  251


Lambda      K        H        a         alpha
   0.309    0.124    0.349    0.792     4.96 

Gapped
Lambda      K        H        a         alpha    sigma
   0.267   0.0410    0.140     1.90     42.6     43.6 

Effective search space used: 5012683362530


  Database: nr
    Posted date:  Sep 23, 2015 12:05 AM
  Number of letters in database: 26,053,659,533
  Number of sequences in database:  71,551,133


Matrix: BLOSUM62
Gap Penalties: Existence: 11, Extension: 1
Neighboring words threshold: 11
Window for multiple hits: 40
```
