# Supplementary material for: Comparative genomics provides new insights into the diversity, physiology, and sexuality of the only industrially exploited tremellomycete: Phaffia rhodozyma
Source: BMC Genomics. 2016 Nov 9;17:901. doi: 10.1186/s12864-016-3244-7 (PMC5103461; doi:10.1186/s12864-016-3244-7)
Supplement: Additional file 6: — List of orphan genes with links to PFAM (related to Additional file 1: Table S1). (ZIP 1428 kb) [file 12864_2016_3244_MOESM6_ESM.zip › BLAST_HTML_FTR/G05272_P.html]

BLAST Search Results


```
BLASTP 2.2.27+


Reference:
Stephen F. Altschul, Thomas L. Madden, Alejandro A. Schäffer,
Jinghui Zhang, Zheng Zhang, Webb Miller, and David J. Lipman (1997),
"Gapped BLAST and PSI-BLAST: a new generation of protein database
search programs", Nucleic Acids Res. 25:3389-3402.


Reference for
composition-based statistics:
Alejandro A. Schäffer, L. Aravind, Thomas L. Madden, Sergei
Shavirin, John L. Spouge, Yuri I. Wolf, Eugene V. Koonin, and
Stephen F. Altschul (2001), "Improving the accuracy of PSI-BLAST
protein database searches with composition-based statistics and
other refinements", Nucleic Acids Res. 29:2994-3005.


Database: nr
           71,551,133 sequences; 26,053,659,533 total letters


Query= G05272_P

Length=143
                                                                      Score     E
Sequences producing significant alignments:                          (Bits)  Value

emb|CED82392.1|  hypothetical protein [Xanthophyllomyces dendrorh...   291    2e-98
ref|XP_011471151.1|  PREDICTED: NT-3 growth factor receptor-like ...  39.3    0.71 
ref|XP_011471150.1|  PREDICTED: NT-3 growth factor receptor-like ...  39.3    0.71 
gb|ENH81593.1|  zinc-binding dehydrogenase [Colletotrichum orbicu...  36.2    7.6  
ref|WP_052262368.1|  hypothetical protein [Pseudoalteromonas lute...  36.2    8.5  


 >emb|CED82392.1| hypothetical protein [Xanthophyllomyces dendrorhous]
Length=142

 Score =  291 bits (746),  Expect = 2e-98, Method: Compositional matrix adjust.
 Identities = 142/142 (100%), Positives = 142/142 (100%), Gaps = 0/142 (0%)

Query  1    MNISRTVLFTTGKQLSFIPSSSRAPALAKRSYAGSDHANTSKPTPPDQESKEPQSSSQAK  60
            MNISRTVLFTTGKQLSFIPSSSRAPALAKRSYAGSDHANTSKPTPPDQESKEPQSSSQAK
Sbjct  1    MNISRTVLFTTGKQLSFIPSSSRAPALAKRSYAGSDHANTSKPTPPDQESKEPQSSSQAK  60

Query  61   AWTVAAVGSVCGIIFYNFNPFRTLSPPINMTGKETSAAYESQDHSQGVKGGGGIGKVVGG  120
            AWTVAAVGSVCGIIFYNFNPFRTLSPPINMTGKETSAAYESQDHSQGVKGGGGIGKVVGG
Sbjct  61   AWTVAAVGSVCGIIFYNFNPFRTLSPPINMTGKETSAAYESQDHSQGVKGGGGIGKVVGG  120

Query  121  SDTQNRESSAWKSTAATFENKK  142
            SDTQNRESSAWKSTAATFENKK
Sbjct  121  SDTQNRESSAWKSTAATFENKK  142


>ref|XP_011471151.1| PREDICTED: NT-3 growth factor receptor-like isoform X2 [Oryzias 
latipes]
Length=816

 Score = 39.3 bits (90),  Expect = 0.71, Method: Composition-based stats.
 Identities = 20/73 (27%), Positives = 35/73 (48%), Gaps = 8/73 (11%)

Query  40   TSKPTPPDQESKEPQSSSQAKAWTVAAVGSVCGI--IFYNFNPFRTLS------PPINMT  91
            +S P PP  E+KEPQ  S   +  V   G  C +  +F+    +R  S      P   M+
Sbjct  407  SSSPVPPISETKEPQEDSMRVSVAVGVAGFACAVLTVFFLLIKYRRRSKLTMKGPTAGMS  466

Query  92   GKETSAAYESQDH  104
            G+++++ +   +H
Sbjct  467  GEDSASPFHHINH  479


>ref|XP_011471150.1| PREDICTED: NT-3 growth factor receptor-like isoform X1 [Oryzias 
latipes]
Length=817

 Score = 39.3 bits (90),  Expect = 0.71, Method: Composition-based stats.
 Identities = 20/73 (27%), Positives = 35/73 (48%), Gaps = 8/73 (11%)

Query  40   TSKPTPPDQESKEPQSSSQAKAWTVAAVGSVCGI--IFYNFNPFRTLS------PPINMT  91
            +S P PP  E+KEPQ  S   +  V   G  C +  +F+    +R  S      P   M+
Sbjct  407  SSSPVPPISETKEPQEDSMRVSVAVGVAGFACAVLTVFFLLIKYRRRSKLTMKGPTAGMS  466

Query  92   GKETSAAYESQDH  104
            G+++++ +   +H
Sbjct  467  GEDSASPFHHINH  479


>gb|ENH81593.1| zinc-binding dehydrogenase [Colletotrichum orbiculare MAFF 240422]
Length=492

 Score = 36.2 bits (82),  Expect = 7.6, Method: Composition-based stats.
 Identities = 19/62 (31%), Positives = 30/62 (48%), Gaps = 0/62 (0%)

Query  5    RTVLFTTGKQLSFIPSSSRAPALAKRSYAGSDHANTSKPTPPDQESKEPQSSSQAKAWTV  64
            R V+     Q  F+   + APAL     A    A++S P+ P+  + EP+ S +A  W  
Sbjct  391  RMVVSLDDTQAGFVRDPTAAPALVDSPIAERSEADSSLPSSPEFTAAEPERSRRASLWQS  450

Query  65   AA  66
            A+
Sbjct  451  AS  452


>ref|WP_052262368.1| hypothetical protein [Pseudoalteromonas luteoviolacea]
Length=328

 Score = 36.2 bits (82),  Expect = 8.5, Method: Compositional matrix adjust.
 Identities = 33/125 (26%), Positives = 53/125 (42%), Gaps = 2/125 (2%)

Query  18   IPSSSRAPALAKRSYAGSDHANTSKPTPPDQESKEPQSSSQAKAWTVAAVGSVCGIIFYN  77
            +PS   A  L   S A  DH +T+  +P D    +   +  + A+     G+  G+   +
Sbjct  129  VPSDMPAVELKANSLAKFDHVSTNAKSPNDSSFLQFLITDNSTAYFERMSGNSVGVYGSS  188

Query  78   FNPFRTLSPPINMTGKETSAAYESQ-DHSQGVKGGGGIGKVVGGSDTQNRESSAWKSTAA  136
            F  F   S  I +   +TSAA  +Q +H + V+  G  G  +G + +       W   AA
Sbjct  189  FVEFSKRSDQIKLEVYDTSAAQSAQSNHFRSVQVAGN-GYFLGDNQSNVNLLMIWSKGAA  247

Query  137  TFENK  141
               NK
Sbjct  248  EINNK  252


Lambda      K        H        a         alpha
   0.312    0.125    0.365    0.792     4.96 

Gapped
Lambda      K        H        a         alpha    sigma
   0.267   0.0410    0.140     1.90     42.6     43.6 

Effective search space used: 641414800915


  Database: nr
    Posted date:  Sep 23, 2015 12:05 AM
  Number of letters in database: 26,053,659,533
  Number of sequences in database:  71,551,133


Matrix: BLOSUM62
Gap Penalties: Existence: 11, Extension: 1
Neighboring words threshold: 11
Window for multiple hits: 40
```
