# Supplementary material for: Comparative genomics provides new insights into the diversity, physiology, and sexuality of the only industrially exploited tremellomycete: Phaffia rhodozyma
Source: BMC Genomics. 2016 Nov 9;17:901. doi: 10.1186/s12864-016-3244-7 (PMC5103461; doi:10.1186/s12864-016-3244-7)
Supplement: Additional file 6: — List of orphan genes with links to PFAM (related to Additional file 1: Table S1). (ZIP 1428 kb) [file 12864_2016_3244_MOESM6_ESM.zip › BLAST_HTML_FTR/G01638_P.html]

BLAST Search Results


```
BLASTP 2.2.27+


Reference:
Stephen F. Altschul, Thomas L. Madden, Alejandro A. Schäffer,
Jinghui Zhang, Zheng Zhang, Webb Miller, and David J. Lipman (1997),
"Gapped BLAST and PSI-BLAST: a new generation of protein database
search programs", Nucleic Acids Res. 25:3389-3402.


Reference for
composition-based statistics:
Alejandro A. Schäffer, L. Aravind, Thomas L. Madden, Sergei
Shavirin, John L. Spouge, Yuri I. Wolf, Eugene V. Koonin, and
Stephen F. Altschul (2001), "Improving the accuracy of PSI-BLAST
protein database searches with composition-based statistics and
other refinements", Nucleic Acids Res. 29:2994-3005.


Database: nr
           71,551,133 sequences; 26,053,659,533 total letters


Query= G01638_P

Length=180
                                                                      Score     E
Sequences producing significant alignments:                          (Bits)  Value

emb|CDZ98182.1|  hypothetical protein [Xanthophyllomyces dendrorh...   351    8e-121
ref|WP_041963974.1|  hypothetical protein [Bacillus selenatarsena...  39.3    1.5   
ref|XP_001700339.1|  predicted protein [Chlamydomonas reinhardtii...  37.7    5.4   
gb|EAR17290.1|  Putative glycosyltransferase [Synechococcus sp. W...  37.0    8.6   


 >emb|CDZ98182.1| hypothetical protein [Xanthophyllomyces dendrorhous]
Length=179

 Score =  351 bits (900),  Expect = 8e-121, Method: Compositional matrix adjust.
 Identities = 177/179 (99%), Positives = 177/179 (99%), Gaps = 0/179 (0%)

Query  1    MSAEKGPVYPYRSSERRVSNKEPKGKTIRWVLIVVIGMLALLSQVVTKPDLFPYKDIKMS  60
            MSAEKGPVYPYRSSER VSNKEPKGKTIRWVLIVVIGMLALLSQVVTKPDLFPYKDIKMS
Sbjct  1    MSAEKGPVYPYRSSERWVSNKEPKGKTIRWVLIVVIGMLALLSQVVTKPDLFPYKDIKMS  60

Query  61   IPSRPISPTFLFSSPHPSPITPSDDLASSSSSNSPSDSFYKRSFSFPTSFTTSFGNWKVN  120
            IPSRPISPTFLFSSPHPSPITPSDDLASSSSSNSPSDSFYKRSFSFPTSFTTSFGNWKVN
Sbjct  61   IPSRPISPTFLFSSPHPSPITPSDDLASSSSSNSPSDSFYKRSFSFPTSFTTSFGNWKVN  120

Query  121  WSKENETPMIGSVPASGLRNAGLLRFLEHGRWSLMERPTYHTSEQISSSHPGTSSHASS  179
            WSKENET MIGSVPASGLRNAGLLRFLEHGRWSLMERPTYHTSEQISSSHPGTSSHASS
Sbjct  121  WSKENETLMIGSVPASGLRNAGLLRFLEHGRWSLMERPTYHTSEQISSSHPGTSSHASS  179


>ref|WP_041963974.1| hypothetical protein [Bacillus selenatarsenatis]
 dbj|GAM12021.1| hypothetical protein SAMD00020551_0140 [Bacillus selenatarsenatis 
SF-1]
Length=462

 Score = 39.3 bits (90),  Expect = 1.5, Method: Compositional matrix adjust.
 Identities = 36/125 (29%), Positives = 57/125 (46%), Gaps = 12/125 (10%)

Query  33   IVVIGMLALLSQVVTKPDLFPYKDIKMSIPSRPISPTFLFSSPHPSPITPSDDLASSSSS  92
            ++V+G+ A+L+ VV  P + PY D       + IS     S+  PS      +     ++
Sbjct  267  LIVLGIFAMLALVVI-PKV-PYLDYA---ADQIISG---LSANAPSEGGEDSEGEDGDNA  318

Query  93   NSPSDSFYKRSFSFPTSFTTSFGNWKVNWSKENETPMIGSVPASGLRNAGLLRFLEHGRW  152
            NS  D   KRS    +SF   +  W +N  K  E+P+IGS P       G +RF ++   
Sbjct  319  NSEDDERLKRSVESVSSFKNRYYYWNLNLEKFKESPIIGSGP----MKEGFVRFADNSYL  374

Query  153  SLMER  157
              + R
Sbjct  375  YTLAR  379


>ref|XP_001700339.1| predicted protein [Chlamydomonas reinhardtii]
 gb|EDO98181.1| predicted protein, partial [Chlamydomonas reinhardtii]
Length=1420

 Score = 37.7 bits (86),  Expect = 5.4, Method: Composition-based stats.
 Identities = 36/131 (27%), Positives = 59/131 (45%), Gaps = 17/131 (13%)

Query  63   SRPISPTFLFSSPHPSPITPSDDLASSSSSNSPSDSFYKRSFSF---PTSFTTSFGNWKV  119
            S P+  T  +S P P+   P+ +LA+SS     S +F + S      P++ TT+ G+   
Sbjct  30   SGPVPLTPAYSEPQPAQFQPAKELATSSVKGRLSTAFARVSLGKADEPSAQTTNGGSNGA  89

Query  120  NWSKENETPMIGSVPASGLRNAGLLRFL------EHGRWSLMERPTYHTSEQISSSH---  170
              S        G+    GL +  LL  L      + G W  +ER    T++ +S++H   
Sbjct  90   LTSPRGTGA--GAASNGGLAHEALLSLLPVLTSLDGGWWDQIERAVVATAQHLSATHACV  147

Query  171  ---PGTSSHAS  178
                G S++AS
Sbjct  148  FFISGDSTYAS  158


>gb|EAR17290.1| Putative glycosyltransferase [Synechococcus sp. WH 7805]
Length=333

 Score = 37.0 bits (84),  Expect = 8.6, Method: Compositional matrix adjust.
 Identities = 24/87 (28%), Positives = 39/87 (45%), Gaps = 15/87 (17%)

Query  98   SFYKRSFSFPTSFTTSFGNWKVNWSKENETPMIGSVPASGLRNAGLLRFLEHGRWSL---  154
             FY     F + F   F   + +WSK+ ET          L+NA  L  L+H  W++   
Sbjct  37   EFYYNEHGFDSDFDPEFAKQQSDWSKKTETY---------LKNANSLLNLQHATWNVTPT  87

Query  155  -MERPTY--HTSEQISSSHPGTSSHAS  178
              +R ++     ++ S+ H G S+HA 
Sbjct  88   NFQRSSFPIQFHQRFSTIHDGISNHAQ  114


Lambda      K        H        a         alpha
   0.316    0.130    0.393    0.792     4.96 

Gapped
Lambda      K        H        a         alpha    sigma
   0.267   0.0410    0.140     1.90     42.6     43.6 

Effective search space used: 641460036520


  Database: nr
    Posted date:  Sep 23, 2015 12:05 AM
  Number of letters in database: 26,053,659,533
  Number of sequences in database:  71,551,133


Matrix: BLOSUM62
Gap Penalties: Existence: 11, Extension: 1
Neighboring words threshold: 11
Window for multiple hits: 40
```
