# Supplementary material for: Comparative genomics provides new insights into the diversity, physiology, and sexuality of the only industrially exploited tremellomycete: Phaffia rhodozyma
Source: BMC Genomics. 2016 Nov 9;17:901. doi: 10.1186/s12864-016-3244-7 (PMC5103461; doi:10.1186/s12864-016-3244-7)
Supplement: Additional file 6: — List of orphan genes with links to PFAM (related to Additional file 1: Table S1). (ZIP 1428 kb) [file 12864_2016_3244_MOESM6_ESM.zip › BLAST_HTML_FTR/G02906_P.html]

BLAST Search Results


```
BLASTP 2.2.27+


Reference:
Stephen F. Altschul, Thomas L. Madden, Alejandro A. Schäffer,
Jinghui Zhang, Zheng Zhang, Webb Miller, and David J. Lipman (1997),
"Gapped BLAST and PSI-BLAST: a new generation of protein database
search programs", Nucleic Acids Res. 25:3389-3402.


Reference for
composition-based statistics:
Alejandro A. Schäffer, L. Aravind, Thomas L. Madden, Sergei
Shavirin, John L. Spouge, Yuri I. Wolf, Eugene V. Koonin, and
Stephen F. Altschul (2001), "Improving the accuracy of PSI-BLAST
protein database searches with composition-based statistics and
other refinements", Nucleic Acids Res. 29:2994-3005.


Database: nr
           71,551,133 sequences; 26,053,659,533 total letters


Query= G02906_P

Length=1146
                                                                      Score     E
Sequences producing significant alignments:                          (Bits)  Value

emb|CED83476.1|  hypothetical protein [Xanthophyllomyces dendrorh...  1932    0.0  


 >emb|CED83476.1| hypothetical protein [Xanthophyllomyces dendrorhous]
Length=1073

 Score = 1932 bits (5005),  Expect = 0.0, Method: Compositional matrix adjust.
 Identities = 1042/1145 (91%), Positives = 1053/1145 (92%), Gaps = 72/1145 (6%)

Query  1     MNQFSSLPILQPPSVVSFSDSSANHKTSPLGRSSICVLSSLPNSSVLTDRLVQNLVQTQT  60
             MNQFSSLPILQPPSVVSFSDSSANHKTSPLGRSSICVLSSLPNSSVLTDRLVQNLVQTQT
Sbjct  1     MNQFSSLPILQPPSVVSFSDSSANHKTSPLGRSSICVLSSLPNSSVLTDRLVQNLVQTQT  60

Query  61    YHTTGSVDEIKTPVKLTTRLKSYFPSSESIKRLRPIKSFSKLKHKALGSTTKSPIVSTSL  120
             YHTTGSVDEIKTPVKLTTRLKSY PSSESIKRLRPIKSFSKLKHKALGSTTKSPIVSTSL
Sbjct  61    YHTTGSVDEIKTPVKLTTRLKSYLPSSESIKRLRPIKSFSKLKHKALGSTTKSPIVSTSL  120

Query  121   FTSLDLNVEVRPNGARALQSWSHSTASKSTFRRPGLSSCIRPGLSLDSPPGLNVREPANL  180
             FTSLDLNVEVRPNGARALQSWSHSTASKSTFRRPGLSSCIRPGLSLDSPPGLNVREPANL
Sbjct  121   FTSLDLNVEVRPNGARALQSWSHSTASKSTFRRPGLSSCIRPGLSLDSPPGLNVREPANL  180

Query  181   DGDLPKVIEIVQNPLLGKLSFEQQSINSRMEQTRAEREREAWKKRKSKTGVSWEERLEVL  240
             DGDLPKVIEIVQNPLLGKLSFEQQSINSRMEQTRAEREREAWKKRKSKTGVSWEERLEVL
Sbjct  181   DGDLPKVIEIVQNPLLGKLSFEQQSINSRMEQTRAEREREAWKKRKSKTGVSWEERLEVL  240

Query  241   DVCRGEISFGSKAGETHEGTRGFDAKADPSNATSSTKKNSILDVQTLPEIVRSNRIMNRR  300
             DVCRGEISFGSKAGETHEGTRGFDAKADPSNATSSTKKNSILDVQTLPEIVRSNRIMNRR
Sbjct  241   DVCRGEISFGSKAGETHEGTRGFDAKADPSNATSSTKKNSILDVQTLPEIVRSNRIMNRR  300

Query  301   LSRHGLRPSKSFDLDSRLPTIECSPPTSPTNSLQKLDDNSVANSNLSGHSILAPSETRLT  360
             LSRHGLRPSKSFDLDSRLPTIECSPPTSPTNSLQKLDDNSVANSNLSGHSILAPSETRLT
Sbjct  301   LSRHGLRPSKSFDLDSRLPTIECSPPTSPTNSLQKLDDNSVANSNLSGHSILAPSETRLT  360

Query  361   WARQGYRRQVRVPRRIASAISLPPLIPIPPLDPTPPNFPIDESPKPIEHTMSNPIGALSY  420
             WARQGYRRQVRVPRRIASAISLPPLIPIPPLDPTPPNFPIDESPKPIEHTMSNPIGALSY
Sbjct  361   WARQGYRRQVRVPRRIASAISLPPLIPIPPLDPTPPNFPIDESPKPIEHTMSNPIGALSY  420

Query  421   SSASRPSALPPSSVGASPQTDMTTVGKSQPSANPSTVLTPINTQLDRPGSIWRSQIIGRY  480
             SSASRPSALPPSSVGASPQTDMTTVGKSQPSANPSTVLTPINTQLDRPGSIWRSQIIGRY
Sbjct  421   SSASRPSALPPSSVGASPQTDMTTVGKSQPSANPSTVLTPINTQLDRPGSIWRSQIIGRY  480

Query  481   YDTSGFLEGSESEIDPSPLKGLEYDLARIISSDEGIEGAPAKKDRNEDLPPRLELHETFS  540
             YDTSGFLEGSESEIDPSPLKGLEYDLARIISSDEGIEGAPAKKDRNEDLPPRLELHETFS
Sbjct  481   YDTSGFLEGSESEIDPSPLKGLEYDLARIISSDEGIEGAPAKKDRNEDLPPRLELHETFS  540

Query  541   PILFSLDFSLTSENAEHSGSFPLKERLESQNQKIFTLIDPIVSPREDPYVHIPRARWLSM  600
             PILFSLDFSLTSENAEHSGSFPLKERLESQNQKIFTLIDPIVSPREDPYVHIPRARWLSM
Sbjct  541   PILFSLDFSLTSENAEHSGSFPLKERLESQNQKIFTLIDPIVSPREDPYVHIPRARWLSM  600

Query  601   DAQSALSAVNPFCIATSDSTSTVTTSSNATNPELLKSLCPILPYHTSPSPSSSCSPHRTS  660
             DAQSALSAVNPFCIATSDSTSTVTTSSNATNPE LKSLCPILPYHTSPSPSSSCSPHRTS
Sbjct  601   DAQSALSAVNPFCIATSDSTSTVTTSSNATNPEFLKSLCPILPYHTSPSPSSSCSPHRTS  660

Query  661   LNQPLHEALPLKLIAISLYLAQPSPIIDPMDVPLPSSPMDEISGFSFSAYSTLWTPPRQS  720
             LNQPLHEALPLKLIAISLYLAQPSPIIDPMDVPLPSSPMDEISGFSFSAYSTLWTPPRQS
Sbjct  661   LNQPLHEALPLKLIAISLYLAQPSPIIDPMDVPLPSSPMDEISGFSFSAYSTLWTPPRQS  720

Query  721   RPQSHHILEPISTYTPPITEADQADSLTSSLPSSPSLSATSSIIDESGDLSISSNLEAQV  780
             RPQSHHILEPISTYTPPITEADQADSLTSSLPSSPSLSATSSIIDESGDLSISSNLEAQV
Sbjct  721   RPQSHHILEPISTYTPPITEADQADSLTSSLPSSPSLSATSSIIDESGDLSISSNLEAQV  780

Query  781   KFGTASRLTSSVPRVIYQPSLSAGLNPLLKHYLHNDHVEDDNRRKDGEEGSDDESGYKYH  840
             KFGTASRLTSSVPRVIYQPSLSAGLNPLLKHYLHNDHVEDDNRRKDGEEGSDDESGYKYH
Sbjct  781   KFGTASRLTSSVPRVIYQPSLSAGLNPLLKHYLHNDHVEDDNRRKDGEEGSDDESGYKYH  840

Query  841   GRGSDSDQDLSPCVARSVFLSSRVTRKPTIVPQTWKSPSPSKPSGRAIKAGPVTISPSAG  900
             GRGSDSDQDLSPCVARSVFLSSRVTRKPTIVPQTWKSPSPSKPSGRAIKAGPVTISPSAG
Sbjct  841   GRGSDSDQDLSPCVARSVFLSSRVTRKPTIVPQTWKSPSPSKPSGRAIKAGPVTISPSAG  900

Query  901   MSFTPSFTSRERNRSSFRLNSSLIAEFGQGALSTSTPTTSEVSLQMFRRRIKDVLEAGIG  960
             MSFTPSFTSRERNRSSFRLNSSLIAEFGQGALSTSTPTTSEVSLQMFRRRIKDVLEAGI 
Sbjct  901   MSFTPSFTSRERNRSSFRLNSSLIAEFGQGALSTSTPTTSEVSLQMFRRRIKDVLEAGIE  960

Query  961   DSFDCSSVEVSQMKSGPGSIDRFISELINEGGVQEGKDPKQEEQEGDEEEEEEEEEEVEH  1020
             +                  + +F                         + + +  +EV H
Sbjct  961   E------------------VVKF-------------------------QVDFKTHDEVGH  977

Query  1021  DDSFTYALEHPEEVVKFQVDFKTHDEVGHIGSSTHAKYSTENKTTTYVSTVGLSSPTTPY  1080
               S T+A        K+  + KT   V  +G                     LSSPTTPY
Sbjct  978   IGSSTHA--------KYSTENKTTTYVSTVG---------------------LSSPTTPY  1008

Query  1081  IDNQRQRQGQMEPEDEFDDEHGNSSDLGSTGREIMRLAREKGQSKLLKRAAGRSIRNKLA  1140
             IDNQRQRQGQMEPEDEFDDEHGNSSDLGSTGREIMRLAREKGQSKLLKRAAGRSIRNKLA
Sbjct  1009  IDNQRQRQGQMEPEDEFDDEHGNSSDLGSTGREIMRLAREKGQSKLLKRAAGRSIRNKLA  1068

Query  1141  SNRGG  1145
             SNRGG
Sbjct  1069  SNRGG  1073


Lambda      K        H        a         alpha
   0.310    0.128    0.363    0.792     4.96 

Gapped
Lambda      K        H        a         alpha    sigma
   0.267   0.0410    0.140     1.90     42.6     43.6 

Effective search space used: 14230977971208


  Database: nr
    Posted date:  Sep 23, 2015 12:05 AM
  Number of letters in database: 26,053,659,533
  Number of sequences in database:  71,551,133


Matrix: BLOSUM62
Gap Penalties: Existence: 11, Extension: 1
Neighboring words threshold: 11
Window for multiple hits: 40
```
