# Supplementary material for: Comparative genomics provides new insights into the diversity, physiology, and sexuality of the only industrially exploited tremellomycete: Phaffia rhodozyma
Source: BMC Genomics. 2016 Nov 9;17:901. doi: 10.1186/s12864-016-3244-7 (PMC5103461; doi:10.1186/s12864-016-3244-7)
Supplement: Additional file 6: — List of orphan genes with links to PFAM (related to Additional file 1: Table S1). (ZIP 1428 kb) [file 12864_2016_3244_MOESM6_ESM.zip › BLAST_HTML_FTR/G04790_P.html]

BLAST Search Results


```
BLASTP 2.2.27+


Reference:
Stephen F. Altschul, Thomas L. Madden, Alejandro A. Schäffer,
Jinghui Zhang, Zheng Zhang, Webb Miller, and David J. Lipman (1997),
"Gapped BLAST and PSI-BLAST: a new generation of protein database
search programs", Nucleic Acids Res. 25:3389-3402.


Reference for
composition-based statistics:
Alejandro A. Schäffer, L. Aravind, Thomas L. Madden, Sergei
Shavirin, John L. Spouge, Yuri I. Wolf, Eugene V. Koonin, and
Stephen F. Altschul (2001), "Improving the accuracy of PSI-BLAST
protein database searches with composition-based statistics and
other refinements", Nucleic Acids Res. 29:2994-3005.


Database: nr
           71,551,133 sequences; 26,053,659,533 total letters


Query= G04790_P

Length=172
                                                                      Score     E
Sequences producing significant alignments:                          (Bits)  Value

emb|CED85238.1|  hypothetical protein [Xanthophyllomyces dendrorh...   345    6e-119
ref|XP_012686000.1|  PREDICTED: neurogenic locus notch homolog pr...  38.9    1.8   


 >emb|CED85238.1| hypothetical protein [Xanthophyllomyces dendrorhous]
Length=171

 Score =  345 bits (886),  Expect = 6e-119, Method: Compositional matrix adjust.
 Identities = 171/171 (100%), Positives = 171/171 (100%), Gaps = 0/171 (0%)

Query  1    MSGLPPSTLYPTVFALASSIVIVLFIGLSMGVRQQRRREQRRLVYQLAYEERMALYKERA  60
            MSGLPPSTLYPTVFALASSIVIVLFIGLSMGVRQQRRREQRRLVYQLAYEERMALYKERA
Sbjct  1    MSGLPPSTLYPTVFALASSIVIVLFIGLSMGVRQQRRREQRRLVYQLAYEERMALYKERA  60

Query  61   RPDMFEVGGGKGRWAGTEQVDCDKINVDLPISALVLPHHPGVKVLPSVAPPVMICVIILQ  120
            RPDMFEVGGGKGRWAGTEQVDCDKINVDLPISALVLPHHPGVKVLPSVAPPVMICVIILQ
Sbjct  61   RPDMFEVGGGKGRWAGTEQVDCDKINVDLPISALVLPHHPGVKVLPSVAPPVMICVIILQ  120

Query  121  PKPPISDFPETRGEPDEEEEGLGVDVDLGMETINVVGDWRGVTRQAGLGFE  171
            PKPPISDFPETRGEPDEEEEGLGVDVDLGMETINVVGDWRGVTRQAGLGFE
Sbjct  121  PKPPISDFPETRGEPDEEEEGLGVDVDLGMETINVVGDWRGVTRQAGLGFE  171


>ref|XP_012686000.1| PREDICTED: neurogenic locus notch homolog protein 1 [Clupea harengus]
Length=2492

 Score = 38.9 bits (89),  Expect = 1.8, Method: Composition-based stats.
 Identities = 17/61 (28%), Positives = 35/61 (57%), Gaps = 1/61 (2%)

Query  2     SGLPPSTLYPTVFALASSIVIVLFIGLSMGVRQQRRREQRRLVYQLAYEERMALYKERAR  61
             S  PP+ LYP ++ + + ++++ F+G+ M   ++RRRE  +L +   ++      K+R  
Sbjct  1724  SAPPPNELYP-IYVVLAGLIMLAFVGVGMVASRKRRRENGQLWFPEGFKTSETNKKKRRE  1782

Query  62    P  62
             P
Sbjct  1783  P  1783


Lambda      K        H        a         alpha
   0.323    0.143    0.438    0.792     4.96 

Gapped
Lambda      K        H        a         alpha    sigma
   0.267   0.0410    0.140     1.90     42.6     43.6 

Effective search space used: 644956994916


  Database: nr
    Posted date:  Sep 23, 2015 12:05 AM
  Number of letters in database: 26,053,659,533
  Number of sequences in database:  71,551,133


Matrix: BLOSUM62
Gap Penalties: Existence: 11, Extension: 1
Neighboring words threshold: 11
Window for multiple hits: 40
```
