# Supplementary material for: Comparative genomics provides new insights into the diversity, physiology, and sexuality of the only industrially exploited tremellomycete: Phaffia rhodozyma
Source: BMC Genomics. 2016 Nov 9;17:901. doi: 10.1186/s12864-016-3244-7 (PMC5103461; doi:10.1186/s12864-016-3244-7)
Supplement: Additional file 6: — List of orphan genes with links to PFAM (related to Additional file 1: Table S1). (ZIP 1428 kb) [file 12864_2016_3244_MOESM6_ESM.zip › BLAST_HTML_FTR/G04363_P.html]

BLAST Search Results


```
BLASTP 2.2.27+


Reference:
Stephen F. Altschul, Thomas L. Madden, Alejandro A. Schäffer,
Jinghui Zhang, Zheng Zhang, Webb Miller, and David J. Lipman (1997),
"Gapped BLAST and PSI-BLAST: a new generation of protein database
search programs", Nucleic Acids Res. 25:3389-3402.


Reference for
composition-based statistics:
Alejandro A. Schäffer, L. Aravind, Thomas L. Madden, Sergei
Shavirin, John L. Spouge, Yuri I. Wolf, Eugene V. Koonin, and
Stephen F. Altschul (2001), "Improving the accuracy of PSI-BLAST
protein database searches with composition-based statistics and
other refinements", Nucleic Acids Res. 29:2994-3005.


Database: nr
           71,551,133 sequences; 26,053,659,533 total letters


Query= G04363_P

Length=217
                                                                      Score     E
Sequences producing significant alignments:                          (Bits)  Value

emb|CED85041.1|  hypothetical protein [Xanthophyllomyces dendrorh...   435    7e-153
emb|CED83218.1|  hypothetical protein [Xanthophyllomyces dendrorh...  43.5    0.069 


 >emb|CED85041.1| hypothetical protein [Xanthophyllomyces dendrorhous]
Length=216

 Score =  435 bits (1119),  Expect = 7e-153, Method: Compositional matrix adjust.
 Identities = 216/216 (100%), Positives = 216/216 (100%), Gaps = 0/216 (0%)

Query  1    MFSTSFPSSSIISYSRSVDSALPIVRIRTVSSDARSFDWAEDVDRYYENETRGRGRSDAK  60
            MFSTSFPSSSIISYSRSVDSALPIVRIRTVSSDARSFDWAEDVDRYYENETRGRGRSDAK
Sbjct  1    MFSTSFPSSSIISYSRSVDSALPIVRIRTVSSDARSFDWAEDVDRYYENETRGRGRSDAK  60

Query  61   KPLRFLFPTAQEPNPHPCLQAIFRRSSAPAYVSTETYAAINVKPSASPSYSFSSLPSSSS  120
            KPLRFLFPTAQEPNPHPCLQAIFRRSSAPAYVSTETYAAINVKPSASPSYSFSSLPSSSS
Sbjct  61   KPLRFLFPTAQEPNPHPCLQAIFRRSSAPAYVSTETYAAINVKPSASPSYSFSSLPSSSS  120

Query  121  SSSTVAPSVEEDEWEETPEKEEVEWIEHTSISTSNLEAKSNPANVKYISVDPNAEECDDP  180
            SSSTVAPSVEEDEWEETPEKEEVEWIEHTSISTSNLEAKSNPANVKYISVDPNAEECDDP
Sbjct  121  SSSTVAPSVEEDEWEETPEKEEVEWIEHTSISTSNLEAKSNPANVKYISVDPNAEECDDP  180

Query  181  VQKIYSTVPWAVIGAGAGKEGVQALEQWGWKTLWQR  216
            VQKIYSTVPWAVIGAGAGKEGVQALEQWGWKTLWQR
Sbjct  181  VQKIYSTVPWAVIGAGAGKEGVQALEQWGWKTLWQR  216


>emb|CED83218.1| hypothetical protein [Xanthophyllomyces dendrorhous]
Length=242

 Score = 43.5 bits (101),  Expect = 0.069, Method: Compositional matrix adjust.
 Identities = 17/31 (55%), Positives = 23/31 (74%), Gaps = 0/31 (0%)

Query  43  VDRYYENETRGRGRSDAKKPLRFLFPTAQEP  73
           V+   E+  RGRGRSD +KPLRF +PT ++P
Sbjct  28  VEEVEEDNDRGRGRSDQRKPLRFFYPTVRQP  58


Lambda      K        H        a         alpha
   0.312    0.125    0.377    0.792     4.96 

Gapped
Lambda      K        H        a         alpha    sigma
   0.267   0.0410    0.140     1.90     42.6     43.6 

Effective search space used: 1149771635813


  Database: nr
    Posted date:  Sep 23, 2015 12:05 AM
  Number of letters in database: 26,053,659,533
  Number of sequences in database:  71,551,133


Matrix: BLOSUM62
Gap Penalties: Existence: 11, Extension: 1
Neighboring words threshold: 11
Window for multiple hits: 40
```
