# Supplementary material for: Comparative genomics provides new insights into the diversity, physiology, and sexuality of the only industrially exploited tremellomycete: Phaffia rhodozyma
Source: BMC Genomics. 2016 Nov 9;17:901. doi: 10.1186/s12864-016-3244-7 (PMC5103461; doi:10.1186/s12864-016-3244-7)
Supplement: Additional file 6: — List of orphan genes with links to PFAM (related to Additional file 1: Table S1). (ZIP 1428 kb) [file 12864_2016_3244_MOESM6_ESM.zip › BLAST_HTML_FTR/G03278_P.html]

BLAST Search Results


```
BLASTP 2.2.27+


Reference:
Stephen F. Altschul, Thomas L. Madden, Alejandro A. Schäffer,
Jinghui Zhang, Zheng Zhang, Webb Miller, and David J. Lipman (1997),
"Gapped BLAST and PSI-BLAST: a new generation of protein database
search programs", Nucleic Acids Res. 25:3389-3402.


Reference for
composition-based statistics:
Alejandro A. Schäffer, L. Aravind, Thomas L. Madden, Sergei
Shavirin, John L. Spouge, Yuri I. Wolf, Eugene V. Koonin, and
Stephen F. Altschul (2001), "Improving the accuracy of PSI-BLAST
protein database searches with composition-based statistics and
other refinements", Nucleic Acids Res. 29:2994-3005.


Database: nr
           71,551,133 sequences; 26,053,659,533 total letters


Query= G03278_P

Length=182
                                                                      Score     E
Sequences producing significant alignments:                          (Bits)  Value

emb|CDZ97798.1|  hypothetical protein [Xanthophyllomyces dendrorh...   362    3e-125
ref|XP_008955940.1|  PREDICTED: protein FAM47E isoform X2 [Pan pa...  39.3    1.3   
ref|XP_008955939.1|  PREDICTED: protein FAM47E isoform X1 [Pan pa...  39.3    1.6   
ref|XP_010419275.1|  PREDICTED: serine carboxypeptidase-like 10 [...  39.3    1.8   
ref|XP_008955942.1|  PREDICTED: protein FAM47E isoform X4 [Pan pa...  38.9    2.3   
ref|XP_011123238.1|  hypothetical protein AOL_s00081g118 [Arthrob...  37.7    5.6   
ref|WP_008087183.1|  histidine triad protein [Streptococcus ictal...  36.2    6.4   
gb|KGY15028.1|  hypothetical protein PABG_12143 [Paracoccidioides...  37.4    7.1   
ref|XP_010763449.1|  hypothetical protein PADG_08122 [Paracoccidi...  37.4    7.1   


 >emb|CDZ97798.1| hypothetical protein [Xanthophyllomyces dendrorhous]
Length=181

 Score =  362 bits (930),  Expect = 3e-125, Method: Compositional matrix adjust.
 Identities = 180/181 (99%), Positives = 180/181 (99%), Gaps = 0/181 (0%)

Query  1    MKTSEPLAVLLVAHHTINSLLSLNSQHLTLSTATIIKSLFLLDELSKLYPQLRVIEPNAG  60
            MKTSEPLAVLLVAHHTINSLLSLNSQHLTLSTATIIKSLFLLDELSKLYPQLRVIEPNAG
Sbjct  1    MKTSEPLAVLLVAHHTINSLLSLNSQHLTLSTATIIKSLFLLDELSKLYPQLRVIEPNAG  60

Query  61   ERMLIASWWIVGDSDQKRLLEESMLVTSVFQEVKGGISVLLRPSKLFKTKSTVLRVAPSR  120
            ERMLIASWWIVGDSDQKRLLEESMLVTSVFQEVKGGISVLLRPSKLFKTKSTVLRVAPSR
Sbjct  61   ERMLIASWWIVGDSDQKRLLEESMLVTSVFQEVKGGISVLLRPSKLFKTKSTVLRVAPSR  120

Query  121  NQSVTSSGHTIIPPLSPCRSIKKTSPPNFCRPLPKIANSNERRQQNPAVNLIAEEELFHA  180
            NQSVTSSGH IIPPLSPCRSIKKTSPPNFCRPLPKIANSNERRQQNPAVNLIAEEELFHA
Sbjct  121  NQSVTSSGHAIIPPLSPCRSIKKTSPPNFCRPLPKIANSNERRQQNPAVNLIAEEELFHA  180

Query  181  L  181
            L
Sbjct  181  L  181


>ref|XP_008955940.1| PREDICTED: protein FAM47E isoform X2 [Pan paniscus]
Length=330

 Score = 39.3 bits (90),  Expect = 1.3, Method: Compositional matrix adjust.
 Identities = 34/102 (33%), Positives = 51/102 (50%), Gaps = 11/102 (11%)

Query  30   LSTATIIKSLFLLDELSKLYPQLRVIEPNAGERM---LIASWWIVGDSDQKRLLEESMLV  86
            LS A   +  FL D  + L P    + PN  E M   L++    V D DQK  LE++   
Sbjct  104  LSPAQQARKAFLEDVEAHLTPHPLALYPNLEEAMPIQLLSKVLEVLDPDQK--LEDTW--  159

Query  87   TSVFQEVKGGISVLLRPSKLFKTKSTVLRVAPSRNQSVTSSG  128
             +  Q+ + G+     P+KL K  ST + + PSR  SV+++G
Sbjct  160  -AYCQDTRKGMK---EPTKLLKKHSTQVYLGPSRKTSVSNTG  197


>ref|XP_008955939.1| PREDICTED: protein FAM47E isoform X1 [Pan paniscus]
Length=356

 Score = 39.3 bits (90),  Expect = 1.6, Method: Compositional matrix adjust.
 Identities = 34/102 (33%), Positives = 51/102 (50%), Gaps = 11/102 (11%)

Query  30   LSTATIIKSLFLLDELSKLYPQLRVIEPNAGERM---LIASWWIVGDSDQKRLLEESMLV  86
            LS A   +  FL D  + L P    + PN  E M   L++    V D DQK  LE++   
Sbjct  104  LSPAQQARKAFLEDVEAHLTPHPLALYPNLEEAMPIQLLSKVLEVLDPDQK--LEDTW--  159

Query  87   TSVFQEVKGGISVLLRPSKLFKTKSTVLRVAPSRNQSVTSSG  128
             +  Q+ + G+     P+KL K  ST + + PSR  SV+++G
Sbjct  160  -AYCQDTRKGMK---EPTKLLKKHSTQVYLGPSRKTSVSNTG  197


>ref|XP_010419275.1| PREDICTED: serine carboxypeptidase-like 10 [Camelina sativa]
Length=527

 Score = 39.3 bits (90),  Expect = 1.8, Method: Compositional matrix adjust.
 Identities = 32/108 (30%), Positives = 53/108 (49%), Gaps = 18/108 (17%)

Query  67   SWWIVGDSDQKRLLEESMLVTSVFQEVKGGISVLL---RPSKLFKTKSTVLRVAPSRNQS  123
            S+++ GDS         M+V ++ QE+  G+S+ +    P +  ++ S    + P   +S
Sbjct  166  SFYVTGDS------YSGMIVPALVQEISKGVSLYIISSPPLRQLQSPSPAPHLVPPLGES  219

Query  124  VTSSGHTIIPPLSPCRSIKKT-SPPNFC---RPLPKIANSNERRQQNP  167
            + SS H    PL P R I +  S P FC   RP+ K++N    R  +P
Sbjct  220  IKSSSH----PLVPVREIHQCPSRPGFCPFHRPI-KLSNLTSYRSMDP  262


>ref|XP_008955942.1| PREDICTED: protein FAM47E isoform X4 [Pan paniscus]
Length=408

 Score = 38.9 bits (89),  Expect = 2.3, Method: Compositional matrix adjust.
 Identities = 34/106 (32%), Positives = 52/106 (49%), Gaps = 11/106 (10%)

Query  30   LSTATIIKSLFLLDELSKLYPQLRVIEPNAGERM---LIASWWIVGDSDQKRLLEESMLV  86
            LS A   +  FL D  + L P    + PN  E M   L++    V D DQK  LE++   
Sbjct  104  LSPAQQARKAFLEDVEAHLTPHPLALYPNLEEAMPIQLLSKVLEVLDPDQK--LEDTW--  159

Query  87   TSVFQEVKGGISVLLRPSKLFKTKSTVLRVAPSRNQSVTSSGHTII  132
             +  Q+ + G+     P+KL K  ST + + PSR  SV+++G  + 
Sbjct  160  -AYCQDTRKGMK---EPTKLLKKHSTQVYLGPSRKTSVSNTGQWLY  201


>ref|XP_011123238.1| hypothetical protein AOL_s00081g118 [Arthrobotrys oligospora 
ATCC 24927]
 gb|EGX48122.1| hypothetical protein AOL_s00081g118 [Arthrobotrys oligospora 
ATCC 24927]
Length=2330

 Score = 37.7 bits (86),  Expect = 5.6, Method: Compositional matrix adjust.
 Identities = 23/63 (37%), Positives = 30/63 (48%), Gaps = 6/63 (10%)

Query  101  LRPSKLFKTKSTVLRVAPSRNQSVTSS------GHTIIPPLSPCRSIKKTSPPNFCRPLP  154
            LR SKLF+ +S      PSRNQ V S         +++      R  KK SPP F R   
Sbjct  716  LRQSKLFREQSNTYSGTPSRNQHVPSKPRQRSVALSVVDACERYRKNKKASPPQFMRIAE  775

Query  155  KIA  157
            ++A
Sbjct  776  RLA  778


>ref|WP_008087183.1| histidine triad protein [Streptococcus ictaluri]
 gb|EHI70867.1| protein hit [Streptococcus ictaluri 707-05]
Length=139

 Score = 36.2 bits (82),  Expect = 6.4, Method: Compositional matrix adjust.
 Identities = 24/68 (35%), Positives = 38/68 (56%), Gaps = 11/68 (16%)

Query  123  SVTSSGHTIIPPLSPCRSI----KKTSPPNFCRPLPKIANSNERRQQNPAVNLIAEEE--  176
            S T+ GHT++ P    R++    ++T+   F R LPKIA + ++    PA+N+IA  E  
Sbjct  32   SQTTPGHTLLIPKKHTRNVLEMDEETAAATFAR-LPKIARAVQKATGAPAMNIIANNEEL  90

Query  177  ----LFHA  180
                +FHA
Sbjct  91   AGQTVFHA  98


>gb|KGY15028.1| hypothetical protein PABG_12143 [Paracoccidioides brasiliensis 
Pb03]
Length=530

 Score = 37.4 bits (85),  Expect = 7.1, Method: Compositional matrix adjust.
 Identities = 27/80 (34%), Positives = 39/80 (49%), Gaps = 3/80 (4%)

Query  96   GISVLLRPSKLFKTKSTV-LRVAPSRNQSVTSSGHTIIPPLSPCRSIKKTSPPNFCRPLP  154
            G S L  P       ST  + + P+ NQ  +S+G+  IPPL+P  +    +PP    P  
Sbjct  232  GFSKLQNPDSADDLVSTTPMSLTPNHNQVSSSNGNPRIPPLTPAAAFTNANPPKLAPPAD  291

Query  155  KIA--NSNERRQQNPAVNLI  172
             I+   S  RR+ NP  +LI
Sbjct  292  IISGNGSATRRRPNPNDSLI  311


>ref|XP_010763449.1| hypothetical protein PADG_08122 [Paracoccidioides brasiliensis 
Pb18]
 gb|EEH43302.2| hypothetical protein PADG_08122 [Paracoccidioides brasiliensis 
Pb18]
Length=530

 Score = 37.4 bits (85),  Expect = 7.1, Method: Compositional matrix adjust.
 Identities = 27/80 (34%), Positives = 39/80 (49%), Gaps = 3/80 (4%)

Query  96   GISVLLRPSKLFKTKSTV-LRVAPSRNQSVTSSGHTIIPPLSPCRSIKKTSPPNFCRPLP  154
            G S L  P       ST  + + P+ NQ  +S+G+  IPPL+P  +    +PP    P  
Sbjct  232  GFSKLRNPDSADDLVSTTPMSLTPNHNQVSSSNGNPRIPPLTPAAAFTNANPPKLAPPAD  291

Query  155  KIA--NSNERRQQNPAVNLI  172
             I+   S  RR+ NP  +LI
Sbjct  292  IISGNGSATRRRPNPNDSLI  311


Lambda      K        H        a         alpha
   0.320    0.133    0.381    0.792     4.96 

Gapped
Lambda      K        H        a         alpha    sigma
   0.267   0.0410    0.140     1.90     42.6     43.6 

Effective search space used: 673533038346


  Database: nr
    Posted date:  Sep 23, 2015 12:05 AM
  Number of letters in database: 26,053,659,533
  Number of sequences in database:  71,551,133


Matrix: BLOSUM62
Gap Penalties: Existence: 11, Extension: 1
Neighboring words threshold: 11
Window for multiple hits: 40
```
