# Supplementary material for: Comparative genomics provides new insights into the diversity, physiology, and sexuality of the only industrially exploited tremellomycete: Phaffia rhodozyma
Source: BMC Genomics. 2016 Nov 9;17:901. doi: 10.1186/s12864-016-3244-7 (PMC5103461; doi:10.1186/s12864-016-3244-7)
Supplement: Additional file 6: — List of orphan genes with links to PFAM (related to Additional file 1: Table S1). (ZIP 1428 kb) [file 12864_2016_3244_MOESM6_ESM.zip › BLAST_HTML_FTR/G04824_P.html]

BLAST Search Results


```
BLASTP 2.2.27+


Reference:
Stephen F. Altschul, Thomas L. Madden, Alejandro A. Schäffer,
Jinghui Zhang, Zheng Zhang, Webb Miller, and David J. Lipman (1997),
"Gapped BLAST and PSI-BLAST: a new generation of protein database
search programs", Nucleic Acids Res. 25:3389-3402.


Reference for
composition-based statistics:
Alejandro A. Schäffer, L. Aravind, Thomas L. Madden, Sergei
Shavirin, John L. Spouge, Yuri I. Wolf, Eugene V. Koonin, and
Stephen F. Altschul (2001), "Improving the accuracy of PSI-BLAST
protein database searches with composition-based statistics and
other refinements", Nucleic Acids Res. 29:2994-3005.


Database: nr
           71,551,133 sequences; 26,053,659,533 total letters


Query= G04824_P

Length=339
                                                                      Score     E
Sequences producing significant alignments:                          (Bits)  Value

emb|CDZ98774.1|  hypothetical protein [Xanthophyllomyces dendrorh...   629    0.0  


 >emb|CDZ98774.1| hypothetical protein [Xanthophyllomyces dendrorhous]
Length=396

 Score =  629 bits (1623),  Expect = 0.0, Method: Compositional matrix adjust.
 Identities = 321/356 (90%), Positives = 322/356 (90%), Gaps = 34/356 (10%)

Query  17   EDMMSTPNLARGRARPAESVDDDTDGPSGALQETSAIAELSPIRASFTRLRKKLDVQQLS  76
            EDMMSTPNLARGRARPAESVDDDT+GPSGALQETSAIAELSPIRASFTRLRKKLDVQQLS
Sbjct  41   EDMMSTPNLARGRARPAESVDDDTEGPSGALQETSAIAELSPIRASFTRLRKKLDVQQLS  100

Query  77   SAPNAVCAMSSVKTEVDYSRTRTASLNTSKRARQEISVHGYNTTAVAAESLDGSAAYKRN  136
            SAPNAVCAMSSVKTEVDYSRTRTASLNTSKRARQEISVHGYNTTAVAAESLDGSAAYKRN
Sbjct  101  SAPNAVCAMSSVKTEVDYSRTRTASLNTSKRARQEISVHGYNTTAVAAESLDGSAAYKRN  160

Query  137  RQSSSTPRPTDSALVSHSSRPLSTTHHSNKNMKFTVPRPFTFTASASSTDTASVVPDRQR  196
            RQSSSTPRPTDSALVSHSSRPLSTTHHSNKNMKFTVPRPFTFTASASSTDTASVVPDRQR
Sbjct  161  RQSSSTPRPTDSALVSHSSRPLSTTHHSNKNMKFTVPRPFTFTASASSTDTASVVPDRQR  220

Query  197  YYPYPAALSTKSTSSTQESALRASRSSNRDALSTQGREKRPEKRCISPRAGRVPSFREIH  256
            YYPYPAALSTKSTSSTQESALRASRSSNRDALSTQGREKRPEKRCISPRAGRVPSFREIH
Sbjct  221  YYPYPAALSTKSTSSTQESALRASRSSNRDALSTQGREKRPEKRCISPRAGRVPSFREIH  280

Query  257  LQDEARQARRTERIRSERAGEIEKEERGLGRR----------------------------  288
            LQDEARQARRTERIRSERAGEIEKEERGLGRR                            
Sbjct  281  LQDEARQARRTERIRSERAGEIEKEERGLGRRVEQRGRERARWENERRERERERERGREE  340

Query  289  ------KHEELQISQERKATVIRANPISEMYSSTLPHASSTIARPHARTNIRTQLD  338
                  KHEELQISQERKATVIRANPISEMYSSTLPHASSTIARPHARTNIRTQLD
Sbjct  341  EQKREQKHEELQISQERKATVIRANPISEMYSSTLPHASSTIARPHARTNIRTQLD  396


Lambda      K        H        a         alpha
   0.311    0.121    0.326    0.792     4.96 

Gapped
Lambda      K        H        a         alpha    sigma
   0.267   0.0410    0.140     1.90     42.6     43.6 

Effective search space used: 2866894428600


  Database: nr
    Posted date:  Sep 23, 2015 12:05 AM
  Number of letters in database: 26,053,659,533
  Number of sequences in database:  71,551,133


Matrix: BLOSUM62
Gap Penalties: Existence: 11, Extension: 1
Neighboring words threshold: 11
Window for multiple hits: 40
```
