# Supplementary material for: Comparative genomics provides new insights into the diversity, physiology, and sexuality of the only industrially exploited tremellomycete: Phaffia rhodozyma
Source: BMC Genomics. 2016 Nov 9;17:901. doi: 10.1186/s12864-016-3244-7 (PMC5103461; doi:10.1186/s12864-016-3244-7)
Supplement: Additional file 6: — List of orphan genes with links to PFAM (related to Additional file 1: Table S1). (ZIP 1428 kb) [file 12864_2016_3244_MOESM6_ESM.zip › BLAST_HTML_FTR/G02403_P.html]

BLAST Search Results


```
BLASTP 2.2.27+


Reference:
Stephen F. Altschul, Thomas L. Madden, Alejandro A. Schäffer,
Jinghui Zhang, Zheng Zhang, Webb Miller, and David J. Lipman (1997),
"Gapped BLAST and PSI-BLAST: a new generation of protein database
search programs", Nucleic Acids Res. 25:3389-3402.


Reference for
composition-based statistics:
Alejandro A. Schäffer, L. Aravind, Thomas L. Madden, Sergei
Shavirin, John L. Spouge, Yuri I. Wolf, Eugene V. Koonin, and
Stephen F. Altschul (2001), "Improving the accuracy of PSI-BLAST
protein database searches with composition-based statistics and
other refinements", Nucleic Acids Res. 29:2994-3005.


Database: nr
           71,551,133 sequences; 26,053,659,533 total letters


Query= G02403_P

Length=1325
                                                                      Score     E
Sequences producing significant alignments:                          (Bits)  Value

emb|CDZ98735.1|  hypothetical protein [Xanthophyllomyces dendrorh...  2657    0.0  


 >emb|CDZ98735.1| hypothetical protein [Xanthophyllomyces dendrorhous]
Length=1324

 Score = 2657 bits (6886),  Expect = 0.0, Method: Compositional matrix adjust.
 Identities = 1323/1324 (99%), Positives = 1323/1324 (99%), Gaps = 0/1324 (0%)

Query  1     MTPNWLSKSTTITSSNPQASPSEAGPKLTSNQTSLTHLGFRGSSPLSSSAAFSPLTAVID  60
             MTPNWLSKSTTITSSNPQASPSEAGPKLTSNQTSLTHLGFRGSSPLSSSAAFSPLTAVID
Sbjct  1     MTPNWLSKSTTITSSNPQASPSEAGPKLTSNQTSLTHLGFRGSSPLSSSAAFSPLTAVID  60

Query  61    TAGRRRANFPSARQTDLTEYFRTHGSERTRNASGMKPRARTEEVNGRGKERETDWENEGI  120
             TAGRRRANFPSARQTDLTEYFRTHGSERTRNASGMKPRARTEEVNGRGKERETDWENEGI
Sbjct  61    TAGRRRANFPSARQTDLTEYFRTHGSERTRNASGMKPRARTEEVNGRGKERETDWENEGI  120

Query  121   QVIGHRLSRSTTPMSGDHTARRVANSSTTTMFSMSSPSQAHLIKSSVASEWTKNGRSRLR  180
             QVIGHRLSRSTTPMSGDHTARRVANSSTTTMFSMSSPSQAHLIKSSVASEWTKNGRSRLR
Sbjct  121   QVIGHRLSRSTTPMSGDHTARRVANSSTTTMFSMSSPSQAHLIKSSVASEWTKNGRSRLR  180

Query  181   PLSSEDEIDVKLELSPQEIAQQARQEDAGFERADKRRKRSPLAAPFKEAMTSLNEAEDKG  240
             PLSSEDEIDVKLELSPQEIAQQARQEDAGFERADKRRKRSPLAAPFKEAMTSLNEAEDKG
Sbjct  181   PLSSEDEIDVKLELSPQEIAQQARQEDAGFERADKRRKRSPLAAPFKEAMTSLNEAEDKG  240

Query  241   MESVLRSRSARAERLMNDESSTTGSASILSMVSPNASKDPEKPTSRVLQSGLVTSRRTIS  300
             MESVLRSRSARAERLMNDESSTTGSASILSMVSPNASKDPEKPTSRV QSGLVTSRRTIS
Sbjct  241   MESVLRSRSARAERLMNDESSTTGSASILSMVSPNASKDPEKPTSRVSQSGLVTSRRTIS  300

Query  301   PSSQTAHSGELNFDQPAAFRASTLHPTPICIDPSEPCPTPVPSSPLLSESLLPLLPASYP  360
             PSSQTAHSGELNFDQPAAFRASTLHPTPICIDPSEPCPTPVPSSPLLSESLLPLLPASYP
Sbjct  301   PSSQTAHSGELNFDQPAAFRASTLHPTPICIDPSEPCPTPVPSSPLLSESLLPLLPASYP  360

Query  361   SPIQHSESLLRVFAPMGKDSAAHLRMLRRDQFAWESDQAFAKRKKELAEVEARKAEEEKA  420
             SPIQHSESLLRVFAPMGKDSAAHLRMLRRDQFAWESDQAFAKRKKELAEVEARKAEEEKA
Sbjct  361   SPIQHSESLLRVFAPMGKDSAAHLRMLRRDQFAWESDQAFAKRKKELAEVEARKAEEEKA  420

Query  421   AREKVRGVWQPPSRPSKKQRASDETEKSREQPSVLITGPSKDIQGRGAREIFQEELEEEE  480
             AREKVRGVWQPPSRPSKKQRASDETEKSREQPSVLITGPSKDIQGRGAREIFQEELEEEE
Sbjct  421   AREKVRGVWQPPSRPSKKQRASDETEKSREQPSVLITGPSKDIQGRGAREIFQEELEEEE  480

Query  481   EDAILQAITVSSTTPGSSSKHSRHHHLSPIRVVGPFRVQGAPSPKRARPASSQQTYSHTQ  540
             EDAILQAITVSSTTPGSSSKHSRHHHLSPIRVVGPFRVQGAPSPKRARPASSQQTYSHTQ
Sbjct  481   EDAILQAITVSSTTPGSSSKHSRHHHLSPIRVVGPFRVQGAPSPKRARPASSQQTYSHTQ  540

Query  541   VSENSSQTQSQPLPIGSVQGQPIVHPNKLQRSRSVINPLRDDDKFSTSSSSHSETHDESQ  600
             VSENSSQTQSQPLPIGSVQGQPIVHPNKLQRSRSVINPLRDDDKFSTSSSSHSETHDESQ
Sbjct  541   VSENSSQTQSQPLPIGSVQGQPIVHPNKLQRSRSVINPLRDDDKFSTSSSSHSETHDESQ  600

Query  601   LPAIITASRSDLGILPHFHLSPPLSNTLDTNQPISRRLSTPSMQKRSDRILVEQTPTPPR  660
             LPAIITASRSDLGILPHFHLSPPLSNTLDTNQPISRRLSTPSMQKRSDRILVEQTPTPPR
Sbjct  601   LPAIITASRSDLGILPHFHLSPPLSNTLDTNQPISRRLSTPSMQKRSDRILVEQTPTPPR  660

Query  661   GRTSSSWHINHGPSLDSGQSSEPIVRDWTYDGFRDSDSEDGLVESSVLPERSRLLSSKKK  720
             GRTSSSWHINHGPSLDSGQSSEPIVRDWTYDGFRDSDSEDGLVESSVLPERSRLLSSKKK
Sbjct  661   GRTSSSWHINHGPSLDSGQSSEPIVRDWTYDGFRDSDSEDGLVESSVLPERSRLLSSKKK  720

Query  721   GEIEPQSVEKLRSGDSAVQSSVPLASSMLPKRREKKLKQQHQDANLTMMSEESGIGNRFI  780
             GEIEPQSVEKLRSGDSAVQSSVPLASSMLPKRREKKLKQQHQDANLTMMSEESGIGNRFI
Sbjct  721   GEIEPQSVEKLRSGDSAVQSSVPLASSMLPKRREKKLKQQHQDANLTMMSEESGIGNRFI  780

Query  781   CSSVPLESSKRMARRQSFSKKGNDELDGVQNSLKTESMDRPDVIRREQDEPSHSTRFMIE  840
             CSSVPLESSKRMARRQSFSKKGNDELDGVQNSLKTESMDRPDVIRREQDEPSHSTRFMIE
Sbjct  781   CSSVPLESSKRMARRQSFSKKGNDELDGVQNSLKTESMDRPDVIRREQDEPSHSTRFMIE  840

Query  841   KQTSRNNSLSPDKSSPLRHLPSSHLIHSSFNSTSVTQSQESRSHLFSEDDEIAVPSSQLN  900
             KQTSRNNSLSPDKSSPLRHLPSSHLIHSSFNSTSVTQSQESRSHLFSEDDEIAVPSSQLN
Sbjct  841   KQTSRNNSLSPDKSSPLRHLPSSHLIHSSFNSTSVTQSQESRSHLFSEDDEIAVPSSQLN  900

Query  901   ERLSPLRRLGSQPMRPKNKLTSLRVPRFPLQSARPGLDIPSRPPPPAVESVKTISSRKRN  960
             ERLSPLRRLGSQPMRPKNKLTSLRVPRFPLQSARPGLDIPSRPPPPAVESVKTISSRKRN
Sbjct  901   ERLSPLRRLGSQPMRPKNKLTSLRVPRFPLQSARPGLDIPSRPPPPAVESVKTISSRKRN  960

Query  961   SSSWNFSPLIAHPLVSSQKATSRSSSPSALLFSPSSSQTPPAKRRAKTPNSQLDQPSFKD  1020
             SSSWNFSPLIAHPLVSSQKATSRSSSPSALLFSPSSSQTPPAKRRAKTPNSQLDQPSFKD
Sbjct  961   SSSWNFSPLIAHPLVSSQKATSRSSSPSALLFSPSSSQTPPAKRRAKTPNSQLDQPSFKD  1020

Query  1021  EKEPQQSSLLAHFRRTTIPNPKRLARLHGLDEELDDIQLSDEGNDEEATEMLKKENGEKN  1080
             EKEPQQSSLLAHFRRTTIPNPKRLARLHGLDEELDDIQLSDEGNDEEATEMLKKENGEKN
Sbjct  1021  EKEPQQSSLLAHFRRTTIPNPKRLARLHGLDEELDDIQLSDEGNDEEATEMLKKENGEKN  1080

Query  1081  SGNLLEPEVALTEPASSSEFVIPNSQPSPKNRIVLVQRKAQRVMGRVPSPPPRNILLPRL  1140
             SGNLLEPEVALTEPASSSEFVIPNSQPSPKNRIVLVQRKAQRVMGRVPSPPPRNILLPRL
Sbjct  1081  SGNLLEPEVALTEPASSSEFVIPNSQPSPKNRIVLVQRKAQRVMGRVPSPPPRNILLPRL  1140

Query  1141  DSKPIITRTSDSETDDQNEDENDIVPDSQFEEGKTILAGRTPYTSGGFSRMYGRAMMTGN  1200
             DSKPIITRTSDSETDDQNEDENDIVPDSQFEEGKTILAGRTPYTSGGFSRMYGRAMMTGN
Sbjct  1141  DSKPIITRTSDSETDDQNEDENDIVPDSQFEEGKTILAGRTPYTSGGFSRMYGRAMMTGN  1200

Query  1201  ISVKEPEGRFDRSVQEDRSSIRSNRPSMITMAEKNGSECIEEEGDTTIVPDEPPTDDDSD  1260
             ISVKEPEGRFDRSVQEDRSSIRSNRPSMITMAEKNGSECIEEEGDTTIVPDEPPTDDDSD
Sbjct  1201  ISVKEPEGRFDRSVQEDRSSIRSNRPSMITMAEKNGSECIEEEGDTTIVPDEPPTDDDSD  1260

Query  1261  PNVTVVPTQYYVVDEFGSTEPVLEASSLTSLSSDSMFVDTLTRRSDESISFWKFAAPSSD  1320
             PNVTVVPTQYYVVDEFGSTEPVLEASSLTSLSSDSMFVDTLTRRSDESISFWKFAAPSSD
Sbjct  1261  PNVTVVPTQYYVVDEFGSTEPVLEASSLTSLSSDSMFVDTLTRRSDESISFWKFAAPSSD  1320

Query  1321  DPST  1324
             DPST
Sbjct  1321  DPST  1324


Lambda      K        H        a         alpha
   0.308    0.124    0.345    0.792     4.96 

Gapped
Lambda      K        H        a         alpha    sigma
   0.267   0.0410    0.140     1.90     42.6     43.6 

Effective search space used: 16722138480348


  Database: nr
    Posted date:  Sep 23, 2015 12:05 AM
  Number of letters in database: 26,053,659,533
  Number of sequences in database:  71,551,133


Matrix: BLOSUM62
Gap Penalties: Existence: 11, Extension: 1
Neighboring words threshold: 11
Window for multiple hits: 40
```
