# Supplementary material for: Comparative genomics provides new insights into the diversity, physiology, and sexuality of the only industrially exploited tremellomycete: Phaffia rhodozyma
Source: BMC Genomics. 2016 Nov 9;17:901. doi: 10.1186/s12864-016-3244-7 (PMC5103461; doi:10.1186/s12864-016-3244-7)
Supplement: Additional file 6: — List of orphan genes with links to PFAM (related to Additional file 1: Table S1). (ZIP 1428 kb) [file 12864_2016_3244_MOESM6_ESM.zip › BLAST_HTML_FTR/G01210_P.html]

BLAST Search Results


```
BLASTP 2.2.27+


Reference:
Stephen F. Altschul, Thomas L. Madden, Alejandro A. Schäffer,
Jinghui Zhang, Zheng Zhang, Webb Miller, and David J. Lipman (1997),
"Gapped BLAST and PSI-BLAST: a new generation of protein database
search programs", Nucleic Acids Res. 25:3389-3402.


Reference for
composition-based statistics:
Alejandro A. Schäffer, L. Aravind, Thomas L. Madden, Sergei
Shavirin, John L. Spouge, Yuri I. Wolf, Eugene V. Koonin, and
Stephen F. Altschul (2001), "Improving the accuracy of PSI-BLAST
protein database searches with composition-based statistics and
other refinements", Nucleic Acids Res. 29:2994-3005.


Database: nr
           71,551,133 sequences; 26,053,659,533 total letters


Query= G01210_P

Length=260
                                                                      Score     E
Sequences producing significant alignments:                          (Bits)  Value

emb|CED82892.1|  hypothetical protein [Xanthophyllomyces dendrorh...   482    4e-170
gb|EMD39227.1|  hypothetical protein CERSUDRAFT_93275 [Ceriporiop...  40.4    1.3   
gb|EKC99920.1|  hypothetical protein A1Q2_05744 [Trichosporon asa...  40.4    1.3   
gb|EJT45964.1|  hypothetical protein A1Q1_05584 [Trichosporon asa...  40.4    1.3   
gb|KIK60449.1|  hypothetical protein GYMLUDRAFT_43760 [Gymnopus l...  40.0    1.4   
gb|EWM21533.1|  hypothetical protein Naga_100650g2 [Nannochlorops...  40.0    1.8   
gb|KEZ41383.1|  hypothetical protein SAPIO_CDS7506 [Scedosporium ...  39.7    2.8   
emb|CCX53937.1|  chromosome partition protein Smc [Veillonella sp...  39.3    3.9   
ref|WP_001284181.1|  membrane protein [Bacillus cereus] >gb|EJR46...  39.3    4.2   
gb|KDQ19035.1|  hypothetical protein BOTBODRAFT_126892 [Botryobas...  38.5    5.6   
gb|EPS94561.1|  hypothetical protein FOMPIDRAFT_1081423, partial ...  37.4    5.9   
ref|XP_013779584.1|  PREDICTED: ribosome maturation protein SBDS-...  38.1    6.3   
ref|WP_041060676.1|  histidine kinase [Vibrio owensii]                38.5    6.5   


 >emb|CED82892.1| hypothetical protein [Xanthophyllomyces dendrorhous]
Length=238

 Score =  482 bits (1240),  Expect = 4e-170, Method: Compositional matrix adjust.
 Identities = 238/238 (100%), Positives = 238/238 (100%), Gaps = 0/238 (0%)

Query  22   MSESSVAQEEALCFLTENELTRSGSDVSVTETDVSSAVRHALNESMAKSLQTFIRYLNPD  81
            MSESSVAQEEALCFLTENELTRSGSDVSVTETDVSSAVRHALNESMAKSLQTFIRYLNPD
Sbjct  1    MSESSVAQEEALCFLTENELTRSGSDVSVTETDVSSAVRHALNESMAKSLQTFIRYLNPD  60

Query  82   NSRACHPLNLVQQIADAQSTVRENESELDRLYESISSDRERVTQLISDVQALLANILGTW  141
            NSRACHPLNLVQQIADAQSTVRENESELDRLYESISSDRERVTQLISDVQALLANILGTW
Sbjct  61   NSRACHPLNLVQQIADAQSTVRENESELDRLYESISSDRERVTQLISDVQALLANILGTW  120

Query  142  PPIIFSAESSIHTNLLQKLSALLLKLSLLPLRQHDILLKSHPNLQAAMATYQETILRRTA  201
            PPIIFSAESSIHTNLLQKLSALLLKLSLLPLRQHDILLKSHPNLQAAMATYQETILRRTA
Sbjct  121  PPIIFSAESSIHTNLLQKLSALLLKLSLLPLRQHDILLKSHPNLQAAMATYQETILRRTA  180

Query  202  RAQKETDELKGEIRKYQMVTERSKGEFERLMRTKSGLDAARAKVELELEELRRAIMEH  259
            RAQKETDELKGEIRKYQMVTERSKGEFERLMRTKSGLDAARAKVELELEELRRAIMEH
Sbjct  181  RAQKETDELKGEIRKYQMVTERSKGEFERLMRTKSGLDAARAKVELELEELRRAIMEH  238


>gb|EMD39227.1| hypothetical protein CERSUDRAFT_93275 [Ceriporiopsis subvermispora 
B]
Length=282

 Score = 40.4 bits (93),  Expect = 1.3, Method: Compositional matrix adjust.
 Identities = 53/185 (29%), Positives = 79/185 (43%), Gaps = 25/185 (14%)

Query  89   LNLVQQIADAQSTVRENESELDRLYESISSDRERVTQLISDVQAL-------LANILGTW  141
             +++   +DA       ESEL +   +    R R+ QL+ +V  +       L   L T 
Sbjct  98   FSILDYWSDADQGTSTAESELKQQASNSREQRTRIQQLVEEVNDVHPRLEEDLRTALLTL  157

Query  142  PPIIFSAESSIHTNLLQKLSALLLKLSLLPLRQHDILLKSHPNLQAAMATYQETI-----  196
            P II ++  +    L   +   LLKLSL   R H + L SH +     AT  E +     
Sbjct  158  PSIINASRDAEADALALSIETSLLKLSLFRARTH-LALYSHTSPTDPRATMSEALSAVQA  216

Query  197  -LRRTARAQKET----DELKGEIRKYQMVTERSKGEFERLMRTKSGLDAARAKVELE--L  249
             L+  AR Q E     DE  GE      + ER  G +++++      D AR K E E   
Sbjct  217  HLQEKAREQAEEEARLDEAIGEYEAVMSLVERGGGGYKQVVE-----DMARVKRETEECR  271

Query  250  EELRR  254
            ++LRR
Sbjct  272  KDLRR  276


>gb|EKC99920.1| hypothetical protein A1Q2_05744 [Trichosporon asahii var. asahii 
CBS 8904]
Length=335

 Score = 40.4 bits (93),  Expect = 1.3, Method: Compositional matrix adjust.
 Identities = 28/86 (33%), Positives = 45/86 (52%), Gaps = 8/86 (9%)

Query  90   NLVQQIADAQSTVRENESELDRLYESIS----SDRERVTQLISDVQALLANILGTW--PP  143
            N +  IA   + V   + E + L+ SI+     DR  V  LI+ ++ALLA  L  W   P
Sbjct  160  NRIDPIATVVAEVEAKKDEANDLFRSINLAPNKDRRTVDALIAYIRALLA--LAPWYASP  217

Query  144  IIFSAESSIHTNLLQKLSALLLKLSL  169
            ++FS + +    + Q  SALLL +++
Sbjct  218  LVFSGKQAFDFGIAQLQSALLLNIAI  243


>gb|EJT45964.1| hypothetical protein A1Q1_05584 [Trichosporon asahii var. asahii 
CBS 2479]
Length=335

 Score = 40.4 bits (93),  Expect = 1.3, Method: Compositional matrix adjust.
 Identities = 28/86 (33%), Positives = 45/86 (52%), Gaps = 8/86 (9%)

Query  90   NLVQQIADAQSTVRENESELDRLYESIS----SDRERVTQLISDVQALLANILGTW--PP  143
            N +  IA   + V   + E + L+ SI+     DR  V  LI+ ++ALLA  L  W   P
Sbjct  160  NRIDPIATVVAEVEAKKDEANDLFRSINLAPNKDRRTVDALIAYIRALLA--LAPWYASP  217

Query  144  IIFSAESSIHTNLLQKLSALLLKLSL  169
            ++FS + +    + Q  SALLL +++
Sbjct  218  LVFSGKQAFDFGIAQLQSALLLNIAI  243


>gb|KIK60449.1| hypothetical protein GYMLUDRAFT_43760 [Gymnopus luxurians FD-317 
M1]
Length=249

 Score = 40.0 bits (92),  Expect = 1.4, Method: Compositional matrix adjust.
 Identities = 63/235 (27%), Positives = 110/235 (47%), Gaps = 26/235 (11%)

Query  36   LTENELTRSGSDVSVTETDVSSAVRHALNESM-AKSLQ------TFI-RYLNPDNSRACH  87
            L + EL RS + + VT +D SS V   ++ES+ A+ LQ      +FI  YL+  +S A  
Sbjct  15   LDDQELARSLTQLRVT-SDSSSLVDKLISESVEARLLQEIESRLSFISEYLSQHDSDA--  71

Query  88   PLNLVQQIADAQSTVRENESELDRLYESISSDRERVTQLISDVQALLANILGTWPPIIFS  147
              N++++I      + + ES++  ++ +      R+ +L + +QA L + + T PPI+  
Sbjct  72   --NVLERIHQELENILDLESQVSSIWSTPKDHITRINELHTRLQAELMDAMSTVPPILNE  129

Query  148  AESSIHTNLLQKLSALLLKLSLLPLRQHDILLKSHPNLQAAMATYQETILRRTARAQKET  207
               +        + A L+KLSL   + H  L     + Q   AT    +     + + E 
Sbjct  130  KRKADGALTAASIEASLVKLSLFRAQAHHKLYGFASDTQPD-ATMAHALSIAYDKLKDEA  188

Query  208  DELKGEIRKYQMVTERSKGEFERLMRTKSGLDAAR--------AKVELELEELRR  254
            D LK E    Q + ++ +  + RL+R   G ++ R         +VE E EE +R
Sbjct  189  DHLKEE---EQALDDQIEA-YARLIRLADGGNSGRFAQIVEDYVQVEKETEECKR  239


>gb|EWM21533.1| hypothetical protein Naga_100650g2 [Nannochloropsis gaditana]
Length=583

 Score = 40.0 bits (92),  Expect = 1.8, Method: Compositional matrix adjust.
 Identities = 27/62 (44%), Positives = 40/62 (65%), Gaps = 4/62 (6%)

Query  194  ETILRRTARAQKET-DELKGEIRKYQMVTERSKGEFERLMRTKSGLDAARAKVELELEEL  252
            E  LRRT +AQ +T   L+ E+R  +   E+ KG++ERL + K+GL+AA A+  L L+  
Sbjct  379  EEELRRTLKAQADTLQALRAELRDQE---EKWKGQYERLRKEKAGLEAAGAETGLALQWR  435

Query  253  RR  254
            RR
Sbjct  436  RR  437


>gb|KEZ41383.1| hypothetical protein SAPIO_CDS7506 [Scedosporium apiospermum]
Length=404

 Score = 39.7 bits (91),  Expect = 2.8, Method: Compositional matrix adjust.
 Identities = 33/116 (28%), Positives = 49/116 (42%), Gaps = 2/116 (2%)

Query  47   DVSVTETDVSSAV-RHALNESMAKSLQTFIRYLNPDNSRACHPLNLVQQIADAQSTVREN  105
            D   TE  ++ A+   AL E  A S  TF+ Y  P N R    L      A   S  R N
Sbjct  222  DYLTTELGIAPALLPSALLERYAMS-TTFLSYFGPPNPRFLEKLENAFPTATGPSHFRWN  280

Query  106  ESELDRLYESISSDRERVTQLISDVQALLANILGTWPPIIFSAESSIHTNLLQKLS  161
            ES ++ LY+     + R   +++     L  + G  PP+     +S   N ++KL 
Sbjct  281  ESHVEALYQIAQGIQHRAASILAAATISLLRVAGEVPPVPDVKSASPPRNGVKKLG  336


>emb|CCX53937.1| chromosome partition protein Smc [Veillonella sp. CAG:933]
Length=1183

 Score = 39.3 bits (90),  Expect = 3.9, Method: Compositional matrix adjust.
 Identities = 34/124 (27%), Positives = 59/124 (48%), Gaps = 12/124 (10%)

Query  113  YESISSDRERVTQLISDVQALLANILGTWPPIIFS---AESSIHTNLLQKLSAL------  163
            YE +   +    Q I+D Q+ +   +    P+       E  ++T L QKLS L      
Sbjct  818  YEQLKQQQSYGEQQINDWQSAIEQNVARMKPLQMQLDVREQQVNTELPQKLSDLEHAYEA  877

Query  164  LLKLSLLPLRQHDILLKSHPNLQAAMATY---QETILRRTARAQKETDELKGEIRKYQMV  220
              K +    +Q D L +SH   Q  +A Y   +ET+  R  R Q+   +++G++ KY+M 
Sbjct  878  QAKATAELAKQRDALYESHSEQQRTLADYSTERETLDTRQKRVQQRLVQMEGQLAKYEMN  937

Query  221  TERS  224
            +E++
Sbjct  938  SEQA  941


>ref|WP_001284181.1| membrane protein [Bacillus cereus]
 gb|EJR46414.1| hypothetical protein IIK_04216 [Bacillus cereus VD102]
 gb|KLA05157.1| hypothetical protein B4153_5838 [Bacillus cereus]
Length=1355

 Score = 39.3 bits (90),  Expect = 4.2, Method: Composition-based stats.
 Identities = 39/147 (27%), Positives = 65/147 (44%), Gaps = 12/147 (8%)

Query  17   LGRLAMSESSVAQEEALCFLTENELTRSGSDVSVTETDVSSAVRHALNESMAKSLQTFIR  76
            L RL    SS AQ+       E EL    + +       ++A+ H   ES  + L   + 
Sbjct  120  LQRLGQENSSAAQDLKKLGAAETELINQSAKLRAEYDFQNAALGHNATES--EKLSAKLH  177

Query  77   YLNPDNSRACHPL-NLVQQIADAQSTVRENESELDRLYESISSDRERVTQLISDVQALLA  135
            YL      A     N  QQ+A A+S   EN +E+++L   +   R    QL ++VQ   A
Sbjct  178  YLGQAQQNASQQTQNCAQQLAAAKSRYGENSAEVNKLETKLLQLRSAEQQLENEVQQ--A  235

Query  136  NILGTWPPIIFSAESSIHTNLLQKLSA  162
            N        + S ++++ + + +K+SA
Sbjct  236  N-------SVLSEQANVASKVSEKMSA  255


>gb|KDQ19035.1| hypothetical protein BOTBODRAFT_126892 [Botryobasidium botryosum 
FD-172 SS1]
Length=289

 Score = 38.5 bits (88),  Expect = 5.6, Method: Compositional matrix adjust.
 Identities = 44/161 (27%), Positives = 69/161 (43%), Gaps = 10/161 (6%)

Query  90   NLVQQIADAQSTVRENESELDRLYESISSDRERVTQLISDVQALLANILGTWPPIIFSAE  149
            ++   I   Q+T RE   ++  + E I    E+  ++   +Q  LA IL T+PPI+ +  
Sbjct  109  DVAGDIEKEQATQRETVKDISLVVERILELVEQTNEIHPRLQESLALILKTYPPILRANR  168

Query  150  SSIHTNLLQKLSALLLKLSLLPLRQHDILLKSHPNLQAAMATYQETILRRTARAQKETDE  209
            S  H  L   +   LLKLSL+       L    P+  AA +    T+ +  AR      E
Sbjct  169  SIYHDLLSLTIETALLKLSLMRSTTATSLYNYTPSADAASSQPSRTVSQTLARIHARLSE  228

Query  210  LKGEIRKYQMVTERSKGE-----FERLMRTKSGLDAARAKV  245
                 RK QM  E +  +     + RL++   G     A+V
Sbjct  229  -----RKLQMDAEEAALDERLESYTRLIKLVDGPQGGFAQV  264


>gb|EPS94561.1| hypothetical protein FOMPIDRAFT_1081423, partial [Fomitopsis 
pinicola FP-58527 SS1]
Length=153

 Score = 37.4 bits (85),  Expect = 5.9, Method: Compositional matrix adjust.
 Identities = 38/125 (30%), Positives = 56/125 (45%), Gaps = 19/125 (15%)

Query  120  RERVTQLISDVQAL-------LANILGTWPPIIFSAESSIHTNLLQKLSALLLKLSLLPL  172
            RE +TQL+ D+  L       L + L T PPI+ +  ++    L   + A L+KLSL+  
Sbjct  29   REEITQLVQDINQLHPTLDEELIDALSTLPPILNATRTAEADLLSTTIEASLMKLSLIRT  88

Query  173  RQHDILLKSHPNLQAAMATYQETI------LRRTARAQ-KETDELKGEIRKYQ----MVT  221
            R H + L  H +     AT    +      LR   RAQ  E  EL  ++  Y+    +V 
Sbjct  89   RTH-VALYGHTSPSRPQATMGRALSVAVDKLRTKQRAQADEEHELDAQLAAYESMLSLVG  147

Query  222  ERSKG  226
             R  G
Sbjct  148  GREGG  152


>ref|XP_013779584.1| PREDICTED: ribosome maturation protein SBDS-like [Limulus polyphemus]
 ref|XP_013779585.1| PREDICTED: ribosome maturation protein SBDS-like [Limulus polyphemus]
Length=249

 Score = 38.1 bits (87),  Expect = 6.3, Method: Compositional matrix adjust.
 Identities = 24/77 (31%), Positives = 44/77 (57%), Gaps = 5/77 (6%)

Query  22   MSESSVAQEEAL--CFLTENELTRSGSDVSVTETDVSSAVRHALNESMAKSLQTFI--RY  77
            +S+  VA++E L  CF TEN++      ++  E  VS   R A  ESM K + T +  + 
Sbjct  60   VSKGQVAKKEDLIKCFKTENQVEICKEILAKGELQVSEKERQAQLESMFKDIATIVSDKC  119

Query  78   LNPDNSRACHPLNLVQQ  94
            +NP+  R  +P++++++
Sbjct  120  INPETKRP-YPVSMIEK  135


>ref|WP_041060676.1| histidine kinase [Vibrio owensii]
Length=1187

 Score = 38.5 bits (88),  Expect = 6.5, Method: Compositional matrix adjust.
 Identities = 37/135 (27%), Positives = 68/135 (50%), Gaps = 21/135 (16%)

Query  52   ETDVSSAVRHA-----LNESMAKSLQ-----TFIRYLNPDNSRACHPLNLVQQIADAQST  101
            ++D++ A+++      L E+++++L      T ++ L     R    L+LV Q AD ++T
Sbjct  205  DSDLNDAIKYTDELEQLTETLSEALHNKYELTLVQALKNSQKRYSSALSLVNQSADNEAT  264

Query  102  VRENESELDRLYESISSDRERVTQLISDVQALLANILGTWPPIIFSAESSIHTNLLQKLS  161
             +   S  ++L  S+ + RER+ Q +   QA +AN+           ES ++ +L   + 
Sbjct  265  SKNLRSSSEQLARSVDALRERIRQDLIQAQANVANL-----------ESDMNMSLEAGIQ  313

Query  162  ALLLKLSLLPLRQHD  176
            A LLK S+   RQ D
Sbjct  314  ATLLKQSVGKARQAD  328


Lambda      K        H        a         alpha
   0.317    0.128    0.343    0.792     4.96 

Gapped
Lambda      K        H        a         alpha    sigma
   0.267   0.0410    0.140     1.90     42.6     43.6 

Effective search space used: 1755527656966


  Database: nr
    Posted date:  Sep 23, 2015 12:05 AM
  Number of letters in database: 26,053,659,533
  Number of sequences in database:  71,551,133


Matrix: BLOSUM62
Gap Penalties: Existence: 11, Extension: 1
Neighboring words threshold: 11
Window for multiple hits: 40
```
