# Supplementary material for: Comparative genomics provides new insights into the diversity, physiology, and sexuality of the only industrially exploited tremellomycete: Phaffia rhodozyma
Source: BMC Genomics. 2016 Nov 9;17:901. doi: 10.1186/s12864-016-3244-7 (PMC5103461; doi:10.1186/s12864-016-3244-7)
Supplement: Additional file 6: — List of orphan genes with links to PFAM (related to Additional file 1: Table S1). (ZIP 1428 kb) [file 12864_2016_3244_MOESM6_ESM.zip › BLAST_HTML_FTR/G04829_P.html]

BLAST Search Results


```
BLASTP 2.2.27+


Reference:
Stephen F. Altschul, Thomas L. Madden, Alejandro A. Schäffer,
Jinghui Zhang, Zheng Zhang, Webb Miller, and David J. Lipman (1997),
"Gapped BLAST and PSI-BLAST: a new generation of protein database
search programs", Nucleic Acids Res. 25:3389-3402.


Reference for
composition-based statistics:
Alejandro A. Schäffer, L. Aravind, Thomas L. Madden, Sergei
Shavirin, John L. Spouge, Yuri I. Wolf, Eugene V. Koonin, and
Stephen F. Altschul (2001), "Improving the accuracy of PSI-BLAST
protein database searches with composition-based statistics and
other refinements", Nucleic Acids Res. 29:2994-3005.


Database: nr
           71,551,133 sequences; 26,053,659,533 total letters


Query= G04829_P

Length=795
                                                                      Score     E
Sequences producing significant alignments:                          (Bits)  Value

emb|CDZ98768.1|  hypothetical protein [Xanthophyllomyces dendrorh...  1338    0.0  
emb|CDZ98853.1|  Glia maturation factor beta [Xanthophyllomyces d...  55.1    4e-04


 >emb|CDZ98768.1| hypothetical protein [Xanthophyllomyces dendrorhous]
Length=767

 Score = 1338 bits (3462),  Expect = 0.0, Method: Compositional matrix adjust.
 Identities = 753/808 (93%), Positives = 753/808 (93%), Gaps = 55/808 (7%)

Query  1    MSVPSPIIIQELIDQLLPTAPSTSAPLHLLPKLQGLVSLLRYWNAHLCSEIRRASRAKSI  60
            MSVPSPIIIQELIDQLLPTAPSTSAPLHLLPKLQGLVSLLRYWNAHLCSEIRRASRAKSI
Sbjct  1    MSVPSPIIIQELIDQLLPTAPSTSAPLHLLPKLQGLVSLLRYWNAHLCSEIRRASRAKSI  60

Query  61   QWEKLEEKDRRILKRMSNVRCEKDLDEKGEAFLLILASWH--------------SLRLVN  106
            QWEKLEEKDRRILKRMSNVRCEKDLDEKGEAFLLILASWH              SLRLVN
Sbjct  61   QWEKLEEKDRRILKRMSNVRCEKDLDEKGEAFLLILASWHIQHHLMTNPRAHQHSLRLVN  120

Query  107  LWFPPSPLPSSYFIGEPSVLDPKTVHATNTKFAKFRTNALVKVQGVRKIINRTEGSVESL  166
            LWFPPSPLPSSYFIGEPSVLDPKTVHATNTKFAKFRTNALVKVQGVRKIINRTEGSVESL
Sbjct  121  LWFPPSPLPSSYFIGEPSVLDPKTVHATNTKFAKFRTNALVKVQGVRKIINRTEGSVESL  180

Query  167  AKALRGEAEAPTTSFLRFDLRLQWESESTGLSNWAKKVVQLWMLMDRLFPSLPEEALSKT  226
            AKALRGEAEAPTTSFLRFDLRLQWESESTGLSNWAKKVVQLWMLMDRLFPSLPEEALSKT
Sbjct  181  AKALRGEAEAPTTSFLRFDLRLQWESESTGLSNWAKKVVQLWMLMDRLFPSLPEEALSKT  240

Query  227  SPHREEVKNIPIHTSVSVPHRVISDSTILSFSTPDHSIPPQQRSGYPHTNDSTYTPSTHL  286
            SPHREEVKNIPIHTSVSVPHRVISDSTILSFSTPDHSIPPQQRSGYPHTNDSTYTPSTHL
Sbjct  241  SPHREEVKNIPIHTSVSVPHRVISDSTILSFSTPDHSIPPQQRSGYPHTNDSTYTPSTHL  300

Query  287  SLPTTSTKTSPLPIRSNDIITAHAESISDLESRSRHFPSSSSLSISPFIKPQATASLDDF  346
            SLPTTSTKTSPLPIRSNDIITAHAESISDLESRSRHFPSSSSLSISPFIKPQATASLDDF
Sbjct  301  SLPTTSTKTSPLPIRSNDIITAHAESISDLESRSRHFPSSSSLSISPFIKPQATASLDDF  360

Query  347  VADSQPFDSLPPSYSFKKTNTTDISLENISDIEIISPRATSRVQPVKPLISSPPSTSYLV  406
            VADSQPFDSLPPSYSFKKTNTTDISLENISDIEIISPRATSRVQPVKPLISSPPST    
Sbjct  361  VADSQPFDSLPPSYSFKKTNTTDISLENISDIEIISPRATSRVQPVKPLISSPPST----  416

Query  407  HSDSRSRELFHSPRAPPPPPPQQQTAAAHRKSLKDARTLGSRRKSGGRPRLSFEPDFDLS  466
                                                 TLGSRRKSGGRPRLSFEPDFDLS
Sbjct  417  -------------------------------------TLGSRRKSGGRPRLSFEPDFDLS  439

Query  467  VIPPEPQSNVKPRLYPEGTADSSSKGLPVNTVSAIRDARPPVDHKDTLPRGQQRAIELIS  526
            VIPPEPQSNVKPRLYPEGTADSSSKGLPVNTVSAIRDARPPVDHKDTLPRGQQRAIELIS
Sbjct  440  VIPPEPQSNVKPRLYPEGTADSSSKGLPVNTVSAIRDARPPVDHKDTLPRGQQRAIELIS  499

Query  527  SSPIDDEAEDDESLPVADSQEIIDDKPANEQYLDSVSISPARSLPRKAATAFSFPDLSPR  586
            SSPIDDEAEDDESLPVADSQEIIDDKPANEQYLDSVSISPARSLPRKAATAFSFPDLSPR
Sbjct  500  SSPIDDEAEDDESLPVADSQEIIDDKPANEQYLDSVSISPARSLPRKAATAFSFPDLSPR  559

Query  587  MKRAHRSLLYLPNHHGIYSSSSGSDSDPISDSSDDDSQDDLVGLNHRTIVRASGTNSIGQ  646
            MKRAHRSLLYLPNHHGIYSSSSGSDSDPISDSSDDDSQDDLVGLNHRTIVRASGTNSIGQ
Sbjct  560  MKRAHRSLLYLPNHHGIYSSSSGSDSDPISDSSDDDSQDDLVGLNHRTIVRASGTNSIGQ  619

Query  647  ITRQAEVISKRKRDTIKDESEESEDGEERFRLRNQMKIQRLDLKVEEKNPDNQLRVFTQG  706
            ITRQAEVISKRKRDTIKDESEESEDGEERFRLRNQMKIQRLDLKVEEKNPDNQLRVFTQG
Sbjct  620  ITRQAEVISKRKRDTIKDESEESEDGEERFRLRNQMKIQRLDLKVEEKNPDNQLRVFTQG  679

Query  707  GRAGSLEQSTSLTTSSSAFDSEILDSSFSAGGRDSESESAVSSLIADDPTYSSSDREEDT  766
            GRAGSLEQSTSLTTSSSAFDSEILDSSFSAGGRDSESESAVSSLIADDPTYSSSDREEDT
Sbjct  680  GRAGSLEQSTSLTTSSSAFDSEILDSSFSAGGRDSESESAVSSLIADDPTYSSSDREEDT  739

Query  767  TATTTITVVTGSIEVQDSQEMNSSLGYM  794
            TATTTITVVTGSIEVQDSQEMNSSLGYM
Sbjct  740  TATTTITVVTGSIEVQDSQEMNSSLGYM  767


>emb|CDZ98853.1| Glia maturation factor beta [Xanthophyllomyces dendrorhous]
Length=1176

 Score = 55.1 bits (131),  Expect = 4e-04, Method: Compositional matrix adjust.
 Identities = 54/228 (24%), Positives = 100/228 (44%), Gaps = 16/228 (7%)

Query  7    IIIQELIDQLLPTAPSTSAPLHLLPKLQGLVSLLRYWNAHLCSEIRRA--SRAKSIQWEK  64
            ++   LI  L      T  P +L  ++  L+SLLR ++  L ++I+ A  + A+    E 
Sbjct  731  LVFLTLIHVLPVLYSDTHMPTYLFQQVPPLISLLRAYHNALNNQIQEAILAEARGEMPEG  790

Query  65   LEEKDRRILKRMSNVRCEKD--LDEKGEAFLLILASWHSLRLVNLWFPPSPLPSSYFIGE  122
              +K+R+ L  +   +C+ D      GE + ++   WH + ++ +     P   S  + +
Sbjct  791  WSQKERKKLWNLKE-KCKLDPRFKRPGE-YEIMFKYWHQMTMMLV----KPHQVSAIVDQ  844

Query  123  PSVLDPKTVHATNTKFAKFRTNALVKVQGVRKIINRTEGSVESLAKAL-RGEAEAPTTSF  181
             +   P+  H  N+K    R      ++ V + +   +G   +L+ AL       P +S 
Sbjct  845  LNAHFPQ--HLENSKTRPVRHVLEQMLKQVVQFVQSYKGPPGNLSSALYNARITVPASSI  902

Query  182  LRFDLRLQ---WESESTGLSNWAKKVVQLWMLMDRLFPSLPEEALSKT  226
             +    L    W   S  + +WA++V + W  M  LFP  P +A S T
Sbjct  903  NKHPATLTFPLWTENSWAIVHWAQRVHEGWQTMRTLFPDEPVQATSHT  950


Lambda      K        H        a         alpha
   0.312    0.128    0.360    0.792     4.96 

Gapped
Lambda      K        H        a         alpha    sigma
   0.267   0.0410    0.140     1.90     42.6     43.6 

Effective search space used: 9334590689496


  Database: nr
    Posted date:  Sep 23, 2015 12:05 AM
  Number of letters in database: 26,053,659,533
  Number of sequences in database:  71,551,133


Matrix: BLOSUM62
Gap Penalties: Existence: 11, Extension: 1
Neighboring words threshold: 11
Window for multiple hits: 40
```
