# Supplementary material for: Comparative genomics provides new insights into the diversity, physiology, and sexuality of the only industrially exploited tremellomycete: Phaffia rhodozyma
Source: BMC Genomics. 2016 Nov 9;17:901. doi: 10.1186/s12864-016-3244-7 (PMC5103461; doi:10.1186/s12864-016-3244-7)
Supplement: Additional file 6: — List of orphan genes with links to PFAM (related to Additional file 1: Table S1). (ZIP 1428 kb) [file 12864_2016_3244_MOESM6_ESM.zip › BLAST_HTML_FTR/G02259_P.html]

BLAST Search Results


```
BLASTP 2.2.27+


Reference:
Stephen F. Altschul, Thomas L. Madden, Alejandro A. Schäffer,
Jinghui Zhang, Zheng Zhang, Webb Miller, and David J. Lipman (1997),
"Gapped BLAST and PSI-BLAST: a new generation of protein database
search programs", Nucleic Acids Res. 25:3389-3402.


Reference for
composition-based statistics:
Alejandro A. Schäffer, L. Aravind, Thomas L. Madden, Sergei
Shavirin, John L. Spouge, Yuri I. Wolf, Eugene V. Koonin, and
Stephen F. Altschul (2001), "Improving the accuracy of PSI-BLAST
protein database searches with composition-based statistics and
other refinements", Nucleic Acids Res. 29:2994-3005.


Database: nr
           71,551,133 sequences; 26,053,659,533 total letters


Query= G02259_P

Length=136
                                                                      Score     E
Sequences producing significant alignments:                          (Bits)  Value

emb|CED82024.1|  hypothetical protein [Xanthophyllomyces dendrorh...   266    4e-88
gb|ELU10278.1|  hypothetical protein CAPTEDRAFT_201255 [Capitella...  38.1    1.2  
ref|WP_008418524.1|  ATP synthase subunit I [Halalkalicoccus jeot...  37.7    1.9  
ref|WP_052535339.1|  hypothetical protein [Candidatus Phaeomarino...  37.4    2.5  
emb|CDX54043.1|  hypothetical protein MPL1032_180320 [Mesorhizobi...  36.6    4.4  
emb|CDX54278.1|  hypothetical protein MPL3365_190151 [Mesorhizobi...  36.6    5.8  
emb|CDX19759.1|  hypothetical protein MPL3356_300107 [Mesorhizobi...  36.2    7.8  


 >emb|CED82024.1| hypothetical protein [Xanthophyllomyces dendrorhous]
Length=164

 Score =  266 bits (679),  Expect = 4e-88, Method: Compositional matrix adjust.
 Identities = 135/135 (100%), Positives = 135/135 (100%), Gaps = 0/135 (0%)

Query  1    MTPVPTTPIKAEEPTDALSSALSALSFIPLDSPPLSDRPALLPSLSSSSSIELTATSSST  60
            MTPVPTTPIKAEEPTDALSSALSALSFIPLDSPPLSDRPALLPSLSSSSSIELTATSSST
Sbjct  30   MTPVPTTPIKAEEPTDALSSALSALSFIPLDSPPLSDRPALLPSLSSSSSIELTATSSST  89

Query  61   GSSSCSDCSDCARKSELRDIEEIHLDLIAQANGKAHGGFPGFPLGSVGQGATAAAGVGGR  120
            GSSSCSDCSDCARKSELRDIEEIHLDLIAQANGKAHGGFPGFPLGSVGQGATAAAGVGGR
Sbjct  90   GSSSCSDCSDCARKSELRDIEEIHLDLIAQANGKAHGGFPGFPLGSVGQGATAAAGVGGR  149

Query  121  ARKRREGKKTTDEST  135
            ARKRREGKKTTDEST
Sbjct  150  ARKRREGKKTTDEST  164


>gb|ELU10278.1| hypothetical protein CAPTEDRAFT_201255 [Capitella teleta]
Length=229

 Score = 38.1 bits (87),  Expect = 1.2, Method: Compositional matrix adjust.
 Identities = 22/57 (39%), Positives = 30/57 (53%), Gaps = 1/57 (2%)

Query  69   SDCARKSELRDIEEIHLDLIAQANGKAHGGFPGFPLGSVGQGATAAAGVGGRARKRR  125
            S+C R+ E R  + IHL+   QA+GK    F G  +G VG+  T    + G A  RR
Sbjct  8    SNCRREIEKRKRQGIHLEFEKQADGKMDSHF-GLEIGKVGKRRTLTRALTGAASTRR  63


>ref|WP_008418524.1| ATP synthase subunit I [Halalkalicoccus jeotgali]
 gb|ADJ13771.1| V-type ATPase 116 kDa subunit [Halalkalicoccus jeotgali B3]
 gb|ELY34183.1| V-type ATPase 116 kDa subunit [Halalkalicoccus jeotgali B3]
Length=747

 Score = 37.7 bits (86),  Expect = 1.9, Method: Composition-based stats.
 Identities = 22/67 (33%), Positives = 32/67 (48%), Gaps = 0/67 (0%)

Query  69   SDCARKSELRDIEEIHLDLIAQANGKAHGGFPGFPLGSVGQGATAAAGVGGRARKRREGK  128
            +D     E  ++E +  D +A  +G AHG  P        Q ATAA G G  A  R +G 
Sbjct  294  ADLGEHVECEELERVDYDDVASGHGHAHGEDPSDAGDGDRQSATAADGEGQSAEVRPDGG  353

Query  129  KTTDEST  135
            ++  ES+
Sbjct  354  RSASESS  360


>ref|WP_052535339.1| hypothetical protein [Candidatus Phaeomarinobacter ectocarpi]
 emb|CDO59289.1| Para-aminobenzoate synthase, aminase component [Candidatus Phaeomarinobacter 
ectocarpi]
Length=454

 Score = 37.4 bits (85),  Expect = 2.5, Method: Compositional matrix adjust.
 Identities = 32/84 (38%), Positives = 37/84 (44%), Gaps = 10/84 (12%)

Query  14   PTDALSSALSALSFIPLDSPPLSDRPALLPSLSSSSSIELTATSSSTGSSSCSDCS----  69
            P DA   A  A    P D+P   DR A L +L     IE  + S +  +  CSD      
Sbjct  129  PQDANKDADHAFVLSPWDTPVTQDRRAALKAL-----IETASRSRAPATRPCSDPEATID  183

Query  70   -DCARKSELRDIEEIHLDLIAQAN  92
             D  RKS  R IE IH   I QAN
Sbjct  184  PDAYRKSVARVIELIHAGDIFQAN  207


>emb|CDX54043.1| hypothetical protein MPL1032_180320 [Mesorhizobium plurifarium]
Length=520

 Score = 36.6 bits (83),  Expect = 4.4, Method: Composition-based stats.
 Identities = 23/55 (42%), Positives = 29/55 (53%), Gaps = 6/55 (11%)

Query  77   LRDIEEIHLDLIAQANGKAHGGFPG----FPLGSVGQGATAAAGVGGRARKRREG  127
            +RD++  HL  I  A+G  H GF G      LG V  GA A AGV  R  +R +G
Sbjct  292  VRDVD--HLQPITHAHGDGHAGFEGQGLHQRLGHVDHGAAAQAGVAERYGRRSDG  344


>emb|CDX54278.1| hypothetical protein MPL3365_190151 [Mesorhizobium plurifarium]
Length=520

 Score = 36.6 bits (83),  Expect = 5.8, Method: Composition-based stats.
 Identities = 23/55 (42%), Positives = 29/55 (53%), Gaps = 6/55 (11%)

Query  77   LRDIEEIHLDLIAQANGKAHGGFPG----FPLGSVGQGATAAAGVGGRARKRREG  127
            +RD++  HL  I  A+G  H GF G      LG V  GA A AGV  R  +R +G
Sbjct  292  VRDVD--HLQPITHAHGDGHAGFEGQGLHQRLGHVDHGAAAQAGVAERYGRRPDG  344


>emb|CDX19759.1| hypothetical protein MPL3356_300107 [Mesorhizobium plurifarium]
Length=520

 Score = 36.2 bits (82),  Expect = 7.8, Method: Composition-based stats.
 Identities = 23/55 (42%), Positives = 29/55 (53%), Gaps = 6/55 (11%)

Query  77   LRDIEEIHLDLIAQANGKAHGGFPG----FPLGSVGQGATAAAGVGGRARKRREG  127
            +RD++  HL  I  A+G  H GF G      LG V  GA A AGV  R  +R +G
Sbjct  292  VRDVD--HLQPITHAHGDGHAGFEGQGLHQRLGHVDHGAAAQAGVAERYGRRPDG  344


Lambda      K        H        a         alpha
   0.312    0.128    0.363    0.792     4.96 

Gapped
Lambda      K        H        a         alpha    sigma
   0.267   0.0410    0.140     1.90     42.6     43.6 

Effective search space used: 637685094878


  Database: nr
    Posted date:  Sep 23, 2015 12:05 AM
  Number of letters in database: 26,053,659,533
  Number of sequences in database:  71,551,133


Matrix: BLOSUM62
Gap Penalties: Existence: 11, Extension: 1
Neighboring words threshold: 11
Window for multiple hits: 40
```
