# Supplementary material for: Comparative genomics provides new insights into the diversity, physiology, and sexuality of the only industrially exploited tremellomycete: Phaffia rhodozyma
Source: BMC Genomics. 2016 Nov 9;17:901. doi: 10.1186/s12864-016-3244-7 (PMC5103461; doi:10.1186/s12864-016-3244-7)
Supplement: Additional file 6: — List of orphan genes with links to PFAM (related to Additional file 1: Table S1). (ZIP 1428 kb) [file 12864_2016_3244_MOESM6_ESM.zip › BLAST_HTML_FTR/G01299_P.html]

BLAST Search Results


```
BLASTP 2.2.27+


Reference:
Stephen F. Altschul, Thomas L. Madden, Alejandro A. Schäffer,
Jinghui Zhang, Zheng Zhang, Webb Miller, and David J. Lipman (1997),
"Gapped BLAST and PSI-BLAST: a new generation of protein database
search programs", Nucleic Acids Res. 25:3389-3402.


Reference for
composition-based statistics:
Alejandro A. Schäffer, L. Aravind, Thomas L. Madden, Sergei
Shavirin, John L. Spouge, Yuri I. Wolf, Eugene V. Koonin, and
Stephen F. Altschul (2001), "Improving the accuracy of PSI-BLAST
protein database searches with composition-based statistics and
other refinements", Nucleic Acids Res. 29:2994-3005.


Database: nr
           71,551,133 sequences; 26,053,659,533 total letters


Query= G01299_P

Length=200
                                                                      Score     E
Sequences producing significant alignments:                          (Bits)  Value

emb|CDZ98480.1|  hypothetical protein [Xanthophyllomyces dendrorh...   221    5e-70
gb|KGB78804.1|  NADH-ubiquinone oxidoreductase subunit 8 [Cryptoc...  40.4    0.49 
gb|KIR85185.1|  NADH-ubiquinone oxidoreductase subunit 8, partial...  40.8    0.53 
ref|XP_003193382.1|  NADH-ubiquinone oxidoreductase 23 kDa subuni...  40.8    0.53 
gb|KIR46369.1|  NADH-ubiquinone oxidoreductase subunit 8 [Cryptoc...  40.8    0.54 
ref|XP_012048681.1|  NADH-ubiquinone oxidoreductase subunit 8 [Cr...  39.7    0.97 
dbj|GAN08523.1|  NADH-ubiquinone oxidoreductase 23 kda subunit pr...  39.7    1.1  
ref|WP_040254381.1|  hypothetical protein [Psychroserpens mesophi...  39.3    1.2  
ref|XP_568110.1|  hypothetical protein [Cryptococcus neoformans v...  39.3    1.5  


 >emb|CDZ98480.1| hypothetical protein [Xanthophyllomyces dendrorhous]
Length=124

 Score =  221 bits (562),  Expect = 5e-70, Method: Compositional matrix adjust.
 Identities = 107/107 (100%), Positives = 107/107 (100%), Gaps = 0/107 (0%)

Query  1    MNNLPRTSLRALRASSTTTATILGQTSIWNQLPRRTFLTSSASRAGLKNLHQPIVHTVIP  60
            MNNLPRTSLRALRASSTTTATILGQTSIWNQLPRRTFLTSSASRAGLKNLHQPIVHTVIP
Sbjct  1    MNNLPRTSLRALRASSTTTATILGQTSIWNQLPRRTFLTSSASRAGLKNLHQPIVHTVIP  60

Query  61   PSKEPITGPTEIVPGYAKVGYGVHPVADYDKGLSVMDKSMHLFFFGE  107
            PSKEPITGPTEIVPGYAKVGYGVHPVADYDKGLSVMDKSMHLFFFGE
Sbjct  61   PSKEPITGPTEIVPGYAKVGYGVHPVADYDKGLSVMDKSMHLFFFGE  107


>gb|KGB78804.1| NADH-ubiquinone oxidoreductase subunit 8 [Cryptococcus gattii 
R265]
 gb|KIR25220.1| NADH-ubiquinone oxidoreductase subunit 8 [Cryptococcus gattii 
LA55]
 gb|KIR36590.1| NADH-ubiquinone oxidoreductase subunit 8 [Cryptococcus gattii 
MMRL2647]
 gb|KIR38993.1| NADH-ubiquinone oxidoreductase subunit 8 [Cryptococcus gattii 
Ram5]
 gb|KIR76020.1| NADH-ubiquinone oxidoreductase subunit 8 [Cryptococcus gattii 
CA1014]
 gb|KIR95963.1| NADH-ubiquinone oxidoreductase subunit 8 [Cryptococcus gattii 
CBS 10090]
 gb|KIS02459.1| NADH-ubiquinone oxidoreductase subunit 8 [Cryptococcus gattii 
2001/935-1]
 gb|KIY58906.1| NADH-ubiquinone oxidoreductase subunit 8 [Cryptococcus gattii 
99/473]
Length=211

 Score = 40.4 bits (93),  Expect = 0.49, Method: Compositional matrix adjust.
 Identities = 23/60 (38%), Positives = 32/60 (53%), Gaps = 16/60 (27%)

Query  139  QKFPTHST--SSIDYVHQILFQSDPNLSLLYVSLVVSRILRGAWVVFEQMFRTSFHMATP  196
            QK+P +ST  S+ID   Q+LF              ++ I+RG WVVFEQ FR  + +  P
Sbjct  65   QKYPDYSTGPSAIDKASQLLF--------------LTEIVRGMWVVFEQFFRPPYTIMYP  110


>gb|KIR85185.1| NADH-ubiquinone oxidoreductase subunit 8, partial [Cryptococcus 
gattii IND107]
Length=281

 Score = 40.8 bits (94),  Expect = 0.53, Method: Compositional matrix adjust.
 Identities = 23/60 (38%), Positives = 32/60 (53%), Gaps = 16/60 (27%)

Query  139  QKFPTHST--SSIDYVHQILFQSDPNLSLLYVSLVVSRILRGAWVVFEQMFRTSFHMATP  196
            QK+P +ST  S+ID   Q+LF              ++ I+RG WVVFEQ FR  + +  P
Sbjct  65   QKYPDYSTGPSAIDKASQLLF--------------LTEIVRGMWVVFEQFFRPPYTIMYP  110


>ref|XP_003193382.1| NADH-ubiquinone oxidoreductase 23 kDa subunit, mitochondrial 
precursor [Cryptococcus gattii WM276]
 gb|ADV21595.1| NADH-ubiquinone oxidoreductase 23 kDa subunit, mitochondrial 
precursor, putative [Cryptococcus gattii WM276]
 gb|KIR53901.1| NADH-ubiquinone oxidoreductase subunit 8 [Cryptococcus gattii 
Ru294]
 gb|KIR81151.1| NADH-ubiquinone oxidoreductase subunit 8 [Cryptococcus gattii 
EJB2]
 gb|KIY34101.1| NADH-ubiquinone oxidoreductase subunit 8 [Cryptococcus gattii 
E566]
 gb|KJE03770.1| NADH-ubiquinone oxidoreductase subunit 8 [Cryptococcus gattii 
NT-10]
Length=280

 Score = 40.8 bits (94),  Expect = 0.53, Method: Compositional matrix adjust.
 Identities = 23/60 (38%), Positives = 32/60 (53%), Gaps = 16/60 (27%)

Query  139  QKFPTHST--SSIDYVHQILFQSDPNLSLLYVSLVVSRILRGAWVVFEQMFRTSFHMATP  196
            QK+P +ST  S+ID   Q+LF              ++ I+RG WVVFEQ FR  + +  P
Sbjct  65   QKYPDYSTGPSAIDKASQLLF--------------LTEIVRGMWVVFEQFFRPPYTIMYP  110


>gb|KIR46369.1| NADH-ubiquinone oxidoreductase subunit 8 [Cryptococcus gattii 
CA1280]
 gb|KIR59547.1| NADH-ubiquinone oxidoreductase subunit 8 [Cryptococcus gattii 
CA1873]
Length=280

 Score = 40.8 bits (94),  Expect = 0.54, Method: Compositional matrix adjust.
 Identities = 23/60 (38%), Positives = 32/60 (53%), Gaps = 16/60 (27%)

Query  139  QKFPTHST--SSIDYVHQILFQSDPNLSLLYVSLVVSRILRGAWVVFEQMFRTSFHMATP  196
            QK+P +ST  S+ID   Q+LF              ++ I+RG WVVFEQ FR  + +  P
Sbjct  65   QKYPDYSTGPSAIDKASQLLF--------------LTEIVRGMWVVFEQFFRPPYTIMYP  110


>ref|XP_012048681.1| NADH-ubiquinone oxidoreductase subunit 8 [Cryptococcus neoformans 
var. grubii H99]
 gb|AFR94306.1| NADH-ubiquinone oxidoreductase subunit 8 [Cryptococcus neoformans 
var. grubii H99]
Length=235

 Score = 39.7 bits (91),  Expect = 0.97, Method: Compositional matrix adjust.
 Identities = 23/60 (38%), Positives = 31/60 (52%), Gaps = 16/60 (27%)

Query  139  QKFPTHST--SSIDYVHQILFQSDPNLSLLYVSLVVSRILRGAWVVFEQMFRTSFHMATP  196
            QK+P +ST  S+ID   QILF              ++ I+RG WVV EQ FR  + +  P
Sbjct  65   QKYPDYSTGPSAIDKASQILF--------------LTEIVRGMWVVLEQFFRPPYTIMYP  110


>dbj|GAN08523.1| NADH-ubiquinone oxidoreductase 23 kda subunit protein [Mucor 
ambiguus]
Length=220

 Score = 39.7 bits (91),  Expect = 1.1, Method: Compositional matrix adjust.
 Identities = 18/57 (32%), Positives = 30/57 (53%), Gaps = 0/57 (0%)

Query  140  KFPTHSTSSIDYVHQILFQSDPNLSLLYVSLVVSRILRGAWVVFEQMFRTSFHMATP  196
            +F  H+ S+ D  HQI  +    L +    L+++ ++RG WVV E  FR  + +  P
Sbjct  39   RFAQHAASTKDISHQISGKPQGALDIAAQHLLLTELMRGMWVVLENFFRPPYTIYYP  95


>ref|WP_040254381.1| hypothetical protein [Psychroserpens mesophilus]
Length=206

 Score = 39.3 bits (90),  Expect = 1.2, Method: Compositional matrix adjust.
 Identities = 27/83 (33%), Positives = 40/83 (48%), Gaps = 14/83 (17%)

Query  94   SVMDKSMHLFFFGEEIWRMEDAIWGNHLIFCHSD--------WVVEPGMEVPPQKFPTHS  145
            +VMD+S+HL F    + R E+ I   HL+ CH D        W+VE      P KF T  
Sbjct  40   AVMDRSLHLIFGFTSLLRNENYIGACHLVRCHLDNILRFSGAWLVE-----NPHKFATDI  94

Query  146  TSSIDYVHQILFQSDPNLSLLYV  168
             + I  + +I+ +   NL   Y+
Sbjct  95   MNGIQ-IDKIIDRDGENLKDWYL  116


>ref|XP_568110.1| hypothetical protein [Cryptococcus neoformans var. neoformans 
JEC21]
 ref|XP_773492.1| hypothetical protein CNBI1060 [Cryptococcus neoformans var. neoformans 
B-3501A]
 gb|EAL18845.1| hypothetical protein CNBI1060 [Cryptococcus neoformans var. neoformans 
B-3501A]
 gb|AAW46593.1| conserved hypothetical protein [Cryptococcus neoformans var. 
neoformans JEC21]
Length=273

 Score = 39.3 bits (90),  Expect = 1.5, Method: Compositional matrix adjust.
 Identities = 23/60 (38%), Positives = 31/60 (52%), Gaps = 16/60 (27%)

Query  139  QKFPTHST--SSIDYVHQILFQSDPNLSLLYVSLVVSRILRGAWVVFEQMFRTSFHMATP  196
            QK+P +ST  S+ID   QILF              ++ I+RG WVV EQ FR  + +  P
Sbjct  65   QKYPDYSTGPSAIDKASQILF--------------LTEIVRGMWVVLEQFFRPPYTIMYP  110


Lambda      K        H        a         alpha
   0.324    0.136    0.431    0.792     4.96 

Gapped
Lambda      K        H        a         alpha    sigma
   0.267   0.0410    0.140     1.90     42.6     43.6 

Effective search space used: 901845308298


  Database: nr
    Posted date:  Sep 23, 2015 12:05 AM
  Number of letters in database: 26,053,659,533
  Number of sequences in database:  71,551,133


Matrix: BLOSUM62
Gap Penalties: Existence: 11, Extension: 1
Neighboring words threshold: 11
Window for multiple hits: 40
```
