# Supplementary material for: Comparative genomics provides new insights into the diversity, physiology, and sexuality of the only industrially exploited tremellomycete: Phaffia rhodozyma
Source: BMC Genomics. 2016 Nov 9;17:901. doi: 10.1186/s12864-016-3244-7 (PMC5103461; doi:10.1186/s12864-016-3244-7)
Supplement: Additional file 6: — List of orphan genes with links to PFAM (related to Additional file 1: Table S1). (ZIP 1428 kb) [file 12864_2016_3244_MOESM6_ESM.zip › BLAST_HTML_FTR/G00632_P.html]

BLAST Search Results


```
BLASTP 2.2.27+


Reference:
Stephen F. Altschul, Thomas L. Madden, Alejandro A. Schäffer,
Jinghui Zhang, Zheng Zhang, Webb Miller, and David J. Lipman (1997),
"Gapped BLAST and PSI-BLAST: a new generation of protein database
search programs", Nucleic Acids Res. 25:3389-3402.


Reference for
composition-based statistics:
Alejandro A. Schäffer, L. Aravind, Thomas L. Madden, Sergei
Shavirin, John L. Spouge, Yuri I. Wolf, Eugene V. Koonin, and
Stephen F. Altschul (2001), "Improving the accuracy of PSI-BLAST
protein database searches with composition-based statistics and
other refinements", Nucleic Acids Res. 29:2994-3005.


Database: nr
           71,551,133 sequences; 26,053,659,533 total letters


Query= G00632_P

Length=420
                                                                      Score     E
Sequences producing significant alignments:                          (Bits)  Value

emb|CED82543.1|  hypothetical protein [Xanthophyllomyces dendrorh...   828    0.0  
emb|CED82526.1|  hypothetical protein [Xanthophyllomyces dendrorh...  98.6    1e-18
emb|CED82534.1|  hypothetical protein [Xanthophyllomyces dendrorh...  74.7    7e-11


 >emb|CED82543.1| hypothetical protein [Xanthophyllomyces dendrorhous]
Length=419

 Score =  828 bits (2138),  Expect = 0.0, Method: Compositional matrix adjust.
 Identities = 419/419 (100%), Positives = 419/419 (100%), Gaps = 0/419 (0%)

Query  1    MLPGSESNTLAAGPRFLLPKSEAAASIRIVPRTPADFGMNRSGFFTKKTTRPAEKEPKSQ  60
            MLPGSESNTLAAGPRFLLPKSEAAASIRIVPRTPADFGMNRSGFFTKKTTRPAEKEPKSQ
Sbjct  1    MLPGSESNTLAAGPRFLLPKSEAAASIRIVPRTPADFGMNRSGFFTKKTTRPAEKEPKSQ  60

Query  61   PVQVVTCIHLLPPELDSNTLRKFFWTSGIRVLEAWKPIGKRSGMIQTSFEDQFKALRRIN  120
            PVQVVTCIHLLPPELDSNTLRKFFWTSGIRVLEAWKPIGKRSGMIQTSFEDQFKALRRIN
Sbjct  61   PVQVVTCIHLLPPELDSNTLRKFFWTSGIRVLEAWKPIGKRSGMIQTSFEDQFKALRRIN  120

Query  121  REPRPWGILQTQSAGLATVAIVLSPLEESSFRLASGKFSIDPNRKDNNASKISNPSTSSS  180
            REPRPWGILQTQSAGLATVAIVLSPLEESSFRLASGKFSIDPNRKDNNASKISNPSTSSS
Sbjct  121  REPRPWGILQTQSAGLATVAIVLSPLEESSFRLASGKFSIDPNRKDNNASKISNPSTSSS  180

Query  181  SSSLSSSSSSSSPLLASTLATAKTKQSTQPIGSPLPVSVRSNAPEPKQKGSVKAMAKLEK  240
            SSSLSSSSSSSSPLLASTLATAKTKQSTQPIGSPLPVSVRSNAPEPKQKGSVKAMAKLEK
Sbjct  181  SSSLSSSSSSSSPLLASTLATAKTKQSTQPIGSPLPVSVRSNAPEPKQKGSVKAMAKLEK  240

Query  241  AQKSPQTKNVSTQNVSSKGDSSTSADVVFKQNTSNVPPPPKTQKRPSETNIAGTPSRPKP  300
            AQKSPQTKNVSTQNVSSKGDSSTSADVVFKQNTSNVPPPPKTQKRPSETNIAGTPSRPKP
Sbjct  241  AQKSPQTKNVSTQNVSSKGDSSTSADVVFKQNTSNVPPPPKTQKRPSETNIAGTPSRPKP  300

Query  301  ITRQSDKKQREMSPPKINKQVNVGSVVKQTKSPITEAKSRVKTPTEKRTDTNDKIERVKT  360
            ITRQSDKKQREMSPPKINKQVNVGSVVKQTKSPITEAKSRVKTPTEKRTDTNDKIERVKT
Sbjct  301  ITRQSDKKQREMSPPKINKQVNVGSVVKQTKSPITEAKSRVKTPTEKRTDTNDKIERVKT  360

Query  361  TGEQKTQPSASSPSLSAKEKKKKKDLSMERSKAIQKVLTSVYKADPKPEEGDRTSSLAL  419
            TGEQKTQPSASSPSLSAKEKKKKKDLSMERSKAIQKVLTSVYKADPKPEEGDRTSSLAL
Sbjct  361  TGEQKTQPSASSPSLSAKEKKKKKDLSMERSKAIQKVLTSVYKADPKPEEGDRTSSLAL  419


>emb|CED82526.1| hypothetical protein [Xanthophyllomyces dendrorhous]
Length=980

 Score = 98.6 bits (244),  Expect = 1e-18, Method: Compositional matrix adjust.
 Identities = 57/143 (40%), Positives = 80/143 (56%), Gaps = 6/143 (4%)

Query  63   QVVTCIHLLPPELDSNTLRKFFWTSGIRVLEAWKPIGKRSGMIQTSFEDQFKALRRINRE  122
            QV+T IH LP ELDS T R FFW+ G+ V+EAWK  GKRSG+IQTS  DQ      ++  
Sbjct  135  QVITYIHRLPLELDSITFRDFFWSRGVHVVEAWKATGKRSGIIQTSISDQDLVCDTMDGT  194

Query  123  PRPWGILQTQSAGLATVAIVLSPLEESSFRLASGK-----FSIDPNRKDN-NASKISNPS  176
              PWG+L  +    A+  I LS  +E +F+ AS +     ++ID N   N  A  + + +
Sbjct  195  QAPWGLLIAKPGDTASTMIKLSEADEHAFQKASSQNPDSSYTIDKNVTSNPKAESLDDMT  254

Query  177  TSSSSSSLSSSSSSSSPLLASTL  199
              +S +S+  SS + S  L   L
Sbjct  255  DVASEASVKISSDNQSDELRQEL  277


>emb|CED82534.1| hypothetical protein [Xanthophyllomyces dendrorhous]
Length=916

 Score = 74.7 bits (182),  Expect = 7e-11, Method: Compositional matrix adjust.
 Identities = 46/103 (45%), Positives = 59/103 (57%), Gaps = 0/103 (0%)

Query  49   TTRPAEKEPKSQPVQVVTCIHLLPPELDSNTLRKFFWTSGIRVLEAWKPIGKRSGMIQTS  108
            + R   KE      QV+T I  LPP+++S +LR FFW++ I VL+A K  G+ SG+IQTS
Sbjct  63   SVRTETKEIPRDGHQVITSIFPLPPDMNSYSLRNFFWSNSINVLDARKSTGEVSGLIQTS  122

Query  109  FEDQFKALRRINREPRPWGILQTQSAGLATVAIVLSPLEESSF  151
             EDQ K L  I+     WG L    A  A  AIV SP +E  F
Sbjct  123  QEDQQKLLSSIHDIQAHWGNLTAVPASPAMTAIVPSPKQERKF  165


Lambda      K        H        a         alpha
   0.307    0.121    0.330    0.792     4.96 

Gapped
Lambda      K        H        a         alpha    sigma
   0.267   0.0410    0.140     1.90     42.6     43.6 

Effective search space used: 4033391761128


  Database: nr
    Posted date:  Sep 23, 2015 12:05 AM
  Number of letters in database: 26,053,659,533
  Number of sequences in database:  71,551,133


Matrix: BLOSUM62
Gap Penalties: Existence: 11, Extension: 1
Neighboring words threshold: 11
Window for multiple hits: 40
```
