# Supplementary material for: Comparative genomics provides new insights into the diversity, physiology, and sexuality of the only industrially exploited tremellomycete: Phaffia rhodozyma
Source: BMC Genomics. 2016 Nov 9;17:901. doi: 10.1186/s12864-016-3244-7 (PMC5103461; doi:10.1186/s12864-016-3244-7)
Supplement: Additional file 6: — List of orphan genes with links to PFAM (related to Additional file 1: Table S1). (ZIP 1428 kb) [file 12864_2016_3244_MOESM6_ESM.zip › BLAST_HTML_FTR/G05523_P.html]

BLAST Search Results


```
BLASTP 2.2.27+


Reference:
Stephen F. Altschul, Thomas L. Madden, Alejandro A. Schäffer,
Jinghui Zhang, Zheng Zhang, Webb Miller, and David J. Lipman (1997),
"Gapped BLAST and PSI-BLAST: a new generation of protein database
search programs", Nucleic Acids Res. 25:3389-3402.


Reference for
composition-based statistics:
Alejandro A. Schäffer, L. Aravind, Thomas L. Madden, Sergei
Shavirin, John L. Spouge, Yuri I. Wolf, Eugene V. Koonin, and
Stephen F. Altschul (2001), "Improving the accuracy of PSI-BLAST
protein database searches with composition-based statistics and
other refinements", Nucleic Acids Res. 29:2994-3005.


Database: nr
           71,551,133 sequences; 26,053,659,533 total letters


Query= G05523_P

Length=243
                                                                      Score     E
Sequences producing significant alignments:                          (Bits)  Value

emb|CDZ97509.1|  hypothetical protein [Xanthophyllomyces dendrorh...   453    2e-159
ref|XP_008071145.1|  PREDICTED: thyroid receptor-interacting prot...  41.6    0.75  
ref|XP_008071146.1|  PREDICTED: thyroid receptor-interacting prot...  41.2    0.83  
ref|WP_015611529.1|  hypothetical protein [Streptomyces fulvissim...  38.5    5.0   
ref|WP_019885226.1|  peptidase S8 [Streptomyces purpureus]            37.7    9.7   


 >emb|CDZ97509.1| hypothetical protein [Xanthophyllomyces dendrorhous]
Length=221

 Score =  453 bits (1166),  Expect = 2e-159, Method: Compositional matrix adjust.
 Identities = 221/221 (100%), Positives = 221/221 (100%), Gaps = 0/221 (0%)

Query  22   MKENLAAHIHIHLLFSPGQESTWTLLERKFPKASEKDFNAAISLLEMDDVISVDPHPEFA  81
            MKENLAAHIHIHLLFSPGQESTWTLLERKFPKASEKDFNAAISLLEMDDVISVDPHPEFA
Sbjct  1    MKENLAAHIHIHLLFSPGQESTWTLLERKFPKASEKDFNAAISLLEMDDVISVDPHPEFA  60

Query  82   QEMLVKIDYNHSIPPPPLPVPGFRFPGEHDCPHGTKNASTVAGSRASPADPFVLPTKKSV  141
            QEMLVKIDYNHSIPPPPLPVPGFRFPGEHDCPHGTKNASTVAGSRASPADPFVLPTKKSV
Sbjct  61   QEMLVKIDYNHSIPPPPLPVPGFRFPGEHDCPHGTKNASTVAGSRASPADPFVLPTKKSV  120

Query  142  GLKPAAFNLDVSSQRRPLMISPDPSPPILGELISRTNSGIFAEGAWSHTPPRMRDALGEE  201
            GLKPAAFNLDVSSQRRPLMISPDPSPPILGELISRTNSGIFAEGAWSHTPPRMRDALGEE
Sbjct  121  GLKPAAFNLDVSSQRRPLMISPDPSPPILGELISRTNSGIFAEGAWSHTPPRMRDALGEE  180

Query  202  EILIEGQVWRPENEETKGNETKRTFRGVGDGAAGWQAMLVA  242
            EILIEGQVWRPENEETKGNETKRTFRGVGDGAAGWQAMLVA
Sbjct  181  EILIEGQVWRPENEETKGNETKRTFRGVGDGAAGWQAMLVA  221


>ref|XP_008071145.1| PREDICTED: thyroid receptor-interacting protein 11 isoform X1 
[Tarsius syrichta]
Length=1828

 Score = 41.6 bits (96),  Expect = 0.75, Method: Composition-based stats.
 Identities = 34/111 (31%), Positives = 48/111 (43%), Gaps = 23/111 (21%)

Query  83    EMLVKI---DYNHSIPPPPLPVPGFRF---PG----EHDCPHGTKNASTVAGSRASPADP  132
             E+ VK    + + SIPPP L VPG +    PG    + + P   K+A+     R +   P
Sbjct  1711  ELFVKFLETESHPSIPPPKLSVPGMKLLDSPGRRKQDTNVPESYKDATDSRTGRRTDVSP  1770

Query  133   FVLPTKKSVGL-KPAAFN------------LDVSSQRRPLMISPDPSPPIL  170
             F+ P   +V L  PA               LDV     PL +SPD S  ++
Sbjct  1771  FLAPRSAAVPLVNPAGLGPGGSGHLLLKPILDVLPTFTPLPVSPDSSAGVV  1821


>ref|XP_008071146.1| PREDICTED: thyroid receptor-interacting protein 11 isoform X2 
[Tarsius syrichta]
Length=1805

 Score = 41.2 bits (95),  Expect = 0.83, Method: Composition-based stats.
 Identities = 34/111 (31%), Positives = 48/111 (43%), Gaps = 23/111 (21%)

Query  83    EMLVKI---DYNHSIPPPPLPVPGFRF---PG----EHDCPHGTKNASTVAGSRASPADP  132
             E+ VK    + + SIPPP L VPG +    PG    + + P   K+A+     R +   P
Sbjct  1688  ELFVKFLETESHPSIPPPKLSVPGMKLLDSPGRRKQDTNVPESYKDATDSRTGRRTDVSP  1747

Query  133   FVLPTKKSVGL-KPAAFN------------LDVSSQRRPLMISPDPSPPIL  170
             F+ P   +V L  PA               LDV     PL +SPD S  ++
Sbjct  1748  FLAPRSAAVPLVNPAGLGPGGSGHLLLKPILDVLPTFTPLPVSPDSSAGVV  1798


>ref|WP_015611529.1| hypothetical protein [Streptomyces fulvissimus]
 gb|AGK80216.1| Tetratricopeptide TPR_1 repeat-containing protein [Streptomyces 
fulvissimus DSM 40593]
Length=451

 Score = 38.5 bits (88),  Expect = 5.0, Method: Compositional matrix adjust.
 Identities = 39/125 (31%), Positives = 55/125 (44%), Gaps = 9/125 (7%)

Query  119  ASTVAGSRASPADPFVLPTKKSVGLKPAAFNLDVSSQRRPLMISPDPSPPI-LGELISRT  177
            A T  G RA+P DP +L T+    L        V+  R  + I+P P   + LGEL    
Sbjct  249  AETRTGLRAAPDDPALLETRARAHLARGDTTRAVADYRAAVAIAPLPHHLLGLGELEQSL  308

Query  178  NSGIFAEGAWS--HTPPRMRDALGE----EEILIEGQVWRPENEETKGNETKRT--FRGV  229
             +G  AE +++      R+R+A G+    + IL E     P    T   +T RT  F  V
Sbjct  309  GNGKQAEESYALLRAQDRIREAAGDPADTDAILFEADHGNPRRAVTLAEQTLRTRPFVAV  368

Query  230  GDGAA  234
             D  A
Sbjct  369  HDAYA  373


>ref|WP_019885226.1| peptidase S8 [Streptomyces purpureus]
Length=511

 Score = 37.7 bits (86),  Expect = 9.7, Method: Compositional matrix adjust.
 Identities = 30/101 (30%), Positives = 40/101 (40%), Gaps = 1/101 (1%)

Query  44   WTLLERKFPKASEKDFNAAISLLEMDDVISVDPHPEFAQEMLVKIDYNHSIPPPPLPVPG  103
            W L   K  KA +K   +    + + D    D HP+ A         N  +   P   PG
Sbjct  157  WDLPAIKADKAHQKSLGSGKVTVAVIDTGVDDTHPDLAPNFDRAASAN-CVTGAPDTTPG  215

Query  104  FRFPGEHDCPHGTKNASTVAGSRASPADPFVLPTKKSVGLK  144
               PG H+  HGT  A TVA ++       V P  K  G+K
Sbjct  216  SWRPGPHESDHGTHVAGTVAAAKNGVGITGVAPGVKVSGIK  256


Lambda      K        H        a         alpha
   0.317    0.135    0.413    0.792     4.96 

Gapped
Lambda      K        H        a         alpha    sigma
   0.267   0.0410    0.140     1.90     42.6     43.6 

Effective search space used: 1513897829155


  Database: nr
    Posted date:  Sep 23, 2015 12:05 AM
  Number of letters in database: 26,053,659,533
  Number of sequences in database:  71,551,133


Matrix: BLOSUM62
Gap Penalties: Existence: 11, Extension: 1
Neighboring words threshold: 11
Window for multiple hits: 40
```
