# Supplementary material for: Comparative genomics provides new insights into the diversity, physiology, and sexuality of the only industrially exploited tremellomycete: Phaffia rhodozyma
Source: BMC Genomics. 2016 Nov 9;17:901. doi: 10.1186/s12864-016-3244-7 (PMC5103461; doi:10.1186/s12864-016-3244-7)
Supplement: Additional file 6: — List of orphan genes with links to PFAM (related to Additional file 1: Table S1). (ZIP 1428 kb) [file 12864_2016_3244_MOESM6_ESM.zip › BLAST_HTML_FTR/G02779_P.html]

BLAST Search Results


```
BLASTP 2.2.27+


Reference:
Stephen F. Altschul, Thomas L. Madden, Alejandro A. Schäffer,
Jinghui Zhang, Zheng Zhang, Webb Miller, and David J. Lipman (1997),
"Gapped BLAST and PSI-BLAST: a new generation of protein database
search programs", Nucleic Acids Res. 25:3389-3402.


Reference for
composition-based statistics:
Alejandro A. Schäffer, L. Aravind, Thomas L. Madden, Sergei
Shavirin, John L. Spouge, Yuri I. Wolf, Eugene V. Koonin, and
Stephen F. Altschul (2001), "Improving the accuracy of PSI-BLAST
protein database searches with composition-based statistics and
other refinements", Nucleic Acids Res. 29:2994-3005.


Database: nr
           71,551,133 sequences; 26,053,659,533 total letters


Query= G02779_P

Length=741
                                                                      Score     E
Sequences producing significant alignments:                          (Bits)  Value

emb|CDZ97125.1|  Basic-leucine zipper domain [Xanthophyllomyces d...  1275    0.0  


 >emb|CDZ97125.1| Basic-leucine zipper domain [Xanthophyllomyces dendrorhous]
Length=634

 Score = 1275 bits (3299),  Expect = 0.0, Method: Compositional matrix adjust.
 Identities = 633/634 (99%), Positives = 634/634 (100%), Gaps = 0/634 (0%)

Query  107  MLNSGSAPSPNQPPQIPQAILQQLLQEPSILSLLSRLEQQQPNTSGSPSGSLSHNSFPSN  166
            MLNSGSAPSPNQPPQIPQAILQQLLQEPSILSLLSRLEQQQPNTSGSPSGSLSHNSFPSN
Sbjct  1    MLNSGSAPSPNQPPQIPQAILQQLLQEPSILSLLSRLEQQQPNTSGSPSGSLSHNSFPSN  60

Query  167  SSFTAVPGIPTPLSNGFTPEPVTTRSGRPSRPPQPSLQLSHNLSHADDQLAALHQAWAGL  226
            SSFTAVPGIPTPLSNGFTPEPVTTRSGRPSRPPQPSLQLSHNLSHADDQLAALHQAWAGL
Sbjct  61   SSFTAVPGIPTPLSNGFTPEPVTTRSGRPSRPPQPSLQLSHNLSHADDQLAALHQAWAGL  120

Query  227  DTSFLDGIAGSLNISNSLPSSGPISAHLPTDPFTSSASLLEPSPSASNVTGTVTASSTNL  286
            DTSFLDGIAGSLNISNSLPSSGPISAHLPTDPFTSSASLLEPSPSASNVTGTVTASSTNL
Sbjct  121  DTSFLDGIAGSLNISNSLPSSGPISAHLPTDPFTSSASLLEPSPSASNVTGTVTASSTNL  180

Query  287  DLGWWWPFQDEENEDEDPSYDPSMAYPNLTPPTFNLITPGPSTTNSLAEESSQPAASTSG  346
            DLGWWWPFQDEENEDEDPSYDPSMAYPNLTPPTFNLITPGPSTTNSLAEESSQPAASTSG
Sbjct  181  DLGWWWPFQDEENEDEDPSYDPSMAYPNLTPPTFNLITPGPSTTNSLAEESSQPAASTSG  240

Query  347  SMERLLNNASKTSSKRKGKGKAILEEALEASITNTSVNIDTTKFNTTESAPANLFFNNDD  406
            SMERLLNNASKTSSKRKGKGKAILEEALEASITNTSVNIDTTKFNTTESAPANLFFNNDD
Sbjct  241  SMERLLNNASKTSSKRKGKGKAILEEALEASITNTSVNIDTTKFNTTESAPANLFFNNDD  300

Query  407  VAAGSSIQDEEDDDEGEPGRLPTPGGRLLRKRKVKERSPPPPSKKVKLTAEESAARRKAR  466
            VAAGSSIQDEEDDDEGEPGRLPTPGGRLLRKRKVKERSPPPPSKKVKLTAEESAARRKAR
Sbjct  301  VAAGSSIQDEEDDDEGEPGRLPTPGGRLLRKRKVKERSPPPPSKKVKLTAEESAARRKAR  360

Query  467  NKELASNSRKRQRDYVVGLEARIIELEAEVKKYKLVFQQAARSIKPGPGKNIYDVVSGGI  526
            NKELASNSRKRQRDYVVGLEARIIELEAEVKKYKLVFQQAARSIKPGPGKNIYDVVSGGI
Sbjct  361  NKELASNSRKRQRDYVVGLEARIIELEAEVKKYKLVFQQAARSIKPGPGKNIYDVVSGGI  420

Query  527  KNPHPFANLPSIPASVPYKASPLKKNSSKKKHTEGEEEEQELPAEVAWPEDEEEDDEWQS  586
            KNPHPFANLPSIPASVPYKASPLKKNSSKKKHTEGEEEEQELPAEVAWPEDEEEDDEWQS
Sbjct  421  KNPHPFANLPSIPASVPYKASPLKKNSSKKKHTEGEEEEQELPAEVAWPEDEEEDDEWQS  480

Query  587  QGEDKEVRIHHDSDERQGEGHNDNHLEDIEGRPAKVLETSIGPSEEGVKQTRAEEVRGNE  646
            QGEDKEVRIHHDSDERQGEGH+DNHLEDIEGRPAKVLETSIGPSEEGVKQTRAEEVRGNE
Sbjct  481  QGEDKEVRIHHDSDERQGEGHDDNHLEDIEGRPAKVLETSIGPSEEGVKQTRAEEVRGNE  540

Query  647  EEEEDEDDDEDDEDSDSEDSALEDEDDEDLYQPIIDVPIPIELAPPPPTTQTETALGGSD  706
            EEEEDEDDDEDDEDSDSEDSALEDEDDEDLYQPIIDVPIPIELAPPPPTTQTETALGGSD
Sbjct  541  EEEEDEDDDEDDEDSDSEDSALEDEDDEDLYQPIIDVPIPIELAPPPPTTQTETALGGSD  600

Query  707  GPMDAPGSIPAGEKVGNQEIMDLLKKLLAKMGGA  740
            GPMDAPGSIPAGEKVGNQEIMDLLKKLLAKMGGA
Sbjct  601  GPMDAPGSIPAGEKVGNQEIMDLLKKLLAKMGGA  634


Lambda      K        H        a         alpha
   0.304    0.124    0.347    0.792     4.96 

Gapped
Lambda      K        H        a         alpha    sigma
   0.267   0.0410    0.140     1.90     42.6     43.6 

Effective search space used: 8542031102652


  Database: nr
    Posted date:  Sep 23, 2015 12:05 AM
  Number of letters in database: 26,053,659,533
  Number of sequences in database:  71,551,133


Matrix: BLOSUM62
Gap Penalties: Existence: 11, Extension: 1
Neighboring words threshold: 11
Window for multiple hits: 40
```
