# Supplementary material for: Comparative genomics provides new insights into the diversity, physiology, and sexuality of the only industrially exploited tremellomycete: Phaffia rhodozyma
Source: BMC Genomics. 2016 Nov 9;17:901. doi: 10.1186/s12864-016-3244-7 (PMC5103461; doi:10.1186/s12864-016-3244-7)
Supplement: Additional file 6: — List of orphan genes with links to PFAM (related to Additional file 1: Table S1). (ZIP 1428 kb) [file 12864_2016_3244_MOESM6_ESM.zip › BLAST_HTML_FTR/G05949_P.html]

BLAST Search Results


```
BLASTP 2.2.27+


Reference:
Stephen F. Altschul, Thomas L. Madden, Alejandro A. Schäffer,
Jinghui Zhang, Zheng Zhang, Webb Miller, and David J. Lipman (1997),
"Gapped BLAST and PSI-BLAST: a new generation of protein database
search programs", Nucleic Acids Res. 25:3389-3402.


Reference for
composition-based statistics:
Alejandro A. Schäffer, L. Aravind, Thomas L. Madden, Sergei
Shavirin, John L. Spouge, Yuri I. Wolf, Eugene V. Koonin, and
Stephen F. Altschul (2001), "Improving the accuracy of PSI-BLAST
protein database searches with composition-based statistics and
other refinements", Nucleic Acids Res. 29:2994-3005.


Database: nr
           71,551,133 sequences; 26,053,659,533 total letters


Query= G05949_P

Length=473
                                                                      Score     E
Sequences producing significant alignments:                          (Bits)  Value

emb|CED84879.1|  hypothetical protein [Xanthophyllomyces dendrorh...   739    0.0  
ref|WP_053309378.1|  succinylglutamate desuccinylase [Vibrio algi...  40.0    4.1  
ref|XP_005797699.1|  PREDICTED: protein FAM13A-like [Xiphophorus ...  40.0    5.4  


 >emb|CED84879.1| hypothetical protein [Xanthophyllomyces dendrorhous]
Length=443

 Score =  739 bits (1907),  Expect = 0.0, Method: Compositional matrix adjust.
 Identities = 425/463 (92%), Positives = 427/463 (92%), Gaps = 29/463 (6%)

Query  10   VERLPSNILSSIQLAIGTTHPTALRDHLAWSRSHPILRAFYQPWWERQINNSGWGLPNPA  69
            VERLPSNILSSIQLAIGTTHPTALRDHLAWSRSHPILRAFYQPWWERQINNSGWGLPNPA
Sbjct  10   VERLPSNILSSIQLAIGTTHPTALRDHLAWSRSHPILRAFYQPWWERQINNSGWGLPNPA  69

Query  70   PPSPVHALCYYLHHQTTCAVQVLRPWAYEDDAASPGSSRKKIPDDRGEWIALCWSTHSPE  129
            PPSPVHALCYYLHHQTTCAVQVLRPWAYEDDAASPGSSRKKIPDDRGEWIALCWSTHSPE
Sbjct  70   PPSPVHALCYYLHHQTTCAVQVLRPWAYEDDAASPGSSRKKIPDDRGEWIALCWSTHSPE  129

Query  130  KYGIWPPLSKSGQTITPNPLLSDTFLPLTPPPPAWTHPALSLPTHQAHTTSPLSRHPTYA  189
            KYGIWPPLSKSGQTITPNPLLSDTFLPLTPPPPAWTHPALSLPTHQAHTTSPLSRHPTYA
Sbjct  130  KYGIWPPLSKSGQTITPNPLLSDTFLPLTPPPPAWTHPALSLPTHQAHTTSPLSRHPTYA  189

Query  190  HQLASFPARAELEVLFLWGTPSDHSLDHHKTLPAGQAEWEKGRALVRVNRQDGRPITVRD  249
            HQLASFPARAELEVLFLWGTPSDHSLDHHKTLPAGQAEWEKGRALVRVNRQDGRPITVRD
Sbjct  190  HQLASFPARAELEVLFLWGTPSDHSLDHHKTLPAGQAEWEKGRALVRVNRQDGRPITVRD  249

Query  250  LWQAILDFSKASLSVPVLPLPPPNLPCSSHSLSPSPNGNSLTPLNVPNQVHDRFPHLDTY  309
            LWQAILDF                          SP G   T      ++HDRFPHLDTY
Sbjct  250  LWQAILDFWT------------------------SPLGWQET-----KRLHDRFPHLDTY  280

Query  310  LSSDRPTKGEMNGRLTKIQRDNQRWAWLGNYNVFTGLLSAIPSPASSSTPRDRFVAWLIT  369
            LSSDRPTKGEMNGRLTKIQRDNQRWAWLGNYNVFTGLLSAIPSPASSSTPRDRFVAWLIT
Sbjct  281  LSSDRPTKGEMNGRLTKIQRDNQRWAWLGNYNVFTGLLSAIPSPASSSTPRDRFVAWLIT  340

Query  370  PSELARFHDQGEAEQLAVGSKRKAQDDAEADLDLDLDLDLDNSKDKGKGRKENSGPTRSR  429
            PSELARFHDQGEAEQLAVGSKRKAQDDAEADLDLDLDLDLDNSKDKGKGRKENSGPTRSR
Sbjct  341  PSELARFHDQGEAEQLAVGSKRKAQDDAEADLDLDLDLDLDNSKDKGKGRKENSGPTRSR  400

Query  430  PRTQMQPQAQPQAHSKPKAQVHVGDQIWRRIDSTTDEAVSGLY  472
            PRTQMQPQAQPQAHSKPKAQVHVGDQIWRRIDSTTDEAVSGLY
Sbjct  401  PRTQMQPQAQPQAHSKPKAQVHVGDQIWRRIDSTTDEAVSGLY  443


>ref|WP_053309378.1| succinylglutamate desuccinylase [Vibrio alginolyticus]
 gb|KOE87381.1| succinylglutamate desuccinylase [Vibrio alginolyticus]
Length=342

 Score = 40.0 bits (92),  Expect = 4.1, Method: Compositional matrix adjust.
 Identities = 32/96 (33%), Positives = 43/96 (45%), Gaps = 11/96 (11%)

Query  166  HPALSLPTHQAHTTSPLSRHPTYAHQLASFPARAELEVLFLWGTPSD----HSLDHHK--  219
            H A+ L  H +   SP SRHP  +H L SF   A +E + L  +PS     +S +H    
Sbjct  155  HCAIRLSKHYSFAVSPKSRHPVRSHALMSFIDSAHVEAVLLSNSPSSTFSWYSAEHFAAQ  214

Query  220  --TLPAGQAEWEKGRALVRVNRQDGRPITVRDLWQA  253
              TL  GQ        L R+   D   + +RDL  A
Sbjct  215  ALTLELGQVARIGENNLKRLVAFD---LAMRDLVSA  247


>ref|XP_005797699.1| PREDICTED: protein FAM13A-like [Xiphophorus maculatus]
Length=728

 Score = 40.0 bits (92),  Expect = 5.4, Method: Compositional matrix adjust.
 Identities = 29/80 (36%), Positives = 37/80 (46%), Gaps = 6/80 (8%)

Query  233  ALVRVNRQDGRPITVRDLWQAILDFSKASLSVPVLPLPPPNLPCSSHSLSPSPNGNSLTP  292
            +LVR  R DG P   R   Q     S  S S+P++      LP +S   SPSP     +P
Sbjct  19   SLVRKERNDGMPQVARQQRQPQGSPSTRSFSIPIV------LPLTSELTSPSPEVTDTSP  72

Query  293  LNVPNQVHDRFPHLDTYLSS  312
            L  P+      PH+D  LSS
Sbjct  73   LPEPDSSRTASPHVDFSLSS  92


Lambda      K        H        a         alpha
   0.317    0.132    0.429    0.792     4.96 

Gapped
Lambda      K        H        a         alpha    sigma
   0.267   0.0410    0.140     1.90     42.6     43.6 

Effective search space used: 4796096431269


  Database: nr
    Posted date:  Sep 23, 2015 12:05 AM
  Number of letters in database: 26,053,659,533
  Number of sequences in database:  71,551,133


Matrix: BLOSUM62
Gap Penalties: Existence: 11, Extension: 1
Neighboring words threshold: 11
Window for multiple hits: 40
```
