# Supplementary material for: Comparative genomics provides new insights into the diversity, physiology, and sexuality of the only industrially exploited tremellomycete: Phaffia rhodozyma
Source: BMC Genomics. 2016 Nov 9;17:901. doi: 10.1186/s12864-016-3244-7 (PMC5103461; doi:10.1186/s12864-016-3244-7)
Supplement: Additional file 6: — List of orphan genes with links to PFAM (related to Additional file 1: Table S1). (ZIP 1428 kb) [file 12864_2016_3244_MOESM6_ESM.zip › BLAST_HTML_FTR/G04555_P.html]

BLAST Search Results


```
BLASTP 2.2.27+


Reference:
Stephen F. Altschul, Thomas L. Madden, Alejandro A. Schäffer,
Jinghui Zhang, Zheng Zhang, Webb Miller, and David J. Lipman (1997),
"Gapped BLAST and PSI-BLAST: a new generation of protein database
search programs", Nucleic Acids Res. 25:3389-3402.


Reference for
composition-based statistics:
Alejandro A. Schäffer, L. Aravind, Thomas L. Madden, Sergei
Shavirin, John L. Spouge, Yuri I. Wolf, Eugene V. Koonin, and
Stephen F. Altschul (2001), "Improving the accuracy of PSI-BLAST
protein database searches with composition-based statistics and
other refinements", Nucleic Acids Res. 29:2994-3005.


Database: nr
           71,551,133 sequences; 26,053,659,533 total letters


Query= G04555_P

Length=398
                                                                      Score     E
Sequences producing significant alignments:                          (Bits)  Value

emb|CED83318.1|  hypothetical protein [Xanthophyllomyces dendrorh...   792    0.0  
gb|KIM94758.1|  hypothetical protein OIDMADRAFT_82992, partial [O...  41.2    0.26 
ref|WP_028582389.1|  hypothetical protein [Desulfobulbus japonicus]   42.7    0.50 
gb|KIO08221.1|  hypothetical protein M404DRAFT_997147 [Pisolithus...  41.6    1.5  


 >emb|CED83318.1| hypothetical protein [Xanthophyllomyces dendrorhous]
Length=397

 Score =  792 bits (2045),  Expect = 0.0, Method: Compositional matrix adjust.
 Identities = 397/397 (100%), Positives = 397/397 (100%), Gaps = 0/397 (0%)

Query  1    MPASKRGIDKANLSSDEILANQRERNRKKQAALRARRAQKLLDLEQEIARLGGNHHPSAT  60
            MPASKRGIDKANLSSDEILANQRERNRKKQAALRARRAQKLLDLEQEIARLGGNHHPSAT
Sbjct  1    MPASKRGIDKANLSSDEILANQRERNRKKQAALRARRAQKLLDLEQEIARLGGNHHPSAT  60

Query  61   NGTGAHFGSSLVGSQLMDGSSQGSVHLDGQREIRKLSVVIGRLVGKLKEFGVDDEEIRSM  120
            NGTGAHFGSSLVGSQLMDGSSQGSVHLDGQREIRKLSVVIGRLVGKLKEFGVDDEEIRSM
Sbjct  61   NGTGAHFGSSLVGSQLMDGSSQGSVHLDGQREIRKLSVVIGRLVGKLKEFGVDDEEIRSM  120

Query  121  TEDGLEEELGEQGIVENLMQAEDIERDGTEKSEREAQEAQYADYIRLSGGDKTSSLSSGQ  180
            TEDGLEEELGEQGIVENLMQAEDIERDGTEKSEREAQEAQYADYIRLSGGDKTSSLSSGQ
Sbjct  121  TEDGLEEELGEQGIVENLMQAEDIERDGTEKSEREAQEAQYADYIRLSGGDKTSSLSSGQ  180

Query  181  TVPYPTNWPSPPRVDPNGHSDPPASTTSSGDPLGPYPVSQPSSGTSVAGGTNMIGIDGLL  240
            TVPYPTNWPSPPRVDPNGHSDPPASTTSSGDPLGPYPVSQPSSGTSVAGGTNMIGIDGLL
Sbjct  181  TVPYPTNWPSPPRVDPNGHSDPPASTTSSGDPLGPYPVSQPSSGTSVAGGTNMIGIDGLL  240

Query  241  TDSSSNGIQARKPSSDNLIGNPSPNTPPGSAFPFAFLFSGPGPAGSILPQHELQQQQSFI  300
            TDSSSNGIQARKPSSDNLIGNPSPNTPPGSAFPFAFLFSGPGPAGSILPQHELQQQQSFI
Sbjct  241  TDSSSNGIQARKPSSDNLIGNPSPNTPPGSAFPFAFLFSGPGPAGSILPQHELQQQQSFI  300

Query  301  RSLSTDGGLSSEMISREPGQDMPVEGEGESTGEGIGAGVEQAEGEEVRDETQGAGGEADG  360
            RSLSTDGGLSSEMISREPGQDMPVEGEGESTGEGIGAGVEQAEGEEVRDETQGAGGEADG
Sbjct  301  RSLSTDGGLSSEMISREPGQDMPVEGEGESTGEGIGAGVEQAEGEEVRDETQGAGGEADG  360

Query  361  VSTNEGFLVEGQMPSPESGHMPPPESVDVEPTYDHPE  397
            VSTNEGFLVEGQMPSPESGHMPPPESVDVEPTYDHPE
Sbjct  361  VSTNEGFLVEGQMPSPESGHMPPPESVDVEPTYDHPE  397


>gb|KIM94758.1| hypothetical protein OIDMADRAFT_82992, partial [Oidiodendron 
maius Zn]
Length=96

 Score = 41.2 bits (95),  Expect = 0.26, Method: Compositional matrix adjust.
 Identities = 22/73 (30%), Positives = 41/73 (56%), Gaps = 1/73 (1%)

Query  5   KRGID-KANLSSDEILANQRERNRKKQAALRARRAQKLLDLEQEIARLGGNHHPSATNGT  63
           KR  D + N++S  +L+ +R +NR  Q A R+R+ +++ ++E+E+  L   H+  A +  
Sbjct  20  KRKTDGRDNVTSSHVLSRRRAQNRVSQRAFRSRKQKRMKEMEEELTTLQERHNELAKSYE  79

Query  64  GAHFGSSLVGSQL  76
                 SL+  QL
Sbjct  80  ALQMEYSLMKQQL  92


>ref|WP_028582389.1| hypothetical protein [Desulfobulbus japonicus]
Length=343

 Score = 42.7 bits (99),  Expect = 0.50, Method: Compositional matrix adjust.
 Identities = 31/91 (34%), Positives = 50/91 (55%), Gaps = 9/91 (10%)

Query  81   SQGSVHLDGQ-----REIRKLSVVIGRLVGKLKEFGVDDEEIRSMTEDGLEEELGEQGIV  135
            SQG+ HL GQ     R++   + ++  + GK+   G D  +I+ M +DGLE ++G  G  
Sbjct  194  SQGT-HLSGQIGDLIRKVIDGNQLLYDIDGKMSGIGDDIGDIKDMMKDGLEADIGNPG--  250

Query  136  ENLMQAEDIERDGTEKSEREAQEAQYADYIR  166
             +L +AE +  D  E  E +    Q+ADYI+
Sbjct  251  -DLPEAEYVGDDLEEVEEIDPLAEQFADYIQ  280


>gb|KIO08221.1| hypothetical protein M404DRAFT_997147 [Pisolithus tinctorius 
Marx 270]
Length=498

 Score = 41.6 bits (96),  Expect = 1.5, Method: Compositional matrix adjust.
 Identities = 33/91 (36%), Positives = 45/91 (49%), Gaps = 18/91 (20%)

Query  189  PSPPRVDPNGHSDPPASTTSSGDPLGPYPVSQPSSGTSVAGGTNMIGIDGL---------  239
            PSP  +DPN  +  PAS  +   P GP+PV + S G+S+ GG   +   G+         
Sbjct  332  PSPAPIDPNALNRSPASIPAHPSP-GPHPVGEGSPGSSIPGGVPTVAETGVPLSAGPSGP  390

Query  240  ------LTD--SSSNGIQARKPSSDNLIGNP  262
                  L D  S S+G Q RK S D+L G+P
Sbjct  391  GPASGSLKDVRSGSHGDQVRKDSVDDLYGSP  421


Lambda      K        H        a         alpha
   0.308    0.130    0.368    0.792     4.96 

Gapped
Lambda      K        H        a         alpha    sigma
   0.267   0.0410    0.140     1.90     42.6     43.6 

Effective search space used: 3701052365080


  Database: nr
    Posted date:  Sep 23, 2015 12:05 AM
  Number of letters in database: 26,053,659,533
  Number of sequences in database:  71,551,133


Matrix: BLOSUM62
Gap Penalties: Existence: 11, Extension: 1
Neighboring words threshold: 11
Window for multiple hits: 40
```
