# Supplementary material for: Comparative genomics provides new insights into the diversity, physiology, and sexuality of the only industrially exploited tremellomycete: Phaffia rhodozyma
Source: BMC Genomics. 2016 Nov 9;17:901. doi: 10.1186/s12864-016-3244-7 (PMC5103461; doi:10.1186/s12864-016-3244-7)
Supplement: Additional file 6: — List of orphan genes with links to PFAM (related to Additional file 1: Table S1). (ZIP 1428 kb) [file 12864_2016_3244_MOESM6_ESM.zip › BLAST_HTML_FTR/G05542_P.html]

BLAST Search Results


```
BLASTP 2.2.27+


Reference:
Stephen F. Altschul, Thomas L. Madden, Alejandro A. Schäffer,
Jinghui Zhang, Zheng Zhang, Webb Miller, and David J. Lipman (1997),
"Gapped BLAST and PSI-BLAST: a new generation of protein database
search programs", Nucleic Acids Res. 25:3389-3402.


Reference for
composition-based statistics:
Alejandro A. Schäffer, L. Aravind, Thomas L. Madden, Sergei
Shavirin, John L. Spouge, Yuri I. Wolf, Eugene V. Koonin, and
Stephen F. Altschul (2001), "Improving the accuracy of PSI-BLAST
protein database searches with composition-based statistics and
other refinements", Nucleic Acids Res. 29:2994-3005.


Database: nr
           71,551,133 sequences; 26,053,659,533 total letters


Query= G05542_P

Length=348
                                                                      Score     E
Sequences producing significant alignments:                          (Bits)  Value

emb|CED83732.1|  hypothetical protein [Xanthophyllomyces dendrorh...   490    3e-170
emb|CED84825.1|  hypothetical protein [Xanthophyllomyces dendrorh...  92.8    2e-18 
gb|ETO01732.1|  hypothetical protein RFI_35707, partial [Reticulo...  41.2    1.1   


 >emb|CED83732.1| hypothetical protein [Xanthophyllomyces dendrorhous]
Length=335

 Score =  490 bits (1261),  Expect = 3e-170, Method: Compositional matrix adjust.
 Identities = 334/347 (96%), Positives = 334/347 (96%), Gaps = 12/347 (3%)

Query  1    MISTPVIVLGLAALVSASQASAAVIGLKAGAAVGGAGLGAKVGAGVHVRAPSSYSGAAGG  60
            MISTPVIVLGLAALVSASQASAAVIGLKAGAAVGGAGLGAKVGAGVHVRAPSSYSGAAGG
Sbjct  1    MISTPVIVLGLAALVSASQASAAVIGLKAGAAVGGAGLGAKVGAGVHVRAPSSYSGAAGG  60

Query  61   GSAYGGGSVNVDDDHRVHVSGGGYGGSHGGGMTYVRRSRASARGLDGLDGLEDVLKNVLD  120
            GSAYGGGSVNVDDDHRVHVSGGGYGGSHGGGMTYVRRSRASARGLDGLDGLEDVLKNVLD
Sbjct  61   GSAYGGGSVNVDDDHRVHVSGGGYGGSHGGGMTYVRRSRASARGLDGLDGLEDVLKNVLD  120

Query  121  SDDISALADVSGSISISANIKAQLSVDGLFKTPDNTLAYQCPDKQWAPPAQYNFGYFDVD  180
            SDDISA            NIKAQLSVDGLFKTPDNTLAYQCPDKQWAPPAQYNFGYFDVD
Sbjct  121  SDDISA------------NIKAQLSVDGLFKTPDNTLAYQCPDKQWAPPAQYNFGYFDVD  168

Query  181  TGAWVDDQAQVQTYLQGLGYAHLDISGSLDLFAHANVNPAILPTGDNTQNKGKCGYLVPA  240
            TGAWVDDQAQVQTYLQGLGYAHLDISGSLDLFAHANVN AILPTGDNTQNKGKCGYLVPA
Sbjct  169  TGAWVDDQAQVQTYLQGLGYAHLDISGSLDLFAHANVNAAILPTGDNTQNKGKCGYLVPA  228

Query  241  APAAPATKAAVATPSSACDVASTAVPTGTTAAGVLDVSATIFVALQANADVNLGLSADVD  300
            APAAPATKAAVATPSSACDVASTAVPTGTTAAGVLDVSATIFVALQANADVNLGLSADVD
Sbjct  229  APAAPATKAAVATPSSACDVASTAVPTGTTAAGVLDVSATIFVALQANADVNLGLSADVD  288

Query  301  ADVDADINANVDANLNAQADVSVDVKTLLNSSGFFQLAEPKSKDCGC  347
            ADVDADINANVDANLNAQADVSVDVKTLLNSSGFFQLAEPKSKDCGC
Sbjct  289  ADVDADINANVDANLNAQADVSVDVKTLLNSSGFFQLAEPKSKDCGC  335


>emb|CED84825.1| hypothetical protein [Xanthophyllomyces dendrorhous]
Length=257

 Score = 92.8 bits (229),  Expect = 2e-18, Method: Compositional matrix adjust.
 Identities = 45/124 (36%), Positives = 65/124 (52%), Gaps = 6/124 (5%)

Query  127  LADVSGSISISANIKAQLSVDGLFKTPDNTLAYQCPDKQWAPPAQYNFGYFDVDTGAWVD  186
            L     ++ +SA + A+L+V GL +  D++L Y CP K W  P  Y FGY+   TG W++
Sbjct  90   LLSAKANVDVSATVAAELAVSGLSRKKDHSLHYTCPTKGWKAPPAYEFGYWHPTTGVWIN  149

Query  187  DQAQVQTYLQGLGYAHLDISGSLDLFAHANVNPAILPTG------DNTQNKGKCGYLVPA  240
            D+  V  YL   GY HL +   +DLF   +   A + +G       +   KGKCGY VP 
Sbjct  150  DKTAVDAYLSVQGYVHLGLDVVVDLFVDIDATVAAVASGVIASRPADLTKKGKCGYWVPK  209

Query  241  APAA  244
            +  A
Sbjct  210  STTA  213


>gb|ETO01732.1| hypothetical protein RFI_35707, partial [Reticulomyxa filosa]
Length=303

 Score = 41.2 bits (95),  Expect = 1.1, Method: Compositional matrix adjust.
 Identities = 17/43 (40%), Positives = 34/43 (79%), Gaps = 2/43 (5%)

Query  285  LQANADVNLGLSADVDADVDADINANVDANLNAQADVSVDVKT  327
            + ANAD+N+ ++ D++ D++ DINA+++A++N  AD++ D+ T
Sbjct  58   IDANADINVDINVDINVDINTDINADINADIN--ADINTDINT  98


 Score = 40.0 bits (92),  Expect = 2.4, Method: Compositional matrix adjust.
 Identities = 16/50 (32%), Positives = 34/50 (68%), Gaps = 0/50 (0%)

Query  271  AAGVLDVSATIFVALQANADVNLGLSADVDADVDADINANVDANLNAQAD  320
            A  + ++ A   + +  N D+N+ ++ D++AD++ADINA+++ ++N  AD
Sbjct  52   AIAITNIDANADINVDINVDINVDINTDINADINADINADINTDINTDAD  101


 Score = 38.5 bits (88),  Expect = 8.1, Method: Compositional matrix adjust.
 Identities = 15/40 (38%), Positives = 29/40 (73%), Gaps = 0/40 (0%)

Query  288  NADVNLGLSADVDADVDADINANVDANLNAQADVSVDVKT  327
            N D+N+ ++ D++ D++ADINA+++A++N   +   D KT
Sbjct  65   NVDINVDINVDINTDINADINADINADINTDINTDADAKT  104


Lambda      K        H        a         alpha
   0.315    0.132    0.383    0.792     4.96 

Gapped
Lambda      K        H        a         alpha    sigma
   0.267   0.0410    0.140     1.90     42.6     43.6 

Effective search space used: 3004139374650


  Database: nr
    Posted date:  Sep 23, 2015 12:05 AM
  Number of letters in database: 26,053,659,533
  Number of sequences in database:  71,551,133


Matrix: BLOSUM62
Gap Penalties: Existence: 11, Extension: 1
Neighboring words threshold: 11
Window for multiple hits: 40
```
