# Supplementary material for: Comparative genomics provides new insights into the diversity, physiology, and sexuality of the only industrially exploited tremellomycete: Phaffia rhodozyma
Source: BMC Genomics. 2016 Nov 9;17:901. doi: 10.1186/s12864-016-3244-7 (PMC5103461; doi:10.1186/s12864-016-3244-7)
Supplement: Additional file 6: — List of orphan genes with links to PFAM (related to Additional file 1: Table S1). (ZIP 1428 kb) [file 12864_2016_3244_MOESM6_ESM.zip › BLAST_HTML_FTR/G03963_P.html]

BLAST Search Results


```
BLASTP 2.2.27+


Reference:
Stephen F. Altschul, Thomas L. Madden, Alejandro A. Schäffer,
Jinghui Zhang, Zheng Zhang, Webb Miller, and David J. Lipman (1997),
"Gapped BLAST and PSI-BLAST: a new generation of protein database
search programs", Nucleic Acids Res. 25:3389-3402.


Reference for
composition-based statistics:
Alejandro A. Schäffer, L. Aravind, Thomas L. Madden, Sergei
Shavirin, John L. Spouge, Yuri I. Wolf, Eugene V. Koonin, and
Stephen F. Altschul (2001), "Improving the accuracy of PSI-BLAST
protein database searches with composition-based statistics and
other refinements", Nucleic Acids Res. 29:2994-3005.


Database: nr
           71,551,133 sequences; 26,053,659,533 total letters


Query= G03963_P

Length=977
                                                                      Score     E
Sequences producing significant alignments:                          (Bits)  Value

emb|CDZ98660.1|  Zinc finger, C2H2-like [Xanthophyllomyces dendro...  1997    0.0  
ref|XP_727008.1|  hypothetical protein [Plasmodium yoelii yoelii ...  44.7    0.36 
gb|EEQ45895.1|  conserved hypothetical protein [Candida albicans ...  43.5    1.3  


 >emb|CDZ98660.1| Zinc finger, C2H2-like [Xanthophyllomyces dendrorhous]
Length=976

 Score = 1997 bits (5173),  Expect = 0.0, Method: Compositional matrix adjust.
 Identities = 974/976 (99%), Positives = 975/976 (99%), Gaps = 0/976 (0%)

Query  1    MEDSPQPSPAAVPTGKIFTCHYCEPPHVFSKEGERKVHHRITHQASSMINHPVTGESYEC  60
            MEDSPQPSPAAVPTGKIFTCHYCEPPHVFSKEGERKVHHRITHQASSMINHPVTGESYEC
Sbjct  1    MEDSPQPSPAAVPTGKIFTCHYCEPPHVFSKEGERKVHHRITHQASSMINHPVTGESYEC  60

Query  61   FRDPTSGTFSCPRCISHADRDPVRLKRHVLSCLSKWPEKSYTCEDCDDSIEYPTIIHLKD  120
            FRDPTSGTFSCPRCISHADRDPVRLKRHVLSCLSKWPEKSYTCEDCDDSIEYPTIIHLKD
Sbjct  61   FRDPTSGTFSCPRCISHADRDPVRLKRHVLSCLSKWPEKSYTCEDCDDSIEYPTIIHLKD  120

Query  121  HHRVVHQETTPINHPSGQVLDVPRSPGSHTFTCPRCVGFSHKDPDRLTWHCTTSCTSVFP  180
            HHRVVHQETTPINHPSGQVLDVPRSPGSHTFTCPRCVGFSHKDPDRLTWHCTTSCTSVFP
Sbjct  121  HHRVVHQETTPINHPSGQVLDVPRSPGSHTFTCPRCVGFSHKDPDRLTWHCTTSCTSVFP  180

Query  181  PLSAYPPTFFKGYASHAPTSPGAQSDPEPTFVAATLPVSASPLPRIGSFSADGSPVASAN  240
            PLSAYPPTFFKGYASHAPTSPGAQSDPEPTFVAATLPVSASPLPRIGSFSADGSPVASAN
Sbjct  181  PLSAYPPTFFKGYASHAPTSPGAQSDPEPTFVAATLPVSASPLPRIGSFSADGSPVASAN  240

Query  241  GGSVLIKYRQKRFYCRLCTPQVSFDNADTKEAHVQALHPDIKKKKRRPTETWSVAGGGSG  300
            GGSVLIKYRQKRFYCRLCTPQVSFDNADTKEAHVQALHPD+KKKKRRPTETWSVAGGGSG
Sbjct  241  GGSVLIKYRQKRFYCRLCTPQVSFDNADTKEAHVQALHPDVKKKKRRPTETWSVAGGGSG  300

Query  301  LRNEIGEDEPEGEPDGQEEETQEPAVKRRIKPRKSRAKPKNLDLEGAHPTGLSFDSPGGP  360
            LRNEIGEDEPEGEPDGQEEETQEPAVKRRIKPRKSRAKPKNLDLEGAHPTGLSFDSPGGP
Sbjct  301  LRNEIGEDEPEGEPDGQEEETQEPAVKRRIKPRKSRAKPKNLDLEGAHPTGLSFDSPGGP  360

Query  361  DQGLLSPGGIMFHQSPAPGSNQANQFYPHAGWPSAYLPPPPRPGAGPHSTSTSPSLSATS  420
            DQGLLSPGGIMFHQSPAPGSNQANQFYPHAGWPSAYLPPPPRPGAGPHSTSTSPSLSATS
Sbjct  361  DQGLLSPGGIMFHQSPAPGSNQANQFYPHAGWPSAYLPPPPRPGAGPHSTSTSPSLSATS  420

Query  421  TSYPGQSIIPSQPNRLFGQIPSFDAFRAQYPAQQQQQYYQQHQEQQLYQQMLQQQQHQQQ  480
            TSYPGQSII SQPNRLFGQIPSFDAFRAQYPAQQQQQYYQQHQEQQLYQQMLQQQQHQQQ
Sbjct  421  TSYPGQSIIQSQPNRLFGQIPSFDAFRAQYPAQQQQQYYQQHQEQQLYQQMLQQQQHQQQ  480

Query  481  QQQQPPSRSTSTPGPTSQPKIPPRQPSPKLPTPPPDDRPSCPFCIVYPSVRFDSPDEQAE  540
            QQQQPPSRSTSTPGPTSQPKIPPRQPSPKLPTPPPDDRPSCPFCIVYPSVRFDSPDEQAE
Sbjct  481  QQQQPPSRSTSTPGPTSQPKIPPRQPSPKLPTPPPDDRPSCPFCIVYPSVRFDSPDEQAE  540

Query  541  HEKRLHRWPLKRKRAILDLVHVRITRPWAASQLEKNHQESNEATGEDKTKTDGKNDERDE  600
            HEKRLHRWPLKRKRAILDLVHVRITRPWAASQLEKNHQESNEATGEDKTKTDGKNDERDE
Sbjct  541  HEKRLHRWPLKRKRAILDLVHVRITRPWAASQLEKNHQESNEATGEDKTKTDGKNDERDE  600

Query  601  KEREQEQLHKDKQTDQDEDGEKEEKGRWECTECIPHINFQTESTKEFHRRVSHLTTVDVI  660
            KEREQEQLHKDKQTDQDEDGEKEEKGRWECTECIPHINFQTESTKEFHRRVSHLTTVDVI
Sbjct  601  KEREQEQLHKDKQTDQDEDGEKEEKGRWECTECIPHINFQTESTKEFHRRVSHLTTVDVI  660

Query  661  LPTGESVQVVRSAVRGGFPCPVCNPEEEPTEEPSIEEIQHTPETDGNVDKDVSDDVGKDT  720
            LPTGESVQVVRSAVRGGFPCPVCNPEEEPTEEPSIEEIQHTPETDGNVDKDVSDDVGKDT
Sbjct  661  LPTGESVQVVRSAVRGGFPCPVCNPEEEPTEEPSIEEIQHTPETDGNVDKDVSDDVGKDT  720

Query  721  NEDKMDVEVDTVTTEKEEKDEKDENDEKDKEGKEDKESKEDEESKEDREDKEDKEDKKEK  780
            NEDKMDVEVDTVTTEKEEKDEKDENDEKDKEGKEDKESKEDEESKEDREDKEDKEDKKEK
Sbjct  721  NEDKMDVEVDTVTTEKEEKDEKDENDEKDKEGKEDKESKEDEESKEDREDKEDKEDKKEK  780

Query  781  EEGKEEEGEEKKKTGYVSWDPERLKAHLKECTLVREPSPAGSPLPNQSILTSLPSPKNND  840
            EEGKEEEGEEKKKTGYVSWDPERLKAHLKECTLVREPSPAGSPLPNQSILTSLPSPKNND
Sbjct  781  EEGKEEEGEEKKKTGYVSWDPERLKAHLKECTLVREPSPAGSPLPNQSILTSLPSPKNND  840

Query  841  DAAAAAPVVENSITKPPSDPKEPAEHIPVPTSLETTAETGFVRSVTQDLPSHSSPLKLIE  900
            DAAAAAPVVENSITKPPSDPKEPAEHIPVPTSLETTAETGFVRSVTQDLPSHSSPLKLIE
Sbjct  841  DAAAAAPVVENSITKPPSDPKEPAEHIPVPTSLETTAETGFVRSVTQDLPSHSSPLKLIE  900

Query  901  KNLMSTESRLSMSPPLTSAVPASASTSVSTEHQPVQANGQLESSPDGQKAIGGTEPSRRP  960
            KNLMSTESRLSMSPPLTSAVPASASTSVSTEHQPVQANGQLESSPDGQKAIGGTEPSRRP
Sbjct  901  KNLMSTESRLSMSPPLTSAVPASASTSVSTEHQPVQANGQLESSPDGQKAIGGTEPSRRP  960

Query  961  LSTSTSKYSLSSLLNN  976
            LSTSTSKYSLSSLLNN
Sbjct  961  LSTSTSKYSLSSLLNN  976


>ref|XP_727008.1| hypothetical protein [Plasmodium yoelii yoelii 17XNL]
 gb|EAA18573.1| hypothetical protein [Plasmodium yoelii yoelii]
 emb|CDU16359.1| erythrocyte membrane associated protein 2, putative [Plasmodium 
yoelii]
 emb|CDZ10886.1| erythrocyte membrane associated protein 2, putative [Plasmodium 
yoelii]
Length=334

 Score = 44.7 bits (104),  Expect = 0.36, Method: Compositional matrix adjust.
 Identities = 33/83 (40%), Positives = 51/83 (61%), Gaps = 2/83 (2%)

Query  720  TNEDKMDVEVDTVTTEKEEKDEKDENDEKDKEGKEDKESKEDEESKEDREDKEDKEDKKE  779
             NEDK D E D    E +E D +DE D +DK+  EDK+  ED++  ED+ED ED++D ++
Sbjct  73   NNEDKQDNE-DKQDNEDQE-DNEDEQDNEDKQDNEDKQDNEDKQDNEDQEDNEDEQDNED  130

Query  780  KEEGKEEEGEEKKKTGYVSWDPE  802
            K++ +++E  E K+      D E
Sbjct  131  KQDNEDQEDNEDKQDNEDKQDNE  153


 Score = 42.7 bits (99),  Expect = 1.5, Method: Compositional matrix adjust.
 Identities = 32/97 (33%), Positives = 59/97 (61%), Gaps = 4/97 (4%)

Query  710  KDVSDDVGKDTNEDKMDVEVDTVTTEKEEKDEKDENDEK----DKEGKEDKESKEDEESK  765
            +D  D+  K  NED+ D E +    +K++ ++K +N++K    D+E  ED++  ED++  
Sbjct  75   EDKQDNEDKQDNEDQEDNEDEQDNEDKQDNEDKQDNEDKQDNEDQEDNEDEQDNEDKQDN  134

Query  766  EDREDKEDKEDKKEKEEGKEEEGEEKKKTGYVSWDPE  802
            ED+ED EDK+D ++K++ +++E  E K+      D E
Sbjct  135  EDQEDNEDKQDNEDKQDNEDQEDNEDKQDNEDKQDNE  171


>gb|EEQ45895.1| conserved hypothetical protein [Candida albicans WO-1]
Length=1189

 Score = 43.5 bits (101),  Expect = 1.3, Method: Compositional matrix adjust.
 Identities = 31/75 (41%), Positives = 48/75 (64%), Gaps = 0/75 (0%)

Query  737  EEKDEKDENDEKDKEGKEDKESKEDEESKEDREDKEDKEDKKEKEEGKEEEGEEKKKTGY  796
            EE  + +E  +KD+E K+D+ESK+DEESK+D E K+D+E  K+K+E K +  E+  K   
Sbjct  633  EESTKSNEESKKDEESKKDEESKKDEESKKDEESKKDEETSKKKDEDKTQANEDSTKGKK  692

Query  797  VSWDPERLKAHLKEC  811
            V  D ++   + KE 
Sbjct  693  VEIDVKKEDTNEKEI  707


Lambda      K        H        a         alpha
   0.309    0.128    0.385    0.792     4.96 

Gapped
Lambda      K        H        a         alpha    sigma
   0.267   0.0410    0.140     1.90     42.6     43.6 

Effective search space used: 11859684529920


  Database: nr
    Posted date:  Sep 23, 2015 12:05 AM
  Number of letters in database: 26,053,659,533
  Number of sequences in database:  71,551,133


Matrix: BLOSUM62
Gap Penalties: Existence: 11, Extension: 1
Neighboring words threshold: 11
Window for multiple hits: 40
```
