# Supplementary material for: Comparative genomics provides new insights into the diversity, physiology, and sexuality of the only industrially exploited tremellomycete: Phaffia rhodozyma
Source: BMC Genomics. 2016 Nov 9;17:901. doi: 10.1186/s12864-016-3244-7 (PMC5103461; doi:10.1186/s12864-016-3244-7)
Supplement: Additional file 6: — List of orphan genes with links to PFAM (related to Additional file 1: Table S1). (ZIP 1428 kb) [file 12864_2016_3244_MOESM6_ESM.zip › BLAST_HTML_FTR/G02866_P.html]

BLAST Search Results


```
BLASTP 2.2.27+


Reference:
Stephen F. Altschul, Thomas L. Madden, Alejandro A. Schäffer,
Jinghui Zhang, Zheng Zhang, Webb Miller, and David J. Lipman (1997),
"Gapped BLAST and PSI-BLAST: a new generation of protein database
search programs", Nucleic Acids Res. 25:3389-3402.


Reference for
composition-based statistics:
Alejandro A. Schäffer, L. Aravind, Thomas L. Madden, Sergei
Shavirin, John L. Spouge, Yuri I. Wolf, Eugene V. Koonin, and
Stephen F. Altschul (2001), "Improving the accuracy of PSI-BLAST
protein database searches with composition-based statistics and
other refinements", Nucleic Acids Res. 29:2994-3005.


Database: nr
           71,551,133 sequences; 26,053,659,533 total letters


Query= G02866_P

Length=438
                                                                      Score     E
Sequences producing significant alignments:                          (Bits)  Value

emb|CDZ98050.1|  hypothetical protein [Xanthophyllomyces dendrorh...   844    0.0  
gb|ETO82680.1|  TKL protein kinase [Phytophthora parasitica P1976]    41.2    2.0  
gb|ETP51781.1|  TKL protein kinase [Phytophthora parasitica P10297]   41.2    2.1  
gb|ETI53997.1|  TKL protein kinase [Phytophthora parasitica P1569]    41.2    2.2  
gb|ETP23800.1|  TKL protein kinase [Phytophthora parasitica CJ01A1]   41.2    2.6  
gb|ETI53994.1|  TKL protein kinase [Phytophthora parasitica P1569]    40.8    2.8  
ref|XP_008900341.1|  TKL protein kinase [Phytophthora parasitica ...  40.8    2.9  
gb|ETP23797.1|  TKL protein kinase [Phytophthora parasitica CJ01A1]   40.8    2.9  
emb|CCA70101.1|  hypothetical protein PIIN_04041 [Piriformospora ...  39.3    7.6  


 >emb|CDZ98050.1| hypothetical protein [Xanthophyllomyces dendrorhous]
Length=454

 Score =  844 bits (2181),  Expect = 0.0, Method: Compositional matrix adjust.
 Identities = 425/427 (99%), Positives = 426/427 (99%), Gaps = 0/427 (0%)

Query  11   SSTPSAVITTASVTLPSTGSTTPTALALTTTTAAAITTSGAPPSAALLTSTALTSAQSAI  70
            SSTPSAVITTASVTLPSTGSTTPTALALTTTTAAAITTSGAPPSAALLTSTALTSAQSAI
Sbjct  28   SSTPSAVITTASVTLPSTGSTTPTALALTTTTAAAITTSGAPPSAALLTSTALTSAQSAI  87

Query  71   VSSSISTSYANIPPASNDTMTKARITAIETGMSTALLATQEADTSSTTAVIMTQSSSKVK  130
            VSSSISTSYANIPPASNDTMTKARITAIETGMSTALLATQEADTSSTT VIMTQSSSKVK
Sbjct  88   VSSSISTSYANIPPASNDTMTKARITAIETGMSTALLATQEADTSSTTTVIMTQSSSKVK  147

Query  131  KSSTSSRSSSATTRLSSASSSASTAIANADSSSSTSLGVGSIAGIVAALCVFLLVIGCFF  190
            KSSTSSRSSSATTRLSSASSSASTAIANADSSSSTSLGVGSIAGIVAALCVFLLVIGCFF
Sbjct  148  KSSTSSRSSSATTRLSSASSSASTAIANADSSSSTSLGVGSIAGIVAALCVFLLVIGCFF  207

Query  191  VIRSNARQKRKRAVEAYAPYSDWNNGRGSTFELQDEDSGSTGGMIERKPSWRDQGRPRDM  250
            VIRSNARQKRKRAVEAYAPYSDWNNGRGSTFELQDEDSGSTGGMIERKPSWRDQGRPRDM
Sbjct  208  VIRSNARQKRKRAVEAYAPYSDWNNGRGSTFELQDEDSGSTGGMIERKPSWRDQGRPRDM  267

Query  251  GGPRPPTMIESGRAGMGAIGSLGRVPSTSTPVPQLSKSAQPQTSDTTVPSFSPGQIIQPL  310
            GGPRPPTMIESGRAGMGAIGSLGRVPSTSTPVPQLSKSAQPQTSDTTVPSFSPGQIIQPL
Sbjct  268  GGPRPPTMIESGRAGMGAIGSLGRVPSTSTPVPQLSKSAQPQTSDTTVPSFSPGQIIQPL  327

Query  311  FTAGLAPLPPVAISSTYGEPGPNDGTRWEAQGGLGTGMGYPVGAAGYVQQRQQQPSPQHQ  370
            FTAGLAPLPPVAISSTYGEPGPNDGTRWEAQGG+GTGMGYPVGAAGYVQQRQQQPSPQHQ
Sbjct  328  FTAGLAPLPPVAISSTYGEPGPNDGTRWEAQGGVGTGMGYPVGAAGYVQQRQQQPSPQHQ  387

Query  371  AYTNGPMYPHSHPLSHSLQPTLSSQQPYPYQNQQPFTVQSTVGQVQMQQPSTNRQTMFNP  430
            AYTNGPMYPHSHPLSHSLQPTLSSQQPYPYQNQQPFTVQSTVGQVQMQQPSTNRQTMFNP
Sbjct  388  AYTNGPMYPHSHPLSHSLQPTLSSQQPYPYQNQQPFTVQSTVGQVQMQQPSTNRQTMFNP  447

Query  431  DDAYGGM  437
            DDAYGGM
Sbjct  448  DDAYGGM  454


>gb|ETO82680.1| TKL protein kinase [Phytophthora parasitica P1976]
Length=675

 Score = 41.2 bits (95),  Expect = 2.0, Method: Compositional matrix adjust.
 Identities = 24/57 (42%), Positives = 37/57 (65%), Gaps = 2/57 (4%)

Query  147  SASSSASTAIANADSSSSTSLGVGSIAGIVAALCVFLLVIGCFFVIRSNARQKRKRA  203
            S SS A +  +NA + SS+S+G G+I GI+ A  VF+LV+  FF+ +   R K +R+
Sbjct  270  SGSSLADSNNSNA-TKSSSSIGPGAIVGIIVAAVVFMLVLFAFFIAQRR-RNKHRRS  324


>gb|ETP51781.1| TKL protein kinase [Phytophthora parasitica P10297]
Length=675

 Score = 41.2 bits (95),  Expect = 2.1, Method: Compositional matrix adjust.
 Identities = 24/57 (42%), Positives = 37/57 (65%), Gaps = 2/57 (4%)

Query  147  SASSSASTAIANADSSSSTSLGVGSIAGIVAALCVFLLVIGCFFVIRSNARQKRKRA  203
            S SS A +  +NA + SS+S+G G+I GI+ A  VF+LV+  FF+ +   R K +R+
Sbjct  270  SGSSLADSNNSNA-TKSSSSIGPGAIVGIIVAAVVFMLVLFAFFIAQRR-RNKHRRS  324


>gb|ETI53997.1| TKL protein kinase [Phytophthora parasitica P1569]
Length=675

 Score = 41.2 bits (95),  Expect = 2.2, Method: Compositional matrix adjust.
 Identities = 24/57 (42%), Positives = 37/57 (65%), Gaps = 2/57 (4%)

Query  147  SASSSASTAIANADSSSSTSLGVGSIAGIVAALCVFLLVIGCFFVIRSNARQKRKRA  203
            S SS A +  +NA + SS+S+G G+I GI+ A  VF+LV+  FF+ +   R K +R+
Sbjct  270  SGSSLADSNNSNA-TKSSSSIGPGAIVGIIVAAVVFMLVLFAFFIAQRR-RNKHRRS  324


>gb|ETP23800.1| TKL protein kinase [Phytophthora parasitica CJ01A1]
Length=675

 Score = 41.2 bits (95),  Expect = 2.6, Method: Compositional matrix adjust.
 Identities = 25/57 (44%), Positives = 36/57 (63%), Gaps = 2/57 (4%)

Query  147  SASSSASTAIANADSSSSTSLGVGSIAGIVAALCVFLLVIGCFFVIRSNARQKRKRA  203
            S SS A +  +NA  SSS S+G G+I GI+ A  VF+LV+  FF+ +   R K +R+
Sbjct  270  SGSSLADSNNSNATKSSS-SIGPGAIVGIIVAAVVFMLVLFAFFIAQRR-RNKHRRS  324


>gb|ETI53994.1| TKL protein kinase [Phytophthora parasitica P1569]
Length=625

 Score = 40.8 bits (94),  Expect = 2.8, Method: Compositional matrix adjust.
 Identities = 24/57 (42%), Positives = 37/57 (65%), Gaps = 2/57 (4%)

Query  147  SASSSASTAIANADSSSSTSLGVGSIAGIVAALCVFLLVIGCFFVIRSNARQKRKRA  203
            S SS A +  +NA + SS+S+G G+I GI+ A  VF+LV+  FF+ +   R K +R+
Sbjct  222  SGSSLADSNNSNA-TKSSSSIGPGAIVGIIVAAVVFMLVLFAFFIAQRR-RNKHRRS  276


>ref|XP_008900341.1| TKL protein kinase [Phytophthora parasitica INRA-310]
 gb|ETN14058.1| TKL protein kinase [Phytophthora parasitica INRA-310]
 gb|ETO82677.1| TKL protein kinase [Phytophthora parasitica P1976]
 gb|ETP51793.1| TKL protein kinase [Phytophthora parasitica P10297]
Length=625

 Score = 40.8 bits (94),  Expect = 2.9, Method: Compositional matrix adjust.
 Identities = 24/57 (42%), Positives = 37/57 (65%), Gaps = 2/57 (4%)

Query  147  SASSSASTAIANADSSSSTSLGVGSIAGIVAALCVFLLVIGCFFVIRSNARQKRKRA  203
            S SS A +  +NA + SS+S+G G+I GI+ A  VF+LV+  FF+ +   R K +R+
Sbjct  222  SGSSLADSNNSNA-TKSSSSIGPGAIVGIIVAAVVFMLVLFAFFIAQRR-RNKHRRS  276


>gb|ETP23797.1| TKL protein kinase [Phytophthora parasitica CJ01A1]
Length=625

 Score = 40.8 bits (94),  Expect = 2.9, Method: Compositional matrix adjust.
 Identities = 24/57 (42%), Positives = 37/57 (65%), Gaps = 2/57 (4%)

Query  147  SASSSASTAIANADSSSSTSLGVGSIAGIVAALCVFLLVIGCFFVIRSNARQKRKRA  203
            S SS A +  +NA + SS+S+G G+I GI+ A  VF+LV+  FF+ +   R K +R+
Sbjct  222  SGSSLADSNNSNA-TKSSSSIGPGAIVGIIVAAVVFMLVLFAFFIAQRR-RNKHRRS  276


>emb|CCA70101.1| hypothetical protein PIIN_04041 [Piriformospora indica DSM 11827]
Length=399

 Score = 39.3 bits (90),  Expect = 7.6, Method: Compositional matrix adjust.
 Identities = 29/86 (34%), Positives = 45/86 (52%), Gaps = 13/86 (15%)

Query  164  STSLGVGSIAGIVAALCVFLLVIGCFFV--IRSNARQKRKRAVEAYAPYSDWNNGRGSTF  221
            S  +G+GSIAGI  A  V + VI  F V  IR   R+K+ + ++   P++  +  R ST 
Sbjct  134  SDGMGIGSIAGIAVAAIVGIAVISAFVVWLIR---RKKKNQDIDE-EPFNRNSFMRNSTV  189

Query  222  ELQDEDSG------STGGMIERKPSW  241
             + D+D G          M ER+P++
Sbjct  190  -IPDDDVGIPSRTRPQPNMAERQPTY  214


Lambda      K        H        a         alpha
   0.309    0.122    0.344    0.792     4.96 

Gapped
Lambda      K        H        a         alpha    sigma
   0.267   0.0410    0.140     1.90     42.6     43.6 

Effective search space used: 4269878954484


  Database: nr
    Posted date:  Sep 23, 2015 12:05 AM
  Number of letters in database: 26,053,659,533
  Number of sequences in database:  71,551,133


Matrix: BLOSUM62
Gap Penalties: Existence: 11, Extension: 1
Neighboring words threshold: 11
Window for multiple hits: 40
```
