# Supplementary material for: Comparative genomics provides new insights into the diversity, physiology, and sexuality of the only industrially exploited tremellomycete: Phaffia rhodozyma
Source: BMC Genomics. 2016 Nov 9;17:901. doi: 10.1186/s12864-016-3244-7 (PMC5103461; doi:10.1186/s12864-016-3244-7)
Supplement: Additional file 6: — List of orphan genes with links to PFAM (related to Additional file 1: Table S1). (ZIP 1428 kb) [file 12864_2016_3244_MOESM6_ESM.zip › BLAST_HTML_FTR/G03801_P.html]

BLAST Search Results


```
BLASTP 2.2.27+


Reference:
Stephen F. Altschul, Thomas L. Madden, Alejandro A. Schäffer,
Jinghui Zhang, Zheng Zhang, Webb Miller, and David J. Lipman (1997),
"Gapped BLAST and PSI-BLAST: a new generation of protein database
search programs", Nucleic Acids Res. 25:3389-3402.


Reference for
composition-based statistics:
Alejandro A. Schäffer, L. Aravind, Thomas L. Madden, Sergei
Shavirin, John L. Spouge, Yuri I. Wolf, Eugene V. Koonin, and
Stephen F. Altschul (2001), "Improving the accuracy of PSI-BLAST
protein database searches with composition-based statistics and
other refinements", Nucleic Acids Res. 29:2994-3005.


Database: nr
           71,551,133 sequences; 26,053,659,533 total letters


Query= G03801_P

Length=848
                                                                      Score     E
Sequences producing significant alignments:                          (Bits)  Value

emb|CDZ98077.1|  hypothetical protein [Xanthophyllomyces dendrorh...  1687    0.0  


 >emb|CDZ98077.1| hypothetical protein [Xanthophyllomyces dendrorhous]
Length=847

 Score = 1687 bits (4369),  Expect = 0.0, Method: Compositional matrix adjust.
 Identities = 847/847 (100%), Positives = 847/847 (100%), Gaps = 0/847 (0%)

Query  1    MVQPSPTPVSYPIYSPPRQSLHFLRYLTKSSTSPLPSNLAVVALLTLLFATYHVTRFVKY  60
            MVQPSPTPVSYPIYSPPRQSLHFLRYLTKSSTSPLPSNLAVVALLTLLFATYHVTRFVKY
Sbjct  1    MVQPSPTPVSYPIYSPPRQSLHFLRYLTKSSTSPLPSNLAVVALLTLLFATYHVTRFVKY  60

Query  61   QQVKRRWRASERARRELIKKESERSSASGVASSSTLPNGGSSSSVGKSSVSPYIGERPSR  120
            QQVKRRWRASERARRELIKKESERSSASGVASSSTLPNGGSSSSVGKSSVSPYIGERPSR
Sbjct  61   QQVKRRWRASERARRELIKKESERSSASGVASSSTLPNGGSSSSVGKSSVSPYIGERPSR  120

Query  121  SELGAKKRKKSKASMTATTTTAAVVDTNRSSSVATTISPASSITPFPKEIDEPVIVTTKP  180
            SELGAKKRKKSKASMTATTTTAAVVDTNRSSSVATTISPASSITPFPKEIDEPVIVTTKP
Sbjct  121  SELGAKKRKKSKASMTATTTTAAVVDTNRSSSVATTISPASSITPFPKEIDEPVIVTTKP  180

Query  181  ASQSSGLSLRIDTKASEDTSRDSSDSKFQVVLPFTSKRRNKINRSTSSPNSSSTPTVGSN  240
            ASQSSGLSLRIDTKASEDTSRDSSDSKFQVVLPFTSKRRNKINRSTSSPNSSSTPTVGSN
Sbjct  181  ASQSSGLSLRIDTKASEDTSRDSSDSKFQVVLPFTSKRRNKINRSTSSPNSSSTPTVGSN  240

Query  241  SQSRDNSTDPSSPPASTTTVIDSSNNNDHDEIVEKFAADGQSTASSCSGLTGQDGDFSPA  300
            SQSRDNSTDPSSPPASTTTVIDSSNNNDHDEIVEKFAADGQSTASSCSGLTGQDGDFSPA
Sbjct  241  SQSRDNSTDPSSPPASTTTVIDSSNNNDHDEIVEKFAADGQSTASSCSGLTGQDGDFSPA  300

Query  301  SSIQTTPTTSINSSTQSLAISSAKTHPVWPSSPLDQAGPPKETSPSLEHEVESLRASLST  360
            SSIQTTPTTSINSSTQSLAISSAKTHPVWPSSPLDQAGPPKETSPSLEHEVESLRASLST
Sbjct  301  SSIQTTPTTSINSSTQSLAISSAKTHPVWPSSPLDQAGPPKETSPSLEHEVESLRASLST  360

Query  361  ALSTLSSMQSTLASSQSSLLGSHAQLSDVTAERDTLARSATTFKSELADLTTSIARSESR  420
            ALSTLSSMQSTLASSQSSLLGSHAQLSDVTAERDTLARSATTFKSELADLTTSIARSESR
Sbjct  361  ALSTLSSMQSTLASSQSSLLGSHAQLSDVTAERDTLARSATTFKSELADLTTSIARSESR  420

Query  421  AVSAEEKQTELSAEVEQLRKDLERARREVEDGRRWGEERRKESEDLRRKEKAGRREIEKF  480
            AVSAEEKQTELSAEVEQLRKDLERARREVEDGRRWGEERRKESEDLRRKEKAGRREIEKF
Sbjct  421  AVSAEEKQTELSAEVEQLRKDLERARREVEDGRRWGEERRKESEDLRRKEKAGRREIEKF  480

Query  481  KEEGRKWEVYEREFRRREYELRFQLHHLSTMHTTLLHHARQLEAHLRETNYAFAPMPVQT  540
            KEEGRKWEVYEREFRRREYELRFQLHHLSTMHTTLLHHARQLEAHLRETNYAFAPMPVQT
Sbjct  481  KEEGRKWEVYEREFRRREYELRFQLHHLSTMHTTLLHHARQLEAHLRETNYAFAPMPVQT  540

Query  541  PMAIPPASSGSSSSTSVSTLAGASIPAYPASPSPSSQVGSLQSPRQPRSPMGFPVGVFVS  600
            PMAIPPASSGSSSSTSVSTLAGASIPAYPASPSPSSQVGSLQSPRQPRSPMGFPVGVFVS
Sbjct  541  PMAIPPASSGSSSSTSVSTLAGASIPAYPASPSPSSQVGSLQSPRQPRSPMGFPVGVFVS  600

Query  601  IADPMSAPTASSQPHPYQYPHSHAHVQTHSGRGKPRRGMSSSAHEESSPGPSSGSVSVPV  660
            IADPMSAPTASSQPHPYQYPHSHAHVQTHSGRGKPRRGMSSSAHEESSPGPSSGSVSVPV
Sbjct  601  IADPMSAPTASSQPHPYQYPHSHAHVQTHSGRGKPRRGMSSSAHEESSPGPSSGSVSVPV  660

Query  661  SGSDPSPAPPASSSSSSEEVTKDVLTSILKTSSTLHNGSSHPPLRPPPLISSTSSFLGHP  720
            SGSDPSPAPPASSSSSSEEVTKDVLTSILKTSSTLHNGSSHPPLRPPPLISSTSSFLGHP
Sbjct  661  SGSDPSPAPPASSSSSSEEVTKDVLTSILKTSSTLHNGSSHPPLRPPPLISSTSSFLGHP  720

Query  721  TSQTQPTVGMSKEGSTENGDEGKGRESEEDEDQEISFASIMSYDANASPLPRPEGDDIED  780
            TSQTQPTVGMSKEGSTENGDEGKGRESEEDEDQEISFASIMSYDANASPLPRPEGDDIED
Sbjct  721  TSQTQPTVGMSKEGSTENGDEGKGRESEEDEDQEISFASIMSYDANASPLPRPEGDDIED  780

Query  781  GQIGSGGGFFGDQSSRKEQDEDERFAIGLDLPQPTSGSEVVNDRKKEADEESVATPTVEA  840
            GQIGSGGGFFGDQSSRKEQDEDERFAIGLDLPQPTSGSEVVNDRKKEADEESVATPTVEA
Sbjct  781  GQIGSGGGFFGDQSSRKEQDEDERFAIGLDLPQPTSGSEVVNDRKKEADEESVATPTVEA  840

Query  841  PIGSNGT  847
            PIGSNGT
Sbjct  841  PIGSNGT  847


Lambda      K        H        a         alpha
   0.305    0.120    0.328    0.792     4.96 

Gapped
Lambda      K        H        a         alpha    sigma
   0.267   0.0410    0.140     1.90     42.6     43.6 

Effective search space used: 10048569038064


  Database: nr
    Posted date:  Sep 23, 2015 12:05 AM
  Number of letters in database: 26,053,659,533
  Number of sequences in database:  71,551,133


Matrix: BLOSUM62
Gap Penalties: Existence: 11, Extension: 1
Neighboring words threshold: 11
Window for multiple hits: 40
```
