# Supplementary material for: Comparative genomics provides new insights into the diversity, physiology, and sexuality of the only industrially exploited tremellomycete: Phaffia rhodozyma
Source: BMC Genomics. 2016 Nov 9;17:901. doi: 10.1186/s12864-016-3244-7 (PMC5103461; doi:10.1186/s12864-016-3244-7)
Supplement: Additional file 6: — List of orphan genes with links to PFAM (related to Additional file 1: Table S1). (ZIP 1428 kb) [file 12864_2016_3244_MOESM6_ESM.zip › BLAST_HTML_FTR/G05788_P.html]

BLAST Search Results


```
BLASTP 2.2.27+


Reference:
Stephen F. Altschul, Thomas L. Madden, Alejandro A. Schäffer,
Jinghui Zhang, Zheng Zhang, Webb Miller, and David J. Lipman (1997),
"Gapped BLAST and PSI-BLAST: a new generation of protein database
search programs", Nucleic Acids Res. 25:3389-3402.


Reference for
composition-based statistics:
Alejandro A. Schäffer, L. Aravind, Thomas L. Madden, Sergei
Shavirin, John L. Spouge, Yuri I. Wolf, Eugene V. Koonin, and
Stephen F. Altschul (2001), "Improving the accuracy of PSI-BLAST
protein database searches with composition-based statistics and
other refinements", Nucleic Acids Res. 29:2994-3005.


Database: nr
           71,551,133 sequences; 26,053,659,533 total letters


Query= G05788_P

Length=315
                                                                      Score     E
Sequences producing significant alignments:                          (Bits)  Value

emb|CDZ96912.1|  hypothetical protein [Xanthophyllomyces dendrorh...   645    0.0  
ref|WP_023519501.1|  transcriptional antiterminator [Enterococcus...  48.9    0.005
ref|WP_010735716.1|  hypothetical protein [Enterococcus mundtii] ...  47.4    0.014
gb|KLT45178.1|  hypothetical protein CC85DRAFT_282672 [Trichospor...  45.1    0.043
ref|WP_045421908.1|  23S rRNA methyltransferase [Vibrio campbellii]   45.4    0.046
ref|WP_050914398.1|  23S rRNA methyltransferase [Vibrio harveyi]      44.3    0.11 
ref|WP_011939579.1|  pyridoxine 5'-phosphate synthase [Geobacter ...  43.1    0.18 
ref|WP_012128915.1|  23S rRNA (uracil(1939)-C(5))-methyltransfera...  43.5    0.21 
ref|WP_010650350.1|  23S rRNA methyltransferase [Vibrio campbellii]   43.5    0.22 
gb|EJT51935.1|  hypothetical protein A1Q1_06804 [Trichosporon asa...  42.7    0.26 
ref|WP_041485613.1|  hypothetical protein [gamma proteobacterium ...  40.0    0.51 
ref|XP_007874280.1|  hypothetical protein PNEG_02286 [Pneumocysti...  40.0    3.2  
ref|XP_662350.1|  hypothetical protein AN4746.2 [Aspergillus nidu...  39.7    3.5  
ref|XP_013325118.1|  hypothetical protein T310_7543 [Rasamsonia e...  39.3    4.2  
ref|XP_013869298.1|  PREDICTED: sickle tail protein homolog [Aust...  38.9    7.7  


 >emb|CDZ96912.1| hypothetical protein [Xanthophyllomyces dendrorhous]
Length=314

 Score =  645 bits (1664),  Expect = 0.0, Method: Compositional matrix adjust.
 Identities = 314/314 (100%), Positives = 314/314 (100%), Gaps = 0/314 (0%)

Query  1    MSFPSSLRSALSSRAPFSHLSPIFLASHRLYATVPSSPKSTTTKGIINQAAAERVESARL  60
            MSFPSSLRSALSSRAPFSHLSPIFLASHRLYATVPSSPKSTTTKGIINQAAAERVESARL
Sbjct  1    MSFPSSLRSALSSRAPFSHLSPIFLASHRLYATVPSSPKSTTTKGIINQAAAERVESARL  60

Query  61   KAEELRAQAAKLADDVDIALQTKEGRRRLNEMPIVISVYDNPVPYRSKNKDDTLIEYIQH  120
            KAEELRAQAAKLADDVDIALQTKEGRRRLNEMPIVISVYDNPVPYRSKNKDDTLIEYIQH
Sbjct  61   KAEELRAQAAKLADDVDIALQTKEGRRRLNEMPIVISVYDNPVPYRSKNKDDTLIEYIQH  120

Query  121  RFGAFYQSRDAMTEYFKTPKWSKAELMYRLGFGEGVERRSSLLKELEGRFEAFKKAEVIG  180
            RFGAFYQSRDAMTEYFKTPKWSKAELMYRLGFGEGVERRSSLLKELEGRFEAFKKAEVIG
Sbjct  121  RFGAFYQSRDAMTEYFKTPKWSKAELMYRLGFGEGVERRSSLLKELEGRFEAFKKAEVIG  180

Query  181  DRRTVETVSCPPIRQSVMTKLNSPTRNSRLRQATWTKTRSVSKPKLISFFRMPIHPKLVM  240
            DRRTVETVSCPPIRQSVMTKLNSPTRNSRLRQATWTKTRSVSKPKLISFFRMPIHPKLVM
Sbjct  181  DRRTVETVSCPPIRQSVMTKLNSPTRNSRLRQATWTKTRSVSKPKLISFFRMPIHPKLVM  240

Query  241  WQALVRFHTEQTIQIPATKPTEQEIVKTQTLVEDVLFERRDWLEPKPDWRIRDFVPTHGI  300
            WQALVRFHTEQTIQIPATKPTEQEIVKTQTLVEDVLFERRDWLEPKPDWRIRDFVPTHGI
Sbjct  241  WQALVRFHTEQTIQIPATKPTEQEIVKTQTLVEDVLFERRDWLEPKPDWRIRDFVPTHGI  300

Query  301  KEDYTKELPIERFQ  314
            KEDYTKELPIERFQ
Sbjct  301  KEDYTKELPIERFQ  314


>ref|WP_023519501.1| transcriptional antiterminator [Enterococcus mundtii]
 dbj|BAO06692.1| transcriptional antiterminator [Enterococcus mundtii QU 25]
Length=490

 Score = 48.9 bits (115),  Expect = 0.005, Method: Compositional matrix adjust.
 Identities = 58/219 (26%), Positives = 97/219 (44%), Gaps = 42/219 (19%)

Query  108  KNKDDTLIEYIQHRFGAFYQSRDAMTEYFKTPKWSKAELM---YRLG-----FGEGVERR  159
            KNK + +IEY++++FG   ++RD +        W+   ++   Y L      F + V+R 
Sbjct  285  KNKLNQVIEYLENKFGIICENRDQLIH---DIYWTTMNMVRPTYILHNKKREFFQNVKRD  341

Query  160  S-SLLKELEGRFEAFKKAEVIGDRRTVETVSCPPIRQSVMTKLNSPTRNSRLRQATWTKT  218
            S +L+K+LE RF  F+  + +G      +     I QSV   L S +         W + 
Sbjct  342  SPTLVKDLEDRF--FEIYQTLG--SAFHSYLDEMIHQSVFKLLTSWS-------DLWVQV  390

Query  219  RSVSKPKL-ISFFRMPIHPKLVMWQALVRFHTEQTIQI----PATKPTEQEIVKTQTLVE  273
            R   K KL ++      +  + M +  ++F+    I I    PATK     + K   L+ 
Sbjct  391  RK-QKIKLNVALLLDSSYDHMCMLKEEIKFYFRHNINIEILHPATKIQNDYLKKFDCLLT  449

Query  274  DVLFERRDWLEPKPDWRIRDFVPTHGIKEDYTKELPIER  312
            D+ F   D+ E          VPT GI  +Y  E+ I++
Sbjct  450  DIYF--NDYFE----------VPTLGIS-NYLDEVIIDK  475


>ref|WP_010735716.1| hypothetical protein [Enterococcus mundtii]
 gb|EOH61331.1| hypothetical protein UAC_01896 [Enterococcus mundtii ATCC 882]
 gb|EOU12396.1| hypothetical protein I587_00942 [Enterococcus mundtii ATCC 882]
Length=490

 Score = 47.4 bits (111),  Expect = 0.014, Method: Compositional matrix adjust.
 Identities = 58/219 (26%), Positives = 96/219 (44%), Gaps = 42/219 (19%)

Query  108  KNKDDTLIEYIQHRFGAFYQSRDAMTEYFKTPKWSKAELM---YRLG-----FGEGVERR  159
            KNK + +IEY++++FG   ++RD +        W+   ++   Y L      F + V+R 
Sbjct  285  KNKLNQVIEYLENKFGIVCENRDQLIH---DIYWTTMNMVRPTYILHNKKREFFQNVKRD  341

Query  160  S-SLLKELEGRFEAFKKAEVIGDRRTVETVSCPPIRQSVMTKLNSPTRNSRLRQATWTKT  218
            S +L+K+LE RF  F+  + +G      +     I QSV   L S +         W + 
Sbjct  342  SPTLVKDLEDRF--FEIYQTLG--SAFHSYLDEMIHQSVFKLLTSWS-------DLWVQV  390

Query  219  RSVSKPKL-ISFFRMPIHPKLVMWQALVRFHTEQTIQI----PATKPTEQEIVKTQTLVE  273
            R   K KL ++      +  + M +  ++F+    I I    PATK     + K   L+ 
Sbjct  391  RK-QKIKLHVALLLDSSYDHMCMLKEEIKFYFRHNINIEILHPATKIQNDYLKKFDCLLT  449

Query  274  DVLFERRDWLEPKPDWRIRDFVPTHGIKEDYTKELPIER  312
            D+ F   D+ E          VPT GI  +Y  E  I++
Sbjct  450  DIYF--NDYFE----------VPTLGIS-NYLDEAIIDK  475


>gb|KLT45178.1| hypothetical protein CC85DRAFT_282672 [Trichosporon oleaginosus]
Length=270

 Score = 45.1 bits (105),  Expect = 0.043, Method: Compositional matrix adjust.
 Identities = 43/173 (25%), Positives = 76/173 (44%), Gaps = 26/173 (15%)

Query  129  RDAMTEYFKTPKWSKAELMYRLGFGEGVERRSSLLKELEGRFEAFKKAEVIGDRRTVETV  188
            +D +  + KT  +S        G G+ +E    ++ E   R+EAF +A+   D R + ++
Sbjct  115  KDGLVPWVKTGWFS--------GSGKAME---GIMPEFVHRYEAFNRAQASADARALPSL  163

Query  189  SCPPIRQSVMTKLNSPTRNSRLRQATWTKTRSVSKPKLISFFRMPI----HPKLVMWQAL  244
            +  P     + +        +   A W      + PKL     +P+     PKL   QA 
Sbjct  164  ASGP----ALKRARDVAAKMQRVNAAWAVEAEHAPPKLQWVRYVPLMGVDGPKLA--QAC  217

Query  245  VRFHTEQTIQIPATKPTEQEIVKTQTLVEDVLFERRDWLEPKPDWRIRDFVPT  297
            V F T Q +    T P  +   K+Q + E+V+FER +   P   W++++ + T
Sbjct  218  VTFDTTQAL---TTGPKGKRETKSQRVRENVVFERSN--APNSPWKVKNMLET  265


>ref|WP_045421908.1| 23S rRNA methyltransferase [Vibrio campbellii]
Length=439

 Score = 45.4 bits (106),  Expect = 0.046, Method: Compositional matrix adjust.
 Identities = 37/138 (27%), Positives = 60/138 (43%), Gaps = 11/138 (8%)

Query  162  LLKELEGRFEAFKKAEVIGDRRTVETVSCPPIRQSVMTKLNSPTRNSRLRQATWTKTRSV  221
            LL E+    +AFKK E +G    V   + P I    ++KL     NS +  A    T   
Sbjct  179  LLPEIYSTLKAFKKPEQLGHVELVLGDNGPCITLRHLSKLTEKETNSLVELAKRHNT---  235

Query  222  SKPKLISFFRMPIHPKLVMWQALVRFHTEQTIQIPATKPTEQEIVKTQTLVEDVLFERRD  281
                  S + MP   +L + +  V F+ E  + +P T     ++   Q + + ++ +  D
Sbjct  236  ------SLYLMPETDQLDLVEGEVPFYQEAGVTVPFTPNNFIQV--NQAVNQKMVAQAVD  287

Query  282  WLEPKPDWRIRDFVPTHG  299
            WL+PK D R+ D     G
Sbjct  288  WLDPKSDERVLDLFCGLG  305


>ref|WP_050914398.1| 23S rRNA methyltransferase [Vibrio harveyi]
Length=439

 Score = 44.3 bits (103),  Expect = 0.11, Method: Compositional matrix adjust.
 Identities = 36/138 (26%), Positives = 61/138 (44%), Gaps = 11/138 (8%)

Query  162  LLKELEGRFEAFKKAEVIGDRRTVETVSCPPIRQSVMTKLNSPTRNSRLRQATWTKTRSV  221
            LL E+    +AFKK E +G    V   + P I    ++KL     NS +          +
Sbjct  179  LLPEIYSTLKAFKKPEQLGHVELVLGDNGPCITLRHLSKLTEKETNSLVE---------L  229

Query  222  SKPKLISFFRMPIHPKLVMWQALVRFHTEQTIQIPATKPTEQEIVKTQTLVEDVLFERRD  281
            +K    S + MP   +L + +  V F+ E  + +P T     ++   Q + + ++ +  D
Sbjct  230  AKRHQASLYLMPETDQLDLVEGEVPFYQEAGVTVPFTPNNFIQV--NQAVNQKMVAQAVD  287

Query  282  WLEPKPDWRIRDFVPTHG  299
            WL+PK D R+ D     G
Sbjct  288  WLDPKSDERVLDLFCGLG  305


>ref|WP_011939579.1| pyridoxine 5'-phosphate synthase [Geobacter uraniireducens]
 sp|A5G535|PDXJ_GEOUR RecName: Full=Pyridoxine 5'-phosphate synthase; Short=PNP synthase
 gb|ABQ26903.1| pyridoxine 5'-phosphate synthase [Geobacter uraniireducens Rf4]
Length=239

 Score = 43.1 bits (100),  Expect = 0.18, Method: Compositional matrix adjust.
 Identities = 27/81 (33%), Positives = 47/81 (58%), Gaps = 2/81 (2%)

Query  75   DVDIALQT-KEGRRRLNEMPIVISVYDNPVPYRSKNKDDTLIEYIQHRFGAFYQSRDAMT  133
            DV + L++ KE  +RL +  +++S++ +P P + K  D T  +YI+   GAF ++RD  +
Sbjct  106  DVRLNLESIKEAVQRLQDGGLIVSLFIDPDPDQIKAADKTGADYIEIHTGAFAEARDWKS  165

Query  134  EYFKTPKWSKA-ELMYRLGFG  153
            E  +  K   A +L  +LG G
Sbjct  166  EQAELAKIENAIKLAGKLGMG  186


>ref|WP_012128915.1| 23S rRNA (uracil(1939)-C(5))-methyltransferase RlmD [Vibrio campbellii]
 sp|A7MTS9|RLMD_VIBCB RecName: Full=23S rRNA (uracil(1939)-C(5))-methyltransferase 
RlmD; AltName: Full=23S rRNA(m5U1939)-methyltransferase
 gb|ABU72465.1| hypothetical protein VIBHAR_03529 [Vibrio campbellii ATCC BAA-1116]
 gb|AGU95353.1| 23S rRNA methyltransferase [Vibrio campbellii ATCC BAA-1116]
Length=439

 Score = 43.5 bits (101),  Expect = 0.21, Method: Compositional matrix adjust.
 Identities = 35/138 (25%), Positives = 60/138 (43%), Gaps = 11/138 (8%)

Query  162  LLKELEGRFEAFKKAEVIGDRRTVETVSCPPIRQSVMTKLNSPTRNSRLRQATWTKTRSV  221
            LL E+    +AFKK E +G    V   + P I    + KL     N+ +          +
Sbjct  179  LLPEIYSTLKAFKKPEQLGHVELVLGDNAPCITLRHLNKLTEKETNTLVE---------L  229

Query  222  SKPKLISFFRMPIHPKLVMWQALVRFHTEQTIQIPATKPTEQEIVKTQTLVEDVLFERRD  281
            +K    S + MP   +L + +  V F+ E  + +P T     ++   Q + + ++ +  D
Sbjct  230  AKRHQASLYLMPETDQLDLVEGEVPFYQEAGVTVPFTPNNFIQV--NQAVNQKMVAQAVD  287

Query  282  WLEPKPDWRIRDFVPTHG  299
            WL+PK D R+ D     G
Sbjct  288  WLDPKSDERVLDLFCGLG  305


>ref|WP_010650350.1| 23S rRNA methyltransferase [Vibrio campbellii]
Length=439

 Score = 43.5 bits (101),  Expect = 0.22, Method: Compositional matrix adjust.
 Identities = 35/138 (25%), Positives = 60/138 (43%), Gaps = 11/138 (8%)

Query  162  LLKELEGRFEAFKKAEVIGDRRTVETVSCPPIRQSVMTKLNSPTRNSRLRQATWTKTRSV  221
            LL E+    +AFKK E +G    V   + P I    + KL     N+ +          +
Sbjct  179  LLPEIYSTLKAFKKPEQLGHVELVLGDNAPCITLRHLNKLTEKETNTLVE---------L  229

Query  222  SKPKLISFFRMPIHPKLVMWQALVRFHTEQTIQIPATKPTEQEIVKTQTLVEDVLFERRD  281
            +K    S + MP   +L + +  V F+ E  + +P T     ++   Q + + ++ +  D
Sbjct  230  AKRHQASLYLMPETDQLDLVEGKVPFYQEAGVTVPFTPNNFIQV--NQAVNQKMVAQAVD  287

Query  282  WLEPKPDWRIRDFVPTHG  299
            WL+PK D R+ D     G
Sbjct  288  WLDPKSDERVLDLFCGLG  305


>gb|EJT51935.1| hypothetical protein A1Q1_06804 [Trichosporon asahii var. asahii 
CBS 2479]
 gb|EKD02544.1| hypothetical protein A1Q2_03140 [Trichosporon asahii var. asahii 
CBS 8904]
Length=237

 Score = 42.7 bits (99),  Expect = 0.26, Method: Compositional matrix adjust.
 Identities = 37/148 (25%), Positives = 66/148 (45%), Gaps = 24/148 (16%)

Query  166  LEGRFEAFKKAEVIGDRRTVETVSCPPIRQSVMTKLNSPTRNSRLRQATWTKTRSVSKPK  225
            LE  ++ + +A+  GD   ++ +S  P     + +  +  +    ++  W   R V+ P+
Sbjct  103  LEDVYKEYNQAQARGDITALKNLSMGP----ALDRATTVAKALSGKKGQWRFIRHVTPPR  158

Query  226  LISFFRMPI--HPKLVMWQALVRFHTEQTIQIPATKPTEQEIVKTQTLVEDVLFERRDWL  283
            ++S    P+  H K+   Q  V++ TEQ    P   P      KT  + E ++F+R    
Sbjct  159  IVSVRTGPMDMHGKMTATQITVQYDTEQEFTAPKRAP------KTSRVREYIVFDRPH--  210

Query  284  EPKPDWRIRDFVPTHGIKEDYTKELPIE  311
            EP    +I+DFV          KE PI+
Sbjct  211  EPSGSLKIKDFV----------KETPID  228


>ref|WP_041485613.1| hypothetical protein [gamma proteobacterium HdN1]
Length=107

 Score = 40.0 bits (92),  Expect = 0.51, Method: Compositional matrix adjust.
 Identities = 29/91 (32%), Positives = 42/91 (46%), Gaps = 12/91 (13%)

Query  52   AERVESAR--LKAEELRAQAAKLADDVDIALQTKEGRRRLNEMPIVISVYDNPVPYRSKN  109
            A+RVE+ R  ++ +E  A     A  +DI+ Q    RRRL           N     SK 
Sbjct  27   AQRVEAQRALVEGQEC-AMVEGKAKRMDISKQKLGKRRRLGR---------NAEGSFSKT  76

Query  110  KDDTLIEYIQHRFGAFYQSRDAMTEYFKTPK  140
            +D T++    HRFG ++Q R  +     TPK
Sbjct  77   RDCTVVNACNHRFGNYFQGRTIINRLTSTPK  107


>ref|XP_007874280.1| hypothetical protein PNEG_02286 [Pneumocystis murina B123]
 gb|EMR09331.1| hypothetical protein PNEG_02286 [Pneumocystis murina B123]
Length=557

 Score = 40.0 bits (92),  Expect = 3.2, Method: Compositional matrix adjust.
 Identities = 28/121 (23%), Positives = 60/121 (50%), Gaps = 8/121 (7%)

Query  168  GRF-EAFKKAEVIGDRRTVETVSCPPIRQSVMTKLNSPTRNSRLRQATWTKTRSVSKPKL  226
            G+F E   +A   GD + ++++      +++ +++       R +   W   R +  PKL
Sbjct  404  GKFYEKVNQAYANGDIKEIDSICGKAYAKTLKSQIAKRPEALRFK---WVLHRMIKPPKL  460

Query  227  ISF--FRMPIHPKLVMWQALVRFHTEQTIQIPATKPTEQEIVKTQTLVEDVLFERRDWLE  284
            +SF   ++ I     + QA+ R H+EQ++ I +    ++E +K    +E  +F+ + W+ 
Sbjct  461  VSFSQAQVEIDSNKYLAQAVYRIHSEQSLTIFSDGLKKEEFLK--EFIEYYVFQYKTWVL  518

Query  285  P  285
            P
Sbjct  519  P  519


>ref|XP_662350.1| hypothetical protein AN4746.2 [Aspergillus nidulans FGSC A4]
 gb|EAA60788.1| predicted protein [Aspergillus nidulans FGSC A4]
 tpe|CBF76876.1| TPA: conserved hypothetical protein [Aspergillus nidulans FGSC 
A4]
Length=398

 Score = 39.7 bits (91),  Expect = 3.5, Method: Compositional matrix adjust.
 Identities = 39/189 (21%), Positives = 81/189 (43%), Gaps = 36/189 (19%)

Query  132  MTEYFKTPK--------WSKAELMYRLGFGEG------VERRSSLLKELEGR-FEAFKKA  176
            M   F+ PK        W K+     LG  +G      ++ R  + +E+  R + AF K 
Sbjct  178  MPSIFQQPKERLHYEWLWLKSWFQNFLGKTDGRGLPLRLKERRQVAREMHQRMYSAFAK-  236

Query  177  EVIGDRRTVETVSCPPIRQSVMTKLNSPTRNSRLRQATWTKTRSVSKP-------KLIS-  228
               GD  T+  + C  +  +++ ++ +  +  ++   TW+  + +  P       +++S 
Sbjct  237  ---GDSATLRNICCTGLANNLIGRIEARRKGEKV---TWSLDKYIRSPSTWFTGMRVVSD  290

Query  229  -FFRMPIHPKLVMWQALVRFHTEQTI-----QIPATKPTEQEIVKTQTLVEDVLFERRDW  282
               ++P  P   + Q ++R  + Q+      QIP    + +  VK Q   E ++ ++  W
Sbjct  291  RATQIPELPDSGVRQVVLRITSRQSTGKVKPQIPGVAVSAENAVKQQDCTEYIVLQKLRW  350

Query  283  LEPKPDWRI  291
            +  +  WRI
Sbjct  351  MGEEESWRI  359


>ref|XP_013325118.1| hypothetical protein T310_7543 [Rasamsonia emersonii CBS 393.64]
 gb|KKA18506.1| hypothetical protein T310_7543 [Rasamsonia emersonii CBS 393.64]
Length=322

 Score = 39.3 bits (90),  Expect = 4.2, Method: Compositional matrix adjust.
 Identities = 47/196 (24%), Positives = 82/196 (42%), Gaps = 38/196 (19%)

Query  125  FYQSRDAM-TEYFKTPKWSK---AELMYRLGFGEGV-----ERRSSLLKELEGRFEAFKK  175
            F+Q R+ +  E+     W++     L Y   F +G+     ERR    +  +  + AF  
Sbjct  98   FHQPRERLRMEWLSLKMWAQNLMGVLAYSKYFNKGLPLRFRERRKVARELHQKMYTAF--  155

Query  176  AEVIGDRRTVETVSCPPIRQSVMTKLNSPTRNSRLRQATWTKTRSVSKPKLISFF-----  230
                GD  T+  + C  +  S+  ++     N R+   TW+  + +  P   +FF     
Sbjct  156  --AAGDVHTLRKICCTGLANSLSARIAKRPSNERV---TWSLDKYIRSPS--TFFTGVRV  208

Query  231  ------RMPIHPKLVMWQALVRFHTEQ-TIQIPAT--------KPTEQEIVKTQTLVEDV  275
                  ++P  PK  + Q +VR  + Q T  +P T        +PT    VK Q   E +
Sbjct  209  VSDRATQIPEMPKSGIRQVVVRITSRQSTGTVPVTLKRGSKEAEPTGPPKVKEQDCTEYI  268

Query  276  LFERRDWLEPKPDWRI  291
            + +R  W+  + +WRI
Sbjct  269  VLQRIMWMGEEEEWRI  284


>ref|XP_013869298.1| PREDICTED: sickle tail protein homolog [Austrofundulus limnaeus]
Length=1801

 Score = 38.9 bits (89),  Expect = 7.7, Method: Compositional matrix adjust.
 Identities = 44/150 (29%), Positives = 63/150 (42%), Gaps = 19/150 (13%)

Query  159   RSSLLKELEGR-FEAFKKAEVIGDRRTVETVSCPPIRQSVMTKLNSPTRNSRLRQATWTK  217
             RS+  KE EG   +  + +  + +R        PP      +K    T  S++ +AT+T 
Sbjct  1168  RSTCTKEGEGNEMKKLQASAELSNRNN------PPDGFKQNSKGKRKTSPSKVSKATFTN  1221

Query  218   TRSVSKPKLISFFR------MPIHPKLVM--WQALVRFHTEQTIQIPATKPT----EQEI  265
               SVS  KL  F R           K VM    AL+R   E+ I  P    T    E   
Sbjct  1222  NSSVSSDKLKDFHRDGSTRGFKHTEKKVMKPLSALIRAVVEEQISPPGAPQTSSVLEPPC  1281

Query  266   VKTQTLVEDVLFERRDWLEPKPDWRIRDFV  295
             V  QT  E+V  ++R +L+  PD   +D V
Sbjct  1282  VSVQTNTEEVTSQKRGFLKNTPDQTNKDKV  1311


Lambda      K        H        a         alpha
   0.320    0.133    0.390    0.792     4.96 

Gapped
Lambda      K        H        a         alpha    sigma
   0.267   0.0410    0.140     1.90     42.6     43.6 

Effective search space used: 2527963281195


  Database: nr
    Posted date:  Sep 23, 2015 12:05 AM
  Number of letters in database: 26,053,659,533
  Number of sequences in database:  71,551,133


Matrix: BLOSUM62
Gap Penalties: Existence: 11, Extension: 1
Neighboring words threshold: 11
Window for multiple hits: 40
```
