# Supplementary material for: Comparative genomics provides new insights into the diversity, physiology, and sexuality of the only industrially exploited tremellomycete: Phaffia rhodozyma
Source: BMC Genomics. 2016 Nov 9;17:901. doi: 10.1186/s12864-016-3244-7 (PMC5103461; doi:10.1186/s12864-016-3244-7)
Supplement: Additional file 6: — List of orphan genes with links to PFAM (related to Additional file 1: Table S1). (ZIP 1428 kb) [file 12864_2016_3244_MOESM6_ESM.zip › BLAST_HTML_FTR/G00515_P.html]

BLAST Search Results


```
BLASTP 2.2.27+


Reference:
Stephen F. Altschul, Thomas L. Madden, Alejandro A. Schäffer,
Jinghui Zhang, Zheng Zhang, Webb Miller, and David J. Lipman (1997),
"Gapped BLAST and PSI-BLAST: a new generation of protein database
search programs", Nucleic Acids Res. 25:3389-3402.


Reference for
composition-based statistics:
Alejandro A. Schäffer, L. Aravind, Thomas L. Madden, Sergei
Shavirin, John L. Spouge, Yuri I. Wolf, Eugene V. Koonin, and
Stephen F. Altschul (2001), "Improving the accuracy of PSI-BLAST
protein database searches with composition-based statistics and
other refinements", Nucleic Acids Res. 29:2994-3005.


Database: nr
           71,551,133 sequences; 26,053,659,533 total letters


Query= G00515_P

Length=481
                                                                      Score     E
Sequences producing significant alignments:                          (Bits)  Value

emb|CED83825.1|  hypothetical protein [Xanthophyllomyces dendrorh...   813    0.0  
ref|XP_007381883.1|  hypothetical protein PUNSTDRAFT_43308 [Punct...  41.2    2.0  


 >emb|CED83825.1| hypothetical protein [Xanthophyllomyces dendrorhous]
Length=473

 Score =  813 bits (2100),  Expect = 0.0, Method: Compositional matrix adjust.
 Identities = 470/480 (98%), Positives = 471/480 (98%), Gaps = 7/480 (1%)

Query  1    MSYPSINAKSRALPPATMPPRRRTFNLHSLVRRNSHELPLMKPSYALDIPSDISPVRPSR  60
            MSYPSINAKSRALPPATMPPRRRTFNLH LVRRNSHELPLMKPSYALDIPSDISPVRPSR
Sbjct  1    MSYPSINAKSRALPPATMPPRRRTFNLHLLVRRNSHELPLMKPSYALDIPSDISPVRPSR  60

Query  61   QLSSSVDETNQSVFDFAHSYSHSPPPATPQLLHTRSASSTLQSRSNLLPTCLLKEDEKVE  120
            QLSSSVDETNQSVFDFAHSYSHSPPPATPQLLHTRSASST QSRSNLLPTCLLKEDEKVE
Sbjct  61   QLSSSVDETNQSVFDFAHSYSHSPPPATPQLLHTRSASSTPQSRSNLLPTCLLKEDEKVE  120

Query  121  DESGSIGSRSTSSSGIPIFFKERGGEYELFDSPFTPPTLPIGLEEEDKMNVFGSYPISPR  180
            DESGSIGSRSTSSSGIPIFFKERGGEYELFDSPFTPPTLPIGLEEEDKMNVFGSYPISPR
Sbjct  121  DESGSIGSRSTSSSGIPIFFKERGGEYELFDSPFTPPTLPIGLEEEDKMNVFGSYPISPR  180

Query  181  TTSFPQATVRPQPQDFYSPRDKFPVSMSYSSSFSVPSISVSPSSIPCARMSPISHAKAPF  240
            TTSFPQATVRPQPQDFYSPRDKFPVSMSYSSSFSVPSISVSPSSIPCARMSPISHAKAPF
Sbjct  181  TTSFPQATVRPQPQDFYSPRDKFPVSMSYSSSFSVPSISVSPSSIPCARMSPISHAKAPF  240

Query  241  NAILLSQKTMQLPSQPAGFPSSQRKSLTLIQLQVAASTFVIPMELIARYPSHLARFISGC  300
            NAILLSQKTMQLPSQPAGFPSSQRKSLTLIQLQVAASTFVIPMELIARYPSHLARFISGC
Sbjct  241  NAILLSQKTMQLPSQPAGFPSSQRKSLTLIQLQVAASTFVIPMELIARYPSHLARFISGC  300

Query  301  TDDDWRATDSDDSFSEAETDFDDDQTSSESSFSYLSPSGFFVSDGEIDGPTTPRAATTNF  360
            TDDDWRATDSDDSFSEAETDFDDDQTSSESSFSY       +SDGEIDGPTTPRAATTNF
Sbjct  301  TDDDWRATDSDDSFSEAETDFDDDQTSSESSFSY-------LSDGEIDGPTTPRAATTNF  353

Query  361  ARVPPPRPPRPETPPMSPSRPSMRRLEILLCRSPEPYIAIDYFFSTGLLLPDLRSMLESE  420
            ARVPPPRPPRPETPPMSPSRPSMRRLEILLCRSPEPYIAIDYFFSTGLLLPDLRSMLESE
Sbjct  354  ARVPPPRPPRPETPPMSPSRPSMRRLEILLCRSPEPYIAIDYFFSTGLLLPDLRSMLESE  413

Query  421  NMLKGCPLCGDSQRASRLREVENEASWLGLDCLAQVCRAERKRSGIEETRPTKKVGDGWI  480
            NMLKGCPLCGDSQRASRLREVENEASWLGLDCLAQVCRAERKRSGIEETRPTKKVGDGWI
Sbjct  414  NMLKGCPLCGDSQRASRLREVENEASWLGLDCLAQVCRAERKRSGIEETRPTKKVGDGWI  473


>ref|XP_007381883.1| hypothetical protein PUNSTDRAFT_43308 [Punctularia strigosozonata 
HHB-11173 SS5]
 gb|EIN10371.1| hypothetical protein PUNSTDRAFT_43308 [Punctularia strigosozonata 
HHB-11173 SS5]
Length=445

 Score = 41.2 bits (95),  Expect = 2.0, Method: Compositional matrix adjust.
 Identities = 47/186 (25%), Positives = 80/186 (43%), Gaps = 29/186 (16%)

Query  239  PFNAILLSQKTMQLPSQPAGFPSSQRKSLTLIQLQVAASTFVIPMELIARYPSHLARFIS  298
            PF+AILLS         P G    ++   T++ L+ + +T+   +  +    SHL+ +++
Sbjct  172  PFDAILLSDA-------PPGNADPKK---TIVVLETSTATYKTTLATLTSSESHLSTYLT  221

Query  299  GCTDDD----WRATDSDDSFSEAETDFDDDQTSSESSFS-YLSPSGFFVSDGE-------  346
               +D        + +DD+ S   T  + +     S F  +L+ +G   +          
Sbjct  222  SLREDGEDEVVVGSHADDTASVYSTQSETNTDLFNSLFQRHLATTGVITNRKPRVSPVHI  281

Query  347  -IDGPTTPRAATTNFARVP-----PPRPPRPETPPMSPSRPSMRRLEILL-CRSPEPYIA  399
             +D P+ P A   N+ R P     PP  PR     +S S PS  RLE LL  R    Y+ 
Sbjct  282  FLDRPSAPYAHILNYLRTPSTPDAPPMLPRAVQLFLSSSYPSRDRLESLLELRDEAKYLG  341

Query  400  IDYFFS  405
            +D  ++
Sbjct  342  LDALYT  347


Lambda      K        H        a         alpha
   0.316    0.131    0.391    0.792     4.96 

Gapped
Lambda      K        H        a         alpha    sigma
   0.267   0.0410    0.140     1.90     42.6     43.6 

Effective search space used: 4878014257268


  Database: nr
    Posted date:  Sep 23, 2015 12:05 AM
  Number of letters in database: 26,053,659,533
  Number of sequences in database:  71,551,133


Matrix: BLOSUM62
Gap Penalties: Existence: 11, Extension: 1
Neighboring words threshold: 11
Window for multiple hits: 40
```
