# Supplementary material for: Comparative genomics provides new insights into the diversity, physiology, and sexuality of the only industrially exploited tremellomycete: Phaffia rhodozyma
Source: BMC Genomics. 2016 Nov 9;17:901. doi: 10.1186/s12864-016-3244-7 (PMC5103461; doi:10.1186/s12864-016-3244-7)
Supplement: Additional file 6: — List of orphan genes with links to PFAM (related to Additional file 1: Table S1). (ZIP 1428 kb) [file 12864_2016_3244_MOESM6_ESM.zip › BLAST_HTML_FTR/G01736_P.html]

BLAST Search Results


```
BLASTP 2.2.27+


Reference:
Stephen F. Altschul, Thomas L. Madden, Alejandro A. Schäffer,
Jinghui Zhang, Zheng Zhang, Webb Miller, and David J. Lipman (1997),
"Gapped BLAST and PSI-BLAST: a new generation of protein database
search programs", Nucleic Acids Res. 25:3389-3402.


Reference for
composition-based statistics:
Alejandro A. Schäffer, L. Aravind, Thomas L. Madden, Sergei
Shavirin, John L. Spouge, Yuri I. Wolf, Eugene V. Koonin, and
Stephen F. Altschul (2001), "Improving the accuracy of PSI-BLAST
protein database searches with composition-based statistics and
other refinements", Nucleic Acids Res. 29:2994-3005.


Database: nr
           71,551,133 sequences; 26,053,659,533 total letters


Query= G01736_P

Length=422
                                                                      Score     E
Sequences producing significant alignments:                          (Bits)  Value

emb|CDZ96196.1|  hypothetical protein [Xanthophyllomyces dendrorh...   864    0.0  
ref|WP_020892638.1|  hypothetical protein [Cyclobacterium qasimii...  43.1    0.49 
ref|WP_014020049.1|  hypothetical protein [Cyclobacterium marinum...  40.4    3.7  


 >emb|CDZ96196.1| hypothetical protein [Xanthophyllomyces dendrorhous]
Length=421

 Score =  864 bits (2232),  Expect = 0.0, Method: Compositional matrix adjust.
 Identities = 421/421 (100%), Positives = 421/421 (100%), Gaps = 0/421 (0%)

Query  1    MFASTRATQHLHLRPAAARFFSSTLATPQSSGPKSSQQANPENAASMEFVPQGTPPVSRA  60
            MFASTRATQHLHLRPAAARFFSSTLATPQSSGPKSSQQANPENAASMEFVPQGTPPVSRA
Sbjct  1    MFASTRATQHLHLRPAAARFFSSTLATPQSSGPKSSQQANPENAASMEFVPQGTPPVSRA  60

Query  61   SRGSRGRGDGGGSVKLLRVYWNKHRSPDRIRSSQAAHGQFDDAPAQFKNKPSGNRANRPS  120
            SRGSRGRGDGGGSVKLLRVYWNKHRSPDRIRSSQAAHGQFDDAPAQFKNKPSGNRANRPS
Sbjct  61   SRGSRGRGDGGGSVKLLRVYWNKHRSPDRIRSSQAAHGQFDDAPAQFKNKPSGNRANRPS  120

Query  121  ESRRSDDSVTIQLDSPTRRRMEERTPQGQIIKHYQDKAQGKFARFGPGRGGAPNSSGERR  180
            ESRRSDDSVTIQLDSPTRRRMEERTPQGQIIKHYQDKAQGKFARFGPGRGGAPNSSGERR
Sbjct  121  ESRRSDDSVTIQLDSPTRRRMEERTPQGQIIKHYQDKAQGKFARFGPGRGGAPNSSGERR  180

Query  181  PYVPRENGPSSRYGGAGGARDRKPSNKSAAPRKQQYTGDPYRTIRELPTMAPSTLPGLHL  240
            PYVPRENGPSSRYGGAGGARDRKPSNKSAAPRKQQYTGDPYRTIRELPTMAPSTLPGLHL
Sbjct  181  PYVPRENGPSSRYGGAGGARDRKPSNKSAAPRKQQYTGDPYRTIRELPTMAPSTLPGLHL  240

Query  241  LYQSDNILAGEEDAPTTAPPKITKGPKANVFPPQTAFQQKIHNVVNDQAPYAGIKMLRKK  300
            LYQSDNILAGEEDAPTTAPPKITKGPKANVFPPQTAFQQKIHNVVNDQAPYAGIKMLRKK
Sbjct  241  LYQSDNILAGEEDAPTTAPPKITKGPKANVFPPQTAFQQKIHNVVNDQAPYAGIKMLRKK  300

Query  301  NPLPFLPDTLSGNQPRLIKQRAKEYDMMVSPEFADVEGINEKIDNERVGGDYSRYYDRER  360
            NPLPFLPDTLSGNQPRLIKQRAKEYDMMVSPEFADVEGINEKIDNERVGGDYSRYYDRER
Sbjct  301  NPLPFLPDTLSGNQPRLIKQRAKEYDMMVSPEFADVEGINEKIDNERVGGDYSRYYDRER  360

Query  361  LDQDVKTLNNGDKSAKENGKILAEYGVSWNGDLGMREREMIKSLVDEFMGMKLTKPKETA  420
            LDQDVKTLNNGDKSAKENGKILAEYGVSWNGDLGMREREMIKSLVDEFMGMKLTKPKETA
Sbjct  361  LDQDVKTLNNGDKSAKENGKILAEYGVSWNGDLGMREREMIKSLVDEFMGMKLTKPKETA  420

Query  421  K  421
            K
Sbjct  421  K  421


>ref|WP_020892638.1| hypothetical protein [Cyclobacterium qasimii]
 gb|EPR65827.1| hypothetical protein ADICYQ_5175 [Cyclobacterium qasimii M12-11B]
Length=481

 Score = 43.1 bits (100),  Expect = 0.49, Method: Compositional matrix adjust.
 Identities = 46/168 (27%), Positives = 67/168 (40%), Gaps = 17/168 (10%)

Query  211  PRKQQYTGDPYRTIRELPTMAPSTLPGLHLLYQSDNILAGEEDAPTTAPPKITKGPKANV  270
            P K + +   YR       M   T  G    +  D I+ GE +  +  PPK+ KG K N 
Sbjct  302  PLKNEVSVGNYRLRENFSNMEVQTGEG----FTYDFIITGEGNISSIRPPKVNKGQKLNT  357

Query  271  FPPQTAFQ-QKIHNVVNDQAPYAGIKMLRKKNPLPFLPDTLSGNQPRLIKQRAKEYDMMV  329
            F P    Q  +    V     ++    L +   +P L D     +      +  EYD ++
Sbjct  358  FDPNEQVQINRGRGKVTGMKEFSYFITLNEAEVVP-LKDHF---EWIYFNPQLAEYDTLI  413

Query  330  SPEFADVEG---INEKIDNERVGGDYSRYYDRERLDQDVKTLNNGDKS  374
                  V G   IN+ I + R+GG     YD   ++ D K LN G KS
Sbjct  414  PQAVVSVTGESRINQAISSSRLGG----LYDLIEVE-DNKLLNQGYKS  456


>ref|WP_014020049.1| hypothetical protein [Cyclobacterium marinum]
 gb|AEL25754.1| hypothetical protein Cycma_2007 [Cyclobacterium marinum DSM 745]
Length=481

 Score = 40.4 bits (93),  Expect = 3.7, Method: Compositional matrix adjust.
 Identities = 49/170 (29%), Positives = 68/170 (40%), Gaps = 21/170 (12%)

Query  211  PRKQQYTGDPYRTIRELPTMAPSTLPGLHLLYQSDNILAGEEDAPTTAPPKITKGPKANV  270
            P K + +   YR    +  +   T  G    +  D I+ GE +  +  PPKI KG K N 
Sbjct  302  PLKNEVSVGNYRLRENISDLEVKTGEG----FTYDFIITGEGNISSIRPPKIKKGQKLNT  357

Query  271  FPPQTAFQ-QKIHNVVNDQAPYAGIKMLRKKNPLPFLPDTLSG--NQPRLIKQRAKEYDM  327
            F P    Q  +    V     ++    L +   +P L D        P L K     YD 
Sbjct  358  FDPNEQVQINRGRGKVTGMKEFSYFMTLNEAEVVP-LKDHFEWIYFNPELAK-----YDT  411

Query  328  MVSPEFADVEG---INEKIDNERVGGDYSRYYDRERLDQDVKTLNNGDKS  374
            +V      V G   IN+ I + R+GG     YD   + +D K LN G KS
Sbjct  412  LVPQAVVRVTGESRINQAISSSRLGG----LYDLIEV-EDNKLLNQGYKS  456


Lambda      K        H        a         alpha
   0.313    0.131    0.384    0.792     4.96 

Gapped
Lambda      K        H        a         alpha    sigma
   0.267   0.0410    0.140     1.90     42.6     43.6 

Effective search space used: 4063604433496


  Database: nr
    Posted date:  Sep 23, 2015 12:05 AM
  Number of letters in database: 26,053,659,533
  Number of sequences in database:  71,551,133


Matrix: BLOSUM62
Gap Penalties: Existence: 11, Extension: 1
Neighboring words threshold: 11
Window for multiple hits: 40
```
