# Supplementary material for: Comparative genomics provides new insights into the diversity, physiology, and sexuality of the only industrially exploited tremellomycete: Phaffia rhodozyma
Source: BMC Genomics. 2016 Nov 9;17:901. doi: 10.1186/s12864-016-3244-7 (PMC5103461; doi:10.1186/s12864-016-3244-7)
Supplement: Additional file 6: — List of orphan genes with links to PFAM (related to Additional file 1: Table S1). (ZIP 1428 kb) [file 12864_2016_3244_MOESM6_ESM.zip › BLAST_HTML_FTR/G04938_P.html]

BLAST Search Results


```
BLASTP 2.2.27+


Reference:
Stephen F. Altschul, Thomas L. Madden, Alejandro A. Schäffer,
Jinghui Zhang, Zheng Zhang, Webb Miller, and David J. Lipman (1997),
"Gapped BLAST and PSI-BLAST: a new generation of protein database
search programs", Nucleic Acids Res. 25:3389-3402.


Reference for
composition-based statistics:
Alejandro A. Schäffer, L. Aravind, Thomas L. Madden, Sergei
Shavirin, John L. Spouge, Yuri I. Wolf, Eugene V. Koonin, and
Stephen F. Altschul (2001), "Improving the accuracy of PSI-BLAST
protein database searches with composition-based statistics and
other refinements", Nucleic Acids Res. 29:2994-3005.


Database: nr
           71,551,133 sequences; 26,053,659,533 total letters


Query= G04938_P

Length=220
                                                                      Score     E
Sequences producing significant alignments:                          (Bits)  Value

emb|CED83421.1|  hypothetical protein [Xanthophyllomyces dendrorh...   290    1e-95
gb|KIL85072.1|  hypothetical protein FAVG1_11500 [Fusarium avenac...  44.7    0.037
gb|EXL70475.1|  hypothetical protein FOPG_13728 [Fusarium oxyspor...  43.5    0.071
gb|EGU75523.1|  hypothetical protein FOXB_13972 [Fusarium oxyspor...  43.5    0.072
ref|XP_003193725.1|  hypothetical protein CGB_D6430W [Cryptococcu...  43.5    0.10 
gb|KIR80870.1|  hypothetical protein I306_02327 [Cryptococcus gat...  43.5    0.10 
gb|KIR57121.1|  hypothetical protein I315_00284 [Cryptococcus gat...  43.5    0.11 
gb|EXM17501.1|  hypothetical protein FOTG_14340 [Fusarium oxyspor...  42.7    0.11 
gb|EWY99821.1|  hypothetical protein FOYG_03759 [Fusarium oxyspor...  42.0    0.23 
ref|XP_013132474.1|  PREDICTED: uncharacterized protein LOC100703...  41.2    0.50 
ref|XP_012049423.1|  hypothetical protein CNAG_01255 [Cryptococcu...  41.2    0.60 
gb|EMT63128.1|  hypothetical protein FOC4_g10013336 [Fusarium oxy...  40.0    1.1  
gb|KOG02963.1|  Uncharacterized protein ABJ98_1989 [Pseudomonas s...  39.3    2.5  
ref|XP_007135679.1|  hypothetical protein PHAVU_010G149300g [Phas...  39.3    3.0  
ref|XP_002845802.1|  HMG-CoA reductase [Arthroderma otae CBS 1134...  38.9    3.9  
gb|EXK88781.1|  hypothetical protein FOQG_08071 [Fusarium oxyspor...  38.5    4.0  
gb|KIO28875.1|  hypothetical protein M407DRAFT_21952 [Tulasnella ...  38.1    4.1  
gb|EXL56165.1|  hypothetical protein FOCG_03850 [Fusarium oxyspor...  38.1    4.3  
gb|EXA44284.1|  hypothetical protein FOVG_05756 [Fusarium oxyspor...  38.1    4.6  
ref|XP_641794.1|  hypothetical protein DDB_G0279325 [Dictyosteliu...  38.5    5.0  
gb|EWZ36703.1|  hypothetical protein FOZG_10669 [Fusarium oxyspor...  37.7    6.0  
gb|EXM19741.1|  hypothetical protein FOTG_12370 [Fusarium oxyspor...  37.7    6.4  
gb|EXK38733.1|  hypothetical protein FOMG_06273 [Fusarium oxyspor...  37.7    6.7  
gb|EWY90105.1|  hypothetical protein FOYG_07720 [Fusarium oxyspor...  37.7    7.0  
gb|EMD39191.1|  hypothetical protein CERSUDRAFT_112868 [Ceriporio...  37.4    9.0  
ref|XP_002604710.1|  hypothetical protein BRAFLDRAFT_80319 [Branc...  37.7    9.0  
gb|KKR33274.1|  Alpha-amylase [Parcubacteria (Falkowbacteria) bac...  37.7    9.4  
gb|ENH67497.1|  hypothetical protein FOC1_g10010774 [Fusarium oxy...  37.4    9.6  


 >emb|CED83421.1| hypothetical protein [Xanthophyllomyces dendrorhous]
Length=195

 Score =  290 bits (741),  Expect = 1e-95, Method: Compositional matrix adjust.
 Identities = 148/148 (100%), Positives = 148/148 (100%), Gaps = 0/148 (0%)

Query  72   ATLVDRAPFRPFPFSTIYPMPRASSSVLVASSSHTHASIKNGTTSSGLSRSVLIAVIIST  131
            ATLVDRAPFRPFPFSTIYPMPRASSSVLVASSSHTHASIKNGTTSSGLSRSVLIAVIIST
Sbjct  48   ATLVDRAPFRPFPFSTIYPMPRASSSVLVASSSHTHASIKNGTTSSGLSRSVLIAVIIST  107

Query  132  IITGLVLSALIGYLLYRRWNIRRNQSSESPPSTFAQQDRDPRLSSSSSTRTLFEKDLEKS  191
            IITGLVLSALIGYLLYRRWNIRRNQSSESPPSTFAQQDRDPRLSSSSSTRTLFEKDLEKS
Sbjct  108  IITGLVLSALIGYLLYRRWNIRRNQSSESPPSTFAQQDRDPRLSSSSSTRTLFEKDLEKS  167

Query  192  DRMTPSTRREKTPITSSASSVTSLDENG  219
            DRMTPSTRREKTPITSSASSVTSLDENG
Sbjct  168  DRMTPSTRREKTPITSSASSVTSLDENG  195


>gb|KIL85072.1| hypothetical protein FAVG1_11500 [Fusarium avenaceum]
Length=293

 Score = 44.7 bits (104),  Expect = 0.037, Method: Compositional matrix adjust.
 Identities = 16/40 (40%), Positives = 28/40 (70%), Gaps = 0/40 (0%)

Query  117  SGLSRSVLIAVIISTIITGLVLSALIGYLLYRRWNIRRNQ  156
            SGLS + L+ + +   + GL++S  +G+ L+RR++ RRNQ
Sbjct  191  SGLSTAALVGISVGVTVAGLLISGAVGFFLWRRFDRRRNQ  230


>gb|EXL70475.1| hypothetical protein FOPG_13728 [Fusarium oxysporum f. sp. conglutinans 
race 2 54008]
Length=261

 Score = 43.5 bits (101),  Expect = 0.071, Method: Compositional matrix adjust.
 Identities = 18/56 (32%), Positives = 34/56 (61%), Gaps = 1/56 (2%)

Query  114  TTSSGLSRSVLIAVIISTIITGLVLSALIGYLLYRRWNIRRNQSSESP-PSTFAQQ  168
            ++ SGLSRS +  V +   I GL++   +G++++R+W  R+N ++ +  P  F Q 
Sbjct  173  SSKSGLSRSEIAGVAVGATIGGLLILGCVGWIVWRKWARRKNDAALAELPGNFNQD  228


>gb|EGU75523.1| hypothetical protein FOXB_13972 [Fusarium oxysporum Fo5176]
Length=260

 Score = 43.5 bits (101),  Expect = 0.072, Method: Compositional matrix adjust.
 Identities = 18/56 (32%), Positives = 34/56 (61%), Gaps = 1/56 (2%)

Query  114  TTSSGLSRSVLIAVIISTIITGLVLSALIGYLLYRRWNIRRNQSSESP-PSTFAQQ  168
            ++ SGLSRS +  V +   I GL++   +G++++R+W  R+N ++ +  P  F Q 
Sbjct  172  SSKSGLSRSEIAGVAVGATIGGLLILGCVGWIVWRKWARRKNDAALAELPGNFNQD  227


>ref|XP_003193725.1| hypothetical protein CGB_D6430W [Cryptococcus gattii WM276]
 gb|ADV21938.1| hypothetical protein CND03720 [Cryptococcus gattii WM276]
 gb|KIY35728.1| hypothetical protein I305_01979 [Cryptococcus gattii E566]
 gb|KJE03397.1| hypothetical protein I311_02959 [Cryptococcus gattii NT-10]
Length=462

 Score = 43.5 bits (101),  Expect = 0.10, Method: Compositional matrix adjust.
 Identities = 34/115 (30%), Positives = 57/115 (50%), Gaps = 20/115 (17%)

Query  98   VLVASSSHTHASIKNGTT--------SSGLSRSVLIAVIISTIITGLVLSALIGYLLYRR  149
            V  AS S TH+S  + T+        SSGLS+  LIA+I+   + GL   A IG+  +R+
Sbjct  203  VTTASRSATHSSTASATSEADNASSKSSGLSKPALIAIIVVASVVGL---AAIGWTAFRK  259

Query  150  WNIRRNQSSESP--PSTFAQQDRDPRLSSSSSTRTLFEKDLEKSDRMTPSTRREK  202
            W +R +   +S   P  F+ Q+        +     FEK L+++   + + R+ +
Sbjct  260  WKLRPSNRFDSKMMPIDFSPQN-------GNMDDDFFEKTLQRTTSQSSANRQRQ  307


>gb|KIR80870.1| hypothetical protein I306_02327 [Cryptococcus gattii EJB2]
Length=462

 Score = 43.5 bits (101),  Expect = 0.10, Method: Compositional matrix adjust.
 Identities = 34/115 (30%), Positives = 57/115 (50%), Gaps = 20/115 (17%)

Query  98   VLVASSSHTHASIKNGTT--------SSGLSRSVLIAVIISTIITGLVLSALIGYLLYRR  149
            V  AS S TH+S  + T+        SSGLS+  LIA+I+   + GL   A IG+  +R+
Sbjct  203  VTTASRSATHSSTASATSEADNASSKSSGLSKPALIAIIVVASVVGL---AAIGWTAFRK  259

Query  150  WNIRRNQSSESP--PSTFAQQDRDPRLSSSSSTRTLFEKDLEKSDRMTPSTRREK  202
            W +R +   +S   P  F+ Q+        +     FEK L+++   + + R+ +
Sbjct  260  WKLRPSNRFDSKMMPIDFSPQN-------GNMDDDFFEKTLQRTTSQSSANRQRQ  307


>gb|KIR57121.1| hypothetical protein I315_00284 [Cryptococcus gattii Ru294]
Length=464

 Score = 43.5 bits (101),  Expect = 0.11, Method: Compositional matrix adjust.
 Identities = 34/115 (30%), Positives = 57/115 (50%), Gaps = 20/115 (17%)

Query  98   VLVASSSHTHASIKNGTT--------SSGLSRSVLIAVIISTIITGLVLSALIGYLLYRR  149
            V  AS S TH+S  + T+        SSGLS+  LIA+I+   + GL   A IG+  +R+
Sbjct  205  VTTASRSATHSSTASATSEADNASSKSSGLSKPALIAIIVVASVVGL---AAIGWTAFRK  261

Query  150  WNIRRNQSSESP--PSTFAQQDRDPRLSSSSSTRTLFEKDLEKSDRMTPSTRREK  202
            W +R +   +S   P  F+ Q+        +     FEK L+++   + + R+ +
Sbjct  262  WKLRPSNRFDSKMMPIDFSPQN-------GNMDDDFFEKTLQRTTSQSSANRQRQ  309


>gb|EXM17501.1| hypothetical protein FOTG_14340 [Fusarium oxysporum f. sp. vasinfectum 
25433]
Length=222

 Score = 42.7 bits (99),  Expect = 0.11, Method: Compositional matrix adjust.
 Identities = 15/45 (33%), Positives = 30/45 (67%), Gaps = 0/45 (0%)

Query  114  TTSSGLSRSVLIAVIISTIITGLVLSALIGYLLYRRWNIRRNQSS  158
            ++ SGLSRS +  V +   I GL++   +G++++R+W  R+N ++
Sbjct  134  SSKSGLSRSEIAGVAVGATIGGLLILGCVGWIVWRKWARRKNDAA  178


>gb|EWY99821.1| hypothetical protein FOYG_03759 [Fusarium oxysporum FOSC 3-a]
Length=244

 Score = 42.0 bits (97),  Expect = 0.23, Method: Compositional matrix adjust.
 Identities = 14/45 (31%), Positives = 29/45 (64%), Gaps = 0/45 (0%)

Query  114  TTSSGLSRSVLIAVIISTIITGLVLSALIGYLLYRRWNIRRNQSS  158
            ++ SGLSR  +  V +   I GL++   +G++++R+W  R+N ++
Sbjct  156  SSKSGLSRGEIAGVAVGATIGGLLILGCVGWIVWRKWARRKNDAA  200


>ref|XP_013132474.1| PREDICTED: uncharacterized protein LOC100703105 [Oreochromis 
niloticus]
Length=332

 Score = 41.2 bits (95),  Expect = 0.50, Method: Compositional matrix adjust.
 Identities = 14/35 (40%), Positives = 26/35 (74%), Gaps = 0/35 (0%)

Query  120  SRSVLIAVIISTIITGLVLSALIGYLLYRRWNIRR  154
            S   LIAV++  I+  ++++A+IG+++YRRW  +R
Sbjct  252  SNDALIAVVVCVILGMIIIAAVIGFVVYRRWKTQR  286


>ref|XP_012049423.1| hypothetical protein CNAG_01255 [Cryptococcus neoformans var. 
grubii H99]
 gb|AFR94891.1| hypothetical protein CNAG_01255 [Cryptococcus neoformans var. 
grubii H99]
Length=461

 Score = 41.2 bits (95),  Expect = 0.60, Method: Compositional matrix adjust.
 Identities = 27/91 (30%), Positives = 47/91 (52%), Gaps = 12/91 (13%)

Query  114  TTSSGLSRSVLIAVIISTIITGLVLSALIGYLLYRRWNIRRNQSSESP--PSTFAQQDRD  171
            T SSGLS+  LIA+I+   + GL   A IG+  +R+W +R +   +S   P  F+ Q+  
Sbjct  226  TGSSGLSKPALIAIIVVASVVGL---AAIGWTAFRKWKLRPSNRFDSKMMPIDFSPQN--  280

Query  172  PRLSSSSSTRTLFEKDLEKSDRMTPSTRREK  202
                  +     FEK L+++   + + R+ +
Sbjct  281  -----DTMDDDFFEKTLQRTTSQSSANRQRQ  306


>gb|EMT63128.1| hypothetical protein FOC4_g10013336 [Fusarium oxysporum f. sp. 
cubense race 4]
 gb|EXL98612.1| hypothetical protein FOIG_09368 [Fusarium oxysporum f. sp. cubense 
tropical race 4 54006]
Length=273

 Score = 40.0 bits (92),  Expect = 1.1, Method: Compositional matrix adjust.
 Identities = 19/60 (32%), Positives = 31/60 (52%), Gaps = 0/60 (0%)

Query  95   SSSVLVASSSHTHASIKNGTTSSGLSRSVLIAVIISTIITGLVLSALIGYLLYRRWNIRR  154
            +S     SS  TH S  N     GLS + L+ + +   + GL++S  +G  L+RR+  +R
Sbjct  149  ASETSTGSSETTHHSATNSENDPGLSTAALVGISVGVTVAGLLISGGVGLCLWRRFTRKR  208


>gb|KOG02963.1| Uncharacterized protein ABJ98_1989 [Pseudomonas syringae pv. 
aceris]
Length=600

 Score = 39.3 bits (90),  Expect = 2.5, Method: Compositional matrix adjust.
 Identities = 36/98 (37%), Positives = 44/98 (45%), Gaps = 13/98 (13%)

Query  55   TYPSPLKLFDSSVG-WNEATLVDRA-PFRPFPFSTIYPMPRASSSVLVASSSHTHASIKN  112
             YP   KL DS+ G WN   LV    P +P+PFS      RA S +       +H     
Sbjct  351  VYP---KLLDSATGAWNPRKLVQFGEPLQPWPFSHFKTRVRAKSEIDALQKIRSHGDYWK  407

Query  113  GTTSSGLSR--SVLIAVIIST-----IITGLVLSALIG  143
               SSGLS   SVL  V I       ++ GL L AL+G
Sbjct  408  EEASSGLSECVSVLGGVAIVFPELMPVVAGLSL-ALVG  444


>ref|XP_007135679.1| hypothetical protein PHAVU_010G149300g [Phaseolus vulgaris]
 gb|ESW07673.1| hypothetical protein PHAVU_010G149300g [Phaseolus vulgaris]
Length=566

 Score = 39.3 bits (90),  Expect = 3.0, Method: Compositional matrix adjust.
 Identities = 19/51 (37%), Positives = 30/51 (59%), Gaps = 2/51 (4%)

Query  115  TSSGLSRSVLIAVIISTII--TGLVLSALIGYLLYRRWNIRRNQSSESPPS  163
            T SGLS   L  +II  ++   G+++   I + +YR W  +R+Q+S S PS
Sbjct  57   TGSGLSPGTLSGIIIGAVLGAVGMLIVGGIFFCVYRNWKKKRSQNSYSQPS  107


>ref|XP_002845802.1| HMG-CoA reductase [Arthroderma otae CBS 113480]
 gb|EEQ32852.1| HMG-CoA reductase [Arthroderma otae CBS 113480]
Length=1138

 Score = 38.9 bits (89),  Expect = 3.9, Method: Compositional matrix adjust.
 Identities = 34/121 (28%), Positives = 56/121 (46%), Gaps = 7/121 (6%)

Query  89   YPMPRASSSVLVASSSHTHASIKNGTTSSGLSRSVLIAVIISTIITGLVLSALI-GYLL-  146
            Y  P A+   L+ +  +    +  G     + RS+   +I   II  L+LS ++ GYL  
Sbjct  555  YVNPNANVDRLLDTDYNPVLDVVGGRVLESILRSLDDPIISKWIIAALILSLILNGYLFN  614

Query  147  YRRWNIRRNQSSE-----SPPSTFAQQDRDPRLSSSSSTRTLFEKDLEKSDRMTPSTRRE  201
              RW+I+ +QSS      +P  T  + +R P +    + RTL E +    D+M  S   E
Sbjct  615  AARWSIKEDQSSTPSETLTPAPTPVKIERKPIIKEDGTPRTLEECEQMLKDKMVTSLDDE  674

Query  202  K  202
            +
Sbjct  675  E  675


>gb|EXK88781.1| hypothetical protein FOQG_08071 [Fusarium oxysporum f. sp. raphani 
54005]
Length=273

 Score = 38.5 bits (88),  Expect = 4.0, Method: Compositional matrix adjust.
 Identities = 19/62 (31%), Positives = 35/62 (56%), Gaps = 1/62 (2%)

Query  93   RASSSVLVASSSHTHASIKNGTTSSGLSRSVLIAVIISTIITGLVLSALIGYLLYRRWNI  152
            +AS + +V+S +  H S+       GLS + L+ + +   + GL++S  IG  L+RR+  
Sbjct  148  QASETSMVSSETTNH-SVTKSENDPGLSTAALVGISVGVTVAGLLISGGIGLCLWRRFKR  206

Query  153  RR  154
            +R
Sbjct  207  KR  208


>gb|KIO28875.1| hypothetical protein M407DRAFT_21952 [Tulasnella calospora MUT 
4182]
Length=242

 Score = 38.1 bits (87),  Expect = 4.1, Method: Compositional matrix adjust.
 Identities = 30/85 (35%), Positives = 45/85 (53%), Gaps = 6/85 (7%)

Query  84   PFSTIYPMPRASSSVLVASSSH--THASIKNGTTSSGLSRSVLIAVIISTIITGLVLSAL  141
            P ST Y  P AS S  + +S    + A+ + G+ S+G   +     I   +I G+ L AL
Sbjct  45   PESTYYGTPTASISYRINTSGDVTSAAATETGSPSTGGKSNA--GPIAGGVIGGIALLAL  102

Query  142  IGYLLYRRWNIRRNQSSESPPSTFA  166
            IG L++RR  +RR  + + PPS  A
Sbjct  103  IGGLVWRR--MRRRDNEDVPPSPTA  125


>gb|EXL56165.1| hypothetical protein FOCG_03850 [Fusarium oxysporum f. sp. radicis-lycopersici 
26381]
Length=273

 Score = 38.1 bits (87),  Expect = 4.3, Method: Compositional matrix adjust.
 Identities = 21/71 (30%), Positives = 34/71 (48%), Gaps = 4/71 (6%)

Query  84   PFSTIYPMPRASSSVLVASSSHTHASIKNGTTSSGLSRSVLIAVIISTIITGLVLSALIG  143
            P ST+   P  S+     S   TH S        GLS + L+ + +   + GL++S  +G
Sbjct  142  PASTLSQAPETST----GSPETTHHSATKSENDPGLSTAALVGISVGVTVAGLLISGAVG  197

Query  144  YLLYRRWNIRR  154
              L+RR+  +R
Sbjct  198  LCLWRRFTRKR  208


>gb|EXA44284.1| hypothetical protein FOVG_05756 [Fusarium oxysporum f. sp. pisi 
HDV247]
Length=273

 Score = 38.1 bits (87),  Expect = 4.6, Method: Compositional matrix adjust.
 Identities = 19/62 (31%), Positives = 35/62 (56%), Gaps = 1/62 (2%)

Query  93   RASSSVLVASSSHTHASIKNGTTSSGLSRSVLIAVIISTIITGLVLSALIGYLLYRRWNI  152
            +AS + +V+S +  H S+       GLS + L+ + +   + GL++S  IG  L+RR+  
Sbjct  148  QASETSMVSSETTNH-SVTKSENDPGLSTATLVGISVGVTVAGLLISGGIGLCLWRRFKR  206

Query  153  RR  154
            +R
Sbjct  207  KR  208


>ref|XP_641794.1| hypothetical protein DDB_G0279325 [Dictyostelium discoideum AX4]
 gb|EAL67811.1| hypothetical protein DDB_G0279325 [Dictyostelium discoideum AX4]
Length=1436

 Score = 38.5 bits (88),  Expect = 5.0, Method: Compositional matrix adjust.
 Identities = 21/53 (40%), Positives = 36/53 (68%), Gaps = 1/53 (2%)

Query  97    SVLVASSSHTHASIKNGTTSSGLSRSVLIAVIISTIITGLVLSALIGYLLYRR  149
             SVL+ S+S T  SI N + +SGLS++ LI +II ++   +V++  + YL++R 
Sbjct  1367  SVLLESNSPTDNSICN-SNNSGLSKTQLIGIIIGSVFFAVVIAIGVTYLVFRN  1418


>gb|EWZ36703.1| hypothetical protein FOZG_10669 [Fusarium oxysporum Fo47]
Length=273

 Score = 37.7 bits (86),  Expect = 6.0, Method: Compositional matrix adjust.
 Identities = 21/72 (29%), Positives = 34/72 (47%), Gaps = 4/72 (6%)

Query  83   FPFSTIYPMPRASSSVLVASSSHTHASIKNGTTSSGLSRSVLIAVIISTIITGLVLSALI  142
             P ST+   P  S+     S   TH S        GLS + L+ + +   + GL++S  +
Sbjct  141  IPASTLSQAPETST----GSPETTHHSATKSENDPGLSTAALVGISVGVTVAGLLISGGV  196

Query  143  GYLLYRRWNIRR  154
            G  L+RR+  +R
Sbjct  197  GLCLWRRFTRKR  208


>gb|EXM19741.1| hypothetical protein FOTG_12370 [Fusarium oxysporum f. sp. vasinfectum 
25433]
Length=273

 Score = 37.7 bits (86),  Expect = 6.4, Method: Compositional matrix adjust.
 Identities = 18/62 (29%), Positives = 35/62 (56%), Gaps = 1/62 (2%)

Query  93   RASSSVLVASSSHTHASIKNGTTSSGLSRSVLIAVIISTIITGLVLSALIGYLLYRRWNI  152
            +AS + +V+S +  H S+       GLS + L+ + +   + GL++S  +G  L+RR+  
Sbjct  148  QASETSMVSSETTNH-SVTKSENDPGLSTAALVGISVGVTVAGLLISGGVGLCLWRRFTR  206

Query  153  RR  154
            +R
Sbjct  207  KR  208


>gb|EXK38733.1| hypothetical protein FOMG_06273 [Fusarium oxysporum f. sp. melonis 
26406]
 gb|KNB01802.1| hypothetical protein FOXG_04945 [Fusarium oxysporum f. sp. lycopersici 
4287]
Length=273

 Score = 37.7 bits (86),  Expect = 6.7, Method: Compositional matrix adjust.
 Identities = 21/72 (29%), Positives = 34/72 (47%), Gaps = 4/72 (6%)

Query  83   FPFSTIYPMPRASSSVLVASSSHTHASIKNGTTSSGLSRSVLIAVIISTIITGLVLSALI  142
             P ST+   P  S+     S   TH S        GLS + L+ + +   + GL++S  +
Sbjct  141  IPASTLSQAPETST----GSPETTHHSATKSENDPGLSTAALVGISVGVTVAGLLISGGV  196

Query  143  GYLLYRRWNIRR  154
            G  L+RR+  +R
Sbjct  197  GLCLWRRFTRKR  208


>gb|EWY90105.1| hypothetical protein FOYG_07720 [Fusarium oxysporum FOSC 3-a]
Length=273

 Score = 37.7 bits (86),  Expect = 7.0, Method: Compositional matrix adjust.
 Identities = 21/72 (29%), Positives = 34/72 (47%), Gaps = 4/72 (6%)

Query  83   FPFSTIYPMPRASSSVLVASSSHTHASIKNGTTSSGLSRSVLIAVIISTIITGLVLSALI  142
             P ST+   P  S+     S   TH S        GLS + L+ + +   + GL++S  +
Sbjct  141  IPASTLSQAPETST----GSPETTHHSATKSGNDPGLSTAALVGISVGVTVAGLLISGGV  196

Query  143  GYLLYRRWNIRR  154
            G  L+RR+  +R
Sbjct  197  GLCLWRRFTRKR  208


>gb|EMD39191.1| hypothetical protein CERSUDRAFT_112868 [Ceriporiopsis subvermispora 
B]
Length=399

 Score = 37.4 bits (85),  Expect = 9.0, Method: Compositional matrix adjust.
 Identities = 23/83 (28%), Positives = 38/83 (46%), Gaps = 5/83 (6%)

Query  121  RSVLIAVIISTIITGLVLSALIGYLLYRRWNIRRNQSSESPPSTFAQQDRDPRLSSSSST  180
            R  L   I+ST ++  ++   +G  +YR W  R  Q    PP  + Q +  P       +
Sbjct  129  RRTLAGSIVSTALSAALIGTAVGLTVYRLWRDRSKQPESLPPPPYEQGEWVP-----PKS  183

Query  181  RTLFEKDLEKSDRMTPSTRREKT  203
            + L E +     R+TPS+ R+K 
Sbjct  184  QPLPEPEPITVSRVTPSSPRKKA  206


>ref|XP_002604710.1| hypothetical protein BRAFLDRAFT_80319 [Branchiostoma floridae]
 gb|EEN60721.1| hypothetical protein BRAFLDRAFT_80319 [Branchiostoma floridae]
Length=1805

 Score = 37.7 bits (86),  Expect = 9.0, Method: Composition-based stats.
 Identities = 22/79 (28%), Positives = 41/79 (52%), Gaps = 2/79 (3%)

Query  86    STIYPMPRASSSVLVASSSHTHASI-KNGTTSSGLSRSVLIAVIISTIITGLVLSALIGY  144
             ST+   PR  +      S  T AS  K+ +    L    ++A+I+S ++  L+++A + +
Sbjct  1696  STVAKPPRTENVATDGPSHMTPASQEKDPSAQHDLETGAIVAIILSCVLV-LLIAAAVPF  1754

Query  145   LLYRRWNIRRNQSSESPPS  163
              +YRRW+  R   ++ P S
Sbjct  1755  FIYRRWSSTRGSRTDVPHS  1773


>gb|KKR33274.1| Alpha-amylase [Parcubacteria (Falkowbacteria) bacterium GW2011_GWF2_39_8]
Length=855

 Score = 37.7 bits (86),  Expect = 9.4, Method: Compositional matrix adjust.
 Identities = 26/84 (31%), Positives = 48/84 (57%), Gaps = 6/84 (7%)

Query  101  ASSSHTHASIKNGTTSSGLSRSVLIAVIISTIITGLVLSA---LIGYLLYRRWNIRRNQS  157
            A+S+   A+  NGT++SGL++ ++ A+ +S     L  S+    +G + YR   I RN S
Sbjct  278  ATSTEVTATTLNGTSTSGLTKPIVTAIPVSATQINLTWSSSTQGVGDISYR---IYRNGS  334

Query  158  SESPPSTFAQQDRDPRLSSSSSTR  181
            + +  +TF  +  D  L++S++ R
Sbjct  335  TSTTATTFGNEFNDVGLATSTTYR  358


>gb|ENH67497.1| hypothetical protein FOC1_g10010774 [Fusarium oxysporum f. sp. 
cubense race 1]
Length=273

 Score = 37.4 bits (85),  Expect = 9.6, Method: Compositional matrix adjust.
 Identities = 19/62 (31%), Positives = 36/62 (58%), Gaps = 1/62 (2%)

Query  93   RASSSVLVASSSHTHASIKNGTTSSGLSRSVLIAVIISTIITGLVLSALIGYLLYRRWNI  152
            +AS + +V+S + T+ S+       GLS + L+ + +   + GL++S  IG  L+RR+  
Sbjct  148  QASETSMVSSET-TNNSVTKSENDPGLSTAALVGISVGVTVAGLLISGGIGLCLWRRFTR  206

Query  153  RR  154
            +R
Sbjct  207  KR  208


Lambda      K        H        a         alpha
   0.319    0.131    0.377    0.792     4.96 

Gapped
Lambda      K        H        a         alpha    sigma
   0.267   0.0410    0.140     1.90     42.6     43.6 

Effective search space used: 1175905893600


  Database: nr
    Posted date:  Sep 23, 2015 12:05 AM
  Number of letters in database: 26,053,659,533
  Number of sequences in database:  71,551,133


Matrix: BLOSUM62
Gap Penalties: Existence: 11, Extension: 1
Neighboring words threshold: 11
Window for multiple hits: 40
```
