# Supplementary material for: Comparative genomics provides new insights into the diversity, physiology, and sexuality of the only industrially exploited tremellomycete: Phaffia rhodozyma
Source: BMC Genomics. 2016 Nov 9;17:901. doi: 10.1186/s12864-016-3244-7 (PMC5103461; doi:10.1186/s12864-016-3244-7)
Supplement: Additional file 6: — List of orphan genes with links to PFAM (related to Additional file 1: Table S1). (ZIP 1428 kb) [file 12864_2016_3244_MOESM6_ESM.zip › BLAST_HTML_FTR/G05580_P.html]

BLAST Search Results


```
BLASTP 2.2.27+


Reference:
Stephen F. Altschul, Thomas L. Madden, Alejandro A. Schäffer,
Jinghui Zhang, Zheng Zhang, Webb Miller, and David J. Lipman (1997),
"Gapped BLAST and PSI-BLAST: a new generation of protein database
search programs", Nucleic Acids Res. 25:3389-3402.


Reference for
composition-based statistics:
Alejandro A. Schäffer, L. Aravind, Thomas L. Madden, Sergei
Shavirin, John L. Spouge, Yuri I. Wolf, Eugene V. Koonin, and
Stephen F. Altschul (2001), "Improving the accuracy of PSI-BLAST
protein database searches with composition-based statistics and
other refinements", Nucleic Acids Res. 29:2994-3005.


Database: nr
           71,551,133 sequences; 26,053,659,533 total letters


Query= G05580_P

Length=136
                                                                      Score     E
Sequences producing significant alignments:                          (Bits)  Value

emb|CDZ96258.1|  hypothetical protein [Xanthophyllomyces dendrorh...   280    4e-93
emb|CDZ96163.1|  von Willebrand factor, type A [Xanthophyllomyces...  39.7    0.50 
ref|WP_045101083.1|  protein HflC [Aliivibrio wodanis] >emb|CED70...  38.5    0.96 
ref|XP_011480960.1|  PREDICTED: THAP domain-containing protein 1 ...  37.0    3.4  
ref|WP_004398346.1|  hypothetical protein [Vibrio nigripulchritud...  35.4    9.8  


 >emb|CDZ96258.1| hypothetical protein [Xanthophyllomyces dendrorhous]
Length=194

 Score =  280 bits (715),  Expect = 4e-93, Method: Compositional matrix adjust.
 Identities = 135/135 (100%), Positives = 135/135 (100%), Gaps = 0/135 (0%)

Query  1    MYKVVASPNLDQRMKSHPPLKIKTRTQLVSLITSFGAFQDKTLIQRALDDYCRGKLGNKS  60
            MYKVVASPNLDQRMKSHPPLKIKTRTQLVSLITSFGAFQDKTLIQRALDDYCRGKLGNKS
Sbjct  60   MYKVVASPNLDQRMKSHPPLKIKTRTQLVSLITSFGAFQDKTLIQRALDDYCRGKLGNKS  119

Query  61   SKERLEQKLHMVEATMYDYQDLGLPDYSSEFIHLRKTLGLQSSKIFTQARSPMSFPEKKE  120
            SKERLEQKLHMVEATMYDYQDLGLPDYSSEFIHLRKTLGLQSSKIFTQARSPMSFPEKKE
Sbjct  120  SKERLEQKLHMVEATMYDYQDLGLPDYSSEFIHLRKTLGLQSSKIFTQARSPMSFPEKKE  179

Query  121  NEHDPFETLSTLGKY  135
            NEHDPFETLSTLGKY
Sbjct  180  NEHDPFETLSTLGKY  194


>emb|CDZ96163.1| von Willebrand factor, type A [Xanthophyllomyces dendrorhous]
Length=440

 Score = 39.7 bits (91),  Expect = 0.50, Method: Composition-based stats.
 Identities = 23/82 (28%), Positives = 42/82 (51%), Gaps = 1/82 (1%)

Query  31   LITSFGAFQDKTLIQRALDDYCRGKLGNKSSKERLEQKLHMVEATMYDYQDLGLPDYSSE  90
            + T+  A  D T     +  YC  K+    S+     KLH++E  +  ++   +PD  ++
Sbjct  1    MTTTARAASDPTYAHDIILSYCSRKIVRGRSRYETGYKLHLLEWELV-FRKYPIPDLGNQ  59

Query  91   FIHLRKTLGLQSSKIFTQARSP  112
             + +R+TLGL  S IF ++ +P
Sbjct  60   VLEVRETLGLPESHIFRRSSNP  81


>ref|WP_045101083.1| protein HflC [Aliivibrio wodanis]
 emb|CED70374.1| HflC protein [Aliivibrio wodanis]
Length=294

 Score = 38.5 bits (88),  Expect = 0.96, Method: Compositional matrix adjust.
 Identities = 30/94 (32%), Positives = 44/94 (47%), Gaps = 2/94 (2%)

Query  3    KVVASPNLDQRMKSHPPLKIKTRTQLVSLITSFGAFQDKTLIQRALDDYCRGKLGNKSSK  62
            + V S   D  + S+   KIK   Q             ++L+QR + D  R ++GNK+ K
Sbjct  78   RFVTSEKKDVIIDSYVKWKIKDFGQFYLATGGGNILTAESLLQRRVSDGLRAEIGNKTVK  137

Query  63   ERLEQKLHMVEAT-MYDYQDLGLPDYSSEFIHLR  95
            E + +K   V AT + D Q+ G  D   E I LR
Sbjct  138  EIVSEKREQVMATVLLDSQE-GTGDLGIEVIDLR  170


>ref|XP_011480960.1| PREDICTED: THAP domain-containing protein 1 A-like [Oryzias latipes]
Length=351

 Score = 37.0 bits (84),  Expect = 3.4, Method: Compositional matrix adjust.
 Identities = 24/56 (43%), Positives = 32/56 (57%), Gaps = 1/56 (2%)

Query  62   KERLEQKLHMVEATMYD-YQDLGLPDYSSEFIHLRKTLGLQSSKIFTQARSPMSFP  116
            KERL+QKL  VEA  YD +Q  G   Y+ E  +   TL +QS K +   R+ +S P
Sbjct  261  KERLQQKLDTVEAFPYDLFQLAGSSGYTEEQKNFALTLHMQSPKAYKFLRTKISLP  316


>ref|WP_004398346.1| hypothetical protein [Vibrio nigripulchritudo]
 gb|EGU61525.1| Cyanase [Vibrio nigripulchritudo ATCC 27043]
Length=265

 Score = 35.4 bits (80),  Expect = 9.8, Method: Compositional matrix adjust.
 Identities = 24/97 (25%), Positives = 48/97 (49%), Gaps = 4/97 (4%)

Query  29   VSLITSFGAFQDKTLIQRALDDY-CRGKLGNKSSKERLEQKLHMVEATMYDYQDLGLPDY  87
            + ++  FG  +   L+Q  +D   C+GK+G   +++  E  + + EA  +  + LG P+ 
Sbjct  29   MGMVAKFGDTESLELLQNTIDYLTCKGKIGQSFNEDEKEFMVELFEAMSWGGKYLGYPEA  88

Query  88   SSEFIHLRKTLGLQ---SSKIFTQARSPMSFPEKKEN  121
            S+   H     G +    S+++T++R  M   E  +N
Sbjct  89   STLANHYVNGNGHKISLPSEVYTKSRVVMDVCEGMKN  125


Lambda      K        H        a         alpha
   0.321    0.135    0.387    0.792     4.96 

Gapped
Lambda      K        H        a         alpha    sigma
   0.267   0.0410    0.140     1.90     42.6     43.6 

Effective search space used: 637685094878


  Database: nr
    Posted date:  Sep 23, 2015 12:05 AM
  Number of letters in database: 26,053,659,533
  Number of sequences in database:  71,551,133


Matrix: BLOSUM62
Gap Penalties: Existence: 11, Extension: 1
Neighboring words threshold: 11
Window for multiple hits: 40
```
