# Supplementary material for: Comparative genomics provides new insights into the diversity, physiology, and sexuality of the only industrially exploited tremellomycete: Phaffia rhodozyma
Source: BMC Genomics. 2016 Nov 9;17:901. doi: 10.1186/s12864-016-3244-7 (PMC5103461; doi:10.1186/s12864-016-3244-7)
Supplement: Additional file 6: — List of orphan genes with links to PFAM (related to Additional file 1: Table S1). (ZIP 1428 kb) [file 12864_2016_3244_MOESM6_ESM.zip › BLAST_HTML_FTR/G05668_P.html]

BLAST Search Results


```
BLASTP 2.2.27+


Reference:
Stephen F. Altschul, Thomas L. Madden, Alejandro A. Schäffer,
Jinghui Zhang, Zheng Zhang, Webb Miller, and David J. Lipman (1997),
"Gapped BLAST and PSI-BLAST: a new generation of protein database
search programs", Nucleic Acids Res. 25:3389-3402.


Reference for
composition-based statistics:
Alejandro A. Schäffer, L. Aravind, Thomas L. Madden, Sergei
Shavirin, John L. Spouge, Yuri I. Wolf, Eugene V. Koonin, and
Stephen F. Altschul (2001), "Improving the accuracy of PSI-BLAST
protein database searches with composition-based statistics and
other refinements", Nucleic Acids Res. 29:2994-3005.


Database: nr
           71,551,133 sequences; 26,053,659,533 total letters


Query= G05668_P

Length=157
                                                                      Score     E
Sequences producing significant alignments:                          (Bits)  Value

emb|CED83024.1|  hypothetical protein [Xanthophyllomyces dendrorh...   326    3e-111
ref|WP_023767493.1|  glycosyl transferase [Mesorhizobium sp. LNHC...  44.7    0.015 
ref|WP_023775857.1|  glycosyl transferase [Mesorhizobium sp. LNHC...  41.6    0.14  
ref|WP_013891503.1|  glycosyl transferase [Mesorhizobium opportun...  40.8    0.25  
ref|WP_027040498.1|  glycosyl transferase [Mesorhizobium sp. URHC...  38.9    1.3   
gb|EXX54156.1|  hypothetical protein RirG_237250 [Rhizophagus irr...  36.2    2.7   
ref|XP_011012926.1|  PREDICTED: probable serine/threonine-protein...  38.1    3.1   
ref|XP_002310436.2|  hypothetical protein POPTR_0007s01990g [Popu...  37.7    3.1   
ref|XP_011026415.1|  PREDICTED: probable serine/threonine-protein...  37.7    3.3   
ref|WP_006325284.1|  glycosyltransferase sugar-binding region con...  37.7    3.4   
ref|WP_019862818.1|  glycosyl transferase [Mesorhizobium loti]        36.6    7.7   
ref|WP_038651693.1|  glycosyl transferase [Mesorhizobium huakuii]...  36.6    8.3   


 >emb|CED83024.1| hypothetical protein [Xanthophyllomyces dendrorhous]
Length=196

 Score =  326 bits (836),  Expect = 3e-111, Method: Compositional matrix adjust.
 Identities = 156/156 (100%), Positives = 156/156 (100%), Gaps = 0/156 (0%)

Query  1    MFNKAVDQGDMSDAAWELKRIDDKNEKAARQEVAVTLFEKDELSPEEIVKHLKSERKLSI  60
            MFNKAVDQGDMSDAAWELKRIDDKNEKAARQEVAVTLFEKDELSPEEIVKHLKSERKLSI
Sbjct  41   MFNKAVDQGDMSDAAWELKRIDDKNEKAARQEVAVTLFEKDELSPEEIVKHLKSERKLSI  100

Query  61   ATVRKYLIQHAMVNEDFYHLLEDPDVWERFSGAKGAYQSTPNIKVAISLHKRFEQMDIDR  120
            ATVRKYLIQHAMVNEDFYHLLEDPDVWERFSGAKGAYQSTPNIKVAISLHKRFEQMDIDR
Sbjct  101  ATVRKYLIQHAMVNEDFYHLLEDPDVWERFSGAKGAYQSTPNIKVAISLHKRFEQMDIDR  160

Query  121  EDHLVRRYSNKGGGIRPYHAGPKKYKYPRNGYKRRT  156
            EDHLVRRYSNKGGGIRPYHAGPKKYKYPRNGYKRRT
Sbjct  161  EDHLVRRYSNKGGGIRPYHAGPKKYKYPRNGYKRRT  196


>ref|WP_023767493.1| glycosyl transferase [Mesorhizobium sp. LNHC232B00]
 gb|ESY65027.1| glycosyl transferase [Mesorhizobium sp. LNHC232B00]
Length=524

 Score = 44.7 bits (104),  Expect = 0.015, Method: Composition-based stats.
 Identities = 44/151 (29%), Positives = 65/151 (43%), Gaps = 16/151 (11%)

Query  8    QGDMSDAAWELKRIDDKNEKAARQEVAVTLFEKDELSPEEIVKHLKSE----RKLSIATV  63
            +GD  D +WE  R+       A +   + L ++   +  +  K  K      R+ +IA V
Sbjct  305  EGDSLDGSWE--RLQAATAGLAGKYRDIVLLQRRVGTSLDRAKRAKPRMQRVRRGAIAKV  362

Query  64   RKYLIQHAMVNEDFYHLLEDPDVWERFSG-------AKGAYQSTPN-IKVAISLHKRFEQ  115
            R YLI H +  +D + L  D DVW RF G       A G   + PN +KVA         
Sbjct  363  RNYLIDHGLSKDDDWALWIDIDVW-RFPGDALNRLIATGHRIAVPNCVKVAGGGSFDLNS  421

Query  116  MDIDREDHLVRRYSNKGGGI-RPYHAGPKKY  145
              I R+    R Y +  GG+ +P    P +Y
Sbjct  422  FIIRRQTRDYRYYRDIRGGLHQPPAQTPNRY  452


>ref|WP_023775857.1| glycosyl transferase [Mesorhizobium sp. LNHC221B00]
 gb|ESY83599.1| glycosyl transferase [Mesorhizobium sp. LNHC221B00]
Length=524

 Score = 41.6 bits (96),  Expect = 0.14, Method: Composition-based stats.
 Identities = 41/151 (27%), Positives = 65/151 (43%), Gaps = 16/151 (11%)

Query  8    QGDMSDAAWELKRIDDKNEKAARQEVAVTLFEKDELSPEEIVKHLKSE----RKLSIATV  63
            +GD  D +WE  R+       A +   + L ++   +  +  K  K      R+ +IA V
Sbjct  305  EGDSLDGSWE--RLQAATAGLAGKYRDIVLLQRRVGTSLDRAKRAKPRMQRVRRGAIAKV  362

Query  64   RKYLIQHAMVNEDFYHLLEDPDVWERFSG-------AKGAYQSTPN-IKVAISLHKRFEQ  115
            R YLI H +  +D + +  D DVW RF G       A G   + PN +K+A         
Sbjct  363  RNYLIDHGLGKDDDWAVWIDIDVW-RFPGDALNRLIATGHRIAVPNCVKIAGGGSFDLNS  421

Query  116  MDIDREDHLVRRYSNKGGGI-RPYHAGPKKY  145
              + R+    R Y +  GG+ +P    P +Y
Sbjct  422  FIVRRQTRDYRYYRDIRGGLHQPPAQTPNRY  452


>ref|WP_013891503.1| glycosyl transferase [Mesorhizobium opportunistum]
 gb|AEH84755.1| glycosyltransferase sugar-binding region containing DXD motif 
[Mesorhizobium opportunistum WSM2075]
Length=524

 Score = 40.8 bits (94),  Expect = 0.25, Method: Composition-based stats.
 Identities = 40/151 (26%), Positives = 65/151 (43%), Gaps = 16/151 (11%)

Query  8    QGDMSDAAWELKRIDDKNEKAARQEVAVTLFEKDELSPEEIVKH----LKSERKLSIATV  63
            +GD  D +WE  R+         +   + L ++   +  +  K     ++  R+ +IA V
Sbjct  305  EGDSLDGSWE--RLQAATAGLTGKYRDIVLLQRRVGTSLDRAKRARPRMQRVRRGAIAKV  362

Query  64   RKYLIQHAMVNEDFYHLLEDPDVWERFSG-------AKGAYQSTPN-IKVAISLHKRFEQ  115
            R YLI H +  +D + L  D DVW RF G       A G   + PN +K+A         
Sbjct  363  RNYLIDHGLSKDDDWALWIDIDVW-RFPGDALNRLIATGHRIAVPNCVKIAGGGSFDLNS  421

Query  116  MDIDREDHLVRRYSNKGGGI-RPYHAGPKKY  145
              + R+    R Y +  GG+ +P    P +Y
Sbjct  422  FIVRRQTRDYRYYRDIRGGLHQPPAQTPNRY  452


>ref|WP_027040498.1| glycosyl transferase [Mesorhizobium sp. URHC0008]
Length=524

 Score = 38.9 bits (89),  Expect = 1.3, Method: Composition-based stats.
 Identities = 41/151 (27%), Positives = 64/151 (42%), Gaps = 16/151 (11%)

Query  8    QGDMSDAAWELKRIDDKNEKAARQEVAVTLFEKDELSPEEIVKHLKSE----RKLSIATV  63
            +GD  D +WE  R+       A +   + L ++   +  +  K  K      R+ +IA V
Sbjct  305  EGDSLDGSWE--RLQAATTGLAGKYRDIVLLQRQVGTRLDRAKRAKPRMQRVRRGAIAKV  362

Query  64   RKYLIQHAMVNEDFYHLLEDPDVWERFSG-------AKGAYQSTPN-IKVAISLHKRFEQ  115
            R YLI H +  +D + L  D DVW RF         A G   + PN +K+A         
Sbjct  363  RNYLIDHGLKEDDDWALWIDIDVW-RFPTDALNRLIATGHRIAVPNCVKIAGGGSFDLNS  421

Query  116  MDIDREDHLVRRYSNKGGGI-RPYHAGPKKY  145
              + R+    R Y +  GG+ +P    P +Y
Sbjct  422  FVVRRQIKDYRYYRDIRGGLHQPPAQTPNRY  452


>gb|EXX54156.1| hypothetical protein RirG_237250 [Rhizophagus irregularis DAOM 
197198w]
Length=95

 Score = 36.2 bits (82),  Expect = 2.7, Method: Compositional matrix adjust.
 Identities = 19/52 (37%), Positives = 29/52 (56%), Gaps = 4/52 (8%)

Query  72   MVNEDFYHLLE-DPDVWERFSGAKGAYQSTPNIKVAISLHKRFEQMDIDRED  122
            M +ED   L E +PD+W R SG +   Q TP++K+      R E+++  R D
Sbjct  1    MQSEDISSLKEENPDLWLRLSGLR---QGTPSLKLRTKFRSRIEELEKSRSD  49


>ref|XP_011012926.1| PREDICTED: probable serine/threonine-protein kinase GCN2 [Populus 
euphratica]
 ref|XP_011012927.1| PREDICTED: probable serine/threonine-protein kinase GCN2 [Populus 
euphratica]
 ref|XP_011012928.1| PREDICTED: probable serine/threonine-protein kinase GCN2 [Populus 
euphratica]
Length=1256

 Score = 38.1 bits (87),  Expect = 3.1, Method: Compositional matrix adjust.
 Identities = 34/108 (31%), Positives = 51/108 (47%), Gaps = 14/108 (13%)

Query  6     VDQGDMSDAAWELKRIDDKNEKAARQEVAVTLFEKDELSPEEIVKHLKSERKLSIATVRK  65
             ++ GD+ DA W    I  ++    RQ+VA  L     L P+       SERKL  A +R+
Sbjct  945   LNHGDLLDAIWSWVGIKPEH----RQKVAELLSLMGSLRPQ------SSERKLKWAVIRR  994

Query  66    YLIQHAMVNEDFYHLLEDPDVWERFSGAKGAYQSTPNIKVAISLHKRF  113
              L+Q   + E   + L+   V  RF GA  A Q+ P ++ A+    R 
Sbjct  995   QLLQELNLAEAVVNRLQ--TVGLRFCGA--ADQALPRLRGALPADNRI  1038


>ref|XP_002310436.2| hypothetical protein POPTR_0007s01990g [Populus trichocarpa]
 gb|EEE90886.2| hypothetical protein POPTR_0007s01990g [Populus trichocarpa]
Length=1163

 Score = 37.7 bits (86),  Expect = 3.1, Method: Compositional matrix adjust.
 Identities = 34/108 (31%), Positives = 51/108 (47%), Gaps = 14/108 (13%)

Query  6     VDQGDMSDAAWELKRIDDKNEKAARQEVAVTLFEKDELSPEEIVKHLKSERKLSIATVRK  65
             ++ GD+ DA W    I  ++    RQ+VA  L     L P+       SERKL  A +R+
Sbjct  945   LNHGDLLDAIWSWVGIKPEH----RQKVAELLSLMGSLRPQ------SSERKLKWAVIRR  994

Query  66    YLIQHAMVNEDFYHLLEDPDVWERFSGAKGAYQSTPNIKVAISLHKRF  113
              L+Q   + E   + L+   V  RF GA  A Q+ P ++ A+    R 
Sbjct  995   QLLQELNLAEAVVNRLQ--TVGLRFCGA--ADQALPRLRGALPADNRI  1038


>ref|XP_011026415.1| PREDICTED: probable serine/threonine-protein kinase GCN2 [Populus 
euphratica]
 ref|XP_011026416.1| PREDICTED: probable serine/threonine-protein kinase GCN2 [Populus 
euphratica]
 ref|XP_011026417.1| PREDICTED: probable serine/threonine-protein kinase GCN2 [Populus 
euphratica]
Length=1256

 Score = 37.7 bits (86),  Expect = 3.3, Method: Compositional matrix adjust.
 Identities = 34/108 (31%), Positives = 51/108 (47%), Gaps = 14/108 (13%)

Query  6     VDQGDMSDAAWELKRIDDKNEKAARQEVAVTLFEKDELSPEEIVKHLKSERKLSIATVRK  65
             ++ GD+ DA W    I  ++    RQ+VA  L     L P+       SERKL  A +R+
Sbjct  945   LNHGDLLDAIWSWVGIKPEH----RQKVAELLSLMGSLRPQ------SSERKLKWAVIRR  994

Query  66    YLIQHAMVNEDFYHLLEDPDVWERFSGAKGAYQSTPNIKVAISLHKRF  113
              L+Q   + E   + L+   V  RF GA  A Q+ P ++ A+    R 
Sbjct  995   QLLQELNLAEAVVNRLQ--TVGLRFCGA--ADQALPRLRGALPADNRI  1038


>ref|WP_006325284.1| glycosyltransferase sugar-binding region containing DXD motif 
[Mesorhizobium sp. STM 4661]
 emb|CCV09792.1| Glycosyltransferase sugar-binding region containing DXD motif 
[Mesorhizobium sp. STM 4661]
Length=524

 Score = 37.7 bits (86),  Expect = 3.4, Method: Composition-based stats.
 Identities = 28/87 (32%), Positives = 42/87 (48%), Gaps = 7/87 (8%)

Query  8    QGDMSDAAWELKRIDDKNEKAARQEVAVTLFEKDELSPEEIVKHLK----SERKLSIATV  63
            +GD SD +WE  R+       A     + L +K   +  +  K  K     ER+ +IA +
Sbjct  305  EGDSSDGSWE--RLKAATAPLAASYRGIVLLQKHLGTSLDRDKRAKPRRQRERRGAIARI  362

Query  64   RKYLIQHAMVNEDFYHLLEDPDVWERF  90
            R +LI H +  +D + L  D DVW RF
Sbjct  363  RNHLIDHGLDADDAWALWIDIDVW-RF  388


>ref|WP_019862818.1| glycosyl transferase [Mesorhizobium loti]
Length=524

 Score = 36.6 bits (83),  Expect = 7.7, Method: Composition-based stats.
 Identities = 26/89 (29%), Positives = 43/89 (48%), Gaps = 7/89 (8%)

Query  8    QGDMSDAAWELKRIDDKNEKAARQEVAVTLFEKDELSPEEIVKH----LKSERKLSIATV  63
            +GD  D +WE  R+       A +   + L ++   +  +  K     ++  R+ +IA V
Sbjct  305  EGDSLDGSWE--RLQAATTGLAGKYRDIVLLQRQVGTRLDRAKRARPSMQRVRRGAIAKV  362

Query  64   RKYLIQHAMVNEDFYHLLEDPDVWERFSG  92
            R YLI H +  +D + L  D D+W RF G
Sbjct  363  RNYLIDHGLKEDDDWALWIDIDIW-RFPG  390


>ref|WP_038651693.1| glycosyl transferase [Mesorhizobium huakuii]
 gb|AID34074.1| anp1 family protein [Mesorhizobium huakuii 7653R]
Length=524

 Score = 36.6 bits (83),  Expect = 8.3, Method: Composition-based stats.
 Identities = 26/89 (29%), Positives = 43/89 (48%), Gaps = 7/89 (8%)

Query  8    QGDMSDAAWELKRIDDKNEKAARQEVAVTLFEKDELSPEEIVKH----LKSERKLSIATV  63
            +GD  D +WE  R+       A +   + L ++   +  +  K     ++  R+ +IA V
Sbjct  305  EGDSLDGSWE--RLQAATTGLAGRYRDIVLLQRQVGTRLDRAKRARPSMQRVRRGAIAKV  362

Query  64   RKYLIQHAMVNEDFYHLLEDPDVWERFSG  92
            R YLI H +  +D + L  D D+W RF G
Sbjct  363  RNYLIDHGLKEDDDWALWIDIDIW-RFPG  390


Lambda      K        H        a         alpha
   0.320    0.136    0.404    0.792     4.96 

Gapped
Lambda      K        H        a         alpha    sigma
   0.267   0.0410    0.140     1.90     42.6     43.6 

Effective search space used: 646298372201


  Database: nr
    Posted date:  Sep 23, 2015 12:05 AM
  Number of letters in database: 26,053,659,533
  Number of sequences in database:  71,551,133


Matrix: BLOSUM62
Gap Penalties: Existence: 11, Extension: 1
Neighboring words threshold: 11
Window for multiple hits: 40
```
