# Supplementary material for: Comparative genomics provides new insights into the diversity, physiology, and sexuality of the only industrially exploited tremellomycete: Phaffia rhodozyma
Source: BMC Genomics. 2016 Nov 9;17:901. doi: 10.1186/s12864-016-3244-7 (PMC5103461; doi:10.1186/s12864-016-3244-7)
Supplement: Additional file 6: — List of orphan genes with links to PFAM (related to Additional file 1: Table S1). (ZIP 1428 kb) [file 12864_2016_3244_MOESM6_ESM.zip › BLAST_HTML_FTR/G02713_P.html]

BLAST Search Results


```
BLASTP 2.2.27+


Reference:
Stephen F. Altschul, Thomas L. Madden, Alejandro A. Schäffer,
Jinghui Zhang, Zheng Zhang, Webb Miller, and David J. Lipman (1997),
"Gapped BLAST and PSI-BLAST: a new generation of protein database
search programs", Nucleic Acids Res. 25:3389-3402.


Reference for
composition-based statistics:
Alejandro A. Schäffer, L. Aravind, Thomas L. Madden, Sergei
Shavirin, John L. Spouge, Yuri I. Wolf, Eugene V. Koonin, and
Stephen F. Altschul (2001), "Improving the accuracy of PSI-BLAST
protein database searches with composition-based statistics and
other refinements", Nucleic Acids Res. 29:2994-3005.


Database: nr
           71,551,133 sequences; 26,053,659,533 total letters


Query= G02713_P

Length=974
                                                                      Score     E
Sequences producing significant alignments:                          (Bits)  Value

emb|CED84977.1|  hypothetical protein [Xanthophyllomyces dendrorh...  1942    0.0  


 >emb|CED84977.1| hypothetical protein [Xanthophyllomyces dendrorhous]
Length=973

 Score = 1942 bits (5030),  Expect = 0.0, Method: Compositional matrix adjust.
 Identities = 973/973 (100%), Positives = 973/973 (100%), Gaps = 0/973 (0%)

Query  1    MPFGFASSSNSSGTSQKSQVPSDQNAIPTPTITTTYSLHPTSQPVPSQGRINQAQATKGR  60
            MPFGFASSSNSSGTSQKSQVPSDQNAIPTPTITTTYSLHPTSQPVPSQGRINQAQATKGR
Sbjct  1    MPFGFASSSNSSGTSQKSQVPSDQNAIPTPTITTTYSLHPTSQPVPSQGRINQAQATKGR  60

Query  61   RSISSNSSLPRTIKSRISTGNSTSTGTGIGPDSIRERNSLRTNGSIGRTSRTKSINGGGG  120
            RSISSNSSLPRTIKSRISTGNSTSTGTGIGPDSIRERNSLRTNGSIGRTSRTKSINGGGG
Sbjct  61   RSISSNSSLPRTIKSRISTGNSTSTGTGIGPDSIRERNSLRTNGSIGRTSRTKSINGGGG  120

Query  121  SPRTNSTQTRSLTEIPLSSSTKNTTQTVGKPKNIERSIGSSPSTQTHLQSSEESKSNSGK  180
            SPRTNSTQTRSLTEIPLSSSTKNTTQTVGKPKNIERSIGSSPSTQTHLQSSEESKSNSGK
Sbjct  121  SPRTNSTQTRSLTEIPLSSSTKNTTQTVGKPKNIERSIGSSPSTQTHLQSSEESKSNSGK  180

Query  181  HAPQVADRQISESSGVAQRSNSFSKPNKNKTNLLWRLRSPSKVKDGASRTRDRDQFKTST  240
            HAPQVADRQISESSGVAQRSNSFSKPNKNKTNLLWRLRSPSKVKDGASRTRDRDQFKTST
Sbjct  181  HAPQVADRQISESSGVAQRSNSFSKPNKNKTNLLWRLRSPSKVKDGASRTRDRDQFKTST  240

Query  241  TPVLKSPSGSITSTSRTRISAKASLNSGLKGPNKVSPAVNPGQEPDSSPVSVLRSRSTVD  300
            TPVLKSPSGSITSTSRTRISAKASLNSGLKGPNKVSPAVNPGQEPDSSPVSVLRSRSTVD
Sbjct  241  TPVLKSPSGSITSTSRTRISAKASLNSGLKGPNKVSPAVNPGQEPDSSPVSVLRSRSTVD  300

Query  301  RFKPGGLTRDHKDGDGAECEGETKGMSRSWSTDLPTATRKIVSNGLVLPSVKHPIHIASP  360
            RFKPGGLTRDHKDGDGAECEGETKGMSRSWSTDLPTATRKIVSNGLVLPSVKHPIHIASP
Sbjct  301  RFKPGGLTRDHKDGDGAECEGETKGMSRSWSTDLPTATRKIVSNGLVLPSVKHPIHIASP  360

Query  361  SPSPLPLAPDQDFPVWAGNHESAANVGMTDFDSATSAPTVPVTTATTAILSASQSSSSSK  420
            SPSPLPLAPDQDFPVWAGNHESAANVGMTDFDSATSAPTVPVTTATTAILSASQSSSSSK
Sbjct  361  SPSPLPLAPDQDFPVWAGNHESAANVGMTDFDSATSAPTVPVTTATTAILSASQSSSSSK  420

Query  421  APRISATFGSRANGAGMPIPIFLNGIPRTNYSRPMHHRSLSYQSTASSVSSSSSAAALAA  480
            APRISATFGSRANGAGMPIPIFLNGIPRTNYSRPMHHRSLSYQSTASSVSSSSSAAALAA
Sbjct  421  APRISATFGSRANGAGMPIPIFLNGIPRTNYSRPMHHRSLSYQSTASSVSSSSSAAALAA  480

Query  481  SEAIPEFTTNNHSDSQNGSRRNSTLLPSMINPTVLARSRHRPGSIRSNSSFEMSKSSSVH  540
            SEAIPEFTTNNHSDSQNGSRRNSTLLPSMINPTVLARSRHRPGSIRSNSSFEMSKSSSVH
Sbjct  481  SEAIPEFTTNNHSDSQNGSRRNSTLLPSMINPTVLARSRHRPGSIRSNSSFEMSKSSSVH  540

Query  541  DRTEEISTTKLPILGRNSSRSSSRSRYSSISTNSSTTSLSVKDSNGHQPNSNYSTARFLR  600
            DRTEEISTTKLPILGRNSSRSSSRSRYSSISTNSSTTSLSVKDSNGHQPNSNYSTARFLR
Sbjct  541  DRTEEISTTKLPILGRNSSRSSSRSRYSSISTNSSTTSLSVKDSNGHQPNSNYSTARFLR  600

Query  601  FSDLFPAPPNHPSFIRSRSNKGEALTPMSTPIVVEGDLTTRQFETESTESKHEIEIKSPV  660
            FSDLFPAPPNHPSFIRSRSNKGEALTPMSTPIVVEGDLTTRQFETESTESKHEIEIKSPV
Sbjct  601  FSDLFPAPPNHPSFIRSRSNKGEALTPMSTPIVVEGDLTTRQFETESTESKHEIEIKSPV  660

Query  661  LDDVAAKDKDGTEALKSDAKDDPVSVQKDESTAWNDLGGGLYVCVSCVKHGYSSVSSSED  720
            LDDVAAKDKDGTEALKSDAKDDPVSVQKDESTAWNDLGGGLYVCVSCVKHGYSSVSSSED
Sbjct  661  LDDVAAKDKDGTEALKSDAKDDPVSVQKDESTAWNDLGGGLYVCVSCVKHGYSSVSSSED  720

Query  721  SGKPNGSGVDDDEEEEEEEIIWKVVIKKKRPIQPLNTNLYADQTPRREYERKNNLGSSLN  780
            SGKPNGSGVDDDEEEEEEEIIWKVVIKKKRPIQPLNTNLYADQTPRREYERKNNLGSSLN
Sbjct  721  SGKPNGSGVDDDEEEEEEEIIWKVVIKKKRPIQPLNTNLYADQTPRREYERKNNLGSSLN  780

Query  781  SSTNTTVTSTTMASNSSKTFGSSAGIITPGQTPTKRTTMNDLHSVSPLHPTEAISPSTNN  840
            SSTNTTVTSTTMASNSSKTFGSSAGIITPGQTPTKRTTMNDLHSVSPLHPTEAISPSTNN
Sbjct  781  SSTNTTVTSTTMASNSSKTFGSSAGIITPGQTPTKRTTMNDLHSVSPLHPTEAISPSTNN  840

Query  841  TLSLTLSLSPSTFSTTSGRLTFLPIPSLVPPSPTKETPSLFKSSVSSSPTSLTSPIGAWS  900
            TLSLTLSLSPSTFSTTSGRLTFLPIPSLVPPSPTKETPSLFKSSVSSSPTSLTSPIGAWS
Sbjct  841  TLSLTLSLSPSTFSTTSGRLTFLPIPSLVPPSPTKETPSLFKSSVSSSPTSLTSPIGAWS  900

Query  901  REQMDGTQRRSPGKNHTRTRSEFGQGWSGRSPNKRPVSRLHERTWSDFGMGVGKGLSASP  960
            REQMDGTQRRSPGKNHTRTRSEFGQGWSGRSPNKRPVSRLHERTWSDFGMGVGKGLSASP
Sbjct  901  REQMDGTQRRSPGKNHTRTRSEFGQGWSGRSPNKRPVSRLHERTWSDFGMGVGKGLSASP  960

Query  961  KHYSLKDLVGESG  973
            KHYSLKDLVGESG
Sbjct  961  KHYSLKDLVGESG  973


Lambda      K        H        a         alpha
   0.305    0.121    0.337    0.792     4.96 

Gapped
Lambda      K        H        a         alpha    sigma
   0.267   0.0410    0.140     1.90     42.6     43.6 

Effective search space used: 11816082748560


  Database: nr
    Posted date:  Sep 23, 2015 12:05 AM
  Number of letters in database: 26,053,659,533
  Number of sequences in database:  71,551,133


Matrix: BLOSUM62
Gap Penalties: Existence: 11, Extension: 1
Neighboring words threshold: 11
Window for multiple hits: 40
```
