# Supplementary material for: Comparative genomics provides new insights into the diversity, physiology, and sexuality of the only industrially exploited tremellomycete: Phaffia rhodozyma
Source: BMC Genomics. 2016 Nov 9;17:901. doi: 10.1186/s12864-016-3244-7 (PMC5103461; doi:10.1186/s12864-016-3244-7)
Supplement: Additional file 6: — List of orphan genes with links to PFAM (related to Additional file 1: Table S1). (ZIP 1428 kb) [file 12864_2016_3244_MOESM6_ESM.zip › BLAST_HTML_FTR/G02549_P.html]

BLAST Search Results


```
BLASTP 2.2.27+


Reference:
Stephen F. Altschul, Thomas L. Madden, Alejandro A. Schäffer,
Jinghui Zhang, Zheng Zhang, Webb Miller, and David J. Lipman (1997),
"Gapped BLAST and PSI-BLAST: a new generation of protein database
search programs", Nucleic Acids Res. 25:3389-3402.


Reference for
composition-based statistics:
Alejandro A. Schäffer, L. Aravind, Thomas L. Madden, Sergei
Shavirin, John L. Spouge, Yuri I. Wolf, Eugene V. Koonin, and
Stephen F. Altschul (2001), "Improving the accuracy of PSI-BLAST
protein database searches with composition-based statistics and
other refinements", Nucleic Acids Res. 29:2994-3005.


Database: nr
           71,551,133 sequences; 26,053,659,533 total letters


Query= G02549_P

Length=233
                                                                      Score     E
Sequences producing significant alignments:                          (Bits)  Value

emb|CDZ97745.1|  hypothetical protein [Xanthophyllomyces dendrorh...   461    1e-162
gb|KDN47431.1|  hypothetical protein RSAG8_03571, partial [Rhizoc...  46.6    0.009 
emb|CDO75208.1|  hypothetical protein BN946_scf184794.g15 [Tramet...  45.4    0.027 
gb|KEP53758.1|  putative transmembrane protein [Rhizoctonia solan...  44.7    0.037 
gb|EUC66218.1|  transmembrane protein, putative [Rhizoctonia sola...  44.7    0.039 
gb|ELU45328.1|  hypothetical protein AG1IA_00651 [Rhizoctonia sol...  43.5    0.099 
ref|WP_005895237.1|  membrane protein [Fusobacterium nucleatum] >...  43.5    0.15  
emb|CCO27501.1|  hypothetical protein BN14_01481 [Rhizoctonia sol...  41.2    0.48  
gb|EJU06308.1|  hypothetical protein DACRYDRAFT_19522 [Dacryopina...  41.6    0.49  
gb|KII94551.1|  hypothetical protein PLICRDRAFT_47589 [Plicaturop...  41.2    0.62  
gb|EIF33701.1|  transcriptional regulator [Burkholderia sp. Ch1-1]    39.3    1.9   
gb|KIM22894.1|  hypothetical protein M408DRAFT_28341 [Serendipita...  38.9    3.6   
gb|EMZ16778.1|  YSIRK family gram-positive signal peptide [Lactob...  38.5    5.7   
ref|WP_036967819.1|  hypothetical protein [Providencia alcalifaci...  38.5    6.4   
ref|WP_003442551.1|  MULTISPECIES: tetraacyldisaccharide 4'-kinas...  37.4    9.8   
gb|KIK36056.1|  hypothetical protein CY34DRAFT_811647 [Suillus lu...  37.4    9.9   


 >emb|CDZ97745.1| hypothetical protein [Xanthophyllomyces dendrorhous]
Length=232

 Score =  461 bits (1187),  Expect = 1e-162, Method: Compositional matrix adjust.
 Identities = 231/232 (99%), Positives = 232/232 (100%), Gaps = 0/232 (0%)

Query  1    MPPVVPIFLSVAALSGLAYAFVYEPYLADRLETWLYGVLETRRQNRRQPVRAQPAEWAQT  60
            MPPVVPIFLSVAALSGLAYAFVYEPYLADRLETWLYGVLETRRQNRRQPVRAQPAEWAQT
Sbjct  1    MPPVVPIFLSVAALSGLAYAFVYEPYLADRLETWLYGVLETRRQNRRQPVRAQPAEWAQT  60

Query  61   SSLDIDMMSSGTWDDRSMNIGLIHSREPIESESGLLRHRRAKDTTRTLFDYADRIDLPPL  120
            SSLDIDMMSSGTWDDRSMNIGL+HSREPIESESGLLRHRRAKDTTRTLFDYADRIDLPPL
Sbjct  61   SSLDIDMMSSGTWDDRSMNIGLMHSREPIESESGLLRHRRAKDTTRTLFDYADRIDLPPL  120

Query  121  LPVSSIRTPSNELMSIPALTPDPTFFTPATSDPPSQCLTPTDPQTPKRPSSPSGMTTSSD  180
            LPVSSIRTPSNELMSIPALTPDPTFFTPATSDPPSQCLTPTDPQTPKRPSSPSGMTTSSD
Sbjct  121  LPVSSIRTPSNELMSIPALTPDPTFFTPATSDPPSQCLTPTDPQTPKRPSSPSGMTTSSD  180

Query  181  MLSSVSDISRHVLSRSGSLSSGIGSVSGSIGFETDDPFGAEDESSDGGWSEV  232
            MLSSVSDISRHVLSRSGSLSSGIGSVSGSIGFETDDPFGAEDESSDGGWSEV
Sbjct  181  MLSSVSDISRHVLSRSGSLSSGIGSVSGSIGFETDDPFGAEDESSDGGWSEV  232


>gb|KDN47431.1| hypothetical protein RSAG8_03571, partial [Rhizoctonia solani 
AG-8 WAC10335]
Length=298

 Score = 46.6 bits (109),  Expect = 0.009, Method: Compositional matrix adjust.
 Identities = 33/93 (35%), Positives = 47/93 (51%), Gaps = 5/93 (5%)

Query  11   VAALSGLAYA-FVYEPYLADRLETWLYGVLETRRQNRRQPVRAQPAEWAQTSSLDIDMMS  69
            +A  +G A+  FVY+PYL+ RL  ++  + E R Q  RQPV    A  ++ +S  +D  S
Sbjct  13   IAVGAGFAFKQFVYDPYLSHRLHEFMEDLHEQREQRHRQPVEPHRASPSRGTSASVDSFS  72

Query  70   SGTWDDRSMNIGLIHSREPIE-SESGLLRHRRA  101
                  R+ + GL    E  E S S  LR R A
Sbjct  73   E---KRRASDAGLATGTEMREVSSSNSLRRRGA  102


>emb|CDO75208.1| hypothetical protein BN946_scf184794.g15 [Trametes cinnabarina]
Length=344

 Score = 45.4 bits (106),  Expect = 0.027, Method: Compositional matrix adjust.
 Identities = 30/70 (43%), Positives = 37/70 (53%), Gaps = 8/70 (11%)

Query  21  FVYEPYLADRLETWLYGVLETRRQNRRQ---PVRA--QPAEWAQTSSLD---IDMMSSGT  72
           FVYEP++A ++ETW    LE RRQ +RQ   PV A   P E    +SL     D      
Sbjct  16  FVYEPHIAPKVETWAESFLENRRQKKRQRQGPVLADPHPLEQGDENSLRRLFSDPRDKKN  75

Query  73  WDDRSMNIGL  82
            DD SM+I L
Sbjct  76  DDDCSMSIEL  85


>gb|KEP53758.1| putative transmembrane protein [Rhizoctonia solani 123E]
Length=303

 Score = 44.7 bits (104),  Expect = 0.037, Method: Compositional matrix adjust.
 Identities = 22/60 (37%), Positives = 33/60 (55%), Gaps = 1/60 (2%)

Query  11  VAALSGLAYA-FVYEPYLADRLETWLYGVLETRRQNRRQPVRAQPAEWAQTSSLDIDMMS  69
           +A  +G A+  FVY+PYLA R+  ++  + E R Q  RQPV    A  ++  S  +D  S
Sbjct  13  IAVGAGFAFKQFVYDPYLAHRIHEFMEDLHEQREQRHRQPVEPHLASPSRGKSASVDSFS  72


>gb|EUC66218.1| transmembrane protein, putative [Rhizoctonia solani AG-3 Rhs1AP]
Length=303

 Score = 44.7 bits (104),  Expect = 0.039, Method: Compositional matrix adjust.
 Identities = 32/92 (35%), Positives = 43/92 (47%), Gaps = 3/92 (3%)

Query  11   VAALSGLAYA-FVYEPYLADRLETWLYGVLETRRQNRRQPVRAQPAEWAQTSSLDIDMMS  69
            +A  +G A+  FVY+PYLA R+  ++  + E R Q  RQPV    A  ++  S  +D  S
Sbjct  13   IAVGAGFAFKQFVYDPYLAHRIHDFMEDLHEQREQRHRQPVEPHLASPSRGKSASVDSFS  72

Query  70   SG-TWDDRSMNIGLIHSREPIE-SESGLLRHR  99
                  D   N G     E  E S S  LR R
Sbjct  73   EKRRASDDGNNAGSTTGTEMREVSSSASLRRR  104


>gb|ELU45328.1| hypothetical protein AG1IA_00651 [Rhizoctonia solani AG-1 IA]
Length=305

 Score = 43.5 bits (101),  Expect = 0.099, Method: Compositional matrix adjust.
 Identities = 22/60 (37%), Positives = 32/60 (53%), Gaps = 1/60 (2%)

Query  11  VAALSGLAYA-FVYEPYLADRLETWLYGVLETRRQNRRQPVRAQPAEWAQTSSLDIDMMS  69
           +A  +G A+  FVY+PYL+ RL  ++  + E R Q  RQPV    A   +  S  +D  S
Sbjct  13  IAVGAGFAFKQFVYDPYLSHRLHEFMEDLHEQRNQRHRQPVEPHLASPRRGKSTSVDSFS  72


>ref|WP_005895237.1| membrane protein [Fusobacterium nucleatum]
 gb|EDK87851.1| outer membrane protein [Fusobacterium nucleatum subsp. polymorphum 
ATCC 10953]
 emb|CKH20167.1| Uncharacterised protein [Fusobacterium nucleatum subsp. polymorphum]
Length=3025

 Score = 43.5 bits (101),  Expect = 0.15, Method: Composition-based stats.
 Identities = 30/104 (29%), Positives = 52/104 (50%), Gaps = 3/104 (3%)

Query  86   REPIESESGLLRHRRAKDTTRTLFDYADRIDLPPLLPVSSIRTPSNELMSIPALTPDPTF  145
            REPI +E  +L +   K+ T+ +      I  PP LPV ++    N  ++ P +T +P  
Sbjct  212  REPI-NEVEILANVSPKEVTKNIKPINPNISEPPELPVPTVNPQVNTPLAAPTIT-NPEI  269

Query  146  FTPATSDPPSQCLTPTDPQ-TPKRPSSPSGMTTSSDMLSSVSDI  188
              P+    P+  +  T P  TP   ++PS + T +  LS+V+ +
Sbjct  270  VPPSIPSAPTINIGVTSPTITPLEVTTPSDVGTIAVNLSTVNPV  313


>emb|CCO27501.1| hypothetical protein BN14_01481 [Rhizoctonia solani AG-1 IB]
 emb|CEL63897.1| hypothetical protein RSOLAG1IB_05662 [Rhizoctonia solani AG-1 
IB]
Length=306

 Score = 41.2 bits (95),  Expect = 0.48, Method: Compositional matrix adjust.
 Identities = 18/41 (44%), Positives = 26/41 (63%), Gaps = 1/41 (2%)

Query  11  VAALSGLAYA-FVYEPYLADRLETWLYGVLETRRQNRRQPV  50
           +A  +G A+  FVY+PYL+ RL  ++  + E R Q  RQPV
Sbjct  13  IAVGAGFAFKQFVYDPYLSHRLHDFMEDLHEQRNQRHRQPV  53


>gb|EJU06308.1| hypothetical protein DACRYDRAFT_19522 [Dacryopinax sp. DJM-731 
SS1]
Length=389

 Score = 41.6 bits (96),  Expect = 0.49, Method: Compositional matrix adjust.
 Identities = 19/49 (39%), Positives = 30/49 (61%), Gaps = 6/49 (12%)

Query  6   PIFLSVAALS---GLAYA---FVYEPYLADRLETWLYGVLETRRQNRRQ  48
           P+F+ V  +    G AYA   F+YEPY+A +LE W    +E+RR+ + +
Sbjct  4   PVFIYVGGVIIAIGAAYALKQFIYEPYVAPQLEKWAQAFIESRRRAKEE  52


>gb|KII94551.1| hypothetical protein PLICRDRAFT_47589 [Plicaturopsis crispa FD-325 
SS-3]
Length=321

 Score = 41.2 bits (95),  Expect = 0.62, Method: Compositional matrix adjust.
 Identities = 19/52 (37%), Positives = 27/52 (52%), Gaps = 5/52 (10%)

Query  5   VPIFLSVAALSGLAYA-----FVYEPYLADRLETWLYGVLETRRQNRRQPVR  51
            P    VA + G+A       FVYEP++A ++E W    +E R+Q R Q  R
Sbjct  3   APAVYIVAVIGGVAAVIAFKEFVYEPHIAPKMEQWAASFVEKRKQKRLQKQR  54


>gb|EIF33701.1| transcriptional regulator [Burkholderia sp. Ch1-1]
Length=266

 Score = 39.3 bits (90),  Expect = 1.9, Method: Compositional matrix adjust.
 Identities = 20/92 (22%), Positives = 41/92 (45%), Gaps = 3/92 (3%)

Query  124  SSIRTPSNELMSIPALTPDPTFFTPATSDPPSQCLTPTDPQT---PKRPSSPSGMTTSSD  180
             ++++P  ++  +    P      PA +   SQ   P + +    P+RP  PSG     D
Sbjct  9    QAVKSPPKDIAGVKGRKPRAALRRPAETQTDSQADAPANLKAAAPPRRPGRPSGAARGPD  68

Query  181  MLSSVSDISRHVLSRSGSLSSGIGSVSGSIGF  212
              + + D +  + +R G + + +G ++   GF
Sbjct  69   QRNRLLDAALVLFARQGIVDTTLGEIAREAGF  100


>gb|KIM22894.1| hypothetical protein M408DRAFT_28341 [Serendipita vermifera MAFF 
305830]
Length=370

 Score = 38.9 bits (89),  Expect = 3.6, Method: Compositional matrix adjust.
 Identities = 18/44 (41%), Positives = 27/44 (61%), Gaps = 2/44 (5%)

Query  10  SVAALSGLA--YAFVYEPYLADRLETWLYGVLETRRQNRRQPVR  51
           +V A+ G+   + F+YEP +A +LE ++   LE RR  RRQ  R
Sbjct  12  AVIAVGGIVAFHQFIYEPLIAPKLEVFVENWLEQRRLKRRQLTR  55


>gb|EMZ16778.1| YSIRK family gram-positive signal peptide [Lactobacillus sp. 
ASF360]
Length=1777

 Score = 38.5 bits (88),  Expect = 5.7, Method: Composition-based stats.
 Identities = 23/53 (43%), Positives = 28/53 (53%), Gaps = 4/53 (8%)

Query  128   TPSNELMSIPALTPDPTFFTPATSDPPSQCLTPTDPQTPKRPSSPSGMTTSSD  180
             TPS    S P    +P   TP+T   PS   TP++P TP  PS PS  TT S+
Sbjct  1610  TPSEP--STPTTPSEPD--TPSTPSEPSTPTTPSEPDTPSTPSEPSTPTTPSE  1658


>ref|WP_036967819.1| hypothetical protein [Providencia alcalifaciens]
 gb|EUD06272.1| filamentous hemagglutinin family N-terminal domain protein [Providencia 
alcalifaciens R90-1475]
Length=2540

 Score = 38.5 bits (88),  Expect = 6.4, Method: Composition-based stats.
 Identities = 22/58 (38%), Positives = 28/58 (48%), Gaps = 2/58 (3%)

Query  121   LPVSSIRTPSNELMSIPALTPDPTFFTPATSDPPSQCLTPTDPQTPKRPSSPSGMTTS  178
             L  S   + S+E  S P  +  P+   P TS+ PS+  TP  P  P  P  PS  TTS
Sbjct  2381  LDASKQPSSSSEKPSQPTTSEKPS--KPTTSEKPSKPTTPEKPSKPTTPEKPSKPTTS  2436


>ref|WP_003442551.1| MULTISPECIES: tetraacyldisaccharide 4'-kinase [Pseudomonas]
 gb|EMI06460.1| tetraacyldisaccharide 4'-kinase [Pseudomonas sp. Lz4W]
Length=336

 Score = 37.4 bits (85),  Expect = 9.8, Method: Compositional matrix adjust.
 Identities = 25/82 (30%), Positives = 39/82 (48%), Gaps = 8/82 (10%)

Query  75   DRSMNIGLIHSREPIE---SESGLLRHRRAKDTTRTLFDYADRIDLPPLLPVSSIRTPSN  131
            DRS  +  + + EP++   S+ GL  +R A+D    L D A  +     LP   +R P+ 
Sbjct  131  DRSRAVQALLASEPLDLILSDDGLQHYRLARDLELVLIDNARGLGNGHCLPAGPLREPAE  190

Query  132  ELMSIPALTPDPTFFTPATSDP  153
             L+S+     D   +  A SDP
Sbjct  191  RLLSV-----DAVLYNGAASDP  207


>gb|KIK36056.1| hypothetical protein CY34DRAFT_811647 [Suillus luteus UH-Slu-Lm8-n1]
Length=410

 Score = 37.4 bits (85),  Expect = 9.9, Method: Compositional matrix adjust.
 Identities = 15/32 (47%), Positives = 19/32 (59%), Gaps = 0/32 (0%)

Query  19  YAFVYEPYLADRLETWLYGVLETRRQNRRQPV  50
           + FVYEP++A  +E W    L  RR  RR PV
Sbjct  23  HEFVYEPHIAPAIERWAEDFLANRRARRRGPV  54


Lambda      K        H        a         alpha
   0.316    0.132    0.395    0.792     4.96 

Gapped
Lambda      K        H        a         alpha    sigma
   0.267   0.0410    0.140     1.90     42.6     43.6 

Effective search space used: 1357825888005


  Database: nr
    Posted date:  Sep 23, 2015 12:05 AM
  Number of letters in database: 26,053,659,533
  Number of sequences in database:  71,551,133


Matrix: BLOSUM62
Gap Penalties: Existence: 11, Extension: 1
Neighboring words threshold: 11
Window for multiple hits: 40
```
