# Supplementary material for: Comparative genomics provides new insights into the diversity, physiology, and sexuality of the only industrially exploited tremellomycete: Phaffia rhodozyma
Source: BMC Genomics. 2016 Nov 9;17:901. doi: 10.1186/s12864-016-3244-7 (PMC5103461; doi:10.1186/s12864-016-3244-7)
Supplement: Additional file 6: — List of orphan genes with links to PFAM (related to Additional file 1: Table S1). (ZIP 1428 kb) [file 12864_2016_3244_MOESM6_ESM.zip › BLAST_HTML_FTR/G00082_P.html]

BLAST Search Results


```
BLASTP 2.2.27+


Reference:
Stephen F. Altschul, Thomas L. Madden, Alejandro A. Schäffer,
Jinghui Zhang, Zheng Zhang, Webb Miller, and David J. Lipman (1997),
"Gapped BLAST and PSI-BLAST: a new generation of protein database
search programs", Nucleic Acids Res. 25:3389-3402.


Reference for
composition-based statistics:
Alejandro A. Schäffer, L. Aravind, Thomas L. Madden, Sergei
Shavirin, John L. Spouge, Yuri I. Wolf, Eugene V. Koonin, and
Stephen F. Altschul (2001), "Improving the accuracy of PSI-BLAST
protein database searches with composition-based statistics and
other refinements", Nucleic Acids Res. 29:2994-3005.


Database: nr
           71,551,133 sequences; 26,053,659,533 total letters


Query= G00082_P

Length=1580
                                                                      Score     E
Sequences producing significant alignments:                          (Bits)  Value

emb|CED84271.1|  hypothetical protein [Xanthophyllomyces dendrorh...  3115    0.0  


 >emb|CED84271.1| hypothetical protein [Xanthophyllomyces dendrorhous]
Length=1579

 Score = 3115 bits (8076),  Expect = 0.0, Method: Compositional matrix adjust.
 Identities = 1579/1579 (100%), Positives = 1579/1579 (100%), Gaps = 0/1579 (0%)

Query  1     MSFPSAADVPGLSDDQTVNSPQRSPSIRERTASFTNRIRTSSTSSLNKLKRSASSASVSP  60
             MSFPSAADVPGLSDDQTVNSPQRSPSIRERTASFTNRIRTSSTSSLNKLKRSASSASVSP
Sbjct  1     MSFPSAADVPGLSDDQTVNSPQRSPSIRERTASFTNRIRTSSTSSLNKLKRSASSASVSP  60

Query  61    STVGHADGPPISPSAGSSSSERKNRRISLSKIFSGSSGNGRNSDKNKSDHSAPEGISPDS  120
             STVGHADGPPISPSAGSSSSERKNRRISLSKIFSGSSGNGRNSDKNKSDHSAPEGISPDS
Sbjct  61    STVGHADGPPISPSAGSSSSERKNRRISLSKIFSGSSGNGRNSDKNKSDHSAPEGISPDS  120

Query  121   PSSGSRMSLSKSMSFGSLRSKKEAKSLHRSSHQSQRTHRTPLTPEALPPQLSRSVTPPGQ  180
             PSSGSRMSLSKSMSFGSLRSKKEAKSLHRSSHQSQRTHRTPLTPEALPPQLSRSVTPPGQ
Sbjct  121   PSSGSRMSLSKSMSFGSLRSKKEAKSLHRSSHQSQRTHRTPLTPEALPPQLSRSVTPPGQ  180

Query  181   SPNPSLPRSTYTGIPSPSSRRVVSPAPMTANTSTFSSAASSPSRIPRRIRVSSNPSLPPQ  240
             SPNPSLPRSTYTGIPSPSSRRVVSPAPMTANTSTFSSAASSPSRIPRRIRVSSNPSLPPQ
Sbjct  181   SPNPSLPRSTYTGIPSPSSRRVVSPAPMTANTSTFSSAASSPSRIPRRIRVSSNPSLPPQ  240

Query  241   IDTLTSPVRARASTSHMATPDNQQHLPQVLQAKTPIASSSSSRSLSRASQAPSFLLEDPF  300
             IDTLTSPVRARASTSHMATPDNQQHLPQVLQAKTPIASSSSSRSLSRASQAPSFLLEDPF
Sbjct  241   IDTLTSPVRARASTSHMATPDNQQHLPQVLQAKTPIASSSSSRSLSRASQAPSFLLEDPF  300

Query  301   STPPPPEVVQPSTSSATAVSPSSSPSTAFDLLQMIQRERSNKSAHTTSTNGERTSSNLKR  360
             STPPPPEVVQPSTSSATAVSPSSSPSTAFDLLQMIQRERSNKSAHTTSTNGERTSSNLKR
Sbjct  301   STPPPPEVVQPSTSSATAVSPSSSPSTAFDLLQMIQRERSNKSAHTTSTNGERTSSNLKR  360

Query  361   SSSQAESILGGVLEREVEGGGRVSRSPSATRRRRLTGDMMRPREVSLSAKAESLNSPDMK  420
             SSSQAESILGGVLEREVEGGGRVSRSPSATRRRRLTGDMMRPREVSLSAKAESLNSPDMK
Sbjct  361   SSSQAESILGGVLEREVEGGGRVSRSPSATRRRRLTGDMMRPREVSLSAKAESLNSPDMK  420

Query  421   SQAGGSSGVDSMILTPESPRSQHSPVAFGEDSILGRGVQEALGPPLREPARIFSLPSSPP  480
             SQAGGSSGVDSMILTPESPRSQHSPVAFGEDSILGRGVQEALGPPLREPARIFSLPSSPP
Sbjct  421   SQAGGSSGVDSMILTPESPRSQHSPVAFGEDSILGRGVQEALGPPLREPARIFSLPSSPP  480

Query  481   SSVTPDKIPIPLTDELMEALKLGRVMPQSSPLPKAESTGKDVSTVDARSEEIEAEEAAPE  540
             SSVTPDKIPIPLTDELMEALKLGRVMPQSSPLPKAESTGKDVSTVDARSEEIEAEEAAPE
Sbjct  481   SSVTPDKIPIPLTDELMEALKLGRVMPQSSPLPKAESTGKDVSTVDARSEEIEAEEAAPE  540

Query  541   HQLERAIPDEALTSDEPANRARTPSVEARLRETEVFNSGANANVSSNDREDVRDDAPSNL  600
             HQLERAIPDEALTSDEPANRARTPSVEARLRETEVFNSGANANVSSNDREDVRDDAPSNL
Sbjct  541   HQLERAIPDEALTSDEPANRARTPSVEARLRETEVFNSGANANVSSNDREDVRDDAPSNL  600

Query  601   PRLGLDPEHVPLTGRAEHAQEVQEGAGIDEDDDHLITPTTTERSSAISSPRLSYISEESS  660
             PRLGLDPEHVPLTGRAEHAQEVQEGAGIDEDDDHLITPTTTERSSAISSPRLSYISEESS
Sbjct  601   PRLGLDPEHVPLTGRAEHAQEVQEGAGIDEDDDHLITPTTTERSSAISSPRLSYISEESS  660

Query  661   NAAGDVGGDGDNDQESESGEDINSEDIREILKGFSSRQPMSGRTLDESQSGTFENVEGHK  720
             NAAGDVGGDGDNDQESESGEDINSEDIREILKGFSSRQPMSGRTLDESQSGTFENVEGHK
Sbjct  661   NAAGDVGGDGDNDQESESGEDINSEDIREILKGFSSRQPMSGRTLDESQSGTFENVEGHK  720

Query  721   NVENEKNDYMPEARGGHQDSSKAPDSSISASSVQVSNTTTKPISDEHEPTLVSAMRLPKE  780
             NVENEKNDYMPEARGGHQDSSKAPDSSISASSVQVSNTTTKPISDEHEPTLVSAMRLPKE
Sbjct  721   NVENEKNDYMPEARGGHQDSSKAPDSSISASSVQVSNTTTKPISDEHEPTLVSAMRLPKE  780

Query  781   SSGLSESIQSDLSSTRNDSRSEPMPSSGSSAKSPPVVDDAPTTSDDHSLLGREEEVKHIK  840
             SSGLSESIQSDLSSTRNDSRSEPMPSSGSSAKSPPVVDDAPTTSDDHSLLGREEEVKHIK
Sbjct  781   SSGLSESIQSDLSSTRNDSRSEPMPSSGSSAKSPPVVDDAPTTSDDHSLLGREEEVKHIK  840

Query  841   VEQPVDEKSGDDQPVDVLSPLTEGARSDLRSQGEDDAPQDSPQKKEEGERTPVGLQSESL  900
             VEQPVDEKSGDDQPVDVLSPLTEGARSDLRSQGEDDAPQDSPQKKEEGERTPVGLQSESL
Sbjct  841   VEQPVDEKSGDDQPVDVLSPLTEGARSDLRSQGEDDAPQDSPQKKEEGERTPVGLQSESL  900

Query  901   LGDGSSQKQVETDEARQEVAHESSRLDDKTEEAKEGFHTTLEQTTDCPSPLISSSSENPL  960
             LGDGSSQKQVETDEARQEVAHESSRLDDKTEEAKEGFHTTLEQTTDCPSPLISSSSENPL
Sbjct  901   LGDGSSQKQVETDEARQEVAHESSRLDDKTEEAKEGFHTTLEQTTDCPSPLISSSSENPL  960

Query  961   DAEPTNNTEDSQIEKQEVPDSPILPKYLPLRLLTSSSIPTDNSHLDQNPVSLNTPTHDPE  1020
             DAEPTNNTEDSQIEKQEVPDSPILPKYLPLRLLTSSSIPTDNSHLDQNPVSLNTPTHDPE
Sbjct  961   DAEPTNNTEDSQIEKQEVPDSPILPKYLPLRLLTSSSIPTDNSHLDQNPVSLNTPTHDPE  1020

Query  1021  LASTHLSDEHNQNFSGLPADDVVHDHKSESAGLVNEPAKEAEIEKAERDIPTYLTPVVKI  1080
             LASTHLSDEHNQNFSGLPADDVVHDHKSESAGLVNEPAKEAEIEKAERDIPTYLTPVVKI
Sbjct  1021  LASTHLSDEHNQNFSGLPADDVVHDHKSESAGLVNEPAKEAEIEKAERDIPTYLTPVVKI  1080

Query  1081  SSSLTPQSSPEAIQEGMYDLGEPSSPLNITDQQTVSHKTSPVDSKLKLSSDPSSDSPGAI  1140
             SSSLTPQSSPEAIQEGMYDLGEPSSPLNITDQQTVSHKTSPVDSKLKLSSDPSSDSPGAI
Sbjct  1081  SSSLTPQSSPEAIQEGMYDLGEPSSPLNITDQQTVSHKTSPVDSKLKLSSDPSSDSPGAI  1140

Query  1141  ILPPDVPSTTTIDDVSPSTIKESMPTTPSLEREDRAIQEGASASIAEQTLKQVLDPKRVL  1200
             ILPPDVPSTTTIDDVSPSTIKESMPTTPSLEREDRAIQEGASASIAEQTLKQVLDPKRVL
Sbjct  1141  ILPPDVPSTTTIDDVSPSTIKESMPTTPSLEREDRAIQEGASASIAEQTLKQVLDPKRVL  1200

Query  1201  IEDKTGELASYEARIASADRNLVDKGKEHRQDSLVRDSGAQTKLTRSESSESLPKEPLNK  1260
             IEDKTGELASYEARIASADRNLVDKGKEHRQDSLVRDSGAQTKLTRSESSESLPKEPLNK
Sbjct  1201  IEDKTGELASYEARIASADRNLVDKGKEHRQDSLVRDSGAQTKLTRSESSESLPKEPLNK  1260

Query  1261  AFPSHHISEDLQHEEQPGSALPSTAEDDPGVSLGKEQDRKAEISSATLPSSTHPTSMNTQ  1320
             AFPSHHISEDLQHEEQPGSALPSTAEDDPGVSLGKEQDRKAEISSATLPSSTHPTSMNTQ
Sbjct  1261  AFPSHHISEDLQHEEQPGSALPSTAEDDPGVSLGKEQDRKAEISSATLPSSTHPTSMNTQ  1320

Query  1321  FSVNGETKDEPPKHFSDELSAETTPSISKTHPQPLEPNTAVISDQPLPPKATSTPLVSSP  1380
             FSVNGETKDEPPKHFSDELSAETTPSISKTHPQPLEPNTAVISDQPLPPKATSTPLVSSP
Sbjct  1321  FSVNGETKDEPPKHFSDELSAETTPSISKTHPQPLEPNTAVISDQPLPPKATSTPLVSSP  1380

Query  1381  TPFVAADVSSSSSRPNLPSRALTTPTRSSTPQRPQLPRAATTNSINTFELPNPFASLLNS  1440
             TPFVAADVSSSSSRPNLPSRALTTPTRSSTPQRPQLPRAATTNSINTFELPNPFASLLNS
Sbjct  1381  TPFVAADVSSSSSRPNLPSRALTTPTRSSTPQRPQLPRAATTNSINTFELPNPFASLLNS  1440

Query  1441  QRFFIRIPVSLLPTRLASFAQQPTFSIPSFFPGAVDPSEGSRSRSHSPDSVQTIGYASTS  1500
             QRFFIRIPVSLLPTRLASFAQQPTFSIPSFFPGAVDPSEGSRSRSHSPDSVQTIGYASTS
Sbjct  1441  QRFFIRIPVSLLPTRLASFAQQPTFSIPSFFPGAVDPSEGSRSRSHSPDSVQTIGYASTS  1500

Query  1501  NDGTEPVSGFGSGLESGKALLGTAASIVGTSVKWGFGWPVLVPLRAGQYVLTTLSSHAGL  1560
             NDGTEPVSGFGSGLESGKALLGTAASIVGTSVKWGFGWPVLVPLRAGQYVLTTLSSHAGL
Sbjct  1501  NDGTEPVSGFGSGLESGKALLGTAASIVGTSVKWGFGWPVLVPLRAGQYVLTTLSSHAGL  1560

Query  1561  GVDSAVREKDKSVTTVANS  1579
             GVDSAVREKDKSVTTVANS
Sbjct  1561  GVDSAVREKDKSVTTVANS  1579


Lambda      K        H        a         alpha
   0.304    0.122    0.331    0.792     4.96 

Gapped
Lambda      K        H        a         alpha    sigma
   0.267   0.0410    0.140     1.90     42.6     43.6 

Effective search space used: 20160527462020


  Database: nr
    Posted date:  Sep 23, 2015 12:05 AM
  Number of letters in database: 26,053,659,533
  Number of sequences in database:  71,551,133


Matrix: BLOSUM62
Gap Penalties: Existence: 11, Extension: 1
Neighboring words threshold: 11
Window for multiple hits: 40
```
