# Supplementary material for: Comparative genomics provides new insights into the diversity, physiology, and sexuality of the only industrially exploited tremellomycete: Phaffia rhodozyma
Source: BMC Genomics. 2016 Nov 9;17:901. doi: 10.1186/s12864-016-3244-7 (PMC5103461; doi:10.1186/s12864-016-3244-7)
Supplement: Additional file 6: — List of orphan genes with links to PFAM (related to Additional file 1: Table S1). (ZIP 1428 kb) [file 12864_2016_3244_MOESM6_ESM.zip › BLAST_HTML_FTR/G01128_P.html]

BLAST Search Results


```
BLASTP 2.2.27+


Reference:
Stephen F. Altschul, Thomas L. Madden, Alejandro A. Schäffer,
Jinghui Zhang, Zheng Zhang, Webb Miller, and David J. Lipman (1997),
"Gapped BLAST and PSI-BLAST: a new generation of protein database
search programs", Nucleic Acids Res. 25:3389-3402.


Reference for
composition-based statistics:
Alejandro A. Schäffer, L. Aravind, Thomas L. Madden, Sergei
Shavirin, John L. Spouge, Yuri I. Wolf, Eugene V. Koonin, and
Stephen F. Altschul (2001), "Improving the accuracy of PSI-BLAST
protein database searches with composition-based statistics and
other refinements", Nucleic Acids Res. 29:2994-3005.


Database: nr
           71,551,133 sequences; 26,053,659,533 total letters


Query= G01128_P

Length=119
                                                                      Score     E
Sequences producing significant alignments:                          (Bits)  Value

emb|CDZ97312.1|  hypothetical protein [Xanthophyllomyces dendrorh...   183    7e-57
ref|WP_044654993.1|  membrane protein [Bacteroides acidifaciens]      40.4    0.19 
gb|KIL57648.1|  hypothetical protein M378DRAFT_171546 [Amanita mu...  38.9    0.63 
ref|WP_032854986.1|  membrane protein [Bacteroides caccae]            38.5    0.76 
ref|WP_005680711.1|  membrane protein [Bacteroides caccae] >gb|EI...  38.5    0.77 
ref|XP_008588585.1|  PREDICTED: E3 ubiquitin-protein ligase RNF21...  38.5    0.87 
gb|EDM22612.1|  hypothetical protein BACCAC_01000 [Bacteroides ca...  37.7    1.4  
ref|WP_034224109.1|  hypothetical protein [Lachnospiraceae bacter...  37.0    2.4  
gb|EOS30272.1|  hypothetical protein C804_03295 [Lachnospiraceae ...  37.0    2.6  
emb|CCZ71783.1|  uncharacterized protein BN535_01532 [Bacteroides...  37.0    3.0  
gb|KOP48811.1|  putative transporter [Madurella mycetomatis]          36.6    3.6  
ref|WP_004436087.1|  hypothetical protein [Bacillus methanolicus]...  35.4    3.9  
gb|EST06827.1|  hypothetical protein PSEUBRA_SCAF25g01068 [Pseudo...  36.2    4.3  
ref|WP_051137230.1|  UDP pyrophosphate synthase [Sphingopyxis bae...  36.2    4.4  
ref|XP_004343947.1|  leucine rich repeat domain containing protei...  36.2    4.7  
ref|WP_043309432.1|  hypothetical protein [Pseudomonas sp. ML96]      35.8    5.6  
ref|WP_037025343.1|  hypothetical protein [Pseudomonas sp. 20_BN]...  35.4    9.9  


 >emb|CDZ97312.1| hypothetical protein [Xanthophyllomyces dendrorhous]
Length=105

 Score =  183 bits (465),  Expect = 7e-57, Method: Compositional matrix adjust.
 Identities = 93/93 (100%), Positives = 93/93 (100%), Gaps = 0/93 (0%)

Query  1   MFARSILRSARPQVLMATRPISRSVVLNKGAMDSLREKASDVASKVSDATSSAATNSNAD  60
           MFARSILRSARPQVLMATRPISRSVVLNKGAMDSLREKASDVASKVSDATSSAATNSNAD
Sbjct  1   MFARSILRSARPQVLMATRPISRSVVLNKGAMDSLREKASDVASKVSDATSSAATNSNAD  60

Query  61  VSNKSSTSDMPPELAELTDPHAGEKVDKVLRAQ  93
           VSNKSSTSDMPPELAELTDPHAGEKVDKVLRAQ
Sbjct  61  VSNKSSTSDMPPELAELTDPHAGEKVDKVLRAQ  93


>ref|WP_044654993.1| membrane protein [Bacteroides acidifaciens]
Length=739

 Score = 40.4 bits (93),  Expect = 0.19, Method: Composition-based stats.
 Identities = 31/91 (34%), Positives = 49/91 (54%), Gaps = 4/91 (4%)

Query  1    MFARSILRSARPQVLMATRPISRSVVLNKGAMDSLREKASDVASKVSDA--TSSAATNSN  58
            +FA +  RS   ++       ++ V+ N   +D LRE AS +  K+S+A   SS+ T++N
Sbjct  281  LFAFNSTRSMLEELEKHMDSNAKQVLKNLEFVDKLRE-ASTLTGKISEAEAMSSSQTDNN  339

Query  59   ADVSN-KSSTSDMPPELAELTDPHAGEKVDK  88
            A + N K    ++  EL ELTD + G K  K
Sbjct  340  ARIGNDKKKLGELRQELRELTDSYVGHKYTK  370


>gb|KIL57648.1| hypothetical protein M378DRAFT_171546 [Amanita muscaria Koide 
BX008]
Length=1062

 Score = 38.9 bits (89),  Expect = 0.63, Method: Composition-based stats.
 Identities = 25/81 (31%), Positives = 38/81 (47%), Gaps = 6/81 (7%)

Query  36   REKASDVASKVSDATSSAATNSNADVSNKSSTSDMP--PELAELTDPHAGEKVDKVLRAQ  93
            R +AS V   +   TS         V+  SST+D P  P+LAEL D +A   +   +R +
Sbjct  693  RRRASSVVISIKRKTSQLLEA----VTPSSSTNDAPVTPKLAELLDSYAKSDIAIAIREE  748

Query  94   GKDLGKHEGDKASDGNGHVVP  114
            G+  G+  G +     G  +P
Sbjct  749  GEAFGREHGRQHGQEPGQELP  769


>ref|WP_032854986.1| membrane protein [Bacteroides caccae]
Length=740

 Score = 38.5 bits (88),  Expect = 0.76, Method: Composition-based stats.
 Identities = 29/97 (30%), Positives = 52/97 (54%), Gaps = 8/97 (8%)

Query  1    MFARSILRSARPQVLMATRPISRSVVLNKGAMDSLREKASDVASKVSDATSSAATNSNAD  60
            MFA +  RS   ++       ++ ++ N   +  ++E ASD+  ++S+  + + +N N D
Sbjct  281  MFALNSSRSMLDELERHMDSNAKQIIKNMEFVGKIKE-ASDITGRISEVEAMSDSN-NKD  338

Query  61   VS----NKSSTSDMPPELAELTDPHAGEKVDK--VLR  91
            ++    NK   S++  EL+ELTD + G K  K  VLR
Sbjct  339  ITALNNNKKRLSELKKELSELTDAYVGHKYSKEGVLR  375


>ref|WP_005680711.1| membrane protein [Bacteroides caccae]
 gb|EIY21681.1| hypothetical protein HMPREF1061_01412 [Bacteroides caccae CL03T12C61]
Length=740

 Score = 38.5 bits (88),  Expect = 0.77, Method: Composition-based stats.
 Identities = 29/97 (30%), Positives = 52/97 (54%), Gaps = 8/97 (8%)

Query  1    MFARSILRSARPQVLMATRPISRSVVLNKGAMDSLREKASDVASKVSDATSSAATNSNAD  60
            MFA +  RS   ++       ++ ++ N   +  ++E ASD+  ++S+  + + +N N D
Sbjct  281  MFALNSSRSMLDELERHMDSNAKQIIKNMEFVGKIKE-ASDITGRISEVEAMSDSN-NKD  338

Query  61   VS----NKSSTSDMPPELAELTDPHAGEKVDK--VLR  91
            ++    NK   S++  EL+ELTD + G K  K  VLR
Sbjct  339  ITALNNNKKRLSELKKELSELTDAYVGHKYSKEGVLR  375


>ref|XP_008588585.1| PREDICTED: E3 ubiquitin-protein ligase RNF213, partial [Galeopterus 
variegatus]
Length=4580

 Score = 38.5 bits (88),  Expect = 0.87, Method: Composition-based stats.
 Identities = 23/71 (32%), Positives = 34/71 (48%), Gaps = 3/71 (4%)

Query  36   REKASDVASKVSDATSSAATNSNADVSNKSSTSDMPPELAELTDPH---AGEKVDKVLRA  92
            R++A   AS   DA +     +  +V +K+ T+  PP     +  H   AGEK  K  R 
Sbjct  265  RQQAGAPASGAVDAAAEPVKGAEEEVKDKTQTTKQPPATTTTSSRHCQEAGEKAGKDERV  324

Query  93   QGKDLGKHEGD  103
            + KD  K EG+
Sbjct  325  KPKDPKKPEGN  335


>gb|EDM22612.1| hypothetical protein BACCAC_01000 [Bacteroides caccae ATCC 43185]
Length=705

 Score = 37.7 bits (86),  Expect = 1.4, Method: Composition-based stats.
 Identities = 29/97 (30%), Positives = 52/97 (54%), Gaps = 8/97 (8%)

Query  1    MFARSILRSARPQVLMATRPISRSVVLNKGAMDSLREKASDVASKVSDATSSAATNSNAD  60
            MFA +  RS   ++       ++ ++ N   +  ++E ASD+  ++S+  + + +N N D
Sbjct  246  MFALNSSRSMLDELERHMDSNAKQIIKNMEFVGKIKE-ASDITGRISEVEAMSDSN-NKD  303

Query  61   VS----NKSSTSDMPPELAELTDPHAGEKVDK--VLR  91
            ++    NK   S++  EL+ELTD + G K  K  VLR
Sbjct  304  ITALNNNKKRLSELKKELSELTDAYVGHKYSKEGVLR  340


>ref|WP_034224109.1| hypothetical protein [Lachnospiraceae bacterium A4]
Length=282

 Score = 37.0 bits (84),  Expect = 2.4, Method: Compositional matrix adjust.
 Identities = 25/71 (35%), Positives = 38/71 (54%), Gaps = 2/71 (3%)

Query  29   KGAMDSLREKASDVASKVSDATSSAATNSNADVSNKSSTSDMPPELAE--LTDPHAGEKV  86
            K  +  LREK  ++   V++ ++    +  A+ SNK  +  + PEL E   TDP A EK 
Sbjct  103  KSLLAELREKYGNMDISVAEWSTDEEQDYYAEQSNKDYSVLINPELLEKMATDPAAREKY  162

Query  87   DKVLRAQGKDL  97
            +KVL   G +L
Sbjct  163  EKVLSGAGDEL  173


>gb|EOS30272.1| hypothetical protein C804_03295 [Lachnospiraceae bacterium A4]
Length=280

 Score = 37.0 bits (84),  Expect = 2.6, Method: Compositional matrix adjust.
 Identities = 25/71 (35%), Positives = 38/71 (54%), Gaps = 2/71 (3%)

Query  29   KGAMDSLREKASDVASKVSDATSSAATNSNADVSNKSSTSDMPPELAE--LTDPHAGEKV  86
            K  +  LREK  ++   V++ ++    +  A+ SNK  +  + PEL E   TDP A EK 
Sbjct  101  KSLLAELREKYGNMDISVAEWSTDEEQDYYAEQSNKDYSVLINPELLEKMATDPAAREKY  160

Query  87   DKVLRAQGKDL  97
            +KVL   G +L
Sbjct  161  EKVLSGAGDEL  171


>emb|CCZ71783.1| uncharacterized protein BN535_01532 [Bacteroides caccae CAG:21]
Length=740

 Score = 37.0 bits (84),  Expect = 3.0, Method: Composition-based stats.
 Identities = 28/97 (29%), Positives = 52/97 (54%), Gaps = 8/97 (8%)

Query  1    MFARSILRSARPQVLMATRPISRSVVLNKGAMDSLREKASDVASKVSDATSSAATNSNAD  60
            MFA +  RS   ++       ++ ++ N   +  ++E ASD+  ++S+  + + +N N D
Sbjct  281  MFALNSSRSMLDELERHMDSNAKQIIKNMEFVGKIKE-ASDITGRISEVEAMSDSN-NKD  338

Query  61   VS----NKSSTSDMPPELAELTDPHAGEKVDK--VLR  91
            ++    NK   S++  +L+ELTD + G K  K  VLR
Sbjct  339  ITALNNNKKRLSELKKKLSELTDAYVGHKYSKEGVLR  375


>gb|KOP48811.1| putative transporter [Madurella mycetomatis]
Length=514

 Score = 36.6 bits (83),  Expect = 3.6, Method: Composition-based stats.
 Identities = 18/51 (35%), Positives = 31/51 (61%), Gaps = 1/51 (2%)

Query  42  VASKVSDATSSAATNSNADVSNKSSTSD-MPPELAELTDPHAGEKVDKVLR  91
           ++  +S A +  AT SN D    SS+ D +PP++A L +   G+K +K++R
Sbjct  1   MSDAMSPAKADPATMSNTDEKPASSSDDGLPPDVAALMEEFTGDKYNKLMR  51


>ref|WP_004436087.1| hypothetical protein [Bacillus methanolicus]
 gb|EIJ77970.1| hypothetical protein PB1_10379 [Bacillus methanolicus PB1]
Length=130

 Score = 35.4 bits (80),  Expect = 3.9, Method: Compositional matrix adjust.
 Identities = 22/60 (37%), Positives = 34/60 (57%), Gaps = 1/60 (2%)

Query  30  GAMDSLREKASDV-ASKVSDATSSAATNSNADVSNKSSTSDMPPELAELTDPHAGEKVDK  88
           G  DS  EK  D+ A ++S+AT + + NS+ DV+ K +   MP  LA L    A +++ K
Sbjct  33  GLRDSKEEKVEDLLAKQLSEATVNQSGNSDVDVNVKVNIDTMPIALALLCQSFANKQLTK  92


>gb|EST06827.1| hypothetical protein PSEUBRA_SCAF25g01068 [Pseudozyma brasiliensis 
GHG001]
Length=580

 Score = 36.2 bits (82),  Expect = 4.3, Method: Compositional matrix adjust.
 Identities = 28/99 (28%), Positives = 41/99 (41%), Gaps = 18/99 (18%)

Query  16   MATRPISRSVVLNKGAMDSLREKASDVASKVSDATSSAATNSNADVSNKSSTSDMPPELA  75
            ++T P+SR V ++ G  D                   A   + A  S +SS  DMP  L 
Sbjct  426  VSTAPVSRKVSIDVGLADR------------------APRATQAPQSTESSPQDMPSVLQ  467

Query  76   ELTDPHAGEKVDKVLRAQGKDLGKHEGDKASDGNGHVVP  114
              TDP A +KV    R +G  +G H+     +G  +  P
Sbjct  468  RTTDPEAEDKVSTYNRMRGHTIGVHDERARHEGEHYTRP  506


>ref|WP_051137230.1| UDP pyrophosphate synthase [Sphingopyxis baekryungensis]
Length=230

 Score = 36.2 bits (82),  Expect = 4.4, Method: Compositional matrix adjust.
 Identities = 18/49 (37%), Positives = 29/49 (59%), Gaps = 0/49 (0%)

Query  24   SVVLNKGAMDSLREKASDVASKVSDATSSAATNSNADVSNKSSTSDMPP  72
            +V LN GA D L    + +A +++D T +A + +   VS +  T+DMPP
Sbjct  124  AVALNYGAQDELLRATASLAGQIADGTLAADSLTPDHVSAQLDTADMPP  172


>ref|XP_004343947.1| leucine rich repeat domain containing protein [Acanthamoeba castellanii 
str. Neff]
 gb|ELR20544.1| leucine rich repeat domain containing protein [Acanthamoeba castellanii 
str. Neff]
Length=984

 Score = 36.2 bits (82),  Expect = 4.7, Method: Composition-based stats.
 Identities = 17/57 (30%), Positives = 32/57 (56%), Gaps = 0/57 (0%)

Query  24   SVVLNKGAMDSLREKASDVASKVSDATSSAATNSNADVSNKSSTSDMPPELAELTDP  80
            ++VL +  +  LR ++ D +S  S A  S A N  ADV+++    ++ PE +++  P
Sbjct  612  ALVLLRAGIAWLRRRSGDTSSATSSAVPSPAINRAADVASELVGHELTPEASDVRKP  668


>ref|WP_043309432.1| hypothetical protein [Pseudomonas sp. ML96]
Length=615

 Score = 35.8 bits (81),  Expect = 5.6, Method: Compositional matrix adjust.
 Identities = 20/44 (45%), Positives = 25/44 (57%), Gaps = 2/44 (5%)

Query  74   LAELTDPHAGEKVDKVLRAQGKDLGKHEGDKASDGNGHVVPGGH  117
            ++ L DP   E VD VL A GK LG+  GD   D NG ++  GH
Sbjct  238  VSSLLDPLPAETVDVVLDADGKVLGRIVGDNVVDMNGRII--GH  279


>ref|WP_037025343.1| hypothetical protein [Pseudomonas sp. 20_BN]
 emb|CDZ95493.1| hypothetical protein BN1079_02828 [Pseudomonas sp. 20_BN]
Length=677

 Score = 35.4 bits (80),  Expect = 9.9, Method: Compositional matrix adjust.
 Identities = 20/44 (45%), Positives = 25/44 (57%), Gaps = 2/44 (5%)

Query  74   LAELTDPHAGEKVDKVLRAQGKDLGKHEGDKASDGNGHVVPGGH  117
            +A L DP   E VD VL A GK LG+  GD   D +G ++  GH
Sbjct  255  VANLLDPLPAETVDVVLDANGKLLGRIVGDNVVDHDGRII--GH  296


Lambda      K        H        a         alpha
   0.312    0.126    0.344    0.792     4.96 

Gapped
Lambda      K        H        a         alpha    sigma
   0.267   0.0410    0.140     1.90     42.6     43.6 

Effective search space used: 656708649135


  Database: nr
    Posted date:  Sep 23, 2015 12:05 AM
  Number of letters in database: 26,053,659,533
  Number of sequences in database:  71,551,133


Matrix: BLOSUM62
Gap Penalties: Existence: 11, Extension: 1
Neighboring words threshold: 11
Window for multiple hits: 40
```
