# Supplementary material for: Comparative genomics provides new insights into the diversity, physiology, and sexuality of the only industrially exploited tremellomycete: Phaffia rhodozyma
Source: BMC Genomics. 2016 Nov 9;17:901. doi: 10.1186/s12864-016-3244-7 (PMC5103461; doi:10.1186/s12864-016-3244-7)
Supplement: Additional file 6: — List of orphan genes with links to PFAM (related to Additional file 1: Table S1). (ZIP 1428 kb) [file 12864_2016_3244_MOESM6_ESM.zip › BLAST_HTML_FTR/G00541_P.html]

BLAST Search Results


```
BLASTP 2.2.27+


Reference:
Stephen F. Altschul, Thomas L. Madden, Alejandro A. Schäffer,
Jinghui Zhang, Zheng Zhang, Webb Miller, and David J. Lipman (1997),
"Gapped BLAST and PSI-BLAST: a new generation of protein database
search programs", Nucleic Acids Res. 25:3389-3402.


Reference for
composition-based statistics:
Alejandro A. Schäffer, L. Aravind, Thomas L. Madden, Sergei
Shavirin, John L. Spouge, Yuri I. Wolf, Eugene V. Koonin, and
Stephen F. Altschul (2001), "Improving the accuracy of PSI-BLAST
protein database searches with composition-based statistics and
other refinements", Nucleic Acids Res. 29:2994-3005.


Database: nr
           71,551,133 sequences; 26,053,659,533 total letters


Query= G00541_P

Length=789
                                                                      Score     E
Sequences producing significant alignments:                          (Bits)  Value

emb|CED83856.1|  hypothetical protein [Xanthophyllomyces dendrorh...  1582    0.0  
gb|KIR29002.1|  hypothetical protein I309_02044 [Cryptococcus gat...  48.9    0.020
gb|KDQ20592.1|  hypothetical protein BOTBODRAFT_40719 [Botryobasi...  48.9    0.024
gb|KGB79432.1|  hypothetical protein CNBG_5270 [Cryptococcus gatt...  48.5    0.032
gb|KIR34807.1|  hypothetical protein I352_03059 [Cryptococcus gat...  48.5    0.034
ref|XP_012049959.1|  hypothetical protein CNAG_02121 [Cryptococcu...  48.1    0.042
gb|KIJ69811.1|  hypothetical protein HYDPIDRAFT_104438 [Hydnomeru...  48.1    0.045
gb|KIK96136.1|  hypothetical protein PAXRUDRAFT_826294 [Paxillus ...  47.8    0.054
gb|KIR46107.1|  hypothetical protein I312_04650 [Cryptococcus gat...  47.4    0.062
ref|XP_003194412.1|  hypothetical protein CGB_E5430W [Cryptococcu...  47.4    0.063
gb|KIR51605.1|  hypothetical protein I315_05828 [Cryptococcus gat...  47.4    0.063
gb|KIR58865.1|  hypothetical protein I314_05278 [Cryptococcus gat...  47.4    0.064
gb|KIY42899.1|  hypothetical protein FISHEDRAFT_68277 [Fistulina ...  47.0    0.096
gb|KIR84974.1|  hypothetical protein I308_04724 [Cryptococcus gat...  46.6    0.10 
gb|KIM29494.1|  hypothetical protein M408DRAFT_114339 [Serendipit...  45.8    0.16 
gb|KNZ76045.1|  hypothetical protein J132_00320 [Termitomyces sp....  45.1    0.34 
ref|XP_003037978.1|  hypothetical protein SCHCODRAFT_102708 [Schi...  45.1    0.39 
ref|XP_775141.1|  hypothetical protein CNBE4150 [Cryptococcus neo...  44.3    0.65 
ref|XP_571071.1|  hypothetical protein [Cryptococcus neoformans v...  43.9    0.71 
emb|CCO30621.1|  hypothetical protein BN14_04651 [Rhizoctonia sol...  41.6    4.1  
emb|CEL59390.1|  hypothetical protein RSOLAG1IB_03323 [Rhizoctoni...  41.6    4.2  
ref|XP_001876534.1|  predicted protein [Laccaria bicolor S238N-H8...  40.8    6.5  


 >emb|CED83856.1| hypothetical protein [Xanthophyllomyces dendrorhous]
Length=788

 Score = 1582 bits (4096),  Expect = 0.0, Method: Compositional matrix adjust.
 Identities = 787/788 (99%), Positives = 788/788 (100%), Gaps = 0/788 (0%)

Query  1    MTSPDITSGSPESDIHLLVQIQLKDDPPGFTLVPSTQPRYNNPKNMFPSPPVPALPSSPP  60
            MTSPDITSGSPESDIHLLVQIQLKDDPPGFTLVPSTQPRYNNPKNMFPSPPVPALPSSPP
Sbjct  1    MTSPDITSGSPESDIHLLVQIQLKDDPPGFTLVPSTQPRYNNPKNMFPSPPVPALPSSPP  60

Query  61   SLSSFLPSLPEPLFLPSPYSPAHPAFHALSSKAERYLDGQRTIFLSQIEQAIQVHRERWD  120
            SLSSFLPSLPEPLFLPSPYSPAHPAFHALSSKAERYLDGQRTIFLSQIE+AIQVHRERWD
Sbjct  61   SLSSFLPSLPEPLFLPSPYSPAHPAFHALSSKAERYLDGQRTIFLSQIERAIQVHRERWD  120

Query  121  TVQDQVKREVSEIWELFEKELGETKYKSQPDSSTTTDNTHTTASVGSTPNSPRNGQRTEG  180
            TVQDQVKREVSEIWELFEKELGETKYKSQPDSSTTTDNTHTTASVGSTPNSPRNGQRTEG
Sbjct  121  TVQDQVKREVSEIWELFEKELGETKYKSQPDSSTTTDNTHTTASVGSTPNSPRNGQRTEG  180

Query  181  FVPSKVSSSAETSPSRHPSAEPKRSKMTTHASDRTLSPASNGIGSRPVSSSSSAENLISS  240
            FVPSKVSSSAETSPSRHPSAEPKRSKMTTHASDRTLSPASNGIGSRPVSSSSSAENLISS
Sbjct  181  FVPSKVSSSAETSPSRHPSAEPKRSKMTTHASDRTLSPASNGIGSRPVSSSSSAENLISS  240

Query  241  SHQHHGPSLLGASLRNSYMPNRSIPSTSTSHSLSVLNGIAHVKSSNASSSVSSRPIASPT  300
            SHQHHGPSLLGASLRNSYMPNRSIPSTSTSHSLSVLNGIAHVKSSNASSSVSSRPIASPT
Sbjct  241  SHQHHGPSLLGASLRNSYMPNRSIPSTSTSHSLSVLNGIAHVKSSNASSSVSSRPIASPT  300

Query  301  HHSQIAACPADKDMATSLLVSNWTSPPNLIFRGSTVNQTGAHLAQGVTGVGPGVETRKVD  360
            HHSQIAACPADKDMATSLLVSNWTSPPNLIFRGSTVNQTGAHLAQGVTGVGPGVETRKVD
Sbjct  301  HHSQIAACPADKDMATSLLVSNWTSPPNLIFRGSTVNQTGAHLAQGVTGVGPGVETRKVD  360

Query  361  TAEVVSARPEDEATRGLERAMEEEKRERSEKRVKKRVAFKEADNEEEKFKSKIADKEPKD  420
            TAEVVSARPEDEATRGLERAMEEEKRERSEKRVKKRVAFKEADNEEEKFKSKIADKEPKD
Sbjct  361  TAEVVSARPEDEATRGLERAMEEEKRERSEKRVKKRVAFKEADNEEEKFKSKIADKEPKD  420

Query  421  RKRDTEEEEEDEGNGKDESDHEDVFDFEPPLPHASFVSEPSLLDYESGNPSSLSLEPLSS  480
            RKRDTEEEEEDEGNGKDESDHEDVFDFEPPLPHASFVSEPSLLDYESGNPSSLSLEPLSS
Sbjct  421  RKRDTEEEEEDEGNGKDESDHEDVFDFEPPLPHASFVSEPSLLDYESGNPSSLSLEPLSS  480

Query  481  LAPSGTGLTDEKVEEDEPMYALDGSQSLRAVTSTFGGRGDAPQEKQRIDPREVEREQKQQ  540
            LAPSGTGLTDEKVEEDEPMYALDGSQSLRAVTSTFGGRGDAPQEKQRIDPREVEREQKQQ
Sbjct  481  LAPSGTGLTDEKVEEDEPMYALDGSQSLRAVTSTFGGRGDAPQEKQRIDPREVEREQKQQ  540

Query  541  SDVDRVAASMGKLTAAYLPSHRAASRDKDKKWHLYGRLAPPPHSLADEVDSSGPKVHYGQ  600
            SDVDRVAASMGKLTAAYLPSHRAASRDKDKKWHLYGRLAPPPHSLADEVDSSGPKVHYGQ
Sbjct  541  SDVDRVAASMGKLTAAYLPSHRAASRDKDKKWHLYGRLAPPPHSLADEVDSSGPKVHYGQ  600

Query  601  SLPIAIALPSNVASQPGAPQGKELERKTSLADREMFVPPLRKAMRVASKSPSTAARRAST  660
            SLPIAIALPSNVASQPGAPQGKELERKTSLADREMFVPPLRKAMRVASKSPSTAARRAST
Sbjct  601  SLPIAIALPSNVASQPGAPQGKELERKTSLADREMFVPPLRKAMRVASKSPSTAARRAST  660

Query  661  SPDLTDHPTPFGPRRVYSHPTQVPSPIPESPQSTPPPPPPPTLAPSDDGSALITRVNAGT  720
            SPDLTDHPTPFGPRRVYSHPTQVPSPIPESPQSTPPPPPPPTLAPSDDGSALITRVNAGT
Sbjct  661  SPDLTDHPTPFGPRRVYSHPTQVPSPIPESPQSTPPPPPPPTLAPSDDGSALITRVNAGT  720

Query  721  QDRPSYLSKRTASYLSAASSLDPGPMLEQAGGDSDPESDNDEKGFIAPHLKSKAERGTDV  780
            QDRPSYLSKRTASYLSAASSLDPGPMLEQAGGDSDPESDNDEKGFIAPHLKSKAERGTDV
Sbjct  721  QDRPSYLSKRTASYLSAASSLDPGPMLEQAGGDSDPESDNDEKGFIAPHLKSKAERGTDV  780

Query  781  GWASLVDR  788
            GWASLVDR
Sbjct  781  GWASLVDR  788


>gb|KIR29002.1| hypothetical protein I309_02044 [Cryptococcus gattii LA55]
 gb|KIR93084.1| hypothetical protein I304_02747 [Cryptococcus gattii CBS 10090]
Length=774

 Score = 48.9 bits (115),  Expect = 0.020, Method: Compositional matrix adjust.
 Identities = 44/111 (40%), Positives = 57/111 (51%), Gaps = 11/111 (10%)

Query  546  VAASMGKLTAAYLPSHRAASRDKDKKWHLYGRLAPPPHS-LADEVDSSGPKVHYGQSLPI  604
            + A++    AA LPSHRAA R  ++   +Y  L     S  +D          +  S+PI
Sbjct  565  LEANLSHTFAADLPSHRAAWRRIEQNGSMYEALRRERRSDTSDNAADDSELSKFATSVPI  624

Query  605  AIA-LPSNVASQPGAPQGKELERKTSLADR-EMFVPPLRKAMR---VASKS  650
            AI  + +  A+ P A     LERKTSL DR  +FVP LR AMR   VAS S
Sbjct  625  AINPIRAGQANVPVA-----LERKTSLTDRPRIFVPGLRSAMREKGVASNS  670


>gb|KDQ20592.1| hypothetical protein BOTBODRAFT_40719 [Botryobasidium botryosum 
FD-172 SS1]
Length=697

 Score = 48.9 bits (115),  Expect = 0.024, Method: Compositional matrix adjust.
 Identities = 32/116 (28%), Positives = 53/116 (46%), Gaps = 3/116 (3%)

Query  28   PGFTLVPSTQPRYNNPKNMFPSPPVPALPSSPPSLSSFLPSLPEPLFLPSPYSPAHPAFH  87
            P FT  P    R   P  +  S   P +    P ++   P+LP P+FL  P++P+HP F 
Sbjct  128  PQFT--PRFHLRLPEPLGVVTSYSSPEVSDVAPPINPVFPTLP-PMFLSPPFTPSHPVFL  184

Query  88   ALSSKAERYLDGQRTIFLSQIEQAIQVHRERWDTVQDQVKREVSEIWELFEKELGE  143
             L+ +A    D  R    +++E   +   E     +  V+ EV  +W L+++   E
Sbjct  185  YLAKRANESSDALRIKAQAELEDWTRRKMEEIQAAESVVRNEVDLLWTLWKEAWQE  240


>gb|KGB79432.1| hypothetical protein CNBG_5270 [Cryptococcus gattii R265]
 gb|KIR73595.1| hypothetical protein I310_02267 [Cryptococcus gattii CA1014]
Length=774

 Score = 48.5 bits (114),  Expect = 0.032, Method: Compositional matrix adjust.
 Identities = 44/111 (40%), Positives = 57/111 (51%), Gaps = 11/111 (10%)

Query  546  VAASMGKLTAAYLPSHRAASRDKDKKWHLYGRLAPPPHS-LADEVDSSGPKVHYGQSLPI  604
            + A++    AA LPSHRAA R  ++   +Y  L     S  +D          +  S+PI
Sbjct  565  LEANLSHTFAADLPSHRAAWRRIEQNGSMYEALRRERRSDTSDNAADDSELSKFATSVPI  624

Query  605  AIA-LPSNVASQPGAPQGKELERKTSLADR-EMFVPPLRKAMR---VASKS  650
            AI  + +  A+ P A     LERKTSL DR  +FVP LR AMR   VAS S
Sbjct  625  AINPIRAGQANVPVA-----LERKTSLTDRPGIFVPGLRSAMREKGVASNS  670


>gb|KIR34807.1| hypothetical protein I352_03059 [Cryptococcus gattii MMRL2647]
 gb|KIR38267.1| hypothetical protein I313_05840 [Cryptococcus gattii Ram5]
 gb|KIR99652.1| hypothetical protein L804_03284 [Cryptococcus gattii 2001/935-1]
 gb|KIY58155.1| hypothetical protein I307_02403 [Cryptococcus gattii 99/473]
Length=774

 Score = 48.5 bits (114),  Expect = 0.034, Method: Compositional matrix adjust.
 Identities = 44/111 (40%), Positives = 57/111 (51%), Gaps = 11/111 (10%)

Query  546  VAASMGKLTAAYLPSHRAASRDKDKKWHLYGRLAPPPHS-LADEVDSSGPKVHYGQSLPI  604
            + A++    AA LPSHRAA R  ++   +Y  L     S  +D          +  S+PI
Sbjct  565  LEANLSHTFAADLPSHRAAWRRIEQNGSMYEALRRERRSDTSDNAADDSELSKFATSVPI  624

Query  605  AIA-LPSNVASQPGAPQGKELERKTSLADR-EMFVPPLRKAMR---VASKS  650
            AI  + +  A+ P A     LERKTSL DR  +FVP LR AMR   VAS S
Sbjct  625  AINPIRAGQANVPVA-----LERKTSLTDRPGIFVPGLRSAMREKGVASNS  670


>ref|XP_012049959.1| hypothetical protein CNAG_02121 [Cryptococcus neoformans var. 
grubii H99]
 gb|AFR95698.1| hypothetical protein CNAG_02121 [Cryptococcus neoformans var. 
grubii H99]
Length=778

 Score = 48.1 bits (113),  Expect = 0.042, Method: Compositional matrix adjust.
 Identities = 43/104 (41%), Positives = 56/104 (54%), Gaps = 10/104 (10%)

Query  546  VAASMGKLTAAYLPSHRAASRDKDKKWHLYGRL--APPPHSLADEVDSSGPKVHYGQSLP  603
            + A++    AA LPSHRAA R  ++   +Y  L     P +  + VD S     +  S+P
Sbjct  568  LEANLSHTFAADLPSHRAAWRRIEQNGSMYEALRGGRRPDTNDNPVDDS-EMSKFATSVP  626

Query  604  IAIA-LPSNVASQPGAPQGKELERKTSLADR-EMFVPPLRKAMR  645
            IAI  + +  AS P A     LERKTSL DR  +FVP LR AMR
Sbjct  627  IAINPIRAGQASVPVA-----LERKTSLTDRPGIFVPGLRPAMR  665


>gb|KIJ69811.1| hypothetical protein HYDPIDRAFT_104438 [Hydnomerulius pinastri 
MD-312]
Length=847

 Score = 48.1 bits (113),  Expect = 0.045, Method: Compositional matrix adjust.
 Identities = 29/87 (33%), Positives = 46/87 (53%), Gaps = 3/87 (3%)

Query  60   PSLS--SFLPSLPEPLFLPSPYSPAHPAFHALSSKAERYLDGQRTIFLSQIEQAIQVHRE  117
            PSLS  S L  L +PLF P+P+ P+HP F  LSS A    D  R+     +   I+    
Sbjct  182  PSLSAGSLLSDL-KPLFPPAPFVPSHPVFSHLSSIASGKSDSLRSAAEEHLAAIIRDKIA  240

Query  118  RWDTVQDQVKREVSEIWELFEKELGET  144
              +  +D+++ +V ++W  F + +GE 
Sbjct  241  ELEKAEDKLRGDVEDLWRKFIENMGEV  267


>gb|KIK96136.1| hypothetical protein PAXRUDRAFT_826294 [Paxillus rubicundulus 
Ve08.2h10]
Length=823

 Score = 47.8 bits (112),  Expect = 0.054, Method: Compositional matrix adjust.
 Identities = 26/92 (28%), Positives = 49/92 (53%), Gaps = 1/92 (1%)

Query  54   ALPSSPPSLSSFLPSLPEPLFLPSPYSPAHPAFHALSSKAERYLDGQRTIFLSQIEQAIQ  113
            +LP + PS+   L +L +PLF P+P+ P H  F  LS+ A +  +  R+     +   ++
Sbjct  169  SLPQTGPSVGPLLSNL-KPLFPPAPFVPCHLVFSHLSALAVKQSESLRSSADEHLAAIVR  227

Query  114  VHRERWDTVQDQVKREVSEIWELFEKELGETK  145
                  +  +D+++ EV E+W  F + +GE +
Sbjct  228  DKIAELERAEDKLRMEVEELWRNFVENVGEVE  259


>gb|KIR46107.1| hypothetical protein I312_04650 [Cryptococcus gattii CA1280]
Length=775

 Score = 47.4 bits (111),  Expect = 0.062, Method: Compositional matrix adjust.
 Identities = 44/111 (40%), Positives = 57/111 (51%), Gaps = 11/111 (10%)

Query  546  VAASMGKLTAAYLPSHRAASRDKDKKWHLYGRLAPPPHS-LADEVDSSGPKVHYGQSLPI  604
            + A++    AA LPSHRAA R  ++   +Y  L     S  +D          +  S+PI
Sbjct  565  LEANLSHTFAADLPSHRAAWRRIEQNGSMYEALRRERRSDTSDNAADDSELSKFATSVPI  624

Query  605  AIA-LPSNVASQPGAPQGKELERKTSLADR-EMFVPPLRKAMR---VASKS  650
            AI  + +  A+ P A     LERKTSL DR  +FVP LR AMR   VAS S
Sbjct  625  AINPIRAGQANVPVA-----LERKTSLTDRPGIFVPGLRPAMREKGVASNS  670


>ref|XP_003194412.1| hypothetical protein CGB_E5430W [Cryptococcus gattii WM276]
 gb|ADV22625.1| Hypothetical Protein CGB_E5430W [Cryptococcus gattii WM276]
 gb|KIR78386.1| hypothetical protein I306_04662 [Cryptococcus gattii EJB2]
 gb|KIY33087.1| hypothetical protein I305_04415 [Cryptococcus gattii E566]
 gb|KJE02900.1| hypothetical protein I311_03402 [Cryptococcus gattii NT-10]
Length=775

 Score = 47.4 bits (111),  Expect = 0.063, Method: Compositional matrix adjust.
 Identities = 44/111 (40%), Positives = 57/111 (51%), Gaps = 11/111 (10%)

Query  546  VAASMGKLTAAYLPSHRAASRDKDKKWHLYGRLAPPPHS-LADEVDSSGPKVHYGQSLPI  604
            + A++    AA LPSHRAA R  ++   +Y  L     S  +D          +  S+PI
Sbjct  565  LEANLSHTFAADLPSHRAAWRRIEQNGSMYEALRRERRSDTSDNAADDSELSKFATSVPI  624

Query  605  AIA-LPSNVASQPGAPQGKELERKTSLADR-EMFVPPLRKAMR---VASKS  650
            AI  + +  A+ P A     LERKTSL DR  +FVP LR AMR   VAS S
Sbjct  625  AINPIRAGQANVPVA-----LERKTSLTDRPGIFVPGLRPAMREKGVASNS  670


>gb|KIR51605.1| hypothetical protein I315_05828 [Cryptococcus gattii Ru294]
Length=776

 Score = 47.4 bits (111),  Expect = 0.063, Method: Compositional matrix adjust.
 Identities = 44/111 (40%), Positives = 57/111 (51%), Gaps = 11/111 (10%)

Query  546  VAASMGKLTAAYLPSHRAASRDKDKKWHLYGRLAPPPHS-LADEVDSSGPKVHYGQSLPI  604
            + A++    AA LPSHRAA R  ++   +Y  L     S  +D          +  S+PI
Sbjct  565  LEANLSHTFAADLPSHRAAWRRIEQNGSMYEALRRERRSDTSDNAADDSELSKFATSVPI  624

Query  605  AIA-LPSNVASQPGAPQGKELERKTSLADR-EMFVPPLRKAMR---VASKS  650
            AI  + +  A+ P A     LERKTSL DR  +FVP LR AMR   VAS S
Sbjct  625  AINPIRAGQANVPVA-----LERKTSLTDRPGIFVPGLRPAMREKGVASNS  670


>gb|KIR58865.1| hypothetical protein I314_05278 [Cryptococcus gattii CA1873]
Length=775

 Score = 47.4 bits (111),  Expect = 0.064, Method: Compositional matrix adjust.
 Identities = 44/111 (40%), Positives = 57/111 (51%), Gaps = 11/111 (10%)

Query  546  VAASMGKLTAAYLPSHRAASRDKDKKWHLYGRLAPPPHS-LADEVDSSGPKVHYGQSLPI  604
            + A++    AA LPSHRAA R  ++   +Y  L     S  +D          +  S+PI
Sbjct  565  LEANLSHTFAADLPSHRAAWRRIEQNGSMYEALRRERRSDTSDNAADDSELSKFATSVPI  624

Query  605  AIA-LPSNVASQPGAPQGKELERKTSLADR-EMFVPPLRKAMR---VASKS  650
            AI  + +  A+ P A     LERKTSL DR  +FVP LR AMR   VAS S
Sbjct  625  AINPIRAGQANVPVA-----LERKTSLTDRPGIFVPGLRPAMREKGVASNS  670


>gb|KIY42899.1| hypothetical protein FISHEDRAFT_68277 [Fistulina hepatica ATCC 
64428]
Length=793

 Score = 47.0 bits (110),  Expect = 0.096, Method: Compositional matrix adjust.
 Identities = 47/131 (36%), Positives = 58/131 (44%), Gaps = 26/131 (20%)

Query  532  EVEREQKQQSDVDRVAASMGKLTAAYLPSHRAASRDKDKKWHLYGRLAPPPHSLADEVDS  591
            E ER     +++D     + KL AA + SHR+A +     W ++ R     H  A+EVD 
Sbjct  601  EAERGDDVVAEMDPREEQVRKLLAAGVASHRSAWKRDGNAWQMFLRRRAE-HGQAEEVDE  659

Query  592  S--------GPKVHYGQSLPIAIALPSNVASQPGAPQGKELERKTSLADR-EMFVPPL--  640
                        VH G SLPIAI       S PGAP     + KTSL DR   FVP L  
Sbjct  660  EFNELDELHAESVHVG-SLPIAIR------SPPGAPSLASYQPKTSLTDRANTFVPRLPK  712

Query  641  -------RKAM  644
                   RKAM
Sbjct  713  RPTSFAYRKAM  723


>gb|KIR84974.1| hypothetical protein I308_04724 [Cryptococcus gattii IND107]
Length=774

 Score = 46.6 bits (109),  Expect = 0.10, Method: Compositional matrix adjust.
 Identities = 39/103 (38%), Positives = 53/103 (51%), Gaps = 8/103 (8%)

Query  546  VAASMGKLTAAYLPSHRAASRDKDKKWHLYGRLAPPPHS-LADEVDSSGPKVHYGQSLPI  604
            + A++ +  AA LPSHR A R  ++   +Y  L     S  +D          +  S+PI
Sbjct  565  LEANLSRTFAADLPSHRGAWRRIEQNGSMYEALRRERRSDTSDNAADDSELSKFATSVPI  624

Query  605  AIA-LPSNVASQPGAPQGKELERKTSLADR-EMFVPPLRKAMR  645
            AI  + +  A+ P A     LERKTSL DR  +FVP LR AMR
Sbjct  625  AINPIRAGQANVPVA-----LERKTSLTDRPGIFVPGLRPAMR  662


>gb|KIM29494.1| hypothetical protein M408DRAFT_114339 [Serendipita vermifera 
MAFF 305830]
Length=572

 Score = 45.8 bits (107),  Expect = 0.16, Method: Compositional matrix adjust.
 Identities = 20/66 (30%), Positives = 38/66 (58%), Gaps = 0/66 (0%)

Query  73   LFLPSPYSPAHPAFHALSSKAERYLDGQRTIFLSQIEQAIQVHRERWDTVQDQVKREVSE  132
            LF P P+ P HP F +L+ +A++    +RT+   +I Q ++  R      ++ +K+EV  
Sbjct  12   LFPPIPFVPNHPLFTSLAREAKKASTSKRTVVEEEIRQFVEQKRNEVIEAENILKQEVQT  71

Query  133  IWELFE  138
            IW+ ++
Sbjct  72   IWKAWK  77


>gb|KNZ76045.1| hypothetical protein J132_00320 [Termitomyces sp. J132]
Length=799

 Score = 45.1 bits (105),  Expect = 0.34, Method: Compositional matrix adjust.
 Identities = 27/80 (34%), Positives = 43/80 (54%), Gaps = 12/80 (15%)

Query  65   FLPSLPEPLFLPSPYSPAHPAFHALSS-----------KAERYLDGQRTIFLSQIEQAIQ  113
            F  +L +P+FLP P++P+HP F  L+S            AE Y+  Q  I +S+IE    
Sbjct  191  FFANL-QPVFLPPPFTPSHPVFSYLASHAMQESQAVRDAAEEYMTEQMKIKISEIEFTDV  249

Query  114  VHRERWDTVQDQVKREVSEI  133
              R + +T+  +V+  VS+I
Sbjct  250  ELRRKVNTLWKRVREGVSQI  269


>ref|XP_003037978.1| hypothetical protein SCHCODRAFT_102708 [Schizophyllum commune 
H4-8]
 gb|EFJ03076.1| hypothetical protein SCHCODRAFT_102708, partial [Schizophyllum 
commune H4-8]
Length=811

 Score = 45.1 bits (105),  Expect = 0.39, Method: Compositional matrix adjust.
 Identities = 35/127 (28%), Positives = 54/127 (43%), Gaps = 5/127 (4%)

Query  82   AHPAFHALSSKAERYLDGQRTIFLSQIEQAIQVHRERWDTVQDQVKREVSEIWELFEKEL  141
             HP F  LSS A    +G R      I   +Q   +    V+D +K+E  ++W+ F   +
Sbjct  205  KHPVFQHLSSLATSKSEGIRKEAEDFISNVVQTKVKEIQKVEDGLKKETEDVWKTFLGAI  264

Query  142  GETKYKSQPDSSTTTDNTHTTASVGSTPNSPRNGQRTEGFVPSKVSSSAETSPSRHPSAE  201
               K + Q   ++   +    A   S   +   G     FVPSKV     T+P R  SA 
Sbjct  265  ASVKSRQQERLTSPRSSISYPAGAFSPNGTGTPGSVIRDFVPSKV-----TAPRRSSSAA  319

Query  202  PKRSKMT  208
            P+ S ++
Sbjct  320  PRTSILS  326


>ref|XP_775141.1| hypothetical protein CNBE4150 [Cryptococcus neoformans var. neoformans 
B-3501A]
 gb|EAL20494.1| hypothetical protein CNBE4150 [Cryptococcus neoformans var. neoformans 
B-3501A]
Length=824

 Score = 44.3 bits (103),  Expect = 0.65, Method: Compositional matrix adjust.
 Identities = 41/109 (38%), Positives = 57/109 (52%), Gaps = 16/109 (15%)

Query  544  DRVAASMGKLTAAYLPSHRAASRDKDKKWHLY-----GRLAPPPHSLADEVDSSGPKVHY  598
            + + A++    AA LPSHRAA R  ++   +Y      R + P  +L D+ + S     +
Sbjct  612  NMLEANLSHTFAADLPSHRAAWRRIEQNGSMYEALRRERRSHPNDNLVDDSELS----KF  667

Query  599  GQSLPIAIA-LPSNVASQPGAPQGKELERKTSLADR-EMFVPPLRKAMR  645
              S+PIAI  + +  AS P A     LERKTSL DR  + VP L  AMR
Sbjct  668  ATSVPIAINPIRAGQASVPVA-----LERKTSLTDRPGIVVPGLGPAMR  711


>ref|XP_571071.1| hypothetical protein [Cryptococcus neoformans var. neoformans 
JEC21]
 gb|AAW43764.1| expressed protein [Cryptococcus neoformans var. neoformans JEC21]
Length=782

 Score = 43.9 bits (102),  Expect = 0.71, Method: Compositional matrix adjust.
 Identities = 41/109 (38%), Positives = 57/109 (52%), Gaps = 16/109 (15%)

Query  544  DRVAASMGKLTAAYLPSHRAASRDKDKKWHLY-----GRLAPPPHSLADEVDSSGPKVHY  598
            + + A++    AA LPSHRAA R  ++   +Y      R + P  +L D+ + S     +
Sbjct  570  NMLEANLSHTFAADLPSHRAAWRRIEQNGSMYEALRRERRSHPNDNLVDDSELS----KF  625

Query  599  GQSLPIAIA-LPSNVASQPGAPQGKELERKTSLADR-EMFVPPLRKAMR  645
              S+PIAI  + +  AS P A     LERKTSL DR  + VP L  AMR
Sbjct  626  ATSVPIAINPIRAGQASVPVA-----LERKTSLTDRPGIVVPGLGPAMR  669


>emb|CCO30621.1| hypothetical protein BN14_04651 [Rhizoctonia solani AG-1 IB]
Length=737

 Score = 41.6 bits (96),  Expect = 4.1, Method: Compositional matrix adjust.
 Identities = 37/98 (38%), Positives = 49/98 (50%), Gaps = 9/98 (9%)

Query  550  MGKLTAAYLPSHRAASRDKDKKWHLYG---RLAPPPH--SLADEVDSSGPKVHYGQSLPI  604
            + +L AA  PSHRAA R   K W L+    + A  P   S+    DS+G K  +      
Sbjct  559  LLELVAANYPSHRAAWRPNGKAWELFDARRKFADSPDSASMTSSEDSAGGK--WDNPSQF  616

Query  605  AIALPSNVASQPGAPQGKELERKTSLADR-EMFVPPLR  641
            A +LP  +A+ P A    E E KTSL ++    VPPLR
Sbjct  617  ATSLPIGIAAGPLA-SSAEREPKTSLHNKPGALVPPLR  653


>emb|CEL59390.1| hypothetical protein RSOLAG1IB_03323 [Rhizoctonia solani AG-1 
IB]
Length=737

 Score = 41.6 bits (96),  Expect = 4.2, Method: Compositional matrix adjust.
 Identities = 37/98 (38%), Positives = 49/98 (50%), Gaps = 9/98 (9%)

Query  550  MGKLTAAYLPSHRAASRDKDKKWHLYG---RLAPPPH--SLADEVDSSGPKVHYGQSLPI  604
            + +L AA  PSHRAA R   K W L+    + A  P   S+    DS+G K  +      
Sbjct  559  LLELVAANYPSHRAAWRPNGKAWELFDARRKFADSPDSASMTSSEDSAGGK--WDNPSQF  616

Query  605  AIALPSNVASQPGAPQGKELERKTSLADR-EMFVPPLR  641
            A +LP  +A+ P A    E E KTSL ++    VPPLR
Sbjct  617  ATSLPIGIAAGPLA-SSAEREPKTSLHNKPGALVPPLR  653


>ref|XP_001876534.1| predicted protein [Laccaria bicolor S238N-H82]
 gb|EDR12270.1| predicted protein [Laccaria bicolor S238N-H82]
Length=818

 Score = 40.8 bits (94),  Expect = 6.5, Method: Compositional matrix adjust.
 Identities = 18/73 (25%), Positives = 38/73 (52%), Gaps = 0/73 (0%)

Query  73   LFLPSPYSPAHPAFHALSSKAERYLDGQRTIFLSQIEQAIQVHRERWDTVQDQVKREVSE  132
            LF P P++P+H  F  L+S AE+     R     QI   ++      + + ++++++V  
Sbjct  190  LFPPPPFTPSHQTFLHLASLAEKESQAHRVAAEQQIADLVREKVADMERIDNELRQQVEV  249

Query  133  IWELFEKELGETK  145
            +W  F++ + E +
Sbjct  250  LWWKFKRSINEVQ  262


Lambda      K        H        a         alpha
   0.308    0.125    0.357    0.792     4.96 

Gapped
Lambda      K        H        a         alpha    sigma
   0.267   0.0410    0.140     1.90     42.6     43.6 

Effective search space used: 9246528513180


  Database: nr
    Posted date:  Sep 23, 2015 12:05 AM
  Number of letters in database: 26,053,659,533
  Number of sequences in database:  71,551,133


Matrix: BLOSUM62
Gap Penalties: Existence: 11, Extension: 1
Neighboring words threshold: 11
Window for multiple hits: 40
```
