# Supplementary material for: Comparative genomics provides new insights into the diversity, physiology, and sexuality of the only industrially exploited tremellomycete: Phaffia rhodozyma
Source: BMC Genomics. 2016 Nov 9;17:901. doi: 10.1186/s12864-016-3244-7 (PMC5103461; doi:10.1186/s12864-016-3244-7)
Supplement: Additional file 6: — List of orphan genes with links to PFAM (related to Additional file 1: Table S1). (ZIP 1428 kb) [file 12864_2016_3244_MOESM6_ESM.zip › BLAST_HTML_FTR/G00192_P.html]

BLAST Search Results


```
BLASTP 2.2.27+


Reference:
Stephen F. Altschul, Thomas L. Madden, Alejandro A. Schäffer,
Jinghui Zhang, Zheng Zhang, Webb Miller, and David J. Lipman (1997),
"Gapped BLAST and PSI-BLAST: a new generation of protein database
search programs", Nucleic Acids Res. 25:3389-3402.


Reference for
composition-based statistics:
Alejandro A. Schäffer, L. Aravind, Thomas L. Madden, Sergei
Shavirin, John L. Spouge, Yuri I. Wolf, Eugene V. Koonin, and
Stephen F. Altschul (2001), "Improving the accuracy of PSI-BLAST
protein database searches with composition-based statistics and
other refinements", Nucleic Acids Res. 29:2994-3005.


Database: nr
           71,551,133 sequences; 26,053,659,533 total letters


Query= G00192_P

Length=412
                                                                      Score     E
Sequences producing significant alignments:                          (Bits)  Value

emb|CED84042.1|  hypothetical protein [Xanthophyllomyces dendrorh...   374    3e-124
ref|WP_053474391.1|  translation initiation factor IF-2 [Flavobac...  40.0    4.8   


 >emb|CED84042.1| hypothetical protein [Xanthophyllomyces dendrorhous]
Length=311

 Score =  374 bits (961),  Expect = 3e-124, Method: Compositional matrix adjust.
 Identities = 266/274 (97%), Positives = 268/274 (98%), Gaps = 5/274 (2%)

Query  18   SSQTVLSANQTFSSEIIFHTQAIVRSTHIMSRSSSDSATTLVVSSTPDTVPGSSVSSTSS  77
            +SQTVLSANQTFSSE+      IVRSTHIMSRSSSDSATTLVVSSTPDTVPGSSVSSTSS
Sbjct  39   ASQTVLSANQTFSSEV-----TIVRSTHIMSRSSSDSATTLVVSSTPDTVPGSSVSSTSS  93

Query  78   TPRMPRRSQTPPPSPPSPSFPSFVSSARSIHHSDAYPFMLDREAVERRRLRKVKKLEEKL  137
            TPRMPRRSQTPPPSPPSPSFPSFVSSARSIHHSDAYPFMLDREAVERRRLRKVKKLEEKL
Sbjct  94   TPRMPRRSQTPPPSPPSPSFPSFVSSARSIHHSDAYPFMLDREAVERRRLRKVKKLEEKL  153

Query  138  AWEESCRKEREARNRREVEEWERKQQEIKAAAAVTAVATTITTISIEAPPITPTPDPPDV  197
            AWEESCRKEREARNRREVEEWERKQQEIKAAAAVTAVATTITTISIEAPPITPTPDPPDV
Sbjct  154  AWEESCRKEREARNRREVEEWERKQQEIKAAAAVTAVATTITTISIEAPPITPTPDPPDV  213

Query  198  PADEAILTPVVAIESPGPEPTPIQVQTAPSIPPSPLILPKSGSSEPVVNVNRRIEIEILD  257
            PADEAILTPVVAIESPGPEPTPIQVQTAPSIPPSPLILPKSGSSEPVVNVNRRIEIEILD
Sbjct  214  PADEAILTPVVAIESPGPEPTPIQVQTAPSIPPSPLILPKSGSSEPVVNVNRRIEIEILD  273

Query  258  SDSDSDDEIQIIESCPKPVVSSRASLPNTRLLPI  291
            SDSDSDDEIQIIESCPKPVVSSRASLPNTRLLPI
Sbjct  274  SDSDSDDEIQIIESCPKPVVSSRASLPNTRLLPI  307


>ref|WP_053474391.1| translation initiation factor IF-2 [Flavobacterium sp. VMW]
 gb|KOP35805.1| translation initiation factor IF-2 [Flavobacterium sp. VMW]
Length=956

 Score = 40.0 bits (92),  Expect = 4.8, Method: Compositional matrix adjust.
 Identities = 39/128 (30%), Positives = 62/128 (48%), Gaps = 19/128 (15%)

Query  134  EEKLAWEESCRKEREAR---NRREVEEWERKQQEIKAAAAVTAVATTITTISIEAPPITP  190
            EEK   +E+ R ERE      RR+ EE +++Q+ IKA A VT          ++   I  
Sbjct  69   EEKRKEKEALRVEREKEIEDKRRQDEERQKQQEIIKARAVVTG--------PVQVGKIDL  120

Query  191  TPDPPDVPADEAILTPVVAIE----SPGPEPTPIQVQTAPSIPPSPLILPKSGSSEPVVN  246
             P  P V  +E    PV A E    +P     P+Q +TAPS P +P++  +    +P++ 
Sbjct  121  NPKKPAVAPEE----PVKAEEPKTVTPSQTEKPVQKETAPSEPVAPVVSEEKKVEKPIIT  176

Query  247  VNRRIEIE  254
              + ++ E
Sbjct  177  EKKEVKAE  184


Lambda      K        H        a         alpha
   0.310    0.126    0.352    0.792     4.96 

Gapped
Lambda      K        H        a         alpha    sigma
   0.267   0.0410    0.140     1.90     42.6     43.6 

Effective search space used: 3912541071656


  Database: nr
    Posted date:  Sep 23, 2015 12:05 AM
  Number of letters in database: 26,053,659,533
  Number of sequences in database:  71,551,133


Matrix: BLOSUM62
Gap Penalties: Existence: 11, Extension: 1
Neighboring words threshold: 11
Window for multiple hits: 40
```
