# Supplementary material for: Comparative genomics provides new insights into the diversity, physiology, and sexuality of the only industrially exploited tremellomycete: Phaffia rhodozyma
Source: BMC Genomics. 2016 Nov 9;17:901. doi: 10.1186/s12864-016-3244-7 (PMC5103461; doi:10.1186/s12864-016-3244-7)
Supplement: Additional file 6: — List of orphan genes with links to PFAM (related to Additional file 1: Table S1). (ZIP 1428 kb) [file 12864_2016_3244_MOESM6_ESM.zip › BLAST_HTML_FTR/G01831_P.html]

BLAST Search Results


```
BLASTP 2.2.27+


Reference:
Stephen F. Altschul, Thomas L. Madden, Alejandro A. Schäffer,
Jinghui Zhang, Zheng Zhang, Webb Miller, and David J. Lipman (1997),
"Gapped BLAST and PSI-BLAST: a new generation of protein database
search programs", Nucleic Acids Res. 25:3389-3402.


Reference for
composition-based statistics:
Alejandro A. Schäffer, L. Aravind, Thomas L. Madden, Sergei
Shavirin, John L. Spouge, Yuri I. Wolf, Eugene V. Koonin, and
Stephen F. Altschul (2001), "Improving the accuracy of PSI-BLAST
protein database searches with composition-based statistics and
other refinements", Nucleic Acids Res. 29:2994-3005.


Database: nr
           71,551,133 sequences; 26,053,659,533 total letters


Query= G01831_P

Length=341
                                                                      Score     E
Sequences producing significant alignments:                          (Bits)  Value

emb|CED83623.1|  hypothetical protein [Xanthophyllomyces dendrorh...   455    3e-157
ref|WP_025335640.1|  hypothetical protein [Paenibacillus sabinae]...  42.7    0.62  


 >emb|CED83623.1| hypothetical protein [Xanthophyllomyces dendrorhous]
Length=307

 Score =  455 bits (1171),  Expect = 3e-157, Method: Compositional matrix adjust.
 Identities = 305/340 (90%), Positives = 307/340 (90%), Gaps = 33/340 (10%)

Query  1    MSIAPTQFSRHRRSKSYPPRNPPSSGITFTLSDHFTSCLTLCFTCFPSFSRRPSEESSSD  60
            MSIAPTQFSRHRRSKSYPPRNPPSSGITFTLSDHFTSCLTLCFTCFPSFSRRPSEESSSD
Sbjct  1    MSIAPTQFSRHRRSKSYPPRNPPSSGITFTLSDHFTSCLTLCFTCFPSFSRRPSEESSSD  60

Query  61   ELGFPRDTLDSLLPAFSSEHGNHRDDADDDAAAWGSDALSLRSQFGTSKRSSRERNPRGA  120
            ELGFPRDTLDSLLPAFSSEHGNHRDDADDDAAAWGSDALSLRSQFGTSKRSSRERNPRGA
Sbjct  61   ELGFPRDTLDSLLPAFSSEHGNHRDDADDDAAAWGSDALSLRSQFGTSKRSSRERNPRGA  120

Query  121  WRSWLGRLIAGRGERLSLEVEEGVDDERDFLEDPVIDWQIEDQTRMFNDNGVDRDSNVDR  180
            WRSWLGRLIAGRGERLSLEVEEGVDDERDFLEDPVIDWQIEDQTR+FNDNGVDRDSNVDR
Sbjct  121  WRSWLGRLIAGRGERLSLEVEEGVDDERDFLEDPVIDWQIEDQTRIFNDNGVDRDSNVDR  180

Query  181  RSRTIDTLPDRIFIDPTEVYQAKQRTDEEERREEGEGEGEGEAEWGAFLSAPLTRRSPNM  240
            RSRTIDTLPD                                 +WGAFLSAPLTRRSPNM
Sbjct  181  RSRTIDTLPD---------------------------------QWGAFLSAPLTRRSPNM  207

Query  241  TTSTSPSPSSSTLPTARAYPFPTSTSASTHLAEPNIPELEPSSTPGPLASISSSSSSSSA  300
            TTSTSPSPSSSTLPTARAYPFPTSTSASTHLAEPNIPELEPSSTPGPLASISSSSSSSSA
Sbjct  208  TTSTSPSPSSSTLPTARAYPFPTSTSASTHLAEPNIPELEPSSTPGPLASISSSSSSSSA  267

Query  301  SSSESKHRSSRSSKSFNKKKSPPPSNEVEPTPAEDVPPSV  340
            SSSESKHRSSRSSKSFNKKKSPPPSNEVEPTPAEDVPPSV
Sbjct  268  SSSESKHRSSRSSKSFNKKKSPPPSNEVEPTPAEDVPPSV  307


>ref|WP_025335640.1| hypothetical protein [Paenibacillus sabinae]
 gb|AHV98143.1| lpxtg-motif cell wall anchor domain-containing protein [Paenibacillus 
sabinae T27]
Length=1201

 Score = 42.7 bits (99),  Expect = 0.62, Method: Composition-based stats.
 Identities = 23/61 (38%), Positives = 35/61 (57%), Gaps = 5/61 (8%)

Query  243   STSPSPSSSTLPTARAYPFPTSTSASTHLAEPNIPELEPSSTPGPLASISSSSSSSSASS  302
             S SP+PS++  P   A P P+ T +    A P      PS+TP P+AS++ S+  S+A S
Sbjct  1015  SASPTPSATPSPVVSASPVPSVTPSPVVSASPT-----PSATPSPVASVTPSAEVSAAPS  1069

Query  303   S  303
             +
Sbjct  1070  A  1070


Lambda      K        H        a         alpha
   0.310    0.126    0.371    0.792     4.96 

Gapped
Lambda      K        H        a         alpha    sigma
   0.267   0.0410    0.140     1.90     42.6     43.6 

Effective search space used: 2897393305500


  Database: nr
    Posted date:  Sep 23, 2015 12:05 AM
  Number of letters in database: 26,053,659,533
  Number of sequences in database:  71,551,133


Matrix: BLOSUM62
Gap Penalties: Existence: 11, Extension: 1
Neighboring words threshold: 11
Window for multiple hits: 40
```
