# Supplementary material for: Comparative genomics provides new insights into the diversity, physiology, and sexuality of the only industrially exploited tremellomycete: Phaffia rhodozyma
Source: BMC Genomics. 2016 Nov 9;17:901. doi: 10.1186/s12864-016-3244-7 (PMC5103461; doi:10.1186/s12864-016-3244-7)
Supplement: Additional file 6: — List of orphan genes with links to PFAM (related to Additional file 1: Table S1). (ZIP 1428 kb) [file 12864_2016_3244_MOESM6_ESM.zip › BLAST_HTML_FTR/G05502_P.html]

BLAST Search Results


```
BLASTP 2.2.27+


Reference:
Stephen F. Altschul, Thomas L. Madden, Alejandro A. Schäffer,
Jinghui Zhang, Zheng Zhang, Webb Miller, and David J. Lipman (1997),
"Gapped BLAST and PSI-BLAST: a new generation of protein database
search programs", Nucleic Acids Res. 25:3389-3402.


Reference for
composition-based statistics:
Alejandro A. Schäffer, L. Aravind, Thomas L. Madden, Sergei
Shavirin, John L. Spouge, Yuri I. Wolf, Eugene V. Koonin, and
Stephen F. Altschul (2001), "Improving the accuracy of PSI-BLAST
protein database searches with composition-based statistics and
other refinements", Nucleic Acids Res. 29:2994-3005.


Database: nr
           71,551,133 sequences; 26,053,659,533 total letters


Query= G05502_P

Length=167
                                                                      Score     E
Sequences producing significant alignments:                          (Bits)  Value

emb|CED83395.1|  hypothetical protein [Xanthophyllomyces dendrorh...   325    4e-111
emb|CDZ97547.1|  hypothetical protein [Xanthophyllomyces dendrorh...  45.4    0.006 
ref|WP_051502066.1|  hypothetical protein [Lactobacillus fabiferm...  38.9    1.2   
ref|WP_024626020.1|  hypothetical protein [Lactobacillus fabiferm...  38.1    2.4   


 >emb|CED83395.1| hypothetical protein [Xanthophyllomyces dendrorhous]
Length=166

 Score =  325 bits (833),  Expect = 4e-111, Method: Compositional matrix adjust.
 Identities = 166/166 (100%), Positives = 166/166 (100%), Gaps = 0/166 (0%)

Query  1    MLTLSICCIVLLPGGAICAVQAAPTGTTNENGKRFALPSGSVCSYQCPTLSSIPDDSDQR  60
            MLTLSICCIVLLPGGAICAVQAAPTGTTNENGKRFALPSGSVCSYQCPTLSSIPDDSDQR
Sbjct  1    MLTLSICCIVLLPGGAICAVQAAPTGTTNENGKRFALPSGSVCSYQCPTLSSIPDDSDQR  60

Query  61   TSYPPDGITGAPYGTTGTTCTYNYKRTVPAACLYTRSTSVLNSSSSSNLACPSTLTSSGD  120
            TSYPPDGITGAPYGTTGTTCTYNYKRTVPAACLYTRSTSVLNSSSSSNLACPSTLTSSGD
Sbjct  61   TSYPPDGITGAPYGTTGTTCTYNYKRTVPAACLYTRSTSVLNSSSSSNLACPSTLTSSGD  120

Query  121  CPASATYSGTPVKLLSWTATTKYKTCNFPGDTYDASGTCSYTNSNG  166
            CPASATYSGTPVKLLSWTATTKYKTCNFPGDTYDASGTCSYTNSNG
Sbjct  121  CPASATYSGTPVKLLSWTATTKYKTCNFPGDTYDASGTCSYTNSNG  166


>emb|CDZ97547.1| hypothetical protein [Xanthophyllomyces dendrorhous]
Length=223

 Score = 45.4 bits (106),  Expect = 0.006, Method: Compositional matrix adjust.
 Identities = 57/168 (34%), Positives = 74/168 (44%), Gaps = 19/168 (11%)

Query  6    ICCIVLLPGGAICAVQAAPTGTTNENGKRFALPSGSV--CSYQCPTLSSIPDDSDQRTSY  63
            +  +VLL   A     A    +  E     A P  S   C Y CPT S   D S  R   
Sbjct  8    LASVVLLSAAA----SAQNHSSVLEKRDTAAQPQASTYSCEYTCPTWSYQDDIS--RVVL  61

Query  64   PPDGITGAPYGTTGTTCTYNYKRTVPAA-CLY-TRSTSVLNSSSSSNLACPSTLTSSGDC  121
             P     + Y T    C Y ++RT  A  C Y T   SV N S  + L+CPST TS+GDC
Sbjct  62   SPS--YSSMYDTKTMLCGYGWQRTEDAGTCRYNTAGQSVYNKSDKTALSCPST-TSNGDC  118

Query  122  PASATYSGTPVKL--LSWTATTKYKTCNFPGD-TYDASGTCSYTNSNG  166
            P+   +     KL     T++T+Y  C++  D TY A   C Y  + G
Sbjct  119  PSPVAFGDASTKLSYKESTSSTEY-LCHYTIDKTYAAK--CFYNKAKG  163


>ref|WP_051502066.1| hypothetical protein [Lactobacillus fabifermentans]
 gb|ETY73669.1| hypothetical protein LFAB_11110 [Lactobacillus fabifermentans 
T30PCM01]
Length=195

 Score = 38.9 bits (89),  Expect = 1.2, Method: Compositional matrix adjust.
 Identities = 29/91 (32%), Positives = 43/91 (47%), Gaps = 6/91 (7%)

Query  1   MLTLSICCIVLLPGGAICAVQAAPTGTTNENGKRFALPSGSVCSYQCPTLSSIPDD----  56
           +L L+   +  L GG + A QAA TGT   +   F + SG +     PTL+    D    
Sbjct  8   ILVLATVALATLVGGTLAA-QAADTGTDGTSVGEFTVASGKLSLDAVPTLAFKGTDVASL  66

Query  57  -SDQRTSYPPDGITGAPYGTTGTTCTYNYKR  86
            S  + +Y  D +TG+   +TG T T +  R
Sbjct  67  ASGTKLAYNSDAVTGSGKTSTGNTLTVSDFR  97


>ref|WP_024626020.1| hypothetical protein [Lactobacillus fabifermentans]
Length=195

 Score = 38.1 bits (87),  Expect = 2.4, Method: Compositional matrix adjust.
 Identities = 29/91 (32%), Positives = 43/91 (47%), Gaps = 6/91 (7%)

Query  1   MLTLSICCIVLLPGGAICAVQAAPTGTTNENGKRFALPSGSVCSYQCPTLSSIPDD----  56
           +L L+   +  L GG + A QAA TGT   +   F + SG +     PTL+    D    
Sbjct  8   ILGLATVALATLVGGTLAA-QAADTGTDGTSVGEFTVASGKLSLDAVPTLAFKGTDVASL  66

Query  57  -SDQRTSYPPDGITGAPYGTTGTTCTYNYKR  86
            S  + +Y  D +TG+   +TG T T +  R
Sbjct  67  ASGTKLAYNSDAVTGSGKTSTGNTLTVSDFR  97


Lambda      K        H        a         alpha
   0.316    0.129    0.408    0.792     4.96 

Gapped
Lambda      K        H        a         alpha    sigma
   0.267   0.0410    0.140     1.90     42.6     43.6 

Effective search space used: 639295408288


  Database: nr
    Posted date:  Sep 23, 2015 12:05 AM
  Number of letters in database: 26,053,659,533
  Number of sequences in database:  71,551,133


Matrix: BLOSUM62
Gap Penalties: Existence: 11, Extension: 1
Neighboring words threshold: 11
Window for multiple hits: 40
```
