# Supplementary material for: Comparative genomics provides new insights into the diversity, physiology, and sexuality of the only industrially exploited tremellomycete: Phaffia rhodozyma
Source: BMC Genomics. 2016 Nov 9;17:901. doi: 10.1186/s12864-016-3244-7 (PMC5103461; doi:10.1186/s12864-016-3244-7)
Supplement: Additional file 6: — List of orphan genes with links to PFAM (related to Additional file 1: Table S1). (ZIP 1428 kb) [file 12864_2016_3244_MOESM6_ESM.zip › BLAST_HTML_FTR/G01819_P.html]

BLAST Search Results


```
BLASTP 2.2.27+


Reference:
Stephen F. Altschul, Thomas L. Madden, Alejandro A. Schäffer,
Jinghui Zhang, Zheng Zhang, Webb Miller, and David J. Lipman (1997),
"Gapped BLAST and PSI-BLAST: a new generation of protein database
search programs", Nucleic Acids Res. 25:3389-3402.


Reference for
composition-based statistics:
Alejandro A. Schäffer, L. Aravind, Thomas L. Madden, Sergei
Shavirin, John L. Spouge, Yuri I. Wolf, Eugene V. Koonin, and
Stephen F. Altschul (2001), "Improving the accuracy of PSI-BLAST
protein database searches with composition-based statistics and
other refinements", Nucleic Acids Res. 29:2994-3005.


Database: nr
           71,551,133 sequences; 26,053,659,533 total letters


Query= G01819_P

Length=973
                                                                      Score     E
Sequences producing significant alignments:                          (Bits)  Value

emb|CED83635.1|  hypothetical protein [Xanthophyllomyces dendrorh...  1776    0.0  


 >emb|CED83635.1| hypothetical protein [Xanthophyllomyces dendrorhous]
Length=931

 Score = 1776 bits (4600),  Expect = 0.0, Method: Compositional matrix adjust.
 Identities = 925/947 (98%), Positives = 925/947 (98%), Gaps = 22/947 (2%)

Query  26   TASQAAYRAGSQRIPIETRSIHSLSVSRQSLSGHLPQTILNTCSSLQASYQQSNQSASYA  85
            TASQAAYRAGSQRIPIETRSIHSLS                      ASYQQSNQSASYA
Sbjct  7    TASQAAYRAGSQRIPIETRSIHSLS----------------------ASYQQSNQSASYA  44

Query  86   PSDKILSKDSARKEPNFDAFLSLRNATPRKSRQKKPTSWTPNHGNRSEKRSTSSSKPYNS  145
            PSDKILSKDSARKEPNFDAFLSLRNATPRKSRQKKPTSWTPNHGNRSEKRSTSSSKPYNS
Sbjct  45   PSDKILSKDSARKEPNFDAFLSLRNATPRKSRQKKPTSWTPNHGNRSEKRSTSSSKPYNS  104

Query  146  EETASAIPTTWNRSQPWSRPRPPINASKPTPPIQLSQPETISSVPTIESYTPSFSFSSPT  205
            EETASAIPTTWNRSQPWSRPRPPINASKPTPPIQLSQPETISSVPTIESYTPSFSFSSPT
Sbjct  105  EETASAIPTTWNRSQPWSRPRPPINASKPTPPIQLSQPETISSVPTIESYTPSFSFSSPT  164

Query  206  APVSTPRSSPSISKHDLLALMQHARPSSSSSPAQPKLPLTFHIPTDLLEAVENQKQGDSK  265
            APVSTPRSSPSISKHDLLALMQHARPSSSSSPAQPKLPLTFHIPTDLLEAVENQKQGDSK
Sbjct  165  APVSTPRSSPSISKHDLLALMQHARPSSSSSPAQPKLPLTFHIPTDLLEAVENQKQGDSK  224

Query  266  NLLESLLVSVMSRSHQAQQVMTPGSTSPRQTADGFSRSSTEQDNAHPIRTADIDRLILRI  325
            NLLESLLVSVMSRSHQAQQVMTPGSTSPRQTADGFSRSSTEQDNAHPIRTADIDRLILRI
Sbjct  225  NLLESLLVSVMSRSHQAQQVMTPGSTSPRQTADGFSRSSTEQDNAHPIRTADIDRLILRI  284

Query  326  LSPAYENPANISLLLNLLPMLLSFAPERSRYMSPKTIAFLLFNSSDPIKGFEYLDSQCQP  385
            LSPAYENPANISLLLNLLPMLLSFAPERSRYMSPKTIAFLLFNSSDPIKGFEYLDSQCQP
Sbjct  285  LSPAYENPANISLLLNLLPMLLSFAPERSRYMSPKTIAFLLFNSSDPIKGFEYLDSQCQP  344

Query  386  EDQAEMFSPTDLGLIWKAWVGSTQPYQALQHAQTRLRAMGLSANEETFDDHILGILLRYP  445
            EDQAEMFSPTDLGLIWKAWVGSTQPYQALQHAQTRLRAMGLSANEETFDDHILGILLRYP
Sbjct  345  EDQAEMFSPTDLGLIWKAWVGSTQPYQALQHAQTRLRAMGLSANEETFDDHILGILLRYP  404

Query  446  AVHHLETVVRNLPSDFQKASRGQPTEFIVTMQDVLVRLFDLAEIAEDKEFLYWISQQICQ  505
            AVHHLETVVRNLPSDFQKASRGQPTEFIVTMQDVLVRLFDLAEIAEDKEFLYWISQQICQ
Sbjct  405  AVHHLETVVRNLPSDFQKASRGQPTEFIVTMQDVLVRLFDLAEIAEDKEFLYWISQQICQ  464

Query  506  LSRVEPIKKQAQSVCQQIQAAAEVVVLDFVERWDLPGMRTQISNLTRYLDPLDSIIIPPR  565
            LSRVEPIKKQAQSVCQQIQAAAEVVVLDFVERWDLPGMRTQISNLTRYLDPLDSIIIPPR
Sbjct  465  LSRVEPIKKQAQSVCQQIQAAAEVVVLDFVERWDLPGMRTQISNLTRYLDPLDSIIIPPR  524

Query  566  WTEQNASKDDTVSGCSSCTPVDLDILDRISRTWADDRKGRLWKALVKGSALEDGQSWKSM  625
            WTEQNASKDDTVSGCSSCTPVDLDILDRISRTWADDRKGRLWKALVKGSALEDGQSWKSM
Sbjct  525  WTEQNASKDDTVSGCSSCTPVDLDILDRISRTWADDRKGRLWKALVKGSALEDGQSWKSM  584

Query  626  FQIYLLGSKGIHHELQRPDEMIFPVIDTLGKNLGRYTGDQKKKFAREVLVDKAFIHTLMA  685
            FQIYLLGSKGIHHELQRPDEMIFPVIDTLGKNLGRYTGDQKKKFAREVLVDKAFIHTLMA
Sbjct  585  FQIYLLGSKGIHHELQRPDEMIFPVIDTLGKNLGRYTGDQKKKFAREVLVDKAFIHTLMA  644

Query  686  TPAFRLTPSDSTGISISTPAIVPAALSDQAGRIIRTILEPFLRTPVLPRTENKLYDALLE  745
            TPAFRLTPSDSTGISISTPAIVPAALSDQAGRIIRTILEPFLRTPVLPRTENKLYDALLE
Sbjct  645  TPAFRLTPSDSTGISISTPAIVPAALSDQAGRIIRTILEPFLRTPVLPRTENKLYDALLE  704

Query  746  SLSHLTTYQPVLGLGYSKDALRHLLTLAPSPALMVRTIRLFLLFRPHQTASSATTAAAND  805
            SLSHLTTYQPVLGLGYSKDALRHLLTLAPSPALMVRTIRLFLLFRPHQTASSATTAAAND
Sbjct  705  SLSHLTTYQPVLGLGYSKDALRHLLTLAPSPALMVRTIRLFLLFRPHQTASSATTAAAND  764

Query  806  EHGWSRSILSPELAAETANSILSRLYSTPDVSQPGPILDLVDLSNEIVGVDTDVETKLSW  865
            EHGWSRSILSPELAAETANSILSRLYSTPDVSQPGPILDLVDLSNEIVGVDTDVETKLSW
Sbjct  765  EHGWSRSILSPELAAETANSILSRLYSTPDVSQPGPILDLVDLSNEIVGVDTDVETKLSW  824

Query  866  LRRLLRRYIEILTLLDLPAANPTSNEGPSLDRRENKRPGSKGTVNKAYRAHQLGEIQNAI  925
            LRRLLRRYIEILTLLDLPAANPTSNEGPSLDRRENKRPGSKGTVNKAYRAHQLGEIQNAI
Sbjct  825  LRRLLRRYIEILTLLDLPAANPTSNEGPSLDRRENKRPGSKGTVNKAYRAHQLGEIQNAI  884

Query  926  IKVERWAADRPGLFAPSSSTSTVKSSGDVRGLIAQIKLKSDRASRLV  972
            IKVERWAADRPGLFAPSSSTSTVKSSGDVRGLIAQIKLKSDRASRLV
Sbjct  885  IKVERWAADRPGLFAPSSSTSTVKSSGDVRGLIAQIKLKSDRASRLV  931


Lambda      K        H        a         alpha
   0.316    0.130    0.378    0.792     4.96 

Gapped
Lambda      K        H        a         alpha    sigma
   0.267   0.0410    0.140     1.90     42.6     43.6 

Effective search space used: 11801548821440


  Database: nr
    Posted date:  Sep 23, 2015 12:05 AM
  Number of letters in database: 26,053,659,533
  Number of sequences in database:  71,551,133


Matrix: BLOSUM62
Gap Penalties: Existence: 11, Extension: 1
Neighboring words threshold: 11
Window for multiple hits: 40
```
