# Supplementary material for: Comparative genomics provides new insights into the diversity, physiology, and sexuality of the only industrially exploited tremellomycete: Phaffia rhodozyma
Source: BMC Genomics. 2016 Nov 9;17:901. doi: 10.1186/s12864-016-3244-7 (PMC5103461; doi:10.1186/s12864-016-3244-7)
Supplement: Additional file 6: — List of orphan genes with links to PFAM (related to Additional file 1: Table S1). (ZIP 1428 kb) [file 12864_2016_3244_MOESM6_ESM.zip › BLAST_HTML_FTR/G04588_P.html]

BLAST Search Results


```
BLASTP 2.2.27+


Reference:
Stephen F. Altschul, Thomas L. Madden, Alejandro A. Schäffer,
Jinghui Zhang, Zheng Zhang, Webb Miller, and David J. Lipman (1997),
"Gapped BLAST and PSI-BLAST: a new generation of protein database
search programs", Nucleic Acids Res. 25:3389-3402.


Reference for
composition-based statistics:
Alejandro A. Schäffer, L. Aravind, Thomas L. Madden, Sergei
Shavirin, John L. Spouge, Yuri I. Wolf, Eugene V. Koonin, and
Stephen F. Altschul (2001), "Improving the accuracy of PSI-BLAST
protein database searches with composition-based statistics and
other refinements", Nucleic Acids Res. 29:2994-3005.


Database: nr
           71,551,133 sequences; 26,053,659,533 total letters


Query= G04588_P

Length=219
                                                                      Score     E
Sequences producing significant alignments:                          (Bits)  Value

emb|CED83906.1|  hypothetical protein [Xanthophyllomyces dendrorh...   270    9e-89
ref|XP_004385310.1|  PREDICTED: A disintegrin and metalloproteina...  38.5    4.8  


 >emb|CED83906.1| hypothetical protein [Xanthophyllomyces dendrorhous]
Length=131

 Score =  270 bits (689),  Expect = 9e-89, Method: Compositional matrix adjust.
 Identities = 131/131 (100%), Positives = 131/131 (100%), Gaps = 0/131 (0%)

Query  88   MSLNKSSPYTCPSKLVQIHSDYSRNSDSANEAFSMYRSSARARSNSLSSNAESFVSNGGS  147
            MSLNKSSPYTCPSKLVQIHSDYSRNSDSANEAFSMYRSSARARSNSLSSNAESFVSNGGS
Sbjct  1    MSLNKSSPYTCPSKLVQIHSDYSRNSDSANEAFSMYRSSARARSNSLSSNAESFVSNGGS  60

Query  148  VSSSNSAAAPFVWMTVLRSRPGLAEELTTFLAGASQRILEKEPGVLFHRTLRCVDDEDDD  207
            VSSSNSAAAPFVWMTVLRSRPGLAEELTTFLAGASQRILEKEPGVLFHRTLRCVDDEDDD
Sbjct  61   VSSSNSAAAPFVWMTVLRSRPGLAEELTTFLAGASQRILEKEPGVLFHRTLRCVDDEDDD  120

Query  208  VVGTKFLAYAE  218
            VVGTKFLAYAE
Sbjct  121  VVGTKFLAYAE  131


>ref|XP_004385310.1| PREDICTED: A disintegrin and metalloproteinase with thrombospondin 
motifs 12 [Trichechus manatus latirostris]
Length=1466

 Score = 38.5 bits (88),  Expect = 4.8, Method: Composition-based stats.
 Identities = 43/171 (25%), Positives = 75/171 (44%), Gaps = 19/171 (11%)

Query  1    MTCSRLNRLRGISCKEAGIDPNSLEDIKKSRMMRAIVHVQHSSVVL------VFATAALP  54
            ++C    R+R ++C +   +P  +     SR +  +     +  VL      +F    LP
Sbjct  826  VSCGGGVRIRSVTCAKNNDEPCDVTKKPNSRALCGLQQCPSTRRVLKPNRGMIFNRKNLP  885

Query  55   AEATSSTKETRPM-----SYEMLSHLTIFDLFNHHTVQMSLNKSSPYTCPSKLVQIHSDY  109
               TS     +P+     S  M++  T+ +  N  T+ +++   SP T       +    
Sbjct  886  ---TSEKDPVKPIPSPTSSPRMVTTPTVLEPMN--TIAVTVKSPSPTTTSQG--NLDGKQ  938

Query  110  SRNSDSANEAFSMYRSSARARSN-SLSSNAESFVSNGGSVSSSNSAAAPFV  159
             +NS + +E  S Y SSA   S  +L+S + S   N G+VSSS++   P V
Sbjct  939  WQNSSTQSELDSHYLSSAGITSQPTLTSWSLSIEPNEGNVSSSDTVPTPEV  989


Lambda      K        H        a         alpha
   0.318    0.127    0.361    0.792     4.96 

Gapped
Lambda      K        H        a         alpha    sigma
   0.267   0.0410    0.140     1.90     42.6     43.6 

Effective search space used: 1181272228575


  Database: nr
    Posted date:  Sep 23, 2015 12:05 AM
  Number of letters in database: 26,053,659,533
  Number of sequences in database:  71,551,133


Matrix: BLOSUM62
Gap Penalties: Existence: 11, Extension: 1
Neighboring words threshold: 11
Window for multiple hits: 40
```
