# Supplementary material for: Comparative genomics provides new insights into the diversity, physiology, and sexuality of the only industrially exploited tremellomycete: Phaffia rhodozyma
Source: BMC Genomics. 2016 Nov 9;17:901. doi: 10.1186/s12864-016-3244-7 (PMC5103461; doi:10.1186/s12864-016-3244-7)
Supplement: Additional file 6: — List of orphan genes with links to PFAM (related to Additional file 1: Table S1). (ZIP 1428 kb) [file 12864_2016_3244_MOESM6_ESM.zip › BLAST_HTML_FTR/G00168_P.html]

BLAST Search Results


```
BLASTP 2.2.27+


Reference:
Stephen F. Altschul, Thomas L. Madden, Alejandro A. Schäffer,
Jinghui Zhang, Zheng Zhang, Webb Miller, and David J. Lipman (1997),
"Gapped BLAST and PSI-BLAST: a new generation of protein database
search programs", Nucleic Acids Res. 25:3389-3402.


Reference for
composition-based statistics:
Alejandro A. Schäffer, L. Aravind, Thomas L. Madden, Sergei
Shavirin, John L. Spouge, Yuri I. Wolf, Eugene V. Koonin, and
Stephen F. Altschul (2001), "Improving the accuracy of PSI-BLAST
protein database searches with composition-based statistics and
other refinements", Nucleic Acids Res. 29:2994-3005.


Database: nr
           71,551,133 sequences; 26,053,659,533 total letters


Query= G00168_P

Length=453
                                                                      Score     E
Sequences producing significant alignments:                          (Bits)  Value

emb|CED84017.1|  hypothetical protein [Xanthophyllomyces dendrorh...   896    0.0  
ref|XP_013097908.1|  PREDICTED: DNA-directed RNA polymerase II su...  42.7    1.0  


 >emb|CED84017.1| hypothetical protein [Xanthophyllomyces dendrorhous]
Length=452

 Score =  896 bits (2315),  Expect = 0.0, Method: Compositional matrix adjust.
 Identities = 452/452 (100%), Positives = 452/452 (100%), Gaps = 0/452 (0%)

Query  1    MRHSSFSSDSSSSSHTSSSGSSSAGSSYDLPTPPRSTSPLAPPDIRRGSSQSTNSTHSLG  60
            MRHSSFSSDSSSSSHTSSSGSSSAGSSYDLPTPPRSTSPLAPPDIRRGSSQSTNSTHSLG
Sbjct  1    MRHSSFSSDSSSSSHTSSSGSSSAGSSYDLPTPPRSTSPLAPPDIRRGSSQSTNSTHSLG  60

Query  61   SSSNFLGWQPSSQHHQHSSTKTSSQTQEPITPPWSPDCPLGAFAVESPADRPSSSTHRMN  120
            SSSNFLGWQPSSQHHQHSSTKTSSQTQEPITPPWSPDCPLGAFAVESPADRPSSSTHRMN
Sbjct  61   SSSNFLGWQPSSQHHQHSSTKTSSQTQEPITPPWSPDCPLGAFAVESPADRPSSSTHRMN  120

Query  121  PLGDRLHPAQYGPNGSTSLWQSQRLFGLDSFPTNAPSSTLDDDDDDDDSRSGTDSVTTYS  180
            PLGDRLHPAQYGPNGSTSLWQSQRLFGLDSFPTNAPSSTLDDDDDDDDSRSGTDSVTTYS
Sbjct  121  PLGDRLHPAQYGPNGSTSLWQSQRLFGLDSFPTNAPSSTLDDDDDDDDSRSGTDSVTTYS  180

Query  181  ARSSLDSRTSLEDQDQDTLSPCPPVCVVSEVPSLSSSGLSSPSSPPPQPPSHPPSLSLEP  240
            ARSSLDSRTSLEDQDQDTLSPCPPVCVVSEVPSLSSSGLSSPSSPPPQPPSHPPSLSLEP
Sbjct  181  ARSSLDSRTSLEDQDQDTLSPCPPVCVVSEVPSLSSSGLSSPSSPPPQPPSHPPSLSLEP  240

Query  241  LLSSQLTYALSNPLLHGDILPKENVSLSALSPSHLSSVRSYHHHHHHHHHRRHWSYRSPS  300
            LLSSQLTYALSNPLLHGDILPKENVSLSALSPSHLSSVRSYHHHHHHHHHRRHWSYRSPS
Sbjct  241  LLSSQLTYALSNPLLHGDILPKENVSLSALSPSHLSSVRSYHHHHHHHHHRRHWSYRSPS  300

Query  301  SHFAPRLPLSLKLVHTAFHILIPCLTFFILGSIALWAAGTVYYYLGLWIFGFWSDAYKHC  360
            SHFAPRLPLSLKLVHTAFHILIPCLTFFILGSIALWAAGTVYYYLGLWIFGFWSDAYKHC
Sbjct  301  SHFAPRLPLSLKLVHTAFHILIPCLTFFILGSIALWAAGTVYYYLGLWIFGFWSDAYKHC  360

Query  361  FWVMVGLACLGFAYYGFWLVVKIGSGLADTWDIDLHALDDFDWVDSFVTVDPRSVNPQKE  420
            FWVMVGLACLGFAYYGFWLVVKIGSGLADTWDIDLHALDDFDWVDSFVTVDPRSVNPQKE
Sbjct  361  FWVMVGLACLGFAYYGFWLVVKIGSGLADTWDIDLHALDDFDWVDSFVTVDPRSVNPQKE  420

Query  421  QDLELGVSYRNRSASTSRAWSFETERVRETSV  452
            QDLELGVSYRNRSASTSRAWSFETERVRETSV
Sbjct  421  QDLELGVSYRNRSASTSRAWSFETERVRETSV  452


>ref|XP_013097908.1| PREDICTED: DNA-directed RNA polymerase II subunit RPB1 [Stomoxys 
calcitrans]
Length=1892

 Score = 42.7 bits (99),  Expect = 1.0, Method: Composition-based stats.
 Identities = 33/116 (28%), Positives = 53/116 (46%), Gaps = 4/116 (3%)

Query  6     FSSDSSSSSHTSSSGSSSAGSSYDLPTPP--RSTSPL-APPDIRRGSSQSTNSTHSLGSS  62
             +   + S  +T  S   S  S    PT P    TSP  +P +    +  S ++T    S 
Sbjct  1772  YDGSAGSPQYTPGSPQYSPASPKYSPTSPLYSPTSPQHSPANQYSPTGSSYSATSPRYSP  1831

Query  63    SNFLGWQP-SSQHHQHSSTKTSSQTQEPITPPWSPDCPLGAFAVESPADRPSSSTH  117
             +N   + P S+++   S T T ++   P +P +SP  P   ++  SPA  PSS T+
Sbjct  1832  NNMSVYSPGSTKYSPTSPTYTPTRNYSPASPMYSPTAPSHGYSPTSPAYSPSSPTY  1887


Lambda      K        H        a         alpha
   0.315    0.129    0.408    0.792     4.96 

Gapped
Lambda      K        H        a         alpha    sigma
   0.267   0.0410    0.140     1.90     42.6     43.6 

Effective search space used: 4495400730249


  Database: nr
    Posted date:  Sep 23, 2015 12:05 AM
  Number of letters in database: 26,053,659,533
  Number of sequences in database:  71,551,133


Matrix: BLOSUM62
Gap Penalties: Existence: 11, Extension: 1
Neighboring words threshold: 11
Window for multiple hits: 40
```
