# Supplementary material for: Comparative genomics provides new insights into the diversity, physiology, and sexuality of the only industrially exploited tremellomycete: Phaffia rhodozyma
Source: BMC Genomics. 2016 Nov 9;17:901. doi: 10.1186/s12864-016-3244-7 (PMC5103461; doi:10.1186/s12864-016-3244-7)
Supplement: Additional file 6: — List of orphan genes with links to PFAM (related to Additional file 1: Table S1). (ZIP 1428 kb) [file 12864_2016_3244_MOESM6_ESM.zip › BLAST_HTML_FTR/G00592_P.html]

BLAST Search Results


```
BLASTP 2.2.27+


Reference:
Stephen F. Altschul, Thomas L. Madden, Alejandro A. Schäffer,
Jinghui Zhang, Zheng Zhang, Webb Miller, and David J. Lipman (1997),
"Gapped BLAST and PSI-BLAST: a new generation of protein database
search programs", Nucleic Acids Res. 25:3389-3402.


Reference for
composition-based statistics:
Alejandro A. Schäffer, L. Aravind, Thomas L. Madden, Sergei
Shavirin, John L. Spouge, Yuri I. Wolf, Eugene V. Koonin, and
Stephen F. Altschul (2001), "Improving the accuracy of PSI-BLAST
protein database searches with composition-based statistics and
other refinements", Nucleic Acids Res. 29:2994-3005.


Database: nr
           71,551,133 sequences; 26,053,659,533 total letters


Query= G00592_P

Length=171
                                                                      Score     E
Sequences producing significant alignments:                          (Bits)  Value

emb|CED82581.1|  hypothetical protein [Xanthophyllomyces dendrorh...   302    2e-101
ref|XP_006466139.1|  PREDICTED: 3-oxoacyl-[acyl-carrier-protein] ...  37.4    3.5   


 >emb|CED82581.1| hypothetical protein [Xanthophyllomyces dendrorhous]
Length=203

 Score =  302 bits (774),  Expect = 2e-101, Method: Compositional matrix adjust.
 Identities = 156/189 (83%), Positives = 156/189 (83%), Gaps = 33/189 (17%)

Query  15   CLASPPATSATFSSTSLIIINNILDYPTGLVGAARHGRLSEYLPDVCRSPCSNYTDWEA-  73
            CLASPPATSATFSSTSLIIINNILDYPTGLVGAARHGRLSEYLPDVCRSPCSNYTDWEA 
Sbjct  15   CLASPPATSATFSSTSLIIINNILDYPTGLVGAARHGRLSEYLPDVCRSPCSNYTDWEAS  74

Query  74   --------------------------------VKDPTLFSPRRYVSLETQSNIIQMSCRG  101
                                            VKDPTLFSPRRYVSLETQSNIIQMSCRG
Sbjct  75   CLVDMSHSECGLKFCTRPMALSYYSCVNCMLYVKDPTLFSPRRYVSLETQSNIIQMSCRG  134

Query  102  FGRSTGPLIELASYLQIGMNTNNVTDFKPPDVRAIAVTVSSARSEWGSRSRSRLGMVGIG  161
            FGRSTGPLIELASYLQIGMNTNNVTDFKPPDVRAIAVTVSSARSEWGSRSRSRLGMVGIG
Sbjct  135  FGRSTGPLIELASYLQIGMNTNNVTDFKPPDVRAIAVTVSSARSEWGSRSRSRLGMVGIG  194

Query  162  IGVLGWSLL  170
            IGVLGWSLL
Sbjct  195  IGVLGWSLL  203


>ref|XP_006466139.1| PREDICTED: 3-oxoacyl-[acyl-carrier-protein] synthase II, chloroplastic-like 
[Citrus sinensis]
Length=156

 Score = 37.4 bits (85),  Expect = 3.5, Method: Compositional matrix adjust.
 Identities = 18/49 (37%), Positives = 27/49 (55%), Gaps = 2/49 (4%)

Query  60   VCRSPCSNYTDWEAVKDPTLFSPRRYVSLETQSNIIQMSC--RGFGRST  106
            +C SP +NY ++  +K   L SP+R+  L ++ NI    C   GF R T
Sbjct  10   LCTSPAANYGNYHPLKSSMLKSPKRHKKLTSRCNIGSTFCIRNGFSRKT  58


Lambda      K        H        a         alpha
   0.323    0.137    0.416    0.792     4.96 

Gapped
Lambda      K        H        a         alpha    sigma
   0.267   0.0410    0.140     1.90     42.6     43.6 

Effective search space used: 647747489103


  Database: nr
    Posted date:  Sep 23, 2015 12:05 AM
  Number of letters in database: 26,053,659,533
  Number of sequences in database:  71,551,133


Matrix: BLOSUM62
Gap Penalties: Existence: 11, Extension: 1
Neighboring words threshold: 11
Window for multiple hits: 40
```
