# Supplementary material for: Comparative genomics provides new insights into the diversity, physiology, and sexuality of the only industrially exploited tremellomycete: Phaffia rhodozyma
Source: BMC Genomics. 2016 Nov 9;17:901. doi: 10.1186/s12864-016-3244-7 (PMC5103461; doi:10.1186/s12864-016-3244-7)
Supplement: Additional file 6: — List of orphan genes with links to PFAM (related to Additional file 1: Table S1). (ZIP 1428 kb) [file 12864_2016_3244_MOESM6_ESM.zip › BLAST_HTML_FTR/G05590_P.html]

BLAST Search Results


```
BLASTP 2.2.27+


Reference:
Stephen F. Altschul, Thomas L. Madden, Alejandro A. Schäffer,
Jinghui Zhang, Zheng Zhang, Webb Miller, and David J. Lipman (1997),
"Gapped BLAST and PSI-BLAST: a new generation of protein database
search programs", Nucleic Acids Res. 25:3389-3402.


Reference for
composition-based statistics:
Alejandro A. Schäffer, L. Aravind, Thomas L. Madden, Sergei
Shavirin, John L. Spouge, Yuri I. Wolf, Eugene V. Koonin, and
Stephen F. Altschul (2001), "Improving the accuracy of PSI-BLAST
protein database searches with composition-based statistics and
other refinements", Nucleic Acids Res. 29:2994-3005.


Database: nr
           71,551,133 sequences; 26,053,659,533 total letters


Query= G05590_P

Length=106
                                                                      Score     E
Sequences producing significant alignments:                          (Bits)  Value

emb|CED85587.1|  hypothetical protein [Xanthophyllomyces dendrorh...   209    5e-67
ref|WP_028243167.1|  hypothetical protein [Pseudobutyrivibrio rum...  39.3    0.34 
ref|WP_014623895.1|  histidine kinase [Spirochaeta thermophila] >...  38.9    0.52 
ref|WP_013313037.1|  histidine kinase [Spirochaeta thermophila] >...  38.9    0.52 
ref|WP_013169276.1|  coagulation factor 5/8 type domain-containin...  38.9    0.54 
ref|YP_004414702.1|  glycoprotein [Aguacate virus] >gb|AEB70968.1...  38.1    1.0  
ref|WP_028292621.1|  hypothetical protein [Oceanobacter kriegii]      36.2    3.5  
emb|CAP08935.1|  fish virus induced TRIM protein [Oncorhynchus my...  36.2    3.8  
ref|XP_003475973.2|  PREDICTED: rab-3A-interacting protein [Cavia...  35.8    4.6  
emb|CAP08943.1|  fish virus induced TRIM protein [Oncorhynchus my...  35.8    4.6  
ref|WP_011196706.1|  3-hydroxybutyryl-CoA dehydrogenase [Symbioba...  35.4    5.2  
ref|WP_012940595.1|  family 2 glycosyl transferase [Archaeoglobus...  35.8    5.2  
ref|XP_010863383.1|  PREDICTED: tripartite motif-containing prote...  35.0    8.5  
ref|XP_010863384.1|  PREDICTED: tripartite motif-containing prote...  35.0    8.9  


 >emb|CED85587.1| hypothetical protein [Xanthophyllomyces dendrorhous]
Length=105

 Score =  209 bits (531),  Expect = 5e-67, Method: Compositional matrix adjust.
 Identities = 105/105 (100%), Positives = 105/105 (100%), Gaps = 0/105 (0%)

Query  1    MSDLKHLEKSIKKESKQSVKELDQAHKDLEKAIKAEKEASVLTHAEHKMQNAKRDVMKSE  60
            MSDLKHLEKSIKKESKQSVKELDQAHKDLEKAIKAEKEASVLTHAEHKMQNAKRDVMKSE
Sbjct  1    MSDLKHLEKSIKKESKQSVKELDQAHKDLEKAIKAEKEASVLTHAEHKMQNAKRDVMKSE  60

Query  61   NEIARAQGLVHQLDSEVAEKKTLIDNLQSASKTQENPSLPPGECF  105
            NEIARAQGLVHQLDSEVAEKKTLIDNLQSASKTQENPSLPPGECF
Sbjct  61   NEIARAQGLVHQLDSEVAEKKTLIDNLQSASKTQENPSLPPGECF  105


>ref|WP_028243167.1| hypothetical protein [Pseudobutyrivibrio ruminis]
Length=1141

 Score = 39.3 bits (90),  Expect = 0.34, Method: Compositional matrix adjust.
 Identities = 28/83 (34%), Positives = 46/83 (55%), Gaps = 6/83 (7%)

Query  14   ESKQSVKELDQAHKDLEKAIKAEKEASVLTHAEHKMQNAKRDVMKSENEIARAQGLVHQL  73
            ++KQ+ +   QA KD E A    KE +    AE  ++ A  +V + ENEI + QG++   
Sbjct  672  QNKQAYENAKQAAKDYEAA----KEKT--NQAEKILEAANAEVTRLENEILKQQGIIDNA  725

Query  74   DSEVAEKKTLIDNLQSASKTQEN  96
            DS ++ + ++I+N     KT EN
Sbjct  726  DSTISAQNSIINNASDDKKTAEN  748


>ref|WP_014623895.1| histidine kinase [Spirochaeta thermophila]
 gb|AEJ60492.1| multi-sensor hybrid histidine kinase [Spirochaeta thermophila 
DSM 6578]
Length=900

 Score = 38.9 bits (89),  Expect = 0.52, Method: Composition-based stats.
 Identities = 27/98 (28%), Positives = 47/98 (48%), Gaps = 8/98 (8%)

Query  4    LKHLEKSIKKESKQSVKELDQAHKDLEKAIKAEKEASVLTHAEHKMQNAKRDVM------  57
            L+     I++E  +  +EL +A++ LEKA +A+ E   L +  H+M+     +M      
Sbjct  373  LEEYSTGIEEEVARRTRELREANRLLEKANRAKSE--FLANVSHEMRTPLHAIMGFAEAL  430

Query  58   KSENEIARAQGLVHQLDSEVAEKKTLIDNLQSASKTQE  95
            K E +    +G +  L SE    K LID +    K ++
Sbjct  431  KGEQDPREREGHLQLLLSEAQRLKVLIDEILDVEKMEQ  468


>ref|WP_013313037.1| histidine kinase [Spirochaeta thermophila]
 gb|ADN01196.1| putative histidine kinase [Spirochaeta thermophila DSM 6192]
Length=900

 Score = 38.9 bits (89),  Expect = 0.52, Method: Composition-based stats.
 Identities = 27/98 (28%), Positives = 47/98 (48%), Gaps = 8/98 (8%)

Query  4    LKHLEKSIKKESKQSVKELDQAHKDLEKAIKAEKEASVLTHAEHKMQNAKRDVM------  57
            L+     I++E  +  +EL +A++ LEKA +A+ E   L +  H+M+     +M      
Sbjct  373  LEEYSTGIEEEVARRTRELREANRLLEKANRAKSE--FLANVSHEMRTPLHAIMGFAEAL  430

Query  58   KSENEIARAQGLVHQLDSEVAEKKTLIDNLQSASKTQE  95
            K E +    +G +  L SE    K LID +    K ++
Sbjct  431  KGEQDPREREGHLQLLLSEAQRLKVLIDEILDVEKMEQ  468


>ref|WP_013169276.1| coagulation factor 5/8 type domain-containing protein [Arcanobacterium 
haemolyticum]
 gb|ADH91778.1| coagulation factor 5/8 type domain protein [Arcanobacterium haemolyticum 
DSM 20595]
Length=982

 Score = 38.9 bits (89),  Expect = 0.54, Method: Composition-based stats.
 Identities = 32/94 (34%), Positives = 46/94 (49%), Gaps = 7/94 (7%)

Query  3    DLKHLEKSIKKESKQSVKELDQAHKDLEKAIKAEKEASVLTHAEHKMQNAKRDVMKSENE  62
            DLK +EK ++ +  +  K+LDQA KD E A+  +KE      AE   Q AK  + K  +E
Sbjct  794  DLKKIEK-LEADKAELEKKLDQATKDKEVALTKKKE------AEDAAQKAKESLTKINDE  846

Query  63   IARAQGLVHQLDSEVAEKKTLIDNLQSASKTQEN  96
            +A+ Q    +        KT ID L    +  EN
Sbjct  847  LAKLQKEYEKAIDGKEALKTKIDTLNKQRQNLEN  880


>ref|YP_004414702.1| glycoprotein [Aguacate virus]
 gb|AEB70968.1| glycoprotein [Aguacate virus]
Length=1320

 Score = 38.1 bits (87),  Expect = 1.0, Method: Composition-based stats.
 Identities = 24/80 (30%), Positives = 46/80 (58%), Gaps = 15/80 (19%)

Query  1    MSDLKHLEKSIKKESKQSVKELDQAHKDLEK----------AIKAEKEASVLTH-----A  45
            + D K  E+S K++SKQ  +ELD   KD++K           +++E+  S ++       
Sbjct  169  LRDSKIREESAKEKSKQLTEELDTMKKDIDKFKGRTDELILHLESERLESRISRNKSRWL  228

Query  46   EHKMQNAKRDVMKSENEIAR  65
            EH++++AK DV++ +N++ R
Sbjct  229  EHELKDAKEDVIRLQNDMLR  248


>ref|WP_028292621.1| hypothetical protein [Oceanobacter kriegii]
Length=884

 Score = 36.2 bits (82),  Expect = 3.5, Method: Compositional matrix adjust.
 Identities = 28/103 (27%), Positives = 50/103 (49%), Gaps = 2/103 (2%)

Query  1    MSDLKHLEKSIKKESKQSVKELDQAHKDLEKAIKAEKEASVLTHAEHKM--QNAKRDVMK  58
            ++++ H  KS   E   + +    AH  L +AI++  +A VL  A+ K+   N++ +   
Sbjct  64   LNEVMHNLKSTNSELVTANEAATHAHDLLRQAIESISDAFVLYDADRKLVLANSRFNQFW  123

Query  59   SENEIARAQGLVHQLDSEVAEKKTLIDNLQSASKTQENPSLPP  101
              + IA +QG+  Q    +A+K  LID       +  NP+  P
Sbjct  124  ESSGIAISQGMDQQRIHRLAQKYRLIDRYYGDVPSNSNPATSP  166


>emb|CAP08935.1| fish virus induced TRIM protein [Oncorhynchus mykiss]
Length=552

 Score = 36.2 bits (82),  Expect = 3.8, Method: Composition-based stats.
 Identities = 25/97 (26%), Positives = 51/97 (53%), Gaps = 13/97 (13%)

Query  1    MSDLKHLEKSIKKESKQSVKELDQAHKDLEKAI-KAEKEASVLTHAEHKMQNAKRDVMKS  59
            M +L+   +S+K+ ++ +V++ DQ   +L ++I +   E   L  A+ K Q         
Sbjct  220  MKELQQAVESLKRSAQSAVEDSDQIFTELIRSIERRSSEVKELIRAQEKAQ---------  270

Query  60   ENEIARAQGLVHQLDSEVAEKKTLIDNLQSASKTQEN  96
               +++A+GL+ QL  E+AE +     L+  S T+++
Sbjct  271  ---VSQAEGLLEQLKQEIAELRKRSTELEQLSHTEDH  304


>ref|XP_003475973.2| PREDICTED: rab-3A-interacting protein [Cavia porcellus]
Length=460

 Score = 35.8 bits (81),  Expect = 4.6, Method: Compositional matrix adjust.
 Identities = 27/80 (34%), Positives = 41/80 (51%), Gaps = 7/80 (9%)

Query  23   DQAHKDLEKAIKAEKEASVLTHAEHKMQNAKRDVMKSENEIARAQGLVHQLDSEVAEKKT  82
            DQ  ++LE+   +  E ++L   E+  + A R  + SE     A+G +  L +EVA  KT
Sbjct  187  DQLGQELEELTASLFEVALLVVRENSFKQASRTTLASE-----ARGKIDVLQAEVAALKT  241

Query  83   LIDNLQSASKTQENPSLPPG  102
            L+ +    S TQE   LP G
Sbjct  242  LVLSSSPTSPTQE--PLPGG  259


>emb|CAP08943.1| fish virus induced TRIM protein [Oncorhynchus mykiss]
Length=551

 Score = 35.8 bits (81),  Expect = 4.6, Method: Composition-based stats.
 Identities = 26/97 (27%), Positives = 49/97 (51%), Gaps = 13/97 (13%)

Query  1    MSDLKHLEKSIKKESKQSVKELDQAHKDLEKAI-KAEKEASVLTHAEHKMQNAKRDVMKS  59
            + +LK   KS K+ ++ +V++ DQ   +L ++I +   E   L  A+ K Q         
Sbjct  220  LKELKQAVKSFKRSAQSAVEDSDQIFTELIRSIERRSSEVKELIRAQEKAQ---------  270

Query  60   ENEIARAQGLVHQLDSEVAEKKTLIDNLQSASKTQEN  96
               ++RA+ L+ QL  E+AE +     L+  S T+++
Sbjct  271  ---VSRAEKLLEQLKQEIAELRKRSTELEQLSHTEDH  304


>ref|WP_011196706.1| 3-hydroxybutyryl-CoA dehydrogenase [Symbiobacterium thermophilum]
 dbj|BAD41569.1| 3-hydroxybutyryl-CoA dehydrogenase [Symbiobacterium thermophilum 
IAM 14863]
Length=296

 Score = 35.4 bits (80),  Expect = 5.2, Method: Compositional matrix adjust.
 Identities = 22/72 (31%), Positives = 38/72 (53%), Gaps = 0/72 (0%)

Query  21   ELDQAHKDLEKAIKAEKEASVLTHAEHKMQNAKRDVMKSENEIARAQGLVHQLDSEVAEK  80
            EL+QA + L+ A++ E E   LT +E +   A+  +    NE+A+A  ++  L  E+AE 
Sbjct  38   ELEQARRQLDLALQHEIEKWALTQSEKRAILARISMTTDINELAKADFVIATLVVEIAED  97

Query  81   KTLIDNLQSASK  92
            K +   L    +
Sbjct  98   KEIFRTLDQVCR  109


>ref|WP_012940595.1| family 2 glycosyl transferase [Archaeoglobus profundus]
 gb|ADB58259.1| glycosyl transferase family 2 [Archaeoglobus profundus DSM 5631]
Length=1164

 Score = 35.8 bits (81),  Expect = 5.2, Method: Composition-based stats.
 Identities = 26/91 (29%), Positives = 51/91 (56%), Gaps = 5/91 (5%)

Query  9    KSIKKESKQSVKELDQAH---KDLEKAIKAEKEASVLTHAEHKMQNAKRDVMKSENEIAR  65
            +S+K E ++ +++LD+     +DL   I  EK    L     +  N K  + +++NEI R
Sbjct  292  ESLKNELQRLLRDLDKKSALIQDLNNEI--EKLKIDLKDKIQENINLKNKLFETKNEIKR  349

Query  66   AQGLVHQLDSEVAEKKTLIDNLQSASKTQEN  96
             Q ++ +L+ +VA+K  LI  L++ ++  +N
Sbjct  350  LQEIIKRLEEKVADKNNLIAKLEAENEALKN  380


>ref|XP_010863383.1| PREDICTED: tripartite motif-containing protein 16-like protein 
isoform X1 [Esox lucius]
Length=451

 Score = 35.0 bits (79),  Expect = 8.5, Method: Composition-based stats.
 Identities = 22/90 (24%), Positives = 55/90 (61%), Gaps = 7/90 (8%)

Query  14   ESKQSVKELDQAHKDLEKAIK-----AEKEASVLTHAEHKMQNAKRDVMKSEN--EIARA  66
            E ++ +KE+ QA + L+++ +     +EK  + L H+  + ++  +D+++++   ++++A
Sbjct  115  EREKEMKEVQQAVESLKRSAQTAVEDSEKIFTELIHSIERRRSELKDLIRAQEKAQVSQA  174

Query  67   QGLVHQLDSEVAEKKTLIDNLQSASKTQEN  96
            +GL+ +L  E+AE K     L+  S T+++
Sbjct  175  EGLLEKLKQEIAELKKTSTVLEQISHTEDH  204


>ref|XP_010863384.1| PREDICTED: tripartite motif-containing protein 16-like protein 
isoform X2 [Esox lucius]
Length=441

 Score = 35.0 bits (79),  Expect = 8.9, Method: Composition-based stats.
 Identities = 22/90 (24%), Positives = 55/90 (61%), Gaps = 7/90 (8%)

Query  14   ESKQSVKELDQAHKDLEKAIK-----AEKEASVLTHAEHKMQNAKRDVMKSEN--EIARA  66
            E ++ +KE+ QA + L+++ +     +EK  + L H+  + ++  +D+++++   ++++A
Sbjct  115  EREKEMKEVQQAVESLKRSAQTAVEDSEKIFTELIHSIERRRSELKDLIRAQEKAQVSQA  174

Query  67   QGLVHQLDSEVAEKKTLIDNLQSASKTQEN  96
            +GL+ +L  E+AE K     L+  S T+++
Sbjct  175  EGLLEKLKQEIAELKKTSTVLEQISHTEDH  204


Lambda      K        H        a         alpha
   0.310    0.124    0.325    0.792     4.96 

Gapped
Lambda      K        H        a         alpha    sigma
   0.267   0.0410    0.140     1.90     42.6     43.6 

Effective search space used: 641307061298


  Database: nr
    Posted date:  Sep 23, 2015 12:05 AM
  Number of letters in database: 26,053,659,533
  Number of sequences in database:  71,551,133


Matrix: BLOSUM62
Gap Penalties: Existence: 11, Extension: 1
Neighboring words threshold: 11
Window for multiple hits: 40
```
