# Supplementary material for: Comparative genomics provides new insights into the diversity, physiology, and sexuality of the only industrially exploited tremellomycete: Phaffia rhodozyma
Source: BMC Genomics. 2016 Nov 9;17:901. doi: 10.1186/s12864-016-3244-7 (PMC5103461; doi:10.1186/s12864-016-3244-7)
Supplement: Additional file 6: — List of orphan genes with links to PFAM (related to Additional file 1: Table S1). (ZIP 1428 kb) [file 12864_2016_3244_MOESM6_ESM.zip › BLAST_HTML_FTR/G00304_P.html]

BLAST Search Results


```
BLASTP 2.2.27+


Reference:
Stephen F. Altschul, Thomas L. Madden, Alejandro A. Schäffer,
Jinghui Zhang, Zheng Zhang, Webb Miller, and David J. Lipman (1997),
"Gapped BLAST and PSI-BLAST: a new generation of protein database
search programs", Nucleic Acids Res. 25:3389-3402.


Reference for
composition-based statistics:
Alejandro A. Schäffer, L. Aravind, Thomas L. Madden, Sergei
Shavirin, John L. Spouge, Yuri I. Wolf, Eugene V. Koonin, and
Stephen F. Altschul (2001), "Improving the accuracy of PSI-BLAST
protein database searches with composition-based statistics and
other refinements", Nucleic Acids Res. 29:2994-3005.


Database: nr
           71,551,133 sequences; 26,053,659,533 total letters


Query= G00304_P

Length=1051
                                                                      Score     E
Sequences producing significant alignments:                          (Bits)  Value

emb|CDZ98232.1|  hypothetical protein [Xanthophyllomyces dendrorh...  1671    0.0  
emb|CCO27071.1|  hypothetical protein BN14_01105 [Rhizoctonia sol...  47.8    0.068
gb|KEP55405.1|  putative C-4 methylsterol oxidase [Rhizoctonia so...  47.8    0.071
gb|EUC64297.1|  C-4 methylsterol oxidase, putative [Rhizoctonia s...  47.4    0.093
emb|CEL60963.1|  nuclear receptor coactivator 6 [Rhizoctonia sola...  47.4    0.11 
gb|KDN46217.1|  hypothetical protein RSAG8_04444, partial [Rhizoc...  46.6    0.16 
gb|KDQ57842.1|  hypothetical protein JAAARDRAFT_194109 [Jaapia ar...  45.4    0.39 
gb|KDE03947.1|  hypothetical protein MVLG_05581 [Microbotryum vio...  45.4    0.43 
gb|EJT52165.1|  hypothetical protein A1Q1_06271 [Trichosporon asa...  44.7    0.56 
ref|XP_012051497.1|  hypothetical protein CNAG_04333 [Cryptococcu...  44.7    0.68 
gb|KIR41455.1|  hypothetical protein I313_02584 [Cryptococcus gat...  43.9    1.0  
gb|KGB76881.1|  hypothetical protein CNBG_2719 [Cryptococcus gatt...  43.9    1.0  
gb|KIR29162.1|  hypothetical protein I309_01748 [Cryptococcus gat...  43.9    1.0  
gb|KIR34160.1|  hypothetical protein I352_03395 [Cryptococcus gat...  43.9    1.1  
gb|KIR98532.1|  hypothetical protein L804_04106 [Cryptococcus gat...  43.9    1.1  
ref|XP_007001196.1|  hypothetical protein TREMEDRAFT_58248 [Treme...  43.5    1.2  
gb|KIR88286.1|  hypothetical protein I308_01348 [Cryptococcus gat...  43.1    1.7  
dbj|BAJ97644.1|  predicted protein [Hordeum vulgare subsp. vulgare]   42.4    2.1  
dbj|BAK02682.1|  predicted protein [Hordeum vulgare subsp. vulgar...  42.4    2.2  
ref|XP_773751.1|  hypothetical protein CNBH2040 [Cryptococcus neo...  42.7    2.2  
ref|NP_001149731.1|  phosphosulfolactate synthase-related protein...  42.0    2.4  
gb|KIR81291.1|  hypothetical protein I306_01524 [Cryptococcus gat...  42.7    2.6  
ref|XP_003195803.1|  hypothetical protein CGB_H3190C [Cryptococcu...  42.4    2.8  
gb|KJE03849.1|  hypothetical protein I311_02307 [Cryptococcus gat...  42.4    2.8  
emb|CUA66886.1|  Formin-like protein 20 [Rhizoctonia solani]          42.4    3.5  
gb|KIR53430.1|  hypothetical protein I315_04022 [Cryptococcus gat...  41.6    4.8  
ref|XP_007317349.1|  hypothetical protein SERLADRAFT_436985 [Serp...  42.0    4.9  
ref|XP_006460824.1|  hypothetical protein AGABI2DRAFT_117746 [Aga...  40.8    9.5  


 >emb|CDZ98232.1| hypothetical protein [Xanthophyllomyces dendrorhous]
Length=824

 Score = 1671 bits (4328),  Expect = 0.0, Method: Compositional matrix adjust.
 Identities = 824/824 (100%), Positives = 824/824 (100%), Gaps = 0/824 (0%)

Query  227   MDSISFVYDPPMISDEFLKGPAVALSPEAEAFVLSSPFNPMGLKVKPTIGEETQPKRLQF  286
             MDSISFVYDPPMISDEFLKGPAVALSPEAEAFVLSSPFNPMGLKVKPTIGEETQPKRLQF
Sbjct  1     MDSISFVYDPPMISDEFLKGPAVALSPEAEAFVLSSPFNPMGLKVKPTIGEETQPKRLQF  60

Query  287   TEKKAPLNIHQGSPINPDLLKSIAPYIPTSLRTGTEKKFVAPVAPKVKKTVPIHQEGFKS  346
             TEKKAPLNIHQGSPINPDLLKSIAPYIPTSLRTGTEKKFVAPVAPKVKKTVPIHQEGFKS
Sbjct  61    TEKKAPLNIHQGSPINPDLLKSIAPYIPTSLRTGTEKKFVAPVAPKVKKTVPIHQEGFKS  120

Query  347   IPVMVGPESLPYARNPSGVDGSVLDSDGRLNVWRPSVDDPEPEHLDHLHFQRRPGAPNPP  406
             IPVMVGPESLPYARNPSGVDGSVLDSDGRLNVWRPSVDDPEPEHLDHLHFQRRPGAPNPP
Sbjct  121   IPVMVGPESLPYARNPSGVDGSVLDSDGRLNVWRPSVDDPEPEHLDHLHFQRRPGAPNPP  180

Query  407   HRAVSQEEDNSSAYAHPSMFRVYTMRDKAAMMNKSQQPFSTNKVSASYIPSSFPVRSPSM  466
             HRAVSQEEDNSSAYAHPSMFRVYTMRDKAAMMNKSQQPFSTNKVSASYIPSSFPVRSPSM
Sbjct  181   HRAVSQEEDNSSAYAHPSMFRVYTMRDKAAMMNKSQQPFSTNKVSASYIPSSFPVRSPSM  240

Query  467   STNPSHVSSLPIPSSRSPLPSGNRLDHAQHSLHSNQSAHREHDFSHLHPSYPVSSSDPRR  526
             STNPSHVSSLPIPSSRSPLPSGNRLDHAQHSLHSNQSAHREHDFSHLHPSYPVSSSDPRR
Sbjct  241   STNPSHVSSLPIPSSRSPLPSGNRLDHAQHSLHSNQSAHREHDFSHLHPSYPVSSSDPRR  300

Query  527   FNELPTGQQYPSQGPDGFLQAQMGLVHDPMFLGQDILHLTASLLNLGLDPAVVLLHTARH  586
             FNELPTGQQYPSQGPDGFLQAQMGLVHDPMFLGQDILHLTASLLNLGLDPAVVLLHTARH
Sbjct  301   FNELPTGQQYPSQGPDGFLQAQMGLVHDPMFLGQDILHLTASLLNLGLDPAVVLLHTARH  360

Query  587   MALMNSGLPSELIPYASRANASMHLGFDRSPSPGGPQIFSPQHVENSTEWATPASNSSVH  646
             MALMNSGLPSELIPYASRANASMHLGFDRSPSPGGPQIFSPQHVENSTEWATPASNSSVH
Sbjct  361   MALMNSGLPSELIPYASRANASMHLGFDRSPSPGGPQIFSPQHVENSTEWATPASNSSVH  420

Query  647   YHAPLHVAPRISPSLSPTLVDVHQDVLPHLIDLAALDSDPVLPKIQVESIAHYRDDQVRI  706
             YHAPLHVAPRISPSLSPTLVDVHQDVLPHLIDLAALDSDPVLPKIQVESIAHYRDDQVRI
Sbjct  421   YHAPLHVAPRISPSLSPTLVDVHQDVLPHLIDLAALDSDPVLPKIQVESIAHYRDDQVRI  480

Query  707   PSPYPIGDFIVPSLEHEKQLLKELKPAANLFAPPMDALPMAFESVSKPREESFLSTVHSV  766
             PSPYPIGDFIVPSLEHEKQLLKELKPAANLFAPPMDALPMAFESVSKPREESFLSTVHSV
Sbjct  481   PSPYPIGDFIVPSLEHEKQLLKELKPAANLFAPPMDALPMAFESVSKPREESFLSTVHSV  540

Query  767   ELPIVKTRSLTESALKPSWEEPIFLPAGSGAGFANGGLRLCGPRAKVPRRSIEEMALEAI  826
             ELPIVKTRSLTESALKPSWEEPIFLPAGSGAGFANGGLRLCGPRAKVPRRSIEEMALEAI
Sbjct  541   ELPIVKTRSLTESALKPSWEEPIFLPAGSGAGFANGGLRLCGPRAKVPRRSIEEMALEAI  600

Query  827   EVACTWEEPAESSALKRERISLTEAIEATKEAARQEAIRKTREENEMKMRVLAEIEVANS  886
             EVACTWEEPAESSALKRERISLTEAIEATKEAARQEAIRKTREENEMKMRVLAEIEVANS
Sbjct  601   EVACTWEEPAESSALKRERISLTEAIEATKEAARQEAIRKTREENEMKMRVLAEIEVANS  660

Query  887   RMKNQQLKEEKGGGKKIVEVKAGLPGRVVSGAAGDPSKSKKESYLTLGGLNKMFSEPPLG  946
             RMKNQQLKEEKGGGKKIVEVKAGLPGRVVSGAAGDPSKSKKESYLTLGGLNKMFSEPPLG
Sbjct  661   RMKNQQLKEEKGGGKKIVEVKAGLPGRVVSGAAGDPSKSKKESYLTLGGLNKMFSEPPLG  720

Query  947   VNVSDSPLHQTRRVISEPVYKQNFRSSSTALMSGLPQKPAVKSSATTPSKSKLPVRAKAL  1006
             VNVSDSPLHQTRRVISEPVYKQNFRSSSTALMSGLPQKPAVKSSATTPSKSKLPVRAKAL
Sbjct  721   VNVSDSPLHQTRRVISEPVYKQNFRSSSTALMSGLPQKPAVKSSATTPSKSKLPVRAKAL  780

Query  1007  PVSKTSDSDTAGSVEHTARVSRKSKNSRPSNRAQVSKPFSSVLK  1050
             PVSKTSDSDTAGSVEHTARVSRKSKNSRPSNRAQVSKPFSSVLK
Sbjct  781   PVSKTSDSDTAGSVEHTARVSRKSKNSRPSNRAQVSKPFSSVLK  824


>emb|CCO27071.1| hypothetical protein BN14_01105 [Rhizoctonia solani AG-1 IB]
Length=502

 Score = 47.8 bits (112),  Expect = 0.068, Method: Compositional matrix adjust.
 Identities = 37/119 (31%), Positives = 54/119 (45%), Gaps = 24/119 (20%)

Query  284  LQFTEKKAPLNIHQGSPINPDLLKSIAPYIPTSLRTGTEKKFVAPVAPKVKKTVPIHQEG  343
            +++ E++    + QG  I P++  +     P S   G++  F  P  P            
Sbjct  332  VEYLEREHRYTVDQGQ-IGPNINTNGDSRAPISADNGSDWIFFDPPRP------------  378

Query  344  FKSIPVMVGPESLPYARNPSGVDGSVLDSDGRLN--VWRPSVDDP------EPEHLDHL  394
               IP + GP SLPYAR PSG +G VL+    L+  VW  S  DP      EP+H + L
Sbjct  379  ---IPALHGPSSLPYARCPSGAEGVVLNDQQELDGVVWGLSERDPRSRPPDEPKHTEKL  434


>gb|KEP55405.1| putative C-4 methylsterol oxidase [Rhizoctonia solani 123E]
Length=855

 Score = 47.8 bits (112),  Expect = 0.071, Method: Compositional matrix adjust.
 Identities = 45/117 (38%), Positives = 53/117 (45%), Gaps = 23/117 (20%)

Query  345  KSIPVMVGPESLPYARNPSGVDGSVLDSDGRLN--VWRPSVDDPEPEHLDHLHFQRRPGA  402
            + IP + GP SLPYAR PSG +G VLD    LN  VW  S  DP   H    H  RR GA
Sbjct  413  RPIPALHGPPSLPYARCPSGAEGVVLDDQQELNGVVWGLSEKDPR-SHCPDEH--RRTGA  469

Query  403  ------------PNPPHRAVSQEEDNSSAYAHPSMFRVYTMRDKAAMMNKSQQPFST  447
                        P PP     +E+   SA A     RV  +R  A+  N   +P ST
Sbjct  470  SSKEQLLLAIKDPRPPRPVTREEKRIESATAS----RVSEIRPLASAHNP--RPLST  520


>gb|EUC64297.1| C-4 methylsterol oxidase, putative [Rhizoctonia solani AG-3 Rhs1AP]
Length=539

 Score = 47.4 bits (111),  Expect = 0.093, Method: Compositional matrix adjust.
 Identities = 42/109 (39%), Positives = 49/109 (45%), Gaps = 21/109 (19%)

Query  345  KSIPVMVGPESLPYARNPSGVDGSVLDSDGRLN--VWRPSVDDPEPEHLDHLHFQRRPGA  402
            + IP + GP SLPYAR PSG +G VLD    LN  VW  S  DP   H    H  RR GA
Sbjct  413  RPIPALHGPPSLPYARCPSGAEGVVLDDQQELNGVVWGLSEKDPR-SHCPDEH--RRTGA  469

Query  403  ------------PNPPHRAVSQEEDNSSAYAHPSMFRVYTMRDKAAMMN  439
                        P PP     +E+   SA A     RV  +R  A+  N
Sbjct  470  SSKEQLLLAIKDPRPPRPVTREEKRIESATAS----RVSEIRPLASAHN  514


>emb|CEL60963.1| nuclear receptor coactivator 6 [Rhizoctonia solani AG-1 IB]
Length=883

 Score = 47.4 bits (111),  Expect = 0.11, Method: Compositional matrix adjust.
 Identities = 37/119 (31%), Positives = 54/119 (45%), Gaps = 24/119 (20%)

Query  284  LQFTEKKAPLNIHQGSPINPDLLKSIAPYIPTSLRTGTEKKFVAPVAPKVKKTVPIHQEG  343
            +++ E++    + QG  I P++  +     P S   G++  F  P  P            
Sbjct  361  VEYLEREHRYTVDQGQ-IGPNINTNEDSRAPISADNGSDWIFFDPPRP------------  407

Query  344  FKSIPVMVGPESLPYARNPSGVDGSVLDSDGRLN--VWRPSVDDP------EPEHLDHL  394
               IP + GP SLPYAR PSG +G VL+    L+  VW  S  DP      EP+H + L
Sbjct  408  ---IPALHGPSSLPYARCPSGAEGVVLNDQQELDGVVWGLSERDPRSRPPDEPKHTEKL  463


>gb|KDN46217.1| hypothetical protein RSAG8_04444, partial [Rhizoctonia solani 
AG-8 WAC10335]
Length=968

 Score = 46.6 bits (109),  Expect = 0.16, Method: Compositional matrix adjust.
 Identities = 25/50 (50%), Positives = 29/50 (58%), Gaps = 2/50 (4%)

Query  345  KSIPVMVGPESLPYARNPSGVDGSVLDSDGRLN--VWRPSVDDPEPEHLD  392
            + IP + GP SLPYAR PSG +G VLD    L+  VW  S  DP   H D
Sbjct  418  RPIPALHGPPSLPYARCPSGAEGVVLDDQQELDGVVWGLSEKDPRSRHPD  467


>gb|KDQ57842.1| hypothetical protein JAAARDRAFT_194109 [Jaapia argillacea MUCL 
33604]
Length=785

 Score = 45.4 bits (106),  Expect = 0.39, Method: Compositional matrix adjust.
 Identities = 24/64 (38%), Positives = 35/64 (55%), Gaps = 10/64 (16%)

Query  335  KTVPIHQEGF--------KSIPVMVGPESLPYARNPSGVDGSVLDSDGRL--NVWRPSVD  384
            KTVP+   G+        + IP + GP SLPYAR PSG +G++++    L   +W    D
Sbjct  206  KTVPLDDAGWIAWTSSPPRPIPALHGPLSLPYARCPSGAEGTIIEEQDHLPRMIWGLGSD  265

Query  385  DPEP  388
            D +P
Sbjct  266  DSQP  269


>gb|KDE03947.1| hypothetical protein MVLG_05581 [Microbotryum violaceum p1A1 
Lamole]
Length=959

 Score = 45.4 bits (106),  Expect = 0.43, Method: Compositional matrix adjust.
 Identities = 20/46 (43%), Positives = 27/46 (59%), Gaps = 0/46 (0%)

Query  339  IHQEGFKSIPVMVGPESLPYARNPSGVDGSVLDSDGRLNVWRPSVD  384
            + Q GF +IP + GP SLPYAR PSG+D  +   D   + W  + D
Sbjct  401  LAQRGFSTIPSLHGPLSLPYARCPSGIDAYLFSMDKEEDPWTFAFD  446


>gb|EJT52165.1| hypothetical protein A1Q1_06271 [Trichosporon asahii var. asahii 
CBS 2479]
 gb|EKC98703.1| hypothetical protein A1Q2_06935 [Trichosporon asahii var. asahii 
CBS 8904]
Length=582

 Score = 44.7 bits (104),  Expect = 0.56, Method: Compositional matrix adjust.
 Identities = 22/40 (55%), Positives = 27/40 (68%), Gaps = 0/40 (0%)

Query  332  KVKKTVPIHQEGFKSIPVMVGPESLPYARNPSGVDGSVLD  371
            KVK   P+     + IP++ GP SLPYARNPSGVD +V D
Sbjct  194  KVKPIRPVAGARERPIPLLHGPLSLPYARNPSGVDATVAD  233


>ref|XP_012051497.1| hypothetical protein CNAG_04333 [Cryptococcus neoformans var. 
grubii H99]
 gb|AFR97064.2| hypothetical protein CNAG_04333 [Cryptococcus neoformans var. 
grubii H99]
Length=714

 Score = 44.7 bits (104),  Expect = 0.68, Method: Compositional matrix adjust.
 Identities = 26/54 (48%), Positives = 32/54 (59%), Gaps = 3/54 (6%)

Query  321  TEKKFVAPVAPKVKKTVP---IHQEGFKSIPVMVGPESLPYARNPSGVDGSVLD  371
            +EKKF +    ++KK      I     K IP + GP SLPYARNPSGVD +V D
Sbjct  216  SEKKFTSWRRDQIKKAKRPELILSSTPKPIPTLYGPLSLPYARNPSGVDATVAD  269


>gb|KIR41455.1| hypothetical protein I313_02584 [Cryptococcus gattii Ram5]
 gb|KIY56541.1| hypothetical protein I307_04004 [Cryptococcus gattii 99/473]
Length=603

 Score = 43.9 bits (102),  Expect = 1.0, Method: Compositional matrix adjust.
 Identities = 19/27 (70%), Positives = 21/27 (78%), Gaps = 0/27 (0%)

Query  345  KSIPVMVGPESLPYARNPSGVDGSVLD  371
            K IP + GP SLPYARNPSGVD +V D
Sbjct  247  KPIPTLYGPLSLPYARNPSGVDATVAD  273


>gb|KGB76881.1| hypothetical protein CNBG_2719 [Cryptococcus gattii R265]
 gb|KIR71697.1| hypothetical protein I310_04375 [Cryptococcus gattii CA1014]
Length=603

 Score = 43.9 bits (102),  Expect = 1.0, Method: Compositional matrix adjust.
 Identities = 19/27 (70%), Positives = 21/27 (78%), Gaps = 0/27 (0%)

Query  345  KSIPVMVGPESLPYARNPSGVDGSVLD  371
            K IP + GP SLPYARNPSGVD +V D
Sbjct  247  KPIPTLYGPLSLPYARNPSGVDATVAD  273


>gb|KIR29162.1| hypothetical protein I309_01748 [Cryptococcus gattii LA55]
 gb|KIR91280.1| hypothetical protein I304_04749 [Cryptococcus gattii CBS 10090]
Length=603

 Score = 43.9 bits (102),  Expect = 1.0, Method: Compositional matrix adjust.
 Identities = 19/27 (70%), Positives = 21/27 (78%), Gaps = 0/27 (0%)

Query  345  KSIPVMVGPESLPYARNPSGVDGSVLD  371
            K IP + GP SLPYARNPSGVD +V D
Sbjct  247  KPIPTLYGPLSLPYARNPSGVDATVAD  273


>gb|KIR34160.1| hypothetical protein I352_03395 [Cryptococcus gattii MMRL2647]
Length=603

 Score = 43.9 bits (102),  Expect = 1.1, Method: Compositional matrix adjust.
 Identities = 19/27 (70%), Positives = 21/27 (78%), Gaps = 0/27 (0%)

Query  345  KSIPVMVGPESLPYARNPSGVDGSVLD  371
            K IP + GP SLPYARNPSGVD +V D
Sbjct  247  KPIPTLYGPLSLPYARNPSGVDATVAD  273


>gb|KIR98532.1| hypothetical protein L804_04106 [Cryptococcus gattii 2001/935-1]
Length=603

 Score = 43.9 bits (102),  Expect = 1.1, Method: Compositional matrix adjust.
 Identities = 19/27 (70%), Positives = 21/27 (78%), Gaps = 0/27 (0%)

Query  345  KSIPVMVGPESLPYARNPSGVDGSVLD  371
            K IP + GP SLPYARNPSGVD +V D
Sbjct  247  KPIPTLYGPLSLPYARNPSGVDATVAD  273


>ref|XP_007001196.1| hypothetical protein TREMEDRAFT_58248 [Tremella mesenterica DSM 
1558]
 gb|EIW72095.1| hypothetical protein TREMEDRAFT_58248 [Tremella mesenterica DSM 
1558]
Length=583

 Score = 43.5 bits (101),  Expect = 1.2, Method: Compositional matrix adjust.
 Identities = 23/50 (46%), Positives = 29/50 (58%), Gaps = 0/50 (0%)

Query  335  KTVPIHQEGFKSIPVMVGPESLPYARNPSGVDGSVLDSDGRLNVWRPSVD  384
            KT  I     K I  + GP SLPYARNPSGVD ++ + D     ++ SVD
Sbjct  239  KTSKITSTQMKRITALYGPLSLPYARNPSGVDATLPEDDIVPATYQTSVD  288


>gb|KIR88286.1| hypothetical protein I308_01348 [Cryptococcus gattii IND107]
Length=600

 Score = 43.1 bits (100),  Expect = 1.7, Method: Compositional matrix adjust.
 Identities = 18/25 (72%), Positives = 20/25 (80%), Gaps = 0/25 (0%)

Query  347  IPVMVGPESLPYARNPSGVDGSVLD  371
            IP + GP SLPYARNPSGVD +V D
Sbjct  249  IPALYGPLSLPYARNPSGVDATVTD  273


>dbj|BAJ97644.1| predicted protein [Hordeum vulgare subsp. vulgare]
Length=294

 Score = 42.4 bits (98),  Expect = 2.1, Method: Compositional matrix adjust.
 Identities = 47/167 (28%), Positives = 62/167 (37%), Gaps = 28/167 (17%)

Query  461  VRSPSMSTNPSHVSSLPIPSSRSPLPSGNRLDHAQHSLHSNQSAHREHDFSHLHPSYPVS  520
            +RSP  S  P+H +   I  S  P   G +     HSL   +      D +H H  Y VS
Sbjct  34   MRSPCYSFRPAHHALQEILDSLGPFVDGLKFSGGSHSLMGKELIREITDLAHKHDMY-VS  92

Query  521  SSDPRRFNELPTGQQYPSQGPDGFLQAQMGLVHDPMFLGQDILHLTASLLNLGLDPAVVL  580
            + D          +    QGP  F Q     V +   LG D + L A  L L   P   +
Sbjct  93   TGD--------WAEHLLRQGPSSFKQ----YVEECKELGFDTIELNAGSLKL---PEEAI  137

Query  581  LHTARHMALMNSGLPSELIPYASRANASMHLGFDRSPSP-GGPQIFS  626
            L   R   + N+GL         RA     + FD S  P  G + F 
Sbjct  138  LRLVR--LIKNTGL---------RAKPLFSVKFDSSEMPAAGDRAFG  173


>dbj|BAK02682.1| predicted protein [Hordeum vulgare subsp. vulgare]
 dbj|BAJ96192.1| predicted protein [Hordeum vulgare subsp. vulgare]
Length=294

 Score = 42.4 bits (98),  Expect = 2.2, Method: Compositional matrix adjust.
 Identities = 47/167 (28%), Positives = 62/167 (37%), Gaps = 28/167 (17%)

Query  461  VRSPSMSTNPSHVSSLPIPSSRSPLPSGNRLDHAQHSLHSNQSAHREHDFSHLHPSYPVS  520
            +RSP  S  P+H +   I  S  P   G +     HSL   +      D +H H  Y VS
Sbjct  34   MRSPCYSFRPAHHALQEILDSLGPFVDGLKFSGGSHSLMGKELIREITDLAHKHDMY-VS  92

Query  521  SSDPRRFNELPTGQQYPSQGPDGFLQAQMGLVHDPMFLGQDILHLTASLLNLGLDPAVVL  580
            + D          +    QGP  F Q     V +   LG D + L A  L L   P   +
Sbjct  93   TGD--------WAEHLLRQGPSSFKQ----YVEECKELGFDTIELNAGSLKL---PEEAI  137

Query  581  LHTARHMALMNSGLPSELIPYASRANASMHLGFDRSPSP-GGPQIFS  626
            L   R   + N+GL         RA     + FD S  P  G + F 
Sbjct  138  LRLVR--LIKNTGL---------RAKPLFSVKFDSSEMPAAGDRAFG  173


>ref|XP_773751.1| hypothetical protein CNBH2040 [Cryptococcus neoformans var. neoformans 
B-3501A]
 gb|EAL19104.1| hypothetical protein CNBH2040 [Cryptococcus neoformans var. neoformans 
B-3501A]
Length=718

 Score = 42.7 bits (99),  Expect = 2.2, Method: Compositional matrix adjust.
 Identities = 19/27 (70%), Positives = 20/27 (74%), Gaps = 0/27 (0%)

Query  345  KSIPVMVGPESLPYARNPSGVDGSVLD  371
            K IP   GP SLPYARNPSGVD +V D
Sbjct  246  KPIPTFYGPLSLPYARNPSGVDATVAD  272


>ref|NP_001149731.1| phosphosulfolactate synthase-related protein [Zea mays]
 gb|ACG36578.1| phosphosulfolactate synthase-related protein [Zea mays]
 gb|AFW69113.1| phosphosulfolactate synthase protein isoform 1 [Zea mays]
 gb|AFW69114.1| phosphosulfolactate synthase protein isoform 2 [Zea mays]
 gb|AFW69115.1| phosphosulfolactate synthase protein isoform 3 [Zea mays]
Length=299

 Score = 42.0 bits (97),  Expect = 2.4, Method: Compositional matrix adjust.
 Identities = 49/167 (29%), Positives = 64/167 (38%), Gaps = 28/167 (17%)

Query  461  VRSPSMSTNPSHVSSLPIPSSRSPLPSGNRLDHAQHSLHSNQSAHREHDFSHLHPSYPVS  520
            +RSP  S  P++ +   I  S SP   G +     HSL   +      D +H H  Y VS
Sbjct  37   MRSPFYSFRPANQALQEILDSLSPFVDGLKFSGGCHSLMGKELVREITDLAHRHDIY-VS  95

Query  521  SSDPRRFNELPTGQQYPSQGPDGFLQAQMGLVHDPMFLGQDILHLTASLLNLGLDPAVVL  580
            + D          +    QGP  F Q     V +   LG D + L A  LNL   P   L
Sbjct  96   TGD--------WAEHLLRQGPSSFKQ----YVEECKALGFDTIELNAGSLNL---PEEAL  140

Query  581  LHTARHMALMNSGLPSELIPYASRANASMHLGFDRSPSPG-GPQIFS  626
            L   R   + +SGL         RA     + FD S  P  G + F 
Sbjct  141  LRLVR--LIKSSGL---------RAKPMFSVKFDSSDIPASGDRAFG  176


>gb|KIR81291.1| hypothetical protein I306_01524 [Cryptococcus gattii EJB2]
Length=600

 Score = 42.7 bits (99),  Expect = 2.6, Method: Compositional matrix adjust.
 Identities = 24/53 (45%), Positives = 30/53 (57%), Gaps = 3/53 (6%)

Query  322  EKKFVAPVAPKVKKTVP---IHQEGFKSIPVMVGPESLPYARNPSGVDGSVLD  371
            EKK  +    ++KK      I     + IP + GP SLPYARNPSGVD +V D
Sbjct  221  EKKLTSWRRDQIKKAKQPELILTSTLRPIPTLYGPLSLPYARNPSGVDATVPD  273


>ref|XP_003195803.1| hypothetical protein CGB_H3190C [Cryptococcus gattii WM276]
 gb|ADV24016.1| Hypothetical Protein CGB_H3190C [Cryptococcus gattii WM276]
 gb|KIY35021.1| hypothetical protein I305_02586 [Cryptococcus gattii E566]
Length=600

 Score = 42.4 bits (98),  Expect = 2.8, Method: Compositional matrix adjust.
 Identities = 24/53 (45%), Positives = 30/53 (57%), Gaps = 3/53 (6%)

Query  322  EKKFVAPVAPKVKKTVP---IHQEGFKSIPVMVGPESLPYARNPSGVDGSVLD  371
            EKK  +    ++KK      I     + IP + GP SLPYARNPSGVD +V D
Sbjct  221  EKKLTSWRRDQIKKAKQPELILTSTLRPIPTLYGPLSLPYARNPSGVDATVPD  273


>gb|KJE03849.1| hypothetical protein I311_02307 [Cryptococcus gattii NT-10]
Length=600

 Score = 42.4 bits (98),  Expect = 2.8, Method: Compositional matrix adjust.
 Identities = 24/53 (45%), Positives = 30/53 (57%), Gaps = 3/53 (6%)

Query  322  EKKFVAPVAPKVKKTVP---IHQEGFKSIPVMVGPESLPYARNPSGVDGSVLD  371
            EKK  +    ++KK      I     + IP + GP SLPYARNPSGVD +V D
Sbjct  221  EKKLTSWRRDQIKKAKQPELILTSTLRPIPTLYGPLSLPYARNPSGVDATVPD  273


>emb|CUA66886.1| Formin-like protein 20 [Rhizoctonia solani]
Length=783

 Score = 42.4 bits (98),  Expect = 3.5, Method: Compositional matrix adjust.
 Identities = 24/51 (47%), Positives = 28/51 (55%), Gaps = 2/51 (4%)

Query  345  KSIPVMVGPESLPYARNPSGVDGSVLDSDGRLN--VWRPSVDDPEPEHLDH  393
            + IP + GP SLPYAR PSG +G VLD    L+  VW  S  D    H D 
Sbjct  338  RPIPALHGPPSLPYARCPSGAEGVVLDDQQELDGVVWGLSDKDAWSHHPDE  388


>gb|KIR53430.1| hypothetical protein I315_04022 [Cryptococcus gattii Ru294]
Length=600

 Score = 41.6 bits (96),  Expect = 4.8, Method: Compositional matrix adjust.
 Identities = 18/25 (72%), Positives = 20/25 (80%), Gaps = 0/25 (0%)

Query  347  IPVMVGPESLPYARNPSGVDGSVLD  371
            IP + GP SLPYARNPSGVD +V D
Sbjct  249  IPTLYGPLSLPYARNPSGVDATVPD  273


>ref|XP_007317349.1| hypothetical protein SERLADRAFT_436985 [Serpula lacrymans var. 
lacrymans S7.9]
 gb|EGO25227.1| hypothetical protein SERLADRAFT_436985 [Serpula lacrymans var. 
lacrymans S7.9]
Length=857

 Score = 42.0 bits (97),  Expect = 4.9, Method: Compositional matrix adjust.
 Identities = 21/54 (39%), Positives = 31/54 (57%), Gaps = 5/54 (9%)

Query  347  IPVMVGPESLPYARNPSGVDGSVLDSDGRLN--VWRPSVDDPE---PEHLDHLH  395
            IP + GP SLPYAR PSG +G++++    ++  +W    DDP      H  H+H
Sbjct  232  IPALHGPLSLPYARCPSGAEGTIIEEPDNMSRMIWGLGPDDPRGASQAHHSHVH  285


>ref|XP_006460824.1| hypothetical protein AGABI2DRAFT_117746 [Agaricus bisporus var. 
bisporus H97]
 gb|EKV47170.1| hypothetical protein AGABI2DRAFT_117746 [Agaricus bisporus var. 
bisporus H97]
Length=728

 Score = 40.8 bits (94),  Expect = 9.5, Method: Compositional matrix adjust.
 Identities = 32/107 (30%), Positives = 53/107 (50%), Gaps = 17/107 (16%)

Query  337  VPIHQEGFKSIPVMVGPESLPYARNPSGVDGSVLDSDGRLN-VWRPSVDDPEPEHLDHLH  395
            +P+     K IP + GP SLPYAR PSG +G++++ +   + +W           LD   
Sbjct  225  IPVKGSPPKPIPALHGPLSLPYARCPSGAEGTLVEGEDLTHMIW----------GLDSHS  274

Query  396  FQRRPGAPNP--PHRAVSQEEDNSSAYAHPSMFR---VYTMRDKAAM  437
             Q   G P P   HR  +    N+S+   P++++    YT+RD+  +
Sbjct  275  LQAVQGKPTPIGMHRRKAFHP-NTSSRKEPNLYQEDITYTLRDQDVI  320


Lambda      K        H        a         alpha
   0.313    0.129    0.372    0.792     4.96 

Gapped
Lambda      K        H        a         alpha    sigma
   0.267   0.0410    0.140     1.90     42.6     43.6 

Effective search space used: 12857052252443


  Database: nr
    Posted date:  Sep 23, 2015 12:05 AM
  Number of letters in database: 26,053,659,533
  Number of sequences in database:  71,551,133


Matrix: BLOSUM62
Gap Penalties: Existence: 11, Extension: 1
Neighboring words threshold: 11
Window for multiple hits: 40
```
