# Supplementary material for: Comparative genomics provides new insights into the diversity, physiology, and sexuality of the only industrially exploited tremellomycete: Phaffia rhodozyma
Source: BMC Genomics. 2016 Nov 9;17:901. doi: 10.1186/s12864-016-3244-7 (PMC5103461; doi:10.1186/s12864-016-3244-7)
Supplement: Additional file 6: — List of orphan genes with links to PFAM (related to Additional file 1: Table S1). (ZIP 1428 kb) [file 12864_2016_3244_MOESM6_ESM.zip › BLAST_HTML_FTR/G01612_P.html]

BLAST Search Results


```
BLASTP 2.2.27+


Reference:
Stephen F. Altschul, Thomas L. Madden, Alejandro A. Schäffer,
Jinghui Zhang, Zheng Zhang, Webb Miller, and David J. Lipman (1997),
"Gapped BLAST and PSI-BLAST: a new generation of protein database
search programs", Nucleic Acids Res. 25:3389-3402.


Reference for
composition-based statistics:
Alejandro A. Schäffer, L. Aravind, Thomas L. Madden, Sergei
Shavirin, John L. Spouge, Yuri I. Wolf, Eugene V. Koonin, and
Stephen F. Altschul (2001), "Improving the accuracy of PSI-BLAST
protein database searches with composition-based statistics and
other refinements", Nucleic Acids Res. 29:2994-3005.


Database: nr
           71,551,133 sequences; 26,053,659,533 total letters


Query= G01612_P

Length=543
                                                                      Score     E
Sequences producing significant alignments:                          (Bits)  Value

emb|CDZ98209.1|  hypothetical protein [Xanthophyllomyces dendrorh...  1087    0.0  
ref|XP_772482.1|  hypothetical protein CNBL0970 [Cryptococcus neo...  50.4    0.002
ref|XP_007864297.1|  hypothetical protein GLOTRDRAFT_137554 [Gloe...  47.4    0.028
gb|ELU42675.1|  hypothetical protein AG1IA_03294 [Rhizoctonia sol...  41.6    2.1  
emb|CDH49422.1|  hypothetical protein RO3G_13248 [Lichtheimia cor...  40.0    8.2  


 >emb|CDZ98209.1| hypothetical protein [Xanthophyllomyces dendrorhous]
Length=542

 Score = 1087 bits (2810),  Expect = 0.0, Method: Compositional matrix adjust.
 Identities = 542/542 (100%), Positives = 542/542 (100%), Gaps = 0/542 (0%)

Query  1    MLHRSLAHTFHDKENTYHLPSRTPLRIGLAGSSFATAGARTVGRPNVKTPFTKTSVLLGG  60
            MLHRSLAHTFHDKENTYHLPSRTPLRIGLAGSSFATAGARTVGRPNVKTPFTKTSVLLGG
Sbjct  1    MLHRSLAHTFHDKENTYHLPSRTPLRIGLAGSSFATAGARTVGRPNVKTPFTKTSVLLGG  60

Query  61   KPPQTTQTGRGKEKIWTEGGVNLGNSKTGEGKDGQMIEPRRLFTLQTNRGSPKASGTLQL  120
            KPPQTTQTGRGKEKIWTEGGVNLGNSKTGEGKDGQMIEPRRLFTLQTNRGSPKASGTLQL
Sbjct  61   KPPQTTQTGRGKEKIWTEGGVNLGNSKTGEGKDGQMIEPRRLFTLQTNRGSPKASGTLQL  120

Query  121  NSSSKDGALAKPNSNQPPPTLKKSRPAPTPQHVLRTPHPTQRTSLGQSHSPTQQLTNILS  180
            NSSSKDGALAKPNSNQPPPTLKKSRPAPTPQHVLRTPHPTQRTSLGQSHSPTQQLTNILS
Sbjct  121  NSSSKDGALAKPNSNQPPPTLKKSRPAPTPQHVLRTPHPTQRTSLGQSHSPTQQLTNILS  180

Query  181  TPLPSTNRTRRRSKQLHTPLSSSTSSNNLSRSPNKSSNVLSTHSPSRQVDPGFSTPAARM  240
            TPLPSTNRTRRRSKQLHTPLSSSTSSNNLSRSPNKSSNVLSTHSPSRQVDPGFSTPAARM
Sbjct  181  TPLPSTNRTRRRSKQLHTPLSSSTSSNNLSRSPNKSSNVLSTHSPSRQVDPGFSTPAARM  240

Query  241  RYPVVDFEIEDPDVLGAGPGLGELVEENVLEIEEEDDEIEEMNRGFVDEPFELPYDTLDM  300
            RYPVVDFEIEDPDVLGAGPGLGELVEENVLEIEEEDDEIEEMNRGFVDEPFELPYDTLDM
Sbjct  241  RYPVVDFEIEDPDVLGAGPGLGELVEENVLEIEEEDDEIEEMNRGFVDEPFELPYDTLDM  300

Query  301  TDLGQRVRSTAIFLGGSSFFDLPPPVDIVLDDDYRDGSLDLNLKDLNSDEELYVLLNQPR  360
            TDLGQRVRSTAIFLGGSSFFDLPPPVDIVLDDDYRDGSLDLNLKDLNSDEELYVLLNQPR
Sbjct  301  TDLGQRVRSTAIFLGGSSFFDLPPPVDIVLDDDYRDGSLDLNLKDLNSDEELYVLLNQPR  360

Query  361  CPTPSTISARANSNSRDSRSTCIGFSSLPPSVLSTTKRDAPSSASNHLSTVRAPSSTPSV  420
            CPTPSTISARANSNSRDSRSTCIGFSSLPPSVLSTTKRDAPSSASNHLSTVRAPSSTPSV
Sbjct  361  CPTPSTISARANSNSRDSRSTCIGFSSLPPSVLSTTKRDAPSSASNHLSTVRAPSSTPSV  420

Query  421  SRRPLSSVSTSSKSSATATTKTQNGPPPTIRPLSIMSSTTPSASSLSGSKIVRPGAPSAL  480
            SRRPLSSVSTSSKSSATATTKTQNGPPPTIRPLSIMSSTTPSASSLSGSKIVRPGAPSAL
Sbjct  421  SRRPLSSVSTSSKSSATATTKTQNGPPPTIRPLSIMSSTTPSASSLSGSKIVRPGAPSAL  480

Query  481  RQSRATPEQKGVSSGSARLNGKSKWAALQARDHRLGRELLAGLATELDQDQTHDDLFEGF  540
            RQSRATPEQKGVSSGSARLNGKSKWAALQARDHRLGRELLAGLATELDQDQTHDDLFEGF
Sbjct  481  RQSRATPEQKGVSSGSARLNGKSKWAALQARDHRLGRELLAGLATELDQDQTHDDLFEGF  540

Query  541  EF  542
            EF
Sbjct  541  EF  542


>ref|XP_772482.1| hypothetical protein CNBL0970 [Cryptococcus neoformans var. neoformans 
B-3501A]
 gb|EAL17835.1| hypothetical protein CNBL0970 [Cryptococcus neoformans var. neoformans 
B-3501A]
Length=296

 Score = 50.4 bits (119),  Expect = 0.002, Method: Compositional matrix adjust.
 Identities = 66/226 (29%), Positives = 96/226 (42%), Gaps = 58/226 (26%)

Query  2    LHRSLAHTFH-----DKENTYHLPSRTPLRIGLAGSSFATAGARTVGRPNVKTPFTKTSV  56
            +HR+  H        +KEN + LPS+TP RIG         G + +G      P T   +
Sbjct  9    IHRTYIHPTTSKGNVNKENAHALPSKTPSRIG---------GKQLIG------PATGMRM  53

Query  57   LLGGKPPQTTQTGRGKEKIWTEGGVNLGNSKTGEGKDGQMIEPRRLFTLQTNRGSPKASG  116
             LG K       GR +  +  +G         G+G++   IEP+RLF             
Sbjct  54   ALGAK-----TEGRDRNVLRQQG------EGKGKGREVDDIEPKRLF-------------  89

Query  117  TLQLNSSSKDGALAKPNSNQPP-PTLKKSRPAPTPQHVLRTPHPTQRTSLGQSHSPTQQL  175
               +NSS      +K  S+ PP P+L    PAP      R   P+Q  +L ++ +PT + 
Sbjct  90   ---VNSSKDSIPPSKSLSSMPPIPSLPTRTPAP------RRIAPSQSQTL-RTPAPTFKF  139

Query  176  TNILSTPLPSTNRTRRRSKQLHTPLSSSTSSNNLSRSPNKSSNVLS  221
                 TPLPS  RT RRS+Q    LSS+    +L     K    ++
Sbjct  140  VEPQPTPLPSATRTGRRSRQS---LSSTPGKGDLGLDKQKGQQFVT  182


>ref|XP_007864297.1| hypothetical protein GLOTRDRAFT_137554 [Gloeophyllum trabeum 
ATCC 11539]
 gb|EPQ57153.1| hypothetical protein GLOTRDRAFT_137554 [Gloeophyllum trabeum 
ATCC 11539]
Length=456

 Score = 47.4 bits (111),  Expect = 0.028, Method: Compositional matrix adjust.
 Identities = 60/219 (27%), Positives = 100/219 (46%), Gaps = 40/219 (18%)

Query  265  VEENVLEIEEEDDEIEEMNRGFVDEPFELPYDTLDMTDLGQRVRSTAIFLGGSSFFDLPP  324
             E+  +E+E+ DDEIE M    +  P+E P++  D   LG+ +  TA       + D PP
Sbjct  178  AEQEAVEVED-DDEIEYMPPTAIIPPYEPPFEMPDYKVLGKELLQTAF---SYPYDDGPP  233

Query  325  P-VDI-VLDDDY---RDGSLDLNLKDLNSDEELYVLLNQPRCPTPSTISARANSNSRDSR  379
            P +D+ V D+ +    +   +L L +L+ D  L      P    PS+ SA  +S++R +R
Sbjct  234  PALDLSVYDEKFFASMEAECELELPELDDDNLL------PAEKRPSS-SATKSSDTRTTR  286

Query  380  STCIGFSSLPPSVLSTTKRDAPSSASNHLSTVRAPS---STPSVSRRPLSSVSTSSKSSA  436
            S  +  SS      +TT R +P +A       RA +   +TP  S     ++ST S    
Sbjct  287  SRTVKVSS------NTTSRASPETAPTRRPITRAAAKGGTTPGSSETQPRALSTRS----  336

Query  437  TATTKTQNGPPPTIRPLSIMSSTTPSASSLSGSKIVRPG  475
                       P +R  S+ S+   + S++  + + RP 
Sbjct  337  -----------PLMRATSVASTRPTTRSAVKATAVARPA  364


>gb|ELU42675.1| hypothetical protein AG1IA_03294 [Rhizoctonia solani AG-1 IA]
Length=494

 Score = 41.6 bits (96),  Expect = 2.1, Method: Compositional matrix adjust.
 Identities = 57/209 (27%), Positives = 90/209 (43%), Gaps = 23/209 (11%)

Query  233  FSTPAARMRYPVVDFEIEDPDVLGAGPGLGELVEENVLEIEEEDDEIEEMNRGFVDEPFE  292
            F TP AR R    D     PD +  G  L   +E  V ++++E++E+E M    +   +E
Sbjct  156  FQTPDARGRPAHWDVGDIGPD-MDEGVQLSNDIEAKVRQVQDEEEELEYMPPTAIVPEYE  214

Query  293  LPYDTLDMTDLGQRVRSTAIFLGGSSFFDLPPPVDIVLDDDYRD-GSLDLNLKDLNSDEE  351
              ++  D+   GQ V S        S++   P  +I + +  RD G +D    DL S  +
Sbjct  215  PLFEMPDLKVFGQTVYSLT-----HSYW---PKDEINVLNAIRDEGIIDARF-DLPSSHD  265

Query  352  LYVLLNQPRCPTPSTIS--------ARANSNSRDSRSTCIGFSSLPPSVLSTTKRD----  399
            L +L      P P  IS         R  S S  +RS   G     P+ ++  + D    
Sbjct  266  LSILDPPEDNPFPVRISFADSKKPETRGRSASVSARSGMSGIVRARPASVADVRTDNISR  325

Query  400  APSSASNHLSTVRAPSSTPSVSRRPLSSV  428
            APS  S+   +   P + P++S R  S+V
Sbjct  326  APSRTSSRPGSTVPPRTNPAISARSTSTV  354


>emb|CDH49422.1| hypothetical protein RO3G_13248 [Lichtheimia corymbifera JMRC:FSU:9682]
Length=921

 Score = 40.0 bits (92),  Expect = 8.2, Method: Compositional matrix adjust.
 Identities = 51/164 (31%), Positives = 80/164 (49%), Gaps = 19/164 (12%)

Query  349  DEELYVLLNQPRCPT------PSTISARANSNSRDSRSTC------IGFSSLPPSVLSTT  396
            D++L V  +  R P+       STI+  A ++S  ++  C      I  S+ P    + T
Sbjct  185  DDDLDVKFDSARSPSRHHQQKQSTITTNATTSSTRAKHCCSGKRHNITTSATPKHEQNAT  244

Query  397  KRDAPSSASNHLSTVRAPSSTP--SVSRRPLSSVSTSSKSSATATTKTQNGPPPTIRPLS  454
             R + SS +N   T  APS++   S++ R  S   TSSKS+A  TT ++   P  +R  S
Sbjct  245  MRKSKSSNTN--VTTEAPSTSQQRSIASRRTSIHPTSSKSTAMGTTVSKQQRPTMLRKSS  302

Query  455  IMSSTTPSASSLSGSKIVRPGAPSALRQSRATPEQKGVSSGSAR  498
            + SST  +   ++   + R    S LR+S + P Q+  SS S R
Sbjct  303  VPSSTKSTV--IASPALRRASEGSVLRKS-SVPHQRSSSSLSTR  343


Lambda      K        H        a         alpha
   0.310    0.127    0.363    0.792     4.96 

Gapped
Lambda      K        H        a         alpha    sigma
   0.267   0.0410    0.140     1.90     42.6     43.6 

Effective search space used: 5763081237795


  Database: nr
    Posted date:  Sep 23, 2015 12:05 AM
  Number of letters in database: 26,053,659,533
  Number of sequences in database:  71,551,133


Matrix: BLOSUM62
Gap Penalties: Existence: 11, Extension: 1
Neighboring words threshold: 11
Window for multiple hits: 40
```
