# Supplementary material for: Comparative genomics provides new insights into the diversity, physiology, and sexuality of the only industrially exploited tremellomycete: Phaffia rhodozyma
Source: BMC Genomics. 2016 Nov 9;17:901. doi: 10.1186/s12864-016-3244-7 (PMC5103461; doi:10.1186/s12864-016-3244-7)
Supplement: Additional file 6: — List of orphan genes with links to PFAM (related to Additional file 1: Table S1). (ZIP 1428 kb) [file 12864_2016_3244_MOESM6_ESM.zip › BLAST_HTML_FTR/G02711_P.html]

BLAST Search Results


```
BLASTP 2.2.27+


Reference:
Stephen F. Altschul, Thomas L. Madden, Alejandro A. Schäffer,
Jinghui Zhang, Zheng Zhang, Webb Miller, and David J. Lipman (1997),
"Gapped BLAST and PSI-BLAST: a new generation of protein database
search programs", Nucleic Acids Res. 25:3389-3402.


Reference for
composition-based statistics:
Alejandro A. Schäffer, L. Aravind, Thomas L. Madden, Sergei
Shavirin, John L. Spouge, Yuri I. Wolf, Eugene V. Koonin, and
Stephen F. Altschul (2001), "Improving the accuracy of PSI-BLAST
protein database searches with composition-based statistics and
other refinements", Nucleic Acids Res. 29:2994-3005.


Database: nr
           71,551,133 sequences; 26,053,659,533 total letters


Query= G02711_P

Length=109
                                                                      Score     E
Sequences producing significant alignments:                          (Bits)  Value

emb|CED84975.1|  hypothetical protein [Xanthophyllomyces dendrorh...   222    4e-72
ref|XP_003880657.1|  hypothetical protein NCLIV_010920 [Neospora ...  37.7    1.4  
gb|KIK70351.1|  hypothetical protein GYMLUDRAFT_32354 [Gymnopus l...  35.4    5.6  


 >emb|CED84975.1| hypothetical protein [Xanthophyllomyces dendrorhous]
Length=108

 Score =  222 bits (565),  Expect = 4e-72, Method: Compositional matrix adjust.
 Identities = 108/108 (100%), Positives = 108/108 (100%), Gaps = 0/108 (0%)

Query  1    MNALFNTPSHIIFTPFAPMPVRPARDEPLPVPFFRRIKMTKASFKDVSSAKPTKTSSSQY  60
            MNALFNTPSHIIFTPFAPMPVRPARDEPLPVPFFRRIKMTKASFKDVSSAKPTKTSSSQY
Sbjct  1    MNALFNTPSHIIFTPFAPMPVRPARDEPLPVPFFRRIKMTKASFKDVSSAKPTKTSSSQY  60

Query  61   SKSVESSQTDVKEKALKRERRSVSNCSSLDMRELEGAYWENAAKKMGI  108
            SKSVESSQTDVKEKALKRERRSVSNCSSLDMRELEGAYWENAAKKMGI
Sbjct  61   SKSVESSQTDVKEKALKRERRSVSNCSSLDMRELEGAYWENAAKKMGI  108


>ref|XP_003880657.1| hypothetical protein NCLIV_010920 [Neospora caninum Liverpool]
 emb|CBZ50624.1| hypothetical protein NCLIV_010920 [Neospora caninum Liverpool]
 tpe|CEL65236.1| TPA: RNA-directed RNA polymerase [Neospora caninum Liverpool]
Length=1244

 Score = 37.7 bits (86),  Expect = 1.4, Method: Compositional matrix adjust.
 Identities = 23/60 (38%), Positives = 33/60 (55%), Gaps = 0/60 (0%)

Query  29   LPVPFFRRIKMTKASFKDVSSAKPTKTSSSQYSKSVESSQTDVKEKALKRERRSVSNCSS  88
            L   F+R I   K   K+ SSA PT  S S  +K+ ++SQT  KE  L+ E+R   + +S
Sbjct  92   LSFDFYRDIHDEKCRRKETSSAAPTPASRSSVAKTGDASQTTGKESVLEPEQRGGGHSTS  151


>gb|KIK70351.1| hypothetical protein GYMLUDRAFT_32354 [Gymnopus luxurians FD-317 
M1]
Length=248

 Score = 35.4 bits (80),  Expect = 5.6, Method: Compositional matrix adjust.
 Identities = 31/91 (34%), Positives = 44/91 (48%), Gaps = 10/91 (11%)

Query  12   IFTPFAPMPVRPARDEPLPVPFFRRIKMTKASFKDVSSAKPTKTSSSQYSKSVESSQTDV  71
            I T  A   V PA   P PV    +  MT+A +    S KP K + SQ S+ ++  Q+D 
Sbjct  112  IATLVAEKCVDPATQRPYPVGMIEK-AMTEAGY----SLKPNKNAKSQVSECIKILQSDS  166

Query  72   K---EKALKRERRSVSNCSSLDMRE--LEGA  97
                ++A  R R ++ N     +RE  LEGA
Sbjct  167  TLPIQRARMRVRVTMPNADGKRLREKILEGA  197


Lambda      K        H        a         alpha
   0.319    0.129    0.374    0.792     4.96 

Gapped
Lambda      K        H        a         alpha    sigma
   0.267   0.0410    0.140     1.90     42.6     43.6 

Effective search space used: 657415113344


  Database: nr
    Posted date:  Sep 23, 2015 12:05 AM
  Number of letters in database: 26,053,659,533
  Number of sequences in database:  71,551,133


Matrix: BLOSUM62
Gap Penalties: Existence: 11, Extension: 1
Neighboring words threshold: 11
Window for multiple hits: 40
```
