# Supplementary material for: Comparative genomics provides new insights into the diversity, physiology, and sexuality of the only industrially exploited tremellomycete: Phaffia rhodozyma
Source: BMC Genomics. 2016 Nov 9;17:901. doi: 10.1186/s12864-016-3244-7 (PMC5103461; doi:10.1186/s12864-016-3244-7)
Supplement: Additional file 6: — List of orphan genes with links to PFAM (related to Additional file 1: Table S1). (ZIP 1428 kb) [file 12864_2016_3244_MOESM6_ESM.zip › BLAST_HTML_FTR/G00410_P.html]

BLAST Search Results


```
BLASTP 2.2.27+


Reference:
Stephen F. Altschul, Thomas L. Madden, Alejandro A. Schäffer,
Jinghui Zhang, Zheng Zhang, Webb Miller, and David J. Lipman (1997),
"Gapped BLAST and PSI-BLAST: a new generation of protein database
search programs", Nucleic Acids Res. 25:3389-3402.


Reference for
composition-based statistics:
Alejandro A. Schäffer, L. Aravind, Thomas L. Madden, Sergei
Shavirin, John L. Spouge, Yuri I. Wolf, Eugene V. Koonin, and
Stephen F. Altschul (2001), "Improving the accuracy of PSI-BLAST
protein database searches with composition-based statistics and
other refinements", Nucleic Acids Res. 29:2994-3005.


Database: nr
           71,551,133 sequences; 26,053,659,533 total letters


Query= G00410_P

Length=215
                                                                      Score     E
Sequences producing significant alignments:                          (Bits)  Value

emb|CED82872.1|  hypothetical protein [Xanthophyllomyces dendrorh...   434    2e-152
ref|WP_038009319.1|  beta-ketoacyl synthase [Tannerella sp. oral ...  37.4    9.7   


 >emb|CED82872.1| hypothetical protein [Xanthophyllomyces dendrorhous]
Length=214

 Score =  434 bits (1115),  Expect = 2e-152, Method: Compositional matrix adjust.
 Identities = 214/214 (100%), Positives = 214/214 (100%), Gaps = 0/214 (0%)

Query  1    MSGYQTARSLPTITKNDPLFGSSTSANRLCLGFDSEPRKSAPDGQVGRKRRLISFEDGET  60
            MSGYQTARSLPTITKNDPLFGSSTSANRLCLGFDSEPRKSAPDGQVGRKRRLISFEDGET
Sbjct  1    MSGYQTARSLPTITKNDPLFGSSTSANRLCLGFDSEPRKSAPDGQVGRKRRLISFEDGET  60

Query  61   MPTATTTASSSLSRTQTESTSYLLSFPDPFHSIPFYYATPPIKRPNQLSDPTASDTVVPN  120
            MPTATTTASSSLSRTQTESTSYLLSFPDPFHSIPFYYATPPIKRPNQLSDPTASDTVVPN
Sbjct  61   MPTATTTASSSLSRTQTESTSYLLSFPDPFHSIPFYYATPPIKRPNQLSDPTASDTVVPN  120

Query  121  SFSSASSNSSLSNVSDFAPRTPKEIVRQVRQVRPSIEKKDDTTDTVSDWLSGMDGLDERE  180
            SFSSASSNSSLSNVSDFAPRTPKEIVRQVRQVRPSIEKKDDTTDTVSDWLSGMDGLDERE
Sbjct  121  SFSSASSNSSLSNVSDFAPRTPKEIVRQVRQVRPSIEKKDDTTDTVSDWLSGMDGLDERE  180

Query  181  ATAETESTRPTERPSHHSAIHQTGRGRKGGRDFW  214
            ATAETESTRPTERPSHHSAIHQTGRGRKGGRDFW
Sbjct  181  ATAETESTRPTERPSHHSAIHQTGRGRKGGRDFW  214


>ref|WP_038009319.1| beta-ketoacyl synthase [Tannerella sp. oral taxon BU063]
 gb|ETK02935.1| beta-ketoacyl synthase [Tannerella sp. oral taxon BU063 isolate 
Cell 2]
Length=411

 Score = 37.4 bits (85),  Expect = 9.7, Method: Compositional matrix adjust.
 Identities = 31/110 (28%), Positives = 46/110 (42%), Gaps = 9/110 (8%)

Query  31   LGFDSEPRKSAPDGQVGRKRRLISFEDGETMPTATTTASSSLSRT----QTESTSYLLSF  86
            +G+ +    S P G     R L +  DG TMP  T+T S +         +E    LL  
Sbjct  293  IGYINAHATSTPVGDRNEARALTALFDGHTMPPVTSTKSMTGHEMWMAGASEVVYSLLMM  352

Query  87   PDPFHSIPFYYATP-----PIKRPNQLSDPTASDTVVPNSFSSASSNSSL  131
               F +    +  P     P++ P +  D  A DT + NSF    +NS+L
Sbjct  353  RGQFIAPHLNFEHPDDDTAPLRIPTERLDDYAFDTFLSNSFGFGGTNSTL  402


Lambda      K        H        a         alpha
   0.313    0.127    0.370    0.792     4.96 

Gapped
Lambda      K        H        a         alpha    sigma
   0.267   0.0410    0.140     1.90     42.6     43.6 

Effective search space used: 1118271043051


  Database: nr
    Posted date:  Sep 23, 2015 12:05 AM
  Number of letters in database: 26,053,659,533
  Number of sequences in database:  71,551,133


Matrix: BLOSUM62
Gap Penalties: Existence: 11, Extension: 1
Neighboring words threshold: 11
Window for multiple hits: 40
```
