# Supplementary material for: Comparative genomics provides new insights into the diversity, physiology, and sexuality of the only industrially exploited tremellomycete: Phaffia rhodozyma
Source: BMC Genomics. 2016 Nov 9;17:901. doi: 10.1186/s12864-016-3244-7 (PMC5103461; doi:10.1186/s12864-016-3244-7)
Supplement: Additional file 6: — List of orphan genes with links to PFAM (related to Additional file 1: Table S1). (ZIP 1428 kb) [file 12864_2016_3244_MOESM6_ESM.zip › BLAST_HTML_FTR/G02395_P.html]

BLAST Search Results


```
BLASTP 2.2.27+


Reference:
Stephen F. Altschul, Thomas L. Madden, Alejandro A. Schäffer,
Jinghui Zhang, Zheng Zhang, Webb Miller, and David J. Lipman (1997),
"Gapped BLAST and PSI-BLAST: a new generation of protein database
search programs", Nucleic Acids Res. 25:3389-3402.


Reference for
composition-based statistics:
Alejandro A. Schäffer, L. Aravind, Thomas L. Madden, Sergei
Shavirin, John L. Spouge, Yuri I. Wolf, Eugene V. Koonin, and
Stephen F. Altschul (2001), "Improving the accuracy of PSI-BLAST
protein database searches with composition-based statistics and
other refinements", Nucleic Acids Res. 29:2994-3005.


Database: nr
           71,551,133 sequences; 26,053,659,533 total letters


Query= G02395_P

Length=327
                                                                      Score     E
Sequences producing significant alignments:                          (Bits)  Value

emb|CDZ98743.1|  hypothetical protein [Xanthophyllomyces dendrorh...   592    0.0  
gb|EUC54226.1|  hypothetical protein RSOL_033600 [Rhizoctonia sol...  49.7    0.002
gb|KEP51915.1|  hypothetical protein V565_053490 [Rhizoctonia sol...  49.3    0.002
ref|XP_002475721.1|  predicted protein [Postia placenta Mad-698-R...  42.4    0.35 
ref|WP_010630180.1|  5-hydroxymethyluracil DNA glycosylase [Halom...  40.8    1.1  
emb|CUA76614.1|  hypothetical protein RSOLAG22IIIB_06375 [Rhizoct...  39.3    3.6  
emb|CEL59064.1|  hypothetical protein RSOLAG1IB_09051 [Rhizoctoni...  38.5    9.7  


 >emb|CDZ98743.1| hypothetical protein [Xanthophyllomyces dendrorhous]
Length=299

 Score =  592 bits (1526),  Expect = 0.0, Method: Compositional matrix adjust.
 Identities = 298/326 (91%), Positives = 299/326 (92%), Gaps = 27/326 (8%)

Query  1    MLFPAAFTVACLALVNTLVDASPTVTSSSSFIHAPRSEVEAHQKKSSRMIPLPRWLQDPK  60
            MLFPAAFTVACLALVNTLVDASPTVTSSSSFIHAPRSEVEAHQKKSSRMIPLPRWLQDPK
Sbjct  1    MLFPAAFTVACLALVNTLVDASPTVTSSSSFIHAPRSEVEAHQKKSSRMIPLPRWLQDPK  60

Query  61   EASSYLPRDKVVRRAAAQPSVSAVPKTYKGQIYVDFVDKSSTDKSGYLSSYLDTFGQYGT  120
            EASSYLPRDKVVRRAAAQPSVSAVPKTYKGQIYVDFVDKSSTDKSGYLSSYLDTFGQYGT
Sbjct  61   EASSYLPRDKVVRRAAAQPSVSAVPKTYKGQIYVDFVDKSSTDKSGYLSSYLDTFGQYGT  120

Query  121  IVDNKTTPLIVDFKYGKNESTNIEIKNGEKDYTDLGLVLAYGLKGADFGPKNPKHVSPFV  180
            IVDNKTTPLIVDFKYGKNESTNIEIKNGEKDYTDLGLVLAYGLKGADFGPKNP       
Sbjct  121  IVDNKTTPLIVDFKYGKNESTNIEIKNGEKDYTDLGLVLAYGLKGADFGPKNP-------  173

Query  181  FQTRSDYYRLTVTLCSRLISSVLVLAGVSQRTVPITGGSGDTLKSTVGLSTGFNLPAQSK  240
                                +VLVLAGVSQRTVPITGGSGDTLKSTVGLSTGFNLPAQSK
Sbjct  174  --------------------NVLVLAGVSQRTVPITGGSGDTLKSTVGLSTGFNLPAQSK  213

Query  241  VWVGDNVVFKKCEGDILIPYWIDLAGEAIKGKLYYVEFDDGEKPTIIVTTDVTLLPKDHV  300
            VWVGDNVVFKKCEGDILIPYWIDLAGEAIKGKLYYVEFDDGEKPTIIVTTDVTLLPKDHV
Sbjct  214  VWVGDNVVFKKCEGDILIPYWIDLAGEAIKGKLYYVEFDDGEKPTIIVTTDVTLLPKDHV  273

Query  301  KFFEIVLLYVSKPDVCYPKDYFSGLN  326
            KFFEIVLLYVSKPDVCYPKDYFSGLN
Sbjct  274  KFFEIVLLYVSKPDVCYPKDYFSGLN  299


>gb|EUC54226.1| hypothetical protein RSOL_033600 [Rhizoctonia solani AG-3 Rhs1AP]
Length=295

 Score = 49.7 bits (117),  Expect = 0.002, Method: Compositional matrix adjust.
 Identities = 59/246 (24%), Positives = 93/246 (38%), Gaps = 46/246 (19%)

Query  70   KVVRRAAAQP--SVSAVPKTYKGQIYVDFVDKSSTDKSGYLSSYLDTFGQYGTIVDNKTT  127
            K   RAA+ P   +S +P     Q   + + K++    GYL+  L+ FGQYG     +  
Sbjct  84   KRATRAASAPRAEISPIPPV---QRSCNILAKAADTTLGYLAPRLNIFGQYGVFQAGQAG  140

Query  128  PLIVDFKY--GKNESTNIEIKNGE-KDYTDLGLVLAYGLKGADFGPKNPKHVSPFVFQTR  184
             L V   Y  G + S ++   NG  K Y  +G  + Y  +  + GP    H   +V  TR
Sbjct  141  ALEVSISYVPGSDASVDLVPTNGPTKAYPYMGAAVGYASESGNLGPGTQSHA--YVAATR  198

Query  185  SDYYRLTVTLCSRLISSVLVLAGVSQRTVPITGGSGDTLKSTVGLSTGFNLPAQSKVWVG  244
                                      R+ P++G       ++V ++TG     +S +WV 
Sbjct  199  Q----------------------TPPRSPPVSG------DNSVSVATGIPADYESAIWVY  230

Query  245  DNVVFKKCEGDILIPYWIDLAGEAIKGKLYYVEFDDGEKPTIIVTTDVTLLPKDHVKFFE  304
            D +  K      +   W++  G      L Y   +DG    I+     TL       + E
Sbjct  231  DPLTSK------IRAQWVNTDGSTPVTHLLYS--NDGSDALILTGDPDTLNSAFGSNYPE  282

Query  305  IVLLYV  310
            I L  V
Sbjct  283  ITLTCV  288


>gb|KEP51915.1| hypothetical protein V565_053490 [Rhizoctonia solani 123E]
Length=295

 Score = 49.3 bits (116),  Expect = 0.002, Method: Compositional matrix adjust.
 Identities = 59/246 (24%), Positives = 93/246 (38%), Gaps = 46/246 (19%)

Query  70   KVVRRAAAQP--SVSAVPKTYKGQIYVDFVDKSSTDKSGYLSSYLDTFGQYGTIVDNKTT  127
            K   RAA+ P   +S +P     Q   + + K++    GYL+  L+ FGQYG     +  
Sbjct  84   KRATRAASAPRAEISPIPPV---QRSCNILAKAADTTLGYLAPRLNIFGQYGVFQAGQAG  140

Query  128  PLIVDFKY--GKNESTNIEIKNGE-KDYTDLGLVLAYGLKGADFGPKNPKHVSPFVFQTR  184
             L V   Y  G + S ++   NG  K Y  +G  + Y  +  + GP    H   +V  TR
Sbjct  141  ALEVSISYVPGSDASVDLVPTNGPTKAYPYMGAAVGYASESGNLGPGTQSHA--YVAATR  198

Query  185  SDYYRLTVTLCSRLISSVLVLAGVSQRTVPITGGSGDTLKSTVGLSTGFNLPAQSKVWVG  244
                                      R+ P++G       ++V ++TG     +S +WV 
Sbjct  199  Q----------------------TPPRSPPVSG------DNSVSVATGIPADYESAIWVY  230

Query  245  DNVVFKKCEGDILIPYWIDLAGEAIKGKLYYVEFDDGEKPTIIVTTDVTLLPKDHVKFFE  304
            D +  K      +   W++  G      L Y   +DG    I+     TL       + E
Sbjct  231  DPLTSK------IRAQWVNTDGSTPVTHLLYS--NDGSDALILTGDPDTLNSAFGSNYPE  282

Query  305  IVLLYV  310
            I L  V
Sbjct  283  ITLTCV  288


>ref|XP_002475721.1| predicted protein [Postia placenta Mad-698-R]
 gb|EED79079.1| predicted protein [Postia placenta Mad-698-R]
Length=281

 Score = 42.4 bits (98),  Expect = 0.35, Method: Compositional matrix adjust.
 Identities = 32/119 (27%), Positives = 49/119 (41%), Gaps = 4/119 (3%)

Query  52   LPRWLQDPKEASSYLPRDKVVRRAAAQPSVSAVPKTYKGQIYVDFVDKSSTDKSGYLSSY  111
            L R L   +   ++  R  +  RA+A P  +  P T  G I V     + T  S ++S  
Sbjct  65   LARGLAPNRPRFNHAARRGLAPRASAAPDPNPCP-TMTGTIRVA---GAGTSASTFVSRV  120

Query  112  LDTFGQYGTIVDNKTTPLIVDFKYGKNESTNIEIKNGEKDYTDLGLVLAYGLKGADFGP  170
             + FG+YG   D     L+         S ++   NG  D+T LG +  +     D GP
Sbjct  121  PNVFGEYGVTTDAANALLVQYANCAGAASADLVTLNGIADFTHLGGITGFSSPSGDLGP  179


>ref|WP_010630180.1| 5-hydroxymethyluracil DNA glycosylase [Halomonas sp. KM-1]
Length=277

 Score = 40.8 bits (94),  Expect = 1.1, Method: Compositional matrix adjust.
 Identities = 23/62 (37%), Positives = 32/62 (52%), Gaps = 2/62 (3%)

Query  58   DPKEASSYLPRDKVVRRAAAQPSVSAVPKTYKGQIYVDFVDKSSTDKSGYLSSYLDTFGQ  117
            DP+ A+  + RD+  R AAA   V A   T  G    DFV  S T + GY    L+ +G+
Sbjct  190  DPRRAAGRISRDRYERLAAAIREVLAAAITQGGTTLRDFV--SGTGEPGYFKQRLNVYGR  247

Query  118  YG  119
            +G
Sbjct  248  HG  249


>emb|CUA76614.1| hypothetical protein RSOLAG22IIIB_06375 [Rhizoctonia solani]
Length=287

 Score = 39.3 bits (90),  Expect = 3.6, Method: Compositional matrix adjust.
 Identities = 24/74 (32%), Positives = 33/74 (45%), Gaps = 3/74 (4%)

Query  106  GYLSSYLDTFGQYGTIVDNKTTPLIVDFKYGKNESTNIE--IKNGE-KDYTDLGLVLAYG  162
            GY+   L  FGQYG    N+   L V F Y     T ++    NG    Y  +G  + Y 
Sbjct  112  GYIGRELILFGQYGLFESNQRAALEVSFSYSPGSDTPVDWHATNGPTAAYPYVGGAVGYY  171

Query  163  LKGADFGPKNPKHV  176
              GA+ GP +  H+
Sbjct  172  SNGANLGPGSSNHI  185


>emb|CEL59064.1| hypothetical protein RSOLAG1IB_09051 [Rhizoctonia solani AG-1 
IB]
Length=387

 Score = 38.5 bits (88),  Expect = 9.7, Method: Compositional matrix adjust.
 Identities = 24/75 (32%), Positives = 43/75 (57%), Gaps = 3/75 (4%)

Query  89   KGQIYVDFVDKSSTDKSGYLSSYLDTFGQYGTIVDNKTTPLIVDFKYGKNESTNIEIK-N  147
            +G I V+ V +      GYLSS  + +G+YGT+ D  T  L V F+  +++S  ++++  
Sbjct  203  QGTITVEVVSEGG-KPLGYLSSQRNAYGEYGTLQDTATGALSVTFRPSRSKSNQLDLRVA  261

Query  148  GEK-DYTDLGLVLAY  161
            GE+ D   LG ++ +
Sbjct  262  GERADMAMLGGIIGF  276


Lambda      K        H        a         alpha
   0.319    0.137    0.406    0.792     4.96 

Gapped
Lambda      K        H        a         alpha    sigma
   0.267   0.0410    0.140     1.90     42.6     43.6 

Effective search space used: 2711815156191


  Database: nr
    Posted date:  Sep 23, 2015 12:05 AM
  Number of letters in database: 26,053,659,533
  Number of sequences in database:  71,551,133


Matrix: BLOSUM62
Gap Penalties: Existence: 11, Extension: 1
Neighboring words threshold: 11
Window for multiple hits: 40
```
