# Supplementary material for: Comparative genomics provides new insights into the diversity, physiology, and sexuality of the only industrially exploited tremellomycete: Phaffia rhodozyma
Source: BMC Genomics. 2016 Nov 9;17:901. doi: 10.1186/s12864-016-3244-7 (PMC5103461; doi:10.1186/s12864-016-3244-7)
Supplement: Additional file 6: — List of orphan genes with links to PFAM (related to Additional file 1: Table S1). (ZIP 1428 kb) [file 12864_2016_3244_MOESM6_ESM.zip › BLAST_HTML_FTR/G04721_P.html]

BLAST Search Results


```
BLASTP 2.2.27+


Reference:
Stephen F. Altschul, Thomas L. Madden, Alejandro A. Schäffer,
Jinghui Zhang, Zheng Zhang, Webb Miller, and David J. Lipman (1997),
"Gapped BLAST and PSI-BLAST: a new generation of protein database
search programs", Nucleic Acids Res. 25:3389-3402.


Reference for
composition-based statistics:
Alejandro A. Schäffer, L. Aravind, Thomas L. Madden, Sergei
Shavirin, John L. Spouge, Yuri I. Wolf, Eugene V. Koonin, and
Stephen F. Altschul (2001), "Improving the accuracy of PSI-BLAST
protein database searches with composition-based statistics and
other refinements", Nucleic Acids Res. 29:2994-3005.


Database: nr
           71,551,133 sequences; 26,053,659,533 total letters


Query= G04721_P

Length=362
                                                                      Score     E
Sequences producing significant alignments:                          (Bits)  Value

emb|CDZ97706.1|  hypothetical protein [Xanthophyllomyces dendrorh...   627    0.0  


 >emb|CDZ97706.1| hypothetical protein [Xanthophyllomyces dendrorhous]
Length=361

 Score =  627 bits (1616),  Expect = 0.0, Method: Compositional matrix adjust.
 Identities = 345/361 (96%), Positives = 345/361 (96%), Gaps = 16/361 (4%)

Query  1    MVSTTNTKGLSSGPSIRGGIGIWGACLEQANLTRSSQLRLCTFASPLSVPKLVLLPGPLP  60
            MVSTTNTKGLSSGPSIRGGIGIWGACLEQANLTRSSQLRLCTFASPLSVPKLVLLPGPLP
Sbjct  1    MVSTTNTKGLSSGPSIRGGIGIWGACLEQANLTRSSQLRLCTFASPLSVPKLVLLPGPLP  60

Query  61   TLPSYLPPYFSPVLSFFLVLALACALIQHALRSYILWGEIAEYGRISTGGKKDDLSDGSP  120
            TLPSYLPPYFSPVLSFFLVLALACALIQHALRSYILWGEIAEYGRISTGGKKDDLSDGSP
Sbjct  61   TLPSYLPPYFSPVLSFFLVLALACALIQHALRSYILWGEIAEYGRISTGGKKDDLSDGSP  120

Query  121  SYQPDAHDGPNRFGGSRWKNMWET----------------IGILLFPLLPSIYILTSAIS  164
            SYQPDAHDGPNRFGGSRWKNMWET                IGILLFPLLPSIYILTSAIS
Sbjct  121  SYQPDAHDGPNRFGGSRWKNMWETVSREIAVVTAQSCIIVIGILLFPLLPSIYILTSAIS  180

Query  165  SIEGDIVTSMLFVPGWGTALLVLSVICTLNLRSSTSRSHSRSRFGRSTSGHCRRSSMLPF  224
            SIEGDIVTSMLFVPGWGTALLVLSVICTLNLRSSTSRSHSRSRFGRSTSGHCRRSSMLPF
Sbjct  181  SIEGDIVTSMLFVPGWGTALLVLSVICTLNLRSSTSRSHSRSRFGRSTSGHCRRSSMLPF  240

Query  225  TEIKALQPHNGHDQDVQSNSQTNADREVDNLDPPLYRSIHPLPLPTPSSRRKDSPSQGTT  284
            TEIKALQPHNGHDQDVQSNSQTNADREVDNLDPPLYRSIHPLPLPTPSSRRKDSPSQGTT
Sbjct  241  TEIKALQPHNGHDQDVQSNSQTNADREVDNLDPPLYRSIHPLPLPTPSSRRKDSPSQGTT  300

Query  285  RLHSLSSDQNRTQMNEQKEQSQPKAFWVPVSAFAEMSEGEADASETINNEGMQVVQVEIW  344
            RLHSLSSDQNRTQMNEQKEQSQPKAFWVPVSAFAEMSEGEADASETINNEGMQVVQVEIW
Sbjct  301  RLHSLSSDQNRTQMNEQKEQSQPKAFWVPVSAFAEMSEGEADASETINNEGMQVVQVEIW  360

Query  345  A  345
            A
Sbjct  361  A  361


Lambda      K        H        a         alpha
   0.318    0.134    0.411    0.792     4.96 

Gapped
Lambda      K        H        a         alpha    sigma
   0.267   0.0410    0.140     1.90     42.6     43.6 

Effective search space used: 3187356336570


  Database: nr
    Posted date:  Sep 23, 2015 12:05 AM
  Number of letters in database: 26,053,659,533
  Number of sequences in database:  71,551,133


Matrix: BLOSUM62
Gap Penalties: Existence: 11, Extension: 1
Neighboring words threshold: 11
Window for multiple hits: 40
```
