# Supplementary material for: Comparative genomics provides new insights into the diversity, physiology, and sexuality of the only industrially exploited tremellomycete: Phaffia rhodozyma
Source: BMC Genomics. 2016 Nov 9;17:901. doi: 10.1186/s12864-016-3244-7 (PMC5103461; doi:10.1186/s12864-016-3244-7)
Supplement: Additional file 6: — List of orphan genes with links to PFAM (related to Additional file 1: Table S1). (ZIP 1428 kb) [file 12864_2016_3244_MOESM6_ESM.zip › BLAST_HTML_FTR/G01776_P.html]

BLAST Search Results


```
BLASTP 2.2.27+


Reference:
Stephen F. Altschul, Thomas L. Madden, Alejandro A. Schäffer,
Jinghui Zhang, Zheng Zhang, Webb Miller, and David J. Lipman (1997),
"Gapped BLAST and PSI-BLAST: a new generation of protein database
search programs", Nucleic Acids Res. 25:3389-3402.


Reference for
composition-based statistics:
Alejandro A. Schäffer, L. Aravind, Thomas L. Madden, Sergei
Shavirin, John L. Spouge, Yuri I. Wolf, Eugene V. Koonin, and
Stephen F. Altschul (2001), "Improving the accuracy of PSI-BLAST
protein database searches with composition-based statistics and
other refinements", Nucleic Acids Res. 29:2994-3005.


Database: nr
           71,551,133 sequences; 26,053,659,533 total letters


Query= G01776_P

Length=277
                                                                      Score     E
Sequences producing significant alignments:                          (Bits)  Value

emb|CDZ96979.1|  hypothetical protein [Xanthophyllomyces dendrorh...   516    0.0  
emb|CDZ96973.1|  hypothetical protein [Xanthophyllomyces dendrorh...  57.0    5e-06
gb|KIJ55993.1|  hypothetical protein M422DRAFT_239170 [Sphaerobol...  37.7    6.2  


 >emb|CDZ96979.1| hypothetical protein [Xanthophyllomyces dendrorhous]
Length=276

 Score =  516 bits (1330),  Expect = 0.0, Method: Compositional matrix adjust.
 Identities = 276/276 (100%), Positives = 276/276 (100%), Gaps = 0/276 (0%)

Query  1    MWINILFFSAFTAFTALIGASTALAVPTPAVVERAAIEDRGLFDQLLGLGTVSQLQAITS  60
            MWINILFFSAFTAFTALIGASTALAVPTPAVVERAAIEDRGLFDQLLGLGTVSQLQAITS
Sbjct  1    MWINILFFSAFTAFTALIGASTALAVPTPAVVERAAIEDRGLFDQLLGLGTVSQLQAITS  60

Query  61   TLKSSVAPTLTQIQTSTSGSGLTNILSNAGLSSSASSTSTASVSTAGLITLLNDLSTLIP  120
            TLKSSVAPTLTQIQTSTSGSGLTNILSNAGLSSSASSTSTASVSTAGLITLLNDLSTLIP
Sbjct  61   TLKSSVAPTLTQIQTSTSGSGLTNILSNAGLSSSASSTSTASVSTAGLITLLNDLSTLIP  120

Query  121  ADQFNAIKTAIQNLPANISNDLYTLIAKVISVVKQIAAAQKSSASLVSSGSNGLLASLLG  180
            ADQFNAIKTAIQNLPANISNDLYTLIAKVISVVKQIAAAQKSSASLVSSGSNGLLASLLG
Sbjct  121  ADQFNAIKTAIQNLPANISNDLYTLIAKVISVVKQIAAAQKSSASLVSSGSNGLLASLLG  180

Query  181  GTSVLTNLQSVITQVVNSLDTINSGVTASTSVAGVDAVAQVFASLKTPTDASLAAIVANS  240
            GTSVLTNLQSVITQVVNSLDTINSGVTASTSVAGVDAVAQVFASLKTPTDASLAAIVANS
Sbjct  181  GTSVLTNLQSVITQVVNSLDTINSGVTASTSVAGVDAVAQVFASLKTPTDASLAAIVANS  240

Query  241  AKIISGFMALLKPLISNLTGLLNQLGLGSLTSTLKF  276
            AKIISGFMALLKPLISNLTGLLNQLGLGSLTSTLKF
Sbjct  241  AKIISGFMALLKPLISNLTGLLNQLGLGSLTSTLKF  276


>emb|CDZ96973.1| hypothetical protein [Xanthophyllomyces dendrorhous]
Length=283

 Score = 57.0 bits (136),  Expect = 5e-06, Method: Compositional matrix adjust.
 Identities = 73/244 (30%), Positives = 126/244 (52%), Gaps = 19/244 (8%)

Query  13   AFTALIGASTALAVPTPAVVERAAIEDRGLFDQLLGLGTVSQLQAITSTLKSSVAPTLTQ  72
            A     GAS+ LA P   V +RA +               S LQ++TS L S+V   ++ 
Sbjct  6    AMAMFFGASSVLAAPAAVVEDRALLGGLLGGSSTSTSSATSGLQSLTSNLLSTVTGLVST  65

Query  73   IQTSTSGSGLTNILSNAGLSSSASSTSTASVSTAGLITLLNDLSTLIPADQFNAIKTAIQ  132
            I+++ SG+   ++L+  G            VST GL + +  L++ +  DQ + I +A+ 
Sbjct  66   IESTVSGTDAASLLNVNG-----------DVSTDGLTSTVTGLTSALTGDQISTITSALD  114

Query  133  NLPANISNDLYTLIAKVISVVKQIAAAQ-------KSSASLVSSGSNGLLASLLGGTSVL  185
            ++P  +   L+ L+  V+ +V Q+  A          ++   S     L   L GG S  
Sbjct  115  DIPL-VGQPLHDLLETVLELVNQLGLANVASGALASGASGSSSGLGGLLGGLLGGGASSA  173

Query  186  TNLQSVITQVVNSLDTINSGVTASTSVAGVDAVAQVFASLKTPTDASLAAIVANSAKIIS  245
            TNLQ++ITQVV+ L +++SGV+ ++   G++AV  +  +LK  TD +L+ ++ NS KI+S
Sbjct  174  TNLQNIITQVVSGLSSVSSGVSGASGSTGLNAVTTILDTLKDSTDTNLSGLLGNSNKIVS  233

Query  246  GFMA  249
            G ++
Sbjct  234  GLLS  237


>gb|KIJ55993.1| hypothetical protein M422DRAFT_239170 [Sphaerobolus stellatus 
SS14]
Length=192

 Score = 37.7 bits (86),  Expect = 6.2, Method: Compositional matrix adjust.
 Identities = 29/91 (32%), Positives = 48/91 (53%), Gaps = 17/91 (19%)

Query  5   ILFFSAFTAFTALIGASTALAVPTPAVVERAAIEDRGLFDQLLGLGTVSQLQAITSTL--  62
           ++F     +F +L G  +A+A P P V E+ AIE R     +LG+  VS LQ+ TS++  
Sbjct  1   MIFSRVLLSFLSL-GVISAVASPAPVVEEKRAIEKRADVSDVLGV--VSTLQSSTSSILP  57

Query  63  ------------KSSVAPTLTQIQTSTSGSG  81
                         +++P LTQ+QT+ + +G
Sbjct  58  QMDNLVSTNQATTDNISPLLTQLQTALTTAG  88


Lambda      K        H        a         alpha
   0.315    0.127    0.326    0.792     4.96 

Gapped
Lambda      K        H        a         alpha    sigma
   0.267   0.0410    0.140     1.90     42.6     43.6 

Effective search space used: 1994867848521


  Database: nr
    Posted date:  Sep 23, 2015 12:05 AM
  Number of letters in database: 26,053,659,533
  Number of sequences in database:  71,551,133


Matrix: BLOSUM62
Gap Penalties: Existence: 11, Extension: 1
Neighboring words threshold: 11
Window for multiple hits: 40
```
