# Supplementary material for: Comparative genomics provides new insights into the diversity, physiology, and sexuality of the only industrially exploited tremellomycete: Phaffia rhodozyma
Source: BMC Genomics. 2016 Nov 9;17:901. doi: 10.1186/s12864-016-3244-7 (PMC5103461; doi:10.1186/s12864-016-3244-7)
Supplement: Additional file 6: — List of orphan genes with links to PFAM (related to Additional file 1: Table S1). (ZIP 1428 kb) [file 12864_2016_3244_MOESM6_ESM.zip › BLAST_HTML_FTR/G02822_P.html]

BLAST Search Results


```
BLASTP 2.2.27+


Reference:
Stephen F. Altschul, Thomas L. Madden, Alejandro A. Schäffer,
Jinghui Zhang, Zheng Zhang, Webb Miller, and David J. Lipman (1997),
"Gapped BLAST and PSI-BLAST: a new generation of protein database
search programs", Nucleic Acids Res. 25:3389-3402.


Reference for
composition-based statistics:
Alejandro A. Schäffer, L. Aravind, Thomas L. Madden, Sergei
Shavirin, John L. Spouge, Yuri I. Wolf, Eugene V. Koonin, and
Stephen F. Altschul (2001), "Improving the accuracy of PSI-BLAST
protein database searches with composition-based statistics and
other refinements", Nucleic Acids Res. 29:2994-3005.


Database: nr
           71,551,133 sequences; 26,053,659,533 total letters


Query= G02822_P

Length=1056
                                                                      Score     E
Sequences producing significant alignments:                          (Bits)  Value

emb|CED84852.1|  hypothetical protein [Xanthophyllomyces dendrorh...  2092    0.0  


 >emb|CED84852.1| hypothetical protein [Xanthophyllomyces dendrorhous]
Length=1119

 Score = 2092 bits (5419),  Expect = 0.0, Method: Compositional matrix adjust.
 Identities = 1054/1055 (99%), Positives = 1055/1055 (100%), Gaps = 0/1055 (0%)

Query  1     MGFFHRTSDSGSSSVALSRSSTDSYASTADTSDTSYQGSASVSPNTPLPKSSSSSSLRAF  60
             MGFFHRTSDSGSSSVALSRSSTDSYASTADTSDTSYQGSASVSPNTPLPKSSSSSSLRAF
Sbjct  65    MGFFHRTSDSGSSSVALSRSSTDSYASTADTSDTSYQGSASVSPNTPLPKSSSSSSLRAF  124

Query  61    FASPKQFIFPPPAVFYPASTTTTDHMSYRSRGTSFDRQHSSGQVTPNGSAGLERKTSRRK  120
             FASPKQFIFPPPAVFYPASTTTTDHMSYRSRGTSFDRQHSSGQVTPNGSAGLERKTSRRK
Sbjct  125   FASPKQFIFPPPAVFYPASTTTTDHMSYRSRGTSFDRQHSSGQVTPNGSAGLERKTSRRK  184

Query  121   QRSQSFHVLTPSSSQTWLSPDGSTRSRSDFLLEIACNTEKVPLVSSTSSRRNSFVLMSAC  180
             QRSQSFHVLTPSSSQTWLSPDGSTRSRSDFLLEIACNTEKVPLVSSTSSRRNSFVLMSAC
Sbjct  185   QRSQSFHVLTPSSSQTWLSPDGSTRSRSDFLLEIACNTEKVPLVSSTSSRRNSFVLMSAC  244

Query  181   HSTNSQRDSSVDSLGSSVSRRRTRSIEYAAAAGTPTASTSAAGTPIGTPQSINMKQLSPL  240
             HSTNSQRDSSVDSLGSSVSRRRTRSIEYAAAAGTPTASTSAAGTPIGTPQSINMKQLSPL
Sbjct  245   HSTNSQRDSSVDSLGSSVSRRRTRSIEYAAAAGTPTASTSAAGTPIGTPQSINMKQLSPL  304

Query  241   QEFRRSNSATRPFPLRSNTAPPVSPTQLSKIPTSNQPVSFIDALAESARERPDRADGPND  300
             QEFRRSNSATRPFPLRSNTAPPVSPTQLSKIPTSNQPVSFIDALAESARERPDRADGPND
Sbjct  305   QEFRRSNSATRPFPLRSNTAPPVSPTQLSKIPTSNQPVSFIDALAESARERPDRADGPND  364

Query  301   ENERDEEDDEDDDDRSTMKPNQELQPRLKIGSSTSSSSAGFRADSPSGRSLQGRRPIGNR  360
             ENERDEEDDEDDDDRSTMKPNQELQPRLKIGSSTSSSSAGFRADSPSGRSLQGRRPIGNR
Sbjct  365   ENERDEEDDEDDDDRSTMKPNQELQPRLKIGSSTSSSSAGFRADSPSGRSLQGRRPIGNR  424

Query  361   GYTTTSVSTSTSSPNATTSMIYIDRVIRTTRHPIPSCQKSTRTSTVHSVPFPSTASSKNL  420
             GYTTTSVSTSTSSPNATTSMIYIDRVIRTTRHPIPSCQKSTRTSTVHSVPFPSTASSKNL
Sbjct  425   GYTTTSVSTSTSSPNATTSMIYIDRVIRTTRHPIPSCQKSTRTSTVHSVPFPSTASSKNL  484

Query  421   ALSSSREQFNQLEIRNIPLITTSPPSVLTGSTVTFKPKSRRTLSLPFQPHLALSIIEPAE  480
             ALSSSREQFNQLEIRNIPLITTSPPSVLTGSTVTFKPKSRRTLSLPFQPHLALSIIEPAE
Sbjct  485   ALSSSREQFNQLEIRNIPLITTSPPSVLTGSTVTFKPKSRRTLSLPFQPHLALSIIEPAE  544

Query  481   PNEADVVTPTATTFPPTSTSGAAAAAAAASAALSPSSSTSTITPRPRTHSTFSRPLTTSL  540
             PNEADVVTPTATTFPPTSTSGAAAAAAAASAALSPSSSTSTITPRPRTHSTFSRPLTTSL
Sbjct  545   PNEADVVTPTATTFPPTSTSGAAAAAAAASAALSPSSSTSTITPRPRTHSTFSRPLTTSL  604

Query  541   KGVLPEVAPSQIPSSASMACLSTFSSWDFPPARAESLAAKHKVLSPLESYVFPTTSRSST  600
             KGVLPEVAPSQIPSSASMACLSTFSSWDFPPARAESLAAKHKVLSPLESYVFPTTSRSST
Sbjct  605   KGVLPEVAPSQIPSSASMACLSTFSSWDFPPARAESLAAKHKVLSPLESYVFPTTSRSST  664

Query  601   MSSSNSLMLPPPVPPPRSIRRSIVGGQPTALVERALNSKATTARPNVKSYSAVPTIPLAS  660
             MSSSNSLMLPPPVPPPRSIRRSIVGGQPTALVERALNSKATTARPNVKSYSAVPTIPLAS
Sbjct  665   MSSSNSLMLPPPVPPPRSIRRSIVGGQPTALVERALNSKATTARPNVKSYSAVPTIPLAS  724

Query  661   SSTAPTLTSTTIAQTSSSSSSTTSLSSTALSLAALTGPSRTTLKRSMTVNSIVHIQGPSQ  720
             SSTAPTLTSTTIAQTSSSSSSTTSLSSTALSLAALTGPSRTTLKRSMTVNSIVHIQGPSQ
Sbjct  725   SSTAPTLTSTTIAQTSSSSSSTTSLSSTALSLAALTGPSRTTLKRSMTVNSIVHIQGPSQ  784

Query  721   LSPPTVPSRHPNRAKLRNPQSYSLQNCKTISVQTNTGSSRSHSSPNSASSSTALSSSPVY  780
             LSPPTVPSRHPNRAKLRNPQSYSLQNCKTISVQTNTGSSRSHSSPNSASSSTALSSSPVY
Sbjct  785   LSPPTVPSRHPNRAKLRNPQSYSLQNCKTISVQTNTGSSRSHSSPNSASSSTALSSSPVY  844

Query  781   MFPLESSNGSDHSTKRSLSPSLNGYVSDSGHNHHRHLHEMGFGSISTPAGWADRRGSHSS  840
             MFPLESSNGSDHS+KRSLSPSLNGYVSDSGHNHHRHLHEMGFGSISTPAGWADRRGSHSS
Sbjct  845   MFPLESSNGSDHSSKRSLSPSLNGYVSDSGHNHHRHLHEMGFGSISTPAGWADRRGSHSS  904

Query  841   SSSLRQEVFPFEKSRPLDSSHTAFSPISSERSSAGQSAANYNFNVHRPGIGDRRQTTGGP  900
             SSSLRQEVFPFEKSRPLDSSHTAFSPISSERSSAGQSAANYNFNVHRPGIGDRRQTTGGP
Sbjct  905   SSSLRQEVFPFEKSRPLDSSHTAFSPISSERSSAGQSAANYNFNVHRPGIGDRRQTTGGP  964

Query  901   GPSVRQMERTLSIDSESSSSGFSSFAIGNYDPSVNMTAIDPGMETGLSTGGTSGATGKTR  960
             GPSVRQMERTLSIDSESSSSGFSSFAIGNYDPSVNMTAIDPGMETGLSTGGTSGATGKTR
Sbjct  965   GPSVRQMERTLSIDSESSSSGFSSFAIGNYDPSVNMTAIDPGMETGLSTGGTSGATGKTR  1024

Query  961   SRSSLSLAGLLGGFTKRGSSFWDGASKKRDDDGDQIDEFGRQKVVHSFSDGGERNDGSSR  1020
             SRSSLSLAGLLGGFTKRGSSFWDGASKKRDDDGDQIDEFGRQKVVHSFSDGGERNDGSSR
Sbjct  1025  SRSSLSLAGLLGGFTKRGSSFWDGASKKRDDDGDQIDEFGRQKVVHSFSDGGERNDGSSR  1084

Query  1021  RGRGRGRDEKREQERPGQEEDEDEDEEEFCSFGNM  1055
             RGRGRGRDEKREQERPGQEEDEDEDEEEFCSFGNM
Sbjct  1085  RGRGRGRDEKREQERPGQEEDEDEDEEEFCSFGNM  1119


Lambda      K        H        a         alpha
   0.308    0.121    0.338    0.792     4.96 

Gapped
Lambda      K        H        a         alpha    sigma
   0.267   0.0410    0.140     1.90     42.6     43.6 

Effective search space used: 12929364132378


  Database: nr
    Posted date:  Sep 23, 2015 12:05 AM
  Number of letters in database: 26,053,659,533
  Number of sequences in database:  71,551,133


Matrix: BLOSUM62
Gap Penalties: Existence: 11, Extension: 1
Neighboring words threshold: 11
Window for multiple hits: 40
```
