# Supplementary material for: Comparative genomics provides new insights into the diversity, physiology, and sexuality of the only industrially exploited tremellomycete: Phaffia rhodozyma
Source: BMC Genomics. 2016 Nov 9;17:901. doi: 10.1186/s12864-016-3244-7 (PMC5103461; doi:10.1186/s12864-016-3244-7)
Supplement: Additional file 6: — List of orphan genes with links to PFAM (related to Additional file 1: Table S1). (ZIP 1428 kb) [file 12864_2016_3244_MOESM6_ESM.zip › BLAST_HTML_FTR/G04122_P.html]

BLAST Search Results


```
BLASTP 2.2.27+


Reference:
Stephen F. Altschul, Thomas L. Madden, Alejandro A. Schäffer,
Jinghui Zhang, Zheng Zhang, Webb Miller, and David J. Lipman (1997),
"Gapped BLAST and PSI-BLAST: a new generation of protein database
search programs", Nucleic Acids Res. 25:3389-3402.


Reference for
composition-based statistics:
Alejandro A. Schäffer, L. Aravind, Thomas L. Madden, Sergei
Shavirin, John L. Spouge, Yuri I. Wolf, Eugene V. Koonin, and
Stephen F. Altschul (2001), "Improving the accuracy of PSI-BLAST
protein database searches with composition-based statistics and
other refinements", Nucleic Acids Res. 29:2994-3005.


Database: nr
           71,551,133 sequences; 26,053,659,533 total letters


Query= G04122_P

Length=626
                                                                      Score     E
Sequences producing significant alignments:                          (Bits)  Value

emb|CDZ97013.1|  hypothetical protein [Xanthophyllomyces dendrorh...  1243    0.0  


 >emb|CDZ97013.1| hypothetical protein [Xanthophyllomyces dendrorhous]
Length=625

 Score = 1243 bits (3216),  Expect = 0.0, Method: Compositional matrix adjust.
 Identities = 625/625 (100%), Positives = 625/625 (100%), Gaps = 0/625 (0%)

Query  1    MNSSIFSPIDSPCSSFSIESSLGYEEYPLSPLSYYTEPSSPFSFASLGGLAQQLCSSSIG  60
            MNSSIFSPIDSPCSSFSIESSLGYEEYPLSPLSYYTEPSSPFSFASLGGLAQQLCSSSIG
Sbjct  1    MNSSIFSPIDSPCSSFSIESSLGYEEYPLSPLSYYTEPSSPFSFASLGGLAQQLCSSSIG  60

Query  61   SVSSTSASSSNSSPFALESPSSPPFEDLMNDLYPSSDDERLVTPTLSSRQPLSAENVPFL  120
            SVSSTSASSSNSSPFALESPSSPPFEDLMNDLYPSSDDERLVTPTLSSRQPLSAENVPFL
Sbjct  61   SVSSTSASSSNSSPFALESPSSPPFEDLMNDLYPSSDDERLVTPTLSSRQPLSAENVPFL  120

Query  121  QQREQTTPAFVLNKMNRVKPSRPMSSSAKSNGTISLAALQPAPLSLGLESHSFSQVVDLG  180
            QQREQTTPAFVLNKMNRVKPSRPMSSSAKSNGTISLAALQPAPLSLGLESHSFSQVVDLG
Sbjct  121  QQREQTTPAFVLNKMNRVKPSRPMSSSAKSNGTISLAALQPAPLSLGLESHSFSQVVDLG  180

Query  181  TSSWEDKQSESMLDVYTSFECLESSLLSSTSETTPTVQYATPVRAAHRTRSNPALQSTNF  240
            TSSWEDKQSESMLDVYTSFECLESSLLSSTSETTPTVQYATPVRAAHRTRSNPALQSTNF
Sbjct  181  TSSWEDKQSESMLDVYTSFECLESSLLSSTSETTPTVQYATPVRAAHRTRSNPALQSTNF  240

Query  241  AGRQRLPAKASLSTLREDSNFDFSSVFDGTSGASVAGSSSTAAQPSMFAVLGGYGGENQS  300
            AGRQRLPAKASLSTLREDSNFDFSSVFDGTSGASVAGSSSTAAQPSMFAVLGGYGGENQS
Sbjct  241  AGRQRLPAKASLSTLREDSNFDFSSVFDGTSGASVAGSSSTAAQPSMFAVLGGYGGENQS  300

Query  301  LGHSWESRTVGMDAYRSDYTLRCLDLENLDISMLASSDGEDIKETTRPSSATLYSGPVDF  360
            LGHSWESRTVGMDAYRSDYTLRCLDLENLDISMLASSDGEDIKETTRPSSATLYSGPVDF
Sbjct  301  LGHSWESRTVGMDAYRSDYTLRCLDLENLDISMLASSDGEDIKETTRPSSATLYSGPVDF  360

Query  361  SPLSPPHISDLPALLPPPIPKKSKRRPKKAVVVSGADATPIATASNSASTSSTEPEDVDV  420
            SPLSPPHISDLPALLPPPIPKKSKRRPKKAVVVSGADATPIATASNSASTSSTEPEDVDV
Sbjct  361  SPLSPPHISDLPALLPPPIPKKSKRRPKKAVVVSGADATPIATASNSASTSSTEPEDVDV  420

Query  421  ARRRIEASRPIMICGWHDPGSLISESVFETSLSSPVPPAPTTIKAPMIPARNAARKLKAV  480
            ARRRIEASRPIMICGWHDPGSLISESVFETSLSSPVPPAPTTIKAPMIPARNAARKLKAV
Sbjct  421  ARRRIEASRPIMICGWHDPGSLISESVFETSLSSPVPPAPTTIKAPMIPARNAARKLKAV  480

Query  481  AMAAVTPVSSSPFSTSSPESFGSDSMSCSSLPSSPITTLQKPIKLRPALSGRAGSSGSSP  540
            AMAAVTPVSSSPFSTSSPESFGSDSMSCSSLPSSPITTLQKPIKLRPALSGRAGSSGSSP
Sbjct  481  AMAAVTPVSSSPFSTSSPESFGSDSMSCSSLPSSPITTLQKPIKLRPALSGRAGSSGSSP  540

Query  541  SRPKTSAITSIGKREQTGPVAYSFINYTVNDATKLMTGVAPSGNARKACGSVSSSTTTVA  600
            SRPKTSAITSIGKREQTGPVAYSFINYTVNDATKLMTGVAPSGNARKACGSVSSSTTTVA
Sbjct  541  SRPKTSAITSIGKREQTGPVAYSFINYTVNDATKLMTGVAPSGNARKACGSVSSSTTTVA  600

Query  601  PGSAKRKRLETDEEDGPQRGGKRRA  625
            PGSAKRKRLETDEEDGPQRGGKRRA
Sbjct  601  PGSAKRKRLETDEEDGPQRGGKRRA  625


Lambda      K        H        a         alpha
   0.309    0.123    0.344    0.792     4.96 

Gapped
Lambda      K        H        a         alpha    sigma
   0.267   0.0410    0.140     1.90     42.6     43.6 

Effective search space used: 6950641744788


  Database: nr
    Posted date:  Sep 23, 2015 12:05 AM
  Number of letters in database: 26,053,659,533
  Number of sequences in database:  71,551,133


Matrix: BLOSUM62
Gap Penalties: Existence: 11, Extension: 1
Neighboring words threshold: 11
Window for multiple hits: 40
```
