# Supplementary material for: Comparative genomics provides new insights into the diversity, physiology, and sexuality of the only industrially exploited tremellomycete: Phaffia rhodozyma
Source: BMC Genomics. 2016 Nov 9;17:901. doi: 10.1186/s12864-016-3244-7 (PMC5103461; doi:10.1186/s12864-016-3244-7)
Supplement: Additional file 6: — List of orphan genes with links to PFAM (related to Additional file 1: Table S1). (ZIP 1428 kb) [file 12864_2016_3244_MOESM6_ESM.zip › BLAST_HTML_FTR/G03726_P.html]

BLAST Search Results


```
BLASTP 2.2.27+


Reference:
Stephen F. Altschul, Thomas L. Madden, Alejandro A. Schäffer,
Jinghui Zhang, Zheng Zhang, Webb Miller, and David J. Lipman (1997),
"Gapped BLAST and PSI-BLAST: a new generation of protein database
search programs", Nucleic Acids Res. 25:3389-3402.


Reference for
composition-based statistics:
Alejandro A. Schäffer, L. Aravind, Thomas L. Madden, Sergei
Shavirin, John L. Spouge, Yuri I. Wolf, Eugene V. Koonin, and
Stephen F. Altschul (2001), "Improving the accuracy of PSI-BLAST
protein database searches with composition-based statistics and
other refinements", Nucleic Acids Res. 29:2994-3005.


Database: nr
           71,551,133 sequences; 26,053,659,533 total letters


Query= G03726_P

Length=196
                                                                      Score     E
Sequences producing significant alignments:                          (Bits)  Value

emb|CDZ96253.1|  BRCT domain [Xanthophyllomyces dendrorhous]           163    1e-45
gb|KKR22387.1|  Chromosome segregation ATPase-like protein [Parcu...  36.2    8.5  


 >emb|CDZ96253.1| BRCT domain [Xanthophyllomyces dendrorhous]
Length=268

 Score =  163 bits (412),  Expect = 1e-45, Method: Compositional matrix adjust.
 Identities = 82/84 (98%), Positives = 83/84 (99%), Gaps = 0/84 (0%)

Query  112  QRFGKIKVETSPDPSTFLAKTFMSSTIQAVKVEEGFDKDDFIDPRLTKKKRVHAKLRSGL  171
            + FGKIKVETSPDPSTFLAKTFMSSTIQAVKVEEGFDKDDFIDPRLTKKKRVHAKLRSGL
Sbjct  185  EPFGKIKVETSPDPSTFLAKTFMSSTIQAVKVEEGFDKDDFIDPRLTKKKRVHAKLRSGL  244

Query  172  TLSSTVKEKTRAKRHLKNLITRSI  195
            TLSSTVKEKTRAKRHLKNLITRSI
Sbjct  245  TLSSTVKEKTRAKRHLKNLITRSI  268


 Score =  139 bits (349),  Expect = 3e-36, Method: Compositional matrix adjust.
 Identities = 62/62 (100%), Positives = 62/62 (100%), Gaps = 0/62 (0%)

Query  1   MQQQSNFFSPFGSAWFSASSDRLHVQRWRENGGTVVENSSSYVVSDVKIVFCDGRCDPLY  60
           MQQQSNFFSPFGSAWFSASSDRLHVQRWRENGGTVVENSSSYVVSDVKIVFCDGRCDPLY
Sbjct  1   MQQQSNFFSPFGSAWFSASSDRLHVQRWRENGGTVVENSSSYVVSDVKIVFCDGRCDPLY  60

Query  61  AK  62
           AK
Sbjct  61  AK  62


>gb|KKR22387.1| Chromosome segregation ATPase-like protein [Parcubacteria (Uhrbacteria) 
bacterium GW2011_GWE1_39_46]
 gb|KKR63705.1| Chromosome segregation ATPase-like protein [Parcubacteria (Uhrbacteria) 
bacterium GW2011_GWC2_40_450]
 gb|KKR89351.1| Chromosome segregation ATPase-like protein [Parcubacteria (Uhrbacteria) 
bacterium GW2011_GWE2_41_1153]
 8 more sequence titles

gb|KKR89793.1| Chromosome segregation ATPase-like protein [Parcubacteria (Uhrbacteria) 
bacterium GW2011_GWD2_41_121]
 gb|KKR95663.1| Chromosome segregation ATPase-like protein [Parcubacteria (Uhrbacteria) 
bacterium GW2011_GWD1_41_16]
 gb|KKR98450.1| Chromosome segregation ATPase-like protein [Parcubacteria (Uhrbacteria) 
bacterium GW2011_GWC1_41_20]
 gb|KKS05660.1| Chromosome segregation ATPase-like protein [Parcubacteria (Uhrbacteria) 
bacterium GW2011_GWB2_41_36]
 gb|KKS07404.1| Chromosome segregation ATPase-like protein [Parcubacteria (Uhrbacteria) 
bacterium GW2011_GWF2_41_40]
 gb|KKS10051.1| Chromosome segregation ATPase-like protein [Parcubacteria (Uhrbacteria) 
bacterium GW2011_GWF2_41_430]
 gb|KKS17785.1| Chromosome segregation ATPase-like protein [Parcubacteria (Uhrbacteria) 
bacterium GW2011_GWB1_41_7]
 gb|KKS50798.1| Chromosome segregation ATPase-like protein [Parcubacteria (Uhrbacteria) 
bacterium GW2011_GWA2_42_220]

Length=152

 Score = 36.2 bits (82),  Expect = 8.5, Method: Compositional matrix adjust.
 Identities = 24/75 (32%), Positives = 38/75 (51%), Gaps = 3/75 (4%)

Query  87   VDLMLFKFGTHDTDIEKFYASFALFQRFGKIK---VETSPDPSTFLAKTFMSSTIQAVKV  143
            +D +  K   HD DI++  A+ A  Q   K+K   +E   +  T + K     T+QA  +
Sbjct  71   IDFLAQKMIEHDEDIKEIKATMATKQDLAKLKSEILEPLDEMITLMKKRDEEMTMQAHGI  130

Query  144  EEGFDKDDFIDPRLT  158
            +   D+DD ID R+T
Sbjct  131  QRLNDRDDKIDARIT  145


Lambda      K        H        a         alpha
   0.325    0.137    0.407    0.792     4.96 

Gapped
Lambda      K        H        a         alpha    sigma
   0.267   0.0410    0.140     1.90     42.6     43.6 

Effective search space used: 858243526938


  Database: nr
    Posted date:  Sep 23, 2015 12:05 AM
  Number of letters in database: 26,053,659,533
  Number of sequences in database:  71,551,133


Matrix: BLOSUM62
Gap Penalties: Existence: 11, Extension: 1
Neighboring words threshold: 11
Window for multiple hits: 40
```
