# Supplementary material for: Comparative genomics provides new insights into the diversity, physiology, and sexuality of the only industrially exploited tremellomycete: Phaffia rhodozyma
Source: BMC Genomics. 2016 Nov 9;17:901. doi: 10.1186/s12864-016-3244-7 (PMC5103461; doi:10.1186/s12864-016-3244-7)
Supplement: Additional file 6: — List of orphan genes with links to PFAM (related to Additional file 1: Table S1). (ZIP 1428 kb) [file 12864_2016_3244_MOESM6_ESM.zip › BLAST_HTML_FTR/G05771_P.html]

BLAST Search Results


```
BLASTP 2.2.27+


Reference:
Stephen F. Altschul, Thomas L. Madden, Alejandro A. Schäffer,
Jinghui Zhang, Zheng Zhang, Webb Miller, and David J. Lipman (1997),
"Gapped BLAST and PSI-BLAST: a new generation of protein database
search programs", Nucleic Acids Res. 25:3389-3402.


Reference for
composition-based statistics:
Alejandro A. Schäffer, L. Aravind, Thomas L. Madden, Sergei
Shavirin, John L. Spouge, Yuri I. Wolf, Eugene V. Koonin, and
Stephen F. Altschul (2001), "Improving the accuracy of PSI-BLAST
protein database searches with composition-based statistics and
other refinements", Nucleic Acids Res. 29:2994-3005.


Database: nr
           71,551,133 sequences; 26,053,659,533 total letters


Query= G05771_P

Length=1391
                                                                      Score     E
Sequences producing significant alignments:                          (Bits)  Value

emb|CED84635.1|  hypothetical protein [Xanthophyllomyces dendrorh...  2771    0.0  
ref|XP_007843784.1|  hypothetical protein Moror_6530 [Moniliophth...  72.0    2e-09
gb|KIR56700.1|  hypothetical protein I315_00879 [Cryptococcus gat...  65.9    4e-07
gb|KIY31725.1|  hypothetical protein I305_05680 [Cryptococcus gat...  65.1    6e-07
gb|KIR81577.1|  hypothetical protein I306_01418 [Cryptococcus gat...  63.9    1e-06
ref|XP_003195625.1|  hypothetical protein CGB_H1760C [Cryptococcu...  63.9    1e-06
gb|KIR45285.1|  hypothetical protein I312_05324 [Cryptococcus gat...  57.4    1e-04
ref|XP_001833285.2|  hypothetical protein CC1G_04264 [Coprinopsis...  56.6    3e-04
ref|XP_012051532.1|  hypothetical protein CNAG_04434 [Cryptococcu...  56.2    3e-04
gb|KDE04562.1|  hypothetical protein, variant [Microbotryum viola...  56.2    3e-04
gb|KIR59288.1|  hypothetical protein I314_04803 [Cryptococcus gat...  53.9    0.001
gb|KIR88183.1|  hypothetical protein I308_01243 [Cryptococcus gat...  52.8    0.003
ref|XP_007882279.1|  hypothetical protein PFL1_06545 [Pseudozyma ...  48.9    0.059
emb|CUA69368.1|  Proteoglycan 4 [Rhizoctonia solani]                  47.8    0.11 
gb|EGN92458.1|  hypothetical protein SERLA73DRAFT_79593 [Serpula ...  45.8    0.39 
gb|EMS25332.1|  hypothetical protein RHTO_03060 [Rhodosporidium t...  42.7    3.7  


 >emb|CED84635.1| hypothetical protein [Xanthophyllomyces dendrorhous]
Length=1390

 Score = 2771 bits (7182),  Expect = 0.0, Method: Compositional matrix adjust.
 Identities = 1390/1390 (100%), Positives = 1390/1390 (100%), Gaps = 0/1390 (0%)

Query  1     MDHPPLTPTRITDNLPSTTSTSVCSPTQLINHDHGHDASDTESTTAETASTCSSFAEWSS  60
             MDHPPLTPTRITDNLPSTTSTSVCSPTQLINHDHGHDASDTESTTAETASTCSSFAEWSS
Sbjct  1     MDHPPLTPTRITDNLPSTTSTSVCSPTQLINHDHGHDASDTESTTAETASTCSSFAEWSS  60

Query  61    GSDSDSNPNRIFYGPTRTPERKLLQKVRHKDSREFHRRKTLWEDPSVRAADRSILSPTKS  120
             GSDSDSNPNRIFYGPTRTPERKLLQKVRHKDSREFHRRKTLWEDPSVRAADRSILSPTKS
Sbjct  61    GSDSDSNPNRIFYGPTRTPERKLLQKVRHKDSREFHRRKTLWEDPSVRAADRSILSPTKS  120

Query  121   SQCELSVVTSVRSPCSTPPPRNVRRPSPQTRLESPNKYIDNPASSLSTPPASPPHSTTTP  180
             SQCELSVVTSVRSPCSTPPPRNVRRPSPQTRLESPNKYIDNPASSLSTPPASPPHSTTTP
Sbjct  121   SQCELSVVTSVRSPCSTPPPRNVRRPSPQTRLESPNKYIDNPASSLSTPPASPPHSTTTP  180

Query  181   SPPTRSPKFPPSPSAVPVCGSPFPTYITSRARTFHHNRRKPSDLAKADESFSSADGSMSA  240
             SPPTRSPKFPPSPSAVPVCGSPFPTYITSRARTFHHNRRKPSDLAKADESFSSADGSMSA
Sbjct  181   SPPTRSPKFPPSPSAVPVCGSPFPTYITSRARTFHHNRRKPSDLAKADESFSSADGSMSA  240

Query  241   DSEDEFEYQPEPESVHQPEAAPHAELDANEEDEGEWETEGEHEEEEEEEEEEEADVPKEE  300
             DSEDEFEYQPEPESVHQPEAAPHAELDANEEDEGEWETEGEHEEEEEEEEEEEADVPKEE
Sbjct  241   DSEDEFEYQPEPESVHQPEAAPHAELDANEEDEGEWETEGEHEEEEEEEEEEEADVPKEE  300

Query  301   DIDETGCALDQSVESAKQEELEGEREREPEEEFVVAQPTEDCISGDPVEAGTEPIAMIPF  360
             DIDETGCALDQSVESAKQEELEGEREREPEEEFVVAQPTEDCISGDPVEAGTEPIAMIPF
Sbjct  301   DIDETGCALDQSVESAKQEELEGEREREPEEEFVVAQPTEDCISGDPVEAGTEPIAMIPF  360

Query  361   DIVTEETAEEHAQDVTEITVEETIPIITPQVASVVTEETIEEIAEDSTMKILQEDFEDIS  420
             DIVTEETAEEHAQDVTEITVEETIPIITPQVASVVTEETIEEIAEDSTMKILQEDFEDIS
Sbjct  361   DIVTEETAEEHAQDVTEITVEETIPIITPQVASVVTEETIEEIAEDSTMKILQEDFEDIS  420

Query  421   KKTFNETPANLVEESMEQSTELPVEVYTKETITESTEERAEVATSDAQYAMDLESADGSG  480
             KKTFNETPANLVEESMEQSTELPVEVYTKETITESTEERAEVATSDAQYAMDLESADGSG
Sbjct  421   KKTFNETPANLVEESMEQSTELPVEVYTKETITESTEERAEVATSDAQYAMDLESADGSG  480

Query  481   DKSETDVQKDVRGEESGKVVVNGDSSEFTNRTEVEEVEEDVGNAGGVADEESVVSADHIE  540
             DKSETDVQKDVRGEESGKVVVNGDSSEFTNRTEVEEVEEDVGNAGGVADEESVVSADHIE
Sbjct  481   DKSETDVQKDVRGEESGKVVVNGDSSEFTNRTEVEEVEEDVGNAGGVADEESVVSADHIE  540

Query  541   PCADENQEISGQGEDEVLRLPPDETRDDLGESNVEKFQPSDQPRLDVLTFSQSEEGSAAL  600
             PCADENQEISGQGEDEVLRLPPDETRDDLGESNVEKFQPSDQPRLDVLTFSQSEEGSAAL
Sbjct  541   PCADENQEISGQGEDEVLRLPPDETRDDLGESNVEKFQPSDQPRLDVLTFSQSEEGSAAL  600

Query  601   LDGVSPVSLCHQTPTSSPKLSPDQLAAHSITPNTSAGLDEPAICERIQPAYTNAQSNVLV  660
             LDGVSPVSLCHQTPTSSPKLSPDQLAAHSITPNTSAGLDEPAICERIQPAYTNAQSNVLV
Sbjct  601   LDGVSPVSLCHQTPTSSPKLSPDQLAAHSITPNTSAGLDEPAICERIQPAYTNAQSNVLV  660

Query  661   VSSLNPKAVLPEPFSEPVRSTPKRVQKSPVKSSHSKRKSSRSQPSSSSTTAAAASQRILS  720
             VSSLNPKAVLPEPFSEPVRSTPKRVQKSPVKSSHSKRKSSRSQPSSSSTTAAAASQRILS
Sbjct  661   VSSLNPKAVLPEPFSEPVRSTPKRVQKSPVKSSHSKRKSSRSQPSSSSTTAAAASQRILS  720

Query  721   KPSEFALNEAESTRPQEPITSPGPLRQTFPNSVASTFSSGSGSDSTSASTFISIPPVSVL  780
             KPSEFALNEAESTRPQEPITSPGPLRQTFPNSVASTFSSGSGSDSTSASTFISIPPVSVL
Sbjct  721   KPSEFALNEAESTRPQEPITSPGPLRQTFPNSVASTFSSGSGSDSTSASTFISIPPVSVL  780

Query  781   KPLSVSLSLPSATGTASSSPAFSSSRLQGPSRSFGSNAESKVPKRDINVPERSALSKSAF  840
             KPLSVSLSLPSATGTASSSPAFSSSRLQGPSRSFGSNAESKVPKRDINVPERSALSKSAF
Sbjct  781   KPLSVSLSLPSATGTASSSPAFSSSRLQGPSRSFGSNAESKVPKRDINVPERSALSKSAF  840

Query  841   RPTLSNKARGYIPGVSVKSRGGYSSPSKSTILGPPVRVLHSPTTRSSSSTTATFLGSHSI  900
             RPTLSNKARGYIPGVSVKSRGGYSSPSKSTILGPPVRVLHSPTTRSSSSTTATFLGSHSI
Sbjct  841   RPTLSNKARGYIPGVSVKSRGGYSSPSKSTILGPPVRVLHSPTTRSSSSTTATFLGSHSI  900

Query  901   GPLSRSTGSVSPAPGIAPTVLPRVRPLGGTSSGSLGHGLQKDVPSEALTVGLTRKTFDQH  960
             GPLSRSTGSVSPAPGIAPTVLPRVRPLGGTSSGSLGHGLQKDVPSEALTVGLTRKTFDQH
Sbjct  901   GPLSRSTGSVSPAPGIAPTVLPRVRPLGGTSSGSLGHGLQKDVPSEALTVGLTRKTFDQH  960

Query  961   RTKDHSAKVEGGAGPGLGNSVRSVSSSLIPTSASASAFTSVPLSSAPSLVSPSTKTTGQD  1020
             RTKDHSAKVEGGAGPGLGNSVRSVSSSLIPTSASASAFTSVPLSSAPSLVSPSTKTTGQD
Sbjct  961   RTKDHSAKVEGGAGPGLGNSVRSVSSSLIPTSASASAFTSVPLSSAPSLVSPSTKTTGQD  1020

Query  1021  RPSGPASSLRSIPTRRSARAAGEPVGSAAPTIALPPVSHRSRTGFGGGSGGGVLTTSSAG  1080
             RPSGPASSLRSIPTRRSARAAGEPVGSAAPTIALPPVSHRSRTGFGGGSGGGVLTTSSAG
Sbjct  1021  RPSGPASSLRSIPTRRSARAAGEPVGSAAPTIALPPVSHRSRTGFGGGSGGGVLTTSSAG  1080

Query  1081  AVHSIASSTIPSLAMKPRRLPHSALNGSTASSSSASLPTVLTPPPPVPPLPSALAAPPAM  1140
             AVHSIASSTIPSLAMKPRRLPHSALNGSTASSSSASLPTVLTPPPPVPPLPSALAAPPAM
Sbjct  1081  AVHSIASSTIPSLAMKPRRLPHSALNGSTASSSSASLPTVLTPPPPVPPLPSALAAPPAM  1140

Query  1141  ANPVVPAPPPMLSKDLASLTDHNTIVNGVLFCALTRTVVRREGEHRPSSPGPKKKSPLAM  1200
             ANPVVPAPPPMLSKDLASLTDHNTIVNGVLFCALTRTVVRREGEHRPSSPGPKKKSPLAM
Sbjct  1141  ANPVVPAPPPMLSKDLASLTDHNTIVNGVLFCALTRTVVRREGEHRPSSPGPKKKSPLAM  1200

Query  1201  AAASSSSSRSTSGAGTGRGRKVSFGSESEEEEEEEDGAWPGLNGGGSVSDQEKKTHLLGP  1260
             AAASSSSSRSTSGAGTGRGRKVSFGSESEEEEEEEDGAWPGLNGGGSVSDQEKKTHLLGP
Sbjct  1201  AAASSSSSRSTSGAGTGRGRKVSFGSESEEEEEEEDGAWPGLNGGGSVSDQEKKTHLLGP  1260

Query  1261  GEDEVFVSPIRKNRSSATCGRKQLWSGSTSAVEARRSSDRMVKWKKSLVVSNSRSGADRT  1320
             GEDEVFVSPIRKNRSSATCGRKQLWSGSTSAVEARRSSDRMVKWKKSLVVSNSRSGADRT
Sbjct  1261  GEDEVFVSPIRKNRSSATCGRKQLWSGSTSAVEARRSSDRMVKWKKSLVVSNSRSGADRT  1320

Query  1321  YQSTEEISAELKKSCIKPTASLDVHGNLDLTKLPPLTSIIKRTKVTYVRFVYDDDLPKAP  1380
             YQSTEEISAELKKSCIKPTASLDVHGNLDLTKLPPLTSIIKRTKVTYVRFVYDDDLPKAP
Sbjct  1321  YQSTEEISAELKKSCIKPTASLDVHGNLDLTKLPPLTSIIKRTKVTYVRFVYDDDLPKAP  1380

Query  1381  AGEKKKKRGK  1390
             AGEKKKKRGK
Sbjct  1381  AGEKKKKRGK  1390


>ref|XP_007843784.1| hypothetical protein Moror_6530 [Moniliophthora roreri MCA 2997]
 gb|ESK96952.1| hypothetical protein Moror_6530 [Moniliophthora roreri MCA 2997]
Length=490

 Score = 72.0 bits (175),  Expect = 2e-09, Method: Compositional matrix adjust.
 Identities = 98/347 (28%), Positives = 139/347 (40%), Gaps = 56/347 (16%)

Query  1053  ALPPVSHRSRTGFG-GGSGGGVLTTSSAGAVHSIASSTIPSLAMKPRRLPHSALNGSTAS  1111
             A PPV +RSR G G  G+     + S AG  H        S   KP       +  S+ S
Sbjct  165   ASPPVDYRSRLGEGPEGASSEPASASEAGEEHETVCLRRTSRVRKP------VVGLSSGS  218

Query  1112  SSSASLPTVLTPPPPVPPLPSALAAPP-AMANPVVPAPPPMLSKDLASLTDHNTIVNGVL  1170
             +S ++ P    PP     +P  L   P A+ N V          DL  LT  NT  N   
Sbjct  219   TSRSTQPRRRKPP-----VPQYLGTGPFAIMNAV----------DLRQLTSTNTTRNQEY  263

Query  1171  FCALTRT-VVRREGEHRPSSPGPKKKS------------PLAMAAASSSSSRSTSGAGTG  1217
               A   T ++R+EG  RP SPG K K+              A A   +  S+   G    
Sbjct  264   LTATLETEIIRKEG-PRPESPGIKAKTVSQKEQEEKEKEQKARAERRARRSKKKLGEDMT  322

Query  1218  RGRKVSFGSESEEEEEEEDGAWPGLNGGGSVSDQEKKTHLLGPGEDEVFVSPIRKNRSSA  1277
              G     GS++  +E++ED AW  +    S      + H  G GEDE + +P R  R   
Sbjct  323   NGED-DAGSDANSDEDKEDDAWDPIESSPSA-----RRHTRGAGEDEDYETPRRIKRLRL  376

Query  1278  TCGRKQLWSGSTSAVEARRSSDRMVKWKKSLVVSNSRSGADRTYQSTEEISAELKKSCIK  1337
                  ++  G  S    RR     V+W + L    +    D       +      K C+ 
Sbjct  377   DDHENEVGGGRNSLNGKRR-----VQWDRGLF---TEVYLDEVQPRPRQTIVAAAKGCLA  428

Query  1338  PTA---SLDVHGNLDLTKLPPLTSIIKRTKVTYVRFVYDDDLPKAPA  1381
             PTA    LD  GNL  ++  PL  +++   +   RFVYD+D+ + PA
Sbjct  429   PTAKALKLDDLGNLPNSE-SPLKDLVQEN-IIVKRFVYDNDVTEEPA  473


>gb|KIR56700.1| hypothetical protein I315_00879 [Cryptococcus gattii Ru294]
Length=1011

 Score = 65.9 bits (159),  Expect = 4e-07, Method: Compositional matrix adjust.
 Identities = 69/247 (28%), Positives = 105/247 (43%), Gaps = 29/247 (12%)

Query  1147  APPPMLSKDLASLTDHNTIVNGVLFCALTRTVVRREGEHRPSSPGPKKKSPLAMAAASSS  1206
             A P +  K L + T  NT  N V  CA+ R ++R+ G  RP SP  K ++      A   
Sbjct  788   AAPALTEKQLKTTTARNTARNQVYHCAIDRKIIRQRGP-RPPSPTSKIRTTSEREEADKK  846

Query  1207  SSRSTSGAGTGRGRKVSFGSESEEEEEEEDGAWPGLNGGGSVSDQEKKTHLLGPGEDEVF  1266
              SR T         K   G  SEEE+E+          G  V ++ +     G  +D  +
Sbjct  847   KSRETRA-------KRRMGIHSEEEKEK----------GQQVIEKLEVAKAPGDEDDWTY  889

Query  1267  VSPIR-KNRSSATCGRKQLWSGSTSAVEARRSSDRMVKWKKSLVV---SNSRSGADRTYQ  1322
              +P R   R+  T    +       A E R+     ++W + + V     + SG++++  
Sbjct  890   KTPQRPLKRARRTSEADEHHDDGGDAEEERKK----LRWDRVVSVIRDDGNASGSNKSSD  945

Query  1323  STEEISAELKKSCIKPTASLDVHGNLDLTKLPPLTSIIKRTKVTYVRFVYDDDLP-KAPA  1381
               +E   E  KSCIK    LD HGN+   + P     +KRT+V      YD + P  APA
Sbjct  946   DGKEKDGEALKSCIKTKVPLDEHGNVLDAQRP--VDNLKRTRVVVTAVFYDGEEPVPAPA  1003

Query  1382  GEKKKKR  1388
                + K+
Sbjct  1004  TATRSKK  1010


>gb|KIY31725.1| hypothetical protein I305_05680 [Cryptococcus gattii E566]
Length=1009

 Score = 65.1 bits (157),  Expect = 6e-07, Method: Compositional matrix adjust.
 Identities = 69/247 (28%), Positives = 105/247 (43%), Gaps = 29/247 (12%)

Query  1147  APPPMLSKDLASLTDHNTIVNGVLFCALTRTVVRREGEHRPSSPGPKKKSPLAMAAASSS  1206
             A P +  K L + T  NT  N V  CA+ R ++R+ G  RP SP  K ++      A   
Sbjct  786   AAPALTEKQLKTTTARNTARNQVYHCAIDRKIIRQRGP-RPPSPTSKIRTTSEREEADKK  844

Query  1207  SSRSTSGAGTGRGRKVSFGSESEEEEEEEDGAWPGLNGGGSVSDQEKKTHLLGPGEDEVF  1266
              SR T         K   G  SEEE+E+          G  V ++ +     G  +D  +
Sbjct  845   KSRETRA-------KRRMGIHSEEEKEK----------GQQVIEKLEVAKAPGDEDDWTY  887

Query  1267  VSPIR-KNRSSATCGRKQLWSGSTSAVEARRSSDRMVKWKKSLVV---SNSRSGADRTYQ  1322
              +P R   R+  T    +       A E R+     ++W + + V     + SG++++  
Sbjct  888   KTPQRPLKRARRTSEADEHHDDDGDAEEERKK----LRWDRVVSVIRDDGNASGSNKSSD  943

Query  1323  STEEISAELKKSCIKPTASLDVHGNLDLTKLPPLTSIIKRTKVTYVRFVYDDDLPK-APA  1381
               +E   E  KSCIK    LD HGN+   + P     +KRT+V      YD + P  APA
Sbjct  944   DGKEKDGEELKSCIKTKVPLDEHGNVLDAQRP--VDNLKRTRVVVTAVFYDGEEPVPAPA  1001

Query  1382  GEKKKKR  1388
                + K+
Sbjct  1002  TATRSKK  1008


>gb|KIR81577.1| hypothetical protein I306_01418 [Cryptococcus gattii EJB2]
 gb|KJE00511.1| hypothetical protein I311_05919 [Cryptococcus gattii NT-10]
Length=1009

 Score = 63.9 bits (154),  Expect = 1e-06, Method: Compositional matrix adjust.
 Identities = 69/247 (28%), Positives = 105/247 (43%), Gaps = 29/247 (12%)

Query  1147  APPPMLSKDLASLTDHNTIVNGVLFCALTRTVVRREGEHRPSSPGPKKKSPLAMAAASSS  1206
             A P +  K L + T  NT  N V  CA+ R ++R+ G  RP SP  K ++      A   
Sbjct  786   AAPALTEKQLKTTTARNTARNQVYHCAIDRKIIRQLGP-RPPSPTSKIRTTSEREEADKK  844

Query  1207  SSRSTSGAGTGRGRKVSFGSESEEEEEEEDGAWPGLNGGGSVSDQEKKTHLLGPGEDEVF  1266
              SR T         K   G  SEEE+E+          G  V ++ +     G  +D  +
Sbjct  845   KSRETRA-------KRRMGIHSEEEKEK----------GQQVIEKLEVAKAPGDEDDWTY  887

Query  1267  VSPIR-KNRSSATCGRKQLWSGSTSAVEARRSSDRMVKWKKSLVV---SNSRSGADRTYQ  1322
              +P R   R+  T    +       A E R+     ++W + + V     + SG++++  
Sbjct  888   KTPQRPLKRARRTSEADEHHDDDGDAEEERKK----LRWDRVVSVIRDDGNASGSNKSSD  943

Query  1323  STEEISAELKKSCIKPTASLDVHGNLDLTKLPPLTSIIKRTKVTYVRFVYDDDLPK-APA  1381
               +E   E  KSCIK    LD HGN+   + P     +KRT+V      YD + P  APA
Sbjct  944   DGKEKDGEELKSCIKTKVPLDEHGNVLDAQRP--VDNLKRTRVVVTAVFYDGEEPVPAPA  1001

Query  1382  GEKKKKR  1388
                + K+
Sbjct  1002  TATRSKK  1008


>ref|XP_003195625.1| hypothetical protein CGB_H1760C [Cryptococcus gattii WM276]
 gb|ADV23838.1| hypothetical protein CNI01230 [Cryptococcus gattii WM276]
Length=1009

 Score = 63.9 bits (154),  Expect = 1e-06, Method: Compositional matrix adjust.
 Identities = 69/247 (28%), Positives = 105/247 (43%), Gaps = 29/247 (12%)

Query  1147  APPPMLSKDLASLTDHNTIVNGVLFCALTRTVVRREGEHRPSSPGPKKKSPLAMAAASSS  1206
             A P +  K L + T  NT  N V  CA+ R ++R+ G  RP SP  K ++      A   
Sbjct  786   AAPALTEKQLKTTTARNTARNQVYHCAIDRKIIRQLGP-RPPSPTSKIRTTSEREEADKK  844

Query  1207  SSRSTSGAGTGRGRKVSFGSESEEEEEEEDGAWPGLNGGGSVSDQEKKTHLLGPGEDEVF  1266
              SR T         K   G  SEEE+E+          G  V ++ +     G  +D  +
Sbjct  845   KSRETRA-------KRRMGIHSEEEKEK----------GQQVIEKLEVAKAPGDEDDWTY  887

Query  1267  VSPIR-KNRSSATCGRKQLWSGSTSAVEARRSSDRMVKWKKSLVV---SNSRSGADRTYQ  1322
              +P R   R+  T    +       A E R+     ++W + + V     + SG++++  
Sbjct  888   KTPQRPLKRARRTSEADEHHDDDGDAEEERKK----LRWDRVVSVIRDDGNASGSNKSSD  943

Query  1323  STEEISAELKKSCIKPTASLDVHGNLDLTKLPPLTSIIKRTKVTYVRFVYDDDLPK-APA  1381
               +E   E  KSCIK    LD HGN+   + P     +KRT+V      YD + P  APA
Sbjct  944   DGKEKDGEELKSCIKTKVPLDEHGNVLDAQRP--VDNLKRTRVVVTAVFYDGEEPVPAPA  1001

Query  1382  GEKKKKR  1388
                + K+
Sbjct  1002  TATRSKK  1008


>gb|KIR45285.1| hypothetical protein I312_05324 [Cryptococcus gattii CA1280]
Length=1046

 Score = 57.4 bits (137),  Expect = 1e-04, Method: Compositional matrix adjust.
 Identities = 62/243 (26%), Positives = 98/243 (40%), Gaps = 21/243 (9%)

Query  1147  APPPMLSKDLASLTDHNTIVNGVLFCALTRTVVRREGEHRPSSPGPKKKSPLAMAAASSS  1206
             A P +  K L + T  NT  N V  CA+ R V+R+ G  RP SP  K ++ L    A   
Sbjct  823   AAPALTEKQLKTTTARNTARNQVYHCAIDRKVIRQRGP-RPPSPTSKIRTTLEREEADKK  881

Query  1207  SSRSTSGAGTGRGRKVSFGSESEEEEEEEDGAWPGLNGGGSVSDQEKKTHLLGPGEDEVF  1266
              SR           K   G  S+E  + +      L    +  D++  T+       +  
Sbjct  882   MSREARA-------KRRMGIHSDEMMDGKQQVIEKLEVAKAPGDEDDWTY-------KTP  927

Query  1267  VSPIRKNRSSATCGRKQLWSGSTSAVEARRSSDRMVKWKKSLVVSNSRSGADRTYQSTEE  1326
               P+++ R ++               + +   DR+V   +      + SG+ ++    +E
Sbjct  928   QRPLKRARRTSEADEHDDDDDGAEEEKKKLRWDRVVSVIRD---DGNASGSAKSSDDGKE  984

Query  1327  ISAELKKSCIKPTASLDVHGNLDLTKLPPLTSIIKRTKVTYVRFVYDDDLPK-APAGEKK  1385
                E  KSCIK    LD HGN+   + P     +KRT+V      YD + P  APA   +
Sbjct  985   KDGEELKSCIKTKVPLDEHGNVLDAQRP--VDNLKRTRVVVTAVFYDGEEPVPAPATATR  1042

Query  1386  KKR  1388
              K+
Sbjct  1043  SKK  1045


>ref|XP_001833285.2| hypothetical protein CC1G_04264 [Coprinopsis cinerea okayama7#130]
 gb|EAU88558.2| hypothetical protein CC1G_04264 [Coprinopsis cinerea okayama7#130]
Length=1225

 Score = 56.6 bits (135),  Expect = 3e-04, Method: Compositional matrix adjust.
 Identities = 69/241 (29%), Positives = 95/241 (39%), Gaps = 41/241 (17%)

Query  1151  MLSKDLASLTDHNTIVNGVLFCALTRTVVRREGEHRPSSPGPKKKSPLAMAAASSSSSRS  1210
             M +  L SLT  NT+ N         T + R+   RP SP  K K+   +A       + 
Sbjct  985   MSATALKSLTAANTVRNQAYLSVKLETAIIRKDGARPESPAVKIKT---IAQREEEEKQK  1041

Query  1211  TSGAGTGRGRKVSFGS---ESEEEEEEEDGAWPGLNGGGSVSDQEKKTHLLGPGEDEVFV  1267
                    R  + S GS    S+ E + ED A P    G S  D     H  G GEDE + 
Sbjct  1042  LKMERAQRYARRSGGSIPGSSDVEPDSEDDALP----GASSMDVSPARHQRGAGEDEDYE  1097

Query  1268  SPIRKNRSSATCGRKQLWSGSTSAVEARRSSDRMVKWKKSL----VVSNSRSGADRTYQS  1323
             +P R          K++        E      R VKW + L     +   + G+ R Y  
Sbjct  1098  TPHR--------AFKRMRLTENGVEEEEEEEKRRVKWDRRLFSVTFLDEIKLGS-RPY--  1146

Query  1324  TEEISAELKKSCIKPTAS---LDVHGNL-----DLTKLPPLTSIIKRTKVTYVRFVYDDD  1375
              ++I+ +L K C+ P A    LD  GNL      L  LP    I+K       +FVYD D
Sbjct  1147  PKKITTQL-KGCLAPAAKQLPLDTLGNLPEDKAPLVDLPSENVIVK-------KFVYDSD  1198

Query  1376  L  1376
             +
Sbjct  1199  V  1199


>ref|XP_012051532.1| hypothetical protein CNAG_04434 [Cryptococcus neoformans var. 
grubii H99]
 gb|AFR97154.2| hypothetical protein CNAG_04434 [Cryptococcus neoformans var. 
grubii H99]
Length=1034

 Score = 56.2 bits (134),  Expect = 3e-04, Method: Compositional matrix adjust.
 Identities = 67/245 (27%), Positives = 98/245 (40%), Gaps = 30/245 (12%)

Query  1151  MLSKDLASLTDHNTIVNGVLFCALTRTVVRREGEHRPSSPGPKKKSPLAMAAASSSSSRS  1210
             +  K L + T  NT  N V  CA+ R V+R+ G  RP SP  K ++      A   +SR 
Sbjct  812   LTEKQLKTTTARNTARNQVYHCAIDRKVIRQRGP-RPPSPTSKIRTTSERDEAEKKNSRE  870

Query  1211  TSGAGTGRGRKVSFGSESEEEEEEEDGAWPGLNGGGSVSDQEKKTHLLGPGEDEVFVSPI  1270
                       K   G +SEEEE    G  P +         EK      PG+D+ +    
Sbjct  871   ARA-------KRRMGIQSEEEE----GKLPVI---------EKLEMAKAPGDDDDWTYKT  910

Query  1271  RKNRSSATCGRKQLWSGSTS--AVEARRSSDRMVKWKKSLVV----SNSRSGADRTYQST  1324
                  S    ++ L    TS    + +    + ++W + + V     N+   A       
Sbjct  911   PPPPPSQQQLQRPLKRARTSEGKEDKKGEEKKKLRWDRVVSVIRDDGNASGSAKSLSDDG  970

Query  1325  EEISAELKKSCIKPTASLDVHGNLDLTKLPPLTSIIKRTKVTYVRFVYDDDLP-KAPAGE  1383
             +E   E  KSCIK    LD HGN+   + P     +KRT+V      YD + P  APA  
Sbjct  971   KEKDGEELKSCIKTKVPLDEHGNVLDAQRP--VHNLKRTRVVVTAVFYDGEEPVPAPATA  1028

Query  1384  KKKKR  1388
              + K+
Sbjct  1029  TRSKK  1033


>gb|KDE04562.1| hypothetical protein, variant [Microbotryum violaceum p1A1 Lamole]
Length=1364

 Score = 56.2 bits (134),  Expect = 3e-04, Method: Compositional matrix adjust.
 Identities = 71/238 (30%), Positives = 100/238 (42%), Gaps = 25/238 (11%)

Query  1148  PPPMLSKD-LASLTDHNTIVNGVLFCALTRTVVRREGEHRPSSPGPKKKSPLAMAAASSS  1206
             P P+L++D L  LT  NT  N + F      V   E   RP SP  K +  +     S +
Sbjct  1145  PAPVLTQDELTRLTQRNTKKNQLHFNHHQVIVTHLEVP-RPPSPTSKIRKSIGEYGVSGA  1203

Query  1207  SSRSTSGAGTGRG---RKVSFGSESEEEEEEEDGAWPGLNGGGSVS--DQEKKTHLLGPG  1261
             +S         R    R  + GSE +    E D        GG V+  +Q K TH   PG
Sbjct  1204  TSVGREAKAAKRRNALRSSTDGSEVDLLNSELDATGGASATGGGVARVEQLKTTHYRAPG  1263

Query  1262  EDEVFVSPIRKNRSSATCGRKQLWSGSTSAVEARRSSDRMVKWKKSLVVSNSRSGADRTY  1321
             ++E + SP+R + SS     K+  SG+T       +  R VKW K+LV           Y
Sbjct  1264  DEEEYSSPVRPSTSSNE--NKKRGSGATKKTSVGSAPARRVKWDKALV-----------Y  1310

Query  1322  QSTEEISAELK-KSCIKPTASLDVHGNLDLTKLPPLTSIIKRTKVTYVRFVYDDDLPK  1378
             +   E   E+K    +K  A LD  GNL +       S+ K   V   + +Y DD P+
Sbjct  1311  EGPLEGQGEVKVDGILKVKAPLDSFGNLTVNN----GSLGKAVPVQIKKLIYIDDPPE  1364


>gb|KIR59288.1| hypothetical protein I314_04803 [Cryptococcus gattii CA1873]
Length=1046

 Score = 53.9 bits (128),  Expect = 0.001, Method: Compositional matrix adjust.
 Identities = 62/244 (25%), Positives = 100/244 (41%), Gaps = 23/244 (9%)

Query  1147  APPPMLSKDLASLTDHNTIVNGVLFCALTRTVVRREGEHRPSSPGPKKKSPLAMAAASSS  1206
             A P +  K L + T  NT  N V  CA+ R V+R+ G  RP SP  K ++      A   
Sbjct  823   AAPALTEKQLKTTTARNTARNQVYHCAIDRKVIRQRGP-RPPSPTSKIRTTSEREEADKK  881

Query  1207  SSRSTSGAGTGRGRKVSFGSESEEEEEEEDGAWPGLNGGGSVSDQEKKTHLLGPGEDEVF  1266
              SR           K   G  S+E  + +      L    +  D++  T+       +  
Sbjct  882   MSREARA-------KRRMGIHSDEMMDGKQQVIEKLEVAKAPGDEDDWTY-------KTP  927

Query  1267  VSPIRKNRSSATCGRKQLWSGSTSAVEARRSSDRMVKWKKSLVVSNSR-SGADRTYQSTE  1325
               P+++ R ++               + +   DR+V    S++  ++  SG+ ++    +
Sbjct  928   QRPLKRARRTSEADEHDDDDDGAEEEKKKLRWDRVV----SVIRDDANASGSAKSSDDGK  983

Query  1326  EISAELKKSCIKPTASLDVHGNLDLTKLPPLTSIIKRTKVTYVRFVYDDDLPK-APAGEK  1384
             E   E  KSCIK    LD HGN+   + P     +KRT+V      YD + P  APA   
Sbjct  984   EKDGEELKSCIKTKVPLDEHGNVLDAQRP--VDNLKRTRVVVTAVFYDGEEPVPAPATAT  1041

Query  1385  KKKR  1388
             + K+
Sbjct  1042  RSKK  1045


>gb|KIR88183.1| hypothetical protein I308_01243 [Cryptococcus gattii IND107]
Length=1029

 Score = 52.8 bits (125),  Expect = 0.003, Method: Compositional matrix adjust.
 Identities = 63/245 (26%), Positives = 100/245 (41%), Gaps = 27/245 (11%)

Query  1149  PPMLSKDLASLTDHNTIVNGVLFCALTRTVVRREGEHRPSSPGPKKKSPLAMAAASSSSS  1208
             P +  K L + T  NT  N V  CA+ R V+ + G  RP SP  K ++      A    S
Sbjct  806   PALTEKQLKTTTARNTARNQVYHCAIDRKVIHQRGP-RPPSPTSKIRTTSEREEADKKKS  864

Query  1209  RSTSGAGTGRGRKVSFGSESEEEEEEEDGAWPGLNGGGSVSDQEKKTHLLGPGEDEVFVS  1268
             R          R++   S+     E  DG       G  V ++ +     G  +D  + +
Sbjct  865   REARA-----KRRMGIHSD-----EMMDG-----KQGQPVIEKLEVAKAPGDEDDWTYKT  909

Query  1269  PIR-KNRSSATCGRKQLWSGSTSAVEARRSSDRMVKWKKSLVV---SNSRSGADRTYQST  1324
             P R   R+  T    +      +  E ++     ++W + + V     + SG+ ++    
Sbjct  910   PQRPLKRARRTSEADEHDDDDDNGAEEKKK----LRWDRVVSVIRDDGNASGSAKSSDDG  965

Query  1325  EEISAELKKSCIKPTASLDVHGNLDLTKLPPLTSIIKRTKVTYVRFVYDDDLP-KAPAGE  1383
             +E   E  KSCIK    LD HGN+   + P     +KRT+V      YD + P  APA  
Sbjct  966   KEKDGEELKSCIKTKVPLDEHGNVLDAQRPVYN--LKRTRVVVTAVFYDGEEPVPAPAAA  1023

Query  1384  KKKKR  1388
              + K+
Sbjct  1024  TRSKK  1028


>ref|XP_007882279.1| hypothetical protein PFL1_06545 [Pseudozyma flocculosa PF-1]
 gb|EPQ25871.1| hypothetical protein PFL1_06545 [Pseudozyma flocculosa PF-1]
Length=1815

 Score = 48.9 bits (115),  Expect = 0.059, Method: Compositional matrix adjust.
 Identities = 69/254 (27%), Positives = 104/254 (41%), Gaps = 43/254 (17%)

Query  1139  AMANPVVPAPP-----PMLSKDLASLTDHNTIVNGVLFCALTRTVVRREGEHRPSSPGPK  1193
             AMA P+ PA       P+ + +L  LT+ +T  NGV    L   V RR G+ RP SP  K
Sbjct  1587  AMAAPLGPATSSNSLLPLSATELTRLTNQHTRRNGVNAAKLLVKVERRGGQ-RPPSPSTK  1645

Query  1194  KKSPLAMAAASSSSSRSTSGAGTGRGR--------KVSFGSESEEEEEEEDGAWPGLNGG  1245
              K          S+S S   A   R R        +     + E + +EE  +W G    
Sbjct  1646  LKE---------SASSSLVAAERRRKRMQEDEYDDEFDDDDDDEADSDEERASWSG----  1692

Query  1246  GSVSDQEKKTHLLGPGEDEVFVSPIR---KNRSSATCGRKQLWSGSTSAVEARRSSDRMV  1302
                   E+K H +G G+DEV+ +P R     R    C  K+L++G +     + + +  +
Sbjct  1693  ------ERKRHRMGRGDDEVYTTPPRLASSQRRVRWC--KRLFAGPS----GKEAGELPL  1740

Query  1303  KWKKSLVVSNSRSGADRTYQSTEEISAELKKSCIKPT-ASLDVHGNLDLTKLPPLTSIIK  1361
                                   +E  A  +KS +      LD  GN D   L PL+  ++
Sbjct  1741  ADLGGGDGGGGDGDVGDGAGGRQERGARPRKSSLASRDYKLDRFGNPDEATLQPLSPRMR  1800

Query  1362  RTKVTYVRFVYDDD  1375
             + KVT  + VYD +
Sbjct  1801  KVKVTIHKIVYDGE  1814


>emb|CUA69368.1| Proteoglycan 4 [Rhizoctonia solani]
Length=1011

 Score = 47.8 bits (112),  Expect = 0.11, Method: Compositional matrix adjust.
 Identities = 52/221 (24%), Positives = 81/221 (37%), Gaps = 50/221 (23%)

Query  1154  KDLASLTDHNTIVNGVLFCALTRTVVRREGEHRPSSPGPKKKSPLAMAAASSSSSRSTSG  1213
             K+L  LT  NT  N    C +           RP+SPG                SR  + 
Sbjct  829   KELQKLTMKNTRQNNTYECVVVERAETYVDRPRPTSPG----------------SRVPTK  872

Query  1214  AGTGRGRKVSFGSESEEEEEEEDGAWPGLNGGGSVSDQEKKTHLLGPGEDEVFVSPIRKN  1273
             A   R        +S +E  +   A     G  S++    +   L PG+D  +V P+RK 
Sbjct  873   AQKAR----VLERQSRDERAQRRSAARDGEGDSSLTSTGVEERRLAPGDDVEWVEPVRKG  928

Query  1274  RSSATCGRKQLWSGSTSAVEARRSSDRMVKWKKSLVVSNSRSGADRTYQSTEEISAELKK  1333
                                       R V+W ++L   +   G  RT +++  +S  + +
Sbjct  929   --------------------------RHVRWAQTLAHDDVDKGP-RTPETSAAVS--VGR  959

Query  1334  SCIKPTASLDVHGNLDLTKLPPLTSIIKRTKVTYVRFVYDD  1374
              C+  T  LD HGN  +   P     +K  +V   R+VY+D
Sbjct  960   GCLARTYELDRHGN-SVNPAPGPAPTLKPLRVVITRYVYND  999


>gb|EGN92458.1| hypothetical protein SERLA73DRAFT_79593 [Serpula lacrymans var. 
lacrymans S7.3]
Length=1300

 Score = 45.8 bits (107),  Expect = 0.39, Method: Compositional matrix adjust.
 Identities = 61/236 (26%), Positives = 94/236 (40%), Gaps = 39/236 (17%)

Query  1156  LASLTDHNTIVNGVLFCALTRTVVRREGEHRPSSPGPKKKSPLAMAAASSSSSR------  1209
             L +LT  NT  N      L   VVR+EG  RP SP  K ++ L          R      
Sbjct  1064  LKALTSSNTTKNQHFSVKLETKVVRKEG-IRPESPTMKARTILQKQREEKDKQRKDRAAR  1122

Query  1210  ----STSGAGTGRGRKVSFGSESEEEEEEEDGAWPGLNGGGSVSDQEK-KTHLLGPGEDE  1264
                 S  G   G    V+   +    E ++D            SD+ +   H  GPG++E
Sbjct  1123  RARRSEDGPELGDADAVTESGDRSAMESDDD----------ENSDRARLGKHRRGPGDEE  1172

Query  1265  VFVSPIRKNRSSATCGRKQLWSGSTSAVEARRSSDRMVKWKKSLVVSNSRSGADRTY--Q  1322
              + +P R +R      R +   G    VE ++   + VKW + L  +      D  +   
Sbjct  1173  DYETPERISRP---VKRLKCVDGE-GQVEVQK---KQVKWDRGLSTT---IYLDELHPKP  1222

Query  1323  STEEISAELKKSCIKPTAS---LDVHGNLDLTKLPPLTSIIKRTKVTYVRFVYDDD  1375
              T  ++   +K C+ P      LD  GN+ +    PLT ++    +T  +FVYD+D
Sbjct  1223  KTPSMNRSTRKGCLTPAVKALRLDTLGNI-MNANTPLTDLVHEN-ITVTKFVYDND  1276


>gb|EMS25332.1| hypothetical protein RHTO_03060 [Rhodosporidium toruloides NP11]
 emb|CDR43176.1| RHTO0S07e09142g1_1 [Rhodosporidium toruloides]
Length=1336

 Score = 42.7 bits (99),  Expect = 3.7, Method: Compositional matrix adjust.
 Identities = 73/269 (27%), Positives = 109/269 (41%), Gaps = 39/269 (14%)

Query  1118  PTVLTPPPPVPPLPSALAAPPAMANPVVP-APPPMLSKD-LASLTDHNTIVNGVLFCALT  1175
             PTV++PP PV        A PA   P+    P P +++D L  LT  NT  N V F  + 
Sbjct  1095  PTVVSPPAPV------EDAQPATTRPLSSFRPAPAVTQDELNRLTQRNTKKNQVAFNRIK  1148

Query  1176  RTVVRREGEHRPSSPGPKKKSPL----AMAAASSSSSRSTSGAGTGRGRKVSF-GSESEE  1230
                V  +   RP SP  K +       ++A  ++   R    A      + S  GSE   
Sbjct  1149  LETVFLDC-ARPPSPTSKIRKAFGSEGSLARTTTKEGREARAAKRRNALRSSLDGSELAA  1207

Query  1231  EEEEEDGAWPGLNGGGSVSDQEKKTHLLGPGEDEVFVSPIRKNRS--SATCGRKQ--LWS  1286
               EE          G S+  +  K H    G+DE + +P +   +  SA  GRK+     
Sbjct  1208  LAEELKA-----ESGSSMEAEPPKQHFRAAGDDEQYFTPQKAGGAWKSAAVGRKRSPGSG  1262

Query  1287  GSTSAVEARRSSDRMVKWKKSLVVSNSRSGADRTYQSTEEISAELKKSCIKPTASLDVHG  1346
              S+++    R   + VKW ++LV        DR  +S  +       S +KP   LD  G
Sbjct  1263  SSSASASPSRQETKRVKWDRALVYEGP---LDRDARSNGD-------SILKPV-DLDAWG  1311

Query  1347  NLDLTKLPPLTSIIKRTKVTYVRFVYDDD  1375
             N        + S+ K T +T    V+ D+
Sbjct  1312  NSTS-----IASLGKPTSITIRMRVFKDE  1335


Lambda      K        H        a         alpha
   0.305    0.123    0.343    0.792     4.96 

Gapped
Lambda      K        H        a         alpha    sigma
   0.267   0.0410    0.140     1.90     42.6     43.6 

Effective search space used: 17569748855667


  Database: nr
    Posted date:  Sep 23, 2015 12:05 AM
  Number of letters in database: 26,053,659,533
  Number of sequences in database:  71,551,133


Matrix: BLOSUM62
Gap Penalties: Existence: 11, Extension: 1
Neighboring words threshold: 11
Window for multiple hits: 40
```
