# Supplementary material for: Comparative genomics provides new insights into the diversity, physiology, and sexuality of the only industrially exploited tremellomycete: Phaffia rhodozyma
Source: BMC Genomics. 2016 Nov 9;17:901. doi: 10.1186/s12864-016-3244-7 (PMC5103461; doi:10.1186/s12864-016-3244-7)
Supplement: Additional file 6: — List of orphan genes with links to PFAM (related to Additional file 1: Table S1). (ZIP 1428 kb) [file 12864_2016_3244_MOESM6_ESM.zip › BLAST_HTML_FTR/G05909_P.html]

BLAST Search Results


```
BLASTP 2.2.27+


Reference:
Stephen F. Altschul, Thomas L. Madden, Alejandro A. Schäffer,
Jinghui Zhang, Zheng Zhang, Webb Miller, and David J. Lipman (1997),
"Gapped BLAST and PSI-BLAST: a new generation of protein database
search programs", Nucleic Acids Res. 25:3389-3402.


Reference for
composition-based statistics:
Alejandro A. Schäffer, L. Aravind, Thomas L. Madden, Sergei
Shavirin, John L. Spouge, Yuri I. Wolf, Eugene V. Koonin, and
Stephen F. Altschul (2001), "Improving the accuracy of PSI-BLAST
protein database searches with composition-based statistics and
other refinements", Nucleic Acids Res. 29:2994-3005.


Database: nr
           71,551,133 sequences; 26,053,659,533 total letters


Query= G05909_P

Length=381
                                                                      Score     E
Sequences producing significant alignments:                          (Bits)  Value

emb|CDZ97237.1|  hypothetical protein [Xanthophyllomyces dendrorh...   717    0.0  
gb|KIO26746.1|  hypothetical protein M407DRAFT_197114 [Tulasnella...  45.4    0.041
ref|XP_007867541.1|  hypothetical protein GLOTRDRAFT_139571 [Gloe...  41.2    1.3  


 >emb|CDZ97237.1| hypothetical protein [Xanthophyllomyces dendrorhous]
Length=380

 Score =  717 bits (1850),  Expect = 0.0, Method: Compositional matrix adjust.
 Identities = 380/380 (100%), Positives = 380/380 (100%), Gaps = 0/380 (0%)

Query  1    MSNPIPISSAPSHPLPVPVPNTHQSSSSSRSFSHSYSHPYPFSQGSPGTSFSPTNPSPIL  60
            MSNPIPISSAPSHPLPVPVPNTHQSSSSSRSFSHSYSHPYPFSQGSPGTSFSPTNPSPIL
Sbjct  1    MSNPIPISSAPSHPLPVPVPNTHQSSSSSRSFSHSYSHPYPFSQGSPGTSFSPTNPSPIL  60

Query  61   SSALNMPGPPVGLSLTSSSFTQTRSFLGGAHPNTSLFYAAGSSPPAHLFPSTYTGPSSSS  120
            SSALNMPGPPVGLSLTSSSFTQTRSFLGGAHPNTSLFYAAGSSPPAHLFPSTYTGPSSSS
Sbjct  61   SSALNMPGPPVGLSLTSSSFTQTRSFLGGAHPNTSLFYAAGSSPPAHLFPSTYTGPSSSS  120

Query  121  SSSSISTPASSARRSFSATSKPTLGLSLPSTSPHLPSTSSSSGSPPQKDCQWATAWASGI  180
            SSSSISTPASSARRSFSATSKPTLGLSLPSTSPHLPSTSSSSGSPPQKDCQWATAWASGI
Sbjct  121  SSSSISTPASSARRSFSATSKPTLGLSLPSTSPHLPSTSSSSGSPPQKDCQWATAWASGI  180

Query  181  NGSVAAGIKSPGLYGGSALADEDEDDDSLPGPLASSLPILTIPAASTAQKSLPQGPGPGP  240
            NGSVAAGIKSPGLYGGSALADEDEDDDSLPGPLASSLPILTIPAASTAQKSLPQGPGPGP
Sbjct  181  NGSVAAGIKSPGLYGGSALADEDEDDDSLPGPLASSLPILTIPAASTAQKSLPQGPGPGP  240

Query  241  GAGPSSQTAGGPPTRRVSIAQPPQESSQRGQNVLRRLSLSSGSLLRPSIPAPSPITPPSS  300
            GAGPSSQTAGGPPTRRVSIAQPPQESSQRGQNVLRRLSLSSGSLLRPSIPAPSPITPPSS
Sbjct  241  GAGPSSQTAGGPPTRRVSIAQPPQESSQRGQNVLRRLSLSSGSLLRPSIPAPSPITPPSS  300

Query  301  QPTTAISYSFPNATPGSGPSVQFSNSFEPAQHQPRPAEIKAPSVPSIGPRRKSVKSSGGK  360
            QPTTAISYSFPNATPGSGPSVQFSNSFEPAQHQPRPAEIKAPSVPSIGPRRKSVKSSGGK
Sbjct  301  QPTTAISYSFPNATPGSGPSVQFSNSFEPAQHQPRPAEIKAPSVPSIGPRRKSVKSSGGK  360

Query  361  RAISPTGERMLVGHAGGGMF  380
            RAISPTGERMLVGHAGGGMF
Sbjct  361  RAISPTGERMLVGHAGGGMF  380


>gb|KIO26746.1| hypothetical protein M407DRAFT_197114 [Tulasnella calospora MUT 
4182]
Length=245

 Score = 45.4 bits (106),  Expect = 0.041, Method: Compositional matrix adjust.
 Identities = 48/131 (37%), Positives = 63/131 (48%), Gaps = 25/131 (19%)

Query  254  TRRVSIAQPPQES-------SQRGQNVLRRLSLSSGSLLRPSIPAPSPITPPSSQPTTAI  306
            TRR S AQ P +S       S RG  VLRRLSLS+    RP+    + +  P++ P +A+
Sbjct  131  TRRGSWAQNPLQSTTGQVPTSDRGTGVLRRLSLSN--TFRPNFSTSTNV--PAAPPPSAV  186

Query  307  SYSFPNATPGSGPSVQFSNSFEPAQHQPRPAEIKAPSVPSIGPRRKSVKSSGGKRAISPT  366
            S S P  + G            P   +PRP   + P   S+       + SG KR ISP 
Sbjct  187  SAS-PVLSRGV-----------PTLGEPRPG--RKPRSASLAVPGSGQEGSGKKRGISPM  232

Query  367  GERMLVGHAGG  377
            GER+L GH  G
Sbjct  233  GERLLKGHFDG  243


>ref|XP_007867541.1| hypothetical protein GLOTRDRAFT_139571 [Gloeophyllum trabeum 
ATCC 11539]
 gb|EPQ54235.1| hypothetical protein GLOTRDRAFT_139571 [Gloeophyllum trabeum 
ATCC 11539]
Length=265

 Score = 41.2 bits (95),  Expect = 1.3, Method: Compositional matrix adjust.
 Identities = 45/111 (41%), Positives = 54/111 (49%), Gaps = 13/111 (12%)

Query  269  RGQNVLRRLSLSSGSLLRPSIPAPSPITPPSSQPTTAISYSFPNATPGSGPSVQFSNSF-  327
            RG  +LRRLSLS G+  RP IP P   T PS+          P  TP S PS    +SF 
Sbjct  164  RGAGLLRRLSLS-GAFARPPIPGP---TSPSAN------VHVPPPTPMS-PSAAPPSSFA  212

Query  328  -EPAQHQPRPAEIKAPSVPSIGPRRKSVKSSGGKRAISPTGERMLVGHAGG  377
             EP  +        A S P   PR  +V +   +RA SP GER+L GH  G
Sbjct  213  AEPVSNMSSVPNPPASSSPRRKPRSATVNTGSIRRAPSPMGERILKGHFDG  263


Lambda      K        H        a         alpha
   0.308    0.125    0.368    0.792     4.96 

Gapped
Lambda      K        H        a         alpha    sigma
   0.267   0.0410    0.140     1.90     42.6     43.6 

Effective search space used: 3475736195593


  Database: nr
    Posted date:  Sep 23, 2015 12:05 AM
  Number of letters in database: 26,053,659,533
  Number of sequences in database:  71,551,133


Matrix: BLOSUM62
Gap Penalties: Existence: 11, Extension: 1
Neighboring words threshold: 11
Window for multiple hits: 40
```
