# Supplementary material for: Comparative genomics provides new insights into the diversity, physiology, and sexuality of the only industrially exploited tremellomycete: Phaffia rhodozyma
Source: BMC Genomics. 2016 Nov 9;17:901. doi: 10.1186/s12864-016-3244-7 (PMC5103461; doi:10.1186/s12864-016-3244-7)
Supplement: Additional file 6: — List of orphan genes with links to PFAM (related to Additional file 1: Table S1). (ZIP 1428 kb) [file 12864_2016_3244_MOESM6_ESM.zip › BLAST_HTML_FTR/G01261_P.html]

BLAST Search Results


```
BLASTP 2.2.27+


Reference:
Stephen F. Altschul, Thomas L. Madden, Alejandro A. Schäffer,
Jinghui Zhang, Zheng Zhang, Webb Miller, and David J. Lipman (1997),
"Gapped BLAST and PSI-BLAST: a new generation of protein database
search programs", Nucleic Acids Res. 25:3389-3402.


Reference for
composition-based statistics:
Alejandro A. Schäffer, L. Aravind, Thomas L. Madden, Sergei
Shavirin, John L. Spouge, Yuri I. Wolf, Eugene V. Koonin, and
Stephen F. Altschul (2001), "Improving the accuracy of PSI-BLAST
protein database searches with composition-based statistics and
other refinements", Nucleic Acids Res. 29:2994-3005.


Database: nr
           71,551,133 sequences; 26,053,659,533 total letters


Query= G01261_P

Length=166
                                                                      Score     E
Sequences producing significant alignments:                          (Bits)  Value

emb|CED85555.1|  hypothetical protein [Xanthophyllomyces dendrorh...   328    5e-112
gb|KIK54643.1|  hypothetical protein GYMLUDRAFT_249360 [Gymnopus ...  38.1    3.4   
ref|XP_001313447.1|  hypothetical protein [Trichomonas vaginalis ...  36.6    9.3   


 >emb|CED85555.1| hypothetical protein [Xanthophyllomyces dendrorhous]
Length=165

 Score =  328 bits (840),  Expect = 5e-112, Method: Compositional matrix adjust.
 Identities = 165/165 (100%), Positives = 165/165 (100%), Gaps = 0/165 (0%)

Query  1    MDTPINTSAPTSLDSTSFSHPAPSIPTPTTDTELYSSDEDPSTNPIGGDGGSAFETPCSS  60
            MDTPINTSAPTSLDSTSFSHPAPSIPTPTTDTELYSSDEDPSTNPIGGDGGSAFETPCSS
Sbjct  1    MDTPINTSAPTSLDSTSFSHPAPSIPTPTTDTELYSSDEDPSTNPIGGDGGSAFETPCSS  60

Query  61   QQSNPSRNPESSLRESLWSATSTNEPKGLVRGEGRRGRQNDGENGQNGGRLSSQMEATGE  120
            QQSNPSRNPESSLRESLWSATSTNEPKGLVRGEGRRGRQNDGENGQNGGRLSSQMEATGE
Sbjct  61   QQSNPSRNPESSLRESLWSATSTNEPKGLVRGEGRRGRQNDGENGQNGGRLSSQMEATGE  120

Query  121  GASGPNGGTYAQGGTKDDRLLGEREFDALNEEIGDPFDTDEIDDF  165
            GASGPNGGTYAQGGTKDDRLLGEREFDALNEEIGDPFDTDEIDDF
Sbjct  121  GASGPNGGTYAQGGTKDDRLLGEREFDALNEEIGDPFDTDEIDDF  165


>gb|KIK54643.1| hypothetical protein GYMLUDRAFT_249360 [Gymnopus luxurians FD-317 
M1]
Length=561

 Score = 38.1 bits (87),  Expect = 3.4, Method: Compositional matrix adjust.
 Identities = 29/91 (32%), Positives = 42/91 (46%), Gaps = 23/91 (25%)

Query  10   PTSLDSTSFSHPAPSIPTPTTDTELYSSDEDPSTNPIGGDGGSAFETPCSSQQSNPSRN-  68
            P+S  ST+ S P P  PTP T+  L  +  D  T+P          TP S+Q +NP R+ 
Sbjct  283  PSSQPSTALSRPEPRWPTPATEPSLIVAYHDILTHP----------TPSSAQTANPQRHK  332

Query  69   ------------PESSLRESLWSATSTNEPK  87
                        P   L ++L+S   TN+P+
Sbjct  333  VAMALLALTQSTPRWDLPDTLFSNLVTNQPR  363


>ref|XP_001313447.1| hypothetical protein [Trichomonas vaginalis G3]
 gb|EAY00518.1| conserved hypothetical protein [Trichomonas vaginalis G3]
Length=606

 Score = 36.6 bits (83),  Expect = 9.3, Method: Compositional matrix adjust.
 Identities = 25/77 (32%), Positives = 39/77 (51%), Gaps = 10/77 (13%)

Query  1    MDTPINTSAPTSLDSTSFSHPAPSIPTPTTDTELYSSDEDPSTNPIGGDGGSAFETPCSS  60
            ++TP  T A T  D+  F+ P  +       T  Y++++ PS++PIG    +AFETP  +
Sbjct  405  IETPFETFAITPFDTNHFT-PFETF----VQTHFYTAEQTPSSSPIGQKDRTAFETPVRT  459

Query  61   QQSNPSRN-----PESS  72
                P +      PESS
Sbjct  460  FYLTPMQTEKIPVPESS  476


Lambda      K        H        a         alpha
   0.306    0.129    0.376    0.792     4.96 

Gapped
Lambda      K        H        a         alpha    sigma
   0.267   0.0410    0.140     1.90     42.6     43.6 

Effective search space used: 642014351342


  Database: nr
    Posted date:  Sep 23, 2015 12:05 AM
  Number of letters in database: 26,053,659,533
  Number of sequences in database:  71,551,133


Matrix: BLOSUM62
Gap Penalties: Existence: 11, Extension: 1
Neighboring words threshold: 11
Window for multiple hits: 40
```
