# Supplementary material for: Comparative genomics provides new insights into the diversity, physiology, and sexuality of the only industrially exploited tremellomycete: Phaffia rhodozyma
Source: BMC Genomics. 2016 Nov 9;17:901. doi: 10.1186/s12864-016-3244-7 (PMC5103461; doi:10.1186/s12864-016-3244-7)
Supplement: Additional file 6: — List of orphan genes with links to PFAM (related to Additional file 1: Table S1). (ZIP 1428 kb) [file 12864_2016_3244_MOESM6_ESM.zip › BLAST_HTML_FTR/G03610_P.html]

BLAST Search Results


```
BLASTP 2.2.27+


Reference:
Stephen F. Altschul, Thomas L. Madden, Alejandro A. Schäffer,
Jinghui Zhang, Zheng Zhang, Webb Miller, and David J. Lipman (1997),
"Gapped BLAST and PSI-BLAST: a new generation of protein database
search programs", Nucleic Acids Res. 25:3389-3402.


Reference for
composition-based statistics:
Alejandro A. Schäffer, L. Aravind, Thomas L. Madden, Sergei
Shavirin, John L. Spouge, Yuri I. Wolf, Eugene V. Koonin, and
Stephen F. Altschul (2001), "Improving the accuracy of PSI-BLAST
protein database searches with composition-based statistics and
other refinements", Nucleic Acids Res. 29:2994-3005.


Database: nr
           71,551,133 sequences; 26,053,659,533 total letters


Query= G03610_P

Length=540
                                                                      Score     E
Sequences producing significant alignments:                          (Bits)  Value

emb|CED84945.1|  hypothetical protein [Xanthophyllomyces dendrorh...  1048    0.0  
ref|XP_008635069.1|  PREDICTED: laminin subunit alpha-3 [Corvus b...  40.8    4.5  
ref|XP_010394743.1|  PREDICTED: LOW QUALITY PROTEIN: laminin subu...  40.8    4.5  
gb|AEV37249.1|  ABC transporter ATP-binding protein [Pseudovibrio...  40.0    8.0  
ref|WP_041769004.1|  glycosyl transferase family 1 [Pseudovibrio ...  39.7    8.5  
gb|EEA92775.1|  ABC transporter, nucleotide binding/ATPase protei...  39.7    8.8  
ref|WP_037037932.1|  glycosyl transferase family 1 [Pseudovibrio ...  39.7    9.0  


 >emb|CED84945.1| hypothetical protein [Xanthophyllomyces dendrorhous]
Length=583

 Score = 1048 bits (2709),  Expect = 0.0, Method: Compositional matrix adjust.
 Identities = 539/583 (92%), Positives = 539/583 (92%), Gaps = 44/583 (8%)

Query  1    MSTLRKPEELFEWLTVDLAFPASQTLKLSQFTRLCRGSLHLLLHLLSLHLLGIVPTLRTR  60
            MSTLRKPEELFEWLTVDLAFPASQTLKLSQFTRLCRGSLHLLLHLLSLHLLGIVPTLRTR
Sbjct  1    MSTLRKPEELFEWLTVDLAFPASQTLKLSQFTRLCRGSLHLLLHLLSLHLLGIVPTLRTR  60

Query  61   SELHRFQALSISNQLPGPTRSLLFPSESSYATCHRTHAHMLRLCDKLAKAQKELNSILAL  120
            SELHRFQALSISNQLPGPTRSLLFPSESSYATCHRTHAHMLRLCDKLAKAQKELNSILAL
Sbjct  61   SELHRFQALSISNQLPGPTRSLLFPSESSYATCHRTHAHMLRLCDKLAKAQKELNSILAL  120

Query  121  TQDGERSRDQEKERGVRQSGRLKVLEETKIGWIETWERLEKLRGMDAAESGTTSLDHIIL  180
            TQDGERSRDQEKERGVRQSGRLKVLEETKIGWIETWERLEKLRGMDAAESGTTSLDHIIL
Sbjct  121  TQDGERSRDQEKERGVRQSGRLKVLEETKIGWIETWERLEKLRGMDAAESGTTSLDHIIL  180

Query  181  QKVLDQLTRFVDSATKSKSRATQYPTSLDIFNIRDL-----------------AERQDTK  223
            QKVLDQLTRFVDSATKSKSRATQYPTSLDIFNIRDL                 AERQDTK
Sbjct  181  QKVLDQLTRFVDSATKSKSRATQYPTSLDIFNIRDLFEPLKEWITKINHRRERAERQDTK  240

Query  224  GGDNTLEMDHLSDTLFDLHRLHIELAGRSFSKTQSSAKLSSDMEPERPAEDFVGHNQRNY  283
            GGDNTLEMDHLSDTLFDLHRLHIELAGRSFSKTQSSAKLSSDMEPERPAEDFVGHNQRNY
Sbjct  241  GGDNTLEMDHLSDTLFDLHRLHIELAGRSFSKTQSSAKLSSDMEPERPAEDFVGHNQRNY  300

Query  284  RLKWEEKLRRRLEDRYNGDQDKVQEKMKSVLQKVQQRTDIAFEKQINQTSIEAIMNNRSI  343
            RLKWEEKLRRRLEDRYNGDQDKVQEKMKSVLQKVQQRTDIAFEKQINQTSIEAIMNNRSI
Sbjct  301  RLKWEEKLRRRLEDRYNGDQDKVQEKMKSVLQKVQQRTDIAFEKQINQTSIEAIMNNRSI  360

Query  344  DAVKLNNLKKVWKGREERKLYLDALDVQW---------------------------IDKT  376
            DAVKLNNLKKVWKGREERKLYLDALDVQW                           IDKT
Sbjct  361  DAVKLNNLKKVWKGREERKLYLDALDVQWRKITRDVYSLLQVHERLVQSISQHTQSIDKT  420

Query  377  CIVRDNVLDAIRSSIVGLEVELENQVSQTCQKEDATSGHPNARTKNGLQEEVFGKLAELT  436
            CIVRDNVLDAIRSSIVGLEVELENQVSQTCQKEDATSGHPNARTKNGLQEEVFGKLAELT
Sbjct  421  CIVRDNVLDAIRSSIVGLEVELENQVSQTCQKEDATSGHPNARTKNGLQEEVFGKLAELT  480

Query  437  FEGKVLVMERALRERAALTKMIDALGHFREALTAAEKFVDTNTLLNTENSQSMKWKSQLD  496
            FEGKVLVMERALRERAALTKMIDALGHFREALTAAEKFVDTNTLLNTENSQSMKWKSQLD
Sbjct  481  FEGKVLVMERALRERAALTKMIDALGHFREALTAAEKFVDTNTLLNTENSQSMKWKSQLD  540

Query  497  KLHDALSQRSLDIQEAGKEFSVYKHEMDALMTGKIGQRGKKLG  539
            KLHDALSQRSLDIQEAGKEFSVYKHEMDALMTGKIGQRGKKLG
Sbjct  541  KLHDALSQRSLDIQEAGKEFSVYKHEMDALMTGKIGQRGKKLG  583


>ref|XP_008635069.1| PREDICTED: laminin subunit alpha-3 [Corvus brachyrhynchos]
Length=3242

 Score = 40.8 bits (94),  Expect = 4.5, Method: Composition-based stats.
 Identities = 43/163 (26%), Positives = 81/163 (50%), Gaps = 13/163 (8%)

Query  105   DKLAKAQKELNSILALTQDGERSRDQE---KERGVRQSGRLKVL--EETKIGWIETWERL  159
             D+L   + +L S+ A T   E+ RD E   K+  V  +    V+  + +K+  +ET E L
Sbjct  1757  DELQLIKSQLQSVHASTHTLEQMRDLETRIKDLKVLLNNYRSVVHNQGSKVDELET-EFL  1815

Query  160   EKLRGMDAAESGTTSLDHIILQKVLDQLTRFVDSATKSKSRATQYPTSLDIFNIRDLAER  219
             +  R ++A +     +++   +++ +    F  +  K K   +Q    ++  NI+ L E+
Sbjct  1816  KLDRDLNALQE-KAEMNYKTAERLFNN---FGQTQQKGKDLVSQIQIVVN--NIQVLLEQ  1869

Query  220   -QDTKGGDNTLEMDHLSDTLFDLHRLHIELAGRSFSKTQSSAK  261
                T GG N L +   S+ L + HR+  E+  R+FS+ Q+ A+
Sbjct  1870  IAGTNGGGNNLPLGDASEELAEAHRMMAEMRNRNFSQLQAEAE  1912


>ref|XP_010394743.1| PREDICTED: LOW QUALITY PROTEIN: laminin subunit alpha-3 [Corvus 
cornix cornix]
Length=3290

 Score = 40.8 bits (94),  Expect = 4.5, Method: Composition-based stats.
 Identities = 43/163 (26%), Positives = 81/163 (50%), Gaps = 13/163 (8%)

Query  105   DKLAKAQKELNSILALTQDGERSRDQE---KERGVRQSGRLKVL--EETKIGWIETWERL  159
             D+L   + +L S+ A T   E+ RD E   K+  V  +    V+  + +K+  +ET E L
Sbjct  1805  DELQLIKSQLQSVHASTHTLEQMRDLETRIKDLKVLLNNYRSVVHNQGSKVDELET-EFL  1863

Query  160   EKLRGMDAAESGTTSLDHIILQKVLDQLTRFVDSATKSKSRATQYPTSLDIFNIRDLAER  219
             +  R ++A +     +++   +++ +    F  +  K K   +Q    ++  NI+ L E+
Sbjct  1864  KLDRDLNALQE-KAEMNYKTAERLFNN---FGQTQQKGKDLVSQIQIVVN--NIQVLLEQ  1917

Query  220   -QDTKGGDNTLEMDHLSDTLFDLHRLHIELAGRSFSKTQSSAK  261
                T GG N L +   S+ L + HR+  E+  R+FS+ Q+ A+
Sbjct  1918  IAGTNGGGNNLPLGDASEELAEAHRMMAEMRNRNFSQLQAEAE  1960


>gb|AEV37249.1| ABC transporter ATP-binding protein [Pseudovibrio sp. FO-BEG1]
Length=657

 Score = 40.0 bits (92),  Expect = 8.0, Method: Compositional matrix adjust.
 Identities = 22/59 (37%), Positives = 36/59 (61%), Gaps = 0/59 (0%)

Query  106  KLAKAQKELNSILALTQDGERSRDQEKERGVRQSGRLKVLEETKIGWIETWERLEKLRG  164
            K  K  K+L+++LA  +    + D+ KE  +R++  +KVLEET+  W+E    LE+L G
Sbjct  598  KYQKYIKKLDALLADPRIYTEAPDKAKEHAIRRAAYVKVLEETEENWLEMSAELEELSG  656


>ref|WP_041769004.1| glycosyl transferase family 1 [Pseudovibrio sp. FO-BEG1]
Length=630

 Score = 39.7 bits (91),  Expect = 8.5, Method: Compositional matrix adjust.
 Identities = 22/59 (37%), Positives = 36/59 (61%), Gaps = 0/59 (0%)

Query  106  KLAKAQKELNSILALTQDGERSRDQEKERGVRQSGRLKVLEETKIGWIETWERLEKLRG  164
            K  K  K+L+++LA  +    + D+ KE  +R++  +KVLEET+  W+E    LE+L G
Sbjct  571  KYQKYIKKLDALLADPRIYTEAPDKAKEHAIRRAAYVKVLEETEENWLEMSAELEELSG  629


>gb|EEA92775.1| ABC transporter, nucleotide binding/ATPase protein [Pseudovibrio 
sp. JE062]
Length=650

 Score = 39.7 bits (91),  Expect = 8.8, Method: Compositional matrix adjust.
 Identities = 22/59 (37%), Positives = 36/59 (61%), Gaps = 0/59 (0%)

Query  106  KLAKAQKELNSILALTQDGERSRDQEKERGVRQSGRLKVLEETKIGWIETWERLEKLRG  164
            K  K  K+L+++LA  +    + D+ KE  +R++  +KVLEET+  W+E    LE+L G
Sbjct  591  KYQKYIKKLDALLADPRIYTEAPDKAKEHAIRRAAYVKVLEETEENWLEMSAELEELSG  649


>ref|WP_037037932.1| glycosyl transferase family 1 [Pseudovibrio sp. JE062]
Length=630

 Score = 39.7 bits (91),  Expect = 9.0, Method: Compositional matrix adjust.
 Identities = 22/59 (37%), Positives = 36/59 (61%), Gaps = 0/59 (0%)

Query  106  KLAKAQKELNSILALTQDGERSRDQEKERGVRQSGRLKVLEETKIGWIETWERLEKLRG  164
            K  K  K+L+++LA  +    + D+ KE  +R++  +KVLEET+  W+E    LE+L G
Sbjct  571  KYQKYIKKLDALLADPRIYTEAPDKAKEHAIRRAAYVKVLEETEENWLEMSAELEELSG  629


Lambda      K        H        a         alpha
   0.317    0.131    0.370    0.792     4.96 

Gapped
Lambda      K        H        a         alpha    sigma
   0.267   0.0410    0.140     1.90     42.6     43.6 

Effective search space used: 5718406189440


  Database: nr
    Posted date:  Sep 23, 2015 12:05 AM
  Number of letters in database: 26,053,659,533
  Number of sequences in database:  71,551,133


Matrix: BLOSUM62
Gap Penalties: Existence: 11, Extension: 1
Neighboring words threshold: 11
Window for multiple hits: 40
```
