# Supplementary material for: Comparative genomics provides new insights into the diversity, physiology, and sexuality of the only industrially exploited tremellomycete: Phaffia rhodozyma
Source: BMC Genomics. 2016 Nov 9;17:901. doi: 10.1186/s12864-016-3244-7 (PMC5103461; doi:10.1186/s12864-016-3244-7)
Supplement: Additional file 6: — List of orphan genes with links to PFAM (related to Additional file 1: Table S1). (ZIP 1428 kb) [file 12864_2016_3244_MOESM6_ESM.zip › BLAST_HTML_FTR/G03783_P.html]

BLAST Search Results


```
BLASTP 2.2.27+


Reference:
Stephen F. Altschul, Thomas L. Madden, Alejandro A. Schäffer,
Jinghui Zhang, Zheng Zhang, Webb Miller, and David J. Lipman (1997),
"Gapped BLAST and PSI-BLAST: a new generation of protein database
search programs", Nucleic Acids Res. 25:3389-3402.


Reference for
composition-based statistics:
Alejandro A. Schäffer, L. Aravind, Thomas L. Madden, Sergei
Shavirin, John L. Spouge, Yuri I. Wolf, Eugene V. Koonin, and
Stephen F. Altschul (2001), "Improving the accuracy of PSI-BLAST
protein database searches with composition-based statistics and
other refinements", Nucleic Acids Res. 29:2994-3005.


Database: nr
           71,551,133 sequences; 26,053,659,533 total letters


Query= G03783_P

Length=1269
                                                                      Score     E
Sequences producing significant alignments:                          (Bits)  Value

emb|CDZ98058.1|  hypothetical protein [Xanthophyllomyces dendrorh...  2233    0.0  
gb|KNZ59051.1|  hypothetical protein VP01_180g6 [Puccinia sorghi]     53.1    0.002
gb|EPE07756.1|  myb dna binding protein [Ophiostoma piceae UAMH 1...  48.9    0.050
gb|KNF05609.1|  hypothetical protein PSTG_01418 [Puccinia striifo...  47.8    0.12 
gb|ENH88019.1|  myb dna binding protein [Colletotrichum orbicular...  44.7    0.79 
ref|XP_501039.1|  YALI0B18018p [Yarrowia lipolytica] >emb|CAG8329...  43.9    1.3  


 >emb|CDZ98058.1| hypothetical protein [Xanthophyllomyces dendrorhous]
Length=1248

 Score = 2233 bits (5786),  Expect = 0.0, Method: Compositional matrix adjust.
 Identities = 1248/1268 (98%), Positives = 1248/1268 (98%), Gaps = 20/1268 (2%)

Query  1     MSTRRVTRSTFSLSQIGLADLETTPRPRKAAVAKRTATTKAGRSPLSRTRRELRSTSITS  60
             MSTRRVTRSTFSLSQIGLADLETTPRPRKAAVAKRTATTKAGRSPLSRTRRELRSTSITS
Sbjct  1     MSTRRVTRSTFSLSQIGLADLETTPRPRKAAVAKRTATTKAGRSPLSRTRRELRSTSITS  60

Query  61    QTGSFQPEETNDDREHRSEPGILDPPSVPVEQSTVYNIRSLSGQPSVLDSPSTNNTSSPN  120
             QTGSFQPEETNDDREHRSEPGILDPPSVPVEQSTVYNIRSLSGQPSVLDSPSTNNTSSPN
Sbjct  61    QTGSFQPEETNDDREHRSEPGILDPPSVPVEQSTVYNIRSLSGQPSVLDSPSTNNTSSPN  120

Query  121   SIRLNPSLTPPPSALTVPFVPPAVSRGEITTLVFASNQLLSLYIDSKNQGRPVDSHREAI  180
             SIRLNPSLTPPPSALTVPFVPPAVSRGEITTLVFASNQLLSLYIDSKNQGRPVDSHREAI
Sbjct  121   SIRLNPSLTPPPSALTVPFVPPAVSRGEITTLVFASNQLLSLYIDSKNQGRPVDSHREAI  180

Query  181   FWQLLQATRALFPSPSPSSFLLSAPSVDSSGQPIAPTSDPTELTAFRLSDYAVFLHAIAG  240
             FWQLLQATRALFPSPSPSSFLLSAPSVDSSGQPIAPTSDPTELTAFRLSDYAVFLHAIAG
Sbjct  181   FWQLLQATRALFPSPSPSSFLLSAPSVDSSGQPIAPTSDPTELTAFRLSDYAVFLHAIAG  240

Query  241   IRGTAASEAQLDELQESWRMFLQLFKGTSGAVGMGSAFRHDGLLTLLLDIGTQLYILKRP  300
             IRGTAASEAQLDELQESWRMFLQLFKGTSGAVGMGSAFRHDGLLTLLLDIGTQLYILKRP
Sbjct  241   IRGTAASEAQLDELQESWRMFLQLFKGTSGAVGMGSAFRHDGLLTLLLDIGTQLYILKRP  300

Query  301   ALSLLQQSQLLDELFSSSLVSRRTSQQGLRTKALKTLDDERWLTMSRARVAELNVLQSNS  360
             ALSLLQQSQLLDELFSSSLVSRRTSQQGLRTKALKTLDDERWLTMSRARVAELNVLQSNS
Sbjct  301   ALSLLQQSQLLDELFSSSLVSRRTSQQGLRTKALKTLDDERWLTMSRARVAELNVLQSNS  360

Query  361   EIRFPFSSFQREVINFVQSRQALLLDHSQPSIPDRLDLDNSQTMLQTARSDFRRRPRLSE  420
             EIRFPFSSFQREVINFVQSRQALLLDHSQPSIPDRLDLDNSQTMLQTARSDFRRRPRLSE
Sbjct  361   EIRFPFSSFQREVINFVQSRQALLLDHSQPSIPDRLDLDNSQTMLQTARSDFRRRPRLSE  420

Query  421   PIHHIRPYPQNPSVPISVRTSYSASLSSRYPNAPAPDSRRVSAPLPQREPLRTPVGLSRR  480
             PIHHIRPYPQNPSVPISVRTSYSASLSSRYPNAPAPDSRRVSAPLPQREPLRTPVGLSRR
Sbjct  421   PIHHIRPYPQNPSVPISVRTSYSASLSSRYPNAPAPDSRRVSAPLPQREPLRTPVGLSRR  480

Query  481   RGSYAHYPDDGVGDIPPIQSELRSRTKPRYSALSIPPAPSSPLMVEDRSPLEESRILVLS  540
             RGSYAHYPDDGVGDIPPIQSELRSRTKPRYSALSIPPAPSSPLMVEDRSPLEESRILVLS
Sbjct  481   RGSYAHYPDDGVGDIPPIQSELRSRTKPRYSALSIPPAPSSPLMVEDRSPLEESRILVLS  540

Query  541   ESPSNKGWEGNDTMDAEELEDLLGKNEEEEVMDEGRTLFANGDATSVQGSDEDELSTGKD  600
             ESPSNKGWEGNDTMDAEELEDLLGKNEEEEVMDEGRTLFANGDATSVQGSDEDELSTGKD
Sbjct  541   ESPSNKGWEGNDTMDAEELEDLLGKNEEEEVMDEGRTLFANGDATSVQGSDEDELSTGKD  600

Query  601   RAQSRLSSTSIKVEENELVDELDLRAIRRPREESSGSFLRESQINQVVPSDPLARDVASD  660
             RAQSRLSSTSIKVEENELVDELDLRAIRRPREESSGSFLRESQINQVVPSDPLARDVASD
Sbjct  601   RAQSRLSSTSIKVEENELVDELDLRAIRRPREESSGSFLRESQINQVVPSDPLARDVASD  660

Query  661   RLYINADEVLEATFALTREDVLFSQPQSQSQPQSQSNSQLQPLAQVSPTSSISAEFIASL  720
             RLYINADEVLEATFALTREDVLFSQPQSQSQPQSQSNSQLQPLAQVSPTSSISAEFIASL
Sbjct  661   RLYINADEVLEATFALTREDVLFSQPQSQSQPQSQSNSQLQPLAQVSPTSSISAEFIASL  720

Query  721   KARTAELKRSKAHRDHARSSSASTTHTVATVLAQVGVNPPSLLHDSEKAAAPLANDLNED  780
             KARTAELKRSKAHRDHARSSSASTTHTVATVLAQVGVNPPSLLHDSEKAAAPLANDLNED
Sbjct  721   KARTAELKRSKAHRDHARSSSASTTHTVATVLAQVGVNPPSLLHDSEKAAAPLANDLNED  780

Query  781   EETELAMELLKLRQLPALSTEKVNGNEVRKEDGDLLVIEKTPQPSTDTASHPMIFPSSPT  840
             EETELAMELLKLRQLPALSTEKVNGNEVRKEDGDLLVIEKTPQPSTDTASHPMIFPSSPT
Sbjct  781   EETELAMELLKLRQLPALSTEKVNGNEVRKEDGDLLVIEKTPQPSTDTASHPMIFPSSPT  840

Query  841   PVHITSVPSAHSLKETTKIPYRPPTLTPEPFGSEAQPVPGFQKDVTEQVAHPPISSFQNT  900
             PVHITSVPSAHSLKETTKIPYRPPTLTPEPFGSEAQPVPGFQKDVTEQVAHPPISSFQNT
Sbjct  841   PVHITSVPSAHSLKETTKIPYRPPTLTPEPFGSEAQPVPGFQKDVTEQVAHPPISSFQNT  900

Query  901   ALEQTETAPSSSLILPVSSQSTSLRRPFNKRKMFDTDSEESDSSIDDSFLRARTGRKPMS  960
             ALEQTETAPSSSLILPVSSQSTSLRRPFNKRKMFDTDSEESDSSIDDSFLRARTGRKPMS
Sbjct  901   ALEQTETAPSSSLILPVSSQSTSLRRPFNKRKMFDTDSEESDSSIDDSFLRARTGRKPMS  960

Query  961   AKILPPSWPSRVMSSVAPPDTSPYPSSSGQHMSPMPSDYRQTTPPSSPIHFRPTSSFSRK  1020
             AKILPPSWPSRVMSSVAPPDTSPYPSSSGQHMSPMPSDYRQTTPPSSPIHFRPTSSFSRK
Sbjct  961   AKILPPSWPSRVMSSVAPPDTSPYPSSSGQHMSPMPSDYRQTTPPSSPIHFRPTSSFSRK  1020

Query  1021  RQRQLSPPRMSSPPASVSLRCPSSSPPSTQPTSSKSQRRIVSSSSRSHTPALTSSYAGNE  1080
             RQRQLSPPRMSSPPASVSLRCPSSSPPSTQPTSSKSQRRIVSSSSRSHTPALTSSYAGNE
Sbjct  1021  RQRQLSPPRMSSPPASVSLRCPSSSPPSTQPTSSKSQRRIVSSSSRSHTPALTSSYAGNE  1080

Query  1081  NHRNGWSPVETRFFVRVAQEVGLDTSNCWTAILERHGAQGRYSRVLSKRSNVNLKDKMRL  1140
             NHRNGWSPVETRFFVRVAQEVGLDTSNCWTAILERHGAQGRYSR                
Sbjct  1081  NHRNGWSPVETRFFVRVAQEVGLDTSNCWTAILERHGAQGRYSR----------------  1124

Query  1141  ILLKDYPYPVKVPNGMVRYLPSGDSRKIAWLKGGRTHAETRKAEPDVEREVDMLCEQARR  1200
                 DYPYPVKVPNGMVRYLPSGDSRKIAWLKGGRTHAETRKAEPDVEREVDMLCEQARR
Sbjct  1125  ----DYPYPVKVPNGMVRYLPSGDSRKIAWLKGGRTHAETRKAEPDVEREVDMLCEQARR  1180

Query  1201  RRIELEGESEEEEDSEPESEPDLESEDGDEEEEQVEDEEEAEEEEEVLDVLTLGKAEESP  1260
             RRIELEGESEEEEDSEPESEPDLESEDGDEEEEQVEDEEEAEEEEEVLDVLTLGKAEESP
Sbjct  1181  RRIELEGESEEEEDSEPESEPDLESEDGDEEEEQVEDEEEAEEEEEVLDVLTLGKAEESP  1240

Query  1261  LFFQGSDD  1268
             LFFQGSDD
Sbjct  1241  LFFQGSDD  1248


>gb|KNZ59051.1| hypothetical protein VP01_180g6 [Puccinia sorghi]
Length=937

 Score = 53.1 bits (126),  Expect = 0.002, Method: Compositional matrix adjust.
 Identities = 29/85 (34%), Positives = 43/85 (51%), Gaps = 5/85 (6%)

Query  1083  RNGWSPVETRFFVRVAQEVGLDTSNCWTAILERHGAQGRYSRVLSKRSNVNLKDKMRLIL  1142
             R  WSP E +  ++   +     SNC   IL+RHG  G  S++ + R++V+LKDK   + 
Sbjct  801   RTFWSPDEEKLLIKEVVKWHA-KSNCMAEILKRHGPHGSLSKIFAHRNSVHLKDKAVNLS  859

Query  1143  LKDY----PYPVKVPNGMVRYLPSG  1163
              K Y      P KV +   R+ P G
Sbjct  860   TKWYRDEAKLPKKVRHAFSRFPPKG  884


>gb|EPE07756.1| myb dna binding protein [Ophiostoma piceae UAMH 11346]
Length=2097

 Score = 48.9 bits (115),  Expect = 0.050, Method: Compositional matrix adjust.
 Identities = 38/119 (32%), Positives = 57/119 (48%), Gaps = 19/119 (16%)

Query  1058  RRIVSSSSRSHTPALTSSYAGNENHRNGWSPVETRFFVRVAQEVGLDTSNC--WTAILER  1115
             R+  ++ +++HT        G  + R  WSP E +  +      GLD      W+ IL+ 
Sbjct  1253  RQAAATKTQTHT-----RREGLHSTRRPWSPDEEQALM-----TGLDMVKGPHWSQILQL  1302

Query  1116  HGAQGRYSRVLSKRSNVNLKDK---MRLILLK---DYPYPVKVPNGMVRY-LPSGDSRK  1167
              GA G  S +L  RS V LKDK   ++L  LK   + PY +K   G ++   PS  +RK
Sbjct  1303  FGANGTISDILKDRSQVQLKDKARNLKLFFLKANTEMPYYLKCVTGELKTRAPSQAARK  1361


>gb|KNF05609.1| hypothetical protein PSTG_01418 [Puccinia striiformis f. sp. 
tritici PST-78]
Length=1476

 Score = 47.8 bits (112),  Expect = 0.12, Method: Compositional matrix adjust.
 Identities = 21/68 (31%), Positives = 35/68 (51%), Gaps = 1/68 (1%)

Query  1070  PALTSSYAGNENHRNGWSPVETRFFVRVAQEVGLDTSNCWTAILERHGAQGRYSRVLSKR  1129
             P+ + S +     R  W+  E    ++  ++     +NC   I++RHG +G  SR  + R
Sbjct  1325  PSRSRSKSNQPGQRTFWTDAEENLLIKEVKKFS-KKNNCMAEIIKRHGPRGTISRTFAHR  1383

Query  1130  SNVNLKDK  1137
             + VNLKDK
Sbjct  1384  TGVNLKDK  1391


>gb|ENH88019.1| myb dna binding protein [Colletotrichum orbiculare MAFF 240422]
Length=1052

 Score = 44.7 bits (104),  Expect = 0.79, Method: Compositional matrix adjust.
 Identities = 34/108 (31%), Positives = 50/108 (46%), Gaps = 13/108 (12%)

Query  1078  GNENHRNGWSPVETRFFVRVAQEVGLDTSNC--WTAILERHGAQGRYSRVLSKRSNVNLK  1135
             G  + R  W+  E +  +      GLD      W+ IL   GA+G YS +L  R+ V LK
Sbjct  718   GTTSTRRPWTQEEEKALM-----TGLDLVRGPHWSQILTLFGAEGTYSTILKDRTQVQLK  772

Query  1136  DKMRLILLKDYPYPVKVPNGMVRYLP--SGDSRKIAWLKGGRTHAETR  1181
             DK R + L    + +K  + M  YL   +G+ +  A  +  R  AE R
Sbjct  773   DKARNLKL----FFLKTNSEMPYYLQAVTGELKTRAPTQAARKEAEER  816


>ref|XP_501039.1| YALI0B18018p [Yarrowia lipolytica]
 emb|CAG83292.1| YALI0B18018p [Yarrowia lipolytica CLIB122]
Length=710

 Score = 43.9 bits (102),  Expect = 1.3, Method: Compositional matrix adjust.
 Identities = 32/91 (35%), Positives = 43/91 (47%), Gaps = 8/91 (9%)

Query  1086  WSPVETRFFVRVAQEVGLDTSN--CWTAILERHGAQGRYSRVLSKRSNVNLKDKMRLILL  1143
             WS  E       A   GL T N   W+ ILE +G  G  S VL  R+ V LKDK R + L
Sbjct  444   WSKAE-----EAALMNGLRTVNGPYWSQILEIYGPGGTVSEVLKDRNQVQLKDKARNLKL  498

Query  1144  KDYPYPVKVPNGMVRYLPSGDSRKIAWLKGG  1174
                   VKVP+ + +++  G  +     +GG
Sbjct  499   YFLKAGVKVPDCL-QFVTGGIKKGTRGRRGG  528


Lambda      K        H        a         alpha
   0.310    0.126    0.353    0.792     4.96 

Gapped
Lambda      K        H        a         alpha    sigma
   0.267   0.0410    0.140     1.90     42.6     43.6 

Effective search space used: 15916252288524


  Database: nr
    Posted date:  Sep 23, 2015 12:05 AM
  Number of letters in database: 26,053,659,533
  Number of sequences in database:  71,551,133


Matrix: BLOSUM62
Gap Penalties: Existence: 11, Extension: 1
Neighboring words threshold: 11
Window for multiple hits: 40
```
