# Supplementary material for: Comparative genomics provides new insights into the diversity, physiology, and sexuality of the only industrially exploited tremellomycete: Phaffia rhodozyma
Source: BMC Genomics. 2016 Nov 9;17:901. doi: 10.1186/s12864-016-3244-7 (PMC5103461; doi:10.1186/s12864-016-3244-7)
Supplement: Additional file 6: — List of orphan genes with links to PFAM (related to Additional file 1: Table S1). (ZIP 1428 kb) [file 12864_2016_3244_MOESM6_ESM.zip › BLAST_HTML_FTR/G00960_P.html]

BLAST Search Results


```
BLASTP 2.2.27+


Reference:
Stephen F. Altschul, Thomas L. Madden, Alejandro A. Schäffer,
Jinghui Zhang, Zheng Zhang, Webb Miller, and David J. Lipman (1997),
"Gapped BLAST and PSI-BLAST: a new generation of protein database
search programs", Nucleic Acids Res. 25:3389-3402.


Reference for
composition-based statistics:
Alejandro A. Schäffer, L. Aravind, Thomas L. Madden, Sergei
Shavirin, John L. Spouge, Yuri I. Wolf, Eugene V. Koonin, and
Stephen F. Altschul (2001), "Improving the accuracy of PSI-BLAST
protein database searches with composition-based statistics and
other refinements", Nucleic Acids Res. 29:2994-3005.


Database: nr
           71,551,133 sequences; 26,053,659,533 total letters


Query= G00960_P

Length=86
                                                                      Score     E
Sequences producing significant alignments:                          (Bits)  Value

emb|CED84774.1|  hypothetical protein [Xanthophyllomyces dendrorh...   171    1e-52
gb|KGQ00836.1|  hypothetical protein PAAG_12501 [Paracoccidioides...  34.7    2.9  
ref|WP_022873740.1|  hypothetical protein [Nesterenkonia alba]        35.0    6.4  


 >emb|CED84774.1| hypothetical protein [Xanthophyllomyces dendrorhous]
Length=85

 Score =  171 bits (432),  Expect = 1e-52, Method: Compositional matrix adjust.
 Identities = 85/85 (100%), Positives = 85/85 (100%), Gaps = 0/85 (0%)

Query  1   MASRTASKAFDYIQRGFSVALVVLSISGATAGYMIHSERMSIAGEYEKRLAEYNVALANE  60
           MASRTASKAFDYIQRGFSVALVVLSISGATAGYMIHSERMSIAGEYEKRLAEYNVALANE
Sbjct  1   MASRTASKAFDYIQRGFSVALVVLSISGATAGYMIHSERMSIAGEYEKRLAEYNVALANE  60

Query  61  KAADSVASALPTTNAPTPITPSPSS  85
           KAADSVASALPTTNAPTPITPSPSS
Sbjct  61  KAADSVASALPTTNAPTPITPSPSS  85


>gb|KGQ00836.1| hypothetical protein PAAG_12501 [Paracoccidioides sp. 'lutzii' 
Pb01]
Length=118

 Score = 34.7 bits (78),  Expect = 2.9, Method: Compositional matrix adjust.
 Identities = 16/41 (39%), Positives = 21/41 (51%), Gaps = 0/41 (0%)

Query  39  RMSIAGEYEKRLAEYNVALANEKAADSVASALPTTNAPTPI  79
           +MSI GE E+ L  +N  L  E A   V    P  +AP P+
Sbjct  55  KMSITGELERTLPSFNSVLERELATKLVCCFGPVVSAPAPV  95


>ref|WP_022873740.1| hypothetical protein [Nesterenkonia alba]
Length=512

 Score = 35.0 bits (79),  Expect = 6.4, Method: Compositional matrix adjust.
 Identities = 27/85 (32%), Positives = 39/85 (46%), Gaps = 9/85 (11%)

Query  8    KAFDYIQRGFSVALVVLSISGATAGYMIHSERMSIAGEYEKRLAEYNVALANEKAAD---  64
            K  DYI+RG       L  +G T       +R +  GE+ +   E  V  A +K A+   
Sbjct  153  KLDDYIRRGVEHVAQSLEEAGVTERLQRAQQRTARTGEHMRTKIEKTVQAAQDKLAEENR  212

Query  65   ----SVASALPTTNAPTPITPSPSS  85
                  ASALPT   PT ++P+ +S
Sbjct  213  QRTAQQASALPT--VPTELSPAENS  235


Lambda      K        H        a         alpha
   0.318    0.127    0.346    0.792     4.96 

Gapped
Lambda      K        H        a         alpha    sigma
   0.267   0.0410    0.140     1.90     42.6     43.6 

Effective search space used: 637282103608


  Database: nr
    Posted date:  Sep 23, 2015 12:05 AM
  Number of letters in database: 26,053,659,533
  Number of sequences in database:  71,551,133


Matrix: BLOSUM62
Gap Penalties: Existence: 11, Extension: 1
Neighboring words threshold: 11
Window for multiple hits: 40
```
