# Supplementary material for: Comparative genomics provides new insights into the diversity, physiology, and sexuality of the only industrially exploited tremellomycete: Phaffia rhodozyma
Source: BMC Genomics. 2016 Nov 9;17:901. doi: 10.1186/s12864-016-3244-7 (PMC5103461; doi:10.1186/s12864-016-3244-7)
Supplement: Additional file 6: — List of orphan genes with links to PFAM (related to Additional file 1: Table S1). (ZIP 1428 kb) [file 12864_2016_3244_MOESM6_ESM.zip › BLAST_HTML_FTR/G04394_P.html]

BLAST Search Results


```
BLASTP 2.2.27+


Reference:
Stephen F. Altschul, Thomas L. Madden, Alejandro A. Schäffer,
Jinghui Zhang, Zheng Zhang, Webb Miller, and David J. Lipman (1997),
"Gapped BLAST and PSI-BLAST: a new generation of protein database
search programs", Nucleic Acids Res. 25:3389-3402.


Reference for
composition-based statistics:
Alejandro A. Schäffer, L. Aravind, Thomas L. Madden, Sergei
Shavirin, John L. Spouge, Yuri I. Wolf, Eugene V. Koonin, and
Stephen F. Altschul (2001), "Improving the accuracy of PSI-BLAST
protein database searches with composition-based statistics and
other refinements", Nucleic Acids Res. 29:2994-3005.


Database: nr
           71,551,133 sequences; 26,053,659,533 total letters


Query= G04394_P

Length=663
                                                                      Score     E
Sequences producing significant alignments:                          (Bits)  Value

emb|CED83407.1|  hypothetical protein [Xanthophyllomyces dendrorh...  1000    0.0  
gb|KLO20400.1|  hypothetical protein SCHPADRAFT_897694 [Schizopor...  90.1    2e-15
gb|KIP10560.1|  hypothetical protein PHLGIDRAFT_219210 [Phlebiops...  87.8    1e-14
ref|XP_007268518.1|  hypothetical protein FOMMEDRAFT_158372 [Fomi...  81.6    1e-12
ref|XP_007847837.1|  hypothetical protein Moror_9102 [Moniliophth...  73.6    3e-10
emb|CCO32579.1|  hypothetical protein BN14_06641 [Rhizoctonia sol...  59.7    6e-06
ref|XP_006460408.1|  hypothetical protein AGABI2DRAFT_117359 [Aga...  59.7    6e-06
gb|KNZ73659.1|  hypothetical protein J132_10460 [Termitomyces sp....  59.7    7e-06
emb|CEL62781.1|  hypothetical protein RSOLAG1IB_10473 [Rhizoctoni...  59.7    7e-06
gb|KDQ65136.1|  hypothetical protein JAAARDRAFT_188385 [Jaapia ar...  59.3    8e-06
ref|XP_002470398.1|  predicted protein [Postia placenta Mad-698-R...  57.0    5e-05
ref|XP_007327866.1|  hypothetical protein AGABI1DRAFT_105472 [Aga...  54.3    3e-04
gb|KIM83320.1|  hypothetical protein PILCRDRAFT_819569 [Piloderma...  53.9    4e-04
gb|KII96121.1|  hypothetical protein PLICRDRAFT_170702 [Plicaturo...  53.9    5e-04
gb|KIJ99457.1|  hypothetical protein K443DRAFT_679965 [Laccaria a...  52.8    0.001
ref|XP_007355941.1|  hypothetical protein AURDEDRAFT_154733 [Auri...  52.4    0.001
ref|XP_776116.1|  hypothetical protein CNBD1640 [Cryptococcus neo...  52.4    0.001
emb|CUA69007.1|  hypothetical protein RSOLAG22IIIB_08259 [Rhizoct...  52.4    0.001
gb|KIR89184.1|  hypothetical protein I308_00186 [Cryptococcus gat...  51.2    0.004
gb|EPS99705.1|  hypothetical protein FOMPIDRAFT_89881 [Fomitopsis...  50.8    0.004
gb|ELU37580.1|  hypothetical protein AG1IA_08390 [Rhizoctonia sol...  50.8    0.004
gb|EMD31767.1|  hypothetical protein CERSUDRAFT_119350 [Ceriporio...  49.7    0.009
gb|KIN98457.1|  hypothetical protein M404DRAFT_1005307 [Pisolithu...  48.9    0.014
gb|KIR68723.1|  hypothetical protein I314_01147 [Cryptococcus gat...  48.9    0.015
gb|KIR49684.1|  hypothetical protein I312_00773, partial [Cryptoc...  48.9    0.016
gb|EUC56753.1|  hypothetical protein RSOL_194520 [Rhizoctonia sol...  48.9    0.017
gb|KIR27314.1|  hypothetical protein I309_03781 [Cryptococcus gat...  48.9    0.017
gb|KGB75079.1|  hypothetical protein CNBG_0917 [Cryptococcus gatt...  48.9    0.018
gb|KIR57022.1|  hypothetical protein I315_00182 [Cryptococcus gat...  48.1    0.034
gb|KIR80452.1|  hypothetical protein I306_02429 [Cryptococcus gat...  47.8    0.037
ref|XP_003193823.1|  hypothetical protein CGB_D7700C [Cryptococcu...  47.8    0.038
gb|KIJ61031.1|  hypothetical protein HYDPIDRAFT_31729 [Hydnomerul...  47.4    0.050
gb|KEP49340.1|  S1/P1 nuclease [Rhizoctonia solani 123E]              47.4    0.056
gb|KIR36053.1|  hypothetical protein I352_00996 [Cryptococcus gat...  47.0    0.060
gb|KIK21937.1|  hypothetical protein PISMIDRAFT_532418 [Pisolithu...  46.6    0.082
emb|CUA76453.1|  putative WD repeat-containing protein alr2800 [N...  46.6    0.11 
ref|XP_008035934.1|  hypothetical protein TRAVEDRAFT_144804 [Tram...  45.8    0.13 
ref|XP_003036973.1|  hypothetical protein SCHCODRAFT_103389 [Schi...  45.4    0.17 
ref|XP_007311998.1|  hypothetical protein SERLADRAFT_431589 [Serp...  44.7    0.28 
gb|KIJ19748.1|  hypothetical protein PAXINDRAFT_166002 [Paxillus ...  44.7    0.31 
gb|KJA18214.1|  hypothetical protein HYPSUDRAFT_45548 [Hypholoma ...  41.6    2.8  


 >emb|CED83407.1| hypothetical protein [Xanthophyllomyces dendrorhous]
Length=659

 Score = 1000 bits (2585),  Expect = 0.0, Method: Compositional matrix adjust.
 Identities = 658/662 (99%), Positives = 658/662 (99%), Gaps = 3/662 (0%)

Query  1    MRFKLSADTPTGPLKAWVLVPESLLPSGTIDHLESYLVDLFPSLCRADDDRGSVTKPKLR  60
            MRFKLSADTPTGPLKAWVLVPESLLPSGTIDHLESYLVDLFPSLCRADDDRGSVTKPKLR
Sbjct  1    MRFKLSADTPTGPLKAWVLVPESLLPSGTIDHLESYLVDLFPSLCRADDDRGSVTKPKLR  60

Query  61   LSIDGFDLLADTSINVIAPDDVVRVTLASISQTALIASGTKRKALSASSPESSPVASKRA  120
            LSIDGFDLLADTSINVIAPDDVVRVTLASISQTALIASGTKRKALSASSPESSPVASKRA
Sbjct  61   LSIDGFDLLADTSINVIAPDDVVRVTLASISQTALIASGTKRKALSASSPESSPVASKRA  120

Query  121  RKSSPPSAGTTTKERESKKKARKNAKDARKEVKKAKKERQIKEEAERKVRKEHKRTIKKS  180
            RKSSPPSAGTTTKERESKKKARKNAKDARKEVKKAKKERQIKEEAERKVRKEHKRTIKKS
Sbjct  121  RKSSPPSAGTTTKERESKKKARKNAKDARKEVKKAKKERQIKEEAERKVRKEHKRTIKKS  180

Query  181  VPVESSESEDSSSSESSSSSSSSSSPSSSSSSAPSVQSSKKPQPAPKSFSNVTPASENLP  240
            VPVESSESEDSSSSESSSSSSSSSSPSSSSSSAPSVQSSKKPQPAPKSFSNVTPASENLP
Sbjct  181  VPVESSESEDSSSSESSSSSSSSSSPSSSSSSAPSVQSSKKPQPAPKSFSNVTPASENLP  240

Query  241  TRSSRSISSTIILTPIIPANPPVPPGAGSTATQERNIRRRRKKAALKAENAAAARSAAVT  300
            TRSSRSISSTIILTPIIPANPPVPPGAGSTATQERNIRRRRKKAALKAENAAAARSAAVT
Sbjct  241  TRSSRSISSTIILTPIIPANPPVPPGAGSTATQERNIRRRRKKAALKAENAAAARSAAVT  300

Query  301  VPKLPSLLPPPASPSPFVESISSSSSSSSSSSSSSSSSSSSSSSSSSPSSPRVAASTLLP  360
            VPKLPSLLPPPASPSPFVESISSSSSSSSSSSSSSSSSSSSSSS SSP   RVAASTLLP
Sbjct  301  VPKLPSLLPPPASPSPFVESISSSSSSSSSSSSSSSSSSSSSSSPSSP---RVAASTLLP  357

Query  361  TQRSLESVVPLSIAFNASSNRNKSSAYRKQAAGSAPTRISYNSVPPPSSLSASAVSATPT  420
            TQRSLESVVPLSIAFNASSNRNKSSAYRKQAAGSAPTRISYNSVPPPSSLSASAVSATPT
Sbjct  358  TQRSLESVVPLSIAFNASSNRNKSSAYRKQAAGSAPTRISYNSVPPPSSLSASAVSATPT  417

Query  421  RPEPFFAKLNSVTTPTNNNNHNKSFIRFDAPSDREVLPGNMWVSSVDVESDSWARVVGSG  480
            RPEPFFAKLNSVTTPTNNNNHNKSFIRFDAPSDREVLPGNMWVSSVDVESDSWARVVGSG
Sbjct  418  RPEPFFAKLNSVTTPTNNNNHNKSFIRFDAPSDREVLPGNMWVSSVDVESDSWARVVGSG  477

Query  481  AWASYFGEPEPEPEPEAEADSIPRSRTRTDTEVFLDYGEDEEEGVERVLAPSTSTSTLPS  540
            AWASYFGEPEPEPEPEAEADSIPRSRTRTDTEVFLDYGEDEEEGVERVLAPSTSTSTLPS
Sbjct  478  AWASYFGEPEPEPEPEAEADSIPRSRTRTDTEVFLDYGEDEEEGVERVLAPSTSTSTLPS  537

Query  541  ASSIVSEQTVGWESEWAALEDAWGSLDGESHIKSSGLKQGSVIGWNDVTLNPKTYTPELT  600
            ASSIVSEQTVGWESEWAALEDAWGSLDGESHIKSSGLKQGSVIGWNDVTLNPKTYTPELT
Sbjct  538  ASSIVSEQTVGWESEWAALEDAWGSLDGESHIKSSGLKQGSVIGWNDVTLNPKTYTPELT  597

Query  601  LLVALVRSVCTDSNSIRYILKPRPRLEHDYDTEESNNGVDEDEVLECPIAEIRALNGKVL  660
            LLVALVRSVCTDSNSIRYILKPRPRLEHDYDTEESNNGVDEDEVLECPIAEIRALNGKVL
Sbjct  598  LLVALVRSVCTDSNSIRYILKPRPRLEHDYDTEESNNGVDEDEVLECPIAEIRALNGKVL  657

Query  661  KF  662
            KF
Sbjct  658  KF  659


>gb|KLO20400.1| hypothetical protein SCHPADRAFT_897694 [Schizopora paradoxa]
Length=564

 Score = 90.1 bits (222),  Expect = 2e-15, Method: Compositional matrix adjust.
 Identities = 188/672 (28%), Positives = 277/672 (41%), Gaps = 131/672 (19%)

Query  1    MRFKLSADTPTGPLKAWVLVPESLLPSGTIDHLESYLVDLFPSLCRADDDRGSVTKPKLR  60
            MR +LS+  P  P++AW  V  +   S +I  L+  + +  PSL  AD D   +T     
Sbjct  1    MRVRLSSKPPLPPIRAWFSVTTT---SQSIAELKHAICEEIPSLRSADVDAEDLT-----  52

Query  61   LSIDGFDLLADTSINVIAPDDVVRVTLASISQTALIASGTKRKALSASSPESSPVASKRA  120
            LS+D FDLL D ++      DV+R          +  S TKR   S        V +   
Sbjct  53   LSVDDFDLLDDGNV------DVLR------ENDLVCISATKRATTSLKRKREDEVVA---  97

Query  121  RKSSPPSAGTTTKERESKKKARKNAKDARKEVKKAKKERQIKEEAERKVRKEHKRTIKKS  180
              SS    G     R SK +A+  + D+  E+    K R     A R +    K   KK 
Sbjct  98   --SSSLQNGV----RNSKNRAQ--SLDSEGEI--GTKGRH----ASRSISTASKPLQKKQ  143

Query  181  VPVESSESEDSSSSESSSSSSSSSSPSSSSSSAPSVQSSKKPQPAPKSFSNVTPASENLP  240
             P +SS S  SSS  SSSSSSSS S S S S + S  SS      P S + +T ++  LP
Sbjct  144  RPAKSSSSSASSSESSSSSSSSSDSDSDSDSDSDSSDSSSSATEIPSSVTKITKSA--LP  201

Query  241  TRSSRSISSTI-ILTPIIPANPP-VPPGAGSTATQERNIRRRRKKAALKAENAAAARSAA  298
                   +    IL+    +NP  VPPG G   TQ RN RRRRKK A   E  AA R+ A
Sbjct  202  QALQALEAQAASILSQ--QSNPLLVPPGEGKRTTQSRNERRRRKKIA---EREAAERTLA  256

Query  299  VTVPKLPSLLPPPASPSPFVESISSSSSSSSSSSSSSSSSSSSSSSSSSPSSPRVAASTL  358
                                   +++ S  +S+++        +S   +P+ P  A  +L
Sbjct  257  A----------------------ANALSDPNSTNAVPLGVRVQTSVEETPAEP--AQGSL  292

Query  359  LPTQRSLESVVPLSIAFNASSNRNKSSAYRKQAAGSAPTRISYNSVPPPSSLSASAVSAT  418
               Q + E  V  S+     SN+NK   ++K    + P RI ++         +  V++ 
Sbjct  293  SQIQTTTEQRVGTSL-----SNKNKKRGFKKAMENAIPERIVFSE--------SLEVTSE  339

Query  419  PTRPEPFFAKLNSVTTPTNNNNHNKSFIRFDAPSDRE---VLPGNMWVSSVDVESD---S  472
              R E           P  + N+         PS+++   +LP NM+V+SV++E+D   +
Sbjct  340  TVRHE---------IAPATSRNNKSQIPHLVTPSEKQSLGLLPNNMFVTSVELENDGQGN  390

Query  473  WARVVGSGAWASYFGEPEPEPEPEAEADSIPRSRTRTDTEVFLDYGEDEEEGVERVLAPS  532
            W R        SY G         A+A+          TE  LDYG  + E   R   P 
Sbjct  391  WDRKKKKKMRESYSGYSGQLQNGFADAEV---------TE--LDYGTPDVEEEARPKKPD  439

Query  533  TSTSTLPSASSIVSEQTVGWESEWAALEDAWGSLDGESHIKSSGLKQGSVIGWNDVTLNP  592
            T ++                  +W ++E  W  +      K   L+ G ++GW  + +NP
Sbjct  440  TGSAV-----------------DWNSIEQKWDQVPVIEDFKD--LRSGCLMGWKTLGINP  480

Query  593  KTYTPELTLLVALVRSVCTDSNSIRYILKPRPRLEHDYDTEESNNGVDED--EVLECPIA  650
             T +PE+ + +A V SV   + S   +L  RP  E D     +    DED  +V      
Sbjct  481  VTCSPEMLVQLARVLSVHQLTRSATVLLLKRPGAE-DIGFRGTRRAEDEDDQQVERDAGE  539

Query  651  EIRALNGKVLKF  662
            EI  L  +V  F
Sbjct  540  EIEDLVEEVFDF  551


>gb|KIP10560.1| hypothetical protein PHLGIDRAFT_219210 [Phlebiopsis gigantea 
11061_1 CR5-6]
Length=512

 Score = 87.8 bits (216),  Expect = 1e-14, Method: Compositional matrix adjust.
 Identities = 173/635 (27%), Positives = 260/635 (41%), Gaps = 160/635 (25%)

Query  1    MRFKLSADTPTGPLKAWVLVPESLLPSGTIDHLESYLVDLFPSLCRADDDRGSVTKPKLR  60
            MR K+    P  P K W +VP       T+  L+  L    P L ++  D        L 
Sbjct  1    MRIKVECSPPLPPSKVWFIVPV----VSTVAELKHALWAELPGLQQSSTD-------NLT  49

Query  61   LSIDGFDLLADTSINVIAPDDVVRVTLASISQTALIASGTKRKALSASSPESSPVASKRA  120
            L +DGF+L+  +SI+VI   ++V     ++ QT  + S  KRK    S+ E S  + KRA
Sbjct  50   LVLDGFELIDSSSIDVIRDGELV-----TVKQT--MTSTVKRK----STEEPSLPSPKRA  98

Query  121  RKSSPPSAGTTTKERESKKKARKNAKDARKEVKKAKKERQIKEEAERKVRKEHKRTIKKS  180
            R++  P    +   R +  +  +   D  K++   K   + K+ +               
Sbjct  99   RRTVDPVVNISA--RTASVQPPQKPADVNKQLGIPKTNARGKQSSSSSSSS---------  147

Query  181  VPVESSESEDSSSSESSSSSSSSSSPSSSSSSAPSVQSSKKPQPAPKSFSNVTPASENLP  240
                 S+S   SSS+S S SS+  S SS SSSAPS + SK   PA KS  N  P ++  P
Sbjct  148  ----DSDSSSDSSSDSDSDSSADDSSSSDSSSAPSERPSK---PATKSQMNGIP-TQREP  199

Query  241  TRSSRSISSTIILTPIIPANPPVPPGAGSTATQERNIRRRRKKAALKAENAAAARSAAVT  300
              S++ +++             VPPG G  ATQ RN RRRRKK   +             
Sbjct  200  AASAKPVAAQH-----------VPPGQGKAATQSRNARRRRKKMYER-------------  235

Query  301  VPKLPSLLPPPASPSPFVESISSSSSSSSSSSSSSSSSSSSSSSSSSPSSPRVAASTLLP  360
                   LP PA P                            S + +P  PR  A    P
Sbjct  236  -------LPLPAEP---------------------------LSVNETPLGPRQTARPESP  261

Query  361  TQR--SLESVVPLSIAFNASSNRNKSSAYRKQAAGSAPTRISYNSVPPPSSLSASAVSAT  418
              R  ++ +  P+ +   + SN+NK   +++  A S P +I +++        ASA    
Sbjct  262  QPRPATVANAAPV-VMMASLSNKNKRKGFKQAMANSLPPKIVFSN-------PASAEEIL  313

Query  419  PTRPEPFFAKLNSVTTPTNNNNHNKS-FIRFDAPSDREV---LPGNMWVSSVDVESDSWA  474
            P           S T+P        S F R  APS+++    LP NM+V+S+DVE     
Sbjct  314  PF----------STTSPGEIQAAAPSIFPRLVAPSEKQANGQLPPNMFVTSIDVEEGLRK  363

Query  475  RVVGSGAWASYFGEPEPEPEPEAEADSIPRSRTRTDTEVFLDYGE-DEEEGVERVLAPST  533
                +   A+          P A      R  T     + LDYGE D+    +R L+ S+
Sbjct  364  GKKKNKKKAT----------PAAV-----RHETAEVEYMVLDYGEPDDHIPAQRDLS-SS  407

Query  534  STSTLPSASSIVSEQTVGWESEWAALEDAWGSLDGESHIKS-SGLKQGSVIGWNDVTLNP  592
            +T+T                 +WA +E  W   DG + I   + +K G ++GW  + +NP
Sbjct  408  ATAT---------------HKDWAKVESGW---DGYAKIADVAQIKPGGIVGWKALGINP  449

Query  593  KTYTPELTLLVALVRSVCTDSNSIRYILKPRPRLE  627
             TYTPE  + V  V +   D   ++ + KP   LE
Sbjct  450  ATYTPEWLVNVGQVVT-AGDKVVVKPLHKPGDVLE  483


>ref|XP_007268518.1| hypothetical protein FOMMEDRAFT_158372 [Fomitiporia mediterranea 
MF3/22]
 gb|EJD01241.1| hypothetical protein FOMMEDRAFT_158372 [Fomitiporia mediterranea 
MF3/22]
Length=560

 Score = 81.6 bits (200),  Expect = 1e-12, Method: Compositional matrix adjust.
 Identities = 160/634 (25%), Positives = 265/634 (42%), Gaps = 172/634 (27%)

Query  1    MRFKLSADTPTGPLKAWVLVPESLLPSGTIDHL--ESYLVDLFPSLCRADDDRGSVTKPK  58
            MRF+L+   P   LKAW  +P+    + T+D L  ++ +    P L   D+D    +  +
Sbjct  1    MRFRLATRPPLRALKAWFDIPDD---NSTLDILGVKAQICSRVPQLL--DEDGSHFSASE  55

Query  59   LRLSIDGFDLLADTSINVIAPDDVVRVTLASISQTALIASGTKRKALSASSPESSPVASK  118
            + L ID F+LL D+S+ VI  +D+V +T                                
Sbjct  56   INLIIDDFELLDDSSLQVIKENDLVHIT--------------------------------  83

Query  119  RARKSSPPSAGTTTKERESKKKARKNAKDARKEVKKAKKERQIKEEAERKVRKEHKRTIK  178
                          K++ S +K RK   D             I    +R+V    KR  +
Sbjct  84   -------------KKQKVSIQKKRKAVDDT-----------TISSPKKRRVDLRAKRPSR  119

Query  179  KSVPVESSESEDSSSSESSSSSSSSSSPSSSSSSAPSVQSSKKPQPAPKSFSNVTPASEN  238
             S+P  SS S +SSS E++SS SSSSS SSS+SS+ S  S+     +  S   V  +  +
Sbjct  120  TSLPSISSSSSESSSDETTSSDSSSSSDSSSASSSSSSTSTSSSSASSSSSVEVQSSKRH  179

Query  239  LPTRSSRSISSTIILTPIIPANPPVPPGAGSTATQERNIRRRRKKAALKAENAAAARSAA  298
            +  + +R  +S+++          VPPG G  +TQ+RN RRR K+   + E+    ++AA
Sbjct  180  V-LQPARHTNSSVL---------SVPPGQGRPSTQKRNARRRLKR-IYQREHKQLEQAAA  228

Query  299  VTVPKLPSLLPPPASPSPFVESISSSSSSSSSSSSSSSSSSSSSSSSSSPSSPRVAASTL  358
              +P         A+  P       +  S S + + + ++  S ++S+SP    +   ++
Sbjct  229  QALPN------ESANAIPL-----GTHRSGSLNHTKNGNAEVSQNASTSP----LEVESM  273

Query  359  LPTQRSLESVVPLSIAFNASSNRNKSSAYRKQAAGSAPTRISYN--SVPPPSSL---SAS  413
            + T R                N+NK   +++  AG    +I ++  +VPP ++    SA 
Sbjct  274  VTTLR----------------NKNKKKGFKQAMAGLTSQKIVFSDETVPPNTATTFHSAE  317

Query  414  AVSATPTRPE-PFFAKLNSVTTPTNNNNHNKSFIRFDAPSDREVLPGNMWVSSVDVESDS  472
            + S+   RP  P F       TP+                 + +LP NM+V+SV+VE + 
Sbjct  318  STSSATARPSAPHF------LTPS-------------VKQAQGLLPSNMFVTSVEVEGEK  358

Query  473  WARVVGSGAWASYFGEPEPEPEPEAEADSIPRSRTRTDTE-VFLDYGEDEEEGVERVLAP  531
            W R         Y G+       +A A+ +    T T  E V L+YGE            
Sbjct  359  WDRGKKKKQRDQY-GQ-------DAAAEELHVDETATKQEDVTLNYGEP-----------  399

Query  532  STSTSTLPSASSIVSEQTVGWESEWAALEDAWGSLD-GESHIKSSGLKQGSVIGWNDVTL  590
                                 E  WA++E  W +L    S  + S L  G+V+G+  + +
Sbjct  400  ---------------------EINWASIEQTWDTLKPLSSLSEVSSLNAGTVVGFKALAI  438

Query  591  NPKTYTPELTLLVALVRSVCTDSNSIRYILKPRP  624
            NP T+TPE  + +  V+ +  D +S+   + PRP
Sbjct  439  NPATFTPETLVHLGRVKEIRHDDSSVVLEMIPRP  472


>ref|XP_007847837.1| hypothetical protein Moror_9102 [Moniliophthora roreri MCA 2997]
 gb|ESK92851.1| hypothetical protein Moror_9102 [Moniliophthora roreri MCA 2997]
Length=528

 Score = 73.6 bits (179),  Expect = 3e-10, Method: Compositional matrix adjust.
 Identities = 108/398 (27%), Positives = 167/398 (42%), Gaps = 82/398 (21%)

Query  263  VPPGAGSTATQERNIRRRRKKAALKAENAAAARSAAVTVPKLPSLLPPPASPSPFVESIS  322
            VPPG GST T +RN RRR KK   K E AA        VP  PS+LPP  + S  +  + 
Sbjct  188  VPPGHGSTQTHKRNQRRRLKK---KYEAAAKG-----LVP--PSVLPPKGTSSVNLAPLG  237

Query  323  SSSSSSSSSSSSSSSSSSSSSSSSSPSSPRVAASTLLPTQRSLESVVPLSIAFNASSNRN  382
                 +S+   S    S                      Q   +  + LS+ F+  +   
Sbjct  238  PKVIPTSAPIQSERQQSH--------------------IQHEPQDKLNLSM-FSLGNKNK  276

Query  383  KSSAYRKQAAGSAPTRISYNSVP--PPSSLSASAVSATPTR-PEPFFAKLNSVTTPTNNN  439
            K    +     SA  +I + +V   PP    ASA+  TPT  P+P               
Sbjct  277  KKGFKQASTLPSAQKKIIFETVASEPPLPF-ASALVPTPTHPPQP---------------  320

Query  440  NHNKSFIRFDAPSDRE---VLPGNMWVSSVDVESDSWARVVGSGAWASYFGEPEPEPEPE  496
            N +K   R  +PS+++   +LP NM+V+SVDVE DS         W  +F + +      
Sbjct  321  NGHKPLPRLISPSEKQEKGLLPSNMFVTSVDVEGDS--------PW-KHFKKTKARKNAL  371

Query  497  AEADSIPRSRTRTDTEVFLD--YGEDEEEGVERVLAPSTSTSTLPSASSIVSEQTVGWES  554
             + D  P S        +LD    + EEE  +  +AP     T+ + ++I  E       
Sbjct  372  VQVDENPLS--------WLDDITTQREEEVEDEPIAPECGEPTVVNETAI--EVDAKATL  421

Query  555  EWAALEDAWGSL----DGESHIKSSGLKQGSVIGWNDVTLNPKTYTPELTLLVALVRSVC  610
             W   E+ + +L      E    +  ++ G V+GW  + LNP T+ PE+ L +A V S  
Sbjct  422  IWTVAENTFDTLSIIQKAEGKALTDVIRPGVVVGWKALALNPVTFCPEIMLHLATVLSPS  481

Query  611  TD--SNSIRYILKPRPRLEHDYDTEESNNGVDEDEVLE  646
             D  + SIR ++  RP +E   + ++ +  V  + + E
Sbjct  482  PDLSTVSIRKMI--RPGVEEYAEDDDCDEIVATETIFE  517


>emb|CCO32579.1| hypothetical protein BN14_06641 [Rhizoctonia solani AG-1 IB]
Length=543

 Score = 59.7 bits (143),  Expect = 6e-06, Method: Compositional matrix adjust.
 Identities = 106/398 (27%), Positives = 160/398 (40%), Gaps = 126/398 (32%)

Query  221  KPQPAPKSFSNVTPASENLPTRSSRSISSTIILTPIIPANPPVPPGAGSTATQERNIRRR  280
            KPQP P+   +V   S+  P + +   SST   T    + PPVPPG G   T+ RN RRR
Sbjct  199  KPQPIPRKPLSVRQPSQ--PAQVATQPSSTRAST----SQPPVPPGEGKATTKSRNARRR  252

Query  281  --RKKAALKAENAAAARSAAVTVPKLPSLLPPPASPSPFVESISSSSSSSSSSSSSSSSS  338
              RK  A    N  A    A   P      P  A+P+P                      
Sbjct  253  ELRKHQA----NGTAFSQQATPTPD-----PKTATPTP----------------------  281

Query  339  SSSSSSSSSPSSPRVAASTLLPTQRSLESVVPLSIAFNASSNRNKSSAYRKQAAGSAPTR  398
                    +P+   + A+ + P+        P++ A +A  N+NK   + +  A S  TR
Sbjct  282  --------APTGDPMVATKIQPS-------APIAPAKHA--NKNKRKGFDRDMAESVATR  324

Query  399  ISYNSVPPPSSLSASAVSATPTRPEPFFAKLNSVTTPTNNNNHNKS-FIRFDA--PSDRE  455
            I Y +   P++   SA+S  P         L  V +P    + NKS +  +    PS+R+
Sbjct  325  IIYGT---PAT---SAISVEP---------LARVDSPAQTASQNKSRYTHYHVVPPSERK  369

Query  456  VLPGNMWVSSVDVESDSWARVVGSGAWASYFGEPEPEPEPEAEADSIPRSRTRTDTEVFL  515
             LP N+ V+SVDVE+      VG      +F                             
Sbjct  370  DLPSNVIVTSVDVEAVDGLEEVG-----EWF-----------------------------  395

Query  516  DYGEDEEEGVERVLAPSTSTSTL-PSASSIVSEQTVGW---ESEWAALEDAWGSLD-GES  570
                   +G +RV   ++  + L P+  S    +T+ W   +SEW  L D +  LD GE 
Sbjct  396  -------DGADRVTKEASPAAVLEPTLDS--KNKTIDWDIVDSEWERLWDTY--LDVGED  444

Query  571  HIKSSGLKQGSVIGWNDVTLNPKTYTPELTLLVALVRS  608
               +  LK G ++G+  +T++P T TP   + +A V S
Sbjct  445  AWNN--LKVGMLLGYKGLTIDPLTCTPGTKIHLARVTS  480


>ref|XP_006460408.1| hypothetical protein AGABI2DRAFT_117359 [Agaricus bisporus var. 
bisporus H97]
 gb|EKV48545.1| hypothetical protein AGABI2DRAFT_117359 [Agaricus bisporus var. 
bisporus H97]
Length=600

 Score = 59.7 bits (143),  Expect = 6e-06, Method: Compositional matrix adjust.
 Identities = 96/386 (25%), Positives = 150/386 (39%), Gaps = 68/386 (18%)

Query  258  PANPPVPPGAGSTATQERNIRRRRKKAALKAENAAAARSAAVTVPKLPSLLPPPASPSPF  317
            P  P VPPG G   T  RN+RR+R +AA    N      A V +    SL  PP   S  
Sbjct  218  PEPPFVPPGQGKPTTHARNLRRKRLRAAHSETN------ATVALGAASSLTSPPQGLSDI  271

Query  318  -VESISSSSSSSSSSSSSSSSSSSSSSSSSSPSSPR--------VAASTLLPTQRS----  364
                + S +   SS +      +       + + P         V  ++L    +     
Sbjct  272  NAMPLGSRTLPQSSKAKRKEKQTDRMRDDETVTRPETEVEDRREVMMASLGNKNKKKGYK  331

Query  365  --LESVVPLSIAFNASSNRNKSSAYRKQAAGSAPTRISYNSVP---PPSSLSASAVSATP  419
              L   VP  I F              +   + P++ S  + P   P S+L +  VS TP
Sbjct  332  KYLMGPVPQKIVFGG------------EGGSAQPSQTSVEASPSSRPASTLKS--VSHTP  377

Query  420  TRPEPFFAKLNSVTTPTNNNNHNKSFIRFDAPSDREVLPGNMWVSSVDVESDSWARVVGS  479
                        V  P+         I      DR  LP N++V+SVDVE+D    +VGS
Sbjct  378  V----------EVAPPSREQRPLPYLIPPSEIQDRGELPPNIFVTSVDVEAD----LVGS  423

Query  480  -GAWASYFGEPEPEPEPEAEADSIPRSRTRTDTEVFLDYGEDEEEGVERVLAPSTSTSTL  538
             G       +     +   +           D +V L YG  ++E         TS  TL
Sbjct  424  NGKKKKNRKKTTTAAQDVYDYTYANDEAYEADLDVTLSYGHGDDE---------TSAETL  474

Query  539  PSASSIVSEQTVGWESEWAALEDAWGSLDGESHIKSSGLKQGSVIGWNDVTLNPKTYTPE  598
             + S + S   +  + +W   E  + +    + +    LKQ  ++GW ++ LNP+T+TPE
Sbjct  475  -NQSQLTSNSKIS-DFDWDQAEQLFDNATKVTELDQ--LKQVRIVGWKNLELNPQTFTPE  530

Query  599  LTLLVALVRSV--CTDSNSIRYILKP  622
            + L+ + V  V   T S  ++ +L+P
Sbjct  531  ILLVASKVLEVSDLTLSVKVQRLLRP  556


>gb|KNZ73659.1| hypothetical protein J132_10460 [Termitomyces sp. J132]
Length=558

 Score = 59.7 bits (143),  Expect = 7e-06, Method: Compositional matrix adjust.
 Identities = 89/373 (24%), Positives = 142/373 (38%), Gaps = 61/373 (16%)

Query  263  VPPGAGSTATQERNIRRRRKKAALKAENAAAARSAAVTVPKLPSLLP-PPASPSPFVESI  321
            VPPG G   T  RN+RRR KK   KA  +A    A       P+L+P  P +       +
Sbjct  196  VPPGHGKPQTHSRNLRRRLKKKHDKAAQSADPAPAPPKNSSAPNLVPLGPNTSRMHARIV  255

Query  322  SSSSSSSSSSSSSSSSSSSSSSSSSSPSSPRVAASTLLPTQR----SLESVVPLSIAFNA  377
            + ++    SS +++     +S   +   +    A T  P QR    +  +V   SI  + 
Sbjct  256  ADTALPDISSRANTQKDDLASRPQTEVDADDRDAGTEPPAQRPQGITTMNVDGSSIMMST  315

Query  378  SSNRNKSSAYRKQAAGSAPTRISYNSVPPPSSLSASAVSATPTRPEPFFAKLNSVTTPTN  437
              N+NK   +++      P +I +            A+ A+    +   A+L      + 
Sbjct  316  LRNKNKRKGFKQSMNAPLPPKIVFQE---------EAMGASEQTAQKLMAELPGSEVASA  366

Query  438  NNNHNKSFIRFDAPSDREV---LPGNMWVSSVDVESDSWARVVGSGAWASYFGEPEPEPE  494
                     R   PS+++    LP  M+V+SVDVE   W                  E E
Sbjct  367  RP-------RLIPPSEKQERGELPPRMFVTSVDVEEGLWDSNQRKKKRKQKQKTDIQENE  419

Query  495  PEAEADSIPRSRTRTDTEVFLDYGEDEEEGVERVLAPSTSTSTLPSASSIVSEQTVGWES  554
             E   D+     T  + +V LDY                            S++ VG   
Sbjct  420  -ETYQDT-----TMLEEDVQLDY----------------------------SDEAVGSAL  445

Query  555  EWAALEDAWGSLDGESHIKSSGLKQGSVIGWNDVTLNPKTYTPELTLLVALVRSVCTDSN  614
            +W  +E  W      + I    L  G+V+GW  + LNP T++PE+ L +A V  +   S 
Sbjct  446  DWDRVEKGWDKFVALADI--GQLAVGTVVGWKGLALNPLTFSPEVMLSLASVTRLPEASA  503

Query  615  SIRYI-LKPRPRL  626
             ++ I +KP  R 
Sbjct  504  ELQIIVIKPFSRF  516


>emb|CEL62781.1| hypothetical protein RSOLAG1IB_10473 [Rhizoctonia solani AG-1 
IB]
Length=554

 Score = 59.7 bits (143),  Expect = 7e-06, Method: Compositional matrix adjust.
 Identities = 106/406 (26%), Positives = 158/406 (39%), Gaps = 142/406 (35%)

Query  221  KPQPAP-KSFSNVTPASENLPTRSSRSISSTIILTPIIPANPPVPPGAGSTATQERNIRR  279
            KPQP P K  S   P+    P + +   SST   T    + PPVPPG G   T+ RN RR
Sbjct  207  KPQPIPRKPLSARQPSQ---PAQVATQPSSTRAST----SQPPVPPGEGKATTKSRNARR  259

Query  280  R--RKKAALKAENAAAARSAAVTVPKLPSLLPPPASPSPFVESISSSSSSSSSSSSSSSS  337
            R  RK  A    N  A    A   P      P  A+P+P                     
Sbjct  260  RELRKHQA----NGTAFSQQATPTPD-----PKTATPTP---------------------  289

Query  338  SSSSSSSSSSPSSPRVAASTLLPTQRSLESVVPLSIAFNASSNRNKSSAYRKQAAGSAPT  397
                     +P+   + A+ + P+        P++ A +A  N+NK   + +  A S  T
Sbjct  290  ---------APTGDPMVATKIQPS-------APIAPAKHA--NKNKRKGFDRDMAESVAT  331

Query  398  RISYNSVPPPSSLSASAVSATPTRPEPFFAKLNSVTTPTNNNNHNKS-FIRFDA--PSDR  454
            RI Y + P PS++S           EP    L  V +P    + NK+ +  +    PS+R
Sbjct  332  RIIYGT-PAPSAISV----------EP----LTRVDSPAQTASQNKARYTHYHVVPPSER  376

Query  455  EVLPGNMWVSSVDVESDSWARVVGSGAWASYFGEPEPEPEPEAEADSIPRSRTRTDTEVF  514
            + LP N+ V+SVDVE+      VG      +F                            
Sbjct  377  KDLPSNVIVTSVDVEAVDGLEEVG-----EWF----------------------------  403

Query  515  LDYGEDEEEGVERVLAPSTSTSTL-PSASSIVSEQTVGW---ESEWAAL--------EDA  562
                    +G +RV   ++  + L P+  S    +T+ W   +SEW  L        EDA
Sbjct  404  --------DGADRVTKEASPAAVLGPTLDS--KNKTIDWDIVDSEWERLWDTYLDVREDA  453

Query  563  WGSLDGESHIKSSGLKQGSVIGWNDVTLNPKTYTPELTLLVALVRS  608
            W           + LK G+++G+  +T++P T TP   + +A V S
Sbjct  454  W-----------NNLKVGTLLGYKGLTIDPLTCTPGTKIHLARVTS  488


>gb|KDQ65136.1| hypothetical protein JAAARDRAFT_188385 [Jaapia argillacea MUCL 
33604]
Length=523

 Score = 59.3 bits (142),  Expect = 8e-06, Method: Compositional matrix adjust.
 Identities = 95/395 (24%), Positives = 156/395 (39%), Gaps = 95/395 (24%)

Query  234  PASENLPTRSSRSISSTIILTPIIPANPPVPPGAGSTATQERNIRRRRKKAALKAENAAA  293
            P  +++P R++    + I  T   P+ PPVPPG G   TQ RN RR+ K+   K   A+A
Sbjct  164  PPKQHVPPRTAPKPKAPI--TARTPSKPPVPPGHGKPQTQNRNHRRKLKRQHEKQAKASA  221

Query  294  --------ARSAAVTVPKLPSLLPPPASPSPFVESISSSSSSSSSSSSSSSSSSSSSSSS  345
                    A S A  +P  P L   P+   P  E +    + +    S+           
Sbjct  222  SGPLLPAHAISTANAIPLGPHL---PSQDQPMDEDVHVEVNETDRQPST-----------  267

Query  346  SSPSSPRVAASTLLPTQRSLESVVPLSIAFNASSNRNKSSAYRKQAAGSAPTRISYNSVP  405
                          P +R       + +A + +SN+NK   ++       P +I + +  
Sbjct  268  --------------PAERM------IMMALSLNSNKNKKRNFKASMNKPLPEKIIFGAS-  306

Query  406  PPSSLSASAVSATPTR--------PEPFFAKLNSVTTPTNNNNHNKSFIRFDAPSDREVL  457
              SS +  A ++TP+         P+P F   +    P +               +R  L
Sbjct  307  --SSSAPLADNSTPSEREWDKDKAPDPQFPHPHPRLVPPSEL------------QERGEL  352

Query  458  PGNMWVSSVDVESDSW-ARVVGSGAWASYFGEPEPEPEPEAEADSIPRSRTRTDTEVFLD  516
            P N++V+SVDVE      +  G G        P  E + E +             EV LD
Sbjct  353  PPNIFVTSVDVEEGMHGKKRKGKGKRRDNVASPPLEGQDERQG------------EVCLD  400

Query  517  YGEDEEEGVERVLAPSTSTSTLPSASSIVSEQTVGWESEWAALEDAWGSLDGESHIKSSG  576
            YG     GV+  ++ +TST+ L    +               ++  W +L   + +    
Sbjct  401  YG--TANGVDEDVS-TTSTNFLKDDGT----------PNIDLVQHKWDTLTVITQLDQ--  445

Query  577  LKQGSVIGWNDVTLNPKTYTPELTLLVALVRSVCT  611
            LK G +IGW  + +NP TY+PE+ L +A + ++ T
Sbjct  446  LKPGILIGWKALGVNPATYSPEILLHLARIIAINT  480


>ref|XP_002470398.1| predicted protein [Postia placenta Mad-698-R]
 gb|EED84423.1| predicted protein [Postia placenta Mad-698-R]
Length=870

 Score = 57.0 bits (136),  Expect = 5e-05, Method: Compositional matrix adjust.
 Identities = 92/389 (24%), Positives = 163/389 (42%), Gaps = 83/389 (21%)

Query  279  RRRKKAAL--KAE-NAAAARSAAV-TVPKLPSLLPPPASPSP--------FVESISSSSS  326
            R    AAL  KAE  A AARS +V T+P +P  L  P++ S           E +++++ 
Sbjct  195  RHHAVAALPPKAELTANAARSKSVPTLPHVPPGLGKPSTQSRNIRRRRKRLYERLAATAE  254

Query  327  SSSSSSSSSSSSS-SSSSSSSSPSSPRVAASTLLPTQRSLESVVPLSIAFNASSNRNKSS  385
             +S ++    + + ++ + SS PS P        P Q   +      +   +  N+NK  
Sbjct  255  PASVNAIPLGTRAPATGAGSSVPSGPSAQ-----PAQNEAQEAPRQPVLMASLQNKNKKK  309

Query  386  AYRKQAAGSAPTRISYNSVPPPSSLSASAVSATPTRPEPFFAKLNSVTTPTNNNNHNKSF  445
             +RK  + + P +I +  +   +++   A+        PF          TN  + ++  
Sbjct  310  GFRKSMSAAVPAKIVFTDLEDETAVVDEAMQEA----MPF----------TNTADRDRGV  355

Query  446  IRFDA----PSDRE---VLPGNMWVSSVDVESDSWARVVGSGAWASYFGEPEPEPEPEAE  498
               +A    PS+++   +LP NM+V+SVDVE   +++           G+ +   E    
Sbjct  356  FMVNARLVPPSEKQERGLLPLNMFVTSVDVEEGLYSKK----------GKKKRNQEQRRP  405

Query  499  ADSIPRSRTRTDTEVFLDYGEDEE----EGVERVLAPSTSTSTLPSASSIVSEQTVGWES  554
            A+ +P           L Y + EE     G+E   A S     +P   SI        E 
Sbjct  406  AE-VPDVGMNAGGNFELPYDDPEELVESNGLEEHSARS-GERPMPVRGSI--------EK  455

Query  555  EWAALEDAWGSLDGESHIKSSGLKQGSVIGWNDVTLNPKTYTPELTLLVALVRSVCTDSN  614
             WA+L     +   +          G+++GW ++ +NP+TYTPE+ L +   R V  D  
Sbjct  456  TWASLAKITAAFQSQV---------GAIVGWKELGINPRTYTPEVLLNIG--RVVKNDEQ  504

Query  615  SIRYILKPRPRLEHDYDTEESNNGVDEDE  643
             +         LE  ++ +E+  G+ ED+
Sbjct  505  LV---------LEPVFEEKETECGLFEDQ  524


>ref|XP_007327866.1| hypothetical protein AGABI1DRAFT_105472 [Agaricus bisporus var. 
burnettii JB137-S8]
 gb|EKM82142.1| hypothetical protein AGABI1DRAFT_105472 [Agaricus bisporus var. 
burnettii JB137-S8]
Length=603

 Score = 54.3 bits (129),  Expect = 3e-04, Method: Compositional matrix adjust.
 Identities = 93/374 (25%), Positives = 151/374 (40%), Gaps = 44/374 (12%)

Query  258  PANPPVPPGAGSTATQERNIRRRRKKAALKAENAAAARSAAVTVPKLPSLLPPPASPSPF  317
            P  P VPPG G   T  RN+RR+R +AA    NA  A  AA       SL  PP   S +
Sbjct  218  PEPPFVPPGQGKPTTHARNLRRKRLRAAHSETNATVALGAAS------SLTSPPQGLS-Y  270

Query  318  VESISSSSSSSSSSSSSSSSSSSSSSSSSSPSSPRVAASTLLPTQRSLESVVPLSIAFNA  377
            + ++   S +   SS +      +       +  R    T +  +R         +   +
Sbjct  271  INAMPLGSRTLPQSSKAKRKEKQTDRMRDDETETR--PETEVEDRRE--------VMMAS  320

Query  378  SSNRNKSSAYRKQAAGSAPTRISYNSVPPPSSLSASAVSATPT-RPEPFFAKLNS----V  432
              N+NK   Y+K   G  P +I +      +  S ++V A+P+ RP      ++     V
Sbjct  321  LGNKNKKKGYKKYLMGPVPQKIVFGGEKGSAQPSQTSVEASPSSRPASTLKSVSHTPVEV  380

Query  433  TTPTNNNNHNKSFIRFDAPSDREVLPGNMWVSSVDVESDSWARVVGSGAWASYFGEPEPE  492
              P+         I      DR  LP N++V+SVDVE+D    V  +G       +    
Sbjct  381  APPSREQRPLPYLIPPSEIQDRGELPPNIFVTSVDVEADL---VSSNGKKKKNRKKATST  437

Query  493  PEPEAEADSIPRSRTRTDTEVFLDYGE--DEEEGVERVLAPSTSTSTLPSASSIVSEQTV  550
             +   +           D +V L YG    E             TST  S  S ++    
Sbjct  438  AQDVYDYTYANDEAHEADLDVTLSYGHGNVE-------------TSTETSHQSQLTSNPK  484

Query  551  GWESEWAALEDAWGSLDGESHIKSSGLKQGSVIGWNDVTLNPKTYTPELTLLVALVRSV-  609
              + +W   E  + +    + +    L+Q  ++GW ++ LNP+T+TPE+ L+ + V  V 
Sbjct  485  ISDFDWDQAEQLFDNATKVTELDQ--LQQVRIVGWKNLELNPQTFTPEILLVASKVLEVS  542

Query  610  -CTDSNSIRYILKP  622
              T S  ++ +L+P
Sbjct  543  DLTSSVKVQRLLRP  556


>gb|KIM83320.1| hypothetical protein PILCRDRAFT_819569 [Piloderma croceum F 1598]
Length=581

 Score = 53.9 bits (128),  Expect = 4e-04, Method: Compositional matrix adjust.
 Identities = 45/148 (30%), Positives = 73/148 (49%), Gaps = 26/148 (18%)

Query  1    MRFKLSADTPTGPLKAWVLVPES----LLPSGTIDHLESYLVDLFPSLCRADDDRGSVTK  56
            MR KL  + P  PLKAW  +P+S    LL   TI+ L+ +L    P L ++      V  
Sbjct  1    MRLKLQTNPPLSPLKAWFPLPKSSPSTLL---TINELKHHLCTHLPVLTKS-----CVHA  52

Query  57   PKLRLSIDGFDLLADTSINVIAPDDVVRVTLASISQTALIASGTKRKALSASSPESSPVA  116
              + L +D F+LL +T + V+   D++ + L     +A      KRKAL   + E+    
Sbjct  53   KDIVLVLDDFELLDETEVGVLRDGDLICIKLDGSRSSA------KRKALDEDTNET----  102

Query  117  SKRARKSS----PPSAGTTTKERESKKK  140
             K+ R+SS    P  +G+ TK  + +++
Sbjct  103  HKKLRQSSIAPLPRVSGSNTKTLKRRRE  130


>gb|KII96121.1| hypothetical protein PLICRDRAFT_170702 [Plicaturopsis crispa 
FD-325 SS-3]
Length=526

 Score = 53.9 bits (128),  Expect = 5e-04, Method: Compositional matrix adjust.
 Identities = 70/291 (24%), Positives = 121/291 (42%), Gaps = 36/291 (12%)

Query  339  SSSSSSSSSPSSPRVAASTLLPTQRSLESVVPLSIAFNASSNRNKSSAYRKQAAGSAPTR  398
            + SSS+++ P  P    S   PT    +      I   +  N+NK   Y+   + + P +
Sbjct  230  AGSSSTNAIPLGPGSTHSDPEPTPVPEKDAADERITMASLRNKNKKRGYKNAMSNALPQK  289

Query  399  ISYNSVPPPSSLSASAVSATPTRPEPFFAKLNSVTTPTNNNNHNKSFIRFDAPSDRE---  455
            I +         S+  V   P R  P  A+ ++       +     + R   PS+++   
Sbjct  290  IVF---------SSDHVEPAPARDVPHSAEADAHLE--QAHRATADYPRLIPPSEKQDAG  338

Query  456  VLPGNMWVSSVDVESDSWARVVGSGAWASYFGEPEPEPEPEAEADSIPRSRTRTDTEVFL  515
             LP N++V+S+DVE     +       AS         E EA               ++L
Sbjct  339  QLPPNLFVTSIDVEEGMHGKKKKKKKRASQLASEPVYAEDEA---------------LYL  383

Query  516  DYGEDEEEGVERVLAPS-TSTSTLPSASSIVSEQTVGWESEWAALEDAWGSLDGESHIKS  574
            +YG  E+   ++V+A    +T   PS    +     G    +  +E  W S    + I  
Sbjct  384  EYGLHED--ADQVMASGDVTTQRNPSGQLPLESLADGSLPNFDVVETRWESYINITDI--  439

Query  575  SGLKQGSVIGWNDVTLNPKTYTPELTLLVALVRSVCTDSNSIRYILKPRPR  625
            S L+   ++GW  + +NP+T+TPE+ L +A V SV  D+ S   +++P  R
Sbjct  440  SQLRPRGLVGWKALAINPRTFTPEMLLNLAHVVSV--DAASGDVVVQPYAR  488


>gb|KIJ99457.1| hypothetical protein K443DRAFT_679965 [Laccaria amethystina LaAM-08-1]
Length=627

 Score = 52.8 bits (125),  Expect = 0.001, Method: Compositional matrix adjust.
 Identities = 105/454 (23%), Positives = 167/454 (37%), Gaps = 90/454 (20%)

Query  214  PSVQSSKKPQPAPK---SFSNVTPASENL-PTRSSRSISSTIILTPIIPANPPVPPGAGS  269
            P  Q+SK PQ +PK      N+T  + N+ P     S+  +  +          PPG GS
Sbjct  177  PVPQTSKVPQTSPKVPAKLPNITLGALNVKPKLPDASVGESFKVQH-------TPPGHGS  229

Query  270  TATQERNIRRRRKKAALK---------AENAAAARSAAVTVPKLPSLLPPPASPSPFVES  320
            + T  RN+RRR K+ A +         +E  A          +  SL PPP S SPF+  
Sbjct  230  SETHARNLRRRYKRQAEREQVQQPKTLSEPPAPPPPTLQQQQRPMSLQPPPISQSPFL--  287

Query  321  ISSSSSSSSSSSSSSSSSSSSSSSSSSPSSPRVAASTLLPTQRSLESVVP---LSIAFNA  377
                 SSSS      +    SS+S+  P  P+   ST        + V P   ++ A N 
Sbjct  288  --PEHSSSSPVPPPPAPPKRSSASNLVPLGPKKIPST------RRDRVGPANDVASALNN  339

Query  378  SSNRNKSSAYRKQAAGSAPTRISYNSVPPPSSLSASAVS-----------ATPTRPEPFF  426
            +   N ++             +  +  P P  +  S  +           A P   +  F
Sbjct  340  AFGSNATTGELNPGIDLDTDVMDVDDEPKPQLMMLSLKNKNKKKGFKQSMAAPIPKKIVF  399

Query  427  AKLNSVTTPTNNNN-HNKSFIRFDAPSD---REVLPGNMWVSSVDVESDSWARV------  476
                 V     +     + ++R   PS+   R  LP NM+V+ VDVE   W  V      
Sbjct  400  GDTEDVQQIVVDVVPSQQEYVRLIPPSEIQGRGELPPNMFVTCVDVERGIWDVVKEHRKP  459

Query  477  -------VGSGAWASYFGEPEPEPEPEAEADSIPRSRTRTDTEVFLDYGEDEEEGVERVL  529
                        WA  + E   EP+ + +                L +    +E  ++  
Sbjct  460  KNSKKKKNKEATWADLWFE---EPQVQGKVSK-------------LVFAGPRQEVTDKWK  503

Query  530  APSTSTSTLPSASSIVSEQTVGWESEWAALEDAWGSLDGESHIKS-SGLKQGSVIGWNDV  588
                    + S S+           +W   E  W   +    +K+   L  GS++GW ++
Sbjct  504  EEEVVEEAVVSGSTAF---------DWVLAEKVW---ETSFEVKTPDQLAVGSLVGWKEL  551

Query  589  TLNPKTYTPELTLLVALVRSVCTDSNSIRYILKP  622
             LN +T +PE  L VA +  +     +IR +L+P
Sbjct  552  GLNLQTVSPENLLFVAKITQIEGTQYTIRQLLRP  585


>ref|XP_007355941.1| hypothetical protein AURDEDRAFT_154733 [Auricularia delicata 
TFB-10046 SS5]
 gb|EJD35966.1| hypothetical protein AURDEDRAFT_154733 [Auricularia delicata 
TFB-10046 SS5]
Length=475

 Score = 52.4 bits (124),  Expect = 0.001, Method: Compositional matrix adjust.
 Identities = 38/115 (33%), Positives = 59/115 (51%), Gaps = 12/115 (10%)

Query  1    MRFKLSADTPTGPLKAWVLVPESLLPSGTIDHLESYLVDLFPSLCRADDDRGSVTKP-KL  59
            MRF+L    P  P KAW +     LP+   D   S ++D+  +LC    +  S + P +L
Sbjct  1    MRFRLHTVPPLPPYKAWYV-----LPAAVTDDASSTVLDIKRALC---SNVLSDSSPDQL  52

Query  60   RLSIDGFDLLADTSINVIAPDDVVRVTLASISQTALIASGTKRKALSASSPESSP  114
             L ID F++L  TS+ V+ P D++     ++S   +   GTKRKA +    + SP
Sbjct  53   ALFIDDFEILDGTSVEVVQPGDLITAKQRAVSAAGV---GTKRKARTDEDIQESP  104


>ref|XP_776116.1| hypothetical protein CNBD1640 [Cryptococcus neoformans var. neoformans 
B-3501A]
 gb|EAL21469.1| hypothetical protein CNBD1640 [Cryptococcus neoformans var. neoformans 
B-3501A]
Length=700

 Score = 52.4 bits (124),  Expect = 0.001, Method: Compositional matrix adjust.
 Identities = 44/135 (33%), Positives = 65/135 (48%), Gaps = 24/135 (18%)

Query  1    MRFKLSADTPTGPLKAWVLVPESLLPSGTIDHLESYLVDLFPSLCRADDDRGSVTKPKLR  60
            MR KLS   P  P +   LVP  +    TI HL+ YL+    S+ +      + +  +L 
Sbjct  1    MRIKLSLLPPFSPSRILFLVPSDV---KTIAHLKKYLIKSLSSIAQH-----ASSSRELL  52

Query  61   LSIDGFDLLADTSINVIAPDDVVRVTLASISQTALIASGTKRKALSASSPESSPVASKRA  120
            L I+GF LL+ + +N+I P DVV V LA  S              SA SP+    + K+ 
Sbjct  53   LEIEGFQLLSGSDLNIIEPTDVVCVRLAPGS--------------SADSPDK--FSDKKR  96

Query  121  RKSSPPSAGTTTKER  135
            +++S P     TK+R
Sbjct  97   KRNSAPKLPKQTKKR  111


>emb|CUA69007.1| hypothetical protein RSOLAG22IIIB_08259 [Rhizoctonia solani]
Length=541

 Score = 52.4 bits (124),  Expect = 0.001, Method: Compositional matrix adjust.
 Identities = 91/376 (24%), Positives = 141/376 (38%), Gaps = 114/376 (30%)

Query  256  IIPANPPVPPGAGSTATQERNIRRRRKKAALKAENAAAARSAAVTVPKLPSLLPPPAS--  313
            + P+ PPVPPG G ++T+ RN RRR  +  + A  A +           P L P PAS  
Sbjct  227  VTPSQPPVPPGQGKSSTKNRNARRRALRKHVAAGTAFS-----------PQLTPTPASAT  275

Query  314  --PSPFVESISSSSSSSSSSSSSSSSSSSSSSSSSSPSSPRVAASTLLPTQRSLESVVPL  371
              P+P  ES+ ++                           +V AST              
Sbjct  276  ATPAPDFESLVAT---------------------------QVKAST--------------  294

Query  372  SIAFNASSNRNKSSAYRKQAAGSAPTRISYNSVPPPSSLSASAVSATPTRPEPFFAKLNS  431
             IA   S+N+NK   + K  A +  TRI+Y + P P+ +SA+    +P    P F     
Sbjct  295  PIALAKSANKNKRKGFDKDMADAVATRITYGT-PAPAPISAAH---SPRTDSPAF-----  345

Query  432  VTTPTNNNNHNKSFIRFDAPSDREVLPGNMWVSSVDVESDSWARVVGSGAWASYFGEPEP  491
               P        +      PS  + LP N+ VSSVDVE+      VG   W  + GE   
Sbjct  346  ---PIATTKSRYTHYHVVPPSQLKDLPSNVIVSSVDVEAADGLEDVGE--W--FDGE---  395

Query  492  EPEPEAEADSIPRSRTRTDTEVFLDYGEDEEEGVERVLAPSTSTSTLPSASSIVSEQTVG  551
                                    ++  +E+     V +P  S +           + + 
Sbjct  396  ------------------------NHAAEEDLTAANVNSPPASNN---------KSKKID  422

Query  552  WE---SEWAALEDAWGSLDGESHIKSSGLKQGSVIGWNDVTLNPKTYTPELTLLVALVRS  608
            WE   S+W   E +WGS       +   L+ G+++ +  + ++P T TP   + +A V S
Sbjct  423  WEVVDSQW---EQSWGSFPTIDQARWDKLQPGTLLAYLGLAIDPVTCTPGTKVHLARVVS  479

Query  609  VCTDSNSIRYILKPRP  624
              +D          RP
Sbjct  480  GPSDGGQAECFFIERP  495


>gb|KIR89184.1| hypothetical protein I308_00186 [Cryptococcus gattii IND107]
Length=622

 Score = 51.2 bits (121),  Expect = 0.004, Method: Compositional matrix adjust.
 Identities = 32/88 (36%), Positives = 49/88 (56%), Gaps = 8/88 (9%)

Query  1   MRFKLSADTPTGPLKAWVLVPESLLPSGTIDHLESYLVDLFPSLCRADDDRGSVTKPKLR  60
           MR KLS   P  P +  +LVP+ +    TI HL+ YL+    S+ +      + +  +L 
Sbjct  1   MRIKLSLLPPFPPSRILLLVPDDV---KTITHLKKYLIKSLSSVAQH-----ASSSQELL  52

Query  61  LSIDGFDLLADTSINVIAPDDVVRVTLA  88
           L I+GF LL+ + +N+I P DVV V +A
Sbjct  53  LEIEGFQLLSGSDLNIIEPTDVVCVRVA  80


>gb|EPS99705.1| hypothetical protein FOMPIDRAFT_89881 [Fomitopsis pinicola FP-58527 
SS1]
Length=557

 Score = 50.8 bits (120),  Expect = 0.004, Method: Compositional matrix adjust.
 Identities = 47/178 (26%), Positives = 76/178 (43%), Gaps = 56/178 (31%)

Query  444  SFIRFDAPSDRE---VLPGNMWVSSVDVESDSWARVVGSGAWASYFGEPEPEPEPEAEAD  500
            +F R   PS+++   ++P NM+V+SVDVE+D                             
Sbjct  368  TFARLVPPSEKQEKGLVPPNMFVTSVDVEAD-----------------------------  398

Query  501  SIPRSRTRTDTEVFLDYGEDEE-------EGVERVLAP-----STSTSTLPSASSIVSEQ  548
             +P  R +   +    + E+EE       E VE  LA      +   S +   +  V+ Q
Sbjct  399  -LPNKRKKKQRQAPEAHYEEEEVIELPYDEPVEAALAGDPVAVTNGVSGMAKTAPAVNRQ  457

Query  549  TVGWESEWAALEDAWGSLDGESHIKSSGLKQGSVIGWNDVTLNPKTYTPELTLLVALV  606
             V  ES W +L             +++ LK G +IGW ++ +NP+T+TPE+ L V  V
Sbjct  458  DV--ESRWTSLPKI---------TEAAQLKPGVIIGWKELGINPRTFTPEMLLNVGKV  504


>gb|ELU37580.1| hypothetical protein AG1IA_08390 [Rhizoctonia solani AG-1 IA]
Length=711

 Score = 50.8 bits (120),  Expect = 0.004, Method: Compositional matrix adjust.
 Identities = 69/269 (26%), Positives = 104/269 (39%), Gaps = 81/269 (30%)

Query  221  KPQPAPK----SFSNVTPASENLPTRSSRSISSTIILTPIIPANPPVPPGAGSTATQERN  276
            KPQP P+    S  ++ P     P+  +R+           P+  PVPPG G T T+ RN
Sbjct  483  KPQPIPRQPLVSRQSLVPTQHVAPSSYTRAT----------PSQLPVPPGEGKTTTKSRN  532

Query  277  IRRRRKKAALKAENAAAARSAAVTVPKLPSLLPPPASPSPFVESISSSSSSSSSSSSSSS  336
             RRR  +    A  A + R+         +  P PA      +SI+++ +  ++      
Sbjct  533  ARRRALRKHQAAGTAFSPRTTPAPTSTTATFTPAPAG-----DSITATKTQVNA------  581

Query  337  SSSSSSSSSSSPSSPRVAASTLLPTQRSLESVVPLSIAFNASSNRNKSSAYRKQAAGSAP  396
                                             P++ A +A  N+NK   + +  A S  
Sbjct  582  ---------------------------------PIAPAKHA--NKNKRKGFDRDMAESVA  606

Query  397  TRISYNSVPPPSSLSASAVSATPTRPEPFFAKLNSVTTPTNNNNHNKS-FIRFDA--PSD  453
            TRI Y +  P         SA+  RP P       V TP  +    KS +  +    PS+
Sbjct  607  TRIIYGTPAP---------SASVERPAP-------VDTPAESTVQTKSRYTHYHVVPPSE  650

Query  454  REVLPGNMWVSSVDVESDSWARVVGSGAW  482
            R+ LP N+ V+SVDVE+       G G W
Sbjct  651  RKDLPANVIVTSVDVEAVDGLE--GVGEW  677


>gb|EMD31767.1| hypothetical protein CERSUDRAFT_119350 [Ceriporiopsis subvermispora 
B]
Length=534

 Score = 49.7 bits (117),  Expect = 0.009, Method: Compositional matrix adjust.
 Identities = 84/358 (23%), Positives = 135/358 (38%), Gaps = 110/358 (31%)

Query  261  PPVPPGAGSTATQERNIRRRRKKAALKAENAAAARSAAVTVPKLPSLLPPPASPSPFVES  320
            PPVPPG G  AT+ RN+RRRRKK     E  AA                 PAS    V +
Sbjct  237  PPVPPGFGKPATKSRNLRRRRKKI---HERLAATEE--------------PAS----VNA  275

Query  321  ISSSSSSSSSSSSSSSSSSSSSSSSSSPSSPRVAASTLLPTQRSLESVVPLSIAFNASSN  380
            I   +      + +  + + +++ + S ++P    ++L                    SN
Sbjct  276  IPLGTREPVDEAPAPPAPAEAATPAESLATPTFMMASL--------------------SN  315

Query  381  RNKSSAYRKQAAGSAPTRISYNSVPPPSSLSASAVSATP------TRPEPFFAKLNSVTT  434
            +NK   +++  A   P RI +               ATP          P+     SV  
Sbjct  316  KNKRKGFKRAMASHIPKRIVFE---------GQEDEATPGDDSEMQETLPYGGADESVVV  366

Query  435  PTNNNNHNKSFIRFDAPSDRE---VLPGNMWVSSVDVESDSWARVVGSGAWASYFGEPEP  491
                    +S  R   PS+++   +LP NM+V+S+DVE                  E  P
Sbjct  367  ------AEQSTARLVPPSEKQELGLLPPNMFVTSIDVE------------------EGLP  402

Query  492  EPEPEAEADSIPRSRTRTDTEVFLDYGEDEEEGVERVLAPSTSTSTLPSASSIVSEQTVG  551
              + + +     ++    DT V LDYGE  E         ++++  +P  + I +     
Sbjct  403  SRKRKRKVVVTQQTVPVEDT-VTLDYGEPLE---------ASNSPEMPDVAQIEAR----  448

Query  552  WESEWAALEDAWGSLDGESHIKSSGLKQGSVIGWNDVTLNPKTYTPELTLLVALVRSV  609
            WES +  + D             S ++ G  + W  + +N  T+TPE  L +A V S 
Sbjct  449  WES-YPRITD------------KSQVQPGMTVAWKALGINRHTFTPEFLLNIAQVMSC  493


>gb|KIN98457.1| hypothetical protein M404DRAFT_1005307 [Pisolithus tinctorius 
Marx 270]
Length=586

 Score = 48.9 bits (115),  Expect = 0.014, Method: Compositional matrix adjust.
 Identities = 35/104 (34%), Positives = 52/104 (50%), Gaps = 10/104 (10%)

Query  1    MRFKLSADTPTGPLKAWVLVPESL--LPS-GTIDHLESYLVDLFPSLCRADDDRGSVTKP  57
            MR K++   P   LKAW  VP +    PS  T+ +L++ L +  P L        +V   
Sbjct  1    MRIKVTTGPPLPLLKAWFPVPSAHPETPSEFTVANLKTRLCNTLPLL-------STVAAS  53

Query  58   KLRLSIDGFDLLADTSINVIAPDDVVRVTLASISQTALIASGTK  101
             LRLSIDGF+LL D  I V+   D+V +   + S+  +   G +
Sbjct  54   SLRLSIDGFELLDDCDIGVVRDGDLVCIEQVNASEKVIKPKGNQ  97


>gb|KIR68723.1| hypothetical protein I314_01147 [Cryptococcus gattii CA1873]
Length=615

 Score = 48.9 bits (115),  Expect = 0.015, Method: Compositional matrix adjust.
 Identities = 43/135 (32%), Positives = 69/135 (51%), Gaps = 20/135 (15%)

Query  1    MRFKLSADTPTGPLKAWVLVPESLLPSGTIDHLESYLVDLFPSLCRADDDRGSVTKPKLR  60
            MR KLS   P    +  +LVP  +    TI HL+ YL++   S+ +      + +  +L 
Sbjct  1    MRIKLSFLPPFPSSRILLLVPNDV---KTITHLKKYLINSLSSVAQH-----ATSSQELI  52

Query  61   LSIDGFDLLADTSINVIAPDDVVRVTLASISQTALIASGTKRKALSASSPESSPVASKRA  120
            L I+GF LL+ + +N+I P DVV V          IA GT  ++  +S  +SS   +K+ 
Sbjct  53   LEIEGFQLLSGSDLNIIEPTDVVCVR---------IAPGTSTESPMSSLNQSS---NKKR  100

Query  121  RKSSPPSAGTTTKER  135
            +++S P     TK+R
Sbjct  101  KRNSAPKLPRQTKKR  115


>gb|KIR49684.1| hypothetical protein I312_00773, partial [Cryptococcus gattii 
CA1280]
Length=716

 Score = 48.9 bits (115),  Expect = 0.016, Method: Compositional matrix adjust.
 Identities = 43/135 (32%), Positives = 69/135 (51%), Gaps = 20/135 (15%)

Query  1    MRFKLSADTPTGPLKAWVLVPESLLPSGTIDHLESYLVDLFPSLCRADDDRGSVTKPKLR  60
            MR KLS   P    +  +LVP  +    TI HL+ YL++   S+ +      + +  +L 
Sbjct  1    MRIKLSFLPPFPSSRILLLVPNDV---KTITHLKKYLINSLSSVAQH-----ATSSQELI  52

Query  61   LSIDGFDLLADTSINVIAPDDVVRVTLASISQTALIASGTKRKALSASSPESSPVASKRA  120
            L I+GF LL+ + +N+I P DVV V          IA GT  ++  +S  +SS   +K+ 
Sbjct  53   LEIEGFQLLSGSDLNIIEPTDVVCVR---------IAPGTSTESPMSSLNQSS---NKKR  100

Query  121  RKSSPPSAGTTTKER  135
            +++S P     TK+R
Sbjct  101  KRNSAPKLPRQTKKR  115


>gb|EUC56753.1| hypothetical protein RSOL_194520 [Rhizoctonia solani AG-3 Rhs1AP]
Length=542

 Score = 48.9 bits (115),  Expect = 0.017, Method: Compositional matrix adjust.
 Identities = 62/258 (24%), Positives = 108/258 (42%), Gaps = 60/258 (23%)

Query  370  PLSIAFNASSNRNKSSAYRKQAAGSAPTRISYNSVPPPSSLSASAVSATPTRPEPFFAKL  429
            P++ A NA  N+NK   + K  AG+  TRI+Y +  P      +AV+  P       A++
Sbjct  296  PIAPAKNA--NKNKRKGFDKDMAGTVATRITYGTPTP------AAVAVEPP------ARV  341

Query  430  NSVTTPTNNNNHNKSFIRFDAPSDREVLPGNMWVSSVDVESDSWARVVGSGAWASYFGEP  489
            +S T P+       +      PS  + LP N+ V+SVDVE+     + G G W       
Sbjct  342  SSPTVPSTTIKSRYTHYHVVPPSQLQDLPSNVIVTSVDVEAVD--GLEGVGEWFD-----  394

Query  490  EPEPEPEAEADSIPRSRTRTDTEVFLDYGEDEEEGVERVLAPSTSTSTLPSASSIVSEQT  549
                    EA+ +                 DE++          + S  P   +  + + 
Sbjct  395  --------EANRV----------------MDEDK---------IAASDKPQPKTSNTSKK  421

Query  550  VGWE---SEWAALEDAWGSLDGESHIKSSGLKQGSVIGWNDVTLNPKTYTPELTLLVALV  606
            + WE   SEW  L D++ +++  +    + L+ G+++ +  +T++P T TP   + V  V
Sbjct  422  IDWEVVDSEWERLWDSFSTIEQAAW---TNLQAGTLLAYPGLTIDPVTCTPCSKIHVVRV  478

Query  607  RSVCTDSNSIRYILKPRP  624
             S  +D  +       RP
Sbjct  479  VSGPSDDGNAECFFIDRP  496


>gb|KIR27314.1| hypothetical protein I309_03781 [Cryptococcus gattii LA55]
 gb|KIR42989.1| hypothetical protein I313_01197 [Cryptococcus gattii Ram5]
 gb|KIR95426.1| hypothetical protein I304_00176 [Cryptococcus gattii CBS 10090]
 gb|KIS01922.1| hypothetical protein L804_00177 [Cryptococcus gattii 2001/935-1]
 gb|KIY55565.1| hypothetical protein I307_05157 [Cryptococcus gattii 99/473]
Length=696

 Score = 48.9 bits (115),  Expect = 0.017, Method: Compositional matrix adjust.
 Identities = 47/137 (34%), Positives = 67/137 (49%), Gaps = 24/137 (18%)

Query  1    MRFKLSADTPTGPLKAWVLVPESLLPSGTIDHLESYLVDLFPSLCRADDDRGSVTKPKLR  60
            MR KLS   P    +  +LVP  +    TI HL+ YL+    S+ +      + +  +L 
Sbjct  1    MRIKLSFLPPFPSSRILLLVPNDV---KTITHLKKYLIKSLSSVAQH-----ASSSQELL  52

Query  61   LSIDGFDLLADTSINVIAPDDVVRVTLASISQTALIASGTKRKALSASSPESSPVAS--K  118
            L I+GF LL+ + +N+I P DVV V          IA GT     SA SP SS   S  K
Sbjct  53   LEIEGFQLLSGSDLNIIEPTDVVCVR---------IAPGT-----SAESPMSSLNQSSNK  98

Query  119  RARKSSPPSAGTTTKER  135
            + +++S P     TK+R
Sbjct  99   KRKRNSAPKLPRQTKKR  115


>gb|KGB75079.1| hypothetical protein CNBG_0917 [Cryptococcus gattii R265]
 gb|KIR75486.1| hypothetical protein I310_00178 [Cryptococcus gattii CA1014]
Length=696

 Score = 48.9 bits (115),  Expect = 0.018, Method: Compositional matrix adjust.
 Identities = 47/137 (34%), Positives = 67/137 (49%), Gaps = 24/137 (18%)

Query  1    MRFKLSADTPTGPLKAWVLVPESLLPSGTIDHLESYLVDLFPSLCRADDDRGSVTKPKLR  60
            MR KLS   P    +  +LVP  +    TI HL+ YL+    S+ +      + +  +L 
Sbjct  1    MRIKLSFLPPFPSSRILLLVPNDV---KTITHLKKYLIKSLSSVAQH-----ASSSQELL  52

Query  61   LSIDGFDLLADTSINVIAPDDVVRVTLASISQTALIASGTKRKALSASSPESSPVAS--K  118
            L I+GF LL+ + +N+I P DVV V          IA GT     SA SP SS   S  K
Sbjct  53   LEIEGFQLLSGSDLNIIEPTDVVCVR---------IAPGT-----SAESPMSSLNQSSNK  98

Query  119  RARKSSPPSAGTTTKER  135
            + +++S P     TK+R
Sbjct  99   KRKRNSAPKLPRQTKKR  115


>gb|KIR57022.1| hypothetical protein I315_00182 [Cryptococcus gattii Ru294]
Length=742

 Score = 48.1 bits (113),  Expect = 0.034, Method: Compositional matrix adjust.
 Identities = 44/137 (32%), Positives = 66/137 (48%), Gaps = 24/137 (18%)

Query  1    MRFKLSADTPTGPLKAWVLVPESLLPSGTIDHLESYLVDLFPSLCRADDDRGSVTKPKLR  60
            MR KLS   P    +  +LVP  +    TI HL+ YL+    S+ +      + +  +L 
Sbjct  1    MRIKLSFLPPFPSSRILLLVPNDV---KTITHLKKYLIKSLSSVAQH-----ASSSHELL  52

Query  61   LSIDGFDLLADTSINVIAPDDVVRVTLASISQTALIASGTKRKALSASSPESSPVAS--K  118
            L I+GF LL+ + +N+I P DVV V +A               + S  SP SS   S  K
Sbjct  53   LEIEGFQLLSGSDLNIIEPTDVVCVRIAP--------------STSTESPMSSLNQSSNK  98

Query  119  RARKSSPPSAGTTTKER  135
            + +++SPP     TK+R
Sbjct  99   KRKRNSPPKPLRQTKKR  115


>gb|KIR80452.1| hypothetical protein I306_02429 [Cryptococcus gattii EJB2]
Length=697

 Score = 47.8 bits (112),  Expect = 0.037, Method: Compositional matrix adjust.
 Identities = 44/137 (32%), Positives = 66/137 (48%), Gaps = 24/137 (18%)

Query  1    MRFKLSADTPTGPLKAWVLVPESLLPSGTIDHLESYLVDLFPSLCRADDDRGSVTKPKLR  60
            MR KLS   P    +  +LVP  +    TI HL+ YL+    S+ +      + +  +L 
Sbjct  1    MRIKLSFLPPFPSSRILLLVPNDV---KTITHLKKYLIKSLSSVAQH-----ASSSHELL  52

Query  61   LSIDGFDLLADTSINVIAPDDVVRVTLASISQTALIASGTKRKALSASSPESSPVAS--K  118
            L I+GF LL+ + +N+I P DVV V +A               + S  SP SS   S  K
Sbjct  53   LEIEGFQLLSGSDLNIIEPTDVVCVRIAP--------------STSTESPMSSLNQSSNK  98

Query  119  RARKSSPPSAGTTTKER  135
            + +++SPP     TK+R
Sbjct  99   KRKRNSPPKPLRQTKKR  115


>ref|XP_003193823.1| hypothetical protein CGB_D7700C [Cryptococcus gattii WM276]
 gb|ADV22036.1| Hypothetical protein CGB_D7700C [Cryptococcus gattii WM276]
Length=723

 Score = 47.8 bits (112),  Expect = 0.038, Method: Compositional matrix adjust.
 Identities = 44/137 (32%), Positives = 66/137 (48%), Gaps = 24/137 (18%)

Query  1    MRFKLSADTPTGPLKAWVLVPESLLPSGTIDHLESYLVDLFPSLCRADDDRGSVTKPKLR  60
            MR KLS   P    +  +LVP  +    TI HL+ YL+    S+ +      + +  +L 
Sbjct  1    MRIKLSFLPPFPSSRILLLVPNDV---KTITHLKKYLIKSLSSVAQH-----ASSSHELL  52

Query  61   LSIDGFDLLADTSINVIAPDDVVRVTLASISQTALIASGTKRKALSASSPESSPVAS--K  118
            L I+GF LL+ + +N+I P DVV V +A               + S  SP SS   S  K
Sbjct  53   LEIEGFQLLSGSDLNIIEPTDVVCVRIAP--------------STSTESPMSSLNQSSNK  98

Query  119  RARKSSPPSAGTTTKER  135
            + +++SPP     TK+R
Sbjct  99   KRKRNSPPKPLRQTKKR  115


>gb|KIJ61031.1| hypothetical protein HYDPIDRAFT_31729 [Hydnomerulius pinastri 
MD-312]
Length=595

 Score = 47.4 bits (111),  Expect = 0.050, Method: Compositional matrix adjust.
 Identities = 29/88 (33%), Positives = 46/88 (52%), Gaps = 4/88 (5%)

Query  1   MRFKLSADTPTGPLKAWVLVPESL---LPSGTIDHLESYLVDLFPSLCRADDDRGSVTKP  57
           MR K+S ++P  PLKAW   P SL    P+   D  +  +  L   +C    D    +  
Sbjct  1   MRIKISTNSPLPPLKAWFPFP-SLPDEGPTSPFDPPKRTIYSLKYLICATLPDLSFCSPS  59

Query  58  KLRLSIDGFDLLADTSINVIAPDDVVRV  85
            LRL+IDGF+LL + ++ V+   D++ +
Sbjct  60  HLRLTIDGFELLDECALTVVRDGDLICI  87


>gb|KEP49340.1| S1/P1 nuclease [Rhizoctonia solani 123E]
Length=892

 Score = 47.4 bits (111),  Expect = 0.056, Method: Compositional matrix adjust.
 Identities = 63/258 (24%), Positives = 107/258 (41%), Gaps = 60/258 (23%)

Query  370  PLSIAFNASSNRNKSSAYRKQAAGSAPTRISYNSVPPPSSLSASAVSATPTRPEPFFAKL  429
            P++ A NA  N+NK   + K  AG+  TRI+Y +  P      +AV+  PT      A++
Sbjct  294  PIAPAKNA--NKNKRKGFDKDMAGTVATRITYGTPTP------AAVAVEPT------ARV  339

Query  430  NSVTTPTNNNNHNKSFIRFDAPSDREVLPGNMWVSSVDVESDSWARVVGSGAWASYFGEP  489
            +S T P        +      PS  + LP N+ V+SVDVE+     + G G W       
Sbjct  340  SSPTVPNTTIKSRYTHYHVVPPSQLQDLPSNVIVTSVDVEAVD--GLEGVGEWFD-----  392

Query  490  EPEPEPEAEADSIPRSRTRTDTEVFLDYGEDEEEGVERVLAPSTSTSTLPSASSIVSEQT  549
                    EA+ +                 DE++          + S  P   +  + + 
Sbjct  393  --------EANRV----------------MDEDK---------IAASDKPQPKTSNTSKK  419

Query  550  VGWE---SEWAALEDAWGSLDGESHIKSSGLKQGSVIGWNDVTLNPKTYTPELTLLVALV  606
            + WE   SEW  L D++ +++  +    + L+ G+++ +  +T++P T TP   + V  V
Sbjct  420  IDWEVVDSEWERLWDSFSTIEQAAW---TNLQAGTLLAYPGLTIDPVTCTPCSKIHVVRV  476

Query  607  RSVCTDSNSIRYILKPRP  624
             S  +D          RP
Sbjct  477  VSGPSDDGKAECFFIDRP  494


>gb|KIR36053.1| hypothetical protein I352_00996 [Cryptococcus gattii MMRL2647]
Length=698

 Score = 47.0 bits (110),  Expect = 0.060, Method: Compositional matrix adjust.
 Identities = 46/137 (34%), Positives = 67/137 (49%), Gaps = 24/137 (18%)

Query  1    MRFKLSADTPTGPLKAWVLVPESLLPSGTIDHLESYLVDLFPSLCRADDDRGSVTKPKLR  60
            MR KLS   P    +  +LVP  +    TI HL+ YL+    S+ +      + +  +L 
Sbjct  1    MRIKLSFLPPFPSSRILLLVPSDV---KTITHLKKYLIKSLSSVAQH-----ASSSQELL  52

Query  61   LSIDGFDLLADTSINVIAPDDVVRVTLASISQTALIASGTKRKALSASSPESSPVAS--K  118
            L I+GF LL+ + +N+I P DVV V          I+ GT     SA SP SS   S  K
Sbjct  53   LEIEGFQLLSGSDLNIIEPTDVVCVR---------ISPGT-----SAESPMSSLNQSSNK  98

Query  119  RARKSSPPSAGTTTKER  135
            + +++S P     TK+R
Sbjct  99   KRKRNSAPKLPRQTKKR  115


>gb|KIK21937.1| hypothetical protein PISMIDRAFT_532418 [Pisolithus microcarpus 
441]
Length=509

 Score = 46.6 bits (109),  Expect = 0.082, Method: Compositional matrix adjust.
 Identities = 35/113 (31%), Positives = 55/113 (49%), Gaps = 8/113 (7%)

Query  1    MRFKLSADTPTGPLKAWVLVPESLLPSGTIDHLESYLVDLFPSLCRADDDRGSVTKPKLR  60
            MR K++   P   L+AW  VP S  P  +    E  + +L   LC      G+V    LR
Sbjct  1    MRIKVTTGPPLPLLRAWFPVP-SPRPESSSTSPELTIANLKTRLCNTLALLGTVPPSALR  59

Query  61   LSIDGFDLLADTSINVIAPDDVVRVTLASISQTALIA-------SGTKRKALS  106
            LSIDGF+LL +  + V+   D+V +   +  ++ +++       SG +RK  S
Sbjct  60   LSIDGFELLDECDLGVVRDGDLVCIEQINPPESIMVSKENQPERSGDRRKHTS  112


>emb|CUA76453.1| putative WD repeat-containing protein alr2800 [Nostoc sp, PCC 
7120] [Rhizoctonia solani]
Length=2541

 Score = 46.6 bits (109),  Expect = 0.11, Method: Compositional matrix adjust.
 Identities = 88/374 (24%), Positives = 141/374 (38%), Gaps = 110/374 (29%)

Query  256   IIPANPPVPPGAGSTATQERNIRRRRKKAALKAENAAAARSAAVTVPKLPSLLPPPASPS  315
             + P+ PPVPPG G ++T+ RN RRR  +  + A  A                  P A+P+
Sbjct  2109  VTPSQPPVPPGQGKSSTKNRNARRRALRKHVAAGTA----------------FTPQATPT  2152

Query  316   PFVESISSSSSSSSSSSSSSSSSSSSSSSSSSPSSPRVAASTLLPTQRSLESVVPLSIAF  375
             P                            +S+  +P  A  +L+ TQ     +    IA 
Sbjct  2153  P----------------------------ASATITPTPAIESLIATQ----VIASTPIAL  2180

Query  376   NASSNRNKSSAYRKQAAGSAPTRISYNSVPPPSSLSASAVSATPTRPEPFFAKLNSVTTP  435
               S+N+NK   + K  A +  TRI+Y + P P+ +S   V   P    P F        P
Sbjct  2181  AKSANKNKRKGFDKDMADAVATRITYGT-PAPAPIS---VEPPPRTDSPAF--------P  2228

Query  436   TNNNNHNKSFIRFDAPSDREVLPGNMWVSSVDVESDSWARVVGSGAWASYFGEPEPEPEP  495
                     +      PS  + LP N+ VSSVDVE+                         
Sbjct  2229  IATTKSRYTHYHVVPPSQLKDLPSNVIVSSVDVEA-------------------------  2263

Query  496   EAEADSIPRSRTRTDTEVFLDYGE--DEEEGVERVLAPSTSTSTLPSASSIVSEQTVGWE  553
                AD +             D GE  D E  V    A + + ++ P++++  + + + WE
Sbjct  2264  ---ADGLE------------DVGEWFDGENHVAEGDATAANVNSPPASNN--TNKKIDWE  2306

Query  554   ---SEWAALEDAWGSLDGESHIKSSGLKQGSVIGWNDVTLNPKTYTPELTLLVALVRSVC  610
                S+W   E +WGS       +   L+ G+++ +  + ++P T TP   + +A V S  
Sbjct  2307  VVDSQW---EQSWGSFPTIDQARWDKLQPGTLLAYLGLAIDPVTCTPGTKIHLARVISGP  2363

Query  611   TDSNSIRYILKPRP  624
             +D          RP
Sbjct  2364  SDGGQAECFFIERP  2377


>ref|XP_008035934.1| hypothetical protein TRAVEDRAFT_144804 [Trametes versicolor FP-101664 
SS1]
 gb|EIW62314.1| hypothetical protein TRAVEDRAFT_144804 [Trametes versicolor FP-101664 
SS1]
Length=545

 Score = 45.8 bits (107),  Expect = 0.13, Method: Compositional matrix adjust.
 Identities = 87/373 (23%), Positives = 143/373 (38%), Gaps = 77/373 (21%)

Query  260  NPPVPPGAGSTATQERNIRRRRKKAALKAENAAAARSAAVTVPKLPSLLPPPASPSPFVE  319
            +P +PPG G   T  RN+RRRRKK   +                  SL   PAS    V 
Sbjct  202  HPSIPPGFGKPTTHSRNLRRRRKKMYERL-----------------SLTAEPAS----VN  240

Query  320  SISSSSSSSSSSSSSSSSSSSSSSSSSSPSSPRVAASTLLPTQ-----RSLESVVPLSIA  374
             I     + +  +S +SS      ++ +P           P Q     +  E+ V    A
Sbjct  241  DIPLGERARTDDASPASSPPEPEPTALAP-----------PAQGKTKGKGKETAVEEHPA  289

Query  375  FNASS--NRNKSSAYRKQAAGSAPTRISYNSVPPPSSLSASAVSATPTRPEPFFAKLNSV  432
            F  +S  N+NK   ++       P +I ++  P P++     V A     E     L S 
Sbjct  290  FLMASLQNKNKRRGFKHALTQGVPAKILFSDAPRPAAEQNMDVDADARVVEAGLM-LES-  347

Query  433  TTPTNNNNHNKSFIRFDAPSDRE---VLPGNMWVSSVDVESDSWARVVGSGAWASYFGEP  489
                   + +++  R   PS+++   +LP NM+V+SVDVE          G W +   + 
Sbjct  348  -----QRSSSRAQPRLVPPSEKQERGLLPANMFVTSVDVE---------EGLWPAKGKKK  393

Query  490  EPEPEPEAEADSIPRSRTRTDTEVFLDYGEDEEEGVERVLAPSTSTSTLPSASSIVSEQT  549
            + +  P  EA                DY E+EE+     L P      +P+A      Q 
Sbjct  394  KKKKAPVKEAQ--------------WDY-EEEEQTFAGGL-PYDDVPEVPAAVPAAGAQD  437

Query  550  VGWESEWAALEDAWGSLDGESHIKSSGLKQGSVIGWNDVTLNPKTYTPELTLLVALVRSV  609
                +E   +   W +L          ++ G+ + W  + +N  T+TPE+ L V  V + 
Sbjct  438  DSASTERVVVAARWDTL--RKITDKEQVQIGTTVAWKALGINFATFTPEVLLHVGRV-AK  494

Query  610  CTDSNSIRYILKP  622
            C +   +  + +P
Sbjct  495  CDEELVVELVAEP  507


>ref|XP_003036973.1| hypothetical protein SCHCODRAFT_103389 [Schizophyllum commune 
H4-8]
 gb|EFJ02071.1| hypothetical protein SCHCODRAFT_103389, partial [Schizophyllum 
commune H4-8]
Length=572

 Score = 45.4 bits (106),  Expect = 0.17, Method: Compositional matrix adjust.
 Identities = 51/176 (29%), Positives = 78/176 (44%), Gaps = 29/176 (16%)

Query  441  HNKSFIRFDAPSDREVLPGNMWVSSVDVESDSWARVVGSGAWASYFGEPEPEPEPEAEAD  500
            H     R   PS+R  LP N++V+SVDVE+   A   G     S + +   E E + EA+
Sbjct  368  HQARLPRLVPPSERTDLPPNVFVTSVDVEAGWEADTSGYKYDTSAYEQTYAE-ENQDEAN  426

Query  501  SIPRSRTRTDTEVFLDYGEDEEEGVERVLAPSTSTSTLPSASSIVSEQTVGWESEWAALE  560
                        + L Y +D ++      APS + +T  SA            S + AL 
Sbjct  427  ------------IQLSYNDDYDDA-----APSLNATTNASAQP----------SPYPALA  459

Query  561  DAWGSLDGESHIKS-SGLKQGSVIGWNDVTLNPKTYTPELTLLVALVRSVCTDSNS  615
             +  S      + S + L+ G  +GW  + LN  T TPE+T+++A V +V  D  S
Sbjct  460  QSLASATASQPVASLAQLEVGKHVGWTSLELNAATMTPEVTVVIAKVVAVDDDGES  515


>ref|XP_007311998.1| hypothetical protein SERLADRAFT_431589 [Serpula lacrymans var. 
lacrymans S7.9]
 gb|EGO04170.1| hypothetical protein SERLA73DRAFT_67886 [Serpula lacrymans var. 
lacrymans S7.3]
 gb|EGO30114.1| hypothetical protein SERLADRAFT_431589 [Serpula lacrymans var. 
lacrymans S7.9]
Length=522

 Score = 44.7 bits (104),  Expect = 0.28, Method: Compositional matrix adjust.
 Identities = 38/125 (30%), Positives = 57/125 (46%), Gaps = 13/125 (10%)

Query  1    MRFKLSADTPTGPLKAWVLVPESLLPSGTIDHLESYLVDLFPSLCRADDDRGSVTKPKLR  60
            MRFK+    P   LKAW  V E    + TI  L+  L+     L      +    K  + 
Sbjct  1    MRFKICTSPPLPDLKAWFAV-ELNEKTRTIWDLKHVLLSDLHVL------QDYQAKDGIS  53

Query  61   LSIDGFDLLADTSINVIAPDDVVRVTLASISQTALI------ASGTKRKALSASSPESSP  114
            LS+DGF+LL +T IN++   D+V + L ++S   L        +G  ++A+  S     P
Sbjct  54   LSMDGFELLDETPINILRDGDLVDIKLDTVSSRPLAKEPLAHVTGKTKQAIKDSHSTDLP  113

Query  115  VASKR  119
               KR
Sbjct  114  AKRKR  118


>gb|KIJ19748.1| hypothetical protein PAXINDRAFT_166002 [Paxillus involutus ATCC 
200175]
Length=588

 Score = 44.7 bits (104),  Expect = 0.31, Method: Compositional matrix adjust.
 Identities = 31/94 (33%), Positives = 45/94 (48%), Gaps = 16/94 (17%)

Query  1   MRFKLSADTPTGPLKAWVLVPES--LLPSG-------TIDHLESYLVDLFPSLCRADDDR  51
           MR K+S + P   L+AW  +P +    PS        T+  L+  L    P+LC      
Sbjct  1   MRIKISTNPPLPALRAWFTIPSTSDRGPSSCHDKSRSTVQSLKFLLCSSLPTLC------  54

Query  52  GSVTKPKLRLSIDGFDLLADTSINVIAPDDVVRV  85
             V    LRLSID F+LL D+ + V+   D+V +
Sbjct  55  -GVAPSCLRLSIDEFELLDDSELTVVRDGDLVCI  87


>gb|KJA18214.1| hypothetical protein HYPSUDRAFT_45548 [Hypholoma sublateritium 
FD-334 SS-4]
Length=587

 Score = 41.6 bits (96),  Expect = 2.8, Method: Compositional matrix adjust.
 Identities = 50/201 (25%), Positives = 69/201 (34%), Gaps = 72/201 (36%)

Query  455  EVLPGNMWVSSVDVESDSWARV-------------------------VGSGAWASYFGEP  489
            E LP NM V+SVDVE+  W                            VG G +A      
Sbjct  382  ERLPRNMIVTSVDVEAGMWDDTSYAEPAKKKKRGKKKAPQDEEYYADVGEGYYA------  435

Query  490  EPEPEPEAEADSIPRSRTRTDTEVFLDYGEDEEEGVERVLAPSTSTSTLPSASSIVSEQT  549
                +  A   ++P         V LDYGE +  G                         
Sbjct  436  ----DGGAWGGAVPADADEPAGPVVLDYGETDAAG------------------------E  467

Query  550  VGWESEWAALEDAWGS----LDGESHIKSSGLKQGSVIGWNDVTLNPKTYTPELTLLVAL  605
             G   +W   E  W S    L G   I +     G+++GW  + +NP+T++PE+ L VA 
Sbjct  468  GGEHFDWDRAERLWDSKSTVLSGPEQIVA-----GALVGWKALAINPRTFSPEVLLSVAH  522

Query  606  V----RSVCTDSNSIRYILKP  622
            V     S       IR I +P
Sbjct  523  VVQPPDSTAGKGVLIRQIQRP  543


Lambda      K        H        a         alpha
   0.305    0.120    0.327    0.792     4.96 

Gapped
Lambda      K        H        a         alpha    sigma
   0.267   0.0410    0.140     1.90     42.6     43.6 

Effective search space used: 7448033162095


  Database: nr
    Posted date:  Sep 23, 2015 12:05 AM
  Number of letters in database: 26,053,659,533
  Number of sequences in database:  71,551,133


Matrix: BLOSUM62
Gap Penalties: Existence: 11, Extension: 1
Neighboring words threshold: 11
Window for multiple hits: 40
```
