# Supplementary material for: Comparative genomics provides new insights into the diversity, physiology, and sexuality of the only industrially exploited tremellomycete: Phaffia rhodozyma
Source: BMC Genomics. 2016 Nov 9;17:901. doi: 10.1186/s12864-016-3244-7 (PMC5103461; doi:10.1186/s12864-016-3244-7)
Supplement: Additional file 6: — List of orphan genes with links to PFAM (related to Additional file 1: Table S1). (ZIP 1428 kb) [file 12864_2016_3244_MOESM6_ESM.zip › BLAST_HTML_FTR/G04818_P.html]

BLAST Search Results


```
BLASTP 2.2.27+


Reference:
Stephen F. Altschul, Thomas L. Madden, Alejandro A. Schäffer,
Jinghui Zhang, Zheng Zhang, Webb Miller, and David J. Lipman (1997),
"Gapped BLAST and PSI-BLAST: a new generation of protein database
search programs", Nucleic Acids Res. 25:3389-3402.


Reference for
composition-based statistics:
Alejandro A. Schäffer, L. Aravind, Thomas L. Madden, Sergei
Shavirin, John L. Spouge, Yuri I. Wolf, Eugene V. Koonin, and
Stephen F. Altschul (2001), "Improving the accuracy of PSI-BLAST
protein database searches with composition-based statistics and
other refinements", Nucleic Acids Res. 29:2994-3005.


Database: nr
           71,551,133 sequences; 26,053,659,533 total letters


Query= G04818_P

Length=403
                                                                      Score     E
Sequences producing significant alignments:                          (Bits)  Value

emb|CED82132.1|  hypothetical protein [Xanthophyllomyces dendrorh...   819    0.0  
emb|CCT65493.1|  uncharacterized protein FFUJ_02437 [Fusarium fuj...  39.3    6.9  


 >emb|CED82132.1| hypothetical protein [Xanthophyllomyces dendrorhous]
Length=402

 Score =  819 bits (2116),  Expect = 0.0, Method: Compositional matrix adjust.
 Identities = 402/402 (100%), Positives = 402/402 (100%), Gaps = 0/402 (0%)

Query  1    MLGRVSVACRRSGRSLFNIAHPSPAFHVHACQTPFSRFASSTSPVDVASSTSLNVPLPSS  60
            MLGRVSVACRRSGRSLFNIAHPSPAFHVHACQTPFSRFASSTSPVDVASSTSLNVPLPSS
Sbjct  1    MLGRVSVACRRSGRSLFNIAHPSPAFHVHACQTPFSRFASSTSPVDVASSTSLNVPLPSS  60

Query  61   PPKSRTVDETECVSASLHPASAETKPEPNTRILKSPPVISNRRLKALKYRLKIPEPLIFL  120
            PPKSRTVDETECVSASLHPASAETKPEPNTRILKSPPVISNRRLKALKYRLKIPEPLIFL
Sbjct  61   PPKSRTVDETECVSASLHPASAETKPEPNTRILKSPPVISNRRLKALKYRLKIPEPLIFL  120

Query  121  NLTGFRRVVIPQAVAVRDQLAKKAKKGSIDPAEFIDEMNKLETERILKIPQDIHDSIRSK  180
            NLTGFRRVVIPQAVAVRDQLAKKAKKGSIDPAEFIDEMNKLETERILKIPQDIHDSIRSK
Sbjct  121  NLTGFRRVVIPQAVAVRDQLAKKAKKGSIDPAEFIDEMNKLETERILKIPQDIHDSIRSK  180

Query  181  RKFWKFIRLPSFGPRSFRKLISAARAATSFFMVTGDRPNLVKAKRELRFLEEHYMLYFPD  240
            RKFWKFIRLPSFGPRSFRKLISAARAATSFFMVTGDRPNLVKAKRELRFLEEHYMLYFPD
Sbjct  181  RKFWKFIRLPSFGPRSFRKLISAARAATSFFMVTGDRPNLVKAKRELRFLEEHYMLYFPD  240

Query  241  IAIRTNSLGDAFSTFGKKKVDSKRLVQIMETGGVEAMIAYRKDLDSGNDTSPVVKTEVPT  300
            IAIRTNSLGDAFSTFGKKKVDSKRLVQIMETGGVEAMIAYRKDLDSGNDTSPVVKTEVPT
Sbjct  241  IAIRTNSLGDAFSTFGKKKVDSKRLVQIMETGGVEAMIAYRKDLDSGNDTSPVVKTEVPT  300

Query  301  KTNSPLNKKRAKSPTESIPTQRETVGDRSQKMIEQAKDGVMDVVGRVQESVSQAIESVPT  360
            KTNSPLNKKRAKSPTESIPTQRETVGDRSQKMIEQAKDGVMDVVGRVQESVSQAIESVPT
Sbjct  301  KTNSPLNKKRAKSPTESIPTQRETVGDRSQKMIEQAKDGVMDVVGRVQESVSQAIESVPT  360

Query  361  YTSASSAEVSKNMVGASSDKDTPTSTKEQGSSEQTKQSRSDP  402
            YTSASSAEVSKNMVGASSDKDTPTSTKEQGSSEQTKQSRSDP
Sbjct  361  YTSASSAEVSKNMVGASSDKDTPTSTKEQGSSEQTKQSRSDP  402


>emb|CCT65493.1| uncharacterized protein FFUJ_02437 [Fusarium fujikuroi IMI 58289]
 gb|KLO99618.1| uncharacterized protein LW94_11105 [Fusarium fujikuroi]
Length=557

 Score = 39.3 bits (90),  Expect = 6.9, Method: Compositional matrix adjust.
 Identities = 46/177 (26%), Positives = 75/177 (42%), Gaps = 28/177 (16%)

Query  230  LEEHYMLY-FP-DIAIRTNSLGDAFSTFGKKKVDSKRLVQIMETGGVEAMIAYRKDLDSG  287
            LEEH+ +Y +P    +   S         +K VD + L +I      E ++         
Sbjct  329  LEEHWRIYAYPFGCNLGFTSRNGCRDHLSQKHVDQRHLSEI------EDLVQL-------  375

Query  288  NDTSPVVKTEVPTKTNSPLNKKRAKSPTESIPTQRETVGDRSQK--MIEQAKDGVMDVVG  345
               S V KTE+P KT  PL KK      ++I T R  +G   ++  ++  + D  M    
Sbjct  376  ---STVGKTEIPDKTTCPLCKKL----LQNIETYRNHIGQHQKQLALLAISLDPTMAEEA  428

Query  346  RVQESVSQAIESVPTYTSASSAEVSKNMVGASSDKD--TPTSTKEQGSSEQTKQSRS  400
             +Q S    I+    Y ++   E S+N    S+DK     ++  + G  EQ+  S+S
Sbjct  429  SLQPSAETEIDE--HYIASQDTETSENEGPVSTDKGKKVASTLDDSGLGEQSDTSKS  483


Lambda      K        H        a         alpha
   0.315    0.129    0.360    0.792     4.96 

Gapped
Lambda      K        H        a         alpha    sigma
   0.267   0.0410    0.140     1.90     42.6     43.6 

Effective search space used: 3776584046000


  Database: nr
    Posted date:  Sep 23, 2015 12:05 AM
  Number of letters in database: 26,053,659,533
  Number of sequences in database:  71,551,133


Matrix: BLOSUM62
Gap Penalties: Existence: 11, Extension: 1
Neighboring words threshold: 11
Window for multiple hits: 40
```
