# Supplementary material for: Comparative genomics provides new insights into the diversity, physiology, and sexuality of the only industrially exploited tremellomycete: Phaffia rhodozyma
Source: BMC Genomics. 2016 Nov 9;17:901. doi: 10.1186/s12864-016-3244-7 (PMC5103461; doi:10.1186/s12864-016-3244-7)
Supplement: Additional file 6: — List of orphan genes with links to PFAM (related to Additional file 1: Table S1). (ZIP 1428 kb) [file 12864_2016_3244_MOESM6_ESM.zip › BLAST_HTML_FTR/G01103_P.html]

BLAST Search Results


```
BLASTP 2.2.27+


Reference:
Stephen F. Altschul, Thomas L. Madden, Alejandro A. Schäffer,
Jinghui Zhang, Zheng Zhang, Webb Miller, and David J. Lipman (1997),
"Gapped BLAST and PSI-BLAST: a new generation of protein database
search programs", Nucleic Acids Res. 25:3389-3402.


Reference for
composition-based statistics:
Alejandro A. Schäffer, L. Aravind, Thomas L. Madden, Sergei
Shavirin, John L. Spouge, Yuri I. Wolf, Eugene V. Koonin, and
Stephen F. Altschul (2001), "Improving the accuracy of PSI-BLAST
protein database searches with composition-based statistics and
other refinements", Nucleic Acids Res. 29:2994-3005.


Database: nr
           71,551,133 sequences; 26,053,659,533 total letters


Query= G01103_P

Length=180
                                                                      Score     E
Sequences producing significant alignments:                          (Bits)  Value

emb|CDZ97338.1|  hypothetical protein [Xanthophyllomyces dendrorh...   362    3e-125
ref|XP_003660661.1|  hypothetical protein MYCTH_2299218 [Myceliop...  38.1    0.47  
ref|XP_010899411.1|  PREDICTED: rap guanine nucleotide exchange f...  40.8    0.56  
emb|CCX09754.1|  Similar to hypothetical protein PTRG_11321 [Pyre...  37.7    0.70  
ref|XP_007307673.1|  hypothetical protein STEHIDRAFT_160170 [Ster...  37.0    1.3   
gb|EMD88230.1|  hypothetical protein COCHEDRAFT_1023386 [Bipolari...  37.0    1.3   
ref|XP_007689136.1|  hypothetical protein COCMIDRAFT_37801 [Bipol...  37.0    1.6   
ref|WP_026847373.1|  MFS transporter [Geodermatophilaceae bacteri...  38.9    1.8   
gb|EMF10875.1|  hypothetical protein SEPMUDRAFT_49643 [Sphaerulin...  36.2    2.5   
ref|WP_014738901.1|  MFS transporter [Modestobacter marinus] >emb...  38.5    2.6   
ref|XP_007709099.1|  hypothetical protein COCCADRAFT_2381 [Bipola...  35.8    3.2   
ref|XP_007699102.1|  hypothetical protein COCSADRAFT_36122 [Bipol...  35.8    3.6   
dbj|GAM40215.1|  long-chain-fatty-acid-CoA ligase [Talaromyces ce...  37.7    5.1   
emb|CCD55303.1|  hypothetical protein BofuT4_P156990.1 [Botrytis ...  35.0    8.1   


 >emb|CDZ97338.1| hypothetical protein [Xanthophyllomyces dendrorhous]
Length=179

 Score =  362 bits (930),  Expect = 3e-125, Method: Compositional matrix adjust.
 Identities = 179/179 (100%), Positives = 179/179 (100%), Gaps = 0/179 (0%)

Query  1    MFSILRSQILSKSPRSHALLPSQLAFSTTLSVFLNPSGPHRQPPKPHPTVVRKLREQEER  60
            MFSILRSQILSKSPRSHALLPSQLAFSTTLSVFLNPSGPHRQPPKPHPTVVRKLREQEER
Sbjct  1    MFSILRSQILSKSPRSHALLPSQLAFSTTLSVFLNPSGPHRQPPKPHPTVVRKLREQEER  60

Query  61   TRRNKDVIIDTKEAEPPSPFSTLSTAYMSIPPQTRLLLGLGLMAFGWLGTVYLGDTPNTK  120
            TRRNKDVIIDTKEAEPPSPFSTLSTAYMSIPPQTRLLLGLGLMAFGWLGTVYLGDTPNTK
Sbjct  61   TRRNKDVIIDTKEAEPPSPFSTLSTAYMSIPPQTRLLLGLGLMAFGWLGTVYLGDTPNTK  120

Query  121  TTVSPRASGSALPTPGERRKQELEMEYMKKQFAEMRKGGGPDALGGSSGFIQVIDPKHS  179
            TTVSPRASGSALPTPGERRKQELEMEYMKKQFAEMRKGGGPDALGGSSGFIQVIDPKHS
Sbjct  121  TTVSPRASGSALPTPGERRKQELEMEYMKKQFAEMRKGGGPDALGGSSGFIQVIDPKHS  179


>ref|XP_003660661.1| hypothetical protein MYCTH_2299218 [Myceliophthora thermophila 
ATCC 42464]
 gb|AEO55416.1| hypothetical protein MYCTH_2299218 [Myceliophthora thermophila 
ATCC 42464]
Length=66

 Score = 38.1 bits (87),  Expect = 0.47, Method: Compositional matrix adjust.
 Identities = 19/50 (38%), Positives = 32/50 (64%), Gaps = 1/50 (2%)

Query  82   TLSTAYMSIPPQTRLLLGLGLMAFGWLGTVYLGDTPNTKTTVSPRASGSA  131
            +L  AY ++PP+T+L +G+GL+A+G LG  + GD   +K   +P  +  A
Sbjct  2    SLLKAYTNLPPKTKLTVGVGLVAWGLLGLTF-GDKIESKLGFTPTEADKA  50


>ref|XP_010899411.1| PREDICTED: rap guanine nucleotide exchange factor 1-like isoform 
X2 [Esox lucius]
Length=1179

 Score = 40.8 bits (94),  Expect = 0.56, Method: Compositional matrix adjust.
 Identities = 20/53 (38%), Positives = 30/53 (57%), Gaps = 5/53 (9%)

Query  116  TPNTKTTVSPRASGSALPTPGERRKQELEMEYMKKQFAEMRKGGGPDALGGSS  168
            +P     VSP + GS+LP    RR+QE E EY++++F+     G   + GG S
Sbjct  266  SPTRVAVVSPVSRGSSLPCRAHRRQQEYEQEYLQRRFS-----GSSQSYGGDS  313


>emb|CCX09754.1| Similar to hypothetical protein PTRG_11321 [Pyrenophora tritici-repentis 
Pt-1C-BFP]; acc. no. XP_001941652 [Pyronema omphalodes 
CBS 100304]
Length=66

 Score = 37.7 bits (86),  Expect = 0.70, Method: Compositional matrix adjust.
 Identities = 19/46 (41%), Positives = 28/46 (61%), Gaps = 1/46 (2%)

Query  82   TLSTAYMSIPPQTRLLLGLGLMAFGWLGTVYLGDTPNTKTTVSPRA  127
            +L   Y S+ P+TR+LLG G++ +G LG +YL D    K   +P A
Sbjct  2    SLINYYRSLHPRTRMLLGAGVIVYGTLG-LYLSDVAEEKLGFTPTA  46


>ref|XP_007307673.1| hypothetical protein STEHIDRAFT_160170 [Stereum hirsutum FP-91666 
SS1]
 gb|EIM83592.1| hypothetical protein STEHIDRAFT_160170 [Stereum hirsutum FP-91666 
SS1]
Length=76

 Score = 37.0 bits (84),  Expect = 1.3, Method: Compositional matrix adjust.
 Identities = 19/42 (45%), Positives = 27/42 (64%), Gaps = 1/42 (2%)

Query  74   AEPPSPFSTLSTAYMSIPPQTRLLLGLGLMAFGWLGTVYLGD  115
            +EPP    TL  +Y ++P QTRL L +GL AF  +G +Y+ D
Sbjct  17   SEPPVYRRTLWQSYAALPAQTRLRLSIGLFAFAAVG-LYVSD  57


>gb|EMD88230.1| hypothetical protein COCHEDRAFT_1023386 [Bipolaris maydis C5]
 gb|ENI02191.1| hypothetical protein COCC4DRAFT_33528 [Bipolaris maydis ATCC 
48331]
Length=67

 Score = 37.0 bits (84),  Expect = 1.3, Method: Compositional matrix adjust.
 Identities = 15/39 (38%), Positives = 27/39 (69%), Gaps = 1/39 (3%)

Query  87   YMSIPPQTRLLLGLGLMAFGWLGTVYLGDTPNTKTTVSP  125
            + SIPP+TR+++G+G+MA+  +G +Y+ D    K   +P
Sbjct  7    FKSIPPKTRMIIGVGVMAYAGVG-LYISDVAEEKLGYTP  44


>ref|XP_007689136.1| hypothetical protein COCMIDRAFT_37801 [Bipolaris oryzae ATCC 
44560]
 gb|EUC44342.1| hypothetical protein COCMIDRAFT_37801 [Bipolaris oryzae ATCC 
44560]
Length=67

 Score = 37.0 bits (84),  Expect = 1.6, Method: Compositional matrix adjust.
 Identities = 15/39 (38%), Positives = 27/39 (69%), Gaps = 1/39 (3%)

Query  87   YMSIPPQTRLLLGLGLMAFGWLGTVYLGDTPNTKTTVSP  125
            + SIPP+TR+++G+G+MA+  +G +Y+ D    K   +P
Sbjct  7    FKSIPPKTRMIIGVGVMAYAGVG-LYISDVAEEKLGYTP  44


>ref|WP_026847373.1| MFS transporter [Geodermatophilaceae bacterium URHA0031]
Length=450

 Score = 38.9 bits (89),  Expect = 1.8, Method: Compositional matrix adjust.
 Identities = 28/95 (29%), Positives = 42/95 (44%), Gaps = 2/95 (2%)

Query  18   ALLPSQLAFSTTLSVFLNPSGPHRQPPKPHPTVVRKLREQEERTRRNKDVIIDTKEAEPP  77
            A L +  +F+ T++  L   G   +P  P      +LRE    TRR+ D+I+    A   
Sbjct  172  AFLINAASFAVTIAALLAMRGSELRPSPPASRSRGQLREALGYTRRHPDLILAMSLAFVA  231

Query  78   SPFSTLSTAYMSIPPQTRLLLGLGLMAFGWLGTVY  112
              F       ++I    R   GLG  AFG++ T Y
Sbjct  232  GTFGF--NTQITIALMAREEFGLGATAFGFMSTAY  264


>gb|EMF10875.1| hypothetical protein SEPMUDRAFT_49643 [Sphaerulina musiva SO2202]
Length=67

 Score = 36.2 bits (82),  Expect = 2.5, Method: Compositional matrix adjust.
 Identities = 26/77 (34%), Positives = 37/77 (48%), Gaps = 11/77 (14%)

Query  81   STLSTAYMSIPPQTRLLLGLGLMAFGWLGTVYLGDTPNTKTTVSPRASGSALPTPGERRK  140
            S L  +Y ++ P+TRLL+G G+MA+G LG             +S RA       P E+ K
Sbjct  2    SGLWQSYRNLAPKTRLLVGGGIMAWGALGLF-----------ISDRAEQFLGLVPTEQDK  50

Query  141  QELEMEYMKKQFAEMRK  157
            + L+    K  F E  K
Sbjct  51   ENLQHAMPKIHFVEKEK  67


>ref|WP_014738901.1| MFS transporter [Modestobacter marinus]
 emb|CCH86293.1| Major facilitator superfamily MFS_1 [Modestobacter marinus]
Length=450

 Score = 38.5 bits (88),  Expect = 2.6, Method: Compositional matrix adjust.
 Identities = 27/95 (28%), Positives = 42/95 (44%), Gaps = 2/95 (2%)

Query  18   ALLPSQLAFSTTLSVFLNPSGPHRQPPKPHPTVVRKLREQEERTRRNKDVIIDTKEAEPP  77
            A L +  +F+ T++  L   G   +P  P      +LRE    TRR+ D+++    A   
Sbjct  172  AFLVNAASFAVTIAALLAMRGSELRPSPPASRSRGQLREALGYTRRHPDLVLAMSLAFVA  231

Query  78   SPFSTLSTAYMSIPPQTRLLLGLGLMAFGWLGTVY  112
              F       ++I    R   GLG  AFG++ T Y
Sbjct  232  GTFGF--NTQITIALMAREEFGLGATAFGFMSTAY  264


>ref|XP_007709099.1| hypothetical protein COCCADRAFT_2381 [Bipolaris zeicola 26-R-13]
 gb|EUC36548.1| hypothetical protein COCCADRAFT_2381 [Bipolaris zeicola 26-R-13]
 gb|EUN22443.1| hypothetical protein COCVIDRAFT_30549 [Bipolaris victoriae FI3]
Length=67

 Score = 35.8 bits (81),  Expect = 3.2, Method: Compositional matrix adjust.
 Identities = 15/39 (38%), Positives = 26/39 (67%), Gaps = 1/39 (3%)

Query  87   YMSIPPQTRLLLGLGLMAFGWLGTVYLGDTPNTKTTVSP  125
            + SIPP+TR+++G+G+MA+  +G +Y+ D    K    P
Sbjct  7    FKSIPPKTRMIIGVGVMAYAGVG-LYISDVAEEKLGYVP  44


>ref|XP_007699102.1| hypothetical protein COCSADRAFT_36122 [Bipolaris sorokiniana 
ND90Pr]
 gb|EMD64744.1| hypothetical protein COCSADRAFT_36122 [Bipolaris sorokiniana 
ND90Pr]
Length=67

 Score = 35.8 bits (81),  Expect = 3.6, Method: Compositional matrix adjust.
 Identities = 14/39 (36%), Positives = 27/39 (69%), Gaps = 1/39 (3%)

Query  87   YMSIPPQTRLLLGLGLMAFGWLGTVYLGDTPNTKTTVSP  125
            + +IPP+TR+++G+G+MA+  +G +Y+ D    K   +P
Sbjct  7    FKNIPPKTRMIIGVGVMAYAGVG-LYISDVAEEKLGYTP  44


>dbj|GAM40215.1| long-chain-fatty-acid-CoA ligase [Talaromyces cellulolyticus]
Length=734

 Score = 37.7 bits (86),  Expect = 5.1, Method: Compositional matrix adjust.
 Identities = 21/59 (36%), Positives = 33/59 (56%), Gaps = 11/59 (19%)

Query  86   AYMSIPPQTRLLLGLGLMAFGWLGTVYLGDTPNTKTTVSPRASGSALPTPGERRKQELE  144
            +Y ++ P+TRLL G+GLMA+  +G            T SP+   +    P E++K+ELE
Sbjct  673  SYKNLSPRTRLLFGVGLMAWAGIG-----------MTASPQIESALGLVPTEQQKEELE  720


>emb|CCD55303.1| hypothetical protein BofuT4_P156990.1 [Botrytis cinerea T4]
Length=72

 Score = 35.0 bits (79),  Expect = 8.1, Method: Compositional matrix adjust.
 Identities = 19/49 (39%), Positives = 30/49 (61%), Gaps = 2/49 (4%)

Query  77   PSPFSTLSTAYMSIPPQTRLLLGLGLMAFGWLGTVYLGDTPNTKTTVSP  125
            PS  S LS+ + ++PP+TR ++G   +A+G +G +YL DT   K    P
Sbjct  8    PSKMSLLSS-FRALPPRTRAMIGASFIAWGTIG-LYLSDTAEKKLGFEP  54


Lambda      K        H        a         alpha
   0.318    0.135    0.397    0.792     4.96 

Gapped
Lambda      K        H        a         alpha    sigma
   0.267   0.0410    0.140     1.90     42.6     43.6 

Effective search space used: 641460036520


  Database: nr
    Posted date:  Sep 23, 2015 12:05 AM
  Number of letters in database: 26,053,659,533
  Number of sequences in database:  71,551,133


Matrix: BLOSUM62
Gap Penalties: Existence: 11, Extension: 1
Neighboring words threshold: 11
Window for multiple hits: 40
```
