# Supplementary material for: Comparative genomics provides new insights into the diversity, physiology, and sexuality of the only industrially exploited tremellomycete: Phaffia rhodozyma
Source: BMC Genomics. 2016 Nov 9;17:901. doi: 10.1186/s12864-016-3244-7 (PMC5103461; doi:10.1186/s12864-016-3244-7)
Supplement: Additional file 6: — List of orphan genes with links to PFAM (related to Additional file 1: Table S1). (ZIP 1428 kb) [file 12864_2016_3244_MOESM6_ESM.zip › BLAST_HTML_FTR/G03468_P.html]

BLAST Search Results


```
BLASTP 2.2.27+


Reference:
Stephen F. Altschul, Thomas L. Madden, Alejandro A. Schäffer,
Jinghui Zhang, Zheng Zhang, Webb Miller, and David J. Lipman (1997),
"Gapped BLAST and PSI-BLAST: a new generation of protein database
search programs", Nucleic Acids Res. 25:3389-3402.


Reference for
composition-based statistics:
Alejandro A. Schäffer, L. Aravind, Thomas L. Madden, Sergei
Shavirin, John L. Spouge, Yuri I. Wolf, Eugene V. Koonin, and
Stephen F. Altschul (2001), "Improving the accuracy of PSI-BLAST
protein database searches with composition-based statistics and
other refinements", Nucleic Acids Res. 29:2994-3005.


Database: nr
           71,551,133 sequences; 26,053,659,533 total letters


Query= G03468_P

Length=560
                                                                      Score     E
Sequences producing significant alignments:                          (Bits)  Value

emb|CED85627.1|  BRCT domain [Xanthophyllomyces dendrorhous]           969    0.0  
ref|XP_007693137.1|  hypothetical protein COCMIDRAFT_109060 [Bipo...  44.7    0.26 
emb|CAZ90042.1|  putative Cytochrome c [Thiomonas arsenitoxydans]     43.5    0.45 
gb|EUN24630.1|  hypothetical protein COCVIDRAFT_39877 [Bipolaris ...  43.9    0.49 
ref|XP_012467107.1|  PREDICTED: DNA polymerase beta isoform X2 [G...  43.9    0.50 
ref|WP_050985992.1|  cytochrome C [Thiomonas arsenitoxydans]          43.1    0.53 
ref|WP_013124332.1|  cytochrome C [Thiomonas intermedia] >sp|D5WY...  43.1    0.54 
ref|XP_007716110.1|  hypothetical protein COCCADRAFT_8231 [Bipola...  43.5    0.55 
emb|CDW93805.1|  Cytochrome c class I [Thiomonas sp. CB2]             43.1    0.57 
gb|KHG01220.1|  DNA polymerase lambda [Gossypium arboreum]            42.4    1.2  
gb|ENI01651.1|  hypothetical protein COCC4DRAFT_147825 [Bipolaris...  42.7    1.2  
gb|EMD94638.1|  hypothetical protein COCHEDRAFT_1191475 [Bipolari...  42.4    1.3  
ref|XP_007705677.1|  hypothetical protein COCSADRAFT_176398 [Bipo...  42.4    1.4  
gb|KMZ90523.1|  formin-binding protein [Plasmodium vivax Mauritan...  42.4    1.7  
gb|KIN03698.1|  hypothetical protein OIDMADRAFT_116894 [Oidiodend...  38.9    6.4  
ref|XP_001617092.1|  formin-binding protein [Plasmodium vivax Sal...  40.4    6.5  
ref|XP_002978521.1|  hypothetical protein SELMODRAFT_418187 [Sela...  39.7    8.0  
gb|AAC51212.1|  MOP2 [Homo sapiens]                                   39.7    10.0 


 >emb|CED85627.1| BRCT domain [Xanthophyllomyces dendrorhous]
Length=486

 Score =  969 bits (2505),  Expect = 0.0, Method: Compositional matrix adjust.
 Identities = 482/484 (99%), Positives = 483/484 (99%), Gaps = 0/484 (0%)

Query  1    MSDFFASLDALNEDPFYQDTRIDLDMDLALSRLHRVHHSSKKRTVRGCHDLELTVNREPT  60
            MSDFFASLDALNEDPFYQDTRIDLDMDLALSRLHRVHHSSKKRTVRGCHDLELTVNREPT
Sbjct  1    MSDFFASLDALNEDPFYQDTRIDLDMDLALSRLHRVHHSSKKRTVRGCHDLELTVNREPT  60

Query  61   SRSKQTLLSPGSLSPSAISPLDTSTSSRSIDLRKVTLLRSLVSGPGSRPKPEQQPPIASN  120
            SRSKQTLLSPGSLSPSAISPLDTSTSSRSIDLRKVTLLRSLVSGPGSRPKPEQQPPIASN
Sbjct  61   SRSKQTLLSPGSLSPSAISPLDTSTSSRSIDLRKVTLLRSLVSGPGSRPKPEQQPPIASN  120

Query  121  QSVNHARTSMNQDAQGEHYFVNQTFSHPPSSDMVSPSSLSFGSGLSEQPDHPIPLHTNPP  180
            QSVNHARTSMNQDAQGEHYFVNQTFSHPPSSDMVSPSSLSFGSGLSEQPDHPIPLHTNPP
Sbjct  121  QSVNHARTSMNQDAQGEHYFVNQTFSHPPSSDMVSPSSLSFGSGLSEQPDHPIPLHTNPP  180

Query  181  ILRSVSPISTQMAFDLLSNVTTPNTSKLEPDERPPVSLSRPPHIENALVAVARRSKQRST  240
            ILRSVSPISTQMAFDLLSNVTTPNTSKLEPDERPPVSLSRPPHIENALVAVARRSKQRST
Sbjct  181  ILRSVSPISTQMAFDLLSNVTTPNTSKLEPDERPPVSLSRPPHIENALVAVARRSKQRST  240

Query  241  DKAVHKEPRHVKRLRLAAAAADTENQTTKMSSVPVGVSARGEEVSKARGKTKSKTGPTLR  300
            DKAVHKEPRHVKRLRLAAAAADTENQTTKMSSVPVGVSARGEEVSKARGKTKSKTGPTLR
Sbjct  241  DKAVHKEPRHVKRLRLAAAAADTENQTTKMSSVPVGVSARGEEVSKARGKTKSKTGPTLR  300

Query  301  SLSAVALRLNEALERQSESESVFIEAPAGTDPGRGSRLHVLAGTTVLILWKSVESLVNEY  360
            SLSAVALRLNEALERQSESESVFIEAPAGTDPGRGSRLHVLAGTTVLILWKSVESLVNEY
Sbjct  301  SLSAVALRLNEALERQSESESVFIEAPAGTDPGRGSRLHVLAGTTVLILWKSVESLVNEY  360

Query  361  QANWIEKISSLGAKIAIEISDDVTHVIPDSSDFPSHLILKRFNVRHADDLPTGIKFVRDH  420
            QANWIEKISSLGAKIAIEISDDVTHVIPDSSDFPSHLILKRFNVRHADDLPTGIKFVRDH
Sbjct  361  QANWIEKISSLGAKIAIEISDDVTHVIPDSSDFPSHLILKRFNVRHADDLPTGIKFVRDH  420

Query  421  WIDYSHKTMSRADEEAFLMFSSHRTTFGIGPSSRSPSNSQPAALFSSLSVPIESTRTVFH  480
            WIDYSHKTMSRADEEAFLMFSSHRTTFGIGPSSRSPSNSQPAALFSSLSVPIESTRTVFH
Sbjct  421  WIDYSHKTMSRADEEAFLMFSSHRTTFGIGPSSRSPSNSQPAALFSSLSVPIESTRTVFH  480

Query  481  SSTL  484
            S T+
Sbjct  481  SPTI  484


>ref|XP_007693137.1| hypothetical protein COCMIDRAFT_109060 [Bipolaris oryzae ATCC 
44560]
 gb|EUC40346.1| hypothetical protein COCMIDRAFT_109060 [Bipolaris oryzae ATCC 
44560]
Length=656

 Score = 44.7 bits (104),  Expect = 0.26, Method: Compositional matrix adjust.
 Identities = 29/97 (30%), Positives = 48/97 (49%), Gaps = 12/97 (12%)

Query  367  KISSLGAKIAIEISDDVTHVIPDSSDFPSHLILKRFNVRHADDLPTGIKFVRDHWIDYSH  426
            +++SLGA IA  ++ + TH+I    D+ S+    +    H  D+P     V   W+D   
Sbjct  39   RLTSLGATIASSVTANTTHLIATEKDYESNSTKTKAAAAH--DVPV----VTIEWLDECE  92

Query  427  KTMSRADEEAFLMFSSHRTTFGIGPSSRSPSNSQPAA  463
               S+ DE  +L+ S      G  P+ R+ SN +PA+
Sbjct  93   SKGSKVDETQYLLSS------GAAPAQRNGSNKRPAS  123


>emb|CAZ90042.1| putative Cytochrome c [Thiomonas arsenitoxydans]
Length=307

 Score = 43.5 bits (101),  Expect = 0.45, Method: Compositional matrix adjust.
 Identities = 36/123 (29%), Positives = 56/123 (46%), Gaps = 10/123 (8%)

Query  80   PLDTST----SSRSIDLRKVTLLRSLVSGPG--SRPKPEQQPPIASNQSVNHARTSMNQD  133
            PL + T     S S  L K   +   V+G G  + P+P+Q P     Q V  A+  +   
Sbjct  137  PLGSKTLVALESYSYWLSKGLPVDEKVAGRGYPNLPEPQQAPDYVRGQKVYEAKCILCHA  196

Query  134  AQGEHYFVNQTFSHPPSSDMVSPSSLSFGSGLSEQPDHPIPLHTNPPILRSVSPISTQMA  193
            A GE  +VN     PP   +  P S ++G+G+    +    ++ N P   S S +S Q A
Sbjct  197  ANGEGQYVNGETVFPP---LWGPKSFNWGAGMGSYKNAAKFIYANMPYGMSYS-LSPQEA  252

Query  194  FDL  196
            +D+
Sbjct  253  WDV  255


>gb|EUN24630.1| hypothetical protein COCVIDRAFT_39877 [Bipolaris victoriae FI3]
Length=656

 Score = 43.9 bits (102),  Expect = 0.49, Method: Compositional matrix adjust.
 Identities = 28/97 (29%), Positives = 48/97 (49%), Gaps = 12/97 (12%)

Query  367  KISSLGAKIAIEISDDVTHVIPDSSDFPSHLILKRFNVRHADDLPTGIKFVRDHWIDYSH  426
            +++SLGA IA  ++ + TH+I    D+ S+    +    H  D+P     V   W+D   
Sbjct  39   RLTSLGATIASSVTANTTHLIATEKDYESNSTKTKAAATH--DVPV----VTIEWLDECE  92

Query  427  KTMSRADEEAFLMFSSHRTTFGIGPSSRSPSNSQPAA  463
               S+ DE  +L+ S      G  P+ ++ SN +PA+
Sbjct  93   SKGSKVDEAQYLLSS------GAAPAQKNGSNKRPAS  123


>ref|XP_012467107.1| PREDICTED: DNA polymerase beta isoform X2 [Gossypium raimondii]
Length=530

 Score = 43.9 bits (102),  Expect = 0.50, Method: Compositional matrix adjust.
 Identities = 32/105 (30%), Positives = 50/105 (48%), Gaps = 14/105 (13%)

Query  340  VLAGTTVLILWKSVESLVNEYQANWIEKISSLGAKIAIEISDDVTHVIPDSSDFPSHLI-  398
            + AG TV ++   V+SL  +    W +K+  +GAK+  ++S+ V+HV   SSD   H + 
Sbjct  18   MFAGMTVFLIEDGVQSLRLQI---WKQKLVQMGAKVETQLSEKVSHVFAISSDALFHHVD  74

Query  399  ---LKRFNVRHADDLPTGIKFVRDHWIDYSHKTMSRADEEAFLMF  440
               L RF     + L  G K   D +I        + D E F +F
Sbjct  75   DDRLARFKGWLEESLAAGEKVSEDFYI-------LKVDPEGFDIF  112


>ref|WP_050985992.1| cytochrome C [Thiomonas arsenitoxydans]
Length=314

 Score = 43.1 bits (100),  Expect = 0.53, Method: Compositional matrix adjust.
 Identities = 36/123 (29%), Positives = 56/123 (46%), Gaps = 10/123 (8%)

Query  80   PLDTST----SSRSIDLRKVTLLRSLVSGPG--SRPKPEQQPPIASNQSVNHARTSMNQD  133
            PL + T     S S  L K   +   V+G G  + P+P+Q P     Q V  A+  +   
Sbjct  144  PLGSKTLVALESYSYWLSKGLPVDEKVAGRGYPNLPEPQQAPDYVRGQKVYEAKCILCHA  203

Query  134  AQGEHYFVNQTFSHPPSSDMVSPSSLSFGSGLSEQPDHPIPLHTNPPILRSVSPISTQMA  193
            A GE  +VN     PP   +  P S ++G+G+    +    ++ N P   S S +S Q A
Sbjct  204  ANGEGQYVNGETVFPP---LWGPKSFNWGAGMGSYKNAAKFIYANMPYGMSYS-LSPQEA  259

Query  194  FDL  196
            +D+
Sbjct  260  WDV  262


>ref|WP_013124332.1| cytochrome C [Thiomonas intermedia]
 sp|D5WYQ5|TSDA_THIK1 RecName: Full=Thiosulfate dehydrogenase; AltName: Full=Tetrathionate 
synthase; Flags: Precursor
 gb|ADG32232.1| cytochrome c class I [Thiomonas intermedia K12]
Length=314

 Score = 43.1 bits (100),  Expect = 0.54, Method: Compositional matrix adjust.
 Identities = 36/123 (29%), Positives = 56/123 (46%), Gaps = 10/123 (8%)

Query  80   PLDTST----SSRSIDLRKVTLLRSLVSGPG--SRPKPEQQPPIASNQSVNHARTSMNQD  133
            PL + T     S S  L K   +   V+G G  + P+P+Q P     Q V  A+  +   
Sbjct  144  PLGSKTLVALESYSYWLSKGLPVDEKVAGRGYPNLPEPQQAPDYVRGQKVYEAKCILCHA  203

Query  134  AQGEHYFVNQTFSHPPSSDMVSPSSLSFGSGLSEQPDHPIPLHTNPPILRSVSPISTQMA  193
            A GE  +VN     PP   +  P S ++G+G+    +    ++ N P   S S +S Q A
Sbjct  204  ANGEGQYVNGETVFPP---LWGPKSFNWGAGMGSYKNAAKFIYANMPYGMSYS-LSPQEA  259

Query  194  FDL  196
            +D+
Sbjct  260  WDV  262


>ref|XP_007716110.1| hypothetical protein COCCADRAFT_8231 [Bipolaris zeicola 26-R-13]
 gb|EUC29582.1| hypothetical protein COCCADRAFT_8231 [Bipolaris zeicola 26-R-13]
Length=656

 Score = 43.5 bits (101),  Expect = 0.55, Method: Compositional matrix adjust.
 Identities = 28/97 (29%), Positives = 48/97 (49%), Gaps = 12/97 (12%)

Query  367  KISSLGAKIAIEISDDVTHVIPDSSDFPSHLILKRFNVRHADDLPTGIKFVRDHWIDYSH  426
            +++SLGA IA  ++ + TH+I    D+ S+    +    H  D+P     V   W+D   
Sbjct  39   RLTSLGATIASSVTANTTHLIATEKDYESNSTKTKAAATH--DVPV----VTIEWLDECE  92

Query  427  KTMSRADEEAFLMFSSHRTTFGIGPSSRSPSNSQPAA  463
               S+ DE  +L+ S      G  P+ ++ SN +PA+
Sbjct  93   SKGSKVDETQYLLSS------GAAPAQKNGSNKRPAS  123


>emb|CDW93805.1| Cytochrome c class I [Thiomonas sp. CB2]
Length=314

 Score = 43.1 bits (100),  Expect = 0.57, Method: Compositional matrix adjust.
 Identities = 36/123 (29%), Positives = 56/123 (46%), Gaps = 10/123 (8%)

Query  80   PLDTST----SSRSIDLRKVTLLRSLVSGPG--SRPKPEQQPPIASNQSVNHARTSMNQD  133
            PL + T     S S  L K   +   V+G G  + P+P+Q P     Q V  A+  +   
Sbjct  144  PLGSKTLVALESYSYWLSKGLPVDEKVAGRGYPNLPEPQQAPDYVRGQKVYEAKCILCHA  203

Query  134  AQGEHYFVNQTFSHPPSSDMVSPSSLSFGSGLSEQPDHPIPLHTNPPILRSVSPISTQMA  193
            A GE  +VN     PP   +  P S ++G+G+    +    ++ N P   S S +S Q A
Sbjct  204  ANGEGQYVNGETVFPP---LWGPKSFNWGAGMGSYKNAAKFIYANMPYGMSYS-LSPQEA  259

Query  194  FDL  196
            +D+
Sbjct  260  WDV  262


>gb|KHG01220.1| DNA polymerase lambda [Gossypium arboreum]
Length=530

 Score = 42.4 bits (98),  Expect = 1.2, Method: Compositional matrix adjust.
 Identities = 31/105 (30%), Positives = 50/105 (48%), Gaps = 14/105 (13%)

Query  340  VLAGTTVLILWKSVESLVNEYQANWIEKISSLGAKIAIEISDDVTHVIPDSSDFPSHLI-  398
            + AG TV ++   V+S+  +    W +K+  +GAK+  ++S+ V+HV   SSD   H + 
Sbjct  18   MFAGMTVFLIEDGVQSVRLQI---WKQKLVQMGAKVETQLSEKVSHVFAISSDALFHHVD  74

Query  399  ---LKRFNVRHADDLPTGIKFVRDHWIDYSHKTMSRADEEAFLMF  440
               L RF     + L  G K   D +I        + D E F +F
Sbjct  75   GDRLARFKGWLEESLAAGEKVSEDFYI-------LKVDPEGFDIF  112


>gb|ENI01651.1| hypothetical protein COCC4DRAFT_147825 [Bipolaris maydis ATCC 
48331]
Length=656

 Score = 42.7 bits (99),  Expect = 1.2, Method: Compositional matrix adjust.
 Identities = 26/97 (27%), Positives = 46/97 (47%), Gaps = 12/97 (12%)

Query  367  KISSLGAKIAIEISDDVTHVIPDSSDFPSHLILKRFNVRHADDLPTGIKFVRDHWIDYSH  426
            +++SLGA IA  ++ + TH+I    D+ S+    +    H       +  V   W+D   
Sbjct  39   RLTSLGATIASSVTANTTHLIATEKDYESNSTKTKAAAAH------NVPVVTIEWLDECE  92

Query  427  KTMSRADEEAFLMFSSHRTTFGIGPSSRSPSNSQPAA  463
               S+ DE  +L+ S      G  P+ ++ SN +PA+
Sbjct  93   SKGSKVDETQYLLSS------GTAPAQKNGSNKRPAS  123


>gb|EMD94638.1| hypothetical protein COCHEDRAFT_1191475 [Bipolaris maydis C5]
Length=656

 Score = 42.4 bits (98),  Expect = 1.3, Method: Compositional matrix adjust.
 Identities = 26/97 (27%), Positives = 46/97 (47%), Gaps = 12/97 (12%)

Query  367  KISSLGAKIAIEISDDVTHVIPDSSDFPSHLILKRFNVRHADDLPTGIKFVRDHWIDYSH  426
            +++SLGA IA  ++ + TH+I    D+ S+    +    H       +  V   W+D   
Sbjct  39   RLTSLGATIASSVTANTTHLIATEKDYESNSTKTKAAAAH------NVPVVTIEWLDECE  92

Query  427  KTMSRADEEAFLMFSSHRTTFGIGPSSRSPSNSQPAA  463
               S+ DE  +L+ S      G  P+ ++ SN +PA+
Sbjct  93   SKGSKVDETQYLLSS------GTAPAQKNGSNKRPAS  123


>ref|XP_007705677.1| hypothetical protein COCSADRAFT_176398 [Bipolaris sorokiniana 
ND90Pr]
 gb|EMD58582.1| hypothetical protein COCSADRAFT_176398 [Bipolaris sorokiniana 
ND90Pr]
Length=658

 Score = 42.4 bits (98),  Expect = 1.4, Method: Compositional matrix adjust.
 Identities = 27/97 (28%), Positives = 48/97 (49%), Gaps = 12/97 (12%)

Query  367  KISSLGAKIAIEISDDVTHVIPDSSDFPSHLILKRFNVRHADDLPTGIKFVRDHWIDYSH  426
            +++SLGA IA  ++ + TH+I    D+ S+    +    H  D+P     +   W+D   
Sbjct  39   RLASLGATIASSVTANTTHLIATEKDYESNSTKTKAAAAH--DVPV----LTIEWLDECE  92

Query  427  KTMSRADEEAFLMFSSHRTTFGIGPSSRSPSNSQPAA  463
               S+ DE  +L+ S      G  P+ ++ SN +PA+
Sbjct  93   SKGSKVDETQYLLSS------GAAPAQKNGSNKRPAS  123


>gb|KMZ90523.1| formin-binding protein [Plasmodium vivax Mauritania I]
Length=880

 Score = 42.4 bits (98),  Expect = 1.7, Method: Compositional matrix adjust.
 Identities = 31/104 (30%), Positives = 49/104 (47%), Gaps = 3/104 (3%)

Query  243  AVHKEPRHVKRLRLAAAAADTENQTTKMSSVPVGVSARGEEVSKARGKTKSKTGPTLRSL  302
            +V  EP  +K+++L  AA D ENQ + +   P   S   E V+K  G+  + T P+    
Sbjct  255  SVWDEPEDIKKIKLECAAEDAENQES-VDKCPNSSSTTHESVNK--GENANNTPPSGFPK  311

Query  303  SAVALRLNEALERQSESESVFIEAPAGTDPGRGSRLHVLAGTTV  346
             AV    ++A+   S   +   E P+  D G  + LH+  G  V
Sbjct  312  EAVNQTTDDAMNNASVDSTTAKEHPSSNDLGMYNYLHMQNGMPV  355


>gb|KIN03698.1| hypothetical protein OIDMADRAFT_116894 [Oidiodendron maius Zn]
Length=171

 Score = 38.9 bits (89),  Expect = 6.4, Method: Composition-based stats.
 Identities = 24/70 (34%), Positives = 35/70 (50%), Gaps = 7/70 (10%)

Query  354  ESLVNEYQANWIEKISSLGAKIAIEISDDVTHVIPDSSDFPSHLILKRFNVRHADDLPTG  413
            E+  +E  ANWI    + G +   E+SDD TH+I        H +LK   V+ A DL T 
Sbjct  41   ENWGHEQMANWIR---AHGGRYEREVSDDTTHLICTV----EHYMLKTVQVKMAQDLGTQ  93

Query  414  IKFVRDHWID  423
             + V   W++
Sbjct  94   CRIVVKDWLE  103


>ref|XP_001617092.1| formin-binding protein [Plasmodium vivax Sal-1]
 gb|EDL47365.1| formin-binding protein, putative [Plasmodium vivax]
 gb|KMZ77913.1| formin-binding protein [Plasmodium vivax India VII]
 gb|KMZ84253.1| formin-binding protein [Plasmodium vivax Brazil I]
 gb|KMZ97141.1| formin-binding protein [Plasmodium vivax North Korean]
Length=880

 Score = 40.4 bits (93),  Expect = 6.5, Method: Compositional matrix adjust.
 Identities = 30/104 (29%), Positives = 48/104 (46%), Gaps = 3/104 (3%)

Query  243  AVHKEPRHVKRLRLAAAAADTENQTTKMSSVPVGVSARGEEVSKARGKTKSKTGPTLRSL  302
            +V  EP  +K+++L  AA D ENQ + +   P   S   E V+K  G+  + T P+    
Sbjct  255  SVWDEPEDIKKIKLECAAEDAENQES-VDKCPNSSSTTHESVNK--GENANNTPPSGFPK  311

Query  303  SAVALRLNEALERQSESESVFIEAPAGTDPGRGSRLHVLAGTTV  346
             A     ++A+   S   +   E P+  D G  + LH+  G  V
Sbjct  312  EAANQTTDDAMNNASVDSTTAKEHPSSNDLGMYNYLHMQNGMPV  355


>ref|XP_002978521.1| hypothetical protein SELMODRAFT_418187 [Selaginella moellendorffii]
 gb|EFJ20507.1| hypothetical protein SELMODRAFT_418187 [Selaginella moellendorffii]
Length=346

 Score = 39.7 bits (91),  Expect = 8.0, Method: Compositional matrix adjust.
 Identities = 32/105 (30%), Positives = 45/105 (43%), Gaps = 14/105 (13%)

Query  334  RGSRLHVLAGTTVLILWKSVESLVNEYQANWIEKISSLGAKIAIEISDDVTHVIPDSSDF  393
            R  + H L+G  ++I  KS  +    ++  W +    LGA+  ++I D VTHV+  S   
Sbjct  251  REVKGHALSGCKIVICAKSQAA----HELLW-DSCQELGAECVVDIDDTVTHVVVASKQQ  305

Query  394  PSHLILKRFNVRHADDLPTGIKFVRDHWIDYSHKTMSRADEEAFL  438
            P  L L             G   V   WI  +H    R DE AFL
Sbjct  306  PQGLEL---------SAQAGKYLVWPSWIHTAHYRCCRPDEAAFL  341


>gb|AAC51212.1| MOP2 [Homo sapiens]
Length=870

 Score = 39.7 bits (91),  Expect = 10.0, Method: Compositional matrix adjust.
 Identities = 50/199 (25%), Positives = 78/199 (39%), Gaps = 26/199 (13%)

Query  169  PDHPIPLHTNPPILRSVSPISTQMAFDLLSNVTTPNTSKLEPDERPPVSL--------SR  220
            P H     TN  I + ++P++    F L        + K EP+ RP  S+        S 
Sbjct  560  PQHCFSAMTN--IFQPLAPVAPHSPFLLDKFQQQLESKKTEPERRPMSSIFFDAGSKASL  617

Query  221  PPHIENALVAVARRSKQRSTDKAVHKEPRHVKRLRLAAAAADTENQTTKMSSVPVGVSAR  280
            PP    A   ++     RS  +     P H    + A      + +T  + + P+G    
Sbjct  618  PPCCGQASTPLSSMGG-RSNTQWPPDPPLHFGPTKWAVG----DQRTEFLGAAPLGPPVS  672

Query  281  GEEVSKAR---GKTKSKTGPTLRSLSAVALRLNEALERQSESESVFIEAPAGTDPGRGSR  337
               VS  +    K     GP + S + VAL     L+RQ E E    + P+G DP  GS 
Sbjct  673  PPHVSTFKTRSAKGFGARGPNVLSPAMVALSNKLKLKRQLEYEKQAFQDPSGGDPPGGST  732

Query  338  LHVLAGTTVLILWKSVESL  356
             H        ++WK +++L
Sbjct  733  SH--------LMWKRMKNL  743


Lambda      K        H        a         alpha
   0.315    0.128    0.371    0.792     4.96 

Gapped
Lambda      K        H        a         alpha    sigma
   0.267   0.0410    0.140     1.90     42.6     43.6 

Effective search space used: 6016239845140


  Database: nr
    Posted date:  Sep 23, 2015 12:05 AM
  Number of letters in database: 26,053,659,533
  Number of sequences in database:  71,551,133


Matrix: BLOSUM62
Gap Penalties: Existence: 11, Extension: 1
Neighboring words threshold: 11
Window for multiple hits: 40
```
