# Supplementary material for: Comparative genomics provides new insights into the diversity, physiology, and sexuality of the only industrially exploited tremellomycete: Phaffia rhodozyma
Source: BMC Genomics. 2016 Nov 9;17:901. doi: 10.1186/s12864-016-3244-7 (PMC5103461; doi:10.1186/s12864-016-3244-7)
Supplement: Additional file 6: — List of orphan genes with links to PFAM (related to Additional file 1: Table S1). (ZIP 1428 kb) [file 12864_2016_3244_MOESM6_ESM.zip › BLAST_HTML_FTR/G04263_P.html]

BLAST Search Results


```
BLASTP 2.2.27+


Reference:
Stephen F. Altschul, Thomas L. Madden, Alejandro A. Schäffer,
Jinghui Zhang, Zheng Zhang, Webb Miller, and David J. Lipman (1997),
"Gapped BLAST and PSI-BLAST: a new generation of protein database
search programs", Nucleic Acids Res. 25:3389-3402.


Reference for
composition-based statistics:
Alejandro A. Schäffer, L. Aravind, Thomas L. Madden, Sergei
Shavirin, John L. Spouge, Yuri I. Wolf, Eugene V. Koonin, and
Stephen F. Altschul (2001), "Improving the accuracy of PSI-BLAST
protein database searches with composition-based statistics and
other refinements", Nucleic Acids Res. 29:2994-3005.


Database: nr
           71,551,133 sequences; 26,053,659,533 total letters


Query= G04263_P

Length=453
                                                                      Score     E
Sequences producing significant alignments:                          (Bits)  Value

emb|CED83678.1|  hypothetical protein [Xanthophyllomyces dendrorh...   728    0.0  


 >emb|CED83678.1| hypothetical protein [Xanthophyllomyces dendrorhous]
Length=448

 Score =  728 bits (1879),  Expect = 0.0, Method: Compositional matrix adjust.
 Identities = 441/452 (98%), Positives = 442/452 (98%), Gaps = 4/452 (1%)

Query  1    MSAPLKNAVHNIEEAFTGKSGNANVSQSGATGTTGSDVNSGVHTSRTSEPIPRLTEQGAL  60
            MSAPLKNAVHNIEEAFTGKSGNANVSQSGATGTTGSDVNSGVHTSRTSEPIPRLTEQGAL
Sbjct  1    MSAPLKNAVHNIEEAFTGKSGNANVSQSGATGTTGSDVNSGVHTSRTSEPIPRLTEQGAL  60

Query  61   HSSDTTDNKRIGTDAANRLDNPHTAAGTQGSHSTGAFDSRTGTQTGSHLPSTTSSSANPL  120
            HSSDTTDNKRIGTDAANRLDNPHTAAG QGSHSTGAFDSRTGTQTGSHLPSTTSSSANPL
Sbjct  61   HSSDTTDNKRIGTDAANRLDNPHTAAGIQGSHSTGAFDSRTGTQTGSHLPSTTSSSANPL  120

Query  121  SSQTGHSSHTASSTAAGVVPGATGHHSHEHSHVPGQTGLVGSTGDHTHQRSQVPGQTGLV  180
            SSQTGHSSHTASSTAAGVVPGATGHHSHEHSHVPGQTGLVGSTGDHTHQRSQVPGQTGLV
Sbjct  121  SSQTGHSSHTASSTAAGVVPGATGHHSHEHSHVPGQTGLVGSTGDHTHQRSQVPGQTGLV  180

Query  181  GSVGDHPHQHTGSTLPLETGLPLGPPIGEVSGVSSGLGHSHTSHTSHTSNTSHNLRDGAI  240
            GSVGDHPHQHTGS LPLETGLPLGPPI EVSGVSSGLGHSHTSHTSHTSNTSHNLRD   
Sbjct  181  GSVGDHPHQHTGSALPLETGLPLGPPIVEVSGVSSGLGHSHTSHTSHTSNTSHNLRD---  237

Query  241  GAGVGAGVGAGVAADTVHSHGVSGTHGAYDTRGLSGAQGVSGAHGISETSTGSSLKDEAL  300
               +GAGVGAGVAADTVHSHGVSGTHGAYDTRGL GAQGVSGAHGISETSTGSSLKDEAL
Sbjct  238  -GAIGAGVGAGVAADTVHSHGVSGTHGAYDTRGLPGAQGVSGAHGISETSTGSSLKDEAL  296

Query  301  HRGSQHTGSATGSGLTGVGTGSAAAAAGAAGIAGAGHHGAEGAHDSKLARAEDKLENRAA  360
            HRGSQHTGSATGSGLTGVGTGSAAAAAGAAGIAGAGHHGAEGAHDSKLARAEDKLENRAA
Sbjct  297  HRGSQHTGSATGSGLTGVGTGSAAAAAGAAGIAGAGHHGAEGAHDSKLARAEDKLENRAA  356

Query  361  AHSGTETGAQVGEHDAHTQFDEHGVRWIRDKEGHPFDATHSTAPKATGTTEAGSVTDATH  420
            AHSGTETGAQVGEHDAHTQFDEHGVRWIRDKEGHPFDATHSTAPKATGTTEAGSVTDATH
Sbjct  357  AHSGTETGAQVGEHDAHTQFDEHGVRWIRDKEGHPFDATHSTAPKATGTTEAGSVTDATH  416

Query  421  ADEHAGVATGEHDAAHKASLAEKVKAAVGLGH  452
            ADEHAGVATGEHDAAHKASLAEKVKAAVGLGH
Sbjct  417  ADEHAGVATGEHDAAHKASLAEKVKAAVGLGH  448


Lambda      K        H        a         alpha
   0.306    0.123    0.355    0.792     4.96 

Gapped
Lambda      K        H        a         alpha    sigma
   0.267   0.0410    0.140     1.90     42.6     43.6 

Effective search space used: 4495400730249


  Database: nr
    Posted date:  Sep 23, 2015 12:05 AM
  Number of letters in database: 26,053,659,533
  Number of sequences in database:  71,551,133


Matrix: BLOSUM62
Gap Penalties: Existence: 11, Extension: 1
Neighboring words threshold: 11
Window for multiple hits: 40
```
