# Supplementary material for: Comparative genomics provides new insights into the diversity, physiology, and sexuality of the only industrially exploited tremellomycete: Phaffia rhodozyma
Source: BMC Genomics. 2016 Nov 9;17:901. doi: 10.1186/s12864-016-3244-7 (PMC5103461; doi:10.1186/s12864-016-3244-7)
Supplement: Additional file 6: — List of orphan genes with links to PFAM (related to Additional file 1: Table S1). (ZIP 1428 kb) [file 12864_2016_3244_MOESM6_ESM.zip › BLAST_HTML_FTR/G01111_P.html]

BLAST Search Results


```
BLASTP 2.2.27+


Reference:
Stephen F. Altschul, Thomas L. Madden, Alejandro A. Schäffer,
Jinghui Zhang, Zheng Zhang, Webb Miller, and David J. Lipman (1997),
"Gapped BLAST and PSI-BLAST: a new generation of protein database
search programs", Nucleic Acids Res. 25:3389-3402.


Reference for
composition-based statistics:
Alejandro A. Schäffer, L. Aravind, Thomas L. Madden, Sergei
Shavirin, John L. Spouge, Yuri I. Wolf, Eugene V. Koonin, and
Stephen F. Altschul (2001), "Improving the accuracy of PSI-BLAST
protein database searches with composition-based statistics and
other refinements", Nucleic Acids Res. 29:2994-3005.


Database: nr
           71,551,133 sequences; 26,053,659,533 total letters


Query= G01111_P

Length=149
                                                                      Score     E
Sequences producing significant alignments:                          (Bits)  Value

emb|CDZ97329.1|  hypothetical protein [Xanthophyllomyces dendrorh...   268    3e-89
ref|WP_052011867.1|  transposase [Youngiibacter fragilis]             38.1    1.5  
gb|ESZ92085.1|  hypothetical protein SBOR_7542 [Sclerotinia borea...  37.4    2.5  
ref|WP_042870961.1|  RTX toxin, partial [Aeromonas piscicola]         38.1    2.5  
ref|XP_001588249.1|  predicted protein [Sclerotinia sclerotiorum ...  37.4    2.7  
gb|EJT50702.1|  hypothetical protein A1Q1_08254 [Trichosporon asa...  36.2    3.5  
gb|KIR46371.1|  hypothetical protein I312_04426 [Cryptococcus gat...  36.6    5.2  
gb|KIR59544.1|  hypothetical protein I314_04531 [Cryptococcus gat...  36.6    5.7  
gb|EKD01864.1|  hypothetical protein A1Q2_03927 [Trichosporon asa...  35.4    6.1  
gb|KIR85183.1|  hypothetical protein I308_04434 [Cryptococcus gat...  36.2    7.0  
ref|WP_051675852.1|  hypothetical protein [Polaromonas glacialis]     36.6    7.1  
ref|XP_008106459.1|  PREDICTED: angiopoietin-1 isoform X2 [Anolis...  36.2    9.5  
ref|WP_010180036.1|  hemolysin-type calcium-binding region [Glaci...  36.2    9.5  
ref|WP_012165873.1|  hemolysin expression modulating protein [Aca...  36.2    9.8  


 >emb|CDZ97329.1| hypothetical protein [Xanthophyllomyces dendrorhous]
Length=135

 Score =  268 bits (685),  Expect = 3e-89, Method: Compositional matrix adjust.
 Identities = 135/135 (100%), Positives = 135/135 (100%), Gaps = 0/135 (0%)

Query  14   MTSVAIPDTPDGPPMLGTIGRDTGLGSGSVLGSSLLGEEWAEFLDADTSGDSLDSGVGSQ  73
            MTSVAIPDTPDGPPMLGTIGRDTGLGSGSVLGSSLLGEEWAEFLDADTSGDSLDSGVGSQ
Sbjct  1    MTSVAIPDTPDGPPMLGTIGRDTGLGSGSVLGSSLLGEEWAEFLDADTSGDSLDSGVGSQ  60

Query  74   FSSGMRGRENFQAQGNEEERREKEKVDRLALDVEKRKTLTSKMTLELQELEARLQAADER  133
            FSSGMRGRENFQAQGNEEERREKEKVDRLALDVEKRKTLTSKMTLELQELEARLQAADER
Sbjct  61   FSSGMRGRENFQAQGNEEERREKEKVDRLALDVEKRKTLTSKMTLELQELEARLQAADER  120

Query  134  ESSLRARQQAVRPSY  148
            ESSLRARQQAVRPSY
Sbjct  121  ESSLRARQQAVRPSY  135


>ref|WP_052011867.1| transposase [Youngiibacter fragilis]
Length=297

 Score = 38.1 bits (87),  Expect = 1.5, Method: Compositional matrix adjust.
 Identities = 36/107 (34%), Positives = 48/107 (45%), Gaps = 9/107 (8%)

Query  8    YTSTPNMTSVAIPDTPD--GPPMLGTIGRDTGLGSGSVLGSSLLGEEWAEFLDADTSGDS  65
            Y   P +  V   D+ +  G  +  T+ R +   SG    S L  EE  E    D S   
Sbjct  128  YLKLPKLGLVRFADSREMKGRILNATVRRKS---SGKFFVSILCEEEICELPKTD-SAVG  183

Query  66   LDSGVG--SQFSSGMRGREN-FQAQGNEEERREKEKVDRLALDVEKR  109
            +D G+   +  S G+R   N F  Q  E  RRE+ K+ R ALD EKR
Sbjct  184  IDLGITDFAVLSDGIRNDNNHFTRQMEERLRREQRKLARRALDAEKR  230


>gb|ESZ92085.1| hypothetical protein SBOR_7542 [Sclerotinia borealis F-4157]
Length=187

 Score = 37.4 bits (85),  Expect = 2.5, Method: Compositional matrix adjust.
 Identities = 22/44 (50%), Positives = 32/44 (73%), Gaps = 4/44 (9%)

Query  103  ALDVEKRKTLTSKMTLELQELEARLQAADERESSLRARQQAVRP  146
            A+  EK+  +++K T EL+ELEARL+A +ER   L+A+Q AV P
Sbjct  42   AIVTEKKNEISTK-TSELEELEARLKATEER---LKAKQAAVSP  81


>ref|WP_042870961.1| RTX toxin, partial [Aeromonas piscicola]
Length=1620

 Score = 38.1 bits (87),  Expect = 2.5, Method: Compositional matrix adjust.
 Identities = 34/122 (28%), Positives = 55/122 (45%), Gaps = 10/122 (8%)

Query  25   GPPMLGTIGRDTGLGSGSVLGSSLLGEEWAEFLDADTSGDSLDSGVGSQFSSGMRGRENF  84
            G   +G +G D  +G  SV G +L G    + L A   GD+L  G G+  +  M  R N+
Sbjct  809  GDHFIGGLGNDVLVGGNSVSGDTLDGGVGNDILVAGLGGDTLFGGAGTDLAVLMGSRANY  868

Query  85   QAQGNEEERREKEKVDRLALDVEKRKTLTSK--MTLELQELEARLQAADERESSLRARQQ  142
                   ERR     + L   V++  T  SK    +EL + +  +   ++ + +L A Q 
Sbjct  869  VI-----ERRSDGGFNFL---VKENGTTISKSLYDIELVQFDDGIYQFNQTDGTLTAVQP  920

Query  143  AV  144
            +V
Sbjct  921  SV  922


>ref|XP_001588249.1| predicted protein [Sclerotinia sclerotiorum 1980]
 gb|EDN94821.1| predicted protein [Sclerotinia sclerotiorum 1980 UF-70]
Length=187

 Score = 37.4 bits (85),  Expect = 2.7, Method: Compositional matrix adjust.
 Identities = 22/44 (50%), Positives = 32/44 (73%), Gaps = 4/44 (9%)

Query  103  ALDVEKRKTLTSKMTLELQELEARLQAADERESSLRARQQAVRP  146
            A+  EK+  +++K T EL+ELEARL+A +ER   L+A+Q AV P
Sbjct  42   AIVTEKKNEISTK-TSELEELEARLKATEER---LKAKQAAVTP  81


>gb|EJT50702.1| hypothetical protein A1Q1_08254 [Trichosporon asahii var. asahii 
CBS 2479]
Length=115

 Score = 36.2 bits (82),  Expect = 3.5, Method: Compositional matrix adjust.
 Identities = 27/74 (36%), Positives = 40/74 (54%), Gaps = 6/74 (8%)

Query  66   LDSGVGSQFSSG---MRGRENFQAQG---NEEERREKEKVDRLALDVEKRKTLTSKMTLE  119
            +D+ VG   SSG    R  + F       NEE+ +  E V  L   +EKR++L   +  E
Sbjct  25   MDAVVGGSHSSGNTTTRQHQPFSDASRRVNEEQPQSAEDVQELERKLEKRRSLIPHLENE  84

Query  120  LQELEARLQAADER  133
            L  LEA+++AA+ER
Sbjct  85   LAALEAQIKAAEER  98


>gb|KIR46371.1| hypothetical protein I312_04426 [Cryptococcus gattii CA1280]
Length=191

 Score = 36.6 bits (83),  Expect = 5.2, Method: Compositional matrix adjust.
 Identities = 27/83 (33%), Positives = 43/83 (52%), Gaps = 12/83 (14%)

Query  63   GDSLDSGV--------GSQFSSGMRGRENF----QAQGNEEERREKEKVDRLALDVEKRK  110
            G   D+GV         +  SSG  G  ++    +A G   + R +E V  L   VEKR+
Sbjct  85   GPGYDAGVPGHGSAEPSTSASSGSHGESDWDVVSKASGMNGDGRSEEAVKDLEQKVEKRR  144

Query  111  TLTSKMTLELQELEARLQAADER  133
            +   ++  +L ELEA+++AA+ER
Sbjct  145  SQLPRLESQLAELEAQIKAAEER  167


>gb|KIR59544.1| hypothetical protein I314_04531 [Cryptococcus gattii CA1873]
Length=191

 Score = 36.6 bits (83),  Expect = 5.7, Method: Compositional matrix adjust.
 Identities = 23/63 (37%), Positives = 37/63 (59%), Gaps = 4/63 (6%)

Query  75   SSGMRGRENF----QAQGNEEERREKEKVDRLALDVEKRKTLTSKMTLELQELEARLQAA  130
            SSG  G  ++    +A G   + R +E V  L   VEKR++   ++  +L ELEA+++AA
Sbjct  105  SSGSHGESDWDVVSKASGMNGDGRSEEAVKDLEQKVEKRRSQLPRLESQLAELEAQIKAA  164

Query  131  DER  133
            +ER
Sbjct  165  EER  167


>gb|EKD01864.1| hypothetical protein A1Q2_03927 [Trichosporon asahii var. asahii 
CBS 8904]
Length=115

 Score = 35.4 bits (80),  Expect = 6.1, Method: Compositional matrix adjust.
 Identities = 27/74 (36%), Positives = 41/74 (55%), Gaps = 6/74 (8%)

Query  66   LDSGVGSQFSSG---MRGRENFQAQG---NEEERREKEKVDRLALDVEKRKTLTSKMTLE  119
            +D+ VG   SSG    R ++ F       NEE+ +  E V  L   +EKR++L   +  E
Sbjct  25   MDAVVGGSHSSGNATSRQQQPFSDASRRVNEEQPQSAEDVQELERKLEKRRSLIPHLENE  84

Query  120  LQELEARLQAADER  133
            L  LEA+++AA+ER
Sbjct  85   LAALEAQIKAAEER  98


>gb|KIR85183.1| hypothetical protein I308_04434 [Cryptococcus gattii IND107]
Length=195

 Score = 36.2 bits (82),  Expect = 7.0, Method: Compositional matrix adjust.
 Identities = 27/83 (33%), Positives = 43/83 (52%), Gaps = 12/83 (14%)

Query  63   GDSLDSGV--------GSQFSSGMRGRENF----QAQGNEEERREKEKVDRLALDVEKRK  110
            G   D+GV         +  SSG  G  ++    +A G   + R +E V  L   VEKR+
Sbjct  89   GPGYDAGVPGHGSAEPSTSASSGSHGESDWDVISKASGMNGDGRSEEAVKDLEQKVEKRR  148

Query  111  TLTSKMTLELQELEARLQAADER  133
            +   ++  +L ELEA+++AA+ER
Sbjct  149  SQLPRLESQLAELEAQIKAAEER  171


>ref|WP_051675852.1| hypothetical protein [Polaromonas glacialis]
Length=1800

 Score = 36.6 bits (83),  Expect = 7.1, Method: Compositional matrix adjust.
 Identities = 21/66 (32%), Positives = 33/66 (50%), Gaps = 2/66 (3%)

Query  39    GSGSVLGSSLLGEEWAEFLDADTSGDSLDSGVGSQFSSGMRGRENFQAQ--GNEEERREK  96
             G+G+ L +S++G   A  L      D L+ G+G+   SG RG +N+     G+     E 
Sbjct  1565  GTGNALANSVIGNSAANTLSGGDGNDILNGGLGADVMSGGRGNDNYTVDDAGDVVSEAEN  1624

Query  97    EKVDRL  102
             E  DR+
Sbjct  1625  EGADRV  1630


>ref|XP_008106459.1| PREDICTED: angiopoietin-1 isoform X2 [Anolis carolinensis]
Length=448

 Score = 36.2 bits (82),  Expect = 9.5, Method: Composition-based stats.
 Identities = 20/52 (38%), Positives = 30/52 (58%), Gaps = 0/52 (0%)

Query  90   EEERREKEKVDRLALDVEKRKTLTSKMTLELQELEARLQAADERESSLRARQ  141
            E E R KE++D L  + E  ++L S+ T  +QELE +L  A    S L+ +Q
Sbjct  149  EMEERHKEELDTLKEEKENLQSLVSRQTYIIQELERQLTKATTNNSILQKQQ  200


>ref|WP_010180036.1| hemolysin-type calcium-binding region [Glaciecola sp. HTCC2999]
Length=10022

 Score = 36.2 bits (82),  Expect = 9.5, Method: Composition-based stats.
 Identities = 26/78 (33%), Positives = 37/78 (47%), Gaps = 2/78 (3%)

Query  5     QARYTSTPNMTSVAIPDTPDGPPMLGTIGRDTGLGSGSVLGSSLLGEEWAEFLDADTSGD  64
             QA +  +   +S  IP  P      G+ G DT  G G     ++ GEE  + LD DT  D
Sbjct  2158  QAIWIESTIDSSAVIPSGPGNDEYPGSEGDDTYNGEGG--DDTIAGEEGNDSLDGDTGND  2215

Query  65    SLDSGVGSQFSSGMRGRE  82
             S+D G G+   +G  G +
Sbjct  2216  SVDGGEGNDTVTGGAGND  2233


>ref|WP_012165873.1| hemolysin expression modulating protein [Acaryochloris marina]
 gb|ABW30658.1| Hemolysin-type calcium-binding repeat, putative [Acaryochloris 
marina MBIC11017]
Length=1054

 Score = 36.2 bits (82),  Expect = 9.8, Method: Compositional matrix adjust.
 Identities = 32/121 (26%), Positives = 55/121 (45%), Gaps = 11/121 (9%)

Query  28   MLGTIGRDT---GLGSGSVLGSS----LLGEEWAEFLDADTSGDSLDSGVGSQFSSGMRG  80
            + G  GRDT   G G+  ++G +    LLG +  ++LD    GD+L+ G G    +G  G
Sbjct  803  ITGGSGRDTLDGGKGNDHLIGDTSSDHLLGGDGNDYLDGGAYGDTLEGGDGDDHLTGGTG  862

Query  81   RENFQAQGNEEERREKEKVDRLALDVEKRKTLTSKMTLELQELEARLQAADERESSLRAR  140
            ++ F      +   E + +D    ++ K K L    T  L  +E  + +  +  + L A 
Sbjct  863  KDRFMGGAGFDTVIESDDID---FELTKNK-LIGTWTDTLDSIEHVILSGGDSNNKLDAS  918

Query  141  Q  141
            Q
Sbjct  919  Q  919


Lambda      K        H        a         alpha
   0.312    0.130    0.357    0.792     4.96 

Gapped
Lambda      K        H        a         alpha    sigma
   0.267   0.0410    0.140     1.90     42.6     43.6 

Effective search space used: 646861734144


  Database: nr
    Posted date:  Sep 23, 2015 12:05 AM
  Number of letters in database: 26,053,659,533
  Number of sequences in database:  71,551,133


Matrix: BLOSUM62
Gap Penalties: Existence: 11, Extension: 1
Neighboring words threshold: 11
Window for multiple hits: 40
```
