# Supplementary material for: Comparative genomics provides new insights into the diversity, physiology, and sexuality of the only industrially exploited tremellomycete: Phaffia rhodozyma
Source: BMC Genomics. 2016 Nov 9;17:901. doi: 10.1186/s12864-016-3244-7 (PMC5103461; doi:10.1186/s12864-016-3244-7)
Supplement: Additional file 6: — List of orphan genes with links to PFAM (related to Additional file 1: Table S1). (ZIP 1428 kb) [file 12864_2016_3244_MOESM6_ESM.zip › BLAST_HTML_FTR/G02416_P.html]

BLAST Search Results


```
BLASTP 2.2.27+


Reference:
Stephen F. Altschul, Thomas L. Madden, Alejandro A. Schäffer,
Jinghui Zhang, Zheng Zhang, Webb Miller, and David J. Lipman (1997),
"Gapped BLAST and PSI-BLAST: a new generation of protein database
search programs", Nucleic Acids Res. 25:3389-3402.


Reference for
composition-based statistics:
Alejandro A. Schäffer, L. Aravind, Thomas L. Madden, Sergei
Shavirin, John L. Spouge, Yuri I. Wolf, Eugene V. Koonin, and
Stephen F. Altschul (2001), "Improving the accuracy of PSI-BLAST
protein database searches with composition-based statistics and
other refinements", Nucleic Acids Res. 29:2994-3005.


Database: nr
           71,551,133 sequences; 26,053,659,533 total letters


Query= G02416_P

Length=365
                                                                      Score     E
Sequences producing significant alignments:                          (Bits)  Value

emb|CED84684.1|  hypothetical protein [Xanthophyllomyces dendrorh...   744    0.0  
ref|XP_003955742.1|  hypothetical protein KAFR_0B03110 [Kazachsta...  44.3    0.21 
emb|CDQ67642.1|  unnamed protein product [Oncorhynchus mykiss]        40.8    2.0  


 >emb|CED84684.1| hypothetical protein [Xanthophyllomyces dendrorhous]
Length=364

 Score =  744 bits (1920),  Expect = 0.0, Method: Compositional matrix adjust.
 Identities = 363/364 (99%), Positives = 364/364 (100%), Gaps = 0/364 (0%)

Query  1    MQPSDHTIDIPSSVSHRSFETAREGDRVQSRRYLNISRLAGRATPLESAARQDVQSSSKK  60
            MQPSDHTIDIPSSVSHRSFETAREGDRVQSRRYLNISRLAGRATPLESAARQDVQSSSKK
Sbjct  1    MQPSDHTIDIPSSVSHRSFETAREGDRVQSRRYLNISRLAGRATPLESAARQDVQSSSKK  60

Query  61   TIKRIKHNTEQTRFSFDTTPVKFHSERESFAPSLTSKQTHQSTNNFITPGTRTSINRSPL  120
            TIKRIKHNTEQTRFSFDTTPVKFHSERESFAPSLTSKQTHQSTNNFITPGTRTSINRSPL
Sbjct  61   TIKRIKHNTEQTRFSFDTTPVKFHSERESFAPSLTSKQTHQSTNNFITPGTRTSINRSPL  120

Query  121  GLAKELSIKNRSFASGRSFKNIVKEDGEDAVVSSGSEGETIRDGEDGASDGEDGEWGEEE  180
            GLAKELSIKNRSFASGRSFKN+VKEDGEDAVVSSGSEGETIRDGEDGASDGEDGEWGEEE
Sbjct  121  GLAKELSIKNRSFASGRSFKNVVKEDGEDAVVSSGSEGETIRDGEDGASDGEDGEWGEEE  180

Query  181  STMMFVETVKKGKDLVEVEESESDNNLLSQLLNADVIEDDKITANLVDLTSPTERFVKDL  240
            STMMFVETVKKGKDLVEVEESESDNNLLSQLLNADVIEDDKITANLVDLTSPTERFVKDL
Sbjct  181  STMMFVETVKKGKDLVEVEESESDNNLLSQLLNADVIEDDKITANLVDLTSPTERFVKDL  240

Query  241  WARQDRHTNSYIQNHQAITRANEGLHRDLSAQNDELMRTYITFKSQQELLVARVESAHQA  300
            WARQDRHTNSYIQNHQAITRANEGLHRDLSAQNDELMRTYITFKSQQELLVARVESAHQA
Sbjct  241  WARQDRHTNSYIQNHQAITRANEGLHRDLSAQNDELMRTYITFKSQQELLVARVESAHQA  300

Query  301  CERRKLEIIKTEENFRQETKEIFERLREEMNISIASFTSRAELTSSEKEANKEVNKMLKA  360
            CERRKLEIIKTEENFRQETKEIFERLREEMNISIASFTSRAELTSSEKEANKEVNKMLKA
Sbjct  301  CERRKLEIIKTEENFRQETKEIFERLREEMNISIASFTSRAELTSSEKEANKEVNKMLKA  360

Query  361  IIGK  364
            IIGK
Sbjct  361  IIGK  364


>ref|XP_003955742.1| hypothetical protein KAFR_0B03110 [Kazachstania africana CBS 
2517]
 emb|CCF56607.1| hypothetical protein KAFR_0B03110 [Kazachstania africana CBS 
2517]
Length=788

 Score = 44.3 bits (103),  Expect = 0.21, Method: Compositional matrix adjust.
 Identities = 42/154 (27%), Positives = 67/154 (44%), Gaps = 8/154 (5%)

Query  1    MQPSDHTIDIPSSVSHRSFETAREGDRVQSRRYLNISRLAGRATPLESAARQDVQSSSKK  60
            + P  H I+I  S     F  A+ G+ +  RR+L I  + G A  + S+    V  +S  
Sbjct  92   VDPLVHLINI--SNEKIDFYIAKIGNPLILRRFLYIFLMTGIAFFVMSSGFLPVDEASGS  149

Query  61   TIKRIKHNT--EQTRFSFDTTPVKFHSERESFAPSLTSKQTHQSTNNFITPGTRTSINRS  118
                  HN   E  R S D + ++   E  S  P ++  +   +  ++I      S+N +
Sbjct  150  RGMFTNHNVLLEYARRSIDLSKLERDLEYISSMPHMSGTKGDAAVRHYIL----ESLNNN  205

Query  119  PLGLAKELSIKNRSFASGRSFKNIVKEDGEDAVV  152
             L L KE      S   G S   I+KE+G+D V+
Sbjct  206  NLKLVKEFEYSTYSNYPGESSLTILKEEGDDVVI  239


>emb|CDQ67642.1| unnamed protein product [Oncorhynchus mykiss]
Length=480

 Score = 40.8 bits (94),  Expect = 2.0, Method: Compositional matrix adjust.
 Identities = 29/73 (40%), Positives = 42/73 (58%), Gaps = 8/73 (11%)

Query  114  SINRSPL--GLAKELS-IKNRSFASGRSFKNIVKEDGEDAVVSSGSEGETIRDGEDGASD  170
             I R+PL  GL K LS + +R    G +   ++  DGED V+  G +G  + DGEDG  D
Sbjct  325  GIARNPLHKGLMKSLSSVTSRGQERGGTEDGVM--DGEDGVMD-GEDG--VMDGEDGVMD  379

Query  171  GEDGEWGEEESTM  183
            GEDG   +++ T+
Sbjct  380  GEDGVMDDQQKTL  392


Lambda      K        H        a         alpha
   0.311    0.126    0.338    0.792     4.96 

Gapped
Lambda      K        H        a         alpha    sigma
   0.267   0.0410    0.140     1.90     42.6     43.6 

Effective search space used: 3232889998521


  Database: nr
    Posted date:  Sep 23, 2015 12:05 AM
  Number of letters in database: 26,053,659,533
  Number of sequences in database:  71,551,133


Matrix: BLOSUM62
Gap Penalties: Existence: 11, Extension: 1
Neighboring words threshold: 11
Window for multiple hits: 40
```
