# Supplementary material for: Comparative genomics provides new insights into the diversity, physiology, and sexuality of the only industrially exploited tremellomycete: Phaffia rhodozyma
Source: BMC Genomics. 2016 Nov 9;17:901. doi: 10.1186/s12864-016-3244-7 (PMC5103461; doi:10.1186/s12864-016-3244-7)
Supplement: Additional file 6: — List of orphan genes with links to PFAM (related to Additional file 1: Table S1). (ZIP 1428 kb) [file 12864_2016_3244_MOESM6_ESM.zip › BLAST_HTML_FTR/G02518_P.html]

BLAST Search Results


```
BLASTP 2.2.27+


Reference:
Stephen F. Altschul, Thomas L. Madden, Alejandro A. Schäffer,
Jinghui Zhang, Zheng Zhang, Webb Miller, and David J. Lipman (1997),
"Gapped BLAST and PSI-BLAST: a new generation of protein database
search programs", Nucleic Acids Res. 25:3389-3402.


Reference for
composition-based statistics:
Alejandro A. Schäffer, L. Aravind, Thomas L. Madden, Sergei
Shavirin, John L. Spouge, Yuri I. Wolf, Eugene V. Koonin, and
Stephen F. Altschul (2001), "Improving the accuracy of PSI-BLAST
protein database searches with composition-based statistics and
other refinements", Nucleic Acids Res. 29:2994-3005.


Database: nr
           71,551,133 sequences; 26,053,659,533 total letters


Query= G02518_P

Length=101
                                                                      Score     E
Sequences producing significant alignments:                          (Bits)  Value

emb|CED82328.1|  hypothetical protein [Xanthophyllomyces dendrorh...   197    8e-63
gb|EAZ07838.1|  hypothetical protein OsI_30096 [Oryza sativa Indi...  43.5    0.014
ref|XP_001745963.1|  hypothetical protein [Monosiga brevicollis M...  36.6    2.4  
ref|WP_035339642.1|  thiol:disulfide interchange protein precurso...  35.8    5.0  


 >emb|CED82328.1| hypothetical protein [Xanthophyllomyces dendrorhous]
Length=100

 Score =  197 bits (501),  Expect = 8e-63, Method: Compositional matrix adjust.
 Identities = 100/100 (100%), Positives = 100/100 (100%), Gaps = 0/100 (0%)

Query  1   MSDRPTNRLRNPFRSSDGQAILVSGFLLSARTTPHSTRAFDPSSLPVTNSPPPTSSPGSP  60
           MSDRPTNRLRNPFRSSDGQAILVSGFLLSARTTPHSTRAFDPSSLPVTNSPPPTSSPGSP
Sbjct  1   MSDRPTNRLRNPFRSSDGQAILVSGFLLSARTTPHSTRAFDPSSLPVTNSPPPTSSPGSP  60

Query  61  QHSSANRVRPRSGSEDTPSNLFTLKEEDEPTKTHCRKALR  100
           QHSSANRVRPRSGSEDTPSNLFTLKEEDEPTKTHCRKALR
Sbjct  61  QHSSANRVRPRSGSEDTPSNLFTLKEEDEPTKTHCRKALR  100


>gb|EAZ07838.1| hypothetical protein OsI_30096 [Oryza sativa Indica Group]
Length=610

 Score = 43.5 bits (101),  Expect = 0.014, Method: Composition-based stats.
 Identities = 23/59 (39%), Positives = 31/59 (53%), Gaps = 0/59 (0%)

Query  36   STRAFDPSSLPVTNSPPPTSSPGSPQHSSANRVRPRSGSEDTPSNLFTLKEEDEPTKTH  94
            ST+  D S L V ++ PP+ S  SP H S    +P +G EDT S+   L  E E  + H
Sbjct  526  STQYIDESPLHVYSARPPSGSSRSPLHDSLGGSKPNAGKEDTLSSSLGLNAEQEDAEVH  584


>ref|XP_001745963.1| hypothetical protein [Monosiga brevicollis MX1]
 gb|EDQ89387.1| predicted protein [Monosiga brevicollis MX1]
Length=1328

 Score = 36.6 bits (83),  Expect = 2.4, Method: Compositional matrix adjust.
 Identities = 25/70 (36%), Positives = 32/70 (46%), Gaps = 6/70 (9%)

Query  31   RTTPHSTRAFDPSSLPVTNSPPPTSSPGSPQHSSAN------RVRPRSGSEDTPSNLFTL  84
             T P+ T A D  +L +TNS  PT+  GS   +S N       V P  GS + P  L   
Sbjct  415  ETQPNITTAADYVTLTITNSLVPTTVVGSDNCTSTNVETTCHWVLPHIGSSNPPQQLSVQ  474

Query  85   KEEDEPTKTH  94
              ED+P   H
Sbjct  475  YTEDQPVSMH  484


>ref|WP_035339642.1| thiol:disulfide interchange protein precursor [Dickeya sp. DW 
0440]
Length=580

 Score = 35.8 bits (81),  Expect = 5.0, Method: Composition-based stats.
 Identities = 18/37 (49%), Positives = 20/37 (54%), Gaps = 1/37 (3%)

Query  34   PHSTRAFDPSSLPVTNSPPPTSSPGSPQHSSANRVRP  70
            P  TR   P S   TN PPPT +   P HSSA  V+P
Sbjct  135  PPETREL-PLSATTTNVPPPTQATARPPHSSATAVQP  170


Lambda      K        H        a         alpha
   0.316    0.129    0.385    0.792     4.96 

Gapped
Lambda      K        H        a         alpha    sigma
   0.267   0.0410    0.140     1.90     42.6     43.6 

Effective search space used: 652397486913


  Database: nr
    Posted date:  Sep 23, 2015 12:05 AM
  Number of letters in database: 26,053,659,533
  Number of sequences in database:  71,551,133


Matrix: BLOSUM62
Gap Penalties: Existence: 11, Extension: 1
Neighboring words threshold: 11
Window for multiple hits: 40
```
