# Supplementary material for: Comparative genomics provides new insights into the diversity, physiology, and sexuality of the only industrially exploited tremellomycete: Phaffia rhodozyma
Source: BMC Genomics. 2016 Nov 9;17:901. doi: 10.1186/s12864-016-3244-7 (PMC5103461; doi:10.1186/s12864-016-3244-7)
Supplement: Additional file 6: — List of orphan genes with links to PFAM (related to Additional file 1: Table S1). (ZIP 1428 kb) [file 12864_2016_3244_MOESM6_ESM.zip › BLAST_HTML_FTR/G00209_P.html]

BLAST Search Results


```
BLASTP 2.2.27+


Reference:
Stephen F. Altschul, Thomas L. Madden, Alejandro A. Schäffer,
Jinghui Zhang, Zheng Zhang, Webb Miller, and David J. Lipman (1997),
"Gapped BLAST and PSI-BLAST: a new generation of protein database
search programs", Nucleic Acids Res. 25:3389-3402.


Reference for
composition-based statistics:
Alejandro A. Schäffer, L. Aravind, Thomas L. Madden, Sergei
Shavirin, John L. Spouge, Yuri I. Wolf, Eugene V. Koonin, and
Stephen F. Altschul (2001), "Improving the accuracy of PSI-BLAST
protein database searches with composition-based statistics and
other refinements", Nucleic Acids Res. 29:2994-3005.


Database: nr
           71,551,133 sequences; 26,053,659,533 total letters


Query= G00209_P

Length=590
                                                                      Score     E
Sequences producing significant alignments:                          (Bits)  Value

emb|CED84060.1|  hypothetical protein [Xanthophyllomyces dendrorh...  1181    0.0  
gb|AFJ73495.1|  pyruvate kinase, partial [Neocallimastix frontalis]   43.9    0.47 


 >emb|CED84060.1| hypothetical protein [Xanthophyllomyces dendrorhous]
Length=618

 Score = 1181 bits (3055),  Expect = 0.0, Method: Compositional matrix adjust.
 Identities = 585/618 (95%), Positives = 586/618 (95%), Gaps = 31/618 (5%)

Query  3    MLSASSRTSAEKQSDAVLPTLTSPKKEHSEAIKPTGENDSQSWPVENAPVESENKHIEKR  62
            MLSASSRTSAEKQSDAVLPTLTSPKKEHSEAIKPTGENDSQSWPVENAPVESENKHIEKR
Sbjct  1    MLSASSRTSAEKQSDAVLPTLTSPKKEHSEAIKPTGENDSQSWPVENAPVESENKHIEKR  60

Query  63   SFFGRSSSHQKKDSLSTPAIIMRFAHSSTPTDGSLRIRVFMDHPTAEPLYVNIIPGVDSL  122
            SFFGRSSSHQKK+SLSTPAIIMRFAHSSTPTDGSLRIRVFMDHPTAEPLYVNIIPGVDSL
Sbjct  61   SFFGRSSSHQKKESLSTPAIIMRFAHSSTPTDGSLRIRVFMDHPTAEPLYVNIIPGVDSL  120

Query  123  HTLKRKILTEDNTRFLATRQNRKSPIEVKWDIPEFLEEEPTEDHYDFDNRLDVYKIS---  179
            HTLKRKILTEDNTRFLATRQNRKSPIEVKWDIPEFLEEEPTEDHYDFDNRLDVYKIS   
Sbjct  121  HTLKRKILTEDNTRFLATRQNRKSPIEVKWDIPEFLEEEPTEDHYDFDNRLDVYKISCSW  180

Query  180  ----------------------------FAQSDQDAKVPLDLGSIFPPDTPEMVEARAKG  211
                                         AQSDQDAKVPLDLGSIFPPDTPEMVEARAKG
Sbjct  181  IHISKMKRTYDHTKVFHSILSSYPSNLIVAQSDQDAKVPLDLGSIFPPDTPEMVEARAKG  240

Query  212  LTELAIRIKPQKSWPILVAFSDVPTHPFVVEYQRGMRVRDLKVIIGEARTNLIIIMGDAS  271
            LTELAIRIKPQKSWPILVAFSDVPTHPFVVEYQRGMRVRDLKVIIGEARTNLIIIMGDAS
Sbjct  241  LTELAIRIKPQKSWPILVAFSDVPTHPFVVEYQRGMRVRDLKVIIGEARTNLIIIMGDAS  300

Query  272  TTTLDGSEFFLFKCAQPGKGTSPRTFPNQIDRYRGLTPATLFDDEEVEFASHRFMRSFRP  331
            TTTLDGSEFFLFKCAQPGKGTSPRTFPNQIDRYRGLTPATLFDDEEVEFASHRFMRSFRP
Sbjct  301  TTTLDGSEFFLFKCAQPGKGTSPRTFPNQIDRYRGLTPATLFDDEEVEFASHRFMRSFRP  360

Query  332  KRGNNPLEKVELVAFMVDDMEEDLLNTLSVNSKAPPRSILSSPAEQYAGLHSIQTPDGSK  391
            KRGNNPLEKVELVAFMVDDMEEDLLNTLSVNSKAPPRSILSSPAEQYAGLHSIQTPDGSK
Sbjct  361  KRGNNPLEKVELVAFMVDDMEEDLLNTLSVNSKAPPRSILSSPAEQYAGLHSIQTPDGSK  420

Query  392  FGSSRSRLSYSEASQSYLTQPIRRSSAAVLPVSRTDSMAVHRESCQPSLQTSATSSPKTA  451
            FGSSRSRLSYSEASQSYLTQPIRRSSAAVLPVSRTDSMAVHRESCQPSLQTSATSSPKTA
Sbjct  421  FGSSRSRLSYSEASQSYLTQPIRRSSAAVLPVSRTDSMAVHRESCQPSLQTSATSSPKTA  480

Query  452  PLSIVSTNMAKKQNKGPRVIESEEFLETESPLAISDPAELSDSDMSGSNLDILKTPPIAL  511
            PLSIVSTNMAKKQNKGPRVIESEEFLETESPLAISDPAELSDSDMSGSNLDILKTPPIAL
Sbjct  481  PLSIVSTNMAKKQNKGPRVIESEEFLETESPLAISDPAELSDSDMSGSNLDILKTPPIAL  540

Query  512  TAANDHGLRAESGSDEKDKADEEDERTMVNGYALIEGALFSLSLKPIDRKGEKLVKNNHK  571
            TAANDHGLRAESGSDEKDKADEEDERTMVNGYALIEGALFSLSLKPIDRKGEKLVKNNHK
Sbjct  541  TAANDHGLRAESGSDEKDKADEEDERTMVNGYALIEGALFSLSLKPIDRKGEKLVKNNHK  600

Query  572  GMFSQEEPLFSVTPVHVG  589
            GMFSQEEPLFSVTPVHVG
Sbjct  601  GMFSQEEPLFSVTPVHVG  618


>gb|AFJ73495.1| pyruvate kinase, partial [Neocallimastix frontalis]
Length=545

 Score = 43.9 bits (102),  Expect = 0.47, Method: Compositional matrix adjust.
 Identities = 46/184 (25%), Positives = 80/184 (43%), Gaps = 35/184 (19%)

Query  16   SDAVLPTLTSPKKEHSEAIKPTGENDSQSWPVENAPVESENKHIEKRSFFGRSSSHQKKD  75
            S A+L  L  PK      I+  G  D +S P+E    + ++ +++  S  G  ++     
Sbjct  34   SVAILGDLCGPK------IRNNGFKDRESIPLE----KGQHLYLKASSELGDENTI----  79

Query  76   SLSTPAIIMRFA--HSSTPTDGSLRIRVFMDHPTAEPLYVNIIPGVDSLHTLKRKILTED  133
             ++TP II +    H     DG++ +RV +   T + L V ++ G +             
Sbjct  80   CMNTPQIIKQLQPEHRVLLDDGNISLRV-IKRITEDTLEVEVLSGTE-------------  125

Query  134  NTRFLATRQNRKSPIEVKWDIPEFLEEEPTEDHYDFDNRLDVYKISFAQSDQDAKVPLDL  193
                L  R+    P ++  DIP   E++  +  + +D RLD   +SF Q  QD +  +DL
Sbjct  126  ----LKARKGINVP-DLLVDIPALTEKDKRDAKFIWDQRLDFIALSFVQRRQDVQDLIDL  180

Query  194  GSIF  197
               F
Sbjct  181  MGEF  184


Lambda      K        H        a         alpha
   0.313    0.130    0.366    0.792     4.96 

Gapped
Lambda      K        H        a         alpha    sigma
   0.267   0.0410    0.140     1.90     42.6     43.6 

Effective search space used: 6417117005316


  Database: nr
    Posted date:  Sep 23, 2015 12:05 AM
  Number of letters in database: 26,053,659,533
  Number of sequences in database:  71,551,133


Matrix: BLOSUM62
Gap Penalties: Existence: 11, Extension: 1
Neighboring words threshold: 11
Window for multiple hits: 40
```
