# Supplementary material for: Comparative genomics provides new insights into the diversity, physiology, and sexuality of the only industrially exploited tremellomycete: Phaffia rhodozyma
Source: BMC Genomics. 2016 Nov 9;17:901. doi: 10.1186/s12864-016-3244-7 (PMC5103461; doi:10.1186/s12864-016-3244-7)
Supplement: Additional file 6: — List of orphan genes with links to PFAM (related to Additional file 1: Table S1). (ZIP 1428 kb) [file 12864_2016_3244_MOESM6_ESM.zip › BLAST_HTML_FTR/G05663_P.html]

BLAST Search Results


```
BLASTP 2.2.27+


Reference:
Stephen F. Altschul, Thomas L. Madden, Alejandro A. Schäffer,
Jinghui Zhang, Zheng Zhang, Webb Miller, and David J. Lipman (1997),
"Gapped BLAST and PSI-BLAST: a new generation of protein database
search programs", Nucleic Acids Res. 25:3389-3402.


Reference for
composition-based statistics:
Alejandro A. Schäffer, L. Aravind, Thomas L. Madden, Sergei
Shavirin, John L. Spouge, Yuri I. Wolf, Eugene V. Koonin, and
Stephen F. Altschul (2001), "Improving the accuracy of PSI-BLAST
protein database searches with composition-based statistics and
other refinements", Nucleic Acids Res. 29:2994-3005.


Database: nr
           71,551,133 sequences; 26,053,659,533 total letters


Query= G05663_P

Length=129
                                                                      Score     E
Sequences producing significant alignments:                          (Bits)  Value

emb|CDZ97488.1|  isocitrate dehydrogenase [Xanthophyllomyces dend...   124    1e-29
emb|CDZ97809.1|  Transposase IS605, OrfB, C-terminal [Xanthophyll...  74.3    1e-12
ref|WP_047187460.1|  short-chain dehydrogenase [Microvirga vignae...  39.7    0.32 
ref|WP_046740468.1|  short-chain dehydrogenase [Lampropedia sp. C...  39.3    0.44 
ref|WP_052455088.1|  short-chain dehydrogenase, partial [Klebsiel...  39.3    0.46 
ref|XP_005996586.1|  PREDICTED: asparagine synthetase domain-cont...  39.3    0.58 
ref|WP_047366121.1|  MULTISPECIES: short-chain dehydrogenase [Ent...  38.9    0.65 
ref|WP_040018942.1|  short-chain dehydrogenase [Enterobacter ludw...  38.9    0.65 
ref|WP_032678818.1|  short-chain dehydrogenase [Enterobacter sp. ...  38.9    0.66 
ref|WP_014171297.1|  MULTISPECIES: short-chain dehydrogenase [Ent...  38.9    0.66 
ref|XP_005996589.1|  PREDICTED: asparagine synthetase domain-cont...  38.9    0.67 
ref|WP_047742224.1|  short-chain dehydrogenase [Enterobacter cloa...  38.5    0.68 
ref|WP_044858643.1|  short-chain dehydrogenase [Enterobacter cloa...  38.5    0.68 
ref|WP_032680432.1|  MULTISPECIES: short-chain dehydrogenase [Ent...  38.5    0.68 
ref|WP_048267682.1|  short-chain dehydrogenase [Klebsiella pneumo...  38.5    0.75 
ref|WP_032455213.1|  MULTISPECIES: short-chain dehydrogenase [Kle...  38.5    0.75 
ref|WP_044521813.1|  MULTISPECIES: short-chain dehydrogenase [Kle...  38.5    0.77 
ref|WP_023288935.1|  short-chain dehydrogenase/reductase SDR [Kle...  38.5    0.77 
ref|WP_040168882.1|  short-chain dehydrogenase [Klebsiella pneumo...  38.5    0.84 
ref|WP_003862216.1|  MULTISPECIES: short-chain dehydrogenase [Ent...  38.1    1.0  
ref|WP_032609987.1|  MULTISPECIES: short-chain dehydrogenase [Ent...  38.1    1.0  
ref|WP_023620857.1|  MULTISPECIES: short-chain dehydrogenase [Ent...  38.1    1.0  
ref|WP_032660057.1|  short-chain dehydrogenase [Enterobacter cloa...  38.1    1.1  
ref|WP_050860982.1|  short-chain dehydrogenase [Enterobacter cloa...  38.1    1.1  
ref|WP_038980951.1|  short-chain dehydrogenase [Enterobacter cloa...  38.1    1.1  
ref|WP_036364567.1|  short-chain dehydrogenase [Microvirga lupini]    38.1    1.1  
ref|WP_014832991.1|  MULTISPECIES: short-chain dehydrogenase [Ent...  38.1    1.1  
ref|WP_049136890.1|  short-chain dehydrogenase [Enterobacter cloa...  38.1    1.1  
ref|WP_038857766.1|  short-chain dehydrogenase [Cronobacter unive...  38.1    1.1  
ref|WP_019846055.1|  short-chain dehydrogenase [Dickeya zeae]         38.1    1.1  
ref|WP_046888505.1|  short-chain dehydrogenase [Enterobacter cloa...  38.1    1.1  
ref|WP_028014159.1|  short-chain dehydrogenase [Enterobacter cloa...  38.1    1.1  
ref|WP_023639989.1|  short-chain dehydrogenase [Dickeya zeae]         38.1    1.1  
ref|WP_045346168.1|  short-chain dehydrogenase [Enterobacter cloa...  38.1    1.2  
ref|WP_013317389.1|  short-chain dehydrogenase [Dickeya dadantii]...  38.1    1.2  
ref|WP_048210587.1|  short-chain dehydrogenase [Enterobacter cloa...  37.7    1.5  
ref|WP_013098400.1|  short-chain dehydrogenase [Enterobacter cloa...  37.7    1.7  
ref|WP_038420466.1|  short-chain dehydrogenase [Enterobacter cloa...  37.4    1.7  
ref|WP_048970757.1|  short-chain dehydrogenase [Enterobacter cloa...  37.4    1.7  
ref|WP_023284879.1|  short-chain dehydrogenase/reductase SDR [Kle...  37.4    1.7  
gb|AGT25156.1|  putative short-chain dehydrogenase/reductase SDR ...  37.4    1.7  
ref|WP_032613441.1|  short-chain dehydrogenase [Leclercia adecarb...  37.4    1.7  
ref|WP_049087277.1|  MULTISPECIES: short-chain dehydrogenase [Ent...  37.4    1.8  
ref|WP_040174808.1|  short-chain dehydrogenase [Klebsiella pneumo...  37.4    1.8  
ref|WP_024360134.1|  MULTISPECIES: short-chain dehydrogenase [Kle...  37.4    1.8  
ref|WP_049182433.1|  short-chain dehydrogenase [Klebsiella pneumo...  37.4    1.9  
ref|WP_032104939.1|  short-chain dehydrogenase [Klebsiella pneumo...  37.4    1.9  
ref|WP_004130709.1|  short-chain dehydrogenase [Klebsiella oxytoc...  37.4    1.9  
ref|WP_040222529.1|  short-chain dehydrogenase [Klebsiella pneumo...  37.4    1.9  
ref|WP_032445768.1|  MULTISPECIES: short-chain dehydrogenase [Ent...  37.4    1.9  
ref|WP_004223790.1|  short-chain dehydrogenase [Klebsiella pneumo...  37.4    1.9  
dbj|BAS36430.1|  3-oxoacyl-ACP reductase [Klebsiella pneumoniae]      37.4    1.9  
ref|WP_048254336.1|  MULTISPECIES: short-chain dehydrogenase [Ent...  37.4    1.9  
ref|WP_012542384.1|  short-chain dehydrogenase [Klebsiella pneumo...  37.4    1.9  
ref|WP_023341222.1|  short-chain dehydrogenase/reductase SDR [Kle...  37.4    1.9  
ref|WP_012068480.1|  short-chain dehydrogenase [Klebsiella pneumo...  37.4    1.9  
ref|WP_048333020.1|  short-chain dehydrogenase [Klebsiella pneumo...  37.4    1.9  
ref|WP_008805763.1|  MULTISPECIES: short-chain dehydrogenase [Ent...  37.4    1.9  
ref|WP_023287402.1|  short-chain dehydrogenase/reductase SDR [Kle...  37.4    1.9  
ref|WP_017900162.1|  MULTISPECIES: short-chain dehydrogenase [Kle...  37.4    1.9  
ref|WP_004886175.1|  MULTISPECIES: 3-oxoacyl-ACP reductase [Enter...  37.4    1.9  
ref|WP_048333495.1|  short-chain dehydrogenase [Klebsiella pneumo...  37.4    2.0  
ref|WP_048268970.1|  short-chain dehydrogenase [Klebsiella pneumo...  37.4    2.0  
ref|WP_045343778.1|  short-chain dehydrogenase [Enterobacter cloa...  37.4    2.0  
ref|WP_043875587.1|  short-chain dehydrogenase [Klebsiella variic...  37.4    2.0  
ref|WP_004135219.1|  MULTISPECIES: putative short-chain dehydroge...  37.4    2.0  
ref|WP_002895750.1|  MULTISPECIES: short-chain dehydrogenase [Ent...  37.4    2.0  
ref|WP_044243604.1|  short-chain dehydrogenase, partial [Klebsiel...  37.4    2.0  
ref|WP_049113343.1|  short-chain dehydrogenase [Klebsiella oxytoca]   37.4    2.0  
ref|WP_048974553.1|  short-chain dehydrogenase [Klebsiella pneumo...  37.4    2.0  
ref|WP_038431907.1|  short-chain dehydrogenase [Klebsiella pneumo...  37.4    2.0  
ref|WP_032411091.1|  short-chain dehydrogenase [Klebsiella pneumo...  37.4    2.0  
ref|WP_016160530.1|  MULTISPECIES: hypothetical protein [Enteroba...  37.4    2.0  
ref|WP_022065514.1|  MULTISPECIES: short-chain dehydrogenase/redu...  37.4    2.0  
gb|ESL29483.1|  short-chain dehydrogenase/reductase SDR [Klebsiel...  37.4    2.0  
emb|CTQ17466.1|  Uncharacterized short-chain type dehydrogenase/r...  37.4    2.0  
ref|WP_040973985.1|  MULTISPECIES: short-chain dehydrogenase [Kle...  37.4    2.0  
ref|WP_032735010.1|  MULTISPECIES: short-chain dehydrogenase [Kle...  37.4    2.0  
ref|WP_048273490.1|  short-chain dehydrogenase [Klebsiella pneumo...  37.4    2.1  
ref|WP_023300845.1|  short-chain dehydrogenase/reductase SDR [Kle...  37.4    2.1  
gb|AIE02442.1|  short-chain dehydrogenase [Klebsiella pneumoniae ...  37.4    2.2  
ref|WP_053194915.1|  short-chain dehydrogenase [Herbaspirillum hi...  37.0    3.1  
ref|WP_007883302.1|  short-chain dehydrogenase [Herbaspirillum sp...  37.0    3.1  
ref|WP_050463195.1|  short-chain dehydrogenase [Herbaspirillum au...  36.6    3.2  
ref|WP_008495475.1|  short-chain dehydrogenase [Acidocella sp. MX...  36.6    3.4  
ref|WP_026439851.1|  short-chain dehydrogenase [Acidocella facilis]   36.6    3.4  
ref|WP_048990457.1|  short-chain dehydrogenase [Klebsiella pneumo...  36.6    3.9  
ref|WP_013973349.1|  MULTISPECIES: short-chain dehydrogenase [Pse...  36.6    3.9  
gb|EJT84616.1|  putative short-chain type regulator [Pseudomonas ...  36.6    4.0  
ref|WP_010799082.1|  hypothetical protein [Pseudomonas sp. HPB007...  36.2    5.0  
ref|WP_019364260.1|  short-chain dehydrogenase [Pseudomonas luteola]  36.2    5.2  
ref|WP_050467269.1|  short-chain dehydrogenase [Herbaspirillum ch...  36.2    5.3  
ref|WP_019437635.1|  3-ketoacyl-ACP reductase [Pseudomonas putida...  36.2    5.5  
ref|WP_014589181.1|  short-chain dehydrogenase [Pseudomonas putid...  36.2    5.6  
ref|WP_044183479.1|  short-chain dehydrogenase [Enterobacter mass...  36.2    5.7  
ref|WP_014754902.1|  short-chain dehydrogenase [Pseudomonas putid...  36.2    5.8  
ref|WP_012054137.1|  short-chain dehydrogenase [Pseudomonas putid...  35.8    6.1  
ref|WP_019752827.1|  3-ketoacyl-ACP reductase [Pseudomonas putida]    35.8    6.1  
ref|WP_005735172.1|  short-chain dehydrogenase [Pseudomonas amygd...  35.4    8.5  
ref|WP_012319952.1|  MULTISPECIES: short-chain dehydrogenase [Bac...  35.4    8.6  
ref|WP_019649740.1|  short-chain dehydrogenase [Pseudomonas sp. 4...  35.4    9.1  
ref|WP_045792147.1|  short-chain dehydrogenase [Pseudomonas fluor...  35.4    9.6  


 >emb|CDZ97488.1| isocitrate dehydrogenase [Xanthophyllomyces dendrorhous]
Length=1099

 Score =  124 bits (310),  Expect = 1e-29, Method: Compositional matrix adjust.
 Identities = 71/112 (63%), Positives = 76/112 (68%), Gaps = 20/112 (18%)

Query  3    SALLSSRTFSPTLRSRRSSPLEGGDVFAGAHTSAASKHAVCQAEGLSAAPGPQSVRVSDV  62
            SALLSSRT SPT  SR SS L+ GDVFAGAHTSAA K                S  VSDV
Sbjct  553  SALLSSRTLSPTPGSRPSSLLDAGDVFAGAHTSAAIKRTT-------------SAGVSDV  599

Query  63   EGVLQVPWRVLWKTQSTWWNRDVNASRNMLHLALHQEKNKGARMPGFDAVAS  114
            EGVL+VPWR       TWWNRDVNASRNMLHLALHQE+N+GARM  FD  A+
Sbjct  600  EGVLEVPWR-------TWWNRDVNASRNMLHLALHQEENEGARMLVFDTAAA  644


>emb|CDZ97809.1| Transposase IS605, OrfB, C-terminal [Xanthophyllomyces dendrorhous]
Length=634

 Score = 74.3 bits (181),  Expect = 1e-12, Method: Compositional matrix adjust.
 Identities = 35/47 (74%), Positives = 39/47 (83%), Gaps = 1/47 (2%)

Query  79   TWWNRDVNASRNMLHLALHQEKNKGARMPGFDAVASD-VETAEAGTE  124
            TWWNRD+NASRNMLHLALHQE N+GARM GFD  A+  V  AEAGT+
Sbjct  588  TWWNRDINASRNMLHLALHQEMNEGARMLGFDITATTLVAEAEAGTD  634


>ref|WP_047187460.1| short-chain dehydrogenase [Microvirga vignae]
 gb|KLK94515.1| short-chain dehydrogenase [Microvirga vignae]
Length=249

 Score = 39.7 bits (91),  Expect = 0.32, Method: Compositional matrix adjust.
 Identities = 25/77 (32%), Positives = 38/77 (49%), Gaps = 10/77 (13%)

Query  1    MVSALLSSRTFSPTLRSRRSSPL---------EGGDVFAGAHTSAASKHAVCQAEGLSAA  51
            +  AL  S+ F P +RSRR   +          GG +F G H SAA    +  A+ ++  
Sbjct  113  LTGALYLSQAFIPHMRSRRRGSIACMSSVSAQRGGGIFGGPHYSAAKAGVLGLAKAMARE  172

Query  52   PGPQSVRVSDVE-GVLQ  67
             GP  +RV+ V  G++Q
Sbjct  173  LGPDGIRVNCVTPGLIQ  189


>ref|WP_046740468.1| short-chain dehydrogenase [Lampropedia sp. CT6]
 gb|KKW69033.1| short-chain dehydrogenase [Lampropedia sp. CT6]
Length=251

 Score = 39.3 bits (90),  Expect = 0.44, Method: Compositional matrix adjust.
 Identities = 25/73 (34%), Positives = 38/73 (52%), Gaps = 10/73 (14%)

Query  5    LLSSRTFSPTLRSRRSSPL---------EGGDVFAGAHTSAASKHAVCQAEGLSAAPGPQ  55
            L  S+ F PT R++RS  +          GG +F G H SAA    +  A+ ++   GP 
Sbjct  118  LYLSQAFIPTFRAQRSGAIACMSSVSAQRGGGIFGGPHYSAAKAGVLGLAKAMARELGPD  177

Query  56   SVRVSDVE-GVLQ  67
            +VRV+ V  G++Q
Sbjct  178  NVRVNCVTPGLIQ  190


>ref|WP_052455088.1| short-chain dehydrogenase, partial [Klebsiella pneumoniae]
Length=226

 Score = 39.3 bits (90),  Expect = 0.46, Method: Compositional matrix adjust.
 Identities = 35/121 (29%), Positives = 53/121 (44%), Gaps = 21/121 (17%)

Query  5    LLSSRTFSPTLRSRRSSPL---------EGGDVFAGAHTSAASKHAVCQAEGLSAAPGPQ  55
            LL S+   PT+R+++S  +          GG +F G H SAA    +  A  ++   GP 
Sbjct  117  LLMSQAVIPTMRAQKSGSIVCISSVSAQRGGGIFGGPHYSAAKAGVLGLARAMARELGPD  176

Query  56   SVRVSDVEGVLQVPWRVLWKTQSTWWNRDVNASRNMLHLALHQEKNKGARMPGFDAVASD  115
            +VRV+ +      P   L +T  T      + + N+L        N G   P  DAV +D
Sbjct  177  NVRVNCIT-----PG--LIQTDITAGKLTDDMTANIL-----AGGNVGLNQPRGDAVNAD  224

Query  116  V  116
            V
Sbjct  225  V  225


>ref|XP_005996586.1| PREDICTED: asparagine synthetase domain-containing protein 1 
isoform X1 [Latimeria chalumnae]
 ref|XP_005996587.1| PREDICTED: asparagine synthetase domain-containing protein 1 
isoform X2 [Latimeria chalumnae]
 ref|XP_005996588.1| PREDICTED: asparagine synthetase domain-containing protein 1 
isoform X3 [Latimeria chalumnae]
Length=630

 Score = 39.3 bits (90),  Expect = 0.58, Method: Compositional matrix adjust.
 Identities = 27/81 (33%), Positives = 39/81 (48%), Gaps = 10/81 (12%)

Query  26   GDVFAGAHTSAASKHAVCQAEGLSAAPGPQSVR--VSDVEGVLQVPWRVLW---KTQSTW  80
            G+VFAG    AA    +   E LS+  G Q +   +S V+G    PW  ++    T S W
Sbjct  80   GEVFAGIDVGAAESDTLVMFEHLSSCDGEQEILSVLSSVQG----PWAFIYYQASTHSLW  135

Query  81   WNRDVNASRNML-HLALHQEK  100
            + RD    R++L H +   EK
Sbjct  136  FGRDFFGRRSLLWHFSSGTEK  156


>ref|WP_047366121.1| MULTISPECIES: short-chain dehydrogenase [Enterobacter]
 gb|KLR48548.1| short-chain dehydrogenase [Enterobacter sp. GN02226]
Length=249

 Score = 38.9 bits (89),  Expect = 0.65, Method: Compositional matrix adjust.
 Identities = 24/73 (33%), Positives = 39/73 (53%), Gaps = 10/73 (14%)

Query  5    LLSSRTFSPTLRSRRSSPL---------EGGDVFAGAHTSAASKHAVCQAEGLSAAPGPQ  55
            LL S+   PTLR+++S  +          GG +F G H SAA    +  A+ ++   GP 
Sbjct  117  LLMSQAVIPTLRAQKSGSIVCISSVSAQRGGGIFGGPHYSAAKAGVLGLAKAMARELGPD  176

Query  56   SVRVSDVE-GVLQ  67
            +VRV+ +  G++Q
Sbjct  177  NVRVNCITPGLIQ  189


>ref|WP_040018942.1| short-chain dehydrogenase [Enterobacter ludwigii]
 gb|KIF88426.1| short-chain dehydrogenase [Enterobacter ludwigii]
Length=249

 Score = 38.9 bits (89),  Expect = 0.65, Method: Compositional matrix adjust.
 Identities = 24/73 (33%), Positives = 39/73 (53%), Gaps = 10/73 (14%)

Query  5    LLSSRTFSPTLRSRRSSPL---------EGGDVFAGAHTSAASKHAVCQAEGLSAAPGPQ  55
            LL S+   PTLR+++S  +          GG +F G H SAA    +  A+ ++   GP 
Sbjct  117  LLMSQAVIPTLRAQKSGSIVCISSVSAQRGGGIFGGPHYSAAKAGVLGLAKAMARELGPD  176

Query  56   SVRVSDVE-GVLQ  67
            +VRV+ +  G++Q
Sbjct  177  NVRVNCITPGLIQ  189


>ref|WP_032678818.1| short-chain dehydrogenase [Enterobacter sp. BIDMC 26]
 gb|EUM27107.1| short-chain dehydrogenase/reductase SDR [Enterobacter sp. BIDMC 
26]
Length=249

 Score = 38.9 bits (89),  Expect = 0.66, Method: Compositional matrix adjust.
 Identities = 24/73 (33%), Positives = 39/73 (53%), Gaps = 10/73 (14%)

Query  5    LLSSRTFSPTLRSRRSSPL---------EGGDVFAGAHTSAASKHAVCQAEGLSAAPGPQ  55
            LL S+   PTLR+++S  +          GG +F G H SAA    +  A+ ++   GP 
Sbjct  117  LLMSQAVIPTLRAQKSGSIVCISSVSAQRGGGIFGGPHYSAAKAGVLGLAKAMARELGPD  176

Query  56   SVRVSDVE-GVLQ  67
            +VRV+ +  G++Q
Sbjct  177  NVRVNCITPGLIQ  189


>ref|WP_014171297.1| MULTISPECIES: short-chain dehydrogenase [Enterobacter]
 gb|AEW74897.1| FabG [Enterobacter cloacae EcWSU1]
 gb|EPR36951.1| 3-oxoacyl-(acyl-carrier-protein) reductase [Enterobacter cloacae 
str. Hanford]
 gb|AHE69557.1| 3-ketoacyl-ACP reductase [Enterobacter cloacae P101]
 gb|KLP36966.1| short-chain dehydrogenase [Enterobacter sp. GN02730]
Length=249

 Score = 38.9 bits (89),  Expect = 0.66, Method: Compositional matrix adjust.
 Identities = 24/73 (33%), Positives = 39/73 (53%), Gaps = 10/73 (14%)

Query  5    LLSSRTFSPTLRSRRSSPL---------EGGDVFAGAHTSAASKHAVCQAEGLSAAPGPQ  55
            LL S+   PTLR+++S  +          GG +F G H SAA    +  A+ ++   GP 
Sbjct  117  LLMSQAVIPTLRAQKSGSIVCISSVSAQRGGGIFGGPHYSAAKAGVLGLAKAMARELGPD  176

Query  56   SVRVSDVE-GVLQ  67
            +VRV+ +  G++Q
Sbjct  177  NVRVNCITPGLIQ  189


>ref|XP_005996589.1| PREDICTED: asparagine synthetase domain-containing protein 1 
isoform X4 [Latimeria chalumnae]
Length=531

 Score = 38.9 bits (89),  Expect = 0.67, Method: Compositional matrix adjust.
 Identities = 27/81 (33%), Positives = 39/81 (48%), Gaps = 10/81 (12%)

Query  26   GDVFAGAHTSAASKHAVCQAEGLSAAPGPQSVR--VSDVEGVLQVPWRVLW---KTQSTW  80
            G+VFAG    AA    +   E LS+  G Q +   +S V+G    PW  ++    T S W
Sbjct  80   GEVFAGIDVGAAESDTLVMFEHLSSCDGEQEILSVLSSVQG----PWAFIYYQASTHSLW  135

Query  81   WNRDVNASRNML-HLALHQEK  100
            + RD    R++L H +   EK
Sbjct  136  FGRDFFGRRSLLWHFSSGTEK  156


>ref|WP_047742224.1| short-chain dehydrogenase [Enterobacter cloacae]
 gb|KLQ65669.1| short-chain dehydrogenase [Enterobacter cloacae]
Length=249

 Score = 38.5 bits (88),  Expect = 0.68, Method: Compositional matrix adjust.
 Identities = 24/73 (33%), Positives = 39/73 (53%), Gaps = 10/73 (14%)

Query  5    LLSSRTFSPTLRSRRSSPL---------EGGDVFAGAHTSAASKHAVCQAEGLSAAPGPQ  55
            LL S+   PTLR+++S  +          GG +F G H SAA    +  A+ ++   GP 
Sbjct  117  LLMSQAVIPTLRAQKSGSIVCISSVSAQRGGGIFGGPHYSAAKAGVLGLAKAMARELGPD  176

Query  56   SVRVSDVE-GVLQ  67
            +VRV+ +  G++Q
Sbjct  177  NVRVNCITPGLIQ  189


>ref|WP_044858643.1| short-chain dehydrogenase [Enterobacter cloacae]
Length=249

 Score = 38.5 bits (88),  Expect = 0.68, Method: Compositional matrix adjust.
 Identities = 24/73 (33%), Positives = 39/73 (53%), Gaps = 10/73 (14%)

Query  5    LLSSRTFSPTLRSRRSSPL---------EGGDVFAGAHTSAASKHAVCQAEGLSAAPGPQ  55
            LL S+   PTLR+++S  +          GG +F G H SAA    +  A+ ++   GP 
Sbjct  117  LLMSQAVIPTLRAQKSGSIVCISSVSAQRGGGIFGGPHYSAAKAGVLGLAKAMARELGPD  176

Query  56   SVRVSDVE-GVLQ  67
            +VRV+ +  G++Q
Sbjct  177  NVRVNCITPGLIQ  189


>ref|WP_032680432.1| MULTISPECIES: short-chain dehydrogenase [Enterobacter cloacae 
complex]
 gb|EUM09203.1| short-chain dehydrogenase/reductase SDR [Enterobacter sp. BIDMC 
30]
 gb|AKM88763.1| short-chain dehydrogenase [Enterobacter cloacae]
Length=249

 Score = 38.5 bits (88),  Expect = 0.68, Method: Compositional matrix adjust.
 Identities = 24/73 (33%), Positives = 39/73 (53%), Gaps = 10/73 (14%)

Query  5    LLSSRTFSPTLRSRRSSPL---------EGGDVFAGAHTSAASKHAVCQAEGLSAAPGPQ  55
            LL S+   PTLR+++S  +          GG +F G H SAA    +  A+ ++   GP 
Sbjct  117  LLMSQAVIPTLRAQKSGSIVCISSVSAQRGGGIFGGPHYSAAKAGVLGLAKAMARELGPD  176

Query  56   SVRVSDVE-GVLQ  67
            +VRV+ +  G++Q
Sbjct  177  NVRVNCITPGLIQ  189


>ref|WP_048267682.1| short-chain dehydrogenase [Klebsiella pneumoniae]
 gb|KMI38328.1| short-chain dehydrogenase/reductase SDR [Klebsiella pneumoniae]
Length=249

 Score = 38.5 bits (88),  Expect = 0.75, Method: Compositional matrix adjust.
 Identities = 23/73 (32%), Positives = 39/73 (53%), Gaps = 10/73 (14%)

Query  5    LLSSRTFSPTLRSRRSSPL---------EGGDVFAGAHTSAASKHAVCQAEGLSAAPGPQ  55
            LL S+  +PT+R+++S  +          GG +F G H SAA    +  A  ++   GP 
Sbjct  117  LLMSQAVTPTMRAQKSGSIVCISSVSAQRGGGIFGGPHYSAAKAGVLGLARAMARELGPD  176

Query  56   SVRVSDVE-GVLQ  67
            +VRV+ +  G++Q
Sbjct  177  NVRVNCITPGLIQ  189


>ref|WP_032455213.1| MULTISPECIES: short-chain dehydrogenase [Klebsiella]
 emb|CDN04382.1| Uncharacterized short-chain type dehydrogenase/reductase y4mP 
[Klebsiella quasipneumoniae subsp. quasipneumoniae]
 gb|KMH16504.1| short-chain dehydrogenase/reductase SDR [Klebsiella pneumoniae]
 gb|KMH51544.1| short-chain dehydrogenase/reductase SDR [Klebsiella pneumoniae]
Length=249

 Score = 38.5 bits (88),  Expect = 0.75, Method: Compositional matrix adjust.
 Identities = 24/73 (33%), Positives = 38/73 (52%), Gaps = 10/73 (14%)

Query  5    LLSSRTFSPTLRSRRSSPL---------EGGDVFAGAHTSAASKHAVCQAEGLSAAPGPQ  55
            LL S+   PT+RS++S  +          GG +F G H SAA    +  A  ++   GP 
Sbjct  117  LLMSQAVIPTMRSQKSGSIVCISSVSAQRGGGIFGGPHYSAAKAGVLGLARAMARELGPD  176

Query  56   SVRVSDVE-GVLQ  67
            +VRV+ +  G++Q
Sbjct  177  NVRVNCITPGLIQ  189


>ref|WP_044521813.1| MULTISPECIES: short-chain dehydrogenase [Klebsiella]
 emb|CDQ13483.1| Uncharacterized short-chain type dehydrogenase/reductase y4mP 
[Klebsiella quasipneumoniae subsp. quasipneumoniae]
 gb|KNG99888.1| short-chain dehydrogenase [Klebsiella quasipneumoniae subsp. 
quasipneumoniae]
Length=249

 Score = 38.5 bits (88),  Expect = 0.77, Method: Compositional matrix adjust.
 Identities = 24/73 (33%), Positives = 38/73 (52%), Gaps = 10/73 (14%)

Query  5    LLSSRTFSPTLRSRRSSPL---------EGGDVFAGAHTSAASKHAVCQAEGLSAAPGPQ  55
            LL S+   PT+RS++S  +          GG +F G H SAA    +  A  ++   GP 
Sbjct  117  LLMSQAVIPTMRSQKSGSIVCISSVSAQRGGGIFGGPHYSAAKAGVLGLARAMARELGPD  176

Query  56   SVRVSDVE-GVLQ  67
            +VRV+ +  G++Q
Sbjct  177  NVRVNCITPGLIQ  189


>ref|WP_023288935.1| short-chain dehydrogenase/reductase SDR [Klebsiella pneumoniae]
 gb|ESL77831.1| short-chain dehydrogenase/reductase SDR [Klebsiella pneumoniae 
UCICRE 14]
Length=249

 Score = 38.5 bits (88),  Expect = 0.77, Method: Compositional matrix adjust.
 Identities = 24/73 (33%), Positives = 38/73 (52%), Gaps = 10/73 (14%)

Query  5    LLSSRTFSPTLRSRRSSPL---------EGGDVFAGAHTSAASKHAVCQAEGLSAAPGPQ  55
            LL S+   PT+RS++S  +          GG +F G H SAA    +  A  ++   GP 
Sbjct  117  LLMSQAVIPTMRSQKSGSIVCISSVSAQRGGGIFGGPHYSAAKAGVLGLARAMARELGPD  176

Query  56   SVRVSDVE-GVLQ  67
            +VRV+ +  G++Q
Sbjct  177  NVRVNCITPGLIQ  189


>ref|WP_040168882.1| short-chain dehydrogenase [Klebsiella pneumoniae]
Length=249

 Score = 38.5 bits (88),  Expect = 0.84, Method: Compositional matrix adjust.
 Identities = 23/73 (32%), Positives = 38/73 (52%), Gaps = 10/73 (14%)

Query  5    LLSSRTFSPTLRSRRSSPL---------EGGDVFAGAHTSAASKHAVCQAEGLSAAPGPQ  55
            LL S+   PT+R+++S  +          GG +F G H SAA    +  A  ++   GP 
Sbjct  117  LLMSQAVIPTMRAQKSGSIVCISSVSAQRGGGIFGGPHYSAAKAGVLGLARAMARELGPD  176

Query  56   SVRVSDVE-GVLQ  67
            +VRV+ +  G++Q
Sbjct  177  NVRVNSITPGLIQ  189


>ref|WP_003862216.1| MULTISPECIES: short-chain dehydrogenase [Enterobacter]
 gb|EIM34631.1| protein FabG [Enterobacter cloacae subsp. cloacae GS1]
 gb|ERP08791.1| short-chain dehydrogenase/reductase SDR [Enterobacter sp. MGH 
14]
 25 more sequence titles

gb|ESM46927.1| short-chain dehydrogenase/reductase SDR [Enterobacter cloacae 
BWH 29]
 gb|ESM88971.1| short-chain dehydrogenase/reductase SDR [Enterobacter sp. MGH 
38]
 emb|CDL32012.1| 3-oxoacyl-[acyl-carrier protein] reductase [Enterobacter cloacae 
ISC8]
 gb|EUL65682.1| short-chain dehydrogenase/reductase SDR [Enterobacter cloacae 
UCI 35]
 gb|EUL70503.1| short-chain dehydrogenase/reductase SDR [Enterobacter cloacae 
UCI 36]
 gb|EUM14956.1| short-chain dehydrogenase/reductase SDR [Enterobacter sp. BIDMC 
29]
 gb|EUN09439.1| short-chain dehydrogenase/reductase SDR [Enterobacter sp. MGH 
3]
 gb|KHG48077.1| KR domain protein [Enterobacter cloacae MRSN 11489]
 gb|AJB83033.1| short-chain dehydrogenase [Enterobacter cloacae]
 gb|KJC02449.1| short-chain dehydrogenase [Enterobacter cloacae]
 gb|KJL63121.1| short-chain dehydrogenase [Enterobacter cloacae]
 gb|KJN06702.1| short-chain dehydrogenase [Enterobacter cloacae]
 gb|KJO83309.1| short-chain dehydrogenase [Enterobacter cloacae]
 gb|KJP04110.1| short-chain dehydrogenase [Enterobacter cloacae]
 gb|KJP31736.1| short-chain dehydrogenase [Enterobacter cloacae]
 gb|KJQ41204.1| short-chain dehydrogenase [Enterobacter cloacae]
 gb|KJX26579.1| short-chain dehydrogenase [Enterobacter cloacae]
 emb|CQR76517.1| 3-oxoacyl-[acyl-carrier-protein] reductase FabG [Enterobacter 
cloacae]
 gb|KLP98919.1| short-chain dehydrogenase [Enterobacter cloacae]
 gb|KLQ04304.1| short-chain dehydrogenase [Enterobacter cloacae]
 gb|KLR17365.1| short-chain dehydrogenase [Enterobacter sp. GN02283]
 gb|KLW15615.1| short-chain dehydrogenase/reductase SDR [Enterobacter sp. BWH52]
 gb|KLW38307.1| short-chain dehydrogenase/reductase SDR [Enterobacter sp. MGH119]
 gb|KLW50534.1| short-chain dehydrogenase/reductase SDR [Enterobacter sp. MGH128]
 gb|KLW89758.1| short-chain dehydrogenase/reductase SDR [Enterobacter sp. BIDMC100]

Length=249

 Score = 38.1 bits (87),  Expect = 1.0, Method: Compositional matrix adjust.
 Identities = 23/73 (32%), Positives = 39/73 (53%), Gaps = 10/73 (14%)

Query  5    LLSSRTFSPTLRSRRSSPL---------EGGDVFAGAHTSAASKHAVCQAEGLSAAPGPQ  55
            LL S+   PT+R+++S  +          GG +F G H SAA    +  A+ ++   GP 
Sbjct  117  LLMSQAVIPTMRAQKSGSIVCISSVSAQRGGGIFGGPHYSAAKAGVLGLAKAMARELGPD  176

Query  56   SVRVSDVE-GVLQ  67
            +VRV+ +  G++Q
Sbjct  177  NVRVNCITPGLIQ  189


>ref|WP_032609987.1| MULTISPECIES: short-chain dehydrogenase [Enterobacter cloacae 
complex]
 gb|EUM76195.1| short-chain dehydrogenase/reductase SDR [Enterobacter sp. MGH 
7]
 gb|KHA95741.1| short-chain dehydrogenase [Enterobacter cloacae]
 71 more sequence titles

gb|KHM02930.1| short-chain dehydrogenase [Enterobacter cloacae]
 gb|KHM04678.1| short-chain dehydrogenase [Enterobacter cloacae]
 gb|KHM08737.1| short-chain dehydrogenase [Enterobacter cloacae]
 gb|KHM17374.1| short-chain dehydrogenase [Enterobacter cloacae]
 gb|KHM25368.1| short-chain dehydrogenase [Enterobacter cloacae]
 gb|KHM27165.1| short-chain dehydrogenase [Enterobacter cloacae]
 gb|KHM41807.1| short-chain dehydrogenase [Enterobacter cloacae]
 gb|KHM41882.1| short-chain dehydrogenase [Enterobacter cloacae]
 gb|KHM61033.1| short-chain dehydrogenase [Enterobacter cloacae]
 gb|KHM64594.1| short-chain dehydrogenase [Enterobacter cloacae]
 gb|KHM65268.1| short-chain dehydrogenase [Enterobacter cloacae]
 gb|KHM72564.1| short-chain dehydrogenase [Enterobacter cloacae]
 gb|KHM78309.1| short-chain dehydrogenase [Enterobacter cloacae]
 gb|KHM80457.1| short-chain dehydrogenase [Enterobacter cloacae]
 gb|KHM85054.1| short-chain dehydrogenase [Enterobacter cloacae]
 gb|KHM88563.1| short-chain dehydrogenase [Enterobacter cloacae]
 gb|KHQ17858.1| short-chain dehydrogenase [Enterobacter cloacae]
 gb|KHQ56377.1| short-chain dehydrogenase [Enterobacter cloacae]
 gb|KJF30670.1| short-chain dehydrogenase/reductase SDR [Enterobacter cloacae 
BIDMC 33A]
 gb|KJH91016.1| short-chain dehydrogenase [Enterobacter cloacae]
 gb|KJH94009.1| short-chain dehydrogenase [Enterobacter cloacae]
 gb|KJI03345.1| short-chain dehydrogenase [Enterobacter cloacae]
 gb|KJI14557.1| short-chain dehydrogenase [Enterobacter cloacae]
 gb|KJI21929.1| short-chain dehydrogenase [Enterobacter cloacae]
 gb|KJI24849.1| short-chain dehydrogenase [Enterobacter cloacae]
 gb|KJI32899.1| short-chain dehydrogenase [Enterobacter cloacae]
 gb|KJI42132.1| short-chain dehydrogenase [Enterobacter cloacae]
 gb|KJI44494.1| short-chain dehydrogenase [Enterobacter cloacae]
 gb|KJI52592.1| short-chain dehydrogenase [Enterobacter cloacae]
 gb|KJI54771.1| short-chain dehydrogenase [Enterobacter cloacae]
 gb|KJI68070.1| short-chain dehydrogenase [Enterobacter cloacae]
 gb|KJI75637.1| short-chain dehydrogenase [Enterobacter cloacae]
 gb|KJI79141.1| short-chain dehydrogenase [Enterobacter cloacae]
 gb|KJI87557.1| short-chain dehydrogenase [Enterobacter cloacae]
 gb|KJI92961.1| short-chain dehydrogenase [Enterobacter cloacae]
 gb|KJI96991.1| short-chain dehydrogenase [Enterobacter cloacae]
 gb|KJJ03302.1| short-chain dehydrogenase [Enterobacter cloacae]
 gb|KJJ08346.1| short-chain dehydrogenase [Enterobacter cloacae]
 gb|KJL94700.1| short-chain dehydrogenase [Enterobacter cloacae]
 gb|KJM20802.1| short-chain dehydrogenase [Enterobacter cloacae]
 gb|KJM22612.1| short-chain dehydrogenase [Enterobacter cloacae]
 gb|KJM33177.1| short-chain dehydrogenase [Enterobacter cloacae]
 gb|KJM48364.1| short-chain dehydrogenase [Enterobacter cloacae]
 gb|KJN79303.1| short-chain dehydrogenase [Enterobacter cloacae]
 gb|KJN88747.1| short-chain dehydrogenase [Enterobacter cloacae]
 gb|KJO10836.1| short-chain dehydrogenase [Enterobacter cloacae]
 gb|KJO22796.1| short-chain dehydrogenase [Enterobacter cloacae]
 gb|KJO71298.1| short-chain dehydrogenase [Enterobacter cloacae]
 gb|KJO76653.1| short-chain dehydrogenase [Enterobacter cloacae]
 gb|KJO87639.1| short-chain dehydrogenase [Enterobacter cloacae]
 gb|KJO88893.1| short-chain dehydrogenase [Enterobacter cloacae]
 gb|KJP22813.1| short-chain dehydrogenase [Enterobacter cloacae]
 gb|KJP25651.1| short-chain dehydrogenase [Enterobacter cloacae]
 gb|KJP26885.1| short-chain dehydrogenase [Enterobacter cloacae]
 gb|KJP49766.1| short-chain dehydrogenase [Enterobacter cloacae]
 gb|KJP50489.1| short-chain dehydrogenase [Enterobacter cloacae]
 gb|KJP61168.1| short-chain dehydrogenase [Enterobacter cloacae]
 gb|KJP67992.1| short-chain dehydrogenase [Enterobacter cloacae]
 gb|KJP78346.1| short-chain dehydrogenase [Enterobacter cloacae]
 gb|KJP97566.1| short-chain dehydrogenase [Enterobacter cloacae]
 gb|KJQ03477.1| short-chain dehydrogenase [Enterobacter cloacae]
 gb|KJQ09715.1| short-chain dehydrogenase [Enterobacter cloacae]
 gb|KJQ12063.1| short-chain dehydrogenase [Enterobacter cloacae]
 gb|KJQ22367.1| short-chain dehydrogenase [Enterobacter cloacae]
 gb|KJQ28151.1| short-chain dehydrogenase [Enterobacter cloacae]
 gb|KJQ30467.1| short-chain dehydrogenase [Enterobacter cloacae]
 gb|KJQ30957.1| short-chain dehydrogenase [Enterobacter cloacae]
 gb|KJQ43225.1| short-chain dehydrogenase [Enterobacter cloacae]
 gb|KLE20581.1| short-chain dehydrogenase [Enterobacter cloacae]
 gb|KLR67115.1| short-chain dehydrogenase [Enterobacter cloacae]
 gb|ALA03470.1| short-chain dehydrogenase [Enterobacter cloacae]

Length=249

 Score = 38.1 bits (87),  Expect = 1.0, Method: Compositional matrix adjust.
 Identities = 23/73 (32%), Positives = 39/73 (53%), Gaps = 10/73 (14%)

Query  5    LLSSRTFSPTLRSRRSSPL---------EGGDVFAGAHTSAASKHAVCQAEGLSAAPGPQ  55
            LL S+   PT+R+++S  +          GG +F G H SAA    +  A+ ++   GP 
Sbjct  117  LLMSQAVIPTMRAQKSGSIVCISSVSAQRGGGIFGGPHYSAAKAGVLGLAKAMARELGPD  176

Query  56   SVRVSDVE-GVLQ  67
            +VRV+ +  G++Q
Sbjct  177  NVRVNCITPGLIQ  189


>ref|WP_023620857.1| MULTISPECIES: short-chain dehydrogenase [Enterobacter]
 gb|KIF94140.1| short-chain dehydrogenase [Enterobacter cloacae]
 gb|KJM35137.1| short-chain dehydrogenase [Enterobacter sp. 42324]
 gb|KJX10773.1| short-chain dehydrogenase [Enterobacter cloacae]
 gb|KLG10645.1| short-chain dehydrogenase [Enterobacter sp. GN02174]
 gb|KLQ16343.1| short-chain dehydrogenase [Enterobacter sp. GN02616]
Length=249

 Score = 38.1 bits (87),  Expect = 1.0, Method: Compositional matrix adjust.
 Identities = 23/73 (32%), Positives = 39/73 (53%), Gaps = 10/73 (14%)

Query  5    LLSSRTFSPTLRSRRSSPL---------EGGDVFAGAHTSAASKHAVCQAEGLSAAPGPQ  55
            LL S+   PT+R+++S  +          GG +F G H SAA    +  A+ ++   GP 
Sbjct  117  LLMSQAVIPTMRAQKSGSIVCISSVSAQRGGGIFGGPHYSAAKAGVLGLAKAMARELGPD  176

Query  56   SVRVSDVE-GVLQ  67
            +VRV+ +  G++Q
Sbjct  177  NVRVNCITPGLIQ  189


>ref|WP_032660057.1| short-chain dehydrogenase [Enterobacter cloacae]
 gb|EUM30064.1| short-chain dehydrogenase/reductase SDR [Enterobacter cloacae 
BIDMC 8]
 gb|KJO30016.1| short-chain dehydrogenase [Enterobacter cloacae]
 gb|KJO91761.1| short-chain dehydrogenase [Enterobacter cloacae]
 gb|KJP00386.1| short-chain dehydrogenase [Enterobacter cloacae]
 gb|KJX44054.1| short-chain dehydrogenase [Enterobacter cloacae]
Length=249

 Score = 38.1 bits (87),  Expect = 1.1, Method: Compositional matrix adjust.
 Identities = 23/73 (32%), Positives = 39/73 (53%), Gaps = 10/73 (14%)

Query  5    LLSSRTFSPTLRSRRSSPL---------EGGDVFAGAHTSAASKHAVCQAEGLSAAPGPQ  55
            LL S+   PT+R+++S  +          GG +F G H SAA    +  A+ ++   GP 
Sbjct  117  LLMSQAVIPTMRAQKSGSIVCISSVSAQRGGGIFGGPHYSAAKAGVLGLAKAMARELGPD  176

Query  56   SVRVSDVE-GVLQ  67
            +VRV+ +  G++Q
Sbjct  177  NVRVNCITPGLIQ  189


>ref|WP_050860982.1| short-chain dehydrogenase [Enterobacter cloacae]
Length=249

 Score = 38.1 bits (87),  Expect = 1.1, Method: Compositional matrix adjust.
 Identities = 23/73 (32%), Positives = 39/73 (53%), Gaps = 10/73 (14%)

Query  5    LLSSRTFSPTLRSRRSSPL---------EGGDVFAGAHTSAASKHAVCQAEGLSAAPGPQ  55
            LL S+   PT+R+++S  +          GG +F G H SAA    +  A+ ++   GP 
Sbjct  117  LLMSQAVIPTMRAQKSGSIVCISSVSAQRGGGIFGGPHYSAAKAGVLGLAKAMARELGPD  176

Query  56   SVRVSDVE-GVLQ  67
            +VRV+ +  G++Q
Sbjct  177  NVRVNCITPGLIQ  189


>ref|WP_038980951.1| short-chain dehydrogenase [Enterobacter cloacae]
Length=249

 Score = 38.1 bits (87),  Expect = 1.1, Method: Compositional matrix adjust.
 Identities = 23/73 (32%), Positives = 39/73 (53%), Gaps = 10/73 (14%)

Query  5    LLSSRTFSPTLRSRRSSPL---------EGGDVFAGAHTSAASKHAVCQAEGLSAAPGPQ  55
            LL S+   PT+R+++S  +          GG +F G H SAA    +  A+ ++   GP 
Sbjct  117  LLMSQAVIPTMRAQKSGSIVCISSVSAQRGGGIFGGPHYSAAKAGVLGLAKAMARELGPD  176

Query  56   SVRVSDVE-GVLQ  67
            +VRV+ +  G++Q
Sbjct  177  NVRVNCITPGLIQ  189


>ref|WP_036364567.1| short-chain dehydrogenase [Microvirga lupini]
Length=249

 Score = 38.1 bits (87),  Expect = 1.1, Method: Compositional matrix adjust.
 Identities = 24/77 (31%), Positives = 37/77 (48%), Gaps = 10/77 (13%)

Query  1    MVSALLSSRTFSPTLRSRRSSPL---------EGGDVFAGAHTSAASKHAVCQAEGLSAA  51
            +   L  S+ F P +RSRR   +          GG +F G H SAA    +  A+ ++  
Sbjct  113  LTGVLYLSQAFIPHMRSRRRGSIACMSSVSAQRGGGIFGGPHYSAAKAGVLGLAKAMARE  172

Query  52   PGPQSVRVSDVE-GVLQ  67
             GP  +RV+ V  G++Q
Sbjct  173  LGPDGIRVNCVTPGLIQ  189


>ref|WP_014832991.1| MULTISPECIES: short-chain dehydrogenase [Enterobacter]
 gb|AFM61223.1| putative short-chain dehydrogenase/reductase SDR [Enterobacter 
cloacae subsp. dissolvens SDM]
 gb|KLQ39554.1| short-chain dehydrogenase [Enterobacter sp. GN02534]
Length=249

 Score = 38.1 bits (87),  Expect = 1.1, Method: Compositional matrix adjust.
 Identities = 23/73 (32%), Positives = 39/73 (53%), Gaps = 10/73 (14%)

Query  5    LLSSRTFSPTLRSRRSSPL---------EGGDVFAGAHTSAASKHAVCQAEGLSAAPGPQ  55
            LL S+   PT+R+++S  +          GG +F G H SAA    +  A+ ++   GP 
Sbjct  117  LLMSQAVIPTMRAQKSGSIVCISSVSAQRGGGIFGGPHYSAAKAGVLGLAKAMARELGPD  176

Query  56   SVRVSDVE-GVLQ  67
            +VRV+ +  G++Q
Sbjct  177  NVRVNCITPGLIQ  189


>ref|WP_049136890.1| short-chain dehydrogenase [Enterobacter cloacae]
Length=249

 Score = 38.1 bits (87),  Expect = 1.1, Method: Compositional matrix adjust.
 Identities = 23/73 (32%), Positives = 39/73 (53%), Gaps = 10/73 (14%)

Query  5    LLSSRTFSPTLRSRRSSPL---------EGGDVFAGAHTSAASKHAVCQAEGLSAAPGPQ  55
            LL S+   PT+R+++S  +          GG +F G H SAA    +  A+ ++   GP 
Sbjct  117  LLMSQAVIPTMRAQKSGSIVCISSVSAQRGGGIFGGPHYSAAKAGVLGLAKAMARELGPD  176

Query  56   SVRVSDVE-GVLQ  67
            +VRV+ +  G++Q
Sbjct  177  NVRVNCITPGLIQ  189


>ref|WP_038857766.1| short-chain dehydrogenase [Cronobacter universalis]
 gb|ALB54137.1| short-chain dehydrogenase [Cronobacter universalis NCTC 9529]
Length=249

 Score = 38.1 bits (87),  Expect = 1.1, Method: Compositional matrix adjust.
 Identities = 23/73 (32%), Positives = 39/73 (53%), Gaps = 10/73 (14%)

Query  5    LLSSRTFSPTLRSRRSSPL---------EGGDVFAGAHTSAASKHAVCQAEGLSAAPGPQ  55
            LL S+   PT+R+++S  +          GG +F G H SAA    +  A+ ++   GP 
Sbjct  117  LLMSQAVIPTMRAQKSGSIVCISSVSAQRGGGIFGGPHYSAAKAGVLGLAKAMARELGPD  176

Query  56   SVRVSDVE-GVLQ  67
            +VRV+ +  G++Q
Sbjct  177  NVRVNCITPGLIQ  189


>ref|WP_019846055.1| short-chain dehydrogenase [Dickeya zeae]
Length=249

 Score = 38.1 bits (87),  Expect = 1.1, Method: Compositional matrix adjust.
 Identities = 23/73 (32%), Positives = 39/73 (53%), Gaps = 10/73 (14%)

Query  5    LLSSRTFSPTLRSRRSSPL---------EGGDVFAGAHTSAASKHAVCQAEGLSAAPGPQ  55
            LL S+   PT+R+++S  +          GG +F G H SAA    +  A+ ++   GP 
Sbjct  117  LLMSQAVIPTMRAQKSGSIVCISSVSAQRGGGIFGGPHYSAAKAGVLGLAKAMARELGPD  176

Query  56   SVRVSDVE-GVLQ  67
            +VRV+ +  G++Q
Sbjct  177  NVRVNCITPGLIQ  189


>ref|WP_046888505.1| short-chain dehydrogenase [Enterobacter cloacae]
 gb|KKY82221.1| short-chain dehydrogenase [Enterobacter cloacae]
Length=249

 Score = 38.1 bits (87),  Expect = 1.1, Method: Compositional matrix adjust.
 Identities = 23/73 (32%), Positives = 39/73 (53%), Gaps = 10/73 (14%)

Query  5    LLSSRTFSPTLRSRRSSPL---------EGGDVFAGAHTSAASKHAVCQAEGLSAAPGPQ  55
            LL S+   PT+R+++S  +          GG +F G H SAA    +  A+ ++   GP 
Sbjct  117  LLMSQAVIPTMRAQKSGSIVCISSVSAQRGGGIFGGPHYSAAKAGVLGLAKAMARELGPD  176

Query  56   SVRVSDVE-GVLQ  67
            +VRV+ +  G++Q
Sbjct  177  NVRVNCITPGLIQ  189


>ref|WP_028014159.1| short-chain dehydrogenase [Enterobacter cloacae]
Length=249

 Score = 38.1 bits (87),  Expect = 1.1, Method: Compositional matrix adjust.
 Identities = 23/73 (32%), Positives = 39/73 (53%), Gaps = 10/73 (14%)

Query  5    LLSSRTFSPTLRSRRSSPL---------EGGDVFAGAHTSAASKHAVCQAEGLSAAPGPQ  55
            LL S+   PT+R+++S  +          GG +F G H SAA    +  A+ ++   GP 
Sbjct  117  LLMSQAVIPTMRAQKSGSIVCISSVSAQRGGGIFGGPHYSAAKAGVLGLAKAMARELGPD  176

Query  56   SVRVSDVE-GVLQ  67
            +VRV+ +  G++Q
Sbjct  177  NVRVNCITPGLIQ  189


>ref|WP_023639989.1| short-chain dehydrogenase [Dickeya zeae]
Length=249

 Score = 38.1 bits (87),  Expect = 1.1, Method: Compositional matrix adjust.
 Identities = 23/73 (32%), Positives = 39/73 (53%), Gaps = 10/73 (14%)

Query  5    LLSSRTFSPTLRSRRSSPL---------EGGDVFAGAHTSAASKHAVCQAEGLSAAPGPQ  55
            LL S+   PT+R+++S  +          GG +F G H SAA    +  A+ ++   GP 
Sbjct  117  LLMSQAVIPTMRAQKSGSIVCISSVSAQRGGGIFGGPHYSAAKAGVLGLAKAMARELGPD  176

Query  56   SVRVSDVE-GVLQ  67
            +VRV+ +  G++Q
Sbjct  177  NVRVNCITPGLIQ  189


>ref|WP_045346168.1| short-chain dehydrogenase [Enterobacter cloacae]
 gb|KJM68262.1| short-chain dehydrogenase [Enterobacter cloacae]
Length=249

 Score = 38.1 bits (87),  Expect = 1.2, Method: Compositional matrix adjust.
 Identities = 23/73 (32%), Positives = 39/73 (53%), Gaps = 10/73 (14%)

Query  5    LLSSRTFSPTLRSRRSSPL---------EGGDVFAGAHTSAASKHAVCQAEGLSAAPGPQ  55
            LL S+   PT+R+++S  +          GG +F G H SAA    +  A+ ++   GP 
Sbjct  117  LLMSQAVIPTMRAQKSGSIVCISSVSAQRGGGIFGGPHYSAAKAGVLGLAKAMARELGPD  176

Query  56   SVRVSDVE-GVLQ  67
            +VRV+ +  G++Q
Sbjct  177  NVRVNCITPGLIQ  189


>ref|WP_013317389.1| short-chain dehydrogenase [Dickeya dadantii]
 gb|ADM97928.1| 3-oxoacyl-[acyl-carrier protein] reductase [Dickeya dadantii 
3937]
Length=249

 Score = 38.1 bits (87),  Expect = 1.2, Method: Compositional matrix adjust.
 Identities = 23/73 (32%), Positives = 39/73 (53%), Gaps = 10/73 (14%)

Query  5    LLSSRTFSPTLRSRRSSPL---------EGGDVFAGAHTSAASKHAVCQAEGLSAAPGPQ  55
            LL S+   PT+R+++S  +          GG +F G H SAA    +  A+ ++   GP 
Sbjct  117  LLMSQAVIPTMRAQKSGSIVCISSVSAQRGGGIFGGPHYSAAKAGVLGLAKAMARELGPD  176

Query  56   SVRVSDVE-GVLQ  67
            +VRV+ +  G++Q
Sbjct  177  NVRVNCITPGLIQ  189


>ref|WP_048210587.1| short-chain dehydrogenase [Enterobacter cloacae]
 gb|KLW08695.1| short-chain dehydrogenase/reductase SDR [Enterobacter cloacae]
 gb|KLW11401.1| short-chain dehydrogenase/reductase SDR [Enterobacter cloacae]
Length=249

 Score = 37.7 bits (86),  Expect = 1.5, Method: Compositional matrix adjust.
 Identities = 23/73 (32%), Positives = 39/73 (53%), Gaps = 10/73 (14%)

Query  5    LLSSRTFSPTLRSRRSSPL---------EGGDVFAGAHTSAASKHAVCQAEGLSAAPGPQ  55
            LL S+   PT+R+++S  +          GG +F G H SAA    +  A+ ++   GP 
Sbjct  117  LLMSQAVIPTMRAQKSGSIVCISSVSAQRGGGIFGGPHYSAARAGVLGLAKAMARELGPD  176

Query  56   SVRVSDVE-GVLQ  67
            +VRV+ +  G++Q
Sbjct  177  NVRVNCITPGLIQ  189


>ref|WP_013098400.1| short-chain dehydrogenase [Enterobacter cloacae]
 ref|YP_003614477.1| putative short-chain dehydrogenase/reductase SDR [Enterobacter 
cloacae subsp. cloacae ATCC 13047]
 gb|ADF63528.1| putative short-chain dehydrogenase/reductase SDR [Enterobacter 
cloacae subsp. cloacae ATCC 13047]
 gb|KGB12959.1| short chain dehydrogenase family protein [Enterobacter cloacae]
Length=249

 Score = 37.7 bits (86),  Expect = 1.7, Method: Compositional matrix adjust.
 Identities = 23/73 (32%), Positives = 39/73 (53%), Gaps = 10/73 (14%)

Query  5    LLSSRTFSPTLRSRRSSPL---------EGGDVFAGAHTSAASKHAVCQAEGLSAAPGPQ  55
            LL S+   PT+R+++S  +          GG +F G H SAA    +  A+ ++   GP 
Sbjct  117  LLMSQAVIPTMRAQQSGSIVCISSVSAQRGGGIFGGPHYSAAKAGVLGLAKAMARELGPD  176

Query  56   SVRVSDVE-GVLQ  67
            +VRV+ +  G++Q
Sbjct  177  NVRVNCITPGLIQ  189


>ref|WP_038420466.1| short-chain dehydrogenase [Enterobacter cloacae]
 gb|AIV31065.1| short-chain dehydrogenase [Enterobacter cloacae]
Length=249

 Score = 37.4 bits (85),  Expect = 1.7, Method: Compositional matrix adjust.
 Identities = 23/73 (32%), Positives = 39/73 (53%), Gaps = 10/73 (14%)

Query  5    LLSSRTFSPTLRSRRSSPL---------EGGDVFAGAHTSAASKHAVCQAEGLSAAPGPQ  55
            LL S+   PT+R+++S  +          GG +F G H SAA    +  A+ ++   GP 
Sbjct  117  LLMSQAVIPTMRAQQSGSIVCISSVSAQRGGGIFGGPHYSAAKAGVLGLAKAMARELGPD  176

Query  56   SVRVSDVE-GVLQ  67
            +VRV+ +  G++Q
Sbjct  177  NVRVNCITPGLIQ  189


>ref|WP_048970757.1| short-chain dehydrogenase [Enterobacter cloacae]
Length=249

 Score = 37.4 bits (85),  Expect = 1.7, Method: Compositional matrix adjust.
 Identities = 23/73 (32%), Positives = 39/73 (53%), Gaps = 10/73 (14%)

Query  5    LLSSRTFSPTLRSRRSSPL---------EGGDVFAGAHTSAASKHAVCQAEGLSAAPGPQ  55
            LL S+   PT+R+++S  +          GG +F G H SAA    +  A+ ++   GP 
Sbjct  117  LLMSQAVIPTMRAQQSGSIVCISSVSAQRGGGIFGGPHYSAAKAGVLGLAKAMARELGPD  176

Query  56   SVRVSDVE-GVLQ  67
            +VRV+ +  G++Q
Sbjct  177  NVRVNCITPGLIQ  189


>ref|WP_023284879.1| short-chain dehydrogenase/reductase SDR [Klebsiella pneumoniae]
 gb|ESL55699.1| short-chain dehydrogenase/reductase SDR [Klebsiella pneumoniae 
BIDMC 24]
Length=249

 Score = 37.4 bits (85),  Expect = 1.7, Method: Compositional matrix adjust.
 Identities = 23/77 (30%), Positives = 39/77 (51%), Gaps = 10/77 (13%)

Query  1    MVSALLSSRTFSPTLRSRRSSPL---------EGGDVFAGAHTSAASKHAVCQAEGLSAA  51
            +   LL S+   PT+R+++S  +          GG +F G H SAA    +  A  ++  
Sbjct  113  LCGTLLMSQAVIPTMRAQKSGSIVCISSVSAQRGGGIFGGPHYSAAKAGVLGLARAMARE  172

Query  52   PGPQSVRVSDVE-GVLQ  67
             GP +VRV+ +  G++Q
Sbjct  173  LGPDNVRVNCITPGLIQ  189


>gb|AGT25156.1| putative short-chain dehydrogenase/reductase SDR [Klebsiella 
pneumoniae JM45]
 gb|AHE45558.1| short-chain type dehydrogenase/reductase [Klebsiella pneumoniae 
subsp. pneumoniae Kp13]
 gb|AHM80524.1| hypothetical protein KPNJ2_03744 [Klebsiella pneumoniae 30684/NJST258_2]
 gb|AHM86162.1| hypothetical protein KPNJ1_03756 [Klebsiella pneumoniae 30660/NJST258_1]
Length=255

 Score = 37.4 bits (85),  Expect = 1.7, Method: Compositional matrix adjust.
 Identities = 23/73 (32%), Positives = 38/73 (52%), Gaps = 10/73 (14%)

Query  5    LLSSRTFSPTLRSRRSSPL---------EGGDVFAGAHTSAASKHAVCQAEGLSAAPGPQ  55
            LL S+   PT+R+++S  +          GG +F G H SAA    +  A  ++   GP 
Sbjct  123  LLMSQAVIPTMRAQKSGSIVCISSVSAQRGGGIFGGPHYSAAKAGVLGLARAMARELGPD  182

Query  56   SVRVSDVE-GVLQ  67
            +VRV+ +  G++Q
Sbjct  183  NVRVNCITPGLIQ  195


>ref|WP_032613441.1| short-chain dehydrogenase [Leclercia adecarboxylata]
 gb|KFC95854.1| 3-oxoacyl-[acyl-carrier protein] reductase [Leclercia adecarboxylata 
ATCC 23216 = NBRC 102595]
Length=249

 Score = 37.4 bits (85),  Expect = 1.7, Method: Compositional matrix adjust.
 Identities = 23/73 (32%), Positives = 39/73 (53%), Gaps = 10/73 (14%)

Query  5    LLSSRTFSPTLRSRRSSPL---------EGGDVFAGAHTSAASKHAVCQAEGLSAAPGPQ  55
            LL S+   PT+R+++S  +          GG +F G H SAA    +  A+ ++   GP 
Sbjct  117  LLMSQAVIPTMRAQQSGSIVCISSVSAQRGGGIFGGPHYSAAKAGVLGLAKAMARELGPD  176

Query  56   SVRVSDVE-GVLQ  67
            +VRV+ +  G++Q
Sbjct  177  NVRVNCITPGLIQ  189


>ref|WP_049087277.1| MULTISPECIES: short-chain dehydrogenase [Enterobacteriaceae]
 dbj|BAS39712.1| 3-oxoacyl-ACP reductase [Klebsiella oxytoca]
Length=249

 Score = 37.4 bits (85),  Expect = 1.8, Method: Compositional matrix adjust.
 Identities = 23/73 (32%), Positives = 38/73 (52%), Gaps = 10/73 (14%)

Query  5    LLSSRTFSPTLRSRRSSPL---------EGGDVFAGAHTSAASKHAVCQAEGLSAAPGPQ  55
            LL S+   PT+R+++S  +          GG +F G H SAA    +  A  ++   GP 
Sbjct  117  LLMSQAVIPTMRAQKSGSIVCISSVSAQRGGGIFGGPHYSAAKAGVLGLARAMARELGPD  176

Query  56   SVRVSDVE-GVLQ  67
            +VRV+ +  G++Q
Sbjct  177  NVRVNCITPGLIQ  189


>ref|WP_040174808.1| short-chain dehydrogenase [Klebsiella pneumoniae]
Length=249

 Score = 37.4 bits (85),  Expect = 1.8, Method: Compositional matrix adjust.
 Identities = 23/73 (32%), Positives = 38/73 (52%), Gaps = 10/73 (14%)

Query  5    LLSSRTFSPTLRSRRSSPL---------EGGDVFAGAHTSAASKHAVCQAEGLSAAPGPQ  55
            LL S+   PT+R+++S  +          GG +F G H SAA    +  A  ++   GP 
Sbjct  117  LLMSQAVIPTMRAQKSGSIVCISSVSAQRGGGIFGGPHYSAAKAGVLGLARAMARELGPD  176

Query  56   SVRVSDVE-GVLQ  67
            +VRV+ +  G++Q
Sbjct  177  NVRVNCITPGLIQ  189


>ref|WP_024360134.1| MULTISPECIES: short-chain dehydrogenase [Klebsiella]
Length=249

 Score = 37.4 bits (85),  Expect = 1.8, Method: Compositional matrix adjust.
 Identities = 23/73 (32%), Positives = 38/73 (52%), Gaps = 10/73 (14%)

Query  5    LLSSRTFSPTLRSRRSSPL---------EGGDVFAGAHTSAASKHAVCQAEGLSAAPGPQ  55
            LL S+   PT+R+++S  +          GG +F G H SAA    +  A  ++   GP 
Sbjct  117  LLMSQAVIPTMRAQKSGSIVCISSVSAQRGGGIFGGPHYSAAKAGVLGLARAMARELGPD  176

Query  56   SVRVSDVE-GVLQ  67
            +VRV+ +  G++Q
Sbjct  177  NVRVNCITPGLIQ  189


>ref|WP_049182433.1| short-chain dehydrogenase [Klebsiella pneumoniae]
Length=249

 Score = 37.4 bits (85),  Expect = 1.9, Method: Compositional matrix adjust.
 Identities = 23/73 (32%), Positives = 38/73 (52%), Gaps = 10/73 (14%)

Query  5    LLSSRTFSPTLRSRRSSPL---------EGGDVFAGAHTSAASKHAVCQAEGLSAAPGPQ  55
            LL S+   PT+R+++S  +          GG +F G H SAA    +  A  ++   GP 
Sbjct  117  LLMSQAVIPTMRAQKSGSIVCISSVSAQRGGGIFGGPHYSAAKAGVLGLARAMARELGPD  176

Query  56   SVRVSDVE-GVLQ  67
            +VRV+ +  G++Q
Sbjct  177  NVRVNCITPGLIQ  189


>ref|WP_032104939.1| short-chain dehydrogenase [Klebsiella pneumoniae]
Length=249

 Score = 37.4 bits (85),  Expect = 1.9, Method: Compositional matrix adjust.
 Identities = 23/73 (32%), Positives = 38/73 (52%), Gaps = 10/73 (14%)

Query  5    LLSSRTFSPTLRSRRSSPL---------EGGDVFAGAHTSAASKHAVCQAEGLSAAPGPQ  55
            LL S+   PT+R+++S  +          GG +F G H SAA    +  A  ++   GP 
Sbjct  117  LLMSQAVIPTMRAQKSGSIVCISSVSAQRGGGIFGGPHYSAAKAGVLGLARAMARELGPD  176

Query  56   SVRVSDVE-GVLQ  67
            +VRV+ +  G++Q
Sbjct  177  NVRVNCITPGLIQ  189


>ref|WP_004130709.1| short-chain dehydrogenase [Klebsiella oxytoca]
 gb|EHT06246.1| hypothetical protein HMPREF9694_04925 [Klebsiella oxytoca 10-5250]
Length=249

 Score = 37.4 bits (85),  Expect = 1.9, Method: Compositional matrix adjust.
 Identities = 23/73 (32%), Positives = 38/73 (52%), Gaps = 10/73 (14%)

Query  5    LLSSRTFSPTLRSRRSSPL---------EGGDVFAGAHTSAASKHAVCQAEGLSAAPGPQ  55
            LL S+   PT+R+++S  +          GG +F G H SAA    +  A  ++   GP 
Sbjct  117  LLMSQAVIPTMRAQKSGSIVCISSVSAQRGGGIFGGPHYSAAKAGVLGLARAMARELGPD  176

Query  56   SVRVSDVE-GVLQ  67
            +VRV+ +  G++Q
Sbjct  177  NVRVNCITPGLIQ  189


>ref|WP_040222529.1| short-chain dehydrogenase [Klebsiella pneumoniae]
Length=249

 Score = 37.4 bits (85),  Expect = 1.9, Method: Compositional matrix adjust.
 Identities = 23/73 (32%), Positives = 38/73 (52%), Gaps = 10/73 (14%)

Query  5    LLSSRTFSPTLRSRRSSPL---------EGGDVFAGAHTSAASKHAVCQAEGLSAAPGPQ  55
            LL S+   PT+R+++S  +          GG +F G H SAA    +  A  ++   GP 
Sbjct  117  LLMSQAVIPTMRAQKSGSIVCISSVSAQRGGGIFGGPHYSAAKAGVLGLARAMARELGPD  176

Query  56   SVRVSDVE-GVLQ  67
            +VRV+ +  G++Q
Sbjct  177  NVRVNCITPGLIQ  189


>ref|WP_032445768.1| MULTISPECIES: short-chain dehydrogenase [Enterobacteriaceae]
 emb|CDI18877.1| Uncharacterized short-chain type dehydrogenase/reductase y4mP 
[Klebsiella pneumoniae subsp. pneumoniae BJ1-GA]
Length=249

 Score = 37.4 bits (85),  Expect = 1.9, Method: Compositional matrix adjust.
 Identities = 23/73 (32%), Positives = 38/73 (52%), Gaps = 10/73 (14%)

Query  5    LLSSRTFSPTLRSRRSSPL---------EGGDVFAGAHTSAASKHAVCQAEGLSAAPGPQ  55
            LL S+   PT+R+++S  +          GG +F G H SAA    +  A  ++   GP 
Sbjct  117  LLMSQAVIPTMRAQKSGSIVCISSVSAQRGGGIFGGPHYSAAKAGVLGLARAMARELGPD  176

Query  56   SVRVSDVE-GVLQ  67
            +VRV+ +  G++Q
Sbjct  177  NVRVNCITPGLIQ  189


>ref|WP_004223790.1| short-chain dehydrogenase [Klebsiella pneumoniae]
 gb|AEJ97307.1| putative short-chain dehydrogenase/reductase SDR [Klebsiella 
pneumoniae KCTC 2242]
 emb|CCN28882.1| oxidoreductase, short chain dehydrogenase/reductase family protein 
[Klebsiella pneumoniae subsp. pneumoniae Ecl8]
 gb|EPF44196.1| putative short-chain dehydrogenase/reductase SDR [Klebsiella 
pneumoniae subsp. pneumoniae B5055]
 emb|CDI18455.1| Uncharacterized short-chain type dehydrogenase/reductase y4mP 
[Klebsiella pneumoniae subsp. pneumoniae T69]
 emb|CDO13632.1| unnamed protein product [Klebsiella pneumoniae]
Length=249

 Score = 37.4 bits (85),  Expect = 1.9, Method: Compositional matrix adjust.
 Identities = 23/73 (32%), Positives = 38/73 (52%), Gaps = 10/73 (14%)

Query  5    LLSSRTFSPTLRSRRSSPL---------EGGDVFAGAHTSAASKHAVCQAEGLSAAPGPQ  55
            LL S+   PT+R+++S  +          GG +F G H SAA    +  A  ++   GP 
Sbjct  117  LLMSQAVIPTMRAQKSGSIVCISSVSAQRGGGIFGGPHYSAAKAGVLGLARAMARELGPD  176

Query  56   SVRVSDVE-GVLQ  67
            +VRV+ +  G++Q
Sbjct  177  NVRVNCITPGLIQ  189


>dbj|BAS36430.1| 3-oxoacyl-ACP reductase [Klebsiella pneumoniae]
Length=249

 Score = 37.4 bits (85),  Expect = 1.9, Method: Compositional matrix adjust.
 Identities = 23/73 (32%), Positives = 38/73 (52%), Gaps = 10/73 (14%)

Query  5    LLSSRTFSPTLRSRRSSPL---------EGGDVFAGAHTSAASKHAVCQAEGLSAAPGPQ  55
            LL S+   PT+R+++S  +          GG +F G H SAA    +  A  ++   GP 
Sbjct  117  LLMSQAVIPTMRAQKSGSIVCISSVSAQRGGGIFGGPHYSAAKAGVLGLARAMARELGPD  176

Query  56   SVRVSDVE-GVLQ  67
            +VRV+ +  G++Q
Sbjct  177  NVRVNCITPGLIQ  189


>ref|WP_048254336.1| MULTISPECIES: short-chain dehydrogenase [Enterobacteriaceae]
 gb|KLZ72120.1| short-chain dehydrogenase/reductase SDR [Klebsiella pneumoniae]
Length=249

 Score = 37.4 bits (85),  Expect = 1.9, Method: Compositional matrix adjust.
 Identities = 23/73 (32%), Positives = 38/73 (52%), Gaps = 10/73 (14%)

Query  5    LLSSRTFSPTLRSRRSSPL---------EGGDVFAGAHTSAASKHAVCQAEGLSAAPGPQ  55
            LL S+   PT+R+++S  +          GG +F G H SAA    +  A  ++   GP 
Sbjct  117  LLMSQAVIPTMRAQKSGSIVCISSVSAQRGGGIFGGPHYSAAKAGVLGLARAMARELGPD  176

Query  56   SVRVSDVE-GVLQ  67
            +VRV+ +  G++Q
Sbjct  177  NVRVNCITPGLIQ  189


>ref|WP_012542384.1| short-chain dehydrogenase [Klebsiella pneumoniae]
 gb|ACI11242.1| oxidoreductase, short chain dehydrogenase/reductase family protein 
[Klebsiella pneumoniae 342]
Length=249

 Score = 37.4 bits (85),  Expect = 1.9, Method: Compositional matrix adjust.
 Identities = 23/73 (32%), Positives = 38/73 (52%), Gaps = 10/73 (14%)

Query  5    LLSSRTFSPTLRSRRSSPL---------EGGDVFAGAHTSAASKHAVCQAEGLSAAPGPQ  55
            LL S+   PT+R+++S  +          GG +F G H SAA    +  A  ++   GP 
Sbjct  117  LLMSQAVIPTMRAQKSGSIVCISSVSAQRGGGIFGGPHYSAAKAGVLGLARAMARELGPD  176

Query  56   SVRVSDVE-GVLQ  67
            +VRV+ +  G++Q
Sbjct  177  NVRVNCITPGLIQ  189


>ref|WP_023341222.1| short-chain dehydrogenase/reductase SDR [Klebsiella pneumoniae]
 gb|ESN41840.1| short-chain dehydrogenase/reductase SDR [Klebsiella pneumoniae 
MGH 19]
Length=249

 Score = 37.4 bits (85),  Expect = 1.9, Method: Compositional matrix adjust.
 Identities = 23/73 (32%), Positives = 38/73 (52%), Gaps = 10/73 (14%)

Query  5    LLSSRTFSPTLRSRRSSPL---------EGGDVFAGAHTSAASKHAVCQAEGLSAAPGPQ  55
            LL S+   PT+R+++S  +          GG +F G H SAA    +  A  ++   GP 
Sbjct  117  LLMSQAVIPTMRAQKSGSIVCISSVSAQRGGGIFGGPHYSAAKAGVLGLARAMARELGPD  176

Query  56   SVRVSDVE-GVLQ  67
            +VRV+ +  G++Q
Sbjct  177  NVRVNCITPGLIQ  189


>ref|WP_012068480.1| short-chain dehydrogenase [Klebsiella pneumoniae]
 gb|ABR76263.1| putative short-chain dehydrogenase/reductase SDR [Klebsiella 
pneumoniae subsp. pneumoniae MGH 78578]
Length=249

 Score = 37.4 bits (85),  Expect = 1.9, Method: Compositional matrix adjust.
 Identities = 23/73 (32%), Positives = 38/73 (52%), Gaps = 10/73 (14%)

Query  5    LLSSRTFSPTLRSRRSSPL---------EGGDVFAGAHTSAASKHAVCQAEGLSAAPGPQ  55
            LL S+   PT+R+++S  +          GG +F G H SAA    +  A  ++   GP 
Sbjct  117  LLMSQAVIPTMRAQKSGSIVCISSVSAQRGGGIFGGPHYSAAKAGVLGLARAMARELGPD  176

Query  56   SVRVSDVE-GVLQ  67
            +VRV+ +  G++Q
Sbjct  177  NVRVNCITPGLIQ  189


>ref|WP_048333020.1| short-chain dehydrogenase [Klebsiella pneumoniae]
 gb|KMH79195.1| short-chain dehydrogenase/reductase SDR [Klebsiella pneumoniae]
Length=249

 Score = 37.4 bits (85),  Expect = 1.9, Method: Compositional matrix adjust.
 Identities = 23/73 (32%), Positives = 38/73 (52%), Gaps = 10/73 (14%)

Query  5    LLSSRTFSPTLRSRRSSPL---------EGGDVFAGAHTSAASKHAVCQAEGLSAAPGPQ  55
            LL S+   PT+R+++S  +          GG +F G H SAA    +  A  ++   GP 
Sbjct  117  LLMSQAVIPTMRAQKSGSIVCISSVSAQRGGGIFGGPHYSAAKAGVLGLARAMARELGPD  176

Query  56   SVRVSDVE-GVLQ  67
            +VRV+ +  G++Q
Sbjct  177  NVRVNCITPGLIQ  189


>ref|WP_008805763.1| MULTISPECIES: short-chain dehydrogenase [Enterobacteriaceae]
 gb|ADC59434.1| short-chain dehydrogenase/reductase SDR [Klebsiella variicola 
At-22]
 gb|EFD85252.1| putative 3-oxoacyl-[acyl-carrier-protein] reductase [Klebsiella 
sp. 1_1_55]
 gb|ESM74058.1| short-chain dehydrogenase/reductase SDR [Klebsiella pneumoniae 
MGH 40]
 gb|AJA96309.1| short-chain dehydrogenase [Klebsiella variicola]
 emb|CEP31365.1| Uncharacterized short-chain type dehydrogenase/reductase y4mP 
[Klebsiella variicola]
 gb|KMH22195.1| short-chain dehydrogenase/reductase SDR [Klebsiella pneumoniae]
 emb|CTP99912.1| Uncharacterized short-chain type dehydrogenase/reductase y4mP 
[Klebsiella variicola]
Length=249

 Score = 37.4 bits (85),  Expect = 1.9, Method: Compositional matrix adjust.
 Identities = 23/73 (32%), Positives = 38/73 (52%), Gaps = 10/73 (14%)

Query  5    LLSSRTFSPTLRSRRSSPL---------EGGDVFAGAHTSAASKHAVCQAEGLSAAPGPQ  55
            LL S+   PT+R+++S  +          GG +F G H SAA    +  A  ++   GP 
Sbjct  117  LLMSQAVIPTMRAQKSGSIVCISSVSAQRGGGIFGGPHYSAAKAGVLGLARAMARELGPD  176

Query  56   SVRVSDVE-GVLQ  67
            +VRV+ +  G++Q
Sbjct  177  NVRVNCITPGLIQ  189


>ref|WP_023287402.1| short-chain dehydrogenase/reductase SDR [Klebsiella pneumoniae]
 gb|ESL65607.1| short-chain dehydrogenase/reductase SDR [Klebsiella pneumoniae 
BIDMC 22]
Length=249

 Score = 37.4 bits (85),  Expect = 1.9, Method: Compositional matrix adjust.
 Identities = 23/73 (32%), Positives = 38/73 (52%), Gaps = 10/73 (14%)

Query  5    LLSSRTFSPTLRSRRSSPL---------EGGDVFAGAHTSAASKHAVCQAEGLSAAPGPQ  55
            LL S+   PT+R+++S  +          GG +F G H SAA    +  A  ++   GP 
Sbjct  117  LLMSQAVIPTMRAQKSGSIVCISSVSAQRGGGIFGGPHYSAAKAGVLGLARAMARELGPD  176

Query  56   SVRVSDVE-GVLQ  67
            +VRV+ +  G++Q
Sbjct  177  NVRVNCITPGLIQ  189


>ref|WP_017900162.1| MULTISPECIES: short-chain dehydrogenase [Klebsiella]
 gb|KMH84721.1| short-chain dehydrogenase/reductase SDR [Klebsiella pneumoniae]
 gb|ALD07603.1| short-chain dehydrogenase [Klebsiella variicola]
 gb|ALD56887.1| short-chain dehydrogenase [Klebsiella pneumoniae subsp. pneumoniae]
Length=249

 Score = 37.4 bits (85),  Expect = 1.9, Method: Compositional matrix adjust.
 Identities = 23/73 (32%), Positives = 38/73 (52%), Gaps = 10/73 (14%)

Query  5    LLSSRTFSPTLRSRRSSPL---------EGGDVFAGAHTSAASKHAVCQAEGLSAAPGPQ  55
            LL S+   PT+R+++S  +          GG +F G H SAA    +  A  ++   GP 
Sbjct  117  LLMSQAVIPTMRAQKSGSIVCISSVSAQRGGGIFGGPHYSAAKAGVLGLARAMARELGPD  176

Query  56   SVRVSDVE-GVLQ  67
            +VRV+ +  G++Q
Sbjct  177  NVRVNCITPGLIQ  189


>ref|WP_004886175.1| MULTISPECIES: 3-oxoacyl-ACP reductase [Enterobacteriaceae]
 gb|EMR23465.1| 3-oxoacyl-ACP reductase [Klebsiella pneumoniae 700603]
 gb|ESM59459.1| short-chain dehydrogenase/reductase SDR [Klebsiella pneumoniae 
MGH 44]
 emb|CDN05701.1| Uncharacterized short-chain type dehydrogenase/reductase y4mP 
[Klebsiella quasipneumoniae subsp. similipneumoniae]
 gb|KII56717.1| short-chain dehydrogenase [Klebsiella pneumoniae]
 emb|CEL81534.1| short chain dehydrogenase/reductase family oxidoreductase [Klebsiella 
pneumoniae ATCC 43816]
 gb|KMI36189.1| short-chain dehydrogenase/reductase SDR [Klebsiella pneumoniae]
 gb|KMI94327.1| short-chain dehydrogenase/reductase SDR [Klebsiella pneumoniae]
Length=249

 Score = 37.4 bits (85),  Expect = 1.9, Method: Compositional matrix adjust.
 Identities = 23/73 (32%), Positives = 38/73 (52%), Gaps = 10/73 (14%)

Query  5    LLSSRTFSPTLRSRRSSPL---------EGGDVFAGAHTSAASKHAVCQAEGLSAAPGPQ  55
            LL S+   PT+R+++S  +          GG +F G H SAA    +  A  ++   GP 
Sbjct  117  LLMSQAVIPTMRAQKSGSIVCISSVSAQRGGGIFGGPHYSAAKAGVLGLARAMARELGPD  176

Query  56   SVRVSDVE-GVLQ  67
            +VRV+ +  G++Q
Sbjct  177  NVRVNCITPGLIQ  189


>ref|WP_048333495.1| short-chain dehydrogenase [Klebsiella pneumoniae]
 gb|KMG99648.1| short-chain dehydrogenase/reductase SDR [Klebsiella pneumoniae]
Length=249

 Score = 37.4 bits (85),  Expect = 2.0, Method: Compositional matrix adjust.
 Identities = 23/73 (32%), Positives = 38/73 (52%), Gaps = 10/73 (14%)

Query  5    LLSSRTFSPTLRSRRSSPL---------EGGDVFAGAHTSAASKHAVCQAEGLSAAPGPQ  55
            LL S+   PT+R+++S  +          GG +F G H SAA    +  A  ++   GP 
Sbjct  117  LLMSQAVIPTMRAQKSGSIVCISSVSAQRGGGIFGGPHYSAAKAGVLGLARAMARELGPD  176

Query  56   SVRVSDVE-GVLQ  67
            +VRV+ +  G++Q
Sbjct  177  NVRVNCITPGLIQ  189


>ref|WP_048268970.1| short-chain dehydrogenase [Klebsiella pneumoniae]
 gb|AKG98370.1| short-chain dehydrogenase [Klebsiella pneumoniae]
 gb|KME98065.1| short-chain dehydrogenase/reductase SDR [Klebsiella pneumoniae]
Length=249

 Score = 37.4 bits (85),  Expect = 2.0, Method: Compositional matrix adjust.
 Identities = 23/73 (32%), Positives = 38/73 (52%), Gaps = 10/73 (14%)

Query  5    LLSSRTFSPTLRSRRSSPL---------EGGDVFAGAHTSAASKHAVCQAEGLSAAPGPQ  55
            LL S+   PT+R+++S  +          GG +F G H SAA    +  A  ++   GP 
Sbjct  117  LLMSQAVIPTMRAQKSGSIVCISSVSAQRGGGIFGGPHYSAAKAGVLGLARAMARELGPD  176

Query  56   SVRVSDVE-GVLQ  67
            +VRV+ +  G++Q
Sbjct  177  NVRVNCITPGLIQ  189


>ref|WP_045343778.1| short-chain dehydrogenase [Enterobacter cloacae]
 gb|KJN25732.1| short-chain dehydrogenase [Enterobacter cloacae]
 gb|KLG04564.1| short-chain dehydrogenase [Enterobacter cloacae]
Length=249

 Score = 37.4 bits (85),  Expect = 2.0, Method: Compositional matrix adjust.
 Identities = 23/73 (32%), Positives = 38/73 (52%), Gaps = 10/73 (14%)

Query  5    LLSSRTFSPTLRSRRSSPL---------EGGDVFAGAHTSAASKHAVCQAEGLSAAPGPQ  55
            LL S+   PT+R ++S  +          GG +F G H SAA    +  A+ ++   GP 
Sbjct  117  LLMSQAVIPTMRVQKSGSIVCISSVSAQRGGGIFGGPHYSAAKAGVLGLAKAMARELGPD  176

Query  56   SVRVSDVE-GVLQ  67
            +VRV+ +  G++Q
Sbjct  177  NVRVNCITPGLIQ  189


>ref|WP_043875587.1| short-chain dehydrogenase [Klebsiella variicola]
 emb|CEL86235.1| Uncharacterized short-chain type dehydrogenase/reductase y4mP 
[Klebsiella variicola]
Length=249

 Score = 37.4 bits (85),  Expect = 2.0, Method: Compositional matrix adjust.
 Identities = 23/73 (32%), Positives = 38/73 (52%), Gaps = 10/73 (14%)

Query  5    LLSSRTFSPTLRSRRSSPL---------EGGDVFAGAHTSAASKHAVCQAEGLSAAPGPQ  55
            LL S+   PT+R+++S  +          GG +F G H SAA    +  A  ++   GP 
Sbjct  117  LLMSQAVIPTMRAQKSGSIVCISSVSAQRGGGIFGGPHYSAAKAGVLGLARAMARELGPD  176

Query  56   SVRVSDVE-GVLQ  67
            +VRV+ +  G++Q
Sbjct  177  NVRVNCITPGLIQ  189


>ref|WP_004135219.1| MULTISPECIES: putative short-chain dehydrogenase/reductase SDR 
[Enterobacteriaceae]
 gb|EKP27924.1| putative short-chain dehydrogenase/reductase SDR [Klebsiella 
oxytoca M5al]
Length=249

 Score = 37.4 bits (85),  Expect = 2.0, Method: Compositional matrix adjust.
 Identities = 23/73 (32%), Positives = 38/73 (52%), Gaps = 10/73 (14%)

Query  5    LLSSRTFSPTLRSRRSSPL---------EGGDVFAGAHTSAASKHAVCQAEGLSAAPGPQ  55
            LL S+   PT+R+++S  +          GG +F G H SAA    +  A  ++   GP 
Sbjct  117  LLMSQAVIPTMRAQKSGSIVCISSVSAQRGGGIFGGPHYSAAKAGVLGLARAMARELGPD  176

Query  56   SVRVSDVE-GVLQ  67
            +VRV+ +  G++Q
Sbjct  177  NVRVNCITPGLIQ  189


>ref|WP_002895750.1| MULTISPECIES: short-chain dehydrogenase [Enterobacteriaceae]
 ref|YP_005225956.1| putative short-chain dehydrogenase/reductase SDR [Klebsiella 
pneumoniae subsp. pneumoniae HS11286]
 dbj|BAH62517.1| putative short-chain dehydrogenase/reductase SDR [Klebsiella 
pneumoniae subsp. pneumoniae NTUH-K2044]
 545 more sequence titles

gb|EEW42619.1| oxidoreductase, short chain dehydrogenase/reductase family protein 
[Klebsiella pneumoniae subsp. rhinoscleromatis ATCC 13884]
 gb|EGF61228.1| putative 3-oxoacyl-[acyl-carrier-protein] reductase [Klebsiella 
sp. MS 92-3]
 gb|EHL93407.1| hypothetical protein HMPREF1024_01691 [Klebsiella sp. 4_1_44FAA]
 gb|AEW60354.1| putative short-chain dehydrogenase/reductase SDR [Klebsiella 
pneumoniae subsp. pneumoniae HS11286]
 gb|EJJ39093.1| putative short-chain dehydrogenase/reductase SDR [Klebsiella 
pneumoniae subsp. pneumoniae KPNIH4]
 gb|EJJ46423.1| putative short-chain dehydrogenase/reductase SDR [Klebsiella 
pneumoniae subsp. pneumoniae KPNIH2]
 gb|EJJ54219.1| putative short-chain dehydrogenase/reductase SDR [Klebsiella 
pneumoniae subsp. pneumoniae KPNIH6]
 gb|EJJ56368.1| putative short-chain dehydrogenase/reductase SDR [Klebsiella 
pneumoniae subsp. pneumoniae KPNIH5]
 gb|EJJ60853.1| putative short-chain dehydrogenase/reductase SDR [Klebsiella 
pneumoniae subsp. pneumoniae KPNIH7]
 gb|EJJ73590.1| putative short-chain dehydrogenase/reductase SDR [Klebsiella 
pneumoniae subsp. pneumoniae KPNIH9]
 gb|EJJ77843.1| putative short-chain dehydrogenase/reductase SDR [Klebsiella 
pneumoniae subsp. pneumoniae KPNIH8]
 gb|EJJ91104.1| putative short-chain dehydrogenase/reductase SDR [Klebsiella 
pneumoniae subsp. pneumoniae KPNIH11]
 gb|EJJ92436.1| putative short-chain dehydrogenase/reductase SDR [Klebsiella 
pneumoniae subsp. pneumoniae KPNIH12]
 gb|EJJ95437.1| putative short-chain dehydrogenase/reductase SDR [Klebsiella 
pneumoniae subsp. pneumoniae KPNIH14]
 gb|EJK08623.1| putative short-chain dehydrogenase/reductase SDR [Klebsiella 
pneumoniae subsp. pneumoniae KPNIH16]
 gb|EJK08915.1| putative short-chain dehydrogenase/reductase SDR [Klebsiella 
pneumoniae subsp. pneumoniae KPNIH17]
 gb|EJK12009.1| putative short-chain dehydrogenase/reductase SDR [Klebsiella 
pneumoniae subsp. pneumoniae KPNIH18]
 gb|EJK21318.1| putative short-chain dehydrogenase/reductase SDR [Klebsiella 
pneumoniae subsp. pneumoniae KPNIH19]
 gb|EJK24413.1| putative short-chain dehydrogenase/reductase SDR [Klebsiella 
pneumoniae subsp. pneumoniae KPNIH20]
 gb|EJK33119.1| putative short-chain dehydrogenase/reductase SDR [Klebsiella 
pneumoniae subsp. pneumoniae KPNIH21]
 gb|EJK35421.1| putative short-chain dehydrogenase/reductase SDR [Klebsiella 
pneumoniae subsp. pneumoniae KPNIH22]
 gb|EJK40243.1| putative short-chain dehydrogenase/reductase SDR [Klebsiella 
pneumoniae subsp. pneumoniae KPNIH23]
 gb|AFQ66686.1| 3-oxoacyl-[acyl-carrier protein] reductase [Klebsiella pneumoniae 
subsp. pneumoniae 1084]
 gb|EKB66691.1| hypothetical protein HMPREF1305_03446 [Klebsiella pneumoniae 
subsp. pneumoniae WGLW1]
 gb|EKB75437.1| hypothetical protein HMPREF1307_03615 [Klebsiella pneumoniae 
subsp. pneumoniae WGLW3]
 gb|EKB78774.1| hypothetical protein HMPREF1306_01380 [Klebsiella pneumoniae 
subsp. pneumoniae WGLW2]
 gb|EKB86404.1| hypothetical protein HMPREF1308_00954 [Klebsiella pneumoniae 
subsp. pneumoniae WGLW5]
 gb|EKF79772.1| Putative short-chain dehydrogenase/reductase SDR [Klebsiella 
pneumoniae subsp. pneumoniae KpQ3]
 emb|CCM81800.1| 3-oxoacyl-[acyl-carrier protein] reductase [Klebsiella pneumoniae 
subsp. pneumoniae ST258-K26BO]
 emb|CCM90398.1| 3-oxoacyl-[acyl-carrier protein] reductase [Klebsiella pneumoniae 
subsp. pneumoniae ST258-K28BO]
 emb|CCM95374.1| 3-oxoacyl-[acyl-carrier protein] reductase [Klebsiella pneumoniae 
subsp. pneumoniae ST512-K30BO]
 gb|EMB10870.1| 3-oxoacyl-ACP reductase [Klebsiella pneumoniae hvKP1]
 gb|EMH93828.1| short chain dehydrogenase family protein [Klebsiella pneumoniae 
JHCK1]
 gb|EMH98832.1| 3-oxoacyl-ACP reductase [Klebsiella pneumoniae RYC492]
 gb|EMI39121.1| short chain dehydrogenase family protein [Klebsiella pneumoniae 
VA360]
 gb|EMR25564.1| 3-oxoacyl-ACP reductase [Klebsiella pneumoniae ATCC BAA-2146]
 gb|EMR26909.1| 3-oxoacyl-ACP reductase [Klebsiella pneumoniae ATCC BAA-1705]
 gb|ENY58271.1| putative short-chain dehydrogenase/reductase SDR [Klebsiella 
pneumoniae subsp. pneumoniae KpMDU1]
 emb|CCI77989.1| unnamed protein product [Klebsiella pneumoniae subsp. rhinoscleromatis 
SB3432]
 gb|EOR17783.1| KR domain protein [Klebsiella pneumoniae UHKPC23]
 gb|EOY64075.1| KR domain protein [Klebsiella pneumoniae KP-7]
 gb|EOY70951.1| KR domain protein [Klebsiella pneumoniae UHKPC40]
 gb|EOY77446.1| KR domain protein [Klebsiella pneumoniae UHKPC81]
 gb|EOY79960.1| KR domain protein [Klebsiella pneumoniae UHKPC09]
 gb|EOY89118.1| KR domain protein [Klebsiella pneumoniae UHKPC27]
 gb|EOY89475.1| KR domain protein [Klebsiella pneumoniae UHKPC01]
 gb|EOY96111.1| KR domain protein [Klebsiella pneumoniae UHKPC26]
 gb|EOY98190.1| KR domain protein [Klebsiella pneumoniae UHKPC24]
 gb|EOZ14121.1| KR domain protein [Klebsiella pneumoniae VAKPC252]
 gb|EOZ17408.1| KR domain protein [Klebsiella pneumoniae UHKPC22]
 gb|EOZ20140.1| KR domain protein [Klebsiella pneumoniae UHKPC04]
 gb|EOZ25892.1| KR domain protein [Klebsiella pneumoniae VAKPC269]
 gb|EOZ28759.1| KR domain protein [Klebsiella pneumoniae VAKPC254]
 gb|EOZ32724.1| KR domain protein [Klebsiella pneumoniae VAKPC280]
 gb|EOZ36883.1| KR domain protein [Klebsiella pneumoniae VAKPC270]
 gb|EOZ44520.1| KR domain protein [Klebsiella pneumoniae VAKPC276]
 gb|EOZ46472.1| KR domain protein [Klebsiella pneumoniae VAKPC297]
 gb|EOZ53823.1| KR domain protein [Klebsiella pneumoniae VAKPC309]
 gb|EOZ63971.1| KR domain protein [Klebsiella pneumoniae KP-11]
 gb|EOZ68255.1| KR domain protein [Klebsiella pneumoniae 361_1301]
 gb|EOZ71678.1| KR domain protein [Klebsiella pneumoniae 440_1540]
 gb|EOZ88818.1| KR domain protein [Klebsiella pneumoniae 540_1460]
 gb|EOZ98975.1| KR domain protein [Klebsiella pneumoniae 646_1568]
 gb|EPA88116.1| KR domain protein [Klebsiella pneumoniae UHKPC57]
 gb|EPB04168.1| KR domain protein [Klebsiella pneumoniae UHKPC 52]
 gb|EPB12216.1| KR domain protein [Klebsiella pneumoniae UHKPC05]
 gb|EPB15884.1| KR domain protein [Klebsiella pneumoniae UHKPC45]
 gb|EPB19198.1| KR domain protein [Klebsiella pneumoniae UHKPC29]
 gb|EPB26614.1| KR domain protein [Klebsiella pneumoniae VAKPC278]
 gb|EPB31329.1| KR domain protein [Klebsiella pneumoniae DMC0526]
 gb|EPB35463.1| KR domain protein [Klebsiella pneumoniae UHKPC48]
 gb|EPB38648.1| KR domain protein [Klebsiella pneumoniae UHKPC32]
 gb|EPN93142.1| KR domain protein [Klebsiella pneumoniae UHKPC28]
 gb|EPN96088.1| KR domain protein [Klebsiella pneumoniae UHKPC47]
 gb|EPN98764.1| KR domain protein [Klebsiella pneumoniae UHKPC69]
 gb|EPO03128.1| KR domain protein [Klebsiella pneumoniae UHKPC77]
 gb|EPO09751.1| KR domain protein [Klebsiella pneumoniae UHKPC96]
 gb|EPO15582.1| KR domain protein [Klebsiella pneumoniae DMC0799]
 gb|EPO28049.1| KR domain protein [Klebsiella pneumoniae UHKPC61]
 gb|EPO30723.1| KR domain protein [Klebsiella pneumoniae DMC1316]
 gb|EPO45697.1| KR domain protein [Klebsiella pneumoniae UHKPC59]
 gb|EPO56278.1| KR domain protein [Klebsiella pneumoniae UHKPC17]
 gb|EPO60551.1| KR domain protein [Klebsiella pneumoniae UHKPC18]
 gb|EPO64518.1| KR domain protein [Klebsiella pneumoniae UHKPC31]
 gb|EPO68629.1| KR domain protein [Klebsiella pneumoniae UHKPC06]
 gb|EPO79442.1| KR domain protein [Klebsiella pneumoniae UHKPC67]
 gb|EPO86475.1| KR domain protein [Klebsiella pneumoniae UHKPC02]
 gb|EPO90810.1| KR domain protein [Klebsiella pneumoniae UHKPC179]
 gb|EPO94439.1| KR domain protein [Klebsiella pneumoniae 160_1080]
 gb|EPP00752.1| KR domain protein [Klebsiella pneumoniae 120_1020]
 gb|EPP00906.1| KR domain protein [Klebsiella pneumoniae 140_1040]
 gb|EPP10188.1| KR domain protein [Klebsiella pneumoniae 280_1220]
 gb|EPS12061.1| putative 3-oxoacyl-[acyl-carrier-protein] reductase [Klebsiella 
pneumoniae subsp. pneumoniae UKKV901664]
 gb|EPS12628.1| putative 3-oxoacyl-[acyl-carrier-protein] reductase [Klebsiella 
pneumoniae subsp. pneumoniae MP14]
 gb|ERI54834.1| putative short-chain dehydrogenase/reductase [Klebsiella pneumoniae 
KP-1]
 gb|ERN60538.1| short-chain dehydrogenase [Klebsiella pneumoniae 303K]
 gb|AGX37075.1| 3-oxoacyl-ACP synthase [Klebsiella pneumoniae CG43]
 gb|ERO73825.1| short-chain dehydrogenase/reductase SDR [Klebsiella pneumoniae 
BIDMC 16]
 gb|ERO78797.1| short-chain dehydrogenase/reductase SDR [Klebsiella pneumoniae 
BIDMC 18C]
 gb|ERO84704.1| short-chain dehydrogenase/reductase SDR [Klebsiella pneumoniae 
BIDMC 12C]
 gb|ESA97477.1| putative 3-oxoacyl-[acyl-carrier-protein] reductase [Klebsiella 
pneumoniae 909957]
 gb|ESL26775.1| short-chain dehydrogenase/reductase SDR [Klebsiella pneumoniae 
BIDMC 41]
 gb|ESL41806.1| short-chain dehydrogenase/reductase SDR [Klebsiella pneumoniae 
BIDMC 36]
 gb|ESL52198.1| short-chain dehydrogenase/reductase SDR [Klebsiella pneumoniae 
BIDMC 25]
 gb|ESL60073.1| short-chain dehydrogenase/reductase SDR [Klebsiella pneumoniae 
BIDMC 23]
 gb|ESL73341.1| short-chain dehydrogenase/reductase SDR [Klebsiella pneumoniae 
BIDMC 21]
 gb|ESL99649.1| short-chain dehydrogenase/reductase SDR [Klebsiella pneumoniae 
UCICRE 7]
 gb|ESM06189.1| short-chain dehydrogenase/reductase SDR [Klebsiella pneumoniae 
UCICRE 6]
 gb|ESM22799.1| short-chain dehydrogenase/reductase SDR [Klebsiella pneumoniae 
UCICRE 4]
 gb|ESM25647.1| short-chain dehydrogenase/reductase SDR [Klebsiella pneumoniae 
UCICRE 2]
 gb|ESM42579.1| short-chain dehydrogenase/reductase SDR [Klebsiella pneumoniae 
BWH 30]
 gb|ESM51400.1| short-chain dehydrogenase/reductase SDR [Klebsiella pneumoniae 
BWH 28]
 gb|ESM57897.1| short-chain dehydrogenase/reductase SDR [Klebsiella pneumoniae 
MGH 48]
 gb|ESM68709.1| short-chain dehydrogenase/reductase SDR [Klebsiella pneumoniae 
MGH 46]
 gb|ESM93532.1| short-chain dehydrogenase/reductase SDR [Klebsiella pneumoniae 
MGH 36]
 gb|ESM95295.1| short-chain dehydrogenase/reductase SDR [Klebsiella pneumoniae 
MGH 32]
 gb|ESM96945.1| short-chain dehydrogenase/reductase SDR [Klebsiella pneumoniae 
MGH 30]
 gb|ESN44529.1| short-chain dehydrogenase/reductase SDR [Klebsiella pneumoniae 
MGH 21]
 gb|ESN49641.1| short-chain dehydrogenase/reductase SDR [Klebsiella pneumoniae 
MGH 18]
 gb|ESN60158.1| short-chain dehydrogenase/reductase SDR [Klebsiella pneumoniae 
MGH 17]
 emb|CDL58751.1| 3-oxoacyl-[acyl-carrier protein] reductase [Escherichia coli 
ISC56]
 emb|CDK78160.1| 3-oxoacyl-[acyl-carrier protein] reductase [Klebsiella pneumoniae 
IS22]
 emb|CDI19573.1| Uncharacterized short-chain type dehydrogenase/reductase y4mP 
[Klebsiella pneumoniae subsp. pneumoniae SA1]
 gb|AHI35129.1| 3-oxoacyl-[acyl-carrier protein] reductase [Klebsiella pneumoniae 
ATCC BAA-2146]
 gb|EWD03160.1| putative short-chain dehydrogenase/reductase SDR [Klebsiella 
pneumoniae NB60]
 gb|EWD16507.1| short-chain dehydrogenase/reductase SDR [Klebsiella pneumoniae 
UCI 43]
 gb|EWD18599.1| short-chain dehydrogenase/reductase SDR [Klebsiella pneumoniae 
UCI 42]
 gb|EWD20339.1| short-chain dehydrogenase/reductase SDR [Klebsiella pneumoniae 
UCI 44]
 gb|EWD27693.1| short-chain dehydrogenase/reductase SDR [Klebsiella pneumoniae 
UCI 38]
 gb|EWD29012.1| short-chain dehydrogenase/reductase SDR [Klebsiella pneumoniae 
UCI 41]
 gb|EWD38631.1| short-chain dehydrogenase/reductase SDR [Klebsiella pneumoniae 
UCI 37]
 gb|EWD40374.1| short-chain dehydrogenase/reductase SDR [Klebsiella pneumoniae 
UCI 34]
 gb|EWD50234.1| short-chain dehydrogenase/reductase SDR [Klebsiella pneumoniae 
UCI 26]
 gb|EWD51399.1| short-chain dehydrogenase/reductase SDR [Klebsiella pneumoniae 
UCI 33]
 gb|EWD57285.1| short-chain dehydrogenase/reductase SDR [Klebsiella pneumoniae 
UCI 25]
 gb|EWD63857.1| short-chain dehydrogenase/reductase SDR [Klebsiella pneumoniae 
UCI 22]
 gb|EWD68267.1| short-chain dehydrogenase/reductase SDR [Klebsiella pneumoniae 
UCI 21]
 gb|EWD70207.1| short-chain dehydrogenase/reductase SDR [Klebsiella pneumoniae 
UCI 19]
 gb|EWD77693.1| short-chain dehydrogenase/reductase SDR [Klebsiella pneumoniae 
UCI 20]
 gb|EWD86206.1| short-chain dehydrogenase/reductase SDR [Klebsiella pneumoniae 
BIDMC 45]
 gb|EWD97599.1| short-chain dehydrogenase/reductase SDR [Klebsiella pneumoniae 
BIDMC 52]
 gb|EWD99115.1| short-chain dehydrogenase/reductase SDR [Klebsiella pneumoniae 
BIDMC 53]
 gb|EWE06146.1| short-chain dehydrogenase/reductase SDR [Klebsiella pneumoniae 
BIDMC 51]
 gb|EWE10494.1| short-chain dehydrogenase/reductase SDR [Klebsiella pneumoniae 
BIDMC 48]
 gb|EWE17232.1| short-chain dehydrogenase/reductase SDR [Klebsiella pneumoniae 
BIDMC 47]
 gb|EWE18724.1| short-chain dehydrogenase/reductase SDR [Klebsiella pneumoniae 
BIDMC 46b]
 gb|EWE24074.1| short-chain dehydrogenase/reductase SDR [Klebsiella pneumoniae 
BIDMC 42b]
 gb|EWE26320.1| short-chain dehydrogenase/reductase SDR [Klebsiella pneumoniae 
BIDMC 42a]
 gb|EWE28783.1| short-chain dehydrogenase/reductase SDR [Klebsiella pneumoniae 
BIDMC 46a]
 gb|EWE38028.1| short-chain dehydrogenase/reductase SDR [Klebsiella pneumoniae 
BIDMC 34]
 gb|EWE42493.1| short-chain dehydrogenase/reductase SDR [Klebsiella pneumoniae 
BIDMC 35]
 gb|EWE49614.1| short-chain dehydrogenase/reductase SDR [Klebsiella pneumoniae 
BIDMC 32]
 gb|EWE53512.1| short-chain dehydrogenase/reductase SDR [Klebsiella pneumoniae 
BIDMC 18A]
 gb|EWE59628.1| short-chain dehydrogenase/reductase SDR [Klebsiella pneumoniae 
BIDMC 18D]
 gb|EWE65197.1| hypothetical protein L443_02188 [Klebsiella pneumoniae BIDMC 
14]
 gb|EWE69593.1| short-chain dehydrogenase/reductase SDR [Klebsiella pneumoniae 
BIDMC 13]
 gb|EWE77155.1| short-chain dehydrogenase/reductase SDR [Klebsiella pneumoniae 
BIDMC 12B]
 gb|EWE81621.1| short-chain dehydrogenase/reductase SDR [Klebsiella pneumoniae 
BIDMC 12A]
 gb|EWE89155.1| short-chain dehydrogenase/reductase SDR [Klebsiella pneumoniae 
BIDMC 11]
 gb|EWE89457.1| short-chain dehydrogenase/reductase SDR [Klebsiella pneumoniae 
BIDMC 5]
 gb|EWE97342.1| short-chain dehydrogenase/reductase SDR [Klebsiella pneumoniae 
BIDMC 7B]
 gb|EWF10661.1| short-chain dehydrogenase/reductase SDR [Klebsiella pneumoniae 
BIDMC 2A]
 gb|EWF11482.1| short-chain dehydrogenase/reductase SDR [Klebsiella pneumoniae 
BIDMC 4]
 gb|EWF13819.1| short-chain dehydrogenase/reductase SDR [Klebsiella pneumoniae 
BIDMC 1]
 gb|EWF18184.1| short-chain dehydrogenase/reductase SDR [Klebsiella pneumoniae 
UCICRE 1]
 gb|EWF24436.1| short-chain dehydrogenase/reductase SDR [Klebsiella pneumoniae 
BWH 36]
 gb|EWF28763.1| short-chain dehydrogenase/reductase SDR [Klebsiella pneumoniae 
BWH 41]
 gb|EWF36055.1| short-chain dehydrogenase/reductase SDR [Klebsiella pneumoniae 
BWH 22]
 gb|EWF39789.1| short-chain dehydrogenase/reductase SDR [Klebsiella pneumoniae 
BWH 2]
 gb|EWF43369.1| short-chain dehydrogenase/reductase SDR [Klebsiella pneumoniae 
MGH 47]
 gb|EWF46826.1| short-chain dehydrogenase/reductase SDR [Klebsiella pneumoniae 
BWH 15]
 gb|EWF52242.1| short-chain dehydrogenase/reductase SDR [Klebsiella pneumoniae 
MGH 43]
 gb|EWF63793.1| short-chain dehydrogenase/reductase SDR [Klebsiella pneumoniae 
MGH 45]
 gb|EWF75527.1| short-chain dehydrogenase/reductase SDR [Klebsiella pneumoniae 
MGH 39]
 gb|EWF77259.1| short-chain dehydrogenase/reductase SDR [Klebsiella pneumoniae 
MGH 35]
 gb|EWF88943.1| short-chain dehydrogenase/reductase SDR [Klebsiella pneumoniae 
MGH 31]
 gb|EWF95384.1| short-chain dehydrogenase/reductase SDR [Klebsiella pneumoniae 
MGH 29]
 gb|EWF97897.1| short-chain dehydrogenase/reductase SDR [Klebsiella pneumoniae 
UCICRE 13]
 gb|EWG04862.1| short-chain dehydrogenase/reductase SDR [Klebsiella pneumoniae 
BIDMC 7A]
 gb|EXF40112.1| short-chain dehydrogenase [Klebsiella pneumoniae EGD-HP19-C]
 gb|EYB73604.1| short chain dehydrogenase family protein [Klebsiella pneumoniae 
Kb677]
 gb|EYB79029.1| short chain dehydrogenase family protein [Klebsiella pneumoniae 
Kb140]
 gb|EZQ76077.1| short-chain dehydrogenase/reductase SDR [Klebsiella pneumoniae 
MGH 63]
 gb|EZQ79294.1| short-chain dehydrogenase/reductase SDR [Klebsiella pneumoniae 
CHS 60]
 gb|EZQ84771.1| short-chain dehydrogenase/reductase SDR [Klebsiella pneumoniae 
CHS 47]
 gb|EZQ91816.1| short-chain dehydrogenase/reductase SDR [Klebsiella pneumoniae 
CHS 21]
 gb|EZQ99339.1| short-chain dehydrogenase/reductase SDR [Klebsiella pneumoniae 
CHS 42]
 gb|EZR02311.1| short-chain dehydrogenase/reductase SDR [Klebsiella pneumoniae 
CHS 16]
 gb|EZR07740.1| short-chain dehydrogenase/reductase SDR [Klebsiella pneumoniae 
UCI 60]
 gb|EZR12096.1| short-chain dehydrogenase/reductase SDR [Klebsiella pneumoniae 
UCI 64]
 gb|EZR22123.1| short-chain dehydrogenase/reductase SDR [Klebsiella pneumoniae 
MGH 75]
 gb|KAI87173.1| short-chain dehydrogenase/reductase SDR [Klebsiella pneumoniae 
CHS 08]
 gb|KDG98694.1| hypothetical protein L449_04089 [Klebsiella pneumoniae BIDMC 
18B]
 gb|KDH11858.1| hypothetical protein AE35_04664 [Klebsiella pneumoniae BIDMC 
60]
 gb|KDH16532.1| hypothetical protein AE28_00678 [Klebsiella pneumoniae BIDMC 
54]
 gb|KDH16746.1| hypothetical protein AE29_00819 [Klebsiella pneumoniae BIDMC 
55]
 gb|KDH29067.1| hypothetical protein AE44_03489 [Klebsiella pneumoniae BIDMC 
69]
 gb|KDH29841.1| hypothetical protein AE43_03273 [Klebsiella pneumoniae BIDMC 
68]
 gb|KDH44082.1| hypothetical protein AE08_00917 [Klebsiella pneumoniae BWH 45]
 gb|KDH47280.1| hypothetical protein AE09_00625 [Klebsiella pneumoniae BWH 46]
 gb|KDH56012.1| hypothetical protein AE55_00833 [Klebsiella pneumoniae BWH 47]
 gb|KDH58198.1| hypothetical protein AE56_00695 [Klebsiella pneumoniae BWH 48]
 gb|KDH61258.1| hypothetical protein AE58_03799 [Klebsiella pneumoniae CHS 02]
 gb|KDH71560.1| hypothetical protein AE59_00918 [Klebsiella pneumoniae CHS 03]
 gb|KDH75235.1| hypothetical protein AE61_00924 [Klebsiella pneumoniae CHS 05]
 gb|KDH76872.1| hypothetical protein AE62_04023 [Klebsiella pneumoniae CHS 06]
 gb|KDH86982.1| hypothetical protein AE63_00702 [Klebsiella pneumoniae CHS 07]
 gb|KDH90920.1| hypothetical protein AE67_04445 [Klebsiella pneumoniae CHS 11]
 gb|KDH95677.1| hypothetical protein AE65_00911 [Klebsiella pneumoniae CHS 09]
 gb|KDI04001.1| hypothetical protein AE69_04022 [Klebsiella pneumoniae CHS 13]
 gb|KDI05424.1| hypothetical protein AE68_00929 [Klebsiella pneumoniae CHS 12]
 gb|KDI13702.1| hypothetical protein AE70_00702 [Klebsiella pneumoniae CHS 14]
 gb|KDI16066.1| hypothetical protein AE73_03747 [Klebsiella pneumoniae CHS 17]
 gb|KDI24413.1| hypothetical protein AE74_00925 [Klebsiella pneumoniae CHS 18]
 gb|KDI26469.1| hypothetical protein AE75_03581 [Klebsiella pneumoniae CHS 19]
 gb|KDI38038.1| hypothetical protein AE78_03279 [Klebsiella pneumoniae CHS 22]
 gb|KDI43302.1| hypothetical protein AE76_00511 [Klebsiella pneumoniae CHS 20]
 gb|KDI45311.1| hypothetical protein AE79_00916 [Klebsiella pneumoniae CHS 23]
 gb|KDI51851.1| hypothetical protein AE82_04675 [Klebsiella pneumoniae CHS 26]
 gb|KDI58159.1| hypothetical protein AE80_00913 [Klebsiella pneumoniae CHS 24]
 gb|KDI61825.1| hypothetical protein AE81_00700 [Klebsiella pneumoniae CHS 25]
 gb|KDI64811.1| hypothetical protein AE83_04678 [Klebsiella pneumoniae CHS 27]
 gb|KDI75490.1| hypothetical protein AE84_02881 [Klebsiella pneumoniae CHS 28]
 gb|KDI76792.1| hypothetical protein AE85_00920 [Klebsiella pneumoniae CHS 29]
 gb|KDI87397.1| hypothetical protein AE88_04570 [Klebsiella pneumoniae CHS 32]
 gb|KDI90105.1| hypothetical protein AE87_00696 [Klebsiella pneumoniae CHS 31]
 gb|KDI92948.1| hypothetical protein AE86_01074 [Klebsiella pneumoniae CHS 30]
 gb|KDJ00323.1| hypothetical protein AE89_03443 [Klebsiella pneumoniae CHS 33]
 gb|KDJ02805.1| hypothetical protein AE91_03754 [Klebsiella pneumoniae CHS 35]
 gb|KDJ12518.1| hypothetical protein AE90_00916 [Klebsiella pneumoniae CHS 34]
 gb|KDJ18741.1| hypothetical protein AE93_03933 [Klebsiella pneumoniae CHS 37]
 gb|KDJ20807.1| hypothetical protein AE92_00924 [Klebsiella pneumoniae CHS 36]
 gb|KDJ24512.1| hypothetical protein AE94_03662 [Klebsiella pneumoniae CHS 38]
 gb|KDJ30891.1| hypothetical protein AE95_04756 [Klebsiella pneumoniae CHS 39]
 gb|KDJ35719.1| hypothetical protein AE97_04761 [Klebsiella pneumoniae CHS 41]
 gb|KDJ38789.1| hypothetical protein AE96_03535 [Klebsiella pneumoniae CHS 40]
 gb|KDJ55013.1| hypothetical protein AF00_04177 [Klebsiella pneumoniae CHS 44]
 gb|KDJ56444.1| hypothetical protein AE99_00637 [Klebsiella pneumoniae CHS 43]
 gb|KDJ60755.1| hypothetical protein AF01_00699 [Klebsiella pneumoniae CHS 45]
 gb|KDJ63795.1| hypothetical protein AF02_04699 [Klebsiella pneumoniae CHS 46]
 gb|KDJ75278.1| hypothetical protein AF05_00919 [Klebsiella pneumoniae CHS 49]
 gb|KDJ78137.1| hypothetical protein AF04_00844 [Klebsiella pneumoniae CHS 48]
 gb|KDJ78866.1| hypothetical protein AF06_04283 [Klebsiella pneumoniae CHS 50]
 gb|KDJ85891.1| hypothetical protein AF07_03697 [Klebsiella pneumoniae CHS 51]
 gb|KDJ87299.1| hypothetical protein AF08_04730 [Klebsiella pneumoniae CHS 52]
 gb|KDK02165.1| hypothetical protein AF09_02303 [Klebsiella pneumoniae CHS 53]
 gb|KDK07310.1| hypothetical protein AF10_00922 [Klebsiella pneumoniae CHS 54]
 gb|KDK11085.1| hypothetical protein AF11_00896 [Klebsiella pneumoniae CHS 55]
 gb|KDK19665.1| hypothetical protein AF12_00700 [Klebsiella pneumoniae CHS 56]
 gb|KDK20709.1| hypothetical protein AF13_02732 [Klebsiella pneumoniae CHS 57]
 gb|KDK30116.1| hypothetical protein AF14_00920 [Klebsiella pneumoniae CHS 58]
 gb|KDK34152.1| hypothetical protein AF17_03844 [Klebsiella pneumoniae CHS 61]
 gb|KDK35097.1| hypothetical protein AF15_00919 [Klebsiella pneumoniae CHS 59]
 gb|KDK39575.1| hypothetical protein AF18_04756 [Klebsiella pneumoniae CHS 62]
 gb|KDK49350.1| hypothetical protein AF20_04751 [Klebsiella pneumoniae CHS 64]
 gb|KDK50615.1| hypothetical protein AF19_00921 [Klebsiella pneumoniae CHS 63]
 gb|KDK62137.1| hypothetical protein AF21_00700 [Klebsiella pneumoniae CHS 65]
 gb|KDK69435.1| hypothetical protein AF22_00612 [Klebsiella pneumoniae CHS 66]
 gb|KDK70900.1| hypothetical protein AF26_04288 [Klebsiella pneumoniae CHS 70]
 gb|KDK72694.1| hypothetical protein AF23_00926 [Klebsiella pneumoniae CHS 67]
 gb|KDK78725.1| hypothetical protein AF27_04268 [Klebsiella pneumoniae CHS 71]
 gb|KDK86227.1| hypothetical protein AF28_00921 [Klebsiella pneumoniae CHS 72]
 gb|KDK88660.1| hypothetical protein AF29_04266 [Klebsiella pneumoniae CHS 73]
 gb|KDL01114.1| hypothetical protein AF32_04270 [Klebsiella pneumoniae CHS 76]
 gb|KDL02016.1| hypothetical protein AF30_03341 [Klebsiella pneumoniae CHS 74]
 gb|KDL03470.1| hypothetical protein AF31_00926 [Klebsiella pneumoniae CHS 75]
 gb|KDL16530.1| hypothetical protein AF36_00922 [Klebsiella pneumoniae CHS 80]
 gb|KDL21833.1| hypothetical protein AF37_00920 [Klebsiella pneumoniae MGH 51]
 gb|KDL24129.1| hypothetical protein AF38_02224 [Klebsiella pneumoniae MGH 52]
 gb|KDL32038.1| hypothetical protein AF45_00427 [Klebsiella pneumoniae MGH 59]
 gb|KDL39005.1| hypothetical protein AF46_00642 [Klebsiella pneumoniae MGH 60]
 gb|KDL40851.1| hypothetical protein AF50_00844 [Klebsiella pneumoniae MGH 64]
 gb|KDL48491.1| hypothetical protein AF51_00678 [Klebsiella pneumoniae MGH 65]
 gb|KDL51980.1| hypothetical protein AF52_02253 [Klebsiella pneumoniae MGH 66]
 gb|KDL55355.1| hypothetical protein AD93_00697 [Klebsiella pneumoniae MGH 67]
 gb|KDL63184.1| hypothetical protein AD95_04486 [Klebsiella pneumoniae MGH 69]
 gb|KDL72033.1| hypothetical protein AD97_03823 [Klebsiella pneumoniae MGH 71]
 gb|KDL72896.1| hypothetical protein AD96_00715 [Klebsiella pneumoniae MGH 70]
 gb|KDL79554.1| hypothetical protein AD99_03977 [Klebsiella pneumoniae MGH 73]
 gb|KDL88077.1| hypothetical protein AD98_00834 [Klebsiella pneumoniae MGH 72]
 gb|KDL94765.1| hypothetical protein AE00_00700 [Klebsiella pneumoniae MGH 74]
 gb|KDL99106.1| hypothetical protein AE05_01538 [Klebsiella pneumoniae MGH 79]
 gb|KDM10750.1| hypothetical protein AE14_00923 [Klebsiella pneumoniae UCI 55]
 gb|KDM16387.1| hypothetical protein AE18_00781 [Klebsiella pneumoniae UCI 59]
 gb|KDM21212.1| hypothetical protein AE15_00841 [Klebsiella pneumoniae UCI 56]
 gb|KDM27124.1| hypothetical protein AE21_04090 [Klebsiella pneumoniae UCI 62]
 gb|KDM30748.1| hypothetical protein AE20_00700 [Klebsiella pneumoniae UCI 61]
 gb|KDM41607.1| hypothetical protein AE22_00629 [Klebsiella pneumoniae UCI 63]
 gb|KDM44366.1| hypothetical protein AE26_00916 [Klebsiella pneumoniae UCI 67]
 gb|KDM49800.1| hypothetical protein AE27_00647 [Klebsiella pneumoniae UCI 68]
 gb|AIA35927.1| short-chain dehydrogenase [Klebsiella pneumoniae subsp. pneumoniae 
KPNIH10]
 gb|AIA41223.1| short-chain dehydrogenase [Klebsiella pneumoniae subsp. pneumoniae 
KPNIH27]
 gb|KEC94393.1| short-chain dehydrogenase/reductase SDR [Klebsiella pneumoniae 
UCI 17]
 gb|KEC95610.1| short-chain dehydrogenase/reductase SDR [Klebsiella pneumoniae 
BIDMC 10]
 gb|KEF23991.1| short-chain dehydrogenase/reductase SDR [Klebsiella pneumoniae 
BIDMC 33B]
 gb|KEF67344.1| short-chain dehydrogenase [Klebsiella pneumoniae 160_1080]
 gb|KEF74135.1| short-chain dehydrogenase [Klebsiella pneumoniae UHKPC 52]
 gb|KEF78543.1| short-chain dehydrogenase [Klebsiella pneumoniae UHKPC45]
 gb|KEF82493.1| short-chain dehydrogenase [Klebsiella pneumoniae UHKPC05]
 gb|KEF89300.1| short-chain dehydrogenase [Klebsiella pneumoniae VAKPC278]
 gb|KEG37836.1| short-chain dehydrogenase [Klebsiella pneumoniae]
 gb|AID97544.1| short-chain dehydrogenase [Klebsiella pneumoniae subsp. pneumoniae 
KPNIH24]
 gb|AIE22239.1| short-chain dehydrogenase [Klebsiella pneumoniae subsp. pneumoniae 
KPNIH1]
 gb|AIE27617.1| short-chain dehydrogenase [Klebsiella pneumoniae subsp. pneumoniae 
KPR0928]
 gb|KEP98628.1| short-chain dehydrogenase [Klebsiella pneumoniae]
 gb|AIG82935.1| short-chain dehydrogenase [Klebsiella pneumoniae subsp. pneumoniae 
PittNDM01]
 gb|KFC45672.1| short-chain dehydrogenase [Klebsiella pneumoniae]
 gb|KFG17603.1| short-chain dehydrogenase [Klebsiella pneumoniae]
 gb|KFG17771.1| short-chain dehydrogenase [Klebsiella pneumoniae]
 gb|AIJ43896.1| short-chain dehydrogenase [Klebsiella pneumoniae]
 gb|KFJ73007.1| short chain dehydrogenase family protein [Klebsiella pneumoniae]
 gb|KGJ42339.1| short-chain dehydrogenase [Klebsiella pneumoniae]
 gb|KGJ43281.1| short-chain dehydrogenase [Klebsiella pneumoniae]
 gb|AIT02604.1| 3-oxoacyl-[acyl-carrier-protein] reductase FabG [Klebsiella pneumoniae]
 gb|KGK55037.1| short-chain dehydrogenase [Klebsiella pneumoniae]
 emb|CED74295.1| Uncharacterized short-chain type dehydrogenase/reductase y4mP 
[Klebsiella pneumoniae]
 gb|KGT61937.1| KR domain protein [Klebsiella pneumoniae MRSN 1319]
 gb|AIW70225.1| short-chain dehydrogenase [Klebsiella pneumoniae subsp. pneumoniae]
 gb|AIW75763.1| short-chain dehydrogenase [Klebsiella pneumoniae subsp. pneumoniae]
 gb|AIX00600.1| short-chain dehydrogenase [Klebsiella pneumoniae]
 gb|KGY33319.1| short-chain dehydrogenase [Klebsiella pneumoniae]
 gb|AIX68586.1| short-chain dehydrogenase [Klebsiella pneumoniae subsp. pneumoniae]
 gb|AIX78268.1| short-chain dehydrogenase [Klebsiella pneumoniae subsp. pneumoniae]
 gb|AIX83631.1| short-chain dehydrogenase [Klebsiella pneumoniae subsp. pneumoniae]
 gb|KHF57842.1| putative short-chain dehydrogenase/reductase SDR [Klebsiella 
pneumoniae]
 gb|KHF58421.1| putative short-chain dehydrogenase/reductase SDR [Klebsiella 
pneumoniae]
 gb|KHF67205.1| 3-oxoacyl-[acyl-carrier-protein] reductase FabG [Klebsiella pneumoniae 
subsp. pneumoniae]
 gb|KHF69155.1| putative short-chain dehydrogenase/reductase SDR [Klebsiella 
pneumoniae]
 gb|KHQ23191.1| short-chain dehydrogenase [Klebsiella pneumoniae]
 gb|KHQ28914.1| short-chain dehydrogenase [Klebsiella pneumoniae]
 gb|KHQ35269.1| short-chain dehydrogenase [Klebsiella pneumoniae]
 gb|AJB33341.1| 3-oxoacyl-[acyl-carrier protein] reductase [Klebsiella pneumoniae 
HK787]
 gb|AJB77047.1| short-chain dehydrogenase [Klebsiella pneumoniae]
 gb|AJB58610.1| short-chain dehydrogenase [Klebsiella pneumoniae]
 gb|AJC05530.1| 3-oxoacyl-[acyl-carrier protein] reductase [Klebsiella pneumoniae 
subsp. pneumoniae 1158]
 gb|KII07943.1| short-chain dehydrogenase [Klebsiella pneumoniae]
 emb|CEN63949.1| 3-oxoacyl-ACP reductase [Klebsiella pneumoniae]
 emb|CEO85522.1| 3-oxoacyl-ACP reductase [Klebsiella pneumoniae]
 gb|KJL10043.1| short-chain dehydrogenase [Klebsiella pneumoniae]
 gb|KJL12015.1| short-chain dehydrogenase [Klebsiella pneumoniae]
 gb|AKE76878.1| 3-oxoacyl-[acyl-carrier-protein] reductase FabG [Klebsiella pneumoniae 
subsp. pneumoniae]
 gb|KKJ16935.1| short-chain dehydrogenase [Klebsiella pneumoniae HE12]
 gb|KKJ22186.1| short-chain dehydrogenase [Klebsiella pneumoniae MRSN 6902]
 gb|KKJ28877.1| short-chain dehydrogenase [Klebsiella pneumoniae MRSN 3852]
 gb|KKJ38045.1| short-chain dehydrogenase [Klebsiella pneumoniae MRSN 3562]
 gb|KKJ63680.1| short-chain dehydrogenase [Klebsiella pneumoniae MRSN 2404]
 gb|KLA40934.1| short-chain dehydrogenase [Klebsiella pneumoniae subsp. pneumoniae]
 gb|AKK89499.1| short-chain dehydrogenase [Klebsiella pneumoniae]
 gb|AKL27746.1| short-chain dehydrogenase [Klebsiella pneumoniae]
 gb|AKL47294.1| short-chain dehydrogenase [Klebsiella pneumoniae]
 gb|KLY19293.1| short-chain dehydrogenase/reductase SDR [Klebsiella oxytoca]
 gb|KLY20562.1| short-chain dehydrogenase/reductase SDR [Klebsiella oxytoca]
 gb|KLY33633.1| short-chain dehydrogenase/reductase SDR [Klebsiella pneumoniae]
 gb|KLY45366.1| short-chain dehydrogenase/reductase SDR [Klebsiella pneumoniae]
 gb|KLY48615.1| short-chain dehydrogenase/reductase SDR [Klebsiella pneumoniae]
 gb|KLY53344.1| short-chain dehydrogenase/reductase SDR [Klebsiella pneumoniae]
 gb|KLY67338.1| short-chain dehydrogenase/reductase SDR [Klebsiella pneumoniae]
 gb|KLY68459.1| short-chain dehydrogenase/reductase SDR [Klebsiella pneumoniae]
 gb|KLY69865.1| short-chain dehydrogenase/reductase SDR [Klebsiella pneumoniae]
 gb|KLY77835.1| short-chain dehydrogenase/reductase SDR [Klebsiella pneumoniae]
 gb|KLY83569.1| short-chain dehydrogenase/reductase SDR [Klebsiella pneumoniae]
 gb|KLY85739.1| short-chain dehydrogenase/reductase SDR [Klebsiella pneumoniae]
 gb|KLY97209.1| short-chain dehydrogenase/reductase SDR [Klebsiella pneumoniae]
 gb|KLZ03801.1| short-chain dehydrogenase/reductase SDR [Klebsiella pneumoniae]
 gb|KLZ08349.1| short-chain dehydrogenase/reductase SDR [Klebsiella pneumoniae]
 gb|KLZ14441.1| short-chain dehydrogenase/reductase SDR [Klebsiella pneumoniae]
 gb|KLZ15520.1| short-chain dehydrogenase/reductase SDR [Klebsiella pneumoniae]
 gb|KLZ27846.1| short-chain dehydrogenase/reductase SDR [Klebsiella pneumoniae]
 gb|KLZ29023.1| short-chain dehydrogenase/reductase SDR [Klebsiella pneumoniae]
 gb|KLZ36791.1| short-chain dehydrogenase/reductase SDR [Klebsiella pneumoniae]
 gb|KLZ37606.1| short-chain dehydrogenase/reductase SDR [Klebsiella pneumoniae]
 gb|KLZ47612.1| short-chain dehydrogenase/reductase SDR [Klebsiella pneumoniae]
 gb|KLZ54226.1| short-chain dehydrogenase/reductase SDR [Klebsiella pneumoniae]
 gb|KLZ57144.1| short-chain dehydrogenase/reductase SDR [Klebsiella pneumoniae]
 gb|KLZ58333.1| short-chain dehydrogenase/reductase SDR [Klebsiella pneumoniae]
 gb|KLZ74082.1| short-chain dehydrogenase/reductase SDR [Klebsiella pneumoniae]
 gb|KLZ80714.1| short-chain dehydrogenase/reductase SDR [Klebsiella pneumoniae]
 gb|KLZ85845.1| short-chain dehydrogenase/reductase SDR [Klebsiella pneumoniae]
 gb|KLZ90904.1| short-chain dehydrogenase/reductase SDR [Klebsiella pneumoniae]
 gb|KLZ99508.1| short-chain dehydrogenase/reductase SDR [Klebsiella pneumoniae]
 gb|KMA01387.1| short-chain dehydrogenase/reductase SDR [Klebsiella pneumoniae]
 gb|KMA02679.1| short-chain dehydrogenase/reductase SDR [Klebsiella pneumoniae]
 gb|KMA15508.1| short-chain dehydrogenase/reductase SDR [Klebsiella pneumoniae]
 gb|KMA16502.1| short-chain dehydrogenase/reductase SDR [Klebsiella pneumoniae]
 gb|KMA27950.1| short-chain dehydrogenase/reductase SDR [Klebsiella pneumoniae]
 gb|KMA33122.1| short-chain dehydrogenase/reductase SDR [Klebsiella pneumoniae]
 gb|KMA44484.1| short-chain dehydrogenase/reductase SDR [Klebsiella pneumoniae]
 gb|KMA45291.1| short-chain dehydrogenase/reductase SDR [Klebsiella pneumoniae]
 gb|KMA45823.1| short-chain dehydrogenase/reductase SDR [Klebsiella pneumoniae]
 gb|KMA59973.1| short-chain dehydrogenase/reductase SDR [Klebsiella pneumoniae]
 gb|KMA61512.1| short-chain dehydrogenase/reductase SDR [Klebsiella pneumoniae]
 gb|KMA72072.1| short-chain dehydrogenase/reductase SDR [Klebsiella pneumoniae]
 gb|KMA75149.1| short-chain dehydrogenase/reductase SDR [Klebsiella pneumoniae]
 gb|KMA77431.1| short-chain dehydrogenase/reductase SDR [Klebsiella pneumoniae]
 gb|KMA81930.1| short-chain dehydrogenase/reductase SDR [Klebsiella pneumoniae]
 gb|KMA83516.1| short-chain dehydrogenase/reductase SDR [Klebsiella pneumoniae]
 gb|KMA93457.1| short-chain dehydrogenase/reductase SDR [Klebsiella pneumoniae]
 gb|KMB03015.1| short-chain dehydrogenase/reductase SDR [Klebsiella pneumoniae]
 gb|KMB07674.1| short-chain dehydrogenase/reductase SDR [Klebsiella pneumoniae]
 gb|KMB10165.1| short-chain dehydrogenase/reductase SDR [Klebsiella pneumoniae]
 gb|KMB16695.1| short-chain dehydrogenase/reductase SDR [Klebsiella pneumoniae]
 gb|KMB25155.1| short-chain dehydrogenase/reductase SDR [Klebsiella pneumoniae]
 gb|KMB28899.1| short-chain dehydrogenase/reductase SDR [Klebsiella pneumoniae]
 gb|KMB31013.1| short-chain dehydrogenase/reductase SDR [Klebsiella pneumoniae]
 gb|KMB38560.1| short-chain dehydrogenase/reductase SDR [Klebsiella pneumoniae]
 gb|KMB40015.1| short-chain dehydrogenase/reductase SDR [Klebsiella pneumoniae]
 gb|KMB51060.1| short-chain dehydrogenase/reductase SDR [Klebsiella pneumoniae]
 gb|KMB53670.1| short-chain dehydrogenase/reductase SDR [Klebsiella pneumoniae]
 gb|KMB54427.1| short-chain dehydrogenase/reductase SDR [Klebsiella pneumoniae]
 gb|KMB60082.1| short-chain dehydrogenase/reductase SDR [Klebsiella pneumoniae]
 gb|KMB71498.1| short-chain dehydrogenase/reductase SDR [Klebsiella pneumoniae]
 gb|KMB74616.1| short-chain dehydrogenase/reductase SDR [Klebsiella pneumoniae]
 gb|KMB76242.1| short-chain dehydrogenase/reductase SDR [Klebsiella pneumoniae]
 gb|KMB90097.1| short-chain dehydrogenase/reductase SDR [Klebsiella pneumoniae]
 gb|KMB91780.1| short-chain dehydrogenase/reductase SDR [Klebsiella pneumoniae]
 gb|KMB94808.1| short-chain dehydrogenase/reductase SDR [Klebsiella pneumoniae]
 gb|KMD02319.1| short-chain dehydrogenase/reductase SDR [Klebsiella pneumoniae]
 gb|KMD06774.1| short-chain dehydrogenase/reductase SDR [Klebsiella pneumoniae]
 gb|KMD12135.1| short-chain dehydrogenase/reductase SDR [Klebsiella pneumoniae]
 gb|KMD14167.1| short-chain dehydrogenase/reductase SDR [Klebsiella pneumoniae]
 gb|KMD18203.1| short-chain dehydrogenase/reductase SDR [Klebsiella pneumoniae]
 gb|KMD23495.1| short-chain dehydrogenase/reductase SDR [Klebsiella pneumoniae]
 gb|KMD33139.1| short-chain dehydrogenase/reductase SDR [Klebsiella pneumoniae]
 gb|KMD35744.1| short-chain dehydrogenase/reductase SDR [Klebsiella pneumoniae]
 gb|KMD40573.1| short-chain dehydrogenase/reductase SDR [Klebsiella pneumoniae]
 gb|KMD49453.1| short-chain dehydrogenase/reductase SDR [Klebsiella pneumoniae]
 gb|KMD52342.1| short-chain dehydrogenase/reductase SDR [Klebsiella pneumoniae]
 gb|KMD65035.1| short-chain dehydrogenase/reductase SDR [Klebsiella pneumoniae]
 gb|KMD67440.1| short-chain dehydrogenase/reductase SDR [Klebsiella pneumoniae]
 gb|KMD74763.1| short-chain dehydrogenase/reductase SDR [Klebsiella pneumoniae]
 gb|KMD79857.1| short-chain dehydrogenase/reductase SDR [Klebsiella pneumoniae]
 gb|KMD83971.1| short-chain dehydrogenase/reductase SDR [Klebsiella pneumoniae]
 gb|KMD87046.1| short-chain dehydrogenase/reductase SDR [Klebsiella pneumoniae]
 gb|KMD93600.1| short-chain dehydrogenase/reductase SDR [Klebsiella pneumoniae]
 gb|KME00343.1| short-chain dehydrogenase/reductase SDR [Klebsiella pneumoniae]
 gb|KME05616.1| short-chain dehydrogenase/reductase SDR [Klebsiella pneumoniae]
 gb|KME10076.1| short-chain dehydrogenase/reductase SDR [Klebsiella pneumoniae]
 gb|KME16271.1| short-chain dehydrogenase/reductase SDR [Klebsiella pneumoniae]
 gb|KME17651.1| short-chain dehydrogenase/reductase SDR [Klebsiella pneumoniae]
 gb|KME27625.1| short-chain dehydrogenase/reductase SDR [Klebsiella pneumoniae]
 gb|KME34328.1| short-chain dehydrogenase/reductase SDR [Klebsiella pneumoniae]
 gb|KME38323.1| short-chain dehydrogenase/reductase SDR [Klebsiella pneumoniae]
 gb|KME41354.1| short-chain dehydrogenase/reductase SDR [Klebsiella pneumoniae]
 gb|KME43889.1| short-chain dehydrogenase/reductase SDR [Klebsiella pneumoniae]
 gb|KME50523.1| short-chain dehydrogenase/reductase SDR [Klebsiella pneumoniae]
 gb|KME60884.1| short-chain dehydrogenase/reductase SDR [Klebsiella pneumoniae]
 gb|KME62814.1| short-chain dehydrogenase/reductase SDR [Klebsiella pneumoniae]
 gb|KME69515.1| short-chain dehydrogenase/reductase SDR [Klebsiella pneumoniae]
 gb|KME81190.1| short-chain dehydrogenase/reductase SDR [Klebsiella pneumoniae]
 gb|KME83426.1| short-chain dehydrogenase/reductase SDR [Klebsiella pneumoniae]
 gb|KME90900.1| short-chain dehydrogenase/reductase SDR [Klebsiella pneumoniae]
 gb|KME97338.1| short-chain dehydrogenase/reductase SDR [Klebsiella pneumoniae]
 gb|KMF10059.1| short-chain dehydrogenase/reductase SDR [Klebsiella pneumoniae]
 gb|KMF10620.1| short-chain dehydrogenase/reductase SDR [Klebsiella pneumoniae]
 gb|KMF15979.1| short-chain dehydrogenase/reductase SDR [Klebsiella pneumoniae]
 gb|KMF21540.1| short-chain dehydrogenase/reductase SDR [Klebsiella pneumoniae]
 gb|KMF29161.1| short-chain dehydrogenase/reductase SDR [Klebsiella pneumoniae]
 gb|KMF36920.1| short-chain dehydrogenase/reductase SDR [Klebsiella pneumoniae]
 gb|KMF37377.1| short-chain dehydrogenase/reductase SDR [Klebsiella pneumoniae]
 gb|KMF46103.1| short-chain dehydrogenase/reductase SDR [Klebsiella pneumoniae]
 gb|KMF50465.1| short-chain dehydrogenase/reductase SDR [Klebsiella pneumoniae]
 gb|KMF61333.1| short-chain dehydrogenase/reductase SDR [Klebsiella pneumoniae]
 gb|KMF64920.1| short-chain dehydrogenase/reductase SDR [Klebsiella pneumoniae]
 gb|KMF65510.1| short-chain dehydrogenase/reductase SDR [Klebsiella pneumoniae]
 gb|KMF74618.1| short-chain dehydrogenase/reductase SDR [Klebsiella pneumoniae]
 gb|KMF76008.1| short-chain dehydrogenase/reductase SDR [Klebsiella pneumoniae]
 gb|KMF82736.1| short-chain dehydrogenase/reductase SDR [Klebsiella pneumoniae]
 gb|KMF86892.1| short-chain dehydrogenase/reductase SDR [Klebsiella pneumoniae]
 gb|KMF96608.1| short-chain dehydrogenase/reductase SDR [Klebsiella pneumoniae]
 gb|KMF97988.1| short-chain dehydrogenase/reductase SDR [Klebsiella pneumoniae]
 gb|KMG07146.1| short-chain dehydrogenase/reductase SDR [Klebsiella pneumoniae]
 gb|KMG08529.1| short-chain dehydrogenase/reductase SDR [Klebsiella pneumoniae]
 gb|KMG17798.1| short-chain dehydrogenase/reductase SDR [Klebsiella pneumoniae]
 gb|KMG19502.1| short-chain dehydrogenase/reductase SDR [Klebsiella pneumoniae]
 gb|KMG24640.1| short-chain dehydrogenase/reductase SDR [Klebsiella pneumoniae]
 gb|KMG34279.1| short-chain dehydrogenase/reductase SDR [Klebsiella pneumoniae]
 gb|KMG35041.1| short-chain dehydrogenase/reductase SDR [Klebsiella pneumoniae]
 gb|KMG45616.1| short-chain dehydrogenase/reductase SDR [Klebsiella pneumoniae]
 gb|KMG47696.1| short-chain dehydrogenase/reductase SDR [Klebsiella pneumoniae]
 gb|KMG56222.1| short-chain dehydrogenase/reductase SDR [Klebsiella pneumoniae]
 gb|KMG60283.1| short-chain dehydrogenase/reductase SDR [Klebsiella pneumoniae]
 gb|KMG64974.1| short-chain dehydrogenase/reductase SDR [Klebsiella pneumoniae]
 gb|KMG74134.1| short-chain dehydrogenase/reductase SDR [Klebsiella pneumoniae]
 gb|KMG75253.1| short-chain dehydrogenase/reductase SDR [Klebsiella pneumoniae]
 gb|KMG83413.1| short-chain dehydrogenase/reductase SDR [Klebsiella pneumoniae]
 gb|KMG87994.1| short-chain dehydrogenase/reductase SDR [Klebsiella pneumoniae]
 gb|KMG91658.1| short-chain dehydrogenase/reductase SDR [Klebsiella pneumoniae]
 gb|KMH03910.1| short-chain dehydrogenase/reductase SDR [Klebsiella pneumoniae]
 gb|KMH06906.1| short-chain dehydrogenase/reductase SDR [Klebsiella pneumoniae]
 gb|KMH22692.1| short-chain dehydrogenase/reductase SDR [Klebsiella pneumoniae]
 gb|KMH31677.1| short-chain dehydrogenase/reductase SDR [Klebsiella pneumoniae]
 gb|KMH31859.1| short-chain dehydrogenase/reductase SDR [Klebsiella pneumoniae]
 gb|KMH38643.1| short-chain dehydrogenase/reductase SDR [Klebsiella pneumoniae]
 gb|KMH44507.1| short-chain dehydrogenase/reductase SDR [Klebsiella pneumoniae]
 gb|KMH60932.1| short-chain dehydrogenase/reductase SDR [Klebsiella pneumoniae]
 gb|KMH72447.1| short-chain dehydrogenase/reductase SDR [Klebsiella pneumoniae]
 gb|KMH72995.1| short-chain dehydrogenase/reductase SDR [Klebsiella pneumoniae]
 gb|KMH73960.1| short-chain dehydrogenase/reductase SDR [Klebsiella pneumoniae]
 gb|KMH91081.1| short-chain dehydrogenase/reductase SDR [Klebsiella pneumoniae]
 gb|KMI00846.1| short-chain dehydrogenase/reductase SDR [Klebsiella pneumoniae]
 gb|KMI03583.1| short-chain dehydrogenase/reductase SDR [Klebsiella pneumoniae]
 gb|KMI10233.1| short-chain dehydrogenase/reductase SDR [Klebsiella pneumoniae]
 gb|KMI17211.1| short-chain dehydrogenase/reductase SDR [Klebsiella pneumoniae]
 gb|KMI22488.1| short-chain dehydrogenase/reductase SDR [Klebsiella pneumoniae]
 gb|KMI32464.1| short-chain dehydrogenase/reductase SDR [Klebsiella pneumoniae]
 gb|KMI46011.1| short-chain dehydrogenase/reductase SDR [Klebsiella pneumoniae]
 gb|KMI47550.1| short-chain dehydrogenase/reductase SDR [Klebsiella pneumoniae]
 gb|KMI56227.1| short-chain dehydrogenase/reductase SDR [Klebsiella pneumoniae]
 gb|KMI61125.1| short-chain dehydrogenase/reductase SDR [Klebsiella pneumoniae]
 gb|KMI68646.1| short-chain dehydrogenase/reductase SDR [Klebsiella pneumoniae]
 gb|KMI69468.1| short-chain dehydrogenase/reductase SDR [Klebsiella pneumoniae]
 gb|KMI77680.1| short-chain dehydrogenase/reductase SDR [Klebsiella pneumoniae]
 gb|KMI83841.1| short-chain dehydrogenase/reductase SDR [Klebsiella pneumoniae]
 gb|KMI87177.1| short-chain dehydrogenase/reductase SDR [Klebsiella pneumoniae]
 gb|KMI96139.1| short-chain dehydrogenase/reductase SDR [Klebsiella pneumoniae]
 gb|KMJ01288.1| short-chain dehydrogenase/reductase SDR [Klebsiella pneumoniae]
 gb|KMK49742.1| short-chain dehydrogenase [Klebsiella pneumoniae]
 gb|KMW89052.1| short-chain dehydrogenase/reductase SDR [Klebsiella pneumoniae]
 gb|KMW91413.1| short-chain dehydrogenase/reductase SDR [Klebsiella pneumoniae]
 gb|KMW97050.1| short-chain dehydrogenase/reductase SDR [Klebsiella pneumoniae]
 gb|KMW99621.1| short-chain dehydrogenase/reductase SDR [Klebsiella pneumoniae]
 gb|KMX08244.1| short-chain dehydrogenase/reductase SDR [Klebsiella pneumoniae]
 gb|KMX10727.1| short-chain dehydrogenase/reductase SDR [Klebsiella pneumoniae]
 gb|KMX20949.1| short-chain dehydrogenase/reductase SDR [Klebsiella pneumoniae]
 gb|KMX24920.1| short-chain dehydrogenase/reductase SDR [Klebsiella pneumoniae]
 gb|KMX26119.1| short-chain dehydrogenase/reductase SDR [Klebsiella pneumoniae]
 gb|KMX29987.1| short-chain dehydrogenase/reductase SDR [Klebsiella pneumoniae]
 gb|KMX41355.1| short-chain dehydrogenase/reductase SDR [Klebsiella pneumoniae]
 gb|KMX46206.1| short-chain dehydrogenase/reductase SDR [Klebsiella pneumoniae]
 gb|KMX51921.1| short-chain dehydrogenase/reductase SDR [Klebsiella pneumoniae]
 gb|KMX54772.1| short-chain dehydrogenase/reductase SDR [Klebsiella pneumoniae]
 gb|KMX62389.1| short-chain dehydrogenase/reductase SDR [Klebsiella pneumoniae]
 gb|KMX65239.1| short-chain dehydrogenase/reductase SDR [Klebsiella pneumoniae]
 gb|KMX67152.1| short-chain dehydrogenase/reductase SDR [Klebsiella pneumoniae]
 gb|KMX72950.1| short-chain dehydrogenase/reductase SDR [Klebsiella pneumoniae]
 gb|KMX82829.1| short-chain dehydrogenase/reductase SDR [Klebsiella pneumoniae]
 gb|KMX90485.1| short-chain dehydrogenase/reductase SDR [Klebsiella pneumoniae]
 gb|KMX94427.1| short-chain dehydrogenase/reductase SDR [Klebsiella pneumoniae]
 gb|AKR90283.1| short-chain dehydrogenase [Klebsiella pneumoniae 500_1420]
 gb|AKR84799.1| short-chain dehydrogenase [Klebsiella pneumoniae DMC1097]
 gb|AKR95765.1| short-chain dehydrogenase [Klebsiella pneumoniae UHKPC07]
 gb|AKS01235.1| short-chain dehydrogenase [Klebsiella pneumoniae UHKPC33]
 gb|KOP09090.1| short-chain dehydrogenase [Klebsiella pneumoniae]
 gb|KOP10431.1| short-chain dehydrogenase [Klebsiella pneumoniae]
 gb|KOP17086.1| short-chain dehydrogenase [Klebsiella pneumoniae]

Length=249

 Score = 37.4 bits (85),  Expect = 2.0, Method: Compositional matrix adjust.
 Identities = 23/73 (32%), Positives = 38/73 (52%), Gaps = 10/73 (14%)

Query  5    LLSSRTFSPTLRSRRSSPL---------EGGDVFAGAHTSAASKHAVCQAEGLSAAPGPQ  55
            LL S+   PT+R+++S  +          GG +F G H SAA    +  A  ++   GP 
Sbjct  117  LLMSQAVIPTMRAQKSGSIVCISSVSAQRGGGIFGGPHYSAAKAGVLGLARAMARELGPD  176

Query  56   SVRVSDVE-GVLQ  67
            +VRV+ +  G++Q
Sbjct  177  NVRVNCITPGLIQ  189


>ref|WP_044243604.1| short-chain dehydrogenase, partial [Klebsiella pneumoniae]
Length=231

 Score = 37.4 bits (85),  Expect = 2.0, Method: Compositional matrix adjust.
 Identities = 23/73 (32%), Positives = 38/73 (52%), Gaps = 10/73 (14%)

Query  5    LLSSRTFSPTLRSRRSSPL---------EGGDVFAGAHTSAASKHAVCQAEGLSAAPGPQ  55
            LL S+   PT+R+++S  +          GG +F G H SAA    +  A  ++   GP 
Sbjct  99   LLMSQAVIPTMRAQKSGSIVCISSVSAQRGGGIFGGPHYSAAKAGVLGLARAMARELGPD  158

Query  56   SVRVSDVE-GVLQ  67
            +VRV+ +  G++Q
Sbjct  159  NVRVNCITPGLIQ  171


>ref|WP_049113343.1| short-chain dehydrogenase [Klebsiella oxytoca]
Length=249

 Score = 37.4 bits (85),  Expect = 2.0, Method: Compositional matrix adjust.
 Identities = 23/73 (32%), Positives = 38/73 (52%), Gaps = 10/73 (14%)

Query  5    LLSSRTFSPTLRSRRSSPL---------EGGDVFAGAHTSAASKHAVCQAEGLSAAPGPQ  55
            LL S+   PT+R+++S  +          GG +F G H SAA    +  A  ++   GP 
Sbjct  117  LLMSQAVIPTMRAQKSGSIVCISSVSAQRGGGIFGGPHYSAAKAGVLGLARAMARELGPD  176

Query  56   SVRVSDVE-GVLQ  67
            +VRV+ +  G++Q
Sbjct  177  NVRVNCITPGLIQ  189


>ref|WP_048974553.1| short-chain dehydrogenase [Klebsiella pneumoniae]
Length=249

 Score = 37.4 bits (85),  Expect = 2.0, Method: Compositional matrix adjust.
 Identities = 23/73 (32%), Positives = 38/73 (52%), Gaps = 10/73 (14%)

Query  5    LLSSRTFSPTLRSRRSSPL---------EGGDVFAGAHTSAASKHAVCQAEGLSAAPGPQ  55
            LL S+   PT+R+++S  +          GG +F G H SAA    +  A  ++   GP 
Sbjct  117  LLMSQAVIPTMRAQKSGSIVCISSVSAQRGGGIFGGPHYSAAKAGVLGLARAMARELGPD  176

Query  56   SVRVSDVE-GVLQ  67
            +VRV+ +  G++Q
Sbjct  177  NVRVNCITPGLIQ  189


>ref|WP_038431907.1| short-chain dehydrogenase [Klebsiella pneumoniae]
 gb|AIK80323.1| short chain dehydrogenase family protein [Klebsiella pneumoniae 
subsp. pneumoniae]
 gb|KGB18892.1| short-chain dehydrogenase [Klebsiella pneumoniae]
Length=249

 Score = 37.4 bits (85),  Expect = 2.0, Method: Compositional matrix adjust.
 Identities = 23/73 (32%), Positives = 38/73 (52%), Gaps = 10/73 (14%)

Query  5    LLSSRTFSPTLRSRRSSPL---------EGGDVFAGAHTSAASKHAVCQAEGLSAAPGPQ  55
            LL S+   PT+R+++S  +          GG +F G H SAA    +  A  ++   GP 
Sbjct  117  LLMSQAVIPTMRAQKSGSIVCISSVSAQRGGGIFGGPHYSAAKAGVLGLARAMARELGPD  176

Query  56   SVRVSDVE-GVLQ  67
            +VRV+ +  G++Q
Sbjct  177  NVRVNCITPGLIQ  189


>ref|WP_032411091.1| short-chain dehydrogenase [Klebsiella pneumoniae]
 gb|ETX42211.1| short-chain dehydrogenase/reductase SDR [Klebsiella pneumoniae 
BIDMC 31]
Length=249

 Score = 37.4 bits (85),  Expect = 2.0, Method: Compositional matrix adjust.
 Identities = 23/73 (32%), Positives = 38/73 (52%), Gaps = 10/73 (14%)

Query  5    LLSSRTFSPTLRSRRSSPL---------EGGDVFAGAHTSAASKHAVCQAEGLSAAPGPQ  55
            LL S+   PT+R+++S  +          GG +F G H SAA    +  A  ++   GP 
Sbjct  117  LLMSQAVIPTMRAQKSGSIVCISSVSAQRGGGIFGGPHYSAAKAGVLGLARAMARELGPD  176

Query  56   SVRVSDVE-GVLQ  67
            +VRV+ +  G++Q
Sbjct  177  NVRVNCITPGLIQ  189


>ref|WP_016160530.1| MULTISPECIES: hypothetical protein [Enterobacteriaceae]
 gb|EOQ54843.1| hypothetical protein A1WC_01411 [Klebsiella sp. KTE92]
 gb|ESL89258.1| short-chain dehydrogenase/reductase SDR [Klebsiella pneumoniae 
UCICRE 10]
 8 more sequence titles

gb|EWD82848.1| short-chain dehydrogenase/reductase SDR [Klebsiella pneumoniae 
UCI 18]
 gb|KDL61530.1| hypothetical protein AD94_00944 [Klebsiella pneumoniae MGH 68]
 gb|KHE24053.1| short-chain dehydrogenase [Klebsiella pneumoniae]
 gb|KKY79220.1| short-chain dehydrogenase [Klebsiella pneumoniae]
 gb|KMH63017.1| short-chain dehydrogenase/reductase SDR [Klebsiella pneumoniae]
 emb|CTQ01341.1| Uncharacterized short-chain type dehydrogenase/reductase y4mP 
[Klebsiella variicola]
 emb|CTQ16845.1| Uncharacterized short-chain type dehydrogenase/reductase y4mP 
[Klebsiella variicola]
 emb|CTQ05483.1| Uncharacterized short-chain type dehydrogenase/reductase y4mP 
[Klebsiella variicola]

Length=249

 Score = 37.4 bits (85),  Expect = 2.0, Method: Compositional matrix adjust.
 Identities = 23/73 (32%), Positives = 38/73 (52%), Gaps = 10/73 (14%)

Query  5    LLSSRTFSPTLRSRRSSPL---------EGGDVFAGAHTSAASKHAVCQAEGLSAAPGPQ  55
            LL S+   PT+R+++S  +          GG +F G H SAA    +  A  ++   GP 
Sbjct  117  LLMSQAVIPTMRAQKSGSIVCISSVSAQRGGGIFGGPHYSAAKAGVLGLARAMARELGPD  176

Query  56   SVRVSDVE-GVLQ  67
            +VRV+ +  G++Q
Sbjct  177  NVRVNCITPGLIQ  189


>ref|WP_022065514.1| MULTISPECIES: short-chain dehydrogenase/reductase SDR [Klebsiella]
 emb|CDA01307.1| short-chain dehydrogenase/reductase SDR [Klebsiella variicola 
CAG:634]
 gb|ESN45302.1| short-chain dehydrogenase/reductase SDR [Klebsiella pneumoniae 
MGH 20]
 gb|KFJ96530.1| short-chain dehydrogenase [Klebsiella variicola]
 gb|KMI15571.1| short-chain dehydrogenase/reductase SDR [Klebsiella pneumoniae]
Length=249

 Score = 37.4 bits (85),  Expect = 2.0, Method: Compositional matrix adjust.
 Identities = 23/73 (32%), Positives = 38/73 (52%), Gaps = 10/73 (14%)

Query  5    LLSSRTFSPTLRSRRSSPL---------EGGDVFAGAHTSAASKHAVCQAEGLSAAPGPQ  55
            LL S+   PT+R+++S  +          GG +F G H SAA    +  A  ++   GP 
Sbjct  117  LLMSQAVIPTMRAQKSGSIVCISSVSAQRGGGIFGGPHYSAAKAGVLGLARAMARELGPD  176

Query  56   SVRVSDVE-GVLQ  67
            +VRV+ +  G++Q
Sbjct  177  NVRVNCITPGLIQ  189


>gb|ESL29483.1| short-chain dehydrogenase/reductase SDR [Klebsiella pneumoniae 
BIDMC 40]
Length=204

 Score = 37.4 bits (85),  Expect = 2.0, Method: Compositional matrix adjust.
 Identities = 23/73 (32%), Positives = 38/73 (52%), Gaps = 10/73 (14%)

Query  5    LLSSRTFSPTLRSRRSSPL---------EGGDVFAGAHTSAASKHAVCQAEGLSAAPGPQ  55
            LL S+   PT+R+++S  +          GG +F G H SAA    +  A  ++   GP 
Sbjct  72   LLMSQAVIPTMRAQKSGSIVCISSVSAQRGGGIFGGPHYSAAKAGVLGLARAMARELGPD  131

Query  56   SVRVSDVE-GVLQ  67
            +VRV+ +  G++Q
Sbjct  132  NVRVNCITPGLIQ  144


>emb|CTQ17466.1| Uncharacterized short-chain type dehydrogenase/reductase y4mP 
[Klebsiella variicola]
Length=249

 Score = 37.4 bits (85),  Expect = 2.0, Method: Compositional matrix adjust.
 Identities = 23/73 (32%), Positives = 38/73 (52%), Gaps = 10/73 (14%)

Query  5    LLSSRTFSPTLRSRRSSPL---------EGGDVFAGAHTSAASKHAVCQAEGLSAAPGPQ  55
            LL S+   PT+R+++S  +          GG +F G H SAA    +  A  ++   GP 
Sbjct  117  LLMSQAVIPTMRAQKSGSIVCISSVSAQRGGGIFGGPHYSAAKAGVLGLARAMARELGPD  176

Query  56   SVRVSDVE-GVLQ  67
            +VRV+ +  G++Q
Sbjct  177  NVRVNCITPGLIQ  189


>ref|WP_040973985.1| MULTISPECIES: short-chain dehydrogenase [Klebsiella]
 gb|AJE91931.1| short-chain dehydrogenase [Klebsiella variicola]
Length=249

 Score = 37.4 bits (85),  Expect = 2.0, Method: Compositional matrix adjust.
 Identities = 23/73 (32%), Positives = 38/73 (52%), Gaps = 10/73 (14%)

Query  5    LLSSRTFSPTLRSRRSSPL---------EGGDVFAGAHTSAASKHAVCQAEGLSAAPGPQ  55
            LL S+   PT+R+++S  +          GG +F G H SAA    +  A  ++   GP 
Sbjct  117  LLMSQAVIPTMRAQKSGSIVCISSVSAQRGGGIFGGPHYSAAKAGVLGLARAMARELGPD  176

Query  56   SVRVSDVE-GVLQ  67
            +VRV+ +  G++Q
Sbjct  177  NVRVNCITPGLIQ  189


>ref|WP_032735010.1| MULTISPECIES: short-chain dehydrogenase [Klebsiella]
 gb|KDH30545.1| hypothetical protein AE36_00647 [Klebsiella pneumoniae BIDMC 
61]
 gb|KDM09795.1| hypothetical protein AE06_00633 [Klebsiella pneumoniae MGH 80]
 gb|KDM59011.1| hypothetical protein AE02_00655 [Klebsiella pneumoniae MGH 76]
 gb|KKY91252.1| short-chain dehydrogenase [Klebsiella pneumoniae]
 gb|KNB81177.1| short-chain dehydrogenase [Klebsiella variicola]
 gb|KNB86288.1| short-chain dehydrogenase [Klebsiella variicola]
Length=249

 Score = 37.4 bits (85),  Expect = 2.0, Method: Compositional matrix adjust.
 Identities = 23/73 (32%), Positives = 38/73 (52%), Gaps = 10/73 (14%)

Query  5    LLSSRTFSPTLRSRRSSPL---------EGGDVFAGAHTSAASKHAVCQAEGLSAAPGPQ  55
            LL S+   PT+R+++S  +          GG +F G H SAA    +  A  ++   GP 
Sbjct  117  LLMSQAVIPTMRAQKSGSIVCISSVSAQRGGGIFGGPHYSAAKAGVLGLARAMARELGPD  176

Query  56   SVRVSDVE-GVLQ  67
            +VRV+ +  G++Q
Sbjct  177  NVRVNCITPGLIQ  189


>ref|WP_048273490.1| short-chain dehydrogenase [Klebsiella pneumoniae]
 gb|KMA15029.1| short-chain dehydrogenase/reductase SDR [Klebsiella pneumoniae]
Length=249

 Score = 37.4 bits (85),  Expect = 2.1, Method: Compositional matrix adjust.
 Identities = 23/73 (32%), Positives = 38/73 (52%), Gaps = 10/73 (14%)

Query  5    LLSSRTFSPTLRSRRSSPL---------EGGDVFAGAHTSAASKHAVCQAEGLSAAPGPQ  55
            LL S+   PT+R+++S  +          GG +F G H SAA    +  A  ++   GP 
Sbjct  117  LLMSQAVIPTMRAQKSGSIVCISSVSAQRGGGIFGGPHYSAAKAGVLGLARAMARELGPD  176

Query  56   SVRVSDVE-GVLQ  67
            +VRV+ +  G++Q
Sbjct  177  NVRVNCITPGLIQ  189


>ref|WP_023300845.1| short-chain dehydrogenase/reductase SDR [Klebsiella pneumoniae]
 gb|ESM07913.1| short-chain dehydrogenase/reductase SDR [Klebsiella pneumoniae 
UCICRE 8]
Length=249

 Score = 37.4 bits (85),  Expect = 2.1, Method: Compositional matrix adjust.
 Identities = 23/73 (32%), Positives = 38/73 (52%), Gaps = 10/73 (14%)

Query  5    LLSSRTFSPTLRSRRSSPL---------EGGDVFAGAHTSAASKHAVCQAEGLSAAPGPQ  55
            LL S+   PT+R+++S  +          GG +F G H SAA    +  A  ++   GP 
Sbjct  117  LLMSQAVIPTMRAQKSGSIVCISSVSAQRGGGIFGGPHYSAAKAGVLGLARAMARELGPD  176

Query  56   SVRVSDVE-GVLQ  67
            +VRV+ +  G++Q
Sbjct  177  NVRVNCITPGLIQ  189


>gb|AIE02442.1| short-chain dehydrogenase [Klebsiella pneumoniae subsp. pneumoniae 
KP5-1]
Length=249

 Score = 37.4 bits (85),  Expect = 2.2, Method: Compositional matrix adjust.
 Identities = 23/73 (32%), Positives = 38/73 (52%), Gaps = 10/73 (14%)

Query  5    LLSSRTFSPTLRSRRSSPL---------EGGDVFAGAHTSAASKHAVCQAEGLSAAPGPQ  55
            LL S+   PT+R+++S  +          GG +F G H SAA    +  A  ++   GP 
Sbjct  117  LLMSQAVIPTMRAQKSGSIVCISSVSAQRGGGIFGGPHYSAAKAGVLGLARAMARELGPD  176

Query  56   SVRVS-DVEGVLQ  67
            +VRV+  + G++Q
Sbjct  177  NVRVNCIIPGLIQ  189


>ref|WP_053194915.1| short-chain dehydrogenase [Herbaspirillum hiltneri]
 gb|AKZ61428.1| short-chain dehydrogenase [Herbaspirillum hiltneri N3]
Length=249

 Score = 37.0 bits (84),  Expect = 3.1, Method: Compositional matrix adjust.
 Identities = 23/73 (32%), Positives = 36/73 (49%), Gaps = 10/73 (14%)

Query  5    LLSSRTFSPTLRSRRSSPL---------EGGDVFAGAHTSAASKHAVCQAEGLSAAPGPQ  55
            L  S+   P +RSRRS  +          GG +F G H SAA    +  A  ++   GP 
Sbjct  117  LYMSQALIPHMRSRRSGSIVCLSSVSAQRGGGIFGGPHYSAAKAGVLGLARAMARELGPD  176

Query  56   SVRVSDVE-GVLQ  67
            ++RV+ +  G++Q
Sbjct  177  NIRVNSLTPGLIQ  189


>ref|WP_007883302.1| short-chain dehydrogenase [Herbaspirillum sp. CF444]
 gb|EJL83352.1| dehydrogenase of unknown specificity, short-chain alcohol dehydrogenase 
[Herbaspirillum sp. CF444]
Length=249

 Score = 37.0 bits (84),  Expect = 3.1, Method: Compositional matrix adjust.
 Identities = 23/73 (32%), Positives = 36/73 (49%), Gaps = 10/73 (14%)

Query  5    LLSSRTFSPTLRSRRSSPL---------EGGDVFAGAHTSAASKHAVCQAEGLSAAPGPQ  55
            L  S+   P +RSRRS  +          GG +F G H SAA    +  A  ++   GP 
Sbjct  117  LYMSQALIPHMRSRRSGSIVCLSSVSAQRGGGIFGGPHYSAAKAGVLGLARAMARELGPD  176

Query  56   SVRVSDVE-GVLQ  67
            ++RV+ +  G++Q
Sbjct  177  NIRVNSLTPGLIQ  189


>ref|WP_050463195.1| short-chain dehydrogenase [Herbaspirillum autotrophicum]
Length=249

 Score = 36.6 bits (83),  Expect = 3.2, Method: Compositional matrix adjust.
 Identities = 23/73 (32%), Positives = 36/73 (49%), Gaps = 10/73 (14%)

Query  5    LLSSRTFSPTLRSRRSSPL---------EGGDVFAGAHTSAASKHAVCQAEGLSAAPGPQ  55
            L  S+   P +RSRRS  +          GG +F G H SAA    +  A  ++   GP 
Sbjct  117  LYMSQALIPHMRSRRSGSIVCLSSVSAQRGGGIFGGPHYSAAKAGVLGLARAMARELGPD  176

Query  56   SVRVSDVE-GVLQ  67
            ++RV+ +  G++Q
Sbjct  177  NIRVNSLTPGLIQ  189


>ref|WP_008495475.1| short-chain dehydrogenase [Acidocella sp. MX-AZ02]
 gb|EKM98345.1| putative oxidoreductase [Acidocella sp. MX-AZ02]
Length=253

 Score = 36.6 bits (83),  Expect = 3.4, Method: Compositional matrix adjust.
 Identities = 22/77 (29%), Positives = 37/77 (48%), Gaps = 10/77 (13%)

Query  1    MVSALLSSRTFSPTLRSRRSSPL---------EGGDVFAGAHTSAASKHAVCQAEGLSAA  51
            +   L  S+ F P +R+R+   +          GG +F G H SAA    +  A+ ++  
Sbjct  117  LTGVLYLSQAFIPHMRARKQGSIACMSSVSAQRGGGIFGGPHYSAAKAGVLGLAKAMARE  176

Query  52   PGPQSVRVSDVE-GVLQ  67
             GP  +RV+ V  G++Q
Sbjct  177  LGPDGIRVNSVTPGLIQ  193


>ref|WP_026439851.1| short-chain dehydrogenase [Acidocella facilis]
Length=253

 Score = 36.6 bits (83),  Expect = 3.4, Method: Compositional matrix adjust.
 Identities = 22/77 (29%), Positives = 37/77 (48%), Gaps = 10/77 (13%)

Query  1    MVSALLSSRTFSPTLRSRRSSPL---------EGGDVFAGAHTSAASKHAVCQAEGLSAA  51
            +   L  S+ F P +R+R+   +          GG +F G H SAA    +  A+ ++  
Sbjct  117  LTGVLYLSQAFIPHMRARKQGSIACMSSVSAQRGGGIFGGPHYSAAKAGVLGLAKAMARE  176

Query  52   PGPQSVRVSDVE-GVLQ  67
             GP  +RV+ V  G++Q
Sbjct  177  LGPDGIRVNSVTPGLIQ  193


>ref|WP_048990457.1| short-chain dehydrogenase [Klebsiella pneumoniae]
Length=249

 Score = 36.6 bits (83),  Expect = 3.9, Method: Compositional matrix adjust.
 Identities = 22/73 (30%), Positives = 38/73 (52%), Gaps = 10/73 (14%)

Query  5    LLSSRTFSPTLRSRRSSPL---------EGGDVFAGAHTSAASKHAVCQAEGLSAAPGPQ  55
            LL ++   PT+R+++S  +          GG +F G H SAA    +  A  ++   GP 
Sbjct  117  LLMTQAVIPTMRAQKSGSIVCISSVSAQRGGGIFGGPHYSAAKAGVLGLARAMARELGPD  176

Query  56   SVRVSDVE-GVLQ  67
            +VRV+ +  G++Q
Sbjct  177  NVRVNCITPGLIQ  189


>ref|WP_013973349.1| MULTISPECIES: short-chain dehydrogenase [Pseudomonas]
 gb|AEJ14025.1| putative short-chain type regulator [Pseudomonas putida S16]
 gb|AGA74350.1| putative short-chain type regulator [Pseudomonas putida HB3267]
 7 more sequence titles

gb|ESW38034.1| short-chain dehydrogenase [Pseudomonas taiwanensis SJ9]
 gb|AHC83496.1| short-chain dehydrogenase [Pseudomonas monteilii SB3078]
 gb|AHC88872.1| short-chain dehydrogenase [Pseudomonas monteilii SB3101]
 gb|AHD15397.1| short-chain dehydrogenase [Pseudomonas sp. FGI182]
 gb|AHZ78430.1| short-chain type regulator [Pseudomonas putida]
 gb|KGK26498.1| short-chain dehydrogenase [Pseudomonas plecoglossicida]
 gb|AJG12632.1| putative short-chain type regulator [Pseudomonas plecoglossicida]

Length=249

 Score = 36.6 bits (83),  Expect = 3.9, Method: Compositional matrix adjust.
 Identities = 19/67 (28%), Positives = 34/67 (51%), Gaps = 9/67 (13%)

Query  5    LLSSRTFSPTLRSRRSSPL---------EGGDVFAGAHTSAASKHAVCQAEGLSAAPGPQ  55
            LL S+   PT+R++R+  +          GG +F G H SAA    +   + ++   GP 
Sbjct  117  LLMSQAVIPTMRAQRAGSIICMSSVSAQRGGGIFGGPHYSAAKAGVLGLGKAMARELGPD  176

Query  56   SVRVSDV  62
            ++RV+ +
Sbjct  177  NIRVNSI  183


>gb|EJT84616.1| putative short-chain type regulator [Pseudomonas putida S11]
Length=249

 Score = 36.6 bits (83),  Expect = 4.0, Method: Compositional matrix adjust.
 Identities = 19/67 (28%), Positives = 34/67 (51%), Gaps = 9/67 (13%)

Query  5    LLSSRTFSPTLRSRRSSPL---------EGGDVFAGAHTSAASKHAVCQAEGLSAAPGPQ  55
            LL S+   PT+R++R+  +          GG +F G H SAA    +   + ++   GP 
Sbjct  117  LLMSQAVIPTMRAQRAGSIICMSSVSAQRGGGIFGGPHYSAAKAGVLGLGKAMARELGPD  176

Query  56   SVRVSDV  62
            ++RV+ +
Sbjct  177  NIRVNSI  183


>ref|WP_010799082.1| hypothetical protein [Pseudomonas sp. HPB0071]
 gb|ENA28350.1| hypothetical protein HMPREF1487_08926 [Pseudomonas sp. HPB0071]
Length=249

 Score = 36.2 bits (82),  Expect = 5.0, Method: Compositional matrix adjust.
 Identities = 22/73 (30%), Positives = 37/73 (51%), Gaps = 10/73 (14%)

Query  5    LLSSRTFSPTLRSRRSSPL---------EGGDVFAGAHTSAASKHAVCQAEGLSAAPGPQ  55
            LL S+   P +R ++S  +          GG +F G H SAA    +  A+ ++   GP 
Sbjct  117  LLMSQAVIPAMRQQKSGSIVCLSSVSAQRGGGIFGGPHYSAAKAGVLGLAKAMARELGPD  176

Query  56   SVRVSDVE-GVLQ  67
            +VRV+ +  G++Q
Sbjct  177  NVRVNSITPGLIQ  189


>ref|WP_019364260.1| short-chain dehydrogenase [Pseudomonas luteola]
Length=249

 Score = 36.2 bits (82),  Expect = 5.2, Method: Compositional matrix adjust.
 Identities = 22/73 (30%), Positives = 37/73 (51%), Gaps = 10/73 (14%)

Query  5    LLSSRTFSPTLRSRRSSPL---------EGGDVFAGAHTSAASKHAVCQAEGLSAAPGPQ  55
            LL S+   P +R ++S  +          GG +F G H SAA    +  A+ ++   GP 
Sbjct  117  LLMSQAVIPAMRQQKSGSIVCLSSVSAQRGGGIFGGPHYSAAKAGVLGLAKAMARELGPD  176

Query  56   SVRVSDVE-GVLQ  67
            +VRV+ +  G++Q
Sbjct  177  NVRVNSITPGLIQ  189


>ref|WP_050467269.1| short-chain dehydrogenase [Herbaspirillum chlorophenolicum]
Length=249

 Score = 36.2 bits (82),  Expect = 5.3, Method: Compositional matrix adjust.
 Identities = 21/67 (31%), Positives = 32/67 (48%), Gaps = 9/67 (13%)

Query  5    LLSSRTFSPTLRSRRSSPL---------EGGDVFAGAHTSAASKHAVCQAEGLSAAPGPQ  55
            L  S+   P +RSRRS  +          GG +F G H SAA    +  A  ++   GP 
Sbjct  117  LYMSQALIPHMRSRRSGSIVCLSSVSAQRGGGIFGGPHYSAAKAGVLGLARAMARELGPD  176

Query  56   SVRVSDV  62
            ++RV+ +
Sbjct  177  NIRVNSL  183


>ref|WP_019437635.1| 3-ketoacyl-ACP reductase [Pseudomonas putida]
 gb|AJA13591.1| short-chain dehydrogenase [Pseudomonas putida S12]
Length=249

 Score = 36.2 bits (82),  Expect = 5.5, Method: Compositional matrix adjust.
 Identities = 21/67 (31%), Positives = 33/67 (49%), Gaps = 9/67 (13%)

Query  5    LLSSRTFSPTLRSRRSSPL---------EGGDVFAGAHTSAASKHAVCQAEGLSAAPGPQ  55
            LL S+   P +R +RS  +          GG +F G H SAA    +  A+ ++   GP 
Sbjct  117  LLMSQAVIPLMREQRSGSIVCMSSVSAQRGGGIFGGPHYSAAKAGVLGLAKAMARELGPD  176

Query  56   SVRVSDV  62
            +VRV+ +
Sbjct  177  NVRVNAI  183


>ref|WP_014589181.1| short-chain dehydrogenase [Pseudomonas putida]
 gb|ADR57750.1| Short-chain dehydrogenase/reductase [Pseudomonas putida BIRD-1]
Length=249

 Score = 36.2 bits (82),  Expect = 5.6, Method: Compositional matrix adjust.
 Identities = 21/67 (31%), Positives = 33/67 (49%), Gaps = 9/67 (13%)

Query  5    LLSSRTFSPTLRSRRSSPL---------EGGDVFAGAHTSAASKHAVCQAEGLSAAPGPQ  55
            LL S+   P +R +RS  +          GG +F G H SAA    +  A+ ++   GP 
Sbjct  117  LLMSQAVIPLMREQRSGSIVCMSSVSAQRGGGIFGGPHYSAAKAGVLGLAKAMARELGPD  176

Query  56   SVRVSDV  62
            +VRV+ +
Sbjct  177  NVRVNAI  183


>ref|WP_044183479.1| short-chain dehydrogenase [Enterobacter massiliensis]
Length=249

 Score = 36.2 bits (82),  Expect = 5.7, Method: Compositional matrix adjust.
 Identities = 22/73 (30%), Positives = 37/73 (51%), Gaps = 10/73 (14%)

Query  5    LLSSRTFSPTLRSRRSSPL---------EGGDVFAGAHTSAASKHAVCQAEGLSAAPGPQ  55
            L+ S+   PTLR+++S  +          GG +F G H SAA    +     ++   GP 
Sbjct  117  LIMSQAVIPTLRAQKSGSIVCISSVSAQRGGGIFGGPHYSAAKAGVLGLTRAMARELGPD  176

Query  56   SVRVSDVE-GVLQ  67
            +VRV+ +  G++Q
Sbjct  177  NVRVNAITPGLIQ  189


>ref|WP_014754902.1| short-chain dehydrogenase [Pseudomonas putida]
 gb|AFK69463.1| Short-chain dehydrogenase/reductase [Pseudomonas putida ND6]
Length=249

 Score = 36.2 bits (82),  Expect = 5.8, Method: Compositional matrix adjust.
 Identities = 21/67 (31%), Positives = 33/67 (49%), Gaps = 9/67 (13%)

Query  5    LLSSRTFSPTLRSRRSSPL---------EGGDVFAGAHTSAASKHAVCQAEGLSAAPGPQ  55
            LL S+   P +R +RS  +          GG +F G H SAA    +  A+ ++   GP 
Sbjct  117  LLMSQAVIPLMREQRSGSIVCMSSVSAQRGGGIFGGPHYSAAKAGVLGLAKAMARELGPD  176

Query  56   SVRVSDV  62
            +VRV+ +
Sbjct  177  NVRVNAI  183


>ref|WP_012054137.1| short-chain dehydrogenase [Pseudomonas putida]
 gb|ABQ81396.1| short-chain dehydrogenase/reductase SDR [Pseudomonas putida F1]
Length=249

 Score = 35.8 bits (81),  Expect = 6.1, Method: Compositional matrix adjust.
 Identities = 21/67 (31%), Positives = 33/67 (49%), Gaps = 9/67 (13%)

Query  5    LLSSRTFSPTLRSRRSSPL---------EGGDVFAGAHTSAASKHAVCQAEGLSAAPGPQ  55
            LL S+   P +R +RS  +          GG +F G H SAA    +  A+ ++   GP 
Sbjct  117  LLMSQAVIPLMREQRSGSIVCMSSVSAQRGGGIFGGPHYSAAKAGVLGLAKAMARELGPD  176

Query  56   SVRVSDV  62
            +VRV+ +
Sbjct  177  NVRVNAI  183


>ref|WP_019752827.1| 3-ketoacyl-ACP reductase [Pseudomonas putida]
Length=249

 Score = 35.8 bits (81),  Expect = 6.1, Method: Compositional matrix adjust.
 Identities = 21/67 (31%), Positives = 33/67 (49%), Gaps = 9/67 (13%)

Query  5    LLSSRTFSPTLRSRRSSPL---------EGGDVFAGAHTSAASKHAVCQAEGLSAAPGPQ  55
            LL S+   P +R +RS  +          GG +F G H SAA    +  A+ ++   GP 
Sbjct  117  LLMSQAVIPLMRGQRSGSIVCMSSVSAQRGGGIFGGPHYSAAKAGVLGLAKAMARELGPD  176

Query  56   SVRVSDV  62
            +VRV+ +
Sbjct  177  NVRVNAI  183


>ref|WP_005735172.1| short-chain dehydrogenase [Pseudomonas amygdali]
 gb|EGH06027.1| 3-oxoacyl-(acyl-carrier-protein) reductase [Pseudomonas amygdali 
pv. aesculi str. 0893_23]
Length=249

 Score = 35.4 bits (80),  Expect = 8.5, Method: Compositional matrix adjust.
 Identities = 21/67 (31%), Positives = 33/67 (49%), Gaps = 9/67 (13%)

Query  5    LLSSRTFSPTLRSRRSSPL---------EGGDVFAGAHTSAASKHAVCQAEGLSAAPGPQ  55
            LL S+   PT+R++ S  +          GG +F G H SAA   A+   + ++   GP 
Sbjct  117  LLMSQAVIPTMRAQSSGSIVCMSSVSAQRGGGIFGGPHYSAAKAGALGLGKAMAREFGPD  176

Query  56   SVRVSDV  62
             VRV+ +
Sbjct  177  QVRVNSI  183


>ref|WP_012319952.1| MULTISPECIES: short-chain dehydrogenase [Bacteria]
 gb|ACB24985.1| short-chain dehydrogenase/reductase SDR [Methylobacterium radiotolerans 
JCM 2831]
 gb|KIU32420.1| short-chain dehydrogenase [Methylobacterium radiotolerans]
 dbj|GAN51030.1| 3-oxoacyl-ACP reductase [Methylobacterium sp. ME121]
 gb|KOX54550.1| short-chain dehydrogenase [Asanoa ferruginea]
Length=249

 Score = 35.4 bits (80),  Expect = 8.6, Method: Compositional matrix adjust.
 Identities = 24/73 (33%), Positives = 35/73 (48%), Gaps = 10/73 (14%)

Query  5    LLSSRTFSPTLRSRRSSPL---------EGGDVFAGAHTSAASKHAVCQAEGLSAAPGPQ  55
            L  S+   PT+R R S  +          GG +F G H SAA    +  A+ ++   GP 
Sbjct  117  LYMSQAVIPTMRGRGSGAIVCMSSVSAQRGGGIFGGPHYSAAKGGVLGLAKAMARELGPD  176

Query  56   SVRVSDVE-GVLQ  67
             VRV+ V  G++Q
Sbjct  177  GVRVNSVTPGLIQ  189


>ref|WP_019649740.1| short-chain dehydrogenase [Pseudomonas sp. 45MFCol3.1]
Length=249

 Score = 35.4 bits (80),  Expect = 9.1, Method: Compositional matrix adjust.
 Identities = 21/67 (31%), Positives = 32/67 (48%), Gaps = 9/67 (13%)

Query  5    LLSSRTFSPTLRSRRSSPL---------EGGDVFAGAHTSAASKHAVCQAEGLSAAPGPQ  55
            LL S+   PT+R + S  +          GG +F G H SAA    +  A+ ++   GP 
Sbjct  117  LLMSQAVIPTMRQQSSGSIVCMSSVSAQRGGGIFGGPHYSAAKAGVLGLAKAMARELGPD  176

Query  56   SVRVSDV  62
             VRV+ +
Sbjct  177  KVRVNSI  183


>ref|WP_045792147.1| short-chain dehydrogenase [Pseudomonas fluorescens]
 gb|AJZ96077.1| 3-ketoacyl-ACP reductase [Pseudomonas fluorescens PICF7]
Length=249

 Score = 35.4 bits (80),  Expect = 9.6, Method: Compositional matrix adjust.
 Identities = 21/67 (31%), Positives = 32/67 (48%), Gaps = 9/67 (13%)

Query  5    LLSSRTFSPTLRSRRSSPL---------EGGDVFAGAHTSAASKHAVCQAEGLSAAPGPQ  55
            LL S+   PT+R + S  +          GG +F G H SAA    +  A+ ++   GP 
Sbjct  117  LLMSQAVIPTMRKQSSGSIVCMSSVSAQRGGGIFGGPHYSAAKAGVLGLAKAMARELGPD  176

Query  56   SVRVSDV  62
             VRV+ +
Sbjct  177  KVRVNSI  183


Lambda      K        H        a         alpha
   0.317    0.127    0.387    0.792     4.96 

Gapped
Lambda      K        H        a         alpha    sigma
   0.267   0.0410    0.140     1.90     42.6     43.6 

Effective search space used: 654714264532


  Database: nr
    Posted date:  Sep 23, 2015 12:05 AM
  Number of letters in database: 26,053,659,533
  Number of sequences in database:  71,551,133


Matrix: BLOSUM62
Gap Penalties: Existence: 11, Extension: 1
Neighboring words threshold: 11
Window for multiple hits: 40
```
