# Supplementary material for: Comparative genomics provides new insights into the diversity, physiology, and sexuality of the only industrially exploited tremellomycete: Phaffia rhodozyma
Source: BMC Genomics. 2016 Nov 9;17:901. doi: 10.1186/s12864-016-3244-7 (PMC5103461; doi:10.1186/s12864-016-3244-7)
Supplement: Additional file 6: — List of orphan genes with links to PFAM (related to Additional file 1: Table S1). (ZIP 1428 kb) [file 12864_2016_3244_MOESM6_ESM.zip › BLAST_HTML_FTR/G04292_P.html]

BLAST Search Results


```
BLASTP 2.2.27+


Reference:
Stephen F. Altschul, Thomas L. Madden, Alejandro A. Schäffer,
Jinghui Zhang, Zheng Zhang, Webb Miller, and David J. Lipman (1997),
"Gapped BLAST and PSI-BLAST: a new generation of protein database
search programs", Nucleic Acids Res. 25:3389-3402.


Reference for
composition-based statistics:
Alejandro A. Schäffer, L. Aravind, Thomas L. Madden, Sergei
Shavirin, John L. Spouge, Yuri I. Wolf, Eugene V. Koonin, and
Stephen F. Altschul (2001), "Improving the accuracy of PSI-BLAST
protein database searches with composition-based statistics and
other refinements", Nucleic Acids Res. 29:2994-3005.


Database: nr
           71,551,133 sequences; 26,053,659,533 total letters


Query= G04292_P

Length=424
                                                                      Score     E
Sequences producing significant alignments:                          (Bits)  Value

emb|CDZ97860.1|  hypothetical protein [Xanthophyllomyces dendrorh...   830    0.0  
gb|KDQ28903.1|  hypothetical protein PLEOSDRAFT_1038578 [Pleurotu...  50.1    0.003
gb|KLO15777.1|  hypothetical protein SCHPADRAFT_995640 [Schizopor...  47.4    0.024
gb|KIY45144.1|  hypothetical protein FISHEDRAFT_61320 [Fistulina ...  47.0    0.031
gb|KDQ65014.1|  hypothetical protein JAAARDRAFT_28677 [Jaapia arg...  46.2    0.046
emb|CDO74118.1|  hypothetical protein BN946_scf185043.g168 [Trame...  45.4    0.082
gb|KII95998.1|  hypothetical protein PLICRDRAFT_48921 [Plicaturop...  45.1    0.10 
ref|XP_009544190.1|  hypothetical protein HETIRDRAFT_473198 [Hete...  43.5    0.41 
gb|KIK42754.1|  hypothetical protein CY34DRAFT_754940 [Suillus lu...  41.6    1.4  
ref|XP_008035052.1|  hypothetical protein TRAVEDRAFT_44622 [Trame...  41.6    1.4  
emb|CCO28170.1|  hypothetical protein BN14_02163 [Rhizoctonia sol...  40.4    2.9  
gb|KIK07655.1|  hypothetical protein K443DRAFT_673233 [Laccaria a...  40.0    4.4  
gb|KDQ20597.1|  hypothetical protein BOTBODRAFT_26601 [Botryobasi...  39.7    6.1  
ref|XP_007381781.1|  hypothetical protein PUNSTDRAFT_125204 [Punc...  39.3    8.3  


 >emb|CDZ97860.1| hypothetical protein [Xanthophyllomyces dendrorhous]
Length=423

 Score =  830 bits (2143),  Expect = 0.0, Method: Compositional matrix adjust.
 Identities = 423/423 (100%), Positives = 423/423 (100%), Gaps = 0/423 (0%)

Query  1    MSNSHAGSRSALSSTSPGLSIPTTGRHKVVLDPAMIRALVGSIDHGAHSQGDEIEYDAFK  60
            MSNSHAGSRSALSSTSPGLSIPTTGRHKVVLDPAMIRALVGSIDHGAHSQGDEIEYDAFK
Sbjct  1    MSNSHAGSRSALSSTSPGLSIPTTGRHKVVLDPAMIRALVGSIDHGAHSQGDEIEYDAFK  60

Query  61   YKFKPSSILTSKPGTITPTASKDSSSSLSGSATVHLQGPLEGEVHGFAAKVESEGSARAS  120
            YKFKPSSILTSKPGTITPTASKDSSSSLSGSATVHLQGPLEGEVHGFAAKVESEGSARAS
Sbjct  61   YKFKPSSILTSKPGTITPTASKDSSSSLSGSATVHLQGPLEGEVHGFAAKVESEGSARAS  120

Query  121  GRPGSCQNDHLAVLKWDGKQFSLLPISKTYSLVHDKSLALDSLPVAHSPPPQSERAYKHQ  180
            GRPGSCQNDHLAVLKWDGKQFSLLPISKTYSLVHDKSLALDSLPVAHSPPPQSERAYKHQ
Sbjct  121  GRPGSCQNDHLAVLKWDGKQFSLLPISKTYSLVHDKSLALDSLPVAHSPPPQSERAYKHQ  180

Query  181  QPSTTLISSSTEKRHRPTSPPSSSAPATRDVHMSSSASSSRSSSPEHSVQPRPTVPALPS  240
            QPSTTLISSSTEKRHRPTSPPSSSAPATRDVHMSSSASSSRSSSPEHSVQPRPTVPALPS
Sbjct  181  QPSTTLISSSTEKRHRPTSPPSSSAPATRDVHMSSSASSSRSSSPEHSVQPRPTVPALPS  240

Query  241  ASQPRPTSKPPLSSSSINPSLPTRPTSQPSVVNPDVEILSFPTSSISTSYAPMPSVPAPL  300
            ASQPRPTSKPPLSSSSINPSLPTRPTSQPSVVNPDVEILSFPTSSISTSYAPMPSVPAPL
Sbjct  241  ASQPRPTSKPPLSSSSINPSLPTRPTSQPSVVNPDVEILSFPTSSISTSYAPMPSVPAPL  300

Query  301  PARPMYGGKSFSHSPLMGSPTIPHPQPPADGSSDDDDSDSSESEGEDDDDDFAAQLKQEL  360
            PARPMYGGKSFSHSPLMGSPTIPHPQPPADGSSDDDDSDSSESEGEDDDDDFAAQLKQEL
Sbjct  301  PARPMYGGKSFSHSPLMGSPTIPHPQPPADGSSDDDDSDSSESEGEDDDDDFAAQLKQEL  360

Query  361  AGVSAGGKKAGKGAFGVVGGKGLGLGLGTGHVQGAGKRPVSLNSLMGVEDDDLEQESPSS  420
            AGVSAGGKKAGKGAFGVVGGKGLGLGLGTGHVQGAGKRPVSLNSLMGVEDDDLEQESPSS
Sbjct  361  AGVSAGGKKAGKGAFGVVGGKGLGLGLGTGHVQGAGKRPVSLNSLMGVEDDDLEQESPSS  420

Query  421  DED  423
            DED
Sbjct  421  DED  423


>gb|KDQ28903.1| hypothetical protein PLEOSDRAFT_1038578 [Pleurotus ostreatus 
PC15]
Length=431

 Score = 50.1 bits (118),  Expect = 0.003, Method: Compositional matrix adjust.
 Identities = 39/147 (27%), Positives = 68/147 (46%), Gaps = 19/147 (13%)

Query  21   IPTTGRHKVVLDPAMIRALVGSIDHGAHSQGDEI---EYDAFKYKFKPSSILTSKPGTIT  77
            +PTTGRH+V +  ++ RAL       A ++   +   ++ +F+Y FKP S+  +KPGT  
Sbjct  9    MPTTGRHRVDIGSSLGRALKARKGGAAPAKRSNLPSRDFYSFRYTFKPPSVDNTKPGTAE  68

Query  78   PTASKDSSSSLSGSATVHLQGPLEGEVHGFAAKVESEGSARASGRPGSCQNDHLAVLKWD  137
             TA K+S+S +    +       EGE   F    E            + + D + +   +
Sbjct  69   MTAGKESTSVMVEHPSTQ-----EGESFIFKGPEEP-----------AKELDCVLIYDEE  112

Query  138  GKQFSLLPISKTYSLVHDKSLALDSLP  164
               F+L  +    +L +DK  A  ++P
Sbjct  113  TGTFTLERVESFVALKYDKKTASSTMP  139


>gb|KLO15777.1| hypothetical protein SCHPADRAFT_995640 [Schizopora paradoxa]
Length=535

 Score = 47.4 bits (111),  Expect = 0.024, Method: Compositional matrix adjust.
 Identities = 38/140 (27%), Positives = 62/140 (44%), Gaps = 22/140 (16%)

Query  21   IPTTGRHKVVLDPAMIRALVGSIDHGAHSQGDE--IEYDAFKYKFKPSSILTSKPGTITP  78
            +P  GRHKV +  ++ RAL       A +  +    +Y  F+Y FKP S+ TSKPG +  
Sbjct  14   VPPPGRHKVDVGGSLKRALRARKGMPAPTNKNMPMSDYYLFRYNFKPESVDTSKPGVVEV  73

Query  79   TASKDSSSSLSGSATVHLQGPL--EGEVHGFAAKVESEGSARASGRPGSCQNDHLAVLKW  136
               K+++       TV ++ P+    +VH F    E            S + + + +   
Sbjct  74   KRGKEAT-------TVQVERPIVQSEDVHFFTGNEEP-----------SKEVECVLIFDE  115

Query  137  DGKQFSLLPISKTYSLVHDK  156
            +   F+L  +  T SL H K
Sbjct  116  ETNTFTLEKLDSTLSLTHSK  135


>gb|KIY45144.1| hypothetical protein FISHEDRAFT_61320 [Fistulina hepatica ATCC 
64428]
Length=443

 Score = 47.0 bits (110),  Expect = 0.031, Method: Compositional matrix adjust.
 Identities = 57/200 (29%), Positives = 87/200 (44%), Gaps = 28/200 (14%)

Query  21   IPTTGRHKVVLDPAMIRALVGSIDH-GAHSQGDEIEYDAFKYKFKPSSILTSKPGTITPT  79
            +P  G H+V +  ++ RAL        A S+  E ++ +F+Y FKP S+  +KPG ++ T
Sbjct  10   LPVQGLHQVNIGSSLTRALKARNGMPAAKSKFPERDFYSFRYNFKPPSVDATKPGNLSIT  69

Query  80   ASKDSSSSLSGSATVHLQGPLEGEVHGFAAKVESEGSARASGRPGSCQNDHLAVLKWDGK  139
             S D+S      ATV  Q    G+ H F       GSA  +      + D + +   + +
Sbjct  70   RSGDTS-----QATV-TQPSQTGDTHMFI------GSASPAK-----EVDCILIYDEETQ  112

Query  140  QFSLLPISKTYSLVHDKSL---ALDSLPVAHSPPPQSERAYKHQQPSTTLISSSTEKRHR  196
             F L  +    SL ++K +   A D LP      PQ     + ++ S      S E    
Sbjct  113  TFMLEKLDSYMSLKYEKKISAQADDVLPAM----PQEALKEEEEEASEEEGEISMEPTGL  168

Query  197  PTSPPS---SSAPATRDVHM  213
            P  PPS    SAP  R  H+
Sbjct  169  PQRPPSPRPVSAPPARPAHL  188


>gb|KDQ65014.1| hypothetical protein JAAARDRAFT_28677 [Jaapia argillacea MUCL 
33604]
Length=428

 Score = 46.2 bits (108),  Expect = 0.046, Method: Compositional matrix adjust.
 Identities = 44/162 (27%), Positives = 71/162 (44%), Gaps = 25/162 (15%)

Query  21   IPTTGRHKVVLDPAMIRALVGS--IDHGAHSQGDEIEYDAFKYKFKPSSILTSKPGTITP  78
            +P TGRH+V +  ++ RAL        GA  +  +  + + +Y FKP SI  SK G+I  
Sbjct  6    MPATGRHQVEIGSSLGRALKARKGTSIGAAKRIPDRNFHSCRYNFKPESIDPSKTGSIEV  65

Query  79   TASKDSSSSLSGSATVHLQGPLEGEVHGFAAKVESEGSARASGRPGSCQNDHLAVLKWDG  138
               KD++     S TV       GE H F         A    +   C      VL +D 
Sbjct  66   KKGKDNT-----SVTVERASTQAGENHIFVG-------AELPAKEWEC------VLIYDE  107

Query  139  K--QFSLLPISKTYSLVHDK---SLALDSLPVAHSPPPQSER  175
            +  +F+L  +     L +D+   ++   + P++  PPP  E+
Sbjct  108  ELGRFTLEKLDSYVQLNYDRKSSTMTASARPISPLPPPADEK  149


>emb|CDO74118.1| hypothetical protein BN946_scf185043.g168 [Trametes cinnabarina]
Length=502

 Score = 45.4 bits (106),  Expect = 0.082, Method: Compositional matrix adjust.
 Identities = 39/137 (28%), Positives = 66/137 (48%), Gaps = 21/137 (15%)

Query  24   TGRHKVVLDPAMIRALV---GSIDHGAHSQGDEIEYDAFKYKFKPSSILTSKPGTITPTA  80
            TGRH++ +  +++RAL    G     + ++ D  E+ +F+Y FKP S+  +KPG+I    
Sbjct  12   TGRHEINIGSSLMRALKARKGGPVKNSKAKPDR-EFYSFRYNFKPESVDPTKPGSIEVKR  70

Query  81   SKDSSSSLSGSATVHLQGPLEGEVHGFAAKVESEGSARASGRPGSCQNDHLAVLKWDGK-  139
             +D     +G  +V++  P     HG    V  E +AR          ++  VL +D + 
Sbjct  71   PRDE----AGPTSVNVVRPSTQNDHGVNF-VGQEKAAR----------EYDCVLIYDEEL  115

Query  140  -QFSLLPISKTYSLVHD  155
              F+L  I    +L HD
Sbjct  116  GTFTLEKIESCVTLHHD  132


>gb|KII95998.1| hypothetical protein PLICRDRAFT_48921 [Plicaturopsis crispa FD-325 
SS-3]
Length=405

 Score = 45.1 bits (105),  Expect = 0.10, Method: Compositional matrix adjust.
 Identities = 39/146 (27%), Positives = 67/146 (46%), Gaps = 23/146 (16%)

Query  14   STSPGLSIPTTGRHKVVLDPAMIRALVGSIDHGAHSQGD---EIEYDAFKYKFKPSSILT  70
            +T+    +P  GRH+V+L  ++ RAL       A ++     + ++ + +Y FKP S+ +
Sbjct  2    ATTSNKWMPLKGRHEVLLGSSLRRALKARKGTAAPNKKAGPPDRDFYSLRYNFKPESVDS  61

Query  71   SKPGTITPTASKDSSSSLSGSATVHLQGPLEGEVHGFAAKVESEGSARASGRPGSCQNDH  130
             KPGTI     KD++     + TV       GE+H F  +   E +A+          D 
Sbjct  62   VKPGTIEVKRGKDAT-----TVTVERSSSQGGEMHVFTGQ---EQAAK----------DW  103

Query  131  LAVLKWDGK--QFSLLPISKTYSLVH  154
              VL +D +   F+L  +    +L H
Sbjct  104  ECVLIYDEELGTFTLEKLDSFVNLNH  129


>ref|XP_009544190.1| hypothetical protein HETIRDRAFT_473198 [Heterobasidion irregulare 
TC 32-1]
 gb|ETW84529.1| hypothetical protein HETIRDRAFT_473198 [Heterobasidion irregulare 
TC 32-1]
Length=449

 Score = 43.5 bits (101),  Expect = 0.41, Method: Compositional matrix adjust.
 Identities = 49/186 (26%), Positives = 78/186 (42%), Gaps = 29/186 (16%)

Query  13   SSTSPGLSIPTTGRHKVVLDPAMIRALVGSI-DHGAHSQGDEIEYDAFKYKFKPSSILTS  71
            SSTSP    P  GR++V +  ++++ L   +  + A     E ++ +F+Y FKP SI  +
Sbjct  3    SSTSPW--SPAPGRYEVQMGQSLVKDLKMRMGTYNAKRALPEKDFYSFRYNFKPESIDPT  60

Query  72   KPGTITPTASKDSSSSLSGSATVHLQGPLEGEVHGFAAKVESEGSARASGRPGSCQNDHL  131
            KPGTI     K+ +       T  L      E H F      EG  + +        D  
Sbjct  61   KPGTIEVQQGKEGTGVRVERPTSQL-----NEAHEF------EGDEKPA-------KDFE  102

Query  132  AVLKWD--GKQFSLLPISKTYSLVHDKSLALDSLPVA------HSPPPQSERAYKHQQPS  183
             VL +D     ++L  +    SL H + +   + P A      H+P P S      + P+
Sbjct  103  CVLIYDPGMGTYTLEKLDSLISLNHKRRVTTRARPSASASPLPHAPEPPSNARSAFKAPT  162

Query  184  TTLISS  189
            T  I +
Sbjct  163  TEEIEA  168


>gb|KIK42754.1| hypothetical protein CY34DRAFT_754940 [Suillus luteus UH-Slu-Lm8-n1]
Length=395

 Score = 41.6 bits (96),  Expect = 1.4, Method: Compositional matrix adjust.
 Identities = 50/198 (25%), Positives = 78/198 (39%), Gaps = 51/198 (26%)

Query  21   IPTTGRHKVVLDPAMIRALVGS--IDHGAHSQGDEIEYDAFKYKFKPSSILTSKPGTITP  78
            +PTTGRH V +  ++ RAL     +     S   + ++ +F+Y FKP SI  S+ GTI  
Sbjct  9    MPTTGRHSVAIGTSLRRALRARKGVAPPKRSNLPDKDFYSFRYNFKPESIEPSRSGTIEV  68

Query  79   TASKDSSSSLSGSATVHLQGP--LEGEVHGFAAKVESEGSARASGRPGSCQNDHLAVLKW  136
               KD +S       + L+ P    GE H F         A    +   C      VL +
Sbjct  69   KRGKDLTS-------ITLERPSTQAGENHLFKG-------AEQQVKEYDC------VLIY  108

Query  137  DGK--QFSLLPISKTYSLVHDKSLALDSLPVAHSPPPQSERAYKHQQPSTTLISSSTEKR  194
            D +   F+L  +       +DK +            P S  ++  + PS           
Sbjct  109  DEELGTFTLEKVECFMGFTYDKKV------------PASSSSFARKSPSA----------  146

Query  195  HRPTSPPSSSAPATRDVH  212
               T+PP++    TRD+ 
Sbjct  147  ---TTPPAAQLKDTRDLE  161


>ref|XP_008035052.1| hypothetical protein TRAVEDRAFT_44622 [Trametes versicolor FP-101664 
SS1]
 gb|EIW61799.1| hypothetical protein TRAVEDRAFT_44622 [Trametes versicolor FP-101664 
SS1]
Length=483

 Score = 41.6 bits (96),  Expect = 1.4, Method: Compositional matrix adjust.
 Identities = 42/149 (28%), Positives = 72/149 (48%), Gaps = 22/149 (15%)

Query  12   LSSTSPGLSIPTTGRHKVVLDPAMIRAL---VGSIDHGAHSQGDEIEYDAFKYKFKPSSI  68
            ++ST+    +PT GRH++ +  +++R+L    G     + ++ D  E+ +F+Y FKP S+
Sbjct  1    MTSTASNAWMPT-GRHEINIGTSLMRSLKARKGGPVKNSKAKPDR-EFYSFRYNFKPESV  58

Query  69   LTSKPGTITPTASKDSSSSLSGSATVHLQGPLEGEVHGFAAKVESEGSARASGRPGSCQN  128
              +KPG+I    +K+      G ++V++  P      G    V   G  R S R   C  
Sbjct  59   DPTKPGSIEIKKAKEE----GGPSSVNVTRPSTQNETG----VNYVGQER-SARDVDC--  107

Query  129  DHLAVLKWDG--KQFSLLPISKTYSLVHD  155
                VL +D   + F+L  I    +L HD
Sbjct  108  ----VLIYDEELQTFTLEKIETYLTLQHD  132


>emb|CCO28170.1| hypothetical protein BN14_02163 [Rhizoctonia solani AG-1 IB]
Length=434

 Score = 40.4 bits (93),  Expect = 2.9, Method: Compositional matrix adjust.
 Identities = 34/98 (35%), Positives = 48/98 (49%), Gaps = 16/98 (16%)

Query  18   GLSIPTTGRHKVVLDPAMIRALVGSIDHGAHSQGDEIE--YDAFKYKFKPSSILTSKPGT  75
            G+++P  GR  V LD +++R L       A  + + I+  Y +FKY FKP SI   KPG 
Sbjct  12   GVNVPE-GRCTVDLDDSVLRML-----EPAQHRKNPIQSSYHSFKYHFKPESIDVRKPGK  65

Query  76   IT----PTASKDSSSSLSGSATVHLQGPLEGEVHGFAA  109
            +     P A+ D   +L     V L GP   + H F A
Sbjct  66   VQMPNPPRATGDGGVALD----VELLGPNPEDKHLFTA  99


>gb|KIK07655.1| hypothetical protein K443DRAFT_673233 [Laccaria amethystina LaAM-08-1]
Length=437

 Score = 40.0 bits (92),  Expect = 4.4, Method: Compositional matrix adjust.
 Identities = 39/143 (27%), Positives = 61/143 (43%), Gaps = 26/143 (18%)

Query  25   GRHKVVLDPAMIRALVGSIDHGA------HSQGDEIEYDAFKYKFKPSSILTSKPGTITP  78
            GRH V +  ++ RAL      G+       S   + ++ +F+YKFKP S+  +KPGTI  
Sbjct  12   GRHPVNVGSSLGRALKARKLKGSEPATAKRSNLPDRDFYSFRYKFKPPSVDMTKPGTIEV  71

Query  79   TASKDSSSSLSGSATVHLQGPLEGEVHGFAAKVESEGSARASGRPGSCQNDHLAVLKWDG  138
                DSS     S TV   G   GE   F         + A+ +   C      VL +D 
Sbjct  72   KRGGDSS-----SVTVEYPGSQAGEKQVFMG-------SEAAAKEWEC------VLIYDE  113

Query  139  K--QFSLLPISKTYSLVHDKSLA  159
            +   ++L  +    +L + +  A
Sbjct  114  ETGTYTLEKLDSCMTLTYQRKAA  136


>gb|KDQ20597.1| hypothetical protein BOTBODRAFT_26601 [Botryobasidium botryosum 
FD-172 SS1]
Length=435

 Score = 39.7 bits (91),  Expect = 6.1, Method: Compositional matrix adjust.
 Identities = 38/142 (27%), Positives = 63/142 (44%), Gaps = 24/142 (17%)

Query  22   PTTGRHKVVLDPAMIRALVGSIDHGAHSQGDEIEYDAFKYKFKPSSILTSKPGTITPTAS  81
            P  G+  V +  + +RAL    +  A    D+  Y +FKYKF+P S+  ++PG I     
Sbjct  12   PPAGKCAVNVGASFLRALQLPKNFPASGSTDDDHY-SFKYKFRPESVDATRPGRI-----  65

Query  82   KDSSSSLS-GSATVHLQ-GPLEGEVHGFAAKVESEGSARASGRPGSCQNDHLAVLKW--D  137
             D+   LS G +T  L+ G  + E H F    +                D   VL +  D
Sbjct  66   -DNLQPLSDGRSTFQLERGGTQNERHIFTGTQQ-------------IPKDWECVLIYDED  111

Query  138  GKQFSLLPISKTYSLVHDKSLA  159
             + F+L  +    +L +D++L+
Sbjct  112  TQSFTLERLDACMNLTYDQALS  133


>ref|XP_007381781.1| hypothetical protein PUNSTDRAFT_125204 [Punctularia strigosozonata 
HHB-11173 SS5]
 gb|EIN10269.1| hypothetical protein PUNSTDRAFT_125204 [Punctularia strigosozonata 
HHB-11173 SS5]
Length=460

 Score = 39.3 bits (90),  Expect = 8.3, Method: Compositional matrix adjust.
 Identities = 22/59 (37%), Positives = 33/59 (56%), Gaps = 3/59 (5%)

Query  21  IPTTGRHKVVLDPAMIRALVGSIDHGAHSQGDEI---EYDAFKYKFKPSSILTSKPGTI  76
           +P TG+H V +  ++ RAL         ++   I   E+ +FK+ FKP SI  +KPGTI
Sbjct  5   VPATGKHSVNIGSSVRRALKARKGIAPPTKKGNIPDREFYSFKWGFKPESIDPTKPGTI  63


Lambda      K        H        a         alpha
   0.307    0.125    0.357    0.792     4.96 

Gapped
Lambda      K        H        a         alpha    sigma
   0.267   0.0410    0.140     1.90     42.6     43.6 

Effective search space used: 4093817105864


  Database: nr
    Posted date:  Sep 23, 2015 12:05 AM
  Number of letters in database: 26,053,659,533
  Number of sequences in database:  71,551,133


Matrix: BLOSUM62
Gap Penalties: Existence: 11, Extension: 1
Neighboring words threshold: 11
Window for multiple hits: 40
```
