# Supplementary material for: Comparative genomics provides new insights into the diversity, physiology, and sexuality of the only industrially exploited tremellomycete: Phaffia rhodozyma
Source: BMC Genomics. 2016 Nov 9;17:901. doi: 10.1186/s12864-016-3244-7 (PMC5103461; doi:10.1186/s12864-016-3244-7)
Supplement: Additional file 6: — List of orphan genes with links to PFAM (related to Additional file 1: Table S1). (ZIP 1428 kb) [file 12864_2016_3244_MOESM6_ESM.zip › BLAST_HTML_FTR/G04472_P.html]

BLAST Search Results


```
BLASTP 2.2.27+


Reference:
Stephen F. Altschul, Thomas L. Madden, Alejandro A. Schäffer,
Jinghui Zhang, Zheng Zhang, Webb Miller, and David J. Lipman (1997),
"Gapped BLAST and PSI-BLAST: a new generation of protein database
search programs", Nucleic Acids Res. 25:3389-3402.


Reference for
composition-based statistics:
Alejandro A. Schäffer, L. Aravind, Thomas L. Madden, Sergei
Shavirin, John L. Spouge, Yuri I. Wolf, Eugene V. Koonin, and
Stephen F. Altschul (2001), "Improving the accuracy of PSI-BLAST
protein database searches with composition-based statistics and
other refinements", Nucleic Acids Res. 29:2994-3005.


Database: nr
           71,551,133 sequences; 26,053,659,533 total letters


Query= G04472_P

Length=546
                                                                      Score     E
Sequences producing significant alignments:                          (Bits)  Value

emb|CED83717.1|  hypothetical protein [Xanthophyllomyces dendrorh...   920    0.0  
emb|CDZ97563.1|  hypothetical protein [Xanthophyllomyces dendrorh...  48.9    0.014
ref|XP_008541191.1|  PREDICTED: forkhead-associated domain-contai...  43.9    0.41 
ref|XP_005607566.1|  PREDICTED: LOW QUALITY PROTEIN: forkhead-ass...  40.0    7.4  


 >emb|CED83717.1| hypothetical protein [Xanthophyllomyces dendrorhous]
Length=490

 Score =  920 bits (2379),  Expect = 0.0, Method: Compositional matrix adjust.
 Identities = 467/505 (92%), Positives = 469/505 (93%), Gaps = 34/505 (7%)

Query  41   IAEVRQQKADAIKLADTWKERYESSATGGQAAALNRCQRELIEARSEIKLKSALFDEKAR  100
             ++VRQQKADAIKLADTWKERYESSATGGQAAALNRCQRELIEARSEIKLKSALFDEKAR
Sbjct  20   FSQVRQQKADAIKLADTWKERYESSATGGQAAALNRCQRELIEARSEIKLKSALFDEKAR  79

Query  101  IANETATFLANRHAEDVRIIETERLLYQHKQEGFEAMERMWTEKSKEYEDDIANLSRGDK  160
            IANETATFLANRHAEDVRIIETERLLYQHKQEGFEAMERMWTEKSKEYEDDIANLSRGDK
Sbjct  80   IANETATFLANRHAEDVRIIETERLLYQHKQEGFEAMERMWTEKSKEYEDDIANLSRGDK  139

Query  161  CRTSLTSIKEQEYLRSIEQLSQQIAEHNKMIEQAKSVFEVLNAEKLAMASEIQRKDTDLQ  220
            CRTSLTSIKEQEYLRSIEQLSQQIAEHNKMIEQAKSVFEVLNAEKLAMASEIQRKDTDLQ
Sbjct  140  CRTSLTSIKEQEYLRSIEQLSQQIAEHNKMIEQAKSVFEVLNAEKLAMASEIQRKDTDLQ  199

Query  221  NLAKELHQARASEIEAKNYMQDANLSLDGAKRKIASLLKKQGIESDERQTSSNRVHELEL  280
            NLAKELHQARASEIEAKNYMQDANLSLDGAKRKIASLLKKQGIESDERQTSSNRVHELEL
Sbjct  200  NLAKELHQARASEIEAKNYMQDANLSLDGAKRKIASLLKKQGIESDERQTSSNRVHELEL  259

Query  281  QLKRAETEVNDLREQLVKSLLEQRKKEKEWDDQRVLMETQARLHKQKVLAITACLGSDQP  340
            QLKRAETEVNDLREQLVKSLLEQ                                   QP
Sbjct  260  QLKRAETEVNDLREQLVKSLLEQH----------------------------------QP  285

Query  341  DSRRDIVQEEGEEEMNVEAAEEARSIARLPGRNTPAPESLSTHSGPFSPKNILSPSSETR  400
            DSRRDIVQEEGEEEMNVEAAEEARSIARLPGRNTPAPESLSTHSGPFSPKNILSPSSETR
Sbjct  286  DSRRDIVQEEGEEEMNVEAAEEARSIARLPGRNTPAPESLSTHSGPFSPKNILSPSSETR  345

Query  401  FHSNHPLSSPRTRAPSSFHNLFSPPSGPASPISRNNSKTISTQSFRLPSQPPEPSPFLMS  460
            FHSNHPLSSPRTRAPSSFHNLFSPPSGPASPISRNNSKTISTQSFRLPSQPPEPSPFLMS
Sbjct  346  FHSNHPLSSPRTRAPSSFHNLFSPPSGPASPISRNNSKTISTQSFRLPSQPPEPSPFLMS  405

Query  461  SPRASKRSSALEIIDREQVDDSRDIGKGAAFKRMRLESDRDDRSNRRSETIPGSGASSFP  520
            SPRASKRSSALEIIDREQVDDSRDIGKGAAFKRMRLESDRDDRSNRRSETIPGSGASSFP
Sbjct  406  SPRASKRSSALEIIDREQVDDSRDIGKGAAFKRMRLESDRDDRSNRRSETIPGSGASSFP  465

Query  521  MVFSPNSKRKMSSSPDEVERRVKKG  545
            MVFSPNSKRKMSSSPDEVERRVKKG
Sbjct  466  MVFSPNSKRKMSSSPDEVERRVKKG  490


>emb|CDZ97563.1| hypothetical protein [Xanthophyllomyces dendrorhous]
Length=625

 Score = 48.9 bits (115),  Expect = 0.014, Method: Compositional matrix adjust.
 Identities = 46/179 (26%), Positives = 88/179 (49%), Gaps = 10/179 (6%)

Query  1    METDIQSTQSEQAERIRFLETQIKRQLDEKVKIDQKHRAEIAEVRQQKADAIKLADTWKE  60
            +  D+  T + Q  R+  LE QI+  + +K   ++KHR E+A +R Q ADA+     WK+
Sbjct  20   LSVDLPVTPAPQKARLGSLEQQIRDLVSQKTATERKHRGELAGLRTQLADAVGEIAEWKD  79

Query  61   RYESSATGGQAAALNRCQRELIEARSEIKLKSALFDEKARIA--NETATFLANR----HA  114
            + ++S   G A  L   ++E+   ++E  +  AL   ++ +    +   FL+ +     A
Sbjct  80   KCKASE--GGAKQLISYRQEVDRVKAESAISEALLKSRSDLTLHEQYLKFLSFKGQLNKA  137

Query  115  EDVRIIETERLL-YQHKQEGFE-AMERMWTEKSKEYEDDIANLSRGDKCRTSLTSIKEQ  171
            E V I+   +L  ++ ++   E  ++ +    + + ED  A L   D+ R    S++EQ
Sbjct  138  ESVTIMRDHKLTRFKARETSLERELDSLVATHNLQLEDHEALLVLLDETRLEAQSLREQ  196


>ref|XP_008541191.1| PREDICTED: forkhead-associated domain-containing protein 1-like 
isoform X1 [Equus przewalskii]
Length=493

 Score = 43.9 bits (102),  Expect = 0.41, Method: Compositional matrix adjust.
 Identities = 53/173 (31%), Positives = 83/173 (48%), Gaps = 14/173 (8%)

Query  138  ERMWTEKSKEYEDDIANLSRGDKCRTSLTSIKEQEYLRSIEQLSQQIAEHNKMIEQAKSV  197
            ERM ++K +  ++DI +  R  +   S  S  ++ Y R    L Q ++E N  I   KS 
Sbjct  278  ERMMSQKFQVLDEDIDSQQREIQSLKSQISALQKGYSRV---LCQSLSERNSEITTLKSE  334

Query  198  FEVL---NAEKLAMASEIQR----KDTDLQNLAKELHQARASEIEAKNYMQDANLSLDGA  250
             E L   NA    M S +Q+    KD  +Q L +E++Q + SE + KNY  +A   L   
Sbjct  335  GENLRRDNAISSGMVSSLQKDVLAKDEQVQQLKQEVNQLK-SENKEKNYQLEA---LSSR  390

Query  251  KRKIASLLKKQGIESDERQTSSNRVHELELQLKRAETEVNDLREQLVKSLLEQ  303
               +   LKK+  + + R      +   + Q++  E E+  LRE+L KS  EQ
Sbjct  391  CSVLKEELKKEDAQKEHRDAQEKELKLCKTQIQEMEQEMKKLREELKKSFTEQ  443


>ref|XP_005607566.1| PREDICTED: LOW QUALITY PROTEIN: forkhead-associated domain-containing 
protein 1 [Equus caballus]
Length=1454

 Score = 40.0 bits (92),  Expect = 7.4, Method: Compositional matrix adjust.
 Identities = 52/173 (30%), Positives = 83/173 (48%), Gaps = 14/173 (8%)

Query  138  ERMWTEKSKEYEDDIANLSRGDKCRTSLTSIKEQEYLRSIEQLSQQIAEHNKMIEQAKSV  197
            ERM ++K +  ++DI +  R  +   S  S  ++ Y +    L Q ++E N  I   KS 
Sbjct  278  ERMMSQKFQVLDEDIDSQQREIQSLKSQISALQKGYSQV---LCQSLSERNSEITTLKSE  334

Query  198  FEVL---NAEKLAMASEIQR----KDTDLQNLAKELHQARASEIEAKNYMQDANLSLDGA  250
             E L   NA    M S +Q+    KD  +Q L +E++Q + SE + KNY  +A   L   
Sbjct  335  GENLRRDNAISSGMVSSLQKDVLAKDEQVQQLKQEVNQLK-SENKEKNYQLEA---LSSR  390

Query  251  KRKIASLLKKQGIESDERQTSSNRVHELELQLKRAETEVNDLREQLVKSLLEQ  303
               +   LKK+  + + R      +   + Q++  E E+  LRE+L KS  EQ
Sbjct  391  CSVLKEELKKEDAQKEHRDAQEKELKLCKTQIQEMEQEMKKLREELKKSFTEQ  443


Lambda      K        H        a         alpha
   0.311    0.125    0.335    0.792     4.96 

Gapped
Lambda      K        H        a         alpha    sigma
   0.267   0.0410    0.140     1.90     42.6     43.6 

Effective search space used: 5807756286150


  Database: nr
    Posted date:  Sep 23, 2015 12:05 AM
  Number of letters in database: 26,053,659,533
  Number of sequences in database:  71,551,133


Matrix: BLOSUM62
Gap Penalties: Existence: 11, Extension: 1
Neighboring words threshold: 11
Window for multiple hits: 40
```
