# Supplementary material for: Comparative genomics provides new insights into the diversity, physiology, and sexuality of the only industrially exploited tremellomycete: Phaffia rhodozyma
Source: BMC Genomics. 2016 Nov 9;17:901. doi: 10.1186/s12864-016-3244-7 (PMC5103461; doi:10.1186/s12864-016-3244-7)
Supplement: Additional file 6: — List of orphan genes with links to PFAM (related to Additional file 1: Table S1). (ZIP 1428 kb) [file 12864_2016_3244_MOESM6_ESM.zip › BLAST_HTML_FTR/G00647_P.html]

BLAST Search Results


```
BLASTP 2.2.27+


Reference:
Stephen F. Altschul, Thomas L. Madden, Alejandro A. Schäffer,
Jinghui Zhang, Zheng Zhang, Webb Miller, and David J. Lipman (1997),
"Gapped BLAST and PSI-BLAST: a new generation of protein database
search programs", Nucleic Acids Res. 25:3389-3402.


Reference for
composition-based statistics:
Alejandro A. Schäffer, L. Aravind, Thomas L. Madden, Sergei
Shavirin, John L. Spouge, Yuri I. Wolf, Eugene V. Koonin, and
Stephen F. Altschul (2001), "Improving the accuracy of PSI-BLAST
protein database searches with composition-based statistics and
other refinements", Nucleic Acids Res. 29:2994-3005.


Database: nr
           71,551,133 sequences; 26,053,659,533 total letters


Query= G00647_P

Length=961
                                                                      Score     E
Sequences producing significant alignments:                          (Bits)  Value

emb|CED82526.1|  hypothetical protein [Xanthophyllomyces dendrorh...  1980    0.0  
emb|CED82534.1|  hypothetical protein [Xanthophyllomyces dendrorh...   256    2e-68
emb|CED82543.1|  hypothetical protein [Xanthophyllomyces dendrorh...  95.9    2e-17
ref|WP_053585048.1|  hypothetical protein [Lysinibacillus contami...  43.9    0.95 
ref|WP_025153878.1|  phosphoesterase [Morganella morganii]            43.5    0.98 
gb|KJF78490.1|  phosphoesterase [Morganella morganii]                 43.5    0.99 
ref|WP_024475057.1|  phosphoesterase [Morganella morganii] >gb|KG...  42.0    2.8  
ref|WP_014673981.1|  GntR family transcriptional regulator [Strep...  39.7    9.0  


 >emb|CED82526.1| hypothetical protein [Xanthophyllomyces dendrorhous]
Length=980

 Score = 1980 bits (5129),  Expect = 0.0, Method: Compositional matrix adjust.
 Identities = 960/980 (98%), Positives = 960/980 (98%), Gaps = 20/980 (2%)

Query  1    MSMIFRTVKSIRSPSILPAKSPFSLVNAAGPHSIRSSRYIRRIHRFEQTSGSVSTTGHED  60
            MSMIFRTVKSIRSPSILPAKSPFSLVNAAGPHSIRSSRYIRRIHRFEQTSGSVSTTGHED
Sbjct  1    MSMIFRTVKSIRSPSILPAKSPFSLVNAAGPHSIRSSRYIRRIHRFEQTSGSVSTTGHED  60

Query  61   HNTQGVDTKGGQDRPQVMAGQAELGLQEAAGSKTIKNEFQKAKDKDLCTQRAEGEGSSEW  120
            HNTQGVDTKGGQDRPQVMAGQAELGLQEAAGSKTIKNEFQKAKDKDLCTQRAEGEGSSEW
Sbjct  61   HNTQGVDTKGGQDRPQVMAGQAELGLQEAAGSKTIKNEFQKAKDKDLCTQRAEGEGSSEW  120

Query  121  ENEAEEDDEIEYNDQVITYIHRLPLELDSITFRDFFWSRGVHVVEAWKATGKRSGIIQTS  180
            ENEAEEDDEIEYNDQVITYIHRLPLELDSITFRDFFWSRGVHVVEAWKATGKRSGIIQTS
Sbjct  121  ENEAEEDDEIEYNDQVITYIHRLPLELDSITFRDFFWSRGVHVVEAWKATGKRSGIIQTS  180

Query  181  ISDQDLVCDTMDGTQAPWGLLIAKPGDTASTMIKLSEADEHAFQKASSQNPDSSYTIDKN  240
            ISDQDLVCDTMDGTQAPWGLLIAKPGDTASTMIKLSEADEHAFQKASSQNPDSSYTIDKN
Sbjct  181  ISDQDLVCDTMDGTQAPWGLLIAKPGDTASTMIKLSEADEHAFQKASSQNPDSSYTIDKN  240

Query  241  VTSNPKAESLDDMTDVASEASVKISSDNQSDELRQELYLQRIPNMKTLQIDIDPKAEVTL  300
            VTSNPKAESLDDMTDVASEASVKISSDNQSDELRQELYLQRIPNMKTLQIDIDPKAEVTL
Sbjct  241  VTSNPKAESLDDMTDVASEASVKISSDNQSDELRQELYLQRIPNMKTLQIDIDPKAEVTL  300

Query  301  NETAVIEALTSRGYKVPAYHIKDNRILFDVGDLSAEGFREILAKSHPTIKANVLEVVAFS  360
            NETAVIEALTSRGYKVPAYHIKDNRILFDVGDLSAEGFREILAKSHPTIKANVLEVVAFS
Sbjct  301  NETAVIEALTSRGYKVPAYHIKDNRILFDVGDLSAEGFREILAKSHPTIKANVLEVVAFS  360

Query  361  WRSSFFSESALYEVNSRTSLLITSLSPTTSGKLVLRYLLELAPQLKLSSIRWIEKRVRET  420
            WRSSFFSESALYEVNSRTSLLITSLSPTTSGKLVLRYLLELAPQLKLSSIRWIEKRVRET
Sbjct  361  WRSSFFSESALYEVNSRTSLLITSLSPTTSGKLVLRYLLELAPQLKLSSIRWIEKRVRET  420

Query  421  WAIVTVSGQQAFESVLNVHQQPIGGVPVDVRAMPIETKGSIKGPTKSAWDIFRIHNFPFN  480
            WAIVTVSGQQAFESVLNVHQQPIGGVPVDVRAMPIETKGSIKGPTKSAWDIFRIHNFPFN
Sbjct  421  WAIVTVSGQQAFESVLNVHQQPIGGVPVDVRAMPIETKGSIKGPTKSAWDIFRIHNFPFN  480

Query  481  LTDDQVRNLFSGGEKSPAVEFIGRINPRAATFKLLNPSVEARRDLMSMQMSVIHGRALQL  540
            LTDDQVRNLFSGGEKSPAVEFIGRINPRAATFKLLNPSVEARRDLMSMQMSVIHGRALQL
Sbjct  481  LTDDQVRNLFSGGEKSPAVEFIGRINPRAATFKLLNPSVEARRDLMSMQMSVIHGRALQL  540

Query  541  EIVTPQDLEKPKHYTNSIAGSQISRSSKRTDGPDAGPRLLQRFRKNNYKDAAPVMGNDEL  600
            EIVTPQDLEKPKHYTNSIAGSQISRSSKRTDGPDAGPRLLQRFRKNNYKDAAPVMGNDEL
Sbjct  541  EIVTPQDLEKPKHYTNSIAGSQISRSSKRTDGPDAGPRLLQRFRKNNYKDAAPVMGNDEL  600

Query  601  IRT--------------------LGQELKGLQFAFVRAKREEGLQEGPNPEIVPNFLKRI  640
            IRT                    LGQELKGLQFAFVRAKREEGLQEGPNPEIVPNFLKRI
Sbjct  601  IRTMRVFSLSCYIILRNAMEIIRLGQELKGLQFAFVRAKREEGLQEGPNPEIVPNFLKRI  660

Query  641  CASPTNLEPWRSLRYEVFTAQKMNHQQQLRSQQIAENEVRQKIEQGLREIMYHPTHGIHA  700
            CASPTNLEPWRSLRYEVFTAQKMNHQQQLRSQQIAENEVRQKIEQGLREIMYHPTHGIHA
Sbjct  661  CASPTNLEPWRSLRYEVFTAQKMNHQQQLRSQQIAENEVRQKIEQGLREIMYHPTHGIHA  720

Query  701  LRPFDFKSLPQPPYRFPLSKMFQRIVLQDPSVLVSMNDLTPNPLSQVDQKSELQQAPHDV  760
            LRPFDFKSLPQPPYRFPLSKMFQRIVLQDPSVLVSMNDLTPNPLSQVDQKSELQQAPHDV
Sbjct  721  LRPFDFKSLPQPPYRFPLSKMFQRIVLQDPSVLVSMNDLTPNPLSQVDQKSELQQAPHDV  780

Query  761  HRVRTSDPLSNTERMLLRGRMRAFSPFFFDIFESYRKRSELSIFLQRLQQAYSQLEDKKG  820
            HRVRTSDPLSNTERMLLRGRMRAFSPFFFDIFESYRKRSELSIFLQRLQQAYSQLEDKKG
Sbjct  781  HRVRTSDPLSNTERMLLRGRMRAFSPFFFDIFESYRKRSELSIFLQRLQQAYSQLEDKKG  840

Query  821  GDSEIIPKFHYRLWSILGDLGSLVSLRTEVCQHIEKQKLDIASVDILRIAQIQAAKSYYE  880
            GDSEIIPKFHYRLWSILGDLGSLVSLRTEVCQHIEKQKLDIASVDILRIAQIQAAKSYYE
Sbjct  841  GDSEIIPKFHYRLWSILGDLGSLVSLRTEVCQHIEKQKLDIASVDILRIAQIQAAKSYYE  900

Query  881  GLKEVIYHPTHGLDAKFSLNYRHPVEPPYQNELACRFQRDMTLAKICEKSTAVLPRRMTV  940
            GLKEVIYHPTHGLDAKFSLNYRHPVEPPYQNELACRFQRDMTLAKICEKSTAVLPRRMTV
Sbjct  901  GLKEVIYHPTHGLDAKFSLNYRHPVEPPYQNELACRFQRDMTLAKICEKSTAVLPRRMTV  960

Query  941  VKYGLKKPSQTERPSAVISP  960
            VKYGLKKPSQTERPSAVISP
Sbjct  961  VKYGLKKPSQTERPSAVISP  980


>emb|CED82534.1| hypothetical protein [Xanthophyllomyces dendrorhous]
Length=916

 Score =  256 bits (655),  Expect = 2e-68, Method: Compositional matrix adjust.
 Identities = 218/803 (27%), Positives = 363/803 (45%), Gaps = 75/803 (9%)

Query  135  QVITYIHRLPLELDSITFRDFFWSRGVHVVEAWKATGKRSGIIQTSISDQDLVCDTMDGT  194
            QVIT I  LP +++S + R+FFWS  ++V++A K+TG+ SG+IQTS  DQ  +  ++   
Sbjct  77   QVITSIFPLPPDMNSYSLRNFFWSNSINVLDARKSTGEVSGLIQTSQEDQQKLLSSIHDI  136

Query  195  QAPWGLLIAKPGDTASTMIKLSEADEHAFQKASSQNPDSSYTIDKNVTSNPKAESLDDMT  254
            QA WG L A P   A T I  S   E  F +A S +             NP+        
Sbjct  137  QAHWGNLTAVPASPAMTAIVPSPKQERKFLEALSTSKRGQ---------NPRV-------  180

Query  255  DVASEASVKISSDNQSDELRQE----LYLQRIPNMKTLQIDIDPKAEVTLNETAVIEALT  310
             V+S A V +++  +S ++ +     L+L  IP + T+QI    K         + E L 
Sbjct  181  -VSSSAQVGLANKYESRDISRSDSRILFLSSIPELVTMQISFSKKFPRKHIANDLRELLL  239

Query  311  SRGYKVPAYHIKDNRILFDVGDLSAEGFREILAKSHPTIKANVLEVVAFSWRSSFFSESA  370
              G+ VP+  + + +IL D+G +S    ++++ +  P +++++L++    W SS   ++A
Sbjct  240  REGWAVPSIAMLNQQILVDIGGISVYELKKMVMRVQPELQSDMLDIAPLYWMSSSSDKNA  299

Query  371  LYEVNSRTSLLITSLSPTTSGKLVLRYLLELAPQLKLSSIRWIEKRVRETWAIVTVSGQQ  430
                 S  +LLI+SLSP  S + ++ Y+  L P +  +SI  I K++ E  A++ V  ++
Sbjct  300  NRNCLSGRTLLISSLSPAMSCQDIMLYIRTLIPDIHPNSINRIRKKIGECTAVIPVQDRE  359

Query  431  AFESVLNVHQQPIGGVPVDVRAMPIETKGS-IKGPTKSAWDIFRIHNFPFNLTDDQVRNL  489
              +S+L +H +  GG PV++R +P +T  S I G     W+ FR+HN P N+  DQ+ + 
Sbjct  360  TLQSILGLHGRCFGGRPVNIRVIPAKTDSSDIIGLEHEKWNTFRVHNLPSNIQKDQIDSC  419

Query  490  FSGGEKSPAVEFIGRINPRAATFKLLNPSVEARRDLMSMQMSVIHGRALQLEIVTPQDLE  549
                   P ++ +   + R A F L NPS E+R  +  +  + IHG+ + +EI+ PQDL 
Sbjct  420  LDSLGGMPPMKLLRWESNRTAIFSLSNPSEESRESIKRVHGNSIHGKTITIEIL-PQDLR  478

Query  550  KPKHYTNSIAGSQISRSSKRTDGPDAGPRLLQRFRKNNYKDAAPVMGNDELIRTLGQELK  609
                   ++    I +S    DG          +R+N  KD   ++  DEL       LK
Sbjct  479  SSTKGALAVEDVSIQQSPTH-DGD---------YRENVPKDTGRLV--DEL-----SYLK  521

Query  610  GLQFAFVRAKREEGLQEGPNPEIVPNFLKRICASPTNLEPWRSLRYEVFTAQKMNHQQQL  669
             L  ++                  P +L+    S    +  RS    +      N +Q  
Sbjct  522  SLFASY-----------------APQYLQLYVMSQAKFQ--RSFLKGISQGTITNTKQMY  562

Query  670  RS-QQIAENEVRQKIEQGL---REIMYHPTHGIHALRPFDFKSLPQPPYRFPLSKMFQRI  725
            +S +Q  +   R  IE  L   REI+YHP  G+     FDF     PPY    +KMF + 
Sbjct  563  KSDEQDHQTTKRLLIESDLLSVREILYHPNCGLFTPTSFDFDRRYSPPYLTECAKMFYKE  622

Query  726  VLQDPS-----VLVSMNDLTPNPLSQVDQKSELQQAPHDVHRVRTSDPLSNTERMLLRGR  780
            V   P       L S ++L+   +   +   +  +   D+ +   SD  S      L  +
Sbjct  623  VRGYPDGSLMQGLRSKSELSARSMDVSEWTVKFGRIVQDLVQSGVSDSYSVPVVKDLEKQ  682

Query  781  MRAFSPFFFDIFESYRKRSELSIFLQRLQQAYSQLEDKKGGD-----SEIIPKFHYRLWS  835
            +  +S       +  R    +  +++  +  Y Q     G       ++++  F  R  +
Sbjct  683  IANYSTSIRTHIDDARDLLMVVSYIKYCRAVYYQSHQVDGQKPMNVFNDLVQGFDRRFEA  742

Query  836  ILGDLGSLVSLRTEVCQHIEKQKLDIASVDILR-IAQIQAAKSYYEGLKEVIYHPTHGLD  894
               +L  L  LR  + + + +  L    VD L  +   +A   Y EGL E++ HPTHG +
Sbjct  743  HKKNLKGLQKLRNNIDRGLIRHGLQ-PCVDFLHMLVSTRARAEYVEGLLEIVRHPTHGFN  801

Query  895  AKFSLNYRHPVEPPYQNELACRF  917
              F  +      PPY   LA  F
Sbjct  802  GAFPFDGEVIPNPPYATTLARTF  824


 Score = 47.8 bits (112),  Expect = 0.062, Method: Compositional matrix adjust.
 Identities = 28/98 (29%), Positives = 46/98 (47%), Gaps = 2/98 (2%)

Query  631  EIVPNFLKRICASPTNLEPWRSLRYEVFTAQKMNHQQQLRS--QQIAENEVRQKIEQGLR  688
            ++V  F +R  A   NL+  + LR  +      +  Q        +     R +  +GL 
Sbjct  731  DLVQGFDRRFEAHKKNLKGLQKLRNNIDRGLIRHGLQPCVDFLHMLVSTRARAEYVEGLL  790

Query  689  EIMYHPTHGIHALRPFDFKSLPQPPYRFPLSKMFQRIV  726
            EI+ HPTHG +   PFD + +P PPY   L++ F  ++
Sbjct  791  EIVRHPTHGFNGAFPFDGEVIPNPPYATTLARTFGSLI  828


>emb|CED82543.1| hypothetical protein [Xanthophyllomyces dendrorhous]
Length=419

 Score = 95.9 bits (237),  Expect = 2e-17, Method: Compositional matrix adjust.
 Identities = 49/110 (45%), Positives = 65/110 (59%), Gaps = 5/110 (5%)

Query  135  QVITYIHRLPLELDSITFRDFFWSRGVHVVEAWKATGKRSGIIQTSISDQDLVCDTMDGT  194
            QV+T IH LP ELDS T R FFW+ G+ V+EAWK  GKRSG+IQTS  DQ      ++  
Sbjct  63   QVVTCIHLLPPELDSNTLRKFFWTSGIRVLEAWKPIGKRSGMIQTSFEDQFKALRRINRE  122

Query  195  QAPWGLLIAKPGDTASTMIKLSEADEHAFQKASSQNPDSSYTIDKNVTSN  244
              PWG+L  +    A+  I LS  +E +F+ AS +     ++ID N   N
Sbjct  123  PRPWGILQTQSAGLATVAIVLSPLEESSFRLASGK-----FSIDPNRKDN  167


>ref|WP_053585048.1| hypothetical protein [Lysinibacillus contaminans]
 gb|KOS66352.1| membrane protein [Lysinibacillus contaminans]
Length=728

 Score = 43.9 bits (102),  Expect = 0.95, Method: Compositional matrix adjust.
 Identities = 30/95 (32%), Positives = 45/95 (47%), Gaps = 3/95 (3%)

Query  183  DQDLVCDTMDGTQAPWGLLIAKPGDTASTMIKLSEADEHAFQKASSQNPDSSYT---IDK  239
            +  L  D     Q  +G L AKP +  S +    E    AFQ ASS+N  +  T    +K
Sbjct  83   ENGLTTDDFLAIQKVYGELKAKPLNNQSLIPPYDEMPPQAFQAASSENGSTLVTPVFFEK  142

Query  240  NVTSNPKAESLDDMTDVASEASVKISSDNQSDELR  274
            +  ++   ESLDD+T +    +V++  D  SD L 
Sbjct  143  DAATDALQESLDDLTKIMGTHNVQLDKDLTSDALH  177


>ref|WP_025153878.1| phosphoesterase [Morganella morganii]
Length=354

 Score = 43.5 bits (101),  Expect = 0.98, Method: Compositional matrix adjust.
 Identities = 24/81 (30%), Positives = 42/81 (52%), Gaps = 0/81 (0%)

Query  99   FQKAKDKDLCTQRAEGEGSSEWENEAEEDDEIEYNDQVITYIHRLPLELDSITFRDFFWS  158
            F+K ++  +C  RA+   ++ +  +AE+  E       I   +R  +E+D IT+ D   S
Sbjct  131  FEKGQEFYMCASRADNLNAAYFSPQAEDWLEYIKASSAIPGFYREGVEIDGITYHDGGIS  190

Query  159  RGVHVVEAWKATGKRSGIIQT  179
              V V+EAW+    R  +I+T
Sbjct  191  DAVPVIEAWRRGATRIVVIRT  211


>gb|KJF78490.1| phosphoesterase [Morganella morganii]
Length=354

 Score = 43.5 bits (101),  Expect = 0.99, Method: Compositional matrix adjust.
 Identities = 24/81 (30%), Positives = 42/81 (52%), Gaps = 0/81 (0%)

Query  99   FQKAKDKDLCTQRAEGEGSSEWENEAEEDDEIEYNDQVITYIHRLPLELDSITFRDFFWS  158
            F+K ++  +C  RA+   ++ +  +AE+  E       I   +R  +E+D IT+ D   S
Sbjct  131  FEKGQEFYMCASRADNLNAAYFSPQAEDWLEYIKASSAIPGFYREGVEIDGITYHDGGIS  190

Query  159  RGVHVVEAWKATGKRSGIIQT  179
              V V+EAW+    R  +I+T
Sbjct  191  DAVPVIEAWRRGATRIVVIRT  211


>ref|WP_024475057.1| phosphoesterase [Morganella morganii]
 gb|KGP43152.1| phosphoesterase [Morganella morganii]
Length=354

 Score = 42.0 bits (97),  Expect = 2.8, Method: Compositional matrix adjust.
 Identities = 23/81 (28%), Positives = 42/81 (52%), Gaps = 0/81 (0%)

Query  99   FQKAKDKDLCTQRAEGEGSSEWENEAEEDDEIEYNDQVITYIHRLPLELDSITFRDFFWS  158
            F++ ++  +C  RA+   ++ +  +AE+  E       I   +R  +E+D IT+ D   S
Sbjct  131  FEQGQEFYMCASRADNLSAAYFSPQAEDWLEYIKASSAIPGFYREGVEIDGITYHDGGIS  190

Query  159  RGVHVVEAWKATGKRSGIIQT  179
              V V+EAW+    R  +I+T
Sbjct  191  DAVPVIEAWRRGATRIVVIRT  211


>ref|WP_014673981.1| GntR family transcriptional regulator [Streptomyces hygroscopicus]
 gb|AEY90668.1| GntR-family transcriptional regulator [Streptomyces hygroscopicus 
subsp. jinggangensis 5008]
 gb|AGF64826.1| GntR-family transcriptional regulator [Streptomyces hygroscopicus 
subsp. jinggangensis TL01]
Length=231

 Score = 39.7 bits (91),  Expect = 9.0, Method: Composition-based stats.
 Identities = 17/56 (30%), Positives = 31/56 (55%), Gaps = 0/56 (0%)

Query  295  KAEVTLNETAVIEALTSRGYKVPAYHIKDNRILFDVGDLSAEGFREILAKSHPTIK  350
            +A V L+   ++EA   RG++VP Y + D R + +   L  +G  ++L+  HP  +
Sbjct  53   EALVDLSAQGILEADQHRGFRVPEYSVTDYRDMIEARSLVTDGMFQVLSAGHPAFR  108


Lambda      K        H        a         alpha
   0.317    0.132    0.382    0.792     4.96 

Gapped
Lambda      K        H        a         alpha    sigma
   0.267   0.0410    0.140     1.90     42.6     43.6 

Effective search space used: 11627141696000


  Database: nr
    Posted date:  Sep 23, 2015 12:05 AM
  Number of letters in database: 26,053,659,533
  Number of sequences in database:  71,551,133


Matrix: BLOSUM62
Gap Penalties: Existence: 11, Extension: 1
Neighboring words threshold: 11
Window for multiple hits: 40
```
