# Supplementary material for: Comparative genomics provides new insights into the diversity, physiology, and sexuality of the only industrially exploited tremellomycete: Phaffia rhodozyma
Source: BMC Genomics. 2016 Nov 9;17:901. doi: 10.1186/s12864-016-3244-7 (PMC5103461; doi:10.1186/s12864-016-3244-7)
Supplement: Additional file 6: — List of orphan genes with links to PFAM (related to Additional file 1: Table S1). (ZIP 1428 kb) [file 12864_2016_3244_MOESM6_ESM.zip › BLAST_HTML_FTR/G03952_P.html]

BLAST Search Results


```
BLASTP 2.2.27+


Reference:
Stephen F. Altschul, Thomas L. Madden, Alejandro A. Schäffer,
Jinghui Zhang, Zheng Zhang, Webb Miller, and David J. Lipman (1997),
"Gapped BLAST and PSI-BLAST: a new generation of protein database
search programs", Nucleic Acids Res. 25:3389-3402.


Reference for
composition-based statistics:
Alejandro A. Schäffer, L. Aravind, Thomas L. Madden, Sergei
Shavirin, John L. Spouge, Yuri I. Wolf, Eugene V. Koonin, and
Stephen F. Altschul (2001), "Improving the accuracy of PSI-BLAST
protein database searches with composition-based statistics and
other refinements", Nucleic Acids Res. 29:2994-3005.


Database: nr
           71,551,133 sequences; 26,053,659,533 total letters


Query= G03952_P

Length=1204
                                                                      Score     E
Sequences producing significant alignments:                          (Bits)  Value

emb|CDZ98671.1|  hypothetical protein [Xanthophyllomyces dendrorh...  1952    0.0  
ref|XP_645312.1|  hypothetical protein DDB_G0272472 [Dictyosteliu...  41.6    6.8  


 >emb|CDZ98671.1| hypothetical protein [Xanthophyllomyces dendrorhous]
Length=1181

 Score = 1952 bits (5056),  Expect = 0.0, Method: Compositional matrix adjust.
 Identities = 1180/1203 (98%), Positives = 1180/1203 (98%), Gaps = 22/1203 (2%)

Query  1     MSITTLSNSATMSTPQGLVSNVSSAISALPNILVPSSSPHDSVHPHPHDSLREDLPRSPS  60
             MSITTLSNSATMSTPQGLVSNVSSAISALPNILVPSSSPHDSVHPHPHDSLREDLPRSPS
Sbjct  1     MSITTLSNSATMSTPQGLVSNVSSAISALPNILVPSSSPHDSVHPHPHDSLREDLPRSPS  60

Query  61    PLSSPSRKVRQADIVQVPIRIHPKPFAQNGQQGIRLMFKPERVQTDTGKIHQVSHSDTET  120
             PLSSPSRKVRQADIVQVPIRIHPKPFAQNGQQGIRLMFKPERVQTDTGKIHQVSHSDTET
Sbjct  61    PLSSPSRKVRQADIVQVPIRIHPKPFAQNGQQGIRLMFKPERVQTDTGKIHQVSHSDTET  120

Query  121   ASRLDVSDGTDQDSEEEDEDDDEDEDDEDYTEHEYQPTRPMSVSTRKSLAPPGTEPAAAP  180
             ASRLDVSDGTDQDSEEEDEDDDEDEDDEDYTEHEYQPTRPMSVSTRKSLAPPGTEPAAAP
Sbjct  121   ASRLDVSDGTDQDSEEEDEDDDEDEDDEDYTEHEYQPTRPMSVSTRKSLAPPGTEPAAAP  180

Query  181   GETIPAFAPVPTTRKNALPLPRGRANSPRGRLPSPTPSIPSTRQRSRSRSHTLTSPLPVS  240
             GETIPAFAPVPTTRKNALPLPRGRANSPRGRLPSPTPSIPSTRQRSRSRSHTLTSPLPVS
Sbjct  181   GETIPAFAPVPTTRKNALPLPRGRANSPRGRLPSPTPSIPSTRQRSRSRSHTLTSPLPVS  240

Query  241   CSASVDHELSPAIESNSASVLTSAAVASSPSSSSSPSSSSSSTSKRSVPLSEFNIDSFSG  300
             CSASVDHELSPAIESNSASVLT                      KRSVPLSEFNIDSFSG
Sbjct  241   CSASVDHELSPAIESNSASVLT----------------------KRSVPLSEFNIDSFSG  278

Query  301   SKRTRRISHSPIQPTSAQGPNRRSRSSSPGASVDSSSTSSNNTGPRSTSPVKVDRRRKGN  360
             SKRTRRISHSPIQPTSAQGPNRRSRSSSPGASVDSSSTSSNNTGPRSTSPVKVDRRRKGN
Sbjct  279   SKRTRRISHSPIQPTSAQGPNRRSRSSSPGASVDSSSTSSNNTGPRSTSPVKVDRRRKGN  338

Query  361   GGGRWPSGAPRSSTVSLNNSGPSLSDGQSEVNQPNGVLDVKMEEQSTELLLNVGKPKTLV  420
             GGGRWPSGAPRSSTVSLNNSGPSLSDGQSEVNQPNGVLDVKMEEQSTELLLNVGKPKTLV
Sbjct  339   GGGRWPSGAPRSSTVSLNNSGPSLSDGQSEVNQPNGVLDVKMEEQSTELLLNVGKPKTLV  398

Query  421   DGAQNDEGKEYVSELRTNAERASIISPIKATDVPSSAPPPPSSESPSSLSKIDSVESSFN  480
             DGAQNDEGKEYVSELRTNAERASIISPIKATDVPSSAPPPPSSESPSSLSKIDSVESSFN
Sbjct  399   DGAQNDEGKEYVSELRTNAERASIISPIKATDVPSSAPPPPSSESPSSLSKIDSVESSFN  458

Query  481   SLKRIVPKIVLVQPNSFSDGSHTSTPALLTNGSTKPAPTSVVPSKPKASRETTPAGQPSK  540
              LKRIVPKIVLVQPNSFSDGSHTSTPALLTNGSTKPAPTSVVPSKPKASRETTPAGQPSK
Sbjct  459   PLKRIVPKIVLVQPNSFSDGSHTSTPALLTNGSTKPAPTSVVPSKPKASRETTPAGQPSK  518

Query  541   KALHAAEMKLLRARKRQERLELVEIELKMLDELYESVANNTDQSLVECWADLQRRYTGAS  600
             KALHAAEMKLLRARKRQERLELVEIELKMLDELYESVANNTDQSLVECWADLQRRYTGAS
Sbjct  519   KALHAAEMKLLRARKRQERLELVEIELKMLDELYESVANNTDQSLVECWADLQRRYTGAS  578

Query  601   TLIENRKRQAEAELMRVYDADISFTWSWWNISRFDTLNKEKAELSMKLQKLGREHRQIQT  660
             TLIENRKRQAEAELMRVYDADISFTWSWWNISRFDTLNKEKAELSMKLQKLGREHRQIQT
Sbjct  579   TLIENRKRQAEAELMRVYDADISFTWSWWNISRFDTLNKEKAELSMKLQKLGREHRQIQT  638

Query  661   AALPPSERFLPVGSRAPLVPNPRYVVHTPEYRLSNDNLEKQEKKRKKRRLVEPESSDDES  720
             AALPPSERFLPVGSRAPLVPNPRYVVHTPEYRLSNDNLEKQEKKRKKRRLVEPESSDDES
Sbjct  639   AALPPSERFLPVGSRAPLVPNPRYVVHTPEYRLSNDNLEKQEKKRKKRRLVEPESSDDES  698

Query  721   IGVKGLRPDDIESDLNKLGLGRAEKEKERLRLIGARQEEERMKREEEERIRAQLEWEAKE  780
             IGVKGLRPDDIESDLNKLGLGRAEKEKERLRLIGARQEEERMKREEEERIRAQLEWEAKE
Sbjct  699   IGVKGLRPDDIESDLNKLGLGRAEKEKERLRLIGARQEEERMKREEEERIRAQLEWEAKE  758

Query  781   AKEAERKLEEQEWERQRAMSVLEAQRLEAEKREQDAVDYRSNRDSGSYPLPMAKPSRTRD  840
             AKEAERKLEEQEWERQRAMSVLEAQRLEAEKREQDAVDYRSNRDSGSYPLPMAKPSRTRD
Sbjct  759   AKEAERKLEEQEWERQRAMSVLEAQRLEAEKREQDAVDYRSNRDSGSYPLPMAKPSRTRD  818

Query  841   PSGKPKSKAGKSTSTSKPPSRSAGMPAPPPPSAPTLPSSSSSSSDIRPIRPVKMEPSSGP  900
             PSGKPKSKAGKSTSTSKPPSRSAGMPAPPPPSAPTLPSSSSSSSDIRPIRPVKMEPSSGP
Sbjct  819   PSGKPKSKAGKSTSTSKPPSRSAGMPAPPPPSAPTLPSSSSSSSDIRPIRPVKMEPSSGP  878

Query  901   IFPSNAPLSPVIPPSAHPWNDPHSREPASYYLGGGSSHLPYPSPHYGPSSYPNMSLPGGY  960
             IFPSNAPLSPVIPPSAHPWNDPHSREPASYYLGGGSSHLPYPSPHYGPSSYPNMSLPGGY
Sbjct  879   IFPSNAPLSPVIPPSAHPWNDPHSREPASYYLGGGSSHLPYPSPHYGPSSYPNMSLPGGY  938

Query  961   PQHFFPSPLNWIPGQSANQESTHQSVGPQGGSSGSSGSSRNVTLSTQDHPSVHPFHLNPY  1020
             PQHFFPSPLNWIPGQSANQESTHQSVGPQGGSSGSSGSSRNVTLSTQDHPSVHPFHLNPY
Sbjct  939   PQHFFPSPLNWIPGQSANQESTHQSVGPQGGSSGSSGSSRNVTLSTQDHPSVHPFHLNPY  998

Query  1021  VSFSSMGDNRSLLSHVPQQQQQQQRPNQHPYQHQPYQSQHQQQQPYSISPSHHEHILSTQ  1080
             VSFSSMGDNRSLLSHVPQQQQQQQRPNQHPYQHQPYQSQHQQQQPYSISPSHHEHILSTQ
Sbjct  999   VSFSSMGDNRSLLSHVPQQQQQQQRPNQHPYQHQPYQSQHQQQQPYSISPSHHEHILSTQ  1058

Query  1081  SHPHHHHVAHRHHQQPSHTPSGSQSGLLVDPPHRPLSTSVSSSTTWRPAGGSKPIEPVRS  1140
             SHPHHHHVAHRHHQQPSHTPSGSQSGLLVDPPHRPLSTSVSSSTTWRPAGGSKPIEPVRS
Sbjct  1059  SHPHHHHVAHRHHQQPSHTPSGSQSGLLVDPPHRPLSTSVSSSTTWRPAGGSKPIEPVRS  1118

Query  1141  NYLPHSFLNPLPSVPPNQAHHPHASQQQQLPPLPFGSSNHQLHPLTGPGHPLWGTNYGVG  1200
             NYLPHSFLNPLPSVPPNQAHHPHASQQQQLPPLPFGSSNHQLHPLTGPGHPLWGTNYGVG
Sbjct  1119  NYLPHSFLNPLPSVPPNQAHHPHASQQQQLPPLPFGSSNHQLHPLTGPGHPLWGTNYGVG  1178

Query  1201  NSR  1203
             NSR
Sbjct  1179  NSR  1181


>ref|XP_645312.1| hypothetical protein DDB_G0272472 [Dictyostelium discoideum AX4]
 sp|Q75JP5|Y2471_DICDI RecName: Full=Calponin homology domain-containing protein DDB_G0272472
 gb|EAL71388.1| hypothetical protein DDB_G0272472 [Dictyostelium discoideum AX4]
Length=1508

 Score = 41.6 bits (96),  Expect = 6.8, Method: Compositional matrix adjust.
 Identities = 51/134 (38%), Positives = 71/134 (53%), Gaps = 29/134 (22%)

Query  701  QEKK--RKKRRLVEPESSDDESIGVK----GLRPDDIESDLNKLGLGRAEKEKERLRLIG  754
            QEKK   +K+R+ E +   DE +  K     L  + IE +L  L L    KE E  RL+ 
Sbjct  588  QEKKLIEEKKRIAEEKRISDEILAKKQLAEKLEKERIEKELEDLRLA---KELEEKRLLA  644

Query  755  ARQEEE---RMKREEEERIRAQLEWEAKE---AKEAERKLEEQE----WERQRAMSVLEA  804
             RQE+E   ++KRE       +LE EA++   A+E ERK  E+E     E+QR    LE 
Sbjct  645  LRQEKELAEKLKRE-------RLEKEAEDKRIAQEIERKRLEKEKQDQLEKQRK---LEQ  694

Query  805  QRLEAEKREQDAVD  818
            QRL+ EK E++  D
Sbjct  695  QRLQKEKDEKELAD  708


Lambda      K        H        a         alpha
   0.307    0.125    0.361    0.792     4.96 

Gapped
Lambda      K        H        a         alpha    sigma
   0.267   0.0410    0.140     1.90     42.6     43.6 

Effective search space used: 14980848673014


  Database: nr
    Posted date:  Sep 23, 2015 12:05 AM
  Number of letters in database: 26,053,659,533
  Number of sequences in database:  71,551,133


Matrix: BLOSUM62
Gap Penalties: Existence: 11, Extension: 1
Neighboring words threshold: 11
Window for multiple hits: 40
```
