# Supplementary material for: Comparative genomics provides new insights into the diversity, physiology, and sexuality of the only industrially exploited tremellomycete: Phaffia rhodozyma
Source: BMC Genomics. 2016 Nov 9;17:901. doi: 10.1186/s12864-016-3244-7 (PMC5103461; doi:10.1186/s12864-016-3244-7)
Supplement: Additional file 6: — List of orphan genes with links to PFAM (related to Additional file 1: Table S1). (ZIP 1428 kb) [file 12864_2016_3244_MOESM6_ESM.zip › BLAST_HTML_FTR/G01418_P.html]

BLAST Search Results


```
BLASTP 2.2.27+


Reference:
Stephen F. Altschul, Thomas L. Madden, Alejandro A. Schäffer,
Jinghui Zhang, Zheng Zhang, Webb Miller, and David J. Lipman (1997),
"Gapped BLAST and PSI-BLAST: a new generation of protein database
search programs", Nucleic Acids Res. 25:3389-3402.


Reference for
composition-based statistics:
Alejandro A. Schäffer, L. Aravind, Thomas L. Madden, Sergei
Shavirin, John L. Spouge, Yuri I. Wolf, Eugene V. Koonin, and
Stephen F. Altschul (2001), "Improving the accuracy of PSI-BLAST
protein database searches with composition-based statistics and
other refinements", Nucleic Acids Res. 29:2994-3005.


Database: nr
           71,551,133 sequences; 26,053,659,533 total letters


Query= G01418_P

Length=372
                                                                      Score     E
Sequences producing significant alignments:                          (Bits)  Value

emb|CDZ96712.1|  hypothetical protein [Xanthophyllomyces dendrorh...   744    0.0  
emb|CCZ41546.1|  cell surface protein [Clostridium sp. CAG:122]       60.5    2e-06
ref|XP_013422765.1|  hypothetical protein M436DRAFT_86260 [Aureob...  41.6    1.3  


 >emb|CDZ96712.1| hypothetical protein [Xanthophyllomyces dendrorhous]
Length=371

 Score =  744 bits (1920),  Expect = 0.0, Method: Compositional matrix adjust.
 Identities = 371/371 (100%), Positives = 371/371 (100%), Gaps = 0/371 (0%)

Query  1    MSTDPIEDQTDQGFLKTLALCRKAQMMKNRLQRIGSKLAPVYRNPADSPPAALAHTPAPE  60
            MSTDPIEDQTDQGFLKTLALCRKAQMMKNRLQRIGSKLAPVYRNPADSPPAALAHTPAPE
Sbjct  1    MSTDPIEDQTDQGFLKTLALCRKAQMMKNRLQRIGSKLAPVYRNPADSPPAALAHTPAPE  60

Query  61   SLTVGAYHPDLSFAYKPNASLTLPKSLTNDHVGRVLGKRKADEQTSMDEKVNGQKSRPNL  120
            SLTVGAYHPDLSFAYKPNASLTLPKSLTNDHVGRVLGKRKADEQTSMDEKVNGQKSRPNL
Sbjct  61   SLTVGAYHPDLSFAYKPNASLTLPKSLTNDHVGRVLGKRKADEQTSMDEKVNGQKSRPNL  120

Query  121  YHHLLTHRPNHITDTSNEFHEAGKISARHSTRTNQGIEARPLGSRGSFGRSTSTIIRPSP  180
            YHHLLTHRPNHITDTSNEFHEAGKISARHSTRTNQGIEARPLGSRGSFGRSTSTIIRPSP
Sbjct  121  YHHLLTHRPNHITDTSNEFHEAGKISARHSTRTNQGIEARPLGSRGSFGRSTSTIIRPSP  180

Query  181  TSIYMNELPDELASNASSCSSIAAAVTPKTTNSIVFHPSIPPVVKSDPSARPLAKHSVPP  240
            TSIYMNELPDELASNASSCSSIAAAVTPKTTNSIVFHPSIPPVVKSDPSARPLAKHSVPP
Sbjct  181  TSIYMNELPDELASNASSCSSIAAAVTPKTTNSIVFHPSIPPVVKSDPSARPLAKHSVPP  240

Query  241  DPVKTDKAFVSNSPLLVHPHPSPIHPSIHPLNLPPSPCSPNSILSFPSSFASTKPATSPS  300
            DPVKTDKAFVSNSPLLVHPHPSPIHPSIHPLNLPPSPCSPNSILSFPSSFASTKPATSPS
Sbjct  241  DPVKTDKAFVSNSPLLVHPHPSPIHPSIHPLNLPPSPCSPNSILSFPSSFASTKPATSPS  300

Query  301  PTSITKQTATTMDLLPVTPTPLSADSCTSTRQIKGPLGVTSPSPTLAPRFQPPTGPTDSW  360
            PTSITKQTATTMDLLPVTPTPLSADSCTSTRQIKGPLGVTSPSPTLAPRFQPPTGPTDSW
Sbjct  301  PTSITKQTATTMDLLPVTPTPLSADSCTSTRQIKGPLGVTSPSPTLAPRFQPPTGPTDSW  360

Query  361  PDFGYLGSSIK  371
            PDFGYLGSSIK
Sbjct  361  PDFGYLGSSIK  371


>emb|CCZ41546.1| cell surface protein [Clostridium sp. CAG:122]
Length=1187

 Score = 60.5 bits (145),  Expect = 2e-06, Method: Composition-based stats.
 Identities = 49/158 (31%), Positives = 66/158 (42%), Gaps = 9/158 (6%)

Query  207  TPKTTNSIVFHPSIPPVVKSDPSARPLAKHSVPPDPVKTDKAFV--SNSPLLVHPHPSPI  264
            TP  + S    PS  P   + P+  P     + P P KT    V  + +P    P P+PI
Sbjct  839  TPAVSASPTPKPSASPTPTAPPTLIPTVSPVMTPAPAKTPTVTVKPTKTPAATVPSPTPI  898

Query  265  HPSIHPLNLPPSPCSPNSILSFPSSFASTKPATSPSPTSITKQTATTMDLLPVTPTPLSA  324
                 P+ + P+    NS  + P+   S+KP   P PTS  K T    D    TP P   
Sbjct  899  MTGSPPV-VSPTAIPINSTSAMPNGHVSSKPDKEPEPTSGNKTTPNPTD--RTTPQPADN  955

Query  325  DSCTSTRQIKGPLGVTSPSPTLAPRFQPPTGPTDSWPD  362
             S +   +   PL   SP+PT A   QP + P  S  D
Sbjct  956  ASPSPANE---PLYTISPTPTSATP-QPTSVPGLSEKD  989


>ref|XP_013422765.1| hypothetical protein M436DRAFT_86260 [Aureobasidium namibiae 
CBS 147.97]
 gb|KEQ68520.1| hypothetical protein M436DRAFT_86260 [Aureobasidium namibiae 
CBS 147.97]
Length=792

 Score = 41.6 bits (96),  Expect = 1.3, Method: Compositional matrix adjust.
 Identities = 22/46 (48%), Positives = 29/46 (63%), Gaps = 3/46 (7%)

Query  120  LYHHLLTHRPNHITDTSNEFHEAGKISARHSTRTNQGIEARPLGSR  165
            L   L T RP+ I +T   F+++G+IS R STR+NQ    R LGSR
Sbjct  360  LVKDLPTERPSSILNTPTNFYQSGRISLRRSTRSNQ---RRSLGSR  402


Lambda      K        H        a         alpha
   0.313    0.128    0.384    0.792     4.96 

Gapped
Lambda      K        H        a         alpha    sigma
   0.267   0.0410    0.140     1.90     42.6     43.6 

Effective search space used: 3339135209740


  Database: nr
    Posted date:  Sep 23, 2015 12:05 AM
  Number of letters in database: 26,053,659,533
  Number of sequences in database:  71,551,133


Matrix: BLOSUM62
Gap Penalties: Existence: 11, Extension: 1
Neighboring words threshold: 11
Window for multiple hits: 40
```
