# Supplementary material for: Comparative genomics provides new insights into the diversity, physiology, and sexuality of the only industrially exploited tremellomycete: Phaffia rhodozyma
Source: BMC Genomics. 2016 Nov 9;17:901. doi: 10.1186/s12864-016-3244-7 (PMC5103461; doi:10.1186/s12864-016-3244-7)
Supplement: Additional file 6: — List of orphan genes with links to PFAM (related to Additional file 1: Table S1). (ZIP 1428 kb) [file 12864_2016_3244_MOESM6_ESM.zip › BLAST_HTML_FTR/G03030_P.html]

BLAST Search Results


```
BLASTP 2.2.27+


Reference:
Stephen F. Altschul, Thomas L. Madden, Alejandro A. Schäffer,
Jinghui Zhang, Zheng Zhang, Webb Miller, and David J. Lipman (1997),
"Gapped BLAST and PSI-BLAST: a new generation of protein database
search programs", Nucleic Acids Res. 25:3389-3402.


Reference for
composition-based statistics:
Alejandro A. Schäffer, L. Aravind, Thomas L. Madden, Sergei
Shavirin, John L. Spouge, Yuri I. Wolf, Eugene V. Koonin, and
Stephen F. Altschul (2001), "Improving the accuracy of PSI-BLAST
protein database searches with composition-based statistics and
other refinements", Nucleic Acids Res. 29:2994-3005.


Database: nr
           71,551,133 sequences; 26,053,659,533 total letters


Query= G03030_P

Length=384
                                                                      Score     E
Sequences producing significant alignments:                          (Bits)  Value

emb|CED84411.1|  hypothetical protein [Xanthophyllomyces dendrorh...   776    0.0  
emb|CED83466.1|  cytochrome c subunit vib [Xanthophyllomyces dend...   138    1e-32
ref|WP_018190620.1|  cysteine desulfurase [Leifsonia sp. 109]         44.3    0.18 
ref|XP_007854249.1|  hypothetical protein Moror_4928 [Moniliophth...  39.3    7.5  


 >emb|CED84411.1| hypothetical protein [Xanthophyllomyces dendrorhous]
Length=383

 Score =  776 bits (2003),  Expect = 0.0, Method: Compositional matrix adjust.
 Identities = 383/383 (100%), Positives = 383/383 (100%), Gaps = 0/383 (0%)

Query  1    MSSMFSSKIYQKIVTYRHLDLYSTYRDDPSTKEERQRAVYQDLAKFSLVSREWWNMITPL  60
            MSSMFSSKIYQKIVTYRHLDLYSTYRDDPSTKEERQRAVYQDLAKFSLVSREWWNMITPL
Sbjct  1    MSSMFSSKIYQKIVTYRHLDLYSTYRDDPSTKEERQRAVYQDLAKFSLVSREWWNMITPL  60

Query  61   LYSDIDLMAVDIDNFFGAIDSKPSFGSLVKHISLPSNCYDLVGDREDYFPNLQISIGLEY  120
            LYSDIDLMAVDIDNFFGAIDSKPSFGSLVKHISLPSNCYDLVGDREDYFPNLQISIGLEY
Sbjct  61   LYSDIDLMAVDIDNFFGAIDSKPSFGSLVKHISLPSNCYDLVGDREDYFPNLQISIGLEY  120

Query  121  TPGFGDDDYEADAEKGEKEDEAQIGADLRGLEEEVLCFTDWERRISIRNLEVLSAESDGW  180
            TPGFGDDDYEADAEKGEKEDEAQIGADLRGLEEEVLCFTDWERRISIRNLEVLSAESDGW
Sbjct  121  TPGFGDDDYEADAEKGEKEDEAQIGADLRGLEEEVLCFTDWERRISIRNLEVLSAESDGW  180

Query  181  QSVTRLLFSLRTIFDLSSLEGLTFIEISDPESPVSPTIDLSAVPMFDHLRVLLTELSLTV  240
            QSVTRLLFSLRTIFDLSSLEGLTFIEISDPESPVSPTIDLSAVPMFDHLRVLLTELSLTV
Sbjct  181  QSVTRLLFSLRTIFDLSSLEGLTFIEISDPESPVSPTIDLSAVPMFDHLRVLLTELSLTV  240

Query  241  RELSLTTPSIDLFRLFTRTLSNAYRMSIPSTLASLVLAQECHPTVTSLWITTEPGLEPQD  300
            RELSLTTPSIDLFRLFTRTLSNAYRMSIPSTLASLVLAQECHPTVTSLWITTEPGLEPQD
Sbjct  241  RELSLTTPSIDLFRLFTRTLSNAYRMSIPSTLASLVLAQECHPTVTSLWITTEPGLEPQD  300

Query  301  SFPRPQDFLADTKIQDRLRQAFPKLEEISTDPIVSESATEEEIKKDGEAVRTLARELGCD  360
            SFPRPQDFLADTKIQDRLRQAFPKLEEISTDPIVSESATEEEIKKDGEAVRTLARELGCD
Sbjct  301  SFPRPQDFLADTKIQDRLRQAFPKLEEISTDPIVSESATEEEIKKDGEAVRTLARELGCD  360

Query  361  RLGIRLVGLDGVLWNQIEGEKEI  383
            RLGIRLVGLDGVLWNQIEGEKEI
Sbjct  361  RLGIRLVGLDGVLWNQIEGEKEI  383


>emb|CED83466.1| cytochrome c subunit vib [Xanthophyllomyces dendrorhous]
Length=590

 Score =  138 bits (347),  Expect = 1e-32, Method: Compositional matrix adjust.
 Identities = 107/350 (31%), Positives = 170/350 (49%), Gaps = 38/350 (11%)

Query  9    IYQKIVTYRHLDLYS-TYRDDPSTKEERQRAVYQDLAKFSLVSREWWNMITPLLYSDIDL  67
            IY++++    LD  S  + +    K+ER+  +++DL    LVS+EW  +  P LY DIDL
Sbjct  15   IYREVIGQLFLDSISPNWLEREEIKDERRIKLFEDLLSCCLVSKEWLAITRPFLYLDIDL  74

Query  68   MAVD-IDNFFGAIDSKPSFGSLVKHISLPSNCYDLVGDREDYFPNLQISIGLEYTPGFGD  126
              +D +D  F  +   P     VKH+S P    +L+  +   FP  Q+S           
Sbjct  75   SCIDNLDALFNTLSLNPYIRPYVKHLSFPMTSIELLETKRHLFPCAQLSFPY--------  126

Query  127  DDYEADAEKGEKEDEAQIGADLRGLEEEVLCFTDWERRISIRNLEVLSAESDGWQSVTRL  186
             D++ D            G+ + G+ + +L FT WER+I +R + + S +   W+   R+
Sbjct  127  -DFDTD------------GSLIDGIPDSLLSFTRWERKIQLRKIVIASWQGRSWEPAARV  173

Query  187  LFSLRTIFDLSSLEGLTFIEISDPESPVSPT-IDLSAVPMFDHL-----RVLLTELSLTV  240
            L     IFDL+ LE L F E+     PV  T ++L+  P    L     R+LL      +
Sbjct  174  LIRASEIFDLTQLEALEFSEL-----PVGDTELELAENPKSSDLLEPLRRILLDSFGPKM  228

Query  241  RELSLTTPSIDLFRLFTRTLSNAYRMSIPSTLASLVLAQECHPTVTSLWITTEPGLEPQD  300
            +EL +TT S  L  LF  +L +  R+SI + LA+++L +  +P + SL I T+P +EP  
Sbjct  229  QELKITTTSRLLLLLFLDSLPDLQRLSISADLANMILNRTSYPRLESLQIMTDPDIEPSP  288

Query  301  SFPRPQDFLADTKIQDRLRQAFPKLEEISTDPIVSESATEEEIKKDGEAV  350
                P    +     +RL +AFP L  I   P +S    E++I  D E +
Sbjct  289  IQEHPDLLFSRNDTLERLARAFPLLRVIGASPYMS----EQDIYADAELI  334


>ref|WP_018190620.1| cysteine desulfurase [Leifsonia sp. 109]
Length=429

 Score = 44.3 bits (103),  Expect = 0.18, Method: Compositional matrix adjust.
 Identities = 47/188 (25%), Positives = 87/188 (46%), Gaps = 18/188 (10%)

Query  202  LTFIEISDPESPVSPTIDLSAVPMFDHLRVLLTELSLTVRELSLTTPSIDL-FRLFTRTL  260
            + F  +S+    +SP  +L A+   +H  +++ +   +V  L++  P++D+ F +F+   
Sbjct  182  VAFAHVSNVLGGISPVAELVAL-AHEHGALVVLDACQSVPHLAVDLPALDVDFAVFS---  237

Query  261  SNAYRMSIPSTLASLV----LAQECHPTVTSLWITTEPGLEPQDSFPRPQDFLADTKIQD  316
               ++M  P+ + +L     L     P +T   + T+  LE  +  P PQ F A T+   
Sbjct  238  --GHKMLGPTGVGALYGRSELLNALPPFLTGGSMITQVTLEGAEYLPAPQRFEAGTQRVS  295

Query  317  R---LRQAFPKLEEISTDPI-VSESATEEEI--KKDG-EAVRTLARELGCDRLGIRLVGL  369
            +   L  A   L+ +    I   E A  E +  + DG E VR L    G  R+G+  V +
Sbjct  296  QAVALAAAVDYLDAVGMPAIEAHEEALGERLLARLDGVEGVRVLGPGAGVPRVGLASVVV  355

Query  370  DGVLWNQI  377
            DG+  + +
Sbjct  356  DGIHAHDV  363


>ref|XP_007854249.1| hypothetical protein Moror_4928 [Moniliophthora roreri MCA 2997]
 gb|ESK86455.1| hypothetical protein Moror_4928 [Moniliophthora roreri MCA 2997]
Length=514

 Score = 39.3 bits (90),  Expect = 7.5, Method: Compositional matrix adjust.
 Identities = 37/99 (37%), Positives = 53/99 (54%), Gaps = 8/99 (8%)

Query  2    SSMF-SSKIYQKIVTYRHLDLYSTYRDDPSTKEERQRAVYQDL-AKFSL--VSREWWNMI  57
            SS+F   +I QKI    HL ++     D  ++  R  A  Q++  K SL  V R+W+N  
Sbjct  25   SSIFLPQEILQKIFE-EHL-IFPPTLIDMHSQTWRHPAYRQNMKTKTSLIFVCRDWYNAA  82

Query  58   TPLLYSDIDLMA-VDIDNFFGAIDSKP-SFGSLVKHISL  94
              LLY D+ +   V++D F   + S P S G+LVK ISL
Sbjct  83   IHLLYRDVSIWHLVELDMFIHTLQSSPRSLGTLVKEISL  121


Lambda      K        H        a         alpha
   0.319    0.137    0.399    0.792     4.96 

Gapped
Lambda      K        H        a         alpha    sigma
   0.267   0.0410    0.140     1.90     42.6     43.6 

Effective search space used: 3521269857544


  Database: nr
    Posted date:  Sep 23, 2015 12:05 AM
  Number of letters in database: 26,053,659,533
  Number of sequences in database:  71,551,133


Matrix: BLOSUM62
Gap Penalties: Existence: 11, Extension: 1
Neighboring words threshold: 11
Window for multiple hits: 40
```
