# Supplementary material for: Comparative genomics provides new insights into the diversity, physiology, and sexuality of the only industrially exploited tremellomycete: Phaffia rhodozyma
Source: BMC Genomics. 2016 Nov 9;17:901. doi: 10.1186/s12864-016-3244-7 (PMC5103461; doi:10.1186/s12864-016-3244-7)
Supplement: Additional file 6: — List of orphan genes with links to PFAM (related to Additional file 1: Table S1). (ZIP 1428 kb) [file 12864_2016_3244_MOESM6_ESM.zip › BLAST_HTML_FTR/G02033_P.html]

BLAST Search Results


```
BLASTP 2.2.27+


Reference:
Stephen F. Altschul, Thomas L. Madden, Alejandro A. Schäffer,
Jinghui Zhang, Zheng Zhang, Webb Miller, and David J. Lipman (1997),
"Gapped BLAST and PSI-BLAST: a new generation of protein database
search programs", Nucleic Acids Res. 25:3389-3402.


Reference for
composition-based statistics:
Alejandro A. Schäffer, L. Aravind, Thomas L. Madden, Sergei
Shavirin, John L. Spouge, Yuri I. Wolf, Eugene V. Koonin, and
Stephen F. Altschul (2001), "Improving the accuracy of PSI-BLAST
protein database searches with composition-based statistics and
other refinements", Nucleic Acids Res. 29:2994-3005.


Database: nr
           71,551,133 sequences; 26,053,659,533 total letters


Query= G02033_P

Length=163
                                                                      Score     E
Sequences producing significant alignments:                          (Bits)  Value

emb|CED82237.1|  hypothetical protein [Xanthophyllomyces dendrorh...   275    2e-91
emb|CDZ98203.1|  hypothetical protein [Xanthophyllomyces dendrorh...  79.0    3e-15
emb|CDZ98286.1|  hypothetical protein [Xanthophyllomyces dendrorh...  66.2    9e-11
emb|CED82021.1|  hypothetical protein [Xanthophyllomyces dendrorh...  53.1    1e-06
emb|CDZ98287.1|  hypothetical protein [Xanthophyllomyces dendrorh...  54.3    2e-06
emb|CDZ97624.1|  hypothetical protein [Xanthophyllomyces dendrorh...  46.2    7e-04
ref|XP_006029775.1|  PREDICTED: alpha-1-acid glycoprotein 2 [Alli...  41.2    0.18 


 >emb|CED82237.1| hypothetical protein [Xanthophyllomyces dendrorhous]
Length=169

 Score =  275 bits (703),  Expect = 2e-91, Method: Compositional matrix adjust.
 Identities = 145/149 (97%), Positives = 146/149 (98%), Gaps = 0/149 (0%)

Query  1    MSNNTYTPLPVSQEHLGDSLATSPVFSQNHAASSNPASYPPPPVHETPLAENLCSYKLSL  60
            MSNNTYTPLPVSQEHLGDSLATSPVFSQNHAASSNPASYPPPPVHETPLAENLCSYKLSL
Sbjct  1    MSNNTYTPLPVSQEHLGDSLATSPVFSQNHAASSNPASYPPPPVHETPLAENLCSYKLSL  60

Query  61   PGAFTSDLDVICTRGSSKEDIVDQLSSISSNILSRTPLDRIIFQIPVGGQWSTVLDSSFD  120
            PGAFTSDLDVICTRGSSKEDIVDQLSSISSNILSRTPLDRIIFQIPVGGQWSTVLDSSFD
Sbjct  61   PGAFTSDLDVICTRGSSKEDIVDQLSSISSNILSRTPLDRIIFQIPVGGQWSTVLDSSFD  120

Query  121  DAIVRGRPDRVRVSIALSRKEWWRNLDLI  149
            DAIVRGRPDRVRVSIALSRKEWWR   L+
Sbjct  121  DAIVRGRPDRVRVSIALSRKEWWRTYGLL  149


>emb|CDZ98203.1| hypothetical protein [Xanthophyllomyces dendrorhous]
Length=150

 Score = 79.0 bits (193),  Expect = 3e-15, Method: Compositional matrix adjust.
 Identities = 50/150 (33%), Positives = 82/150 (55%), Gaps = 17/150 (11%)

Query  1    MSNNTYTPLPVSQEHLGDSLATSPVFSQNHAAS---------SNPASYPPPPVHETPLAE  51
            M++N YTPLP + E    SL   P++S++             SNP SYPP  ++ TP   
Sbjct  1    MTSNPYTPLPSTDESAESSL---PLYSKDSRLEGTVPQDVPVSNPTSYPPTALNPTPSGR  57

Query  52   NLCSYKLSLPGAFTSDLD--VICTRGSSKEDIVDQLSSISSNILSRTPLDRIIFQIPVGG  109
               ++K+  P   +SD    V+CT G +KE+ ++ L     ++LSR  ++R++FQIPVG 
Sbjct  58   RFRAFKVVWP---SSDWKHTVMCTWGLTKEETIESLRLALPDVLSRRSVERMVFQIPVGE  114

Query  110  QWSTVLDSSFDDAIVRGRPDRVRVSIALSR  139
             W+ V +S ++D I     + +R+S   +R
Sbjct  115  DWADVSESDWNDLIAGQTFEEIRLSSYFAR  144


>emb|CDZ98286.1| hypothetical protein [Xanthophyllomyces dendrorhous]
Length=141

 Score = 66.2 bits (160),  Expect = 9e-11, Method: Compositional matrix adjust.
 Identities = 35/87 (40%), Positives = 48/87 (55%), Gaps = 0/87 (0%)

Query  51   ENLCSYKLSLPGAFTSDLDVICTRGSSKEDIVDQLSSISSNILSRTPLDRIIFQIPVGGQ  110
            ENL +     PG       VI TRG SKE+ V+QL  I         +DR+ F++ +  Q
Sbjct  7    ENLFTCTFWPPGVHPKPSMVILTRGMSKEETVNQLRHIFLEAFGEVSVDRVNFKVKINDQ  66

Query  111  WSTVLDSSFDDAIVRGRPDRVRVSIAL  137
            W+ V DSS+DD +VRGRPD   ++I  
Sbjct  67   WAIVSDSSWDDVMVRGRPDSADITITF  93


>emb|CED82021.1| hypothetical protein [Xanthophyllomyces dendrorhous]
Length=71

 Score = 53.1 bits (126),  Expect = 1e-06, Method: Compositional matrix adjust.
 Identities = 28/70 (40%), Positives = 45/70 (64%), Gaps = 1/70 (1%)

Query  55   SYKLSLPGAFTSDLDVICTRGSSKEDIVDQLSSISSNILSRTPLDRIIFQIPVGGQWSTV  114
            S+++  P  F  +  VIC  G +K++ V++L S   + LSR  ++R+ FQIPVG  W+ V
Sbjct  2    SFRVVWP-FFGWERAVICELGLTKKETVERLRSALPDDLSRESVERMDFQIPVGEGWADV  60

Query  115  LDSSFDDAIV  124
            +DS +DD I+
Sbjct  61   MDSDWDDLII  70


>emb|CDZ98287.1| hypothetical protein [Xanthophyllomyces dendrorhous]
Length=171

 Score = 54.3 bits (129),  Expect = 2e-06, Method: Compositional matrix adjust.
 Identities = 33/106 (31%), Positives = 54/106 (51%), Gaps = 1/106 (1%)

Query  32   ASSNPASYPPPPVHETPLAENLCSYKLSLPGAFTSDLDVICTRGSSKEDIVDQLSSISSN  91
             S +P+S     + E   A N+ ++K++     +  L V+ + G S+ED + QL  I   
Sbjct  17   GSPSPSSQTATTIKEAS-ARNIVAFKINTGSVPSRSLRVLFSGGLSREDYIKQLRFIHPE  75

Query  92   ILSRTPLDRIIFQIPVGGQWSTVLDSSFDDAIVRGRPDRVRVSIAL  137
            I  R   +++ F I VG  W  V DSS+DD +V+ +   V V I +
Sbjct  76   IFDRVSSNKVTFHILVGLDWFLVSDSSWDDVMVKRQQHAVLVKIKM  121


>emb|CDZ97624.1| hypothetical protein [Xanthophyllomyces dendrorhous]
Length=120

 Score = 46.2 bits (108),  Expect = 7e-04, Method: Compositional matrix adjust.
 Identities = 29/95 (31%), Positives = 49/95 (52%), Gaps = 6/95 (6%)

Query  60   LPGAFTSDL------DVICTRGSSKEDIVDQLSSISSNILSRTPLDRIIFQIPVGGQWST  113
            +PG+ T+ +       V+C  G  KE   + L     + L +   +R+ FQIP G  W++
Sbjct  1    MPGSITNAMPTEFKRTVMCEWGLIKEQTAENLRQAVPDFLGKRSAERMTFQIPAGETWAS  60

Query  114  VLDSSFDDAIVRGRPDRVRVSIALSRKEWWRNLDL  148
            VLD++++D +V      VRV I  +  E +R+  L
Sbjct  61   VLDNNWNDFMVGRTYPEVRVVIIKTGFEEFRDAML  95


>ref|XP_006029775.1| PREDICTED: alpha-1-acid glycoprotein 2 [Alligator sinensis]
Length=204

 Score = 41.2 bits (95),  Expect = 0.18, Method: Compositional matrix adjust.
 Identities = 35/129 (27%), Positives = 56/129 (43%), Gaps = 9/129 (7%)

Query  37   ASYPPPPVHETPLAENLCSYKLSLPGAFTSDLDVICTRGSSKEDIVDQLSSISSNILSRT  96
            AS+  P   E  L +N  +Y    PG    +L VI     ++  IVD+ S I+ +  + T
Sbjct  49   ASHYKPHQREMELLKN--AYFFFYPGKHEDELQVIQVMRFNESCIVDKKSYITIDRSNST  106

Query  97   PLDRIIFQIPVGGQWSTVLDSSFDDAIVRGRPDRVRVSIALSRKEW---WRNLDLIERNE  153
                +  Q P G   + +L SSFDD ++     +V   I+ S +       +L+  +   
Sbjct  107  ----MTIQGPYGNDTAQLLKSSFDDTLIMYHVQQVEKGISFSARSQNVSKEHLEEFKAQA  162

Query  154  TCLSPVNDD  162
             CL    DD
Sbjct  163  ACLGFTEDD  171


Lambda      K        H        a         alpha
   0.318    0.133    0.405    0.792     4.96 

Gapped
Lambda      K        H        a         alpha    sigma
   0.267   0.0410    0.140     1.90     42.6     43.6 

Effective search space used: 650171180504


  Database: nr
    Posted date:  Sep 23, 2015 12:05 AM
  Number of letters in database: 26,053,659,533
  Number of sequences in database:  71,551,133


Matrix: BLOSUM62
Gap Penalties: Existence: 11, Extension: 1
Neighboring words threshold: 11
Window for multiple hits: 40
```
