# Supplementary material for: Comparative genomics provides new insights into the diversity, physiology, and sexuality of the only industrially exploited tremellomycete: Phaffia rhodozyma
Source: BMC Genomics. 2016 Nov 9;17:901. doi: 10.1186/s12864-016-3244-7 (PMC5103461; doi:10.1186/s12864-016-3244-7)
Supplement: Additional file 6: — List of orphan genes with links to PFAM (related to Additional file 1: Table S1). (ZIP 1428 kb) [file 12864_2016_3244_MOESM6_ESM.zip › BLAST_HTML_FTR/G01507_P.html]

BLAST Search Results


```
BLASTP 2.2.27+


Reference:
Stephen F. Altschul, Thomas L. Madden, Alejandro A. Schäffer,
Jinghui Zhang, Zheng Zhang, Webb Miller, and David J. Lipman (1997),
"Gapped BLAST and PSI-BLAST: a new generation of protein database
search programs", Nucleic Acids Res. 25:3389-3402.


Reference for
composition-based statistics:
Alejandro A. Schäffer, L. Aravind, Thomas L. Madden, Sergei
Shavirin, John L. Spouge, Yuri I. Wolf, Eugene V. Koonin, and
Stephen F. Altschul (2001), "Improving the accuracy of PSI-BLAST
protein database searches with composition-based statistics and
other refinements", Nucleic Acids Res. 29:2994-3005.


Database: nr
           71,551,133 sequences; 26,053,659,533 total letters


Query= G01507_P

Length=337
                                                                      Score     E
Sequences producing significant alignments:                          (Bits)  Value

emb|CED83088.1|  hypothetical protein [Xanthophyllomyces dendrorh...   491    6e-171
emb|CEL51874.1|  hypothetical protein RSOLAG1IB_00411 [Rhizoctoni...  38.9    5.4   
emb|CUA74976.1|  hypothetical protein RSOLAG22IIIB_01611 [Rhizoct...  38.1    9.1   


 >emb|CED83088.1| hypothetical protein [Xanthophyllomyces dendrorhous]
Length=326

 Score =  491 bits (1263),  Expect = 6e-171, Method: Compositional matrix adjust.
 Identities = 311/336 (93%), Positives = 312/336 (93%), Gaps = 10/336 (3%)

Query  1    MADTTEVVPISTIAAEDVVEDASEEVVAPAIVEEVPVIEESAAEVEAPIETIAASPTEVA  60
            MADTTEVVPISTIAAEDVVEDASEEVVAPAIVEEVPVIEESAAEVEAPIETIAASPTEVA
Sbjct  1    MADTTEVVPISTIAAEDVVEDASEEVVAPAIVEEVPVIEESAAEVEAPIETIAASPTEVA  60

Query  61   PEAVPVAIPAKATTTEVPATKPASPKKEGFFAKFISGLKEDKKIAAEKKAAAKANKGKWS  120
            PEAVPVAIPAKATTTEVPATKPASPKKEGFFAKFISGLKEDKKIAAEKKAAAKANK    
Sbjct  61   PEAVPVAIPAKATTTEVPATKPASPKKEGFFAKFISGLKEDKKIAAEKKAAAKANKAAAE  120

Query  121  NVVVVLAAAEKKAAEEAAIKKAEESSSKKTEEAAVVEDEAPVVAAAIPKSDDTPVVAPTE  180
                  AA +K             SSSKKTEEAAVVEDEAPVVAAAIPKSDDTPVVAPTE
Sbjct  121  KKAAEEAAIKKAEE----------SSSKKTEEAAVVEDEAPVVAAAIPKSDDTPVVAPTE  170

Query  181  ETAEADVVEPKEEAVLVAADAPTPAVSELKTVESPADVALGAEVEVPEETAEAVTIETAV  240
            ETAEADVVEPKEEAVLVAADAPTPAVSELKTVESPADVALGAEVEVPEETAEAVTIETAV
Sbjct  171  ETAEADVVEPKEEAVLVAADAPTPAVSELKTVESPADVALGAEVEVPEETAEAVTIETAV  230

Query  241  VPEPASVVAPAAVEEKEAKPLAPKAARRLSARIVGIFKGSKSPVSTPTVEKLPESAPVID  300
            VPEPASVVAPAAVEEKEAKPLAPKAARRLSARIVGIFKGSKSPVSTPTVEKLPESAPVID
Sbjct  231  VPEPASVVAPAAVEEKEAKPLAPKAARRLSARIVGIFKGSKSPVSTPTVEKLPESAPVID  290

Query  301  AVPTSEPLSESITPAVEEAAVAPVIEAEAPKSIVVA  336
            AVPTSEPLSESITPAVEEAAVAPVIEAEAPKSIVVA
Sbjct  291  AVPTSEPLSESITPAVEEAAVAPVIEAEAPKSIVVA  326


>emb|CEL51874.1| hypothetical protein RSOLAG1IB_00411 [Rhizoctonia solani AG-1 
IB]
Length=309

 Score = 38.9 bits (89),  Expect = 5.4, Method: Compositional matrix adjust.
 Identities = 20/39 (51%), Positives = 23/39 (59%), Gaps = 1/39 (3%)

Query  263  PKAARRLSARIVGIFKGSKSPVST-PTVEKLPESAPVID  300
            PKA RRLSAR+ G FK    P  T P   K+ E+ P ID
Sbjct  228  PKAGRRLSARVTGFFKPKHKPEETSPLPAKVDENPPKID  266


>emb|CUA74976.1| hypothetical protein RSOLAG22IIIB_01611 [Rhizoctonia solani]
Length=303

 Score = 38.1 bits (87),  Expect = 9.1, Method: Compositional matrix adjust.
 Identities = 19/38 (50%), Positives = 23/38 (61%), Gaps = 1/38 (3%)

Query  264  KAARRLSARIVGIFKGSKSPVST-PTVEKLPESAPVID  300
            KA RRLSAR+ G FK    P  T P   K+ E+ P+ID
Sbjct  223  KAGRRLSARVTGFFKPKHKPEETSPLPAKVDENPPMID  260


Lambda      K        H        a         alpha
   0.303    0.120    0.308    0.792     4.96 

Gapped
Lambda      K        H        a         alpha    sigma
   0.267   0.0410    0.140     1.90     42.6     43.6 

Effective search space used: 2836395551700


  Database: nr
    Posted date:  Sep 23, 2015 12:05 AM
  Number of letters in database: 26,053,659,533
  Number of sequences in database:  71,551,133


Matrix: BLOSUM62
Gap Penalties: Existence: 11, Extension: 1
Neighboring words threshold: 11
Window for multiple hits: 40
```
