# Supplementary material for: Comparative genomics provides new insights into the diversity, physiology, and sexuality of the only industrially exploited tremellomycete: Phaffia rhodozyma
Source: BMC Genomics. 2016 Nov 9;17:901. doi: 10.1186/s12864-016-3244-7 (PMC5103461; doi:10.1186/s12864-016-3244-7)
Supplement: Additional file 6: — List of orphan genes with links to PFAM (related to Additional file 1: Table S1). (ZIP 1428 kb) [file 12864_2016_3244_MOESM6_ESM.zip › BLAST_HTML_FTR/G00129_P.html]

BLAST Search Results


```
BLASTP 2.2.27+


Reference:
Stephen F. Altschul, Thomas L. Madden, Alejandro A. Schäffer,
Jinghui Zhang, Zheng Zhang, Webb Miller, and David J. Lipman (1997),
"Gapped BLAST and PSI-BLAST: a new generation of protein database
search programs", Nucleic Acids Res. 25:3389-3402.


Reference for
composition-based statistics:
Alejandro A. Schäffer, L. Aravind, Thomas L. Madden, Sergei
Shavirin, John L. Spouge, Yuri I. Wolf, Eugene V. Koonin, and
Stephen F. Altschul (2001), "Improving the accuracy of PSI-BLAST
protein database searches with composition-based statistics and
other refinements", Nucleic Acids Res. 29:2994-3005.


Database: nr
           71,551,133 sequences; 26,053,659,533 total letters


Query= G00129_P

Length=815
                                                                      Score     E
Sequences producing significant alignments:                          (Bits)  Value

emb|CED83974.1|  hypothetical protein [Xanthophyllomyces dendrorh...  1619    0.0  


 >emb|CED83974.1| hypothetical protein [Xanthophyllomyces dendrorhous]
Length=805

 Score = 1619 bits (4193),  Expect = 0.0, Method: Compositional matrix adjust.
 Identities = 804/805 (99%), Positives = 805/805 (100%), Gaps = 0/805 (0%)

Query  10   MYSPNSPVFAHHSSDDDGDHAVFCFCGNEVEPDEEGDMGIYCSTACARKDALEALSSNHN  69
            MYSPNSPVFAHHSSDDDGDHAVFCFCGNEVEPDEEGDMGIYCSTACARKDALEALSSNHN
Sbjct  1    MYSPNSPVFAHHSSDDDGDHAVFCFCGNEVEPDEEGDMGIYCSTACARKDALEALSSNHN  60

Query  70   QPQLSSSNSVPPGSFSYLNSTRQPIPSASCPDSLAYSQSGQVVFPPPLSEGNLSGLSPAL  129
            QPQLSSSNSVPPGSFSYLNSTRQPIPSASCPDSLAYSQSGQVVFPPPLSEGNLSGLSPAL
Sbjct  61   QPQLSSSNSVPPGSFSYLNSTRQPIPSASCPDSLAYSQSGQVVFPPPLSEGNLSGLSPAL  120

Query  130  STASSMSSMASSEGMSSHYRRMEKERVALQRQRKKEREEAKQAAAHAAAMEILSTQRRIR  189
            STASSMSSMASSEGMSSHYRRMEKERVALQRQRKKEREEAKQAAAHAAAMEILSTQRRIR
Sbjct  121  STASSMSSMASSEGMSSHYRRMEKERVALQRQRKKEREEAKQAAAHAAAMEILSTQRRIR  180

Query  190  QQRAEYHLRTASSNSISSKPPSSYSFSANMNNTDINSHISSGTTLSRNNTASSTSSTQSR  249
            QQRAEYHLRTASSNSISSKPPSSYSFSANMNNTDINSHISSGTTLSRNNTASSTSSTQSR
Sbjct  181  QQRAEYHLRTASSNSISSKPPSSYSFSANMNNTDINSHISSGTTLSRNNTASSTSSTQSR  240

Query  250  YISTHPSRAKTPDLIHHGRNESLGSNTSSISSAASVAWGWGSRSGWGSQSREGGEEEIEN  309
            YISTHPSRAKTPDLIHHGRNESLGSNTSSISSAASVAWGWGSRSGWGSQSREGGEEEIEN
Sbjct  241  YISTHPSRAKTPDLIHHGRNESLGSNTSSISSAASVAWGWGSRSGWGSQSREGGEEEIEN  300

Query  310  PYLIREEDEELADDDLPDGHHQLPLDSVNLKQTGSLMKRSKANLSIVDALDQAEPQQTDR  369
            PYLIREEDEELADDDLPDGHHQLPLDSVNLKQTGSLMKRSKANLSIVDALDQAEPQQTDR
Sbjct  301  PYLIREEDEELADDDLPDGHHQLPLDSVNLKQTGSLMKRSKANLSIVDALDQAEPQQTDR  360

Query  370  GGLKMGALLDDILDMERGFTVGGSSSPRSNVDGRSDAGASYIPGISPSHPTNNSTSTPRT  429
            GGLKMGALLDDILDMERGFTVGGSSSPRSNVDGRSDAGASYIPGISPSHPTNNSTSTPRT
Sbjct  361  GGLKMGALLDDILDMERGFTVGGSSSPRSNVDGRSDAGASYIPGISPSHPTNNSTSTPRT  420

Query  430  HTNPLHNERSLFTTPMTDANTLEIDAAPIQSISTSSSADMLRTPNEQTGPVSKDVPPHLA  489
            HTNPLHNERSLFTTPMTDA+TLEIDAAPIQSISTSSSADMLRTPNEQTGPVSKDVPPHLA
Sbjct  421  HTNPLHNERSLFTTPMTDADTLEIDAAPIQSISTSSSADMLRTPNEQTGPVSKDVPPHLA  480

Query  490  GITQGRLKRPFVSTPPSSPHTTLQPESYVSRSHHPTSSSLSSISTAPSASTRSISISKFS  549
            GITQGRLKRPFVSTPPSSPHTTLQPESYVSRSHHPTSSSLSSISTAPSASTRSISISKFS
Sbjct  481  GITQGRLKRPFVSTPPSSPHTTLQPESYVSRSHHPTSSSLSSISTAPSASTRSISISKFS  540

Query  550  TSTPPGPSSYSTLGATSSRPCPRLPTAPIPRPRRSTLSTEQHAHHLGLAHRRSVSTPFDI  609
            TSTPPGPSSYSTLGATSSRPCPRLPTAPIPRPRRSTLSTEQHAHHLGLAHRRSVSTPFDI
Sbjct  541  TSTPPGPSSYSTLGATSSRPCPRLPTAPIPRPRRSTLSTEQHAHHLGLAHRRSVSTPFDI  600

Query  610  SLSQPSALTSTPFLSSSHALSPRAGPNLSKKPKHRRSFSANEASSTAGWLEDSSSMASPL  669
            SLSQPSALTSTPFLSSSHALSPRAGPNLSKKPKHRRSFSANEASSTAGWLEDSSSMASPL
Sbjct  601  SLSQPSALTSTPFLSSSHALSPRAGPNLSKKPKHRRSFSANEASSTAGWLEDSSSMASPL  660

Query  670  PPLPVIHSRPRTLVYSPEDSMGDRTVVDLSCSPSQSAREAGGLKLGWELPLSPPSLLGWE  729
            PPLPVIHSRPRTLVYSPEDSMGDRTVVDLSCSPSQSAREAGGLKLGWELPLSPPSLLGWE
Sbjct  661  PPLPVIHSRPRTLVYSPEDSMGDRTVVDLSCSPSQSAREAGGLKLGWELPLSPPSLLGWE  720

Query  730  SDIRDSRSTIQPPITLTSSVLEDENLNLAPPLMLGSDQESGVYMDPDEGRRRVESGQENG  789
            SDIRDSRSTIQPPITLTSSVLEDENLNLAPPLMLGSDQESGVYMDPDEGRRRVESGQENG
Sbjct  721  SDIRDSRSTIQPPITLTSSVLEDENLNLAPPLMLGSDQESGVYMDPDEGRRRVESGQENG  780

Query  790  GGEGMSAWTRSGERLRAVLGWGDRD  814
            GGEGMSAWTRSGERLRAVLGWGDRD
Sbjct  781  GGEGMSAWTRSGERLRAVLGWGDRD  805


Lambda      K        H        a         alpha
   0.309    0.124    0.357    0.792     4.96 

Gapped
Lambda      K        H        a         alpha    sigma
   0.267   0.0410    0.140     1.90     42.6     43.6 

Effective search space used: 9628131277216


  Database: nr
    Posted date:  Sep 23, 2015 12:05 AM
  Number of letters in database: 26,053,659,533
  Number of sequences in database:  71,551,133


Matrix: BLOSUM62
Gap Penalties: Existence: 11, Extension: 1
Neighboring words threshold: 11
Window for multiple hits: 40
```
