# Supplementary material for: Comparative genomics provides new insights into the diversity, physiology, and sexuality of the only industrially exploited tremellomycete: Phaffia rhodozyma
Source: BMC Genomics. 2016 Nov 9;17:901. doi: 10.1186/s12864-016-3244-7 (PMC5103461; doi:10.1186/s12864-016-3244-7)
Supplement: Additional file 6: — List of orphan genes with links to PFAM (related to Additional file 1: Table S1). (ZIP 1428 kb) [file 12864_2016_3244_MOESM6_ESM.zip › BLAST_HTML_FTR/G01076_P.html]

BLAST Search Results


```
BLASTP 2.2.27+


Reference:
Stephen F. Altschul, Thomas L. Madden, Alejandro A. Schäffer,
Jinghui Zhang, Zheng Zhang, Webb Miller, and David J. Lipman (1997),
"Gapped BLAST and PSI-BLAST: a new generation of protein database
search programs", Nucleic Acids Res. 25:3389-3402.


Reference for
composition-based statistics:
Alejandro A. Schäffer, L. Aravind, Thomas L. Madden, Sergei
Shavirin, John L. Spouge, Yuri I. Wolf, Eugene V. Koonin, and
Stephen F. Altschul (2001), "Improving the accuracy of PSI-BLAST
protein database searches with composition-based statistics and
other refinements", Nucleic Acids Res. 29:2994-3005.


Database: nr
           71,551,133 sequences; 26,053,659,533 total letters


Query= G01076_P

Length=692
                                                                      Score     E
Sequences producing significant alignments:                          (Bits)  Value

emb|CDZ97367.1|  hypothetical protein [Xanthophyllomyces dendrorh...  1398    0.0  
ref|WP_043329095.1|  histidine ammonia-lyase [Curvibacter gracilis]   41.2    3.7  


 >emb|CDZ97367.1| hypothetical protein [Xanthophyllomyces dendrorhous]
Length=691

 Score = 1398 bits (3618),  Expect = 0.0, Method: Compositional matrix adjust.
 Identities = 691/691 (100%), Positives = 691/691 (100%), Gaps = 0/691 (0%)

Query  1    MRALQKALVPVPSTKRASLIRCLSYISSARPAAPPFLPKASTGPPSQAASSCSLPDSQGD  60
            MRALQKALVPVPSTKRASLIRCLSYISSARPAAPPFLPKASTGPPSQAASSCSLPDSQGD
Sbjct  1    MRALQKALVPVPSTKRASLIRCLSYISSARPAAPPFLPKASTGPPSQAASSCSLPDSQGD  60

Query  61   WKEPFQRSPILPHMDPSFSSASLPPLPAKPFTKNPLESIKSYMEVSNKVHPESSSPVLIP  120
            WKEPFQRSPILPHMDPSFSSASLPPLPAKPFTKNPLESIKSYMEVSNKVHPESSSPVLIP
Sbjct  61   WKEPFQRSPILPHMDPSFSSASLPPLPAKPFTKNPLESIKSYMEVSNKVHPESSSPVLIP  120

Query  121  SFQPQVDTTYSVMLCNLPHTAQSQDVYAFLSRALDDDRDLPTFSFVTSPLADPSVPRTVV  180
            SFQPQVDTTYSVMLCNLPHTAQSQDVYAFLSRALDDDRDLPTFSFVTSPLADPSVPRTVV
Sbjct  121  SFQPQVDTTYSVMLCNLPHTAQSQDVYAFLSRALDDDRDLPTFSFVTSPLADPSVPRTVV  180

Query  181  VYARDVVQFEQLLALLPRNGPSSANPFDLIPPASPPLPQWRQIGGRTIYAHDPQPKKSKW  240
            VYARDVVQFEQLLALLPRNGPSSANPFDLIPPASPPLPQWRQIGGRTIYAHDPQPKKSKW
Sbjct  181  VYARDVVQFEQLLALLPRNGPSSANPFDLIPPASPPLPQWRQIGGRTIYAHDPQPKKSKW  240

Query  241  RSLERTKDRFVDEDGYHFLLSGVSTTFGLAKVRRVLNRFCQWEGDLEGSRIRALGPEDAP  300
            RSLERTKDRFVDEDGYHFLLSGVSTTFGLAKVRRVLNRFCQWEGDLEGSRIRALGPEDAP
Sbjct  241  RSLERTKDRFVDEDGYHFLLSGVSTTFGLAKVRRVLNRFCQWEGDLEGSRIRALGPEDAP  300

Query  301  SRPYHVILPIRAVLLQPVFDQLFQSSTTVFQPRSEHPSEGYLSSNGPIWLSNTNPIELNI  360
            SRPYHVILPIRAVLLQPVFDQLFQSSTTVFQPRSEHPSEGYLSSNGPIWLSNTNPIELNI
Sbjct  301  SRPYHVILPIRAVLLQPVFDQLFQSSTTVFQPRSEHPSEGYLSSNGPIWLSNTNPIELNI  360

Query  361  LERSLSPHRTSVRPPVLRVELRAEDNQPRSSSISAATSSDRNFRPVRPPETYQDWWKPSF  420
            LERSLSPHRTSVRPPVLRVELRAEDNQPRSSSISAATSSDRNFRPVRPPETYQDWWKPSF
Sbjct  361  LERSLSPHRTSVRPPVLRVELRAEDNQPRSSSISAATSSDRNFRPVRPPETYQDWWKPSF  420

Query  421  QSLKALSYLQNLSFLTSFSLSSLSTRIASPGFSSSSSSAQFGKGVSRSYWKHHVYRRDRF  480
            QSLKALSYLQNLSFLTSFSLSSLSTRIASPGFSSSSSSAQFGKGVSRSYWKHHVYRRDRF
Sbjct  421  QSLKALSYLQNLSFLTSFSLSSLSTRIASPGFSSSSSSAQFGKGVSRSYWKHHVYRRDRF  480

Query  481  NLERSAPAWRRTYLPIYSALLLPLVPASLLSAQNHSFTDLYPVSSVHAYPPALESYVKPH  540
            NLERSAPAWRRTYLPIYSALLLPLVPASLLSAQNHSFTDLYPVSSVHAYPPALESYVKPH
Sbjct  481  NLERSAPAWRRTYLPIYSALLLPLVPASLLSAQNHSFTDLYPVSSVHAYPPALESYVKPH  540

Query  541  LNRVAVEKKKNCEAARVVVSGLPHSLQEGELVRWMKAQRLGDWLQIIRREPTKDRIKSFW  600
            LNRVAVEKKKNCEAARVVVSGLPHSLQEGELVRWMKAQRLGDWLQIIRREPTKDRIKSFW
Sbjct  541  LNRVAVEKKKNCEAARVVVSGLPHSLQEGELVRWMKAQRLGDWLQIIRREPTKDRIKSFW  600

Query  601  SAARLLGGSEQAEPSKGKEAGKTEGWLGDGLTSASVFKLTQNTSRTTSDFLIILPTKNEA  660
            SAARLLGGSEQAEPSKGKEAGKTEGWLGDGLTSASVFKLTQNTSRTTSDFLIILPTKNEA
Sbjct  601  SAARLLGGSEQAEPSKGKEAGKTEGWLGDGLTSASVFKLTQNTSRTTSDFLIILPTKNEA  660

Query  661  FRLARALDDAEFRFSKQNYTIYKLRARVWHV  691
            FRLARALDDAEFRFSKQNYTIYKLRARVWHV
Sbjct  661  FRLARALDDAEFRFSKQNYTIYKLRARVWHV  691


>ref|WP_043329095.1| histidine ammonia-lyase [Curvibacter gracilis]
Length=529

 Score = 41.2 bits (95),  Expect = 3.7, Method: Compositional matrix adjust.
 Identities = 29/89 (33%), Positives = 44/89 (49%), Gaps = 8/89 (9%)

Query  201  PSSANPFDLIPPASPPLPQWRQI--GGRTIYAHDPQPKKSKWRSLERTKDRFVDEDGYHF  258
            P+SA    L+ P    L QWR+I  GG T+  H  +  ++   + +R   R VDED   +
Sbjct  13   PTSAAAELLLEPGRVSLAQWRRIAAGGLTLRLH--ESARTGLLAAQRCVQRIVDEDQVVY  70

Query  259  LLSGVSTTFG-LAKVRRVLNRFCQWEGDL  286
               G++T FG LA  R    R  + + +L
Sbjct  71   ---GINTGFGKLASTRIAHERLAELQRNL  96


Lambda      K        H        a         alpha
   0.318    0.132    0.401    0.792     4.96 

Gapped
Lambda      K        H        a         alpha    sigma
   0.267   0.0410    0.140     1.90     42.6     43.6 

Effective search space used: 7875741997146


  Database: nr
    Posted date:  Sep 23, 2015 12:05 AM
  Number of letters in database: 26,053,659,533
  Number of sequences in database:  71,551,133


Matrix: BLOSUM62
Gap Penalties: Existence: 11, Extension: 1
Neighboring words threshold: 11
Window for multiple hits: 40
```
