# Supplementary material for: Comparative genomics provides new insights into the diversity, physiology, and sexuality of the only industrially exploited tremellomycete: Phaffia rhodozyma
Source: BMC Genomics. 2016 Nov 9;17:901. doi: 10.1186/s12864-016-3244-7 (PMC5103461; doi:10.1186/s12864-016-3244-7)
Supplement: Additional file 6: — List of orphan genes with links to PFAM (related to Additional file 1: Table S1). (ZIP 1428 kb) [file 12864_2016_3244_MOESM6_ESM.zip › BLAST_HTML_FTR/G01771_P.html]

BLAST Search Results


```
BLASTP 2.2.27+


Reference:
Stephen F. Altschul, Thomas L. Madden, Alejandro A. Schäffer,
Jinghui Zhang, Zheng Zhang, Webb Miller, and David J. Lipman (1997),
"Gapped BLAST and PSI-BLAST: a new generation of protein database
search programs", Nucleic Acids Res. 25:3389-3402.


Reference for
composition-based statistics:
Alejandro A. Schäffer, L. Aravind, Thomas L. Madden, Sergei
Shavirin, John L. Spouge, Yuri I. Wolf, Eugene V. Koonin, and
Stephen F. Altschul (2001), "Improving the accuracy of PSI-BLAST
protein database searches with composition-based statistics and
other refinements", Nucleic Acids Res. 29:2994-3005.


Database: nr
           71,551,133 sequences; 26,053,659,533 total letters


Query= G01771_P

Length=284
                                                                      Score     E
Sequences producing significant alignments:                          (Bits)  Value

emb|CDZ96973.1|  hypothetical protein [Xanthophyllomyces dendrorh...   489    4e-172
emb|CDZ96979.1|  hypothetical protein [Xanthophyllomyces dendrorh...   101    1e-21 
ref|WP_044356590.1|  membrane protein [Burkholderia pseudomallei]...  41.2    0.89  
ref|WP_011851537.1|  membrane protein [Burkholderia pseudomallei]...  40.8    1.2   
gb|KGC53894.1|  putative transmembrane protein [Burkholderia pseu...  40.8    1.4   
ref|WP_038777157.1|  membrane protein [Burkholderia pseudomallei]...  40.8    1.5   
ref|WP_038777378.1|  membrane protein [Burkholderia pseudomallei]...  40.8    1.5   
ref|WP_043311923.1|  membrane protein [Burkholderia pseudomallei]...  40.8    1.5   
ref|WP_038760726.1|  membrane protein [Burkholderia pseudomallei]...  40.8    1.5   
ref|WP_038760000.1|  membrane protein [Burkholderia pseudomallei]     40.8    1.5   
ref|WP_038736435.1|  membrane protein [Burkholderia pseudomallei]...  40.8    1.5   
ref|WP_038726018.1|  membrane protein [Burkholderia pseudomallei]...  40.8    1.5   
ref|WP_038772140.1|  membrane protein [Burkholderia pseudomallei]...  40.8    1.5   
ref|WP_038781107.1|  membrane protein [Burkholderia pseudomallei]...  40.8    1.5   
gb|AJX85637.1|  hypothetical protein BH02_258 [Burkholderia pseud...  40.8    1.5   
gb|KGD47406.1|  putative transmembrane protein [Burkholderia pseu...  40.4    1.6   
gb|AIV64635.1|  putative transmembrane protein [Burkholderia pseu...  40.4    1.6   
ref|WP_038732303.1|  membrane protein [Burkholderia pseudomallei]...  40.4    1.7   
ref|WP_050864536.1|  hypothetical protein [Burkholderia pseudomal...  40.4    2.1   
ref|WP_038752545.1|  membrane protein [Burkholderia pseudomallei]     40.0    2.6   
ref|WP_041197152.1|  membrane protein [Burkholderia pseudomallei]...  40.0    2.6   
ref|WP_004191362.1|  membrane protein [Burkholderia mallei] >gb|A...  40.0    2.7   
ref|WP_024900466.1|  membrane protein [Burkholderia mallei] >gb|A...  40.0    2.7   
ref|WP_050868030.1|  hypothetical protein [Burkholderia pseudomal...  40.0    2.7   
ref|WP_038727716.1|  MULTISPECIES: membrane protein [pseudomallei...  40.0    2.7   
ref|WP_053811533.1|  hypothetical protein [Burkholderia mallei] >...  39.7    2.9   
gb|EDS87783.1|  conserved hypothetical protein [Burkholderia pseu...  39.7    3.1   
gb|ABA49191.1|  hypothetical protein BURPS1710b_1936 [Burkholderi...  39.7    3.1   
gb|EEC33528.1|  conserved hypothetical protein [Burkholderia pseu...  39.7    3.2   
gb|AIS30135.1|  putative transmembrane protein [Burkholderia mall...  39.7    3.2   
gb|EEP85279.1|  lipoprotein [Burkholderia mallei GB8 horse 4] >gb...  39.7    3.2   
gb|KGX45198.1|  hypothetical protein Y043_4969 [Burkholderia pseu...  39.7    3.3   
ref|WP_011857895.1|  membrane protein [Burkholderia mallei] >gb|A...  39.7    3.4   
ref|WP_011832185.1|  membrane protein [Burkholderia mallei] >gb|A...  39.7    3.4   
ref|WP_004266755.1|  membrane protein [Burkholderia mallei] >gb|E...  39.7    3.4   
ref|WP_043299750.1|  membrane protein [Burkholderia sp. TSV202]       39.7    3.5   
ref|WP_050865816.1|  hypothetical protein [Burkholderia pseudomal...  39.7    3.5   
ref|WP_044360470.1|  membrane protein [Burkholderia pseudomallei]...  39.7    3.5   
ref|WP_043275366.1|  membrane protein [Burkholderia pseudomallei]     39.7    3.5   
ref|WP_011854067.1|  membrane protein [Burkholderia pseudomallei]...  39.7    3.5   
ref|WP_038736266.1|  membrane protein [Burkholderia pseudomallei]     39.7    3.5   
emb|CPF11207.1|  lipoprotein [Burkholderia pseudomallei]              39.7    3.5   
ref|WP_038744376.1|  membrane protein [Burkholderia pseudomallei]     39.7    3.5   
ref|WP_038788311.1|  membrane protein [Burkholderia pseudomallei]     39.7    3.5   
ref|WP_041198994.1|  membrane protein [Burkholderia pseudomallei]...  39.3    3.7   
ref|WP_004550548.1|  hypothetical protein [Burkholderia pseudomal...  39.3    3.8   
ref|WP_025986021.1|  membrane protein [Burkholderia pseudomallei]...  39.3    3.8   
ref|WP_038717868.1|  membrane protein [Burkholderia pseudomallei]...  39.3    3.8   
ref|WP_041219129.1|  membrane protein [Burkholderia pseudomallei]     39.3    3.8   
ref|WP_038792516.1|  membrane protein [Burkholderia pseudomallei]     39.3    3.8   
ref|WP_050043010.1|  hypothetical protein [Burkholderia pseudomal...  39.3    3.8   
ref|WP_023360518.1|  hypothetical protein [Burkholderia pseudomal...  39.3    3.8   
ref|WP_038795520.1|  membrane protein [Burkholderia pseudomallei]     39.3    3.8   
ref|WP_004552870.1|  hypothetical protein [Burkholderia pseudomal...  39.3    3.8   
ref|WP_038739045.1|  membrane protein [Burkholderia sp. MSHR44]       39.3    3.8   
ref|WP_038753270.1|  membrane protein [Burkholderia pseudomallei]     39.3    3.8   
ref|WP_024430414.1|  membrane protein [Burkholderia pseudomallei]...  39.3    3.8   
ref|WP_038766399.1|  membrane protein [Burkholderia pseudomallei]...  39.3    3.8   
ref|WP_038757948.1|  membrane protein [Burkholderia pseudomallei]     39.3    3.8   
ref|WP_038729505.1|  membrane protein [Burkholderia pseudomallei]...  39.3    3.8   
ref|WP_020850685.1|  hypothetical protein [Burkholderia pseudomal...  39.3    3.9   
ref|WP_038789791.1|  membrane protein [Burkholderia pseudomallei]     39.3    3.9   
gb|KGU98613.1|  hypothetical protein X885_3206 [Burkholderia pseu...  39.3    4.0   
ref|WP_038765304.1|  membrane protein [Burkholderia pseudomallei]...  39.3    4.0   
ref|WP_004521663.1|  membrane protein [Burkholderia pseudomallei]...  39.3    4.0   
ref|WP_038771759.1|  membrane protein [Burkholderia pseudomallei]     39.3    4.0   
ref|WP_025986495.1|  membrane protein [Burkholderia pseudomallei]...  39.3    4.0   
ref|WP_024428749.1|  membrane protein [Burkholderia pseudomallei]...  39.3    4.1   
ref|WP_004526863.1|  MULTISPECIES: membrane protein [Burkholderia...  39.3    4.1   
ref|WP_031313408.1|  membrane protein [Burkholderia pseudomallei]     39.3    4.1   
ref|WP_038775869.1|  membrane protein [Burkholderia pseudomallei]     39.3    4.1   
ref|WP_038718253.1|  membrane protein [Burkholderia pseudomallei]     39.3    4.2   
ref|WP_038768443.1|  membrane protein [Burkholderia pseudomallei]...  39.3    4.2   
gb|AJX36279.1|  hypothetical protein DP45_02490 [Burkholderia pse...  39.3    4.2   
gb|KGU77041.1|  putative transmembrane protein [Burkholderia pseu...  39.3    4.3   
gb|AIV48230.1|  hypothetical protein X988_1416 [Burkholderia pseu...  39.3    4.3   
gb|AIP05596.1|  putative transmembrane protein [Burkholderia pseu...  39.3    4.3   
gb|KGW22311.1|  putative transmembrane protein [Burkholderia pseu...  39.3    4.3   
gb|KGS26498.1|  putative transmembrane protein [Burkholderia pseu...  39.3    4.3   
gb|KGD45241.1|  hypothetical protein DP44_5997 [Burkholderia pseu...  39.3    4.3   
gb|AIO86742.1|  putative transmembrane protein [Burkholderia pseu...  39.3    4.3   
gb|KGU71435.1|  putative transmembrane protein [Burkholderia pseu...  39.3    4.3   
gb|AIP48314.1|  hypothetical protein DR56_3040 [Burkholderia pseu...  39.3    4.3   
gb|AIP51884.1|  putative transmembrane protein [Burkholderia pseu...  39.3    4.3   
gb|KGV23356.1|  putative transmembrane protein [Burkholderia pseu...  39.3    4.3   
gb|ACQ95514.1|  collagen triple helix repeat protein [Burkholderi...  39.3    4.3   
gb|KGC47533.1|  putative transmembrane protein [Burkholderia pseu...  39.3    4.3   
gb|KGV70413.1|  putative transmembrane protein [Burkholderia pseu...  39.3    4.3   
ref|WP_009937686.1|  hypothetical protein, partial [Burkholderia ...  39.3    4.3   
gb|EBA46384.1|  ISBma2, transposase [Burkholderia pseudomallei 30...  39.3    4.3   
ref|WP_050856926.1|  hypothetical protein [Burkholderia pseudomal...  39.3    4.4   
ref|WP_038730104.1|  membrane protein [Burkholderia sp. ABCPW 1] ...  39.3    4.4   
gb|EMP77057.1|  hypothetical protein D512_09113 [Burkholderia pse...  39.3    4.4   
gb|EQA89458.1|  hypothetical protein M218_08355 [Burkholderia pse...  39.3    4.4   
gb|KGD58869.1|  putative transmembrane protein [Burkholderia pseu...  39.3    4.5   
gb|AIP13048.1|  putative transmembrane protein [Burkholderia pseu...  39.3    4.5   
gb|KGX08649.1|  putative transmembrane protein [Burkholderia sp. ...  39.3    4.5   
gb|KGC29861.1|  putative transmembrane protein [Burkholderia pseu...  39.3    4.5   
gb|EDO83984.1|  conserved hypothetical protein [Burkholderia pseu...  39.3    4.5   
gb|KGC49187.1|  putative transmembrane protein [Burkholderia pseu...  39.3    4.5   
gb|KGC69984.1|  putative transmembrane protein [Burkholderia pseu...  39.3    4.5   
gb|KGW62006.1|  putative transmembrane protein [Burkholderia pseu...  39.3    4.5   
gb|EEH24272.1|  conserved hypothetical protein [Burkholderia pseu...  39.3    4.8   
gb|AIV76499.1|  putative transmembrane protein [Burkholderia sp. ...  38.9    5.2   
gb|AIV52115.1|  putative transmembrane protein [Burkholderia pseu...  38.9    5.2   
gb|EES26072.1|  conserved hypothetical protein [Burkholderia pseu...  38.9    5.2   
gb|AIV59718.1|  putative transmembrane protein [Burkholderia pseu...  38.9    5.3   
gb|KGW30836.1|  putative transmembrane protein [Burkholderia pseu...  38.9    5.3   
gb|KGU64818.1|  putative transmembrane protein [Burkholderia pseu...  38.9    5.3   
ref|WP_041196235.1|  membrane protein [Burkholderia pseudomallei]...  38.9    6.3   


 >emb|CDZ96973.1| hypothetical protein [Xanthophyllomyces dendrorhous]
Length=283

 Score =  489 bits (1260),  Expect = 4e-172, Method: Compositional matrix adjust.
 Identities = 283/283 (100%), Positives = 283/283 (100%), Gaps = 0/283 (0%)

Query  1    MLVLPAMAMFFGASSVLAAPAAVVEDRALLGGLLGGSSTSTSSATSGLQSLTSNLLSTVT  60
            MLVLPAMAMFFGASSVLAAPAAVVEDRALLGGLLGGSSTSTSSATSGLQSLTSNLLSTVT
Sbjct  1    MLVLPAMAMFFGASSVLAAPAAVVEDRALLGGLLGGSSTSTSSATSGLQSLTSNLLSTVT  60

Query  61   GLVSTIESTVSGTDAASLLNVNGDVSTDGLTSTVTGLTSALTGDQISTITSALDDIPLVG  120
            GLVSTIESTVSGTDAASLLNVNGDVSTDGLTSTVTGLTSALTGDQISTITSALDDIPLVG
Sbjct  61   GLVSTIESTVSGTDAASLLNVNGDVSTDGLTSTVTGLTSALTGDQISTITSALDDIPLVG  120

Query  121  QPLHDLLETVLELVNQLGLANVASGALASGASGSSSGLGGLLGGLLGGGASSATNLQNII  180
            QPLHDLLETVLELVNQLGLANVASGALASGASGSSSGLGGLLGGLLGGGASSATNLQNII
Sbjct  121  QPLHDLLETVLELVNQLGLANVASGALASGASGSSSGLGGLLGGLLGGGASSATNLQNII  180

Query  181  TQVVSGLSSVSSGVSGASGSTGLNAVTTILDTLKDSTDTNLSGLLGNSNKIVSGLLSTVT  240
            TQVVSGLSSVSSGVSGASGSTGLNAVTTILDTLKDSTDTNLSGLLGNSNKIVSGLLSTVT
Sbjct  181  TQVVSGLSSVSSGVSGASGSTGLNAVTTILDTLKDSTDTNLSGLLGNSNKIVSGLLSTVT  240

Query  241  GLLGANGLLGGLLSGLGLSSGTSGLTSLLSGLGLDSLTSTLGL  283
            GLLGANGLLGGLLSGLGLSSGTSGLTSLLSGLGLDSLTSTLGL
Sbjct  241  GLLGANGLLGGLLSGLGLSSGTSGLTSLLSGLGLDSLTSTLGL  283


>emb|CDZ96979.1| hypothetical protein [Xanthophyllomyces dendrorhous]
Length=276

 Score =  101 bits (252),  Expect = 1e-21, Method: Compositional matrix adjust.
 Identities = 101/296 (34%), Positives = 156/296 (53%), Gaps = 50/296 (17%)

Query  6    AMAMFFGASSVLAAP------AAVVEDRALLGGLLGGSSTSTSSATSGLQSLTSNLLSTV  59
            A     GAS+ LA P       A +EDR L   LLG  + S       LQ++TS L S+V
Sbjct  13   AFTALIGASTALAVPTPAVVERAAIEDRGLFDQLLGLGTVS------QLQAITSTLKSSV  66

Query  60   TGLVSTIESTVSGTDAASLLNVNG-----------DVSTDGLTSTVTGLTSALTGDQIST  108
               ++ I+++ SG+   ++L+  G            VST GL + +  L++ +  DQ + 
Sbjct  67   APTLTQIQTSTSGSGLTNILSNAGLSSSASSTSTASVSTAGLITLLNDLSTLIPADQFNA  126

Query  109  ITSALDDIPL-VGQPLHDLLETVLELVNQLGLANVASGALASGASGSSSGLGGLLGGLLG  167
            I +A+ ++P  +   L+ L+  V+ +V Q+      + A  S AS  SSG  GLL  LLG
Sbjct  127  IKTAIQNLPANISNDLYTLIAKVISVVKQI------AAAQKSSASLVSSGSNGLLASLLG  180

Query  168  GGASSATNLQNIITQVVSGLSSVSSGVSGASGSTGLNAVTTILDTLKDSTDTNLSGLLGN  227
            G  S  TNLQ++ITQVV+ L +++SGV+ ++   G++AV  +  +LK  TD +L+ ++ N
Sbjct  181  G-TSVLTNLQSVITQVVNSLDTINSGVTASTSVAGVDAVAQVFASLKTPTDASLAAIVAN  239

Query  228  SNKIVSGLLSTVTGLLGANGLLGGLLSGLGLSSGTSGLTSLLSGLGLDSLTSTLGL  283
            S KI+SG ++ +  L+                   S LT LL+ LGL SLTSTL  
Sbjct  240  SAKIISGFMALLKPLI-------------------SNLTGLLNQLGLGSLTSTLKF  276


>ref|WP_044356590.1| membrane protein [Burkholderia pseudomallei]
 gb|KIX47491.1| membrane protein [Burkholderia pseudomallei]
Length=506

 Score = 41.2 bits (95),  Expect = 0.89, Method: Compositional matrix adjust.
 Identities = 58/169 (34%), Positives = 91/169 (54%), Gaps = 14/169 (8%)

Query  87   TDGLTSTVTGLTSALTGDQISTITSALDDIPLVGQPLHDLLETVLELVNQLG--LANVAS  144
            T  ++S +T +TS +TG      T  + ++  +G P++ LL T+   +NQ G  ++   +
Sbjct  262  TQTVSSAITPITSMVTGT-----TQTVGNVTGLGAPVNTLLGTIGGGLNQAGALISKTGN  316

Query  145  GALASGASGSSSGLGGLL---GGLLGGGASSATNLQNIITQVVSGLSSVSSGVSGASGST  201
              + +G   + S  G  +   GGLL GG S ATN    IT  V GL+   SGVSGA+  T
Sbjct  317  NPVTTGLGQTVSATGNTITSVGGLLTGG-SGATNPLAPITAAVGGLTGTLSGVSGATSGT  375

Query  202  GLNAVTTILDTLKDSTDTNLSGLLGNSNKI--VSGLLSTVTGLL-GANG  247
             L  +T ++ T+  +      G  G+++ +  V+GL+STVTG L GA G
Sbjct  376  PLAPLTNVVSTVTGALSGATGGATGSTSPLAPVTGLVSTVTGALSGATG  424


>ref|WP_011851537.1| membrane protein [Burkholderia pseudomallei]
 gb|ABN84241.1| conserved hypothetical protein [Burkholderia pseudomallei 668]
Length=534

 Score = 40.8 bits (94),  Expect = 1.2, Method: Compositional matrix adjust.
 Identities = 57/169 (34%), Positives = 91/169 (54%), Gaps = 14/169 (8%)

Query  87   TDGLTSTVTGLTSALTGDQISTITSALDDIPLVGQPLHDLLETVLELVNQLG--LANVAS  144
            T  ++S +T +TS +TG      T  + ++  +G P++ LL T+   +NQ G  ++   +
Sbjct  262  TQTVSSAITPITSMVTGT-----TQTVGNVTGLGAPVNTLLGTIGGGLNQAGALISKTGN  316

Query  145  GALASGASGSSSGLGGLL---GGLLGGGASSATNLQNIITQVVSGLSSVSSGVSGASGST  201
              + +G   + S  G  +   GGLL GG S ATN    IT  V GL+   SGVSGA+  T
Sbjct  317  NPVTTGLGQTVSATGNTITSVGGLLTGG-SGATNPLAPITAAVGGLTGTLSGVSGATSGT  375

Query  202  GLNAVTTILDTLKDSTDTNLSGLLGNSNKI--VSGLLSTVTGLL-GANG  247
             L  +T ++ T+  +      G  G+++ +  ++GL+STVTG L GA G
Sbjct  376  PLAPLTNVVSTVTGALSGATGGATGSTSPLAPITGLVSTVTGALSGATG  424


>gb|KGC53894.1| putative transmembrane protein [Burkholderia pseudomallei]
 gb|KGV13109.1| putative transmembrane protein [Burkholderia pseudomallei MSHR4300]
 gb|AJX79410.1| hypothetical protein BG16_307 [Burkholderia pseudomallei MSHR2543]
Length=512

 Score = 40.8 bits (94),  Expect = 1.4, Method: Compositional matrix adjust.
 Identities = 54/164 (33%), Positives = 88/164 (54%), Gaps = 13/164 (8%)

Query  87   TDGLTSTVTGLTSALTGDQISTITSALDDIPLVGQPLHDLLETVLELVNQLG--LANVAS  144
            T  ++S +T +TS +TG      T  + ++  +G P++ LL T+   +NQ G  ++   +
Sbjct  240  TQTVSSAITPITSMVTGT-----TQTVGNVTGLGAPVNTLLGTIGGGLNQAGALISKTGN  294

Query  145  GALASGASGSSSGLGGLL---GGLLGGGASSATNLQNIITQVVSGLSSVSSGVSGASGST  201
              + +G   + S  G  +   GGLL GG S ATN    IT  V GL+   SGVSGA+  T
Sbjct  295  NPVTTGLGQTVSATGNTITSVGGLLTGG-SGATNPLAPITAAVGGLTGTLSGVSGATSGT  353

Query  202  GLNAVTTILDTLKDSTDTNLSGLLGNSNKI--VSGLLSTVTGLL  243
             L  +T ++ T+  +      G  G+++ +  ++GL+STVTG L
Sbjct  354  PLAPLTNVVSTVTGALSGATGGATGSTSPLAPITGLVSTVTGAL  397


>ref|WP_038777157.1| membrane protein [Burkholderia pseudomallei]
 gb|KGS87560.1| putative transmembrane protein [Burkholderia pseudomallei MSHR5596]
Length=530

 Score = 40.8 bits (94),  Expect = 1.5, Method: Compositional matrix adjust.
 Identities = 54/164 (33%), Positives = 88/164 (54%), Gaps = 13/164 (8%)

Query  87   TDGLTSTVTGLTSALTGDQISTITSALDDIPLVGQPLHDLLETVLELVNQLG--LANVAS  144
            T  ++S +T +TS +TG      T  + ++  +G P++ LL T+   +NQ G  ++   +
Sbjct  262  TQTVSSAITPITSMVTGT-----TQTVGNVTGLGAPVNTLLGTIGGGLNQAGALISKTGN  316

Query  145  GALASGASGSSSGLGGLL---GGLLGGGASSATNLQNIITQVVSGLSSVSSGVSGASGST  201
              + +G   + S  G  +   GGLL GG S ATN    IT  V GL+   SGVSGA+  T
Sbjct  317  NPVTTGLGQTVSATGNTITSVGGLLTGG-SGATNPLAPITAAVGGLTGTLSGVSGATSGT  375

Query  202  GLNAVTTILDTLKDSTDTNLSGLLGNSNKI--VSGLLSTVTGLL  243
             L  +T ++ T+  +      G  G+++ +  ++GL+STVTG L
Sbjct  376  PLAPLTNVVSTVTGALSGATGGATGSTSPLAPITGLVSTVTGAL  419


>ref|WP_038777378.1| membrane protein [Burkholderia pseudomallei]
 gb|KGC98654.1| putative transmembrane protein [Burkholderia pseudomallei]
Length=530

 Score = 40.8 bits (94),  Expect = 1.5, Method: Compositional matrix adjust.
 Identities = 54/164 (33%), Positives = 88/164 (54%), Gaps = 13/164 (8%)

Query  87   TDGLTSTVTGLTSALTGDQISTITSALDDIPLVGQPLHDLLETVLELVNQLG--LANVAS  144
            T  ++S +T +TS +TG      T  + ++  +G P++ LL T+   +NQ G  ++   +
Sbjct  262  TQTVSSAITPITSMVTGT-----TQTVGNVTGLGAPVNTLLGTIGGGLNQAGALISKTGN  316

Query  145  GALASGASGSSSGLGGLL---GGLLGGGASSATNLQNIITQVVSGLSSVSSGVSGASGST  201
              + +G   + S  G  +   GGLL GG S ATN    IT  V GL+   SGVSGA+  T
Sbjct  317  NPVTTGLGQTVSATGNTITSVGGLLTGG-SGATNPLAPITAAVGGLTGTLSGVSGATSGT  375

Query  202  GLNAVTTILDTLKDSTDTNLSGLLGNSNKI--VSGLLSTVTGLL  243
             L  +T ++ T+  +      G  G+++ +  ++GL+STVTG L
Sbjct  376  PLAPLTNVVSTVTGALSGATGGATGSTSPLAPITGLVSTVTGAL  419


>ref|WP_043311923.1| membrane protein [Burkholderia pseudomallei]
 gb|KGS59709.1| putative transmembrane protein [Burkholderia pseudomallei MSHR5609]
 gb|KGW87623.1| putative transmembrane protein [Burkholderia pseudomallei MSHR456]
Length=534

 Score = 40.8 bits (94),  Expect = 1.5, Method: Compositional matrix adjust.
 Identities = 54/164 (33%), Positives = 88/164 (54%), Gaps = 13/164 (8%)

Query  87   TDGLTSTVTGLTSALTGDQISTITSALDDIPLVGQPLHDLLETVLELVNQLG--LANVAS  144
            T  ++S +T +TS +TG      T  + ++  +G P++ LL T+   +NQ G  ++   +
Sbjct  262  TQTVSSAITPITSMVTGT-----TQTVGNVTGLGAPVNTLLGTIGGGLNQAGALISKTGN  316

Query  145  GALASGASGSSSGLGGLL---GGLLGGGASSATNLQNIITQVVSGLSSVSSGVSGASGST  201
              + +G   + S  G  +   GGLL GG S ATN    IT  V GL+   SGVSGA+  T
Sbjct  317  NPVTTGLGQTVSATGNTITSVGGLLTGG-SGATNPLAPITAAVGGLTGTLSGVSGATSGT  375

Query  202  GLNAVTTILDTLKDSTDTNLSGLLGNSNKI--VSGLLSTVTGLL  243
             L  +T ++ T+  +      G  G+++ +  ++GL+STVTG L
Sbjct  376  PLAPLTNVVSTVTGALSGATGGATGSTSPLAPITGLVSTVTGAL  419


>ref|WP_038760726.1| membrane protein [Burkholderia pseudomallei]
 gb|KGS52387.1| putative transmembrane protein [Burkholderia pseudomallei MSHR5492]
 gb|KGX99469.1| putative transmembrane protein [Burkholderia pseudomallei A79D]
 gb|KGY00434.1| putative transmembrane protein [Burkholderia pseudomallei A79C]
Length=534

 Score = 40.8 bits (94),  Expect = 1.5, Method: Compositional matrix adjust.
 Identities = 57/169 (34%), Positives = 91/169 (54%), Gaps = 14/169 (8%)

Query  87   TDGLTSTVTGLTSALTGDQISTITSALDDIPLVGQPLHDLLETVLELVNQLG--LANVAS  144
            T  ++S +T +TS +TG      T  + ++  +G P++ LL T+   +NQ G  ++   +
Sbjct  262  TQTVSSAITPITSMVTGT-----TQTVGNVTGLGAPVNTLLGTIGGGLNQAGALISKTGN  316

Query  145  GALASGASGSSSGLGGLL---GGLLGGGASSATNLQNIITQVVSGLSSVSSGVSGASGST  201
              + +G   + S  G  +   GGLL GG S ATN    IT  V GL+   SGVSGA+  T
Sbjct  317  NPVTTGLGQTVSATGNTITSVGGLLTGG-SGATNPLAPITAAVGGLTGTLSGVSGATSGT  375

Query  202  GLNAVTTILDTLKDSTDTNLSGLLGNSNKI--VSGLLSTVTGLL-GANG  247
             L  +T ++ T+  +      G  G+++ +  ++GL+STVTG L GA G
Sbjct  376  PLAPLTNVVSTVTGALSGATGGATGSTSPLAPITGLVSTVTGALSGATG  424


>ref|WP_038760000.1| membrane protein [Burkholderia pseudomallei]
Length=535

 Score = 40.8 bits (94),  Expect = 1.5, Method: Compositional matrix adjust.
 Identities = 54/164 (33%), Positives = 88/164 (54%), Gaps = 13/164 (8%)

Query  87   TDGLTSTVTGLTSALTGDQISTITSALDDIPLVGQPLHDLLETVLELVNQLG--LANVAS  144
            T  ++S +T +TS +TG      T  + ++  +G P++ LL T+   +NQ G  ++   +
Sbjct  259  TQTVSSAITPITSMVTGT-----TQTVGNVTGLGAPVNTLLGTIGGGLNQAGALISKTGN  313

Query  145  GALASGASGSSSGLGGLL---GGLLGGGASSATNLQNIITQVVSGLSSVSSGVSGASGST  201
              + +G   + S  G  +   GGLL GG S ATN    IT  V GL+   SGVSGA+  T
Sbjct  314  NPVTTGLGQTVSATGNTITSVGGLLTGG-SGATNPLAPITAAVGGLTGTLSGVSGATSGT  372

Query  202  GLNAVTTILDTLKDSTDTNLSGLLGNSNKI--VSGLLSTVTGLL  243
             L  +T ++ T+  +      G  G+++ +  ++GL+STVTG L
Sbjct  373  PLAPLTNVVSTVTGALSGATGGATGSTSPLAPITGLVSTVTGAL  416


>ref|WP_038736435.1| membrane protein [Burkholderia pseudomallei]
 gb|KGX60847.1| putative transmembrane protein [Burkholderia pseudomallei TSV32]
Length=537

 Score = 40.8 bits (94),  Expect = 1.5, Method: Compositional matrix adjust.
 Identities = 57/169 (34%), Positives = 91/169 (54%), Gaps = 14/169 (8%)

Query  87   TDGLTSTVTGLTSALTGDQISTITSALDDIPLVGQPLHDLLETVLELVNQLG--LANVAS  144
            T  ++S +T +TS +TG      T  + ++  +G P++ LL T+   +NQ G  ++   +
Sbjct  265  TQTVSSAITPITSMVTGT-----TQTVGNVTGLGAPVNTLLGTIGGGLNQAGALISKTGN  319

Query  145  GALASGASGSSSGLGGLL---GGLLGGGASSATNLQNIITQVVSGLSSVSSGVSGASGST  201
              + +G   + S  G  +   GGLL GG S ATN    IT  V GL+   SGVSGA+  T
Sbjct  320  NPVTTGLGQTVSATGNTITSVGGLLTGG-SGATNPLAPITAAVGGLTGTLSGVSGATSGT  378

Query  202  GLNAVTTILDTLKDSTDTNLSGLLGNSNKI--VSGLLSTVTGLL-GANG  247
             L  +T ++ T+  +      G  G+++ +  ++GL+STVTG L GA G
Sbjct  379  PLAPLTNVVSTVTGALSGATGGATGSTSPLAPITGLVSTVTGALSGATG  427


>ref|WP_038726018.1| membrane protein [Burkholderia pseudomallei]
 gb|KGV62778.1| putative transmembrane protein [Burkholderia pseudomallei ABCPW 
91]
 gb|KGW78110.1| putative transmembrane protein [Burkholderia pseudomallei MSHR2990]
Length=537

 Score = 40.8 bits (94),  Expect = 1.5, Method: Compositional matrix adjust.
 Identities = 57/169 (34%), Positives = 91/169 (54%), Gaps = 14/169 (8%)

Query  87   TDGLTSTVTGLTSALTGDQISTITSALDDIPLVGQPLHDLLETVLELVNQLG--LANVAS  144
            T  ++S +T +TS +TG      T  + ++  +G P++ LL T+   +NQ G  ++   +
Sbjct  265  TQTVSSAITPITSMVTGT-----TQTVGNVTGLGAPVNTLLGTIGGGLNQAGALISKTGN  319

Query  145  GALASGASGSSSGLGGLL---GGLLGGGASSATNLQNIITQVVSGLSSVSSGVSGASGST  201
              + +G   + S  G  +   GGLL GG S ATN    IT  V GL+   SGVSGA+  T
Sbjct  320  NPVTTGLGQTVSATGNTITSVGGLLTGG-SGATNPLAPITAAVGGLTGTLSGVSGATSGT  378

Query  202  GLNAVTTILDTLKDSTDTNLSGLLGNSNKI--VSGLLSTVTGLL-GANG  247
             L  +T ++ T+  +      G  G+++ +  ++GL+STVTG L GA G
Sbjct  379  PLAPLTNVVSTVTGALSGATGGATGSTSPLAPITGLVSTVTGALSGATG  427


>ref|WP_038772140.1| membrane protein [Burkholderia pseudomallei]
 gb|KGD12315.1| putative transmembrane protein [Burkholderia pseudomallei]
Length=537

 Score = 40.8 bits (94),  Expect = 1.5, Method: Compositional matrix adjust.
 Identities = 54/164 (33%), Positives = 88/164 (54%), Gaps = 13/164 (8%)

Query  87   TDGLTSTVTGLTSALTGDQISTITSALDDIPLVGQPLHDLLETVLELVNQLG--LANVAS  144
            T  ++S +T +TS +TG      T  + ++  +G P++ LL T+   +NQ G  ++   +
Sbjct  265  TQTVSSAITPITSMVTGT-----TQTVGNVTGLGAPVNTLLGTIGGGLNQAGALISKTGN  319

Query  145  GALASGASGSSSGLGGLL---GGLLGGGASSATNLQNIITQVVSGLSSVSSGVSGASGST  201
              + +G   + S  G  +   GGLL GG S ATN    IT  V GL+   SGVSGA+  T
Sbjct  320  NPVTTGLGQTVSATGNTITSVGGLLTGG-SGATNPLAPITAAVGGLTGTLSGVSGATSGT  378

Query  202  GLNAVTTILDTLKDSTDTNLSGLLGNSNKI--VSGLLSTVTGLL  243
             L  +T ++ T+  +      G  G+++ +  ++GL+STVTG L
Sbjct  379  PLAPLTNVVSTVTGALSGATGGATGSTSPLAPITGLVSTVTGAL  422


>ref|WP_038781107.1| membrane protein [Burkholderia pseudomallei]
 gb|KGU94132.1| putative transmembrane protein [Burkholderia pseudomallei MSHR4377]
Length=540

 Score = 40.8 bits (94),  Expect = 1.5, Method: Compositional matrix adjust.
 Identities = 57/169 (34%), Positives = 91/169 (54%), Gaps = 14/169 (8%)

Query  87   TDGLTSTVTGLTSALTGDQISTITSALDDIPLVGQPLHDLLETVLELVNQLG--LANVAS  144
            T  ++S +T +TS +TG      T  + ++  +G P++ LL T+   +NQ G  ++   +
Sbjct  268  TQTVSSAITPITSMVTGT-----TQTVGNVTGLGAPVNTLLGTIGGGLNQAGALISKTGN  322

Query  145  GALASGASGSSSGLGGLL---GGLLGGGASSATNLQNIITQVVSGLSSVSSGVSGASGST  201
              + +G   + S  G  +   GGLL GG S ATN    IT  V GL+   SGVSGA+  T
Sbjct  323  NPVTTGLGQTVSATGNTITSVGGLLTGG-SGATNPLAPITAAVGGLTGTLSGVSGATSGT  381

Query  202  GLNAVTTILDTLKDSTDTNLSGLLGNSNKI--VSGLLSTVTGLL-GANG  247
             L  +T ++ T+  +      G  G+++ +  ++GL+STVTG L GA G
Sbjct  382  PLAPLTNVVSTVTGALSGATGGATGSTSPLAPITGLVSTVTGALSGATG  430


>gb|AJX85637.1| hypothetical protein BH02_258 [Burkholderia pseudomallei]
Length=512

 Score = 40.8 bits (94),  Expect = 1.5, Method: Compositional matrix adjust.
 Identities = 57/169 (34%), Positives = 91/169 (54%), Gaps = 14/169 (8%)

Query  87   TDGLTSTVTGLTSALTGDQISTITSALDDIPLVGQPLHDLLETVLELVNQLG--LANVAS  144
            T  ++S +T +TS +TG      T  + ++  +G P++ LL T+   +NQ G  ++   +
Sbjct  240  TQTVSSAITPITSMVTGT-----TQTVGNVTGLGAPVNTLLGTIGGGLNQAGALISKTGN  294

Query  145  GALASGASGSSSGLGGLL---GGLLGGGASSATNLQNIITQVVSGLSSVSSGVSGASGST  201
              + +G   + S  G  +   GGLL GG S ATN    IT  V GL+   SGVSGA+  T
Sbjct  295  NPVTTGLGQTVSATGNTITSVGGLLTGG-SGATNPLAPITAAVGGLTGTLSGVSGATSGT  353

Query  202  GLNAVTTILDTLKDSTDTNLSGLLGNSNKI--VSGLLSTVTGLL-GANG  247
             L  +T ++ T+  +      G  G+++ +  ++GL+STVTG L GA G
Sbjct  354  PLAPLTNVVSTVTGALSGATGGATGSTSPLAPITGLVSTVTGALSGATG  402


>gb|KGD47406.1| putative transmembrane protein [Burkholderia pseudomallei]
 gb|AIV94806.1| putative transmembrane protein [Burkholderia pseudomallei A79A]
 gb|AIV90459.1| putative transmembrane protein [Burkholderia pseudomallei B03]
Length=512

 Score = 40.4 bits (93),  Expect = 1.6, Method: Compositional matrix adjust.
 Identities = 57/169 (34%), Positives = 91/169 (54%), Gaps = 14/169 (8%)

Query  87   TDGLTSTVTGLTSALTGDQISTITSALDDIPLVGQPLHDLLETVLELVNQLG--LANVAS  144
            T  ++S +T +TS +TG      T  + ++  +G P++ LL T+   +NQ G  ++   +
Sbjct  240  TQTVSSAITPITSMVTGT-----TQTVGNVTGLGAPVNTLLGTIGGGLNQAGALISKTGN  294

Query  145  GALASGASGSSSGLGGLL---GGLLGGGASSATNLQNIITQVVSGLSSVSSGVSGASGST  201
              + +G   + S  G  +   GGLL GG S ATN    IT  V GL+   SGVSGA+  T
Sbjct  295  NPVTTGLGQTVSATGNTITSVGGLLTGG-SGATNPLAPITAAVGGLTGTLSGVSGATSGT  353

Query  202  GLNAVTTILDTLKDSTDTNLSGLLGNSNKI--VSGLLSTVTGLL-GANG  247
             L  +T ++ T+  +      G  G+++ +  ++GL+STVTG L GA G
Sbjct  354  PLAPLTNVVSTVTGALSGATGGATGSTSPLAPITGLVSTVTGALSGATG  402


>gb|AIV64635.1| putative transmembrane protein [Burkholderia pseudomallei K42]
Length=515

 Score = 40.4 bits (93),  Expect = 1.6, Method: Compositional matrix adjust.
 Identities = 57/169 (34%), Positives = 91/169 (54%), Gaps = 14/169 (8%)

Query  87   TDGLTSTVTGLTSALTGDQISTITSALDDIPLVGQPLHDLLETVLELVNQLG--LANVAS  144
            T  ++S +T +TS +TG      T  + ++  +G P++ LL T+   +NQ G  ++   +
Sbjct  243  TQTVSSAITPITSMVTGT-----TQTVGNVTGLGAPVNTLLGTIGGGLNQAGALISKTGN  297

Query  145  GALASGASGSSSGLGGLL---GGLLGGGASSATNLQNIITQVVSGLSSVSSGVSGASGST  201
              + +G   + S  G  +   GGLL GG S ATN    IT  V GL+   SGVSGA+  T
Sbjct  298  NPVTTGLGQTVSATGNTITSVGGLLTGG-SGATNPLAPITAAVGGLTGTLSGVSGATSGT  356

Query  202  GLNAVTTILDTLKDSTDTNLSGLLGNSNKI--VSGLLSTVTGLL-GANG  247
             L  +T ++ T+  +      G  G+++ +  ++GL+STVTG L GA G
Sbjct  357  PLAPLTNVVSTVTGALSGATGGATGSTSPLAPITGLVSTVTGALSGATG  405


>ref|WP_038732303.1| membrane protein [Burkholderia pseudomallei]
 gb|KGV91414.1| putative transmembrane protein [Burkholderia pseudomallei ABCPW 
30]
Length=534

 Score = 40.4 bits (93),  Expect = 1.7, Method: Compositional matrix adjust.
 Identities = 54/164 (33%), Positives = 88/164 (54%), Gaps = 13/164 (8%)

Query  87   TDGLTSTVTGLTSALTGDQISTITSALDDIPLVGQPLHDLLETVLELVNQLG--LANVAS  144
            T  ++S +T +TS +TG      T  + ++  +G P++ LL T+   +NQ G  ++   +
Sbjct  262  TQTVSSAITPITSMVTGT-----TQTVGNVTGLGAPVNTLLGTIGGGLNQAGALISKTGN  316

Query  145  GALASGASGSSSGLGGLL---GGLLGGGASSATNLQNIITQVVSGLSSVSSGVSGASGST  201
              + +G   + S  G  +   GGLL GG S ATN    IT  V GL+   SGVSGA+  T
Sbjct  317  NPVTTGLGQTVSATGNTITSVGGLLTGG-SGATNPLAPITAAVGGLTGTLSGVSGATSGT  375

Query  202  GLNAVTTILDTLKDSTDTNLSGLLGNSNKI--VSGLLSTVTGLL  243
             L  +T ++ T+  +      G  G+++ +  ++GL+STVTG L
Sbjct  376  PLAPLTNVVSTVTGALSGATGGATGSTSPLAPITGLVSTVTGAL  419


>ref|WP_050864536.1| hypothetical protein [Burkholderia pseudomallei]
 gb|KNA35974.1| membrane protein [Burkholderia pseudomallei]
Length=542

 Score = 40.4 bits (93),  Expect = 2.1, Method: Compositional matrix adjust.
 Identities = 54/164 (33%), Positives = 88/164 (54%), Gaps = 13/164 (8%)

Query  87   TDGLTSTVTGLTSALTGDQISTITSALDDIPLVGQPLHDLLETVLELVNQLG--LANVAS  144
            T  ++S +T +TS +TG      T  + ++  +G P++ LL T+   +NQ G  ++   +
Sbjct  271  TQTVSSAITPITSLVTGT-----TQTVGNVTGLGAPVNTLLGTIGGGLNQAGALISKTGN  325

Query  145  GALASGASGSSSGLGGLL---GGLLGGGASSATNLQNIITQVVSGLSSVSSGVSGASGST  201
              + +G   + S  G  +   GGLL GG S ATN    IT  V GL+   SGVSGA+  T
Sbjct  326  NPVTTGLGQTVSATGNTITSVGGLLTGG-SGATNPLAPITAAVGGLTGTLSGVSGATSGT  384

Query  202  GLNAVTTILDTLKDSTDTNLSGLLGNSNKI--VSGLLSTVTGLL  243
             L  +T ++ T+  +      G  G+++ +  ++GL+STVTG L
Sbjct  385  PLAPLTNVVSTVTGALSGATGGATGSTSPLAPITGLVSTVTGAL  428


>ref|WP_038752545.1| membrane protein [Burkholderia pseudomallei]
Length=533

 Score = 40.0 bits (92),  Expect = 2.6, Method: Compositional matrix adjust.
 Identities = 59/172 (34%), Positives = 91/172 (53%), Gaps = 21/172 (12%)

Query  87   TDGLTSTVTGLTSALTGDQISTITSALDDIPLVGQPLHDLLETVLELVNQLG--LANVAS  144
            T  ++S +T +TS +TG      T  + ++  +G P++ LL T+   +NQ G  ++   +
Sbjct  262  TQTVSSAITPITSMVTGT-----TQTVGNVTGLGAPVNTLLGTIGGGLNQAGALISKTGN  316

Query  145  GALASGASGSSSGLGGLL---GGLLGGGASSATNLQNIITQVVSGLSSVSSGVSGASGST  201
              + +G   + S  G  +   GGLL GG S ATN    IT  V GL+   SGVSGA+  T
Sbjct  317  NPVTTGLGQTVSATGNTITSVGGLLTGG-SGATNPLAPITAAVGGLTGTLSGVSGATSGT  375

Query  202  GLNAVTTILDTLKDSTDTNLSGLLGNSNKI-----VSGLLSTVTGLL-GANG  247
             L  +T ++ T+  +    LSG  G++        ++GL+STVTG L GA G
Sbjct  376  PLAPLTNVVSTVTGA----LSGATGSATSTSPLAPITGLVSTVTGALSGATG  423


>ref|WP_041197152.1| membrane protein [Burkholderia pseudomallei]
 gb|KGV73934.1| putative transmembrane protein [Burkholderia pseudomallei MSHR4299]
Length=529

 Score = 40.0 bits (92),  Expect = 2.6, Method: Compositional matrix adjust.
 Identities = 56/167 (34%), Positives = 88/167 (53%), Gaps = 20/167 (12%)

Query  87   TDGLTSTVTGLTSALTGDQISTITSALDDIPLVGQPLHDLLETVLELVNQLG--LANVAS  144
            T  ++S +T +TS +TG      T  + ++  +G P++ LL T+   +NQ G  ++   +
Sbjct  262  TQTVSSAITPITSMVTGT-----TQTVGNVTGLGAPVNTLLGTIGGGLNQAGALISKTGN  316

Query  145  GALASGASGSSSGLGGLL---GGLLGGGASSATNLQNIITQVVSGLSSVSSGVSGASGST  201
              + +G   + S  G  +   GGLL GG S ATN    IT  V GL+   SGVSGA+  T
Sbjct  317  NPVTTGLGQTVSATGNTITSVGGLLTGG-SGATNPLAPITAAVGGLTGTLSGVSGATSGT  375

Query  202  GLNAVTTILDTLKDSTDTNLSGLLGNSNKI-----VSGLLSTVTGLL  243
             L  +T ++ T+  +    LSG  G++        ++GL+STVTG L
Sbjct  376  PLAPLTNVVSTVTGA----LSGATGSATSTSPLAPITGLVSTVTGAL  418


>ref|WP_004191362.1| membrane protein [Burkholderia mallei]
 gb|ABM50454.1| hypothetical protein BMASAVP1_A1489 [Burkholderia mallei SAVP1]
 gb|EDK56361.1| conserved hypothetical protein [Burkholderia mallei FMH]
 7 more sequence titles

gb|EDK60522.1| conserved hypothetical protein [Burkholderia mallei JHU]
 gb|EDP88548.1| conserved hypothetical protein [Burkholderia mallei ATCC 10399]
 gb|AIO82549.1| membrane protein [Burkholderia mallei]
 gb|AIP77862.1| putative transmembrane protein [Burkholderia mallei]
 gb|AJX47417.1| putative transmembrane protein [Burkholderia mallei]
 gb|AJX54202.1| putative transmembrane protein [Burkholderia mallei]
 gb|AJX63608.1| putative transmembrane protein [Burkholderia mallei]

Length=517

 Score = 40.0 bits (92),  Expect = 2.7, Method: Compositional matrix adjust.
 Identities = 59/172 (34%), Positives = 91/172 (53%), Gaps = 21/172 (12%)

Query  87   TDGLTSTVTGLTSALTGDQISTITSALDDIPLVGQPLHDLLETVLELVNQLG--LANVAS  144
            T  ++S +T +TS +TG      T  + ++  +G P++ LL T+   +NQ G  ++   +
Sbjct  250  TQTVSSAITPITSMVTGT-----TQTVGNVTGLGAPVNTLLGTIGGGLNQAGALISKTGN  304

Query  145  GALASGASGSSSGLGGLL---GGLLGGGASSATNLQNIITQVVSGLSSVSSGVSGASGST  201
              + +G   + S  G  +   GGLL GG S ATN    IT  V GL+   SGVSGA+  T
Sbjct  305  NPVTTGLGQTVSATGNTITSVGGLLTGG-SGATNPLAPITAAVGGLTGTLSGVSGATSGT  363

Query  202  GLNAVTTILDTLKDSTDTNLSGLLGNSNKI-----VSGLLSTVTGLL-GANG  247
             L  +T ++ T+  +    LSG  G++        ++GL+STVTG L GA G
Sbjct  364  PLAPLTNVVSTVTGA----LSGATGSATSTSPLAPITGLVSTVTGALSGATG  411


>ref|WP_024900466.1| membrane protein [Burkholderia mallei]
 gb|AIO59796.1| putative transmembrane protein [Burkholderia mallei]
Length=520

 Score = 40.0 bits (92),  Expect = 2.7, Method: Compositional matrix adjust.
 Identities = 59/172 (34%), Positives = 91/172 (53%), Gaps = 21/172 (12%)

Query  87   TDGLTSTVTGLTSALTGDQISTITSALDDIPLVGQPLHDLLETVLELVNQLG--LANVAS  144
            T  ++S +T +TS +TG      T  + ++  +G P++ LL T+   +NQ G  ++   +
Sbjct  253  TQTVSSAITPITSMVTGT-----TQTVGNVTGLGAPVNTLLGTIGGGLNQAGALISKTGN  307

Query  145  GALASGASGSSSGLGGLL---GGLLGGGASSATNLQNIITQVVSGLSSVSSGVSGASGST  201
              + +G   + S  G  +   GGLL GG S ATN    IT  V GL+   SGVSGA+  T
Sbjct  308  NPVTTGLGQTVSATGNTITSVGGLLTGG-SGATNPLAPITAAVGGLTGTLSGVSGATSGT  366

Query  202  GLNAVTTILDTLKDSTDTNLSGLLGNSNKI-----VSGLLSTVTGLL-GANG  247
             L  +T ++ T+  +    LSG  G++        ++GL+STVTG L GA G
Sbjct  367  PLAPLTNVVSTVTGA----LSGATGSATSTSPLAPITGLVSTVTGALSGATG  414


>ref|WP_050868030.1| hypothetical protein [Burkholderia pseudomallei]
 emb|CPH12275.1| lipoprotein [Burkholderia pseudomallei]
Length=529

 Score = 40.0 bits (92),  Expect = 2.7, Method: Compositional matrix adjust.
 Identities = 59/172 (34%), Positives = 91/172 (53%), Gaps = 21/172 (12%)

Query  87   TDGLTSTVTGLTSALTGDQISTITSALDDIPLVGQPLHDLLETVLELVNQLG--LANVAS  144
            T  ++S +T +TS +TG      T  + ++  +G P++ LL T+   +NQ G  ++   +
Sbjct  262  TQTVSSAITPITSMVTGT-----TQTVGNVTGLGAPVNTLLGTIGGGLNQAGALISKTGN  316

Query  145  GALASGASGSSSGLGGLL---GGLLGGGASSATNLQNIITQVVSGLSSVSSGVSGASGST  201
              + +G   + S  G  +   GGLL GG S ATN    IT  V GL+   SGVSGA+  T
Sbjct  317  NPVTTGLGQTVSATGNTITSVGGLLTGG-SGATNPLAPITAAVGGLTGTLSGVSGATSGT  375

Query  202  GLNAVTTILDTLKDSTDTNLSGLLGNSNKI-----VSGLLSTVTGLL-GANG  247
             L  +T ++ T+  +    LSG  G++        ++GL+STVTG L GA G
Sbjct  376  PLAPLTNVVSTVTGA----LSGATGSATSTSPLAPITGLVSTVTGALSGATG  423


>ref|WP_038727716.1| MULTISPECIES: membrane protein [pseudomallei group]
 gb|AIO94374.1| putative transmembrane protein [Burkholderia pseudomallei 576]
 gb|KGD27743.1| putative transmembrane protein [Burkholderia pseudomallei]
 gb|KGX22816.1| putative transmembrane protein [Burkholderia sp. MSHR4009]
 gb|KGX25452.1| putative transmembrane protein [Burkholderia sp. MSHR4018]
Length=533

 Score = 40.0 bits (92),  Expect = 2.7, Method: Compositional matrix adjust.
 Identities = 59/172 (34%), Positives = 91/172 (53%), Gaps = 21/172 (12%)

Query  87   TDGLTSTVTGLTSALTGDQISTITSALDDIPLVGQPLHDLLETVLELVNQLG--LANVAS  144
            T  ++S +T +TS +TG      T  + ++  +G P++ LL T+   +NQ G  ++   +
Sbjct  262  TQTVSSAITPITSMVTGT-----TQTVGNVTGLGAPVNTLLGTIGGGLNQAGALISKTGN  316

Query  145  GALASGASGSSSGLGGLL---GGLLGGGASSATNLQNIITQVVSGLSSVSSGVSGASGST  201
              + +G   + S  G  +   GGLL GG S ATN    IT  V GL+   SGVSGA+  T
Sbjct  317  NPVTTGLGQTVSATGNTITSVGGLLTGG-SGATNPLAPITAAVGGLTGTLSGVSGATSGT  375

Query  202  GLNAVTTILDTLKDSTDTNLSGLLGNSNKI-----VSGLLSTVTGLL-GANG  247
             L  +T ++ T+  +    LSG  G++        ++GL+STVTG L GA G
Sbjct  376  PLAPLTNVVSTVTGA----LSGATGSATSTSPLAPITGLVSTVTGALSGATG  423


>ref|WP_053811533.1| hypothetical protein [Burkholderia mallei]
 gb|KOT21022.1| putative transmembrane protein [Burkholderia mallei]
Length=500

 Score = 39.7 bits (91),  Expect = 2.9, Method: Compositional matrix adjust.
 Identities = 59/172 (34%), Positives = 91/172 (53%), Gaps = 21/172 (12%)

Query  87   TDGLTSTVTGLTSALTGDQISTITSALDDIPLVGQPLHDLLETVLELVNQLG--LANVAS  144
            T  ++S +T +TS +TG      T  + ++  +G P++ LL T+   +NQ G  ++   +
Sbjct  233  TQTVSSAITPITSMVTGT-----TQTVGNVTGLGAPVNTLLGTIGGGLNQAGALISKTGN  287

Query  145  GALASGASGSSSGLGGLL---GGLLGGGASSATNLQNIITQVVSGLSSVSSGVSGASGST  201
              + +G   + S  G  +   GGLL GG S ATN    IT  V GL+   SGVSGA+  T
Sbjct  288  NPVTTGLGQTVSATGNTITSVGGLLTGG-SGATNPLAPITAAVGGLTGTLSGVSGATSGT  346

Query  202  GLNAVTTILDTLKDSTDTNLSGLLGNSNKI-----VSGLLSTVTGLL-GANG  247
             L  +T ++ T+  +    LSG  G++        ++GL+STVTG L GA G
Sbjct  347  PLAPLTNVVSTVTGA----LSGATGSATSTSPLAPITGLVSTVTGALSGATG  394


>gb|EDS87783.1| conserved hypothetical protein [Burkholderia pseudomallei S13]
Length=561

 Score = 39.7 bits (91),  Expect = 3.1, Method: Compositional matrix adjust.
 Identities = 59/172 (34%), Positives = 90/172 (52%), Gaps = 21/172 (12%)

Query  87   TDGLTSTVTGLTSALTGDQISTITSALDDIPLVGQPLHDLLETVLELVNQLG--LANVAS  144
            T  ++S +T +TS +TG      T  + ++  +G P++ LL T+   +NQ G  ++   +
Sbjct  290  TQTVSSAITPITSMVTGT-----TQTVGNVTGLGAPVNTLLGTIGGGLNQAGALISKTGN  344

Query  145  GALASGASGSSSGLGGLL---GGLLGGGASSATNLQNIITQVVSGLSSVSSGVSGASGST  201
              + +G   + S  G  +   GGLL GG S ATN    IT  V GL+   SGVSGA+  T
Sbjct  345  NPVTTGLGQTVSATGNTITSVGGLLTGG-SGATNPLAPITAAVGGLTGTLSGVSGATSGT  403

Query  202  GLNAVTTILDTLKDSTDTNLSGLLGNSNKI-----VSGLLSTVTGLL-GANG  247
             L  +T ++ T+  +    LSG  G +        ++GL+STVTG L GA G
Sbjct  404  PLAPLTNVVSTVTGA----LSGATGGATSTSPLAPITGLVSTVTGALSGATG  451


>gb|ABA49191.1| hypothetical protein BURPS1710b_1936 [Burkholderia pseudomallei 
1710b]
Length=635

 Score = 39.7 bits (91),  Expect = 3.1, Method: Compositional matrix adjust.
 Identities = 56/167 (34%), Positives = 87/167 (52%), Gaps = 20/167 (12%)

Query  87   TDGLTSTVTGLTSALTGDQISTITSALDDIPLVGQPLHDLLETVLELVNQLG--LANVAS  144
            T  ++S +T +TS +TG      T  + ++  +G P++ LL T+   +NQ G  ++   +
Sbjct  360  TQTVSSAITPITSMVTGT-----TQTVGNVTGLGAPVNTLLGTIGGGLNQAGALISKTGN  414

Query  145  GALASGASGSSSGLGGLL---GGLLGGGASSATNLQNIITQVVSGLSSVSSGVSGASGST  201
              + +G   + S  G  +   GGLL GG S ATN    IT  V GL+   SGVSGA+  T
Sbjct  415  NPVTTGLGQTVSATGNTITSVGGLLTGG-SGATNPLAPITAAVGGLTGTLSGVSGATSGT  473

Query  202  GLNAVTTILDTLKDSTDTNLSGLLGNSNKI-----VSGLLSTVTGLL  243
             L  +T ++ T+  +    LSG  G +        ++GL+STVTG L
Sbjct  474  PLAPLTNVVSTVTGA----LSGATGGATSTSPLAPITGLVSTVTGAL  516


>gb|EEC33528.1| conserved hypothetical protein [Burkholderia pseudomallei 576]
Length=514

 Score = 39.7 bits (91),  Expect = 3.2, Method: Compositional matrix adjust.
 Identities = 59/172 (34%), Positives = 91/172 (53%), Gaps = 21/172 (12%)

Query  87   TDGLTSTVTGLTSALTGDQISTITSALDDIPLVGQPLHDLLETVLELVNQLG--LANVAS  144
            T  ++S +T +TS +TG      T  + ++  +G P++ LL T+   +NQ G  ++   +
Sbjct  243  TQTVSSAITPITSMVTGT-----TQTVGNVTGLGAPVNTLLGTIGGGLNQAGALISKTGN  297

Query  145  GALASGASGSSSGLGGLL---GGLLGGGASSATNLQNIITQVVSGLSSVSSGVSGASGST  201
              + +G   + S  G  +   GGLL GG S ATN    IT  V GL+   SGVSGA+  T
Sbjct  298  NPVTTGLGQTVSATGNTITSVGGLLTGG-SGATNPLAPITAAVGGLTGTLSGVSGATSGT  356

Query  202  GLNAVTTILDTLKDSTDTNLSGLLGNSNKI-----VSGLLSTVTGLL-GANG  247
             L  +T ++ T+  +    LSG  G++        ++GL+STVTG L GA G
Sbjct  357  PLAPLTNVVSTVTGA----LSGATGSATSTSPLAPITGLVSTVTGALSGATG  404


>gb|AIS30135.1| putative transmembrane protein [Burkholderia mallei NCTC 10247]
 gb|AJX44100.1| putative transmembrane protein [Burkholderia mallei]
 gb|KOS78095.1| putative transmembrane protein [Burkholderia mallei]
 6 more sequence titles

gb|KOS78951.1| putative transmembrane protein [Burkholderia mallei]
 gb|KOS85484.1| putative transmembrane protein [Burkholderia mallei]
 gb|KOS97878.1| putative transmembrane protein [Burkholderia mallei]
 gb|KOT03354.1| putative transmembrane protein [Burkholderia mallei]
 gb|KOT13568.1| putative transmembrane protein [Burkholderia mallei]
 gb|KOT24910.1| putative transmembrane protein [Burkholderia mallei]

Length=492

 Score = 39.7 bits (91),  Expect = 3.2, Method: Compositional matrix adjust.
 Identities = 59/172 (34%), Positives = 91/172 (53%), Gaps = 21/172 (12%)

Query  87   TDGLTSTVTGLTSALTGDQISTITSALDDIPLVGQPLHDLLETVLELVNQLG--LANVAS  144
            T  ++S +T +TS +TG      T  + ++  +G P++ LL T+   +NQ G  ++   +
Sbjct  225  TQTVSSAITPITSMVTGT-----TQTVGNVTGLGAPVNTLLGTIGGGLNQAGALISKTGN  279

Query  145  GALASGASGSSSGLGGLL---GGLLGGGASSATNLQNIITQVVSGLSSVSSGVSGASGST  201
              + +G   + S  G  +   GGLL GG S ATN    IT  V GL+   SGVSGA+  T
Sbjct  280  NPVTTGLGQTVSATGNTITSVGGLLTGG-SGATNPLAPITAAVGGLTGTLSGVSGATSGT  338

Query  202  GLNAVTTILDTLKDSTDTNLSGLLGNSNKI-----VSGLLSTVTGLL-GANG  247
             L  +T ++ T+  +    LSG  G++        ++GL+STVTG L GA G
Sbjct  339  PLAPLTNVVSTVTGA----LSGATGSATSTSPLAPITGLVSTVTGALSGATG  386


>gb|EEP85279.1| lipoprotein [Burkholderia mallei GB8 horse 4]
 gb|EES45363.1| conserved hypothetical protein [Burkholderia mallei PRL-20]
 gb|AIO51328.1| putative transmembrane protein [Burkholderia mallei]
 gb|AIO62998.1| putative transmembrane protein [Burkholderia mallei]
 gb|KGC78937.1| putative transmembrane protein [Burkholderia mallei]
 gb|KIY05900.1| hypothetical protein DM79_A1655 [Burkholderia mallei]
 gb|AJX02203.1| hypothetical protein BM45_1644 [Burkholderia mallei]
 gb|AJY35533.1| hypothetical protein BO07_1781 [Burkholderia mallei]
Length=495

 Score = 39.7 bits (91),  Expect = 3.2, Method: Compositional matrix adjust.
 Identities = 59/172 (34%), Positives = 91/172 (53%), Gaps = 21/172 (12%)

Query  87   TDGLTSTVTGLTSALTGDQISTITSALDDIPLVGQPLHDLLETVLELVNQLG--LANVAS  144
            T  ++S +T +TS +TG      T  + ++  +G P++ LL T+   +NQ G  ++   +
Sbjct  228  TQTVSSAITPITSMVTGT-----TQTVGNVTGLGAPVNTLLGTIGGGLNQAGALISKTGN  282

Query  145  GALASGASGSSSGLGGLL---GGLLGGGASSATNLQNIITQVVSGLSSVSSGVSGASGST  201
              + +G   + S  G  +   GGLL GG S ATN    IT  V GL+   SGVSGA+  T
Sbjct  283  NPVTTGLGQTVSATGNTITSVGGLLTGG-SGATNPLAPITAAVGGLTGTLSGVSGATSGT  341

Query  202  GLNAVTTILDTLKDSTDTNLSGLLGNSNKI-----VSGLLSTVTGLL-GANG  247
             L  +T ++ T+  +    LSG  G++        ++GL+STVTG L GA G
Sbjct  342  PLAPLTNVVSTVTGA----LSGATGSATSTSPLAPITGLVSTVTGALSGATG  389


>gb|KGX45198.1| hypothetical protein Y043_4969 [Burkholderia pseudomallei MSHR2138]
Length=511

 Score = 39.7 bits (91),  Expect = 3.3, Method: Compositional matrix adjust.
 Identities = 59/172 (34%), Positives = 91/172 (53%), Gaps = 21/172 (12%)

Query  87   TDGLTSTVTGLTSALTGDQISTITSALDDIPLVGQPLHDLLETVLELVNQLG--LANVAS  144
            T  ++S +T +TS +TG      T  + ++  +G P++ LL T+   +NQ G  ++   +
Sbjct  240  TQTVSSAITPITSMVTGT-----TQTVGNVTGLGAPVNTLLGTIGGGLNQAGALISKTGN  294

Query  145  GALASGASGSSSGLGGLL---GGLLGGGASSATNLQNIITQVVSGLSSVSSGVSGASGST  201
              + +G   + S  G  +   GGLL GG S ATN    IT  V GL+   SGVSGA+  T
Sbjct  295  NPVTTGLGQTVSATGNTITSVGGLLTGG-SGATNPLAPITAAVGGLTGTLSGVSGATSGT  353

Query  202  GLNAVTTILDTLKDSTDTNLSGLLGNSNKI-----VSGLLSTVTGLL-GANG  247
             L  +T ++ T+  +    LSG  G++        ++GL+STVTG L GA G
Sbjct  354  PLAPLTNVVSTVTGA----LSGATGSATSTSPLAPITGLVSTVTGALSGATG  401


>ref|WP_011857895.1| membrane protein [Burkholderia mallei]
 gb|ABO04793.1| putative liporotein [Burkholderia mallei NCTC 10247]
Length=516

 Score = 39.7 bits (91),  Expect = 3.4, Method: Compositional matrix adjust.
 Identities = 59/172 (34%), Positives = 91/172 (53%), Gaps = 21/172 (12%)

Query  87   TDGLTSTVTGLTSALTGDQISTITSALDDIPLVGQPLHDLLETVLELVNQLG--LANVAS  144
            T  ++S +T +TS +TG      T  + ++  +G P++ LL T+   +NQ G  ++   +
Sbjct  249  TQTVSSAITPITSMVTGT-----TQTVGNVTGLGAPVNTLLGTIGGGLNQAGALISKTGN  303

Query  145  GALASGASGSSSGLGGLL---GGLLGGGASSATNLQNIITQVVSGLSSVSSGVSGASGST  201
              + +G   + S  G  +   GGLL GG S ATN    IT  V GL+   SGVSGA+  T
Sbjct  304  NPVTTGLGQTVSATGNTITSVGGLLTGG-SGATNPLAPITAAVGGLTGTLSGVSGATSGT  362

Query  202  GLNAVTTILDTLKDSTDTNLSGLLGNSNKI-----VSGLLSTVTGLL-GANG  247
             L  +T ++ T+  +    LSG  G++        ++GL+STVTG L GA G
Sbjct  363  PLAPLTNVVSTVTGA----LSGATGSATSTSPLAPITGLVSTVTGALSGATG  410


>ref|WP_011832185.1| membrane protein [Burkholderia mallei]
 gb|ABN00897.1| putative liporotein [Burkholderia mallei NCTC 10229]
 gb|KKM44828.1| membrane protein [Burkholderia mallei]
Length=519

 Score = 39.7 bits (91),  Expect = 3.4, Method: Compositional matrix adjust.
 Identities = 59/172 (34%), Positives = 91/172 (53%), Gaps = 21/172 (12%)

Query  87   TDGLTSTVTGLTSALTGDQISTITSALDDIPLVGQPLHDLLETVLELVNQLG--LANVAS  144
            T  ++S +T +TS +TG      T  + ++  +G P++ LL T+   +NQ G  ++   +
Sbjct  252  TQTVSSAITPITSMVTGT-----TQTVGNVTGLGAPVNTLLGTIGGGLNQAGALISKTGN  306

Query  145  GALASGASGSSSGLGGLL---GGLLGGGASSATNLQNIITQVVSGLSSVSSGVSGASGST  201
              + +G   + S  G  +   GGLL GG S ATN    IT  V GL+   SGVSGA+  T
Sbjct  307  NPVTTGLGQTVSATGNTITSVGGLLTGG-SGATNPLAPITAAVGGLTGTLSGVSGATSGT  365

Query  202  GLNAVTTILDTLKDSTDTNLSGLLGNSNKI-----VSGLLSTVTGLL-GANG  247
             L  +T ++ T+  +    LSG  G++        ++GL+STVTG L GA G
Sbjct  366  PLAPLTNVVSTVTGA----LSGATGSATSTSPLAPITGLVSTVTGALSGATG  413


>ref|WP_004266755.1| membrane protein [Burkholderia mallei]
 gb|EDK85534.1| putative lipoprotein [Burkholderia mallei 2002721280]
Length=522

 Score = 39.7 bits (91),  Expect = 3.4, Method: Compositional matrix adjust.
 Identities = 59/172 (34%), Positives = 91/172 (53%), Gaps = 21/172 (12%)

Query  87   TDGLTSTVTGLTSALTGDQISTITSALDDIPLVGQPLHDLLETVLELVNQLG--LANVAS  144
            T  ++S +T +TS +TG      T  + ++  +G P++ LL T+   +NQ G  ++   +
Sbjct  255  TQTVSSAITPITSMVTGT-----TQTVGNVTGLGAPVNTLLGTIGGGLNQAGALISKTGN  309

Query  145  GALASGASGSSSGLGGLL---GGLLGGGASSATNLQNIITQVVSGLSSVSSGVSGASGST  201
              + +G   + S  G  +   GGLL GG S ATN    IT  V GL+   SGVSGA+  T
Sbjct  310  NPVTTGLGQTVSATGNTITSVGGLLTGG-SGATNPLAPITAAVGGLTGTLSGVSGATSGT  368

Query  202  GLNAVTTILDTLKDSTDTNLSGLLGNSNKI-----VSGLLSTVTGLL-GANG  247
             L  +T ++ T+  +    LSG  G++        ++GL+STVTG L GA G
Sbjct  369  PLAPLTNVVSTVTGA----LSGATGSATSTSPLAPITGLVSTVTGALSGATG  416


>ref|WP_043299750.1| membrane protein [Burkholderia sp. TSV202]
Length=517

 Score = 39.7 bits (91),  Expect = 3.5, Method: Compositional matrix adjust.
 Identities = 59/172 (34%), Positives = 90/172 (52%), Gaps = 21/172 (12%)

Query  87   TDGLTSTVTGLTSALTGDQISTITSALDDIPLVGQPLHDLLETVLELVNQLG--LANVAS  144
            T  ++S +T +TS +TG      T  + ++  +G P++ LL T+   +NQ G  ++   +
Sbjct  250  TQTVSSAITPITSMVTGT-----TQTVGNVTGLGAPVNTLLGTIGGGLNQAGALISKTGN  304

Query  145  GALASGASGSSSGLGGLL---GGLLGGGASSATNLQNIITQVVSGLSSVSSGVSGASGST  201
              + +G   + S  G  +   GGLL GG S ATN    IT  V GL+   SGVSGA+  T
Sbjct  305  NPVTTGLGQTVSATGNTITSVGGLLTGG-SGATNPLAPITAAVGGLTGTLSGVSGATSGT  363

Query  202  GLNAVTTILDTLKDSTDTNLSGLLGNSNKI-----VSGLLSTVTGLL-GANG  247
             L  +T ++ T+  +    LSG  G +        ++GL+STVTG L GA G
Sbjct  364  PLAPLTNVVSTVTGA----LSGATGGATSTSPLAPITGLVSTVTGALSGATG  411


>ref|WP_050865816.1| hypothetical protein [Burkholderia pseudomallei]
 emb|CPF67072.1| lipoprotein [Burkholderia pseudomallei]
Length=527

 Score = 39.7 bits (91),  Expect = 3.5, Method: Compositional matrix adjust.
 Identities = 59/172 (34%), Positives = 90/172 (52%), Gaps = 21/172 (12%)

Query  87   TDGLTSTVTGLTSALTGDQISTITSALDDIPLVGQPLHDLLETVLELVNQLG--LANVAS  144
            T  ++S +T +TS +TG      T  + ++  +G P++ LL T+   +NQ G  ++   +
Sbjct  256  TQTVSSAITPITSMVTGT-----TQTVGNVTGLGAPVNTLLGTIGGGLNQAGALISKTGN  310

Query  145  GALASGASGSSSGLGGLL---GGLLGGGASSATNLQNIITQVVSGLSSVSSGVSGASGST  201
              + +G   + S  G  +   GGLL GG S ATN    IT  V GL+   SGVSGA+  T
Sbjct  311  NPVTTGLGQTVSATGNTITSVGGLLTGG-SGATNPLAPITAAVGGLTGTLSGVSGATSGT  369

Query  202  GLNAVTTILDTLKDSTDTNLSGLLGNSNKI-----VSGLLSTVTGLL-GANG  247
             L  +T ++ T+  +    LSG  G +        ++GL+STVTG L GA G
Sbjct  370  PLAPLTNVVSTVTGA----LSGATGGATSTSPLAPITGLVSTVTGALSGATG  417


>ref|WP_044360470.1| membrane protein [Burkholderia pseudomallei]
 gb|KIX40329.1| membrane protein [Burkholderia pseudomallei]
Length=533

 Score = 39.7 bits (91),  Expect = 3.5, Method: Compositional matrix adjust.
 Identities = 59/172 (34%), Positives = 90/172 (52%), Gaps = 21/172 (12%)

Query  87   TDGLTSTVTGLTSALTGDQISTITSALDDIPLVGQPLHDLLETVLELVNQLG--LANVAS  144
            T  ++S +T +TS +TG      T  + ++  +G P++ LL T+   +NQ G  ++   +
Sbjct  262  TQTVSSAITPITSMVTGT-----TQTVGNVTGLGAPVNTLLGTIGGGLNQAGALISKTGN  316

Query  145  GALASGASGSSSGLGGLL---GGLLGGGASSATNLQNIITQVVSGLSSVSSGVSGASGST  201
              + +G   + S  G  +   GGLL GG S ATN    IT  V GL+   SGVSGA+  T
Sbjct  317  NPVTTGLGQTVSATGNTITSVGGLLTGG-SGATNPLAPITAAVGGLTGTLSGVSGATSGT  375

Query  202  GLNAVTTILDTLKDSTDTNLSGLLGNSNKI-----VSGLLSTVTGLL-GANG  247
             L  +T ++ T+  +    LSG  G +        ++GL+STVTG L GA G
Sbjct  376  PLAPLTNVVSTVTGA----LSGATGGATSTSPLAPITGLVSTVTGALSGATG  423


>ref|WP_043275366.1| membrane protein [Burkholderia pseudomallei]
Length=533

 Score = 39.7 bits (91),  Expect = 3.5, Method: Compositional matrix adjust.
 Identities = 59/172 (34%), Positives = 90/172 (52%), Gaps = 21/172 (12%)

Query  87   TDGLTSTVTGLTSALTGDQISTITSALDDIPLVGQPLHDLLETVLELVNQLG--LANVAS  144
            T  ++S +T +TS +TG      T  + ++  +G P++ LL T+   +NQ G  ++   +
Sbjct  262  TQTVSSAITPITSMVTGT-----TQTVGNVTGLGAPVNTLLGTIGGGLNQAGALISKTGN  316

Query  145  GALASGASGSSSGLGGLL---GGLLGGGASSATNLQNIITQVVSGLSSVSSGVSGASGST  201
              + +G   + S  G  +   GGLL GG S ATN    IT  V GL+   SGVSGA+  T
Sbjct  317  NPVTTGLGQTVSATGNTITSVGGLLTGG-SGATNPLAPITAAVGGLTGTLSGVSGATSGT  375

Query  202  GLNAVTTILDTLKDSTDTNLSGLLGNSNKI-----VSGLLSTVTGLL-GANG  247
             L  +T ++ T+  +    LSG  G +        ++GL+STVTG L GA G
Sbjct  376  PLAPLTNVVSTVTGA----LSGATGGATSTSPLAPITGLVSTVTGALSGATG  423


>ref|WP_011854067.1| membrane protein [Burkholderia pseudomallei]
 gb|ABN91146.1| conserved hypothetical protein [Burkholderia pseudomallei 1106a]
 emb|CFL10237.1| lipoprotein [Burkholderia pseudomallei]
 77 more sequence titles

emb|CFB48803.1| lipoprotein [Burkholderia pseudomallei]
 emb|CFD83211.1| lipoprotein [Burkholderia pseudomallei]
 emb|CFD86455.1| lipoprotein [Burkholderia pseudomallei]
 emb|CFK33841.1| lipoprotein [Burkholderia pseudomallei]
 emb|CFK39109.1| lipoprotein [Burkholderia pseudomallei]
 emb|CFK47057.1| lipoprotein [Burkholderia pseudomallei]
 emb|CFK48653.1| lipoprotein [Burkholderia pseudomallei]
 emb|CFK57056.1| lipoprotein [Burkholderia pseudomallei]
 emb|CFK61434.1| lipoprotein [Burkholderia pseudomallei]
 emb|CFK65951.1| lipoprotein [Burkholderia pseudomallei]
 emb|CFK64540.1| lipoprotein [Burkholderia pseudomallei]
 emb|CFK74453.1| lipoprotein [Burkholderia pseudomallei]
 emb|CFK87149.1| lipoprotein [Burkholderia pseudomallei]
 emb|CFK93702.1| lipoprotein [Burkholderia pseudomallei]
 emb|CFK96667.1| lipoprotein [Burkholderia pseudomallei]
 emb|CFL10136.1| lipoprotein [Burkholderia pseudomallei]
 emb|CFL08475.1| lipoprotein [Burkholderia pseudomallei]
 emb|CFL30340.1| lipoprotein [Burkholderia pseudomallei]
 emb|CFL37789.1| lipoprotein [Burkholderia pseudomallei]
 emb|CFL37623.1| lipoprotein [Burkholderia pseudomallei]
 emb|CFL48003.1| lipoprotein [Burkholderia pseudomallei]
 emb|CFL47518.1| lipoprotein [Burkholderia pseudomallei]
 emb|CFL51435.1| lipoprotein [Burkholderia pseudomallei]
 emb|CFT68886.1| lipoprotein [Burkholderia pseudomallei]
 emb|CFT50682.1| lipoprotein [Burkholderia pseudomallei]
 emb|CFT50920.1| lipoprotein [Burkholderia pseudomallei]
 emb|CFT74474.1| lipoprotein [Burkholderia pseudomallei]
 emb|CFT85558.1| lipoprotein [Burkholderia pseudomallei]
 emb|CFT83681.1| lipoprotein [Burkholderia pseudomallei]
 emb|CFU24639.1| lipoprotein [Burkholderia pseudomallei]
 emb|CFV69724.1| lipoprotein [Burkholderia pseudomallei]
 emb|CFV75950.1| lipoprotein [Burkholderia pseudomallei]
 emb|CPE37199.1| lipoprotein [Burkholderia pseudomallei]
 emb|CPF66027.1| lipoprotein [Burkholderia pseudomallei]
 emb|CPE68788.1| lipoprotein [Burkholderia pseudomallei]
 emb|CPE83776.1| lipoprotein [Burkholderia pseudomallei]
 emb|CPE98588.1| lipoprotein [Burkholderia pseudomallei]
 emb|CPF68961.1| lipoprotein [Burkholderia pseudomallei]
 emb|CPF16450.1| lipoprotein [Burkholderia pseudomallei]
 emb|CPH81742.1| lipoprotein [Burkholderia pseudomallei]
 emb|CPE12473.1| lipoprotein [Burkholderia pseudomallei]
 emb|CRY12905.1| lipoprotein [Burkholderia pseudomallei]
 emb|CRY20767.1| lipoprotein [Burkholderia pseudomallei]
 emb|CRY24163.1| lipoprotein [Burkholderia pseudomallei]
 emb|CPH32238.1| lipoprotein [Burkholderia pseudomallei]
 emb|CPF15508.1| lipoprotein [Burkholderia pseudomallei]
 emb|CPE54605.1| lipoprotein [Burkholderia pseudomallei]
 emb|CPG24880.1| lipoprotein [Burkholderia pseudomallei]
 emb|CPH14102.1| lipoprotein [Burkholderia pseudomallei]
 emb|CPF12394.1| lipoprotein [Burkholderia pseudomallei]
 emb|CPF87810.1| lipoprotein [Burkholderia pseudomallei]
 emb|CPE61013.1| lipoprotein [Burkholderia pseudomallei]
 emb|CPG09174.1| lipoprotein [Burkholderia pseudomallei]
 emb|CPF06464.1| lipoprotein [Burkholderia pseudomallei]
 emb|CPE40021.1| lipoprotein [Burkholderia pseudomallei]
 emb|CPF66581.1| lipoprotein [Burkholderia pseudomallei]
 emb|CPF86867.1| lipoprotein [Burkholderia pseudomallei]
 emb|CPE94406.1| lipoprotein [Burkholderia pseudomallei]
 emb|CPG05236.1| lipoprotein [Burkholderia pseudomallei]
 emb|CPF09686.1| lipoprotein [Burkholderia pseudomallei]
 emb|CPH22908.1| lipoprotein [Burkholderia pseudomallei]
 emb|CPG26225.1| lipoprotein [Burkholderia pseudomallei]
 emb|CPG28593.1| lipoprotein [Burkholderia pseudomallei]
 emb|CPF57938.1| lipoprotein [Burkholderia pseudomallei]
 emb|CPG05058.1| lipoprotein [Burkholderia pseudomallei]
 emb|CPF93509.1| lipoprotein [Burkholderia pseudomallei]
 emb|CPE49837.1| lipoprotein [Burkholderia pseudomallei]
 emb|CPE25298.1| lipoprotein [Burkholderia pseudomallei]
 emb|CPE75528.1| lipoprotein [Burkholderia pseudomallei]
 emb|CPE95020.1| lipoprotein [Burkholderia pseudomallei]
 emb|CPF81966.1| lipoprotein [Burkholderia pseudomallei]
 emb|CPF79382.1| lipoprotein [Burkholderia pseudomallei]
 emb|CPE40327.1| lipoprotein [Burkholderia pseudomallei]
 emb|CPE75691.1| lipoprotein [Burkholderia pseudomallei]
 emb|CPE98835.1| lipoprotein [Burkholderia pseudomallei]
 emb|CPE98870.1| lipoprotein [Burkholderia pseudomallei]
 emb|CPG32543.1| lipoprotein [Burkholderia pseudomallei]

Length=533

 Score = 39.7 bits (91),  Expect = 3.5, Method: Compositional matrix adjust.
 Identities = 59/172 (34%), Positives = 90/172 (52%), Gaps = 21/172 (12%)

Query  87   TDGLTSTVTGLTSALTGDQISTITSALDDIPLVGQPLHDLLETVLELVNQLG--LANVAS  144
            T  ++S +T +TS +TG      T  + ++  +G P++ LL T+   +NQ G  ++   +
Sbjct  262  TQTVSSAITPITSMVTGT-----TQTVGNVTGLGAPVNTLLGTIGGGLNQAGALISKTGN  316

Query  145  GALASGASGSSSGLGGLL---GGLLGGGASSATNLQNIITQVVSGLSSVSSGVSGASGST  201
              + +G   + S  G  +   GGLL GG S ATN    IT  V GL+   SGVSGA+  T
Sbjct  317  NPVTTGLGQTVSATGNTITSVGGLLTGG-SGATNPLAPITAAVGGLTGTLSGVSGATSGT  375

Query  202  GLNAVTTILDTLKDSTDTNLSGLLGNSNKI-----VSGLLSTVTGLL-GANG  247
             L  +T ++ T+  +    LSG  G +        ++GL+STVTG L GA G
Sbjct  376  PLAPLTNVVSTVTGA----LSGATGGATSTSPLAPITGLVSTVTGALSGATG  423


>ref|WP_038736266.1| membrane protein [Burkholderia pseudomallei]
Length=535

 Score = 39.7 bits (91),  Expect = 3.5, Method: Compositional matrix adjust.
 Identities = 59/172 (34%), Positives = 90/172 (52%), Gaps = 21/172 (12%)

Query  87   TDGLTSTVTGLTSALTGDQISTITSALDDIPLVGQPLHDLLETVLELVNQLG--LANVAS  144
            T  ++S +T +TS +TG      T  + ++  +G P++ LL T+   +NQ G  ++   +
Sbjct  264  TQTVSSAITPITSMVTGT-----TQTVGNVTGLGAPVNTLLGTIGGGLNQAGALISKTGN  318

Query  145  GALASGASGSSSGLGGLL---GGLLGGGASSATNLQNIITQVVSGLSSVSSGVSGASGST  201
              + +G   + S  G  +   GGLL GG S ATN    IT  V GL+   SGVSGA+  T
Sbjct  319  NPVTTGLGQTVSATGNTITSVGGLLTGG-SGATNPLAPITAAVGGLTGTLSGVSGATSGT  377

Query  202  GLNAVTTILDTLKDSTDTNLSGLLGNSNKI-----VSGLLSTVTGLL-GANG  247
             L  +T ++ T+  +    LSG  G +        ++GL+STVTG L GA G
Sbjct  378  PLAPLTNVVSTVTGA----LSGATGGATSTSPLAPITGLVSTVTGALSGATG  425


>emb|CPF11207.1| lipoprotein [Burkholderia pseudomallei]
Length=537

 Score = 39.7 bits (91),  Expect = 3.5, Method: Compositional matrix adjust.
 Identities = 59/172 (34%), Positives = 90/172 (52%), Gaps = 21/172 (12%)

Query  87   TDGLTSTVTGLTSALTGDQISTITSALDDIPLVGQPLHDLLETVLELVNQLG--LANVAS  144
            T  ++S +T +TS +TG      T  + ++  +G P++ LL T+   +NQ G  ++   +
Sbjct  262  TQTVSSAITPITSMVTGT-----TQTVGNVTGLGAPVNTLLGTIGGGLNQAGALISKTGN  316

Query  145  GALASGASGSSSGLGGLL---GGLLGGGASSATNLQNIITQVVSGLSSVSSGVSGASGST  201
              + +G   + S  G  +   GGLL GG S ATN    IT  V GL+   SGVSGA+  T
Sbjct  317  NPVTTGLGQTVSATGNTITSVGGLLTGG-SGATNPLAPITAAVGGLTGTLSGVSGATSGT  375

Query  202  GLNAVTTILDTLKDSTDTNLSGLLGNSNKI-----VSGLLSTVTGLL-GANG  247
             L  +T ++ T+  +    LSG  G +        ++GL+STVTG L GA G
Sbjct  376  PLAPLTNVVSTVTGA----LSGATGGATSTSPLAPITGLVSTVTGALSGATG  423


>ref|WP_038744376.1| membrane protein [Burkholderia pseudomallei]
Length=540

 Score = 39.7 bits (91),  Expect = 3.5, Method: Compositional matrix adjust.
 Identities = 59/172 (34%), Positives = 90/172 (52%), Gaps = 21/172 (12%)

Query  87   TDGLTSTVTGLTSALTGDQISTITSALDDIPLVGQPLHDLLETVLELVNQLG--LANVAS  144
            T  ++S +T +TS +TG      T  + ++  +G P++ LL T+   +NQ G  ++   +
Sbjct  265  TQTVSSAITPITSMVTGT-----TQTVGNVTGLGAPVNTLLGTIGGGLNQAGALISKTGN  319

Query  145  GALASGASGSSSGLGGLL---GGLLGGGASSATNLQNIITQVVSGLSSVSSGVSGASGST  201
              + +G   + S  G  +   GGLL GG S ATN    IT  V GL+   SGVSGA+  T
Sbjct  320  NPVTTGLGQTVSATGNTITSVGGLLTGG-SGATNPLAPITAAVGGLTGTLSGVSGATSGT  378

Query  202  GLNAVTTILDTLKDSTDTNLSGLLGNSNKI-----VSGLLSTVTGLL-GANG  247
             L  +T ++ T+  +    LSG  G +        ++GL+STVTG L GA G
Sbjct  379  PLAPLTNVVSTVTGA----LSGATGGATSTSPLAPITGLVSTVTGALSGATG  426


>ref|WP_038788311.1| membrane protein [Burkholderia pseudomallei]
Length=545

 Score = 39.7 bits (91),  Expect = 3.5, Method: Compositional matrix adjust.
 Identities = 59/172 (34%), Positives = 90/172 (52%), Gaps = 21/172 (12%)

Query  87   TDGLTSTVTGLTSALTGDQISTITSALDDIPLVGQPLHDLLETVLELVNQLG--LANVAS  144
            T  ++S +T +TS +TG      T  + ++  +G P++ LL T+   +NQ G  ++   +
Sbjct  274  TQTVSSAITPITSMVTGT-----TQTVGNVTGLGAPVNTLLGTIGGGLNQAGALISKTGN  328

Query  145  GALASGASGSSSGLGGLL---GGLLGGGASSATNLQNIITQVVSGLSSVSSGVSGASGST  201
              + +G   + S  G  +   GGLL GG S ATN    IT  V GL+   SGVSGA+  T
Sbjct  329  NPVTTGLGQTVSATGNTITSVGGLLTGG-SGATNPLAPITAAVGGLTGTLSGVSGATSGT  387

Query  202  GLNAVTTILDTLKDSTDTNLSGLLGNSNKI-----VSGLLSTVTGLL-GANG  247
             L  +T ++ T+  +    LSG  G +        ++GL+STVTG L GA G
Sbjct  388  PLAPLTNVVSTVTGA----LSGATGGATSTSPLAPITGLVSTVTGALSGATG  435


>ref|WP_041198994.1| membrane protein [Burkholderia pseudomallei]
 gb|KGX47161.1| putative transmembrane protein [Burkholderia pseudomallei MSHR3709]
Length=535

 Score = 39.3 bits (90),  Expect = 3.7, Method: Compositional matrix adjust.
 Identities = 59/172 (34%), Positives = 90/172 (52%), Gaps = 21/172 (12%)

Query  87   TDGLTSTVTGLTSALTGDQISTITSALDDIPLVGQPLHDLLETVLELVNQLG--LANVAS  144
            T  ++S +T +TS +TG      T  + ++  +G P++ LL T+   +NQ G  ++   +
Sbjct  264  TQTVSSAITPITSMVTGT-----TQTVGNVTGLGAPVNTLLGTIGGGLNQAGALISKTGN  318

Query  145  GALASGASGSSSGLGGLL---GGLLGGGASSATNLQNIITQVVSGLSSVSSGVSGASGST  201
              + +G   + S  G  +   GGLL GG S ATN    IT  V GL+   SGVSGA+  T
Sbjct  319  NPVTTGLGQTVSATGNTITSVGGLLTGG-SGATNPLAPITAAVGGLTGTLSGVSGATSGT  377

Query  202  GLNAVTTILDTLKDSTDTNLSGLLGNSNKI-----VSGLLSTVTGLL-GANG  247
             L  +T ++ T+  +    LSG  G +        ++GL+STVTG L GA G
Sbjct  378  PLAPLTNVVSTVTGA----LSGATGGATSTSPLAPITGLVSTVTGALSGATG  425


>ref|WP_004550548.1| hypothetical protein [Burkholderia pseudomallei]
 ref|YP_108501.1| hypothetical protein BPSL1901 [Burkholderia pseudomallei K96243]
 emb|CAH35901.1| putative membrane protein [Burkholderia pseudomallei K96243]
 9 more sequence titles

gb|EIF76696.1| hypothetical protein BP354E_1420 [Burkholderia pseudomallei 354e]
 gb|EIF81012.1| hypothetical protein BP354A_1793 [Burkholderia pseudomallei 354a]
 gb|AJW54882.1| membrane protein [Burkholderia pseudomallei]
 emb|CFL40603.1| lipoprotein [Burkholderia pseudomallei]
 emb|CFT76789.1| lipoprotein [Burkholderia pseudomallei]
 emb|CPH93433.1| lipoprotein [Burkholderia pseudomallei]
 emb|CPF52017.1| lipoprotein [Burkholderia pseudomallei]
 emb|CPG41503.1| lipoprotein [Burkholderia pseudomallei]
 gb|ALC58726.1| hypothetical protein AMS56_11975 [Burkholderia pseudomallei]

Length=529

 Score = 39.3 bits (90),  Expect = 3.8, Method: Compositional matrix adjust.
 Identities = 56/167 (34%), Positives = 87/167 (52%), Gaps = 20/167 (12%)

Query  87   TDGLTSTVTGLTSALTGDQISTITSALDDIPLVGQPLHDLLETVLELVNQLG--LANVAS  144
            T  ++S +T +TS +TG      T  + ++  +G P++ LL T+   +NQ G  ++   +
Sbjct  262  TQTVSSAITPITSMVTGT-----TQTVGNVTGLGAPVNTLLGTIGGGLNQAGALISKTGN  316

Query  145  GALASGASGSSSGLGGLL---GGLLGGGASSATNLQNIITQVVSGLSSVSSGVSGASGST  201
              + +G   + S  G  +   GGLL GG S ATN    IT  V GL+   SGVSGA+  T
Sbjct  317  NPVTTGLGQTVSATGNTITSVGGLLTGG-SGATNPLAPITAAVGGLTGTLSGVSGATSGT  375

Query  202  GLNAVTTILDTLKDSTDTNLSGLLGNSNKI-----VSGLLSTVTGLL  243
             L  +T ++ T+  +    LSG  G +        ++GL+STVTG L
Sbjct  376  PLAPLTNVVSTVTGA----LSGATGGATSTSPLAPITGLVSTVTGAL  418


>ref|WP_025986021.1| membrane protein [Burkholderia pseudomallei]
 gb|KIX68411.1| membrane protein [Burkholderia pseudomallei]
Length=532

 Score = 39.3 bits (90),  Expect = 3.8, Method: Compositional matrix adjust.
 Identities = 56/167 (34%), Positives = 87/167 (52%), Gaps = 20/167 (12%)

Query  87   TDGLTSTVTGLTSALTGDQISTITSALDDIPLVGQPLHDLLETVLELVNQLG--LANVAS  144
            T  ++S +T +TS +TG      T  + ++  +G P++ LL T+   +NQ G  ++   +
Sbjct  265  TQTVSSAITPITSMVTGT-----TQTVGNVTGLGAPVNTLLGTIGGGLNQAGALISKTGN  319

Query  145  GALASGASGSSSGLGGLL---GGLLGGGASSATNLQNIITQVVSGLSSVSSGVSGASGST  201
              + +G   + S  G  +   GGLL GG S ATN    IT  V GL+   SGVSGA+  T
Sbjct  320  NPVTTGLGQTVSATGNTITSVGGLLTGG-SGATNPLAPITAAVGGLTGTLSGVSGATSGT  378

Query  202  GLNAVTTILDTLKDSTDTNLSGLLGNSNKI-----VSGLLSTVTGLL  243
             L  +T ++ T+  +    LSG  G +        ++GL+STVTG L
Sbjct  379  PLAPLTNVVSTVTGA----LSGATGGATSTSPLAPITGLVSTVTGAL  421


>ref|WP_038717868.1| membrane protein [Burkholderia pseudomallei]
 emb|CFL03854.1| lipoprotein [Burkholderia pseudomallei]
 emb|CFL84341.1| lipoprotein [Burkholderia pseudomallei]
 8 more sequence titles

emb|CFT48392.1| lipoprotein [Burkholderia pseudomallei]
 emb|CPF59789.1| lipoprotein [Burkholderia pseudomallei]
 emb|CRY29051.1| lipoprotein [Burkholderia pseudomallei]
 emb|CPF72630.1| lipoprotein [Burkholderia pseudomallei]
 emb|CPG35913.1| lipoprotein [Burkholderia pseudomallei]
 emb|CPG24974.1| lipoprotein [Burkholderia pseudomallei]
 emb|CPG31069.1| lipoprotein [Burkholderia pseudomallei]
 emb|CPF95554.1| lipoprotein [Burkholderia pseudomallei]

Length=533

 Score = 39.3 bits (90),  Expect = 3.8, Method: Compositional matrix adjust.
 Identities = 56/167 (34%), Positives = 87/167 (52%), Gaps = 20/167 (12%)

Query  87   TDGLTSTVTGLTSALTGDQISTITSALDDIPLVGQPLHDLLETVLELVNQLG--LANVAS  144
            T  ++S +T +TS +TG      T  + ++  +G P++ LL T+   +NQ G  ++   +
Sbjct  262  TQTVSSAITPITSMVTGT-----TQTVGNVTGLGAPVNTLLGTIGGGLNQAGALISKTGN  316

Query  145  GALASGASGSSSGLGGLL---GGLLGGGASSATNLQNIITQVVSGLSSVSSGVSGASGST  201
              + +G   + S  G  +   GGLL GG S ATN    IT  V GL+   SGVSGA+  T
Sbjct  317  NPVTTGLGQTVSATGNTITSVGGLLTGG-SGATNPLAPITAAVGGLTGTLSGVSGATSGT  375

Query  202  GLNAVTTILDTLKDSTDTNLSGLLGNSNKI-----VSGLLSTVTGLL  243
             L  +T ++ T+  +    LSG  G +        ++GL+STVTG L
Sbjct  376  PLAPLTNVVSTVTGA----LSGATGGATSTSPLAPITGLVSTVTGAL  418


>ref|WP_041219129.1| membrane protein [Burkholderia pseudomallei]
Length=537

 Score = 39.3 bits (90),  Expect = 3.8, Method: Compositional matrix adjust.
 Identities = 56/167 (34%), Positives = 87/167 (52%), Gaps = 20/167 (12%)

Query  87   TDGLTSTVTGLTSALTGDQISTITSALDDIPLVGQPLHDLLETVLELVNQLG--LANVAS  144
            T  ++S +T +TS +TG      T  + ++  +G P++ LL T+   +NQ G  ++   +
Sbjct  262  TQTVSSAITPITSMVTGT-----TQTVGNVTGLGAPVNTLLGTIGGGLNQAGALISKTGN  316

Query  145  GALASGASGSSSGLGGLL---GGLLGGGASSATNLQNIITQVVSGLSSVSSGVSGASGST  201
              + +G   + S  G  +   GGLL GG S ATN    IT  V GL+   SGVSGA+  T
Sbjct  317  NPVTTGLGQTVSATGNTITSVGGLLTGG-SGATNPLAPITAAVGGLTGTLSGVSGATSGT  375

Query  202  GLNAVTTILDTLKDSTDTNLSGLLGNSNKI-----VSGLLSTVTGLL  243
             L  +T ++ T+  +    LSG  G +        ++GL+STVTG L
Sbjct  376  PLAPLTNVVSTVTGA----LSGATGGATSTSPLAPITGLVSTVTGAL  418


>ref|WP_038792516.1| membrane protein [Burkholderia pseudomallei]
Length=538

 Score = 39.3 bits (90),  Expect = 3.8, Method: Compositional matrix adjust.
 Identities = 56/167 (34%), Positives = 87/167 (52%), Gaps = 20/167 (12%)

Query  87   TDGLTSTVTGLTSALTGDQISTITSALDDIPLVGQPLHDLLETVLELVNQLG--LANVAS  144
            T  ++S +T +TS +TG      T  + ++  +G P++ LL T+   +NQ G  ++   +
Sbjct  271  TQTVSSAITPITSMVTGT-----TQTVGNVTGLGAPVNTLLGTIGGGLNQAGALISKTGN  325

Query  145  GALASGASGSSSGLGGLL---GGLLGGGASSATNLQNIITQVVSGLSSVSSGVSGASGST  201
              + +G   + S  G  +   GGLL GG S ATN    IT  V GL+   SGVSGA+  T
Sbjct  326  NPVTTGLGQTVSATGNTITSVGGLLTGG-SGATNPLAPITAAVGGLTGTLSGVSGATSGT  384

Query  202  GLNAVTTILDTLKDSTDTNLSGLLGNSNKI-----VSGLLSTVTGLL  243
             L  +T ++ T+  +    LSG  G +        ++GL+STVTG L
Sbjct  385  PLAPLTNVVSTVTGA----LSGATGGATSTSPLAPITGLVSTVTGAL  427


>ref|WP_050043010.1| hypothetical protein [Burkholderia pseudomallei]
Length=524

 Score = 39.3 bits (90),  Expect = 3.8, Method: Compositional matrix adjust.
 Identities = 59/172 (34%), Positives = 90/172 (52%), Gaps = 21/172 (12%)

Query  87   TDGLTSTVTGLTSALTGDQISTITSALDDIPLVGQPLHDLLETVLELVNQLG--LANVAS  144
            T  ++S +T +TS +TG      T  + ++  +G P++ LL T+   +NQ G  ++   +
Sbjct  253  TQTVSSAITPITSMVTGT-----TQTVGNVTGLGAPVNTLLGTIGGGLNQAGALISKTGN  307

Query  145  GALASGASGSSSGLGGLL---GGLLGGGASSATNLQNIITQVVSGLSSVSSGVSGASGST  201
              + +G   + S  G  +   GGLL GG S ATN    IT  V GL+   SGVSGA+  T
Sbjct  308  NPVTTGLGQTVSATGNTITSVGGLLTGG-SGATNPLAPITAAVGGLTGTLSGVSGATSGT  366

Query  202  GLNAVTTILDTLKDSTDTNLSGLLGNSNKI-----VSGLLSTVTGLL-GANG  247
             L  +T ++ T+  +    LSG  G +        ++GL+STVTG L GA G
Sbjct  367  PLAPLTNVVSTVTGA----LSGATGGATSTSPLAPITGLVSTVTGALSGATG  414


>ref|WP_023360518.1| hypothetical protein [Burkholderia pseudomallei]
 gb|AGZ29458.1| putative transmembrane protein [Burkholderia pseudomallei NCTC 
13179]
Length=542

 Score = 39.3 bits (90),  Expect = 3.8, Method: Compositional matrix adjust.
 Identities = 56/167 (34%), Positives = 87/167 (52%), Gaps = 20/167 (12%)

Query  87   TDGLTSTVTGLTSALTGDQISTITSALDDIPLVGQPLHDLLETVLELVNQLG--LANVAS  144
            T  ++S +T +TS +TG      T  + ++  +G P++ LL T+   +NQ G  ++   +
Sbjct  271  TQTVSSAITPITSMVTGT-----TQTVGNVTGLGAPVNTLLGTIGGGLNQAGALISKTGN  325

Query  145  GALASGASGSSSGLGGLL---GGLLGGGASSATNLQNIITQVVSGLSSVSSGVSGASGST  201
              + +G   + S  G  +   GGLL GG S ATN    IT  V GL+   SGVSGA+  T
Sbjct  326  NPVTTGLGQTVSATGNTITSVGGLLTGG-SGATNPLAPITAAVGGLTGTLSGVSGATSGT  384

Query  202  GLNAVTTILDTLKDSTDTNLSGLLGNSNKI-----VSGLLSTVTGLL  243
             L  +T ++ T+  +    LSG  G +        ++GL+STVTG L
Sbjct  385  PLAPLTNVVSTVTGA----LSGATGGATSTSPLAPITGLVSTVTGAL  427


>ref|WP_038795520.1| membrane protein [Burkholderia pseudomallei]
Length=543

 Score = 39.3 bits (90),  Expect = 3.8, Method: Compositional matrix adjust.
 Identities = 56/167 (34%), Positives = 87/167 (52%), Gaps = 20/167 (12%)

Query  87   TDGLTSTVTGLTSALTGDQISTITSALDDIPLVGQPLHDLLETVLELVNQLG--LANVAS  144
            T  ++S +T +TS +TG      T  + ++  +G P++ LL T+   +NQ G  ++   +
Sbjct  272  TQTVSSAITPITSMVTGT-----TQTVGNVTGLGAPVNTLLGTIGGGLNQAGALISKTGN  326

Query  145  GALASGASGSSSGLGGLL---GGLLGGGASSATNLQNIITQVVSGLSSVSSGVSGASGST  201
              + +G   + S  G  +   GGLL GG S ATN    IT  V GL+   SGVSGA+  T
Sbjct  327  NPVTTGLGQTVSATGNTITSVGGLLTGG-SGATNPLAPITAAVGGLTGTLSGVSGATSGT  385

Query  202  GLNAVTTILDTLKDSTDTNLSGLLGNSNKI-----VSGLLSTVTGLL  243
             L  +T ++ T+  +    LSG  G +        ++GL+STVTG L
Sbjct  386  PLAPLTNVVSTVTGA----LSGATGGATSTSPLAPITGLVSTVTGAL  428


>ref|WP_004552870.1| hypothetical protein [Burkholderia pseudomallei]
 gb|EIF65887.1| hypothetical protein BP1026A_1000 [Burkholderia pseudomallei 
1026a]
 gb|AFI66487.1| hypothetical protein BP1026B_I1867 [Burkholderia pseudomallei 
1026b]
 gb|AJX08592.1| putative transmembrane protein [Burkholderia pseudomallei 1026b]
 emb|CFT76463.1| lipoprotein [Burkholderia pseudomallei]
Length=529

 Score = 39.3 bits (90),  Expect = 3.8, Method: Compositional matrix adjust.
 Identities = 59/172 (34%), Positives = 90/172 (52%), Gaps = 21/172 (12%)

Query  87   TDGLTSTVTGLTSALTGDQISTITSALDDIPLVGQPLHDLLETVLELVNQLG--LANVAS  144
            T  ++S +T +TS +TG      T  + ++  +G P++ LL T+   +NQ G  ++   +
Sbjct  262  TQTVSSAITPITSMVTGT-----TQTVGNVTGLGAPVNTLLGTIGGGLNQAGALISKTGN  316

Query  145  GALASGASGSSSGLGGLL---GGLLGGGASSATNLQNIITQVVSGLSSVSSGVSGASGST  201
              + +G   + S  G  +   GGLL GG S ATN    IT  V GL+   SGVSGA+  T
Sbjct  317  NPVTTGLGQTVSATGNTITSVGGLLTGG-SGATNPLAPITAAVGGLTGTLSGVSGATSGT  375

Query  202  GLNAVTTILDTLKDSTDTNLSGLLGNSNKI-----VSGLLSTVTGLL-GANG  247
             L  +T ++ T+  +    LSG  G +        ++GL+STVTG L GA G
Sbjct  376  PLAPLTNVVSTVTGA----LSGATGGATSTSPLAPITGLVSTVTGALSGATG  423


>ref|WP_038739045.1| membrane protein [Burkholderia sp. MSHR44]
Length=532

 Score = 39.3 bits (90),  Expect = 3.8, Method: Compositional matrix adjust.
 Identities = 59/172 (34%), Positives = 90/172 (52%), Gaps = 21/172 (12%)

Query  87   TDGLTSTVTGLTSALTGDQISTITSALDDIPLVGQPLHDLLETVLELVNQLG--LANVAS  144
            T  ++S +T +TS +TG      T  + ++  +G P++ LL T+   +NQ G  ++   +
Sbjct  265  TQTVSSAITPITSMVTGT-----TQTVGNVTGLGAPVNTLLGTIGGGLNQAGALISKTGN  319

Query  145  GALASGASGSSSGLGGLL---GGLLGGGASSATNLQNIITQVVSGLSSVSSGVSGASGST  201
              + +G   + S  G  +   GGLL GG S ATN    IT  V GL+   SGVSGA+  T
Sbjct  320  NPVTTGLGQTVSATGNTITSVGGLLTGG-SGATNPLAPITAAVGGLTGTLSGVSGATSGT  378

Query  202  GLNAVTTILDTLKDSTDTNLSGLLGNSNKI-----VSGLLSTVTGLL-GANG  247
             L  +T ++ T+  +    LSG  G +        ++GL+STVTG L GA G
Sbjct  379  PLAPLTNVVSTVTGA----LSGATGGATSTSPLAPITGLVSTVTGALSGATG  426


>ref|WP_038753270.1| membrane protein [Burkholderia pseudomallei]
Length=533

 Score = 39.3 bits (90),  Expect = 3.8, Method: Compositional matrix adjust.
 Identities = 59/172 (34%), Positives = 90/172 (52%), Gaps = 21/172 (12%)

Query  87   TDGLTSTVTGLTSALTGDQISTITSALDDIPLVGQPLHDLLETVLELVNQLG--LANVAS  144
            T  ++S +T +TS +TG      T  + ++  +G P++ LL T+   +NQ G  ++   +
Sbjct  262  TQTVSSAITPITSMVTGT-----TQTVGNVTGLGAPVNTLLGTIGGGLNQAGALISKTGN  316

Query  145  GALASGASGSSSGLGGLL---GGLLGGGASSATNLQNIITQVVSGLSSVSSGVSGASGST  201
              + +G   + S  G  +   GGLL GG S ATN    IT  V GL+   SGVSGA+  T
Sbjct  317  NPVTTGLGQTVSATGNTITSVGGLLTGG-SGATNPLAPITAAVGGLTGTLSGVSGATSGT  375

Query  202  GLNAVTTILDTLKDSTDTNLSGLLGNSNKI-----VSGLLSTVTGLL-GANG  247
             L  +T ++ T+  +    LSG  G +        ++GL+STVTG L GA G
Sbjct  376  PLAPLTNVVSTVTGA----LSGATGGATSTSPLAPITGLVSTVTGALSGATG  423


>ref|WP_024430414.1| membrane protein [Burkholderia pseudomallei]
 gb|AHE26092.1| putative transmembrane protein [Burkholderia pseudomallei NCTC 
13178]
 gb|KEO67512.1| membrane protein [Burkholderia pseudomallei MSHR5855]
 emb|CFL38409.1| lipoprotein [Burkholderia pseudomallei]
 emb|CFV84456.1| lipoprotein [Burkholderia pseudomallei]
 emb|CPG89326.1| lipoprotein [Burkholderia pseudomallei]
 emb|CPF83755.1| lipoprotein [Burkholderia pseudomallei]
Length=533

 Score = 39.3 bits (90),  Expect = 3.8, Method: Compositional matrix adjust.
 Identities = 59/172 (34%), Positives = 90/172 (52%), Gaps = 21/172 (12%)

Query  87   TDGLTSTVTGLTSALTGDQISTITSALDDIPLVGQPLHDLLETVLELVNQLG--LANVAS  144
            T  ++S +T +TS +TG      T  + ++  +G P++ LL T+   +NQ G  ++   +
Sbjct  262  TQTVSSAITPITSMVTGT-----TQTVGNVTGLGAPVNTLLGTIGGGLNQAGALISKTGN  316

Query  145  GALASGASGSSSGLGGLL---GGLLGGGASSATNLQNIITQVVSGLSSVSSGVSGASGST  201
              + +G   + S  G  +   GGLL GG S ATN    IT  V GL+   SGVSGA+  T
Sbjct  317  NPVTTGLGQTVSATGNTITSVGGLLTGG-SGATNPLAPITAAVGGLTGTLSGVSGATSGT  375

Query  202  GLNAVTTILDTLKDSTDTNLSGLLGNSNKI-----VSGLLSTVTGLL-GANG  247
             L  +T ++ T+  +    LSG  G +        ++GL+STVTG L GA G
Sbjct  376  PLAPLTNVVSTVTGA----LSGATGGATSTSPLAPITGLVSTVTGALSGATG  423


>ref|WP_038766399.1| membrane protein [Burkholderia pseudomallei]
 gb|KGD59666.1| putative transmembrane protein [Burkholderia pseudomallei]
Length=536

 Score = 39.3 bits (90),  Expect = 3.8, Method: Compositional matrix adjust.
 Identities = 59/172 (34%), Positives = 90/172 (52%), Gaps = 21/172 (12%)

Query  87   TDGLTSTVTGLTSALTGDQISTITSALDDIPLVGQPLHDLLETVLELVNQLG--LANVAS  144
            T  ++S +T +TS +TG      T  + ++  +G P++ LL T+   +NQ G  ++   +
Sbjct  265  TQTVSSAITPITSMVTGT-----TQTVGNVTGLGAPVNTLLGTIGGGLNQAGALISKTGN  319

Query  145  GALASGASGSSSGLGGLL---GGLLGGGASSATNLQNIITQVVSGLSSVSSGVSGASGST  201
              + +G   + S  G  +   GGLL GG S ATN    IT  V GL+   SGVSGA+  T
Sbjct  320  NPVTTGLGQTVSATGNTITSVGGLLTGG-SGATNPLAPITAAVGGLTGTLSGVSGATSGT  378

Query  202  GLNAVTTILDTLKDSTDTNLSGLLGNSNKI-----VSGLLSTVTGLL-GANG  247
             L  +T ++ T+  +    LSG  G +        ++GL+STVTG L GA G
Sbjct  379  PLAPLTNVVSTVTGA----LSGATGGATSTSPLAPITGLVSTVTGALSGATG  426


>ref|WP_038757948.1| membrane protein [Burkholderia pseudomallei]
Length=537

 Score = 39.3 bits (90),  Expect = 3.8, Method: Compositional matrix adjust.
 Identities = 59/172 (34%), Positives = 90/172 (52%), Gaps = 21/172 (12%)

Query  87   TDGLTSTVTGLTSALTGDQISTITSALDDIPLVGQPLHDLLETVLELVNQLG--LANVAS  144
            T  ++S +T +TS +TG      T  + ++  +G P++ LL T+   +NQ G  ++   +
Sbjct  262  TQTVSSAITPITSMVTGT-----TQTVGNVTGLGAPVNTLLGTIGGGLNQAGALISKTGN  316

Query  145  GALASGASGSSSGLGGLL---GGLLGGGASSATNLQNIITQVVSGLSSVSSGVSGASGST  201
              + +G   + S  G  +   GGLL GG S ATN    IT  V GL+   SGVSGA+  T
Sbjct  317  NPVTTGLGQTVSATGNTITSVGGLLTGG-SGATNPLAPITAAVGGLTGTLSGVSGATSGT  375

Query  202  GLNAVTTILDTLKDSTDTNLSGLLGNSNKI-----VSGLLSTVTGLL-GANG  247
             L  +T ++ T+  +    LSG  G +        ++GL+STVTG L GA G
Sbjct  376  PLAPLTNVVSTVTGA----LSGATGGATSTSPLAPITGLVSTVTGALSGATG  423


>ref|WP_038729505.1| membrane protein [Burkholderia pseudomallei]
 gb|KKC13459.1| putative transmembrane protein [Burkholderia pseudomallei MSHR1328]
Length=540

 Score = 39.3 bits (90),  Expect = 3.8, Method: Compositional matrix adjust.
 Identities = 59/172 (34%), Positives = 90/172 (52%), Gaps = 21/172 (12%)

Query  87   TDGLTSTVTGLTSALTGDQISTITSALDDIPLVGQPLHDLLETVLELVNQLG--LANVAS  144
            T  ++S +T +TS +TG      T  + ++  +G P++ LL T+   +NQ G  ++   +
Sbjct  273  TQTVSSAITPITSMVTGT-----TQTVGNVTGLGAPVNTLLGTIGGGLNQAGALISKTGN  327

Query  145  GALASGASGSSSGLGGLL---GGLLGGGASSATNLQNIITQVVSGLSSVSSGVSGASGST  201
              + +G   + S  G  +   GGLL GG S ATN    IT  V GL+   SGVSGA+  T
Sbjct  328  NPVTTGLGQTVSATGNTITSVGGLLTGG-SGATNPLAPITAAVGGLTGTLSGVSGATSGT  386

Query  202  GLNAVTTILDTLKDSTDTNLSGLLGNSNKI-----VSGLLSTVTGLL-GANG  247
             L  +T ++ T+  +    LSG  G +        ++GL+STVTG L GA G
Sbjct  387  PLAPLTNVVSTVTGA----LSGATGGATSTSPLAPITGLVSTVTGALSGATG  434


>ref|WP_020850685.1| hypothetical protein [Burkholderia pseudomallei]
 gb|AGR72313.1| putative transmembrane protein [Burkholderia pseudomallei MSHR305]
 gb|AHK65360.1| putative transmembrane protein [Burkholderia pseudomallei MSHR520]
Length=502

 Score = 39.3 bits (90),  Expect = 3.9, Method: Compositional matrix adjust.
 Identities = 56/167 (34%), Positives = 87/167 (52%), Gaps = 20/167 (12%)

Query  87   TDGLTSTVTGLTSALTGDQISTITSALDDIPLVGQPLHDLLETVLELVNQLG--LANVAS  144
            T  ++S +T +TS +TG      T  + ++  +G P++ LL T+   +NQ G  ++   +
Sbjct  262  TQTVSSAITPITSMVTGT-----TQTVGNVTGLGAPVNTLLGTIGGGLNQAGALISKTGN  316

Query  145  GALASGASGSSSGLGGLL---GGLLGGGASSATNLQNIITQVVSGLSSVSSGVSGASGST  201
              + +G   + S  G  +   GGLL GG S ATN    IT  V GL+   SGVSGA+  T
Sbjct  317  NPVTTGLGQTVSATGNTITSVGGLLTGG-SGATNPLAPITAAVGGLTGTLSGVSGATSGT  375

Query  202  GLNAVTTILDTLKDSTDTNLSGLLGNSNKI-----VSGLLSTVTGLL  243
             L  +T ++ T+  +    LSG  G +        ++GL+STVTG L
Sbjct  376  PLAPLTNVVSTVTGA----LSGATGGATSTSPLAPITGLVSTVTGAL  418


>ref|WP_038789791.1| membrane protein [Burkholderia pseudomallei]
Length=532

 Score = 39.3 bits (90),  Expect = 3.9, Method: Compositional matrix adjust.
 Identities = 56/167 (34%), Positives = 87/167 (52%), Gaps = 20/167 (12%)

Query  87   TDGLTSTVTGLTSALTGDQISTITSALDDIPLVGQPLHDLLETVLELVNQLG--LANVAS  144
            T  ++S +T +TS +TG      T  + ++  +G P++ LL T+   +NQ G  ++   +
Sbjct  265  TQTVSSAITPITSMVTGT-----TQTVGNVTGLGAPVNTLLGTIGGGLNQAGALISKTGN  319

Query  145  GALASGASGSSSGLGGLL---GGLLGGGASSATNLQNIITQVVSGLSSVSSGVSGASGST  201
              + +G   + S  G  +   GGLL GG S ATN    IT  V GL+   SGVSGA+  T
Sbjct  320  NPVTTGLGQTVSATGNTITSVGGLLTGG-SGATNPLAPITAAVGGLTGTLSGVSGATSGT  378

Query  202  GLNAVTTILDTLKDSTDTNLSGLLGNSNKI-----VSGLLSTVTGLL  243
             L  +T ++ T+  +    LSG  G +        ++GL+STVTG L
Sbjct  379  PLAPLTNVVSTVTGA----LSGATGGATSTSPLAPITGLVSTVTGAL  421


>gb|KGU98613.1| hypothetical protein X885_3206 [Burkholderia pseudomallei MSHR4372]
Length=510

 Score = 39.3 bits (90),  Expect = 4.0, Method: Compositional matrix adjust.
 Identities = 56/167 (34%), Positives = 87/167 (52%), Gaps = 20/167 (12%)

Query  87   TDGLTSTVTGLTSALTGDQISTITSALDDIPLVGQPLHDLLETVLELVNQLG--LANVAS  144
            T  ++S +T +TS +TG      T  + ++  +G P++ LL T+   +NQ G  ++   +
Sbjct  243  TQTVSSAITPITSMVTGT-----TQTVGNVTGLGAPVNTLLGTIGGGLNQAGALISKTGN  297

Query  145  GALASGASGSSSGLGGLL---GGLLGGGASSATNLQNIITQVVSGLSSVSSGVSGASGST  201
              + +G   + S  G  +   GGLL GG S ATN    IT  V GL+   SGVSGA+  T
Sbjct  298  NPVTTGLGQTVSATGNTITSVGGLLTGG-SGATNPLAPITAAVGGLTGTLSGVSGATSGT  356

Query  202  GLNAVTTILDTLKDSTDTNLSGLLGNSNKI-----VSGLLSTVTGLL  243
             L  +T ++ T+  +    LSG  G +        ++GL+STVTG L
Sbjct  357  PLAPLTNVVSTVTGA----LSGATGGATSTSPLAPITGLVSTVTGAL  399


>ref|WP_038765304.1| membrane protein [Burkholderia pseudomallei]
 emb|CDU28234.1| putative membrane protein [Burkholderia pseudomallei]
 gb|KGW10062.1| putative transmembrane protein [Burkholderia pseudomallei TSV 
25]
 71 more sequence titles

gb|KIX57986.1| membrane protein [Burkholderia pseudomallei]
 emb|CFM57997.1| lipoprotein [Burkholderia pseudomallei]
 emb|CFM80457.1| lipoprotein [Burkholderia pseudomallei]
 emb|CFN15999.1| lipoprotein [Burkholderia pseudomallei]
 emb|CFO50701.1| lipoprotein [Burkholderia pseudomallei]
 emb|CFO67625.1| lipoprotein [Burkholderia pseudomallei]
 emb|CFO88266.1| lipoprotein [Burkholderia pseudomallei]
 emb|CFP06378.1| lipoprotein [Burkholderia pseudomallei]
 emb|CFL95426.1| lipoprotein [Burkholderia pseudomallei]
 emb|CFM09340.1| lipoprotein [Burkholderia pseudomallei]
 emb|CFM31946.1| lipoprotein [Burkholderia pseudomallei]
 emb|CFM63371.1| lipoprotein [Burkholderia pseudomallei]
 emb|CFM96339.1| lipoprotein [Burkholderia pseudomallei]
 emb|CFN30682.1| lipoprotein [Burkholderia pseudomallei]
 emb|CFO08576.1| lipoprotein [Burkholderia pseudomallei]
 emb|CFP24317.1| lipoprotein [Burkholderia pseudomallei]
 emb|CFU15804.1| lipoprotein [Burkholderia pseudomallei]
 emb|CFU27249.1| lipoprotein [Burkholderia pseudomallei]
 emb|CFU52096.1| lipoprotein [Burkholderia pseudomallei]
 emb|CFU64930.1| lipoprotein [Burkholderia pseudomallei]
 emb|CFU73295.1| lipoprotein [Burkholderia pseudomallei]
 emb|CFW14492.1| lipoprotein [Burkholderia pseudomallei]
 emb|CFW46878.1| lipoprotein [Burkholderia pseudomallei]
 emb|CFW79413.1| lipoprotein [Burkholderia pseudomallei]
 emb|CPQ79211.1| lipoprotein [Burkholderia pseudomallei]
 emb|CPK19080.1| lipoprotein [Burkholderia pseudomallei]
 emb|CPP51567.1| lipoprotein [Burkholderia pseudomallei]
 emb|CPK91094.1| lipoprotein [Burkholderia pseudomallei]
 emb|CPN88817.1| lipoprotein [Burkholderia pseudomallei]
 emb|CPM78221.1| lipoprotein [Burkholderia pseudomallei]
 emb|CPN05965.1| lipoprotein [Burkholderia pseudomallei]
 emb|CPK61922.1| lipoprotein [Burkholderia pseudomallei]
 emb|CPP00628.1| lipoprotein [Burkholderia pseudomallei]
 emb|CPK37087.1| lipoprotein [Burkholderia pseudomallei]
 emb|CPK23037.1| lipoprotein [Burkholderia pseudomallei]
 emb|CPO09030.1| lipoprotein [Burkholderia pseudomallei]
 emb|CPM56891.1| lipoprotein [Burkholderia pseudomallei]
 emb|CRY16291.1| lipoprotein [Burkholderia pseudomallei]
 emb|CPL13811.1| lipoprotein [Burkholderia pseudomallei]
 emb|CPJ24072.1| lipoprotein [Burkholderia pseudomallei]
 emb|CPH78415.1| lipoprotein [Burkholderia pseudomallei]
 emb|CPM86741.1| lipoprotein [Burkholderia pseudomallei]
 emb|CPI73692.1| lipoprotein [Burkholderia pseudomallei]
 emb|CPP49244.1| lipoprotein [Burkholderia pseudomallei]
 emb|CPN82954.1| lipoprotein [Burkholderia pseudomallei]
 emb|CPK39812.1| lipoprotein [Burkholderia pseudomallei]
 emb|CPN59011.1| lipoprotein [Burkholderia pseudomallei]
 emb|CPM97656.1| lipoprotein [Burkholderia pseudomallei]
 emb|CPM02821.1| lipoprotein [Burkholderia pseudomallei]
 emb|CPL19913.1| lipoprotein [Burkholderia pseudomallei]
 emb|CPJ42716.1| lipoprotein [Burkholderia pseudomallei]
 emb|CPQ92915.1| lipoprotein [Burkholderia pseudomallei]
 emb|CPH65675.1| lipoprotein [Burkholderia pseudomallei]
 emb|CPQ48886.1| lipoprotein [Burkholderia pseudomallei]
 emb|CPJ37742.1| lipoprotein [Burkholderia pseudomallei]
 emb|CPP88338.1| lipoprotein [Burkholderia pseudomallei]
 emb|CPP65569.1| lipoprotein [Burkholderia pseudomallei]
 emb|CPK49633.1| lipoprotein [Burkholderia pseudomallei]
 emb|CPP93049.1| lipoprotein [Burkholderia pseudomallei]
 emb|CPH98938.1| lipoprotein [Burkholderia pseudomallei]
 emb|CPJ11698.1| lipoprotein [Burkholderia pseudomallei]
 emb|CPM84472.1| lipoprotein [Burkholderia pseudomallei]
 emb|CPL42642.1| lipoprotein [Burkholderia pseudomallei]
 emb|CPN59555.1| lipoprotein [Burkholderia pseudomallei]
 emb|CPM16559.1| lipoprotein [Burkholderia pseudomallei]
 emb|CPQ47378.1| lipoprotein [Burkholderia pseudomallei]
 emb|CPK48141.1| lipoprotein [Burkholderia pseudomallei]
 emb|CPM24295.1| lipoprotein [Burkholderia pseudomallei]
 emb|CPN48190.1| lipoprotein [Burkholderia pseudomallei]
 emb|CPP04680.1| lipoprotein [Burkholderia pseudomallei]
 emb|CPO27271.1| lipoprotein [Burkholderia pseudomallei]

Length=529

 Score = 39.3 bits (90),  Expect = 4.0, Method: Compositional matrix adjust.
 Identities = 56/167 (34%), Positives = 87/167 (52%), Gaps = 20/167 (12%)

Query  87   TDGLTSTVTGLTSALTGDQISTITSALDDIPLVGQPLHDLLETVLELVNQLG--LANVAS  144
            T  ++S +T +TS +TG      T  + ++  +G P++ LL T+   +NQ G  ++   +
Sbjct  262  TQTVSSAITPITSMVTGT-----TQTVGNVTGLGAPVNTLLGTIGGGLNQAGALISKTGN  316

Query  145  GALASGASGSSSGLGGLL---GGLLGGGASSATNLQNIITQVVSGLSSVSSGVSGASGST  201
              + +G   + S  G  +   GGLL GG S ATN    IT  V GL+   SGVSGA+  T
Sbjct  317  NPVTTGLGQTVSATGNTITSVGGLLTGG-SGATNPLAPITAAVGGLTGTLSGVSGATSGT  375

Query  202  GLNAVTTILDTLKDSTDTNLSGLLGNSNKI-----VSGLLSTVTGLL  243
             L  +T ++ T+  +    LSG  G +        ++GL+STVTG L
Sbjct  376  PLAPLTNVVSTVTGA----LSGATGGATSTSPLAPITGLVSTVTGAL  418


>ref|WP_004521663.1| membrane protein [Burkholderia pseudomallei]
 gb|EDU07546.1| conserved hypothetical protein [Burkholderia pseudomallei 1655]
Length=529

 Score = 39.3 bits (90),  Expect = 4.0, Method: Compositional matrix adjust.
 Identities = 56/167 (34%), Positives = 87/167 (52%), Gaps = 20/167 (12%)

Query  87   TDGLTSTVTGLTSALTGDQISTITSALDDIPLVGQPLHDLLETVLELVNQLG--LANVAS  144
            T  ++S +T +TS +TG      T  + ++  +G P++ LL T+   +NQ G  ++   +
Sbjct  262  TQTVSSAITPITSMVTGT-----TQTVGNVTGLGAPVNTLLGTIGGGLNQAGALISKTGN  316

Query  145  GALASGASGSSSGLGGLL---GGLLGGGASSATNLQNIITQVVSGLSSVSSGVSGASGST  201
              + +G   + S  G  +   GGLL GG S ATN    IT  V GL+   SGVSGA+  T
Sbjct  317  NPVTTGLGQTVSATGNTITSVGGLLTGG-SGATNPLAPITAAVGGLTGTLSGVSGATSGT  375

Query  202  GLNAVTTILDTLKDSTDTNLSGLLGNSNKI-----VSGLLSTVTGLL  243
             L  +T ++ T+  +    LSG  G +        ++GL+STVTG L
Sbjct  376  PLAPLTNVVSTVTGA----LSGATGGATSTSPLAPITGLVSTVTGAL  418


>ref|WP_038771759.1| membrane protein [Burkholderia pseudomallei]
Length=533

 Score = 39.3 bits (90),  Expect = 4.0, Method: Compositional matrix adjust.
 Identities = 56/167 (34%), Positives = 87/167 (52%), Gaps = 20/167 (12%)

Query  87   TDGLTSTVTGLTSALTGDQISTITSALDDIPLVGQPLHDLLETVLELVNQLG--LANVAS  144
            T  ++S +T +TS +TG      T  + ++  +G P++ LL T+   +NQ G  ++   +
Sbjct  262  TQTVSSAITPITSMVTGT-----TQTVGNVTGLGAPVNTLLGTIGGGLNQAGALISKTGN  316

Query  145  GALASGASGSSSGLGGLL---GGLLGGGASSATNLQNIITQVVSGLSSVSSGVSGASGST  201
              + +G   + S  G  +   GGLL GG S ATN    IT  V GL+   SGVSGA+  T
Sbjct  317  NPVTTGLGQTVSATGNTITSVGGLLTGG-SGATNPLAPITAAVGGLTGTLSGVSGATSGT  375

Query  202  GLNAVTTILDTLKDSTDTNLSGLLGNSNKI-----VSGLLSTVTGLL  243
             L  +T ++ T+  +    LSG  G +        ++GL+STVTG L
Sbjct  376  PLAPLTNVVSTVTGA----LSGATGGATSTSPLAPITGLVSTVTGAL  418


>ref|WP_025986495.1| membrane protein [Burkholderia pseudomallei]
 gb|AHE35056.1| putative transmembrane protein [Burkholderia pseudomallei NAU20B-16]
 gb|AHG35294.1| putative transmembrane protein [Burkholderia pseudomallei MSHR511]
 gb|AHG66271.1| putative transmembrane protein [Burkholderia pseudomallei MSHR146]
 gb|KJR94720.1| membrane protein [Burkholderia pseudomallei]
Length=533

 Score = 39.3 bits (90),  Expect = 4.0, Method: Compositional matrix adjust.
 Identities = 56/167 (34%), Positives = 87/167 (52%), Gaps = 20/167 (12%)

Query  87   TDGLTSTVTGLTSALTGDQISTITSALDDIPLVGQPLHDLLETVLELVNQLG--LANVAS  144
            T  ++S +T +TS +TG      T  + ++  +G P++ LL T+   +NQ G  ++   +
Sbjct  262  TQTVSSAITPITSMVTGT-----TQTVGNVTGLGAPVNTLLGTIGGGLNQAGALISKTGN  316

Query  145  GALASGASGSSSGLGGLL---GGLLGGGASSATNLQNIITQVVSGLSSVSSGVSGASGST  201
              + +G   + S  G  +   GGLL GG S ATN    IT  V GL+   SGVSGA+  T
Sbjct  317  NPVTTGLGQTVSATGNTITSVGGLLTGG-SGATNPLAPITAAVGGLTGTLSGVSGATSGT  375

Query  202  GLNAVTTILDTLKDSTDTNLSGLLGNSNKI-----VSGLLSTVTGLL  243
             L  +T ++ T+  +    LSG  G +        ++GL+STVTG L
Sbjct  376  PLAPLTNVVSTVTGA----LSGATGGATSTSPLAPITGLVSTVTGAL  418


>ref|WP_024428749.1| membrane protein [Burkholderia pseudomallei]
 gb|ALB14914.1| membrane protein [Burkholderia pseudomallei]
Length=537

 Score = 39.3 bits (90),  Expect = 4.1, Method: Compositional matrix adjust.
 Identities = 56/167 (34%), Positives = 87/167 (52%), Gaps = 20/167 (12%)

Query  87   TDGLTSTVTGLTSALTGDQISTITSALDDIPLVGQPLHDLLETVLELVNQLG--LANVAS  144
            T  ++S +T +TS +TG      T  + ++  +G P++ LL T+   +NQ G  ++   +
Sbjct  262  TQTVSSAITPITSMVTGT-----TQTVGNVTGLGAPVNTLLGTIGGGLNQAGALISKTGN  316

Query  145  GALASGASGSSSGLGGLL---GGLLGGGASSATNLQNIITQVVSGLSSVSSGVSGASGST  201
              + +G   + S  G  +   GGLL GG S ATN    IT  V GL+   SGVSGA+  T
Sbjct  317  NPVTTGLGQTVSATGNTITSVGGLLTGG-SGATNPLAPITAAVGGLTGTLSGVSGATSGT  375

Query  202  GLNAVTTILDTLKDSTDTNLSGLLGNSNKI-----VSGLLSTVTGLL  243
             L  +T ++ T+  +    LSG  G +        ++GL+STVTG L
Sbjct  376  PLAPLTNVVSTVTGA----LSGATGGATSTSPLAPITGLVSTVTGAL  418


>ref|WP_004526863.1| MULTISPECIES: membrane protein [Burkholderia]
 gb|EDO91975.1| conserved hypothetical protein [Burkholderia pseudomallei Pasteur 
52237]
 gb|EET06243.1| conserved hypothetical protein [Burkholderia pseudomallei 1710a]
 gb|EIF64866.1| hypothetical protein BP1258A_1543 [Burkholderia pseudomallei 
1258a]
 gb|EIF67464.1| hypothetical protein BP1258B_1636 [Burkholderia pseudomallei 
1258b]
 gb|ALB95479.1| hypothetical protein AM256_08280 [Burkholderia pseudomallei]
 gb|ALC01545.1| hypothetical protein AM257_08290 [Burkholderia pseudomallei]
Length=537

 Score = 39.3 bits (90),  Expect = 4.1, Method: Compositional matrix adjust.
 Identities = 56/167 (34%), Positives = 87/167 (52%), Gaps = 20/167 (12%)

Query  87   TDGLTSTVTGLTSALTGDQISTITSALDDIPLVGQPLHDLLETVLELVNQLG--LANVAS  144
            T  ++S +T +TS +TG      T  + ++  +G P++ LL T+   +NQ G  ++   +
Sbjct  262  TQTVSSAITPITSMVTGT-----TQTVGNVTGLGAPVNTLLGTIGGGLNQAGALISKTGN  316

Query  145  GALASGASGSSSGLGGLL---GGLLGGGASSATNLQNIITQVVSGLSSVSSGVSGASGST  201
              + +G   + S  G  +   GGLL GG S ATN    IT  V GL+   SGVSGA+  T
Sbjct  317  NPVTTGLGQTVSATGNTITSVGGLLTGG-SGATNPLAPITAAVGGLTGTLSGVSGATSGT  375

Query  202  GLNAVTTILDTLKDSTDTNLSGLLGNSNKI-----VSGLLSTVTGLL  243
             L  +T ++ T+  +    LSG  G +        ++GL+STVTG L
Sbjct  376  PLAPLTNVVSTVTGA----LSGATGGATSTSPLAPITGLVSTVTGAL  418


>ref|WP_031313408.1| membrane protein [Burkholderia pseudomallei]
Length=538

 Score = 39.3 bits (90),  Expect = 4.1, Method: Compositional matrix adjust.
 Identities = 56/167 (34%), Positives = 87/167 (52%), Gaps = 20/167 (12%)

Query  87   TDGLTSTVTGLTSALTGDQISTITSALDDIPLVGQPLHDLLETVLELVNQLG--LANVAS  144
            T  ++S +T +TS +TG      T  + ++  +G P++ LL T+   +NQ G  ++   +
Sbjct  267  TQTVSSAITPITSMVTGT-----TQTVGNVTGLGAPVNTLLGTIGGGLNQAGALISKTGN  321

Query  145  GALASGASGSSSGLGGLL---GGLLGGGASSATNLQNIITQVVSGLSSVSSGVSGASGST  201
              + +G   + S  G  +   GGLL GG S ATN    IT  V GL+   SGVSGA+  T
Sbjct  322  NPVTTGLGQTVSATGNTITSVGGLLTGG-SGATNPLAPITAAVGGLTGTLSGVSGATSGT  380

Query  202  GLNAVTTILDTLKDSTDTNLSGLLGNSNKI-----VSGLLSTVTGLL  243
             L  +T ++ T+  +    LSG  G +        ++GL+STVTG L
Sbjct  381  PLAPLTNVVSTVTGA----LSGATGGATSTSPLAPITGLVSTVTGAL  423


>ref|WP_038775869.1| membrane protein [Burkholderia pseudomallei]
Length=542

 Score = 39.3 bits (90),  Expect = 4.1, Method: Compositional matrix adjust.
 Identities = 56/167 (34%), Positives = 87/167 (52%), Gaps = 20/167 (12%)

Query  87   TDGLTSTVTGLTSALTGDQISTITSALDDIPLVGQPLHDLLETVLELVNQLG--LANVAS  144
            T  ++S +T +TS +TG      T  + ++  +G P++ LL T+   +NQ G  ++   +
Sbjct  275  TQTVSSAITPITSMVTGT-----TQTVGNVTGLGAPVNTLLGTIGGGLNQAGALISKTGN  329

Query  145  GALASGASGSSSGLGGLL---GGLLGGGASSATNLQNIITQVVSGLSSVSSGVSGASGST  201
              + +G   + S  G  +   GGLL GG S ATN    IT  V GL+   SGVSGA+  T
Sbjct  330  NPVTTGLGQTVSATGNTITSVGGLLTGG-SGATNPLAPITAAVGGLTGTLSGVSGATSGT  388

Query  202  GLNAVTTILDTLKDSTDTNLSGLLGNSNKI-----VSGLLSTVTGLL  243
             L  +T ++ T+  +    LSG  G +        ++GL+STVTG L
Sbjct  389  PLAPLTNVVSTVTGA----LSGATGGATSTSPLAPITGLVSTVTGAL  431


>ref|WP_038718253.1| membrane protein [Burkholderia pseudomallei]
Length=533

 Score = 39.3 bits (90),  Expect = 4.2, Method: Compositional matrix adjust.
 Identities = 56/167 (34%), Positives = 87/167 (52%), Gaps = 20/167 (12%)

Query  87   TDGLTSTVTGLTSALTGDQISTITSALDDIPLVGQPLHDLLETVLELVNQLG--LANVAS  144
            T  ++S +T +TS +TG      T  + ++  +G P++ LL T+   +NQ G  ++   +
Sbjct  262  TQTVSSAITPITSMVTGT-----TQTVGNVTGLGAPVNTLLGTIGGGLNQAGALISKTGN  316

Query  145  GALASGASGSSSGLGGLL---GGLLGGGASSATNLQNIITQVVSGLSSVSSGVSGASGST  201
              + +G   + S  G  +   GGLL GG S ATN    IT  V GL+   SGVSGA+  T
Sbjct  317  NPVTTGLGQTVSATGNTITSVGGLLTGG-SGATNPLAPITAAVGGLTGTLSGVSGATSGT  375

Query  202  GLNAVTTILDTLKDSTDTNLSGLLGNSNKI-----VSGLLSTVTGLL  243
             L  +T ++ T+  +    LSG  G +        ++GL+STVTG L
Sbjct  376  PLAPLTNVVSTVTGA----LSGATGGATSTSPLAPITGLVSTVTGAL  418


>ref|WP_038768443.1| membrane protein [Burkholderia pseudomallei]
 gb|KGS50549.1| putative transmembrane protein [Burkholderia pseudomallei MSHR5613]
Length=544

 Score = 39.3 bits (90),  Expect = 4.2, Method: Compositional matrix adjust.
 Identities = 56/167 (34%), Positives = 87/167 (52%), Gaps = 20/167 (12%)

Query  87   TDGLTSTVTGLTSALTGDQISTITSALDDIPLVGQPLHDLLETVLELVNQLG--LANVAS  144
            T  ++S +T +TS +TG      T  + ++  +G P++ LL T+   +NQ G  ++   +
Sbjct  265  TQTVSSAITPITSMVTGT-----TQTVGNVTGLGAPVNTLLGTIGGGLNQAGALISKTGN  319

Query  145  GALASGASGSSSGLGGLL---GGLLGGGASSATNLQNIITQVVSGLSSVSSGVSGASGST  201
              + +G   + S  G  +   GGLL GG S ATN    IT  V GL+   SGVSGA+  T
Sbjct  320  NPVTTGLGQTVSATGNTITSVGGLLTGG-SGATNPLAPITAAVGGLTGTLSGVSGATSGT  378

Query  202  GLNAVTTILDTLKDSTDTNLSGLLGNSNKI-----VSGLLSTVTGLL  243
             L  +T ++ T+  +    LSG  G +        ++GL+STVTG L
Sbjct  379  PLAPLTNVVSTVTGA----LSGATGGTTSTSPLAPITGLVSTVTGAL  421


>gb|AJX36279.1| hypothetical protein DP45_02490 [Burkholderia pseudomallei]
Length=511

 Score = 39.3 bits (90),  Expect = 4.2, Method: Compositional matrix adjust.
 Identities = 56/167 (34%), Positives = 87/167 (52%), Gaps = 20/167 (12%)

Query  87   TDGLTSTVTGLTSALTGDQISTITSALDDIPLVGQPLHDLLETVLELVNQLG--LANVAS  144
            T  ++S +T +TS +TG      T  + ++  +G P++ LL T+   +NQ G  ++   +
Sbjct  240  TQTVSSAITPITSMVTGT-----TQTVGNVTGLGAPVNTLLGTIGGGLNQAGALISKTGN  294

Query  145  GALASGASGSSSGLGGLL---GGLLGGGASSATNLQNIITQVVSGLSSVSSGVSGASGST  201
              + +G   + S  G  +   GGLL GG S ATN    IT  V GL+   SGVSGA+  T
Sbjct  295  NPVTTGLGQTVSATGNTITSVGGLLTGG-SGATNPLAPITAAVGGLTGTLSGVSGATSGT  353

Query  202  GLNAVTTILDTLKDSTDTNLSGLLGNSNKI-----VSGLLSTVTGLL  243
             L  +T ++ T+  +    LSG  G +        ++GL+STVTG L
Sbjct  354  PLAPLTNVVSTVTGA----LSGATGGATSTSPLAPITGLVSTVTGAL  396


>gb|KGU77041.1| putative transmembrane protein [Burkholderia pseudomallei MSHR543]
 gb|KGW63854.1| putative transmembrane protein [Burkholderia pseudomallei MSHR1029]
 gb|AJX82949.1| putative transmembrane protein [Burkholderia pseudomallei 7894]
 gb|AJX28138.1| putative transmembrane protein [Burkholderia pseudomallei K96243]
Length=507

 Score = 39.3 bits (90),  Expect = 4.3, Method: Compositional matrix adjust.
 Identities = 56/167 (34%), Positives = 87/167 (52%), Gaps = 20/167 (12%)

Query  87   TDGLTSTVTGLTSALTGDQISTITSALDDIPLVGQPLHDLLETVLELVNQLG--LANVAS  144
            T  ++S +T +TS +TG      T  + ++  +G P++ LL T+   +NQ G  ++   +
Sbjct  240  TQTVSSAITPITSMVTGT-----TQTVGNVTGLGAPVNTLLGTIGGGLNQAGALISKTGN  294

Query  145  GALASGASGSSSGLGGLL---GGLLGGGASSATNLQNIITQVVSGLSSVSSGVSGASGST  201
              + +G   + S  G  +   GGLL GG S ATN    IT  V GL+   SGVSGA+  T
Sbjct  295  NPVTTGLGQTVSATGNTITSVGGLLTGG-SGATNPLAPITAAVGGLTGTLSGVSGATSGT  353

Query  202  GLNAVTTILDTLKDSTDTNLSGLLGNSNKI-----VSGLLSTVTGLL  243
             L  +T ++ T+  +    LSG  G +        ++GL+STVTG L
Sbjct  354  PLAPLTNVVSTVTGA----LSGATGGATSTSPLAPITGLVSTVTGAL  396


>gb|AIV48230.1| hypothetical protein X988_1416 [Burkholderia pseudomallei TSV 
48]
Length=507

 Score = 39.3 bits (90),  Expect = 4.3, Method: Compositional matrix adjust.
 Identities = 56/167 (34%), Positives = 87/167 (52%), Gaps = 20/167 (12%)

Query  87   TDGLTSTVTGLTSALTGDQISTITSALDDIPLVGQPLHDLLETVLELVNQLG--LANVAS  144
            T  ++S +T +TS +TG      T  + ++  +G P++ LL T+   +NQ G  ++   +
Sbjct  240  TQTVSSAITPITSMVTGT-----TQTVGNVTGLGAPVNTLLGTIGGGLNQAGALISKTGN  294

Query  145  GALASGASGSSSGLGGLL---GGLLGGGASSATNLQNIITQVVSGLSSVSSGVSGASGST  201
              + +G   + S  G  +   GGLL GG S ATN    IT  V GL+   SGVSGA+  T
Sbjct  295  NPVTTGLGQTVSATGNTITSVGGLLTGG-SGATNPLAPITAAVGGLTGTLSGVSGATSGT  353

Query  202  GLNAVTTILDTLKDSTDTNLSGLLGNSNKI-----VSGLLSTVTGLL  243
             L  +T ++ T+  +    LSG  G +        ++GL+STVTG L
Sbjct  354  PLAPLTNVVSTVTGA----LSGATGGATSTSPLAPITGLVSTVTGAL  396


>gb|AIP05596.1| putative transmembrane protein [Burkholderia pseudomallei]
 gb|KGS32343.1| hypothetical protein X989_298 [Burkholderia pseudomallei MSHR4378]
 gb|KGW41491.1| putative transmembrane protein [Burkholderia pseudomallei MSHR1000]
Length=507

 Score = 39.3 bits (90),  Expect = 4.3, Method: Compositional matrix adjust.
 Identities = 56/167 (34%), Positives = 87/167 (52%), Gaps = 20/167 (12%)

Query  87   TDGLTSTVTGLTSALTGDQISTITSALDDIPLVGQPLHDLLETVLELVNQLG--LANVAS  144
            T  ++S +T +TS +TG      T  + ++  +G P++ LL T+   +NQ G  ++   +
Sbjct  240  TQTVSSAITPITSMVTGT-----TQTVGNVTGLGAPVNTLLGTIGGGLNQAGALISKTGN  294

Query  145  GALASGASGSSSGLGGLL---GGLLGGGASSATNLQNIITQVVSGLSSVSSGVSGASGST  201
              + +G   + S  G  +   GGLL GG S ATN    IT  V GL+   SGVSGA+  T
Sbjct  295  NPVTTGLGQTVSATGNTITSVGGLLTGG-SGATNPLAPITAAVGGLTGTLSGVSGATSGT  353

Query  202  GLNAVTTILDTLKDSTDTNLSGLLGNSNKI-----VSGLLSTVTGLL  243
             L  +T ++ T+  +    LSG  G +        ++GL+STVTG L
Sbjct  354  PLAPLTNVVSTVTGA----LSGATGGATSTSPLAPITGLVSTVTGAL  396


>gb|KGW22311.1| putative transmembrane protein [Burkholderia pseudomallei MSHR733]
Length=510

 Score = 39.3 bits (90),  Expect = 4.3, Method: Compositional matrix adjust.
 Identities = 56/167 (34%), Positives = 87/167 (52%), Gaps = 20/167 (12%)

Query  87   TDGLTSTVTGLTSALTGDQISTITSALDDIPLVGQPLHDLLETVLELVNQLG--LANVAS  144
            T  ++S +T +TS +TG      T  + ++  +G P++ LL T+   +NQ G  ++   +
Sbjct  243  TQTVSSAITPITSMVTGT-----TQTVGNVTGLGAPVNTLLGTIGGGLNQAGALISKTGN  297

Query  145  GALASGASGSSSGLGGLL---GGLLGGGASSATNLQNIITQVVSGLSSVSSGVSGASGST  201
              + +G   + S  G  +   GGLL GG S ATN    IT  V GL+   SGVSGA+  T
Sbjct  298  NPVTTGLGQTVSATGNTITSVGGLLTGG-SGATNPLAPITAAVGGLTGTLSGVSGATSGT  356

Query  202  GLNAVTTILDTLKDSTDTNLSGLLGNSNKI-----VSGLLSTVTGLL  243
             L  +T ++ T+  +    LSG  G +        ++GL+STVTG L
Sbjct  357  PLAPLTNVVSTVTGA----LSGATGGATSTSPLAPITGLVSTVTGAL  399


>gb|KGS26498.1| putative transmembrane protein [Burkholderia pseudomallei MSHR5569]
 gb|KGS44173.1| putative transmembrane protein [Burkholderia pseudomallei ABCPW 
107]
 gb|KGS76011.1| putative transmembrane protein [Burkholderia pseudomallei MSHR7500]
 gb|KGS95791.1| putative transmembrane protein [Burkholderia pseudomallei MSHR7498]
 gb|KGW51288.1| putative transmembrane protein [Burkholderia pseudomallei MSHR684]
Length=511

 Score = 39.3 bits (90),  Expect = 4.3, Method: Compositional matrix adjust.
 Identities = 56/167 (34%), Positives = 87/167 (52%), Gaps = 20/167 (12%)

Query  87   TDGLTSTVTGLTSALTGDQISTITSALDDIPLVGQPLHDLLETVLELVNQLG--LANVAS  144
            T  ++S +T +TS +TG      T  + ++  +G P++ LL T+   +NQ G  ++   +
Sbjct  240  TQTVSSAITPITSMVTGT-----TQTVGNVTGLGAPVNTLLGTIGGGLNQAGALISKTGN  294

Query  145  GALASGASGSSSGLGGLL---GGLLGGGASSATNLQNIITQVVSGLSSVSSGVSGASGST  201
              + +G   + S  G  +   GGLL GG S ATN    IT  V GL+   SGVSGA+  T
Sbjct  295  NPVTTGLGQTVSATGNTITSVGGLLTGG-SGATNPLAPITAAVGGLTGTLSGVSGATSGT  353

Query  202  GLNAVTTILDTLKDSTDTNLSGLLGNSNKI-----VSGLLSTVTGLL  243
             L  +T ++ T+  +    LSG  G +        ++GL+STVTG L
Sbjct  354  PLAPLTNVVSTVTGA----LSGATGGATSTSPLAPITGLVSTVTGAL  396


>gb|KGD45241.1| hypothetical protein DP44_5997 [Burkholderia pseudomallei]
 gb|KGS06169.1| hypothetical protein X948_2168 [Burkholderia pseudomallei MSHR5608]
 gb|KGS25762.1| hypothetical protein X962_1106 [Burkholderia pseudomallei MSHR7343]
 gb|KGS74288.1| hypothetical protein X947_1462 [Burkholderia pseudomallei MSHR7334]
 gb|KGV02413.1| hypothetical protein X880_97 [Burkholderia pseudomallei MSHR4032]
 gb|KGV08731.1| hypothetical protein X895_84 [Burkholderia pseudomallei MSHR4503]
Length=511

 Score = 39.3 bits (90),  Expect = 4.3, Method: Compositional matrix adjust.
 Identities = 56/167 (34%), Positives = 87/167 (52%), Gaps = 20/167 (12%)

Query  87   TDGLTSTVTGLTSALTGDQISTITSALDDIPLVGQPLHDLLETVLELVNQLG--LANVAS  144
            T  ++S +T +TS +TG      T  + ++  +G P++ LL T+   +NQ G  ++   +
Sbjct  240  TQTVSSAITPITSMVTGT-----TQTVGNVTGLGAPVNTLLGTIGGGLNQAGALISKTGN  294

Query  145  GALASGASGSSSGLGGLL---GGLLGGGASSATNLQNIITQVVSGLSSVSSGVSGASGST  201
              + +G   + S  G  +   GGLL GG S ATN    IT  V GL+   SGVSGA+  T
Sbjct  295  NPVTTGLGQTVSATGNTITSVGGLLTGG-SGATNPLAPITAAVGGLTGTLSGVSGATSGT  353

Query  202  GLNAVTTILDTLKDSTDTNLSGLLGNSNKI-----VSGLLSTVTGLL  243
             L  +T ++ T+  +    LSG  G +        ++GL+STVTG L
Sbjct  354  PLAPLTNVVSTVTGA----LSGATGGATSTSPLAPITGLVSTVTGAL  396


>gb|AIO86742.1| putative transmembrane protein [Burkholderia pseudomallei]
 gb|KGS62747.1| hypothetical protein X979_330 [Burkholderia pseudomallei MSHR7527]
 gb|KGV73000.1| putative transmembrane protein [Burkholderia pseudomallei MSHR3964]
 gb|KGV88501.1| putative transmembrane protein [Burkholderia pseudomallei MSHR3951]
 gb|KGW00775.1| putative transmembrane protein [Burkholderia pseudomallei MSHR3960]
 gb|KGW04406.1| putative transmembrane protein [Burkholderia pseudomallei MSHR4303]
 gb|KGW34467.1| putative transmembrane protein [Burkholderia pseudomallei MSHR3016]
Length=511

 Score = 39.3 bits (90),  Expect = 4.3, Method: Compositional matrix adjust.
 Identities = 56/167 (34%), Positives = 87/167 (52%), Gaps = 20/167 (12%)

Query  87   TDGLTSTVTGLTSALTGDQISTITSALDDIPLVGQPLHDLLETVLELVNQLG--LANVAS  144
            T  ++S +T +TS +TG      T  + ++  +G P++ LL T+   +NQ G  ++   +
Sbjct  240  TQTVSSAITPITSMVTGT-----TQTVGNVTGLGAPVNTLLGTIGGGLNQAGALISKTGN  294

Query  145  GALASGASGSSSGLGGLL---GGLLGGGASSATNLQNIITQVVSGLSSVSSGVSGASGST  201
              + +G   + S  G  +   GGLL GG S ATN    IT  V GL+   SGVSGA+  T
Sbjct  295  NPVTTGLGQTVSATGNTITSVGGLLTGG-SGATNPLAPITAAVGGLTGTLSGVSGATSGT  353

Query  202  GLNAVTTILDTLKDSTDTNLSGLLGNSNKI-----VSGLLSTVTGLL  243
             L  +T ++ T+  +    LSG  G +        ++GL+STVTG L
Sbjct  354  PLAPLTNVVSTVTGA----LSGATGGATSTSPLAPITGLVSTVTGAL  396


>gb|KGU71435.1| putative transmembrane protein [Burkholderia pseudomallei MSHR4304]
 gb|KGV32461.1| putative transmembrane protein [Burkholderia pseudomallei MSHR4308]
Length=515

 Score = 39.3 bits (90),  Expect = 4.3, Method: Compositional matrix adjust.
 Identities = 56/167 (34%), Positives = 87/167 (52%), Gaps = 20/167 (12%)

Query  87   TDGLTSTVTGLTSALTGDQISTITSALDDIPLVGQPLHDLLETVLELVNQLG--LANVAS  144
            T  ++S +T +TS +TG      T  + ++  +G P++ LL T+   +NQ G  ++   +
Sbjct  240  TQTVSSAITPITSMVTGT-----TQTVGNVTGLGAPVNTLLGTIGGGLNQAGALISKTGN  294

Query  145  GALASGASGSSSGLGGLL---GGLLGGGASSATNLQNIITQVVSGLSSVSSGVSGASGST  201
              + +G   + S  G  +   GGLL GG S ATN    IT  V GL+   SGVSGA+  T
Sbjct  295  NPVTTGLGQTVSATGNTITSVGGLLTGG-SGATNPLAPITAAVGGLTGTLSGVSGATSGT  353

Query  202  GLNAVTTILDTLKDSTDTNLSGLLGNSNKI-----VSGLLSTVTGLL  243
             L  +T ++ T+  +    LSG  G +        ++GL+STVTG L
Sbjct  354  PLAPLTNVVSTVTGA----LSGATGGATSTSPLAPITGLVSTVTGAL  396


>gb|AIP48314.1| hypothetical protein DR56_3040 [Burkholderia pseudomallei MSHR5858]
Length=515

 Score = 39.3 bits (90),  Expect = 4.3, Method: Compositional matrix adjust.
 Identities = 56/167 (34%), Positives = 87/167 (52%), Gaps = 20/167 (12%)

Query  87   TDGLTSTVTGLTSALTGDQISTITSALDDIPLVGQPLHDLLETVLELVNQLG--LANVAS  144
            T  ++S +T +TS +TG      T  + ++  +G P++ LL T+   +NQ G  ++   +
Sbjct  240  TQTVSSAITPITSMVTGT-----TQTVGNVTGLGAPVNTLLGTIGGGLNQAGALISKTGN  294

Query  145  GALASGASGSSSGLGGLL---GGLLGGGASSATNLQNIITQVVSGLSSVSSGVSGASGST  201
              + +G   + S  G  +   GGLL GG S ATN    IT  V GL+   SGVSGA+  T
Sbjct  295  NPVTTGLGQTVSATGNTITSVGGLLTGG-SGATNPLAPITAAVGGLTGTLSGVSGATSGT  353

Query  202  GLNAVTTILDTLKDSTDTNLSGLLGNSNKI-----VSGLLSTVTGLL  243
             L  +T ++ T+  +    LSG  G +        ++GL+STVTG L
Sbjct  354  PLAPLTNVVSTVTGA----LSGATGGATSTSPLAPITGLVSTVTGAL  396


>gb|AIP51884.1| putative transmembrane protein [Burkholderia pseudomallei HBPUB10134a]
 gb|KGD20287.1| putative transmembrane protein [Burkholderia sp. BGJ]
 gb|AIS46069.1| putative transmembrane protein [Burkholderia sp. BGK]
 gb|AIV83098.1| hypothetical protein X978_437 [Burkholderia pseudomallei MSHR3965]
 gb|KGT01137.1| hypothetical protein JT30_364 [Burkholderia pseudomallei]
 gb|KGW14486.1| putative transmembrane protein [Burkholderia pseudomallei MSHR4000]
 gb|AJX59680.1| hypothetical protein DP47_2345 [Burkholderia pseudomallei Pasteur 
52237]
Length=515

 Score = 39.3 bits (90),  Expect = 4.3, Method: Compositional matrix adjust.
 Identities = 56/167 (34%), Positives = 87/167 (52%), Gaps = 20/167 (12%)

Query  87   TDGLTSTVTGLTSALTGDQISTITSALDDIPLVGQPLHDLLETVLELVNQLG--LANVAS  144
            T  ++S +T +TS +TG      T  + ++  +G P++ LL T+   +NQ G  ++   +
Sbjct  240  TQTVSSAITPITSMVTGT-----TQTVGNVTGLGAPVNTLLGTIGGGLNQAGALISKTGN  294

Query  145  GALASGASGSSSGLGGLL---GGLLGGGASSATNLQNIITQVVSGLSSVSSGVSGASGST  201
              + +G   + S  G  +   GGLL GG S ATN    IT  V GL+   SGVSGA+  T
Sbjct  295  NPVTTGLGQTVSATGNTITSVGGLLTGG-SGATNPLAPITAAVGGLTGTLSGVSGATSGT  353

Query  202  GLNAVTTILDTLKDSTDTNLSGLLGNSNKI-----VSGLLSTVTGLL  243
             L  +T ++ T+  +    LSG  G +        ++GL+STVTG L
Sbjct  354  PLAPLTNVVSTVTGA----LSGATGGATSTSPLAPITGLVSTVTGAL  396


>gb|KGV23356.1| putative transmembrane protein [Burkholderia pseudomallei MSHR4462]
 gb|KGX04402.1| putative transmembrane protein [Burkholderia pseudomallei MSHR640]
Length=516

 Score = 39.3 bits (90),  Expect = 4.3, Method: Compositional matrix adjust.
 Identities = 56/167 (34%), Positives = 87/167 (52%), Gaps = 20/167 (12%)

Query  87   TDGLTSTVTGLTSALTGDQISTITSALDDIPLVGQPLHDLLETVLELVNQLG--LANVAS  144
            T  ++S +T +TS +TG      T  + ++  +G P++ LL T+   +NQ G  ++   +
Sbjct  249  TQTVSSAITPITSMVTGT-----TQTVGNVTGLGAPVNTLLGTIGGGLNQAGALISKTGN  303

Query  145  GALASGASGSSSGLGGLL---GGLLGGGASSATNLQNIITQVVSGLSSVSSGVSGASGST  201
              + +G   + S  G  +   GGLL GG S ATN    IT  V GL+   SGVSGA+  T
Sbjct  304  NPVTTGLGQTVSATGNTITSVGGLLTGG-SGATNPLAPITAAVGGLTGTLSGVSGATSGT  362

Query  202  GLNAVTTILDTLKDSTDTNLSGLLGNSNKI-----VSGLLSTVTGLL  243
             L  +T ++ T+  +    LSG  G +        ++GL+STVTG L
Sbjct  363  PLAPLTNVVSTVTGA----LSGATGGATSTSPLAPITGLVSTVTGAL  405


>gb|ACQ95514.1| collagen triple helix repeat protein [Burkholderia pseudomallei 
MSHR346]
 gb|AIP11888.1| putative transmembrane protein [Burkholderia pseudomallei]
Length=516

 Score = 39.3 bits (90),  Expect = 4.3, Method: Compositional matrix adjust.
 Identities = 56/167 (34%), Positives = 87/167 (52%), Gaps = 20/167 (12%)

Query  87   TDGLTSTVTGLTSALTGDQISTITSALDDIPLVGQPLHDLLETVLELVNQLG--LANVAS  144
            T  ++S +T +TS +TG      T  + ++  +G P++ LL T+   +NQ G  ++   +
Sbjct  245  TQTVSSAITPITSMVTGT-----TQTVGNVTGLGAPVNTLLGTIGGGLNQAGALISKTGN  299

Query  145  GALASGASGSSSGLGGLL---GGLLGGGASSATNLQNIITQVVSGLSSVSSGVSGASGST  201
              + +G   + S  G  +   GGLL GG S ATN    IT  V GL+   SGVSGA+  T
Sbjct  300  NPVTTGLGQTVSATGNTITSVGGLLTGG-SGATNPLAPITAAVGGLTGTLSGVSGATSGT  358

Query  202  GLNAVTTILDTLKDSTDTNLSGLLGNSNKI-----VSGLLSTVTGLL  243
             L  +T ++ T+  +    LSG  G +        ++GL+STVTG L
Sbjct  359  PLAPLTNVVSTVTGA----LSGATGGATSTSPLAPITGLVSTVTGAL  401


>gb|KGC47533.1| putative transmembrane protein [Burkholderia pseudomallei]
Length=520

 Score = 39.3 bits (90),  Expect = 4.3, Method: Compositional matrix adjust.
 Identities = 56/167 (34%), Positives = 87/167 (52%), Gaps = 20/167 (12%)

Query  87   TDGLTSTVTGLTSALTGDQISTITSALDDIPLVGQPLHDLLETVLELVNQLG--LANVAS  144
            T  ++S +T +TS +TG      T  + ++  +G P++ LL T+   +NQ G  ++   +
Sbjct  253  TQTVSSAITPITSMVTGT-----TQTVGNVTGLGAPVNTLLGTIGGGLNQAGALISKTGN  307

Query  145  GALASGASGSSSGLGGLL---GGLLGGGASSATNLQNIITQVVSGLSSVSSGVSGASGST  201
              + +G   + S  G  +   GGLL GG S ATN    IT  V GL+   SGVSGA+  T
Sbjct  308  NPVTTGLGQTVSATGNTITSVGGLLTGG-SGATNPLAPITAAVGGLTGTLSGVSGATSGT  366

Query  202  GLNAVTTILDTLKDSTDTNLSGLLGNSNKI-----VSGLLSTVTGLL  243
             L  +T ++ T+  +    LSG  G +        ++GL+STVTG L
Sbjct  367  PLAPLTNVVSTVTGA----LSGATGGATSTSPLAPITGLVSTVTGAL  409


>gb|KGV70413.1| putative transmembrane protein [Burkholderia pseudomallei MSHR4375]
Length=521

 Score = 39.3 bits (90),  Expect = 4.3, Method: Compositional matrix adjust.
 Identities = 56/167 (34%), Positives = 87/167 (52%), Gaps = 20/167 (12%)

Query  87   TDGLTSTVTGLTSALTGDQISTITSALDDIPLVGQPLHDLLETVLELVNQLG--LANVAS  144
            T  ++S +T +TS +TG      T  + ++  +G P++ LL T+   +NQ G  ++   +
Sbjct  250  TQTVSSAITPITSMVTGT-----TQTVGNVTGLGAPVNTLLGTIGGGLNQAGALISKTGN  304

Query  145  GALASGASGSSSGLGGLL---GGLLGGGASSATNLQNIITQVVSGLSSVSSGVSGASGST  201
              + +G   + S  G  +   GGLL GG S ATN    IT  V GL+   SGVSGA+  T
Sbjct  305  NPVTTGLGQTVSATGNTITSVGGLLTGG-SGATNPLAPITAAVGGLTGTLSGVSGATSGT  363

Query  202  GLNAVTTILDTLKDSTDTNLSGLLGNSNKI-----VSGLLSTVTGLL  243
             L  +T ++ T+  +    LSG  G +        ++GL+STVTG L
Sbjct  364  PLAPLTNVVSTVTGA----LSGATGGATSTSPLAPITGLVSTVTGAL  406


>ref|WP_009937686.1| hypothetical protein, partial [Burkholderia pseudomallei]
Length=460

 Score = 39.3 bits (90),  Expect = 4.3, Method: Compositional matrix adjust.
 Identities = 56/167 (34%), Positives = 87/167 (52%), Gaps = 20/167 (12%)

Query  87   TDGLTSTVTGLTSALTGDQISTITSALDDIPLVGQPLHDLLETVLELVNQLG--LANVAS  144
            T  ++S +T +TS +TG      T  + ++  +G P++ LL T+   +NQ G  ++   +
Sbjct  193  TQTVSSAITPITSMVTGT-----TQTVGNVTGLGAPVNTLLGTIGGGLNQAGALISKTGN  247

Query  145  GALASGASGSSSGLGGLL---GGLLGGGASSATNLQNIITQVVSGLSSVSSGVSGASGST  201
              + +G   + S  G  +   GGLL GG S ATN    IT  V GL+   SGVSGA+  T
Sbjct  248  NPVTTGLGQTVSATGNTITSVGGLLTGG-SGATNPLAPITAAVGGLTGTLSGVSGATSGT  306

Query  202  GLNAVTTILDTLKDSTDTNLSGLLGNSNKI-----VSGLLSTVTGLL  243
             L  +T ++ T+  +    LSG  G +        ++GL+STVTG L
Sbjct  307  PLAPLTNVVSTVTGA----LSGATGGATSTSPLAPITGLVSTVTGAL  349


>gb|EBA46384.1| ISBma2, transposase [Burkholderia pseudomallei 305]
 gb|AIP80207.1| hypothetical protein JE55_3196 [Burkholderia pseudomallei]
 gb|KGW56355.1| hypothetical protein Y029_2127 [Burkholderia pseudomallei MSHR303]
Length=480

 Score = 39.3 bits (90),  Expect = 4.3, Method: Compositional matrix adjust.
 Identities = 56/167 (34%), Positives = 87/167 (52%), Gaps = 20/167 (12%)

Query  87   TDGLTSTVTGLTSALTGDQISTITSALDDIPLVGQPLHDLLETVLELVNQLG--LANVAS  144
            T  ++S +T +TS +TG      T  + ++  +G P++ LL T+   +NQ G  ++   +
Sbjct  240  TQTVSSAITPITSMVTGT-----TQTVGNVTGLGAPVNTLLGTIGGGLNQAGALISKTGN  294

Query  145  GALASGASGSSSGLGGLL---GGLLGGGASSATNLQNIITQVVSGLSSVSSGVSGASGST  201
              + +G   + S  G  +   GGLL GG S ATN    IT  V GL+   SGVSGA+  T
Sbjct  295  NPVTTGLGQTVSATGNTITSVGGLLTGG-SGATNPLAPITAAVGGLTGTLSGVSGATSGT  353

Query  202  GLNAVTTILDTLKDSTDTNLSGLLGNSNKI-----VSGLLSTVTGLL  243
             L  +T ++ T+  +    LSG  G +        ++GL+STVTG L
Sbjct  354  PLAPLTNVVSTVTGA----LSGATGGATSTSPLAPITGLVSTVTGAL  396


>ref|WP_050856926.1| hypothetical protein [Burkholderia pseudomallei]
 emb|CFV66135.1| lipoprotein [Burkholderia pseudomallei]
 emb|CPF26978.1| lipoprotein [Burkholderia pseudomallei]
Length=560

 Score = 39.3 bits (90),  Expect = 4.4, Method: Compositional matrix adjust.
 Identities = 59/172 (34%), Positives = 90/172 (52%), Gaps = 21/172 (12%)

Query  87   TDGLTSTVTGLTSALTGDQISTITSALDDIPLVGQPLHDLLETVLELVNQLG--LANVAS  144
            T  ++S +T +TS +TG      T  + ++  +G P++ LL T+   +NQ G  ++   +
Sbjct  262  TQTVSSAITPITSMVTGT-----TQTVGNVTGLGAPVNTLLGTIGGGLNQAGALISKTGN  316

Query  145  GALASGASGSSSGLGGLL---GGLLGGGASSATNLQNIITQVVSGLSSVSSGVSGASGST  201
              + +G   + S  G  +   GGLL GG S ATN    IT  V GL+   SGVSGA+  T
Sbjct  317  NPVTTGLGQTVSATGNTITSVGGLLTGG-SGATNPLAPITAAVGGLTGTLSGVSGATSGT  375

Query  202  GLNAVTTILDTLKDSTDTNLSGLLGNSNKI-----VSGLLSTVTGLL-GANG  247
             L  +T ++ T+  +    LSG  G +        ++GL+STVTG L GA G
Sbjct  376  PLAPLTNVVSTVTGA----LSGATGGATSTSPLAPITGLVSTVTGALSGATG  423


>ref|WP_038730104.1| membrane protein [Burkholderia sp. ABCPW 1]
 gb|KGX09823.1| putative transmembrane protein [Burkholderia sp. ABCPW 1]
Length=529

 Score = 39.3 bits (90),  Expect = 4.4, Method: Compositional matrix adjust.
 Identities = 56/167 (34%), Positives = 87/167 (52%), Gaps = 20/167 (12%)

Query  87   TDGLTSTVTGLTSALTGDQISTITSALDDIPLVGQPLHDLLETVLELVNQLG--LANVAS  144
            T  ++S +T +TS +TG      T  + ++  +G P++ LL T+   +NQ G  ++   +
Sbjct  262  TQTVSSAITPITSMVTGT-----TQTVGNVTGLGAPVNTLLGTIGGGLNQAGALISKTGN  316

Query  145  GALASGASGSSSGLGGLL---GGLLGGGASSATNLQNIITQVVSGLSSVSSGVSGASGST  201
              + +G   + S  G  +   GGLL GG S ATN    IT  V GL+   SGVSGA+  T
Sbjct  317  NPVTTGLGQTVSATGNTITSVGGLLTGG-SGATNPLAPITAAVGGLTGTLSGVSGATSGT  375

Query  202  GLNAVTTILDTLKDSTDTNLSGLLGNSNKI-----VSGLLSTVTGLL  243
             L  +T ++ T+  +    LSG  G +        ++GL+STVTG L
Sbjct  376  PLAPLTNVVSTVTGA----LSGATGGATSTSPLAPITGLVSTVTGAL  418


>gb|EMP77057.1| hypothetical protein D512_09113 [Burkholderia pseudomallei MSHR1043]
Length=530

 Score = 39.3 bits (90),  Expect = 4.4, Method: Compositional matrix adjust.
 Identities = 56/167 (34%), Positives = 87/167 (52%), Gaps = 20/167 (12%)

Query  87   TDGLTSTVTGLTSALTGDQISTITSALDDIPLVGQPLHDLLETVLELVNQLG--LANVAS  144
            T  ++S +T +TS +TG      T  + ++  +G P++ LL T+   +NQ G  ++   +
Sbjct  263  TQTVSSAITPITSMVTGT-----TQTVGNVTGLGAPVNTLLGTIGGGLNQAGALISKTGN  317

Query  145  GALASGASGSSSGLGGLL---GGLLGGGASSATNLQNIITQVVSGLSSVSSGVSGASGST  201
              + +G   + S  G  +   GGLL GG S ATN    IT  V GL+   SGVSGA+  T
Sbjct  318  NPVTTGLGQTVSATGNTITSVGGLLTGG-SGATNPLAPITAAVGGLTGTLSGVSGATSGT  376

Query  202  GLNAVTTILDTLKDSTDTNLSGLLGNSNKI-----VSGLLSTVTGLL  243
             L  +T ++ T+  +    LSG  G +        ++GL+STVTG L
Sbjct  377  PLAPLTNVVSTVTGA----LSGATGGATSTSPLAPITGLVSTVTGAL  419


>gb|EQA89458.1| hypothetical protein M218_08355 [Burkholderia pseudomallei MSHR338]
Length=539

 Score = 39.3 bits (90),  Expect = 4.4, Method: Compositional matrix adjust.
 Identities = 56/167 (34%), Positives = 87/167 (52%), Gaps = 20/167 (12%)

Query  87   TDGLTSTVTGLTSALTGDQISTITSALDDIPLVGQPLHDLLETVLELVNQLG--LANVAS  144
            T  ++S +T +TS +TG      T  + ++  +G P++ LL T+   +NQ G  ++   +
Sbjct  268  TQTVSSAITPITSMVTGT-----TQTVGNVTGLGAPVNTLLGTIGGGLNQAGALISKTGN  322

Query  145  GALASGASGSSSGLGGLL---GGLLGGGASSATNLQNIITQVVSGLSSVSSGVSGASGST  201
              + +G   + S  G  +   GGLL GG S ATN    IT  V GL+   SGVSGA+  T
Sbjct  323  NPVTTGLGQTVSATGNTITSVGGLLTGG-SGATNPLAPITAAVGGLTGTLSGVSGATSGT  381

Query  202  GLNAVTTILDTLKDSTDTNLSGLLGNSNKI-----VSGLLSTVTGLL  243
             L  +T ++ T+  +    LSG  G +        ++GL+STVTG L
Sbjct  382  PLAPLTNVVSTVTGA----LSGATGGATSTSPLAPITGLVSTVTGAL  424


>gb|KGD58869.1| putative transmembrane protein [Burkholderia pseudomallei]
Length=502

 Score = 39.3 bits (90),  Expect = 4.5, Method: Compositional matrix adjust.
 Identities = 59/172 (34%), Positives = 90/172 (52%), Gaps = 21/172 (12%)

Query  87   TDGLTSTVTGLTSALTGDQISTITSALDDIPLVGQPLHDLLETVLELVNQLG--LANVAS  144
            T  ++S +T +TS +TG      T  + ++  +G P++ LL T+   +NQ G  ++   +
Sbjct  231  TQTVSSAITPITSMVTGT-----TQTVGNVTGLGAPVNTLLGTIGGGLNQAGALISKTGN  285

Query  145  GALASGASGSSSGLGGLL---GGLLGGGASSATNLQNIITQVVSGLSSVSSGVSGASGST  201
              + +G   + S  G  +   GGLL GG S ATN    IT  V GL+   SGVSGA+  T
Sbjct  286  NPVTTGLGQTVSATGNTITSVGGLLTGG-SGATNPLAPITAAVGGLTGTLSGVSGATSGT  344

Query  202  GLNAVTTILDTLKDSTDTNLSGLLGNSNKI-----VSGLLSTVTGLL-GANG  247
             L  +T ++ T+  +    LSG  G +        ++GL+STVTG L GA G
Sbjct  345  PLAPLTNVVSTVTGA----LSGATGGATSTSPLAPITGLVSTVTGALSGATG  392


>gb|AIP13048.1| putative transmembrane protein [Burkholderia pseudomallei]
 gb|KGS03520.1| putative transmembrane protein [Burkholderia pseudomallei MSHR7504]
Length=507

 Score = 39.3 bits (90),  Expect = 4.5, Method: Compositional matrix adjust.
 Identities = 59/172 (34%), Positives = 90/172 (52%), Gaps = 21/172 (12%)

Query  87   TDGLTSTVTGLTSALTGDQISTITSALDDIPLVGQPLHDLLETVLELVNQLG--LANVAS  144
            T  ++S +T +TS +TG      T  + ++  +G P++ LL T+   +NQ G  ++   +
Sbjct  240  TQTVSSAITPITSMVTGT-----TQTVGNVTGLGAPVNTLLGTIGGGLNQAGALISKTGN  294

Query  145  GALASGASGSSSGLGGLL---GGLLGGGASSATNLQNIITQVVSGLSSVSSGVSGASGST  201
              + +G   + S  G  +   GGLL GG S ATN    IT  V GL+   SGVSGA+  T
Sbjct  295  NPVTTGLGQTVSATGNTITSVGGLLTGG-SGATNPLAPITAAVGGLTGTLSGVSGATSGT  353

Query  202  GLNAVTTILDTLKDSTDTNLSGLLGNSNKI-----VSGLLSTVTGLL-GANG  247
             L  +T ++ T+  +    LSG  G +        ++GL+STVTG L GA G
Sbjct  354  PLAPLTNVVSTVTGA----LSGATGGATSTSPLAPITGLVSTVTGALSGATG  401


>gb|KGX08649.1| putative transmembrane protein [Burkholderia sp. MSHR44]
Length=510

 Score = 39.3 bits (90),  Expect = 4.5, Method: Compositional matrix adjust.
 Identities = 59/172 (34%), Positives = 90/172 (52%), Gaps = 21/172 (12%)

Query  87   TDGLTSTVTGLTSALTGDQISTITSALDDIPLVGQPLHDLLETVLELVNQLG--LANVAS  144
            T  ++S +T +TS +TG      T  + ++  +G P++ LL T+   +NQ G  ++   +
Sbjct  243  TQTVSSAITPITSMVTGT-----TQTVGNVTGLGAPVNTLLGTIGGGLNQAGALISKTGN  297

Query  145  GALASGASGSSSGLGGLL---GGLLGGGASSATNLQNIITQVVSGLSSVSSGVSGASGST  201
              + +G   + S  G  +   GGLL GG S ATN    IT  V GL+   SGVSGA+  T
Sbjct  298  NPVTTGLGQTVSATGNTITSVGGLLTGG-SGATNPLAPITAAVGGLTGTLSGVSGATSGT  356

Query  202  GLNAVTTILDTLKDSTDTNLSGLLGNSNKI-----VSGLLSTVTGLL-GANG  247
             L  +T ++ T+  +    LSG  G +        ++GL+STVTG L GA G
Sbjct  357  PLAPLTNVVSTVTGA----LSGATGGATSTSPLAPITGLVSTVTGALSGATG  404


>gb|KGC29861.1| putative transmembrane protein [Burkholderia pseudomallei]
 gb|KGC91785.1| putative transmembrane protein [Burkholderia pseudomallei]
Length=511

 Score = 39.3 bits (90),  Expect = 4.5, Method: Compositional matrix adjust.
 Identities = 59/172 (34%), Positives = 90/172 (52%), Gaps = 21/172 (12%)

Query  87   TDGLTSTVTGLTSALTGDQISTITSALDDIPLVGQPLHDLLETVLELVNQLG--LANVAS  144
            T  ++S +T +TS +TG      T  + ++  +G P++ LL T+   +NQ G  ++   +
Sbjct  240  TQTVSSAITPITSMVTGT-----TQTVGNVTGLGAPVNTLLGTIGGGLNQAGALISKTGN  294

Query  145  GALASGASGSSSGLGGLL---GGLLGGGASSATNLQNIITQVVSGLSSVSSGVSGASGST  201
              + +G   + S  G  +   GGLL GG S ATN    IT  V GL+   SGVSGA+  T
Sbjct  295  NPVTTGLGQTVSATGNTITSVGGLLTGG-SGATNPLAPITAAVGGLTGTLSGVSGATSGT  353

Query  202  GLNAVTTILDTLKDSTDTNLSGLLGNSNKI-----VSGLLSTVTGLL-GANG  247
             L  +T ++ T+  +    LSG  G +        ++GL+STVTG L GA G
Sbjct  354  PLAPLTNVVSTVTGA----LSGATGGATSTSPLAPITGLVSTVTGALSGATG  401


>gb|EDO83984.1| conserved hypothetical protein [Burkholderia pseudomallei 406e]
 gb|EXJ01817.1| membrane protein [Burkholderia pseudomallei MSHR6137]
 gb|AIP20524.1| putative transmembrane protein [Burkholderia pseudomallei MSHR5855]
 gb|AIP39473.1| putative transmembrane protein [Burkholderia pseudomallei MSHR5848]
 gb|AIP72852.1| putative transmembrane protein [Burkholderia pseudomallei]
 gb|AJW92559.1| putative transmembrane protein [Burkholderia pseudomallei 406e]
Length=511

 Score = 39.3 bits (90),  Expect = 4.5, Method: Compositional matrix adjust.
 Identities = 59/172 (34%), Positives = 90/172 (52%), Gaps = 21/172 (12%)

Query  87   TDGLTSTVTGLTSALTGDQISTITSALDDIPLVGQPLHDLLETVLELVNQLG--LANVAS  144
            T  ++S +T +TS +TG      T  + ++  +G P++ LL T+   +NQ G  ++   +
Sbjct  240  TQTVSSAITPITSMVTGT-----TQTVGNVTGLGAPVNTLLGTIGGGLNQAGALISKTGN  294

Query  145  GALASGASGSSSGLGGLL---GGLLGGGASSATNLQNIITQVVSGLSSVSSGVSGASGST  201
              + +G   + S  G  +   GGLL GG S ATN    IT  V GL+   SGVSGA+  T
Sbjct  295  NPVTTGLGQTVSATGNTITSVGGLLTGG-SGATNPLAPITAAVGGLTGTLSGVSGATSGT  353

Query  202  GLNAVTTILDTLKDSTDTNLSGLLGNSNKI-----VSGLLSTVTGLL-GANG  247
             L  +T ++ T+  +    LSG  G +        ++GL+STVTG L GA G
Sbjct  354  PLAPLTNVVSTVTGA----LSGATGGATSTSPLAPITGLVSTVTGALSGATG  401


>gb|KGC49187.1| putative transmembrane protein [Burkholderia pseudomallei]
 gb|KGD12894.1| putative transmembrane protein [Burkholderia pseudomallei]
 gb|KGS64689.1| putative transmembrane protein [Burkholderia pseudomallei MSHR4868]
 gb|KGX62802.1| putative transmembrane protein [Burkholderia pseudomallei TSV44]
Length=514

 Score = 39.3 bits (90),  Expect = 4.5, Method: Compositional matrix adjust.
 Identities = 59/172 (34%), Positives = 90/172 (52%), Gaps = 21/172 (12%)

Query  87   TDGLTSTVTGLTSALTGDQISTITSALDDIPLVGQPLHDLLETVLELVNQLG--LANVAS  144
            T  ++S +T +TS +TG      T  + ++  +G P++ LL T+   +NQ G  ++   +
Sbjct  243  TQTVSSAITPITSMVTGT-----TQTVGNVTGLGAPVNTLLGTIGGGLNQAGALISKTGN  297

Query  145  GALASGASGSSSGLGGLL---GGLLGGGASSATNLQNIITQVVSGLSSVSSGVSGASGST  201
              + +G   + S  G  +   GGLL GG S ATN    IT  V GL+   SGVSGA+  T
Sbjct  298  NPVTTGLGQTVSATGNTITSVGGLLTGG-SGATNPLAPITAAVGGLTGTLSGVSGATSGT  356

Query  202  GLNAVTTILDTLKDSTDTNLSGLLGNSNKI-----VSGLLSTVTGLL-GANG  247
             L  +T ++ T+  +    LSG  G +        ++GL+STVTG L GA G
Sbjct  357  PLAPLTNVVSTVTGA----LSGATGGATSTSPLAPITGLVSTVTGALSGATG  404


>gb|KGC69984.1| putative transmembrane protein [Burkholderia pseudomallei]
Length=515

 Score = 39.3 bits (90),  Expect = 4.5, Method: Compositional matrix adjust.
 Identities = 59/172 (34%), Positives = 90/172 (52%), Gaps = 21/172 (12%)

Query  87   TDGLTSTVTGLTSALTGDQISTITSALDDIPLVGQPLHDLLETVLELVNQLG--LANVAS  144
            T  ++S +T +TS +TG      T  + ++  +G P++ LL T+   +NQ G  ++   +
Sbjct  240  TQTVSSAITPITSMVTGT-----TQTVGNVTGLGAPVNTLLGTIGGGLNQAGALISKTGN  294

Query  145  GALASGASGSSSGLGGLL---GGLLGGGASSATNLQNIITQVVSGLSSVSSGVSGASGST  201
              + +G   + S  G  +   GGLL GG S ATN    IT  V GL+   SGVSGA+  T
Sbjct  295  NPVTTGLGQTVSATGNTITSVGGLLTGG-SGATNPLAPITAAVGGLTGTLSGVSGATSGT  353

Query  202  GLNAVTTILDTLKDSTDTNLSGLLGNSNKI-----VSGLLSTVTGLL-GANG  247
             L  +T ++ T+  +    LSG  G +        ++GL+STVTG L GA G
Sbjct  354  PLAPLTNVVSTVTGA----LSGATGGATSTSPLAPITGLVSTVTGALSGATG  401


>gb|KGW62006.1| putative transmembrane protein [Burkholderia pseudomallei MSHR1357]
Length=518

 Score = 39.3 bits (90),  Expect = 4.5, Method: Compositional matrix adjust.
 Identities = 59/172 (34%), Positives = 90/172 (52%), Gaps = 21/172 (12%)

Query  87   TDGLTSTVTGLTSALTGDQISTITSALDDIPLVGQPLHDLLETVLELVNQLG--LANVAS  144
            T  ++S +T +TS +TG      T  + ++  +G P++ LL T+   +NQ G  ++   +
Sbjct  251  TQTVSSAITPITSMVTGT-----TQTVGNVTGLGAPVNTLLGTIGGGLNQAGALISKTGN  305

Query  145  GALASGASGSSSGLGGLL---GGLLGGGASSATNLQNIITQVVSGLSSVSSGVSGASGST  201
              + +G   + S  G  +   GGLL GG S ATN    IT  V GL+   SGVSGA+  T
Sbjct  306  NPVTTGLGQTVSATGNTITSVGGLLTGG-SGATNPLAPITAAVGGLTGTLSGVSGATSGT  364

Query  202  GLNAVTTILDTLKDSTDTNLSGLLGNSNKI-----VSGLLSTVTGLL-GANG  247
             L  +T ++ T+  +    LSG  G +        ++GL+STVTG L GA G
Sbjct  365  PLAPLTNVVSTVTGA----LSGATGGATSTSPLAPITGLVSTVTGALSGATG  412


>gb|EEH24272.1| conserved hypothetical protein [Burkholderia pseudomallei Pakistan 
9]
Length=514

 Score = 39.3 bits (90),  Expect = 4.8, Method: Compositional matrix adjust.
 Identities = 56/167 (34%), Positives = 87/167 (52%), Gaps = 20/167 (12%)

Query  87   TDGLTSTVTGLTSALTGDQISTITSALDDIPLVGQPLHDLLETVLELVNQLG--LANVAS  144
            T  ++S +T +TS +TG      T  + ++  +G P++ LL T+   +NQ G  ++   +
Sbjct  243  TQTVSSAITPITSMVTGT-----TQTVGNVTGLGAPVNTLLGTIGGGLNQAGALISKTGN  297

Query  145  GALASGASGSSSGLGGLL---GGLLGGGASSATNLQNIITQVVSGLSSVSSGVSGASGST  201
              + +G   + S  G  +   GGLL GG S ATN    IT  V GL+   SGVSGA+  T
Sbjct  298  NPVTTGLGQTVSATGNTITSVGGLLTGG-SGATNPLAPITAAVGGLTGTLSGVSGATSGT  356

Query  202  GLNAVTTILDTLKDSTDTNLSGLLGNSNKI-----VSGLLSTVTGLL  243
             L  +T ++ T+  +    LSG  G +        ++GL+STVTG L
Sbjct  357  PLAPLTNVVSTVTGA----LSGATGGATSTSPLAPITGLVSTVTGAL  399


>gb|AIV76499.1| putative transmembrane protein [Burkholderia sp. TSV202]
Length=495

 Score = 38.9 bits (89),  Expect = 5.2, Method: Compositional matrix adjust.
 Identities = 59/172 (34%), Positives = 90/172 (52%), Gaps = 21/172 (12%)

Query  87   TDGLTSTVTGLTSALTGDQISTITSALDDIPLVGQPLHDLLETVLELVNQLG--LANVAS  144
            T  ++S +T +TS +TG      T  + ++  +G P++ LL T+   +NQ G  ++   +
Sbjct  228  TQTVSSAITPITSMVTGT-----TQTVGNVTGLGAPVNTLLGTIGGGLNQAGALISKTGN  282

Query  145  GALASGASGSSSGLGGLL---GGLLGGGASSATNLQNIITQVVSGLSSVSSGVSGASGST  201
              + +G   + S  G  +   GGLL GG S ATN    IT  V GL+   SGVSGA+  T
Sbjct  283  NPVTTGLGQTVSATGNTITSVGGLLTGG-SGATNPLAPITAAVGGLTGTLSGVSGATSGT  341

Query  202  GLNAVTTILDTLKDSTDTNLSGLLGNSNKI-----VSGLLSTVTGLL-GANG  247
             L  +T ++ T+  +    LSG  G +        ++GL+STVTG L GA G
Sbjct  342  PLAPLTNVVSTVTGA----LSGATGGATSTSPLAPITGLVSTVTGALSGATG  389


>gb|AIV52115.1| putative transmembrane protein [Burkholderia pseudomallei MSHR1153]
Length=511

 Score = 38.9 bits (89),  Expect = 5.2, Method: Compositional matrix adjust.
 Identities = 59/172 (34%), Positives = 90/172 (52%), Gaps = 21/172 (12%)

Query  87   TDGLTSTVTGLTSALTGDQISTITSALDDIPLVGQPLHDLLETVLELVNQLG--LANVAS  144
            T  ++S +T +TS +TG      T  + ++  +G P++ LL T+   +NQ G  ++   +
Sbjct  240  TQTVSSAITPITSMVTGT-----TQTVGNVTGLGAPVNTLLGTIGGGLNQAGALISKTGN  294

Query  145  GALASGASGSSSGLGGLL---GGLLGGGASSATNLQNIITQVVSGLSSVSSGVSGASGST  201
              + +G   + S  G  +   GGLL GG S ATN    IT  V GL+   SGVSGA+  T
Sbjct  295  NPVTTGLGQTVSATGNTITSVGGLLTGG-SGATNPLAPITAAVGGLTGTLSGVSGATSGT  353

Query  202  GLNAVTTILDTLKDSTDTNLSGLLGNSNKI-----VSGLLSTVTGLL-GANG  247
             L  +T ++ T+  +    LSG  G +        ++GL+STVTG L GA G
Sbjct  354  PLAPLTNVVSTVTGA----LSGATGGATSTSPLAPITGLVSTVTGALSGATG  401


>gb|EES26072.1| conserved hypothetical protein [Burkholderia pseudomallei 1106b]
 gb|AFR15706.1| hypothetical protein BPC006_I1832 [Burkholderia pseudomallei 
BPC006]
 gb|AIO89440.1| putative transmembrane protein [Burkholderia pseudomallei]
 6 more sequence titles

gb|AIO13267.1| putative transmembrane protein [Burkholderia pseudomallei]
 gb|AIP61282.1| putative transmembrane protein [Burkholderia pseudomallei HBPUB10303a]
 gb|KGC72108.1| putative transmembrane protein [Burkholderia pseudomallei]
 gb|KGV57313.1| putative transmembrane protein [Burkholderia pseudomallei BDU 
2]
 gb|KGX37436.1| putative transmembrane protein [Burkholderia pseudomallei MSHR3335]
 gb|KGX61883.1| putative transmembrane protein [Burkholderia pseudomallei TSV5]

Length=511

 Score = 38.9 bits (89),  Expect = 5.2, Method: Compositional matrix adjust.
 Identities = 59/172 (34%), Positives = 90/172 (52%), Gaps = 21/172 (12%)

Query  87   TDGLTSTVTGLTSALTGDQISTITSALDDIPLVGQPLHDLLETVLELVNQLG--LANVAS  144
            T  ++S +T +TS +TG      T  + ++  +G P++ LL T+   +NQ G  ++   +
Sbjct  240  TQTVSSAITPITSMVTGT-----TQTVGNVTGLGAPVNTLLGTIGGGLNQAGALISKTGN  294

Query  145  GALASGASGSSSGLGGLL---GGLLGGGASSATNLQNIITQVVSGLSSVSSGVSGASGST  201
              + +G   + S  G  +   GGLL GG S ATN    IT  V GL+   SGVSGA+  T
Sbjct  295  NPVTTGLGQTVSATGNTITSVGGLLTGG-SGATNPLAPITAAVGGLTGTLSGVSGATSGT  353

Query  202  GLNAVTTILDTLKDSTDTNLSGLLGNSNKI-----VSGLLSTVTGLL-GANG  247
             L  +T ++ T+  +    LSG  G +        ++GL+STVTG L GA G
Sbjct  354  PLAPLTNVVSTVTGA----LSGATGGATSTSPLAPITGLVSTVTGALSGATG  401


>gb|AIV59718.1| putative transmembrane protein [Burkholderia pseudomallei MSHR2243]
 gb|AIV72192.1| putative transmembrane protein [Burkholderia pseudomallei MSHR62]
 gb|KGU77675.1| putative transmembrane protein [Burkholderia pseudomallei MSHR465J]
 gb|KGW70752.1| putative transmembrane protein [Burkholderia pseudomallei MSHR3458]
Length=513

 Score = 38.9 bits (89),  Expect = 5.3, Method: Compositional matrix adjust.
 Identities = 59/172 (34%), Positives = 90/172 (52%), Gaps = 21/172 (12%)

Query  87   TDGLTSTVTGLTSALTGDQISTITSALDDIPLVGQPLHDLLETVLELVNQLG--LANVAS  144
            T  ++S +T +TS +TG      T  + ++  +G P++ LL T+   +NQ G  ++   +
Sbjct  242  TQTVSSAITPITSMVTGT-----TQTVGNVTGLGAPVNTLLGTIGGGLNQAGALISKTGN  296

Query  145  GALASGASGSSSGLGGLL---GGLLGGGASSATNLQNIITQVVSGLSSVSSGVSGASGST  201
              + +G   + S  G  +   GGLL GG S ATN    IT  V GL+   SGVSGA+  T
Sbjct  297  NPVTTGLGQTVSATGNTITSVGGLLTGG-SGATNPLAPITAAVGGLTGTLSGVSGATSGT  355

Query  202  GLNAVTTILDTLKDSTDTNLSGLLGNSNKI-----VSGLLSTVTGLL-GANG  247
             L  +T ++ T+  +    LSG  G +        ++GL+STVTG L GA G
Sbjct  356  PLAPLTNVVSTVTGA----LSGATGGATSTSPLAPITGLVSTVTGALSGATG  403


>gb|KGW30836.1| putative transmembrane protein [Burkholderia pseudomallei MSHR2451]
Length=518

 Score = 38.9 bits (89),  Expect = 5.3, Method: Compositional matrix adjust.
 Identities = 59/172 (34%), Positives = 90/172 (52%), Gaps = 21/172 (12%)

Query  87   TDGLTSTVTGLTSALTGDQISTITSALDDIPLVGQPLHDLLETVLELVNQLG--LANVAS  144
            T  ++S +T +TS +TG      T  + ++  +G P++ LL T+   +NQ G  ++   +
Sbjct  243  TQTVSSAITPITSMVTGT-----TQTVGNVTGLGAPVNTLLGTIGGGLNQAGALISKTGN  297

Query  145  GALASGASGSSSGLGGLL---GGLLGGGASSATNLQNIITQVVSGLSSVSSGVSGASGST  201
              + +G   + S  G  +   GGLL GG S ATN    IT  V GL+   SGVSGA+  T
Sbjct  298  NPVTTGLGQTVSATGNTITSVGGLLTGG-SGATNPLAPITAAVGGLTGTLSGVSGATSGT  356

Query  202  GLNAVTTILDTLKDSTDTNLSGLLGNSNKI-----VSGLLSTVTGLL-GANG  247
             L  +T ++ T+  +    LSG  G +        ++GL+STVTG L GA G
Sbjct  357  PLAPLTNVVSTVTGA----LSGATGGATSTSPLAPITGLVSTVTGALSGATG  404


>gb|KGU64818.1| putative transmembrane protein [Burkholderia pseudomallei MSHR983]
Length=523

 Score = 38.9 bits (89),  Expect = 5.3, Method: Compositional matrix adjust.
 Identities = 59/172 (34%), Positives = 90/172 (52%), Gaps = 21/172 (12%)

Query  87   TDGLTSTVTGLTSALTGDQISTITSALDDIPLVGQPLHDLLETVLELVNQLG--LANVAS  144
            T  ++S +T +TS +TG      T  + ++  +G P++ LL T+   +NQ G  ++   +
Sbjct  252  TQTVSSAITPITSMVTGT-----TQTVGNVTGLGAPVNTLLGTIGGGLNQAGALISKTGN  306

Query  145  GALASGASGSSSGLGGLL---GGLLGGGASSATNLQNIITQVVSGLSSVSSGVSGASGST  201
              + +G   + S  G  +   GGLL GG S ATN    IT  V GL+   SGVSGA+  T
Sbjct  307  NPVTTGLGQTVSATGNTITSVGGLLTGG-SGATNPLAPITAAVGGLTGTLSGVSGATSGT  365

Query  202  GLNAVTTILDTLKDSTDTNLSGLLGNSNKI-----VSGLLSTVTGLL-GANG  247
             L  +T ++ T+  +    LSG  G +        ++GL+STVTG L GA G
Sbjct  366  PLAPLTNVVSTVTGA----LSGATGGATSTSPLAPITGLVSTVTGALSGATG  413


>ref|WP_041196235.1| membrane protein [Burkholderia pseudomallei]
 gb|KGV37021.1| hypothetical protein X985_4620 [Burkholderia pseudomallei MSHR4012]
 gb|KGV63715.1| putative transmembrane protein [Burkholderia pseudomallei MSHR4003]
Length=540

 Score = 38.9 bits (89),  Expect = 6.3, Method: Compositional matrix adjust.
 Identities = 56/167 (34%), Positives = 87/167 (52%), Gaps = 20/167 (12%)

Query  87   TDGLTSTVTGLTSALTGDQISTITSALDDIPLVGQPLHDLLETVLELVNQLG--LANVAS  144
            T  ++S +T +TS +TG      T  + ++  +G P++ LL T+   +NQ G  ++   +
Sbjct  265  TQTVSSAITPITSMVTGT-----TQTVGNVTGLGAPVNTLLGTIGGGLNQAGALISKTGN  319

Query  145  GALASGASGSSSGLGGLL---GGLLGGGASSATNLQNIITQVVSGLSSVSSGVSGASGST  201
              + +G   + S  G  +   GGLL GG S ATN    IT  V GL+   +GVSGA+  T
Sbjct  320  NPVTTGLGQTVSATGNTITSVGGLLTGG-SGATNPLAPITAAVGGLTGTLNGVSGATSGT  378

Query  202  GLNAVTTILDTLKDSTDTNLSGLLGNSNKI-----VSGLLSTVTGLL  243
             L  +T ++ T+  +    LSG  G +        V+GL+STVTG L
Sbjct  379  PLAPLTNVVSTVTGA----LSGATGGATSTSPLAPVTGLVSTVTGAL  421


Lambda      K        H        a         alpha
   0.310    0.129    0.336    0.792     4.96 

Gapped
Lambda      K        H        a         alpha    sigma
   0.267   0.0410    0.140     1.90     42.6     43.6 

Effective search space used: 2077992996660


  Database: nr
    Posted date:  Sep 23, 2015 12:05 AM
  Number of letters in database: 26,053,659,533
  Number of sequences in database:  71,551,133


Matrix: BLOSUM62
Gap Penalties: Existence: 11, Extension: 1
Neighboring words threshold: 11
Window for multiple hits: 40
```
