# Supplementary material for: Comparative genomics provides new insights into the diversity, physiology, and sexuality of the only industrially exploited tremellomycete: Phaffia rhodozyma
Source: BMC Genomics. 2016 Nov 9;17:901. doi: 10.1186/s12864-016-3244-7 (PMC5103461; doi:10.1186/s12864-016-3244-7)
Supplement: Additional file 6: — List of orphan genes with links to PFAM (related to Additional file 1: Table S1). (ZIP 1428 kb) [file 12864_2016_3244_MOESM6_ESM.zip › BLAST_HTML_FTR/G02662_P.html]

BLAST Search Results


```
BLASTP 2.2.27+


Reference:
Stephen F. Altschul, Thomas L. Madden, Alejandro A. Schäffer,
Jinghui Zhang, Zheng Zhang, Webb Miller, and David J. Lipman (1997),
"Gapped BLAST and PSI-BLAST: a new generation of protein database
search programs", Nucleic Acids Res. 25:3389-3402.


Reference for
composition-based statistics:
Alejandro A. Schäffer, L. Aravind, Thomas L. Madden, Sergei
Shavirin, John L. Spouge, Yuri I. Wolf, Eugene V. Koonin, and
Stephen F. Altschul (2001), "Improving the accuracy of PSI-BLAST
protein database searches with composition-based statistics and
other refinements", Nucleic Acids Res. 29:2994-3005.


Database: nr
           71,551,133 sequences; 26,053,659,533 total letters


Query= G02662_P

Length=601
                                                                      Score     E
Sequences producing significant alignments:                          (Bits)  Value

emb|CDZ97538.1|  hypothetical protein [Xanthophyllomyces dendrorh...   611    0.0  
ref|WP_043100073.1|  hypothetical protein [Oleiagrimonas soli] >g...  43.1    0.75 


 >emb|CDZ97538.1| hypothetical protein [Xanthophyllomyces dendrorhous]
Length=473

 Score =  611 bits (1575),  Expect = 0.0, Method: Compositional matrix adjust.
 Identities = 315/341 (92%), Positives = 315/341 (92%), Gaps = 26/341 (8%)

Query  83   PVLKFRPRPHPPSIRFKARHRPALSAPIAAIPTSSSEDKLSFDLSNFKQLSSWKEKIDVK  142
            PVLKFRPRPHPPSIRFKARHRPALSAPIAAIPTSSSEDKLSFDLSNFKQLSSWKEKIDVK
Sbjct  146  PVLKFRPRPHPPSIRFKARHRPALSAPIAAIPTSSSEDKLSFDLSNFKQLSSWKEKIDVK  205

Query  143  SSSIQESRKKELQLPDCQKKPKGTSRPSNKRRATIQKPTSKTLKQLELMGSDDRFPPYEP  202
            SSSIQESRKKELQLPDCQKKPKGTSRPSNKRRATIQKPTSKTLKQLELMGSDDRFPPYEP
Sbjct  206  SSSIQESRKKELQLPDCQKKPKGTSRPSNKRRATIQKPTSKTLKQLELMGSDDRFPPYEP  265

Query  203  KSDPQQWLSHSSNIDHIKVKPVSAFTTIHQATRFLSPLAELPVECSPVESEPITSSPPRL  262
            KSDPQQWLSHSSNIDHIKVKPVSAFTTIHQATRFLSPLAELPVECSPVESEPITSSPPRL
Sbjct  266  KSDPQQWLSHSSNIDHIKVKPVSAFTTIHQATRFLSPLAELPVECSPVESEPITSSPPRL  325

Query  263  DPPTRLVSVADSIVCSPKFSRSRISRPRNTKIRANDVKKTVNQDLRILNLRNISMDENEV  322
            DPPTRLVSVADSI                          TVNQDLRILNLRNISMDENEV
Sbjct  326  DPPTRLVSVADSI--------------------------TVNQDLRILNLRNISMDENEV  359

Query  323  ELRTPVQSSLRPRRLDLQELSTRPSIDPPDGWEKEPDRQLRLLSSTPHCASASNTVTFPR  382
            ELRTPVQSSLRPRRLDLQELSTRPSIDPPDGWEKEPDRQLRLLSSTPHCASASNTVTFPR
Sbjct  360  ELRTPVQSSLRPRRLDLQELSTRPSIDPPDGWEKEPDRQLRLLSSTPHCASASNTVTFPR  419

Query  383  LYSRPRKRPSFSAEEILERLLPVASQVSNSSWLEKETAAHV  423
            LYSRPRKRPSFSAEEILERLLPVASQVSNSSWLEKETAAHV
Sbjct  420  LYSRPRKRPSFSAEEILERLLPVASQVSNSSWLEKETAAHV  460


 Score = 43.1 bits (100),  Expect = 0.73, Method: Compositional matrix adjust.
 Identities = 18/18 (100%), Positives = 18/18 (100%), Gaps = 0/18 (0%)

Query  1   MSPEGFLVTKRWRKRQPT  18
           MSPEGFLVTKRWRKRQPT
Sbjct  1   MSPEGFLVTKRWRKRQPT  18


>ref|WP_043100073.1| hypothetical protein [Oleiagrimonas soli]
 gb|KGI77796.1| hypothetical protein LF63_0105070 [Oleiagrimonas soli]
Length=390

 Score = 43.1 bits (100),  Expect = 0.75, Method: Compositional matrix adjust.
 Identities = 26/85 (31%), Positives = 42/85 (49%), Gaps = 7/85 (8%)

Query  442  RQDAQSYGCGQQKCPASSVVRRTKSILRHRSTLIFDSAGNSTLRPEKLRRRMTVMFDDHQ  501
            R +A+  GC  Q CP S+V RR   +LR  +    D+  +   R  + R+R   +FD +Q
Sbjct  94   RTEARKRGCSVQTCPESAVERRYDELLRTHA----DNGHSDAERSARFRQRYARLFDRYQ  149

Query  502  ---TVFGSSSSDPQTIKRKLSHIIL  523
               T+   S  D + +KR   H++ 
Sbjct  150  HASTLRTVSDPDLRLLKRAAEHVLF  174


Lambda      K        H        a         alpha
   0.317    0.130    0.384    0.792     4.96 

Gapped
Lambda      K        H        a         alpha    sigma
   0.267   0.0410    0.140     1.90     42.6     43.6 

Effective search space used: 6580138453488


  Database: nr
    Posted date:  Sep 23, 2015 12:05 AM
  Number of letters in database: 26,053,659,533
  Number of sequences in database:  71,551,133


Matrix: BLOSUM62
Gap Penalties: Existence: 11, Extension: 1
Neighboring words threshold: 11
Window for multiple hits: 40
```
