# Supplementary material for: Comparative genomics provides new insights into the diversity, physiology, and sexuality of the only industrially exploited tremellomycete: Phaffia rhodozyma
Source: BMC Genomics. 2016 Nov 9;17:901. doi: 10.1186/s12864-016-3244-7 (PMC5103461; doi:10.1186/s12864-016-3244-7)
Supplement: Additional file 6: — List of orphan genes with links to PFAM (related to Additional file 1: Table S1). (ZIP 1428 kb) [file 12864_2016_3244_MOESM6_ESM.zip › BLAST_HTML_FTR/G04803_P.html]

BLAST Search Results


```
BLASTP 2.2.27+


Reference:
Stephen F. Altschul, Thomas L. Madden, Alejandro A. Schäffer,
Jinghui Zhang, Zheng Zhang, Webb Miller, and David J. Lipman (1997),
"Gapped BLAST and PSI-BLAST: a new generation of protein database
search programs", Nucleic Acids Res. 25:3389-3402.


Reference for
composition-based statistics:
Alejandro A. Schäffer, L. Aravind, Thomas L. Madden, Sergei
Shavirin, John L. Spouge, Yuri I. Wolf, Eugene V. Koonin, and
Stephen F. Altschul (2001), "Improving the accuracy of PSI-BLAST
protein database searches with composition-based statistics and
other refinements", Nucleic Acids Res. 29:2994-3005.


Database: nr
           71,551,133 sequences; 26,053,659,533 total letters


Query= G04803_P

Length=645
                                                                      Score     E
Sequences producing significant alignments:                          (Bits)  Value

emb|CDZ96345.1|  hypothetical protein [Xanthophyllomyces dendrorh...  1075    0.0  


 >emb|CDZ96345.1| hypothetical protein [Xanthophyllomyces dendrorhous]
Length=597

 Score = 1075 bits (2780),  Expect = 0.0, Method: Compositional matrix adjust.
 Identities = 579/596 (97%), Positives = 581/596 (97%), Gaps = 9/596 (2%)

Query  58   LASSSTPSND----FLG-----DIDSASPYETTTGLIRSDDSDEGTRFSEKRIIAFHEQL  108
            LA S+TP +      LG       DSASPYETTTGLIRSDDSDEGTRFSEKRIIAFHEQL
Sbjct  2    LAQSNTPRHQQQPLLLGLRICLQHDSASPYETTTGLIRSDDSDEGTRFSEKRIIAFHEQL  61

Query  109  KSVALKSRWHVQDSQPRPLSVEQARSSISTVPLKLPKPCSAPDSIPAFLTESISQPSSSA  168
            KSVALKSRWHVQDSQPRPLSVEQARSSISTVPLKLPKPCSAPDSIPAFLTESISQPSSSA
Sbjct  62   KSVALKSRWHVQDSQPRPLSVEQARSSISTVPLKLPKPCSAPDSIPAFLTESISQPSSSA  121

Query  169  TPASHSTTQPSTQYIPVTLSQSTPVQPPQRTITSSTTSDQTSNPASKSKSESQLESNLTL  228
            TPASHSTTQPSTQYIPVTLSQSTPVQPPQRTITSSTTSDQTSNPASKSKSESQLESNLTL
Sbjct  122  TPASHSTTQPSTQYIPVTLSQSTPVQPPQRTITSSTTSDQTSNPASKSKSESQLESNLTL  181

Query  229  KPELEPKPGLEWSSQLQPVREESPLTPSIVSTIGSPRIPPSSSSQVHLSRRPVTSVSISR  288
            KPELEPKPGLEWSSQLQPVREESPLTPSIVSTIGSPRIPPSSSSQVHLSRRPVTSVSISR
Sbjct  182  KPELEPKPGLEWSSQLQPVREESPLTPSIVSTIGSPRIPPSSSSQVHLSRRPVTSVSISR  241

Query  289  NVPIASPKLMKAMAPTYGGNKAKPPNFLLPLGPRTPVAPLTLAQLQLTKSKAAASSEKNL  348
            NVPIASPKLMKAMAPTYGGNKAKPPNFLLPLGPRTPVAPLTLAQLQLTKSKAAASSEKNL
Sbjct  242  NVPIASPKLMKAMAPTYGGNKAKPPNFLLPLGPRTPVAPLTLAQLQLTKSKAAASSEKNL  301

Query  349  LRKKVLKAWNTHRRPENSWDKSSPKTSKHVSVTENADSENEFSINNLGISLSRRNSSPTS  408
            LRKKVLKAWNTHRRPENSWDKSSPKTSKHVSVTENADSENEFSINNLGISLSRRNSSPTS
Sbjct  302  LRKKVLKAWNTHRRPENSWDKSSPKTSKHVSVTENADSENEFSINNLGISLSRRNSSPTS  361

Query  409  AVRKLSESVNVPAQFMPLSSSSDTSLVFNNPHTALSNTLLDHLVREKRRLKSSISLAYAQ  468
            AVRKLSESVNVPAQFMPLSSSSDTSLVFNNPHTALSNTLLDHLVREKRRLKSSISLAYAQ
Sbjct  362  AVRKLSESVNVPAQFMPLSSSSDTSLVFNNPHTALSNTLLDHLVREKRRLKSSISLAYAQ  421

Query  469  YCIDTLNQSLGQLEVISSRDPSSSTSQAIPRLLRQYLHQYLLVPAFHTQTRQEVLDRMFR  528
            YCIDTLNQSLGQLEVISSRDPSSSTSQAIPRLLRQYLHQYLLVPAFHTQTRQEVLDRMFR
Sbjct  422  YCIDTLNQSLGQLEVISSRDPSSSTSQAIPRLLRQYLHQYLLVPAFHTQTRQEVLDRMFR  481

Query  529  LGLQPSVDLEILILRALFLRQKIYSKDFAPYGSFIVELSSLQTYFDKLLLGSDTNGRSNN  588
            LGLQPSVDLEILILRALFLRQKIYSKDFAPYGSFIVELSSLQTYFDKLLLGSDTNGRSNN
Sbjct  482  LGLQPSVDLEILILRALFLRQKIYSKDFAPYGSFIVELSSLQTYFDKLLLGSDTNGRSNN  541

Query  589  SSEQGTIDRRRIWSTMYSGFRSMAKNPIPTDLKARLLAEAQKEPVDLVLISKLSTV  644
            SSEQGTIDRRRIWSTMYSGFRSMAKNPIPTDLKARLLAEAQKEPVDLVLISKLSTV
Sbjct  542  SSEQGTIDRRRIWSTMYSGFRSMAKNPIPTDLKARLLAEAQKEPVDLVLISKLSTV  597


Lambda      K        H        a         alpha
   0.312    0.125    0.350    0.792     4.96 

Gapped
Lambda      K        H        a         alpha    sigma
   0.267   0.0410    0.140     1.90     42.6     43.6 

Effective search space used: 7232224246176


  Database: nr
    Posted date:  Sep 23, 2015 12:05 AM
  Number of letters in database: 26,053,659,533
  Number of sequences in database:  71,551,133


Matrix: BLOSUM62
Gap Penalties: Existence: 11, Extension: 1
Neighboring words threshold: 11
Window for multiple hits: 40
```
