# Supplementary material for: Comparative genomics provides new insights into the diversity, physiology, and sexuality of the only industrially exploited tremellomycete: Phaffia rhodozyma
Source: BMC Genomics. 2016 Nov 9;17:901. doi: 10.1186/s12864-016-3244-7 (PMC5103461; doi:10.1186/s12864-016-3244-7)
Supplement: Additional file 6: — List of orphan genes with links to PFAM (related to Additional file 1: Table S1). (ZIP 1428 kb) [file 12864_2016_3244_MOESM6_ESM.zip › BLAST_HTML_FTR/G04434_P.html]

BLAST Search Results


```
BLASTP 2.2.27+


Reference:
Stephen F. Altschul, Thomas L. Madden, Alejandro A. Schäffer,
Jinghui Zhang, Zheng Zhang, Webb Miller, and David J. Lipman (1997),
"Gapped BLAST and PSI-BLAST: a new generation of protein database
search programs", Nucleic Acids Res. 25:3389-3402.


Reference for
composition-based statistics:
Alejandro A. Schäffer, L. Aravind, Thomas L. Madden, Sergei
Shavirin, John L. Spouge, Yuri I. Wolf, Eugene V. Koonin, and
Stephen F. Altschul (2001), "Improving the accuracy of PSI-BLAST
protein database searches with composition-based statistics and
other refinements", Nucleic Acids Res. 29:2994-3005.


Database: nr
           71,551,133 sequences; 26,053,659,533 total letters


Query= G04434_P

Length=324
                                                                      Score     E
Sequences producing significant alignments:                          (Bits)  Value

emb|CED82676.1|  hypothetical protein [Xanthophyllomyces dendrorh...   659    0.0  
gb|ERI86223.1|  ferrous iron transport protein B [Bacteroides pyo...  40.4    2.8  
ref|WP_034543207.1|  iron transporter FeoB [Bacteroides pyogenes]...  40.4    2.8  
ref|WP_017211294.1|  chemotaxis protein CheA [Clostridium beijeri...  40.0    3.5  
ref|WP_041901209.1|  chemotaxis protein CheA [Clostridium beijeri...  39.7    3.8  


 >emb|CED82676.1| hypothetical protein [Xanthophyllomyces dendrorhous]
Length=323

 Score =  659 bits (1699),  Expect = 0.0, Method: Compositional matrix adjust.
 Identities = 322/323 (99%), Positives = 322/323 (99%), Gaps = 0/323 (0%)

Query  1    MNAFAQRSPLDIPELLYLVSNHLCANHSSDPASFRSILPLLTVNHLFHDTILPKLYTSIK  60
            MNAFAQRSPLDIPELLYLVSNHLCANHSSDPASFRSILPLLTVNHLFHDTILPKLYTSIK
Sbjct  1    MNAFAQRSPLDIPELLYLVSNHLCANHSSDPASFRSILPLLTVNHLFHDTILPKLYTSIK  60

Query  61   LAGYDAWQRFLKNGRPGWSSVLEFDLRITDQILESGLWNQFLNRLAQGRFRRLRVLHILF  120
            LAGYDAWQRFLKNGRPGWSSVLEFDLRITDQILESGLWNQFLNRLAQGRFRRLRVLHILF
Sbjct  61   LAGYDAWQRFLKNGRPGWSSVLEFDLRITDQILESGLWNQFLNRLAQGRFRRLRVLHILF  120

Query  121  NCSSAAREKLENVPTDGSEGGVNLEGIHLELTELKLDGTPSESLKRTLLSPIKMTYQHLS  180
            NCSSAAREKLENVPTDGSEGGVNLEGIHLELTELKLDGTPSESLKRTLLSPIKMTYQHLS
Sbjct  121  NCSSAAREKLENVPTDGSEGGVNLEGIHLELTELKLDGTPSESLKRTLLSPIKMTYQHLS  180

Query  181  VSIFQLQINPPNMSIPISVPTTSLRPSEDEQRPYGSMNVHHMKPTLWRRLPTSVERLSFL  240
            VSIFQLQINPPNMSIPI VPTTSLRPSEDEQRPYGSMNVHHMKPTLWRRLPTSVERLSFL
Sbjct  181  VSIFQLQINPPNMSIPIPVPTTSLRPSEDEQRPYGSMNVHHMKPTLWRRLPTSVERLSFL  240

Query  241  LSTPFPCPNSLTHPSWQGENAASQLKLITNFLTDLKERRALGQHLGLKTVDLSAFAGLSR  300
            LSTPFPCPNSLTHPSWQGENAASQLKLITNFLTDLKERRALGQHLGLKTVDLSAFAGLSR
Sbjct  241  LSTPFPCPNSLTHPSWQGENAASQLKLITNFLTDLKERRALGQHLGLKTVDLSAFAGLSR  300

Query  301  TWSGMFDVVVEAAEAMEGLEVLV  323
            TWSGMFDVVVEAAEAMEGLEVLV
Sbjct  301  TWSGMFDVVVEAAEAMEGLEVLV  323


>gb|ERI86223.1| ferrous iron transport protein B [Bacteroides pyogenes F0041]
Length=839

 Score = 40.4 bits (93),  Expect = 2.8, Method: Compositional matrix adjust.
 Identities = 35/113 (31%), Positives = 47/113 (42%), Gaps = 18/113 (16%)

Query  60   KLAGYDAWQRFLKNGRPGWSSVLEFDLRITDQILESGL------WNQFLNRLAQGRFRRL  113
            K+ GY+   R  + G     S  E    ITD +   GL        + L R+A G+ R +
Sbjct  64   KILGYEISLRRQEAGMIEVISEEEAQRNITDTVCREGLPEDVFVKEEALKRIALGKRRTI  123

Query  114  RVLHI---------LFNCSSAAREKLEN---VPTDGSEGGVNLEGIHLELTEL  154
             V  +         LFN +S A E + N   V  D  EG  N EG H  L +L
Sbjct  124  NVALVGNPNCGKTSLFNLASGAHEHVGNYSGVTVDAKEGYFNFEGYHFRLVDL  176


>ref|WP_034543207.1| iron transporter FeoB [Bacteroides pyogenes]
 dbj|GAE22510.1| ferrous iron transport protein B [Bacteroides pyogenes JCM 10003]
Length=828

 Score = 40.4 bits (93),  Expect = 2.8, Method: Compositional matrix adjust.
 Identities = 35/113 (31%), Positives = 47/113 (42%), Gaps = 18/113 (16%)

Query  60   KLAGYDAWQRFLKNGRPGWSSVLEFDLRITDQILESGL------WNQFLNRLAQGRFRRL  113
            K+ GY+   R  + G     S  E    ITD +   GL        + L R+A G+ R +
Sbjct  53   KILGYEISLRRQEAGMIEVISEEEAQRNITDTVCREGLPEDVFVKEEALKRIALGKRRTI  112

Query  114  RVLHI---------LFNCSSAAREKLEN---VPTDGSEGGVNLEGIHLELTEL  154
             V  +         LFN +S A E + N   V  D  EG  N EG H  L +L
Sbjct  113  NVALVGNPNCGKTSLFNLASGAHEHVGNYSGVTVDAKEGYFNFEGYHFRLVDL  165


>ref|WP_017211294.1| chemotaxis protein CheA [Clostridium beijerinckii]
Length=687

 Score = 40.0 bits (92),  Expect = 3.5, Method: Compositional matrix adjust.
 Identities = 29/105 (28%), Positives = 57/105 (54%), Gaps = 7/105 (7%)

Query  97   LWNQFLNRLAQGRFRRLRV----LHILFNCSSAAREKLENVPTDGSEGGVNLEGIHLELT  152
            L ++  + LA+ R  +L+V    + +LF+C     + ++NV  +GSE  ++++GI   L 
Sbjct  63   LTHKMEDVLAEFREGKLKVTQDVVTVLFDCLDTLEKMVDNVQ-EGSEEKIDIDGIMKALA  121

Query  153  ELKLDGTPSESLKRTLLSPIKMTYQH--LSVSIFQLQINPPNMSI  195
            ++K +G  S + + T  S IK   ++  +S   F L +N  + S+
Sbjct  122  DIKENGNKSNAQEETQASEIKSEDENKMISGDEFDLDLNQYDTSV  166


>ref|WP_041901209.1| chemotaxis protein CheA [Clostridium beijerinckii]
 gb|AJH01360.1| CheA signal transduction histidine kinase [Clostridium beijerinckii]
Length=687

 Score = 39.7 bits (91),  Expect = 3.8, Method: Compositional matrix adjust.
 Identities = 29/105 (28%), Positives = 57/105 (54%), Gaps = 7/105 (7%)

Query  97   LWNQFLNRLAQGRFRRLRV----LHILFNCSSAAREKLENVPTDGSEGGVNLEGIHLELT  152
            L ++  + LA+ R  +L+V    + +LF+C     + ++NV  +GSE  ++++GI   L 
Sbjct  63   LTHKMEDVLAEFREGKLKVTQDVVTVLFDCLDTLEKMVDNVQ-EGSEEKIDIDGIMKALA  121

Query  153  ELKLDGTPSESLKRTLLSPIKMTYQH--LSVSIFQLQINPPNMSI  195
            ++K +G  S+  + T  S IK   ++  +S   F L +N  + S+
Sbjct  122  DIKENGNKSDVQEETQASEIKSEDENKMISGDEFDLDLNQYDTSV  166


Lambda      K        H        a         alpha
   0.321    0.136    0.412    0.792     4.96 

Gapped
Lambda      K        H        a         alpha    sigma
   0.267   0.0410    0.140     1.90     42.6     43.6 

Effective search space used: 2665852187442


  Database: nr
    Posted date:  Sep 23, 2015 12:05 AM
  Number of letters in database: 26,053,659,533
  Number of sequences in database:  71,551,133


Matrix: BLOSUM62
Gap Penalties: Existence: 11, Extension: 1
Neighboring words threshold: 11
Window for multiple hits: 40
```
