# Supplementary material for: Comparative genomics provides new insights into the diversity, physiology, and sexuality of the only industrially exploited tremellomycete: Phaffia rhodozyma
Source: BMC Genomics. 2016 Nov 9;17:901. doi: 10.1186/s12864-016-3244-7 (PMC5103461; doi:10.1186/s12864-016-3244-7)
Supplement: Additional file 6: — List of orphan genes with links to PFAM (related to Additional file 1: Table S1). (ZIP 1428 kb) [file 12864_2016_3244_MOESM6_ESM.zip › BLAST_HTML_FTR/G04975_P.html]

BLAST Search Results


```
BLASTP 2.2.27+


Reference:
Stephen F. Altschul, Thomas L. Madden, Alejandro A. Schäffer,
Jinghui Zhang, Zheng Zhang, Webb Miller, and David J. Lipman (1997),
"Gapped BLAST and PSI-BLAST: a new generation of protein database
search programs", Nucleic Acids Res. 25:3389-3402.


Reference for
composition-based statistics:
Alejandro A. Schäffer, L. Aravind, Thomas L. Madden, Sergei
Shavirin, John L. Spouge, Yuri I. Wolf, Eugene V. Koonin, and
Stephen F. Altschul (2001), "Improving the accuracy of PSI-BLAST
protein database searches with composition-based statistics and
other refinements", Nucleic Acids Res. 29:2994-3005.


Database: nr
           71,551,133 sequences; 26,053,659,533 total letters


Query= G04975_P

Length=446
                                                                      Score     E
Sequences producing significant alignments:                          (Bits)  Value

emb|CDZ97499.1|  hypothetical protein [Xanthophyllomyces dendrorh...   905    0.0  
ref|XP_007262820.1|  hypothetical protein FOMMEDRAFT_165323 [Fomi...  41.2    2.5  


 >emb|CDZ97499.1| hypothetical protein [Xanthophyllomyces dendrorhous]
Length=445

 Score =  905 bits (2340),  Expect = 0.0, Method: Compositional matrix adjust.
 Identities = 441/445 (99%), Positives = 441/445 (99%), Gaps = 0/445 (0%)

Query  1    MNEHLSRSQIYLQTTTTTTIATFMPPSSSIKPLPERPFRSTPKGLSGMLSSSSRKRPAED  60
            MNEHLSRSQIYLQTTTTTTIATFMPPSSSIKPLPERPFRSTPKGLSGMLSSSSRKRPAED
Sbjct  1    MNEHLSRSQIYLQTTTTTTIATFMPPSSSIKPLPERPFRSTPKGLSGMLSSSSRKRPAED  60

Query  61   QVEGRSSRARLFEIPDSGSPSSCLVAQKETQNIKPFSISLTPQGGTARQELSLSTPANDR  120
            QVEGRSSRARLFEIPDSGSPSSCLVAQKETQNIKPFSISLTPQGGTARQELSLSTPANDR
Sbjct  61   QVEGRSSRARLFEIPDSGSPSSCLVAQKETQNIKPFSISLTPQGGTARQELSLSTPANDR  120

Query  121  ASVVHQSAQIQSSSASVSSIVSKGAKPVIRPSLHFSTDPLIGEDFQHKSMRLAHRRAIQP  180
            ASVVHQSAQIQSSSASVSSIVSKGAKPVIRPSLHFSTDPLIGEDFQHKSMRLAHRRAIQP
Sbjct  121  ASVVHQSAQIQSSSASVSSIVSKGAKPVIRPSLHFSTDPLIGEDFQHKSMRLAHRRAIQP  180

Query  181  PPPMTPISNPRSSPHSYAIKTQDSRRLNNQLSPDIEDDYSLSEDGYMHQETGGVQFGKTP  240
            PPPMTPISNPRSSPHSYAIKTQDSRRLNNQLSPDIEDDYSLSEDGYMHQETGGVQFGKTP
Sbjct  181  PPPMTPISNPRSSPHSYAIKTQDSRRLNNQLSPDIEDDYSLSEDGYMHQETGGVQFGKTP  240

Query  241  YAVRRDHEGLPAGIFDEDDEDDMFMQLGAEQLLMFQDKKGQADEEVYRGRSVDEDVELDE  300
            YAVRRDHEGLPAGIFDEDDEDDMFMQLGAEQLLMFQDKK QADEE YRGRSVDEDVELDE
Sbjct  241  YAVRRDHEGLPAGIFDEDDEDDMFMQLGAEQLLMFQDKKDQADEEAYRGRSVDEDVELDE  300

Query  301  RQHGFRGKVKFDHTEEGIAEGRFEDDIENTYRGHPIFSSDDHRSTNSLVMLKGGKIGEAY  360
            RQHGFRGK KFDHTEE IAEGRFEDDIENTYRGHPIFSSDDHRSTNSLVMLKGGKIGEAY
Sbjct  301  RQHGFRGKAKFDHTEEDIAEGRFEDDIENTYRGHPIFSSDDHRSTNSLVMLKGGKIGEAY  360

Query  361  EKMKDKYAQETLTAEQWETRGKDLSTRFDSLAQKMVQHTKERIEIHRAATEKLEAYDLSL  420
            EKMKDKYAQETLTAEQWETRGKDLSTRFDSLAQKMVQHTKERIEIHRAATEKLEAYDLSL
Sbjct  361  EKMKDKYAQETLTAEQWETRGKDLSTRFDSLAQKMVQHTKERIEIHRAATEKLEAYDLSL  420

Query  421  SNRSAVLETIQRGLAGKASSLLDRS  445
            SNRSAVLETIQRGLAGKASSLLDRS
Sbjct  421  SNRSAVLETIQRGLAGKASSLLDRS  445


>ref|XP_007262820.1| hypothetical protein FOMMEDRAFT_165323 [Fomitiporia mediterranea 
MF3/22]
 gb|EJD06556.1| hypothetical protein FOMMEDRAFT_165323 [Fomitiporia mediterranea 
MF3/22]
Length=516

 Score = 41.2 bits (95),  Expect = 2.5, Method: Compositional matrix adjust.
 Identities = 20/71 (28%), Positives = 37/71 (52%), Gaps = 2/71 (3%)

Query  358  EAYEKMKDKYAQETLTAEQWETRGKDLSTRFDSLAQKMVQHTKERIEIHRAATEKLEAYD  417
            E YE  K ++   ++  E W+   K++S RF  L   +  H   ++E+H    EK+  + 
Sbjct  426  EKYEAAKKRWTDCSM--EDWKAGAKEMSERFGKLVDFVKDHMTTKLEVHATLHEKVVQHK  483

Query  418  LSLSNRSAVLE  428
            + LS R ++L+
Sbjct  484  VVLSERQSMLK  494


Lambda      K        H        a         alpha
   0.313    0.129    0.361    0.792     4.96 

Gapped
Lambda      K        H        a         alpha    sigma
   0.267   0.0410    0.140     1.90     42.6     43.6 

Effective search space used: 4390157234892


  Database: nr
    Posted date:  Sep 23, 2015 12:05 AM
  Number of letters in database: 26,053,659,533
  Number of sequences in database:  71,551,133


Matrix: BLOSUM62
Gap Penalties: Existence: 11, Extension: 1
Neighboring words threshold: 11
Window for multiple hits: 40
```
