# Supplementary material for: Comparative genomics provides new insights into the diversity, physiology, and sexuality of the only industrially exploited tremellomycete: Phaffia rhodozyma
Source: BMC Genomics. 2016 Nov 9;17:901. doi: 10.1186/s12864-016-3244-7 (PMC5103461; doi:10.1186/s12864-016-3244-7)
Supplement: Additional file 6: — List of orphan genes with links to PFAM (related to Additional file 1: Table S1). (ZIP 1428 kb) [file 12864_2016_3244_MOESM6_ESM.zip › BLAST_HTML_FTR/G04056_P.html]

BLAST Search Results


```
BLASTP 2.2.27+


Reference:
Stephen F. Altschul, Thomas L. Madden, Alejandro A. Schäffer,
Jinghui Zhang, Zheng Zhang, Webb Miller, and David J. Lipman (1997),
"Gapped BLAST and PSI-BLAST: a new generation of protein database
search programs", Nucleic Acids Res. 25:3389-3402.


Reference for
composition-based statistics:
Alejandro A. Schäffer, L. Aravind, Thomas L. Madden, Sergei
Shavirin, John L. Spouge, Yuri I. Wolf, Eugene V. Koonin, and
Stephen F. Altschul (2001), "Improving the accuracy of PSI-BLAST
protein database searches with composition-based statistics and
other refinements", Nucleic Acids Res. 29:2994-3005.


Database: nr
           71,551,133 sequences; 26,053,659,533 total letters


Query= G04056_P

Length=258
                                                                      Score     E
Sequences producing significant alignments:                          (Bits)  Value

emb|CED84825.1|  hypothetical protein [Xanthophyllomyces dendrorh...   509    1e-180
emb|CED83732.1|  hypothetical protein [Xanthophyllomyces dendrorh...  94.7    4e-19 
ref|WP_035916719.1|  LuxR family transcriptional regulator [Fusob...  38.5    4.4   
ref|XP_007263070.1|  NAD-binding protein [Fomitiporia mediterrane...  38.1    6.2   
ref|WP_025048265.1|  C4-dicarboxylate ABC transporter substrate-b...  37.4    9.5   
ref|WP_025044531.1|  C4-dicarboxylate ABC transporter substrate-b...  37.4    9.5   
ref|WP_005958371.1|  LuxR family transcriptional regulator [Fusob...  37.4    9.6   
ref|WP_037907941.1|  C4-dicarboxylate ABC transporter substrate-b...  37.4    9.7   


 >emb|CED84825.1| hypothetical protein [Xanthophyllomyces dendrorhous]
Length=257

 Score =  509 bits (1310),  Expect = 1e-180, Method: Compositional matrix adjust.
 Identities = 257/257 (100%), Positives = 257/257 (100%), Gaps = 0/257 (0%)

Query  1    MLAFSAISLGLALFASAVQAAPVSENAEGMVARYDAHSSSSSKSGSASILGDVVSLTGSD  60
            MLAFSAISLGLALFASAVQAAPVSENAEGMVARYDAHSSSSSKSGSASILGDVVSLTGSD
Sbjct  1    MLAFSAISLGLALFASAVQAAPVSENAEGMVARYDAHSSSSSKSGSASILGDVVSLTGSD  60

Query  61   RSASSSYHNRDLLDLNLDAEVSATLQALGLLSAKANVDVSATVAAELAVSGLSRKKDHSL  120
            RSASSSYHNRDLLDLNLDAEVSATLQALGLLSAKANVDVSATVAAELAVSGLSRKKDHSL
Sbjct  61   RSASSSYHNRDLLDLNLDAEVSATLQALGLLSAKANVDVSATVAAELAVSGLSRKKDHSL  120

Query  121  HYTCPTKGWKAPPAYEFGYWHPTTGVWINDKTAVDAYLSVQGYVHLGLDVVVDLFVDIDA  180
            HYTCPTKGWKAPPAYEFGYWHPTTGVWINDKTAVDAYLSVQGYVHLGLDVVVDLFVDIDA
Sbjct  121  HYTCPTKGWKAPPAYEFGYWHPTTGVWINDKTAVDAYLSVQGYVHLGLDVVVDLFVDIDA  180

Query  181  TVAAVASGVIASRPADLTKKGKCGYWVPKSTTAVVDVVASLEAAVYVDADVSVEALLNTC  240
            TVAAVASGVIASRPADLTKKGKCGYWVPKSTTAVVDVVASLEAAVYVDADVSVEALLNTC
Sbjct  181  TVAAVASGVIASRPADLTKKGKCGYWVPKSTTAVVDVVASLEAAVYVDADVSVEALLNTC  240

Query  241  GFFHLSADAQAAIAATA  257
            GFFHLSADAQAAIAATA
Sbjct  241  GFFHLSADAQAAIAATA  257


>emb|CED83732.1| hypothetical protein [Xanthophyllomyces dendrorhous]
Length=335

 Score = 94.7 bits (234),  Expect = 4e-19, Method: Compositional matrix adjust.
 Identities = 63/210 (30%), Positives = 89/210 (42%), Gaps = 67/210 (32%)

Query  98   DVSATVAAELAVSGLSRKKDHSLHYTCPTKGWKAPPAYEFGYWHPTTGVWINDKTAVDAY  157
            D+SA + A+L+V GL +  D++L Y CP K W  P  Y FGY+   TG W++D+  V  Y
Sbjct  123  DISANIKAQLSVDGLFKTPDNTLAYQCPDKQWAPPAQYNFGYFDVDTGAWVDDQAQVQTY  182

Query  158  LSVQGYVHLGLDVVVDLFVDIDATVAAVASGVIASRPADLTKKGKCGYW-----------  206
            L   GY HL +   +DLF   +   A + +G       +   KGKCGY            
Sbjct  183  LQGLGYAHLDISGSLDLFAHANVNAAILPTG------DNTQNKGKCGYLVPAAPAAPATK  236

Query  207  ----------------VPKSTTA--VVDVVASLEAAVYVDADVS----------------  232
                            VP  TTA  V+DV A++  A+  +ADV+                
Sbjct  237  AAVATPSSACDVASTAVPTGTTAAGVLDVSATIFVALQANADVNLGLSADVDADVDADIN  296

Query  233  ----------------VEALLNTCGFFHLS  246
                            V+ LLN+ GFF L+
Sbjct  297  ANVDANLNAQADVSVDVKTLLNSSGFFQLA  326


>ref|WP_035916719.1| LuxR family transcriptional regulator [Fusobacterium necrophorum]
 gb|KDE63784.1| LuxR family transcriptional regulator [Fusobacterium necrophorum 
BL]
 gb|KDE70409.1| LuxR family transcriptional regulator [Fusobacterium necrophorum 
BFTR-2]
Length=274

 Score = 38.5 bits (88),  Expect = 4.4, Method: Compositional matrix adjust.
 Identities = 24/77 (31%), Positives = 37/77 (48%), Gaps = 1/77 (1%)

Query  30   MVARYDAHSSSSSKSGSASILGDVVSLTGSDRSASSSYHNRDLLDLNLDAEVSATLQALG  89
            +V  YD      +K     I GD  +L+ SD++  S Y++RD +   LD +   T+   G
Sbjct  160  LVKVYDYFQDKDTKENDILIGGDF-NLSASDKAFDSLYNHRDNITCTLDPKTKTTVGTKG  218

Query  90   LLSAKANVDVSATVAAE  106
            L SA  N+ +S     E
Sbjct  219  LSSAYDNIFISKKYTTE  235


>ref|XP_007263070.1| NAD-binding protein [Fomitiporia mediterranea MF3/22]
 gb|EJD06806.1| NAD-binding protein [Fomitiporia mediterranea MF3/22]
Length=261

 Score = 38.1 bits (87),  Expect = 6.2, Method: Compositional matrix adjust.
 Identities = 33/128 (26%), Positives = 51/128 (40%), Gaps = 13/128 (10%)

Query  126  TKGWKAPPAYEFGYWHPTTGVWINDKTAVDAYLSVQGYVHLGLDVVVDLFVDIDATVAAV  185
            T+  +A P   F  + P   +       +  Y         G+DV V+  V     + AV
Sbjct  6    TRDDQATPVTPFTSYTPRVAIVTGSAQGI-GYAIAHRLADDGIDVAVNDLVSKQDQIDAV  64

Query  186  ASGVIASRPADLTKKGKCGYWVPKSTTAVVDVVASLEAAVY----VDADVSVEALLNTCG  241
                      +L KKG+C   VP   ++  DV+A +E  V     VD  V+  A+   C 
Sbjct  65   VD--------ELRKKGRCAIAVPGDISSEADVIAIVEKTVQELGSVDIMVANAAISQLCS  116

Query  242  FFHLSADA  249
            F H S ++
Sbjct  117  FLHTSVES  124


>ref|WP_025048265.1| C4-dicarboxylate ABC transporter substrate-binding protein [Sulfitobacter 
mediterraneus]
 gb|KIN77352.1| TRAP transporter, DctQ subunit [Sulfitobacter mediterraneus KCTC 
32188]
Length=226

 Score = 37.4 bits (85),  Expect = 9.5, Method: Compositional matrix adjust.
 Identities = 19/44 (43%), Positives = 25/44 (57%), Gaps = 0/44 (0%)

Query  165  HLGLDVVVDLFVDIDATVAAVASGVIASRPADLTKKGKCGYWVP  208
            HLG+D V++LF +    V AV +G+I    A L  KG   YW P
Sbjct  76   HLGVDAVINLFNEGPRKVLAVIAGLICVIYAGLLMKGAWDYWAP  119


>ref|WP_025044531.1| C4-dicarboxylate ABC transporter substrate-binding protein [Sulfitobacter 
geojensis]
 gb|KHA52137.1| TRAP dicarboxylate transporter, DctP subunit [Sulfitobacter geojensis]
Length=226

 Score = 37.4 bits (85),  Expect = 9.5, Method: Compositional matrix adjust.
 Identities = 19/44 (43%), Positives = 25/44 (57%), Gaps = 0/44 (0%)

Query  165  HLGLDVVVDLFVDIDATVAAVASGVIASRPADLTKKGKCGYWVP  208
            HLG+D V++LF +    V A+ +GVI    A L  KG   YW P
Sbjct  76   HLGVDAVINLFDEGPRKVLAILAGVICVIYAGLLMKGAWDYWAP  119


>ref|WP_005958371.1| LuxR family transcriptional regulator [Fusobacterium necrophorum]
 gb|EIJ69905.1| endonuclease/exonuclease/phosphatase family protein [Fusobacterium 
necrophorum subsp. funduliforme ATCC 51357]
Length=274

 Score = 37.4 bits (85),  Expect = 9.6, Method: Compositional matrix adjust.
 Identities = 24/77 (31%), Positives = 36/77 (47%), Gaps = 1/77 (1%)

Query  30   MVARYDAHSSSSSKSGSASILGDVVSLTGSDRSASSSYHNRDLLDLNLDAEVSATLQALG  89
            +V  YD       K     I GD  +L+ SD++  S Y++RD +   LD +   T+   G
Sbjct  160  LVKVYDYFQDRDIKENDILIAGDF-NLSASDKAFDSLYNHRDNITCTLDPKTKTTVGTKG  218

Query  90   LLSAKANVDVSATVAAE  106
            L SA  N+ +S     E
Sbjct  219  LSSAYDNIFISKKYTTE  235


>ref|WP_037907941.1| C4-dicarboxylate ABC transporter substrate-binding protein [Sulfitobacter 
mediterraneus]
 gb|KAJ03269.1| C4-dicarboxylate ABC transporter substrate-binding protein [Sulfitobacter 
mediterraneus]
Length=226

 Score = 37.4 bits (85),  Expect = 9.7, Method: Compositional matrix adjust.
 Identities = 19/44 (43%), Positives = 25/44 (57%), Gaps = 0/44 (0%)

Query  165  HLGLDVVVDLFVDIDATVAAVASGVIASRPADLTKKGKCGYWVP  208
            HLG+D V++LF +    V AV +G+I    A L  KG   YW P
Sbjct  76   HLGVDAVINLFNEGPRKVLAVIAGLICVIYAGLLMKGAWDYWAP  119


Lambda      K        H        a         alpha
   0.317    0.128    0.368    0.792     4.96 

Gapped
Lambda      K        H        a         alpha    sigma
   0.267   0.0410    0.140     1.90     42.6     43.6 

Effective search space used: 1724456371002


  Database: nr
    Posted date:  Sep 23, 2015 12:05 AM
  Number of letters in database: 26,053,659,533
  Number of sequences in database:  71,551,133


Matrix: BLOSUM62
Gap Penalties: Existence: 11, Extension: 1
Neighboring words threshold: 11
Window for multiple hits: 40
```
