# Supplementary material for: Comparative genomics provides new insights into the diversity, physiology, and sexuality of the only industrially exploited tremellomycete: Phaffia rhodozyma
Source: BMC Genomics. 2016 Nov 9;17:901. doi: 10.1186/s12864-016-3244-7 (PMC5103461; doi:10.1186/s12864-016-3244-7)
Supplement: Additional file 6: — List of orphan genes with links to PFAM (related to Additional file 1: Table S1). (ZIP 1428 kb) [file 12864_2016_3244_MOESM6_ESM.zip › BLAST_HTML_FTR/G01806_P.html]

BLAST Search Results


```
BLASTP 2.2.27+


Reference:
Stephen F. Altschul, Thomas L. Madden, Alejandro A. Schäffer,
Jinghui Zhang, Zheng Zhang, Webb Miller, and David J. Lipman (1997),
"Gapped BLAST and PSI-BLAST: a new generation of protein database
search programs", Nucleic Acids Res. 25:3389-3402.


Reference for
composition-based statistics:
Alejandro A. Schäffer, L. Aravind, Thomas L. Madden, Sergei
Shavirin, John L. Spouge, Yuri I. Wolf, Eugene V. Koonin, and
Stephen F. Altschul (2001), "Improving the accuracy of PSI-BLAST
protein database searches with composition-based statistics and
other refinements", Nucleic Acids Res. 29:2994-3005.


Database: nr
           71,551,133 sequences; 26,053,659,533 total letters


Query= G01806_P

Length=422
                                                                      Score     E
Sequences producing significant alignments:                          (Bits)  Value

emb|CED83648.1|  hypothetical protein [Xanthophyllomyces dendrorh...   838    0.0  
ref|XP_007849326.1|  hypothetical protein Moror_2759 [Moniliophth...  45.4    0.087


 >emb|CED83648.1| hypothetical protein [Xanthophyllomyces dendrorhous]
Length=421

 Score =  838 bits (2166),  Expect = 0.0, Method: Compositional matrix adjust.
 Identities = 421/421 (100%), Positives = 421/421 (100%), Gaps = 0/421 (0%)

Query  1    MAQCTTSTLTLYGTTTVNSVSGPSVTSQAVYTVPGVETTSYSSICESTGQGILGVPGGCQ  60
            MAQCTTSTLTLYGTTTVNSVSGPSVTSQAVYTVPGVETTSYSSICESTGQGILGVPGGCQ
Sbjct  1    MAQCTTSTLTLYGTTTVNSVSGPSVTSQAVYTVPGVETTSYSSICESTGQGILGVPGGCQ  60

Query  61   GYRTETFVGTGTPTVMTSTVIVPAEQTGAGAVSTYPVSTTVITSCPSGSTGSSTIGNKQD  120
            GYRTETFVGTGTPTVMTSTVIVPAEQTGAGAVSTYPVSTTVITSCPSGSTGSSTIGNKQD
Sbjct  61   GYRTETFVGTGTPTVMTSTVIVPAEQTGAGAVSTYPVSTTVITSCPSGSTGSSTIGNKQD  120

Query  121  ASVSNLAVSGTSTGQSSATPSVQGPSSVGSTVMAVATSVGMDTKTVQVVVTGADGEASVS  180
            ASVSNLAVSGTSTGQSSATPSVQGPSSVGSTVMAVATSVGMDTKTVQVVVTGADGEASVS
Sbjct  121  ASVSNLAVSGTSTGQSSATPSVQGPSSVGSTVMAVATSVGMDTKTVQVVVTGADGEASVS  180

Query  181  TSFQEFSTVYVYTSFVSAGVASSNTIVAASTSASSSSNSSSDSSVNSPGGLAAGIVAGIV  240
            TSFQEFSTVYVYTSFVSAGVASSNTIVAASTSASSSSNSSSDSSVNSPGGLAAGIVAGIV
Sbjct  181  TSFQEFSTVYVYTSFVSAGVASSNTIVAASTSASSSSNSSSDSSVNSPGGLAAGIVAGIV  240

Query  241  FVFAVGLVFWAVLRKSKQNEWSTDDSVLDDPSEKIDTLSIGGAVQRQVTLNRRHQENLSN  300
            FVFAVGLVFWAVLRKSKQNEWSTDDSVLDDPSEKIDTLSIGGAVQRQVTLNRRHQENLSN
Sbjct  241  FVFAVGLVFWAVLRKSKQNEWSTDDSVLDDPSEKIDTLSIGGAVQRQVTLNRRHQENLSN  300

Query  301  TAQQLNASPPNSLFMERIVVSNMSRSRSLRYAPLPGDSPRLDRGPSQSSSRHPHPKLQAH  360
            TAQQLNASPPNSLFMERIVVSNMSRSRSLRYAPLPGDSPRLDRGPSQSSSRHPHPKLQAH
Sbjct  301  TAQQLNASPPNSLFMERIVVSNMSRSRSLRYAPLPGDSPRLDRGPSQSSSRHPHPKLQAH  360

Query  361  AHAHPQAHGQLHRQPSRPSTGPRAEETGSYRPVDGTRILSDGSGRSMSMVSEEEGPPRYQ  420
            AHAHPQAHGQLHRQPSRPSTGPRAEETGSYRPVDGTRILSDGSGRSMSMVSEEEGPPRYQ
Sbjct  361  AHAHPQAHGQLHRQPSRPSTGPRAEETGSYRPVDGTRILSDGSGRSMSMVSEEEGPPRYQ  420

Query  421  I  421
            I
Sbjct  421  I  421


>ref|XP_007849326.1| hypothetical protein Moror_2759 [Moniliophthora roreri MCA 2997]
 gb|ESK91355.1| hypothetical protein Moror_2759 [Moniliophthora roreri MCA 2997]
Length=414

 Score = 45.4 bits (106),  Expect = 0.087, Method: Compositional matrix adjust.
 Identities = 44/159 (28%), Positives = 72/159 (45%), Gaps = 25/159 (16%)

Query  203  SNTIVAASTSASSSSNSSSDSSVNSPGGLAAGIVAGI-VFVFAVGLVFWAVLRKSKQNEW  261
            S T + A   +SS S +   SS N+ G +  G+++GI V + AV  V++   RK ++N+ 
Sbjct  163  STTTMPAEIQSSSPSLAVERSSKNNTGPIIGGVISGIAVLILAVIAVWYIFKRKRRRNDV  222

Query  262  STDDSVLDDPS-----EKIDTL--------------SIGGAVQRQVTLNRRHQENLSNTA  302
              D  V D P+     + +DT+              ++   V R   +  R + N S T 
Sbjct  223  FDDIDVNDFPAAVTNNDNVDTVPNPYVYDPLATQFATLSDRVHRGALMVARRKHNSSTTP  282

Query  303  QQLNASPPNSLFMERIVVSNMSRSRSLRYAPLPGDSPRL  341
             Q + +  + L M     S+MSR+ S    P  G SP +
Sbjct  283  TQWSPTSSHQLLM-----SDMSRANSPPSTPGHGRSPSI  316


Lambda      K        H        a         alpha
   0.309    0.123    0.343    0.792     4.96 

Gapped
Lambda      K        H        a         alpha    sigma
   0.267   0.0410    0.140     1.90     42.6     43.6 

Effective search space used: 4063604433496


  Database: nr
    Posted date:  Sep 23, 2015 12:05 AM
  Number of letters in database: 26,053,659,533
  Number of sequences in database:  71,551,133


Matrix: BLOSUM62
Gap Penalties: Existence: 11, Extension: 1
Neighboring words threshold: 11
Window for multiple hits: 40
```
