# Supplementary material for: Comparative genomics provides new insights into the diversity, physiology, and sexuality of the only industrially exploited tremellomycete: Phaffia rhodozyma
Source: BMC Genomics. 2016 Nov 9;17:901. doi: 10.1186/s12864-016-3244-7 (PMC5103461; doi:10.1186/s12864-016-3244-7)
Supplement: Additional file 6: — List of orphan genes with links to PFAM (related to Additional file 1: Table S1). (ZIP 1428 kb) [file 12864_2016_3244_MOESM6_ESM.zip › BLAST_HTML_FTR/G05174_P.html]

BLAST Search Results


```
BLASTP 2.2.27+


Reference:
Stephen F. Altschul, Thomas L. Madden, Alejandro A. Schäffer,
Jinghui Zhang, Zheng Zhang, Webb Miller, and David J. Lipman (1997),
"Gapped BLAST and PSI-BLAST: a new generation of protein database
search programs", Nucleic Acids Res. 25:3389-3402.


Reference for
composition-based statistics:
Alejandro A. Schäffer, L. Aravind, Thomas L. Madden, Sergei
Shavirin, John L. Spouge, Yuri I. Wolf, Eugene V. Koonin, and
Stephen F. Altschul (2001), "Improving the accuracy of PSI-BLAST
protein database searches with composition-based statistics and
other refinements", Nucleic Acids Res. 29:2994-3005.


Database: nr
           71,551,133 sequences; 26,053,659,533 total letters


Query= G05174_P

Length=240
                                                                      Score     E
Sequences producing significant alignments:                          (Bits)  Value

emb|CED85597.1|  hypothetical protein [Xanthophyllomyces dendrorh...   437    4e-152
ref|WP_026976448.1|  hypothetical protein [Flavobacterium tegetin...  40.8    0.98  
gb|KKR81113.1|  hypothetical protein UU26_C0005G0042 [Microgenoma...  38.9    1.7   
gb|EJO71178.1|  intein N-terminal splicing domain protein [Leptos...  39.3    3.9   
ref|WP_025175841.1|  intein splicing protein [Leptospira kirschneri]  39.3    4.1   


 >emb|CED85597.1| hypothetical protein [Xanthophyllomyces dendrorhous]
Length=277

 Score =  437 bits (1123),  Expect = 4e-152, Method: Compositional matrix adjust.
 Identities = 238/266 (89%), Positives = 239/266 (90%), Gaps = 27/266 (10%)

Query  1    MARSTIHPFVGILGYVESLALVPFVAVLIPTIAFVQLCQDTGVAGDSQKVTNDNRTALLN  60
            MARSTIHPFVGILGYVESLALVPFVAVLIPTIAFVQLCQDTGVAGDSQKVTNDNRTALLN
Sbjct  12   MARSTIHPFVGILGYVESLALVPFVAVLIPTIAFVQLCQDTGVAGDSQKVTNDNRTALLN  71

Query  61   ATGSHERTRSQITIDDSHPLMSMSKTEQSLDVVDRFDGSLHLIIDDVYDPVYDSRTSQSD  120
            ATGSHERTRSQITIDDSHPLMSMSKTEQSLDVVDRFDGSLHLIIDDVYDPVYDSRTSQSD
Sbjct  72   ATGSHERTRSQITIDDSHPLMSMSKTEQSLDVVDRFDGSLHLIIDDVYDPVYDSRTSQSD  131

Query  121  ITSSSGS---------------------------KTEHIPIKLQMEETSTDMPLEDDVNP  153
            ITSSSGS                           +TEHIPIKLQMEETSTDMPLEDDVNP
Sbjct  132  ITSSSGSNSTIIEREGSLAGPDISDEIHPYETHQETEHIPIKLQMEETSTDMPLEDDVNP  191

Query  154  IRIDQTQGAETHYESFRMGHPIETVIRLGMDVENFVERDVEISEQRASPDKHQVSPRGKQ  213
            IRIDQTQGAETHYESFRMGHPIETVIRLGMDVENFVERDVEISEQRASPDKHQVSPRGKQ
Sbjct  192  IRIDQTQGAETHYESFRMGHPIETVIRLGMDVENFVERDVEISEQRASPDKHQVSPRGKQ  251

Query  214  SKKNRKKKLKAKEKVREVKQSGPDKV  239
            SKKNRKKKLKAKEKVREVKQSGPDKV
Sbjct  252  SKKNRKKKLKAKEKVREVKQSGPDKV  277


>ref|WP_026976448.1| hypothetical protein [Flavobacterium tegetincola]
Length=462

 Score = 40.8 bits (94),  Expect = 0.98, Method: Compositional matrix adjust.
 Identities = 21/77 (27%), Positives = 39/77 (51%), Gaps = 0/77 (0%)

Query  10   VGILGYVESLALVPFVAVLIPTIAFVQLCQDTGVAGDSQKVTNDNRTALLNATGSHERTR  69
            +GI+G+ E   + P +A +   IAF+ L   TGVAGD   V  + +   +N     +   
Sbjct  242  IGIIGHSEGGTIAPMLAAIDKNIAFIVLLAGTGVAGDELLVDQNYQVGKINGMTEEQLAA  301

Query  70   SQITIDDSHPLMSMSKT  86
            ++IT  + + ++  + T
Sbjct  302  AKITNQEIYAIVKGTGT  318


>gb|KKR81113.1| hypothetical protein UU26_C0005G0042 [Microgenomates (Daviesbacteria) 
bacterium GW2011_GWC1_40_9]
 gb|KKR82173.1| hypothetical protein UU29_C0017G0009 [Microgenomates (Daviesbacteria) 
bacterium GW2011_GWA2_40_9]
 gb|KKR93635.1| hypothetical protein UU44_C0002G0296 [Microgenomates (Daviesbacteria) 
bacterium GW2011_GWB1_41_15]
 gb|KKS14814.1| hypothetical protein UU73_C0003G0013 [Microgenomates (Daviesbacteria) 
bacterium GW2011_GWA1_41_61]
Length=166

 Score = 38.9 bits (89),  Expect = 1.7, Method: Compositional matrix adjust.
 Identities = 44/158 (28%), Positives = 75/158 (47%), Gaps = 23/158 (15%)

Query  9    FVGILGYVESLALVPFVAVLIPTIAFVQLCQDTGVAGDSQKVTNDNRTALLNATGSHERT  68
            +V I+  V S AL+PF   +I T   V+   +  V   ++ V+   +  + NATG +  T
Sbjct  15   YVAIMAIV-STALIPFAWNIIGT--GVKSAAEQEVFSQARVVSERIKYEIRNATGINNVT  71

Query  69   RSQITIDDSHPLMSMSKTEQSLD--VVDRFDGSLHLIIDDVYDPVYDSRTSQSDITS---  123
             S I         S++K+E SL+  V+D   G +   I        +  +S +++TS   
Sbjct  72   SSSI---------SLAKSEASLNPTVIDFLAGKIR--ISQGGGGAVNLNSSDTNMTSLTF  120

Query  124  ----SSGSKTEHIPIKLQMEETSTDMPLEDDVNPIRID  157
                S+ +KT+HI     ME+  T +  E +V PI ++
Sbjct  121  TNYTSADNKTKHIQFIFTMEDNYTGLRQEYNVPPITVE  158


>gb|EJO71178.1| intein N-terminal splicing domain protein [Leptospira kirschneri 
serovar Grippotyphosa str. RM52]
Length=3060

 Score = 39.3 bits (90),  Expect = 3.9, Method: Composition-based stats.
 Identities = 29/137 (21%), Positives = 64/137 (47%), Gaps = 10/137 (7%)

Query  101   HLIIDDVYDPVYDSRTSQSDITSSSGSKTEHIPIKLQMEETSTDMPLEDDVNPIRIDQTQ  160
             H ++D  Y    +S+ S S+   + G K       +Q E       ++  ++ +R+ Q Q
Sbjct  2417  HGLVDKSYFKANESKVSYSEKLKAFGEK-------IQPETRRRLGEIDQKLSDLRVSQNQ  2469

Query  161   GAETHYESFRMGHPIETVIR---LGMDVENFVERDVEISEQRASPDKHQVSPRGKQSKKN  217
                 +YE+++  HP E   R   +     + + +   + ++R++    +V+P GKQS+ +
Sbjct  2470  QESANYENWKKNHPNEAYKRTQEMETRRNDMLAQQKNLEKERSNLINREVAPFGKQSEIS  2529

Query  218   RKKKLKAKEKVREVKQS  234
             R + L    K+   +++
Sbjct  2530  RLEILGDHNKLTNAQRT  2546


>ref|WP_025175841.1| intein splicing protein [Leptospira kirschneri]
Length=3032

 Score = 39.3 bits (90),  Expect = 4.1, Method: Composition-based stats.
 Identities = 29/137 (21%), Positives = 64/137 (47%), Gaps = 10/137 (7%)

Query  101   HLIIDDVYDPVYDSRTSQSDITSSSGSKTEHIPIKLQMEETSTDMPLEDDVNPIRIDQTQ  160
             H ++D  Y    +S+ S S+   + G K       +Q E       ++  ++ +R+ Q Q
Sbjct  2389  HGLVDKSYFKANESKVSYSEKLKAFGEK-------IQPETRRRLGEIDQKLSDLRVSQNQ  2441

Query  161   GAETHYESFRMGHPIETVIR---LGMDVENFVERDVEISEQRASPDKHQVSPRGKQSKKN  217
                 +YE+++  HP E   R   +     + + +   + ++R++    +V+P GKQS+ +
Sbjct  2442  QESANYENWKKNHPNEAYKRTQEMETRRNDMLAQQKNLEKERSNLINREVAPFGKQSEIS  2501

Query  218   RKKKLKAKEKVREVKQS  234
             R + L    K+   +++
Sbjct  2502  RLEILGDHNKLTNAQRT  2518


Lambda      K        H        a         alpha
   0.316    0.132    0.365    0.792     4.96 

Gapped
Lambda      K        H        a         alpha    sigma
   0.267   0.0410    0.140     1.90     42.6     43.6 

Effective search space used: 1467076246810


  Database: nr
    Posted date:  Sep 23, 2015 12:05 AM
  Number of letters in database: 26,053,659,533
  Number of sequences in database:  71,551,133


Matrix: BLOSUM62
Gap Penalties: Existence: 11, Extension: 1
Neighboring words threshold: 11
Window for multiple hits: 40
```
