# Supplementary material for: Comparative genomics provides new insights into the diversity, physiology, and sexuality of the only industrially exploited tremellomycete: Phaffia rhodozyma
Source: BMC Genomics. 2016 Nov 9;17:901. doi: 10.1186/s12864-016-3244-7 (PMC5103461; doi:10.1186/s12864-016-3244-7)
Supplement: Additional file 6: — List of orphan genes with links to PFAM (related to Additional file 1: Table S1). (ZIP 1428 kb) [file 12864_2016_3244_MOESM6_ESM.zip › BLAST_HTML_FTR/G01956_P.html]

BLAST Search Results


```
BLASTP 2.2.27+


Reference:
Stephen F. Altschul, Thomas L. Madden, Alejandro A. Schäffer,
Jinghui Zhang, Zheng Zhang, Webb Miller, and David J. Lipman (1997),
"Gapped BLAST and PSI-BLAST: a new generation of protein database
search programs", Nucleic Acids Res. 25:3389-3402.


Reference for
composition-based statistics:
Alejandro A. Schäffer, L. Aravind, Thomas L. Madden, Sergei
Shavirin, John L. Spouge, Yuri I. Wolf, Eugene V. Koonin, and
Stephen F. Altschul (2001), "Improving the accuracy of PSI-BLAST
protein database searches with composition-based statistics and
other refinements", Nucleic Acids Res. 29:2994-3005.


Database: nr
           71,551,133 sequences; 26,053,659,533 total letters


Query= G01956_P

Length=434
                                                                      Score     E
Sequences producing significant alignments:                          (Bits)  Value

emb|CDZ96799.1|  hypothetical protein [Xanthophyllomyces dendrorh...   632    0.0  
gb|EJT51263.1|  hypothetical protein A1Q1_07541 [Trichosporon asa...  41.6    1.5  


 >emb|CDZ96799.1| hypothetical protein [Xanthophyllomyces dendrorhous]
Length=445

 Score =  632 bits (1630),  Expect = 0.0, Method: Compositional matrix adjust.
 Identities = 366/450 (81%), Positives = 386/450 (86%), Gaps = 22/450 (5%)

Query  1    MVNPPQLDDHSATVSPTSLYISSVGRLEYLWTKSVDILESSSSENPNSVESFRGPDPVTY  60
            MVNPPQLDDHSATVSPTSLYISSVGRLEYLWTKSVDILESSSSENPNSVESFRGPD V++
Sbjct  1    MVNPPQLDDHSATVSPTSLYISSVGRLEYLWTKSVDILESSSSENPNSVESFRGPD-VSF  59

Query  61   NNRRV-QISFLDAYHGNLA----QTRQVDTRVRESGISIGSEIRRDTL---ALSFDWKPS  112
              R + +IS++     N++     T  V +R+  S ++    +R   L   + S +++ S
Sbjct  60   IGRVLDRISWI--LERNISPCRLHTTTVVSRLVFSMLTTEILLRPGRLIPESESLEYR-S  116

Query  113  PLSFTQTPPLTFSAASNTFSLSYALRKS--SYIRLP-------VIRRSFGPMILLAPLGL  163
             L F +   L  S   N   L +   K   S+  LP       VIRRSFGPMILLAPLGL
Sbjct  117  GLRFEEILWLCLSIG-NLVELLFPSPKHPRSHTYLPFTTYASSVIRRSFGPMILLAPLGL  175

Query  164  LVFFALALGLNGDLWRIGLAFGFFIVSISSSGFGPGETPAGEEPGSTPTPADPGIAPFAV  223
            LVFFALALGLNGDLWRIGLAFGFFIVSISSSGFGPGETPAGEEPGSTPTPADPGIAPFAV
Sbjct  176  LVFFALALGLNGDLWRIGLAFGFFIVSISSSGFGPGETPAGEEPGSTPTPADPGIAPFAV  235

Query  224  RLWLSITLVAILWVMLVVGCARGMTAGQSGNRSSASSTSFAANGNGHVEGQEAEEDQWVK  283
            RLWLSITLVAILWVMLVVGCARGMTAGQSGNRSSASSTSFAANGNGHVEGQEAEEDQWVK
Sbjct  236  RLWLSITLVAILWVMLVVGCARGMTAGQSGNRSSASSTSFAANGNGHVEGQEAEEDQWVK  295

Query  284  EWGKGIGLEARRIRKRTVQDWSPSSRSEPSLLFESRSSFEEEDDERAAPLEETQRQGVLK  343
            EWGKGIGLEARRIRKRTVQDWSPSSRSEPSLLFESRSSFEEEDDERAAPLEETQRQGVLK
Sbjct  296  EWGKGIGLEARRIRKRTVQDWSPSSRSEPSLLFESRSSFEEEDDERAAPLEETQRQGVLK  355

Query  344  IRFPSPFNVIFFILYNIPSFFLSAFCFISASQQTRVRPVLDSWGRSSEKMLAKLLGGLAF  403
            IRFPSPFNVIFFILYNIPSFFLSAFCFISASQQTRVRPVLDSWGRSSEKMLAKLLGGLAF
Sbjct  356  IRFPSPFNVIFFILYNIPSFFLSAFCFISASQQTRVRPVLDSWGRSSEKMLAKLLGGLAF  415

Query  404  GWWDGAMWVWSKLRRGETESARGEITLRGD  433
            GWWDGAMWVWSKLRRGETESARGEITLRGD
Sbjct  416  GWWDGAMWVWSKLRRGETESARGEITLRGD  445


>gb|EJT51263.1| hypothetical protein A1Q1_07541 [Trichosporon asahii var. asahii 
CBS 2479]
 gb|EKD05374.1| hypothetical protein A1Q2_00333 [Trichosporon asahii var. asahii 
CBS 8904]
Length=543

 Score = 41.6 bits (96),  Expect = 1.5, Method: Compositional matrix adjust.
 Identities = 40/162 (25%), Positives = 61/162 (38%), Gaps = 41/162 (25%)

Query  147  VIRRSFGPMILLAPLGLLVFFALALGLNGDLWRIGLAFGFFIVSISSSGFGPGETPAGEE  206
            ++ R      LL PL L ++   A  LNG +W +  + G   V I+ +G           
Sbjct  352  ILARGAKATALLVPLSLGLWAIYAFSLNGKVWAVIESSGD--VDITGNG-----------  398

Query  207  PGSTPTPADPGIAPFAVRLWLSITLVAILWVMLVVGCARGMTAGQSGNRSSASSTSFAAN  266
                  P D G APF  R+ L ITL+ ++ +   +   R M                   
Sbjct  399  -----DPIDVGRAPFETRVALLITLILVIVLSACLSIIRAMA------------------  435

Query  267  GNGHVEGQEAEEDQWVKEWGKGIGLEARRIRKRTVQDWSPSS  308
                  G +  +  W  E+G+GI   ARR     V  + P+S
Sbjct  436  -----PGNDTHDGTWEAEYGEGIAHTARRALAAGVARYLPAS  472


Lambda      K        H        a         alpha
   0.320    0.135    0.413    0.792     4.96 

Gapped
Lambda      K        H        a         alpha    sigma
   0.267   0.0410    0.140     1.90     42.6     43.6 

Effective search space used: 4209739814280


  Database: nr
    Posted date:  Sep 23, 2015 12:05 AM
  Number of letters in database: 26,053,659,533
  Number of sequences in database:  71,551,133


Matrix: BLOSUM62
Gap Penalties: Existence: 11, Extension: 1
Neighboring words threshold: 11
Window for multiple hits: 40
```
