# Supplementary material for: Comparative genomics provides new insights into the diversity, physiology, and sexuality of the only industrially exploited tremellomycete: Phaffia rhodozyma
Source: BMC Genomics. 2016 Nov 9;17:901. doi: 10.1186/s12864-016-3244-7 (PMC5103461; doi:10.1186/s12864-016-3244-7)
Supplement: Additional file 6: — List of orphan genes with links to PFAM (related to Additional file 1: Table S1). (ZIP 1428 kb) [file 12864_2016_3244_MOESM6_ESM.zip › BLAST_HTML_FTR/G05374_P.html]

BLAST Search Results


```
BLASTP 2.2.27+


Reference:
Stephen F. Altschul, Thomas L. Madden, Alejandro A. Schäffer,
Jinghui Zhang, Zheng Zhang, Webb Miller, and David J. Lipman (1997),
"Gapped BLAST and PSI-BLAST: a new generation of protein database
search programs", Nucleic Acids Res. 25:3389-3402.


Reference for
composition-based statistics:
Alejandro A. Schäffer, L. Aravind, Thomas L. Madden, Sergei
Shavirin, John L. Spouge, Yuri I. Wolf, Eugene V. Koonin, and
Stephen F. Altschul (2001), "Improving the accuracy of PSI-BLAST
protein database searches with composition-based statistics and
other refinements", Nucleic Acids Res. 29:2994-3005.


Database: nr
           71,551,133 sequences; 26,053,659,533 total letters


Query= G05374_P

Length=123
                                                                      Score     E
Sequences producing significant alignments:                          (Bits)  Value

emb|CED84184.1|  hypothetical protein [Xanthophyllomyces dendrorh...  79.3    6e-16
ref|WP_050107766.1|  peptidase M26 [Streptococcus pneumoniae] >em...  36.6    3.8  
emb|CEW37849.1|  immunoglobulin A1 protease [Streptococcus pneumo...  36.6    3.8  
ref|WP_051552121.1|  hypothetical protein [Acetobacter persici]       36.2    4.9  


 >emb|CED84184.1| hypothetical protein [Xanthophyllomyces dendrorhous]
Length=129

 Score = 79.3 bits (194),  Expect = 6e-16, Method: Compositional matrix adjust.
 Identities = 40/40 (100%), Positives = 40/40 (100%), Gaps = 0/40 (0%)

Query  1   MLFKSTLFSAFVLSFFLTFVSAVPTKIVERTTSAAKTSQQ  40
           MLFKSTLFSAFVLSFFLTFVSAVPTKIVERTTSAAKTSQQ
Sbjct  1   MLFKSTLFSAFVLSFFLTFVSAVPTKIVERTTSAAKTSQQ  40


>ref|WP_050107766.1| peptidase M26 [Streptococcus pneumoniae]
 emb|CKJ06271.1| immunoglobulin A1 protease [Streptococcus pneumoniae]
Length=2005

 Score = 36.6 bits (83),  Expect = 3.8, Method: Composition-based stats.
 Identities = 30/100 (30%), Positives = 45/100 (45%), Gaps = 2/100 (2%)

Query  21   SAVPTKIVERTTSAAKTSQQTPALHTPASQFPPRTRPLSVERPISLAFTTQTTLSLPSFL  80
            S  P+ + + T S  +T++Q P    P ++     +P + E   S+A          S +
Sbjct  368  SGEPSSVTDATPSNGETTEQ-PGTSAPKAEVTGENKPSTSEPTESVANRASGRTEGDSEV  426

Query  81   TFASTGSPVAAL-EPATRADHTPTNQEETVERGQAQEAAV  119
            T   TG+  A L EP T A  TP N+EE     +A E  V
Sbjct  427  TPGETGTESAELAEPKTEAPTTPENKEEQPGATEAPEQKV  466


>emb|CEW37849.1| immunoglobulin A1 protease [Streptococcus pneumoniae]
 emb|CJE97817.1| immunoglobulin A1 protease [Streptococcus pneumoniae]
Length=2005

 Score = 36.6 bits (83),  Expect = 3.8, Method: Composition-based stats.
 Identities = 30/100 (30%), Positives = 45/100 (45%), Gaps = 2/100 (2%)

Query  21   SAVPTKIVERTTSAAKTSQQTPALHTPASQFPPRTRPLSVERPISLAFTTQTTLSLPSFL  80
            S  P+ + + T S  +T++Q P    P ++     +P + E   S+A          S +
Sbjct  368  SGEPSSVTDATPSNGETTEQ-PGTSAPKAEVTGENKPSTSEPTESVANRASGRTEGDSEV  426

Query  81   TFASTGSPVAAL-EPATRADHTPTNQEETVERGQAQEAAV  119
            T   TG+  A L EP T A  TP N+EE     +A E  V
Sbjct  427  TPGETGTESAELAEPKTEAPTTPENKEEQPGATEAPEQKV  466


>ref|WP_051552121.1| hypothetical protein [Acetobacter persici]
Length=248

 Score = 36.2 bits (82),  Expect = 4.9, Method: Compositional matrix adjust.
 Identities = 31/110 (28%), Positives = 50/110 (45%), Gaps = 8/110 (7%)

Query  10   AFVLSFFLTFVSAVP---TKIVERTTSAAKTSQQTPALHTPASQFPPRTRPLSVERPISL  66
            A+ L F  T+ +A+    + +V+R     K+  Q P L  PA Q P R  P  V   + L
Sbjct  81   AYALGFLRTYGTALGFDASTLVQRYKREGKSGGQKPDLTFPAPQ-PDRRIPPGVSVSLGL  139

Query  67   AFTTQTTLSLPSFLTFASTGS----PVAALEPATRADHTPTNQEETVERG  112
            A    + +    F+  A        PVAA+ P  + ++TP+ Q  ++  G
Sbjct  140  AVILASYVGWYHFIGHAPPAPEHVPPVAAIIPGAQTNNTPSPQVASILPG  189


Lambda      K        H        a         alpha
   0.321    0.127    0.355    0.792     4.96 

Gapped
Lambda      K        H        a         alpha    sigma
   0.267   0.0410    0.140     1.90     42.6     43.6 

Effective search space used: 647263899579


  Database: nr
    Posted date:  Sep 23, 2015 12:05 AM
  Number of letters in database: 26,053,659,533
  Number of sequences in database:  71,551,133


Matrix: BLOSUM62
Gap Penalties: Existence: 11, Extension: 1
Neighboring words threshold: 11
Window for multiple hits: 40
```
