# Supplementary material for: Comparative genomics provides new insights into the diversity, physiology, and sexuality of the only industrially exploited tremellomycete: Phaffia rhodozyma
Source: BMC Genomics. 2016 Nov 9;17:901. doi: 10.1186/s12864-016-3244-7 (PMC5103461; doi:10.1186/s12864-016-3244-7)
Supplement: Additional file 6: — List of orphan genes with links to PFAM (related to Additional file 1: Table S1). (ZIP 1428 kb) [file 12864_2016_3244_MOESM6_ESM.zip › BLAST_HTML_FTR/G05366_P.html]

BLAST Search Results


```
BLASTP 2.2.27+


Reference:
Stephen F. Altschul, Thomas L. Madden, Alejandro A. Schäffer,
Jinghui Zhang, Zheng Zhang, Webb Miller, and David J. Lipman (1997),
"Gapped BLAST and PSI-BLAST: a new generation of protein database
search programs", Nucleic Acids Res. 25:3389-3402.


Reference for
composition-based statistics:
Alejandro A. Schäffer, L. Aravind, Thomas L. Madden, Sergei
Shavirin, John L. Spouge, Yuri I. Wolf, Eugene V. Koonin, and
Stephen F. Altschul (2001), "Improving the accuracy of PSI-BLAST
protein database searches with composition-based statistics and
other refinements", Nucleic Acids Res. 29:2994-3005.


Database: nr
           71,551,133 sequences; 26,053,659,533 total letters


Query= G05366_P

Length=377
                                                                      Score     E
Sequences producing significant alignments:                          (Bits)  Value

emb|CED84886.1|  hypothetical protein [Xanthophyllomyces dendrorh...   722    0.0  
ref|XP_007398026.1|  hypothetical protein PHACADRAFT_259631 [Phan...  39.3    5.6  


 >emb|CED84886.1| hypothetical protein [Xanthophyllomyces dendrorhous]
Length=376

 Score =  722 bits (1864),  Expect = 0.0, Method: Compositional matrix adjust.
 Identities = 376/376 (100%), Positives = 376/376 (100%), Gaps = 0/376 (0%)

Query  1    MSFPTSTSIDLPLSLDPREPPSTPSPHKIRTPKRTTPLPSRTSSPHPVGHHTLSSSPLNR  60
            MSFPTSTSIDLPLSLDPREPPSTPSPHKIRTPKRTTPLPSRTSSPHPVGHHTLSSSPLNR
Sbjct  1    MSFPTSTSIDLPLSLDPREPPSTPSPHKIRTPKRTTPLPSRTSSPHPVGHHTLSSSPLNR  60

Query  61   SASISSGTAKVGSIGGISRDEEKGSDIFERDIEGRGGEIVSLATGELDDSSGPLVHVGPN  120
            SASISSGTAKVGSIGGISRDEEKGSDIFERDIEGRGGEIVSLATGELDDSSGPLVHVGPN
Sbjct  61   SASISSGTAKVGSIGGISRDEEKGSDIFERDIEGRGGEIVSLATGELDDSSGPLVHVGPN  120

Query  121  SPRPHHHHHHHQQQQHHQRQQQSHHVPTPRISATDQLFPTVLDDAIEALSAVGNQDVLVV  180
            SPRPHHHHHHHQQQQHHQRQQQSHHVPTPRISATDQLFPTVLDDAIEALSAVGNQDVLVV
Sbjct  121  SPRPHHHHHHHQQQQHHQRQQQSHHVPTPRISATDQLFPTVLDDAIEALSAVGNQDVLVV  180

Query  181  TPSRSPSRAGSPGGMDRLRRGLGLGSSPDGTGSGSKSPVLSLGEGGRELWDLKMADQSAL  240
            TPSRSPSRAGSPGGMDRLRRGLGLGSSPDGTGSGSKSPVLSLGEGGRELWDLKMADQSAL
Sbjct  181  TPSRSPSRAGSPGGMDRLRRGLGLGSSPDGTGSGSKSPVLSLGEGGRELWDLKMADQSAL  240

Query  241  GEEFLSTSSSPSPPHSLPASPHPLSLSSSSHSLHQAFNSIALPPSYIPTSSNPHSSPHRL  300
            GEEFLSTSSSPSPPHSLPASPHPLSLSSSSHSLHQAFNSIALPPSYIPTSSNPHSSPHRL
Sbjct  241  GEEFLSTSSSPSPPHSLPASPHPLSLSSSSHSLHQAFNSIALPPSYIPTSSNPHSSPHRL  300

Query  301  AFISPLDLRLSPPLSTHSLATLTAGSSLIHLGDRESITASASASTPSSREPSAVGEREAS  360
            AFISPLDLRLSPPLSTHSLATLTAGSSLIHLGDRESITASASASTPSSREPSAVGEREAS
Sbjct  301  AFISPLDLRLSPPLSTHSLATLTAGSSLIHLGDRESITASASASTPSSREPSAVGEREAS  360

Query  361  GRNLEDRLEKVHVVDS  376
            GRNLEDRLEKVHVVDS
Sbjct  361  GRNLEDRLEKVHVVDS  376


>ref|XP_007398026.1| hypothetical protein PHACADRAFT_259631 [Phanerochaete carnosa 
HHB-10118-sp]
 gb|EKM53334.1| hypothetical protein PHACADRAFT_259631 [Phanerochaete carnosa 
HHB-10118-sp]
Length=400

 Score = 39.3 bits (90),  Expect = 5.6, Method: Compositional matrix adjust.
 Identities = 89/294 (30%), Positives = 120/294 (41%), Gaps = 64/294 (22%)

Query  144  HHVPTPRISAT-DQLFPTVLDDAIEALSAVGNQD-----VLVVTP---------------  182
            H +P  ++S   DQ  P+VLD A E L+A  +       + VVTP               
Sbjct  93   HRIPRGKLSEQLDQSVPSVLDSAAEMLTADPDDKDSMDFIAVVTPVSFDHLPPISLGPRS  152

Query  183  ------SRSPSRAGSPGGM-DRLRRGLGLGS-SPDGTG---SGSKSPVLSLGEGGRELWD  231
                  S+  SR+ SP GM +R    LGL S +P  T      ++SPV+S     R    
Sbjct  153  GFTSPISQMSSRSPSPNGMSNRKSVILGLPSPTPSFTMPLPGQTQSPVVSTSPPMRPAVQ  212

Query  232  LKMADQ------SALGEEFLSTSSSPSPPHSLPASPHPLSLSSSSHSLHQAFNSIALPPS  285
                 Q      S     + S  S  S P +     HPL   SSS S            S
Sbjct  213  TNSPSQAPAPSISTPTSAYFSVHSEESSPTTATHVEHPLHTLSSSTSSATPTTVSGGLAS  272

Query  286  YIPTSSNPHSSPHRLAFISPLDLRLSPPLSTHSLATLTAG-------SSLIHLGDRESIT  338
            + P S  P +   RL+F+S  DL  S P STH L++ T         SS+I +   ++  
Sbjct  273  HAPPS--PKNPSKRLSFLSYTDLLTSVPASTHPLSSFTQFNEPPPHLSSVIGIPQAQAQL  330

Query  339  ASASASTPSSREPSA--------------VG---EREASGRNLEDRLEKVHVVD  375
            +S +AS   S + SA              VG   ERE  GR LE+RLE + V D
Sbjct  331  SSGAASIHGSLKASAWITERDPATDLVNDVGGEWEREGLGRGLEERLETLMVND  384


Lambda      K        H        a         alpha
   0.310    0.128    0.371    0.792     4.96 

Gapped
Lambda      K        H        a         alpha    sigma
   0.267   0.0410    0.140     1.90     42.6     43.6 

Effective search space used: 3415024646325


  Database: nr
    Posted date:  Sep 23, 2015 12:05 AM
  Number of letters in database: 26,053,659,533
  Number of sequences in database:  71,551,133


Matrix: BLOSUM62
Gap Penalties: Existence: 11, Extension: 1
Neighboring words threshold: 11
Window for multiple hits: 40
```
