# Supplementary material for: Comparative genomics provides new insights into the diversity, physiology, and sexuality of the only industrially exploited tremellomycete: Phaffia rhodozyma
Source: BMC Genomics. 2016 Nov 9;17:901. doi: 10.1186/s12864-016-3244-7 (PMC5103461; doi:10.1186/s12864-016-3244-7)
Supplement: Additional file 6: — List of orphan genes with links to PFAM (related to Additional file 1: Table S1). (ZIP 1428 kb) [file 12864_2016_3244_MOESM6_ESM.zip › BLAST_HTML_FTR/G02824_P.html]

BLAST Search Results


```
BLASTP 2.2.27+


Reference:
Stephen F. Altschul, Thomas L. Madden, Alejandro A. Schäffer,
Jinghui Zhang, Zheng Zhang, Webb Miller, and David J. Lipman (1997),
"Gapped BLAST and PSI-BLAST: a new generation of protein database
search programs", Nucleic Acids Res. 25:3389-3402.


Reference for
composition-based statistics:
Alejandro A. Schäffer, L. Aravind, Thomas L. Madden, Sergei
Shavirin, John L. Spouge, Yuri I. Wolf, Eugene V. Koonin, and
Stephen F. Altschul (2001), "Improving the accuracy of PSI-BLAST
protein database searches with composition-based statistics and
other refinements", Nucleic Acids Res. 29:2994-3005.


Database: nr
           71,551,133 sequences; 26,053,659,533 total letters


Query= G02824_P

Length=377
                                                                      Score     E
Sequences producing significant alignments:                          (Bits)  Value

emb|CED84850.1|  hypothetical protein [Xanthophyllomyces dendrorh...   557    0.0  
ref|WP_006635882.1|  filamentous hemagglutinin family outer membr...  48.9    0.009


 >emb|CED84850.1| hypothetical protein [Xanthophyllomyces dendrorhous]
Length=518

 Score =  557 bits (1435),  Expect = 0.0, Method: Compositional matrix adjust.
 Identities = 308/308 (100%), Positives = 308/308 (100%), Gaps = 0/308 (0%)

Query  1    MRYHQPNLENEHRDYKSLLLNLRHQDTLDLRQGLISPLVRYTRKKPARSRASEQRSRLED  60
            MRYHQPNLENEHRDYKSLLLNLRHQDTLDLRQGLISPLVRYTRKKPARSRASEQRSRLED
Sbjct  1    MRYHQPNLENEHRDYKSLLLNLRHQDTLDLRQGLISPLVRYTRKKPARSRASEQRSRLED  60

Query  61   ESDMDIGEEDEDGYEDKDDEAAWVIEDGVRKRIKGEDKEEGDGRFSDVFGFSLPPLLQAR  120
            ESDMDIGEEDEDGYEDKDDEAAWVIEDGVRKRIKGEDKEEGDGRFSDVFGFSLPPLLQAR
Sbjct  61   ESDMDIGEEDEDGYEDKDDEAAWVIEDGVRKRIKGEDKEEGDGRFSDVFGFSLPPLLQAR  120

Query  121  PVLPRIPPEDERTLFPLYVSQLPPPPFETLADEILLLAKKFLKQDRKRCLKVKPTRLPAT  180
            PVLPRIPPEDERTLFPLYVSQLPPPPFETLADEILLLAKKFLKQDRKRCLKVKPTRLPAT
Sbjct  121  PVLPRIPPEDERTLFPLYVSQLPPPPFETLADEILLLAKKFLKQDRKRCLKVKPTRLPAT  180

Query  181  MDSCQTVEPSLPTSSLCQPPSAQPQPPISPPSLSSQPPSLFPPTSPHHYQPYCPPKSSSQ  240
            MDSCQTVEPSLPTSSLCQPPSAQPQPPISPPSLSSQPPSLFPPTSPHHYQPYCPPKSSSQ
Sbjct  181  MDSCQTVEPSLPTSSLCQPPSAQPQPPISPPSLSSQPPSLFPPTSPHHYQPYCPPKSSSQ  240

Query  241  TSSITSKELIHTTRSLLAVTLDGLSKVTPVDTFGTARAKKRKLREAESGWERVLNVLSGL  300
            TSSITSKELIHTTRSLLAVTLDGLSKVTPVDTFGTARAKKRKLREAESGWERVLNVLSGL
Sbjct  241  TSSITSKELIHTTRSLLAVTLDGLSKVTPVDTFGTARAKKRKLREAESGWERVLNVLSGL  300

Query  301  DQDSLDPV  308
            DQDSLDPV
Sbjct  301  DQDSLDPV  308


 Score = 71.2 bits (173),  Expect = 4e-10, Method: Compositional matrix adjust.
 Identities = 69/71 (97%), Positives = 69/71 (97%), Gaps = 0/71 (0%)

Query  306  DPVDEDSDLNSEDKEVEEEEEEEEEEEMDREQEEGEDEAEKLEGTGLTIFHSPFIPPRKR  365
            D  DEDSDLNSEDKEVEEEEEEEEEEEMDREQEEGEDEAEKLEGTGLTIFHSPFIPPRKR
Sbjct  448  DIRDEDSDLNSEDKEVEEEEEEEEEEEMDREQEEGEDEAEKLEGTGLTIFHSPFIPPRKR  507

Query  366  SVERSKDIKES  376
            SVERSKDIKES
Sbjct  508  SVERSKDIKES  518


>ref|WP_006635882.1| filamentous hemagglutinin family outer membrane protein [Microcoleus 
vaginatus]
 gb|EGK83617.1| filamentous hemagglutinin family outer membrane protein [Microcoleus 
vaginatus FGP-2]
Length=3022

 Score = 48.9 bits (115),  Expect = 0.009, Method: Composition-based stats.
 Identities = 57/244 (23%), Positives = 95/244 (39%), Gaps = 40/244 (16%)

Query  112   SLPPLLQARPVLPRIPPEDERTLFPLYVSQLPPPPFETLADEILLLAKKFLKQDRKRCLK  171
             ++PPL +  P  P +PP  E T+ P       PP  ET+   I  L +            
Sbjct  2305  TIPPLTETIP--PTLPPLTE-TIPPTI-----PPLTETIPPTIPPLTQT-----------  2345

Query  172   VKPTRLPATMDSCQTVEPSLPTSSLCQPPSAQPQPPISPPSLSSQPPSL------FPPTS  225
             + PT  P T    QT+ P++P       P  Q  PP  PP   + PP++       PPT 
Sbjct  2346  IPPTIPPLT----QTIPPTIP-------PLTQTIPPTIPPLTQTIPPTIPPLTQTIPPTI  2394

Query  226   PHHYQPYCPPKSSSQTSSITSKELIHTTRSLLAVTLDGLSKVTPVDTFGTARAKKRKLRE  285
             P    P  PP + +    I        TR++L       +     +T          + E
Sbjct  2395  PATVPPAIPPLTQTIPPIIPPTIPP-ITRTVLPTI--PPTIPPITETVSPITGTVPPITE  2451

Query  286   AESGWERVLNVLSGLDQDSLDPVDEDSDLN-SEDKEVEEEEEEEEEEEMDREQEEGEDEA  344
                    +L   +  +Q  ++   + ++ N  E+++V E  ++E ++ M   Q+E +   
Sbjct  2452  TTGTVLPILPQTTETNQQVMETNQQVTETNQQENQQVTETNQQENQQVMVINQQENQPVT  2511

Query  345   EKLE  348
               LE
Sbjct  2512  SALE  2515


Lambda      K        H        a         alpha
   0.312    0.132    0.381    0.792     4.96 

Gapped
Lambda      K        H        a         alpha    sigma
   0.267   0.0410    0.140     1.90     42.6     43.6 

Effective search space used: 3415024646325


  Database: nr
    Posted date:  Sep 23, 2015 12:05 AM
  Number of letters in database: 26,053,659,533
  Number of sequences in database:  71,551,133


Matrix: BLOSUM62
Gap Penalties: Existence: 11, Extension: 1
Neighboring words threshold: 11
Window for multiple hits: 40
```
