# Supplementary material for: Comparative genomics provides new insights into the diversity, physiology, and sexuality of the only industrially exploited tremellomycete: Phaffia rhodozyma
Source: BMC Genomics. 2016 Nov 9;17:901. doi: 10.1186/s12864-016-3244-7 (PMC5103461; doi:10.1186/s12864-016-3244-7)
Supplement: Additional file 6: — List of orphan genes with links to PFAM (related to Additional file 1: Table S1). (ZIP 1428 kb) [file 12864_2016_3244_MOESM6_ESM.zip › BLAST_HTML_FTR/G03142_P.html]

BLAST Search Results


```
BLASTP 2.2.27+


Reference:
Stephen F. Altschul, Thomas L. Madden, Alejandro A. Schäffer,
Jinghui Zhang, Zheng Zhang, Webb Miller, and David J. Lipman (1997),
"Gapped BLAST and PSI-BLAST: a new generation of protein database
search programs", Nucleic Acids Res. 25:3389-3402.


Reference for
composition-based statistics:
Alejandro A. Schäffer, L. Aravind, Thomas L. Madden, Sergei
Shavirin, John L. Spouge, Yuri I. Wolf, Eugene V. Koonin, and
Stephen F. Altschul (2001), "Improving the accuracy of PSI-BLAST
protein database searches with composition-based statistics and
other refinements", Nucleic Acids Res. 29:2994-3005.


Database: nr
           71,551,133 sequences; 26,053,659,533 total letters


Query= G03142_P

Length=1219
                                                                      Score     E
Sequences producing significant alignments:                          (Bits)  Value

emb|CDZ96684.1|  hypothetical protein [Xanthophyllomyces dendrorh...  2435    0.0  
ref|XP_009855530.1|  hypothetical protein NEUTE1DRAFT_149556 [Neu...  45.4    0.52 


 >emb|CDZ96684.1| hypothetical protein [Xanthophyllomyces dendrorhous]
Length=1244

 Score = 2435 bits (6312),  Expect = 0.0, Method: Compositional matrix adjust.
 Identities = 1200/1200 (100%), Positives = 1200/1200 (100%), Gaps = 0/1200 (0%)

Query  1     MHPSQLTWSQQETYSTAALIAELNHPDLTIEEAEDIFLNIIHGRLMEGKDRWSVGKELEE  60
             MHPSQLTWSQQETYSTAALIAELNHPDLTIEEAEDIFLNIIHGRLMEGKDRWSVGKELEE
Sbjct  25    MHPSQLTWSQQETYSTAALIAELNHPDLTIEEAEDIFLNIIHGRLMEGKDRWSVGKELEE  84

Query  61    IARSIPVRRTISLSAVRQATFFHLATLSKEAFVTAQSRAKVVTSLKSHERSPHKGKEVLL  120
             IARSIPVRRTISLSAVRQATFFHLATLSKEAFVTAQSRAKVVTSLKSHERSPHKGKEVLL
Sbjct  85    IARSIPVRRTISLSAVRQATFFHLATLSKEAFVTAQSRAKVVTSLKSHERSPHKGKEVLL  144

Query  121   EYVNTMERVTGRWIGVGAGGIKSQTELVQGGLRSIKESVENNKSLSSLVEQIQLQTGHVS  180
             EYVNTMERVTGRWIGVGAGGIKSQTELVQGGLRSIKESVENNKSLSSLVEQIQLQTGHVS
Sbjct  145   EYVNTMERVTGRWIGVGAGGIKSQTELVQGGLRSIKESVENNKSLSSLVEQIQLQTGHVS  204

Query  181   LSQLLNHLRSLAIASDALKESEKVLRAWAQTGLKAGESRFSLGSQLEEIQSMLPHALHPV  240
             LSQLLNHLRSLAIASDALKESEKVLRAWAQTGLKAGESRFSLGSQLEEIQSMLPHALHPV
Sbjct  205   LSQLLNHLRSLAIASDALKESEKVLRAWAQTGLKAGESRFSLGSQLEEIQSMLPHALHPV  264

Query  241   FPSLLESLGLPPSPIHPHAQGVACQPFLIESFLGDTSDPEFAQEVLFEWIWQEKRAWRKI  300
             FPSLLESLGLPPSPIHPHAQGVACQPFLIESFLGDTSDPEFAQEVLFEWIWQEKRAWRKI
Sbjct  265   FPSLLESLGLPPSPIHPHAQGVACQPFLIESFLGDTSDPEFAQEVLFEWIWQEKRAWRKI  324

Query  301   WGPKEKESLRTRLMKMESQLDNAILAPIFHSVMSKTFLFEPSSSISTAPGSLLSLDRSLS  360
             WGPKEKESLRTRLMKMESQLDNAILAPIFHSVMSKTFLFEPSSSISTAPGSLLSLDRSLS
Sbjct  325   WGPKEKESLRTRLMKMESQLDNAILAPIFHSVMSKTFLFEPSSSISTAPGSLLSLDRSLS  384

Query  361   SVKPLQRPNRSHYVLASSFKRAPRTDDESDQDQLTKKNIFSTLPPATSVSLSELQKTLYS  420
             SVKPLQRPNRSHYVLASSFKRAPRTDDESDQDQLTKKNIFSTLPPATSVSLSELQKTLYS
Sbjct  385   SVKPLQRPNRSHYVLASSFKRAPRTDDESDQDQLTKKNIFSTLPPATSVSLSELQKTLYS  444

Query  421   PSLGLSDSISSLDRSPYPKLAINQSRHGNSFAPANSFLTGVTTQNMPRHHRSASSPVYIQ  480
             PSLGLSDSISSLDRSPYPKLAINQSRHGNSFAPANSFLTGVTTQNMPRHHRSASSPVYIQ
Sbjct  445   PSLGLSDSISSLDRSPYPKLAINQSRHGNSFAPANSFLTGVTTQNMPRHHRSASSPVYIQ  504

Query  481   SQAQEMRYTSISDGHLPSPKLAHWRTRSSSFGRSVEDVSMSQGSLPILEEQSEDMSTHID  540
             SQAQEMRYTSISDGHLPSPKLAHWRTRSSSFGRSVEDVSMSQGSLPILEEQSEDMSTHID
Sbjct  505   SQAQEMRYTSISDGHLPSPKLAHWRTRSSSFGRSVEDVSMSQGSLPILEEQSEDMSTHID  564

Query  541   LEEFLVSTFSPPCQQSQSVDGHLGHSSSFRIGGRAVRDMLDNEGTDTFSPKKSELNRDAL  600
             LEEFLVSTFSPPCQQSQSVDGHLGHSSSFRIGGRAVRDMLDNEGTDTFSPKKSELNRDAL
Sbjct  565   LEEFLVSTFSPPCQQSQSVDGHLGHSSSFRIGGRAVRDMLDNEGTDTFSPKKSELNRDAL  624

Query  601   RLNITLPRATASGDSLHLSRFDTDLAGHNFLGDKPVKEEGSLQNLKHPKLQSLTQLSKPP  660
             RLNITLPRATASGDSLHLSRFDTDLAGHNFLGDKPVKEEGSLQNLKHPKLQSLTQLSKPP
Sbjct  625   RLNITLPRATASGDSLHLSRFDTDLAGHNFLGDKPVKEEGSLQNLKHPKLQSLTQLSKPP  684

Query  661   KLEEETSDNSPSKGVSLTPSSLPSDKTMSVPDQPAPPPQKKRFSFFQRRRPAINPPVAVV  720
             KLEEETSDNSPSKGVSLTPSSLPSDKTMSVPDQPAPPPQKKRFSFFQRRRPAINPPVAVV
Sbjct  685   KLEEETSDNSPSKGVSLTPSSLPSDKTMSVPDQPAPPPQKKRFSFFQRRRPAINPPVAVV  744

Query  721   EPESKSIPIQVSQHNTNSKPGEDVSSVAHSSQSVTDPMSCLRSGSFYNSNSHMGEGLNTV  780
             EPESKSIPIQVSQHNTNSKPGEDVSSVAHSSQSVTDPMSCLRSGSFYNSNSHMGEGLNTV
Sbjct  745   EPESKSIPIQVSQHNTNSKPGEDVSSVAHSSQSVTDPMSCLRSGSFYNSNSHMGEGLNTV  804

Query  781   SGLTYSVLEETGEIPRPDSPSSPSDESLLTRVWSHTGHRRLTVINPDVASDSNRSSLSIA  840
             SGLTYSVLEETGEIPRPDSPSSPSDESLLTRVWSHTGHRRLTVINPDVASDSNRSSLSIA
Sbjct  805   SGLTYSVLEETGEIPRPDSPSSPSDESLLTRVWSHTGHRRLTVINPDVASDSNRSSLSIA  864

Query  841   PADILDEDSAKVDPVQNNDIDKEISGPEDEEEEEKDLDAIARVGSPVVEIRSSDAYPSIL  900
             PADILDEDSAKVDPVQNNDIDKEISGPEDEEEEEKDLDAIARVGSPVVEIRSSDAYPSIL
Sbjct  865   PADILDEDSAKVDPVQNNDIDKEISGPEDEEEEEKDLDAIARVGSPVVEIRSSDAYPSIL  924

Query  901   SREEDFPSLSIFSISPRESSNGYREMFRPREAEPIITSILNPPMVSPPVRALSMVNKAMF  960
             SREEDFPSLSIFSISPRESSNGYREMFRPREAEPIITSILNPPMVSPPVRALSMVNKAMF
Sbjct  925   SREEDFPSLSIFSISPRESSNGYREMFRPREAEPIITSILNPPMVSPPVRALSMVNKAMF  984

Query  961   AEQVVPDRSGPLPSTNHTTHDFSSSALTLRGVFDSLPSGVVHSSGFVLEILMNYIDAERG  1020
             AEQVVPDRSGPLPSTNHTTHDFSSSALTLRGVFDSLPSGVVHSSGFVLEILMNYIDAERG
Sbjct  985   AEQVVPDRSGPLPSTNHTTHDFSSSALTLRGVFDSLPSGVVHSSGFVLEILMNYIDAERG  1044

Query  1021  RAMADPRMKGWGDEERGKVGWFLSELEESLVVTNPHLIPTFNRVRILTDCPPQRNYQPRP  1080
             RAMADPRMKGWGDEERGKVGWFLSELEESLVVTNPHLIPTFNRVRILTDCPPQRNYQPRP
Sbjct  1045  RAMADPRMKGWGDEERGKVGWFLSELEESLVVTNPHLIPTFNRVRILTDCPPQRNYQPRP  1104

Query  1081  RNSRSGRASFVSHPAIPSTPRFPSFSASESSTAVTSESSPPLLKTNFQSSRQDINPTAQF  1140
             RNSRSGRASFVSHPAIPSTPRFPSFSASESSTAVTSESSPPLLKTNFQSSRQDINPTAQF
Sbjct  1105  RNSRSGRASFVSHPAIPSTPRFPSFSASESSTAVTSESSPPLLKTNFQSSRQDINPTAQF  1164

Query  1141  RKKLRAHDGEETDTHLDAYAGYKDLTPPAVNGNDTVESRKSRRDRMLFPVALRNETAGQS  1200
             RKKLRAHDGEETDTHLDAYAGYKDLTPPAVNGNDTVESRKSRRDRMLFPVALRNETAGQS
Sbjct  1165  RKKLRAHDGEETDTHLDAYAGYKDLTPPAVNGNDTVESRKSRRDRMLFPVALRNETAGQS  1224


>ref|XP_009855530.1| hypothetical protein NEUTE1DRAFT_149556 [Neurospora tetrasperma 
FGSC 2508]
 gb|EGO51887.1| hypothetical protein NEUTE1DRAFT_149556 [Neurospora tetrasperma 
FGSC 2508]
Length=2645

 Score = 45.4 bits (106),  Expect = 0.52, Method: Compositional matrix adjust.
 Identities = 33/109 (30%), Positives = 51/109 (47%), Gaps = 7/109 (6%)

Query  673  KGVSLTPSSLPSDKTMSVPDQP----APPPQKKRFSFFQRRRPAINPPVAVVEPESKSIP  728
            K +  TP+ L SDK   VP Q       P + +R+   Q R  AI  P  V++    +  
Sbjct  194  KALEQTPTELESDKQDEVPGQKEEVELQPTEAERWVIIQERLKAIIGPTTVIQ---NAEI  250

Query  729  IQVSQHNTNSKPGEDVSSVAHSSQSVTDPMSCLRSGSFYNSNSHMGEGL  777
            I+       S P  + ++ +H+S  V   +SC+ S SFYN  +  G G+
Sbjct  251  IRTIVGFWESIPRAEGAAASHASMIVLTALSCVASVSFYNLTAIHGTGI  299


Lambda      K        H        a         alpha
   0.312    0.128    0.366    0.792     4.96 

Gapped
Lambda      K        H        a         alpha    sigma
   0.267   0.0410    0.140     1.90     42.6     43.6 

Effective search space used: 15196711045824


  Database: nr
    Posted date:  Sep 23, 2015 12:05 AM
  Number of letters in database: 26,053,659,533
  Number of sequences in database:  71,551,133


Matrix: BLOSUM62
Gap Penalties: Existence: 11, Extension: 1
Neighboring words threshold: 11
Window for multiple hits: 40
```
