# Supplementary material for: Comparative genomics provides new insights into the diversity, physiology, and sexuality of the only industrially exploited tremellomycete: Phaffia rhodozyma
Source: BMC Genomics. 2016 Nov 9;17:901. doi: 10.1186/s12864-016-3244-7 (PMC5103461; doi:10.1186/s12864-016-3244-7)
Supplement: Additional file 6: — List of orphan genes with links to PFAM (related to Additional file 1: Table S1). (ZIP 1428 kb) [file 12864_2016_3244_MOESM6_ESM.zip › BLAST_HTML_FTR/G01167_P.html]

BLAST Search Results


```
BLASTP 2.2.27+


Reference:
Stephen F. Altschul, Thomas L. Madden, Alejandro A. Schäffer,
Jinghui Zhang, Zheng Zhang, Webb Miller, and David J. Lipman (1997),
"Gapped BLAST and PSI-BLAST: a new generation of protein database
search programs", Nucleic Acids Res. 25:3389-3402.


Reference for
composition-based statistics:
Alejandro A. Schäffer, L. Aravind, Thomas L. Madden, Sergei
Shavirin, John L. Spouge, Yuri I. Wolf, Eugene V. Koonin, and
Stephen F. Altschul (2001), "Improving the accuracy of PSI-BLAST
protein database searches with composition-based statistics and
other refinements", Nucleic Acids Res. 29:2994-3005.


Database: nr
           71,551,133 sequences; 26,053,659,533 total letters


Query= G01167_P

Length=171
                                                                      Score     E
Sequences producing significant alignments:                          (Bits)  Value

emb|CED82937.1|  hypothetical protein [Xanthophyllomyces dendrorh...   275    3e-91
ref|XP_011207386.1|  PREDICTED: fibrillin-1 isoform X1 [Bactrocer...  43.1    0.061
ref|XP_007297502.1|  Hsp70-like protein [Marssonina brunnea f. sp...  40.4    0.55 
gb|AIE37058.1|  membrane protein [Bacillus thuringiensis serovar ...  38.9    1.9  
ref|WP_036141345.1|  hypothetical protein [Luteibacter sp. 9135]      38.1    3.8  
ref|XP_013865549.1|  PREDICTED: adenylate cyclase type 9 [Austrof...  37.7    4.7  
ref|XP_011664006.1|  PREDICTED: uncharacterized protein LOC105438...  37.4    5.5  
dbj|GAK62257.1|  60S ribosomal protein L23 [Pseudozyma antarctica]    36.6    9.2  


 >emb|CED82937.1| hypothetical protein [Xanthophyllomyces dendrorhous]
Length=161

 Score =  275 bits (703),  Expect = 3e-91, Method: Compositional matrix adjust.
 Identities = 139/139 (100%), Positives = 139/139 (100%), Gaps = 0/139 (0%)

Query  1    MQLTRALKQSFRIRPSLGLAPPFAHLRFTLPSGRFKSFSSSDSAPVQTGIEGSPTTKSMK  60
            MQLTRALKQSFRIRPSLGLAPPFAHLRFTLPSGRFKSFSSSDSAPVQTGIEGSPTTKSMK
Sbjct  1    MQLTRALKQSFRIRPSLGLAPPFAHLRFTLPSGRFKSFSSSDSAPVQTGIEGSPTTKSMK  60

Query  61   ETSEGPTPIDKTSTNQPLTSHATSLPSSLQPPAPPGITQTSKDSQTEEGAQGKGKRTIAE  120
            ETSEGPTPIDKTSTNQPLTSHATSLPSSLQPPAPPGITQTSKDSQTEEGAQGKGKRTIAE
Sbjct  61   ETSEGPTPIDKTSTNQPLTSHATSLPSSLQPPAPPGITQTSKDSQTEEGAQGKGKRTIAE  120

Query  121  RDAEMMQKLLDRDGGQSTL  139
            RDAEMMQKLLDRDGGQSTL
Sbjct  121  RDAEMMQKLLDRDGGQSTL  139


>ref|XP_011207386.1| PREDICTED: fibrillin-1 isoform X1 [Bactrocera dorsalis]
Length=1845

 Score = 43.1 bits (100),  Expect = 0.061, Method: Composition-based stats.
 Identities = 31/93 (33%), Positives = 46/93 (49%), Gaps = 8/93 (9%)

Query  16   SLGLAPPFAHLRFTLPSGR-FKSFSSSDSAPVQTGIEGSPTTKSMKETSEGPTPIDKTST  74
            +LGL P +A LR  L S +   SF+ + S      I  +PT  + + +S     ID+ S 
Sbjct  906  ALGLPPVYASLRALLSSMQPVSSFAKTSS------IMSTPTISNTQNSSPSCLDIDECSI  959

Query  75   NQPLTSH-ATSLPSSLQPPAPPGITQTSKDSQT  106
            +    SH   + P S Q   PPG T +S D++T
Sbjct  960  SNGNCSHFCMNFPGSFQCSCPPGFTLSSVDNRT  992


>ref|XP_007297502.1| Hsp70-like protein [Marssonina brunnea f. sp. 'multigermtubi' 
MB_m1]
 gb|EKD12292.1| Hsp70-like protein [Marssonina brunnea f. sp. 'multigermtubi' 
MB_m1]
Length=824

 Score = 40.4 bits (93),  Expect = 0.55, Method: Composition-based stats.
 Identities = 29/99 (29%), Positives = 40/99 (40%), Gaps = 9/99 (9%)

Query  33   GRFKSFSSSDSAPVQTGIEGSPTTKSMKETSE--------GPTPIDKTST-NQPLTSHAT  83
            GR  S++++   P  TGI G+ TT  M +           GP PI+  ST N P   H  
Sbjct  65   GRPPSYTNNTYPPAPTGINGARTTSPMAQHPRTPPSQVMGGPPPINTASTGNYPPPGHPA  124

Query  84   SLPSSLQPPAPPGITQTSKDSQTEEGAQGKGKRTIAERD  122
             + +  Q   PPG       +Q   GA        A R+
Sbjct  125  GMGAPQQAAGPPGYGPPQYGAQFPPGANSMAAAQYANRN  163


>gb|AIE37058.1| membrane protein [Bacillus thuringiensis serovar kurstaki str. 
HD-1]
Length=739

 Score = 38.9 bits (89),  Expect = 1.9, Method: Composition-based stats.
 Identities = 32/116 (28%), Positives = 61/116 (53%), Gaps = 11/116 (9%)

Query  46   VQTGIEGSPTT-KSMKETSEGPTPIDKTSTNQPLTSHATSLPSS----LQPPAPPGITQT  100
            V  G++G+ +  KS +E  +G     + +  Q L +    + ++    ++P A P +   
Sbjct  557  VSDGVKGTVSEFKSGQE--QGKEKAQENADRQTLNTIGKGIQNNANAGIEPSASPKMANR  614

Query  101  SK-DSQTEEGAQG--KGKRTIAERDAEMMQKLLDRDGGQSTLKDSRPGF-SIERDI  152
            S   S + EG QG  KG+R IAER+ E+  KL +  G +++  + + G  +++RD+
Sbjct  615  SNVASISNEGQQGNVKGERQIAEREIELQNKLRNTGGTEASQGNIKSGTQTVQRDV  670


>ref|WP_036141345.1| hypothetical protein [Luteibacter sp. 9135]
Length=636

 Score = 38.1 bits (87),  Expect = 3.8, Method: Compositional matrix adjust.
 Identities = 21/48 (44%), Positives = 25/48 (52%), Gaps = 0/48 (0%)

Query  85   LPSSLQPPAPPGITQTSKDSQTEEGAQGKGKRTIAERDAEMMQKLLDR  132
            +P+SL P   P I QT K    E  A   GK  I ERD   + KL+DR
Sbjct  127  IPASLMPAGGPDIDQTFKSHLNEYLATDAGKTFIHERDVTQVNKLMDR  174


>ref|XP_013865549.1| PREDICTED: adenylate cyclase type 9 [Austrofundulus limnaeus]
Length=1434

 Score = 37.7 bits (86),  Expect = 4.7, Method: Compositional matrix adjust.
 Identities = 27/81 (33%), Positives = 43/81 (53%), Gaps = 4/81 (5%)

Query  33    GRFKSF---SSSDSAPV-QTGIEGSPTTKSMKETSEGPTPIDKTSTNQPLTSHATSLPSS  88
             G+ K+F    SSDS PV Q  +  SP  ++  + S G +P D+ ++  P TS   S  ++
Sbjct  1273  GQMKTFLFPKSSDSGPVPQYQLSVSPEIRAQVDGSIGRSPTDEIASMVPTTSMIASCTNT  1332

Query  89    LQPPAPPGITQTSKDSQTEEG  109
             + P A  G +  +  SQT+E 
Sbjct  1333  MVPSASAGSSNLTGLSQTKEA  1353


>ref|XP_011664006.1| PREDICTED: uncharacterized protein LOC105438194 isoform X1 [Strongylocentrotus 
purpuratus]
 ref|XP_011664007.1| PREDICTED: uncharacterized protein LOC105438194 isoform X2 [Strongylocentrotus 
purpuratus]
Length=312

 Score = 37.4 bits (85),  Expect = 5.5, Method: Compositional matrix adjust.
 Identities = 20/51 (39%), Positives = 28/51 (55%), Gaps = 0/51 (0%)

Query  52   GSPTTKSMKETSEGPTPIDKTSTNQPLTSHATSLPSSLQPPAPPGITQTSK  102
            G+PTT    ETS  P   +K +TN P+T+ A+  P   +    PG T+ SK
Sbjct  42   GAPTTSGNIETSAAPLTAEKEATNAPVTTEASEAPGETEASEAPGETEASK  92


>dbj|GAK62257.1| 60S ribosomal protein L23 [Pseudozyma antarctica]
Length=252

 Score = 36.6 bits (83),  Expect = 9.2, Method: Compositional matrix adjust.
 Identities = 17/36 (47%), Positives = 25/36 (69%), Gaps = 0/36 (0%)

Query  104  SQTEEGAQGKGKRTIAERDAEMMQKLLDRDGGQSTL  139
            SQT + A+   K  + +RDAE++ KLLDRDGG + +
Sbjct  194  SQTAQTAEPVVKGKMLQRDAELLAKLLDRDGGSAAV  229


Lambda      K        H        a         alpha
   0.312    0.128    0.360    0.792     4.96 

Gapped
Lambda      K        H        a         alpha    sigma
   0.267   0.0410    0.140     1.90     42.6     43.6 

Effective search space used: 647747489103


  Database: nr
    Posted date:  Sep 23, 2015 12:05 AM
  Number of letters in database: 26,053,659,533
  Number of sequences in database:  71,551,133


Matrix: BLOSUM62
Gap Penalties: Existence: 11, Extension: 1
Neighboring words threshold: 11
Window for multiple hits: 40
```
