# Supplementary material for: Comparative genomics provides new insights into the diversity, physiology, and sexuality of the only industrially exploited tremellomycete: Phaffia rhodozyma
Source: BMC Genomics. 2016 Nov 9;17:901. doi: 10.1186/s12864-016-3244-7 (PMC5103461; doi:10.1186/s12864-016-3244-7)
Supplement: Additional file 6: — List of orphan genes with links to PFAM (related to Additional file 1: Table S1). (ZIP 1428 kb) [file 12864_2016_3244_MOESM6_ESM.zip › BLAST_HTML_FTR/G04332_P.html]

BLAST Search Results


```
BLASTP 2.2.27+


Reference:
Stephen F. Altschul, Thomas L. Madden, Alejandro A. Schäffer,
Jinghui Zhang, Zheng Zhang, Webb Miller, and David J. Lipman (1997),
"Gapped BLAST and PSI-BLAST: a new generation of protein database
search programs", Nucleic Acids Res. 25:3389-3402.


Reference for
composition-based statistics:
Alejandro A. Schäffer, L. Aravind, Thomas L. Madden, Sergei
Shavirin, John L. Spouge, Yuri I. Wolf, Eugene V. Koonin, and
Stephen F. Altschul (2001), "Improving the accuracy of PSI-BLAST
protein database searches with composition-based statistics and
other refinements", Nucleic Acids Res. 29:2994-3005.


Database: nr
           71,551,133 sequences; 26,053,659,533 total letters


Query= G04332_P

Length=387
                                                                      Score     E
Sequences producing significant alignments:                          (Bits)  Value

emb|CED83346.1|  hypothetical protein [Xanthophyllomyces dendrorh...   796    0.0  
ref|WP_030468961.1|  hypothetical protein [Lechevalieria aerocolo...  43.1    0.31 
ref|WP_015450836.1|  recombination factor protein RarA [Bifidobac...  43.1    0.42 
ref|WP_020527485.1|  hypothetical protein [Flexithrix dorotheae]      43.5    0.42 
ref|WP_052401493.1|  hypothetical protein [Bifidobacterium thermo...  43.1    0.42 
ref|WP_052363237.1|  hypothetical protein [Bifidobacterium therma...  43.1    0.42 
ref|WP_029575920.1|  hypothetical protein [Bifidobacterium therma...  43.1    0.42 
emb|CEF65134.1|  Translation initiation factor IF-2, mitochondria...  42.4    0.83 
ref|XP_007292699.1|  hypothetical protein MBM_04810 [Marssonina b...  42.0    0.96 
gb|EPS27021.1|  hypothetical protein PDE_01962 [Penicillium oxali...  41.6    1.1  
ref|XP_002306466.2|  hypothetical protein POPTR_0005s18100g [Popu...  40.8    2.6  
ref|XP_007761583.1|  GATA-binding protein, other eukaryote [Clado...  39.7    3.8  
gb|KOM30421.1|  hypothetical protein LR48_Vigan1345s001300 [Vigna...  39.7    6.0  
ref|XP_011005875.1|  PREDICTED: uncharacterized protein LOC105112...  39.7    7.2  
ref|XP_011005876.1|  PREDICTED: uncharacterized protein LOC105112...  39.7    7.4  
ref|XP_011005872.1|  PREDICTED: uncharacterized protein LOC105112...  39.7    7.4  
ref|XP_011005874.1|  PREDICTED: uncharacterized protein LOC105112...  39.7    7.6  
ref|XP_013298196.1|  hypothetical protein NECAME_11997 [Necator a...  39.3    8.3  


 >emb|CED83346.1| hypothetical protein [Xanthophyllomyces dendrorhous]
Length=386

 Score =  796 bits (2055),  Expect = 0.0, Method: Compositional matrix adjust.
 Identities = 385/386 (99%), Positives = 386/386 (100%), Gaps = 0/386 (0%)

Query  1    MAADRQSSDEFSQGPPSNLTRRPQSCAKCRQEGRPPILRADCRVHKRQLKKEHMNHTLAG  60
            MAADRQSSDEFSQGPPSNLTRRPQSCAKCRQEGRPPILRADCRVHKRQLKKEHMNHTLAG
Sbjct  1    MAADRQSSDEFSQGPPSNLTRRPQSCAKCRQEGRPPILRADCRVHKRQLKKEHMNHTLAG  60

Query  61   PGGAGRRPGLRFLENTTYTTLMAPANSASSSTQSVIHPLLSTQTGGPSTDRSSSSFYLHD  120
            PGGAGRRPGLRFLENTTYTTLMAPANSASSSTQSVIHPLLSTQTGGPSTDRSSSSFYLHD
Sbjct  61   PGGAGRRPGLRFLENTTYTTLMAPANSASSSTQSVIHPLLSTQTGGPSTDRSSSSFYLHD  120

Query  121  PRNMPMESLERTPMGAESAEGANVDDGLVRRAYTEQNDLSLGSDGHSTQRDDLEMDDGGM  180
            PRNMPMESLERTPMGAESAEGANVDDGLVRRAYTEQNDLSLGSDGHSTQRDDLEMDDGGM
Sbjct  121  PRNMPMESLERTPMGAESAEGANVDDGLVRRAYTEQNDLSLGSDGHSTQRDDLEMDDGGM  180

Query  181  MDGSGDEKGPGGKRLSLTQARAKAWSTPLAAEESRAKMFSSRWSRVENSAIALGQSTGAY  240
            MDGSGDEKGPGGKRLSLTQARAKAWSTPLAAEESRAKMFSSRWSRVENSAIALGQSTGAY
Sbjct  181  MDGSGDEKGPGGKRLSLTQARAKAWSTPLAAEESRAKMFSSRWSRVENSAIALGQSTGAY  240

Query  241  VALLAWRDDRVQSNTPTATHKTFLPKSHFSQSLQSLPDGTSARIYDHFVNLIRETHPRFI  300
            VALLAWRDDRVQSNTPTATHKTFLPKSHFSQSLQSLPDGTSARIYDHFVNL+RETHPRFI
Sbjct  241  VALLAWRDDRVQSNTPTATHKTFLPKSHFSQSLQSLPDGTSARIYDHFVNLVRETHPRFI  300

Query  301  EAQLESVRREAEEKVFQMEERARMEREEQDRKVKELERRLEEYQKAGTVVASSAATGSSR  360
            EAQLESVRREAEEKVFQMEERARMEREEQDRKVKELERRLEEYQKAGTVVASSAATGSSR
Sbjct  301  EAQLESVRREAEEKVFQMEERARMEREEQDRKVKELERRLEEYQKAGTVVASSAATGSSR  360

Query  361  QAAASDTVTATGIVDDKETVLISDDS  386
            QAAASDTVTATGIVDDKETVLISDDS
Sbjct  361  QAAASDTVTATGIVDDKETVLISDDS  386


>ref|WP_030468961.1| hypothetical protein [Lechevalieria aerocolonigenes]
Length=338

 Score = 43.1 bits (100),  Expect = 0.31, Method: Compositional matrix adjust.
 Identities = 36/128 (28%), Positives = 59/128 (46%), Gaps = 19/128 (15%)

Query  263  FLPKSHFSQSLQSLPDGTSARIYDHFVNLIRETHPRFIEAQLESVRREAE----EKVFQM  318
             L  +H S +L SLP G +A ++     ++R      +EA +E+VR+ AE      V+ +
Sbjct  137  VLTTTHLSVALLSLPSGRAAELF----GVLRVD----VEATIEAVRKAAEPEDGSAVWLL  188

Query  319  EERARMEREEQDRKVKELERRLEEYQKAGTVV-------ASSAATGSSRQAAASDTVTAT  371
             +   +E E     ++ L R +   Q  G  V       A+  A  + RQ  A+D V A 
Sbjct  189  RQAGALEGESGSGTMRWLARLVLRRQSTGGPVLNVVRMEATRLAVAAGRQETAADLVAAV  248

Query  372  GIVDDKET  379
             +VD + T
Sbjct  249  LVVDHQVT  256


>ref|WP_015450836.1| recombination factor protein RarA [Bifidobacterium thermophilum]
 gb|AGH41579.1| recombination factor protein RarA [Bifidobacterium thermophilum 
RBL67]
Length=505

 Score = 43.1 bits (100),  Expect = 0.42, Method: Compositional matrix adjust.
 Identities = 35/114 (31%), Positives = 51/114 (45%), Gaps = 21/114 (18%)

Query  255  TPTATHKTFLPKSHFSQSLQSLPDGTSARIYDHFVNLIRETHPRFIE-----AQLESVR-  308
            +PT +    +P +    +L   P GT      H +    E+H RFIE     A++E +R 
Sbjct  91   SPTTSQSVVVPGA----ALLYGPPGTGKTTIAHLIA--EESHRRFIELSAVNARVEELRS  144

Query  309  ---------REAEEKVFQMEERARMEREEQDRKVKELERRLEEYQKAGTVVASS  353
                     R  EE V  ++E  R  REEQD  +  +E+RL  +  A T   SS
Sbjct  145  SLSAAERLKRRGEETVLFIDEIHRYSREEQDILLPAVEKRLVTFVAATTEAPSS  198


>ref|WP_020527485.1| hypothetical protein [Flexithrix dorotheae]
Length=1173

 Score = 43.5 bits (101),  Expect = 0.42, Method: Composition-based stats.
 Identities = 22/44 (50%), Positives = 30/44 (68%), Gaps = 0/44 (0%)

Query  310  EAEEKVFQMEERARMEREEQDRKVKELERRLEEYQKAGTVVASS  353
            +A +K  Q EERA+   EE++RK KE E RLEE +K+  V+A S
Sbjct  614  QAMKKKRQQEERAKQLAEEKERKRKEQEARLEEIRKSAQVIADS  657


>ref|WP_052401493.1| hypothetical protein [Bifidobacterium thermophilum]
Length=505

 Score = 43.1 bits (100),  Expect = 0.42, Method: Compositional matrix adjust.
 Identities = 35/114 (31%), Positives = 51/114 (45%), Gaps = 21/114 (18%)

Query  255  TPTATHKTFLPKSHFSQSLQSLPDGTSARIYDHFVNLIRETHPRFIE-----AQLESVR-  308
            +PT +    +P +    +L   P GT      H +    E+H RFIE     A++E +R 
Sbjct  91   SPTTSQSVVVPGA----ALLYGPPGTGKTTIAHLIA--EESHRRFIELSAVNARVEELRS  144

Query  309  ---------REAEEKVFQMEERARMEREEQDRKVKELERRLEEYQKAGTVVASS  353
                     R  EE V  ++E  R  REEQD  +  +E+RL  +  A T   SS
Sbjct  145  SLSAAERLKRRGEETVLFIDEIHRYSREEQDILLPAVEKRLVTFVAATTEAPSS  198


>ref|WP_052363237.1| hypothetical protein [Bifidobacterium thermacidophilum]
 gb|KFI99417.1| recombination factor protein RarA [Bifidobacterium thermacidophilum 
subsp. porcinum]
Length=505

 Score = 43.1 bits (100),  Expect = 0.42, Method: Compositional matrix adjust.
 Identities = 35/114 (31%), Positives = 51/114 (45%), Gaps = 21/114 (18%)

Query  255  TPTATHKTFLPKSHFSQSLQSLPDGTSARIYDHFVNLIRETHPRFIE-----AQLESVR-  308
            +PT +    +P +    +L   P GT      H +    E+H RFIE     A++E +R 
Sbjct  91   SPTTSQSVVVPGA----ALLYGPPGTGKTTIAHLIA--EESHRRFIELSAVNARVEELRS  144

Query  309  ---------REAEEKVFQMEERARMEREEQDRKVKELERRLEEYQKAGTVVASS  353
                     R  EE V  ++E  R  REEQD  +  +E+RL  +  A T   SS
Sbjct  145  SLSAAERLKRRGEETVLFIDEIHRYSREEQDILLPAVEKRLVTFVAATTEAPSS  198


>ref|WP_029575920.1| hypothetical protein [Bifidobacterium thermacidophilum]
 gb|KFJ03406.1| AAA family ATPase [Bifidobacterium thermacidophilum subsp. thermacidophilum]
Length=505

 Score = 43.1 bits (100),  Expect = 0.42, Method: Compositional matrix adjust.
 Identities = 35/114 (31%), Positives = 51/114 (45%), Gaps = 21/114 (18%)

Query  255  TPTATHKTFLPKSHFSQSLQSLPDGTSARIYDHFVNLIRETHPRFIE-----AQLESVR-  308
            +PT +    +P +    +L   P GT      H +    E+H RFIE     A++E +R 
Sbjct  91   SPTTSQSVVVPGA----ALLYGPPGTGKTTIAHLIA--EESHRRFIELSAVNARVEELRN  144

Query  309  ---------REAEEKVFQMEERARMEREEQDRKVKELERRLEEYQKAGTVVASS  353
                     R  EE V  ++E  R  REEQD  +  +E+RL  +  A T   SS
Sbjct  145  SLSAAERLKRRGEETVLFIDEIHRYSREEQDILLPAVEKRLVTFVAATTEAPSS  198


>emb|CEF65134.1| Translation initiation factor IF-2, mitochondrial [Strongyloides 
ratti]
Length=1397

 Score = 42.4 bits (98),  Expect = 0.83, Method: Composition-based stats.
 Identities = 21/49 (43%), Positives = 33/49 (67%), Gaps = 3/49 (6%)

Query  299   FIEAQLESVRREAEEKVFQMEERARM---EREEQDRKVKELERRLEEYQ  344
              ++ + E  ++E EEK  Q+EER R+   EREE+ R++KE E R+ E+Q
Sbjct  1067  LLKEKAERTKKEREEKTKQVEERRRLKEQEREEKLREIKEKEDRIREFQ  1115


>ref|XP_007292699.1| hypothetical protein MBM_04810 [Marssonina brunnea f. sp. 'multigermtubi' 
MB_m1]
 gb|EKD17233.1| hypothetical protein MBM_04810 [Marssonina brunnea f. sp. 'multigermtubi' 
MB_m1]
Length=512

 Score = 42.0 bits (97),  Expect = 0.96, Method: Compositional matrix adjust.
 Identities = 25/92 (27%), Positives = 44/92 (48%), Gaps = 10/92 (11%)

Query  246  WRDDRVQSNTPTATHKTFLPKSHFSQSLQSLPDGTSARI----------YDHFVNLIRET  295
            W    V   T  A  + F+P++H  + L+ L D T+ ++          Y  F+  +RE 
Sbjct  402  WWHLVVNLETSIAITQNFVPRAHLGRVLEFLKDNTADQVSGFKKEVTDPYGVFLKRMREE  461

Query  296  HPRFIEAQLESVRREAEEKVFQMEERARMERE  327
            HP  +E  L  + R+AE +  + ++ AR + E
Sbjct  462  HPELLEQALAELERKAEGRKRKWDDVARNDEE  493


>gb|EPS27021.1| hypothetical protein PDE_01962 [Penicillium oxalicum 114-2]
Length=488

 Score = 41.6 bits (96),  Expect = 1.1, Method: Compositional matrix adjust.
 Identities = 26/95 (27%), Positives = 45/95 (47%), Gaps = 9/95 (9%)

Query  245  AWRDDRVQSNTPTATHKTFLPKSHFSQSLQSL---PDGTSA------RIYDHFVNLIRET  295
             W    V   +  A  + F+P++H   +L  L   PD  S         Y+HFV+ +RE 
Sbjct  375  GWWHLVVNLESSIAITQNFIPRAHIGAALDFLANKPDQVSGFRKNVENPYEHFVDGMREA  434

Query  296  HPRFIEAQLESVRREAEEKVFQMEERARMEREEQD  330
            HP  +   LE ++++A+ K  + EE    + E+ +
Sbjct  435  HPELLAQGLEELQKKADGKKRKWEEIVHGKGEQDE  469


>ref|XP_002306466.2| hypothetical protein POPTR_0005s18100g [Populus trichocarpa]
 gb|EEE93462.2| hypothetical protein POPTR_0005s18100g [Populus trichocarpa]
Length=2435

 Score = 40.8 bits (94),  Expect = 2.6, Method: Compositional matrix adjust.
 Identities = 25/92 (27%), Positives = 50/92 (54%), Gaps = 1/92 (1%)

Query  293  RETHPRFIEAQLESVRREAEEKVFQMEERARMEREEQDRKVKELERRLEEYQKAGTVVAS  352
            +E   R  + +LE++RR  E ++ + EE+ R+  EE+ RK    ++ LE  +K     A 
Sbjct  639  KEAEWRAEQERLEAIRRAEEHRIAREEEKQRISMEEERRKHSARQKLLELEEKIAKRQAE  698

Query  353  SAATGSSRQAAASDTVTATGIVDDKETVLISD  384
            +  +G+   +  +D +  TG+V +K+   ++D
Sbjct  699  ATKSGNDNSSGVTDEIM-TGMVTEKDVSRVTD  729


>ref|XP_007761583.1| GATA-binding protein, other eukaryote [Cladophialophora yegresii 
CBS 114405]
 gb|EXJ54068.1| GATA-binding protein, other eukaryote [Cladophialophora yegresii 
CBS 114405]
Length=257

 Score = 39.7 bits (91),  Expect = 3.8, Method: Compositional matrix adjust.
 Identities = 25/67 (37%), Positives = 35/67 (52%), Gaps = 1/67 (1%)

Query  279  GTSARIYDHFVNLIRETHPRFIEAQ-LESVRREAEEKVFQMEERARMEREEQDRKVKELE  337
            G +A  YD  V L      R  E + +  V  + E  + +  +RA  ER+E  RKV+ELE
Sbjct  172  GAAAMSYDDLVGLTNSLRTRVSELEVINMVYHDNESNLCRERDRALQERDEYKRKVEELE  231

Query  338  RRLEEYQ  344
            R+L E Q
Sbjct  232  RQLLEGQ  238


>gb|KOM30421.1| hypothetical protein LR48_Vigan1345s001300 [Vigna angularis]
Length=2363

 Score = 39.7 bits (91),  Expect = 6.0, Method: Compositional matrix adjust.
 Identities = 27/84 (32%), Positives = 48/84 (57%), Gaps = 2/84 (2%)

Query  301  EAQLESVRREAEEKVFQMEERARMEREEQDRKVKELERRLEEYQKAGTVVASSAATGSSR  360
            + ++E+VR+  E+++ + EE+ R+  EE+ RK    ++ LE  QK     A +A +GS+ 
Sbjct  548  QERMEAVRKAEEQRLAREEEKRRILLEEERRKQAAKQKLLELEQKIARRQAEAAKSGSNA  607

Query  361  QAAASDTVTATGIVDDKETVLISD  384
                 + V A  IV++KET   +D
Sbjct  608  PVVVEEKVPA--IVNEKETSRATD  629


>ref|XP_011005875.1| PREDICTED: uncharacterized protein LOC105112028 isoform X3 [Populus 
euphratica]
Length=2429

 Score = 39.7 bits (91),  Expect = 7.2, Method: Compositional matrix adjust.
 Identities = 25/92 (27%), Positives = 50/92 (54%), Gaps = 1/92 (1%)

Query  293  RETHPRFIEAQLESVRREAEEKVFQMEERARMEREEQDRKVKELERRLEEYQKAGTVVAS  352
            +E   R  + +LE++RR  E ++ + EE+ R+  EE+ RK    ++ LE  +K     A 
Sbjct  639  KEAEWRAEQERLEAIRRAEEHRIAREEEKQRIFMEEERRKHSARKKLLELEEKIAKRQAE  698

Query  353  SAATGSSRQAAASDTVTATGIVDDKETVLISD  384
            +  +G+   +  +D +  TG+V +K+   ++D
Sbjct  699  ATKSGNDNSSGVTDEIM-TGMVTEKDVSRVAD  729


>ref|XP_011005876.1| PREDICTED: uncharacterized protein LOC105112028 isoform X4 [Populus 
euphratica]
Length=2426

 Score = 39.7 bits (91),  Expect = 7.4, Method: Compositional matrix adjust.
 Identities = 25/92 (27%), Positives = 50/92 (54%), Gaps = 1/92 (1%)

Query  293  RETHPRFIEAQLESVRREAEEKVFQMEERARMEREEQDRKVKELERRLEEYQKAGTVVAS  352
            +E   R  + +LE++RR  E ++ + EE+ R+  EE+ RK    ++ LE  +K     A 
Sbjct  639  KEAEWRAEQERLEAIRRAEEHRIAREEEKQRIFMEEERRKHSARKKLLELEEKIAKRQAE  698

Query  353  SAATGSSRQAAASDTVTATGIVDDKETVLISD  384
            +  +G+   +  +D +  TG+V +K+   ++D
Sbjct  699  ATKSGNDNSSGVTDEIM-TGMVTEKDVSRVAD  729


>ref|XP_011005872.1| PREDICTED: uncharacterized protein LOC105112028 isoform X1 [Populus 
euphratica]
 ref|XP_011005873.1| PREDICTED: uncharacterized protein LOC105112028 isoform X1 [Populus 
euphratica]
Length=2435

 Score = 39.7 bits (91),  Expect = 7.4, Method: Compositional matrix adjust.
 Identities = 25/92 (27%), Positives = 50/92 (54%), Gaps = 1/92 (1%)

Query  293  RETHPRFIEAQLESVRREAEEKVFQMEERARMEREEQDRKVKELERRLEEYQKAGTVVAS  352
            +E   R  + +LE++RR  E ++ + EE+ R+  EE+ RK    ++ LE  +K     A 
Sbjct  639  KEAEWRAEQERLEAIRRAEEHRIAREEEKQRIFMEEERRKHSARKKLLELEEKIAKRQAE  698

Query  353  SAATGSSRQAAASDTVTATGIVDDKETVLISD  384
            +  +G+   +  +D +  TG+V +K+   ++D
Sbjct  699  ATKSGNDNSSGVTDEIM-TGMVTEKDVSRVAD  729


>ref|XP_011005874.1| PREDICTED: uncharacterized protein LOC105112028 isoform X2 [Populus 
euphratica]
Length=2433

 Score = 39.7 bits (91),  Expect = 7.6, Method: Compositional matrix adjust.
 Identities = 25/92 (27%), Positives = 50/92 (54%), Gaps = 1/92 (1%)

Query  293  RETHPRFIEAQLESVRREAEEKVFQMEERARMEREEQDRKVKELERRLEEYQKAGTVVAS  352
            +E   R  + +LE++RR  E ++ + EE+ R+  EE+ RK    ++ LE  +K     A 
Sbjct  639  KEAEWRAEQERLEAIRRAEEHRIAREEEKQRIFMEEERRKHSARKKLLELEEKIAKRQAE  698

Query  353  SAATGSSRQAAASDTVTATGIVDDKETVLISD  384
            +  +G+   +  +D +  TG+V +K+   ++D
Sbjct  699  ATKSGNDNSSGVTDEIM-TGMVTEKDVSRVAD  729


>ref|XP_013298196.1| hypothetical protein NECAME_11997 [Necator americanus]
 gb|ETN75969.1| hypothetical protein NECAME_11997 [Necator americanus]
Length=734

 Score = 39.3 bits (90),  Expect = 8.3, Method: Compositional matrix adjust.
 Identities = 29/85 (34%), Positives = 45/85 (53%), Gaps = 18/85 (21%)

Query  298  RFIEAQLESVRREAEEKVFQM-EERARMEREEQDRKVKELER-----------------R  339
            R I+ + E++RR+ EEK+ ++ EER R +RE ++R  KEL+R                 R
Sbjct  34   RLIKERHEALRRQHEEKIHKINEERQRQQRELKERHAKELKRQQDVLQRRMALMERDKAR  93

Query  340  LEEYQKAGTVVASSAATGSSRQAAA  364
             +E  +    VAS  +T SSR+  A
Sbjct  94   KQEILEKNHAVASRLSTNSSRKNYA  118


Lambda      K        H        a         alpha
   0.313    0.126    0.358    0.792     4.96 

Gapped
Lambda      K        H        a         alpha    sigma
   0.267   0.0410    0.140     1.90     42.6     43.6 

Effective search space used: 3566803519495


  Database: nr
    Posted date:  Sep 23, 2015 12:05 AM
  Number of letters in database: 26,053,659,533
  Number of sequences in database:  71,551,133


Matrix: BLOSUM62
Gap Penalties: Existence: 11, Extension: 1
Neighboring words threshold: 11
Window for multiple hits: 40
```
