# Supplementary material for: Comparative genomics provides new insights into the diversity, physiology, and sexuality of the only industrially exploited tremellomycete: Phaffia rhodozyma
Source: BMC Genomics. 2016 Nov 9;17:901. doi: 10.1186/s12864-016-3244-7 (PMC5103461; doi:10.1186/s12864-016-3244-7)
Supplement: Additional file 6: — List of orphan genes with links to PFAM (related to Additional file 1: Table S1). (ZIP 1428 kb) [file 12864_2016_3244_MOESM6_ESM.zip › BLAST_HTML_FTR/G01971_P.html]

BLAST Search Results


```
BLASTP 2.2.27+


Reference:
Stephen F. Altschul, Thomas L. Madden, Alejandro A. Schäffer,
Jinghui Zhang, Zheng Zhang, Webb Miller, and David J. Lipman (1997),
"Gapped BLAST and PSI-BLAST: a new generation of protein database
search programs", Nucleic Acids Res. 25:3389-3402.


Reference for
composition-based statistics:
Alejandro A. Schäffer, L. Aravind, Thomas L. Madden, Sergei
Shavirin, John L. Spouge, Yuri I. Wolf, Eugene V. Koonin, and
Stephen F. Altschul (2001), "Improving the accuracy of PSI-BLAST
protein database searches with composition-based statistics and
other refinements", Nucleic Acids Res. 29:2994-3005.


Database: nr
           71,551,133 sequences; 26,053,659,533 total letters


Query= G01971_P

Length=163
                                                                      Score     E
Sequences producing significant alignments:                          (Bits)  Value

emb|CDZ96812.1|  hypothetical protein [Xanthophyllomyces dendrorh...   312    4e-106
dbj|BAN82048.1|  hypothetical protein, partial [Protobothrops fla...  40.4    0.42  
gb|KFV62859.1|  BAG family molecular chaperone regulator 3, parti...  38.1    2.6   
ref|XP_009908710.1|  PREDICTED: BAG family molecular chaperone re...  38.1    2.8   
ref|XP_007478975.1|  PREDICTED: BAG family molecular chaperone re...  38.1    2.8   
ref|XP_007478974.1|  PREDICTED: BAG family molecular chaperone re...  38.1    2.9   
ref|XP_003755368.1|  PREDICTED: BAG family molecular chaperone re...  38.1    3.1   
gb|KFO87231.1|  BAG family molecular chaperone regulator 3, parti...  37.4    4.3   
ref|XP_009693604.1|  PREDICTED: BAG family molecular chaperone re...  37.4    5.1   
gb|KFQ16952.1|  BAG family molecular chaperone regulator 3, parti...  37.4    5.3   
gb|KFV19443.1|  BAG family molecular chaperone regulator 3, parti...  37.4    5.3   
ref|XP_008942080.1|  PREDICTED: BAG family molecular chaperone re...  37.4    5.4   
ref|XP_010130366.1|  PREDICTED: BAG family molecular chaperone re...  37.4    5.6   
gb|KFP56712.1|  BAG family molecular chaperone regulator 3, parti...  37.0    6.4   
ref|XP_009986949.1|  PREDICTED: BAG family molecular chaperone re...  37.0    7.5   
emb|CEL63117.1|  hypothetical protein RSOLAG1IB_05157 [Rhizoctoni...  37.0    7.5   


 >emb|CDZ96812.1| hypothetical protein [Xanthophyllomyces dendrorhous]
Length=162

 Score =  312 bits (800),  Expect = 4e-106, Method: Compositional matrix adjust.
 Identities = 162/162 (100%), Positives = 162/162 (100%), Gaps = 0/162 (0%)

Query  1    MSGISHREELDNDSASTDEDASEEEFEDSEEEQDEQFDTSLAPQLRSELQPQPQPQSSQS  60
            MSGISHREELDNDSASTDEDASEEEFEDSEEEQDEQFDTSLAPQLRSELQPQPQPQSSQS
Sbjct  1    MSGISHREELDNDSASTDEDASEEEFEDSEEEQDEQFDTSLAPQLRSELQPQPQPQSSQS  60

Query  61   LNQIMELEQTLQSLLTEKLPTLNRLQLEGHLTNLLLEADRVDPHGEFDEDAVREKRRELV  120
            LNQIMELEQTLQSLLTEKLPTLNRLQLEGHLTNLLLEADRVDPHGEFDEDAVREKRRELV
Sbjct  61   LNQIMELEQTLQSLLTEKLPTLNRLQLEGHLTNLLLEADRVDPHGEFDEDAVREKRRELV  120

Query  121  RAVERVLGGDEDTKGNADLDAEERRAEEEENEVESELQPLTI  162
            RAVERVLGGDEDTKGNADLDAEERRAEEEENEVESELQPLTI
Sbjct  121  RAVERVLGGDEDTKGNADLDAEERRAEEEENEVESELQPLTI  162


>dbj|BAN82048.1| hypothetical protein, partial [Protobothrops flavoviridis]
Length=327

 Score = 40.4 bits (93),  Expect = 0.42, Method: Compositional matrix adjust.
 Identities = 27/68 (40%), Positives = 38/68 (56%), Gaps = 4/68 (6%)

Query  61   LNQIMELEQTLQSLLTEKLPTLNRLQLEGHLTNLLLEADRVDPHGEFDEDAVREKRRELV  120
            LN++  L+Q + S   +K      L +E +LT  LL  D VDP G  D   VR+ RR+ V
Sbjct  181  LNRVQILKQEVDSFQGKKN-DKKYLWIEEYLTKELLALDSVDPEGRAD---VRQARRDGV  236

Query  121  RAVERVLG  128
            R V+ +LG
Sbjct  237  RKVQNILG  244


>gb|KFV62859.1| BAG family molecular chaperone regulator 3, partial [Picoides 
pubescens]
Length=496

 Score = 38.1 bits (87),  Expect = 2.6, Method: Compositional matrix adjust.
 Identities = 26/67 (39%), Positives = 36/67 (54%), Gaps = 4/67 (6%)

Query  61   LNQIMELEQTLQSLLTEKLPTLNRLQLEGHLTNLLLEADRVDPHGEFDEDAVREKRRELV  120
            L ++  LEQ + S   +K      L +E +LT  LL  D VDP G  D   VR+ RR+ V
Sbjct  349  LEKVQMLEQAVNSFEGKKT-DKKYLMIEEYLTKELLALDSVDPEGRAD---VRQARRDGV  404

Query  121  RAVERVL  127
            R V+ +L
Sbjct  405  RKVQNIL  411


>ref|XP_009908710.1| PREDICTED: BAG family molecular chaperone regulator 3 [Picoides 
pubescens]
Length=511

 Score = 38.1 bits (87),  Expect = 2.8, Method: Compositional matrix adjust.
 Identities = 26/67 (39%), Positives = 36/67 (54%), Gaps = 4/67 (6%)

Query  61   LNQIMELEQTLQSLLTEKLPTLNRLQLEGHLTNLLLEADRVDPHGEFDEDAVREKRRELV  120
            L ++  LEQ + S   +K      L +E +LT  LL  D VDP G  D   VR+ RR+ V
Sbjct  364  LEKVQMLEQAVNSFEGKKT-DKKYLMIEEYLTKELLALDSVDPEGRAD---VRQARRDGV  419

Query  121  RAVERVL  127
            R V+ +L
Sbjct  420  RKVQNIL  426


>ref|XP_007478975.1| PREDICTED: BAG family molecular chaperone regulator 3 isoform 
X2 [Monodelphis domestica]
Length=565

 Score = 38.1 bits (87),  Expect = 2.8, Method: Compositional matrix adjust.
 Identities = 26/67 (39%), Positives = 37/67 (55%), Gaps = 4/67 (6%)

Query  61   LNQIMELEQTLQSLLTEKLPTLNRLQLEGHLTNLLLEADRVDPHGEFDEDAVREKRRELV  120
            L ++  LEQ + S   +K      L +E +LT  LL  D VDP G  D   VR+ RR+ V
Sbjct  416  LEKVQGLEQAVDSFEGKKT-DKQYLMIEEYLTKELLALDSVDPEGRAD---VRQARRDGV  471

Query  121  RAVERVL  127
            R V+++L
Sbjct  472  RKVQQIL  478


>ref|XP_007478974.1| PREDICTED: BAG family molecular chaperone regulator 3 isoform 
X1 [Monodelphis domestica]
Length=586

 Score = 38.1 bits (87),  Expect = 2.9, Method: Compositional matrix adjust.
 Identities = 26/67 (39%), Positives = 37/67 (55%), Gaps = 4/67 (6%)

Query  61   LNQIMELEQTLQSLLTEKLPTLNRLQLEGHLTNLLLEADRVDPHGEFDEDAVREKRRELV  120
            L ++  LEQ + S   +K      L +E +LT  LL  D VDP G  D   VR+ RR+ V
Sbjct  437  LEKVQGLEQAVDSFEGKKT-DKQYLMIEEYLTKELLALDSVDPEGRAD---VRQARRDGV  492

Query  121  RAVERVL  127
            R V+++L
Sbjct  493  RKVQQIL  499


>ref|XP_003755368.1| PREDICTED: BAG family molecular chaperone regulator 3 [Sarcophilus 
harrisii]
Length=573

 Score = 38.1 bits (87),  Expect = 3.1, Method: Compositional matrix adjust.
 Identities = 36/112 (32%), Positives = 56/112 (50%), Gaps = 10/112 (9%)

Query  22   SEEEFEDSEEEQDEQFDTS--LAPQLRSELQPQPQPQSSQSLNQIME----LEQTLQSLL  75
            S  +  D+EE       +S  LAPQ  ++++  P+      +  I+E    LE+ + S  
Sbjct  383  SSSKIVDAEERPTSPIPSSPELAPQKPADVEAVPKHPGVMKVEAILEKVQGLEEAVNSFE  442

Query  76   TEKLPTLNRLQLEGHLTNLLLEADRVDPHGEFDEDAVREKRRELVRAVERVL  127
             +K      L +E +LT  LL  D VDP G  D   VR+ RR+ VR V+++L
Sbjct  443  GKKT-DKKYLMIEEYLTKELLALDSVDPEGCAD---VRQARRDGVRKVQQIL  490


>gb|KFO87231.1| BAG family molecular chaperone regulator 3, partial [Buceros 
rhinoceros silvestris]
Length=477

 Score = 37.4 bits (85),  Expect = 4.3, Method: Compositional matrix adjust.
 Identities = 26/67 (39%), Positives = 36/67 (54%), Gaps = 4/67 (6%)

Query  61   LNQIMELEQTLQSLLTEKLPTLNRLQLEGHLTNLLLEADRVDPHGEFDEDAVREKRRELV  120
            L ++  LEQ + S   +K      L +E +LT  LL  D VDP G  D   VR+ RR+ V
Sbjct  330  LEKVQMLEQAVDSFEGKKT-DKKYLMIEEYLTKELLALDSVDPEGRAD---VRQARRDGV  385

Query  121  RAVERVL  127
            R V+ +L
Sbjct  386  RKVQNIL  392


>ref|XP_009693604.1| PREDICTED: BAG family molecular chaperone regulator 3 [Cariama 
cristata]
Length=512

 Score = 37.4 bits (85),  Expect = 5.1, Method: Compositional matrix adjust.
 Identities = 26/67 (39%), Positives = 36/67 (54%), Gaps = 4/67 (6%)

Query  61   LNQIMELEQTLQSLLTEKLPTLNRLQLEGHLTNLLLEADRVDPHGEFDEDAVREKRRELV  120
            L ++  LEQ + S   +K      L +E +LT  LL  D VDP G  D   VR+ RR+ V
Sbjct  365  LEKVQMLEQAVDSFEGKKT-DKKYLMIEEYLTKELLALDSVDPEGRAD---VRQARRDGV  420

Query  121  RAVERVL  127
            R V+ +L
Sbjct  421  RKVQNIL  427


>gb|KFQ16952.1| BAG family molecular chaperone regulator 3, partial [Merops nubicus]
Length=381

 Score = 37.4 bits (85),  Expect = 5.3, Method: Compositional matrix adjust.
 Identities = 26/67 (39%), Positives = 36/67 (54%), Gaps = 4/67 (6%)

Query  61   LNQIMELEQTLQSLLTEKLPTLNRLQLEGHLTNLLLEADRVDPHGEFDEDAVREKRRELV  120
            L ++  LEQ + S   +K      L +E +LT  LL  D VDP G  D   VR+ RR+ V
Sbjct  234  LEKVQMLEQAVDSFEGKKT-DKKYLMIEEYLTKELLALDSVDPEGRAD---VRQARRDGV  289

Query  121  RAVERVL  127
            R V+ +L
Sbjct  290  RKVQNIL  296


>gb|KFV19443.1| BAG family molecular chaperone regulator 3, partial [Tauraco 
erythrolophus]
Length=497

 Score = 37.4 bits (85),  Expect = 5.3, Method: Compositional matrix adjust.
 Identities = 26/67 (39%), Positives = 36/67 (54%), Gaps = 4/67 (6%)

Query  61   LNQIMELEQTLQSLLTEKLPTLNRLQLEGHLTNLLLEADRVDPHGEFDEDAVREKRRELV  120
            L ++  LEQ + S   +K      L +E +LT  LL  D VDP G  D   VR+ RR+ V
Sbjct  350  LEKVQMLEQAVDSFEGKKT-DKKYLMIEEYLTKELLALDSVDPEGRAD---VRQARRDGV  405

Query  121  RAVERVL  127
            R V+ +L
Sbjct  406  RKVQNIL  412


>ref|XP_008942080.1| PREDICTED: BAG family molecular chaperone regulator 3, partial 
[Merops nubicus]
Length=384

 Score = 37.4 bits (85),  Expect = 5.4, Method: Compositional matrix adjust.
 Identities = 26/67 (39%), Positives = 36/67 (54%), Gaps = 4/67 (6%)

Query  61   LNQIMELEQTLQSLLTEKLPTLNRLQLEGHLTNLLLEADRVDPHGEFDEDAVREKRRELV  120
            L ++  LEQ + S   +K      L +E +LT  LL  D VDP G  D   VR+ RR+ V
Sbjct  237  LEKVQMLEQAVDSFEGKKT-DKKYLMIEEYLTKELLALDSVDPEGRAD---VRQARRDGV  292

Query  121  RAVERVL  127
            R V+ +L
Sbjct  293  RKVQNIL  299


>ref|XP_010130366.1| PREDICTED: BAG family molecular chaperone regulator 3 [Buceros 
rhinoceros silvestris]
Length=580

 Score = 37.4 bits (85),  Expect = 5.6, Method: Compositional matrix adjust.
 Identities = 26/67 (39%), Positives = 36/67 (54%), Gaps = 4/67 (6%)

Query  61   LNQIMELEQTLQSLLTEKLPTLNRLQLEGHLTNLLLEADRVDPHGEFDEDAVREKRRELV  120
            L ++  LEQ + S   +K      L +E +LT  LL  D VDP G  D   VR+ RR+ V
Sbjct  433  LEKVQMLEQAVDSFEGKKT-DKKYLMIEEYLTKELLALDSVDPEGRAD---VRQARRDGV  488

Query  121  RAVERVL  127
            R V+ +L
Sbjct  489  RKVQNIL  495


>gb|KFP56712.1| BAG family molecular chaperone regulator 3, partial [Cariama 
cristata]
Length=501

 Score = 37.0 bits (84),  Expect = 6.4, Method: Compositional matrix adjust.
 Identities = 26/67 (39%), Positives = 36/67 (54%), Gaps = 4/67 (6%)

Query  61   LNQIMELEQTLQSLLTEKLPTLNRLQLEGHLTNLLLEADRVDPHGEFDEDAVREKRRELV  120
            L ++  LEQ + S   +K      L +E +LT  LL  D VDP G  D   VR+ RR+ V
Sbjct  354  LEKVQMLEQAVDSFEGKKT-DKKYLMIEEYLTKELLALDSVDPEGRAD---VRQARRDGV  409

Query  121  RAVERVL  127
            R V+ +L
Sbjct  410  RKVQNIL  416


>ref|XP_009986949.1| PREDICTED: BAG family molecular chaperone regulator 3-like [Tauraco 
erythrolophus]
Length=540

 Score = 37.0 bits (84),  Expect = 7.5, Method: Compositional matrix adjust.
 Identities = 26/67 (39%), Positives = 36/67 (54%), Gaps = 4/67 (6%)

Query  61   LNQIMELEQTLQSLLTEKLPTLNRLQLEGHLTNLLLEADRVDPHGEFDEDAVREKRRELV  120
            L ++  LEQ + S   +K      L +E +LT  LL  D VDP G  D   VR+ RR+ V
Sbjct  393  LEKVQMLEQAVDSFEGKKT-DKKYLMIEEYLTKELLALDSVDPEGRAD---VRQARRDGV  448

Query  121  RAVERVL  127
            R V+ +L
Sbjct  449  RKVQNIL  455


>emb|CEL63117.1| hypothetical protein RSOLAG1IB_05157 [Rhizoctonia solani AG-1 
IB]
Length=450

 Score = 37.0 bits (84),  Expect = 7.5, Method: Compositional matrix adjust.
 Identities = 37/123 (30%), Positives = 59/123 (48%), Gaps = 20/123 (16%)

Query  57   SSQSLNQIMELEQTLQS---------LLTEKLPTL----NRLQLEGH---LTNLLLEADR  100
            S  ++  I+    TLQS          L +  PTL    N   L G+   LT LL + D 
Sbjct  172  SHAAIQSILASFATLQSEFTFPSQLDFLPDSSPTLAYTPNNAPLHGYEHALTGLLTKLDG  231

Query  101  VDPHGEFDEDAVREKRRELVRAVERVLGGDEDTKGNADLDAEERRAEEEENEVE-SELQP  159
            V+ +G+ D   VR  R+E V+A+E+ L   +  K +      ER+AE    E + +E+ P
Sbjct  232  VESYGDGD---VRRARKEAVKAIEKELERLDGMKADRWRYVSERQAEVTTAEADRAEVDP  288

Query  160  LTI  162
            +++
Sbjct  289  VSV  291


Lambda      K        H        a         alpha
   0.307    0.128    0.334    0.792     4.96 

Gapped
Lambda      K        H        a         alpha    sigma
   0.267   0.0410    0.140     1.90     42.6     43.6 

Effective search space used: 650171180504


  Database: nr
    Posted date:  Sep 23, 2015 12:05 AM
  Number of letters in database: 26,053,659,533
  Number of sequences in database:  71,551,133


Matrix: BLOSUM62
Gap Penalties: Existence: 11, Extension: 1
Neighboring words threshold: 11
Window for multiple hits: 40
```
