# Supplementary material for: Comparative genomics provides new insights into the diversity, physiology, and sexuality of the only industrially exploited tremellomycete: Phaffia rhodozyma
Source: BMC Genomics. 2016 Nov 9;17:901. doi: 10.1186/s12864-016-3244-7 (PMC5103461; doi:10.1186/s12864-016-3244-7)
Supplement: Additional file 6: — List of orphan genes with links to PFAM (related to Additional file 1: Table S1). (ZIP 1428 kb) [file 12864_2016_3244_MOESM6_ESM.zip › BLAST_HTML_FTR/G02527_P.html]

BLAST Search Results


```
BLASTP 2.2.27+


Reference:
Stephen F. Altschul, Thomas L. Madden, Alejandro A. Schäffer,
Jinghui Zhang, Zheng Zhang, Webb Miller, and David J. Lipman (1997),
"Gapped BLAST and PSI-BLAST: a new generation of protein database
search programs", Nucleic Acids Res. 25:3389-3402.


Reference for
composition-based statistics:
Alejandro A. Schäffer, L. Aravind, Thomas L. Madden, Sergei
Shavirin, John L. Spouge, Yuri I. Wolf, Eugene V. Koonin, and
Stephen F. Altschul (2001), "Improving the accuracy of PSI-BLAST
protein database searches with composition-based statistics and
other refinements", Nucleic Acids Res. 29:2994-3005.


Database: nr
           71,551,133 sequences; 26,053,659,533 total letters


Query= G02527_P

Length=930
                                                                      Score     E
Sequences producing significant alignments:                          (Bits)  Value

emb|CED82340.1|  hypothetical protein [Xanthophyllomyces dendrorh...  1323    0.0  


 >emb|CED82340.1| hypothetical protein [Xanthophyllomyces dendrorhous]
Length=958

 Score = 1323 bits (3423),  Expect = 0.0, Method: Compositional matrix adjust.
 Identities = 910/944 (96%), Positives = 912/944 (97%), Gaps = 15/944 (2%)

Query  1    MAWGMKTGGEDSEDEGESGHADRFHPSNFPLVPSSIEPGASRVIARRAPSISSQTSRTFE  60
            MAWGMKTGGEDSEDEGESGHADRFHPSNFPLVPSSIEPGASRVIARRAPSISSQTSRTFE
Sbjct  15   MAWGMKTGGEDSEDEGESGHADRFHPSNFPLVPSSIEPGASRVIARRAPSISSQTSRTFE  74

Query  61   LAINAIPSSSALDSHPISSGPSGSSNRRRRVDAAESNAGSRRTTSLAPLGPSASTLYPEP  120
            LAINAIPSSSALDSHPISSGPSGSSNRRRRVDAAESNAGSRRTTSLAPLGPSASTLYPEP
Sbjct  75   LAINAIPSSSALDSHPISSGPSGSSNRRRRVDAAESNAGSRRTTSLAPLGPSASTLYPEP  134

Query  121  RRPARQPSGRFVAPDVRFPPSVSSSSLAVSPTQIGSPASRNPSWASSSSRPSPPQTPRPV  180
            RRPARQPSGRFVAPDVRFPPSVSSSSLAVSPTQIGSPASRNPSWASSSSRPSPPQTPRPV
Sbjct  135  RRPARQPSGRFVAPDVRFPPSVSSSSLAVSPTQIGSPASRNPSWASSSSRPSPPQTPRPV  194

Query  181  SPSRPSSPTQSALSYLNAPSLVSFPSRPESVHGEPVESFGALPGGNHRAILLQRLGDAEE  240
            SPSRPSSPTQSALSYLNAPSLVSFPSRPESVHGEPVESFGALPGGNHRAILLQRLGDAEE
Sbjct  195  SPSRPSSPTQSALSYLNAPSLVSFPSRPESVHGEPVESFGALPGGNHRAILLQRLGDAEE  254

Query  241  AAEEDPRLAQALGVLLEMGYVGSIGSAG-------VPQFIIFQVF-------LRLPDRLP  286
            AAEEDPRLAQALGVLLEMGYVGSIGSAG         +F+  Q         L  P R  
Sbjct  255  AAEEDPRLAQALGVLLEMGYVGSIGSAGNTRPNGESDRFVFVQSAPVHHLPSLSSPARSA  314

Query  287  QTSDL-TSNRRTSSNLTSTTASGSGSSPASRGRPRRRAAGATRRSTRSNREPVALAQANP  345
               D  TSNRRTSSNLTSTTASGSGSSPASRGRPRRRAAGATRRSTRSNREPVALAQANP
Sbjct  315  TPDDRPTSNRRTSSNLTSTTASGSGSSPASRGRPRRRAAGATRRSTRSNREPVALAQANP  374

Query  346  SPASSTRSRNSAVRSSISELVTGHGRDHSSPTTEERQTENRQEKEGEEAVEEPIDESDGR  405
            SPASSTRSRNSAVRSSISELVTGHGRDHSSPTTEERQTENRQEKEGEEAVEEPIDESDGR
Sbjct  375  SPASSTRSRNSAVRSSISELVTGHGRDHSSPTTEERQTENRQEKEGEEAVEEPIDESDGR  434

Query  406  NRVVSNPRPRRRRRPDGPSPLESAESVSVSSLAETTSSYSTARSNTSLPTPSPEIDSRVS  465
            NRVVSNPRPRRRRRPDGPSPLESAESVSVSSLAETTSSYSTARSNTSLPTPSPEIDSRVS
Sbjct  435  NRVVSNPRPRRRRRPDGPSPLESAESVSVSSLAETTSSYSTARSNTSLPTPSPEIDSRVS  494

Query  466  SRSSLLALDTVQPSQHSLIQTVQDDEDIPIALRRTPRSSTAPFAPTSARIGRTRNSSNAS  525
            SRSSLLALDTVQPSQHSLIQTVQDDEDIPIALRRTPRSSTAPFAPTSARIGRTRNSSNAS
Sbjct  495  SRSSLLALDTVQPSQHSLIQTVQDDEDIPIALRRTPRSSTAPFAPTSARIGRTRNSSNAS  554

Query  526  SSTSLSQPPNNRLSSGLPTPITTASERLSPMPLTTSFPPVEIPNVDPEAHPVSDCLRTSQ  585
            SSTSLSQPPNNRLSSGLPTPITTASERLSPMPLTTSFPPVEIPNVDPEAHPVSDCLRTSQ
Sbjct  555  SSTSLSQPPNNRLSSGLPTPITTASERLSPMPLTTSFPPVEIPNVDPEAHPVSDCLRTSQ  614

Query  586  VSQLIPPDVFQSSSVITDPISSLSTSGMSGTHESRAGEDPLRNVPGLFELGRGLFEGFIA  645
            VSQLIPPDVFQSSSVITDPISSLSTSGMSGTHESRAGEDPLRNVPGLFELGRGLFEGFIA
Sbjct  615  VSQLIPPDVFQSSSVITDPISSLSTSGMSGTHESRAGEDPLRNVPGLFELGRGLFEGFIA  674

Query  646  APSSASTLPVVNGEFVGEGRGMTLTRSRSNRSRRRLSASGGTTDQTNNPVMRGTRCSTVP  705
            APSSASTLPVVNGEFVGEGRGMTLTRSRSNRSRRRLS SGGTTDQTNNPVMRGTRCSTVP
Sbjct  675  APSSASTLPVVNGEFVGEGRGMTLTRSRSNRSRRRLSTSGGTTDQTNNPVMRGTRCSTVP  734

Query  706  PLSTPSESETLPTTTTATTMSEYVAHGIQPRLRTFLRSDRPLSTLSTSARAIDSTPFLRN  765
            PLSTPSESETLPTTTTATTMSEYVAHGIQPRLRTFLRSDRPLSTLSTSARAIDSTPFLRN
Sbjct  735  PLSTPSESETLPTTTTATTMSEYVAHGIQPRLRTFLRSDRPLSTLSTSARAIDSTPFLRN  794

Query  766  IRPPTTRSRSSLPSIRTSHPSGPTTVSNFTWTPTQRSTVSSSVTTTMTASASQIPASPTH  825
            IRPPTTRSRSSLPSIRTSHP GPTTVSNFTWTPTQRSTVSSSVTTTMTASASQIPASPTH
Sbjct  795  IRPPTTRSRSSLPSIRTSHPPGPTTVSNFTWTPTQRSTVSSSVTTTMTASASQIPASPTH  854

Query  826  TLLSLTSTMSSENDLPLDRNPPPAYERSLGERAEYVAPPPRPRRGVRQREEATEGNSMSG  885
            TLLSLTSTMSSENDLPLDRNPPPAYERSLGERAEYVAPPPRPRRGVRQREEATEGNSMSG
Sbjct  855  TLLSLTSTMSSENDLPLDRNPPPAYERSLGERAEYVAPPPRPRRGVRQREEATEGNSMSG  914

Query  886  LAGRLLGGGTLRDPDMRPRNSISRGEGEGENRLMARGRSLRGLR  929
            LAGRLLGGGTLRDPDMRPRNSISRGEGEGENRLMARGRSLRGLR
Sbjct  915  LAGRLLGGGTLRDPDMRPRNSISRGEGEGENRLMARGRSLRGLR  958


Lambda      K        H        a         alpha
   0.309    0.124    0.349    0.792     4.96 

Gapped
Lambda      K        H        a         alpha    sigma
   0.267   0.0410    0.140     1.90     42.6     43.6 

Effective search space used: 11176589955280


  Database: nr
    Posted date:  Sep 23, 2015 12:05 AM
  Number of letters in database: 26,053,659,533
  Number of sequences in database:  71,551,133


Matrix: BLOSUM62
Gap Penalties: Existence: 11, Extension: 1
Neighboring words threshold: 11
Window for multiple hits: 40
```
