# Supplementary material for: Comparative genomics provides new insights into the diversity, physiology, and sexuality of the only industrially exploited tremellomycete: Phaffia rhodozyma
Source: BMC Genomics. 2016 Nov 9;17:901. doi: 10.1186/s12864-016-3244-7 (PMC5103461; doi:10.1186/s12864-016-3244-7)
Supplement: Additional file 6: — List of orphan genes with links to PFAM (related to Additional file 1: Table S1). (ZIP 1428 kb) [file 12864_2016_3244_MOESM6_ESM.zip › BLAST_HTML_FTR/G05867_P.html]

BLAST Search Results


```
BLASTP 2.2.27+


Reference:
Stephen F. Altschul, Thomas L. Madden, Alejandro A. Schäffer,
Jinghui Zhang, Zheng Zhang, Webb Miller, and David J. Lipman (1997),
"Gapped BLAST and PSI-BLAST: a new generation of protein database
search programs", Nucleic Acids Res. 25:3389-3402.


Reference for
composition-based statistics:
Alejandro A. Schäffer, L. Aravind, Thomas L. Madden, Sergei
Shavirin, John L. Spouge, Yuri I. Wolf, Eugene V. Koonin, and
Stephen F. Altschul (2001), "Improving the accuracy of PSI-BLAST
protein database searches with composition-based statistics and
other refinements", Nucleic Acids Res. 29:2994-3005.


Database: nr
           71,551,133 sequences; 26,053,659,533 total letters


Query= G05867_P

Length=560
                                                                      Score     E
Sequences producing significant alignments:                          (Bits)  Value

emb|CED83384.1|  hypothetical protein [Xanthophyllomyces dendrorh...  1056    0.0  
ref|WP_015431815.1|  hypothetical protein [Bibersteinia trehalosi...  43.1    0.26 
dbj|GAD91928.1|  conserved hypothetical protein [Byssochlamys spe...  41.2    3.1  


 >emb|CED83384.1| hypothetical protein [Xanthophyllomyces dendrorhous]
Length=529

 Score = 1056 bits (2730),  Expect = 0.0, Method: Compositional matrix adjust.
 Identities = 529/559 (95%), Positives = 529/559 (95%), Gaps = 30/559 (5%)

Query  1    MPDLEKLSRSLKAPPEFPQNGRTPSQAKIASLESQLLTLQTTLSESLNEMSKHCEENFNL  60
            MPDLEKLSRSLKAPPEFPQNGRTPSQAKIASLESQLLTLQTTLSESLNEMSKHCEENFNL
Sbjct  1    MPDLEKLSRSLKAPPEFPQNGRTPSQAKIASLESQLLTLQTTLSESLNEMSKHCEENFNL  60

Query  61   TERVCELEQALYDMSAAERECNRRKNWLEDELNRLRAHSPLFVPTETTRGLSTMSAGVSR  120
            TERVCELEQALYDMSAAERECNRRKNWLEDELNRLRAHSPLFVPTETTRGLSTMSAGVSR
Sbjct  61   TERVCELEQALYDMSAAERECNRRKNWLEDELNRLRAHSPLFVPTETTRGLSTMSAGVSR  120

Query  121  AMHSCKSTKRSDYTLDPAGGSVKTLACAIVGDCFVSVNPESFAPRLNSLNSRPEWYGLQP  180
            AMHSCKSTKRSDYTLDPAGGSVKTLACAI                              P
Sbjct  121  AMHSCKSTKRSDYTLDPAGGSVKTLACAI------------------------------P  150

Query  181  SRMQSTPSPSPNITLRTPARISRSRALPNTTANNNSLQCIPSPNTRIPVLSASYESRINP  240
            SRMQSTPSPSPNITLRTPARISRSRALPNTTANNNSLQCIPSPNTRIPVLSASYESRINP
Sbjct  151  SRMQSTPSPSPNITLRTPARISRSRALPNTTANNNSLQCIPSPNTRIPVLSASYESRINP  210

Query  241  RPASQPLASPLHIGSSHASSQTIISISRSRSTGDVPSPSILKSKTHCSPQPVRTSRSSHV  300
            RPASQPLASPLHIGSSHASSQTIISISRSRSTGDVPSPSILKSKTHCSPQPVRTSRSSHV
Sbjct  211  RPASQPLASPLHIGSSHASSQTIISISRSRSTGDVPSPSILKSKTHCSPQPVRTSRSSHV  270

Query  301  LSSESFGVFDSKRGVIYGNELFTSPIRRSDKKGHDDVWEEGASAWNTLAGSRPDWTGFDS  360
            LSSESFGVFDSKRGVIYGNELFTSPIRRSDKKGHDDVWEEGASAWNTLAGSRPDWTGFDS
Sbjct  271  LSSESFGVFDSKRGVIYGNELFTSPIRRSDKKGHDDVWEEGASAWNTLAGSRPDWTGFDS  330

Query  361  SPEGVVNTDKLLKHMYTPPTMTAEDERSCFLAESNKENYFGRSPLKTPHLLTQRPSVSKA  420
            SPEGVVNTDKLLKHMYTPPTMTAEDERSCFLAESNKENYFGRSPLKTPHLLTQRPSVSKA
Sbjct  331  SPEGVVNTDKLLKHMYTPPTMTAEDERSCFLAESNKENYFGRSPLKTPHLLTQRPSVSKA  390

Query  421  GSKFSTRIPASTSFRSKFTRTAPSFDQLELGFESSDRFDSQRTLVDLDLSSASTRTRVLS  480
            GSKFSTRIPASTSFRSKFTRTAPSFDQLELGFESSDRFDSQRTLVDLDLSSASTRTRVLS
Sbjct  391  GSKFSTRIPASTSFRSKFTRTAPSFDQLELGFESSDRFDSQRTLVDLDLSSASTRTRVLS  450

Query  481  PASPTEQPSTRTGLDGSRSSLDRKPPFSSSHLSFHERNPRSIQNAQSTNRSLKKVIPTRY  540
            PASPTEQPSTRTGLDGSRSSLDRKPPFSSSHLSFHERNPRSIQNAQSTNRSLKKVIPTRY
Sbjct  451  PASPTEQPSTRTGLDGSRSSLDRKPPFSSSHLSFHERNPRSIQNAQSTNRSLKKVIPTRY  510

Query  541  EAGSSRTTRITRTGYATGG  559
            EAGSSRTTRITRTGYATGG
Sbjct  511  EAGSSRTTRITRTGYATGG  529


>ref|WP_015431815.1| hypothetical protein [Bibersteinia trehalosi]
 gb|AGH37676.1| hypothetical protein WQG_3960 [Bibersteinia trehalosi USDA-ARS-USMARC-192]
 gb|AHG82515.1| hypothetical protein F542_18000 [Bibersteinia trehalosi USDA-ARS-USMARC-188]
 gb|AHG84849.1| hypothetical protein F543_19880 [Bibersteinia trehalosi USDA-ARS-USMARC-189]
 gb|AHG85667.1| hypothetical protein F544_4350 [Bibersteinia trehalosi USDA-ARS-USMARC-190]
Length=188

 Score = 43.1 bits (100),  Expect = 0.26, Method: Compositional matrix adjust.
 Identities = 30/97 (31%), Positives = 46/97 (47%), Gaps = 20/97 (21%)

Query  20   NGRTPSQAKIASLESQLLTLQTTLSESLNEMSKHCEENF----------------NLTER  63
            NG+T  QA++  L++QL  LQ T  E + E+ K  E+                  NL E+
Sbjct  22   NGKTELQAEVQHLKAQLSELQKTSDEKIAEIRKEAEDEIAIAQRLADEKVAAAQKNLEEK  81

Query  64   VC----ELEQALYDMSAAERECNRRKNWLEDELNRLR  96
            +     E+E A  +   A +E N +K+ L D LN L+
Sbjct  82   LASLQKEVEAAKKEAEQARKEANEKKDKLSDALNGLK  118


>dbj|GAD91928.1| conserved hypothetical protein [Byssochlamys spectabilis No. 
5]
Length=729

 Score = 41.2 bits (95),  Expect = 3.1, Method: Compositional matrix adjust.
 Identities = 36/127 (28%), Positives = 57/127 (45%), Gaps = 11/127 (9%)

Query  25   SQAKIASLESQLLTLQTTLSESLNEMSKH----CEENFNLTERVCELE----QALYDMSA  76
            S+ K ASLE +  TL+ +LS    EM+K+     E+   L +R  +LE    +    + A
Sbjct  388  SEQKAASLEKENATLKQSLSRVKQEMTKYEGRRKEKEAKLKQREAKLEARNQEYRERLKA  447

Query  77   AERECNRRKNWLEDELNRLRAHSPLFVPTETTRGLSTMSAGVSRAMHSCK---STKRSDY  133
            A +E    +    +E  R++ H      T T+ GLS    G + A    K     +  D+
Sbjct  448  ANQERRDAEQSFSEERQRMQEHIDALQGTITSLGLSKTGTGAAHAYSPAKRFSGVETYDF  507

Query  134  TLDPAGG  140
             L+P  G
Sbjct  508  ALEPQNG  514


Lambda      K        H        a         alpha
   0.312    0.126    0.365    0.792     4.96 

Gapped
Lambda      K        H        a         alpha    sigma
   0.267   0.0410    0.140     1.90     42.6     43.6 

Effective search space used: 6016239845140


  Database: nr
    Posted date:  Sep 23, 2015 12:05 AM
  Number of letters in database: 26,053,659,533
  Number of sequences in database:  71,551,133


Matrix: BLOSUM62
Gap Penalties: Existence: 11, Extension: 1
Neighboring words threshold: 11
Window for multiple hits: 40
```
