# Supplementary material for: Comparative genomics provides new insights into the diversity, physiology, and sexuality of the only industrially exploited tremellomycete: Phaffia rhodozyma
Source: BMC Genomics. 2016 Nov 9;17:901. doi: 10.1186/s12864-016-3244-7 (PMC5103461; doi:10.1186/s12864-016-3244-7)
Supplement: Additional file 6: — List of orphan genes with links to PFAM (related to Additional file 1: Table S1). (ZIP 1428 kb) [file 12864_2016_3244_MOESM6_ESM.zip › BLAST_HTML_FTR/G02994_P.html]

BLAST Search Results


```
BLASTP 2.2.27+


Reference:
Stephen F. Altschul, Thomas L. Madden, Alejandro A. Schäffer,
Jinghui Zhang, Zheng Zhang, Webb Miller, and David J. Lipman (1997),
"Gapped BLAST and PSI-BLAST: a new generation of protein database
search programs", Nucleic Acids Res. 25:3389-3402.


Reference for
composition-based statistics:
Alejandro A. Schäffer, L. Aravind, Thomas L. Madden, Sergei
Shavirin, John L. Spouge, Yuri I. Wolf, Eugene V. Koonin, and
Stephen F. Altschul (2001), "Improving the accuracy of PSI-BLAST
protein database searches with composition-based statistics and
other refinements", Nucleic Acids Res. 29:2994-3005.


Database: nr
           71,551,133 sequences; 26,053,659,533 total letters


Query= G02994_P

Length=89
                                                                      Score     E
Sequences producing significant alignments:                          (Bits)  Value

emb|CED82631.1|  hypothetical protein [Xanthophyllomyces dendrorh...   179    8e-56
ref|WP_007199745.1|  PII uridylyl-transferase [Hoeflea phototroph...  35.0    6.8  
ref|XP_002287139.1|  predicted protein [Thalassiosira pseudonana ...  34.7    8.6  


 >emb|CED82631.1| hypothetical protein [Xanthophyllomyces dendrorhous]
Length=88

 Score =  179 bits (453),  Expect = 8e-56, Method: Compositional matrix adjust.
 Identities = 88/88 (100%), Positives = 88/88 (100%), Gaps = 0/88 (0%)

Query  1   MGIVNKIKEKIVEHDQQANNVEMKQEYLASEDPELRDDVPSNVRASHDASSASQAYKNQA  60
           MGIVNKIKEKIVEHDQQANNVEMKQEYLASEDPELRDDVPSNVRASHDASSASQAYKNQA
Sbjct  1   MGIVNKIKEKIVEHDQQANNVEMKQEYLASEDPELRDDVPSNVRASHDASSASQAYKNQA  60

Query  61  ESDRLTAARSAAVETSVTPEVTSGAGLP  88
           ESDRLTAARSAAVETSVTPEVTSGAGLP
Sbjct  61  ESDRLTAARSAAVETSVTPEVTSGAGLP  88


>ref|WP_007199745.1| PII uridylyl-transferase [Hoeflea phototrophica]
 gb|EDQ35514.1| [Protein-PII] uridylyltransferase [Hoeflea phototrophica DFL-43]
Length=953

 Score = 35.0 bits (79),  Expect = 6.8, Method: Composition-based stats.
 Identities = 18/53 (34%), Positives = 29/53 (55%), Gaps = 0/53 (0%)

Query  10   KIVEHDQQANNVEMKQEYLASEDPELRDDVPSNVRASHDASSASQAYKNQAES  62
            KI   ++Q N V   +  LA ED ELRD +P  + A H ++  ++    +AE+
Sbjct  899  KITNENRQTNIVARLKAVLAKEDDELRDQMPPGMIAPHGSTRRTRTTAAKAET  951


>ref|XP_002287139.1| predicted protein [Thalassiosira pseudonana CCMP1335]
 gb|EED94582.1| predicted protein [Thalassiosira pseudonana CCMP1335]
Length=1014

 Score = 34.7 bits (78),  Expect = 8.6, Method: Compositional matrix adjust.
 Identities = 18/55 (33%), Positives = 34/55 (62%), Gaps = 3/55 (5%)

Query  17   QANNVEMKQEYLASEDPELRDDVPSNVRASHDASSASQAYKNQAESDRLTAARSA  71
            Q NN++ KQ+Y+     E+ D V ++ RA+ D+++ S+   +  + DRL+  +SA
Sbjct  122  QQNNLQNKQKYITQ---EMEDQVMASARATMDSNTVSKVVNSLIDDDRLSKVKSA  173


Lambda      K        H        a         alpha
   0.310    0.122    0.324    0.792     4.96 

Gapped
Lambda      K        H        a         alpha    sigma
   0.267   0.0410    0.140     1.90     42.6     43.6 

Effective search space used: 654964280580


  Database: nr
    Posted date:  Sep 23, 2015 12:05 AM
  Number of letters in database: 26,053,659,533
  Number of sequences in database:  71,551,133


Matrix: BLOSUM62
Gap Penalties: Existence: 11, Extension: 1
Neighboring words threshold: 11
Window for multiple hits: 40
```
