# Supplementary material for: Comparative genomics provides new insights into the diversity, physiology, and sexuality of the only industrially exploited tremellomycete: Phaffia rhodozyma
Source: BMC Genomics. 2016 Nov 9;17:901. doi: 10.1186/s12864-016-3244-7 (PMC5103461; doi:10.1186/s12864-016-3244-7)
Supplement: Additional file 6: — List of orphan genes with links to PFAM (related to Additional file 1: Table S1). (ZIP 1428 kb) [file 12864_2016_3244_MOESM6_ESM.zip › BLAST_HTML_FTR/G02844_P.html]

BLAST Search Results


```
BLASTP 2.2.27+


Reference:
Stephen F. Altschul, Thomas L. Madden, Alejandro A. Schäffer,
Jinghui Zhang, Zheng Zhang, Webb Miller, and David J. Lipman (1997),
"Gapped BLAST and PSI-BLAST: a new generation of protein database
search programs", Nucleic Acids Res. 25:3389-3402.


Reference for
composition-based statistics:
Alejandro A. Schäffer, L. Aravind, Thomas L. Madden, Sergei
Shavirin, John L. Spouge, Yuri I. Wolf, Eugene V. Koonin, and
Stephen F. Altschul (2001), "Improving the accuracy of PSI-BLAST
protein database searches with composition-based statistics and
other refinements", Nucleic Acids Res. 29:2994-3005.


Database: nr
           71,551,133 sequences; 26,053,659,533 total letters


Query= G02844_P

Length=588
                                                                      Score     E
Sequences producing significant alignments:                          (Bits)  Value

emb|CED84712.1|  hypothetical protein [Xanthophyllomyces dendrorh...  1190    0.0  
ref|XP_006257967.1|  PREDICTED: periaxin [Alligator mississippien...  40.4    5.9  
ref|XP_006031475.1|  PREDICTED: periaxin [Alligator sinensis]         40.4    6.3  


 >emb|CED84712.1| hypothetical protein [Xanthophyllomyces dendrorhous]
Length=587

 Score = 1190 bits (3078),  Expect = 0.0, Method: Compositional matrix adjust.
 Identities = 586/587 (99%), Positives = 587/587 (100%), Gaps = 0/587 (0%)

Query  1    MDYPLSINEVNAVTTSSTMTLDQLPFVVLDRLISVIDLSSQASGSSSSSDPNGKNRESLS  60
            MDYPLSINEVNAVTTSSTMTLDQLPFVVLDRLISVIDLSSQASGSSSSSDPNGKNRESLS
Sbjct  1    MDYPLSINEVNAVTTSSTMTLDQLPFVVLDRLISVIDLSSQASGSSSSSDPNGKNRESLS  60

Query  61   YRRPRKVSKSISELAMTSRTFASLTSRLRFRSISLCSCPHGKKFWTWLMDHQEVWGMIQE  120
            YRRPRKVSKSISELAMTSRTFASLTSRLRFRSISLCSCPHGKKFWTWLMDHQEVWGMIQE
Sbjct  61   YRRPRKVSKSISELAMTSRTFASLTSRLRFRSISLCSCPHGKKFWTWLMDHQEVWGMIQE  120

Query  121  ITVTAGCDVTSLVGPQVEMFFGALPSLELVYYEPFERLSDWLAGMISQLPSLQSVVLINS  180
            ITVTAGCDVTSLVGPQVEMFFGALPSLELVYYEPFERLSDWLAGMISQLPSLQSVVLINS
Sbjct  121  ITVTAGCDVTSLVGPQVEMFFGALPSLELVYYEPFERLSDWLAGMISQLPSLQSVVLINS  180

Query  181  GITSSPQLGLFQGLEQLTIYPGFVSPWSETTYRIEQSQPAVVLASAGRREEEESVETEET  240
            GITSSPQLGLFQGLEQLTIYPGFVSPWSETTYRIEQSQPAVVLASAGRREEEESVETEET
Sbjct  181  GITSSPQLGLFQGLEQLTIYPGFVSPWSETTYRIEQSQPAVVLASAGRREEEESVETEET  240

Query  241  TGGTLVKRTPERRTLDSDAINRALAKSARTLKLLHLICPRDAPRLTAETIFTDPSLPTKT  300
            TGGTLVKRTPERRTLDSDAINRALAKSARTLKLLHLICPRDAPRLTAETIFTDPSLPTKT
Sbjct  241  TGGTLVKRTPERRTLDSDAINRALAKSARTLKLLHLICPRDAPRLTAETIFTDPSLPTKT  300

Query  301  ALKLPMLERLFIRPADLSTDLFPDLLLGCPRLRLLSITHMPMGPKSTLCIPENSLPKLEV  360
            ALKLPMLERLFIRPADLSTDLFPDLLLGCPRLRLLSITHMPMGPKSTLCIPENSLPKLEV
Sbjct  301  ALKLPMLERLFIRPADLSTDLFPDLLLGCPRLRLLSITHMPMGPKSTLCIPENSLPKLEV  360

Query  361  LNLIVPEDSYTDSPGLYASAVNLIKGKTQVWKLELDVPFGLMERLFQRLEVPNGVKELTI  420
            LNLIVPEDSYTDSPGLYASAVNLIKGKTQ+WKLELDVPFGLMERLFQRLEVPNGVKELTI
Sbjct  361  LNLIVPEDSYTDSPGLYASAVNLIKGKTQLWKLELDVPFGLMERLFQRLEVPNGVKELTI  420

Query  421  GQWVDQKLLGLIDEKFPGLERLTFESGRWGLDMEDSIHLPLYLAHLRSLRSLHLDFPLSP  480
            GQWVDQKLLGLIDEKFPGLERLTFESGRWGLDMEDSIHLPLYLAHLRSLRSLHLDFPLSP
Sbjct  421  GQWVDQKLLGLIDEKFPGLERLTFESGRWGLDMEDSIHLPLYLAHLRSLRSLHLDFPLSP  480

Query  481  TVLPLATINFFDEAAPFFHILPLIKSTSEEIQKQILPRMVPVVLGLFENSAELEEIVWSF  540
            TVLPLATINFFDEAAPFFHILPLIKSTSEEIQKQILPRMVPVVLGLFENSAELEEIVWSF
Sbjct  481  TVLPLATINFFDEAAPFFHILPLIKSTSEEIQKQILPRMVPVVLGLFENSAELEEIVWSF  540

Query  541  GNTDWFWRMGTYDQLEMDLGFCLGRGRRVMGGSAIKTKTEWADGIRH  587
            GNTDWFWRMGTYDQLEMDLGFCLGRGRRVMGGSAIKTKTEWADGIRH
Sbjct  541  GNTDWFWRMGTYDQLEMDLGFCLGRGRRVMGGSAIKTKTEWADGIRH  587


>ref|XP_006257967.1| PREDICTED: periaxin [Alligator mississippiensis]
Length=867

 Score = 40.4 bits (93),  Expect = 5.9, Method: Compositional matrix adjust.
 Identities = 33/98 (34%), Positives = 50/98 (51%), Gaps = 10/98 (10%)

Query  301  ALKLPMLERLFIRPADLSTDLFPDLLLGCPRLRLLSITHMPMGPKSTLCIPENSLPKLEV  360
            ALK+P ++ +  R AD+     PD+ L   +L +      P GP+    +P+ SLPKLE+
Sbjct  492  ALKMPSIDIVVPRAADVELPPAPDVRLPTAKLEMPG----PEGPEVKFKLPQVSLPKLEL  547

Query  361  LNLIVPEDSYTDSPGLYASAVNLIKGKTQVWKLELDVP  398
               +  E      PG    A+ L+ GK  + KL+L VP
Sbjct  548  AGKV--ELEPEPEPG----ALELLAGKIGMPKLDLSVP  579


>ref|XP_006031475.1| PREDICTED: periaxin [Alligator sinensis]
Length=1093

 Score = 40.4 bits (93),  Expect = 6.3, Method: Compositional matrix adjust.
 Identities = 33/98 (34%), Positives = 49/98 (50%), Gaps = 10/98 (10%)

Query  301  ALKLPMLERLFIRPADLSTDLFPDLLLGCPRLRLLSITHMPMGPKSTLCIPENSLPKLEV  360
            ALK+P ++    R AD+     PD+ L   +L +      P GP+    +P+ SLPKLE+
Sbjct  202  ALKMPSIDIAVPRAADVELPPAPDVRLPTAKLEMPG----PEGPEVKFKLPQVSLPKLEL  257

Query  361  LNLIVPEDSYTDSPGLYASAVNLIKGKTQVWKLELDVP  398
               +  E      PG    A+ L+ GK  + KL+L VP
Sbjct  258  AGKV--ELEPEPEPG----ALELLAGKIGMPKLDLSVP  289


Lambda      K        H        a         alpha
   0.320    0.137    0.410    0.792     4.96 

Gapped
Lambda      K        H        a         alpha    sigma
   0.267   0.0410    0.140     1.90     42.6     43.6 

Effective search space used: 6387476742012


  Database: nr
    Posted date:  Sep 23, 2015 12:05 AM
  Number of letters in database: 26,053,659,533
  Number of sequences in database:  71,551,133


Matrix: BLOSUM62
Gap Penalties: Existence: 11, Extension: 1
Neighboring words threshold: 11
Window for multiple hits: 40
```
